# Cancer genomic profiling identified dihydropyrimidine dehydrogenase deficiency in bladder cancer promotes sensitivity to gemcitabine

**DOI:** 10.1038/s41598-022-12528-3

**Published:** 2022-05-20

**Authors:** Shigehiro Tsukahara, Masaki Shiota, Dai Takamatsu, Shohei Nagakawa, Takashi Matsumoto, Ryo Kiyokoba, Mikako Yagi, Daiki Setoyama, Nozomi Noda, Shinya Matsumoto, Tetsutaro Hayashi, Alberto Contreras-Sanz, Peter C. Black, Junichi Inokuchi, Kenichi Kohashi, Yoshinao Oda, Takeshi Uchiumi, Masatoshi Eto, Dongchon Kang

**Affiliations:** 1 Department of Urology, Graduate School of Medical Sciences, Kyushu University, 3-1-1 Maidashi, Higashi-ku, Fukuoka, 812-8582 Japan; 2 Department of Clinical Chemistry and Laboratory Medicine, Graduate School of Medical Sciences, Kyushu University, 3-1-1 Maidashi, Higashi-ku, Fukuoka, 812-8582 Japan; 3 Department of Anatomic Pathology, Graduate School of Medical Sciences, Kyushu University, Fukuoka, Japan; 4 Department of Urology, Graduate School of Biomedical and Health Sciences, Hiroshima University, Hiroshima, Japan; 5 Department of Urologic Sciences, University of British Columbia, Vancouver, BC Canada; 6 Department of Health and Science, School of Medicine, Kyushu University, Fukuoka, Japan

**Keywords:** Cancer, Oncology, Urology

## Abstract

Chemotherapy is a standard therapy for muscle-invasive bladder cancer (MIBC). However, genomic alterations associated with chemotherapy sensitivity in MIBC have not been fully explored. This study aimed to investigate the genomic landscape of MIBC in association with the response to chemotherapy and to explore the biological role of genomic alterations. Genomic alterations in MIBC were sequenced by targeted exome sequencing of 409 genes. Gene expression in MIBC tissues was analyzed by western blotting, immunohistochemistry, and RNA microarray. Cellular sensitivity to gemcitabine and gemcitabine metabolite was examined in bladder cancer cells after modulation of candidate gene. Targeted exome sequencing in 20 cases with MIBC revealed various genomic alterations including pathogenic missense mutation of *DPYD* gene encoding dihydropyrimidine dehydrogenase (DPD). Conversely, high *DPYD* and DPD expression were associated with poor response to gemcitabine-containing chemotherapy among patients with MIBC, as well as gemcitabine resistance in bladder cancer cells. DPD suppression rendered cells sensitive to gemcitabine, while DPD overexpression made cells gemcitabine-resistant through reduced activity of the cytotoxic gemcitabine metabolite difluorodeoxycytidine diphosphate. This study revealed the novel role of DPD in gemcitabine metabolism. It has been suggested that *DPYD* genomic alterations and DPD expression are potential predictive biomarkers in gemcitabine treatment.

## Introduction

Bladder cancer (BC) is the ninth most commonly diagnosed cancer worldwide^1^. Approximately 10–15% of cases present as muscle-invasive bladder cancer (MIBC)^2^. Surgical management of MIBC alone is inadequate in many cases, and disease control rates are improved with the addition of neoadjuvant chemotherapy (NAC)^3,4^. Currently, gemcitabine/cisplatin (GC) is the most widely used chemotherapy regimen in the peri-operative and advanced settings. Alternatively, gemcitabine/carboplatin (GCarbo) is used to treat advanced disease in cisplatin-ineligible patients, which make up approximately half of the patient population. Meanwhile, methotrexate/vinblastine/doxorubicin/cisplatin (MVAC) chemotherapy is less commonly used due to higher rates of toxicity^1,5,6^.

Approximately 60% of patients with advanced BC will respond to platinum-based chemotherapy, but that response is rarely durable. In the NAC setting, approximately 40% have a major response and are thought to benefit from the chemotherapy, while the other 60% are at risk of complications and delay in definitive surgical intervention without clear benefit^1^. It is therefore critical to predict NAC efficacy in patients with MIBC in order to administer NAC more selectively prior to radical cystectomy^4^. Predictive biomarkers of response to chemotherapy would have significant impact on personalized multimodal therapy in patients with MIBC.

Thus far, several studies have demonstrated the association between specific genomic alterations and tumor response to chemotherapy. In urothelial carcinoma including BC, somatic mutations in DNA damage repair genes (*ERCC2*, *ATM*, *RB1*, *FANCC*, and *BRCA2*) have been correlated with sensitivity to cisplatin^1,7–9^. However, analyses beyond DNA damage repair genes to predict tumor response to chemotherapy in BC has been limited. Therefore, this study aimed to reveal genomic alterations associated with tumor response to platinum-based chemotherapy for BC by targeted exome sequencing of 409 genes. On the basis of the findings obtained by targeted exome sequencing, the biological role and clinical value of identified genomic alterations were explored.

## Results

### Mutation landscape in MIBC by targeted exome sequencing

**Figure 1:**
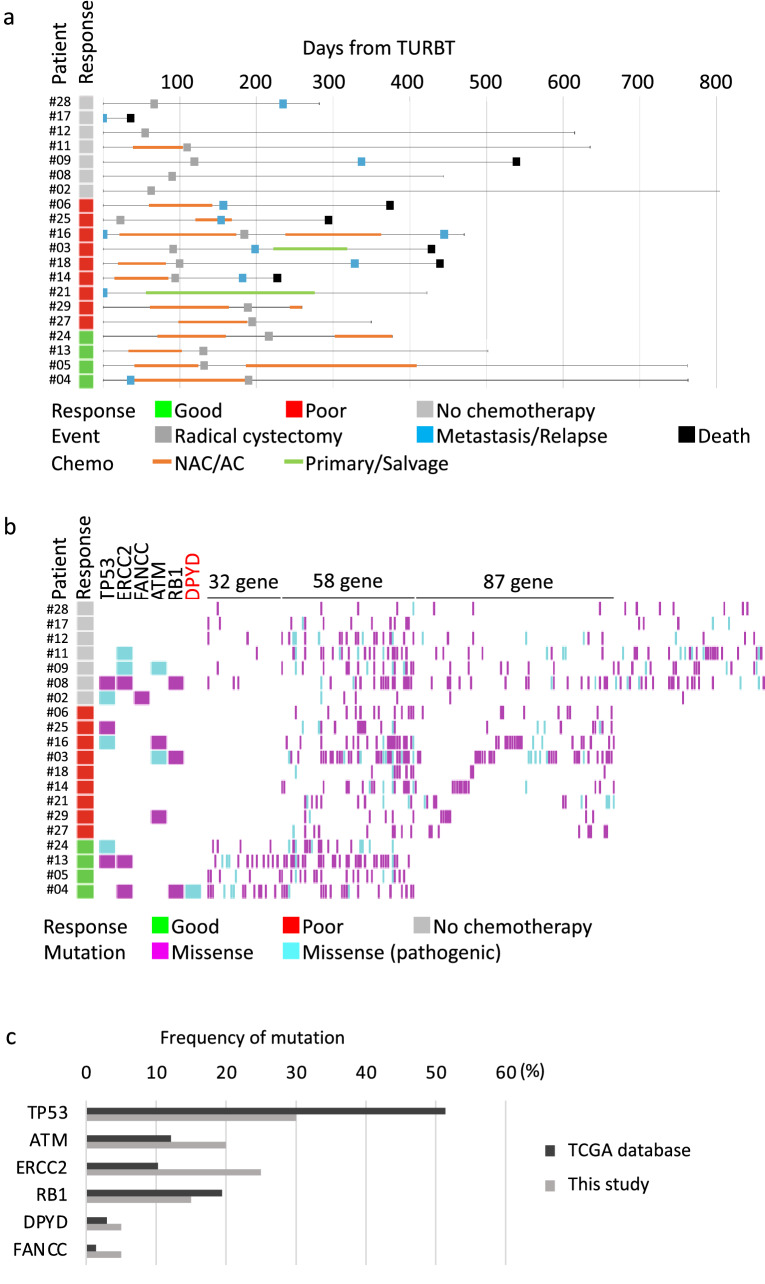
Clinical course and mutation landscape in 20 patients with BC. (**a**) Swimmers’ plot of 20 patients from TURBT. Tumor response to chemotherapy, events, and purpose of chemotherapy are shown. NAC, neoadjuvant chemotherapy; AC, adjuvant chemotherapy. (**b**) Overview of genomic mutations detected in 20 patients with MIBC. Deleterious missense mutations were colored light blue. Genomic alterations in *TP53*, *ERCC2*, *FANCC*, *ATM*, *RB1*, and *DPYD* were shown. RC, radical cystectomy. (**c**) Frequency of genomic alterations (*TP53*, *ERCC2*, *FANCC*, *ATM*, *RB1*, and *DPYD*) detected in this study (gray bar) and TCGA database (black bar) were shown.

To characterize the genomic landscape of MIBC, we performed targeted exome sequencing of 409 cancer-associated genes in MIBC tissues from 20 patients. The clinicopathological characteristics are provided in Supplementary Table S1, and their clinical courses are shown in Fig. 1a. Among 20 patients, 13 received platinum-based chemotherapy. Tumor response to chemotherapy was good (ypT0N0 after NAC or complete response during primary/salvage treatment) in 4 patients, and poor (pN1 after NAC or stable disease or progressive disease after primary/salvage treatment) in 9 patients. During a median follow-up of 14.7 months (interquartile range, 12.2–19.6 months), 11 patients developed metastasis, and seven patients died due to disease progression among 20 patients with MIBC. Notably, five patients among nine poor responders died from BC (Fig. 1a). By targeted exome sequencing, 1159 somatic mutations in total were detected in the tumor tissue from 20 patients (Fig. 1b, Supplementary Table S2). Sequencing of leukocyte-derived gDNA has confirmed that gene mutations are not germline mutations. Tumor mutation burden (TMB) was comparable among tumor tissues with good (25.2 muts/Mb) and poor (21.3 muts/Mb) responses (P = 0.594).

All genomic alterations were classified as non-pathogenic mutation, and pathogenic mutation. The following genomic alterations reported to be associated with response of chemotherapy were detected; *TP53* in 6 cases, *ERCC2* in 5 cases, *FANCC* in 1 case, *ATM* in 4 cases, and *RB1* in 3 cases (Fig. 1b and Supplementary Fig. S1a). Although the frequency of genomic alterations in most genes differed from those reported in TCGA study, the frequency of *DPYD* alteration was comparable with that reported in TCGA study (Fig. 1c and Supplementary Fig. S1b).

**Figure 2:**
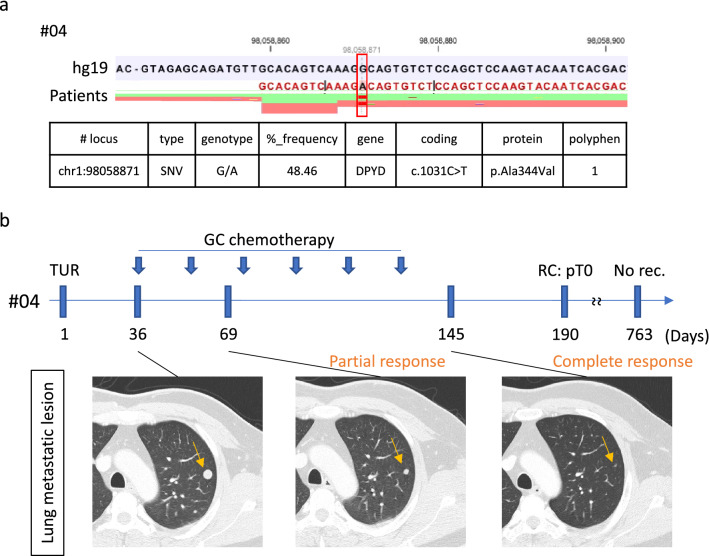
*DPYD* mutation and clinical course of patient #04 who had good response to chemotherapy with gemcitabine. (**a**) Sequence of *DPYD* gene in tumor tissue from patient #04. Width of green and orange bands indicates the forward and reverse read depth, respectively. Red band indicates mutated nucleotide. (**b**) Clinical course of patient #04 from TUR is shown. GC, gemcitabine/cisplatin; TUR, transurethral resection; CR, complete response; RC, radical cystectomy; No rec., no recurrence.

Interestingly, patient #04 harbored a single-nucleotide variant (c.1031 C > T) leading to amino-acid substitution as p.Ala334Val (valiant allele frequency, 48.5%) (Fig. 2a), and showed a complete response of lung metastasis and the primary bladder tumor to 6 cycles of GC chemotherapy followed by cystectomy (Fig. 2b). This missense mutation in *DPYD* gene was judged as pathogenic by Polyphen2 (score = 1.000). Then, among various gene alterations, we focused *DPYD* gene encoding dihydropyrimidine dehydrogenase (DPD) because gemcitabine metabolite structure, difluorodeoxyuridine (dFdU), was similar to 5-FU, to which DPD is well known to be involved in cellular resistance^10,11^.

### DPD expression was associated with response to chemotherapy

**Figure 3:**
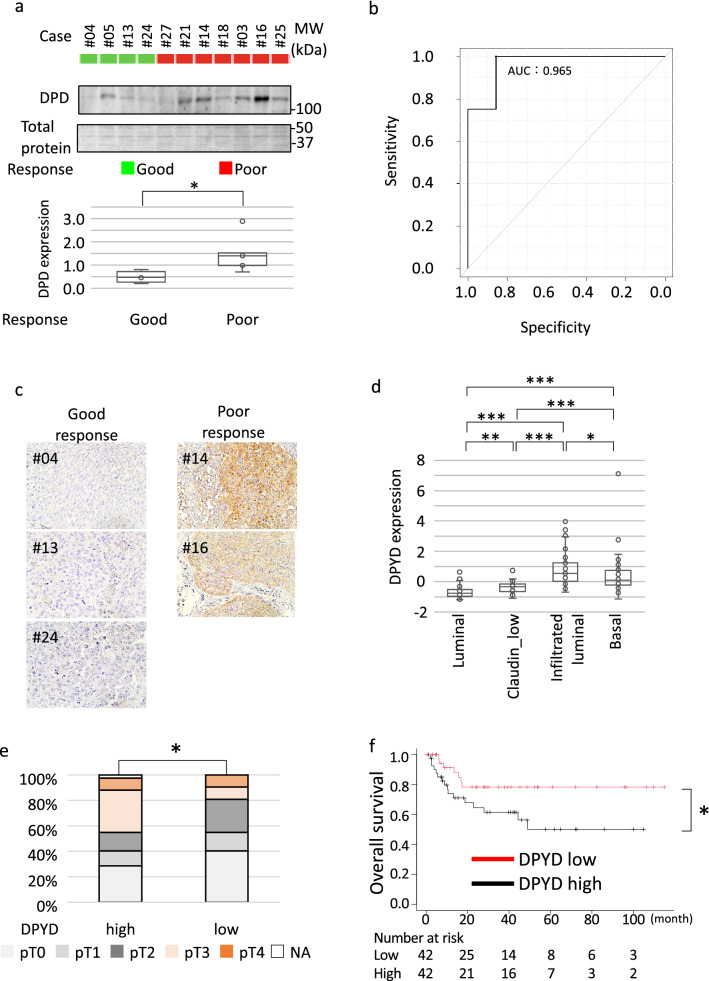
DPD expression was associated with response to gemcitabine-containing chemotherapy. (**a**) (upper panel) Protein levels of DPD in TUR sections from patients with a good response and patients with a poor response excluding the sample from #06 due to poor quality were detected by western blotting. (lower panel) Quantitative protein levels of DPD in good and poor responders were displayed. *P < 0.05. (**b**) Receiver operating characteristic curves for prediction of response to chemotherapy by protein level of DPD are shown. AUC, area under curve. (**c**) Immunohistochemistry of TUR sections from patients with a good response and patients with a poor response are shown. Brown stain represents DPD protein expression. Magnification, × 400. (**d**) *DPYD* mRNA expression levels in 223 patients (GSE87304) were shown by molecular subtypes. *P < 0.05, **P < 0.01, ***P < 0.001. (**e**) Pathological T-stage in patients (GSE87304) with highest (n = 42) and lowest (n = 42) quartile of *DPYD* expression when treated with gemcitabine-containing chemotherapy. *P < 0.05. (**f**) Overall survival in patients (GSE87304) between in patients with highest (n = 42) and lowest (n = 42) quartile of *DPYD* expression when treated with gemcitabine-containing chemotherapy. *P < 0.05. Vertical bar on Kaplan–Meier curve represents censor.

DPD plays a critical role in the metabolism of pyrimidine-based drugs including gemcitabine^12^, and therefore we focused on the clinical significance of DPD in gemcitabine-containing chemotherapy. First, the association between DPD expression and response to chemotherapy was examined in tumors with good and poor responses to chemotherapy. DPD protein expression was lower in tumors with a good response while DPD protein expression was higher in tumors with a poor response (P = 0.012, Fig. 3a, Supplementary Fig. S2a and S2b). The sensitivity and specificity of predicting response from DPD protein expression at a cutoff value of 0.804 were 0.857 and 1.000, respectively (Fig. 3b). Consistently, representative imaging obtained by immunohistochemistry showed an absence of DPD staining in tissues from the good responders, while positive DPD staining was detected in tissues from the poor responders (Fig. 3c).

Next, the association between *DPYD* mRNA expression and tumor response to NAC was explored using microarray data (GSE87304) from 223 patients who received NAC regimens including gemcitabine^13^. When the association between *DPYD* mRNA expression and molecular subtypes, *DPYD* expressions were highest and lowest in claudin-low and luminal subtypes, respectively (Fig. 3d). We separated 169 patients who received gemcitabine-containing NAC from 54 patients who received non-gemcitabine containing MVAC, and compared the highest and lowest quartiles in each treatment group based on *DPYD* expression (Table 1). Residual extravesical extension after gemcitabine-containing NAC (ypT3/4) was found more frequently among tumors with high *DPYD* expression, compared with tumors with low *DPYD* expression (P = 0.019, Fig. 3e). Overall survival was worse in patients with *DPYD*-expressing tumors compared to those with low *DPYD*-expressing tumors after treatment with gemcitabine-containing NAC (Fig. 3f). In contrast, *DPYD* expression was associated with neither tumor response nor overall survival in patients treated with MVAC (Supplementary Figs. S3a and S3b). Similarly, TCGA study showed that disease-free survival was comparable between higher and lower expression of *DPYD* among 137 patients without chemotherapy (Supplementary Fig. S3c)^14^.

**Table 1:** Clinicopathological information of patients.

Variables	*DPYD* expression in patients treated with gemcitabine			*DPYD* expression in patients treated without gemcitabine		
	High (n = 42)	Low (n = 42)	P-value	High (n = 27)	Low (n = 27)	P-value
Median age, years (interquartile range)	66 (57–72)	61.8 (57–73)	0.71	57 (50–66)	56 (53–61)	0.80
**Gender, n (%)**						
Male	28 (66.7%)	34 (81.0%)		19 (70.3%)	20 (74.1%)	
Female	14 (33.3%)	8 (19.0%)	0.21	8 (29.7%)	7 (25.9%)	0.76
**Clinical T-stage, n (%)**						
cT2	17 (40.5%)	24 (57.1%)		5 (18.5%)	11 (40.7%)	
cT3	17 (40.5%)	13 (30.9%)		16 (59.3%)	9 (33.3%)	
cT4	8 (19.0%)	5 (11.9%)	0.34	6 (22.2%)	7 (25.9%)	0.11
**Pathological T-stage, n (%)**						
pT0	14 (33.3%)	20 (47.6%)		12 (44.4%)	11 (40.7%)	
pT1	3 (7.1%)	2 (4.8%)		2 (7.4%)	2 (7.4%)	
pT2	6 (14.3%)	11 (26.2%)		1 (3.7%)	7 (25.9%)	
pT3	14 (33.3%)	5 (11.9%)		4 (14.8%)	5 (18.5%)	
pT4	5 (11.9%)	4 (9.5%)	0.04*	6 (22.2%)	2 (7.4%)	0.38
NA	0 (0%)	0 (0%)		2 (7.4%)	0 (0%)	
**Pathological N-stage, n (%)**						
pN0	33 (78.6%)	31 (73.8%)		17 (63.0%)	12 (44.4%)	
pN1–3	5 (11.9%)	10 (23.8%)	0.26	0 (0%)	0 (0%)	1.00
NA	4 (9.5%)	1 (2.4%)		10 (37.0%)	15 (55.6%)	
**NAC regimen, n (%)**						
GC	37 (88.1%)	36 (85.7%)		0 (0%)	0 (0%)	
GCarbo	5 (11.9%)	6 (14.3%)	0.75	0 (0%)	0 (0%)	
MVAC	0 (0%)	0 (0%)		27 (100%)	27 (100%)	1.00
**Recurrence, n (%)**						
Absence	26 (61.9%)	30 (71.4%)		17 (63.0%)	16 (59.3%)	
Presence	16 (38.1%)	12 (28.6%)	0.49	8 (29.6%)	11 (40.7%)	0.57
NA	0 (0%)	0 (0%)		2 (7.4%)	0 (0%)	
**Survival, n (%)**						
Alive	26 (61.9%)	35 (83.3%)		20 (74.1%)	18 (66.7%)	
Death	16 (38.1%)	7 (16.7%)	0.049*	7 (25.9%)	9 (33.3%)	0.77

NA, not available; NAC, neoadjuvant chemotherapy; GC, gemcitabine/cisplatin; Gcarbo, gemcitabine/carboplatin; MVAC, methotrexate/vinblastine/doxorubicin/cisplatin.

*Statistically significant.

GSEA analysis was performed to ascertain the gene networks associated with *DPYD* expression using the cohort (GSE87304). Although several gene set were significant, there was no apparent functional signature associated with gemcitabine resistance (Supplementary Table S3).

### DPD expression modulated cellular sensitivity to gemcitabine

**Figure 4:**
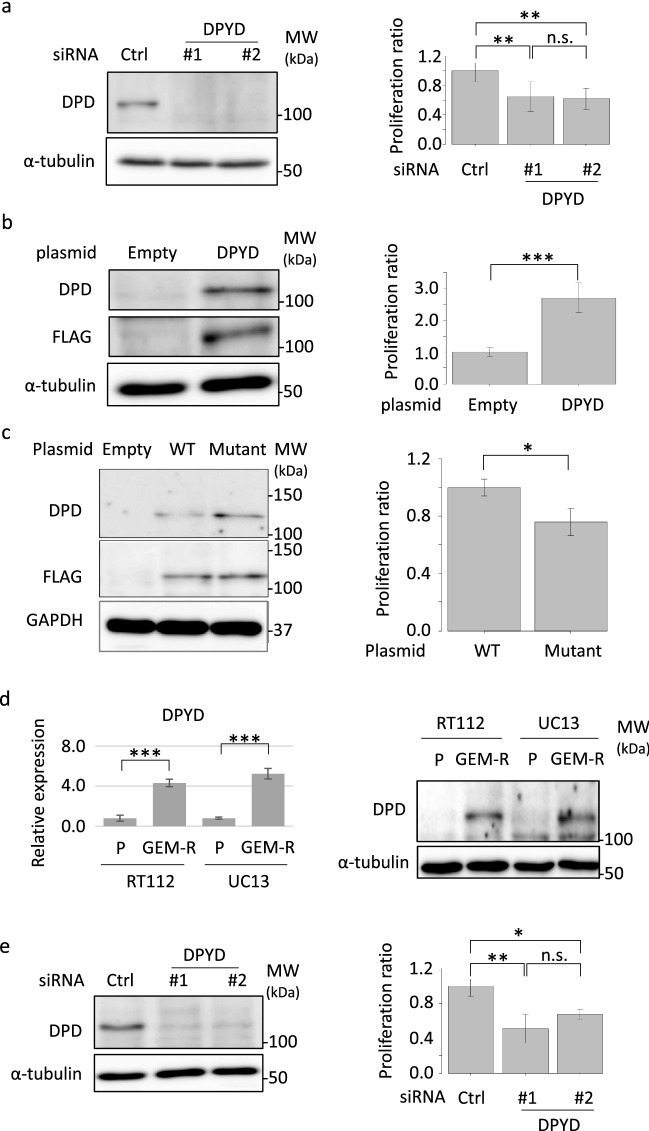
DPD expression modulated cellular sensitivity to gemcitabine. (**a**) (left panel) UM-UC-3 cells were transfected with 25 nM each of the indicated siRNA and incubated for 72 h. Whole-cell extracts from UM-UC-3 cells were subjected to SDS-PAGE followed by western blotting for the indicated proteins. (right panel) UM-UC-3 cells transfected with siRNA were incubated with 0 or 22.0 μM gemcitabine. After 48 h, cell numbers were counted. Relative cell counts when transfected with control siRNA were defined as 1. Boxes, mean; bars, ± SD. **P < 0.01 (one-way ANOVA). (**b**) (left panel) Whole-cell extracts from HEK293 cells and DPD-overexpressing HEK293 cells were subjected to SDS-PAGE followed by western blotting for the indicated proteins. (right panel) HEK293 cells and DPD-overexpressing HEK293 cells were incubated with 0 or 6.67 nM gemcitabine. After 48 h, cell numbers were counted. Relative cell counts of HEK293 cells were defined as 1. Boxes, mean; bars, ± SD. ***P < 0.001 (Student’s t-test). (**c**) (left panel) HEK293 cells were transfected with 5 μg each of the indicated plasmid and incubated for 72 h. Whole-cell extracts from HEK293 cells were subjected to SDS-PAGE followed by western blotting for the indicated proteins. (right panel) HEK293 cells were transfected with 5 μg each of the indicated plasmid and incubated with 0 or 33.3 nM gemcitabine. After 48 h, cell proliferation was measured with CellTiter Glo. Relative cell proliferation when transfected with wild-type *DPYD* expression plasmid was defined as 1. Boxes, mean; bars, ± SD. *P < 0.05 (Student’s t-test.). (**d**) (left panel) After extraction of total RNA from parental (RT112-P and UM-UC-13-P) and gemcitabine-resistant RT112 (RT112 GEM-R) and UM-UC-13 (UM-UC-13 GEM-R) cells and DNA synthesis, quantitative real-time PCR was performed for *DPYD* and *18 s ribosomal RNA*. Each target transcript level was corrected relative to the corresponding *18 s ribosomal RNA* transcript level. The level of each target transcript in parental cells was defined as 1. Boxes, mean; bars, ± SD. ***P < 0.001 (Student’s t-test). (right panel) Whole-cell extracts from parental and GEM-R RT112 and UM-UC-13 cells were subjected to SDS-PAGE followed by western blotting for the indicated proteins. (**e**) (left panel) RT112 GEM-R cells were transfected with 25 nM each of the indicated siRNA and incubated for 72 h. Whole-cell extracts from RT112 GEM-R cells were subjected to SDS-PAGE followed by western blotting for the indicated proteins. (right panel) RT112 GEM-R cells were transfected with siRNA and incubated with 0 or 10.0 μM gemcitabine. After 48 h, cell numbers were counted. Relative cell counts when transfected with control siRNA were defined as 1. Boxes, mean; bars, ± SD. *P < 0.05, **P < 0.01 (one-way ANOVA).

To delineate the association between DPD expression and sensitivity to gemcitabine, DPD expression was modulated and cellular sensitivity to gemcitabine was examined. When DPD expression was reduced using specific siRNA, UM-UC-3 cells became sensitive to gemcitabine (Fig. 4a, Supplementary Fig. S4a and S4d), but not to cisplatin (Supplementary Fig. S5). Conversely, stable *DPYD* overexpression using a *DPYD*-expression plasmid conferred resistance to gemcitabine in HEK293 cells (Fig. 4b and Supplementary Fig. S4e–S4k). However, when mutated *DPYD* (c.1031C > T) found in patient #04 was overexpressed in HEK293 cells, cellular viability was lower compared with cells overexpressing wild-type *DPYD* (Fig. 4c and Supplementary Fig. S4l–S4r).

Next, *DPYD* mRNA and DPD protein expression was compared between parental- (P) and gemcitabine-resistant (GEM-R) cell lines. Higher expression levels of *DPYD* mRNA and DPD protein were observed in RT112 GEM-R and UM-UC-13 GEM-R cells compared with parental cells (Fig. 4d and Supplementary Fig. S4s–S4u). When DPD protein expression was reduced using specific siRNA in RT112 GEM-R cells, gemcitabine resistance was reversed (Fig. 4e, Supplementary Fig. S4v and S4x).

### Gemcitabine metabolism by DPD

**Figure 5:**
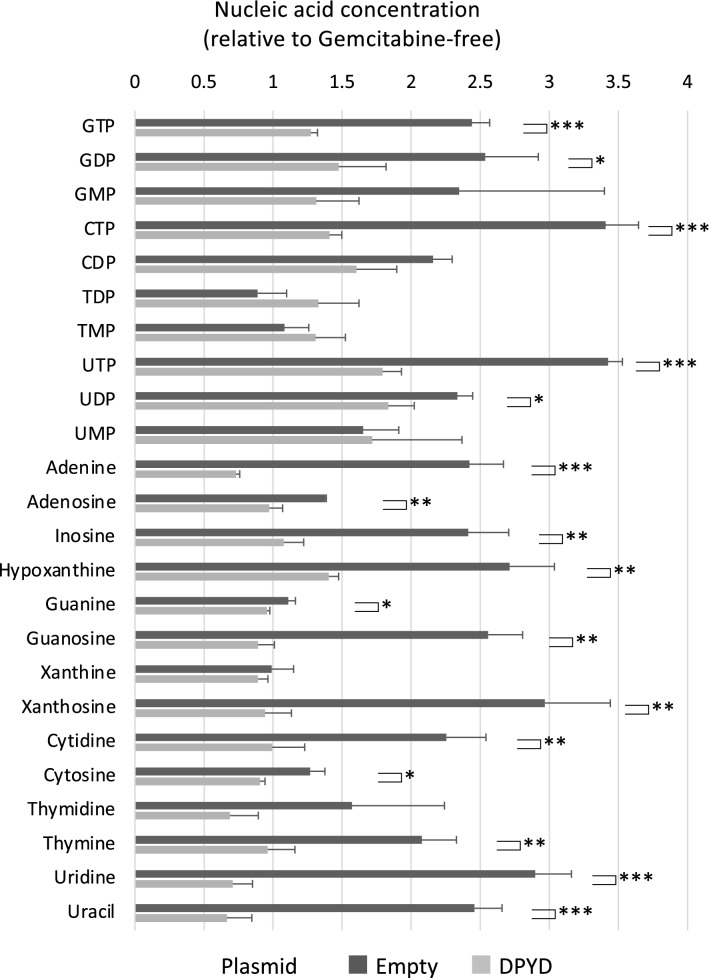
Nucleic acid-related metabolites after exposure to gemcitabine. Nucleic acid-related metabolites in HEK293 cells and DPD-overexpressing HEK293 cells cultured in medium containing 0 or 6.67 nM gemcitabine for 24 h. The concentrations of metabolites when exposed to gemcitabine were corrected against those not exposed to gemcitabine. Boxes, mean; bars, ± SD. *P < 0.05, **P < 0.01, ***P < 0.001 (Student’s t-test).

Finally, we investigated the mechanism of DPD-derived gemcitabine resistance by examining nucleotide metabolism with LC–MS/MS. Difluorodeoxycytidine diphosphate (dFdCDP, a gemcitabine metabolite) is a ribonucleotide reductase (RNR) inhibition. RNR is an enzyme that converts ribose to deoxyribose, which is very important for generating dNTPs for DNA synthesis. As a result of the inhibition of RNR, increased intracellular concentrations of NMP, NDP, and NTP can be observed as an effect of gemcitabine because deoxyribonucleic acid is depleted while ribonucleic acid is stored. As shown in Fig. 5, gemcitabine induced increased levels of ribonucleotides and ribonucleosides in low DPD-expressing HEK293 cells. In contrast, these metabolic changes were abrogated in *DPYD*-overexpressing HEK293 cells (Fig. 5).

## Discussion

Clinical data in this study suggested that genomic alteration in *DPYP* and gene expression level of *DPYP* were associated with response to gemcitabine-containing chemotherapy. In line with this notion, experimental data in this study have shown that DPD promoted resistance to gemcitabine through deactivation of gemcitabine.

DPD expression is associated with tumor response to 5-fluorouracil (5-FU) in BC cells^10–17^. Because 5-FU is metabolized and deactivated by DPD, DPD may protect cells from cytotoxicity by 5-FU (Supplementary Fig. S6)^10^. Similarly, this study revealed that DPD is a critical determinant of chemosensitivity to gemcitabine in BC. Gemcitabine (difluorodeoxycytidine; dFdC) is a pyrimidine antagonist, similar to 5-FU. As a molecular mechanism involved in gemcitabine resistance, gemcitabine metabolism and deactivation by DPD were suggested. Gemcitabine is metabolized to the bioactive form of difluorodeoxycytidine monophosphate (dFdCMP), diphosphate (dFdCDP, a potent inhibitor of the enzyme ribonucleotide reductase), and triphosphate (dFdCTP, incorporated as a false precursor) by dCK, nucleotide monophosphate kinase (NMPK), and nucleotide diphosphokinase (NDPK), respectively (Supplementary Fig. S6). Meanwhile, more than 90% of gemcitabine is inactivated by CDA into dFdU^10^, which may be a substrate of DPD and metabolized by DPD (Supplementary Fig. S6). Then, we hypothesized that impaired activity of DPD by low expression or dysfunction due to mutations may result in decrease inactivation of gemcitabine, and instead increased metabolism of gemcitabine to its bioactive form. Actually, for the first time, to our knowledge, this study showed that DPD robustly modulated nucleotide metabolism, suggesting deactivation of gemcitabine by DPD.

Previous study has reported that DPD expression is associated with the clinical benefit of adjuvant 5-FU chemotherapy for upper urinary tract urothelial carcinoma^16^. Furthermore, in the ESPAC-3(v2) clinical trial, high expression of DPD was associated with poor survival when patients were treated with 5-FU or gemcitabine for pancreatic cancer^18^. Consistently, this study showed that high *DPYD* expression was associated with poor response and survival following administration of gemcitabine-containing chemotherapy. Additionally, in our analysis of MIBC microarray data, high and low *DPYD* expression was associated with poor-prognosis claudin-low and good-prognosis luminal subtypes, respectively. Thus, the molecular subtypes may be associated with prognosis through DPD expression in MIBC.

Genetic variants in *DPYD* were reported to affect 5-FU metabolism and are associated with adverse events in chemotherapy using gemcitabine for pancreatic cancer^19,20^. However, this study notably reported the significance of *DPYD* genomic alterations of tumor cells following gemcitabine-containing chemotherapy. Thus, while germline deleterious loss-of-function in *DPYD* results in increased cytotoxicity by gemcitabine in normal cells, somatic alterations in cancer cells lead to augmented clinical benefit by gemcitabine. Taken together, these findings indicated that *DPYD* mRNA expression and DPD protein expression, as well as genomic alterations in *DPYD*, in cancer cells may be predictive markers of efficacy in 5-FU- and gemcitabine-containing chemotherapy regimens.

However, this study suffers from some limitations. Sample number for analysis of genomic alterations by targeted exome sequencing was small to perform statistical analysis. In addition, there was imbalance of sample number between good and poor responders. HEK293 and UM-UC-3 cells were utilized in in vitro experiments. However, HEK293 cells were not BC cell line, and UM-UC-3 cells are a hypertriploid cell line, which might affect the result in this study (CRL-1749, ATCC, https://www.atcc.org/products/crl-1749).

In conclusion, this study demonstrated the genomic landscape of MIBC, and the novel role of DPD in gemcitabine metabolism, suggesting that DPD is a critical molecule in cellular sensitivity to gemcitabine through gemcitabine deactivation. *DPYD* genomic alterations and expression levels may be useful biomarkers to predict tumor response to gemcitabine-containing chemotherapy in BC.

## Materials and methods

### Sample collection

This study included 20 patients diagnosed with MIBC at Kyushu University Hospital (Fukuoka, Japan) between 2019 and 2021. Of these 20 patients, nine and four patients received chemotherapy as neoadjuvant and primary/salvage treatments, respectively. Tumor tissues and blood samples were banked at − 80 °C until extraction of DNA or protein. Written informed consent was obtained from all patients, and the study was approved by the institutional review board (#2020-254). The study was conducted in accordance with the principles described in the Declaration of Helsinki and the Ethical Guidelines for Epidemiological Research enacted by the Japanese Government.

### Targeted exome sequencing

Tumor DNA was extracted from tissues obtained by transurethral resection (TUR) or radical cystectomy with a DNeasy blood and tissue kit (Qiagen, Hilden, Germany). Germline DNA (gDNA) was extracted from blood collected before undergoing chemotherapy. Targeted exome sequencing was performed using the Ion Ampliseq Comprehensive Cancer Panel (CCP; #4,477,685; Thermo Fisher, Waltham, MA, USA), which consisted of four primer pools totaling nearly 16,000 primer pairs covering 1.23 Mb of 409 genes (Supplementary Table S4)^21^. DNA (10 ng) from 20 tumors and matched gDNA was used for libraries. Libraries were generated by the Ion Torrent Ion Chef system (Thermo Fisher) with the Ion Ampliseq Library kit plus (Thermo Fisher) and sequenced by an Ion PGM Sequencer (Thermo Fisher). We analyzed sequences using Ion Reporter Software 5.18.1.0 (Thermo Fisher, https://ionreporter.thermofisher.com/ir/). The mean depth of read coverage for the target genes was 10^3^ × (interquartile range, 48.5–139). Mutations reported as “pathogenic” or “likely pathogenic” in dbSNP (https://www.ncbi.nlm.nih.gov/snp/), and mutations with Polyphen2 score > 0.9 (http://genetics.bwh.harvard.edu/pph2/) were determined to be pathogenic^22^. A mutation was defined as having a 5.0% allele frequency or greater, and TMB was calculated by dividing the number of mutations by the sequence length of the comprehensive cancer panel (1.23 Mb). Public dataset of the TCGA study (BLCA) were obtained from cBioPortal (www.cbioportal.org), and the frequency of mutation in *TP53*, *ATM*, *ERCC*, *RB1*, *DPYD*, *FANCC* genes was analyzed^23,24^.

### *DYPD* mRNA expression analysis in MIBC tissues

Microarray data (GSE87304) from a multi-institutional cohort of 223 MIBC patients treated with NAC followed by radical cystectomy were used^13^. GC or Gcarbo was administered in 169 patients, and MVAC in 54 patients. Molecular subtypes (basal, claudin-low, luminal, and luminal-infiltrated) were previously reported in this cohort^13^. Patients were divided into *DPYD* high and *DPYD* low groups based on mRNA expression levels. The number of genes analyzed was 46,048 Gene set enrichment analysis (GSEA 4.1.0) was performed with gene sets database of h.all.v7.5.symbols.gmt [Hallmarks], and chip platform of Human_AFFY_HG_U133_MSigDB.v7.5.chip. (https://www.gsea-msigdb.org/gsea/index.jsp)^25^. Public dataset of the TCGA study (BLCA) were obtained from cBioPortal (www.cbioportal.org), and the gene expression of *DPYD* was analyzed^23,24^.

### Immunohistochemistry

TUR sections were deparaffinized and permeabilized with xylene, then activated with 0.01 M citric acid for 10 min. Blocking with 3% hydrogen peroxide for 15 min and Blocking-One (Nacalai Tesque, Kyoto, Japan) for 30 min was performed. Anti-dihydropyrimidine dehydrogenase (DPD) antibody (1:50, ab54797; Abcam, Cambridge, UK) was diluted with Blocking-One and used as the primary antibody with 90 min incubation. Histofine Simple Stain MAX-PO (MULTI) (Nichirei Bioscience Inc, Tokyo, Japan) was used as the secondary antibody with 45 min incubation. The signals were developed by DAB tablet (Fujifilm, Tokyo, Japan) for 20 min, followed by counterstaining with Histofine Mayer’s hematoxylin (415,081; Nichirei Bioscience) for 2 s^26^. After dehydration, sections were encapsulated and observed.

### Cell culture

RT112 and UM-UC-13 human bladder cancer cell lines were provided by the Pathology Core of Bladder Cancer SPORE at MD Anderson Cancer Center, and cultured in MEM/EBSS (Hyclone, GE Healthcare, Chicago, IL, USA) containing 0.1 mM non-essential amino acids, 1 mM sodium pyruvate, 10% fetal bovine serum (FBS), and 1% penicillin/streptomycin^27^. To establish gemcitabine-resistant sublines (RT112 Gem-R and UT-UC-13 Gem-R), RT112 and UM-UC-13 cells were treated serially with increasing concentrations of gemcitabine up to 10 μM and 0.1 μM, respectively. HEK293 cells were cultured in Dulbecco’s modified Eagle’s medium (DMEM; Sigma–Aldrich, St. Louis, MO, USA) supplemented with 10% FBS and 1% penicillin/streptomycin.

### Plasmids and siRNA

C-terminal FLAG-tagged *DPYD* plasmid in the backbone of pcDNA3.1 was obtained from GenScript (cloneID: Ohu19551; GenScript Biotech, Piscataway, NJ, USA). To establish *DPYD* c.1031C > T plasmid, the PrimeSTAR Mutagenesis Basal Kit (Takara, Kusatsu, Japan) was used. The primers, annealing temperature (Tm), and cycle number of mutagenesis reactions were as follows: 5’-GACACTGTCTTTGACTGTGCAACATCT-3’ and 5’-GTCAAAGACAGTGTCTCCAGCTCCAAG-3’, 55 °C, and 30 cycles. Correct introduction of mutations was confirmed by Sanger sequencing. Specific siRNAs against *DPYD* (#1: SASI_Hs01_0018-0530/*DPYD* and #2: SASI_01_0018-0539/*DPYD*) and control siRNA (MISSION siRNA Universal Negative Control #1, SCI001) were obtained from Sigma–Aldrich. Transfection of plasmids and siRNAs into cells was performed using Lipofectamine LTX (Invitrogen, Waltham, MA, USA) and Lipofectamine RNAiMAX (Invitrogen), respectively, in accordance with the manufacturer’s instructions^28^. Stable overexpressing cells were established by transfecting expression plasmid into HEK293 cells as described above, and culturing with medium containing G418 (Nacalai tesque) at 400 μg/mL for at least 2 weeks for selection.

### Cell proliferation assay

Cell proliferation was calculated by cell counting or CellTiter-Glo Luminescent Cell Viability Assay (Promega, Madison, WI, USA). Cell counting was performed using a TC20 Automated Cell Counter (Bio-Rad, Hercules, CA, USA) after incubation in the indicated conditions. For CellTiter-Glo assay, cells were seeded in a 96-well plate with phenol-red free medium and cultured under the indicated conditions. CellTiter reagents were added to the medium and incubated for 10 min, followed by measurement using a 2030 ARVO X2 (PerkinElmer, Waltham, MA, USA)^29^. Cell survival rate when exposed to gemcitabine was corrected with that in the absence of gemcitabine. The proliferation ratio was calculated using the formula below. A proliferation ratio of > 1.0 indicated resistance and that of < 1.0 indicated sensitivity.

$${\mathrm{Proliferation\, ratio}}=\frac{survival \,rate\, (transfected \,cell)}{survival \,rate\, (empty-transfected \,cell)}$$

All values represent the results of three independent experiments.

### Western blot analysis

Cells and tumor tissues were lysed in RIPA buffer (50 mM Tris–HCl, pH 7.5, 1 mM EDTA, 150 mM NaCl, 0.5% NP40). For tissue lysis, 0.1% SDS was added with protease inhibitor cocktail set 1 (Fujifilm). Protein samples were separated by SDS-PAGE gel and transferred onto PVDF membranes. After 30 min blocking with Blocking-One (Nacalai Tesque), membranes were incubated with primary antibody overnight at 4 °C. The following antibodies were used: anti-DPD antibody (1:10,000, ab54797; Abcam), anti-GAPDH antibody (1:10,000, 14C10; Cell Signaling Technology, Danvers, MA, USA), and anti-α-tubulin antibody (1:10,000, PM054; MBL, Tokyo, Japan). Then membranes were incubated with secondary antibody (anti-mouse IgG, 1:20,000 [NA9310V, GE Healthcare]; anti-rabbit IgG, 1:20,000 [Jackson ImmunoResearch, West Grove, PA, USA]) for 1 h at room temperature. Each antibody was diluted in 1 × PBST. ECL substrates (Clarity Western ECL substrate; Bio-Rad) were used for signal detection with an electronically cooled charge-coupled device camera (LAS4000; GE Healthcare). Quantification of protein expression was performed with imageQuant TL (GE Healthcare)^30^. As internal controls, GAPDH or α-tubulin was used for cells, and total protein was used for patients’ tissue samples. Total protein was detected by staining membranes with CBB-R (CBB Stain One Super, 11642-31; Nacalai Tesque) for 15 min.

### Quantitative real-time PCR (qRT-PCR)

RNA from cells was extracted with the RNeasy Mini kit (Qiagen). After treatment with DNase I (Qiagen), reverse transcription with 500 ng RNA using a PrimeScript RT Reagent Kit (Takara) was performed, and cDNA was stored at − 80 °C. mRNA expression was calculated using SYBR Premix ExTaq II (Takara) with a thermal cycler (StepOne plus; Applied Biosystems, Waltham, MA, USA)^31^. Primers, Tm, and cycle numbers were as follows: *DPYD*; 5’-CAACGTAGAGCAGATGTTGCAC-3’ and 5’-GAGCTGTCATGCAGAAATGGTTT-3’, *18 s ribosomal RNA* (internal control); 5’-AAACGGCTACCACATCCAAG-3’ and 5’-CCTCCAATGGATCCTCGTTA-3’, 60 °C, 40 cycles. All values represent the results of three independent experiments.

### Plasma metabolite preparation

Metabolite extraction from cultured cells was described previously^32^. To prepare nucleic acid-related metabolites, approximately 3 × 10^6^ HEK293 cell pellets were added to 500 µL ice-cold 80% methanol, vortexed, sonicated five times (30-s sonication and 30-s cooling) with a BIORUPTOR (Cosmo Bio, Tokyo, Japan), and centrifuged at 21,500×g for 5 min at 4 °C. The supernatants were collected, and 250 µL supernatant was evaporated to dryness, then dissolved in a mobile phase determined by each analytical condition and then subjected to LCMS measurement of nucleotides or nucleosides.

### Liquid chromatography-mass spectrometry

Cellular metabolites were analyzed by liquid chromatography-mass spectrometry using an LCMS-8060 instrument (Shimadzu, Kyoto, Japan). For nucleotides, the prepared sample was separated on a SeQuant® Zic®-pHILIC column (150 × 2.1 mm, 5 μm particle size; Merck, Darmstadt, Germany) with mobile phases consisting of solvent A (10 mM ammonium bicarbonate, 0.1% ammonia, 100 μM medronate)^33^ and solvent B (acetonitrile). The column oven temperature was maintained at 40 °C. The gradient elution program was as follows: flow rate of 0.25 mL/min; 0–2 min, 70% B; 0–1 min, 70–47.5% B; 1–1.5 min, 47.5–30% B; 1.5–3.5 min, 30% B; 3.5–6.5 min, 70% B; and 6.6–12.5 min. The parameters for the heated electrospray ionization source (ESI) in negative ion mode under multiple reaction monitoring (MRM) were as follows: drying gas flow rate, 10 L/min; nebulizer gas flow rate, 3 L/min; heating gas flow rate, 10 L/min; interface temperature, 300 °C; DL temperature, 250 °C; heat block temperature, 400 °C; CID gas, 270 kPa. However, for nucleosides, the sample was separated on a KINETEX C18 column (150 mm × 2.1 mm, 1.7 μm; Phenomenex) with mobile phases consisting of solvent A (0.1% formic acid) and solvent B (0.1% formic acid in acetonitrile). The column oven temperature was 35 °C. The gradient elution program was as follows: flow rate of 0.3 mL/min; 0–2 min, 3% B; 2–5.5 min, 3–6% B; 5.8–8.5 min, 100% B; and 8.6–15 min, 3% B. The parameters for the heated ESI in positive ion mode under multiple reaction monitoring (MRM) were the same as above. Data processing was performed using the LabSolutions LC–MS software program (Shimadzu).

### Statistical analysis

All statistical analyses were performed using EZR software (Saitama Medical Center, Jichi Medical University, Saitama, Japan), a graphical user interface for R 2.13.0^34^. Statistical analyses were performed using Fisher test, Student’s t-test, one-way ANOVA, and Mann–Whitney U test. All *P*-values are two-sided. Levels of statistical significance were set at *P* < 0.05.

### Ethical compliance

The study was approved by Kyushu University Institutional Review Board for Clinical Research (#2020-254).

## Supplementary Information

Supplementary Table S1.

Supplementary Table S2.

Supplementary Figure S1.

Supplementary Figure S2.

Supplementary Figure S3.

Supplementary Table S3.

Supplementary Figure S4.

Supplementary Figure S5.

Supplementary Figure S6.

Supplementary Table S4.

Supplementary Information 11.

## Data Availability

The authors declare that the sequencing data of 20 patients of this study are available within the paper and its Supplementary Information files. The microarray data that support the findings of this study are available from University of British Columbia, which were used under license for the current study, and so are not publicly available. Data are however available from the authors upon reasonable request and with permission of University of British Columbia. Mutation data in TCGA data that support the findings of this study have been deposited in cBioportal with https://pubmed.ncbi.nlm.nih.gov/28988769/.
