# Supplementary material for: Cancer genomic profiling identified dihydropyrimidine dehydrogenase deficiency in bladder cancer promotes sensitivity to gemcitabine
Source: Sci Rep. 2022 May 20;12:8535. doi: 10.1038/s41598-022-12528-3 (PMC9122908; doi:10.1038/s41598-022-12528-3)
Supplement: Supplementary file 1 — Supplementary Table S1. [file 41598_2022_12528_MOESM1_ESM.pdf]

Supplementary Table S1. Clinicopathological informations in 20 cases with mucle invasive bladder cancer

Case No.	Diagnosis	Age, years	Gender	Clinical T-stage	Clinical N-stage	Metastasis	Preoperative chemotherapy	RECIST	2nd-line chemotherapy	RECIST	Radical therapy	Pathological T-stage	Pathological N-stage	Adjuvant chemotherap	Recurrence (day from radical thera	Metastasis	Salvage/primery chemotherap	RECIST	Response to GC/GCarbo	Survival	Remark
2	MIBC	68	M	T2	0	-	-	-	-	-	Radical cystectomy	pT3a	0	-	-	-	-		-	Alive	
3	MIBC	77	M	T3	0	-	-	-	-	-	Radical cystectomy	pT3b+is	0	-	107	Lung, liver, bone	GCarbo	PD	Poor	Dead	
4	MIBC	42	M	T3	0	Lung	GC	CR	-	-	Radical cystectomy	pT0	0	-	-	-	-		Good	Alive	*1
5	MIBC	53	M	T3	0	-	GC	-	-	-	Radical cystectomy	pT0	0	GC	-	-	-		Good	Alive	
6	MIBC	63	M	T3	0	-	-	-	-	-	-	-	-	-	-	-	GC	PD	Poor	Dead	
8	MIBC	81	M	T3	0	-	-	-	-	-	Radiation	-	-	-	-	-	-		-	Dead	
9	MIBC	91	M	T2	0	-	-	-	-	-	Radical cystectomy	pT4+is	0	-	218	Non-regional lymph node	-		-	Dead	
11	MIBC	70	M	T3	0	-	MVAC	-	-	-	Radical cystectomy	pT3+is	0	-	-	-	-		-	Alive	
12	MIBC	72	M	T1	0	-	-	-	-	-	Partial cystectomy	pT3+is	0	-	-	-	-		-	Alive	*2
13	MIBC	70	M	T2	0	-	GC	-	-	-	Radical cystectomy	pT0	0	-	-	-	-		Good	Alive	
14	MIBC	79	F	T2	0	-	GC	-	-	-	Radical cystectomy	pT3b+is	0	-	88	Peritoneum, bone	-		Poor	Dead	
16	MIBC	61	F	T3	1	-	GCarbo	PD	Pembrolizumab	PR	Radical cystectomy	pT0	3	Pembrolizumab	-	-	-		Poor	Alive	*3
17	MIBC	74	F	T2	0	Lung, liver	-	-	-	-	-	-	-	-	-	-	-		-	Dead	
18	MIBC	56	M	T3	0	-	GC	-	-	-	Radical cystectomy	pT3a	0	-	328	Peritoneum	Pembrolizumab		Poor	Alive	
21	MIBC	63	M	T3	1	Non-regional lymph node	-	-	-	-	-	-	-	-	-	-	GC	SD	Poor	Alive	
24	MIBC	73	F	T2	0	-	GC	-	-	-	Radical cystectomy	pT0	0	GC	-	-	-		Good	Alive	
25	MIBC	82	F	T1+is	0	-	-	-	-	-	Radical cystectomy	pT3+is	1	-	154	Non-regional lymph node	GC	PD	Poor	Dead	*4
27	MIBC	71	M	T3	0	-	GC	-	-	-	Radical cystectomy	pT3b+is	1	-	-	-	-		Poor	Alive	
28	MIBC	73	M	T3	0	-	-	-	-	-	Radical cystectomy	pT4a	0	-	-	-	-		-	Alive	
29	MIBC	51	F	T2	1	-	GC	-	-	-	Radical cystectomy	pT2b+is	2	-	-	-	-		Poor	Alive	

*1, Radical cystectomy was performed after distant disease disappeared by GC.
*2, Partial cystectomy was performed for bladder cancer in diverticulum.
*3, Pembrolizumab was administered as 2nd-line chemotherapy since GCarbo response was poor.
*4, Radical cystectomy was performed for BCG-refractory bladder cancer.
