# Supplementary material for: Cancer genomic profiling identified dihydropyrimidine dehydrogenase deficiency in bladder cancer promotes sensitivity to gemcitabine
Source: Sci Rep. 2022 May 20;12:8535. doi: 10.1038/s41598-022-12528-3 (PMC9122908; doi:10.1038/s41598-022-12528-3)
Supplement: Supplementary file 2 — Supplementary Table S2. [file 41598_2022_12528_MOESM2_ESM.pdf]

Supplementary Table S2. Mutated genes detected in 20 cases with muscle invasive bladder cancer

Case No.	Gene	Gene location	Reference	Genotype	Transcript	Exon	AA Change	Function	Coverage	Variant allele frequency
Pt02	PDE4DIP	chr1:144855812	T	T/C	NM_001198834.3	41	p.Ser2247=	synonymous	156	12.18
Pt02	PDE4DIP	chr1:144882730	T	T/G	NM_001198834.3	24	p.Ser1097Arg	missense	361	5.54
Pt02	MDM4	chr1:204512101	T	T/C	NM_002393.5	-	-	unknown	135	5.93
Pt02	TAF1L	chr9:32633325	G	G/A	NM_153809.2	1	p.Gly751=	synonymous	140	6.43
Pt02	FANCC	chr9:98002860	A	A/T	NM_000136.3	-	-	unknown	100	6
Pt02	NUP98	chr11:3740589	A	A/T	NM_016320.5	-	-	unknown	70	5.71
Pt02	ERCC5	chr13:103527850	G	G/A	NM_000123.3	15	p.Gly1053Glu	missense	251	6.37
Pt02	PML	chr15:74336985	G	G/C	NM_033238.3	9	p.Gly762Ala	missense	94	5.32
Pt02	TP53	chr17:7578459	GACGCGGGT	GACGCGGGT/GACGCCGGT	NM_000546.5	5	p.Arg156Gly	missense	81	59.26
Pt02	TP53	chr17:7578504	AGGGC	AGGGC/AGGGT	NM_000546.5	5	p.Cys141Tyr	missense	96	32.29
Pt03	SDHB	chr1:17380497	G	T/T	NM_003000.3	1	p.Ala6=	synonymous	179	100
Pt03	TRIM33	chr1:114940209	G	G/T	NM_015906.4	20	-	unknown	51	49.02
Pt03	TRIM33	chr1:114948281	A	A/G	NM_015906.4	15	p.Ile840Thr	missense	83	60.24
Pt03	PDE4DIP	chr1:144854581	T	T/C	NM_001198834.4	42	p.Thr2297Ala	missense	179	23.46
Pt03	PDE4DIP	chr1:144854594	G	G/T	NM_001198834.4	42	p.Leu2292=	synonymous	177	23.73
Pt03	PDE4DIP	chr1:144854597	TC	CC/CT	NM_001198834.4	42	p.Arg2291Gln	missense	174	CC=39.08,CT=41.38
Pt03	PDE4DIP	chr1:144857068	A	A/T	NM_001198834.4	-	-	unknown	71	49.3
Pt03	PDE4DIP	chr1:144874815	T	T/C	NM_001198834.4	30	p.His1598Arg	missense	308	45.13
Pt03	PDE4DIP	chr1:144922583	G	G/A	NM_001198834.4	7	p.Ser275Leu	missense	358	47.49
Pt03	ITGA10	chr1:145542281	G	G/A	NM_001198834.4	-	-	unknown	188	43.62
Pt03	MCL1	chr1:150551995	G	G/C	NM_021960.5	1	p.Leu4=	synonymous	49	18.37
Pt03	SH2D2A	chr1:156785617	G	A/A	NM_001161441.1	-	-	unknown	104	100
Pt03	DDR2	chr1:162743418	G	T/T	NM_006182.4	-	-	unknown	268	100
Pt03	PBX1	chr1:164529120	G	G/A	NM_002585.4	1	p.Gly21Ser	missense	6	66.67
Pt03	PBX1	chr1:164769086	G	G/A	NM_002585.4	4	p.Glu221Lys	missense	171	8.19
Pt03	TPR	chr1:186313108	C	C/T	NM_003292.3	26	p.Val1178Ile	missense	156	42.31
Pt03	PIK3C2B	chr1:204425028	G	G/A	NM_002646.4	13	p.Phe633=	synonymous	15	40
Pt03	PIK3C2B	chr1:204438643	G	T/T	NM_002646.4	4	p.Leu96=	synonymous	146	100
Pt03	MDM4	chr1:204501383	C	C/T	NM_002393.5	-	-	unknown	200	39.5
Pt03	MDM4	chr1:204512101	T	T/C	NM_002393.5	-	-	unknown	122	7.38
Pt03	MDM4	chr1:204515863	C	T/T	NM_002393.5	-	-	unknown	148	100
Pt03	MTR	chr1:236988593	A	AT/AT	NM_000254.2	-	-	unknown	112	100
Pt03	NCOA1	chr2:24964587	A	A/G	NM_003743.5	-	-	unknown	130	12.31
Pt03	ALK	chr2:29432776	T	T/C	NM_004304.5	-	-	unknown	89	58.43

Pt03	ALK	chr2:29543663	T	C/C	NM_004304.5	7	p.Gln500=	synonymous	123	100
Pt03	ALK	chr2:29940529	A	T/T	NM_004304.5	2	p.Pro234=	synonymous	73	100
Pt03	EML4	chr2:42515388	A	A/G	NM_019063.5	11	p.Ile382Val	missense	74	66.22
Pt03	MSH6	chr2:48010488	G	G/A	NM_000179.3	1	p.Gly39Glu	missense	118	60.17
Pt03	XPO1	chr2:61749690	C	C/T	NM_003400.4	-	-	unknown	78	41.03
Pt03	XPO1	chr2:61749716	C	C/T	NM_003400.4	-	-	unknown	78	41.03
Pt03	AFF3	chr2:100343557	C	T/T	NM_001025108.2	10	p.Ser383Asn	missense	100	100
Pt03	LRP1B	chr2:141122209	G	G/C	NM_018557.3	-	-	unknown	138	9.42
Pt03	LRP1B	chr2:141283603	T	G/G	NM_018557.3	-	-	unknown	124	100
Pt03	LRP1B	chr2:141528592	T	C/C	NM_018557.3	-	-	unknown	163	100
Pt03	LRP1B	chr2:141945949	C	T/T	NM_018557.3	-	-	unknown	143	100
Pt03	LRP1B	chr2:142012036	T	T/G	NM_018557.3	-	-	unknown	97	41.24
Pt03	SF3B1	chr2:198267770	G	G/GAA	NM_012433.4	-	-	unknown	97	50.52
Pt03	ERBB4	chr2:212812097	T	T/C	NM_005235.3	-	-	unknown	94	29.79
Pt03	FN1	chr2:216235089	C	T/T	NM_212482.3	41	p.Val2261Ile	missense	59	100
Pt03	FN1	chr2:216242917	T	A/A	NM_212482.3	35	p.Gly1897=	synonymous	86	100
Pt03	FN1	chr2:216251697	G	G/A	NM_212482.3	-	-	unknown	129	95.35
Pt03	FN1	chr2:216272908	G	G/T	NM_212482.3	17	p.Pro814His	missense	98	10.2
Pt03	FN1	chr2:216274853	AACAC	AAC/A	NM_212482.3	-	-	unknown	116	AAC=91.38,A=6.90
Pt03	FN1	chr2:216283958	T	A/A	NM_212482.3	-	-	unknown	123	100
Pt03	STK36	chr2:219563504	C	C/T	NM_015690.5	26	p.Ser1079=	synonymous	63	44.44
Pt03	PAX3	chr2:223065870	C	C/G	NM_181459.4	10	-	unknown	119	49.58
Pt03	PAX3	chr2:223161625	C	C/G	NM_181459.4	-	-	unknown	81	7.41
Pt03	FANCD2	chr3:10114947	A	A/G	NM_033084.6	28	p.Gln872=	synonymous	207	7.73
Pt03	VHL	chr3:10188341	A	A/T	NM_000551.4	-	-	unknown	126	7.94
Pt03	XPC	chr3:14187449	G	G/T	NM_004628.5	16	p.Gln939Lys	missense	86	53.49
Pt03	TGFBR2	chr3:30686414	A	A/G	NM_001024847.2	-	-	unknown	76	55.26
Pt03	LTF	chr3:46490474	G	G/A	NM_002343.6	9	p.Val364=	synonymous	76	43.42
Pt03	SETD2	chr3:47165053	A	A/T	NM_014159.6	3	p.Leu358Ter	nonsense	128	5.47
Pt03	PBRM1	chr3:52584715	A	A/G	NM_018313.5	-	-	unknown	137	56.2
Pt03	PBRM1	chr3:52584787	T	T/C	NM_018313.5	28	p.Pro1445=	synonymous	140	57.86
Pt03	PBRM1	chr3:52676065	C	C/T	NM_018313.5	-	-	unknown	40	45
Pt03	MAGI1	chr3:65425560	T	T/TCTG	NM_001033057.2	9	p.Gln421dup	nonframeshiftInsertion	55	23.64
Pt03	MAGI1	chr3:65607592	T	T/TCA	NM_001033057.2	-	-	unknown	61	A=6.56,TCA=68.85
Pt03	MAGI1	chr3:65607637	A	G/G	NM_001033057.2	-	-	unknown	69	100
Pt03	GATA2	chr3:128204951	C	C/T	NM_032638.5	3	p.Ala164Thr	missense	191	41.36
Pt03	GATA2	chr3:128205860	G	G/C	NM_032638.5	2	p.Pro5=	synonymous	60	38.33
Pt03	EPHB1	chr3:134968166	C	C/G	NM_004441.5	-	-	unknown	146	12.33
Pt03	PIK3CA	chr3:178942431	C	C/G	NM_006218.4	-	-	unknown	77	64.94
Pt03	LPP	chr3:188327461	C	C/T	NM_005578.5	6	p.Asp314=	synonymous	153	45.1
Pt03	LPP	chr3:188590446	A	G/G	NM_005578.5	10	p.Arg535=	synonymous	144	100

Pt03	TNK2	chr3:195595632	A	G/G	NM_001010938.2	-	-	unknown	163	100
Pt03	TNK2	chr3:195605929	G	G/A	NM_001010938.2	7	p.Asn359=	synonymous	104	56.73
Pt03	PDGFRA	chr4:55161391	T	C/C	NM_006206.6	23	p.Asp1074=	synonymous	110	100
Pt03	KDR	chr4:55972974	T	T/A	NM_002253.3	11	p.Gln472His	missense	160	51.88
Pt03	ADGRL3	chr4:62800554	G	G/A	NM_015236.6	-	-	unknown	153	44.44
Pt03	AFF1	chr4:88053085	C	C/T	NM_001166693.2	-	-	unknown	128	38.28
Pt03	MTRR	chr5:7869235	T	T/C	NM_024091.4	-	-	unknown	127	58.27
Pt03	MTRR	chr5:7870808	G	G/C	NM_024010.4	-	-	unknown	216	5.09
Pt03	MTRR	chr5:7878192	T	T/C	NM_024010.4	5	p.Leu179=	synonymous	147	60.54
Pt03	IL7R	chr5:35861068	T	T/C	NM_002185.5	2	p.Ile66Thr	missense	135	45.93
Pt03	IL7R	chr5:35871190	G	G/A	NM_002185.5	4	p.Val138Ile	missense	140	16.43
Pt03	LIFR	chr5:38493661	T	T/A	NM_002310.6	-	-	unknown	142	11.27
Pt03	LIFR	chr5:38528977	C	A/T	NM_002310.6	-	-	unknown	160	A=10.63,T=80.63
Pt03	LIFR	chr5:38528979	C	C/A	NM_002310.6	-	-	unknown	156	83.97
Pt03	LIFR	chr5:38528981	G	A/C	NM_002310.6	-	-	unknown	156	A=3.85,C=95.51
Pt03	RAD50	chr5:131892979	G	A/A	NM_005732.4	1	-	unknown	127	100
Pt03	CSF1R	chr5:149456811	G	G/A	NM_005211.3	-	-	unknown	177	33.33
Pt03	FLT4	chr5:180048056	C	C/T	NM_182925.5	-	-	unknown	69	98.55
Pt03	FLT4	chr5:180053090	G	A/A	NM_182925.5	-	-	unknown	88	100
Pt03	FLT4	chr5:180053097	C	T/T	NM_182925.5	-	-	unknown	88	100
Pt03	POU5F1	chr6:31133663	T	C/C	NM_002701.6	-	-	unknown	56	100
Pt03	POU5F1	chr6:31138371	G	G/A	NM_002701.6	1	p.Phe9=	synonymous	38	50
Pt03	POU5F1	chr6:31138377	C	C/T	NM_002701.6	1	p.Ser7=	synonymous	38	50
Pt03	NOTCH4	chr6:32190484	G	G/A	NM_004557.4	3	p.Pro85=	synonymous	96	41.67
Pt03	PKHD1	chr6:51524403	G	G/A	NM_138694.4	61	p.His3507=	synonymous	114	51.75
Pt03	PKHD1	chr6:51720838	T	C/C	NM_138694.4	49	p.Leu2588=	synonymous	151	99.34
Pt03	PKHD1	chr6:51720872	A	G/G	NM_138694.4	-	-	unknown	150	99.33
Pt03	PKHD1	chr6:51910905	T	T/C	NM_138694.4	24	p.Asn830Ser	missense	245	58.37
Pt03	DST	chr6:56351972	G	G/C	NM_001144769.5	81	p.Leu4874Val	missense	166	57.23
Pt03	DST	chr6:56359034	C	C/A	NM_001144769.5	-	-	unknown	127	40.94
Pt03	DST	chr6:56374754	C	C/A	NM_001144769.5	-	-	unknown	77	49.35
Pt03	DST	chr6:56417282	C	T/T	NM_001144769.5	55	p.Met3317Ile	missense	206	100
Pt03	DST	chr6:56420538	C	C/T	NM_001144769.5	54	p.Arg2795His	missense	139	39.57
Pt03	DST	chr6:56462687	C	C/G	NM_001144769.5	41	p.Glu1897Gln	missense	133	12.78
Pt03	ADGRB3	chr6:69759148	A	T/T	NM_001704.3	-	-	unknown	32	100
Pt03	EPHA7	chr6:93982124	A	G/G	NM_004440.4	6	p.Ser447=	synonymous	140	100
Pt03	EPHA7	chr6:94124530	C	A/A	NM_004440.4	-	-	unknown	83	100
Pt03	ROS1	chr6:117678083	A	A/G	NM_002944.2	-	-	unknown	88	51.14
Pt03	TNFAIP3	chr6:138195961	TCTC	T/T	NM_001270507.2	-	-	unknown	15	100
Pt03	ESR1	chr6:152382087	C	C/CT	NM_001122740.1	-	-	unknown	128	29.69
Pt03	SYNE1	chr6:152443756	C	C/T	NM_182961.4	146	p.Gly8737Ser	missense	154	44.16

Pt03	SYNE1	chr6:152461048	CTGTT	CTGTT/C	NM_182961.4	-	-	unknown	97	58.76
Pt03	SYNE1	chr6:152461061	G	G/A	NM_182961.4	-	-	unknown	100	59
Pt03	SYNE1	chr6:152542548	A	G/G	NM_182961.4	-	-	unknown	75	100
Pt03	SYNE1	chr6:152629621	C	C/T	NM_182961.4	-	-	unknown	121	9.09
Pt03	SYNE1	chr6:152647681	A	T/T	NM_182961.4	79	p.Leu5015Met	missense	155	99.35
Pt03	SYNE1	chr6:152652034	A	T/T	NM_182961.4	78	p.Ser4596Thr	missense	157	100
Pt03	SYNE1	chr6:152675854	A	A/G	NM_182961.4	67	p.Ser3622=	synonymous	188	35.11
Pt03	SYNE1	chr6:152690559	G	G/A	NM_182961.4	-	-	unknown	73	27.4
Pt03	SYNE1	chr6:152708310	G	G/A	NM_182961.4	54	p.Ala2795Val	missense	91	70.33
Pt03	SYNE1	chr6:152712752	C	C/T	NM_182961.4	-	-	unknown	120	53.33
Pt03	IGF2R	chr6:160453561	T	T/G	NM_000876.3	-	-	unknown	132	37.88
Pt03	IGF2R	chr6:160464289	G	G/A	NM_000876.3	12	p.Gly530=	synonymous	121	38.02
Pt03	IGF2R	chr6:160468180	G	G/A	NM_000876.3	-	-	unknown	155	65.81
Pt03	IGF2R	chr6:160468278	A	G/G	NM_000876.3	16	p.Thr713=	synonymous	156	100
Pt03	IGF2R	chr6:160493834	G	G/A	NM_000876.3	33	p.Ala1536=	synonymous	141	60.28
Pt03	IGF2R	chr6:160494521	C	C/T	NM_000876.3	-	-	unknown	95	41.05
Pt03	CARD11	chr7:2946461	T	T/C	NM_032415.6	25	p.Arg1092=	synonymous	42	52.38
Pt03	CARD11	chr7:2946463	G	G/C	NM_032415.6	25	p.Arg1092Gly	missense	44	6.82
Pt03	CARD11	chr7:2966445	T	G/G	NM_032415.6	-	-	unknown	81	100
Pt03	ETV1	chr7:14025766	T	A/A	NM_001163147.1	-	-	unknown	112	100
Pt03	EGFR	chr7:55227945	A	A/G	NM_005228.5	12	p.Tyr471Cys	missense	206	9.22
Pt03	EGFR	chr7:55229255	G	A/A	NM_005228.5	13	p.Arg521Lys	missense	164	100
Pt03	AKAP9	chr7:91667692	T	T/G	NM_005751.4	-	-	unknown	153	33.33
Pt03	AKAP9	chr7:91714923	G	G/C	NM_005751.4	36	p.Glu2983Gln	missense	221	7.69
Pt03	AKAP9	chr7:91726927	A	A/C	NM_005751.4	42	p.Arg3476=	synonymous	121	32.23
Pt03	TRRAP	chr7:98558880	G	G/C	NM_001244580.1	-	-	unknown	160	43.13
Pt03	EPHB4	chr7:100401270	T	G/G	NM_004444.5	-	-	unknown	140	100
Pt03	PIK3CG	chr7:106513011	C	C/T	NM_002649.3	3	p.Ser675=	synonymous	185	57.3
Pt03	POT1	chr7:124481245	G	G/A	NM_015450.3	-	-	unknown	100	45
Pt03	TRIM24	chr7:138239387	G	G/A	NM_015905.3	-	-	unknown	144	13.89
Pt03	EPHB6	chr7:142562052	C	C/T	NM_004445.6	7	p.Pro165Leu	missense	58	20.69
Pt03	KMT2C	chr7:151882672	C	C/A	NM_170606.3	34	p.Ala1685Ser	missense	145	5.52
Pt03	KMT2C	chr7:151882735	G	G/A	NM_170606.3	-	-	unknown	148	5.41
Pt03	KMT2C	chr7:151902343	G	G/A	NM_170606.3	-	-	unknown	110	32.73
Pt03	KMT2C	chr7:151962257	C	C/T	NM_170606.3	8	p.Pro350=	synonymous	476	5.67
Pt03	KMT2C	chr7:151962265	C	C/T	NM_170606.3	8	p.Asp348Asn	missense	473	5.71
Pt03	KMT2C	chr7:151970951	C	C/T	NM_170606.3	7	p.Arg284Gln	missense	218	6.88
Pt03	ADGRA2	chr8:37686749	A	A/G	NM_032777.10	-	-	unknown	131	47.33
Pt03	ADGRA2	chr8:37693302	T	T/C	NM_032777.10	-	-	unknown	60	98.33
Pt03	ADGRA2	chr8:37693347	C	C/A	NM_032777.10	-	-	unknown	47	51.06
Pt03	PRKDC	chr8:48751781	T	T/C	NM_006904.7	56	-	unknown	181	17.68

Pt03	NBN	chr8:90995019	C	C/T	NM_002485.5	2	p.Leu34=	synonymous	212	51.89
Pt03	UBR5	chr8:103372743	G	A/A	NM_015902.6	-	-	unknown	190	100
Pt03	CSMD3	chr8:113308243	A	A/G	NM_198123.2	-	-	unknown	76	52.63
Pt03	CSMD3	chr8:113363511	T	T/C	NM_198123.2	-	-	unknown	48	60.42
Pt03	MYC	chr8:128750964	C	C/T	NM_002467.6	2	p.Tyr167=	synonymous	92	28.26
Pt03	RECQL4	chr8:145738584	G	G/T	NM_004260.4	-	-	unknown	13	30.77
Pt03	RECQL4	chr8:145741765	G	G/A	NM_004260.4	5	p.Ser246=	synonymous	129	54.26
Pt03	RECQL4	chr8:145742514	A	G/G	NM_004260.4	4	p.Ser92Pro	missense	82	100
Pt03	PTPRD	chr9:8331574	A	A/AAACTTACCATTCCTGAACTGT	NM_002839.4	-	-	unknown	121	41.32
Pt03	PTPRD	chr9:8436703	C	C/G	NM_002839.4	-	-	unknown	89	38.2
Pt03	PTPRD	chr9:8518052	G	G/C	NM_002839.4	21	p.Gln447Glu	missense	152	39.47
Pt03	CDKN2A	chr9:21968712	C	C/A	NM_001195132.1	3	-	unknown	85	51.76
Pt03	TAF1L	chr9:32633325	G	G/A	NM_153809.2	1	p.Gly751=	synonymous	89	5.62
Pt03	SYK	chr9:93641175	C	C/T	NM_003177.7	11	p.Tyr507=	synonymous	142	59.86
Pt03	SYK	chr9:93641199	T	T/C	NM_003177.7	11	p.Leu515=	synonymous	143	60.14
Pt03	ABL1	chr9:133761001	A	G/G	NM_005157.6	11	p.Pro1108=	synonymous	141	100
Pt03	NUP214	chr9:134003900	A	A/G	NM_005085.4	-	-	unknown	148	25
Pt03	NUP214	chr9:134103759	G	G/A	NM_005085.4	-	-	unknown	113	41.59
Pt03	TSC1	chr9:135782221	T	T/C	NM_000368.5	14	p.Glu445=	synonymous	112	33.93
Pt03	NOTCH1	chr9:139391636	G	A/A	NM_017617.5	34	p.Asp2185=	synonymous	108	100
Pt03	NOTCH1	chr9:139397707	G	A/A	NM_017617.5	27	p.Asp1698=	synonymous	92	100
Pt03	NOTCH1	chr9:139405261	C	T/T	NM_017617.5	-	-	unknown	107	100
Pt03	NOTCH1	chr9:139411880	G	A/A	NM_017617.5	-	-	unknown	48	100
Pt03	MLLT10	chr10:21977439	A	A/C	NM_001195626.3	-	-	unknown	193	56.99
Pt03	RET	chr10:43610119	G	G/A	NM_020975.6	11	p.Gly691Ser	missense	154	51.3
Pt03	TET1	chr10:70332580	A	A/G	NM_030625.3	2	p.Asp162Gly	missense	191	48.69
Pt03	FAS	chr10:90771829	T	C/C	NM_000043.6	7	p.Thr214=	synonymous	131	100
Pt03	CYP2C19	chr10:96580439	TG	TG/T	NM_000769.4	-	-	unknown	104	26.92
Pt03	BLNK	chr10:97964261	C	C/G	NM_013314.4	-	-	unknown	162	56.79
Pt03	BLNK	chr10:97990491	C	C/T	NM_013314.4	-	-	unknown	84	59.52
Pt03	BLNK	chr10:97990583	A	A/G	NM_013314.4	4	p.Pro57=	synonymous	71	30.99
Pt03	SUFU	chr10:104268877	G	G/C	NM_016169.4	-	-	unknown	148	54.73
Pt03	SUFU	chr10:104356822	A	G/G	NM_016169.4	-	-	unknown	138	100
Pt03	NUP98	chr11:3740589	A	A/T	NM_016320.5	-	-	unknown	128	13.28
Pt03	NUP98	chr11:3789982	GAAAAAGAAAAA	GAAAAAGAAAAA/G	NM_016320.5	-	-	unknown	81	G=67.90,GAAAAAGAAAA=9.88
Pt03	EXT2	chr11:44257802	T	T/C	NM_000401.3	-	-	unknown	114	30.7

Pt03	MEN1	chr11:64572557	A	G/G	NM_000244.3	9	p.His438=	synonymous	80	100
Pt03	CCND1	chr11:69466115	C	A/A	NM_053056.3	5	-	unknown	67	100
Pt03	GUCY1A2	chr11:106579204	C	A/A	NM_000855.3	-	-	unknown	134	100
Pt03	ATM	chr11:108117816	G	G/C	NM_000051.3	8	p.Glu343Gln	missense	80	21.25
Pt03	ATM	chr11:108183167	A	G/G	NM_000051.3	40	p.Asn1983Ser	missense	143	99.3
Pt03	ADAMTS20	chr12:43847683	A	G/T	NM_025003.5	-	-	unknown	147	G=95.92,T=3.40
Pt03	ARID2	chr12:46215163	G	G/A	NM_152641.4	-	-	unknown	64	57.81
Pt03	ARID2	chr12:46231408	C	C/G	NM_152641.4	10	p.Ile416Met	missense	99	14.14
Pt03	KMT2D	chr12:49434236	G	G/A	NM_003482.4	32	p.Pro2439=	synonymous	34	8.82
Pt03	KMT2D	chr12:49436501	C	C/G	NM_003482.4	-	-	unknown	158	12.66
Pt03	KMT2D	chr12:49441780	G	G/A	NM_003482.4	15	p.Gln1402Ter	nonsense	162	9.88
Pt03	ERBB3	chr12:56493822	A	A/C	NM_001982.4	-	-	unknown	158	41.77
Pt03	DDIT3	chr12:57911160	G	G/A	NM_001195055.1	3	p.Phe33=	synonymous	123	67.48
Pt03	HNF1A	chr12:121416650	A	A/C	NM_000545.8	1	p.Ile27Leu	missense	114	42.98
Pt03	EP400	chr12:132552040	G	G/A	NM_015409.5	-	-	unknown	64	60.94
Pt03	FLT3	chr13:28597447	ATC	ATC/A	NM_004119.3	-	-	unknown	94	A=43.62,ATT=1.06
Pt03	RB1	chr13:49037984	C	C/T	NM_000321.2	-	-	unknown	61	16.39
Pt03	ERCC5	chr13:103527850	G	G/A	NM_000123.3	15	p.Gly1053Glu	missense	154	5.19
Pt03	ERCC5	chr13:103528002	G	C/C	NM_000123.4	15	p.Asp1104His	missense	57	100
Pt03	BCL2L2	chr14:23777099	G	A/A	NM_001199864.2	3	p.Pro41=	synonymous	43	100
Pt03	NIN	chr14:51237701	A	G/G	NM_020921.3	11	p.Val376=	synonymous	130	100
Pt03	HIF1A	chr14:62213848	T	C/C	NM_001530.4	15	-	unknown	153	100
Pt03	TSHR	chr14:81610655	G	G/A	NM_000369.4	10	p.Lys751=	synonymous	133	42.86
Pt03	TRIP11	chr14:92454604	A	A/G	NM_004239.4	-	-	unknown	179	97.77
Pt03	TRIP11	chr14:92484128	T	T/C	NM_004239.4	-	-	unknown	151	5.96
Pt03	DICER1	chr14:95591070	G	A/A	NM_030621.4	-	-	unknown	164	99.39
Pt03	HSP90AA1	chr14:102548151	A	A/G	NM_001017963.3	12	p.Asp821=	synonymous	139	58.27
Pt03	HSP90AA1	chr14:102548224	T	T/C	NM_001017963.3	-	-	unknown	141	41.84
Pt03	THBS1	chr15:39884847	C	C/T	NM_003246.4	17	p.Leu871=	synonymous	145	39.31
Pt03	KNL1	chr15:40917185	C	C/T	NM_144508.5	10	p.Gln1575Ter	nonsense	97	12.37
Pt03	PML	chr15:74317235	C	C/T	NM_033238.3	4	p.Ala407=	synonymous	136	6.62
Pt03	NTRK3	chr15:88423463	C	T/T	NM_001012338.2	-	-	unknown	91	100
Pt03	IDH2	chr15:90634941	A	A/G	NM_002168.4	-	-	unknown	91	62.64
Pt03	IGF1R	chr15:99456470	G	G/T	NM_000875.5	8	p.Gly596Val	missense	120	5.83
Pt03	IGF1R	chr15:99500256	G	G/A	NM_000875.5	-	-	unknown	68	45.59
Pt03	MYH11	chr16:15843899	G	A/A	NM_001040114.1	-	-	unknown	29	100
Pt03	PALB2	chr16:23640944	G	G/A	NM_024675.4	-	-	unknown	109	8.26
Pt03	MMP2	chr16:55523705	T	T/C	NM_004530.6	7	p.Asp383=	synonymous	107	67.29
Pt03	CDH11	chr16:65005462	G	G/A	NM_001797.4	-	-	unknown	127	54.33
Pt03	CDH11	chr16:65025658	T	C/C	NM_001797.4	-	-	unknown	140	100
Pt03	CDH11	chr16:65025718	G	G/A	NM_001797.4	6	p.Thr255Met	missense	141	38.3

Pt03	MAF	chr16:79633805	TGCC	T/T	NM_005360.5	1	-	unknown	31	100
Pt03	FANCA	chr16:89838078	A	G/G	NM_000135.4	-	-	unknown	173	100
Pt03	FANCA	chr16:89845194	A	G/G	NM_000135.4	-	-	unknown	151	98.68
Pt03	FANCA	chr16:89849480	C	T/T	NM_000135.4	16	p.Gly501Ser	missense	53	100
Pt03	FANCA	chr16:89869761	T	C/C	NM_000135.4	-	-	unknown	116	100
Pt03	NLRP1	chr17:5487164	C	G/G	NM_033004.4	1	p.Ser38=	synonymous	96	100
Pt03	TP53	chr17:7577528	GATGGGCCTCCGGTTC	GATGGGCCTCCGGTTC/GATGCTCCGGTTC	NM_000546.5	7	p.Arg249_Pro250delinsSer	nonframeshiftDeletion	148	37.84
Pt03	TP53	chr17:7579470	CGG	CGC/CGC	NM_000546.5	4	p.Pro72Arg	missense	85	100
Pt03	FLCN	chr17:17122327	G	A/A	NM_144997.7	-	-	unknown	60	100
Pt03	NF1	chr17:29553719	GT	GT/G	NM_001042492.3	-	-	unknown	97	6.19
Pt03	NF1	chr17:29663626	A	A/T	NM_001042492.3	-	-	unknown	34	14.71
Pt03	CDK12	chr17:37671910	G	A/A	NM_016507.4	-	-	unknown	124	100
Pt03	ETV4	chr17:41622740	G	G/T	NM_001986.4	-	-	unknown	157	33.12
Pt03	COL1A1	chr17:48265209	G	G/A	NM_000088.4	-	-	unknown	89	69.66
Pt03	COL1A1	chr17:48268223	A	G/G	NM_000088.4	33	p.Thr766=	synonymous	78	100
Pt03	BRIP1	chr17:59763114	A	T/T	NM_032043.3	-	-	unknown	103	100
Pt03	BIRC5	chr17:76219591	G	A/A	NM_001012271.2	5	p.Glu152Lys	missense	113	100
Pt03	CDH2	chr18:25532080	G	G/A	NM_001792.5	16	-	unknown	155	43.23
Pt03	DCC	chr18:50929159	C	C/A	NM_005215.4	19	p.Pro944His	missense	167	22.75
Pt03	DCC	chr18:50936780	G	G/A	NM_005215.4	-	-	unknown	191	44.5
Pt03	DCC	chr18:50936977	G	G/A	NM_005215.4	20	p.Gly1031Arg	missense	58	5.17
Pt03	DCC	chr18:50936994	T	T/C	NM_005215.4	20	p.Pro1036=	synonymous	172	37.79
Pt03	DCC	chr18:50937026	A	A/G	NM_005215.4	-	-	unknown	171	38.01
Pt03	MALT1	chr18:56338830	C	C/T	NM_006785.4	1	-	unknown	64	46.88
Pt03	CDH20	chr18:59157764	T	C/C	NM_031891.4	2	-	unknown	168	98.81
Pt03	STK11	chr19:1219274	G	A/A	NM_000455.5	-	-	unknown	51	100
Pt03	TCF3	chr19:1615796	G	G/A	NM_001136139.4	17	p.Ala492Val	missense	24	50
Pt03	TCF3	chr19:1650134	A	G/G	NM_001136139.4	-	-	unknown	22	100
Pt03	GNA11	chr19:3110193	C	C/G	NM_002067.5	2	p.Ile61Met	missense	160	8.13
Pt03	GNA11	chr19:3119404	C	C/CG	NM_002067.5	-	-	unknown	8	100
Pt03	GNA11	chr19:3119405	TG	GG/GT	NM_002067.5	-	-	unknown	15	GG=46.67,GT=40.00
Pt03	FZR1	chr19:3533274	G	A/A	NM_001136198.1	-	-	unknown	65	100
Pt03	FZR1	chr19:3533275	C	C/A	NM_001136198.1	-	-	unknown	68	48.53
Pt03	MARK4	chr19:45801018	T	C/C	NM_001199867.2	15	p.Arg561=	synonymous	99	100
Pt03	ERCC2	chr19:45868309	T	G/G	NM_000400.4	6	p.Arg156=	synonymous	61	100
Pt03	PPP2R1A	chr19:52719190	A	A/G	NM_014225.6	-	-	unknown	100	53

Pt03	PPP2R1A	chr19:52725338	T	T/C	NM_014225.6	-	-	unknown	86	38.37
Pt03	ASXL1	chr20:31019024	C	C/T	NM_015338.6	-	-	unknown	178	51.69
Pt03	ASXL1	chr20:31022959	T	C/C	NM_015338.6	12	p.Leu815Pro	missense	107	100
Pt03	PLCG1	chr20:39792538	C	T/T	NM_002660.3	-	-	unknown	85	100
Pt03	AURKA	chr20:54961541	A	T/T	NM_003600.4	3	p.Phe31Ile	missense	118	100
Pt03	ERG	chr21:39762883	T	T/C	NM_182918.4	-	-	unknown	135	58.52
Pt03	ITGB2	chr21:46311813	A	G/G	NM_000211.5	11	p.Val441=	synonymous	135	99.26
Pt03	ITGB2	chr21:46314907	T	A/A	NM_000211.5	9	p.Gln354His	missense	70	100
Pt03	SYN3	chr22:33253280	T	C/C	NM_000362.5	3	p.His83=	synonymous	78	100
Pt03	SYN3	chr22:33253292	C	C/T	NM_000362.5	3	p.Ser87=	synonymous	78	61.54
Pt03	MYH9	chr22:36691607	A	C/C	NM_002473.6	26	p.Ala1143=	synonymous	98	100
Pt03	MYH9	chr22:36694954	C	C/G	NM_002473.6	-	-	unknown	25	60
Pt03	MYH9	chr22:36708049	CTCCTG TGA	CTCCTG TGA/C	NM_002473.6	-	-	unknown	72	50
Pt03	MYH9	chr22:36708084	C	C/T	NM_002473.6	-	-	unknown	76	97.37
Pt03	PDGFB	chr22:39636932	A	A/G	NM_002608.4	-	-	unknown	78	5.13
Pt03	CYP2D6	chr22:42523943	A	G/G	NM_000106.6	6	p.Cys296Arg	missense	87	100
Pt03	CYP2D6	chr22:42526549	C	T/T	NM_000106.6	-	-	unknown	101	100
Pt03	CYP2D6	chr22:42526561	GG	TC/TC	NM_000106.6	-	-	unknown	100	100
Pt03	CYP2D6	chr22:42526567	G	A/A	NM_000106.6	-	-	unknown	100	99
Pt03	CYP2D6	chr22:42526571	C	G/G	NM_000106.6	-	-	unknown	100	100
Pt03	CYP2D6	chr22:42526573	T	G/G	NM_000106.6	-	-	unknown	100	100
Pt03	CYP2D6	chr22:42526580	G	C/C	NM_000106.6	-	-	unknown	100	99
Pt04	ARID1A	chr1:27106747	G	G/A	NM_006015.6	20	p.Glu2120Lys	missense	94	48.94
Pt04	CMPK1	chr1:47834231	T	T/C	NM_016308.3	2	p.Tyr88His	missense	197	14.21
Pt04	DPYD	chr1:98058871	G	G/A	NM_000110.3	10	p.Ala344Val	missense	130	48.46
Pt04	PDE4DIP	chr1:144864126	C	C/T	NM_001198834.3	-	-	unknown	51	31.37
Pt04	PDE4DIP	chr1:144864143	G	G/A	NM_001198834.3	36	p.Asn1984=	synonymous	51	31.37
Pt04	PBX1	chr1:164776928	C	C/T	NM_002585.3	-	-	unknown	132	43.94
Pt04	PBX1	chr1:164776953	C	C/T	NM_002585.3	-	-	unknown	127	45.67
Pt04	ABL2	chr1:179086450	G	G/GA	NM_005158.5	-	-	unknown	99	5.05
Pt04	PARP1	chr1:226595478	TCCCCG	TCCCCG /TCCCG	NM_001618.4	-	-	unknown	34	58.82
Pt04	SOX11	chr2:5833746	G	G/A	NM_003108.4	1	p.Arg298Gln	missense	134	22.39
Pt04	EML4	chr2:42396722	A	A/G	NM_019063.5	1	-	unknown	26	30.77
Pt04	MSH6	chr2:48033894	T	T/C	NM_000179.2	-	-	unknown	9	33.33
Pt04	ERCC3	chr2:128028923	C	C/T	NM_000122.1	12	p.Arg645Gln	missense	96	58.33
Pt04	IDH1	chr2:209113154	G	G/C	NM_005896.3	4	p.Pro118Arg	missense	139	9.35
Pt04	FN1	chr2:216272908	G	G/T	NM_212482.3	17	p.Pro814His	missense	79	10.13
Pt04	ITGA9	chr3:37574768	T	T/C	NM_002207.3	-	-	unknown	67	89.55

Pt04	IL7R	chr5:35857177	G	G/C	NM_002185.5	-	-	unknown	87	9.2
Pt04	IL7R	chr5:35876491	T	T/C	NM_002185.5	8	p.Leu428Ser	missense	223	17.49
Pt04	IL6ST	chr5:55248168	C	C/T	NM_002184.4	12	p.Glu488Lys	missense	149	67.79
Pt04	FGFR4	chr5:176523744	T	T/TC	NM_213647.3	16	-	unknown	32	25
Pt04	NSD1	chr5:176637240	G	T/T	NM_022455.4	5	p.Val614Leu	missense	9	100
Pt04	PKHD1	chr6:51882152	C	C/T	NM_138694.4	-	-	unknown	85	44.71
Pt04	ROS1	chr6:117706963	C	C/T	NM_002944.2	15	p.Trp729Ter	nonsense	200	5
Pt04	SBDS	chr7:66460350	G	G/A	NM_016038.4	1	p.Arg19Trp	missense	111	42.34
Pt04	CDK6	chr7:92247319	CCCAGTCTGGGTAGAG	CCCAGTCTGGGTAGAG/C	NM_001145306.1	-	-	unknown	104	54.81
Pt04	RECQL4	chr8:145738583	G	G/T	NM_004260.3	-	-	unknown	14	57.14
Pt04	RECQL4	chr8:145742514	A	A/G	NM_004260.3	4	p.Ser92Pro	missense	104	43.27
Pt04	PAX5	chr9:36923506	C	C/G	NM_016734.3	-	-	unknown	85	36.47
Pt04	NUP214	chr9:134014645	T	T/C	NM_005085.4	-	-	unknown	76	6.58
Pt04	GUCY1A2	chr11:106888774	C	C/G	NM_000855.3	1	p.Arg3Pro	missense	31	58.06
Pt04	KMT2A	chr11:118382642	CGGGTTTTCTTTATTTCCTTTCA	CGGGTTTTCTTTATTTCCTTTCA/C	NM_001197104.1	31	-	unknown	153	26.8
Pt04	ETS1	chr11:128359188	T	T/A	NM_001143820.2	5	p.Lys178Ter	nonsense	127	63.78
Pt04	FOXO1	chr13:41240106	C	C/T	NM_002015.4	1	p.Asp82Asn	missense	16	56.25
Pt04	RB1	chr13:49050906	G	G/T	NM_000321.2	25	p.Glu864Ter	nonsense	73	93.15
Pt04	ERCC5	chr13:103527850	G	G/A	NM_000123.3	15	p.Gly1053Glu	missense	140	5.71
Pt04	IRS2	chr13:110435126	T	T/G	NM_003749.3	1	p.Lys1092Thr	missense	6	50
Pt04	IRS2	chr13:110435914	G	G/A	NM_003749.3	1	p.Pro829=	synonymous	119	60.5
Pt04	TSHR	chr14:81609939	A	A/C	NM_000369.3	10	p.Thr513Pro	missense	108	5.56
Pt04	TCF12	chr15:57383956	C	C/CT	NM_207037.2	-	-	unknown	31	9.68
Pt04	IGF1R	chr15:99192741	TTTC	TTTC/T	NM_000875.5	1	-	unknown	19	21.05
Pt04	CDH5	chr16:66437049	C	C/T	NM_001795.5	12	p.Pro778Ser	missense	52	73.08
Pt04	MAF	chr16:79633805	TGCC	TGCC/T	NM_005360.5	1	-	unknown	21	52.38
Pt04	TP53	chr17:7577157	T	T/A	NM_000546.5	8	-	unknown	98	9082
Pt04	TP53	chr17:7579633	GCCCCCCAG	GCCCCCCAG/GC	NM_000546.5	-	-	unknown	40	10
Pt04	MALT1	chr18:56376590	A	A/AT	NM_006785.4	-	-	unknown	17	23.53
Pt04	TCF3	chr19:1620911	CTC	CGTC/CT	NM_001136139.4	-	-	unknown	12	CGTC=41.67,CT=50.00
Pt04	FZR1	chr19:3533275	C	C/A	NM_001136198.1	-	-	unknown	72	29.17
Pt04	PIK3R2	chr19:45801337	A	A/G	NM_005027.4	7	p.Lys286Arg	missense	74	5.41

Pt04	ERCC2	chr19:45856347	C	C/T	NM_000400.3	19	p.Asp609Asn	missense	129	47.29
Pt04	RUNX1	chr21:36164405	G	G/T	NM_001754.4	9	-	unknown	85	97.65
Pt04	BCR	chr22:23603667	C	C/T	NM_004327.4	4	p.Leu564=	synonymous	83	21.69
Pt04	PDGFB	chr22:39627591	C	C/T	NM_002608.4	-	-	unknown	41	73.17
Pt04	TAF1	chrX:70679458	G	G/A	NM_004606.4	36	p.Gln1727=	synonymous	81	6.17
Pt05	LCK	chr1:32741342	G	G/A	NM_001042771.2	-	-	unknown	100	19
Pt05	TAL1	chr1:47691090	T	T/C	NM_003189.5	-	-	unknown	8	87.5
Pt05	PDE4DIP	chr1:144855782	C	C/T	NM_001198834.3	41	p.Ala2257=	synonymous	71	12.68
Pt05	PDE4DIP	chr1:144855812	T	T/C	NM_001198834.3	41	p.Ser2247=	synonymous	71	23.94
Pt05	PDE4DIP	chr1:144994902	G	G/A	NM_001198834.3	-	-	unknown	499	9.42
Pt05	MTR	chr1:237024404	T	T/C	NM_000254.2	-	-	unknown	11	54.55
Pt05	BAP1	chr3:52439966	G	G/C	NM_004656.4	-	-	unknown	81	29.63
Pt05	PBRM1	chr3:52637714	G	G/T	NM_018313.5	18	p.Leu868Ile	missense	75	30.67
Pt05	KIT	chr4:55569922	T	T/G	NM_000222.2	5	p.His263Gln	missense	153	7.19
Pt05	PKHD1	chr6:51618174	A	A/G	NM_138694.4	-	-	unknown	135	5.19
Pt05	TNFAIP3	chr6:138195961	TCTC	T/T	NM_001270507.2	-	-	unknown	35	100
Pt05	CARD11	chr7:2972136	A	A/T	NM_032415.6	-	-	unknown	74	5.41
Pt05	EPHB4	chr7:100411644	C	C/G	NM_004444.5	9	-	unknown	150	33.33
Pt05	GRM8	chr7:126086145	G	G/A	NM_000845.3	-	-	unknown	158	34.81
Pt05	KMT2C	chr7:151882672	C	C/A	NM_170606.3	34	p.Ala1685Ser	missense	177	5.65
Pt05	FGFR2	chr10:123263459	G	G/C	NM_000141.4	-	-	unknown	126	50
Pt05	KMT2D	chr12:49447254	C	C/T	NM_003482.3	-	-	unknown	122	26.23
Pt05	FOXO1	chr13:41240106	C	C/T	NM_002015.4	1	p.Asp82Asn	missense	20	35
Pt05	CREBBP	chr16:3817843	G	G/C	NM_004380.3	16	p.Ser1043Ter	nonsense	161	43.48
Pt05	MYH11	chr16:15811062	C	C/T	NM_001040114.1	39	p.Lys1820=	synonymous	39	10.26
Pt05	NLRP1	chr17:5421106	C	C/A	NM_033004.4	15	p.Lys1339Asn	missense	196	39.29
Pt05	NF1	chr17:29555994	C	C/T	NM_001042492.2	-	-	unknown	157	40.13
Pt05	ZNF521	chr18:22642721	A	A/G	NM_015461.3	-	-	unknown	74	12.16
Pt05	ZNF521	chr18:22642727	A	A/G	NM_015461.3	-	-	unknown	35	8.57
Pt05	DCC	chr18:50936977	G	G/A	NM_005215.4	20	p.Gly1031Arg	missense	96	9.38
Pt05	RUNX1	chr21:36164405	G	G/T	NM_001754.4	9	-	unknown	57	42.11
Pt05	BCR	chr22:23523630	C	C/A	NM_004327.4	1	p.Ile161=	synonymous	48	95.83
Pt05	TAF1	chrX:70612769	A	A/G	NM_004606.4	20	p.Thr1012=	synonymous	48	6.25
Pt06	PBX1	chr1:164529120	G	G/A	NM_002585.3	1	p.Gly21Ser	missense	24	95.83
Pt06	PIK3C2B	chr1:204400784	A	A/G	NM_002646.3	-	-	unknown	128	5.47
Pt06	EML4	chr2:42396722	A	A/G	NM_019063.5	1	-	unknown	9	33.33
Pt06	MSH2	chr2:47630550	C	G/G	NM_000251.2	-	-	unknown	55	100
Pt06	AFF3	chr2:100210074	G	G/C	NM_001025108.2	14	p.Ser708=	synonymous	56	48.21
Pt06	AFF3	chr2:100210642	T	T/C	NM_001025108.2	14	p.Asn519Ser	missense	90	44.44
Pt06	LRP1B	chr2:141359287	C	C/T	NM_018557.2	-	-	unknown	136	30.88
Pt06	STK36	chr2:219563603	G	G/C	NM_015690.5	26	p.Arg1112=	synonymous	144	11.11
Pt06	EPHB1	chr3:134514579	T	C/C	NM_004441.5	-	-	unknown	95	100

Pt06	NSD2	chr4:1940123	C	C/T	NM_001042424.3	-	-	unknown	25	16
Pt06	KIT	chr4:55598217	T	T/C	NM_000222.2	-	-	unknown	47	14.89
Pt06	IL6ST	chr5:55243425	A	A/G	NM_002184.4	-	-	unknown	59	13.56
Pt06	IL6ST	chr5:55251892	C	C/T	NM_002184.4	10	p.Asp410Asn	missense	88	45.45
Pt06	SYNE1	chr6:152793375	TA	TA/T	NM_182961.4	-	-	unknown	93	38.71
Pt06	AKAP9	chr7:91732045	G	G/A	NM_005751.4	46	p.Gly3745=	synonymous	126	52.38
Pt06	BRAF	chr7:140434630	AAG	AAG/A	NM_004333.6	-	-	unknown	21	9.52
Pt06	KMT2C	chr7:151962257	C	C/T	NM_170606.3	8	p.Pro350=	synonymous	739	8.93
Pt06	KMT2C	chr7:151962265	C	C/T	NM_170606.3	8	p.Asp348Asn	missense	732	8.88
Pt06	WRN	chr8:31000243	T	C/C	NM_000553.5	-	-	unknown	30	100
Pt06	RECQL4	chr8:145737286	G	A/A	NM_004260.3	-	-	unknown	106	100
Pt06	RECQL4	chr8:145738767	CG	C/C	NM_004260.3	15	-	unknown	56	100
Pt06	RECQL4	chr8:145742879	T	T/C	NM_004260.3	3	p.Glu44=	synonymous	80	98.75
Pt06	RALGDS	chr9:135974079	G	C/C	NM_001271775.2	18	p.Leu879=	synonymous	23	100
Pt06	RALGDS	chr9:135974100	G	A/A	NM_001271775.2	18	p.Thr872=	synonymous	23	100
Pt06	NOTCH1	chr9:139396690	C	T/T	NM_017617.5	-	-	unknown	79	100
Pt06	NOTCH1	chr9:139410177	T	C/C	NM_017617.5	-	-	unknown	93	98.92
Pt06	NOTCH1	chr9:139412197	G	A/A	NM_017617.5	-	-	unknown	78	100
Pt06	NFKB2	chr10:104159196	A	G/G	NM_001077494.3	13	p.Pro423=	synonymous	59	100
Pt06	NUP98	chr11:3740588	G	G/T	NM_016320.5	-	-	unknown	90	66.67
Pt06	EXT2	chr11:44117898	T	G/G	NM_000401.3	-	-	unknown	54	100
Pt06	MEN1	chr11:64572018	T	C/C	NM_000244.3	10	p.Thr546Ala	missense	127	99.21
Pt06	KRAS	chr12:25398280	GCCAC	GCCAC/GCCAT	NM_033360.4	2	p.Gly12Asp	missense	101	38.61
Pt06	CDK8	chr13:26967597	A	A/G	NM_001260.3	7	p.Tyr247Cys	missense	134	36.57
Pt06	FLT3	chr13:28609825	A	A/G	NM_004119.2	-	-	unknown	109	26.61
Pt06	TSHR	chr14:81609939	A	A/C	NM_000369.3	10	p.Thr513Pro	missense	97	6.19
Pt06	THBS1	chr15:39884882	G	G/T	NM_003246.4	17	p.Gln882His	missense	104	30.77
Pt06	BUB1B	chr15:40491952	A	G/G	NM_001211.5	-	-	unknown	173	100
Pt06	IGF1R	chr15:99192754	CTTTTTTTTTTT	CTTTTTCTTTTT/C	NM_000875.5	1	-	unknown	28	CTTTTTCTTTTT=28.57,C=50.00,CT=0.00,CTT=7.14,CTTTTTTTTT=14.29
Pt06	MAF	chr16:79633805	TGCC	T/T	NM_005360.5	1	-	unknown	9	100
Pt06	ITGB3	chr17:45331358	C	C/G	NM_000212.2	-	-	unknown	41	46.34
Pt06	COL1A1	chr17:48277059	AGCCCCAGGCCCCAGGCCCCAG	A/A	NM_000088.3	-	-	unknown	9	100
Pt06	ZNF521	chr18:22642721	A	A/G	NM_015461.3	-	-	unknown	74	13.51
Pt06	ZNF521	chr18:22642722	A	A/G	NM_015461.3	-	-	unknown	69	18.84
Pt06	DCC	chr18:50936977	G	G/A	NM_005215.4	20	p.Gly1031Arg	missense	74	5.41

Pt06	SMARCA4	chr19:11095072	C	C/T	NM_001128849.2	-	-	unknown	116	43.97
Pt06	RUNX1	chr21:36164405	G	G/T	NM_001754.4	9	-	unknown	43	97.67
Pt06	BCR	chr22:23653880	G	A/A	NM_004327.4	-	-	unknown	8	100
Pt06	CYP2D6	chr22:42525132	G	G/C	NM_000106.6	3	p.Val136=	synonymous	292	27.4
Pt06	USP9X	chrX:40999853	C	A/A	NM_001039590.3	-	-	unknown	46	100
Pt08	ARID1A	chr1:27023078	G	G/A	NM_006015.6	1	p.Ala62Thr	missense	13	30.77
Pt08	ARID1A	chr1:27105946	G	G/T	NM_006015.6	20	p.Glu1853Ter	nonsense	169	53.25
Pt08	TRIM33	chr1:114940209	G	G/T	NM_015906.4	20	-	unknown	76	26.32
Pt08	TRIM33	chr1:114948124	G	G/C	NM_015906.4	15	p.Val892=	synonymous	138	57.25
Pt08	PDE4DIP	chr1:144882583	T	T/C	NM_001198834.4	24	p.Ile1146Val	missense	421	24.7
Pt08	PDE4DIP	chr1:144886112	G	G/A	NM_001198834.4	23	p.Ser1041Leu	missense	301	14.95
Pt08	NTRK1	chr1:156836690	C	C/G	NM_002529.3	-	-	unknown	126	46.03
Pt08	DDR2	chr1:162743418	G	T/T	NM_006182.4	-	-	unknown	207	100
Pt08	PBX1	chr1:164529120	G	A/A	NM_002585.4	1	p.Gly21Ser	missense	9	100
Pt08	MDM4	chr1:204512101	T	T/C	NM_002393.5	-	-	unknown	116	8.62
Pt08	MTR	chr1:237024391	C	C/CT	NM_000254.2	-	-	unknown	15	13.33
Pt08	MTR	chr1:237024403	T	T/TC	NM_000254.2	-	-	unknown	12	75
Pt08	MTR	chr1:237024404	T	T/C	NM_000254.2	-	-	unknown	17	17.65
Pt08	MTR	chr1:237048563	T	T/A	NM_000254.2	-	-	unknown	151	5.3
Pt08	AKT3	chr1:243736210	CTTTTTTTTTTT	CTTTTTTTTTTT/C	NM_005465.7	-	-	unknown	137	18.98
Pt08	LRP1B	chr2:141459964	C	C/T	NM_018557.3	-	-	unknown	221	42.53
Pt08	NFE2L2	chr2:178097246	G	G/A	NM_006164.5	4	p.Phe156=	synonymous	196	40.31
Pt08	PMS1	chr2:190670539	TAAAAAAA	TAAAAAAA/T	NM_000534.5	-	-	unknown	166	22.29
Pt08	SF3B1	chr2:198269914	GA	GA/G	NM_012433.4	-	-	unknown	95	17.89
Pt08	SF3B1	chr2:198274750	T	T/A	NM_012433.4	-	-	unknown	172	51.16
Pt08	STK36	chr2:219559395	C	G/G	NM_015690.5	-	-	unknown	26	100
Pt08	FANCD2	chr3:10088343	A	A/G	NM_033084.6	15	p.Asn405Ser	missense	180	6.11
Pt08	LTF	chr3:46501268	C	C/T	NM_002343.6	2	p.Ala29Thr	missense	128	21.88
Pt08	GATA2	chr3:128202760	G	G/C	NM_032638.5	4	p.Gly320=	synonymous	293	16.04
Pt08	PIK3CA	chr3:178952085	A	A/G	NM_006218.4	21	p.His1047Arg	missense	156	58.33
Pt08	NSD2	chr4:1940128	C	C/T	NM_001042424.3	-	-	unknown	14	28.57
Pt08	NSD2	chr4:1940139	C	C/T	NM_001042424.3	-	-	unknown	13	53.85
Pt08	NSD2	chr4:1980477	C	C/T	NM_001042424.3	22	p.Phe1313=	synonymous	154	59.74
Pt08	ADGRL3	chr4:62936032	G	G/A	NM_015236.6	25	p.Leu1272=	synonymous	182	58.24
Pt08	AFF1	chr4:88016073	A	A/G	NM_001166693.2	8	p.Tyr412Cys	missense	139	54.68
Pt08	AFF1	chr4:88029381	G	G/A	NM_001166693.2	11	p.Asp483Asn	missense	158	63.92
Pt08	NFKB1	chr4:103516021	G	G/C	NM_003998.4	-	-	unknown	231	35.06
Pt08	MTRR	chr5:7897191	C	C/T	NM_024010.4	14	p.His595Tyr	missense	108	7.41
Pt08	IL7R	chr5:35876109	G	G/A	NM_002185.5	8	p.Glu301Lys	missense	177	57.63

Pt08	LIFR	chr5:38523568	A	A/T	NM_002310.6	5	p.Trp172Arg	missense	242	6.2
Pt08	IL6ST	chr5:55243426	A	A/G	NM_002184.4	-	-	unknown	86	17.44
Pt08	IL6ST	chr5:55243440	A	A/G	NM_002184.4	-	-	unknown	33	21.21
Pt08	NOTCH4	chr6:32166479	C	C/T	NM_004557.4	25	p.Glu1522Lys	missense	175	38.86
Pt08	DST	chr6:56464925	T	T/G	NM_001144769.5	39	p.Glu1760Ala	missense	194	5.67
Pt08	ROS1	chr6:117631482	C	C/T	NM_002944.2	-	-	unknown	70	5.71
Pt08	SYNE1	chr6:152532732	TA	TA/T	NM_182961.4	-	-	unknown	53	9.43
Pt08	RPS6KA2	chr6:166826255	G	A/A	NM_001006932.3	22	p.Leu741=	synonymous	36	100
Pt08	RPS6KA2	chr6:166826304	T	C/C	NM_001006932.3	22	p.Ser724=	synonymous	36	100
Pt08	AKAP9	chr7:91641709	G	G/A	NM_005751.4	-	-	unknown	78	62.82
Pt08	TRRAP	chr7:98513306	C	C/T	NM_001244580.1	-	-	unknown	191	61.26
Pt08	TRRAP	chr7:98574279	C	C/G	NM_001244580.1	54	p.Phe2704Leu	missense	166	62.05
Pt08	EPHB4	chr7:100410477	C	C/A	NM_004444.5	12	p.Gln670His	missense	122	61.48
Pt08	BRAF	chr7:140434588	A	A/G	NM_004333.6	-	-	unknown	71	11.27
Pt08	BRAF	chr7:140434601	A	A/G	NM_004333.6	-	-	unknown	59	5.08
Pt08	BRAF	chr7:140434602	A	A/G	NM_004333.6	-	-	unknown	58	8.62
Pt08	BRAF	chr7:140434611	AAG	AAG/A	NM_004333.6	-	-	unknown	50	A=8.00,AA=72.00,AAAA=2.00
Pt08	KMT2C	chr7:151948998	TA	TA/TT	NM_170606.3	-	-	unknown	163	AA=3.07,TT=11.66
Pt08	CSMD3	chr8:113301623	T	T/C	NM_198123.2	57	p.His3040Arg	missense	134	40.3
Pt08	PTPRD	chr9:8518470	C	C/G	NM_002839.4	-	-	unknown	87	32.18
Pt08	ABL1	chr9:133755930	G	G/A	NM_005157.6	10	p.Val519=	synonymous	178	43.82
Pt08	NOTCH1	chr9:139391636	G	G/A	NM_017617.5	34	p.Asp2185=	synonymous	178	55.06
Pt08	MLLT10	chr10:22023084	T	T/G	NM_001195626.3	-	-	unknown	139	7.19
Pt08	SUFU	chr10:104264107	C	C/T	NM_016169.4	-	-	unknown	30	40
Pt08	NUP98	chr11:3740589	A	A/T	NM_016320.5	-	-	unknown	152	7.24
Pt08	RRM1	chr11:4116181	C	C/T	NM_001033.5	1	-	unknown	188	44.15
Pt08	WT1	chr11:32417819	G	G/A	NM_024426.6	7	p.His416=	synonymous	179	49.16
Pt08	MRE11	chr11:94212930	TAAAAAAA	TAAAAAAA/T	NM_005591.4	-	-	unknown	123	25.2
Pt08	CCND2	chr12:4383158	T	C/C	NM_001759.4	1	-	unknown	14	100
Pt08	KMT2D	chr12:49424534	G	A/A	NM_003482.4	42	p.Pro4563=	synonymous	46	100
Pt08	KMT2D	chr12:49424590	G	G/A	NM_003482.4	-	-	unknown	45	35.56
Pt08	KMT2D	chr12:49443825	A	A/T	NM_003482.4	12	p.Cys1182Ter	nonsense	162	45.06
Pt08	HNF1A	chr12:121434486	G	G/T	NM_000545.8	6	p.Gly417Val	missense	88	54.55
Pt08	EP400	chr12:132547102	G	G/A	NM_015409.5	47	p.Gln2730=	synonymous	46	6.52
Pt08	RB1	chr13:48916831	C	T/T	NM_000321.2	3	p.Gln121Ter	nonsense	215	99.07
Pt08	IRS2	chr13:110436232	G	A/A	NM_003749.3	1	p.Ser723=	synonymous	30	100
Pt08	THBS1	chr15:39884882	G	G/T	NM_003246.4	17	p.Gln882His	missense	122	31.97
Pt08	BUB1B	chr15:40512823	G	G/A	NM_001211.6	23	p.Glu1006Lys	missense	147	41.5
Pt08	IGF1R	chr15:99192744	C	C/CT	NM_000875.5	1	-	unknown	74	CT=5.41,T=4.05
Pt08	PALB2	chr16:23637604	G	G/A	NM_024675.4	7	p.Leu901=	synonymous	138	38.41

Pt08	PALB2	chr16:23641257	G	G/A	NM_024675.4	5	p.Gln740Ter	nonsense	124	20.16
Pt08	TP53	chr17:7577070	GGAGATTCTCTTCCTCTGTGC	GGAGATTCTCTTCCTCTGTGC/GGAGATTCTCTTCCTATGTGC	NM_000546.5	8	p.Glu285Ter	nonsense	89	95.51
Pt08	PER1	chr17:8047118	C	C/T	NM_002616.3	19	p.Gln846=	synonymous	89	94.38
Pt08	PGAP3	chr17:37829129	A	A/G	NM_033419.5	-	-	unknown	68	7.35
Pt08	RARA	chr17:38498990	C	C/G	NM_000964.4	-	-	unknown	240	50
Pt08	ETV4	chr17:41610566	G	G/A	NM_001986.4	7	p.Leu178=	synonymous	165	51.52
Pt08	SEPTIN9	chr17:75369490	CGGCGCCCCAGCCA	CGGCGCCCCAGCCA/C	NM_001113491.2	-	-	unknown	106	57.55
Pt08	RNF213	chr17:78280065	C	C/T	NM_001256071.3	12	p.Gln742Ter	nonsense	132	55.3
Pt08	RNF213	chr17:78363057	G	G/A	NM_001256071.3	65	p.Glu5029Lys	missense	134	36.57
Pt08	RNF213	chr17:78363104	G	G/A	NM_001256071.3	65	p.Met5044Ile	missense	135	58.52
Pt08	ZNF521	chr18:22642721	A	A/G	NM_015461.3	-	-	unknown	81	6.17
Pt08	ZNF521	chr18:22642722	A	A/G	NM_015461.3	-	-	unknown	76	11.84
Pt08	ZNF521	chr18:22642726	A	A/G	NM_015461.3	-	-	unknown	61	9.84
Pt08	ZNF521	chr18:22642731	A	A/G	NM_015461.3	-	-	unknown	37	16.22
Pt08	ZNF521	chr18:22642735	A	A/G	NM_015461.3	-	-	unknown	19	21.05
Pt08	ZNF521	chr18:22671909	C	C/T	NM_015461.3	-	-	unknown	230	53.04
Pt08	MBD1	chr18:47806271	G	G/C	NM_001204136.1	2	p.Ser31Ter	nonsense	147	50.34
Pt08	DCC	chr18:50936994	T	T/C	NM_005215.4	20	p.Pro1036=	synonymous	198	54.55
Pt08	DCC	chr18:50937026	A	A/G	NM_005215.4	-	-	unknown	190	57.37
Pt08	ERCC2	chr19:45872387	T	T/A	NM_000400.4	3	p.Met42Leu	missense	97	57.73
Pt08	ITGB2	chr21:46330647	G	G/A	NM_000211.5	2	p.Leu17=	synonymous	158	49.37
Pt08	MN1	chr22:28195829	A	A/G	NM_002430.3	1	p.Tyr235His	missense	143	95.1
Pt08	NF2	chr22:30051602	T	T/C	NM_000268.4	6	p.Met179Thr	missense	174	95.4
Pt08	MYH9	chr22:36697560	G	G/A	NM_002473.6	-	-	unknown	207	95.65
Pt08	EP300	chr22:41550954	C	T/T	NM_001429.4	-	-	unknown	17	100
Pt08	EP300	chr22:41572852	G	G/C	NM_001429.4	31	p.Asp1713His	missense	167	47.31
Pt08	EP300	chr22:41573760	G	G/T	NM_001429.4	31	p.Met2015Ile	missense	150	44
Pt08	KDM6A	chrX:44935877	CT	C/TC	NM_021140.3	-	-	unknown	29	C=79.31,CTT=0.00,TC=10.34
Pt08	KDM5C	chrX:53223430	T	T/C	NM_004187.5	23	p.Glu1310Gly	missense	71	5.63
Pt08	TAF1	chrX:70612705	CT	CT/C	NM_004606.5	-	-	unknown	80	7.5
Pt08	TAF1	chrX:70612769	A	A/G	NM_004606.5	20	p.Thr992=	synonymous	37	8.11

Pt09	PIK3CD	chr1:9784395	G	G/A	NM_005026.5	22	p.Arg927His	missense	202	11.88
Pt09	MTOR	chr1:11206736	T	T/C	NM_004958.4	32	p.Gln1561=	synonymous	114	10.53
Pt09	JAK1	chr1:65300273	A	A/G	NM_002227.4	25	p.Ile1146Thr	missense	124	28.23
Pt09	ABL2	chr1:179077972	G	G/A	NM_005158.5	12	p.Leu795=	synonymous	147	16.33
Pt09	MDM4	chr1:204512101	T	T/C	NM_002393.5	-	-	unknown	151	7.95
Pt09	MSH6	chr2:48032885	T	T/A	NM_000179.2	-	-	unknown	162	6.17
Pt09	PAX8	chr2:113993153	T	T/G	NM_003466.4	9	p.His302Pro	missense	103	7.77
Pt09	NFE2L2	chr2:178095838	A	A/G	NM_006164.5	5	p.Ile498Thr	missense	115	14.78
Pt09	BAP1	chr3:52442072	T	T/C	NM_004656.4	5	p.Thr93Ala	missense	112	16.96
Pt09	EPHA3	chr3:89259414	T	T/C	NM_005233.6	3	p.Val186=	synonymous	208	26.44
Pt09	NSD2	chr4:1940130	T	T/C	NM_001042424.3	-	-	unknown	40	17.5
Pt09	NSD2	chr4:1940143	C	C/T	NM_001042424.3	-	-	unknown	28	21.43
Pt09	NSD2	chr4:1940148	T	T/C	NM_001042424.3	-	-	unknown	36	22.22
Pt09	NSD2	chr4:1940149	T	T/C	NM_001042424.3	-	-	unknown	34	11.76
Pt09	KIT	chr4:55569922	T	T/G	NM_000222.2	5	p.His263Gln	missense	119	5.04
Pt09	TET2	chr4:106156227	G	G/C	NM_001127208.2	3	p.Met376Ile	missense	100	11
Pt09	FBXW7	chr4:153247168	T	T/A	NM_033632.3	10	p.Tyr545Phe	missense	130	17.69
Pt09	MTRR	chr5:7897191	C	C/T	NM_024010.4	14	p.His595Tyr	missense	69	21.74
Pt09	IL6ST	chr5:55243426	A	A/G	NM_002184.4	-	-	unknown	52	19.23
Pt09	IL6ST	chr5:55243440	A	A/G	NM_002184.4	-	-	unknown	14	42.86
Pt09	NOTCH4	chr6:32166824	G	G/A	NM_004557.4	24	p.Arg1472Trp	missense	246	10.57
Pt09	DAXX	chr6:33290613	T	T/TC	NM_001369203.1	-	-	unknown	47	12.77
Pt09	PKHD1	chr6:51913367	G	G/A	NM_138694.4	23	p.Thr777Met	missense	145	21.38
Pt09	ROS1	chr6:117706963	C	C/T	NM_002944.2	15	p.Trp729Ter	nonsense	168	8.33
Pt09	TNFAIP3	chr6:138195961	TCTC	TCTC/T	NM_001270507.2	-	-	unknown	64	23.44
Pt09	BRAF	chr7:140434594	A	A/G	NM_004333.6	-	-	unknown	62	8.06
Pt09	BRAF	chr7:140434606	AG	AG/AA	NM_004333.6	-	-	unknown	47	AA=12.77,GA=10.64
Pt09	KMT2C	chr7:151882735	G	G/A	NM_170606.3	-	-	unknown	179	5.03
Pt09	KMT2C	chr7:151970951	C	C/T	NM_170606.3	7	p.Arg284Gln	missense	203	7.39
Pt09	KMT2C	chr7:151971006	C	C/T	NM_170606.3	-	-	unknown	208	7.21
Pt09	RECQL4	chr8:145742879	T	T/C	NM_004260.3	3	p.Glu44=	synonymous	98	40.82
Pt09	GATA3	chr10:8100712	A	A/G	NM_001002295.2	3	p.Tyr229Cys	missense	166	12.65
Pt09	MLLT10	chr10:22023084	T	T/G	NM_001195626.2	-	-	unknown	115	5.22
Pt09	ATM	chr11:108188140	A	A/G	NM_000051.3	43	p.Tyr2080Cys	missense	147	20.41
Pt09	ATM	chr11:108236235	A	A/G	NM_000051.3	63	p.Ter3057Trp	stoploss	125	12.8
Pt09	KMT2D	chr12:49421862	A	A/T	NM_003482.3	46	p.Tyr4815Ter	nonsense	133	23.31
Pt09	KMT2D	chr12:49436084	G	G/A	NM_003482.3	28	p.Pro1966Leu	missense	129	19.38
Pt09	HNF1A	chr12:121416587	A	A/G	NM_000545.6	1	p.Ser6Gly	missense	59	5.08
Pt09	IRS2	chr13:110436232	G	A/A	NM_003749.3	1	p.Ser723=	synonymous	15	100
Pt09	HIF1A	chr14:62212360	A	A/T	NM_001530.4	-	-	unknown	61	6.56
Pt09	TSHR	chr14:81609939	A	A/C	NM_000369.3	10	p.Thr513Pro	missense	60	5
Pt09	BCL11B	chr14:99642360	C	C/G	NM_138576.4	4	p.Pro271=	synonymous	32	37.5

Pt09	IL21R	chr16:27441435	C	C/T	NM_021798.4	2	p.Gln15Ter	nonsense	59	13.56
Pt09	MAF	chr16:79633805	TGCCGC C	T/TGCC	NM_005360.5	-	-	unknown	11	T=18.18,TGCC=81.82
Pt09	ETV4	chr17:41622708	C	C/T	NM_001986.4	3	p.Glu30Lys	missense	155	19.35
Pt09	ZNF521	chr18:22642721	A	A/G	NM_015461.3	-	-	unknown	70	8.57
Pt09	ZNF521	chr18:22642722	A	A/G	NM_015461.3	-	-	unknown	70	10
Pt09	ZNF521	chr18:22642730	A	A/G	NM_015461.3	-	-	unknown	37	29.73
Pt09	ZNF521	chr18:22642735	A	A/G	NM_015461.3	-	-	unknown	25	24
Pt09	ZNF521	chr18:22642736	A	A/G	NM_015461.3	-	-	unknown	20	35
Pt09	DCC	chr18:50450106	C	C/T	NM_005215.4	4	p.Leu243=	synonymous	149	14.77
Pt09	DCC	chr18:50936977	G	G/A	NM_005215.4	20	p.Gly1031Arg	missense	65	12.31
Pt09	STK11	chr19:1219443	G	G/GCGG GGGC	NM_000455.4	-	-	unknown	7	85.71
Pt09	CEBPA	chr19:33792731	G	G/GGCG GGT	NM_004364.4	1	p.His195_Pro19 6dup	nonframeshiftI nsertion	15	33.33
Pt09	ERCC2	chr19:45872380	G	G/A	NM_000400.3	3	p.Ser44Leu	missense	113	25.66
Pt09	RUNX1	chr21:36164405	G	G/T	NM_001754.4	9	-	unknown	65	41.54
Pt09	BCR	chr22:23523630	C	C/A	NM_004327.4	1	p.Ile161=	synonymous	70	48.57
Pt09	KDM6A	chrX:44942855	T	T/C	NM_021140.3	23	-	unknown	104	41.35
Pt11	MTOR	chr1:11188506	C	C/A	NM_004958.4	-	-	unknown	74	62.16
Pt11	MUTYH	chr1:45798885	C	C/T	NM_001128425.2	-	-	unknown	163	54.6
Pt11	JAK1	chr1:65307269	C	C/T	NM_002227.4	18	p.Glu807Lys	missense	182	60.99
Pt11	TRIM33	chr1:114940209	G	G/T	NM_015906.4	20	-	unknown	47	23.4
Pt11	TRIM33	chr1:114969990	G	G/GA	NM_015906.4	-	-	unknown	31	22.58
Pt11	PDE4DIP	chr1:144855782	C	C/T	NM_001198834.4	41	p.Ala2257=	synonymous	108	23.15
Pt11	PDE4DIP	chr1:144855812	T	T/C	NM_001198834.4	41	p.Ser2247=	synonymous	106	40.57
Pt11	PDE4DIP	chr1:144994710	G	G/C	NM_001198834.4	1	p.Leu8Val	missense	710	12.11
Pt11	DDR2	chr1:162743418	G	T/T	NM_006182.4	-	-	unknown	257	100
Pt11	PBX1	chr1:164529120	G	G/A	NM_002585.4	1	p.Gly21Ser	missense	9	88.89
Pt11	CDC73	chr1:193219795	A	A/AT	NM_024529.5	-	-	unknown	22	13.64
Pt11	MTR	chr1:236972086	C	C/T	NM_000254.2	-	-	unknown	61	16.39
Pt11	MSH6	chr2:48032885	T	T/A	NM_000179.3	-	-	unknown	147	7.48
Pt11	SF3B1	chr2:198265477	C	C/T	NM_012433.4	18	p.Asp894Asn	missense	145	13.1
Pt11	SF3B1	chr2:198273283	T	T/C	NM_012433.4	8	p.Gly309=	synonymous	95	41.05
Pt11	PAX3	chr2:223158420	G	G/C	NM_181459.4	-	-	unknown	120	15.83
Pt11	RAF1	chr3:12641829	T	T/C	NM_002880.3	-	-	unknown	563	45.29
Pt11	MAGI1	chr3:65376868	G	G/T	NM_001033057.2	14	p.Pro789Thr	missense	132	13.64
Pt11	EPHB1	chr3:134670126	G	G/C	NM_004441.5	-	-	unknown	223	31.84
Pt11	FOXL2	chr3:138665106	GA	GA/TT	NM_023067.4	1	p.Phe153Ter	nonsense	127	10.24
Pt11	FGFR3	chr4:1807584	C	C/A	NM_000142.4	13	p.Pro585Thr	missense	102	6.86
Pt11	NSD2	chr4:1940139	C	C/T	NM_001042424.3	-	-	unknown	13	15.38
Pt11	PDGFRA	chr4:55136787	C	C/CT	NM_006206.6	-	-	unknown	122	9.02

Pt11	KIT	chr4:55569922	T	T/G	NM_000222.3	5	p.His263Gln	missense	87	12.64
Pt11	AFF1	chr4:88016160	A	A/G	NM_001166693.2	-	-	unknown	84	23.81
Pt11	FGFR4	chr5:176519567	G	G/T	NM_213647.3	-	-	unknown	100	15
Pt11	NSD1	chr5:176637240	G	G/T	NM_022455.4	5	p.Val614Leu	missense	74	9.46
Pt11	NSD1	chr5:176710838	T	T/C	NM_022455.4	20	p.Asn2020=	synonymous	210	22.38
Pt11	POU5F1	chr6:31137979	C	C/T	NM_002701.6	-	-	unknown	95	64.21
Pt11	PKHD1	chr6:51513956	A	A/G	NM_138694.4	62	p.Leu3746Pro	missense	176	5.11
Pt11	PKHD1	chr6:51712741	G	G/A	NM_138694.4	50	p.Pro2647Ser	missense	190	42.63
Pt11	ROS1	chr6:117641203	A	A/G	NM_002944.2	-	-	unknown	59	23.73
Pt11	TNFAIP3	chr6:138195961	TCTC	TCTC/T	NM_001270507.2	-	-	unknown	64	18.75
Pt11	SYNE1	chr6:152712752	C	C/T	NM_182961.4	-	-	unknown	73	38.36
Pt11	SYNE1	chr6:152720845	T	T/C	NM_182961.4	48	p.Ala2381=	synonymous	102	26.47
Pt11	AKAP9	chr7:91630620	G	G/T	NM_005751.4	8	p.Met463Ile	missense	111	50.45
Pt11	AKAP9	chr7:91641671	CA	CA/CAA	NM_005751.4	-	-	unknown	30	10
Pt11	AKAP9	chr7:91659190	C	C/CT	NM_005751.4	-	-	unknown	53	9.43
Pt11	CDK6	chr7:92247454	A	A/G	NM_001145306.2	7	p.Ser256Pro	missense	172	45.93
Pt11	TRRAP	chr7:98552765	G	G/A	NM_001244580.1	40	p.Ala1918=	synonymous	111	19.82
Pt11	EPHB4	chr7:100424586	CG	CG/C	NM_004444.5	-	-	unknown	36	100
Pt11	PIK3CG	chr7:106508520	G	G/C	NM_002649.3	2	p.Glu172Gln	missense	95	24.21
Pt11	MET	chr7:116397640	T	T/G	NM_001127500.3	-	-	unknown	85	24.71
Pt11	MET	chr7:116397665	T	T/G	NM_001127500.3	-	-	unknown	83	25.3
Pt11	MET	chr7:116397690	A	A/G	NM_001127500.3	8	-	unknown	67	31.34
Pt11	KMT2C	chr7:151875160	G	G/C	NM_170606.3	-	-	unknown	15	33.33
Pt11	ADGRA2	chr8:37699394	C	C/G	NM_032777.10	19	p.Arg1180Gly	missense	21	71.43
Pt11	UBR5	chr8:103308022	TT	TT/GG	NM_015902.6	-	-	unknown	244	45.49
Pt11	CSMD3	chr8:113395737	A	A/T	NM_198123.2	-	-	unknown	117	11.11
Pt11	TAF1L	chr9:32633325	G	G/A	NM_153809.2	1	p.Gly751=	synonymous	292	6.51
Pt11	RALGDS	chr9:135974079	G	G/C	NM_001271775.2	18	p.Leu879=	synonymous	13	84.62
Pt11	RALGDS	chr9:135974100	G	G/A	NM_001271775.2	18	p.Thr872=	synonymous	13	84.62
Pt11	NOTCH1	chr9:139391636	G	A/A	NM_017617.5	34	p.Asp2185=	synonymous	225	100
Pt11	NOTCH1	chr9:139400380	GC	GC/GCC	NM_017617.5	-	-	unknown	27	22.22
Pt11	NOTCH1	chr9:139407452	C	C/T	NM_017617.5	-	-	unknown	34	88.24
Pt11	RET	chr10:43604610	C	C/A	NM_020975.6	6	p.Pro399Thr	missense	191	12.57
Pt11	BLNK	chr10:97975169	A	A/G	NM_013314.4	-	-	unknown	77	6.49
Pt11	MRE11	chr11:94168955	A	A/T	NM_005591.4	-	-	unknown	310	8.06
Pt11	CCND2	chr12:4383158	T	C/C	NM_001759.4	1	-	unknown	18	100
Pt11	CDK4	chr12:58144445	C	C/T	NM_000075.4	5	p.Arg209His	missense	231	16.02
Pt11	HIF1A	chr14:62201051	C	C/CT	NM_001530.4	-	-	unknown	15	13.33
Pt11	TSHR	chr14:81574840	T	T/C	NM_000369.4	-	-	unknown	265	5.28
Pt11	TRIP11	chr14:92441066	C	C/T	NM_004239.4	19	p.Gly1827Ser	missense	22	22.73
Pt11	TRIP11	chr14:92491753	A	A/T	NM_004239.4	3	p.Leu71=	synonymous	149	68.46

Pt11	BCL11B	chr14:99642360	C	C/G	NM_138576.4	4	p.Pro271=	synonymous	69	27.54
Pt11	MYH11	chr16:15843898	C	CA/CA	NM_001040114.1	-	-	unknown	92	100
Pt11	CDH11	chr16:65016033	C	C/G	NM_001797.4	8	p.Glu391Gln	missense	188	9.04
Pt11	FANCA	chr16:89831243	C	C/A	NM_000135.4	-	-	unknown	12	41.67
Pt11	MAP2K4	chr17:11984633	A	A/G	NM_003010.4	-	-	unknown	97	34.02
Pt11	PGAP3	chr17:37829129	A	A/G	NM_033419.5	-	-	unknown	45	8.89
Pt11	ERBB2	chr17:37868208	C	C/A	NM_004448.3	8	p.Ser310Tyr	missense	167	22.75
Pt11	COL1A1	chr17:48267222	G	G/T	NM_000088.4	37	p.Pro871Thr	missense	67	32.84
Pt11	COL1A1	chr17:48277059	AGCCCC AGGCCC CAGGCC CCAG	A/AGCC CCAGGC CCCAG	NM_000088.4	-	-	unknown	10	A=80.00,AGCCCCAGGCCCC AG=20.00
Pt11	ZNF521	chr18:22642740	A	A/G	NM_015461.3	-	-	unknown	20	30
Pt11	ZNF521	chr18:22642742	A	A/G	NM_015461.3	-	-	unknown	9	33.33
Pt11	DCC	chr18:50683818	C	C/G	NM_005215.4	8	p.Pro452Ala	missense	109	38.53
Pt11	DCC	chr18:50936977	G	G/A	NM_005215.4	20	p.Gly1031Arg	missense	83	6.02
Pt11	STK11	chr19:1219274	G	A/A	NM_000455.5	-	-	unknown	35	100
Pt11	STK11	chr19:1219443	G	G/GCGG GGGC	NM_000455.5	-	-	unknown	22	13.64
Pt11	TCF3	chr19:1625558	G	A/A	NM_001136139.4	-	-	unknown	6	100
Pt11	FZR1	chr19:3533275	C	C/A	NM_001136198.1	-	-	unknown	191	6.28
Pt11	CD79A	chr19:42384978	G	G/T	NM_001783.4	5	p.Arg204=	synonymous	257	37.74
Pt11	ERCC2	chr19:45872377	C	C/G	NM_000400.4	3	p.Gly45Ala	missense	127	38.58
Pt11	ERCC2	chr19:45873775	C	C/T	NM_000400.4	-	-	unknown	169	38.46
Pt11	PPP2R1A	chr19:52719190	A	A/G	NM_014225.6	-	-	unknown	164	45.12
Pt11	ITGB2	chr21:46309312	G	G/A	NM_000211.5	13	p.Arg586Trp	missense	398	19.85
Pt11	MN1	chr22:28193696	T	T/C	NM_002430.3	1	p.Arg946Gly	missense	161	38.51
Pt11	EP300	chr22:41550938	A	A/AT	NM_001429.4	-	-	unknown	27	11.11
Pt12	DDR2	chr1:162743418	G	T/T	NM_006182.4	-	-	unknown	105	100
Pt12	MTR	chr1:237048563	T	T/A	NM_000254.2	-	-	unknown	88	5.68
Pt12	FN1	chr2:216226339	C	C/T	NM_212482.3	46	p.Cys2458Tyr	missense	77	5.19
Pt12	EPHA3	chr3:89521693	T	C/C	NM_005233.6	16	p.Trp924Arg	missense	18	100
Pt12	EPHA3	chr3:89521725	T	T/C	NM_005233.6	16	p.Gly934=	synonymous	20	15
Pt12	ATR	chr3:142188251	C	C/G	NM_001184.4	38	p.Leu2160Phe	missense	102	6.86
Pt12	PIK3CA	chr3:178936096	G	G/T	NM_006218.4	10	p.Gln546His	missense	180	36.67
Pt12	NSD2	chr4:1940139	C	C/T	NM_001042424.3	-	-	unknown	8	50
Pt12	KIT	chr4:55569922	T	T/G	NM_000222.3	5	p.His263Gln	missense	79	7.59
Pt12	IL6ST	chr5:55260184	G	G/GA	NM_002184.4	-	-	unknown	37	8.11
Pt12	CSF1R	chr5:149433596	TG	TG/GA	NM_005211.3	-	-	unknown	59	32.2
Pt12	PKHD1	chr6:51618174	A	A/G	NM_138694.4	-	-	unknown	84	5.95
Pt12	PKHD1	chr6:51640746	C	C/T	NM_138694.4	-	-	unknown	96	11.46

Pt12	PKHD1	chr6:51930868	T	T/C	NM_138694.4	12	p.Leu262=	synonymous	19	47.37
Pt12	SYNE1	chr6:152615232	C	C/G	NM_182961.4	94	p.Glu5905Gln	missense	75	20
Pt12	ETV1	chr7:14017126	TA	TA/TAA	NM_001163147.1	-	-	unknown	74	33.78
Pt12	ETV1	chr7:14025840	C	C/T	NM_001163147.1	-	-	unknown	94	22.34
Pt12	AKAP9	chr7:91700166	G	G/GA	NM_005751.4	-	-	unknown	57	5.26
Pt12	EPHB4	chr7:100424585	CCG	C/CC	NM_004444.5	-	-	unknown	36	C=25.00,CC=69.44,CCC=5.56
Pt12	KMT2C	chr7:151875160	G	G/C	NM_170606.3	-	-	unknown	17	52.94
Pt12	KMT2C	chr7:151877196	C	C/A	NM_170606.3	37	p.Glu2389Ter	nonsense	103	30.1
Pt12	KMT2C	chr7:151962257	C	C/T	NM_170606.3	8	p.Pro350=	synonymous	564	6.91
Pt12	KMT2C	chr7:151962265	C	C/T	NM_170606.3	8	p.Asp348Asn	missense	555	7.21
Pt12	WRN	chr8:31000243	T	T/C	NM_000553.6	-	-	unknown	21	57.14
Pt12	WRN	chr8:31004867	GT	GT/GTT	NM_000553.6	-	-	unknown	58	10.34
Pt12	ADGRA2	chr8:37693347	C	C/A	NM_032777.10	-	-	unknown	49	59.18
Pt12	NBN	chr8:90990596	A	A/AT	NM_002485.5	-	-	unknown	38	7.89
Pt12	RECQL4	chr8:145737040	C	C/T	NM_004260.4	-	-	unknown	74	62.16
Pt12	NUP214	chr9:134067560	T	T/C	NM_005085.4	-	-	unknown	14	28.57
Pt12	NOTCH1	chr9:139391636	G	A/A	NM_017617.5	34	p.Asp2185=	synonymous	183	100
Pt12	NOTCH1	chr9:139400380	G	G/GC	NM_017617.5	-	-	unknown	48	12.5
Pt12	NOTCH1	chr9:139401676	C	C/G	NM_017617.5	-	-	unknown	187	32.09
Pt12	SUFU	chr10:104386934	T	T/C	NM_016169.4	11	p.Ile433=	synonymous	60	5
Pt12	NUP98	chr11:3733839	C	C/T	NM_016320.5	20	p.Thr899=	synonymous	104	16.35
Pt12	KMT2A	chr11:118343801	A	A/G	NM_001197104.2	3	p.Ile643Val	missense	79	37.97
Pt12	CCND2	chr12:4383158	T	C/C	NM_001759.4	1	-	unknown	17	100
Pt12	KMT2D	chr12:49427012	G	G/A	NM_003482.4	40	p.Gln3826Ter	nonsense	46	32.61
Pt12	KMT2D	chr12:49431423	G	G/C	NM_003482.4	35	p.Ser3239Ter	nonsense	147	38.1
Pt12	EP400	chr12:132547090	A	A/G	NM_015409.5	47	p.Gln2726=	synonymous	22	27.27
Pt12	HIF1A	chr14:62200966	C	C/G	NM_001530.4	8	p.Gln331Glu	missense	97	8.25
Pt12	TRIP11	chr14:92454643	C	C/G	NM_004239.4	16	p.Glu1749Gln	missense	102	19.61
Pt12	IGF1R	chr15:99500725	TGG	TGG/T	NM_000875.5	21	-	unknown	64	T=10.94,TG=4.69,TGGG=0.00
Pt12	MYH11	chr16:15843898	C	CA/CA	NM_001040114.1	-	-	unknown	79	100
Pt12	FANCA	chr16:89882311	G	G/C	NM_000135.4	2	p.Gln55Glu	missense	106	7.55
Pt12	NF1	chr17:29657271	G	G/GA	NM_001042492.3	-	-	unknown	30	16.67
Pt12	ERBB2	chr17:37873542	G	G/GC	NM_004448.3	-	-	unknown	67	7.46
Pt12	DCC	chr18:50936994	T	T/C	NM_005215.4	20	p.Pro1036=	synonymous	74	63.51
Pt12	DCC	chr18:50937026	A	A/G	NM_005215.4	-	-	unknown	73	65.75
Pt12	TCF3	chr19:1619482	T	T/TG	NM_001136139.4	-	-	unknown	108	5.56
Pt12	GNA11	chr19:3119404	C	C/CG	NM_002067.5	-	-	unknown	26	100
Pt12	FZR1	chr19:3533275	C	C/A	NM_001136198.1	-	-	unknown	112	6.25
Pt12	SMARCA4	chr19:11141526	C	C/T	NM_001128849.3	25	p.Ala1168Val	missense	57	49.12

Pt12	CCNE1	chr19:30303508	C	C/CGGACG	NM_001238.4	-	-	unknown	48	37.5
Pt12	SRC	chr20:36031561	G	G/C	NM_198291.2	-	-	unknown	17	47.06
Pt12	RUNX1	chr21:36164700	T	T/C	NM_001754.4	9	p.Gln392Arg	missense	48	6.25
Pt12	BCR	chr22:23657735	G	G/A	NM_004327.4	23	-	unknown	37	8.11
Pt12	BCR	chr22:23657766	G	G/A	NM_004327.4	23	-	unknown	37	8.11
Pt12	EP300	chr22:41548219	G	G/T	NM_001429.4	16	p.Glu1003Ter	nonsense	110	11.82
Pt12	EP300	chr22:41550954	C	T/T	NM_001429.4	-	-	unknown	19	100
Pt12	TAF1	chrX:70679458	G	G/A	NM_004606.5	36	p.Gln1707=	synonymous	58	6.9
Pt12	ATRX	chrX:76778878	T	T/C	NM_000489.5	31	p.Asp2234Gly	missense	51	64.71
Pt13	PAX7	chr1:18960888	A	A/G	NM_002584.3	2	p.Ile59Met	missense	48	6.25
Pt13	PDE4DIP	chr1:144854180	T	T/C	NM_001198834.4	43	p.Ala2328=	synonymous	667	17.69
Pt13	PDE4DIP	chr1:144855782	C	C/T	NM_001198834.4	41	p.Ala2257=	synonymous	150	5.33
Pt13	PDE4DIP	chr1:144873887	C	C/T	NM_001198834.4	31	p.Gln1690=	synonymous	266	9.02
Pt13	PDE4DIP	chr1:144915561	G	G/A	NM_001198834.4	14	p.Arg622Ter	nonsense	312	5.77
Pt13	NTRK1	chr1:156845492	A	A/AGT	NM_002529.3	-	-	unknown	110	AGT=21.82,AGTGT=6.36,T=3.64
Pt13	DDR2	chr1:162743418	G	T/T	NM_006182.4	-	-	unknown	307	100
Pt13	MTR	chr1:237058762	C	C/A	NM_000254.2	31	p.Gly1170=	synonymous	203	11.33
Pt13	MSH6	chr2:48032885	T	T/A	NM_000179.3	-	-	unknown	214	10.28
Pt13	LRP1B	chr2:142567910	T	T/C	NM_018557.3	2	p.Gln48Arg	missense	25	72
Pt13	PAX3	chr2:223161889	A	G/G	NM_181459.4	2	p.Gly43=	synonymous	8	100
Pt13	RAF1	chr3:12626123	G	G/A	NM_002880.3	17	p.Leu613=	synonymous	224	22.32
Pt13	LTF	chr3:46480958	C	C/G	NM_002343.6	15	p.Glu579Asp	missense	182	23.08
Pt13	PBRM1	chr3:52620772	AA	AA/GC	NM_018313.5	-	-	unknown	78	14.1
Pt13	ATR	chr3:142231074	AAA	AAA/GCT	NM_001184.4	-	-	unknown	135	11.11
Pt13	ATR	chr3:142277575	A	A/T	NM_001184.4	8	p.Gly592=	synonymous	85	24.71
Pt13	BCL6	chr3:187442662	G	G/GCTCCACCTCCTTCCCTGCC	NM_001706.5	-	-	unknown	206	22.33
Pt13	NSD2	chr4:1940139	C	C/T	NM_001042424.3	-	-	unknown	11	36.36
Pt13	KIT	chr4:55569922	T	T/G	NM_000222.3	5	p.His263Gln	missense	126	7.14
Pt13	CSF1R	chr5:149433596	TG	TG/GA	NM_014983.3	-	-	unknown	97	24.74
Pt13	PKHD1	chr6:51618174	A	A/G	NM_138694.4	-	-	unknown	194	12.89
Pt13	DST	chr6:56476262	T	T/C	NM_001144769.5	-	-	unknown	208	53.85
Pt13	ADGRB3	chr6:70064164	G	G/A	NM_001704.3	27	p.Asp1167Asn	missense	252	9.92
Pt13	PRDM1	chr6:106547244	T	T/C	NM_001198.4	4	p.Tyr161His	missense	166	12.05
Pt13	ESR1	chr6:152129484	C	C/A	NM_001122740.1	2	p.Pro146Gln	missense	8	37.5
Pt13	SYNE1	chr6:152712752	C	C/T	NM_182961.4	-	-	unknown	156	32.69

Pt13	PMS2	chr7:6026496	G	G/T	NM_000535.7	11	p.His634Asn	missense	218	21.1
Pt13	AKAP9	chr7:91630620	G	G/T	NM_005751.4	8	p.Met463Ile	missense	173	31.21
Pt13	AKAP9	chr7:91667692	T	T/G	NM_005751.4	-	-	unknown	124	54.84
Pt13	AKAP9	chr7:91671973	C	C/G	NM_005751.4	-	-	unknown	197	13.2
Pt13	BRAF	chr7:140434586	G	G/A	NM_004333.6	-	-	unknown	51	23.53
Pt13	BRAF	chr7:140434597	G	G/A	NM_004333.6	-	-	unknown	38	52.63
Pt13	BRAF	chr7:140434600	A	A/G	NM_004333.6	-	-	unknown	40	10
Pt13	BRAF	chr7:140434611	AAAG	AAAG/A	NM_004333.6	-	-	unknown	32	A=34.38,AA=18.75,AAAA=12.50
Pt13	BRAF	chr7:140434619	A	A/G	NM_004333.6	-	-	unknown	35	14.29
Pt13	BRAF	chr7:140434620	A	A/G	NM_004333.6	-	-	unknown	35	42.86
Pt13	KMT2C	chr7:151833925	T	T/C	NM_170606.3	59	p.Met4910Val	missense	199	14.57
Pt13	KMT2C	chr7:151962257	C	C/T	NM_170606.3	8	p.Pro350=	synonymous	903	9.63
Pt13	KMT2C	chr7:151962265	C	C/T	NM_170606.3	8	p.Asp348Asn	missense	891	9.76
Pt13	WRN	chr8:30924738	A	A/T	NM_000553.6	-	-	unknown	39	30.77
Pt13	ADGRA2	chr8:37693347	C	C/A	NM_032777.10	-	-	unknown	72	47.22
Pt13	UBR5	chr8:103271151	G	G/T	NM_015902.6	-	-	unknown	207	19.32
Pt13	CSMD3	chr8:113569047	G	G/A	NM_198123.2	25	p.Leu1393=	synonymous	206	14.56
Pt13	PTPRD	chr9:8338847	CAGAGAGAGAGAGAGAGAG	CAGAGAGAGAGAGAGA/CAGAGAGAGAGAGAGA	NM_002839.4	-	-	unknown	102	100
Pt13	PTPRD	chr9:8518470	C	C/G	NM_002839.4	-	-	unknown	56	26.79
Pt13	TAF1L	chr9:32633325	G	G/A	NM_153809.2	1	p.Gly751=	synonymous	166	7.23
Pt13	NOTCH1	chr9:139391636	G	A/A	NM_017617.5	34	p.Asp2185=	synonymous	161	100
Pt13	NUP98	chr11:3721767	C	C/T	NM_016320.5	-	-	unknown	125	13.6
Pt13	WT1	chr11:32456562	G	G/A	NM_024426.6	1	p.Pro115=	synonymous	7	57.14
Pt13	CCND2	chr12:4383158	T	T/C	NM_001759.4	1	-	unknown	26	96.15
Pt13	ADAMTS20	chr12:43792797	G	G/GA	NM_025003.5	-	-	unknown	30	16.67
Pt13	ADAMTS20	chr12:43856797	T	T/C	NM_025003.5	11	p.Ile505Met	missense	337	24.33
Pt13	HNF1A	chr12:121437382	A	G/G	NM_000545.8	9	p.Ser574Gly	missense	7	100
Pt13	IRS2	chr13:110436232	G	G/A	NM_003749.3	1	p.Ser723=	synonymous	12	41.67
Pt13	NIN	chr14:51190230	C	C/G	NM_020921.3	31	p.Arg2118Thr	missense	163	26.99
Pt13	TSHR	chr14:81574844	TT	TT/AC	NM_000369.4	-	-	unknown	305	12.13
Pt13	HSP90AA1	chr14:102568296	G	A/A	NM_001017963.3	2	p.Thr94=	synonymous	36	100
Pt13	TP53	chr17:7579312	C	C/A	NM_000546.5	4	p.Thr125=	synonymous	146	26.03
Pt13	ZNF521	chr18:22642722	A	A/G	NM_015461.3	-	-	unknown	129	27.91

Pt13	ZNF521	chr18:22642723	A	A/G	NM_015461.3	-	-	unknown	127	13.39
Pt13	ZNF521	chr18:22642726	A	A/G	NM_015461.3	-	-	unknown	108	17.59
Pt13	ZNF521	chr18:22642727	A	A/G	NM_015461.3	-	-	unknown	99	7.07
Pt13	ZNF521	chr18:22642728	A	A/G	NM_015461.3	-	-	unknown	94	22.34
Pt13	ZNF521	chr18:22642729	A	A/G	NM_015461.3	-	-	unknown	88	13.64
Pt13	ZNF521	chr18:22642730	A	A/G	NM_015461.3	-	-	unknown	78	16.67
Pt13	DCC	chr18:50936994	T	T/C	NM_005215.4	20	p.Pro1036=	synonymous	236	48.73
Pt13	DCC	chr18:50937026	A	A/G	NM_005215.4	-	-	unknown	237	48.52
Pt13	MALT1	chr18:56390331	C	C/G	NM_006785.4	10	p.Pro357Arg	missense	69	15.94
Pt13	PIK3R2	chr19:18271819	G	G/C	NM_005027.4	-	-	unknown	132	30.3
Pt13	AXL	chr19:41762463	T	T/C	NM_021913.5	18	p.Trp715Arg	missense	156	14.74
Pt13	ERCC2	chr19:45872197	C	C/G	NM_000400.4	4	p.Glu79Asp	missense	153	29.41
Pt13	PPP2R1A	chr19:52719190	A	A/G	NM_014225.6	-	-	unknown	219	30.14
Pt13	RUNX1	chr21:36252849	C	C/G	NM_001754.4	-	-	unknown	183	9.29
Pt13	EP300	chr22:41550954	C	T/T	NM_001429.4	-	-	unknown	11	100
Pt13	USP9X	chrX:40999853	C	A/A	NM_001039590.3	-	-	unknown	69	100
Pt13	KDM6A	chrX:44942036	T	T/G	NM_021140.3	22	-	unknown	112	25.89
Pt13	TAF1	chrX:70613074	TTGTGTG	TTGTGTG/TTG	NM_004606.5	-	-	unknown	9	T=11.11,TTG=55.56
Pt14	NRAS	chr1:115251191	G	G/A	NM_002524.5	5	p.Gln179Ter	nonsense	86	9.3
Pt14	MUC1	chr1:155162067	C	T/T	NM_001204285.2	2	p.Thr22=	synonymous	72	100
Pt14	PARP1	chr1:226570841	T	T/C	NM_001618.4	8	p.Lys352Arg	missense	142	6.34
Pt14	MSH6	chr2:48032885	T	T/A	NM_000179.2	-	-	unknown	81	6.17
Pt14	LRP1B	chr2:141528653	G	G/C	NM_018557.2	-	-	unknown	97	5.15
Pt14	GATA2	chr3:128200806	G	G/A	NM_032638.4	-	-	unknown	32	46.88
Pt14	PIK3CA	chr3:178916876	G	G/C	NM_006218.4	2	p.Arg88Pro	missense	91	40.66
Pt14	FGFR3	chr4:1808286	G	G/A	NM_000142.4	16	p.Val682Ile	missense	39	46.15
Pt14	NSD2	chr4:1940128	C	C/T	NM_001042424.3	-	-	unknown	20	20
Pt14	NSD2	chr4:1940133	T	T/C	NM_001042424.3	-	-	unknown	21	14.29
Pt14	NSD2	chr4:1940148	T	T/C	NM_001042424.3	-	-	unknown	22	27.27
Pt14	FBXW7	chr4:153268263	G	G/A	NM_033632.3	-	-	unknown	50	6
Pt14	LIFR	chr5:38504305	A	A/T	NM_002310.6	-	-	unknown	108	7.41
Pt14	FLT4	chr5:180045700	G	GGGTTACCCTA/GGGTTACCCTA	NM_182925.5	-	-	unknown	8	100

Pt14	FLT4	chr5:180046426	C	CTGGCC GCTTAG CTAAGG CACAG/ CTGGCC GCTTAG CTAAGG CACAG	NM_182925.5	-	-	unknown	28	100
Pt14	FOXO3	chr6:108985092	C	C/G	NM_001455.4	2	p.Ala352=	synonymous	65	6.15
Pt14	ROS1	chr6:117710552	G	G/C	NM_002944.2	12	p.Leu574Val	missense	92	32.61
Pt14	TNFAIP3	chr6:138195961	TCTC	T/T	NM_001270507.2	-	-	unknown	14	100
Pt14	SYNE1	chr6:152532732	TA	TA/T	NM_182961.4	-	-	unknown	41	34.15
Pt14	SYNE1	chr6:152793575	A	A/G	NM_182961.4	-	-	unknown	6	83.33
Pt14	RPS6KA2	chr6:166826405	G	G/A	NM_001006932.3	-	-	unknown	47	6.38
Pt14	BRAF	chr7:140434586	G	G/A	NM_004333.6	-	-	unknown	41	24.39
Pt14	BRAF	chr7:140434602	A	A/G	NM_004333.6	-	-	unknown	25	16
Pt14	BRAF	chr7:140434614	GAAAAA A	AAAAA GA/GAA AAAAA	NM_004333.6	-	-	unknown	19	AAAAAGA=47.37,GAAAAA AA=52.63,GAAAAAAAAG=0 .00
Pt14	KMT2C	chr7:151882735	G	G/A	NM_170606.3	-	-	unknown	167	14.37
Pt14	KMT2C	chr7:151945140	C	C/T	NM_170606.3	14	p.Ser793=	synonymous	88	5.68
Pt14	KMT2C	chr7:151945167	G	G/T	NM_170606.3	14	p.Ser784=	synonymous	86	5.81
Pt14	KMT2C	chr7:151962200	C	C/T	NM_170606.3	8	p.Met369Ile	missense	422	9
Pt14	KMT2C	chr7:151962257	C	C/T	NM_170606.3	8	p.Pro350=	synonymous	402	7.96
Pt14	KMT2C	chr7:151962265	C	C/T	NM_170606.3	8	p.Asp348Asn	missense	393	7.89
Pt14	KMT2C	chr7:151970951	C	C/T	NM_170606.3	7	p.Arg284Gln	missense	175	5.71
Pt14	KMT2C	chr7:151970959	G	G/A	NM_170606.3	-	-	unknown	178	5.06
Pt14	KMT2C	chr7:151971006	C	C/T	NM_170606.3	-	-	unknown	179	6.15
Pt14	KMT2C	chr7:151971015	G	G/A	NM_170606.3	-	-	unknown	182	10.99
Pt14	RECQL4	chr8:145739707	C	C/T	NM_004260.3	11	p.Glu582Lys	missense	65	26.15
Pt14	TLX1	chr10:102894177	T	T/A	NM_005521.4	-	-	unknown	36	8.33
Pt14	FGFR2	chr10:123244924	T	T/C	NM_000141.4	16	p.Asn727Ser	missense	16	18.75
Pt14	KMT2A	chr11:118348873	A	A/T	NM_001197104.1	5	p.Lys1176Ter	nonsense	25	60
Pt14	CCND2	chr12:4383158	T	C/C	NM_001759.4	1	-	unknown	17	100
Pt14	KMT2D	chr12:49433003	A	A/C	NM_003482.3	33	-	unknown	116	34.48
Pt14	IRS2	chr13:110435231	C	C/T	NM_003749.3	1	p.Gly1057Asp	missense	26	46.15
Pt14	IRS2	chr13:110435953	A	A/G	NM_003749.3	1	p.Cys816=	synonymous	119	50.42
Pt14	IRS2	chr13:110436232	G	G/A	NM_003749.3	1	p.Ser723=	synonymous	10	50
Pt14	TSHR	chr14:81574844	TT	TT/AC	NM_000369.3	-	-	unknown	159	6.92

Pt14	BCL11B	chr14:99642288	C	C/G	NM_138576.4	4	p.Thr295=	synonymous	14	35.71
Pt14	BCL11B	chr14:99642360	C	C/G	NM_138576.4	4	p.Pro271=	synonymous	34	32.35
Pt14	MAF	chr16:79633805	TGCC	T/T	NM_005360.5	1	-	unknown	8	100
Pt14	BRIP1	chr17:59934523	G	G/C	NM_032043.2	4	p.Ser92Ter	nonsense	60	31.67
Pt14	SEPTIN9	chr17:75369519	GCCCCG	GCCCCG /GCCCG	NM_001113491.2	-	-	unknown	37	GCCCG=72.97,GCCCGC=5.4 1,GCCG=8.11
Pt14	ZNF521	chr18:22642721	A	A/G	NM_015461.3	-	-	unknown	35	14.29
Pt14	ZNF521	chr18:22642722	A	A/G	NM_015461.3	-	-	unknown	35	22.86
Pt14	MBD1	chr18:47801499	C	C/T	NM_001204136.1	9	p.Pro303=	synonymous	33	9.09
Pt14	DCC	chr18:50936977	G	G/A	NM_005215.4	20	p.Gly1031Arg	missense	41	7.32
Pt14	BCL2	chr18:60985879	T	T/C	NM_000633.2	2	p.Thr7=	synonymous	63	36.51
Pt14	TCF3	chr19:1650134	A	G/G	NM_001136139.4	-	-	unknown	14	100
Pt14	RUNX1	chr21:36164405	G	G/T	NM_001754.4	9	-	unknown	34	20.59
Pt14	BCR	chr22:23523630	C	C/A	NM_004327.4	1	p.Ile161=	synonymous	70	37.14
Pt16	PLEKHG5	chr1:6528469	G	G/A	NM_001265593.1	20	p.Asp878=	synonymous	84	19.05
Pt16	PIK3CD	chr1:9784307	C	C/T	NM_005026.5	-	-	unknown	74	22.97
Pt16	PDE4DIP	chr1:144855782	C	C/T	NM_001198834.3	41	p.Ala2257=	synonymous	69	7.25
Pt16	NTRK1	chr1:156844182	C	C/G	NM_002529.3	9	p.Phe395Leu	missense	136	8.09
Pt16	DNMT3A	chr2:25505279	G	G/A	NM_022552.4	-	-	unknown	83	10.84
Pt16	ALK	chr2:29541191	C	C/T	NM_004304.5	8	p.Pro542=	synonymous	196	19.9
Pt16	CRBN	chr3:3196442	G	G/A	NM_016302.3	7	p.Ser275Leu	missense	191	13.09
Pt16	BAP1	chr3:52437846	C	C/T	NM_004656.4	13	p.Val439Met	missense	170	23.53
Pt16	PIK3CA	chr3:178936082	G	G/A	NM_006218.4	10	p.Glu542Lys	missense	211	5.21
Pt16	KIT	chr4:55569922	T	T/G	NM_000222.2	5	p.His263Gln	missense	113	5.31
Pt16	KIT	chr4:55570026	C	C/T	NM_000222.2	5	p.Ser298Leu	missense	218	6.88
Pt16	KIT	chr4:55598189	A	A/AT	NM_000222.2	-	-	unknown	67	5.97
Pt16	KIT	chr4:55598217	T	T/G	NM_000222.2	-	-	unknown	35	11.43
Pt16	NFKB1	chr4:103498169	G	G/C	NM_003998.4	7	p.Glu182Gln	missense	144	5.56
Pt16	FBXW7	chr4:153271176	C	C/T	NM_033632.3	-	-	unknown	110	8.18
Pt16	IL6ST	chr5:55243426	A	A/G	NM_002184.4	-	-	unknown	51	9.8
Pt16	CSF1R	chr5:149440501	C	C/T	NM_005211.3	14	p.Met631Ile	missense	124	6.45
Pt16	DAXX	chr6:33286507	G	G/A	NM_001145338.1	-	-	unknown	195	5.13
Pt16	PKHD1	chr6:51612611	G	G/C	NM_138694.4	58	p.Ser3268Ter	nonsense	96	6.25
Pt16	ROS1	chr6:117706963	C	C/T	NM_002944.2	15	p.Trp729Ter	nonsense	225	7.56
Pt16	CARD11	chr7:2972136	A	A/T	NM_032415.6	-	-	unknown	58	6.9
Pt16	EPHB4	chr7:100401278	C	C/T	NM_004444.5	-	-	unknown	92	6.52
Pt16	POT1	chr7:124532335	G	G/C	NM_015450.3	6	p.Leu37Val	missense	98	8.16
Pt16	KMT2C	chr7:151882735	G	G/A	NM_170606.3	-	-	unknown	191	5.24
Pt16	PRKDC	chr8:48855876	G	G/A	NM_006904.6	10	p.Leu287=	synonymous	112	7.14
Pt16	CSMD3	chr8:113275803	C	C/T	NM_198123.2	-	-	unknown	123	7.32
Pt16	RECQL4	chr8:145742046	C	C/T	NM_004260.3	5	p.Glu153Lys	missense	140	6.43

Pt16	TAF1L	chr9:32632578	T	T/C	NM_153809.2	1	p.Pro1000=	synonymous	30	10
Pt16	TAF1L	chr9:32633325	G	G/A	NM_153809.2	1	p.Gly751=	synonymous	109	7.34
Pt16	NUP214	chr9:134067569	T	T/A	NM_005085.4	-	-	unknown	28	25
Pt16	FAS	chr10:90773980	G	G/A	NM_000043.6	9	p.Glu261Lys	missense	228	12.28
Pt16	ATM	chr11:108143620	G	G/A	NM_000051.3	-	-	unknown	91	8.79
Pt16	KMT2D	chr12:49426526	G	G/A	NM_003482.3	39	p.Gln3988Ter	nonsense	125	12
Pt16	KMT2D	chr12:49427631	G	G/T	NM_003482.3	39	p.Leu3619=	synonymous	40	17.5
Pt16	ERBB3	chr12:56493405	T	T/A	NM_001982.3	-	-	unknown	107	8.41
Pt16	KNL1	chr15:40943768	G	G/A	NM_144508.5	20	p.Leu2104=	synonymous	94	10.64
Pt16	IDH2	chr15:90628131	C	C/G	NM_002168.3	10	p.Gln396His	missense	90	5.56
Pt16	IGF1R	chr15:99192744	C	C/T	NM_000875.5	1	-	unknown	35	14.29
Pt16	CREBBP	chr16:3781338	C	C/T	NM_004380.3	30	p.Trp1676Ter	nonsense	95	18.95
Pt16	MAF	chr16:79633805	TGCC	T/T	NM_005360.5	1	-	unknown	30	100
Pt16	TP53	chr17:7576828	G	G/C	NM_000546.5	-	-	unknown	84	13.1
Pt16	TP53	chr17:7577518	TGATGG<br>TGAG	TGATGG<br>TGAG/T<br>AATGGT<br>GAG	NM_000546.5	7	p.Ile254=	synonymous	97	8.25
Pt16	TP53	chr17:7578368	CACCAT<br>CGCTAT<br>CTGAGC<br>AGCGCT<br>CATGGT<br>GGGGGC<br>AGCGCC<br>TCACA	CACCAT<br>CGCTAT<br>CTGAGC<br>AGCGCT<br>CATGGT<br>GGGGG<br>CAGCGC<br>CTCACA<br>/CACCA<br>TCGCTA<br>TCTCAG<br>CAGCGC<br>TCATGG<br>TGGGG<br>GCAGC<br>GCCTCA<br>CA	NM_000546.5	5	p.Ser183Ter	nonsense	62	C=0.00,CACCATCGCTATCT<br>CAGCAGCGCTCATGGTGG<br>GGGCAGCGCCTCACA=12.9<br>0,CACCATCGCTATCTGAGC<br>AGCGCTCATGGTGGGGGG<br>CAGCGCCTCACA=1.61,GAC<br>CATCGCTATCTGAGCAGCG<br>CTCATGGTGGGGGCAGCG<br>CCTCACA=0.00
Pt16	TP53	chr17:7579873	G	G/C	NM_000546.5	2	p.Leu14Val	missense	87	11.49
Pt16	ZNF521	chr18:22642722	A	A/G	NM_015461.3	-	-	unknown	97	9.28
Pt16	ZNF521	chr18:22642726	A	A/G	NM_015461.3	-	-	unknown	77	5.19
Pt16	ZNF521	chr18:22642727	A	A/G	NM_015461.3	-	-	unknown	76	5.26

Pt16	DCC	chr18:50936977	G	G/A	NM_005215.4	20	p.Gly1031Arg	missense	49	6.12
Pt16	PIK3R2	chr19:18273972	G	G/C	NM_005027.4	-	-	unknown	42	42.86
Pt16	RUNX1	chr21:36193960	C	C/G	NM_001754.4	-	-	unknown	204	18.14
Pt16	CRKL	chr22:21272502	G	G/C	NM_005207.4	1	p.Asp94His	missense	39	15.38
Pt16	AR	chrX:66942740	C	C/G	NM_000044.6	7	p.Arg841Gly	missense	200	11
Pt17	ITGA10	chr1:145535984	C	C/T	NM_001278267.1	-	-	unknown	131	17.56
Pt17	TPR	chr1:186330701	A	A/G	NM_003292.3	-	-	unknown	57	8.77
Pt17	MTR	chr1:237024404	T	T/C	NM_000254.2	-	-	unknown	16	37.5
Pt17	SOX11	chr2:5833525	GGAC	GGAC/G	NM_003108.4	1	p.Asp233del	nonframeshiftDeletion	24	12.5
Pt17	MSH6	chr2:48032885	T	T/A	NM_000179.2	-	-	unknown	94	5.32
Pt17	LRP1B	chr2:141597707	G	G/A	NM_018557.2	-	-	unknown	136	14.71
Pt17	PBRM1	chr3:52643360	C	C/A	NM_018313.5	17	p.Glu846Ter	nonsense	85	24.71
Pt17	ROS1	chr6:117706963	C	C/T	NM_002944.2	15	p.Trp729Ter	nonsense	147	6.12
Pt17	IKZF1	chr7:50468269	C	C/T	NM_006060.6	8	p.Arg502Trp	missense	97	19.59
Pt17	TRRAP	chr7:98509702	C	C/G	NM_001244580.1	18	p.Leu689Val	missense	77	18.18
Pt17	NUP214	chr9:134014660	T	T/C	NM_005085.4	-	-	unknown	75	10.67
Pt17	FGFR2	chr10:123353266	C	C/A	NM_000141.4	2	p.Arg22=	synonymous	101	6.93
Pt17	ATM	chr11:108203474	G	G/GT	NM_000051.3	-	-	unknown	88	GT=6.82,T=1.14
Pt17	NF1	chr17:29563077	T	T/G	NM_001042492.2	-	-	unknown	91	7.69
Pt17	ZNF521	chr18:22642722	A	A/G	NM_015461.3	-	-	unknown	57	26.32
Pt17	ZNF521	chr18:22642729	A	A/G	NM_015461.3	-	-	unknown	37	24.32
Pt17	ZNF521	chr18:22642732	A	A/G	NM_015461.3	-	-	unknown	28	35.71
Pt17	EP300	chr22:41550954	C	C/T	NM_001429.4	-	-	unknown	48	8.33
Pt17	EP300	chr22:41551021	A	A/G	NM_001429.4	17	p.Arg1055=	synonymous	48	6.25
Pt17	KDM6A	chrX:44938424	A	A/T	NM_021140.3	20	p.Glu991Val	missense	69	28.99
Pt17	KDM5C	chrX:53224232	C	C/G	NM_004187.4	22	p.Asp1107His	missense	99	19.19
Pt17	TAF1	chrX:70612769	A	A/G	NM_004606.4	20	p.Thr1012=	synonymous	56	5.36
Pt17	TAF1	chrX:70679458	G	G/A	NM_004606.4	36	p.Gln1727=	synonymous	104	6.73
Pt18	FANCD2	chr3:10088343	A	A/G	NM_033084.5	15	p.Asn405Ser	missense	157	8.28
Pt18	KIT	chr4:55569922	T	T/G	NM_000222.2	5	p.His263Gln	missense	84	5.95
Pt18	IL6ST	chr5:55243431	A	A/G	NM_002184.4	-	-	unknown	30	26.67
Pt18	ROS1	chr6:117706963	C	C/T	NM_002944.2	15	p.Trp729Ter	nonsense	156	9.62
Pt18	CARD11	chr7:2972136	A	A/T	NM_032415.6	-	-	unknown	65	6.15
Pt18	KMT2C	chr7:151875122	A	A/T	NM_170606.3	-	-	unknown	27	14.81
Pt18	KMT2C	chr7:151948998	T	T/A	NM_170606.3	-	-	unknown	114	6.14
Pt18	KMT2C	chr7:151970951	C	C/T	NM_170606.3	7	p.Arg284Gln	missense	120	5.83
Pt18	KMT2C	chr7:151971006	C	C/T	NM_170606.3	-	-	unknown	123	6.5
Pt18	RECQL4	chr8:145737040	C	C/T	NM_138431.3	-	-	unknown	30	50
Pt18	TAF1L	chr9:32633325	G	G/A	NM_153809.2	1	p.Gly751=	synonymous	122	6.56
Pt18	FGFR2	chr10:123244924	T	T/C	NM_000141.4	16	p.Asn727Ser	missense	50	6
Pt18	ZNF521	chr18:22642726	A	A/G	NM_015461.3	-	-	unknown	52	9.62

Pt18	MYH9	chr22:36702640	G	G/A	NM_002473.5	16	p.Ile619=	synonymous	55	5.45
Pt21	TRIM33	chr1:114964254	G	G/A	NM_015906.4	11	p.Ser622Leu	missense	44	40.91
Pt21	PDE4DIP	chr1:144854180	T	T/C	NM_001198834.4	43	p.Ala2328=	synonymous	104	5.77
Pt21	ARNT	chr1:150789868	C	C/G	NM_001668.4	16	p.Arg516Thr	missense	42	40.48
Pt21	NTRK1	chr1:156834578	C	C/T	NM_002529.3	3	p.Arg116Trp	missense	41	7.32
Pt21	MTR	chr1:237038006	TTT	TTT/TAA	NM_000254.2	-	-	unknown	28	82.14
Pt21	LRP1B	chr2:141264386	A	A/G	NM_018557.3	53	p.Ser2834Pro	missense	47	6.38
Pt21	PPARG	chr3:12434114	G	G/C	NM_015869.5	4	p.Gly161Ala	missense	49	26.53
Pt21	MAGI1	chr3:65422816	G	G/A	NM_001033057.2	-	-	unknown	59	32.2
Pt21	PDGFRA	chr4:55161227	G	G/A	NM_006206.6	-	-	unknown	46	6.52
Pt21	FBXW7	chr4:153247289	G	G/C	NM_033632.3	10	p.Arg505Gly	missense	41	14.63
Pt21	IL6ST	chr5:55265435	T	T/C	NM_002184.4	4	p.Ile105Val	missense	76	6.58
Pt21	PKHD1	chr6:51618174	A	A/G	NM_138694.4	-	-	unknown	69	5.8
Pt21	DST	chr6:56426884	TA	TA/TAA	NM_001144769.5	-	-	unknown	20	10
Pt21	ROS1	chr6:117678087	G	G/GA	NM_002944.2	-	-	unknown	30	10
Pt21	SYNE1	chr6:152631788	AT	AT/A	NM_182961.4	-	-	unknown	33	9.09
Pt21	IGF2R	chr6:160465696	A	A/AAT	NM_000876.3	-	-	unknown	29	10.34
Pt21	MET	chr7:116436022	G	G/A	NM_001127500.3	21	p.Ala1357=	synonymous	31	22.58
Pt21	GRM8	chr7:126086158	G	G/A	NM_000845.3	-	-	unknown	74	22.97
Pt21	KMT2C	chr7:151882672	C	C/A	NM_170606.3	34	p.Ala1685Ser	missense	115	8.7
Pt21	KMT2C	chr7:151962257	C	C/T	NM_170606.3	8	p.Pro350=	synonymous	286	9.79
Pt21	KMT2C	chr7:151962265	C	C/T	NM_170606.3	8	p.Asp348Asn	missense	283	9.54
Pt21	UBR5	chr8:103292625	G	G/C	NM_015902.6	42	p.Gln2000Glu	missense	88	34.09
Pt21	UBR5	chr8:103292712	G	G/A	NM_015902.6	-	-	unknown	90	35.56
Pt21	MAPK8	chr10:49609689	T	T/G	NM_139049.4	4	-	unknown	37	32.43
Pt21	NUMA1	chr11:71716271	G	G/A	NM_006185.4	-	-	unknown	23	43.48
Pt21	NUMA1	chr11:71726807	T	T/C	NM_006185.4	15	p.His581Arg	missense	15	33.33
Pt21	ARID2	chr12:46287170	TTC	TTC/T	NM_152641.4	-	-	unknown	20	15
Pt21	IDH2	chr15:90630723	G	G/T	NM_002168.4	6	p.Leu255Met	missense	39	28.21
Pt21	MMP2	chr16:55525913	G	C/C	NM_004530.6	-	-	unknown	7	100
Pt21	FANCA	chr16:89805498	T	T/TG	NM_001113525.2	-	-	unknown	16	12.5
Pt21	ERBB2	chr17:37865590	C	C/T	NM_004448.3	4	p.Val153=	synonymous	61	32.79
Pt21	COL1A1	chr17:48266160	CAG	CAG/C	NM_000088.4	-	-	unknown	58	15.52
Pt21	DCC	chr18:50936977	G	G/A	NM_005215.4	20	p.Gly1031Arg	missense	22	13.64
Pt21	GNAS	chr20:57428722	C	C/T					33	51.52
Pt21	RUNX1	chr21:36206960	G	G/GA	NM_001754.4	-	-	unknown	25	12
Pt24	DPYD	chr1:98187262	TA	TA/T	NM_000110.4	-	-	unknown	19	10.53
Pt24	PDE4DIP	chr1:144854180	T	T/C	NM_001198834.4	43	p.Ala2328=	synonymous	147	6.12
Pt24	PDE4DIP	chr1:144873887	C	C/T	NM_001198834.4	31	p.Gln1690=	synonymous	108	5.56
Pt24	ARNT	chr1:150786575	G	G/C	NM_001668.4	20	p.Leu697=	synonymous	118	14.41

Pt24	TPR	chr1:186313082	G	G/A	NM_003292.3	26	p.Leu1186=	synonymous	54	53.7
Pt24	MTR	chr1:237048563	T	T/A	NM_000254.2	-	-	unknown	74	5.41
Pt24	FN1	chr2:216298167	C	C/G	NM_212482.3	3	p.Asp99His	missense	45	51.11
Pt24	ITGA9	chr3:37559152	C	C/T	NM_002207.3	-	-	unknown	36	41.67
Pt24	ATR	chr3:142272458	G	G/A	NM_001184.4	-	-	unknown	76	14.47
Pt24	FGFR3	chr4:1803564	C	C/T	NM_000142.4	7	p.Arg248Cys	missense	35	60
Pt24	FGFR3	chr4:1806583	C	C/T	NM_000142.4	10	p.Ser433=	synonymous	71	43.66
Pt24	PDGFRA	chr4:55144063	C	C/A	NM_006206.6	14	p.Pro631His	missense	42	7.14
Pt24	KIT	chr4:55569922	T	T/G	NM_000222.3	5	p.His263Gln	missense	39	7.69
Pt24	FLT4	chr5:180057230	G	G/A	NM_182925.5	4	p.Arg170Cys	missense	16	25
Pt24	SYNE1	chr6:152639218	G	G/C	NM_182961.4	86	p.Gln5524Glu	missense	21	47.62
Pt24	AKAP9	chr7:91712808	G	G/C	NM_005751.4	33	p.Glu2829Gln	missense	33	36.36
Pt24	AKAP9	chr7:91718745	G	G/A	NM_005751.4	38	p.Arg3087Lys	missense	56	26.79
Pt24	BRAF	chr7:140454024	G	G/A	NM_004333.6	14	p.His568=	synonymous	125	40.8
Pt24	KMT2C	chr7:151882672	C	C/A	NM_170606.3	34	p.Ala1685Ser	missense	110	5.45
Pt24	KMT2C	chr7:151882735	G	G/A	NM_170606.3	-	-	unknown	110	5.45
Pt24	ABL1	chr9:133747498	GT	GT/G	NM_005157.6	-	-	unknown	30	10
Pt24	ATM	chr11:108122786	GT	GT/GTT	NM_000051.3	-	-	unknown	22	22.73
Pt24	KMT2A	chr11:118345001	C	C/G	NM_001197104.2	3	p.Leu1043Val	missense	28	64.29
Pt24	MDM2	chr12:69207452	T	T/C	NM_002392.5	-	-	unknown	47	6.38
Pt24	TP53	chr17:7578236	A	A/T	NM_000546.5	6	p.Tyr205Asn	missense	32	78.13
Pt24	ITGB3	chr17:45367012	G	G/A	NM_000212.3	-	-	unknown	32	37.5
Pt24	AURKA	chr20:54948645	G	G/C	NM_003600.4	-	-	unknown	48	56.25
Pt25	PDE4DIP	chr1:144873887	C	C/T	NM_001198834.4	31	p.Gln1690=	synonymous	71	5.63
Pt25	PIK3C2B	chr1:204400784	A	A/G	NM_002646.4	-	-	unknown	40	7.5
Pt25	REL	chr2:61143993	A	A/T	NM_002908.4	-	-	unknown	36	5.56
Pt25	LTF	chr3:46480958	C	C/G	NM_002343.6	15	p.Glu579Asp	missense	20	55
Pt25	GATA2	chr3:128202753	G	G/A	NM_032638.5	4	p.His323Tyr	missense	34	41.18
Pt25	GATA2	chr3:128202760	G	G/C	NM_032638.5	4	p.Gly320=	synonymous	49	24.49
Pt25	ATR	chr3:142266679	C	C/G	NM_001184.4	16	p.Arg1082Pro	missense	58	12.07
Pt25	ATR	chr3:142272275	AAC	AAC/A	NM_001184.4	-	-	unknown	38	15.79
Pt25	LIFR	chr5:38493661	T	T/A	NM_002310.6	-	-	unknown	45	6.67
Pt25	NPM1	chr5:170817036	C	C/CT	NM_002520.6	-	-	unknown	27	11.11
Pt25	NPM1	chr5:170837598	A	A/AAT	NM_002520.6	11	-	unknown	24	20.83
Pt25	PKHD1	chr6:51618174	A	A/G	NM_138694.4	-	-	unknown	55	5.45
Pt25	EPHA7	chr6:93953059	C	C/T	NM_004440.4	17	-	unknown	46	30.43
Pt25	EPHA7	chr6:94124530	C	C/A	NM_004440.4	-	-	unknown	9	44.44
Pt25	AKAP9	chr7:91630620	G	G/T	NM_005751.4	8	p.Met463Ile	missense	27	29.63
Pt25	KMT2C	chr7:151962257	C	C/T	NM_170606.3	8	p.Pro350=	synonymous	197	9.14
Pt25	KMT2C	chr7:151962265	C	C/T	NM_170606.3	8	p.Asp348Asn	missense	192	9.38
Pt25	WRN	chr8:31000243	T	T/C	NM_000553.6	-	-	unknown	6	83.33
Pt25	HOOK3	chr8:42841777	G	G/GT	NM_032410.4	-	-	unknown	27	7.41

Pt25	HOOK3	chr8:42852707	G	G/C	NM_032410.4	16	p.Arg516Thr	missense	31	41.94
Pt25	UBR5	chr8:103335661	C	C/T	NM_015902.6	14	p.Gly554=	synonymous	58	5.17
Pt25	NUP214	chr9:134010257	A	A/AT	NM_005085.4	-	-	unknown	16	37.5
Pt25	NUP98	chr11:3726551	ATCT	ATCT/A	NM_016320.5	22	p.Glu986del	nonframeshiftDeletion	58	13.79
Pt25	NUP98	chr11:3740589	A	A/T	NM_016320.5	-	-	unknown	33	9.09
Pt25	KMT2A	chr11:118342797	C	C/G	NM_001197104.2	3	p.Ser308Ter	nonsense	12	50
Pt25	KMT2A	chr11:118342999	C	C/G	NM_001197104.2	3	p.Leu375=	synonymous	32	40.63
Pt25	CBL	chr11:119077232	GCAC	GCAC/G	NM_005188.4	1	p.His42del	nonframeshiftDeletion	42	11.9
Pt25	KNL1	chr15:40944177	T	T/TTA	NM_144508.5	-	-	unknown	44	6.82
Pt25	FANCA	chr16:89831520	A	A/T	NM_000135.4	-	-	unknown	43	11.63
Pt25	TP53	chr17:7577610	T	T/C	NM_000546.5	7	-	unknown	36	86.11
Pt25	ERBB2	chr17:37873542	GC	GC/GCC	NM_004448.3	-	-	unknown	19	G=5.26,GCC=10.53
Pt25	COL1A1	chr17:48276693	A	A/AG	NM_000088.4	-	-	unknown	16	12.5
Pt25	ZNF521	chr18:22642729	A	A/G	NM_015461.3	-	-	unknown	9	33.33
Pt25	SMAD2	chr18:45395590	G	G/GA	NM_001003652.4	-	-	unknown	30	10
Pt25	STK11	chr19:1226420	C	C/T	NM_000455.5	-	-	unknown	13	46.15
Pt25	PTPRT	chr20:40733280	G	G/A	NM_133170.4	26	p.Arg1176Cys	missense	72	11.11
Pt25	KDM6A	chrX:44949156	G	G/T	NM_021140.3	25	p.Trp1239Cys	missense	30	70
Pt27	TAL1	chr1:47691090	T	T/C	NM_003189.5	-	-	unknown	8	25
Pt27	TRIM33	chr1:114940209	G	G/T	NM_015906.4	20	-	unknown	10	90
Pt27	NTRK1	chr1:156848909	CA	CA/C	NM_002529.3	-	-	unknown	18	50
Pt27	PBX1	chr1:164529120	G	A/A	NM_002585.4	1	p.Gly21Ser	missense	20	95
Pt27	PBX1	chr1:164790885	C	C/A	NM_002585.4	-	-	unknown	215	15.35
Pt27	TPR	chr1:186330695	TAAAAA	TAAAA/TAAAAAG	NM_003292.3	-	-	unknown	31	TAAAAAAA=0.00,TAAAA=58.06,TAAAAAG=38.71
Pt27	MTR	chr1:237015723	G	G/T	NM_000254.2	-	-	unknown	69	13.04
Pt27	MTR	chr1:237038007	TT	TT/TA	NM_000254.2	-	-	unknown	25	AA=8.00,TA=44.00
Pt27	CRBN	chr3:3192669	C	C/T	NM_016302.3	11	p.Thr403=	synonymous	14	78.57
Pt27	XPC	chr3:14206282	TA	TA/T	NM_004628.5	-	-	unknown	143	56.64
Pt27	BAP1	chr3:52443980	G	G/C	NM_004656.4	1	-	unknown	8	25
Pt27	ATR	chr3:142277575	A	A/T	NM_001184.4	8	p.Gly592=	synonymous	62	29.03
Pt27	AFF1	chr4:88056719	GT	GT/G	NM_001166693.2	-	-	unknown	29	79.31
Pt27	IL6ST	chr5:55243425	A	A/G	NM_002184.4	-	-	unknown	24	25
Pt27	IL6ST	chr5:55243426	A	A/G	NM_002184.4	-	-	unknown	23	21.74
Pt27	SYNE1	chr6:152555045	A	A/T	NM_182961.4	112	p.Ser6861Arg	missense	57	17.54
Pt27	SYNE1	chr6:152793572	T	T/A	NM_182961.4	-	-	unknown	88	35.23
Pt27	SYNE1	chr6:152793575	A	A/G	NM_182961.4	-	-	unknown	88	22.73
Pt27	ETV1	chr7:14017126	T	TA/TA	NM_001163147.1	-	-	unknown	58	100

Pt27	EPHB4	chr7:100424577	GCCCCCCCCCG	GCCCCCCCCCC/GCCCCCCCCCCG	NM_004444.5	-	-	unknown	26	GCCCCCCCCCC=0.00,GCCCCCCCCCC=34.62,GCCCCCCCCCCG=53.85,GCCCCCCCCCG=0.00
Pt27	BRAF	chr7:140434597	G	A/GA	NM_004333.6	-	-	unknown	7	A=71.43,GA=28.57
Pt27	KMT2C	chr7:151902167	T	T/A	NM_170606.3	-	-	unknown	57	35.09
Pt27	CSMD3	chr8:113516208	TG	T/TA	NM_198123.2	-	-	unknown	54	T=3.70,TA=96.30
Pt27	PTPRD	chr9:8331574	A	A/AAACTTACCATTCCTGAACTGT	NM_002839.4	-	-	unknown	75	36
Pt27	NUP214	chr9:134014661	T	T/C	NM_005085.4	-	-	unknown	36	33.33
Pt27	NUP214	chr9:134067573	T	T/C	NM_005085.4	-	-	unknown	10	30
Pt27	NUP214	chr9:134067583	CCA	CCA/C	NM_005085.4	-	-	unknown	12	25
Pt27	NFKB2	chr10:104157187	CG	CG/GC	NM_001077494.3	-	-	unknown	5	40
Pt27	RRM1	chr11:4150238	G	G/T	NM_001033.5	-	-	unknown	70	18.57
Pt27	EP400	chr12:132502741	C	C/T	NM_015409.5	-	-	unknown	42	23.81
Pt27	AKT1	chr14:105239766	G	G/GC	NM_001014431.2	-	-	unknown	64	28.13
Pt27	IGF1R	chr15:99482409	AT	AT/A	NM_000875.5	-	-	unknown	116	38.79
Pt27	AURKB	chr17:8113603	GA	GA/G	NM_004217.4	-	-	unknown	85	45.88
Pt27	NF1	chr17:29508414	ATTTTT	ATTTT/ATTTTA	NM_001042492.3	-	-	unknown	22	ATTTT=63.64,ATTTTA=31.82
Pt27	ZNF521	chr18:22642733	A	A/G	NM_015461.3	-	-	unknown	9	33.33
Pt27	ZNF521	chr18:22642738	A	A/G	NM_015461.3	-	-	unknown	7	42.86
Pt27	ZNF521	chr18:22642740	A	A/G	NM_015461.3	-	-	unknown	5	40
Pt27	TCF3	chr19:1615796	G	G/A	NM_001136139.4	17	p.Ala492Val	missense	15	46.67
Pt27	TCF3	chr19:1650134	A	A/G	NM_001136139.4	-	-	unknown	78	33.33
Pt27	GNA11	chr19:3119404	C	CG/CG	NM_002067.5	-	-	unknown	38	100
Pt27	MYH9	chr22:36744886	GCCC	GCCC/GGCT	NM_002473.6	-	-	unknown	77	70.13
Pt28	PLEKHG5	chr1:6530965	C	CG/G	NM_001265593.1	-	-	unknown	29	CG=89.66,G=10.34
Pt28	TRIM33	chr1:114940246	C	C/CTTT	NM_015906.4	20	-	unknown	16	18.75
Pt28	PDE4DIP	chr1:144915561	G	G/A	NM_001198834.4	14	p.Arg622Ter	nonsense	275	33.82
Pt28	NTRK1	chr1:156848909	CA	CA/C	NM_002529.3	-	-	unknown	5	40
Pt28	GATA2	chr3:128202760	G	G/C	NM_032638.5	4	p.Gly320=	synonymous	49	22.45
Pt28	APC	chr5:112111309	TA	AA/T	NM_000038.6	-	-	unknown	91	AA=25.27,T=61.54
Pt28	FGFR4	chr5:176520216	G	G/A	NM_213647.3	9	p.Ala379Thr	missense	16	18.75
Pt28	PKHD1	chr6:51618170	T	G/G	NM_138694.4	-	-	unknown	110	98.18

Pt28	DST	chr6:56476448	ATTTTTTTT	ATTTTTTT/ATTTTTTTA	NM_001144769.5	-	-	unknown	120	ATTTTTTT=41.67,ATTTTTTTA=43.33
Pt28	MAP3K7	chr6:91266359	CA	CA/AC	NM_145331.3	-	-	unknown	132	46.97
Pt28	EPHB4	chr7:100424586	CG	CG/C	NM_004444.5	-	-	unknown	13	53.85
Pt28	SMO	chr7:128851818	T	T/A	NM_005631.5	-	-	unknown	22	22.73
Pt28	BRAF	chr7:140434586	G	G/A	NM_004333.6	-	-	unknown	9	22.22
Pt28	RECQL4	chr8:145736999	C	C/A	NM_138431.3	-	-	unknown	10	20
Pt28	RECQL4	chr8:145741643	TC	TC/CT	NM_004260.4	5	p.Glu287Arg	missense	16	31.25
Pt28	TAF1L	chr9:32632574	C	C/G	NM_153809.2	1	p.Ala1002Pro	missense	21	90.48
Pt28	NTRK3	chr15:88522711	G	G/A	NM_001012338.2	-	-	unknown	56	21.43
Pt28	TP53	chr17:7579643	CCCCCAGCCCTCCAGGT	C/C	NM_000546.5	-	-	unknown	20	100
Pt28	ETV4	chr17:41610736	AG	AG/GA	NM_001986.4	-	-	unknown	14	28.57
Pt28	CIC	chr19:42799393	A	A/G	NM_015125.4	20	-	unknown	6	33.33
Pt28	TOP1	chr20:39658121	CGGGGC	CGGGGC/CGGG	NM_003286.4	-	-	unknown	49	55.1
Pt28	GNAS	chr20:57428437	C	C/G					56	37.5
Pt28	PDGFB	chr22:39636926	GCA	GCA/G	NM_002608.4	-	-	unknown	10	20
Pt28	TFE3	chrX:48895792	G	G/C	NM_006521.6	4	p.Thr237Ser	missense	9	22.22
Pt29	CMPK1	chr1:47799639	G	C/C	NM_016308.3	1	p.Gly8Arg	missense	9	100
Pt29	NTRK1	chr1:156845492	AGTGT	A/AGT	NM_002529.3	-	-	unknown	40	A=20.00,AGT=77.50
Pt29	PBX1	chr1:164529120	G	A/A	NM_002585.4	1	p.Gly21Ser	missense	9	100
Pt29	PBX1	chr1:164790876	GCCCCCCCA	GCCCCCCCA/GCCCCCCA	NM_002585.4	-	-	unknown	63	28.57
Pt29	PBX1	chr1:164790885	C	C/A	NM_002585.4	-	-	unknown	66	18.18
Pt29	CDC73	chr1:193111249	G	G/GT	NM_024529.5	-	-	unknown	7	42.86
Pt29	MDM4	chr1:204507282	T	T/A	NM_002393.5	-	-	unknown	8	25
Pt29	MDM4	chr1:204512099	G	GC/GC	NM_002393.5	-	-	unknown	19	100
Pt29	MTR	chr1:237049605	T	T/G	NM_000254.2	27	p.Leu930Ter	nonsense	9	22.22
Pt29	FH	chr1:241675471	TTA	TTA/T	NM_000143.4	-	-	unknown	9	22.22
Pt29	REL	chr2:61143985	T	T/A	NM_002908.4	-	-	unknown	16	25
Pt29	REL	chr2:61143988	A	A/T	NM_002908.4	-	-	unknown	16	25
Pt29	AFF3	chr2:100210450	AC	AC/A	NM_001025108.2	14	p.Val583Ter	nonsense	48	77.08
Pt29	FOXL2	chr3:138665486	G	G/C	NM_023067.4	1	p.Pro27Ala	missense	7	28.57
Pt29	FOXL2	chr3:138665492	TG	TG/GT	NM_023067.4	1	Lys25Gln	missense	6	33.33

Pt29	FGFR3	chr4:1803307	T	T/C	NM_000142.4	-	-	unknown	74	50
Pt29	ADGRL3	chr4:62775521	C	C/G	NM_015236.6	-	-	unknown	6	33.33
Pt29	ADGRL3	chr4:62775522	T	T/TGTGT TC	NM_015236.6	-	-	unknown	6	33.33
Pt29	FBXW7	chr4:153273704	G	G/GC	NM_033632.3	-	-	unknown	10	40
Pt29	MTRR	chr5:7896970	C	C/CTT	NM_024010.4	-	-	unknown	5	40
Pt29	DST	chr6:56476464	AG	AG/GA	NM_001144769.5	-	-	unknown	25	AA=12.00,GA=16.00
Pt29	KMT2C	chr7:151902167	T	T/A	NM_170606.3	-	-	unknown	8	50
Pt29	KMT2C	chr7:151971043	T	T/C	NM_170606.3	-	-	unknown	11	63.64
Pt29	RECQL4	chr8:145738583	G	G/T	NM_004260.4	-	-	unknown	13	30.77
Pt29	PTCH1	chr9:98209362	G	G/T	NM_000264.5	23	p.Asn1392Lys	missense	59	15.25
Pt29	BLNK	chr10:97990583	A	G/G	NM_013314.4	4	p.Pro57=	synonymous	51	100
Pt29	SUFU	chr10:104263959	C	C/G	NM_016169.4	1	p.Ala17Gly	missense	6	33.33
Pt29	NUMA1	chr11:71720268	TG	TG/CA	NM_006185.4	-	-	unknown	5	40
Pt29	ATM	chr11:108163340	TATT	TATT/G CCA	NM_000051.3	-	-	unknown	6	50
Pt29	MDM2	chr12:69233076	G	G/A	NM_002392.5	11	p.Cys314Tyr	missense	7	28.57
Pt29	HNF1A	chr12:121437382	A	G/G	NM_000545.8	9	p.Ser574Gly	missense	9	100
Pt29	PGAP3	chr17:37829129	A	G/G	NM_033419.5	-	-	unknown	10	100
Pt29	SEPTIN9	chr17:75494705	A	G/G	NM_001113491.2	12	p.Met576Val	missense	23	100
Pt29	SEPTIN9	chr17:75494746	A	G/G	NM_001113491.2	12	-	unknown	5	100
Pt29	STK11	chr19:1219443	G	G/GCGG GGGC	NM_000455.5	-	-	unknown	20	20
Pt29	MYH9	chr22:36708083	G	GT/GT	NM_002473.6	-	-	unknown	83	100
Pt29	SSX1	chrX:48116786	C	C/G	NM_005635.4	-	-	unknown	104	44.23
Pt29	TAF1	chrX:70643940	T	T/TA	NM_004606.5	-	-	unknown	6	50
