# Supplementary material for: Cancer genomic profiling identified dihydropyrimidine dehydrogenase deficiency in bladder cancer promotes sensitivity to gemcitabine
Source: Sci Rep. 2022 May 20;12:8535. doi: 10.1038/s41598-022-12528-3 (PMC9122908; doi:10.1038/s41598-022-12528-3)
Supplement: Supplementary file 3 — Supplementary Figure S1. [file 41598_2022_12528_MOESM3_ESM.pdf]

Supplementary Figure S1 Tsukahara et al.

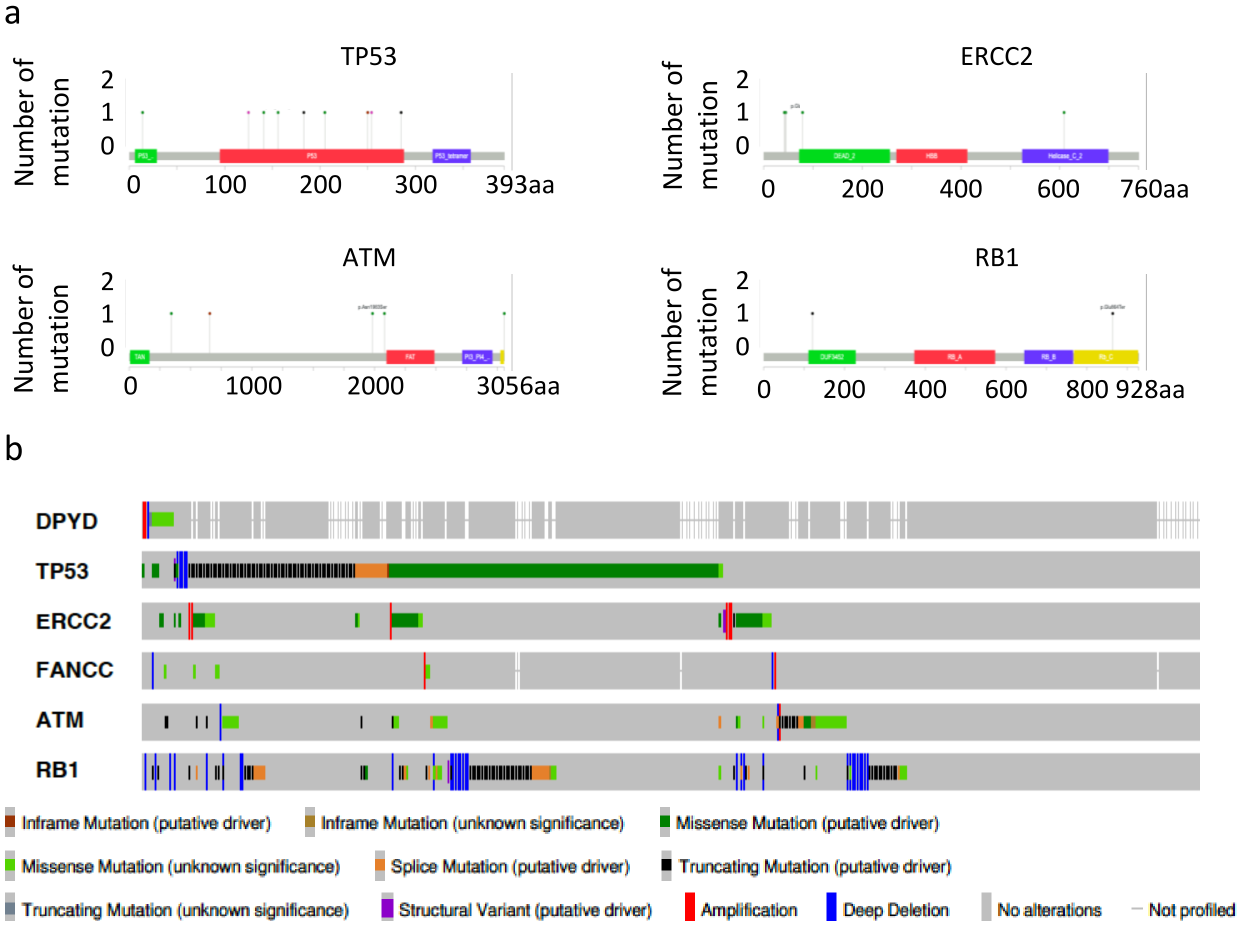

Supplementary Figure S1. Genomic alterations detected in this study and TCGA database. (a) Mutation mapping of *TP53*, *ERCC2*, *ATM*, and *RB1* genes detected in this study. FANCC mutation was not displayed due to intronic mutation. (b) Oncoprint of genomic alterations detected in TCGA database was shown.
