# Supplementary material for: Cancer genomic profiling identified dihydropyrimidine dehydrogenase deficiency in bladder cancer promotes sensitivity to gemcitabine
Source: Sci Rep. 2022 May 20;12:8535. doi: 10.1038/s41598-022-12528-3 (PMC9122908; doi:10.1038/s41598-022-12528-3)
Supplement: Supplementary file 4 — Supplementary Figure S2. [file 41598_2022_12528_MOESM4_ESM.pdf]

Supplementary Figure S2 Tsukahara et al.

a

b

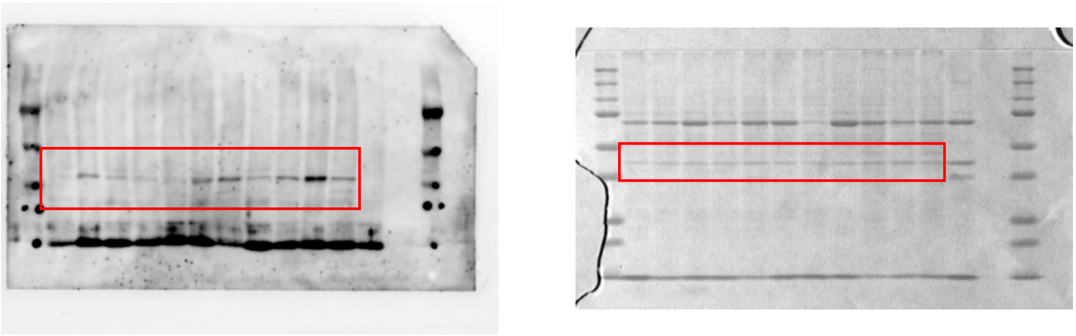

Supplementary Figure S2. Full-length blot and gel for Fig. 3a. (a) is DPD blot and (b) is gel stein. The part surrounded by the red frames are adopted as the actual figures, respectively.
