# Supplementary material for: Cancer genomic profiling identified dihydropyrimidine dehydrogenase deficiency in bladder cancer promotes sensitivity to gemcitabine
Source: Sci Rep. 2022 May 20;12:8535. doi: 10.1038/s41598-022-12528-3 (PMC9122908; doi:10.1038/s41598-022-12528-3)
Supplement: Supplementary file 5 — Supplementary Figure S3. [file 41598_2022_12528_MOESM5_ESM.pdf]

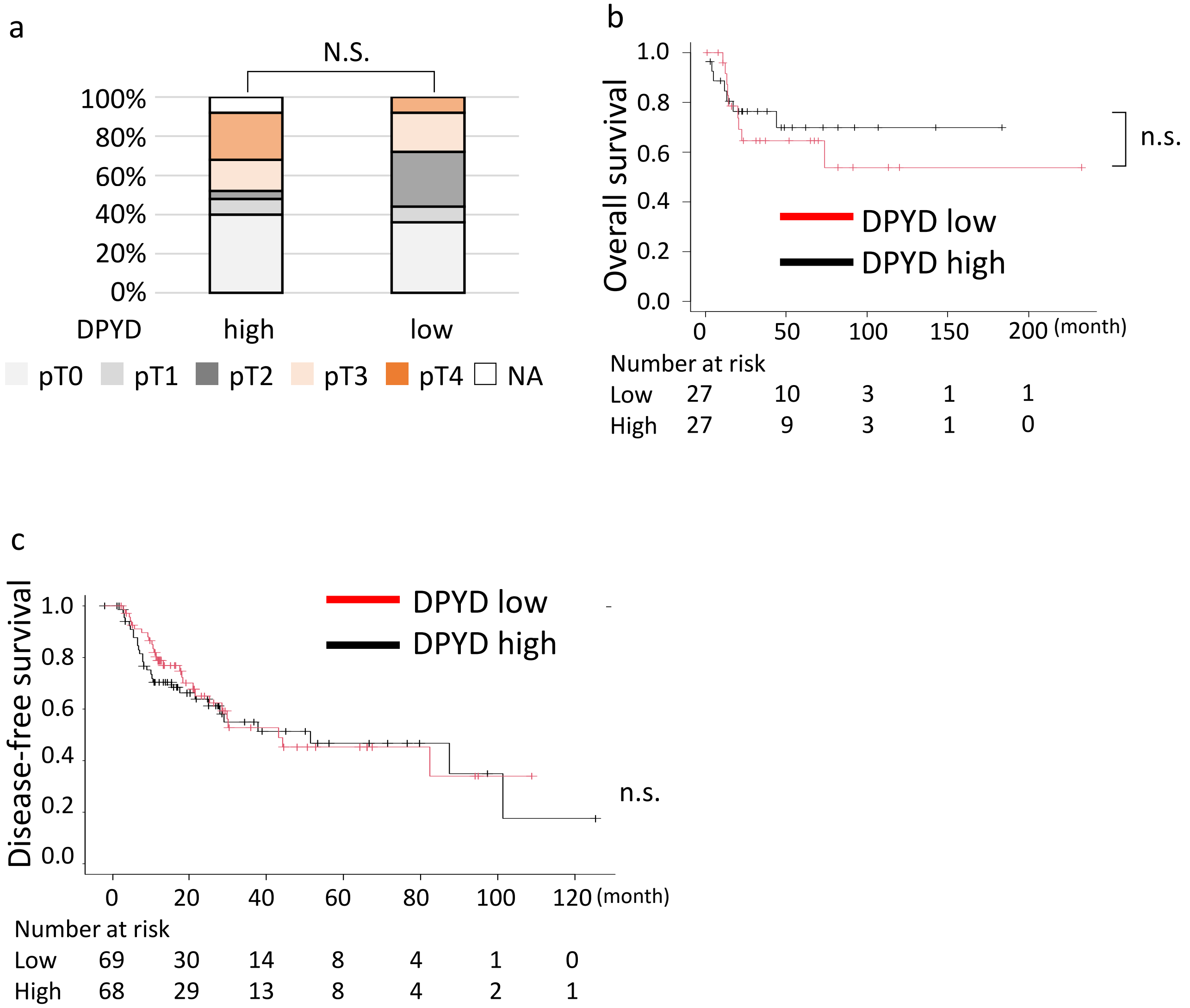


Supplementary Figure S3. *DPYD* expression was not associated with response to chemotherapy without gemcitabine. (a) Pathological T-stage in patients with higher (n = 27) and lower (n = 27) half of *DPYD* expression when treated without gemcitabine-containing chemotherapy. (b) Overall survival in patients between in patients with higher (n = 27) and lower (n = 27) half of *DPYD* expression when treated without gemcitabine-containing chemotherapy. (c) Disease-free survival was indicated with high and low of *DPYD* expression among the patients without peri-operative chemotherapy from TCGA database (n = 137).
