# Supplementary material for: Cancer genomic profiling identified dihydropyrimidine dehydrogenase deficiency in bladder cancer promotes sensitivity to gemcitabine
Source: Sci Rep. 2022 May 20;12:8535. doi: 10.1038/s41598-022-12528-3 (PMC9122908; doi:10.1038/s41598-022-12528-3)
Supplement: Supplementary file 6 — Supplementary Table S3. [file 41598_2022_12528_MOESM6_ESM.pdf]

Supplementary Table S3. Enrichment gene sets and scores (*DPYD* high vs *DPYD* low)

Gene set	Enrichment score	Nominal P-value	False discovery rate Q	Familywise error rate P
Inflammatory response	0.7743	0.0000	0.0000	0.0000
Complement	0.7396	0.0000	0.0000	0.0000
KRAS Signaling up	0.6716	0.0000	0.0000	0.0000
TNF-α Signaling via NF-kb	0.7825	0.0000	0.0000	0.0000
IL6 JAK STAT3 Signaling	0.8118	0.0000	0.0000	0.0000
Allograft rejection	0.8120	0.0000	0.0000	0.0000
Apoptosis	0.7002	0.0000	0.0000	0.0000
Coagulation	0.6563	0.0000	0.0000	0.0000
Epithelial mesenchymal transition	0.8119	0.0000	0.0000	0.0000
IL2 STAT5 Signaling	0.6674	0.0000	0.0000	0.0000
Interferon gamma response	0.5440	0.0000	0.0000	0.0000
Interferon alpha response	0.8675	0.0000	0.0000	0.0000
Apical junction	0.6205	0.0000	0.0000	0.0000
Hypoxia	0.6124	0.0000	0.0001	0.0050
UV response DN	0.6475	0.0000	0.0001	0.0050
Wnt Beta Catenin Signaling	0.6638	0.0000	0.0015	0.0100
Hedgehog Signaling	0.6563	0.0000	0.0014	0.0100
Angiogenesis	0.6830	0.0000	0.0016	0.0140
P53 pathway	0.5348	0.0019	0.0071	0.0460
Myogenesis	0.5163	0.0000	0.0106	0.0680
Peroxisome	-0.3376	0.2874	0.6569	0.7030
Bile acid metabolism	-0.2609	0.4564	0.5108	0.8010
Spermatogenesis	-0.2307	0.5801	0.4363	0.8590
