# Supplementary material for: Cancer genomic profiling identified dihydropyrimidine dehydrogenase deficiency in bladder cancer promotes sensitivity to gemcitabine
Source: Sci Rep. 2022 May 20;12:8535. doi: 10.1038/s41598-022-12528-3 (PMC9122908; doi:10.1038/s41598-022-12528-3)
Supplement: Supplementary file 7 — Supplementary Figure S4. [file 41598_2022_12528_MOESM7_ESM.pdf]

Supplementary Figure S4 Tsukahara et al.

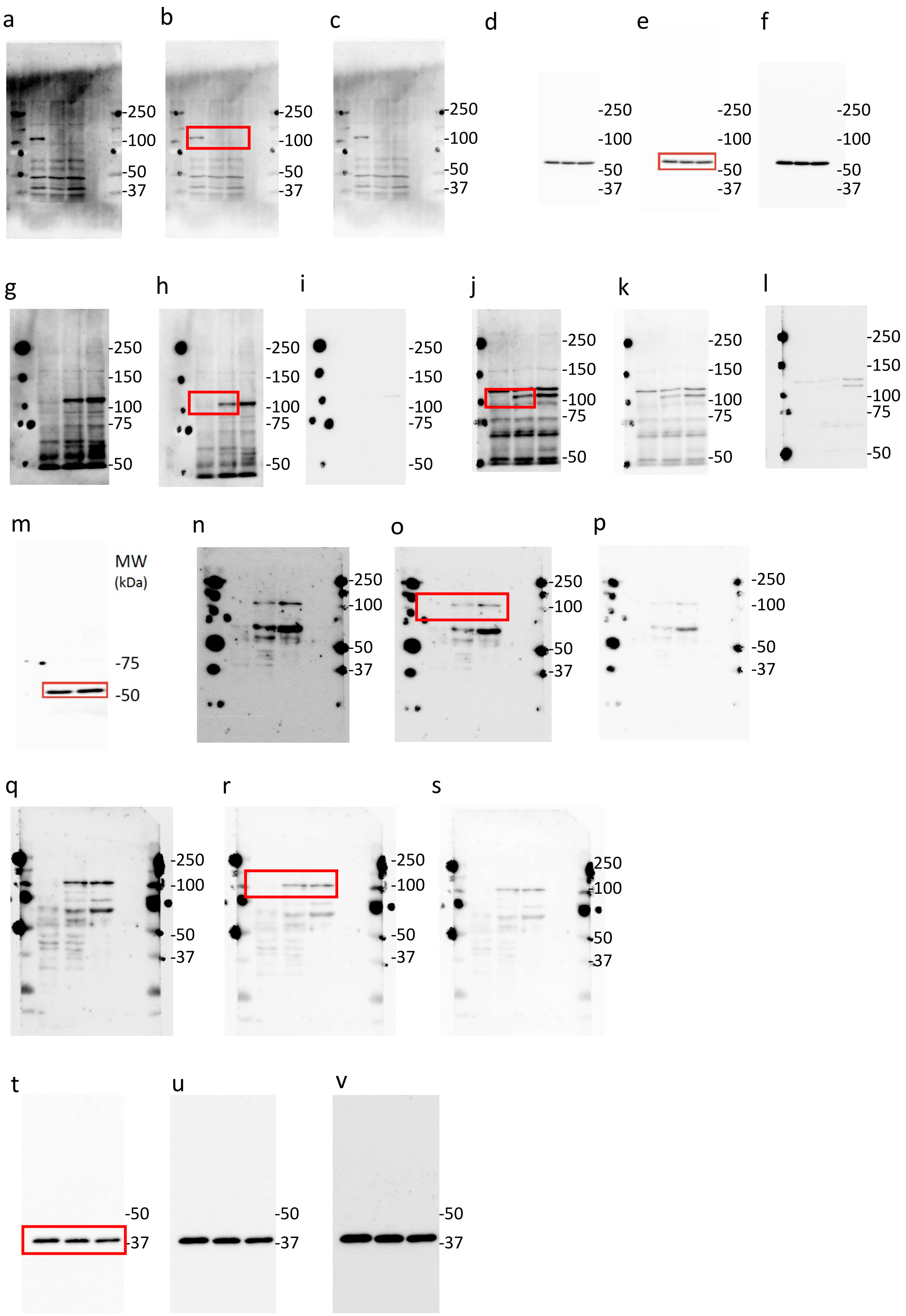

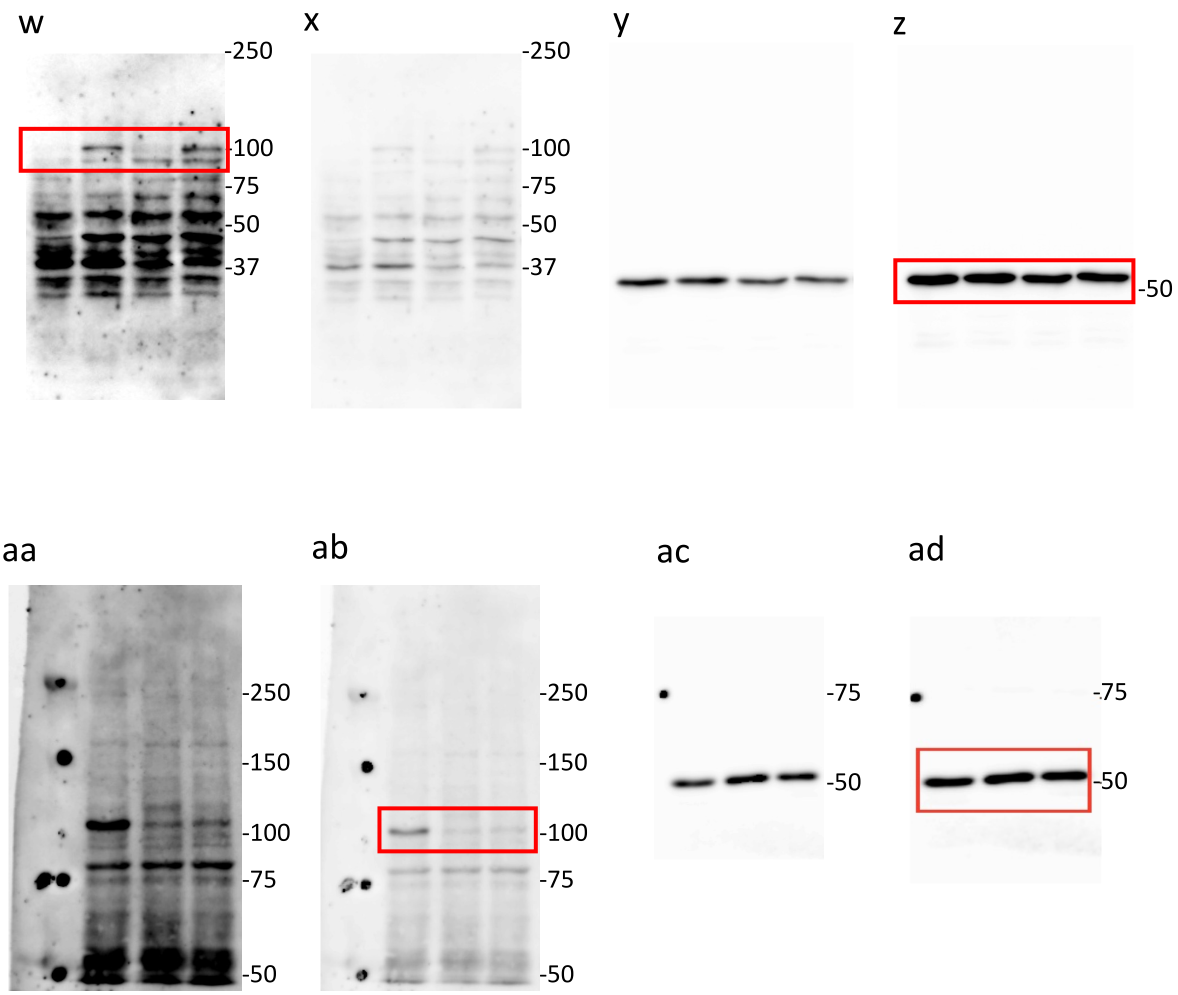

Supplementary Figure S4. Full-length gels and blots of Fig 4. (a, b, c) DPD blots for Fig 4a with different exposure. (d, e, f) α-tubulin blots for Fig 4a with different exposure. (g, h, i) DPD blots for Fig 4b with different exposure. (j, k, l) FLAG blots for Fig 4b with different exposure. (m) α-tubulin blots for Fig 4b. (n, o, p) DPD blots for Fig 4c with different exposure. (q, r, s) FLAG blots for Fig 4c with different exposure. (t, u, v) GAPDH blots for Fig 4c with different exposure. (w, x) DPD blots for Fig 4d with different exposure. (y, z) α-tubulin blots for Fig 4d with different exposure. (aa, ab) DPD blots for Fig 4e with different exposure. (ac, ad) α-tubulin blots for Fig 4e with different exposure. The parts surrounded by the red frames are adopted as the actual figures, respectively.
