# Supplementary material for: Cancer genomic profiling identified dihydropyrimidine dehydrogenase deficiency in bladder cancer promotes sensitivity to gemcitabine
Source: Sci Rep. 2022 May 20;12:8535. doi: 10.1038/s41598-022-12528-3 (PMC9122908; doi:10.1038/s41598-022-12528-3)
Supplement: Supplementary file 8 — Supplementary Figure S5. [file 41598_2022_12528_MOESM8_ESM.pdf]

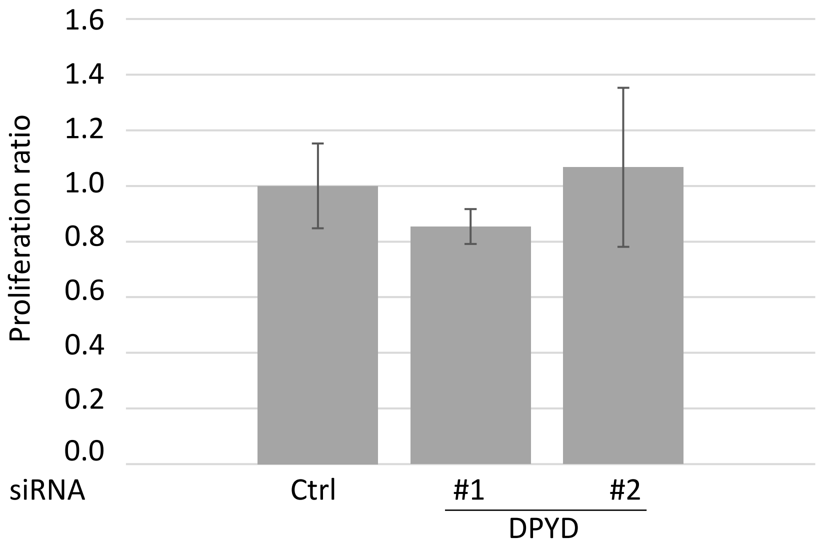

Supplementary Figure S5. UM-UC3 cells transfected with 25 nM each of the indicated siRNA were incubated with 0 or 10.0 μM of cisplatin. After 48 h, cell numbers were counted. Relative cell counts when transfected with control siRNA were defined as 1. Boxes: mean; bars: ± SD.
