# Supplementary material for: Cancer genomic profiling identified dihydropyrimidine dehydrogenase deficiency in bladder cancer promotes sensitivity to gemcitabine
Source: Sci Rep. 2022 May 20;12:8535. doi: 10.1038/s41598-022-12528-3 (PMC9122908; doi:10.1038/s41598-022-12528-3)
Supplement: Supplementary file 9 — Supplementary Figure S6. [file 41598_2022_12528_MOESM9_ESM.pdf]

Supplementary Figure S6 Tsukahara et al.

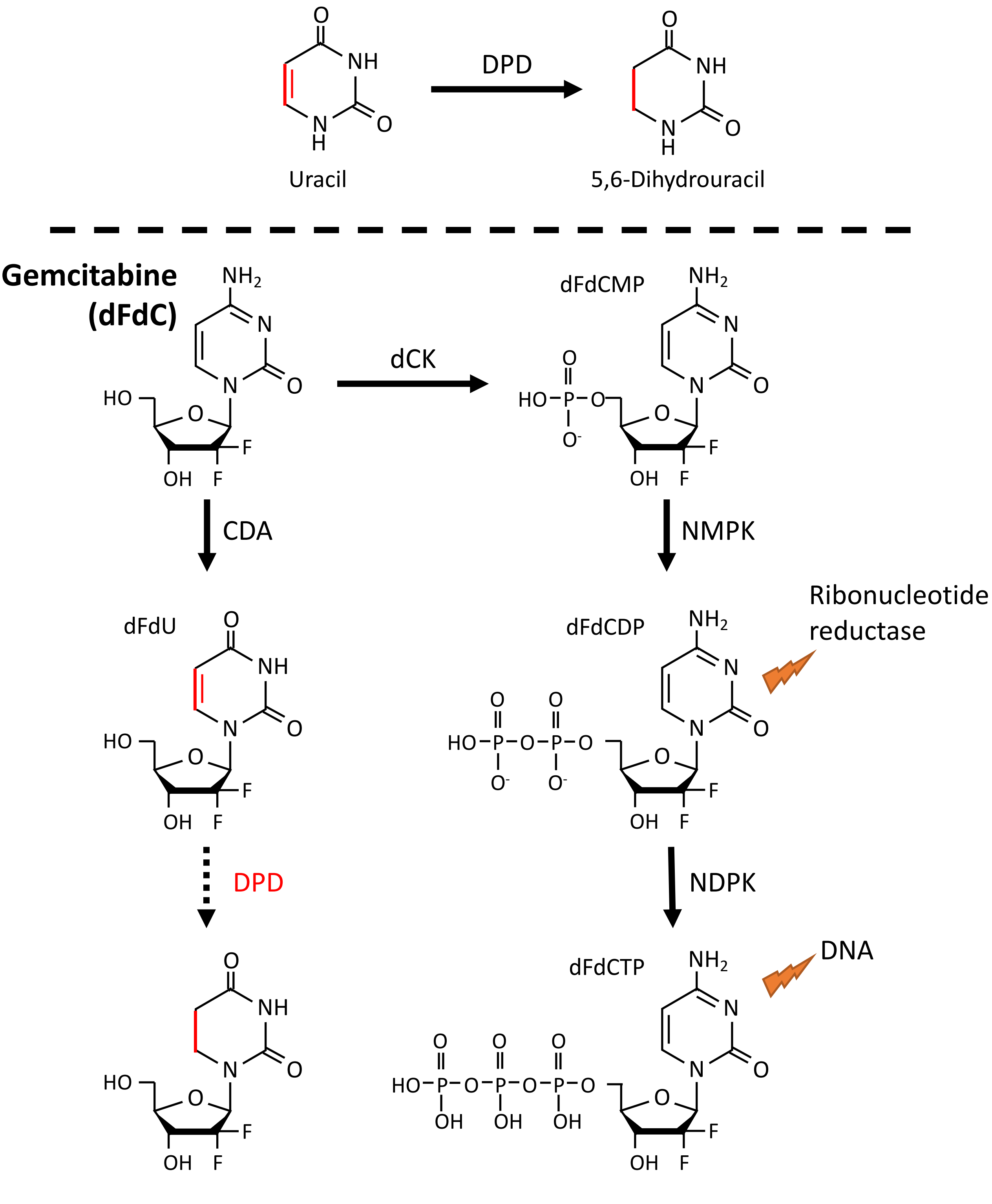

Supplementary Figure S6. Metabolites and enzymes involved in gemcitabine metabolism. dFdC, difluorodeoxycytidine; dFdU, difluorodeoxyuridine; dFdCMP, difluorodeoxycytidine monophosphate; dFdCDP, difluorodeoxycytidine diphosphate; dFdCTP, difluorodeoxycytidine triphosphate; CDA, cytidine deaminase; dCK, deoxycytidine kinase; NMPK, nucleoside monophosphate kinase; NDPK, nucleoside diphosphate kinase.
