# Supplementary material for: Cancer genomic profiling identified dihydropyrimidine dehydrogenase deficiency in bladder cancer promotes sensitivity to gemcitabine
Source: Sci Rep. 2022 May 20;12:8535. doi: 10.1038/s41598-022-12528-3 (PMC9122908; doi:10.1038/s41598-022-12528-3)
Supplement: Supplementary file 10 — Supplementary Table S4. [file 41598_2022_12528_MOESM10_ESM.pdf]

Supplementary Table 4. Genes and primer sets in the Comprehensive Cancer Panel

Chromosome	Gene	Ion_AmpliSeq_Fwd_Primer	Ion_AmpliSeq_Rev_Primer
1	ABL2	CGGGCATGAAAGTGGACATAAAAG	ACTGAAGAAACAGGTGGAGAACAAG
1	ABL2	TCTTGTGCCCCTTCAATGTTCTC	TTTTATTGGTCCCCTTGTCTTCCTC
1	ABL2	CATGTCAAGCAAGATGGCTTCA	GAATTTGCTGGATTACCTCCGAGA
1	ABL2	GTCACCTCTTCTCGGTTGCAT	CAAAGTGGTTTGGAGTGTTTGAATGT
1	ABL2	CACTCTCAGGCACCTTCCA	CCACAGAGACCGGCTTCAATA
1	ABL2	GGTGGTGACCAGGGAGTCTTA	ACGCAGTGCAGCTGAGTATC
1	ABL2	CTGCCATTGATTAGACTGCTGAG	CTTGGTTACAACCAGAATGGTGAGT
1	ABL2	CCATTCTTAGAGCGAACTTCACTCC	TGACAGTACACTGCTTCAGTTGAAATAA
1	ABL2	GGCAAGCATTTTTGGAGGAAATCT	CCTCTCGTTTTCTTCTTTCTTACCTTTC
1	ABL2	gccAGGCCCCTTTATCTCTTA	CCACTGAGAGTGACCCTAATCTCT
1	ABL2	CTTGCTACAAAATCATAAAGTGCAACGA	AATTCCCTTCCCTTCTTATGTCTTTCAG
1	ABL2	AGGTTCCTCTGTGCAAAGCTC	GAGAAATGGAGAATCAGCCCCATA
1	ABL2	GAAGTTACCCGTGAGTTCGTATTTCT	CAGCCTCTTGGAAGATGCCAA
1	ABL2	CTATCCCTGGTGAAGCATGTCTC	TTTTAAGTTACTGATCGGCCATTTCTCA
1	ABL2	CAGTTAGGGCAGTTGGCTCTT	CAAGCTCTTATCTGAGCATCAGGTC
1	ABL2	GGAGCAGCACTTTCCTCTGATTT	CTTCCATGTCCTCAGGGCTTC
1	ABL2	GGTCCTTGTCTCCAGAGGATGT	AACCACAAAGTGCCAGTCCTTA
1	ABL2	GTGTGTTTCAGAGTGGGTGAGA	CCCAAGGGTAAAGAGAAGAATGGTG
1	ABL2	CATTGCCATCCTATCCTGCTCT	GCTTCTTTACACCACGCTTAATCA
1	ABL2	AACTCCAGCCATCCCAAGTC	CACAGCTCTTCCTCTCAGAACAC
1	ABL2	CTCTGTAATGGCTAGATCCCCAGA	GGCCAATGACATGCTTCCAAAA
1	ABL2	GAACTGCTAGCTTATGCCTTCAAAATT	CCTGTCTCAGGTCTATGACCTACT
1	ABL2	GGCTGTTCCATTCGATATCCTTTTTC	ATCGGAGAAGAAACACTAAGTGCTTT
1	ABL2	CTGAACATCGTAAGAGAAGCTCTAAAGA	TCATGCTGGAGCCAAATTTCCTAT
1	ABL2	ACTCTCTGGTGCTGTCCACTTA	GAATCACATTAGGGTTCTCTTTCCCA
1	ABL2	TCACCAACAGTAAATCCAACAGATTCT	ACTGGATATTGAATTTCTGCAAGGAGTT
1	ABL2	AGTGTTCCAAAGTTAAAGAAGGGCAT	GCGGTCAGTATGGAGAGGTT
1	ABL2	GTATTTCTTCCAGACGCCAACGTA	TGATGGGCTGGTGACAACAT
1	ABL2	ACTTGGGTGCTGGGTAGTGTA	AGCTTTTCTCCTTTTCTTACCAGGTG
1	ABL2	GCTGAAGAAACCTGTAGCTCCT	GACACCAGCTGCTTGACTACT
1	ABL2	GATGCAGTCCACATAGCCTGA	CTCAGCAGACAAAATCAGCAAAGAG
1	ABL2	GACAGTAGGTCAGCACATTCCA	CAGTGATGCCTCCACCTCAAG
1	ABL2	CTCCCCTCAGAAATGTGTGCAT	GAGCAAACTGGAACTCAGCCT

1	ABL2	GAGATGGAAGATGTGGGCAGAG	TCCATCTGCTCAGACCCTACAG
1	ABL2	GCTCGTAAGCCCAGTGTCTTTT	GAATCTGGTGCCACCCAAGT
1	ABL2	GTTATCAAGCCTCCCTGAAACTATCA	CTTTTGTGCTTCTGGGCAGAG
1	AKT3	AAATTCCTATCAAGAAATGGAGAACTGCT	TTCCTTCCAGACAAAAGACCGTTT
1	AKT3	AAACGGTCTTTTGTCTGGAAGGA	AACAATTCTTTTCATCTTTCATGCTTACTATT
1	AKT3	CTTACTTGCCACTGAAAAGTTGTTGA	CATGCGTGCTTTCCTCATGTAG
1	AKT3	ACAAACACATGCACACTGCAAA	TGAAACTACTAGGTAAAGGCACTTTTGG
1	AKT3	GCCTTCTCTCGAACCAAAATAACTTTC	GGTTTTGAGTGGATATTGCAAACACATA
1	AKT3	AATGGAGTAAGTCACTGTGGAATTTGA	GCTCAGACTATTACAATAACACCACCT
1	AKT3	CCACATTCATCAGTAATTCACTAAGCGT	ACCAGGACCATGAGAAACTTTTTGAA
1	AKT3	CATCTGAAGAGAGTGTTCGAGGAAATT	GAAGATAATGACTATGGCCGAGCA
1	AKT3	CCTAGGCCCCACCAGTCTAC	TCTCAGACTACCTAGGCTTTGAAAGTTA
1	AKT3	TGTCCCTATAGTCTCTGCAAAAACAAAT	CACACGTTTCTATGGTGCAGAA
1	AKT3	TGTAGATAGTCCAAGGCAGAGACAAT	ATTATTACAATCCTGTCCCTGCTGTTT
1	AKT3	TGAATGCTGTGCTGGGAATAAGT	GACAGAAGCTATCCAGGCTGTAG
1	AKT3	CTTGCCTCTGCAGTCTGTCT	TGTGTTGAGTGTGTTTGATCTCTGA
1	AKT3	GCTTTCAGTTAGAGTGTGTGCCA	CTGGGCTTAACCTCTTCCAATTTTAGA
1	AKT3	AGCAGTGCAGCCAAAATATATACACA	TTTCTGTTCTCATAGCCTTGGTGG
1	AKT3	TTCATCATAAAAGGTGACTAAACCAGCA	TTCCATCCAGGTCAGCAATCAG
1	AKT3	AGTTACCCAGCATGCCACAAT	GGAAGGTCGTGGTGCGTT
1	AKT3	AATTGAAGATGACAGTGAAGTAGCAGAA	ATTAAAATAGATGTTTCCTGCCCACAGA
1	AKT3	ACTTTCCTATTAATGGAACCGAAGCC	GCTGGACAAAGATGGCCACATAAA
1	AKT3	CTATTAATGGAACCGAAGCCTACCT	CACAGTTGGAGAATCTAATGCTGGA
1	AKT3	TGGCCATCTTTGTCCAGCA	TTCCAAAATACCTTACTCAGCATTCAGT
1	AKT3	GTGAATTTCTTACCTTTCCTCTGGAGT	GAATGCCAGTTAATGAAAACAGAACGA
1	AKT3	GATTATAAATGTGTTTGGCTTTGGTCGT	GAGTTCGCACTGGGTTTTGTTTAA
1	AKT3	AGATCAACTTCTAAGACACCACTCACT	GCTGATATCACAAAGTACCATTTCTCCA
1	ARID1A	GCCATCACAGCTTTTGTTTTTCTTGT	CCTTTCAGAAGGTGCAGAAATACTCA
1	ARID1A	TGTGTGATGTATTTGCTCTTGGTTGT	AGGACTCAGAGCTCCTTCTGTC
1	ARID1A	TGGTTCAATAGATGACCTCCCCAT	GGGAGGTATGAGGAGAGAAAGG
1	ARID1A	AGCAGAGTAATCCAGCTCAGTCT	CCCTAAAGCCCAGCACTTGA
1	ARID1A	GCCCCCTCCATCTAACTACCAG	ATTTTCATCCCAGAGTGCAGGAA

1 ARID1A	GAATTCCCAGGCTTAGCCATGATA	GCAATGCTTGATTGGTTCATGGAAG
1 ARID1A	ACCTCTCCTAGCAAGTCTCCA	GGTGATATCCCGCCGAATCAT
1 ARID1A	AGTCGGACAGCATCATGCATC	GGTCAAACAGCTCTCCAAAAGCTA
1 ARID1A	CAGTACCTGCCTCGCACATAG	GGCAGATTAGGCAACCGAATGA
1 ARID1A	CCAGGCTTGTCAACTTACCAGTTT	CATCGCCACATTTCCTAGCC
1 ARID1A	GGTGACCCCTACAGTCGTG	AGAGGCAGATTGAGCCCACTATA
1 ARID1A	CTCATCATCAGTGCATAGCTTCTCA	GGTGAGGACTTTGCTGGTTGT
1 ARID1A	CAACAGGGCCAGACTCCATATT	CTGGGAGTATGGAGGCTGAGA
1 ARID1A	CCACCACAGCTCCAGTCC	GAGGTTGCTGCTGCTGGTA
1 ARID1A	CAGCCACAGGCTCAGTCTC	GCAGGAGGCAGGGATATCTT
1 ARID1A	CAGGTAAGTGCTAGTCATTCTCACT	CCATCTGCCTTGTTGTTCATTTTGG
1 ARID1A	GGGATGTAAAGTTAACTCCAGCCA	TTGTCACTCTCTTTTCTTTTTCCTTCCT
1 ARID1A	CCCAGGATAAGGATGGAGAGCAT	GTGTGTATCTGTCCTCCGGAAG
1 ARID1A	GACTCCTGCGTGTCCTTTGTTA	TGATACCCAGGGTTTGGAGTCA
1 ARID1A	CAGCCTTATCTCCGCGTCA	ACTGTTTTCTCCTCTCACCCGTAT
1 ARID1A	ACATTCCAGAAGCGGAATTCCA	GAGCACAAGTTCAAATAGCAATCAGA T
1 ARID1A	GCCTTAGGAAGAACTTTCCCAAAGAG	CCTCCTTCTGCCATGGAACTG
1 ARID1A	CAGACTCCCCAGTCAACCAG	CAAGGAGTTCCCATGCACTTATCTT
1 ARID1A	GTGGATAGACGACATGGAGGTT	ATCATACAGCAGGATGTTGATGGTAT C
1 ARID1A	GCAGAGAGCACATGGGCATTA	AGAAGATCCCAAACCCTCTCAATCT
1 ARID1A	AACCAGGCGGGAGATATACCT	CATTCAAAGGCATAGAGACACTGGAT
1 ARID1A	GCCAGCTCCTTGAAAAAGCAGTAT	CCGCTGACCCCATCCTTAC
1 ARID1A	accACCCCAGAGTAAGAAGCTTTA	AACTTGGTGATCTTCTCATTGGTTGT
1 ARID1A	GAGGCCATCAAAGCTCAGGTTAA	GGGTACCCATGTCCTTGCTG
1 ARID1A	CAACAGGGACCTCCGTCAG	AACAAGGGTCAAGGTAATCACAATCA
1 ARID1A	ACCTTCCAGAAATCCAGTTCTTCTACT	AGAGGTCCAGAGGTTTCCTACC
1 ARID1A	ACTCTGCCTCTCCCAACTGATA	CAATCAGGCATCGTCGGAAATATTC
1 ARID1A	CATGACAAATCTGCCTGCTGTG	CATGAAGCCAGTGAGTACCTAGAAAG
1 ARID1A	AGGGTTGCTAGAGCTCCTTGTA	GGAGCTGGACTAGACACCTTG
1 ARID1A	CACTGAGCATATCCAGACCCA	TGGTCTGTTGTCCCTGGTGTA
1 ARID1A	GGAAGCATGTGACAACAGCAGA	TCTCCTTGATGGCCTCTGAACT
1 ARID1A	CTACTGGATCCTGGGAGGTTCA	CACTATTCTCTGAAGCTGGCTTGT
1 ARID1A	AGGAGATAGCCTTTTCAGGCAAG	CTTATCTGAGCAGTCCACCACAAAT
1 ARID1A	CTTGACCGAGGATGGAGCTAA	GTCCAGAAGGGTACACAGTGG
1 ARID1A	GCCCCAGCAGAACTCTCA	CCGGAGTGCCACCTCTC
1 ARID1A	CGCCTCCTCGTCGTCTT	GGGTAGCCCTGGTAGCC

1	ARID1A	CTCAACCAACTGCTCACGTC	AACACTGCGAGGCCATGT
1	ARID1A	GGGCTACCAGGGCTACC	GCGTGGTGGCTCCTTTGTT
1	ARID1A	ACATGGCCTCGCAGTGTT	CCCTCCCCACTCAGCTGT
1	ARID1A	GCTTCCAGTAAAGATCGTACAGAAGAAT	AGCTCTGTCTTGCTCTCGAAG
1	ARID1A	CGAACCCCACAGTAAGGATGAGA	AAGTCATTGCCTGGCACAAATG
1	ARID1A	GCCCACCCTAATCCTGTGTT	CCAGTCCATTCCCAGCTACC
1	ARID1A	CCAGCACCCTGAAGCTATAGT	AGGAGAATACATCCCCGAGTCTG
1	ARID1A	GTCCAATACCATTCGAAGCCTGT	CTCACCCCTTGGTCCTGTTC
1	ARID1A	CGAATCTCATGCCTTCCAACC	GTGGCGAAGCCTGATCCATA
1	ARID1A	CAGGCACCACTAACTTATGAAAAGGA	AGATGTTGGCGAGTGTAACCAAG
1	ARID1A	TGCTAGTGAGTGACTAACCAAGTCT	GCTGTCCATGCATTTGACCTC
1	ARID1A	CATGGCTGGAGGCATAAACC	GGACACATCCCTGACCCAAC
1	ARID1A	ATGGCCAATATGCCACCTCA	CTCTGCTTCCCAGGCCTTAC
1	ARID1A	GTGCCATTGCAGTGCAGAAG	CGCCGCATCATGTCCACA
1	ARID1A	CTTGGAGATGCTCCGGGAAAA	CAAACTGCCCAGTGTAGGAGTC
1	ARID1A	CCCACCCTTTGAGCCAACTA	TGACTTGTGAAACCAATGAGTTCATCA
1	ARID1A	TCCATACCCCGAGAGCATTTG	ACATTGTTGTCCTGGATGCTGAG
1	ARID1A	TGGACATCTCGGTATCACCGT	CAGTCAGTTTCTAAGTTCTCCACACA
1	ARID1A	GGTCTTGGAAACCCTCAGCAAA	CGGGTTCTTTCGGTCACTGA
1	ARID1A	TTGTATAGCACTATGGTGCGCTT	CCAGGAGGTTGCCGATACTG
1	ARID1A	CGCCCTCGGAGCTGAAG	CCGTCCTGCAGCTCCTTT
1	ARID1A	GAGCCGGACCTGAAGAACTC	GGTTGCCCGAAGCCGTA
1	ARID1A	GGCCCGCCCTGAACAATAA	CTCCGGCCGTAGGGTTG
1	ARID1A	GGCTTCGGGCAACCCTA	CCGTGGTCGTGAGAGTTCTG
1	ARID1A	GCCGTCTTCCACCAACAACAT	GTAGTAGGAGTTGTACTGGTGGTTG
1	ARID1A	GGCGTGAACCGAACAGATGA	AATGTGATTCTGCATGCTTGGTG
1	ARID1A	CTTTATGTCCCTGAGTGCAGAGTAT	CAACTGCTGCTGCTGAGG
1	ARID1A	GGAGATGTACAGCGTGCCATA	GATAGGCATTGCCATACTGGTTG
1	ARID1A	CCTTCCCCTCAGCAAGATGTATA	ATGTTTTGCTGGGCATTGGTG
1	ARID1A	ACAGCACTATTTGGCTCCAGTT	CAGCCATACTATTAATCCCTTGTCCATA
1	ARID1A	CCATTCCAGTTTGGCCGAGA	CCTGTTGGCATAATTATAGGTCATGTCA
1	ARID1A	CATACAGGCATCAGCTGAGGTT	CCTCTGATCTGTGTGCAGCATT
1	ARID1A	TGATGGGAACTGGACCTCCT	TGACTCTTGAAGAAATCCCTAGTGAGAA
1	ARNT	AACTAGTACCGGAGAACACTTCCT	TGTCTTGTCAACTGGCCTTTGA
1	ARNT	GCTTTCATGGACTCTGTGAGGAA	CTAGGAATGGACTTGGCTCTGTAAAG
1	ARNT	CACGAAGTGAGGTTCCCCATC	GGGTGGGAAAGATGATTATCCTGTT
1	ARNT	TGCTCAACAGATGATTATTTCACCCTTT	CCCCTCTTTACTGTCTACTCTGGTTT
1	ARNT	TGGTGCGATTTTCCTGTTACTCT	CGTACTTCCCAGTTTGGTGTGG
1	ARNT	TCTTGTCCTCTCTTGCCACagata	GAGCTCTGCGGATAAAGAGAGAC

1 ARNT	GGAGGATGGAGTCTGAAAGCTG	AGTGATTGACAGTGTTGACTCTCATAG
1 ARNT	GTCAATATGCTAGGACTGTCTGGTT	GCTTTGAAACAATCCTTTTTCTCTCCTT
1 ARNT	GGTAAAAATGTGATGATCTCTGCAGGTA	GGGCAGTCTGATTGCTTTTAATCTCTT
1 ARNT	ACATACTTCACATTGGTGTTGGTACAG	TCAGGTAGTGAAATTAAAAGGCCAAGT
1 ARNT	GTTCTTAGACCGGAACCGGAA	TGGTGCCTCAAGGACAATGAC
1 ARNT	GGCCCTGTAAAGCAGCACATAT	CCCAACTCCTTTTGGTCCACAG
1 ARNT	CTTTCAAGAGATTCACCCAGATTCCA	CTCGAAAACCAGACAAGCTAACC
1 ARNT	TGAGAAACTGCCATGCGTAAGAT	GGCTTCTCTTTCTTTCCATGCAG
1 ARNT	GCCTGCCACAGTGTTCAGTTA	TGTAGAATTCTGTCATCCTGAAGACCA
1 ARNT	CTTTAGAGGCGCCCCAGTC	GGGAGTGGCCTTTCTTTTCC
1 ARNT	CAGAGTCAACAAACCAATTTAACAGCTA	TCTATGATCAGGTGCACCCAGA
1 ARNT	TGCTCACGAAGTTTATCCACATCA	ATTTGATCTTGGAGGCAGCAGAT
1 ARNT	CTCACATGAGACAATAAACAGAAAGCC	GCATGTGGCCTGAAGTGTCTTA
1 ARNT	ACCCCTTATCCTCACCCCAATA	GTGTGTGACCCAAACTTAATCTTTTTCT
1 ARNT	CTCTTGATCCTTCCTATACCAAATCACA	ACTGCTCTTCACATCTTCCTTCAG
1 ARNT	ACCCTTTCTCTCCCAGCTTCTAAT	CCTCATCATCGTTCAAGTTCTAGTGAG
1 ARNT	TGGCGGTTGTTGAACATGTTG	TGGAGTTGTTTATTCTTCCTTGCTGTAT
1 ARNT	GAAGATCTGCTCTAATTGAACGGGAA	GGAGGTCCCCGTTAAACCTTTTA
1 ARNT	TGGAAAAACACTGGAAGATTACTGACT	CCAGGATAGAGATCCAAGATTTTCAGAA
1 ARNT	CATACCCGCATTGATGTTGTGATAGA	TGGTTCAGCCTGTGACAACC
1 ARNT	CTTGCTGTGTTCTGGTCCTGT	AGTGTTCTCCGGTACTAGTTAGAATGT
1 ARNT	ACAGGGAACACACAGATGATAAGTTT	CCCTCCTGTAACCATTGTCCAG
1 ARNT	ATCTGTCCTGCAGAAGCTGATG	TTAACCTGCAGGAAGTCAAGGG
1 ARNT	TAGGATGAAGTACTCTCCTTAGACCTAA	CTCAGGCTGGATTTTGATGATGATG
1 ARNT	ACCTCAAAAATTTACTGTTCCCTTCTCC	TTCCGGTAGCTTGTGTTGAGAAAT
1 ARNT	GTTTTGGCTGTCTTAGGAACTACATGA	GAGTTCATCTCCCGACACAACA
1 ARNT	GATCCACAAAAGTGAAGATACCCTCAA	ACCATGAGGAGATAGGAAATACATCTGT
1 ARNT	CAAGTTGAAGTGAGATGGTCAGGAATA	GAATTCAAGGTGGAGGAGCCATT
1 ARNT	CCGCTTAATAGCCCTCTGGAC	GATACTGATGATAAGCAAGGACTGTCTT
1 BCL10	ATCCTCCTTGTCCTCGGACT	CCGAGGAGGACCTCACTGAA
1 BCL10	GCGTTACTCACGTCCTTCTTCAC	TCCTCTCCTTCTTCCCCATTACC
1 BCL10	CTGGCCTGCTCTGCATTTT	CCTTGTTGAATCTATTCGGCGAGA
1 BCL10	TTTCAGCACTTCATCTGTAATCTTCTGT	CAAGTAGAAAAAGGGCTGGAAAATTGT

1 BCL10	CCTTTTGGGTTTTCCTGTAAGTAGTCTA	ACCTGTGTGAGAAAATCATAGCTGAG
1 BCL10	TTGCACGTAGATGATCAAAATGTCTCT	TGCCAACTAATAGTCACGTATGTGATTT
1 BCL10	CTGTAGATCTGGTGGCAAAGGA	CATCCAGAAGGAGAATCCAGCAC
1 BCL10	TTCTAGAACAGGCAAATTCAGAGAAG	TTTTCCAGATGGAGCCACGAA
1 BCL10	TCATCTGAATTTGATCTGGAGAGGTTG	TTTAACAAGTCACAAGATGGACAGTGA
1 BCL10	CTTACATTGCATTTTAAAAGACATGCATCA	TTCCCTTAAGATCACGTACTGTTTCAC
1 BCL10	GCAATAAAGTGTCATTGTCGTGAAACA	TCTTCAACTACACTTCCCAGACCT
1 BCL9	CTCTAATTTGTGCTGACCAGCTGTA	CCCCCTGTTTACCCTGATTGG
1 BCL9	CCCCAGCTGGATTCCAAATTCT	CCATTTCCAGCCCCATTCTTCA
1 BCL9	CCATACCCCTAAAGCACTCCCT	GGAGTCCCAGGATCTCTCTGA
1 BCL9	GTATTTCCGCCGACTCCTTTGA	CTGTCGAATGTCAGAGGCCTT
1 BCL9	CTCCAGGCATGATGATGTCCAT	CGGCACATCCCATCTTAGCAG
1 BCL9	AGCGCATGCTTTTTCCTGATG	CTTACCTAGGCTTTGGGATTGGG
1 BCL9	GGGCCAATACAGGCCATGAT	GGTGGTCGGGTCCTATGGT
1 BCL9	CCACCTTCTATGAACTCCCAGTCT	ACCATCATGTCCTGGAGGGAA
1 BCL9	GTCTCCCACGATGCACCAA	GAGGTGGACAGGTGAAGCAG
1 BCL9	AACAAGTGGTTGTCCAGCAGT	GGCAGAGAATGTGGCATGTTGATA
1 BCL9	CTGCTGCTGCTTCCATTAAGTC	GGTGCTTTATGGTTTGGAGGGAT
1 BCL9	GGGTACAGAGCCATTTTCTGATG	TTCGGCCCTTCCATTTCAGAG
1 BCL9	AAACCTCCCCTTCAGAGTCCT	CAGGTCTAGCAGTCTCCAGCTA
1 BCL9	CCTGGCCTTGCTAGTTCATAATCA	ATGGTATAAGGTGTACTAGAGGGAAGAC
1 BCL9	TGTCCCGTTTGTGACTGCAA	TCATGGTGTGCCTATGGCATC
1 BCL9	TGCCTCTGTGAATATCCCTGGAA	GCCACAGTCTTGATAGCATCATGG
1 BCL9	CCCAGTTCCACCCCGTTATA	CACAACCTGCCTTTCTGATTTCTTAC
1 BCL9	GTGGGTTTGTGTTGTGATGCTAT	GGGCTGTAGCATTTGATGGG
1 BCL9	GCACACACCACACTCGATGA	AGCAAGACACAGCCATCAACTTA
1 BCL9	TTCTTTCTATTCCTTCCTCTCCCTTACA	TCTGGATGTGGAAAGAGACGATAGT
1 BCL9	TGTTTTGAAGGGCCAGGTTGAA	TGCAGTCACTGTAGAATCATTAAACCAT
1 BCL9	CTATGCACATTGGATGCACAATAGTTTT	TGAGGGTTGGCATCGGAAC
1 BCL9	CCTTCGGTGTTTACAGACCCA	GCTGTTTACGGCCTGGAACTT
1 BCL9	AGAATCCTCGAATTTCAGGTCCAAA	CATTGGGACCCCATGAGGAG
1 BCL9	GCCGTGGGACCCAACATA	GAGGGAATGGAACCTGCTGT
1 BCL9	CAGCCAGACGCTGCAATATTT	GATGTCCGGAGTTCCCACTG
1 BCL9	GGCTTCCCTCCAGTACAGTCT	GCTTGCAAAGTGCTGCATCT
1 BCL9	GGACTAGCATTACCTGGCATGG	AGGTCCCATCATGCCTTGTTG
1 BCL9	GCAACCTGCCCCAAAGTTCA	TCGGATGACCTCCTGCAGAT
1 BCL9	CATGAGACCACCAGCCTTTCT	CCGATCCTTGCCAGGAATCAT
1 BCL9	GCCCACCCTGATGAGCAAT	GGGATCATGATGTTCTGTTGGG

1	BCL9	GCTCTTTTAATTGCCCTTCTGTTCA	GAGTTGGCTGCAGTCTGGTAT
1	BCL9	CCCTGGTCGGGAACTTGA	AGCAGGCAGCATGTCTGAG
1	BCL9	CAGGACCAGAATTCTTCCCAGA	GGAGCTGGCAGGACTTCCTA
1	BCL9	GGGTAGAAAACAAACTGATTCCTTCTG	TTGGGATCTGCTGAAGAGCTATTG
1	BCL9	CCTGGATTTGCAGGCATGATAAA	CCAGGAGGAAAATTTCGACCATCT
1	BCL9	CAACTCCCAACAATAGGGCAGT	CTCCAGCTGCTCCTGAGATAG
1	BCL9	CCAGATGATGTGCCAAAAATCCC	GCTGCTGCTGAAACATCTCTTC
1	BCL9	CCCCACACTGGGAGAGAATC	ACTTTGTGCTCCTGTGAATTCTTTCT
1	BCL9	CCAAACCCCCAAGGATTGTCT	GGTTATTCCCAGGCTCCATGT
1	BCL9	TGCTGAAATTACGCCCAGGT	GATTCCGCAAGCCACTGTTG
1	BCL9	GATGAACAGGATGATTCCAGGCT	CCCACTAGGAACCATCCCAAAC
1	BCL9	GACCATTCCTTCCCATGTCTCAG	CTGAACTGGAGGAGCTGTGTT
1	BCL9	ACTACCTCTCAACCCTTCCAGTAA	CCCAGCATTGGTGACTGGAC
1	CDC73	AGAAATAGGTCTTCAGCGATCTACTCA	GGTTTCTTTGCTTCTGCTAAAACTTCA
1	CDC73	TTCTTTTATAGTCAAACGAGCTGCAGA	GGTTACTGCAATCAAAGAATCCTTGTT
1	CDC73	TTTATCTTCCCATTTTCATCACGTGGA	GTTGCATCTGGTCTTTTCTTCTTTGTAT
1	CDC73	AAGGTTGTCAACGAGAAAATGAAACTC	AGACTATCTTACCAGTCTTGAGGCATAA
1	CDC73	CTTATAGAGTAGTAGACCAGCCCCTTAA	GCAACGTCATCAACGGCAAT
1	CDC73	CGCACCAAACAGCCTATCC	CTGCCTCTCAAGTTCAACACAAC
1	CDC73	AATATATTTATGATACACTCCAGGAATGCC	GCAGTTATGTCATCATCTAGATCAGTCT
1	CDC73	TGCAATCAAAGCCAAAATTATGGCTAA	GTAAGATAGTTGTTCGTGTCCTCCAT
1	CDC73	CGAGATATTGTCAGCAGAGAGAGAGT	CTTCCACCTAAAAGCAAAGTTTAACCAA
1	CDC73	TCCAAGAACATTTTTGCAATTCTTCAATCT	ATGTCTTCAACGTTACTACACTGCAATA
1	CDC73	gaatCCAGCCTGAAGAGTTGAATTAGA	CTTACAGCTGCACGTCGGA
1	CDC73	CGTGAATCTTTTTATGTCTTCAGCAACA	TGGAATGGGCTTCTGAAAGTTCAATAT
1	CDC73	AGAATATGGTTTTTATGACACAGAGTTGTGA	GGCCTGGTTTTGCAATCATTTACTTTT
1	CDC73	CTGCTGCTGTTGGTTCGT	GGATGTTGTACTGTCGCAGGA
1	CDC73	AAGATGGCGGACGTGCTTA	ACAGCCATGCCGGACTTAC
1	CDC73	AAGGCTTTGTATATTATTGAACCATCACA	GGTAGGTTCCCATAGTGTCAATTTTGAA
1	CDC73	TTTCGTAGAAACGGAAGGCTTCA	ACTCACATAAAATCCCTGGAACAAAAGA
1	CDC73	AATGTCAACTTGTTTTTACATGCATTATTCTAA	GGAGCCAAATAATTCAGAATCTCAAGTG
1	CDC73	CAGGTACATGGTAAAGCATAAATCGC	GAAGCACAAAGCATCAAATAAGGTCTTA
1	CDC73	TGGTAAGGAAAAGTTACATGTAGCGTT	TGTTCAGTCTGTACAATCCCTTCTTTG
1	CDC73	CGCCTTGATAAAGAGAGATTGGCT	AAGAGGTAAATGTAAATGTGGAGAGCAT
1	CDC73	GTTATAATACGGCTTCAGTTGGTGGA	TTCTGAACATTTGGATCCAGACGAA

1 CDC73	AAGCCTTCCATCTGAAGTATGATGAAG	CACACACAAGCATATTTTAGAATCGGAA
1 CDC73	GGACTGTTGCCTAAGGGATTTATAGT	TGATCCATCAGGCAAAAGCCAT
1 CDC73	GCATGGCAGTTCAAAGGTTGG	TGACATACACATCATATGCGCAGAA
1 CDKN2C	CTCTGTAGCATATGCACTTGAAGGATT	GCGAAACCAGTTCGGTCTTTCA
1 CDKN2C	CTACTTAGAGGTGCTAATCCCGATT	CCTTCTTTGGCAGCCAAGTG
1 CDKN2C	GCCTGGTTAGGAGCAAAGGAA	GCATTGACGTTTACATTATTTTGCAACA
1 CDKN2C	ACATCGAGGATAATGAAGGGAACCT	GCTAACAACCTCATTCCTCCCA
1 CDKN2C	GGGACCTAGAGCAACTTACTAGTT	CGTAGGCAACATTATTGACTTGTTTTCC
1 CDKN2C	CTGTGATTTGGCCAGGCTCTA	AGTCAGGAGAGCTACTCAGTTAATTGAT
1 CKS1B	GGGAGGTGGTAGTGGAATACA	AGCTTGAGGCTGAAAAGTAGCTT
1 CKS1B	GTTTTTCTTTCCTTTCTTGGCCTTGT	ATTCAGACATCAGATGGGTTTTAGGG
1 CKS1B	CAAGGACATAGCCAAGCTGGT	CTCAGTGTAGCAGTTCTATATGGTTGTT
1 CKS1B	GTTGGGAGTTGCTTGGAGGT	GCTCACGACCCTCGCTATTG
1 CMPK1	TTGCAGGTTAAAAGAATGGCAAATTCA	AGTGGTTGTATGTAAAAACTGCCAAGTA
1 CMPK1	GCTGTATGGTATTTTAACAGTACCTGCT	GCAGCTATCAAAGCAATATTCAAGCAT
1 CMPK1	GGGCAGATCCAGTGTTCTGTG	AGCATCTATTTTCTTGACTTTCCCCATT
1 CMPK1	CCTTCAGTCAACAAAGCCAATTATTGA	CCTGCTTACCCCAAAACAAATAATCAC
1 CMPK1	aagtataGTCCTACCACTGGGTTGAAT	CATACTGTGAATCTGGGTTCTTCCTTT
1 CMPK1	AGGAGAGCTGCTTCGTGATG	CTGGTTGGCATTCACACTCCTTA
1 CMPK1	TCCTTTAGGAAATGGATCAGACAATGG	AAAAAGAGAACGAAAGATACATCTGCCT
1 CMPK1	CAAGGATGGAACAAGACCATGGAT	AGTTGACTAACAGTTGCTTAAAGCCT
1 CMPK1	GTCAGCTCCCTCAGCGT	GACCAGCGGCTTCATGAGA
1 CMPK1	CCGATTCTCCTCTGCTCTCCA	GCCCGCATCCCGTCAAG
1 DDR2	AAGGAACAGGGTCTACCTCCAT	CGAGTGTTGCTGTCATCAACTTT
1 DDR2	CCTGGTCACAGATCCAATGCTT	TTCTTATATCATGACCATTCTGCACCTC
1 DDR2	TAGATGCATTCTGCTCCTCGAA	TCCAAGTCCTTTAGGAAAATGTCATAGG
1 DDR2	AGGTGCTGGATGGAAATAGTAACC	GCTTCCTGGGCTAGTGACCTA
1 DDR2	TCAGATTTCTCTCTCCTTTTCCTCCT	CCATTGGTGGCACTCTCGTT
1 DDR2	TGTGGCCCGGCTATGACTA	gcctcCTTAAAGAAGTTCCACCAC
1 DDR2	GCCTTGGTGTGCATTCTTCTC	CAGAGAGGGTCATCAGTGATACAC
1 DDR2	CCCAAACATCATCCATCTATTAGCTGT	GAGAAGGAAGACCTGGCTTGTTTA
1 DDR2	CAGGAAATGCCCAGCAAGAGTA	GCACTGACATCTAGGGCAAAATCTT
1 DDR2	TGGAGGGAATGGAAAAATTCAAAGACA	AGGAGTATTCCTGGTCCTAAAGATGAAA

1 DDR2	CCCCGTCTTTGTAATATTCTCTCTCTCT	CTTGGAATGAGGGACGGTTCT
1 DDR2	AGCTGCTGGAGAAGAGATACGA	GGTCTTGTAGGGAGGACCTGAG
1 DDR2	CTTGCATTATTGGTTGGTGGCA	GCTGGTCTTCTAGCATATAGCTGAGTAA
1 DDR2	ACTCACTTGGCTGTGTTTCCTT	AGTGGCCAATTTCCCATTCTAATAAAGTT
1 DDR2	TGCTTGCCTGTGAACCAGTAA	GCAGAAACTCCTTCAGGTCATCAG
1 DDR2	CTTTCTCCTTGCTCTTCTCTTCCA	GGAAAGATGCGATCGTAAGTCGAG
1 DDR2	GCCCTGAGATTCCAGTGGAAC	GTGCCATCCCGACTGTAATTGA
1 DDR2	GAGTTTGCCCCCATGTACAAGA	CCTTCTTCTCCTAGCCTAAGTCAAAAT
1 DDR2	CACCTAGTGAACAAGGGTCCAA	TTGCTGAGGTTTCTCCCTTGAC
1 DDR2	ACCTTAGCAGGGCCAACCTA	TTGACGTCATCCAGGACAAGG
1 DDR2	GAACCTAATGCCATTTCCTTCCC	TGAACATCATCCAGGTATCTGCAAAA
1 DDR2	AGTGCCATCAAGTGTCAATACCA	CATTCCTCCTTCATCACCTTGCT
1 DDR2	ACCATGTCCTCTCTTTTCTCTTTGG	ACATGGGCTTTCTTGATGTAACCAT
1 DDR2	CCTCCTCTCAGAGTTCCTTCCT	CTGTCACTCCTTGGAGGTTCAC
1 DDR2	CCACTATGCAGAGGCTGACATA	TCCTTCTCCCAGCTTCTCTTTGA
1 DDR2	AGTTCCCCAGGAAACTCCTAACTT	GAAGGGACTCTTTGTTCTACTGATCATT
1 DDR2	GGCTCTGACTCACCCTTGTTTT	TCCCCCAGTAAGTCCCTTCAAATTA
1 DDR2	CTACCTTCTGTCTTCTTGTCTATTTCCT	TTCCAAAGTCAGCTATCTTGATTGTGT
1 DDR2	ACGAAACTGTTTAGTGGGTAAGAACT	ACAGGGCTTTAAAATGCTGAGAACTTA
1 DDR2	AGCTGCAGATTATGAAATTTAACAGGGT	GGGAATAGGGCTGTTCTTGACAAAA
1 DDR2	GGTTACTTTGTGGGAGACTTTCACC	ATCCATTCATCCCCAACAGTTCTTAC
1 DPYD	CCTGGTAGCCAGAATCATTACAGG	AGCTTGCTAAGTAATTCAGTGGCTATTT
1 DPYD	CCTCCTTTTCAAGAGGCCCAAA	GACTCTGCTGAAAGTGGTCTTCA
1 DPYD	CGGAAGCACCACTATGGAGAAAC	TCAGTGTGCTAACCATTGATTATTCTGA
1 DPYD	TTCTCCCATGCCATGTGGAC	TTATCAACGGTGAAAGCCTATTGGTAT
1 DPYD	CAGTAAAGTAGGCATACTTATATAGTTGGCA	CGCAAGAAAATCATAGCAGAAAACAAGA
1 DPYD	GGGATAAAACAGTTTCTCTTAAGTGGTG	ATTTTTCTCTTCTCTGAGCTAACATGCT
1 DPYD	AGATATTTGTGCATGGTGATGGTAGTG	TGATCTTTGTGTAGGTGGATGCAATTT
1 DPYD	GGTCCCTCTTCAGTGGCATAT	ATTTTGCAGAACTATTATGGAGCTGCTA
1 DPYD	CCAAGTGGGTTGTCAGAAAATATCATCT	AAGAAATAATGTTTGTCGTAATTTGGCTGTT
1 DPYD	AGAGAAAGTTTTGGTGAGGGCAA	TGTAGAAATGGCCGGATTGAAGT
1 DPYD	CGCTAGCAAGACCAAAAGGATTT	CAGTCACAATATGGAGCTTCCGT
1 DPYD	GGTAGTTCAGGCTTGGCAGAA	TCGGATGCTGTGTTGAAGTGAT
1 DPYD	GCAAGAAGTGGGCAACACCTA	GCATGAAAATGTTGATGTGTCTTGCAT

1 DPYD	CAATATTTACAATAGCGGGCAACTGAT	TGAACAGGATCCAGAGCTGGT
1 DPYD	CAAGCTGTATTCTGTACCCACAGAT	CTAGATGCCTGAAATGTGCAGATG
1 DPYD	ATTAGTTGGACAGCTCTTCTGACAC	TTACTAGACACGGACTCTGAATGAGTAT
1 DPYD	TGTGGAGTGAGGTAATTTTCAGCAT	TGCTGTCTTTAGAGTATCCTGGCT
1 DPYD	GGAGTACAACTCCATATTTCTGATGGT	CTGGAGACACTGCCTTTGACT
1 DPYD	CAACGTAGAGCAGATGTTGCAC	GAGCTGTCATGCAGAAATGGTTT
1 DPYD	GATCTGAAATAGAAACCAAGGCTGAGT	GAACTACAAGACTGGGATGGACAG
1 DPYD	GGTGACTCACAGTAGCTGGACT	TTGGTTGACTGCTCTGTCTTTTCT
1 DPYD	ACATTCACCAACTTATGCCAATTCTCTT	TCCTTTCTGAATATTGAGCTCATCAGTG
1 DPYD	GACACCAATATGCAGCCGTTTT	CATCAGGACATTGTGACAAATGTTTCC
1 DPYD	TGTTATACCTGCTTTACTGCCTTTGG	CCCCTCCTCCTGCTAATGATATTTTAT
1 DPYD	CCATTACCTCAGACTTCTTGGCA	TTTGTGTACAACTGGATAACTACTTGAACTT
1 DPYD	AGAACTGAACCAAAGGCACTGA	CTGTCCCCACGGAAGGTTATAG
1 DPYD	GCATAGCAACAATTCTCCCACCTTTTA	ATTTTCAAGTTTCTTCTCTGTTCTGTTTTGT
1 DPYD	GCAATGAACTCATTTGTTCCCCAA	GCAGAATTGTGAGAAGCTGGAGAATAAT
1 DPYD	AAGAGTCGTGTGCTTGATGTCAT	ACATTTTTGCATAACTGCATAGATACAATTTTT
1 DPYD	ACTCCTTTCTTTTTGAGCAGTACACA	CTGATGCCTGTTTTTCTTTCTCCTTT
1 DPYD	TTTGGAAAGAGCTGAACACAAGGA	CTGCATCAAAATGGTTTCCAGGAC
1 DPYD	GCCTCTCTTTGGTTCATAAGGTGTT	CAGTGACATCAATACCCTCTATTTCTGT
1 DPYD	CTGAAGGCAGTCATTCTTCTGGATAT	GATAATTTGCGGTAAAAGCCTTTCAGT
1 DPYD	CACTGAAAGGCTTTTACCGCAA	TGGAATCTCATAGAATTTTTGGCTGACT
1 DPYD	CTACCCGCAGAGCACTCC	ACTGGCAGACTCGAGACTGTA
1 DPYD	AGTGGAAATGATGGCAAATGCCTA	CAGAAACTATGCAAACTAGTGAAGCAT
1 DPYD	TGTTTTGCTCCATCATTTCTGACACT	CCTGCAAGTATAAGTTGTGCTTCCTT
1 DPYD	ACATCACCACCTGCAAATACCC	GGAATTGTCTGTTGACTAGTGATTGTCT
1 DPYD	GAGTACCCCAATCGAGCCAAA	CATGACAATTGATTTCCCCGTAGG
1 DPYD	TGACTCCAAATAGGAGACGTCAGA	TGGAAGCAGGTCTTTTATTTGGGTAA
1 DPYD	CTTAGGCAAGGTTGGGTGTGA	CATCCAAAGACTTTTTGCCACTTGTAG
1 DPYD	GCAATATTTGGCACCACTGGTT	TGTATCAACTGTGGTAAATGCTACATGA
1 DPYD	TCTGAACAGCTTGCCTAACCC	TCATGTAGCTCATTGTCAAGTTGGATTT
1 DPYD	CTACTGTATTATTTCTCATGGCAGCTCT	GATTGCTAGCATTATGTGCAGTTACAAT
1 DPYD	AGTGAAAAACAATTCCCTGAAAGCTAGA	ATCTGAAAGCCGATGTGGTCA

1 DPYD	ATCCATGCATCTCCTTGCCTAAG	GCAGATGCCCTGGAGTTAAATTTATCAT
1 DPYD	TGGTTCCCCGGATGATTCTG	AAAATTCCTCTGCAAAAATGTGAGAAGG
1 FH	GTAAAGTGACTCATGAATACAGCCTACT	CCAGACCGTGAGATCTACGATGA
1 FH	GTTCTGTCACACCTCCAATCTTAAAGT	CTGGGTTTCTTTTCAACTTGTAATAGTGT
1 FH	GCTGTTCTCAAACACTGATCCACTT	GCTGATGAATGAGTCTCTAATGTTGGTG
1 FH	ACTTACCTATATGAGGATTGAGAGCTGT	GGGATGCTTCAGTTTCCTTTACAGA
1 FH	GGATTCCCACCACGCAGTTT	AACATGTTGCCTTAGTAATGtctctctc
1 FH	GGAATCTCTCCCGCCAAGT	CCGTGCCCTCGTTTTGG
1 FH	ACCATTCGAGCCGCGTT	ACAGCCGCCCAGAAATTCTAC
1 FH	CAGCTGCGGGATAACTTtaaacaa	GTTCTGGTCCTCGGTCAGG
1 FH	ATTCTCCCAGACCTGACCGA	TCTTTAGGCTTGCCTTTTGTCACT
1 FH	GAGCAGCCAGAGCTTCAAATTTATT	CGTATATTGGAACTTTCTGTTTCACTTGC
1 FH	CAGAGTATGGCATGGGTCTGA	ACCCCAGTTATTAAAGCTTTTGGCA
1 FH	GGCCGCTCGCTTCAAGA	GTTTTATGTATAGCTTTTGCATCTGCCA
1 FH	ACTTCTTGTCAAAAAGTGAATGCTTGTT	TGTGCATCCCAACGATCATGTTAATA
1 FH	GCTCACATACTGACCTGGCTTT	CCTCTCGTGGTATGGCAGAC
1 FH	CATTCATATTTGTCTGAGTTCCTGATCCA	TCAAACTCTGTGGCATAATCAGCATT
1 FH	GTTGAACAGAACAACCTCTGTCACT	CATACTCAGGATGCTGTTCCACTTAC
1 FH	TCAAACTCCTATACCTGCCCAAGA	AGTACTGTTACCAGGACTACAGAAGTT
1 FH	GGATTTTGCATCAAGAGCATCATGT	AGAAGTTTGTTTTTGTTGCCTCTGATTT
1 FH	TGCTTGACTTCCAACTGCTACTTATG	GCAGCAATGGACATTTTGAGTTGAA
1 FH	GCTTACCATCATTGGCTTGAAAACA	TCTTTACTCTGTCATTGGTGGTTTTCTT
1 FH	GAGATGCTTAAGTTCAATAGCAGTTTCC	TCTCACAGCAGAGCAGTTTGAC
1 FH	AGCATGTCCTTAGGTTTTACCCATTC	ACTGCTAACCCATATGTCGTCTTTTTAT
1 FH	TCACAAGAATTCAAGACAGGAACACTC	ACTGCTGTTGGTACAGGTTTAAATACT
1 FH	CCACTTTTGCAGCAACCTTTTCT	TCAGGAATTTAGTGGTTATGTTCAACAAGT
1 FH	GGCAGCTTTTATTCTTGTCATTGCATAT	TGCAGTTAGAGTAACTTGTAAGCTATTAGGA
1 IKBKE	ACTCAGTCTCCCCTTGGACA	GTATCTAGGGTGAGGAGCAGACAA
1 IKBKE	CCAAGATGAGCCTCAGACACT	CTACGAGGCGCATGATGTTC
1 IKBKE	ATTGTGCATCGCGACATCAAG	CCAGGTACTCCTCAGTCCCAT
1 IKBKE	TGATGAGAAGTTCGTCTCGGTCT	GAGACATAACTCCTGTGGAAGCA
1 IKBKE	GCAAGGGCTTAGCAGGATCAG	CTCTCCTGGGTCTCCGTGATA
1 IKBKE	CCTCTCCAGATGCTCCCAAAA	GGTTACTGGACCTTTGGAGCTG
1 IKBKE	ACCCCTGACAGTCTCCATGT	ACAACTCGCTGCAGGATGTC
1 IKBKE	GACCAGTTCTTTGCGGAGAC	GCTGCTTGGGTAGAGAAGGTT

1 IKBKE	GCATGCTCTTTCTCTAGGCAGAA	GGCCAGGGTTGCTTCCTAC
1 IKBKE	GGGCTGAGTCCTGCTCTAATTC	GCACCTAGAATCCTTCTGCCAT
1 IKBKE	CCATGGCCTCATTCTGGTTCTC	GCATGGTACAAGGTCACTCCAA
1 IKBKE	TGACTGTGGATCTCTGGAGCA	CCAGTCCGCCCCATTCC
1 IKBKE	CAAATGTGGCCTTTCTCTTTGCT	CAGCTCTGATCCTGCTAAGCC
1 IKBKE	ATGACCCAAGGGTAGGAGGT	GCCCAGCCCTCATGATTACTC
1 IKBKE	CCTCCCAGGAAAAGGGTCTG	CCTACACAAGGTGAAAGCTCCTTA
1 IKBKE	CCTGCCTTGCCTACTGACA	GGACACAGAGGTGACCCTCA
1 IKBKE	CCGACACCAGGAGTACCTCTT	CCCAGGTGGCTCTACTCAC
1 IKBKE	CCCTCTCTGTCCCACCCATA	CAGGAACTCATCCTCAGGCA
1 IKBKE	AGCCCTGAGAATGCCTTTGG	GGAATGATTAGCAGGAGTCAGACA
1 IKBKE	GGACTTGAATGGCTCAGTGTTCT	TGCTTCAGGATGCCTCATGTG
1 IKBKE	GCTGACCTGGCCTTCCT	CAGCAGGGTCCCACTCAC
1 IKBKE	CCCCTCAGACAGGGTCTTTG	ACCTCCTACCCTTGGGTCAT
1 IKBKE	CTTGGGCACTTACGACAAGGT	GGAACACCTGCAGGAGTCTTTT
1 IKBKE	TCTAGGGTGAATTTCAGTCATTTAGCC	TGGGTCCTCTGAAGCCTAGAA
1 IKBKE	GGGAGTGGGACATGTGCTAA	CAATGTTCTGGTGGTTCAGCTTC
1 IKBKE	GTGAGGGAGTTTGAGGTCCTG	AAATCTCTGTTATCATGTGGCCTGATT
1 IKBKE	CTGACCAAAGTATGGCTTCCCT	ACACAGATACACAACCGCTTTCTC
1 IKBKE	CCTCCCTCTTTCCTCTGTGCTATT	CTCATCCTAGACTTCTTGAACTGTTTG TA
1 IKBKE	TGCTGTTTGGACAAGATGAACTTCA	GGTAGCACCAGTGAAAAGGAAGA
1 IKBKE	TGCACACTGTTTCGTTGACTGAT	TGGCGTTGAGAATCGCTCT
1 ITGA10	ACCCCATGATCTCATTCTCTTCCA	GACCTTCAGAGACACTGCCA
1 ITGA10	CCCCAGCTCCAGCTTCTATCT	CCTCTTTTCATCCTAGACCCCAAC
1 ITGA10	CCCCCTATTCTATTCCTTTCCTCCTT	TTGCCTAGTACATGTCCATCCAAC
1 ITGA10	CCAGGTGTTCATCTCTCCTCTCT	ACCACTTCTTCCTTCGTTCGG
1 ITGA10	TGAGTGGTCCCTGGGAGATTT	CTAAGCCTTCATTGCTCTAGCCT
1 ITGA10	CTACAGTTACCTTCCCTTTTCTCTGG	ACACGTCCTGTTTCCTTGTTCTG
1 ITGA10	GATGGAACAACTGATGTCTTACTTGT G	AAATATGTGCACCTTCCCTCCTTT
1 ITGA10	CCTCAGTATTCTCTCTCTTCCTCTCA	GAGCTGGGACCCCTCAC
1 ITGA10	AATGTCTAAATTGAATGTGTCCTCCTG T	CCATCCAAGACAATGACAACATCCAT
1 ITGA10	CCTTCTAGGCTGCCCAACATAC	CCCAGCTCATATTGCCTTCTCTTAC
1 ITGA10	GGATGCATGTAGAAAACTACTCTTCT GT	CTCCCCACTAAAGCAGTGCTTA
1 ITGA10	TCTCCCACTTCTTCCTATTCCCA	ATGCCCTCCTAGACCAGACT
1 ITGA10	CCTCCTTCAACTCCTGGTCTCT	GCTCGTATTGTAAGCATTTTCCTTTCT G
1 ITGA10	TGCTGGTATCTACAACTCTGGAGAA	CTGAGGCCAATACCCCAAtct
1 ITGA10	CCCCATCAGGAGGGTCTTTCA	GGACACCCCTTTGTGACTCATAC
1 ITGA10	GGGCTCCTGTTCCTCTGTAGT	TCTGGAAGAAAGTTCCCTTCATTCTC

1	ITGA10	AGAGGCCAGAGGACTTCTCAT	GTATCTTCCATCCCCACCAGAAAC
1	ITGA10	CCTGATCTGTCTCTCTCTGTCTCTTC	GGGAGTACTAGAAAAGGAAAGGCTTAC
1	ITGA10	AGAGGTTGGGTCTGCCAGATTA	CACATCAGCAAAACCATCTTGGT
1	ITGA10	ATGGGAGCTCTTCCTGATCTGA	CCTGCTTTATTTGGGTACTCCTGAC
1	ITGA10	GGTCGCTGGGATCACCAATT	CCAGCAGTCCATTCATCCAGT
1	ITGA10	AGACATGAGGTTCACCGCATC	GCAGTTACTGCCCTCTCCTTAC
1	ITGA10	GAGCCCTCAACATTATGCCTCT	CCACCGTCAGGGACTTGAAC
1	ITGA10	CTATCCACTTGTTCCATAGGCCAA	CCCATCATCATTATTTCACAGCTCTCA
1	ITGA10	GTCTGTGTTATGGCTGTGACCAT	CCACATTCTCCTCATGCCAAGAC
1	ITGA10	gtgtgtCTGCATACATCTGATCTCA	CCATCATGGGACTCTCCATCAGT
1	ITGA10	CCAGGCTACTGGTGGTTGTC	ACTCTTCCAGATCCAGGGTGAATT
1	ITGA10	CCTCTCCCCTCTGACCCTAC	GGGTATGAGTCTGAGGTCAGAACT
1	ITGA10	GGGAAACTGTCTTCCCCTGAT	GAGTCAGAGAATGGGAGAAGTAATGAAG
1	ITGA10	TGTTTGCCCTGTGCTTACTTACA	AGAAGGGTCAGGCATCTTCTCA
1	ITGA10	CTTGCACTTCTCAGAGGGCTTA	CAGTCAGACAGACTGCCTCTTG
1	ITGA10	GTGTGGTTCAGAGGGACTGTAG	GTCTAGTGAGGAAGGCATGCAA
1	ITGA10	GGCAGGCAGACTGAGATTAAGA	GAATAGGCGTGGGTGATGTTCA
1	ITGA10	TGCTCCCCCTTTAACCTGGA	CCTCCCCTTTGTTAGCACTACTG
1	ITGA10	GGAGTAACTATTTCCCCCATCCC	GAACCAAGTACTCAGCCTTCCT
1	ITGA10	TTTTCTCTTTATTCCCCACTGTCTTCTC	CGTCTTCCAGTGCCATTCGT
1	ITGA10	TCTGTGCTATGGCTTGAAGGAG	CGCCCCCACTAGGGATGTATT
1	ITGA10	gactctgtTTTCCTTCAGGATACATCA	GCAGTGTTGGGAGCAAGGG
1	ITGA10	CCTACTCTGGACCCCATCTTCT	ACAGCCCCATCTTTCTTAAGCTG
1	ITGA10	TCGAGGAAAAGTCATCGCCTTC	GGCCTCTCTCTTACCCACTTCA
1	ITGA10	AGATAAGTCTGAGAGCTACTGGCA	GGGAGGACTTTCTAGACCCTTATTCTA
1	ITGA10	AGACTTCAGCCACAGCATGTT	TCTGGATCACTGGCAATAGTTCTAATTTC
1	ITGA10	AGATCCCAGCTCTTTCCTGAGA	GTCCATTTCTCCAGAGTAATCCTCAC
1	JAK1	TTCCGACAACTCCTGGGAAG	CGCTCTGGGAAATCTGCTACAAT
1	JAK1	TCAGCGTCTTGTCTTTCAAGGG	CTCCATCAGTGTGTCTGTTGTCT
1	JAK1	CATAGAGAGCAAAGGTCAGCCAA	CCATCTCACACTATGCCATGATGA
1	JAK1	AGTTCTGGCAACTGCATCTTCT	GGTTTCTCCCCCTTGCTCCTA
1	JAK1	GGAGGAGTTCAGAGGAAGACACTT	GGACCCTGATGGATTACAAGGATG
1	JAK1	CTTCTTCTCTTCAGAAGTTCCTTCGT	CCAGACGGTCCATCACTTCAG
1	JAK1	TGCAGAACGGAACTCAGCAATT	TCCGGGAAGAGTGGAACAATTTTT
1	JAK1	CAACTCAAGCTGCGAAAACATAATAGA	TTGTTCCTGAAAATGATAGGCCCAA
1	JAK1	ACAATGTGAGTGATTTCAGGGAAGTAAG	GCAACTTAACCCTGTCCCTTTTATGTAT
1	JAK1	GCCATGGGTTGGGCCTAT	GGAGAAAAGCCTTTTTATTGCTACTTAAA
1	JAK1	ACCCACTTAGAGTCAAATTACAAACCAAT	CTTCGGGAAGCCTTAAGGAATATCTT

1 JAK1	GCTGCTGTTTGAGGTTTATTTTGTTCTTA	CTCTCCCATGGTTCCCTCATT
1 JAK1	AAAATGACTCTCTAAAAGGAGACCAACC	GGCCCATTCATCAAGCTCAGT
1 JAK1	CCTAGACAGCACCGTAATGGG	AAGGAGCAGCTTGGCTAAACTT
1 JAK1	ACACCAAAGGCAACTGATAAGGT	TCCCTGGTAGATGGCTACTTCC
1 JAK1	GTAATGATGGGCATCTGCTGTGA	TCTCACTGTGCACCTCCCTA
1 JAK1	GGTACCAGGTCAGTACTTCCCA	CTCTTTGCCCTGTATGACGAGAA
1 JAK1	GGAGCATACCAGAGCTTGGTG	TCTTTGTGGATGAGAGAGTTCCTTATCT
1 JAK1	TTAACCAAGCAGAGGGATGGAC	GGTCTTTTGGAGTCACTCTGCAT
1 JAK1	TGAATCACAGTAAGTCAGCAGCTC	GAAGTTTTGTGTTCTGCTTCCTTTCAA
1 JAK1	AAGCAGCACGGGTGTAACTTA	CCTTTCACTCTGTTTTCTTTTCTCTTCTG
1 JAK1	ccaGAAGGATAAACAGCATACGCTAT	GGTGGAAACGTTCTCTACTACGAAG
1 JAK1	CCAAGATTCCCAGTCACCATCA	GGTGAAATACTTGGCTACCTTGGA
1 JAK1	CAGCACCGTAATGTTTTGTCAAAGTT	GTCCATCAGACAGAGGAACCTTC
1 JAK1	AACATTATTTATCCGCATCCTGGTGA	ACATGTGGATTCATGGACTTAGATTTGT
1 JAK1	CGTGTTTTGGTCGATCTGGGT	CCAGCTTGGGAGACCTCAT
1 JAK1	GATCTGCTTCTTGAGGTGGCT	ACATGTAATTTCTGGTCATTTCCCGAAT
1 JAK1	AACAGAGTGAAAGGCAGTCGATT	CCGCTGCATGAACTATGACC
1 JAK1	CGGAAGAAAGGCCTCTGATTGG	GTTTCTTCCATGTGCCCCTCTT
1 JAK1	CACATTAATATAGCCAGAAAGGGCCTA	GACTTCGGTTTAACCAAAGCAATTGA
1 JAK1	TGACGGTGTAATACTCCTTATCGGTT	GTGCTTGACTTTTACTTCTCTCTCTCA
1 JAK1	ACCCCTTTGAAAGAGAACACACTT	GCTGGCCTGAGACATTCCTATG
1 JAK1	AAAATTAAAGGCAATAGCACCAATAGCC	GGAGAACACTGGACAGCTGAATAA
1 JAK1	TCCTGGGCCCAAACTTCCTA	GTTCTATTTCACCAATTGGCATGGAA
1 JAK1	ACACTGACTGCTCATTGTCGTT	GGTTGTCTGCCTGCTTCTCAG
1 JAK1	GGAGAAGGACTTGATAATCTGTGGAATT	ACAGGTTTATCAACTTATGAGGAAATGCT
1 JAK1	CCTGTTTTGCACTGGCCTTTA	GAGGTAACCACATAGCTGATCTGAAAA
1 JAK1	AGAGGTTCCTTAAGATCTCGATTTCCT	ACAGCACATGCATCTTTCCCT
1 JAK1	AGATTTCCCCAAACACAGCCTT	GCTGAGTTTCGATCGGATCCT
1 JAK1	CACCTGCACCAGATCCTTCTT	GTCTGAGAGCTCGGATCTTGG
1 JAK1	CAAGCCAGTGTCCTGACTGA	CCCTTTGCCTACTTCTCCCTCTA
1 JAK1	CTGGTTTCTGGTGGGACCATTA	CATGCACCGGAAAAGCGATG
1 JAK1	TTTGAATTTCCATGGTGTGGTAAGGA	CAGGTTCCAGACATGGCTATTTTTATG
1 JAK1	GATCTTCTGAACTAGTGTGTTGGTGAA	GATCTTCTATCTGTCGGACAGGGA
1 JAK1	CCTCTGCTGTGTACTCTCCACT	GCCATGGCTTTCTGTGCTAAAATG
1 JAK1	CCTCAGTCTTCTTGGAGCTCCT	ACACTCCCTTTTGCCATAAGAACTTTTA

1 JUN	CCCGTTGCTGGACTGGATT	CAGTAACCCCAAGATCCTGAAACA
1 JUN	CCAGGTTCAGGGTCATGCTC	ACGTGAAGTGACGGACTGTTC
1 JUN	GCAACCCAGTCCAACTTGGATA	GCTAACGCAGCAGTTGCAAAC
1 JUN	CCCGACGGTCTCTCTTCAAAAT	GCTGGAGGAAAAAGTGAAAACCT
1 JUN	GCTCCGAGTTCTGAGCTTTCA	AGTCCCAGGAGCGGATCAA
1 JUN	GGTTCCTCATGCGCTTCCT	ACCTGCCCCAGCAGATG
1 JUN	GCTGGGCAGCGTGTTCT	GAGCTGGAGCGCCTGAT
1 LCK	CCCTGATGCCCCTTGTCTTTA	CAAGGTGGAGAGGGACTCAC
1 LCK	TGGCGGTGAAGGCTTGAG	CGCTCTCCCGGATGAGGA
1 LCK	GGAACACTCACGGCTCCT	CGACTCACCCCGTGTCT
1 LCK	GACTTTCCCACTCCTTCCCTT	GGGTGACCACAGCGTAGAG
1 LCK	GAGGCCAACCTCATGAAGCA	GGAGAGCAGTATCCCCTGGTA
1 LCK	TCTCAGGTCGACGGCTGA	CGACGGGTCCCCACTTAC
1 LCK	AGGGAGGGTACGTGTGAGAT	GTGAATGTCCCGTAGTTAATGGCT
1 LCK	CATTAAGTGGACAGCGCCAGA	CCTTCCCATCACCCTTTCAGTT
1 LCK	AGACCAGTGACGTGAAGACATC	GCAATCTTGCAGCTCAGGGT
1 LCK	GCTGCCAACATTCTGGTGTCT	GCCCAGACCACCCTTAAATCTC
1 LCK	CAGGGTCTGACCCAATCTTCTG	CTTGTCTTTCAGGCCCTGCT
1 LCK	TCAATACGAGGCCTGCCCTAT	CTCCACCAGCTTCAGCGT
1 LCK	GAGGACGAGTGGGAGGTTC	AGCTGTCCTGGGACTCTCAT
1 LCK	CCTTTCACCCATCAACCCGTA	CTGCGCAGGTAGTCAAAGGT
1 LCK	CCAACTCATGAGGCTGTGCT	CAAGTCAGGCTGGAGAGAAAGG
1 LCK	AGACTCACTGCGTTCTTTCGT	GCCGGGAAAAGTGATTCGAG
1 LCK	AACGGTGGCTTCTACATCTCC	GGGAATGGAGTAGGGCATTGAAA
1 LCK	AGGGCAAGGCCCCATTATATC	GATAATGGCAGTTCTCACACACATC
1 LCK	GGAAGATGACTGGATGGAAAACATC	GCATTCTCTACCCAACTCCCTCA
1 LCK	AACTTGGGCCAGCAACTCTT	TTCACGTCACTGGTCTGTTCC
1 MARK1	TGTCATGGTAGAAGTTTGCTCAACAA	ACCTTGAGGTGTCGGTTCTG
1 MARK1	GTTCTGATTGACCACACATAATGTAG AGT	GGGACAGCACTTCTTACCATGTTTAA AT
1 MARK1	GAGTGCAGTGTTTGTGCAAACT	AGAGTGCTGTTGTTTAAGTCACTACTG
1 MARK1	AGTGGAAACTTGTGTCAGAGGTC	AGAACCCGTGTGTTGCTTCA
1 MARK1	TACCATACTGGGCTTCTAAATTTGAT AC	TCATATCACCATCAAGGAGAAGGTTT TC
1 MARK1	TTTCCTCTTAGGCTGAAAACCTTCTC	ACTTCAGGCCCATCATACTTCTTTC
1 MARK1	TGCTCCCGAGCTTTTCCAAG	TGATACCTTTAAATTCTGGCCATCGA AA
1 MARK1	CTCTATACATTAGTCAGTGGCTCCTTG	CGTAAAACTCGCTCTCGCAGTT
1 MARK1	CATCTGAGGGTGGTCTGCAAA	TCTCTTTCTTTTGGTTCCCCTGATG
1 MARK1	CTTTGCCCTCTTGTTCCAGAAGTA	CAGTTATTTGCATCTAACACTTTTCGG A
1 MARK1	TGGACCCTAATGACATGATGAGAGAA A	CCATTAAGTGACAGTCGTGGCAA
1 MARK1	TGGGAGATGGAAGTCTGCAAG	TGTATCTTCCCTGAACCTGTAAATTTG G

1 MARK1	CTTCGTTCAGAGTTTAAAGCATGCAA	CTGTTAGCCTGAGGTCTTTTGGTAT
1 MARK1	TTTCCATGGTCTTCTCAACTGCTT	GCAGTGTGACTTTAATAGGATGACCA G
1 MARK1	CATCCATTCCTCCTGCTGTATCAT	TTTCCTCTCTGGCCCTACAAGA
1 MARK1	CACCAGAAGTCCATGTCCACTT	GTGATGGTGACTATCATGTATTTTCAC A
1 MARK1	ACTAGCAGAAAATTGTGGGCCAA	TCTTCCATGGGCAACTAAGTAATCAA AT
1 MARK1	CTGCTTTACCTTTCTGATTCAGGTGA	GTACATCGCTACAATTATCAGCAAAA CA
1 MARK1	CTTTCGATGGCCAGAATTTAAAGGT	ACAGTCTGTGGACATATAGAAGGGAA T
1 MARK1	GAGTTTTACGAGGGAAGTACCGT	AATGCTCTTCGGTTCCTGGaatattt
1 MARK1	GTAGGGCCAAAAGAAGTAGCCA	CGGTCTGGAGTCGCAGTACTAA
1 MARK1	GCTCAAACTTTAAAAGGATGTAGTGC A	GGCTGGATGTGTGGTTCAGTATA
1 MARK1	TTTTTCTTAGCATACATCTGTGGATGG A	CAATGTGAGGCTGTTCATCTGTTG
1 MARK1	CTTCCCCATCTGCTCACAGTA	CACCCGTTTCATGGGATGGT
1 MARK1	CTTATAATGGGCCACCTGCTTC	GAATCAAAACACGACAGTACATTGGT
1 MARK1	CGGTGTAGAAACTCCATTACGTCAG	CCAGGCAGATATTAAAAGGCATTGTG
1 MARK1	ATCTAGTTAGGAATGAAACAGAAATT GGAT	TTGTAGACTGGTAGGATTTAGCTGAG T
1 MARK1	GTCCGCACCCCTTTCCT	GGGAGGCTCCGGTTACTCA
1 MARK1	CAAAGATGGCAAACTTGCCCAT	GGCATCATTTATTTCATCTCGTGCAA
1 MARK1	ACATTATGGTCACCATGGGCTTT	AGGTGAAAAACTTACTTCAGGTGGTT T
1 MARK1	GCACGAGATGAAATAAATGATGCCTT AA	TATTAGCTCAAGAAATAATTCACAGT AATCTGA
1 MARK1	TCTGTGGATTTTTCTCAAAGTCAGCTT	GGTCCTTTCACAGACATATGTATTCCT T
1 MARK1	ATTCTGGAGGTAGCATGGCAAG	TTCAGCAAGGGCACTAACACA
1 MARK1	TCTTAAAGCATATTCAATCTGTGCTA ACAGT	CAAAGAGAACTTTTCAATCACCCTCA T
1 MARK1	AATCCGAATTAGTAATTTATCTAAAT TGGAGGT	TCACGGTGAACAATGTACTTTTGATG A
1 MARK1	CCTTCACTTTGTTTTGAGGGATCCAA	GTGCTGTAAGTATTTCAAAAGTACGT TTTCA
1 MCL1	CAAACAGCCGTGCGTCATAAA	CAAAACGGGACTGGCTAGTTAAAC
1 MCL1	GGCAAACTTACCCAGCCTCTTT	GGATTGTGACTCTCATTTCTTTTGGTG
1 MCL1	GTCTTCAAGTGTTTAGCCACAAAGG	GACCTGCATGCTTTTCTTTCTCA
1 MCL1	CGACCCTCCGGGAGTCT	GACTTTTGGCTACGGAGAAGGA
1 MCL1	CCTCCCCTCCCCCTATCT	GGCGACTGGCAATGTTTGG
1 MCL1	CGATTACCGCGTTTCTTTTGAGG	CGCCCTAAAACCGTGATAAAGGA
1 MCL1	TGGTGGTGGTTGGTTAAAAGTCA	GTGGAGTTCTTCCATGTAGAGGAC
1 MCL1	CACATTCCTGATGCCACCTTCTA	CCTTCTCTCTGGATAGTAGTGTTGTAA GA
1 MCL1	ATGAGACACGTTTCAACACTGACT	GAGACCTTACGACGGGTTGG
1 MCL1	CTTACCTTGGAAGGCCGTCT	GCAGTCGCTGGAGATTATCTCT

1 MCL1	GCCTGCTCCCGAAGGTA	CCGCTGCTGGAGTTGGTC
1 MCL1	ACCCGTCCGTACTGGTGTTATTA	CGCTTGAGGAGATGGAAGCC
1 MCL1	CCTCTTCGGGCGACATGAT	CACCCTCACGCCAGACTC
1 MDM4	GTAGTGTGACGACATTGAGTTTTGG	CTAACTGCTCTGATACTGACTCATTCAAA
1 MDM4	GTTTCAGATACTACAGATGACTTGTGGTT	GCACACAAACAGAATCAACATGGAATTT
1 MDM4	GGCTTCATGCCCTATTTGCAAG	CTAAGAACATTCTCTGACAGGTTGGAAA
1 MDM4	CTAGATCACTGGTGTGGAATAGCTT	CAGTACCTCTTGCTTGGAGAGTTAAATT
1 MDM4	TGGCAGTGTACTGAATGCAAGA	GGACATCATTTCCTTCATTTTCCTTTTCA
1 MDM4	CACGTCTGATATCACTGCCATACC	CTGTGAGCCAAATCCAAGAATTCC
1 MDM4	ACTTTTTGATCCCTGCAACTCAGT	TTTCTCACATAAGCTACATGGCTTCAA
1 MDM4	ACATGGAGGATTGCCAGAATCTC	ACCAGCTGAATCTCTTTCTTGCAAA
1 MDM4	GAGGTTCTCTTGTTCCATAGTTTCAGAA	TTTGATGGCTTACCTCTTTAACAGTGAA
1 MDM4	TGGTGAGCCAGAATGGAACTG	AATGTATGCATTTATGCTCTGAGGTAGG
1 MDM4	CAGAAGACGATATCCCCACACTG	AGAAATTGTGACATATCCCTCTCCTACT
1 MDM4	GATGAAACATCTAGGCTGGACCTT	TGTCTGTAAATCAGTTGAGCCATTACTT
1 MDM4	CTTGGTGGTTTTTAGGAAACTTGAGAA	GTGTGGTCAAGAGTAACCAAGAATGT
1 MDM4	GGTTGCCTTTGTGTGAATGCTAA	CAGGAGAGATCCTGCAAGCA
1 MDM4	AGTGCTAGAGATGGGAGAAGAATGT	ACTTAGAGTCCTCCAGGTCATCATT
1 MDM4	TTCACAGGTGATTGAAGTGGGAAA	CAGCACTCTTCAACACAAGTGAAC
1 MDM4	ACGGCAAACCACTGATATCTTCAT	TCAAAGCTGTGATACAGACTGAAGAAA
1 MDM4	GCTGAGGTCCTTTTTGTGTGGAA	CAACAAAAGAAGTCACCGTGTGG
1 MDM4	TCTTTCTTTAGCAAACTAACTTACCTTACCTC	GTTGCTTCACCATTATGTACTGACCTA
1 MDM4	TTCCATGCAGGTCATGCACTATT	CGCAAGCCCTCACTGTTTTTAC
1 MPL	CATGGCTCAGTCTGCTTCTCT	TCCAGTACCAGGCAGGGTT
1 MPL	GAAGTCTGACCCTTTTTGTCTCCTA	CTTCGGCTCCACCTGGTC
1 MPL	AGCTGACAGGCAGACCTAGAT	CGAGGGAGATCCCATCAGGTT
1 MPL	CAGCCTGGCAACTCCTACTG	CTTTTATCTCCTCCCCATCTCCCT
1 MPL	CCAGCTTCCTGAAGGGAGGAT	CTGGGCATAGGTGATCTCCAC
1 MPL	CCAACTCACCAGCTGTTCCTTA	GGCACAGTCCAGCACCTAC
1 MPL	AGGGAAAGAAGAGAGAATAGGAGTCAA	GTCCAAGGTAAAGCATTGCAGT
1 MPL	CTTCTTTGACTTTAGTGGCACTTGG	CTCAATCAGCAGTTCAGCTCTCA
1 MPL	CAGAGGCTGAGCCATAGACTG	TCCTCCCAGCTGATCTGAAGTT
1 MPL	CAGTATCATCAAGGCCATGGGT	AGCAGGTTTCTGTGGCAATCA
1 MPL	ACTGGTCCCACGGTCATACA	GGGCAGAAGAAGGTCAGCATA
1 MPL	CAGACGCTGGGCTATCGAA	TCCAGGGACCTTGGTAGGTG
1 MPL	CGCGATCTCGCTACCGTTTA	GCTGTGCGGCTTTGGTG
1 MPL	CTGTGTAGGAGGGACCTCTTCTAT	GAAGAGACGCACTTCCTCCTG

1 MPL	GATACGTGTGCCAGTTTCCAGA	GGTGACAGGAGGATGGCTCTTA
1 MPL	GCAGGCCTGATTCAATGACTCT	CCACAAGGATGTGAATAATGCTGTCA
1 MPL	CTGCCACTTCAAGTCACGAAA	GTGGGAAGAATGAGGAAGAAAGTTCTTA
1 MPL	TGCTTTAATCCAGCGCCTCTC	GCTGCAATCTTCGGTAGTCCAT
1 MPL	GACTCCTTTGCCCCTGTGTT	CTGCTGCCAATAGCTTAGTGGT
1 MPL	ACATTGCCAACCATTCCTACCT	GTAGGGTAGGGAAGTTCACAGAGAA
1 MPL	CCCCAACTCTGACCCTTCTGA	TCCAATTCCAGATGCCCACTG
1 MPL	CAAACTTGCACTGGAGGGAGAT	GTAGTGAGGTCTGTGGGCATTT
1 MTOR	CTTAGAGGTTAGTTTCTTTTCCACCCT	TGAAAGATCCAGACCCTGATCCA
1 MTOR	GGACATTATTGATCACACCTGGGTT	GAGATGGTCTCTTGGGTGAATTTTACTT
1 MTOR	GGAAAAGTGAGGTGTGGAGCTT	GGGTACAGCTCTCACCAAGG
1 MTOR	GGAGGTCCCAAATCCCATGAG	AGCAGCTGGTACACGACAAG
1 MTOR	GAAGCCCATGAGATCTTTGCAGTA	CCAGTTTGAATGCCAGTCATAAGTAAAG
1 MTOR	AGCCCTACTTCCTTAGCACTGTAT	CATCCAGATTGATACCTGGCTACA
1 MTOR	CACCTGTAACCAAGTATCCTCACC	GCAGGCAAAAACTGGGAAACTAA
1 MTOR	GAGGGCCATCCTATTGCGA	ACCACTTGACTCTGATGCAGAAG
1 MTOR	GCATGCTCAAACACCTCCAC	GTTTCTTCCTGGACCCAAACTACAT
1 MTOR	CCTGACATTGTGAGTAGGTGGT	CCTCAGGTGACCTATGCCTACAT
1 MTOR	GGGCACTCTTCCACATGTTTTTC	GTCCAGTTCTGTCTATAACCCAGTTTC
1 MTOR	CCAGCCTCTCGGTTTGTGTTA	GAACATATGAGCCTTTCTTCCCTACAG
1 MTOR	CAGCTCGTTCCCGATATCCA	CTGGCTCCAGGTAAAGAAAATAAACATT
1 MTOR	CCAAACCAGTGGTTAGATGAGAAACT	CAAGAATTGATACGCCCAGACC
1 MTOR	TGAATGAGACGTCCCACCAAG	CAGAGCATCAACCGAAACAAGTTAAAA
1 MTOR	CTGAGATGGGTAATGATGTCTTCCA	GCCAACCCTTTATCCTTCCTCTCT
1 MTOR	GAAGCCCACCCCACTCTAC	TCCCTAGCAAATACCAAAGGCAA
1 MTOR	GAGCCTGGACTCCCCTCT	GATGAGACCTTGGCCAAAGAGAA
1 MTOR	CCGATCATCCCGATTCATGCC	GATGGGATGGGCCTGTATTCTT
1 MTOR	GCCTCAAAAATGACAATGTGCAGA	GTCCCAAGGGTTGTTTCTCTTCTA
1 MTOR	CAAGTAGTAAAGACAACAGGGACTTCAG	CATCTCTTCCCCCTCCTGTTTTAG
1 MTOR	GGAAAAGTGATCACCCGGGAA	CCCTGATCTGTCTTCTGTTTCTCAAAG
1 MTOR	cTCCCAGGCACTTGATGATACTC	CATTTCTAGGTTACAGGCCTGGAT
1 MTOR	AGGCAGGAAAAGCAAGTTGAGTA	CAAACTTGTCCCCGAGCGA
1 MTOR	CAGATCTGGCGGATGATCTCT	TGTCTTTTGGATTGTTCGTGCAGTA
1 MTOR	GTGTGGCATGTGATTCTGTAGTTG	CATTCTCACCCTGATACGTCAACAT
1 MTOR	GCTCCATCTGGCACTTCAGATA	CATCGCCGCTGCAGAAG

1	MTOR	GGGCTGTTCTCGGTGCT	CGATGAGAAGAAGAAACTGCGTCAT
1	MTOR	CCTCGCTCTCACTGTTGCTG	CGAAGCTGTGCTACACTACAAACAT
1	MTOR	GCATGACGCAGTTTCTTCTTCTCA	GTCACAGCTCCAGTGTCTGTC
1	MTOR	CCACCTAGAGCCAGCACCTTA	TGTGAAAAGTGGACCCTGGTTAATG
1	MTOR	ACCCGCCCTGACACACTATA	CAAAGATGACTGGCTGGAATGG
1	MTOR	ATGATGAGTCCTTCAGCAGCTC	AATTGGCCCTTGAAACTGATTGTTTT
1	MTOR	CCATCTTGGCTTGGGTCTCAT	GGAAGATTGGTAGTTTAAGGAGATTTGGA
1	MTOR	AAACAAGAGAAGGCTGTGTGGAT	ACATCGCTGAAGTCACACAGAC
1	MTOR	CCATGAATTCAGCCAAGTTTAAGAGG	ATTTTCCACCTTCTCTGCCTTCA
1	MTOR	CTGGCCATCACCTCGGTTA	TGTTTGTGGCTCTGAATGACCA
1	MTOR	GTGGCAGAATATTTCTACAGGGTTATGT	AGGCATCTCGTTTGTACTTTGGG
1	MTOR	CGCAGAAGCTCAGCTGTAAC	GCATCAATGAGAGCACAATCCC
1	MTOR	GCTCCCGGATCTCAAACACC	TGTTCAAAGGCTCCATTGCTCTT
1	MTOR	GCTGTAGTACTGCAGCACTTTG	TTTTGAGGAGGGAATGTCATGGG
1	MTOR	AAACATGCCTTTCACGTTCCTTTC	TCTCTCCTTCTTCTCTTGGCTTCA
1	MTOR	TGTCCCAAAGCAGAAGTAAAACCA	CCAAGCCTGGGACCTCTATTATCAT
1	MTOR	CATGTGTGGTGCAGAGGAGAAA	CATCACCTTCATCTTCAAGTCCCT
1	MTOR	GCTTTGAGATTCGTCGGAACAC	GATAACTTGAAAACGTGCCTGCTTT
1	MTOR	GGAACTGCACACATTTGAGTCC	GAAATGCTGGTCAACATGGGAAA
1	MTOR	TGGGAATGGAGCCATCTCCTTA	AGCAAGTGCAACCCTTCTTTGA
1	MTOR	GGTAGAACTCATCCAGAGGCAAG	CTAACCATGCTCTGCCTGAAGT
1	MTOR	CACACGGCCACAAAAATGTTG	CAATGAGAGTTCTCGGTTTGCTTTT
1	MTOR	CTGAGAGATCTGGGTGCATGT	TGCTTTCTGTCTATGTGTGTGTGTATAG
1	MTOR	GAAGACTTCTCAAATTGTTGCCATTTCA	ACGGACATGAGTTTGTTTTCCTTCT
1	MTOR	CGCAGATCTTCATGGCCTTTT	GGGATCTCTTGAATGCAGTGGTG
1	MTOR	TCTCCTTTCCCAGTCACCTGAA	GGAGGATCCTTTGATTTACCAGCATC
1	MTOR	TTGGCCACTCCTAAGCATCC	TCTGCACCTCCCAAAATTTAAGGATAT
1	MTOR	CGAGGAAGAAAAACAATCCCACTTG	AATGCAGCCAACAAGATTCTGAAG
1	MTOR	GTTGCTGTGCTCACACATGTT	TCCTTAAAATTCATACCAGCTCTTCCC
1	MTOR	GCTAGTCACTGGTGCGGTTC	TGGCTTTTTGGTGTTTGAATTTTCTGTT
1	MTOR	gggtctGTCTTGCTCAATCAG	CTTTCTGCCACCCTCTTTTTCATT
1	MTOR	GCCACGGGCTTCTTAAATTTCAT	CAGATGCCAATGAGAGGAAAGGT
1	MTOR	CCTTACCTATGGCCAAGATGCC	TCCCACCACCACAGTTAGAGAATTATTA

1 MTOR	ATTTTTCAGTGACAGACATACAGAGAGG	CATCCGGGACTACAGGGAGAA
1 MTOR	GCTCGATGTTGAGAAGGATCTTCTT	TTTACGGTACACTAACCCTGCTTTT
1 MTOR	ATCTCTCTGGAGGATGACGTAGG	TCAGAGCACGATCATTCTTCTCATTG
1 MTOR	CCCAAGAGCTACCACAATTTGCT	TCATCAGAAAGGGACCTGACTCA
1 MTOR	GGATGACAGGGCTGGAATATGAC	ATCGATGCCTTCCAGCACAT
1 MTOR	GCATGGTCTGGACAAAATGCTG	TCATTCTGTGCTCTAGGGTATTCTGT
1 MTOR	GTCACTTTGAGAGCCCCACTC	GAAATGTTTTTGTTCCTCCTGTGCTA
1 MTOR	GGGAAGCCTTCCTTTCAAATCC	GGCCTATGACAAGAAAATGGACAC
1 MTOR	GCTCTGGGTCGTCCTTGTTG	ACAGCAGTCTTTCTTTCCCATGTAA
1 MTOR	CGTCCTTCCTCTCCAACCAAATG	CTTTCTGTGGCTGTGAGGTCT
1 MTOR	GCGAGGCAAATAGACCTTAAACTC	GCTATCTACTTTGTCTTTTCTGTGCTCA
1 MTOR	CAGCTCCAGACTTTCTAAACACAAC	AAGCACAAAGTGAACATTGGCAT
1 MTOR	CATCCCGGGACTGGTCTATC	ATTATGTAACCTGAGAGGAGTATCCGT
1 MTOR	ACTGGCTACTCCCAATTGTCCTA	CCTCCATCCACCTCATCAGTG
1 MTOR	GTCTGGCTAACCACATGAGCAT	CTTTCTATCCAGGCCACTCTCTG
1 MTOR	CAGTGGCGAACAAATTGGGT	AGTAGCCTCAAAATGGAAAATTCAGCTA
1 MTOR	CTTGCTTCTGAGCCTTCCTTGA	GCTGGTTGACCTCTTGTCCATTT
1 MTOR	GATACAGGGCAAGCTCAGGTT	CATTTCTCTACAGGAGGCAGAAGG
1 MTOR	CAAGTGAAGACTGTGGCATCCA	gCCTGAGTAACCTGCTCTCCT
1 MTOR	TGGAAAACCAAATGAAACCATTCAGG	CCGTTCTTTAGCGGTCATGTCAA
1 MTOR	CCAGGCCTAAAATATACCCAACCA	tctcaaaaGTACAAACCAAAGACAGCTTA
1 MTOR	CCATGTGGGTGGGTGGTT	AATGTTCCAGGGCTGCCA
1 MTOR	CATGGCCTACCAGAGTTGCAT	CTAACACGTGTGTGTTCCAACAG
1 MTOR	GCTCCTCCCTGTAGACACTCA	GTTGTCATGGAAATGGCATCCAA
1 MTOR	CATGGCAAGACGGCCAATG	CATCATCACCTTTTGCCTTCCTCT
1 MTOR	TGCTAGTCCCAAAGAGGAGGT	CACCATGCTTTTGCTCCCTTTT
1 MTOR	GGCCAATAACTCCCTAGCCA	TTGAGTGTAACTACCTTTTCCCTTTGTT
1 MTOR	TGTCCCTTTTAAGTAAACACATGACACA	AACCTGTTGTATTGCTCCCATTCTTA
1 MTOR	GGAGAAGAGAGGTCATTTTGCATGA	GCATATGCCAAAGCACTACACTACAA
1 MTOR	GCCTTTCTGGAACTCCAGTTCT	GCTGTGAGGTTTTGCTCTTCTC
1 MTOR	TGTCTGTCCAGACTCCCATCTT	GGAGTCCCTGGATTTCACTGA
1 MTOR	AGGGACAGGAAGTCTGACTGA	CTTGAGGTGCTACTGAATTTTCTGAAG
1 MTOR	GTGAATGATCCGGGAGGCATAG	CTTCCTTGGAGATTGTATGTTAGCAGTTA
1 MTOR	TGTACCCTGGTTCTGCTCAGT	AATCGTGTTCTTGTGCCTATCCA
1 MTOR	TCCTAGATACCTTCATTCCTTTCCCAAA	TGTGGGCAGCATCACTCTTG

1 MTOR	GCTGCCAAGCGTTCGGA	TCAAGATGGGCTACTGAAAATGCT
1 MTOR	GTTTGTGCATAAGGACCAGGGA	CTTCCCAAGAACAGACTGAGTGT
1 MTOR	CAAACTTTCTCACCATGGTTTCAGTT	TCCTTTTCTTTCTGCCTTCTTAGGTG
1 MTOR	TGGGAATGGCAAGCAGTAATTTCA	CCAATCATTCGCATTCAGTCCAT
1 MTOR	TGACTTGCAAAGACGGTGCT	AGGCAGAACACTAAATCCTTTGGTAATT
1 MTOR	CCCACACATCCCACAATGACT	TCTAGCAATGTGAGCGTCCTG
1 MTOR	TTTCCTCATTCCGGCTCTTTAGG	TCTCTCTAAAGAACCTCAGGGCAA
1 MTR	acagttTGGTTAGTGATGAGTTGTATCC	CCAGTTCAGGCCCCTGTAAC
1 MTR	CAGGAAGTATAGACGGCTTTTATGTTGA	CTGATATGATCTGGTGTTGACCCA
1 MTR	ATTTTCCCTGTGTTGCTCCCT	CCACATATAATGCAACAGCTAGCTTAC
1 MTR	gatcgctgACTTAAGGTATCACTGTAA	GGGTCTTTATTCCAGATGAGATCTTCAC
1 MTR	CCATAAGGAACTTCTGCAGCTCT	CATGAAGGTCTACTAACTGTAGGTGAGT
1 MTR	TCAGTTTTGTCTCTAATGGGCTTTCA	AATGCCCAAAGTATAGTTGCACAGTA
1 MTR	GGACACACTCTCAGAAATAAAGACGTT	GGGAAAAACATGCTCAAAAGGACAATTA
1 MTR	GTGGTTTGGGAAAGATACCTGATCT	CCATAGGCCTGAACAAAATGACTTG
1 MTR	CAGCGTCAGCATTGACAACC	ACATCAAAGAAAGGCTTCCAGTCA
1 MTR	GCAGAAGCTGGTGGACTACAT	AGAAAGGAAGGACTTACTGCACTAAC
1 MTR	CAGTGAGTGAAATGTGCTCTAATGT	CACACTCTGATTTTTGTGTCTGTTTCTG
1 MTR	TCTTTTGCTCTTTTCCCTAGGCAA	CATACAAGTTGTGTTCCTCCATTCC
1 MTR	ACCCTAATATCCTAACCATTGGGACT	GGGACAAATTTGAGCCATGACTTG
1 MTR	CCTGTGGAGAGCACGTCTTC	GGAATCGCCCCAGGGATTA
1 MTR	AAAAATGTTCAGCCACTTAGAGATCTCA	CTGATGTTCCTATAATCCGGCCTT
1 MTR	GGGCACAAGAAACTCTAAAGCTCATA	CATCTTAGACACATGGAACAATGGAATT
1 MTR	CTGAATTACTTTGAACTTCGGATGCTTT	GACAAGTTGGAAAGACCTCCACTTAT
1 MTR	GGAAACATTACCTGGAGCCAGA	GCAGGGCTAAGAGTTCTTCTACCATA
1 MTR	tCATTTGACGATACCCGATTGCT	TGCAAGGGAGCGATGAAGTC
1 MTR	TTGCTCATCTATGGCTATCTTGCATT	AGCAAAAATCTGTTTCTACCACTTACCT
1 MTR	GGAGCCATACTACTGCCTCTCA	CCATGATGCTGCTGTAGTCGTC
1 MTR	AGACAGGACCATTATGAGTCTCTCAA	GCCTTTTACACTCCTCAAAACCATTTTA
1 MTR	CAAGGCCTATGAGGATGATGGT	GTTCTAAGGGCTGATCAGTCTTCA
1 MTR	GTGTTTGTTTTTGCAAGTAGTCACCat	GCGTTTCATCATAGTCACCAAAGGT
1 MTR	AATTATCCTTTCTCAGGTCTTCCCAAC	GGATTTCTTCAGGGAAAATTCTCCTTCT
1 MTR	CTCTGTGGAGGTAAGGCAGTT	TTTTGGTTTAACCTGGCTTCCTCA

1 MTR	AGGATACTGAGGAAGCCAGGT	GCAACTGTGAAAATTTCTCCCCATATT
1 MTR	TATTCTGAATTAATGGATACAATGTTTCCTCTT	CTGCAATCAGGAATACCAGGTCTTTAT
1 MTR	CCTAAACAACCCTGCCTTGCT	GTTGAAAGAGGTGAGGTCCTAACTTAC
1 MTR	TCCATTATTTATTGTGCGGAGGAAAAGA	CAAAGGAACCACGTGTGCAAAA
1 MTR	GATTGCTGGGAAGGGTAAGGAATT	CCATCTAGCATGCCATCATCCAT
1 MTR	CCCAGGTGTTGGATGTCAAC	GAGACAAGTAAATCCCCCTGATAAACAT
1 MTR	GGGTGTCCTCATGGTTCTAATTCA	CTGCTCACTGCCACAGTAGG
1 MTR	CCATGAAAGAGTTCGCCGAGAA	CTCGATGTCTGCGAGTCTCC
1 MTR	CACCGAGAAGCTCACCATGT	CCCTAAGTGCCAGTATGCTGATAAG
1 MTR	TCTAAAGCTCCTCCCTCACACAT	CATCCAGCACCATAATCCTCTTCTG
1 MTR	GGGATGAGATCAATGCCATTCTG	GGCCTGGCATGATCTTTAAATTCCT
1 MTR	AACGAAGAACACTTCCGAGGTC	ACATGGGAACCTGGGAATACTTTAC
1 MTR	CTGATGTGCTGGGTGTGATTTATTTT	ACCCCAGCCTTGTCTGTTTTT
1 MTR	TGATGGTAGTTGAATAAAAGTGCTTTGA	GCTGCACCCAAAGCACAATT
1 MTR	AGCATTGGATTAAATTGTGCTTTGGG	TCTCTTACCTCATCTATGCCATGTCTT
1 MTR	TCCTATCTGCTCAGATGGGCTAT	AAGTTGGTGTACGGTCCAATCC
1 MTR	TCCTTTTAAGGTCTAGAGCCCTTCA	CTAGCCAAAGACATGGGCAGAT
1 MTR	AGCATTGTTTGATGGTTATTTTTGGCA	TCTTTTGACTAATCAGTGTGTTCAGCAT
1 MTR	GGTCTACGATGATGCCCACAAT	CCTCTGCGTACAGGTGAATGTC
1 MTR	GCCAGCACAGAGTATCCAAGAC	GCTGGCTGTCAGTACACCTTC
1 MTR	AGAGTTGTATAGGAACTGTCAGTGCT	TGGAATAAAATGTAAGGTCACCCACTT
1 MTR	TTGGACATGTTTTCACAGTAGTTGTAGA	TCTTTCTCTAGTCCATAGCCAAACAGA
1 MTR	AAAGAGCAAAGGAAGTCAGTGTGT	GGAAAGAGTCCGCCCACTTTTA
1 MTR	CAGATTTCAGGGACGATCGTTGA	CCATCAGAAAAGGTTCCCCAAAAA
1 MTR	CCTGTGGCCTCTGTGTGATTTT	GACTAATGCTATTGACAATGCACTTCC
1 MTR	CTGGGTTAAAGTGCTGCCAAG	CAATGGATTAGGCCAAAAGAAACCA
1 MTR	GCAAGGTACTTAATGTTTCTTGGCAAA	CCACAGATATGTTCTTCCTCAATGCATA
1 MTR	TTCTCATCTTTTGCAGGTTGAGGAT	CCCTAGGTTGTATTTCCTTGAGGATCA
1 MTR	TTCCTTTTTCTTTCATCTTCCTCACTGT	CCATTTCCTTGGCAACAAAAATCATTTC
1 MTR	ACTCATCACTCCTTCCCTGGAT	TTTGAAGTGAGGACAAAGCTCATTAGT
1 MUC1	GGAAGGAAAGGCCGATACTCA	CACTTCTCCCCAGTTGTCTACT
1 MUC1	CCCCAACCTTAAGTGCACCAG	TTCCCTCTCACCCCAGTTTTTG
1 MUC1	GAGGTTTGAAATGTGAAAAGACAGGAA	GCCACCACTCTGATACTCCTACC

1 MUC1	CAGGGACTGCACTCACCAA	CCCTGACCACTGCTTTTCCTTTTA
1 MUC1	CATCCTGTCCCTGAGTGGTG	TTACGGGTTCTGGTCATGCAA
1 MUC1	GCCGAAGTCTCCTTTTCTCCAC	TGGTGGCCAGAGCTTATATTTTCTT
1 MUC1	GGCTGGCCTGGTGACTG	AGTATGACCAGCAGCGTACTCT
1 MUC1	CCATCTGCCAGGGAGCAAA	CTCCCCACCCATTTCACCA
1 MUC1	GCTGCCAGCACTAAGGAAAGAG	CGGAAGCAGCCTCTCGATAT
1 MUC1	GACGTCTGAGATCGTCAGGTT	CTGAAGTGCCCATTTCCCTGT
1 MUC1	CCCTGAAGAACCTGAGTGGAGT	CTCACCCTGGATCTCTTTTCCTT
1 MUC1	CTTCTCACCTCTTCCAAGCTCTTT	GAGTACCCCACCTACCACAC
1 MUC1	GGCCCAATCTCACCTTCTCATA	GTGCCGCCGAAAGAACTAC
1 MUC1	GTATCCCGGGCTGGAAAGATG	GCTGGCCATTGTCTATCTCATTG
1 MUC1	GCTATGGCTGGCAAGGGT	GCCCCTCCAGTCCACAA
1 MUTYH	CTACACCCACCCCAAAGTAGA	GTCCTCATGCCAACCCCTTT
1 MUTYH	ATGTAGAACATGTAGGAAACACAAGGAA	GTGACCACCGTACCACCAG
1 MUTYH	CAGCTGCGGTGTGAAATTCC	ATCCACAGGCCTATTTGAACCC
1 MUTYH	CATGAGGACACTGCTGACCT	CCCTCCACCCTAACTCCTCATC
1 MUTYH	ACTCAGGTTAGAGGAAGAACTGGAA	CCCTATGACACTCAACCCTGTG
1 MUTYH	CCACGCCCAGTATCCAGGTA	CCCTGGGAGTGGTCAACTTC
1 MUTYH	CTTGCGGCTGGCCTTTC	GGCCAAAGCCCACTCTCT
1 MUTYH	GCCTGTCCAGGTCCATCTCAT	TGCTAAGCTGGTACGACCAAG
1 MUTYH	TCCATGGTAGGTCCCGTTTCT	GGGATGATTGCTGAGTGTCCTG
1 MUTYH	GTGACTTCAGCTACGTCTCTGAAT	CTGTGTCCCAAGACCCTGATG
1 MUTYH	GGCACAGGGTTGAGTGTCAT	CCACAGTGTGTACCCCACAG
1 MUTYH	GCACAGGCTCTCCACAGG	TGCAAAGGAGCTCTGCTTCA
1 MUTYH	CACCTGATTGGAGTGCAAGAC	TTGACCTCTGATCCTACCCACA
1 MUTYH	GGGACGTCTGAACGGAAGT	TGAAACTGCGCCATCGTCA
1 MUTYH	GGGTGGGCTGTGAGATCA	TCTTGAGTCTTGCACTCCAATCAG
1 MUTYH	GAAGCCTGGAGTGGAGAATGTT	CTCACCTCCCTGTCTTCTTGTCTA
1 MUTYH	CCTTGGGCCACAACCTAGTTC	GGGTCTTTTTGTTTCAGGCCATC
1 MUTYH	CCTTTGCAGACACCCCTGAAG	CTAACTCTTTGGCCCCTCTGTG
1 MUTYH	AACAACAGGATTCTCAGGGAATGG	TCTCCGTGCAGTCGGAAAAA
1 MUTYH	AGATGTGAGACCGAAAGAAATTATCCAG	ACTACAAGGCCTCCCTCCTT
1 MUTYH	ATTCACCTCCTGTGGGTAGGAT	CTTGACCTTGTCTCTTTCTGCCT
1 MUTYH	AGGCCTGTGGATATAGCCTCAA	GAGTTCCCGTCCGTGACC
1 MUTYH	GCCCAACGCTGTAGTTCCTG	AGTGGCATGAGTAACAAGAGAGAATG
1 MYCL1	GTGGCCCCCTCTTGGAT	GCACTCCCAGCCTCGAA
1 MYCL1	GCTTCGAGGCTGGGAGT	CAGGAACTACGCCTCCATCA
1 MYCL1	GTTCCTGCCCCAGCCTTT	GAAATTCGAGCTGGTGCCATC
1 MYCL1	CGGGATTCCGCTTCGTCT	CCAGCGAGGACATCTGGAA
1 MYCL1	CGATGGCACCAGCTCGAATT	AGGGAGCGGACATGGACTA
1 MYCL1	AGTCATAGTCGTAGAAATAGTGCTGGTA	CGACTGTGCCGAGGGAC
1 MYCL1	AGTTGTGCTGTTGCTGATGGA	CTTCTCTTGATCTCTTTTCTCGGACA

1	MYCL1	GTAGCCAGTGAGGTATGCAATTCT	CCCAAAGTAGTGATCCTAAGCAAGG
1	MYCL1	CCACCAGGGCTTGCAAGTATT	TGAGGATGTGACCAAGAGGAAGA
1	MYCL1	GCGCTCCAGGAAGTTGTGAT	CAAGAAGAGGTTCTGGAGAGAGATG
1	MYCL1	TCTCTTCATCCTCCTCATCTTCCTTTT	GCATGAAGCATTTCCACATCTCCA
1	MYCL1	CTGTCCCACCATAACCAAAATGTG	ACAGAGAAAAGACAGCTCCGATG
1	MYCL1	GGGCTCCAATAATCATCAAAGGGA	CTCAAGCTCTTGGGCCATCT
1	MYCL1	GACTACACGGGCAGCTTCA	CAAGCGACTCGGGTAAGGA
1	NOTCH2	GTGCTATATGTCAAAGTGCTAGGCT	GGGAACTCATGGTGTACCCCTATT
1	NOTCH2	CATAGCAGCTGACTTCTCACCAT	AAGGTACCCTGGTTATTGTGGTATTG
1	NOTCH2	AGCAGTTGTTCAGGTGGCAT	TGACAAATACTGTGCAGACCACTTC
1	NOTCH2	CCTGGTCACAGTGGTTGTCTTT	GGTAGGAAAATCCACAGTAGTTCCAA AT
1	NOTCH2	CACACATTCTCTCCCTGCTCTC	ACAATGGGCAGTGTGTGGATAAA
1	NOTCH2	GCTGAGTGAATGGGAAGGAAGTC	AGATCAATGAATGCAGCTCTCATCC
1	NOTCH2	CAGGCACTGGAAACGATTGAC	CACCTTTCTCTCTCTTTAAGCCACTT
1	NOTCH2	CACGTTCCCTCATTCAGGCAT	CTGATGTTGCTCTGTTTGCTTCTTAG
1	NOTCH2	TTTATGACTGGACAGGGCAAACA	TCCGTAGGACACCATCGCT
1	NOTCH2	TCTAACACATCCACCTCCTGCT	AGGGAGCATTGTTTTCACCTTTCA
1	NOTCH2	CCAACTGGCTTAACACAGGTCAA	GCTGTATATGCCCCGAGGGA
1	NOTCH2	GGCATTCGTTCACCTGTGAGT	TGACTAACACTGTCCCCCTTCT
1	NOTCH2	ATCCCCTGAACACTAAGAATGGATT	AGGCACCTGTATTGACCTTGTG
1	NOTCH2	GGCAAGAGCACTTGAAATGGTT	GGTCTGTTTCCAGTAATGGTCCAATAT
1	NOTCH2	GGATCCATGTGGACACCTCATC	ATGGAGTCCATTGTGAGAACAACAT
1	NOTCH2	TCACCTCTCAGTGCACTCATTG	GGATAAGTGCCAGACAGACATGAAT
1	NOTCH2	CTTACAGGGTTCACTCAGACACTC	TGATGCTACTCTTCAGAGGTCTAATTC T
1	NOTCH2	CAGTCTGCCTCTGGTTACCAAT	GATGACTGCCCTAACCACAGG
1	NOTCH2	CACAAACCCCTCCATTCTGACA	CAGGTCGTTTCTTCTGTATGAGATTTC
1	NOTCH2	TGGCTTATTGGCAATGCCTCTT	GCGTAAGCATGGCTCTCTCTG
1	NOTCH2	CGAAGAGTGAAACCTTCAGGCA	CCCTGTCTCTGTGGACTTTCAG
1	NOTCH2	AGTTTAAACATTTGCTGAGCTCAACA G	CCAACCCCCTAAATGGGCAATATA
1	NOTCH2	CAGCCCCTTTGTAGCCTTGT	GACATTTTCTCTTAGTTGTCCTTTGCC
1	NOTCH2	CTGAGACTCAGGCACTGACAA	GAACCTGCTTTGATGACATAAGTGG
1	NOTCH2	AGCACACAGTGGCAAGTGTAG	TGAGGCTGTCAGAAAGTTTTACTGTTT
1	NOTCH2	CCTACCAAGGAGATGAAAAGTGAGA A	TCATGTTACCACTGTCCCTTTTTGAG

1	NOTCH2	CTGACAACATAAACCACAACATCAGAG	CCATCCAGGGCATTGTCTTAAGTTATA
1	NOTCH2	TCCCATTTCGAAGATTGGTAAAACCA	GAATGGAGGGACTTGTGCTGT
1	NOTCH2	TGAAACCATCAGGCATGTTACTGG	TTGCAGTGACTACTTCTGCCTTT
1	NOTCH2	GGTATCTGCTGAAGGTAGGAAACC	GGAGAGGACTGCCAGTACTCA
1	NOTCH2	ACTATCACTCTTTACTCCCATTCAGGA	ACCTAATTGGTACTGGAACAAGTGAAC
1	NOTCH2	CCCACAGATGACAGGTGAGAGA	CTGTTAGACCATTTTGCCAATCGAG
1	NOTCH2	CCCTTCATCATCGACCCAGT	CATGGTGGAAAGTGTTGAAAACCA
1	NOTCH2	GACGATCCATATGGTCTGTGATGT	TTCTGTGATGGGCCTTTTCTGTAG
1	NOTCH2	GCTCTACCAAGAGAAGTTCAGAACA	GACCTCGTTGTGAGATGGACAT
1	NOTCH2	GGGTCTGAATGGCACTCATTG	TTTGATCCCAGTAATTGGTACTTGTGT
1	NOTCH2	GAAGCTGTAAGGAGAAACGGGAAT	CAATAATGTGGAGGCAACTCTTTTGTT
1	NOTCH2	CGGTTGGCCCCATTTTTCAAC	AGATTTCATTGTGCAAGTCATAGTGTCT
1	NOTCH2	TGCCAACAGTCTGAAACATCTGT	GATGAATGTTACAGCAGCCCTTG
1	NOTCH2	CAATGCAGCGACCATCGTT	CATGGTCAGTGTCAGGATGGTA
1	NOTCH2	CTAGATCCGTCCTTCTGCTACCT	AGGCTATGGCTGTGTATGTGTC
1	NOTCH2	GCAGATGCAGGTGTAGGAATCAA	CTGCCTTGGTCTTTGAGAATCCT
1	NOTCH2	CATCTCCACTCCAGCCGTT	GAGACTCAAGAGCCCTAATCAAAAGT
1	NOTCH2	gagtgaatCTCTCACGTATTTGGATCT	TGGTGGCAGAACTGATCAACTG
1	NOTCH2	CTGCATTCACATCCGCTTGG	CAGGTGACTCTCTTTTCTGTACTTTCT
1	NOTCH2	AGTTGTACAATCTCCTGGGAGGT	CTGGGCTATACTGGGAGCTACT
1	NOTCH2	ACTCATCGAGTTGCTCCTCAC	TGTTCTTCTATGACATTTCTCCCTCACT
1	NOTCH2	CTTGGGCTTCCTAAAAACAGCAAT	CCTGTGCACCCTCACCTTG
1	NOTCH2	TGCCTCCATTGACACAAGGT	AAATGTGAGACTGATGTCAATGAGTGT
1	NOTCH2	TGCCTCCATTGACACAAGGTG	CAGAAATGTGAGACTGATGTCAATGAG
1	NOTCH2	CACACTCATTGACATCAGTCTCACAT	GGAGTGCCAATGGACGGA
1	NOTCH2	ATGAGACAGGCAGGCATCC	AAGAGCTCGCTGATGTCAATGA
1	NOTCH2	ttCAAGTGACTCTTCTCATGTTCTTTACC	GCCAAGCGTCTCCTGGAT
1	NOTCH2	CATGTTGTCCTGGGCATTGG	AGAGGACTCTTCTGCTAACATCATCA
1	NOTCH2	GCACCCTGGTAGACCAAGTCT	GGGTTAACTACCTTCTCCTTCTTGAGT
1	NOTCH2	CCACCCACTACTATCTGCCCTT	CCTATTGTGACGTGCCCAATG
1	NOTCH2	GGCCTTGCAGTAAAAGGAGCTT	TCCGTTGGGTTTTCTCTTCCAG
1	NOTCH2	GAGGCTGCTATGTCACAAGAGA	GTATGTACACCCCTGCCTCTG
1	NOTCH2	GGGCCTTCCATATGATCTGATAACC	GTTCCCCAAACCCTTGTGAGA

1	NOTCH2	GACTCTTTGCAAACAGCAGCAT	TCAGAGAACATCCTTGGGATAAGGAA
1	NOTCH2	TTCCCCCAATTGACACTCTTCC	GCCAGTGTGTTCAAGACTCAGA
1	NOTCH2	TCCGTGTTCTTGAAGCAGTGG	CCTGACCTGCACTCTTCTGTTTTA
1	NOTCH2	AAGAAGTGAGTCAAGCAGTATGCAA	GGACAAAAATGAATGCCTTTCGAATCC
1	NOTCH2	ACAAGTTCCTCCATTCTGGCAT	CACCAAGTCCTGTATTTCACTCTATGAC
1	NOTCH2	GGATAAATGACCGGTATGCCCAA	GGCTTCAGTGGTATGGACTGT
1	NOTCH2	GGCAGTCATCAATGTCCTCCTC	GTCATTTATTGGCCCTGTGTTCCTA
1	NOTCH2	AGAAAAGCTTCACTTGGGTCTGG	CACCAATGACTACCTGTGTGTTTG
1	NOTCH2	GCTTCTCACCAGTAAAGGCACTA	GCTACAGTTGTCGCTGCTTG
1	NOTCH2	CTCGTTGATGTCTCCCTCACAA	ATTCTGTACCTCTTCTCTTTCCTCCA
1	NOTCH2	GTGTCAGGTAGGGATGCTCAC	GCAGGTAGCTCAGACCATTCTC
1	NOTCH2	CACTGCCTGGAGACACAATGT	ATGCCCAGCATGCACTATCTT
1	NOTCH2	GCTGGGAAAGGATGATAGGCT	TGCCCACCATGTACCAGATTC
1	NOTCH2	GGCAAACGGGCCATTTCTG	CCCATTGTGACTTTCCAGCTCAT
1	NOTCH2	AGGCTGCATTTCATGAAGGTTAGAA	CTCACACGTATGTTTCCGACAC
1	NOTCH2	TGGTTGGGCAATACTGCCTTTAG	TGAGACCCAGTACAATGAGATGTTTG
1	NOTCH2	GGGATGTAATCATTGGAGAGGATGTG	TCCCTAACCTTGCCAAGGAG
1	NOTCH2	AGCTGGAGCCAGGACCATA	CTCAGTGAGCCAGTTGCTATCC
1	NOTCH2	CTCTACACTGGAGGTGGACTCT	TCAAGTTCATCACCCCACTCTG
1	NOTCH2	CCTACTACCCTTGGCATCCTTTG	AGGCACCGTGTTGACTTCTG
1	NOTCH2	GGTCACATCTGACCAGTCAGAA	AAATGCTGCTGAGCGAACAC
1	NOTCH2	CAAGAACGAGAAACTGAAGTGTGT	CCCACTTCCCTGCTGGGATTATA
1	NOTCH2	AGCTCATCACACTGGTTGTTGA	GCCAGTATTGTGCCGACAAAG
1	NOTCH2	GTTGCAGGCCTCATCACAGA	CTCCTTATTACTCCTGCCAGTGTG
1	NOTCH2	CGGCTACCCGAGAATGGT	TGCTTCTGCCCCAGTCC
1	NOTCH2	CTACTGGCACAGCCTGACTC	GCCATTTTTATTTCCTCACTGATTTCCA
1	NRAS	CTCTACCAGAGTTAATCAACTGATGCAA	TGCCAACAAGGACAGTTGATACAA
1	NRAS	CAGAATATGGGTAAAGATGATCCGACAA	CACTGACAATCCAGCTAATCCAGAA
1	NRAS	GCCAGTTCGTGGGCTTGTT	acccagccTAATCTTGTTTTTCTTATGT
1	NRAS	GGTGGGATCATATTCATCTACAAAGTGG	ACAGGTTCTTGCTGGTGTGAA
1	NRAS	CAGAAGAGGATAGGCAGAAACTCA	AGTGATGATGGGACTCAGGGTT
1	NRAS	ACCACACATGGCAATCCCATAC	TTTGGATTGTGTCCGTTGAGCTA
1	NRAS	CAGAACACAAAGATCATCCTTTCAGAGA	CATACTGGATACAGCTGGACAAGAAG
1	NRAS	TGGTCTCTCATGGCACTGTACT	GCATTGCATTCCCTGTGGTTTT
1	NTRK1	GCGGCTGTGTCTCCTCTCTA	CCTCACAGGCATCACTGAAGTAT
1	NTRK1	CCACATCATCGAGAACCCACA	GAGAAAGGGAGGGATCGTTCAG
1	NTRK1	GCCTAGGAGCAGTAAGGGAGT	AGGACTTGCAGATGGACAAAGG

1 NTRK1	CAAGCTGGCTAAAGCTCCTTCTTA	CCAGCACGTCACGTTCTTC
1 NTRK1	ATGTCACCAGTGACCTCAACAG	TGAGGGAAGAAGAGATGAGTAGGG
1 NTRK1	GGCTTACTACAGGAGGCTCTGA	TCCGTCCACATTTGTTGAGCA
1 NTRK1	TCCTTTCTACGCTGCTCCTTG	AGTCAGGAAGAGCCAGTAGGAAA
1 NTRK1	CTGGCGGCTGGGTCTTTA	CCAGCTGTGCCAGCCAA
1 NTRK1	CCTGCTGGCTTGGCTGA	TCACAGCTCAGTCAGGTTCTCT
1 NTRK1	TGGATAGCCTCCACCACCT	GTCCTGCCTGACCAGCAAA
1 NTRK1	GGGACTGATCCTCCTGCAC	GCCCATTAGCAGCCCAAGTC
1 NTRK1	GGGTTACTGGAGGCTACAGTG	CTGCACAAGCTTCTACTTCCTATCT
1 NTRK1	CAAGACAGTCCCCGCTACA	AGGAGGTTGTGGCACTCAG
1 NTRK1	GCCTTTGGGAAGGTCTTCCTTG	CACTCTGTCCTGATGTGATGGA
1 NTRK1	TCCAGGTCATTGAGGAGGGT	CCACATCCACCGAGGCATT
1 NTRK1	CACGCTGAAGGTCCAGGT	GCCAGCGAAGGTCTTCTCA
1 NTRK1	GACCTCCTGCTGTTGCTCTT	CTCAGTGAAGATGAAGCTGGTCTC
1 NTRK1	TCAATGGCTCCGTGCTCAAT	CGTGTAGTTGCCGTTGTTGAC
1 NTRK1	GGGTGTCTGCGCCTCAA	GGGCAGGGTTCAGGATGG
1 NTRK1	CCTGCCGCTTCCATCCA	GTCCCCGTGCCGCATAT
1 NTRK1	CCCTGCTCATGGTCTTTGAGT	GGGATGTCTATAGGGAAGGGAAGA
1 NTRK1	CCCCTCGTCCCATGAAGGA	CCTCCACCTTGGGCTACTCA
1 NTRK1	tTTTCAGGCCGCCCTCA	ATGATCCAAATGGCTGGTGAGATT
1 NTRK1	CCTATCCCCTCTCCTTTTCTTGTT	GGTCCCGGTGCACAAAATG
1 NTRK1	GTGTACCTGGCGGGTCTG	ACGGTAATAGTCGGTGCTGTAGAT
1 NTRK1	TGATTTTGGCATGAGCAGGGAT	ACCAGAGCCAGTGAATCTCAGA
1 NTRK1	GGTGGGCATGGGAACTCAA	ACAGTCCCCGTTTCCCTCA
1 NTRK1	CCTCTTATCCCCTGTGATCCCT	CATGACACTGCAGCTTCTGTTC
1 NTRK1	GGAACCCTCTGCACTGTTCTT	CCTTACACCACCTCCCTCAC
1 NTRK1	CCCAGGCAATCGACTGCAT	CGTGCACATCCTTGATGCT
1 NTRK1	ACCAGAGGTCTACGCCATCAT	CAGTATTCCGGCTAACCACTCC
1 NTRK1	CAGGCGCCCCTGGAATT	GGGCTGCTTGCCGTAGG
1 NTRK1	TGGTGCTCTGGGAGATCTTCA	GAACCGAGTTCCCTGAAAAAGG
1 NTRK1	TCAGAGTGAGGTCGGGTCA	ACACACACACTTGGTGCTCTT
1 PARP1	AGGAAGGCCTGACCCTGTTA	GGCTTTCTTTTGCTCCTCCAG
1 PARP1	AGCGCTTCGGGTGAATTCATA	CTCCTTAACAAGCTTCCCCTCAG
1 PARP1	AAGCAGACAGTGTAAGGGCATT	GAACAGATGCCGTCCAAGGA
1 PARP1	CATGAAGTGCTCAATGGCATCC	ACATGTGTATCTGACACCCTCCTT
1 PARP1	GCCAGGTTTTTCTCCCAACAGAT	AGCTGGTCTTCAAGAGCGATG
1 PARP1	GACGTCCCCAGTGCAGTAATAG	GCATTTGCCCTCTGACATGTT
1 PARP1	GGACGGCTGCAAGGTAGTC	TCTCAAATCGCTTTTACACCCTGAT
1 PARP1	GGTTCCGTGGACAACAACCT	GCAGAGCCTGTTGAAGTTGTG
1 PARP1	GGCTTCTTCATCCCAAAGTCGT	CTGCTCCCTCAGTCTTGCATT
1 PARP1	AGCCCCTGACTTCCCTCTT	GTAAAGGAAGCCAACATCCGAGT
1 PARP1	AACTCCTGAAGGCTCTTGGTG	AATTTGAACAGAGGAGGTGGAAAAG AT

1 PARP1	GCTTCCTTTACTTCCTCCATCTTCTTATT	GAAGCAAAGGAGAGCTGTGAATG
1 PARP1	TTCTGCTGAACAAATGAGTCACTTCT	CACACACAATGCGTATGACTTGG
1 PARP1	GAAGAAGGGATTCTTACATCGATGACTT	TCCTGGGTCTCTTTTGTGCTAATC
1 PARP1	GGACAGGTACAAGAAGCTGACA	TGATGTGGAAAGTATGAAGAAAGCCA
1 PARP1	CGCAATAACCTCATACTCCACCA	TGGGTCTCTTAGCAATGTGCTTTT
1 PARP1	TGTGCTCACACAGCATACTCAA	CATGTCACTTGTGACTTCTGTTTTTCAG
1 PARP1	CTTACGTTGAGCTAGCACCCTAA	CCAAGTCCAACAGAAGTACGTG
1 PARP1	CTTTTCTATCTTCTCCATACACCCCTTG	AGATGAGCATTGCTGTCTGTTTGAT
1 PARP1	GGCTGTAGCAACAGCTTTGGA	GTTTCGAGAGCCCAGTGTGAA
1 PARP1	CCAGGTCTCACCCCCTAAGT	CGAAATCCCCTGTCTCCTCAG
1 PARP1	TCACATGCGTGTCCCACTTAA	GCCACTGGGTCTTGTCCATAG
1 PARP1	GCCTTCCCGGACACAGTTAA	CGAGTCGAGTACGCCAAGAG
1 PARP1	GATGCTCTCGCTGCATTTCTTG	CAGCGTGTTTCTAGGTCGTG
1 PARP1	GCATCTCCCTGGCTTACTGAC	GGATAAAGAAGCCCTGAAGAAGCA
1 PARP1	CACCCACCCTTCACTCTTGAC	CATGATTGACCGCTGGTACCAT
1 PARP1	CCTGTTCTTGACAAAGCAGCCT	GCTGCCCGTATCATGTTCAC
1 PARP1	GCTCAAAGTTCTTTATGGAGACACCT	CAGCTTCATAACCGAAGATTGCT
1 PARP1	CCAGCAAAGTTGGTGGTCCT	TGTCTTTCAGTCAACCAGAGTAACAAA
1 PARP1	CAAAGAGAGACCCTTGACGGATA	TGCTGTGAACTCCTCTGCTTC
1 PARP1	GTCCCAAATGCTGTACCTGCT	GGAATTCCGAGAAATCTCTTACCTCAA
1 PARP1	GGAGGCGCTGGTTTCTGG	TCAAGGGAGAGCTGGCTTCT
1 PARP1	CCATGCTGCCCCAGTATGTA	ACTTCTCCTGCTTCTGGAAGGT
1 PARP1	CCCATCCACCTCAACGTCAG	CAAGGTCAAGGTCTAGTGGGTCTA
1 PARP1	GCATTGTCCCTGTTGCACAAA	TCCCGGAACAAGGATGAAGTG
1 PARP1	CCGAGTTTCTCAATCATGGCCTT	GCTGGCCTTGCTGAATGATTCT
1 PARP1	GCTGGCAGTCCTGTTTGCTTA	CCTTCTACCTTCCCCTTACTTTTTAGTT
1 PARP1	AATCCAGACAGCAGAATGTCGAA	TCTCTCATATCCTGTGTCTCCTTCAG
1 PARP1	TCAGTCCCAGAGCCAGGTTTA	CTCCCTTTTCCGACCTTCCAG
1 PARP1	TGGGCCTAACGGGTTTCAC	CCTGGTGGACATCGTTAAAGGAA
1 PARP1	CTGCAGCTTGTAGTAGGAGTTGG	CCCTTCTCCGTGAGAGTGCTA
1 PAX7	CTCTTTTGCCTTTGAATTTCTGAGGTT	CAGGGAAGTTCCCTACCTTTGTC
1 PAX7	GTCTACATGTGTTGCAGCTCTCT	TGGGTGGGAAGCCTCCT
1 PAX7	CGGCGTTCAACCACCTTCT	GGCACCACTGAGATGGCA
1 PAX7	CCACTTGCTTAGGACCTCTCTT	TCAGAGTAGCTGGAGAAGCTGT
1 PAX7	ACCAGCTCTGCCTACGGA	GCTGGGCTGAGACCACCTA
1 PAX7	CTACTCTCCCACCTCCACCT	GGCTCAGCTGCCTTCTCA
1 PAX7	CTGGCACTAATGGCCTTTTCC	GTCTCCTGGCTTGATGGAGTC
1 PAX7	TCGGCCACCTCCATCTCA	GGGTCCACGCTGTAGCC
1 PAX7	CCTACTGCCCACCCACCTA	gtagCTCTCCATAAACAAGCAGGA

1	PAX7	CAGTGTCTGCTCTCCATCCTC	GGTGGGCCATCTCCACTATC
1	PAX7	CCTAACCACATCCGCCACAA	GGACCCGGTCTCCTGGTAG
1	PAX7	GCGTCTCCAAGATTCTTTGCC	GGAGCCCCCGACTATAGGAA
1	PAX7	atgggtgTAGATAGAGTGAATGGAAGA	TTCATGCGACGCTGGGT
1	PAX7	CTGTTGATTATCTGGCCAAAAATGTGA	GTTAGGGTTGGGCTGGGAATT
1	PAX7	CGGCACCCTTCCATCTCTCTAA	CCGTGAATGTGGTCCGACT
1	PAX7	GAACCTGACCTCCCACTGAA	CTCCTCGCGGGTGTATATGTC
1	PAX7	TTTGAGAGGACCCACTACCCA	GCCATCCCAAACTTCTGAATCTCT
1	PAX7	GGAAGCGATTTTTGCCGACTT	CCAGCCTGGGCGTTCTTAC
1	PAX7	GATGAATGGATATCTGTAAGGAAGCAG	CGCGGCTAATCGAACTCACTA
1	PAX7	CACAGCATCGACGGCATC	GAGAGTTTCTCCGCTGGAGTT
1	PBX1	GCATTATCGGCAGTTTGATCTTGAGA	GCCAAAGAAAATGCTAAAATGTTGGAAT
1	PBX1	CAGAGGACTGACTTCTCTCCCT	TCAGCCGGGATGCGATTG
1	PBX1	CCCCTTCCCTGTTTATCCTGAA	GCATGGGAATGCATCAGCCT
1	PBX1	CCTTTGGAGATGGACGAGCA	GGTCTGTGATGGTCATAATTTGCTGTA
1	PBX1	GGAAGCAGGACATTGGAGACATTT	CATTGTTTCCCCCTGCAAGAG
1	PBX1	CTGTGATTCAGGTGCCTCACT	GACATATTGGCTGGGTCCCTAC
1	PBX1	TCTTCTGCAATGTTTCTGAAGGATGA	TTGGTAGCAGTGACAGCTGTTT
1	PBX1	AGGAAGCCAATATTTATGCTGCCA	CCCAATGTAGGAACAGCCAGTATAAAA
1	PBX1	GTGGCCTGCCTGATGATGAT	GAGTCCATCACTGTATCCTCCTG
1	PBX1	CTTCGCCATGTTATCAGCCAGA	CCCTATGGCTTTGAAATAATTGGTAGTG
1	PBX1	CAGCTTATGTAGCCAAAGAACACAA	CTTCCGCTAACAGCATGTTGTC
1	PBX1	CACAGACCCCCAGCTGATG	CTGTGAGAGTTTGGCTCTGTAATCT
1	PBX1	TCAGACAACTCAGTGGAGCATTC	GCTTCCTCCACCCTTTCCTAAA
1	PBX1	CAACCAGCATTTCACTTAGTCTTCTCTAT	CATCCTGCTGCTCTCAGTGTT
1	PBX1	CTTGATGCGTGTTGAGGCTTG	CTGAACTTGCGGTGGATGATG
1	PBX1	CAAAGGAGATTGAGCGGATGGT	CAGATCAGGGACTCCCAGAGA
1	PBX1	GGTGTGAGCCTTTTTGTTATTATTTCCT	TGGCTAACTCCTCTTTGGCTTC
1	PBX1	GCAACCCTTACCCCAGTGAG	TGTCCAGTAAAGCAAGACCTCTAAGA
1	PDE4DIP	CCCACACCCTGTGCTCTTA	CTGTAACACCTGCTTCTCTTCCC
1	PDE4DIP	CGGACCAATCCATGGGTCATA	AGAAACCCCCTCAGGTACAGA
1	PDE4DIP	CACTCACATGACTTACCTTCTCAAAGT	ACTAAAGATCGAGGCCCTCCA
1	PDE4DIP	CAGACTATAAATGCACTACATGACACTTG	AGGATGCTGCAGGACCTTCTA
1	PDE4DIP	CTTCTCCAGATCTGTGACTGCAC	GGAATGTGCCAGTTAGAATGATCCT
1	PDE4DIP	CATGTTCCAGCACTTGTTTATTTCGAT	CCTCCAGAAGCTTACAGTGTCC
1	PDE4DIP	GAAATATTAATGCATGGAGCCTGGGATA	AGGAGCTAAAGGCTCAAATTGAGG
1	PDE4DIP	GACACTGAGGAGAATCCTGCTT	GGGACAGTAACAGGTGTCTTCA

1 PDE4DIP	CCTCAGGTGGAGCATTTCTTCC	GCCTAACTTCTTCAAACCATGATTTTCT
1 PDE4DIP	CTCCACTTCAACCTTCAGCTCAA	TGAGAGGCTGTGGACCAAGG
1 PDE4DIP	GCCAGCTAGGAAGAGCCTAGAAT	CCTCAAGCTGCGCATCTAC
1 PDE4DIP	CATGCGCTCCTCCAGGAA	AATCGGGCACGCTCCTC
1 PDE4DIP	GGCGACATGGCCCCTATTG	CGGGTTACACCACTTGTGTCTG
1 PDE4DIP	CAAGAAAGGGCACTGGCATC	GTGCTAGGCAGCAAAGGTATTC
1 PDE4DIP	TGCTGCTCCGAAGCTCAT	TTCTATGCCTCACTCACAAAGGAAG
1 PDE4DIP	tgaaGCTCTGCCTAAAAGTCCCTA	CTGGAGGAATCCATCTGCATCAA
1 PDE4DIP	CCTTCCACGAGCAGTAGAGGT	CTCTCTTCATGTCCTGGTGGTT
1 PDE4DIP	CTACCCTACGCCAAGCATGA	CCAGAGAGTATCCCAGCTGGAG
1 PDE4DIP	AGCCAGCTTCTCTTCTAGTCCAT	GGGTCTTCACCTCATAGTGTCC
1 PDE4DIP	CTGAAGCCCTGGAGAGTAGAAAG	CCCTCTCAGTTACAAGTGATTATTCGT
1 PDE4DIP	CTCTCAGCTTCCAGGGTCTT	CGGGTATATGGAAAGTCAGAAAACATCTT
1 PDE4DIP	CCTTCAGATCTTTGATGTCCTTTCGTA	CTGTGCTCTGAGCAGGGA
1 PDE4DIP	GAGCCCAGTTTTCATTGAAATCATGT	AGCAGCTACAAGAAGAATTGCAGAA
1 PDE4DIP	GCACGAAGCTGTTGGCTCTTA	GCCTCTGTATCTAATAACCATGTGACAAT
1 PDE4DIP	GTGCTTCTGGATGTACTGACCA	ACTGTCCTTACTTTTCCTTTGCTTCA
1 PDE4DIP	CAGAGAAAGAGGAGATGAAGCTTCT	CCATCATCAACCAGTGCATCTCA
1 PDE4DIP	TGCTGTTGGATTCGGCCTTAG	CTGGGTAAACATAAGGCCAGGA
1 PDE4DIP	TCCTGCCTGGGTAGGAAAAGA	GGCAAGTGGGATGTGATGAGG
1 PDE4DIP	TCCCCAGATACACTCCCTTTCT	TGGAAGCCGACTCTTCCTACT
1 PDE4DIP	GTTCTATGGTGCCCCTTGGAG	CTGACACCCCATTTGCTTCTTACTA
1 PDE4DIP	CCAGCTCCACCAGGATACAT	CAGAAGTCCTTAAAACAGCAGGAAAG
1 PDE4DIP	TGACTCAAGGGTTCAACACAAACT	CAATTTGTAGAGGCTGCAGCAC
1 PDE4DIP	CTCTTTCTGCTGTTCACTCTCGT	GCTTTATTCTGCAGCCAACTTGAAA
1 PDE4DIP	GAGCACAAGGAGTACACAGTATTCA	CACTTGGCAGAACTCCAGGAA
1 PDE4DIP	GCCTCGGTCCCAATCTCTT	GGACCAGCCTAATTTCTTATCGTCTC
1 PDE4DIP	CCACCCTCTGGAGCTTCTGTA	CTCCTTGTGATTGTGATTTCTACTCTCTT
1 PDE4DIP	TGGCCTCTGTTTGCTGGATTTT	TTTCTTCCTTGCCGTTTCTTCCTA
1 PDE4DIP	GGGAAGCTGCACGCTGAT	TGAAGACCCCTCCCAAGCTA
1 PDE4DIP	CCATCAGTAGCATCACCCTCT	TCCTTTTCTTGTTCCCTACCTCTTTTT
1 PDE4DIP	CAACAGAACTCTGTAGCTTCCCA	TCCAGTCAGAGCTACAAATCTACGA
1 PDE4DIP	TCTTGGAATTGCCATAAAGTGCCT	GCTCTGGTGTGGCTGAGTA
1 PDE4DIP	ACAGTCCTCCCAATCTGAAGAGT	ACCAAATTTAGCCGTTGGCAGA
1 PDE4DIP	CTGCTGAATGATACTCTCTTGTTCCT	GACTGATTATTGCTCTCTTTATGGCTGTA
1 PDE4DIP	CCAGGAAATGAAATAGAAACAGGACCTT	AGAGGCTCACCAGCAAACTC

1 PDE4DIP	TCTTTTGCATGCCGGGTGA	CCATAAGTTAGCTTCCACTCTTTCCATA
1 PDE4DIP	CCAGTAACTTACCTGTTGTCTTCGG	TCCTGAAGACATACCAGCTATGGAA
1 PDE4DIP	CAACTTTTTCCCGAAGAAGTAAGACTT	TCAAGACATTTGTTCCCGTGCTA
1 PDE4DIP	CTTGCCAATATCCCTTCCTCTTGTaa	CAGCAGCTTTTGGAAGACTTGAG
1 PDE4DIP	CAAGACCTCTTGCTGCTTCTC	AAATCAATCCTGTGTTCCTCTCACC
1 PDE4DIP	GCTGCCAATCGCAAGCA	ACTGGCCCTCTCCTCCTT
1 PDE4DIP	GAGACCACTGGTGTCTCACA	CCTAGGCTTTCATTTTCACTCCATACC
1 PDE4DIP	GAGGAAGGCTAGCCAGCTT	GGTGATGTCTGGTCAGGGAAT
1 PDE4DIP	GATTAGTGTGGCCAGGGAAGAAA	CCTCAGCTCTGCAATGAGAACAG
1 PDE4DIP	CTCTTCCTCCATGCTTTCTCCAT	GACTATGAAGCCATTTGTAAGGTACCTC
1 PDE4DIP	TCGATTGTCTTCCCTGAGGACT	TGAGAGATAACGAATGGGAAACTTTTGA
1 PDE4DIP	TGCAAACAAGGTTTTCCATCCTG	TTACGCAGTCGGCTAGAAGAAG
1 PDE4DIP	GGAGATACTTCTGGCCACCTTTC	GTTTGAGTGCTGGGTGGAGAAT
1 PDE4DIP	CGAAAGCAGAACCTGGAGATTGAG	CCATTGAGTGGTCACCCAGTA
1 PDE4DIP	CGCTCCAAGCTTCTTCCAAGA	GGCTAATCCACTTTCCTTCCATGT
1 PDE4DIP	TGTGTGGCTCCTGGATCTGAT	GTATCGGAAGAATAACGATGACTCTGG
1 PDE4DIP	GGACATGTCAACCGTCCCATT	CGACACAGTTATTGCCCGGAT
1 PDE4DIP	CGCTCAATAGAAAGCGCTTCA	TTCCACACGGCGTCCAA
1 PDE4DIP	GGCGACCTCTCTCTCCCTAA	TGAGACTAAGGAAAGAGCCTGGAAA
1 PDE4DIP	AGAGTCTTGGCCAAACCTATAATACAGA	CTTCATTGACTGCCAAAGAGGATG
1 PDE4DIP	CTAATGTGGATCTGGGTATGCTGA	GTGATCATAGTTCCTGCATCTTCCTAAG
1 PDE4DIP	ACCATGGTGGAGCTATAAGCC	GACATAGTCAGCGAGTACACACA
1 PDE4DIP	TGGAGGCTACCTGAGTGACTG	CAGCCATGCCTTGTCTGACT
1 PDE4DIP	AGAGGTGCTGCTGGGAGA	TGACCTTGGTTTTACCCGTTGT
1 PDE4DIP	CCTGATCACTTCTCATCTGAAGCA	GAGCACAACTGAGCATCTGAAGA
1 PDE4DIP	AACTGTTCCATGCTCTCCTTCTG	CTTTAGGCCTTCTGCCTGGT
1 PDE4DIP	GCCGCAGAACTTCCTGGT	GAGCGGTGCTGGCAAAG
1 PDE4DIP	GCTGGGAAGTTCTGGTTAATGGA	TGGCAGAGGTACGAGCTCT
1 PDE4DIP	CGCAGACAATTGTTCCCCTGAAT	AATGTTACCAGACTCCTTCTCTCTCA
1 PDE4DIP	AAAACAGCCTCTCTCTTACCTCTCT	GCGTATTGCTTCCTCTCTCTTCTTTA
1 PDE4DIP	CTTCTCCAGGCTAAGACATGCT	GCAAGATGGTACAATTCAGAACCTCA
1 PDE4DIP	TTCCCTGCTTTTCAGAGTTTCCT	ATTTGAGGCTAAAAGACATCATTGACCTA
1 PDE4DIP	CCAACTGAAGTCTCTCCTGTCCTA	CCCTCTCTCATTTTCAGACTGAGG
1 PDE4DIP	CATAACTCTGTATTTCTCCAGCTCGT	ACCCTGGGCCTCAGTCA
1 PDE4DIP	GGTGGACCCTGGTAGGCTAAA	GAGTAAAGTCCACAGGGTTCAGAAG

1	PDE4DIP	CTACAAGATCTGCTAACGGATGGT	GCTGGTGGTTTCTACTCTGTGG
1	PDE4DIP	TTTTCACTCAATCTAAGCCCACACTT	TGAAAGAATAAACACAGAACTGGTTGGT
1	PDE4DIP	CATTCCCCTCCTCTTGGTGTTG	CCACAATATCATCAACCTCCTCAAAGAA
1	PDE4DIP	CTTCCTTGCTACTCAGCACAAGT	GGTGATGGTTGAGACTGTGGTAA
1	PDE4DIP	CATGCTTCAGGCTCAGGATACA	CCAGCTTCGTCTTGCTGTGA
1	PDE4DIP	TCTCTCTAAGTCATGATCTCGCTCTC	AGAGCTTTAGGAGTCTTCCTTTCCTT
1	PDE4DIP	CTACTCTCACTCAGACCCTCTTTG	GAACACCATGCTGAGCCTTTG
1	PDE4DIP	ATCTGTATGTTCCTGGTCAGAGACT	GGCAGACATTGTTTCCTCTTTTCAC
1	PDE4DIP	GGAAGTCCCAGACCCTGAC	GGAATCACTTTTCTTTTCTGGACTTGTT
1	PDE4DIP	TCAGCTCCGCATTCTCAAGG	AGTTAATTGCGGGCTGAGATGTT
1	PDE4DIP	TCCAGAGCAACAGCTTTATCATGTATT	GAAACTATTTTTGGTCTCAGGAATTTCGG
1	PDE4DIP	CTGCGCTTCTTGCATGGTT	TTTGGTCACCACGTAGCTCTC
1	PDE4DIP	TTTTTAGCCATGAGTTCAAGTTCCTCT	GAGGCAGCAGATACCACATTATGTTAA
1	PDE4DIP	TGTCCATCGTCTTGCAAGTAAAAATG	AATCCACCCTTTGGTTTGTATTGTATCT
1	PDE4DIP	GAGCTGGCCATGCTCTCA	ACGCGAACCCCTCAGTC
1	PDE4DIP	CCATGCTCTCACCTCATCCA	GGGCTCACTACACGCCTT
1	PDE4DIP	GTCCCCGGACCGGTAAG	CAGGAACCGTAGCTAGGACTG
1	PDE4DIP	GCCAGAGGCCAGCTAGCTATA	CCTTAACAGGTCTTTGAAACCCCTT
1	PDE4DIP	AGGGATAACTCACAGGTGTCTTGT	GGTCTTGATTATAAGCCCATCCAGAG
1	PDE4DIP	ATTTCACTGGAATCGGAAGCCT	GAGACTGAGAGAAGTGCAAAGGA
1	PDE4DIP	TGAACAACAGTCACACTTTCCAAGT	ACTTAGCAAGCACAAAGGTACCC
1	PDE4DIP	GAACTATGATCACAATGACAGTACTGAG	CCCACTGGCTGTCTTGGA
1	PDE4DIP	TCTGTTTTCCAAGACAGCCAGT	GCAAGCCTTAATGGAAAGAAATTCAGAA
1	PDE4DIP	GGTAACTTCAGCTTGTTGGTTAGAGAT	GTGATTTCCTCTTACTAGGCTGCT
1	PDE4DIP	AGGTTGTTTGCATTAATCACTTTCCATC	GAATTCCCCAGCTCTGGTCTT
1	PDE4DIP	CCTTTAGTTGCCATATTTACCATTTGCT	GGGCTCCTGAAAACTGTGGT
1	PDE4DIP	CAAGGCCCACAGCATCCTTA	GGTGTCTTCTCCTCGATCCCT
1	PIK3C2B	CACTTCCTGCTGAGATGGCAA	CCAGCGCAAGCTTAAAGACATC
1	PIK3C2B	CCAGTACAAGGACTCTTTCTGCAT	TCCTCACATCCCTGATCTCATCC
1	PIK3C2B	GCTACCTCCCACCCATTCACT	TCCCCCTACCTTACCCTTCAC
1	PIK3C2B	GCAGCCTCACCCCACTAA	AGTTCAGCATCCGCTACCAG
1	PIK3C2B	CAGTAAGGCTGCCAGCAGATA	CCTGTACCCTTGCCTTTGATCC
1	PIK3C2B	CTCAGATCACTGATGGGCAGT	GCTTGCCTGCCTGACATCTAT
1	PIK3C2B	GGTCCACTGCTTCAGGAGAAC	GGCTCACTGATGCTGACAAGAA
1	PIK3C2B	GGGTTAACAAGGAGCCTGGTTT	CCGAAGAGCTCACTTCTCCAAG
1	PIK3C2B	TCACCTCCGAGTGGCAGTAATAT	CAGCACTCCCTTGAGGGTAAG

1	PIK3C2B	AGACGATGAGGTGGAAGAGGTA	CCTGACCTGCTCCCTTTCTTTTATAG
1	PIK3C2B	GGGTAGGTCCTTCTGCCAAC	GACTGGCTGCAGAAACACAAC
1	PIK3C2B	CCCTACCTTCTCATACTCGTCCTC	GGAACGTGACCCCCATTTCT
1	PIK3C2B	CAGTGCTGATTCGCTCAGG	CCTCTCTTCTTCTTTCTGCTCCTTCTA
1	PIK3C2B	ACTCTGAGAGCCCCAAACCTA	TGTACTCCTCCCCTTCTTTCCAG
1	PIK3C2B	AGACTAGTCCCTGTTTCTGAAGGA	AAGACTATGCCCCCTCAGGT
1	PIK3C2B	AAAACTGGAGGGAGGACCAATC	GCCTCAACTTGCAGCTAGGTT
1	PIK3C2B	TCGCATTCTTTCGGTTGCCATA	CGACCTATGATGCGGAGATGTTG
1	PIK3C2B	CCAGAGGTGTCTTTGCTGAAGTC	GGGTCTGTGGACTATGATGGTA
1	PIK3C2B	AGTTGAGCCTAGTAATTGCATCATTG A	TTTCTCTTTGGTCGAACAATTGCC
1	PIK3C2B	GGATCCGATGCTCTAGCAGTTT	CCGAGCTTCTATCTGGGATACC
1	PIK3C2B	ACCCCTTTCTGGGAGGCA	GGCTCCCTGTCTGGAGACTAT
1	PIK3C2B	GTAGTTCAGAGGAGTTGGCAGT	GCCCTGTTTCTGATTCTGTTCCA
1	PIK3C2B	TCACCCCTACTCCCAACTGA	GTCACTTTCTCCCTCCATCCA
1	PIK3C2B	GGAAAAAGTCAGGATCCCAGCAA	CCTCCCGAACACACACTCTC
1	PIK3C2B	CTGATTCGGCCAGAGCTCTT	TCCTCTTTTCTTATCCTCCTGCCA
1	PIK3C2B	CCCTCCCACCTGATGTGTTCTA	GTTTGACATTGACATTCGGCTACAG
1	PIK3C2B	CAGCCCCTGGATTCGTACTC	ATGACCTCTCCTGTGGTTTTTCC
1	PIK3C2B	CACAACCTTCTGCTCCATCAG	CCCAGGATGCTAATGGTCTTGT
1	PIK3C2B	TTCCAAGGCCAAGAAGCTGT	TGCTGTGTGCCACTCTCTATG
1	PIK3C2B	CTGCTTATTGGCCTCTGAGGA	GTGGCCCCTCCTAATTTGACTT
1	PIK3C2B	AATCCCACCCCATTCTTTAACTCTTAC	ACTGAGTCGTCTGCTCTTCTCTTAA
1	PIK3C2B	GCTTGTGTCCCAGCTCTGAAC	ATGCTGAAGACCACTGGTCAC
1	PIK3C2B	CGGCCAAAATCAATGTGGAACAT	CTTTATGTCCTCTCCTCCCATCTG
1	PIK3C2B	CCAAGTCCTGTTCCTGACTCCTA	GAGGGAGGAGCTAAACGGTTAC
1	PIK3C2B	GCGTGGATCAAGTGCCAGAT	CATTTTCTTTCCCCATCTTCACCTC
1	PIK3C2B	ACCTCCCATCCCGTATCCTTT	GACCTTTGAGGAGTTCCAGGAATTAC
1	PIK3C2B	AGAGCAGCCGCAACTTATTGT	GTACACCCTAAGATCTGGACCTTTG
1	PIK3C2B	AGGGAAGGGCTAAAGCCTCA	TTCCCACTCTCCCTCCTTCTTAG
1	PIK3C2B	GCCCTACCCCACCATTTTCC	GACCCCTATGTGAAAATTTACCTCCTT
1	PIK3C2B	CCTGACAGTATCCCTCTCTTCCTTT	GGTGGTCCAGTCCGTCAAG
1	PIK3C2B	CTTAGTGGTTTTCTGAGGGTCAGG	AATGGTGGTGAAGAGACAGATGTTAA AA
1	PIK3C2B	GGCCACAAGATGAGGTGGAAAT	GTCAACCTGAAGGTGACTGTGTT
1	PIK3C2B	CTGGTTGAGAGCACTGGTGAT	GGTTGGAGACCAAGAGCATGAG
1	PIK3C2B	GCCTCTTGAAGCCTGTCACAC	TCTAAAGGCACAGTGAGGGTTTTT
1	PIK3C2B	ACACGTGTCTACCTCCCACTT	GAAGCTTCATATTCTCTTTCTCCCTTT CT
1	PIK3C2B	AGAAATCCTCTGCGGCAAGAC	GATTTTGTTGACCTTTGCTGCCA
1	PIK3C2B	TTGCGAATGAGGTTGTAGGCT	CCATGTTGACCTTCTACCTGTATGTTC

1	PIK3C2B	CATCTGATGGGATCCCTGCAC	GCCCCTCTCTGAATAGGTCACTT
1	PIK3C2B	CCCTTCACAAGGAGGAGTCTCA	GAACATCCGCCTGCGAGA
1	PIK3C2B	GTCTTCTCCTGAGCCAGGTC	ACTTCCCTCACCCATCTCTATGT
1	PIK3C2B	CAATGTCACACAATGCCACACAT	CTTCACTGTCTATGCCACCCA
1	PIK3C2B	TCACCTGGTAGCCCAGATGAT	GGTGGAGCTGTACTGCAACA
1	PIK3C2B	CCCATCTGAACCATCAAAAATGTAGAG	CTGTTACGCGGTCTCTCTGG
1	PIK3C2B	AGTGAGTTGTAGTTAAGGGTAGGATCAG	CCCTCTCTCATCAGCTGGGAT
1	PIK3C2B	GTCTGGAAGTCTGCGTTGAATG	CTTGTGGAGGCCTCATGACTTT
1	PIK3C2B	CTGTAAAAGTCTACCCCAGGCT	GCATCAGCCGCAAAGAGCTA
1	PIK3C2B	CATACTCCATCTGCAGGGCTTC	GAACTGACGTTGGCAGGAAGTA
1	PIK3C2B	CAGCCGAACTCCGACACATA	CCTCCCTCTTCTTTTTCCCAACAG
1	PIK3C2B	AGACTGTGGTGGTTACCCATATGA	GTGAAGCTGTCCATCTCCTACAAA
1	PIK3C2B	GCATCACCATGATGAAGAGTTTATTGTT	CTTGACCCTTCATGGTTTCATTTGTC
1	PIK3C2B	GTCCACCAGGCTTGAGTAAAGTA	CGTCACTATCTTTTCACCATGGCA
1	PIK3CD	CCCTGAGCGTCTGGGAATC	GTGCATCAGCTCCTTGGTCT
1	PIK3CD	GTCAAGCTGAGCTCTCAGAAGA	GTGGGAGGAACCCTTGTGG
1	PIK3CD	ACCGCGTGAAGAAGCTCATC	GGTCGCACAAGGAGTCAAACT
1	PIK3CD	GAAGAAGCTCATCAACTCACAGATCA	CGAAAGTCGTTCACTTCTGGGT
1	PIK3CD	CCTCCACGAGTTTGACTCCTT	GGGAAACTGTACTGCAGCCA
1	PIK3CD	TGTGCCAATTCTGCGAGGAG	CCTCGCTGCCCTCAAACTTAA
1	PIK3CD	CGGGCCCTTCTGGTCAAC	GACACCTGGCAAACACCATC
1	PIK3CD	CGCTGAGTGCAGCCGTT	AGGAGTGAGGGCTCTGGTT
1	PIK3CD	GCAGAGGACTGACCTCCCT	ACCTGCAGCGTGTAGTCTTC
1	PIK3CD	AGACCCACCTGAGGACATTCA	CTCACCTCCGAGCTGGAC
1	PIK3CD	GGAAGAAGGCCACAGTGTTC	AGAGTGCAGAGGGCCTCA
1	PIK3CD	CAACGAGATGCTGTGCAAGA	TTCTCGATCACGGCGTACAG
1	PIK3CD	TGGCCCGTCTCTGCTTTG	CGGGAGGAGAGTGGTGAGT
1	PIK3CD	AGCAGAGAACCTACCAGAAACTCA	ACACCAAGGGACGATGTGG
1	PIK3CD	GAGCTCCCTCCTGTCCTGA	TCAGCTCGCAGTCCAGGTA
1	PIK3CD	GCAGGTGCTCAAGTACGAGTC	GGCTGGGATTCCCACAGAAC
1	PIK3CD	CTCACCTGCCCTGTCCTTC	ATCTCTGGGACCCTCACACT
1	PIK3CD	CCATTTGCCCGTCCCTCTTC	GCCATGTTGCTCTTGTTGAGTT
1	PIK3CD	TTCAGACACCATCGCCAACAT	CCACTCCACACCTCTTGGAAAG
1	PIK3CD	GGATTCTCTCCTGTCTGACACCTT	GTAGGTGAGGATGAATGGGACAC
1	PIK3CD	GGCTGACCGGTGACTGT	TCTTGCTGCTCCGCTGT
1	PIK3CD	TGTGTTCACCTGCATCAACCA	TGAGGAGGCTGATCTGTGAGTT
1	PIK3CD	AATTTCAAGACCAAGTTTGGAATCAACC	GGGCTCAGGCACTCTCA
1	PIK3CD	GTGTCCTGGCCTCGCTA	CAGTAGGCCTCCAGGATGAG
1	PIK3CD	GGGTCCTGGGAATCCTGGT	CGAGGATGGAGGAGGAATGGA
1	PIK3CD	TCACCTTCCAGCTCCGAGAT	GGATTCCCAGACGCTCAGG
1	PIK3CD	TGACCCCTCACCTGACCAT	CGGAAGGCGCCATCTCAC
1	PIK3CD	CACTTCCTCTGTCCCCTACCT	TCCTGCCCGGCACATAC
1	PIK3CD	CCCCCAGATGCTCTACCTG	AGCTGCCCTCTGGAGAAGT
1	PIK3CD	atgctttTTAATCTTCCCCACCCA	TGCCCACTCTCTCGGATCAT

1 PIK3CD	TGCTGGGCATTGGCGAT	GCCACAGAGCTGTGTGCTA
1 PIK3CD	GGGAGACAAGCTGCACTTTGA	CTCTCCCACCTATCCCAGGAG
1 PIK3CD	GAGTGTGAGGGTCCCAGAGA	CTCGGGCAGGCAGATGAG
1 PIK3CD	GCAGTAACCCCAACACGGATAG	TCTGAAGAGCTCCAGGAGCA
1 PIK3CD	GTGGAACCAGAGCCCTCA	GGGAAGTGCTCCTGGACTT
1 PIK3CD	GAGAAGGACCTGGTGTGGAA	gtagCCAGAGGCTGTGGTC
1 PIK3CD	AGCTGAGGTAACTCATTTTGCCATT	ACCACAACGCTCTGATTCTCC
1 PIK3CD	CCATGGAATTCTGGACCAAGGA	CAAGGTCTGAGGACCGGATG
1 PIK3CD	TGGACACCGCTGTGATTTGT	CAGTTCACTTTGGTTTTCCAGCTC
1 PIK3CD	GTTTAACGAAGCCCTCCGTGA	CAGCCCCTTTAGGACCAATGT
1 PIK3CD	CTGAAGTCTGGTGAGCCCAA	GCCTCCTCGTTGCTGTACAT
1 PIK3CD	CAAGATGAAGCCCCTGTGGAT	GTCTTCTCCACCCCCACTCTA
1 PLEKHG5	GACAAGGTCACTGGTTCTTGAGT	GGAGCCTGAGGATGAAGGAG
1 PLEKHG5	CTGGAATCACCTTGCTGTCCTT	CAGTCTCTGGCCTCCCCTA
1 PLEKHG5	CACGACCAATGGGAACCTGA	CTACCTGGACCAGTCCAACAC
1 PLEKHG5	CCTGTAGGCCTCGAAGGTGA	CCGCCTGGTTCTAACACACAT
1 PLEKHG5	GGGCCGTGGAGGCTTTT	TGTGGGCTAGCGTGATGG
1 PLEKHG5	CCGTACCATCTTGAAGCCTTTGA	GTTCAGCAACATCCCGGAGA
1 PLEKHG5	CCATCACGCTAGCCCACAG	CGGGTGAGGTACCAGGAA
1 PLEKHG5	CACCCTGAGCCCCATCAA	CGGTGAGGAGAAGCTCACA
1 PLEKHG5	CACACCCCCTCCTCCTCA	GGGCATGAAGGACTCCAAGT
1 PLEKHG5	GCAGAATCGGCAAACTCAGG	CTCCACCAGACCAGGGTACT
1 PLEKHG5	CCTCCCCAAATGCGATGCT	AGCTCTACCGAATCAGGACCA
1 PLEKHG5	GGCAGTGAGCGTGGAGTTAA	GCTCCCACAGGAAGAGGTG
1 PLEKHG5	CCGAGGGCAGGTCTCCA	CTCTGCCTGGCTGTTCCA
1 PLEKHG5	CATGGAACATCTGATCACTGGGT	CTGGGAGGAGGAGTACGATGAA
1 PLEKHG5	GAGCCCTGAGTCCTAATACCTG	CCCCTGTCCAGCTGTTGA
1 PLEKHG5	CGGACTTAGACTTGAGCAGGTG	CCCCAACCTCCTTACAAGACTTTG
1 PLEKHG5	GGCATTGTCCTCATCCTCGT	CCCTGACCAGGGTATCTTTCC
1 PLEKHG5	GAGGCACTAGCTCTGCCATT	AGACCTCTCTCAGCACCACT
1 PLEKHG5	GTGGGCGTGGCAGATGA	TGGGCTGACTCTGCTCCT
1 PLEKHG5	CCAGGTATCTCTCTGCCCACT	CACAACCCTCTCCTGCTTTCTC
1 PLEKHG5	cagttacTGAAACACTGACACAGGA	GCCTGTGACAGCAAGTTCCA
1 PLEKHG5	ACATGCCCATCATAATGCATGGT	GATCTCGTGGACCTCCTGAAG
1 PLEKHG5	GGTGGTCATGAGCAGACGTA	CCCTGCAGAGCCTggaag
1 PLEKHG5	GCTCCACGGAGCCGATCTA	AAGTCGGTGCTGAGGAAGAC
1 PLEKHG5	GCTGAAGTGCCACTGtcctc	TGTCCCATGTACCCCAACCT
1 PLEKHG5	CTGGCCATCAGGGTTACCAT	CACAGTGCCAGAGGCTGA
1 PLEKHG5	CCAGCATGTCGCTCAGCTT	CGGGCCTACATCACGGT
1 PLEKHG5	CTTCAGCCTCTGGCACTGT	CGACCTCTTCCGGGCCTA
1 PLEKHG5	GCGCCCTCACCGTGATG	gCGCCGTGGTTGTTCTG
1 PLEKHG5	GAAGGGCGCAGGCTGTA	CCCACACCTCCAGCCTCT
1 PLEKHG5	GTCTACCTCCTGGAAAGATACCCT	CACTGGCGACAGCTGGAA
1 PLEKHG5	CCCGGTTCTTCCAGCTGT	GCAAGAACATGTCGGAGTTCCT
1 PLEKHG5	GCTGTCGCCAGTGTTGGT	CCTGACCACCCTTCCCTGA
1 PLEKHG5	GGCTCTCCAGCCTCCCTA	GTGCCCCGCTGACTGAT

1 PLEKHG5	CCGCCCTCCTTCCTGGTA	GTCACACCATCCCCTGATCC
1 PLEKHG5	GCATCCGGCTCATGCATACA	GCGTCAGCCAAAGAAGGACTC
1 PLEKHG5	GAGACTGACTCCCATCTCAGCTA	TGGTGAGCCGCATCGAC
1 PLEKHG5	CTGCTTTCCACCACCTCGTA	GTCATGACCCTGGACCTGAAG
1 PLEKHG5	CTCCTTTCACTCTGTGTCCTCAAA	TCAAGCATGAATTCAGTCCTGACAA
1 PLEKHG5	TCGAGGTGGAGACCCATGTT	AGACCTGTGTGTCTGATTAGAGAGG
1 PLEKHG5	CCTTCTGCAAAGGCTCAGACT	GAGGCCTCCTACATCAGGAAAC
1 PLEKHG5	CCCCTACTCACGTTGATGATCAC	GCCTCGTGAATGACCCATCC
1 PLEKHG5	GCACTGTGAAACTCATTCAGGTAGA	GCTCGTGGACAAGATTGTGTG
1 PLEKHG5	CTCCTTACCAGGGTCCCGTA	GATGGATGTGTACTGCTTCCTCTTC
1 PLEKHG5	GTCACCAACAGCAGATCCGT	GGTGGGAGGAGTGTTTGACAAAAA
1 PLEKHG5	CCCACTCACTACTCTGCACTC	ACTGACTTGGGCCTTCCCTAA
1 PTGS2	TCAAAAATTCCGCTGCAAGAAGAC	GATTGTACCCGGACAGGATTCT
1 PTGS2	ACGTGTTGAGCAGTTTTCTCCAT	TGCTGTCCTTTAATTGCAGCAAATC
1 PTGS2	TTCCGTGGCAGAAATTCTAAAGTTACT	AACAACTCTATATTGCTGGAACATGGAA
1 PTGS2	TGGTGAATGATTCAACAAACTGGGTAA	CTATCACTGGCATCCCCTTCTG
1 PTGS2	GGTCATGAATTTGAAAGGTGTCAGG	TGAGTGGCTATCACTTCAAACTGAAAT
1 PTGS2	TGTTTGTTGAAAAGTAGTTCTGGGTCAA	CTTGAAAGCTTGTGATCAGATGCAATAA
1 PTGS2	GAGGTCAGAGCGGAAACTCT	CCTCAGACAGCAAAGCCTACC
1 PTGS2	CCGCAACAGGAGTACTGACT	CCACAGTACTACTAAAAGAACGTTCGAC
1 PTGS2	CTTCTACAGTTCAGTCGAACGTTCT	CCTTTACTTCATTCAGTGTTCCAGATCC
1 PTGS2	TTGATGGTGACTGTTTTAATGAGCTCT	GGTGGAGAAGTGGGTTTTCAAATCA
1 PTGS2	GAGACTGAATTGAGGCAGTGTTGA	GCCATCTTTGGTGAAACCATGGTA
1 PTGS2	AGGAGAATGGTGCTCCAACTTC	TTGTATCTCTGTCTTCATCGCCTTC
1 PTGS2	AGGCTAAAAACCTTAGAAAGACACTTGT	CAGTGCACTACATACTTACCCACTT
1 PTGS2	TTATTCACAACGTTCCAAAATCCCTTG	CCCTCCCTTTCTTCGTCTTCTTG
1 PTGS2	AGTTCTTCAAATGATTCATAGGGCTTCA	GTTCCACCCGCAGTACAGAAA
1 PTGS2	TGGTCAATGGAAGCCTGTGATAC	CTAAGGCAAGCTGAATACAAACAGTAAA
1 PTGS2	GTGTGAGTTTTCATTTACCACATCTTGT	GGTGATGAGCAGTTGTTCCAG
1 PTGS2	GTTTACCTATCAGTATTAGCCTGCTTGT	CCTGGTCTGATGATGTATGCCA
1 PTGS2	TGTTCCCGCAGCCAGATT	GATGGAGAGATGTATCCTCCCACA
1 PTGS2	TCTCTGCCTGAGTATCTTTGACTGT	CAGGTATGCTTCCTTTGACTATTAAGACT
1 PTGS2	AGCAATGCAGCCCGTCTTATA	CAACTTACAATGCTGACTATGGCTACA
1 PTGS2	GTTAGAGAAGGCTTCCCAGCTTT	AGGTGCCACTTTCCACATTTTACAATA

1 PTGS2	CTCCTTTAATGTTAGCCCTTGACTATGA	CACTTCACGCATCAGTTTTTCAAGA
1 PTGS2	TGGCCCTCGCTTATGATCTG	CTTCCTGATTCAAATGAGATTGTGGAAA
1 PTGS2	AGGGATGAACTTTCTTCTTAGAAGCAAT	TCTTGGTAGATTGACAGTCACCATCT
1 RNASEL	AGCAGAAAGAAGAAAGGTAAACTGGATT	GTGCCTCTGCAAACTTTTCCTTAG
1 RNASEL	CTGTGGCATCTGCACAAAGTTTTA	GCTCAAAGTAATGAAGAGGtggttcaa
1 RNASEL	TCCATCGTAGATATAAACTTAGAATTGGATTTT	TCATCCGGAATTTGGGAGAACAC
1 RNASEL	GCTTTTCTTCATCAATGTGTTCTCCC	TTGTGTTGCTACCACATTTTTGTGTT
1 RNASEL	GAAGAGACGATGAATGAggtcctt	GCATCTTTGGCTTGATTTATGGCT
1 RNASEL	CTCTGGTTTGCCACTGGTGTA	GCCTTCTGAACATTCCAAAAGTTTTGAC
1 RNASEL	GAATTGTTCATACCTTAGTCGTCCACTT	GATCATTTAGTCATCTTCAGCCGCTA
1 RNASEL	GCAACTCATCCCTCACAAGCAA	AACAAGCCTCAGTGTGATGGAG
1 RNASEL	TGAACTCCAGCAAATCAGTCCAT	CAGATCTGGTGATCTATGTCTACACAAA
1 RNASEL	GGGAAATGCTTTCTATATTCTGTGTTCT	GGGCAAGCATGCTGAACAAT
1 RNASEL	CATCCACATTTACTCTAGGCCTTTCC	GCTGTTCAAGAACTACACTTGTCCT
1 RNASEL	CCCCCATCTCATCAAGGAGAATC	GGTAAGGTCAAAGCCCTAAAATTCCT
1 RNASEL	GCAGATCCTGGTGGGTGTATC	ACTTGTTTGTGTGTGTCACCCT
1 RNASEL	CCTCAAATTCACATTTGCTCCTCTCTTA	CGTGAAGCTGCTGAAACTTTTCC
1 RNASEL	CCAAACACGCTTCCAGAGTCT	GTAGCTGTGAAGACGTTCTGTGA
1 RNASEL	CCTCGCTGTCATAACAAGATCCC	GGAGCAAGAGCACATAGAGATTAATGA
1 RNASEL	GACAAGAGACTTCCCGCTGT	CCTATGATTGGCAAACTCAAGTTCTTT
1 RNASEL	CACTCATTGACATCTGCTCCTTTAGAAA	AAATGAGCAGGGAGGACATTGT
1 RNASEL	ACCATGACGAAGCAGAAGTTCC	GCTGATTAAAGCTGTTCAAAACGAAGAT
1 RNASEL	CTGTTTTGCCATCACTGTCTGT	GGCTATTACGCATCTGCTGCT
1 RNASEL	CCTCCTTCTGAAGTATCAGCAATTTTGT	TCTCTCTCATGGAGCCAAAGAAGA
1 RNASEL	GTCTTCAGCAGGAGGGTGAAAA	AACGTGGAGCCAGTACAGATTG
1 RNASEL	CAATTGCTGGACCAGGTCAAC	AGTGGTAGCAGGTGGCATTTAC
1 RNASEL	TTGACATCAGCCCCATGGTC	GAAAAAGGACACGTAGAGGTCTTGAA
1 RNF2	AATTTAAGTACATTTTTCTCATTGGCATATTCT	ACCTCACAGCCAGATACTTGGATAA
1 RNF2	GTCTTTAGCCCAAAGATACTAGCATTGT	CGACTTGGATAAATTTTGCTGATGAGT
1 RNF2	GGTAACGCCACTGTTGATCAC	GGCCACTGGCTGTTGCTATATAAA
1 RNF2	GGCCAGACCCAAACTTTGATG	CTGTTCATGGCCTGTATCTTCAGT
1 RNF2	GCCAGTGAGAAGCAGTATACCA	CCACCACTTGCCACTCAGTTAG
1 RNF2	CCAGGATCAACAAGCACAATAATCA	GTATTCAGAAAAACAGCTACCCTCTCT

1 RNF2	TTATTGTGGTTTGTGCAGTTTTCATAAT	AGGCTCATTTGTGCTCCTTTGT
1 RNF2	CCCATGGAACTTTATTACGCACCT	TCTGGCTCAGAAAGAAAATTGAACAAC
1 RNF2	AGTGACTCTTTTACAGGAGGCAATAAC	GTGATGATGCAGTCTGCACAAA
1 RNF2	ATGACTACAAAGGAGTGTTTACATCGTT	CCTGGAAAGCACTTTCGTGGTA
1 RNF2	CTAAAGTTCAGTAGCAAACATGCTTCA	ACCATTTTCAATCTGTTGTTTCTTGCC
1 RNF2	CCTTGACTAGACTGCAGCGA	TGTTACTAGGGCCTGCTTCCT
1 RNF2	CAGTAATGCATCCACACATAGCAATC	GGGATGAGGCCTGAATACTAATTCAATT
1 RNF2	TCCAGTAATGGATGGTGCTAGTGA	AGACTGCCTTTTACCTCCAAATTAAACA
1 RNF2	ccatagcaCTTCCCTTCCAAATACTAAA	CCCTTGGCAGTCATCTTAGTCATTAC
1 SDHB	CTCTGAGGCTCCAGGACTCA	CGATGTTCGACGGGACAC
1 SDHB	TCGGATGATCTCAGATTTTTAAGCCTTC	ATCTATCGATGGGACCCAGACA
1 SDHB	CATATGAGGTTTGTCTCCAGCCT	GTGCCAGCAAAATGGAATTATCTTGTAT
1 SDHB	CATGCTGTATTCATGGAAAACCAAGAT	GGAAAGCTATTGCAGAGATCAAGAAAA
1 SDHB	CTTTCTTCTCCTTATAGGTTGCCATCA	GGAAGGAGTTTCACCCAAGATTGT
1 SDHB	CCACAAGTATCTGGAGCCCAAC	GGGAGGTTGAACGTTACATAAATACCA
1 SDHB	GCCACACTCCTGGCAATCAT	CCATTGAGCCTTATTTGAAGAAGAAGGA
1 SDHB	CTGCTTGCCTTCCTGAGATTCA	AGGTGATGATGGAATCTGAtccttttc
1 SDHB	GCTTGAGTTTCAATTTCTCTTAAAGCA	GCCTCTCTTTTCTCCCCATACAG
1 SDHB	CAGGCATATGCTGGTCCCTTT	ACCATCATGAACTGCACAAGGA
1 SDHB	CCCACGTACCTTAGGACAGG	CAGCTAATCATCCCTGGTTTTCCA
1 SDHB	AATACTCAAACAAATCCTGCCCTGA	CAAAAATCTACCCTCTTCCACACATGTA
1 SDHB	CACGGGAACAAGATCCTTTATCACA	GGATGTGTTAAATGTGTGTCTCTTTCAG
1 SDHC	gcctggcTTGGTATTGCAAAATAT	TCTCTTCTTTGGCCGTGGTTC
1 SDHC	TGGTTTTATTTTAGTGCTGTTCCTTTGG	CCACTACCTCTAGATTCTCTGGCT
1 SDHC	GAGAAGCTCCAGAGCCTTTTAAAGA	CAACTCCCAGTCCCACTGAAG
1 SDHC	TGTAACTTATGAGCAGCTGTGACAA	CACAGGGACTTCACAAGTTCCAAATA
1 SDHC	ACTCCCTGGGAACTTTGAGTCT	GAGAAAATGTGCAAATCCCGAATTAACT
1 SDHC	GACTCTCTACTATGGTGTCATCTTTTCC	CATACCTGCACTCAAAGCAATACC
1 SDHC	TCTCTTCCCATGGCGATGTC	GTTCCCTCTAAATCAAGTGCTGAGT
1 SDHC	TCCCTTCACCCCTAAAAATAGAGAAGT	CTGATACAGAGCTGAGGGCTAAAG
1 SDHC	TCTGAGACAGGAACTGTTAATGTCCTA	CATAGAGGACAACACAGTAAGAACCA
1 SDHC	CACAGATGTGGGACCTAGGAAAAG	TGACAAACAGAAAGATGGGTGAATGT
1 TAL1	CGCCGCCGATGTGTAGAA	cgcTGCTCTACAGCCTCA

1	TAL1	GGCTGGCTGAGGCTGTA	CCACCGAGCTGTGCAGAC
1	TAL1	GGTCTGCACAGCTCGGT	GAGCCCCCAGTCATCGAA
1	TAL1	CGCGGCCCTTTAAGTCTCT	GCACCTGGTCCTGCTGA
1	TAL1	CGTTCAGCAGGACCAGGTG	CATCCGCTTGTCTGTCTGTCT
1	TAL1	CGCCCATCTTTGTGAGATACCTA	CCTTCCCCCTATGAGATGGAGAT
1	TAL1	GGGCAGACTCACCATCAGTA	GGATCTTGTGATCTGCCCGTTC
1	TAL1	CGGGCTCCTCCGTGTAG	ACTGGCAAGGACCCTGTG
1	TAL1	GTCTTGCAGGAGGTCATCTGG	CCGCCTGGCCATGAAGTATAT
1	TAL1	CTCCTGGTCATTGAGCAGCTT	GGCAGCAGAATGTGAACGG
1	TAL1	ATCTCATTCTTGCTGAGCTTCTTGT	TTCTCCTAACTCTTGTCCTCCTAATCTC
1	TAL1	GCCCTCCTGGCTGATCCT	CGGCAGCTCCCTGGATG
1	TNFRSF14	CTCCCTGAGGCTGAGTGAAC	GTGGAGCAAACAATGACGATGAC
1	TNFRSF14	ACTGGGTATGGTGGTTTCTCTCA	GACAGAGCTCCAAGAGGTGAC
1	TNFRSF14	CTTGCGAAGTTCCCACTCTCT	GGCTGTGCTGGCCTCTTAC
1	TNFRSF14	CCTCCACGTACCCCTCTCAG	CGTCCTGGACGATGCAGAAG
1	TNFRSF14	ACAGAGAACGCCGTGTGT	GGGACCAGGGTGGAAATGG
1	TNFRSF14	TCTGCTGGAGTTCATCCTGCTA	GTCAGAACTGGGATCTGCGA
1	TNFRSF14	GCCAGGGCATCTCCCAAT	CCAAAGGGCTGCACCTAC
1	TNFRSF14	CTCTGTCCGTCCCTCTCTTCT	CCTCCCCCGGGTTCACTTA
1	TNFRSF14	CCTGACCTGTGTGTCTGTGTAT	CACCATGTACACCAGAAACACCA
1	TNFRSF14	GGGTCTCCACGATTCGTGT	CCCGTGAATGAGGGTATTGTCT
1	TNFRSF14	GGTGAGGCCACAGTCATTGAG	CAGGTGGACAGCCTCTTTCAG
1	TPR	CATTCTCTAGGATCGAAGCTCATTTCT	AGTGTTCAGAATGAAGTACAAGAAGCTC
1	TPR	ACATAGTAGAGAGGGACTCAACAAACA	CTCTTGTGGTGCCACATCGTA
1	TPR	TGAATTGCTTCAGCAAATCCATCAG	TGTGGTGTTTTGTTTGTTAACTCTGTTT
1	TPR	CAACAAGCTGAGGTATGTGACTACTT	TCATCTGAGGTAATATCACAGCATCTAGT
1	TPR	CACTAAGAGACGTTGATTTTGTTGTTGA	AGAGTGCTTTTGATGGAACTTGAAGAA
1	TPR	ACGAATTACGTGGTTACCCCTTG	ATTTGAGGCAGTACAAGTTGAAGGT
1	TPR	AAAACTCACATTCCTCTTTCACCAGAA	CATTTTTAGTGGACTCTTGTGGGCTA
1	TPR	GTATCCATTCCCACATGGTAAGACA	ACCAAGCTGAAACAAAATCAAAATCACT
1	TPR	CTTCTGCAGATTCTCTACTTGACTTTCA	ACTGTTTGTTGTCTTAGGTTATGGAGAC
1	TPR	TGGTTTTCAACTCTGTATTCAGCCA	CTGCTTGTGTCCCTTAGCTACA
1	TPR	TATCATATATGCTTACACGATTTCGGTATCTAA	GGACGTAGGTACAAGACTCAATATGAAG
1	TPR	TCTTCAAAATACCTTATCCTGTTGTGCT	AGGAAACCATCCAGAAGGACTTAGAT
1	TPR	GAGTCTTGTACCTACGTCCAATTTTCTT	TGCATCTTTGACTAACAACCAGAACTTA
1	TPR	TGGATGGTTTCCTTTTCAGTTCTTACTT	ATTGGACTTCAAACTTTGAAAAATGTCTCA
1	TPR	ACTAGAACTTGTTTGTGGCATATCTTCA	TTGTTGATGTTCATTCTTCCTTCTTCCT
1	TPR	TCAACTCACTAACAAGCATCTATTAGCC	GATCATCTTCTAGAGGTGGAGCAATC

1 TPR	CCAATCTCCCTATTTCTCAAGCATTTCT	GGAGAAGTGACTGATCTGTTGCAT
1 TPR	GGACATCAGTTACATCTTCCCATTGAC	TGAAGGCACTAACTGAGAAAAACAAAGA
1 TPR	TGCGATCCTGAGCAATTTCAAGT	TGGCAGTTGCATAAAAACACTCATTG
1 TPR	ACAACATGCTTCCACTCTTGATAACT	GCATGAAGAACTGATGAAGAAAACTGAAA
1 TPR	AGTCTCTCCTTCTCTTCTCTTAGCATTTT	CTCATAGGAACAGGTAGACTTTCAACT
1 TPR	GAGTGGCTATTGCTTATGTTGCAAAA	CAAATGCAGCTTGTTGATTCCATAGT
1 TPR	CGGTACATATCACGCTGACGA	CCTGTGAATTAGGCCGACTTTTTCTA
1 TPR	CACAAAGAATTAGGCTATTTACTGACAGGAT	AGTCCAAGTTGCTTCTCAGTCTTC
1 TPR	ACCTTTACCAGTTCTCTGTGAAGACT	CAAAGAGACAACTGGATACAGAGACA
1 TPR	GAAGATTTGTCTCTGTATCCAGTTGTCT	ccGAGCTCCAAAGGTCTATAAAAAGAT
1 TPR	GCAAAATTCAATGATCATAATGGATGACA	GGGCAAAACTTACTGCTAACTAATCT
1 TPR	AGCAGTAAGTTTTGCCCCCTTT	CACTTGACACCATGTTTTTCAGCATT
1 TPR	GCAACACTATTGCAAATTAGCTCTTGAA	CAAGAACAAGTTACAGATTTGCGATCA
1 TPR	GGTATTTTGTGATCGCAAATCTGTAACT	CACAGTGTTACCTTCTCATAATCTCCAA
1 TPR	AAAACCAATGATGCTTAATCAATATTTTCATGT	GCGGCTTGAGCTAGAGAAACTC
1 TPR	GACTGTGGGACAACCTCTTTTCTA	TCTTATGTGGAGCACAATTCTAATACTG
1 TPR	GAGTCATCAGCGGCACTCTAA	GGAACAACAGGCCAGTATGGA
1 TPR	TTTATTCCTCCTCTCCCTCCCAT	CCCAACAGTTTTATATCCACAATGCCTA
1 TPR	CGTGTTTCCATGTTTTGGAAGAGT	TCAGGTGAGGAAACTGGAGTTAGATATT
1 TPR	TCAGCATTTGCTTCTTGTAAGGGTAA	TTAGTTATCAAGAGTGGAAGCATGTTGT
1 TPR	CAAGGATTTGAGCACTTTCATCCAAT	CAACTTCATCCCAGGATGGTCAA
1 TPR	caATTACAATGACATCATCTCCTTTGCC	AAGGGAGTTACCCAGGGAGATT
1 TPR	GTTTCTTCACTGTCTTCCATAGGTGTAT	GCTGCTAGTCCATAAGTACTCATTATTCTT
1 TPR	acaaaaCACAACTTTGCAGATTGTT	CAACAGGTGATGATGGAGATGAAGTAT
1 TPR	CCTTCAGATTCTGCCTCCACAA	CTGTATTTACTGAGAGCACCACCTC
1 TPR	AGGCATGTTCCGAAGCATCA	GGCAGATAGTAGGTTCTGTTTTATTGGT
1 TPR	AAGGCTTCTTTCCATATACCTTGCAAT	GCATCTAGTAAGTCAACAGAAAGATCCA
1 TPR	GAAAGGAGCTTCCGATATTCTTCTGTAT	TTGATTACTTTACATGCAGGACACCT
1 TPR	TCTGTTGAGATGAACTAAATGGCAACT	AGAAGAGCCATAGAGAGCATGGA
1 TPR	ACGGTTAACCTATTTACCTGTTGTTCC	TGAAGTTCGTTTAAAAGAGTCAGCTGAA
1 TPR	GGCTCTTCTTTTATCATCCTGAAGTTCT	AAAGGTGACAGAAGAAGTGCGT
1 TPR	ACACCCATTTTTACAATGACGTGACT	CAGCTACAGTGATGCCCACTAC

1 TPR	CTAACCTTCCTGTGATTCCACTTGT	GAAATAAGTCAACACCCAGGGCTA
1 TPR	GGCTTGATATTGGCTGTTGGTG	ATTCAAAACAGCTTTAATAAGGCTCTGA
1 TPR	CACCCTACAGAAATTCAGAACTCTGAT	ACTGGAGCGATCTGAAACAGAAAC
1 TPR	TCTATCTGGCTACTAAGCCTTTGTTTG	CTGCTTCATGTAGAAATGGGTTGTAAT
1 TPR	CCACATGCTTTTGAAGATGTTCATTTGA	GGGAGTTACTTAGCTTTTCCTTAGCA
1 TPR	ACTTATAATGCAAGTTGGACAAGGGAT	GAAACTCACTGCCACAACTCAAAAG
1 TPR	CTAGCTTCTCATTTGCTCCTCTCAAA	CCAGTTATGAAATGCTGCAAGATAATGT
1 TPR	GGCTGTAGCTGAAAAGAGTACCT	AGTGGAAATGCCTCTTCCAAAGA
1 TPR	CCTACAGGTGTGACACTTTTCAACT	CAGTTCTTCTTTGCCAAAGCGT
1 TPR	GCTATCCTCTTCCTCTTCACGTGT	CTGTGTTCTCCTTTAGTTAAGTGACCAT
1 TPR	AAAATACCAAAAAGTTCATGGGATTGCT	GCCCCACCTCAGGAGTTG
1 TPR	AGTACTTTTAATACCTGAACTGGTGGTC	ACAGCCATCATCAGCATCTGAAA
1 TPR	GCATGAATGGTCAGTCTTGGG	CAGGTGCTGCAGAATCTTCTTTTT
1 TPR	CTGTTGTTCTCTAGAAACCTCCTGA	ATTCGTACGGTGAACATTCCTGTT
1 TPR	GGTGTAACCATTGGGCGGATAC	TCTATAAGTGCCAGCACATCAGAC
1 TPR	GTGAAATAACAGCTTCGTAAGTGCTTTA	GACTGGAAGAACAAATGAATGGCTTAAAA
1 TPR	ATTTAAAGCACACATACACACACAAAC	GGCTATTGTAGCAGCAAAGTCAAAAA
1 TPR	AACAGATATTTACCAGCTAAGTGTGCAA	GGAACAGACTGTGCAACTTCAGT
1 TPR	CCTGACGAAGTCGTGAAAGTTCA	ATATTGAAATGATTGAATTCCATGGTGAATAC
1 TPR	GTTCTCTAAAAACCTAACCCAGGACAAA	CAAGTACATCACAGACTGTTTCCAC
1 TPR	TCAATAACAGGTACTGGAGCAGGA	GCTGATTTATGAAAGCCAACAAAGCATT
1 TPR	CAAAGATTACTGGGCAGTCAGGTT	CAGGAGGACACTGATAAAGCCA
1 TPR	CTCTCTCAAGTACAGATGATTGCTTGT	TGGTGGTGTTTGAGTATTGTATGTCTTT
1 TPR	AGCAACGGTGTGGTACTTTAGG	ACCCTTCATCTACTACACTGATATGGTT
1 TPR	TCTCATTTGTATTGTTACCACGGCTAAA	ATGAAATAGTGAAAGAAGTGGAAGCCA
1 TPR	TGGCGTTTCAAAATTGGTGCTT	ACAACCCTTTGTCTACTTTCAGCTC
1 TPR	CTTTAACATTCTCTCTCTTTCCTCCCA	CTGCATGCTGCTGATGTTGAA
1 TPR	GCTCCTTCGCAGCTTGTAGAG	CCAATGTGTATCTTTGGACAAGTGCTA
1 TPR	ACCTGTTTTTCCTTGTTCAGGGATT	TTTTAGGTCAGCCTAGCAACAAAGA
1 TPR	GCTGACTCACAAGATCATCCACATC	TGTATTTGAACCTTAATGCTAATTTCATAAGCT
1 TPR	AACAGAAATGGGCTGAGCACTA	GGAAGAATTAGAAGCTGAGAAAAGAGACT

1	TPR	GCTACAAGCACTTCTTCATACCTGTTAAG	TCGCTTTCCTGTATTGCTCTACTTG
1	TPR	AGTTTATGCATTAGGAATCATCTGGTACTTG	CCATTCTAGAGGATGTTAAACGTCTGA
1	TPR	GTTCACCCTTTGTTGTATTGCTTTCTT	cagctgTGCATACTTTGTATTAAAGGT
1	TPR	AGGCTTAAGGATTAGAAAACTTGTTCCA	CTAGTAGTAGTCAACCAAAGCCTTTCA
1	TPR	CTGAAAGGCTTTGGTTGACTACTACTA	AGAATTGAAATCAAATGAACAAGTCTACATCTT
1	TPR	ACCACAGAAATGAAGCAGTAAGAACA	ATTAAGTGACAAGGTCGTTGCCT
1	TPR	GACCTTGTACACCTTCCTTCACA	ATTCTGATAGACTCTTGGCTTGCAG
1	TPR	AGCCTTTCCTAGAATGATCCACTAA	CAGCTGTAGCTAAGATAGTGAAACCT
1	TPR	GCAAGCTCTTCTTCAGACAATATGG	TATAATGTACAGTACATGTCACTTACCTTGAAA
1	TPR	TGTGGTTTATCACATTTTTAGCCAGAAGA	TGTTCCCACTACTCCACTACAAGT
1	TPR	GGGCTGCTACTTGTAGTGGA	TTCTTTCACATCCCTGACTTGTTGAT
1	TPR	GCTGTTGCTAAAACAAAGAAAACAGAGT	GACCCGAAGGCAGTCTGTAG
1	TPR	AGTCAACTGAAGGCCACGTC	GCAGCGTAATAAGCTGTATCTGTTGATA
1	TPR	AATGCATAGGTCTACTCTGACTTCATTT	TTGTGCAACCCACTCAACAGA
1	TPR	GCAGGCTCAATCTGAGGATGA	CAGTTTTTGGAAGCACAAGTGGAT
1	TPR	GACATTAGGACTAGTAGAACGAACGG	TGACATGTACCACAGAATAGGAAGGATA
1	TPR	CTTTCTTCTGTACCTAATTCTGTCCCA	GCAGTAGAGGAACTACACAAACTTTTGA
1	TPR	AAACACTTACCTTCACCAGCTTCTT	GTTGCATTGTTGACTGTTAGGTAACTTT
1	TPR	GTGCTTCTCTTTTGATTTAACAAGTGCT	CGTTATCGACAAAGGGTTGAACTTTTA
1	TPR	CAGCATTTAGACTATCTTGCAGTTCCT	ACGACGAGAAAAAGAAATTGCTGAAAC
1	TPR	CGATAACGCAGACTCTCAACCT	ATTAAAGTCAATGATTTTTGTCATCTTACCAAA
1	TPR	GCCAAAATTCACTGACAAAGGCT	GGAGCATCAAGAGAGACACCTT
1	TPR	CTTGAGGCTCATCTCTCTGCTC	GAGCCTTAGATCAGCAGAAAGATGAA
1	TPR	CGCAGTAATGCGAACATCCAA	GTGCCAATTAAGTGCATAAATGCTGT
1	TPR	GCAGTGTTTTCACAAACAAGTTTGATG	CTAGAAATTGATAGCCAGCAGGAAGA
1	TPR	CAGATGCTTGAACCGGCTCT	AGGGAGCTCTGGATTACTTAAGTGATAA
1	TPR	GGGCCAATCTCTTTTTACCAATTCaa	AGAAGAAAGTATGGGTGGAGGTGA
1	TPR	AAGGACGGAAAGAGCATCTCAC	GCCCAAGTCTGTCCAGAACAA
1	TPR	CTGATCAGCAAGGAACTTTTCAAGT	GCCTTTCTTCTCCGCTTCTACC
1	TRIM33	ACTTCCCCACCGAGCTACTA	ATGTGCTTTCCTTTCCTTGTTTGG
1	TRIM33	TGTTACTTTTGACAGGACTTTAGCAGaa	GTGGCCGATGTCCGTTTG
1	TRIM33	GGACATCGGCCACAAAGTC	GGAGAATTCTTATTTGCCATTATGTGCTT

1	TRIM33	GAAACAGGAATCCATAGCAGAACCA	GTGAAAGACACATCTGAAGCTCCT
1	TRIM33	TTTTTCATCAGAACTGCTAGGAGCTT	GGACATCATGCACAATTTCTTTATTCCT
1	TRIM33	AAAAGTGTAGTGTTGAGGGCTTCAT	CACCTGTACCAACTACAACAACAAC
1	TRIM33	GCTTGTCTAGGATGCTGTTGTGTT	CAGCTTCGACTCCAGCACAT
1	TRIM33	TGATGTTTCTGTGCATATACTTGTTGTTG	CAGCTCCTGGTTATACTCCTAATGTT
1	TRIM33	TGGAGGAACTTGCCCAACTAC	AGAGGAAAACATGGTGATTACTCAACAAA
1	TRIM33	GGCAGACAACTACTGAATCCCA	GGACTGGATATGCACATTTTGTAGAGAT
1	TRIM33	CAATCATATTCAACTTCTGGCTTTCCAAT	CCCAGTCCAGGGAAGTAGCTT
1	TRIM33	GGCAAAAGAAGGAGGTTAACTGTAATC	ACATGTATCGAAGCACATCAAAGAGTAA
1	TRIM33	GACATCTTCTTTCTTCCTGATCAAGTGA	AGATTAGTGCTTATTTCTTCTTTTAGGTATGT
1	TRIM33	CTGCACTTGCATTGTCTTCACA	TCTGTGTACAGTGAAATAGTATATTGAAGAACA
1	TRIM33	AAGCACTTTTTATAGTATTTCCTGGTAAGCA	TGCCCTGTACACAAACAAGAACA
1	TRIM33	TGTTCCAATAGCTGACAGTCTCTACAT	GCTACAGAGTCTGTTGGAGCA
1	TRIM33	ACAGGGCGTTGACCAGATG	CTCCTTCTTTTGCCCCTATAGAATTTT
1	TRIM33	CTCAGAGCTGAAAGAATCAATGAAGTG	CAAGAACCTGGGACTGAAGATGAAATA
1	TRIM33	TTGTTTTACACCTCCTGAAAAGCTACA	TGTTGTTTCTTATTAGCTGTGGGTCAT
1	TRIM33	GTATGTGTCTCACCAGTCGCT	AAGTTACTACAGCAGCAGAATGACAT
1	TRIM33	CCCAATTTGTGAAGTTCATAACATGCTT	CATATGGAATTCTACAGCGAAGCTCTT
1	TRIM33	TTGATAAGGGTGAAAATGGCCACT	ACAGTTGATGATTCAGAGACCTAGGAT
1	TRIM33	GAAGCCTCATTCAACTCAAATACAAACC	ACGAGCCCTACTACAGCAACTAT
1	TRIM33	GACCTCGGTTTGCATTTGCC	GCGATTCTGGCAGATCAAGTAATGATAA
1	TRIM33	CCTGTCTGAGTAGATCTCTGTGAGTTT	GTGAAGGAATATGCATTACCAATAGGTG
1	TRIM33	ACTGTAGGCAGGATATTCCAGCA	GACTTTATACAGCCCCGCAGAA
1	TRIM33	GTCTCTCATCTGACTTTAGGCGTT	GAAAGCAGTTGCATTGTACTTTGAAGAT
1	TRIM33	TCAATATACTCACCTGCCTCGACT	GTGACCGGTAGGTAGAATTCCAAATAT
1	TRIM33	TCCTTCACCAAGTTTGCATGGT	GCCAATCCTATGCAGTCTCATAACTTTA
1	TRIM33	GTAATACCCGTTTGCTTTACCTGATG	TTCAGTTGGAAGATGCTGGCT
1	TRIM33	GATTATCTAAACTACTTGAGCCAGCATCT	GGCTCAGCTTGATATTGGATAATCTGTT
1	TRIM33	AGTATTTTCTCCTTCTAATTCAGTGTTCCAAG	CTGTCCCTGCTGCTAATGGA
1	TRIM33	CCTAAATTGACTACATTCTTTGCCCAGA	CTTTAAGATTACTTTCCAGTTGCGTCAT
1	TRIM33	GGATCACACCGTGCTTTCAAAAT	TGACACTGTGGGAATATATGTGCTTTTT

1	TRIM33	CCTGTTTTATAGAAATACTGTGCACTGG	TTCATCTAACTTGTCATGTTCCAACACT
1	TRIM33	CACTGGTACCTTGGAAAGCTAAGT	TGGCAACAATAAAGATGATGACCCA
1	TRIM33	AGACAGCACACCAGTCTTCATT	CCATGTGAAAATTGAACCTGCAGATATG
1	TRIM33	CCTGACTGTTTGCAGCTTTCATT	TTTGTCATCGTTTTGTTTTAGTTGAGCA
1	TRIM33	AACAGATTTAAACTGGACCCACTTCTT	TTTCAAAATCAGAAGGGTGCAATTGAG
1	TRIM33	TTCTCAAGAAGTTTCGCCAGTAGATT	GTTTCAAGATTGACTCATTTAGCTGTTCT
1	TRIM33	CTCCTCTAGGGTTGCGCAAAT	GCCCTGTCTTCACTCCTTCTG
1	TRIM33	TGGATGTCGCCGTTGCT	CAGCCTCGCTCCTGGAC
1	TRIM33	TGCAAGCTCTGCTGACACA	CTCAGTGGGCACTGGAGTT
1	TRIM33	CAGGTGTCCAGGAGCGA	GGCCCAGGCTGCTTCAT
1	TRIM33	TGTAACTCTAGGCATACTTGTCTTTGGA	CTTTTCAAGCCCATCAGATGAGACT
1	TRIM33	CCTGGTATTCTGGCAGCATTCT	AATTTCTACTTTTTGTAGCCTCCTCGAT
2	ACVR2A	AACAAAAACTGCTGTGGCGTT	CTGCATCTGGGTAATTCTTTCACCTA
2	ACVR2A	GCCAGGTTATCAGCTGGATGTG	TCCCATTTCTTAGTGTGCACAGATG
2	ACVR2A	CCTAAAGTTGCTATCTTGGGAAACAGT	GGTTCTGTCTTTTTCCCAATTAGCATT
2	ACVR2A	GTAGATCAGAAACTCAGGAGTGTCTTT	ACCTTGTTTCACTATTTCAATGGAACCA
2	ACVR2A	GCATTGTTTTGCTACCTGGAAGAATAT	ATGCACTGATCTTTCCCCTTTCAA
2	ACVR2A	CCTGAAAGGGAAACTCACAACCT	CACAGTTCATTCCAAGAGACCACAT
2	ACVR2A	GGTTCACTATCAGACTTTCTTAAGGCTA	GGCAGGTTTGTGGCCATCT
2	ACVR2A	ACATGAGGATATACCTGGCCTAAAAGA	CTGTCTTGTAAAGCATGATTGCATAACA
2	ACVR2A	GTGTTGATGGAATAATTTGCCCCTAC	GGTGGCTTAGGTGTAACTGGATT
2	ACVR2A	CCCTTTTCCACAGCCACTTCA	CCATCTTGTGATGCCTGTACAC
2	ACVR2A	GGGATTGTCATTTGTGCATTTTGG	TGGGTTTCACCACAGTAATATTTCTCAA
2	ACVR2A	TGGTGTGTGTCATGTTCTGCTTAT	ACATATTCGTTAAGCAACTGGGCTT
2	ACVR2A	GGGAAGATTTGGTTGTGTCTGGA	GGCTTCTGTTTTGAGACTATTTCTGAGT
2	ACVR2A	GCCCTGAAGTATATTTTTGTTGCTGT	CAATCTACAGTTGAGCAAACCTACGTAT
2	ACVR2A	TTCTTTCTTTTTGACTCTAGGACAAACAGT	TCAACACTGGTGCCTCGTTTT
2	ACVR2A	TGAAGCATGAGAACATATTACAGTTCAT	CTCTGAGGGATTTATAGCCAGCATT
2	ACVR2A	CCCTGGTAATAGACCACATTTGGTT	CCCTCTAATACCTCTGGAGCCAT
2	ACVR2A	AGGTTGGTACCCGGAGGTA	GCGAGAAGCCAGTTCCCATAG
2	ACVR2A	AGGATAGATATGTATGCCATGGGATTAGT	CCTTGGAGTGTATCATCCCTTTTCA
2	ACVR2A	GTGTACATATTCCCCCTTTTCTGCT	GCCAACCCAAAGTCAGCAATG
2	ACVR2A	CTGTTGAAAAACAACCTGACAGCTT	ACTAATTCAGTCCTGACATGCCATT

2	ACVR2A	CATAGCATGTAAACAGTTGGGAATAGGT	AGAGATGGATGCTGGCCAATTT
2	ACVR2A	GATGAATACATGTTGCCATTTGAGGAG	AAGCTAACTGGATAACTTACAGCATGTT
2	ACVR2A	GGCCTGTTTTAAGAGATTATTGGCAGA	ATCACCTTCCAGAAAATACTTGCAGAA
2	ACVR2A	CGGTTGATTTTACACCAGGAGGTT	GCACCTACCTGAAGAACAGGAGA
2	ACVR2A	TGGCGTTTGCCGTCTTTCTTA	GGACTCTCCGACTCAACACC
2	AFF3	GGCAGGTGGTCATTGAGAATGAT	GTGTTTCAGCTTCTTGTGGTGTT
2	AFF3	TCAGAGATACTTTAGGGACAAAATCCGA	CATCTCTGCCCTTTCTTTCTTCCA
2	AFF3	TCTGCAGACCTTGACCAGAGT	GCAAACCACTGCAACATGAACATC
2	AFF3	GAGGGAATGCACGTACCTGTT	AGTGCTGATTGACTTTAGTTTATTCCCA
2	AFF3	GGGTGTGCGCTCACCTC	ATCCCACAGCGCATCCA
2	AFF3	TGGTGATGCTGACGTGGTT	CATGTCTCCCAACCCCTCTC
2	AFF3	GGAGAGGCTGCCCTGAGA	GCCACTGCGCTTTGCTTT
2	AFF3	TGCATGTGAACGGTGCTGAT	CCTGATGAGTCTCCTAAGCTGAAG
2	AFF3	CGCTGGTTTCCGAAGACGA	GCGGACACTGTGTTCAGAACT
2	AFF3	GTTTTGAAGCTAGGGATGGAGGAA	GCTCTCTCAGGACCTTGCTTG
2	AFF3	GCTGTCTGCCAACACCATCTC	ACAAATCTGCTAAAACCCAGAGAACTAT
2	AFF3	CTGGGCTGAACAGACAGAAGAT	GGCACGTTTAATTCTAGTTACTCTCTCTT
2	AFF3	CATCTACCTTGTAGGGCTCACTG	TTTGTGTGTGTGGTTGTTCTCTCT
2	AFF3	AGTGATGAAGCTGCAGTACACAC	GTTGCACTTAAGCCATAATGACGTTTA
2	AFF3	TGGTTTTAATTGGGAAAGGAAGAGCAT	CACAAATACACCAGCGAGGACTTA
2	AFF3	AGGAACAATTCTGCTCATTCACTAGAC	CTTCTGGGCCTGTTTCTCTTTTC
2	AFF3	CATTAGGTCGGCTGGAAGAAGT	CTGACTCTGTAACATTCCATTACCGTAA
2	AFF3	GCCCGGACTTGCTATCCT	cagcTCCTCCAGCGACTC
2	AFF3	AGATCCGGAGCTGCTCTCT	GTTTGCTCACTTGGCTTTCCTTC
2	AFF3	CCCACTAGAAATGCCTGCATGA	CAATATTGTTTGTTTTTATCCCGTCCTT
2	AFF3	CGACTCTGTCTCAATGAACTCCTT	ACCAAAACCAGGCCCTGTG
2	AFF3	CCCGGCTGAGGTCCTCT	AGTAAAGGCGTGAAGCAGAAGT
2	AFF3	GCCTGGTGGGCTTCTTG	ACAAGGCCCCTGGGAGTA
2	AFF3	GGACTTCTGCTTCACGCCTT	GGAAAGTCCCCGACGTTTG
2	AFF3	GCTCTTGATCTCCTTCTCTCTCAGG	AGTTAATCCCCACAAGCCTCCTAT
2	AFF3	GGCATATTTAAGAACAGGCATCTCTGAT	TCTTAAAGACTGTAAGACTCATATGTCGGT
2	AFF3	GAGCCGCAGGTTTCCTTTCTA	GGTCATCAATCTATTCCATCAGGCTTAA
2	AFF3	CTCCCCTCATCCAGCAAGAAAA	GGCTTTGAACTATGCTGAAGCAG
2	AFF3	CATTTCCACACTCGATAAACGACAATG	CAGAGTTGGGTTTCTCAAATAAGGTGTA
2	AFF3	CACCACATTTAAAAGCGTCTGAACTTA	TGTGTGTTGTTTGTTGTTTAGAAAGCAG

2 AFF3	GGGAATGTTTTCAGAATTGTTGTGAG	ACAAAGATGTTCTGTAGAGCCAAAGAT
2 AFF3	CCACCATTCAGCTTGGTTAACA	TCTTTGTACCTCTTTGTTCCTTCTTTCA
2 AFF3	CCTGCTCCCCAGATGAAACTC	GGTTCTGAATGACCAGTTTTAAACATGAT
2 AFF3	CATGCATTGTCTGGCTTACGG	TGGCACTCACTGTCAGTGTTTT
2 AFF3	AGCAGATTTGTGCAAATCAAGCAA	AGCCCTCGTCTATCTGTAGCA
2 AFF3	GCTGCTGGTGTGGAAGTTGTA	GAGTCATCTCGTTGGAGTTCCC
2 AFF3	AGTCTGAGGAACCCCAGGTTT	CAATAGCGTGGATGTGTTTTTCTTTCTC
2 AFF3	GCACCTGGTTTGCCAATGATG	CCTCCTCCCCGCTTTTTCTAAG
2 AFF3	AGCCTATGGGTGCTCTATCCAA	CACACCTCGGACACACCTG
2 AFF3	CTTGGATTTTGGCAAAGCCTTTTCT	GAGATCAGGTCTCTCTGGGTCA
2 AFF3	ACAGGAGGGTCAGGTCGATTT	CCAGGACCACCAGTGACATC
2 AFF3	CGTGGCTTTCATTTTGGATCAGA	AAGATGTTTTGTTTGTCACCGGTTTT
2 AFF3	GTAGAACTGCTCCTCCAGCTC	GAGTACCCTCTGTCCAAAGCA
2 AFF3	CTCTGATCATTCCCGGAGGAG	CTAAGCAGGATCGTCCCCAAAT
2 AFF3	GGAAATGTTCACCGCAGTCTGA	GGTCCAGTACTCCCAACAGG
2 AFF3	CAGGGTGAGGTCCCTATGACA	GCATGTGTGGCATTCTTAACAACT
2 ALK	AACTGCTTAGTAACTAGCAGAAGTGTTC	GGATATATGCCATACCCCAGCAAA
2 ALK	CTCCAGAACTTCCTGGTTGCT	ggtgtgtCTATATCCATCTCCATGTC
2 ALK	CACAAGTGTCAGTGGATATCAGGAA	GTGACTTCCACCAGGACTGTG
2 ALK	CATAGGCAAGACTCCAGGCTT	AGTGCAACAACGCCTACCA
2 ALK	CCACGCTCAGGTTGGAGTTC	TGAGTGACTGCCTCTCCTCTT
2 ALK	ATCTGGCTCTCATCTTCTCCCT	GCAAACTTACCATGGTTGTGTGT
2 ALK	GCACCAATCTTTCTTCTGCCTTTTG	CCAAATCAAGAAACCTGTTTGAGAGAA
2 ALK	GGTTTCAGCTCCTTGTTTGGGT	CCTGAAATCAACCCTCACCTAACT
2 ALK	CGAAGTCAACTGCCAGACTCTT	GCCATCGGGCTCCTGTG
2 ALK	CACAGCTGCCGTGGAAAG	TGGGTACCAAGGACTGTTCAGA
2 ALK	AGAGCAGAACAGGAGTGAAGTCT	CCAGATGGACTTGCTGGATGG
2 ALK	GGGCATCTCCTTAGAACGCT	TCTGGAGTGCAGCTTTGACTTC
2 ALK	GGGAATACTCCAGCTCACAGG	CTTGGACAGGGCAAAGGATGT
2 ALK	AGGGTGTCTCTCTGTGGCTTTA	GACTCTGTAGGCTGCAGTTCTC
2 ALK	TGCATATAGGGAGCTGAGGGAAT	AGACTCCTTCCCTTTCCTGTCTC
2 ALK	CATATACTTACCATATCGGCTGCGAT	CAAGCACATGGATCAGTGTTTTCTTTA
2 ALK	GGGAGGACTGACCTAAGCAAG	CTCTGCTCTGGTTTGCTACTCA
2 ALK	GGGTGAGGCAGTCTTTACTCA	CGTTGTACACTCATCTTCCTAGGGAT
2 ALK	GGGATCCCAAGGAAGAGAAGTGA	CACTTCCCTTGTGGGAATGTCAA
2 ALK	CCTGTTGCTGGTAGCCGTAA	CTGGAGGGAAGCTGTACTGTC
2 ALK	CTCCCAGTTGCAACGTTAGGT	GGTCCACGGATCCAGAAACAAG
2 ALK	CGTACGTTGGGTTCCACAAG	GAGATCTCTGTTCGAGTCCCTAGA
2 ALK	CACAGACTCTGGAAATGTGTAACCAA	CCCTCTGGAAGGTACATTGCC

2 ALK	TCACGTGTCCCCCTTCCA	GGTTCCTCCTCTCCTGGTCTC
2 ALK	GATCTCTCTTGCAGCCTCGTT	CTTCTCAACACCTCAGCTGACTC
2 ALK	CCTCATCCACGGACTCAGGAT	CAGTATCTCTGATTTTGATGCCATTTGT
2 ALK	GCACAGGAACCTGGTGGAAATC	ACAATGTGATAGAAGAAGAAATCCGTGT
2 ALK	CACTCATGCACGCTTCTGTTC	AGAACTCTGGTTTGGCCACAAA
2 ALK	CCTTTTGTGGCTAGAGGAGTCT	CATCAGTGACCTGAAGGAGGTG
2 ALK	CCGAATGAGGGTGATGTTTTTCC	AGAGCCCTGAGTACAAGCTGA
2 ALK	GGTTGTAGTCGGTCATGATGGT	TGGCCTGTGTAGTGCTTCAA
2 ALK	AGGAGGAGGGTGTTGAGTGTT	CATCTTCAACCTGGAGAAGGATGAC
2 ALK	CCCAACCAGGATGTACAGCAT	AACGTTCTTTGTCCCATTGCAG
2 ALK	TGTGGTCATGGGCCAAATCTC	CTGCAGTCACTGTGAGGTAGAC
2 ALK	CTTTCAGGGTCCATGTGACATTC	CAGTGACCACCTGGAGACATAGTA
2 ALK	GACTAGCATAACGAAGTGACACCTT	GATCCTCTCTGTGGTGACCTCT
2 ALK	ACCAATCATGATGCCGGAGAAAG	TGACTTGGGACCAACTCAAAGG
2 ALK	GGAGTGACACCTTGAACACGAA	CGGAAAATCTTTGCAGGAGGGT
2 ALK	CCACCCCCACTTCTTCATGG	CTCACGCAGGTTCCCTATCT
2 ALK	CCTGAGTCTCCCATCTGTCTATGT	CACACTCCTCAATGGCAGGT
2 ALK	GGGCATCCTTTAGGGTCCTG	TTTTGCCTTTAGTGGTCCTGAAAAT
2 ALK	GCAGGGATACCTGGAGGATGAT	CTAGTTTGGTTTTCCTCTCCTTCCC
2 ALK	TCCCAGGTGCCAGTGACTAA	GGAGAATAACTCCTCGGTTCTAGGG
2 ALK	GCTCCGGAATTGCCGTTTAG	TGTCTGAATGTCTCCCCTGGT
2 ALK	GCTCAAGAGTGAGCCACTTCT	TGACTCTGTCTCCTCTTGTCTTCTC
2 ALK	GTTGATTTCAGGGTGAGAGATGATT	GACAATATCTCCATCAGCCTGGAC
2 ALK	TGAACTCACTGGTGAGGTAGCA	CCTCACACACATTCCTCTTGATGT
2 ALK	CGGTATTTGCCATCTTTAAGACTGTTTC	AGATACCGGATAATGACTCAGTGCT
2 ALK	TGTCTTCAGGCTGATGTTGCC	AGTTCGCACCCTCAACGTATTC
2 ALK	cagccCACTCTAGACTTTTCTTCAT	CAGTGGACTTCTTTGCCCTGAA
2 ALK	AGATTCAGATACCTTCACTGCAGTTC	TTTTCTCCTCTGTTTTCCAATGCAAC
2 ALK	ACTGCAGCAAAGACTGGTTCTC	GCTCTGCAGCAAATTCAACCAC
2 ALK	CCAATGCAGCGAACAATGTTCTG	AGCTGCTGAAAATGTAACTTTGTATCCT
2 ALK	CGATGAAGCAGAGCTGAGGTT	GCAATTCGCGCCTCCCA
2 ALK	CCAGTCCCGAAGATCTGGAAGA	AGTTCAATCTCAGCGAGCTGT
2 ALK	TTGGCGAATCCACCAACTGA	GCTGTCCAGGGTGCTGAAG
2 ALK	GCTCCAGCACCAACTGCTT	GGCTCGCTAGCTCTGGAC
2 ALK	CCTTCAGCACCCTGGACA	CTACTGCTGCCACCATCCT
2 ALK	GCAGTCCAGAGCTAGCGA	CGGGAGCCACTCAGCTACT
2 ALK	CCTCCCGTTTTGCCTGTTGA	GTTTTAATAATTTCCCCTACGGCAGGA
2 ALK	ACAGGAAGAGCACAGTCACTTTG	CTCACTGACAAGCTCCTCGT
2 ALK	AGCTGATAGAGGAGAAGGGTATTGG	CGTGTCCTTGGTGCTAGTGG

2 ALK	GCTCCTTCCCGGTTTTGTTCT	TTCAGCAATCATACCTTGTCCTCTG
2 ALK	GAGGTGGGAGGAGAAATTAGAGAAC	GCTTGTGATTCTTTTCCTCTCTCCTA
2 BCL11A	TCTTCGCACAACGGCTTCTT	GTGTGGCAGTTTTCGGATGG
2 BCL11A	GAACTTAAGGGCTCTCGAGCTT	GCCAAGTCACACCTGTACTTTTT
2 BCL11A	GATCCTGGTATTCTTAGCAGGTTAAAGG	ACGCACAGAACACTCATGGAT
2 BCL11A	CCGTGTTCGCTTTCTAAGTAGATTCTTA	CCTCAGTGATTAAACATTGATGTTGGT
2 BCL11A	CGGACTTGACCGTCATGGG	TCTGCGGCAAGACGTTCAA
2 BCL11A	ACCACCAGGTTGCTCTGAAAT	GCCCTATGCAAAGGTTACTGCAA
2 BCL11A	GCTTGCTACCTGGCTGGAAT	CCTCCCGCCATGGATTTCTC
2 BCL11A	CTGCCAGCTCTCTAAGTCTCCTA	CCACCACCGAGACATCACTTG
2 BCL11A	CCCCCAGGCGCTCTATG	CCTCTCCATGGGATTCATATTGCAG
2 BCL11A	GCACTTCACAACTCCTTACTGCTT	TCAACGTCATCTAGAGGAATTTGCC
2 BCL11A	ACCTGCTATGTGTTCCTGTTTGG	AATGGCAGCCTCTGCTTAGAAA
2 BCL11A	GAAGGTGGCTTATCCACAGCTT	GGATCATGACCTCCTCACCTGT
2 BCL11A	TGGGAAGTTCATCTGGCACTG	TTTCTGTCCTCTCTGGCACTCTA
2 BCL11A	GGGCATATTCTGCACTCATCC	GTTGTTTGTAGCTGTAGTGCTTGATTTT
2 BCL11A	TGCGCGCTCTCGTGattat	GTGAACCGAGCCGTCGT
2 BCL11A	CTACTCTGGGCACAGGCATAG	GACACTTGTGAGTACTGTGGGAAA
2 BCL11A	GGCCATTAACAGTGCCATCGT	GGCATGGTGCTCAGCTC
2 BCL11A	AGTGAGATTGCTACAGTTCTTGAAGAC	AGCTGGACGGAGGGATCT
2 BCL11A	GGAAGGCCTCGCTGAAGTG	CTACGGCTTCGGGCTGA
2 BCL11A	CCCCGCGAGCTGTTCTC	AACGACCCCAACCTGATCC
2 BCL11A	CTCGCTCTCCGTCAGctc	GTCCGTGGTGGCCAAGT
2 BCL11A	GGCCCGGACCACTAATATGG	CCCTTCCTTAGCTTCGGAGACT
2 BCL11A	GGGTCGTTCTCGCTCTTGA	GACGCACATGCACAAATCGT
2 BCL11A	GAGGCAAAAGGCGATTGTCTG	CCCCTTCTCTAAGCGCATCAAG
2 BCL11A	GGCAGGTCGAACTCCTTCTC	GCGAGTCGGACCGCATA
2 BCL11A	GGGTGTCAGGTGGGAGTGA	GGGAAGGACGTTTACAAATGTGAA
2 BCL11A	GTACACGCTAAAAGGCATCTTACAAAT	CCTTATAAATGCGAGCTGTGCAA
2 CREB1	CTGTCCTAAGCACTAGCAGCTT	TCTTCTTCAATCCTTGGCACTCC
2 CREB1	TTTGAATGACTTATCTTCTGATGCACCA	ACTCACTATACTGTCCACTGCTAGTTT
2 CREB1	CGGTGCCAACTCCAATTTACC	GGCACTTGTATTGGTTTCTCTGAAGA
2 CREB1	AAAATCCATTGGCTTTTAGTTGCCATT	TCAGTGGTCTGTGCATACTGTAGA
2 CREB1	CCTGCAAACATTAACCATGACCAAT	ACCACACCTGTGAATTCTGAATTCC
2 CREB1	GTTGCAATGTTTCTGTCTTACACCAT	GGAGGATGCCATAACAACTCCAG
2 CREB1	GCACCCACTAGCACTATTGCC	ATCCAGCAGAATGTGAAGACACA
2 CREB1	TTTCCCGTCCTCTTTTGCTTGTA	CTTTTAGCTCCTCAATCAATGTCTTGTTT
2 CREB1	AGAGTGGCAGTGCTTGAAAATCA	CCAGTCCATTTTCCACCTTAACAGG

2 CREB1	GCCTTTAAAGTCACGAAAGCACAA	GTCATTTGTTGGTTTTCAGCTTCTGT
2 CREB1	CAGAGTGGAGATGCAGCTGTA	CTCTAGGAAGAGACACAAACTGGAAT
2 CREB1	GTGTGGTTGTCTCCCATAAGAACT	TCCTGTGAATCTTCACTTTCTGCAA
2 CREB1	TTGCAGAAAGTGAAGATTCACAGGA	TCTGACATCTCTTTATCTGCAACTACCT
2 CREB1	GAACGTTCATTCAAGTAGGCCAT	CTCCAAATCTAGGACCTAGGACTCA
2 CREB1	ATATTGAACATGAGTACTGCCAAAGAGT	CCATTGGGCAGCTGTACTAGAG
2 CREB1	CATCATCTGCTCCCACCGTAA	ACACATACCTGAACTGTTTGGACTTG
2 DNMT3A	GAGGAGCTGGCAGTGGAA	TGGCTACCACGCCTGAG
2 DNMT3A	GCATTCTTGTCCCCAGCATC	AGCCAAGGTCATTGCAGGAAT
2 DNMT3A	CCTGGTTTTCTTCCACAGCATTC	AAACATGGTCCCCTTGAGTGTC
2 DNMT3A	CTCCCAGCAGGGACACTC	AGTGTAAGCCTCGGCAAACAA
2 DNMT3A	CCCACCAACAAATTAATAAGCCAAACC	GTTCTCAGAGCTAAGTATCAAGGATTTCA
2 DNMT3A	GGTGTGTAGGATGTGACACTCA	GGATCCACCTCTGGCCTCA
2 DNMT3A	TCCCCCATCCTGGGACAA	ACCTGTTGTGCTCACTGCTTAG
2 DNMT3A	CCACCCGTGTCCTTCTTCTA	CCTTTGTTCTTCCCTCTCCACAG
2 DNMT3A	CCTGCTGGAGAGCCAAGT	AGCTGAGACCCTGCCTGA
2 DNMT3A	ATTTTCCACTGCTCTTGAGGCT	GGCGCCTCAGAGCTATTACC
2 DNMT3A	CGCTTCTCCAAGTCCCCATTG	ACCCTCCTCTTGTGTATCTTTCTTACAT
2 DNMT3A	GCCCCACAGCATGGACATA	TCCTGGTGCTGAAGGACTTG
2 DNMT3A	CAATGTAGCGGTCCACCTGAAT	GGGAAAAGATAGGACTTGGGCCTA
2 DNMT3A	GTGGACACAGTCAGCCAGA	CATCCCCTCCCTCTGCTTT
2 DNMT3A	GCCCTGGGATCAAGAACCTTC	GCATTGTGTCTTGGTGGATGAC
2 DNMT3A	CCAGCGGGTGCCTTCAG	GGCCTCGTGACCACTGT
2 DNMT3A	ACTGTTCCCAGGACGTTTGT	GACATCTTATGGTGCACTGAAATGG
2 DNMT3A	GCACAACCCGGGTACCTTT	GCTTATTCCTCTTTTCTCCTCTTCATCT
2 DNMT3A	AAGCCTATGTGCGGAAGCA	CCCATCACGTTGCCTTTATCCTC
2 DNMT3A	AGAGTTCCCAGGCAACAAACTT	AAGAGGTAGTTGGCCTGCTTC
2 DNMT3A	GCTCCACAATGCAGATGAGACA	GATGATCGCCCCTTCTTCTGG
2 DNMT3A	TGGCCACCACATTCTCAAAGAG	CCACACCACTGTCCTATGCAG
2 DNMT3A	GAAAGCTGGGTGCCCTCAT	GAAGCTGTTCCCGGTGTG
2 DNMT3A	CCTTGGCAGTGTCACTCTCAT	ACCCTCACTACTCAGAGTCTGG
2 DNMT3A	GAACATGGCAGAGCAGCTAGT	GGGTAACCTTCCCGGTATGAA
2 DNMT3A	CCAGGAGCTTTCACCAACCTG	CTCATCTTCAAACCGTCTCCTGTT
2 DNMT3A	CCAGCTAAGGAGACCACTGGA	CTTTCTGGAGTGTGCGTACCA
2 DNMT3A	GTAGCCGTCGTCGTCGTA	GGGTCATGTCTTCAGGGCTTAG
2 DNMT3A	AGTAAGTTCTAAGGGTTAGCCTGAA	GGACCCCTACTACATCAGCAAG
2 DNMT3A	GCCACTCGTCCCGCTTG	CGTCAGTGCCCACCCTAATG
2 DNMT3A	GCCAGGCTCCTAGACCCA	CTCCAGATGTTCTTCGCTAATAACCA
2 DNMT3A	CAGCACTCACAAATTCCTGGTC	AGACCCCTGGAACTGCTACAT
2 DNMT3A	AGCCCGTAGGTACCCTTGT	CTGAGAGTCTCCTCTGCTCACT
2 DNMT3A	GCCCGCTGCTCACCTTT	ACGATAATTCCTTCCCCAAAGCC

2	DNMT3A	TCAATGTTCCGGCACTTCTG	CCCAAGGTCAAGGAGATTATTGATGAG
2	DNMT3A	GCAGGCCAACTACCTCTTGT	CTCCTCCAGAAGAGAAGAATCCC
2	DNMT3A	CACATGTCCGTGTACACTTCTTTGTA	GAGGTGGGAACAAGTTGGAGA
2	DNMT3A	AGGGCTGCGGAGATCCT	GCAGCTACTTCCAGAGCTTCA
2	DNMT3A	TGTCGCTACCTCAGTTTGCC	GGTATTTGGTTTCCCAGTCCACTA
2	DNMT3A	GCTCATGTTGGAGACGTCAGTA	GGTCCTGCTGTGTGGTTAGAC
2	DNMT3A	CCCCAGCAGAGGTTCTAGAC	GTTACAGTCTCTCTTCTGCCTCCTA
2	EML4	AATCAGCAAACAAATCAAAGCTCATGAT	CCATCCTTACCTCTATTTCTCTTTCAGG
2	EML4	GATGTTATTAACTGGAGGAGGGAAAGAC	GTGGGACAAAATACCTGAGTTTAGAGTA
2	EML4	TGCTTACAGCTATAAATGCAGGCT	TTCCTTCTGCTACAGCTCTGATTG
2	EML4	TCTAGGTTCCTGATCAGTATGGCA	TCAGTTAACGGTTAATATCACACAGCTT
2	EML4	acctagccAGAATTTTTCTCATCTTTT	GAGCTGACAGGCGATCTTGA
2	EML4	TTTCTGCTGCAAGTACTTCTGATGT	TTCACTGAGGCCACATGATCTTC
2	EML4	TTTGAGGCGTCTTGCAATCTCT	AACTAGTTTTATATTTCCAGCTTCAACTTTCA
2	EML4	AGGATTTTGGGTTATCTTACTATTTGCAGTT	GCATATGCTCATTGGAGTCATCA
2	EML4	GCATATGCTTACTGTATGGGACTG	TCTGCTTTTGCGACTTACGAATAGAT
2	EML4	ACTTCTTAACAGGTGGTTTGTTCTGG	AATTGCAGTGAAGTGCTGTGAATG
2	EML4	TCCATTTCTGAAACAGGCATGTCA	TGATGCTCATGTCTTTTCCACCAG
2	EML4	CAGGTAAGCTGTGTGATATTAACCGTTA	TGAGCACATGTCAAGAGCAAATCTT
2	EML4	CTCACAATGACAGTCACCTGATATCAA	CGGTGTGGGAGTATTAGATTGGATG
2	EML4	GTCTTGCCACACATCCCTTCA	GTTAATAATCACATCCTGCTTCACTCAC
2	EML4	CCGTCTCTTCCACTGAAAGTGT	AGTTTGTTCCAGGCTGTTCTCC
2	EML4	GCTACTGTCCCCATAGGGAGA	CACCACTTCAGATTACTCAGTTCACTTA
2	EML4	GAATAAGCAGTTCTCCCACACTTCT	TTATCTCGTTGCATGGCTCTTCA
2	EML4	GAGACCTTGGTGAGCCTCTTTA	CATGCAGCTGAAGGAAAAGAGTTG
2	EML4	CCCCTCCTTCCAAATGGACTTA	TTCCATTGGTTATACAGGACATGGG
2	EML4	CCAAGCCCTCGAGCAGTTATT	TGCAATGTTAGTTTTTCCCCCTTTCATA
2	EML4	TCTTCAGCTAATTTTTCTGCATCCCT	TCGGCAACAGCTATCACCTTTC
2	EML4	CACTGGTGCGATCCCACAATA	TGGACAGAAGGCCTTATAACATTAGTTTC
2	EML4	GGGTGTGACACTGCTTCCTC	GGGACCAGTCAAGGTGTGT
2	EML4	ACCTTGACTGGTCCCCAGA	GCCCCTGACAGACACATCTTA
2	EML4	GATCAAAGTCCACAAATTCGAGCAT	TCATTACAACCATTCCCTAGCTCTGTA
2	EML4	GTGGATAGGATTCATTCATTAATTGCCAAAT	CCTACAGCCAGGAAGGTACCA

2	EML4	AAGCATCAAGTTGTCATGGCAAAAA	CATATAAGCATGACTCCACCTGAGT
2	EML4	GGTATTCTGTTGTTTCATGTTTCCTGTG	TCTCTAGGTACAGAAGGCATAACACT
2	EML4	GGGAATGGAGATGTTCTTACTGGAG	TGGGACTGAAAGAGTACTATGTGAGAAT
2	EML4	AGGAAATGTTAATTGCTGCTTTTGTGT	GGCTATCATCTGAATTTTCCCAAGAATT
2	EML4	CCATCACCAGCTGAAAAGTCACAT	ACTTACTTGTCTGCAGTTTTGGTAACTT
2	EML4	GTCGAAAATACCTTCAACACCCAAATT	CAAACCAGACATACATAACTGTACATGC
2	EML4	TGGCTAGTTTGAATCAAGATGCACTT	CCTTACAATCCGATCGATTCCTGA
2	EML4	GACATTCCAAATGGCTGCAAACTAA	CACTTCTTTACATCAGGTGTCATTTCCT
2	EML4	CTAGAGAACGAGCGGGTCAG	AAAGTTCCTACCCTTCGCAGAAG
2	EML4	TCTAGGCATAAAGATGTCATCATCAACC	TGAACTGATAAAGTGCACTGGATGAAAA
2	EML4	GGCATTTTCTCAAAATCTTGCCCTAT	TGGTATTTGCATCTGTTGGGTGAAA
2	EML4	AAATGAAGTTGTTTTGGCTGTGGAG	TGATTCTTACCCCAAAAATTCCCTGTT
2	EML4	GAGCGGCAATTCACTAACAAGAA	TGCCATGACAACTTGATGCTTATTAAAC
2	EML4	GTTCAGTCTTCTTCATTGTACCTTCCT	CCGGTCGGAAGAAGGTAAACAT
2	EML4	TTATCGAGGAAAGGACTGTAGAGCTA	AATGATACCAACCATTTCACACAGTCT
2	EML4	CGACACTACCTGGGCCATAC	GAGCTTTCCACAAGAAATCACTTTCAAT
2	EML4	CTGTGTGCTATTGGTGTTTATTTGCAT	CAATAATCTGCAGTGTGGATAGAGTAACA
2	EML4	CCACGTCAGAGTGTGGGATTC	ACTGAGCTTAACTTCTTTCCTGCTTTT
2	EML4	AATGAGACTAAAACTTTGAAGTAGTCATTTTTG	CCGACCGCGCATAAACATTTT
2	EML4	TGTCTTGTGTTTCAACAGAAGGAGAAT	TGAATTTGCTGTCATGGTAGAGAAGG
2	ERBB4	TCTGTTACTTACGTGGACATTTCTTGAC	TGACAGTGGAGCATGTGTTACTC
2	ERBB4	GTTGGATTGTAGACAAAGGTTTGGG	CCTGTTTTGGCAGCGTTTCAT
2	ERBB4	CGGATCCGCCCATTCTGTTTATAAAA	AAAAGCTGAGTACCTGAAGAACAACA
2	ERBB4	GTGCAAGGTTTACACATTTTAATCCCAT	TGTTTTGAGCTTGTTTGCTGAATGTT
2	ERBB4	GCCTTCTCTGGCATTGACAGTA	CACAATGCATCCAATGGTCCAC
2	ERBB4	CACATACTCATCCTCGGCCTTG	ACCTGAATCCAGTGGAGGAGAA
2	ERBB4	TTACCATAGCTCCAAACGTCACTC	GCCAGCCCAAAGACTCACATTTA
2	ERBB4	GAAACTCTTCAGCTTCCAGAGGAA	AACTGTACCAAGATCAATGGGAATTTGA
2	ERBB4	ACCCATGAATACCAGTGACTAGAAAGA	CCCTAACTCTGAGTCTTGTTTCTACAG
2	ERBB4	TGAACTTCGAATGGCGATCGT	CATGTCCAAGAGGACAGTAGCA
2	ERBB4	GGTCAGCACTGTACCTCTGG	AGGAGTGTCTGTGCCCTACA
2	ERBB4	CTTCTGGAATTGTGCTAGTTGGG	CTAACAACGCCTTCTCTCCACAT
2	ERBB4	TTAAATGCCTTAGAGTGTTCCTCAATGT	CAGAAAAGATGGAAACTTTGGACTTCAA

2 ERBB4	CCTTACCTGTCAAGTTCTTTAATCCAAGT	TCGTTACCTGCCTCTGGAGAAT
2 ERBB4	GTTTTGTCCCACGAATAATGCGTAA	TCGTTTTATAACACAGGCAACTGTTTG
2 ERBB4	GGTTGTCTAAAGTAATAACTCCATTGGCT	GAGCTGAAGAGGGTAAAAGTCCTT
2 ERBB4	ACCGTTCCAAAAGCACCTGA	agagGACTGACTATCGGACTGAAA
2 ERBB4	AAATGTGATTGCCTGGGTGTCT	AGGGTATTTGGGTACCTGAAGGA
2 ERBB4	CATAGCGCAACAGTTGCAGTT	TCTCTTCTTGATTTTCTAGGGACCCTT
2 ERBB4	GGTCTATGGCTTCAATTGCATTGT	GCATTGATAACACCCCTACTGCtaatt
2 ERBB4	TGAGGTGAAGGCAACCCTAGAA	AAGGAATGGTGTGCAACCATCT
2 ERBB4	CAACAGCCATCACTGGAACAC	GCCACCAAAGTCATTTGCCATATATCT
2 ERBB4	CTTACCCTATTCGAGTCAATTCTTGCT	GCAAGTTCTTTCAGAATCTCTTGGATGA
2 ERBB4	GCATCCATCATATCTTCCAAATCCTCT	CATATTTGTGTCTGATGGGCAATCTTTC
2 ERBB4	CCAGAGGCATGAAATGGACCAAT	GTCCTTTTTCCCTTTTCGTGTTCTG
2 ERBB4	CAAGCAAGATTGCTCTCAAAAAGATACC	TCTTAGTGAAATCTCCAAACCATGTGAA
2 ERBB4	TGGCTAGCCCAAAATCTGTGATT	GGAAAACCTGGGCATTAACTGAATATC
2 ERBB4	TTTTGACTTAATGCACACACAATGCT	ACCAAACATGACTGACTTCAGTGT
2 ERBB4	ACCAATGGTCACCAGGTTAGAAAAA	CCCAACTTTCAACACATTTTCACCATAT
2 ERBB4	CAGGTCTGCCTGTATGCATCAT	GCAAGTACTATGAAAACTGTGAGGTTG
2 ERBB4	GTTATCTCCAGGTTGCCCATGA	CACGTGTATCTTCTTGTCTTTGCAG
2 ERBB4	AGGAGGGCAGAGCATTTTTCTTA	CTCAGGACCTAAGGACACAGAC
2 ERBB4	CATATTTTCAATATGAAAACTGTTCCAGGTTAG	ACATTGGATCACAACTGCTGCTTAA
2 ERBB4	CACTTACCTTAGCTATCTGGACACA	CACACCTAGTCCGGTTGCT
2 ERBB4	GGATGGTTGGGCTCAGACAC	AAATCATCAGCACCATTAGTACAATCCA
2 ERBB4	GCATCTCTGTATCTTCCACTGGGA	CACCTTACAGACACCGGAATACTG
2 ERBB4	AAACCACAACTGAGCTTACACCA	CCTGCAGGAGTACAGCACAAAATAT
2 ERBB4	GAGGCAAACGTTCTCCTTTCTCTAATAA	TGGTATAGTGCTGGTTTGTTCAACATAT
2 ERBB4	GGGTTGGGATAACAGAGCAACA	TTCAGCACAATCAACCAGAGAATAGT
2 ERBB4	AGCTTTTCTGTTGTCCCGGATT	GAAATCAGCGCAGGAAACATCT
2 ERBB4	ACACAGGTTGCTGTTGTCAGTAATAT	GTTTTCTCACTTCCCCCTCCTTAG
2 ERBB4	GGTACCAGGGATAAAGATACATGTGGAT	GAAAAATGTCCAGATGGCTTACAGG
2 ERBB4	GATAACTAGGAAAGGATTTGAGCGACA	TCACTGTGGATGGAGAATGATGTTTT
2 ERBB4	GCATACTTGAAAATGAAACTGTTTGCC	ATTCCAGAGGAAATGACACCACTTT
2 ERBB4	CATGATACTAAATAAGCCAACACACCAC	TCCTCATGTGGCTGATGTTTTCA
2 ERBB4	CTCACTGTTGGCAAAGGCAAA	AAATTGGACACAGCCCTCCTC

2	ERBB4	ATTGTGAAATACTTACTCCTGACATGGG	CCATGTGGCTTTTCTTTTTCGTTTCT
2	ERBB4	ACCTGATGAAAATCCCCAAACACA	CTGGTCATTGTGGGTCTGACAT
2	ERBB4	TGCTCTTCCTTCTAACATAAACAGCAA	CTTCCTCTGTCTTCTCTCTCTTCATACT
2	ERBB4	AGCATAACTCATTCATCGCCACAT	TGGCAAGATATTGTTCGGAACCC
2	ERBB4	AAGAGTCAAGTTGGAAGGCCAT	CCTTTTCATCAACAAGCAGTTTGACAA
2	ERBB4	TCATTTTGCAGTGAGTTTGCCATT	ACTGTGTAAATTTCCAGGTTGGATGAT
2	ERBB4	CCTTAAATTTAGGTCTACTGTCAGCATCA	GCTTGCTTTCTTTCTCAGATCATTACGA
2	ERBB4	CCCGGGATGGGTGAAGAG	CTGGGTGAGCCTTCTCGTG
2	ERBB4	CCACCTGACTGAGAATCGCT	GGGATATGCCATTTGGACATGTA
2	ERBB4	AAGAGAGAAATCACAGACATACAAAGCA	TTTGATCATGGTCCTATCTCTGTCTTCT
2	ERBB4	ATCTCTGCTATTACTTTACTAAGAATGATGATG	CCAACGCGAGAAATCCCTGAT
2	ERBB4	ATTAAAAACCTTGTTATATAGGCCCAGTT	GCCTGCCCTAGTTCCAAGATG
2	ERCC3	CCACCCCATATGCCACACAAA	TGGTCTAACACAGCTTCATTCTTCTCTA
2	ERCC3	GTAAGTGCTGGGTTAAAGACACCT	GACCCCGTGATCCGAGAATG
2	ERCC3	GCCTCCCCTTCAGAGTTTCTTAA	CCATCTAATTGAATGAGCAGTGGAATCT
2	ERCC3	GAAGGTCAAAGAGGTGGAAGGA	GCAAACATGTACACCCGCTCT
2	ERCC3	GCATCATTTCCTAAAGCGCTTGA	GGAGACTAACATGGGCTGGTT
2	ERCC3	CAGTCGTGGCTGAGCGT	CCGGATTGAGCCGGAAGTC
2	ERCC3	CCCAAGCTAAGGGCATGCTTA	GGGAAGCAGGTGGATGAGTC
2	ERCC3	CCATATTCATCCACTTTGGTGCCT	GGGCTTACTTCTGTGTTGGTATCTTG
2	ERCC3	GGACTCCTTCCTTTCACACAGA	GAGCAAATGGACAAGGATGAAGAAG
2	ERCC3	GACACTGTCTGTGTCTCTTCTTCTT	TTCAGCACCTACCTCTCTCACA
2	ERCC3	GAGTGACCTCCTGCAACAGAA	GTAGGTAGGTGACACTTCGTTTGA
2	ERCC3	GGACATTTGCTTCCGGCAGA	CCCTGGTATAAAGAGGAGAAAAGAGTGA
2	ERCC3	ATCAGGTCCTTCCCATATCCCT	GCACATTTACCTGTTGGATGTTATCTG
2	ERCC3	GGGTATCAAGAAGGCCTTGGTC	AGGACACACAGGAAATGGCTTAC
2	ERCC3	AATCTCTGCCGCTTGGTTGA	TTGTCTTCTTTATTGCCGTTTCTCATTTT
2	ERCC3	GTTTGTCTGTTCAGCAAGTGGAC	GTTGCCATTAGCACCTACTCCAT
2	ERCC3	AGGACCTTTTGGTGGTGTGG	AGTGGAAAGCCCAGTTCAAGATG
2	ERCC3	GATCTGGCTGTCGTCAATGGT	GGGTGGGATTGCACTCTTGTTT
2	ERCC3	CTGCCCATGAGGAATCGATCT	AATTTTCTGATTGGGCCTAAGCTCT
2	ERCC3	CTCCATCCAGTTGGCTTCGT	TGAATGCTCCCATTGTTTCCTCAG
2	ERCC3	GAATTGCTGGTCTCAGCTGTC	ACCCATGTGCATGAGTACAAACT
2	ERCC3	CTGCATACAAGGAGTAGGCAGTT	GTCTGTTTTGTCCATTTGGTATCTCTCA

2 ERCC3	TGGAACCTCCTCACCCATTTACT	TCACGAAACTCGCTGGCAT
2 ERCC3	TCGAAAACGCCAAGTCTTCCT	GGAAGTGACCAAAGCAGGTG
2 ERCC3	GAGAAACTGGCCTGGAGGAAG	TGTACACCATGAACCCCAACAAATTTA
2 ERCC3	CTTGATCAGAAACTGGCAAGCTC	AAACTTGTAAAATGACCCCTGGTTCTTA
2 ERCC3	CAACAGCCTGACCACCTTCTTA	CGGAATGATTCTGTCAACCCTGAT
2 ERCC3	TGTGGGCTTTAGGTCAATGTTGAT	GCACAGTAACAAGATTTTAAAACCCCTT
2 FN1	CTCTTGCTCTTAGCTCCTCCTACT	GTGCGTTACTCACCTGTGAAAAAT
2 FN1	CCCAAAGATAAAAAGACAATGGCAACA	GGCATAAGGTTCGGGAAGAGG
2 FN1	TGACAACTCTGCAACATCTTCCTC	GAGTCTTCCTTCTTGTTCGAAACCA
2 FN1	AGAGTTGCCCACGGTAACAA	GCTTTGGATGTACCGATAATTGCAG
2 FN1	GACCTAAAGTCTCCAATCATTTCTGTGA	GTCAGGGAGAAAATGGCCAGAT
2 FN1	CCAAGACATGTGCAGCTCATC	ACGTCCCAGTTCACCTACTGT
2 FN1	GGTATGGTGTATACTTGCCCTCTGA	AGAGCTTTGTGTCAGCTTTGACTT
2 FN1	GCAGGTCCGCAGTCAGAATC	GGACCAAGTTGATGACACCTCA
2 FN1	TCTGCTCCAGCGAACAACAAT	CAGGTACATGTGTGCTACATAGTGTTAA
2 FN1	GTTGGCTCAAAAGCTGGGAAAAA	ATCTCACATTTCCAAGTACATTCTCAGG
2 FN1	TGGGATACTCACAGGTCTCCA	GCTCAAGTGGTCCTGTCGAA
2 FN1	ggccGAGATGCAGATGATTCTTA	CCGATGTTCAGGACAACAGCAT
2 FN1	CTGATTGCATACAAGTCAATGGCATTT	GGCACTGACTACAAGATCTACCTGTA
2 FN1	CCGAGCATTGTCATTCAAGGTG	AGAAAAATTGCAGGCTAGCTTGAGA
2 FN1	GAAGGCAGCCACTTGACACTA	CTTGTCCAATCTTGTCTCCAAGGA
2 FN1	ACAACCCACAAGGGCTTCATC	AACCACAATTACACTGATTGCACTTC
2 FN1	CATGTTGTCTCTTCTGCCCTCA	CGGTAATCTTTTCCTTCTTTGTTGTGTA
2 FN1	GGGCTTGTTGTCACTTACCTT	AGCCGGTTGTTATGACAATGGA
2 FN1	CTCCCCTGTGCCATTCTCATAA	TCTACACCATCCAAGTCCTGAGAG
2 FN1	GCGCATCTCTTTCCTGTCCAT	GGTGTTTACTTTGCATTCCTTCCTCTA
2 FN1	GTGCCCCTCCTGGAAGAATAT	AGTACGGCCACCAAGAAGTG
2 FN1	GGTGGTGAAGTCAAAGCGAGT	CCGTTGGAAGGAAGCTACCAT
2 FN1	GTAGGAGTTTAAGTGGCCTGGT	TGCCTAAGTCCTGCTTAAGTCATTTT
2 FN1	GGCACTTAATTTTCAGCTTACGTGT	ACAATGTGGGTCCCTCTGTCT
2 FN1	CAGATTCCTCAGTGGGTACTTGG	CCACTAACCTCCAGTTTGTCAATGAAA
2 FN1	CATCTCACCAGGACAGTAGAATCAG	CAACCTTGAGGAGAGAAAACTTAGACAT
2 FN1	CACCTGGTGGTGCAATTAACC	GAGCTATTCCCTGCACCTGAT
2 FN1	GCTCTGCAGTGTCTTCTTCACC	GCATTCACTGATGTGGATGTCGATT
2 FN1	CAAGTGAGGGTGGAGAATTTGG	CCATCAGCAGGAACACCTTTG

2 FN1	GGCTTTCCCAAGCAATTTTGATGG	ATGCTTATTTCACAACTTGCTTTTCACA
2 FN1	ACAGCCCGGTGACTTCTG	GACCTGCAGTTTGTGGAAGTG
2 FN1	GATGGTGACCTTCACGTCTGT	GCCTAACATATCCAAGAAACTGGCTT
2 FN1	CACCTACATTCGGCGGGTA	CTGGGTATGACACTGGAAATGGT
2 FN1	CAGAAGTGCCAGGAAGCTGAAT	GGCTCTAACCTCTCTCGGCTA
2 FN1	TGAATCTTTGGCCAATTGCTGACT	GAGAGTGCCCCTACTACACTG
2 FN1	TTCAAAATTCACCCGTTTGTTGTGT	CAGGAGAAGGTATCCCTATTTTTGAAGA
2 FN1	TAGTATCCTACTGAGGAGTCCACAAAA	AACTCACTGACCTAAGCTTTGTTGATAT
2 FN1	TGTCACTCTCAGGATAGCAGCTT	CTGATCAGAGCTCCTGCACTT
2 FN1	GCCGGGACTCAGGTTATCAA	CCTCTGTGCAATAACTTCCCTTTTCT
2 FN1	GCCCCATGAGAAGTGAAGAGAA	GCCTCTACAGGACAAGAAGCTC
2 FN1	ACCAACTGGGTAGGAAAGTCTTCT	GGAATTCCATCACCCTCACCAAC
2 FN1	ACATACTCTGTGCCTGGAGTGA	TCTTTTACTGTGCACTGGATTGCT
2 FN1	GATCCTGTAGCCAGTGATGGTG	CACTTCCTAAACTGGTGGTGTCT
2 FN1	CTGGATAGTCATACTGCCAACACC	GTTTCTCATGCTTTTGAGCAGAGTTTTA
2 FN1	GGCAAACAGTCAGGAAGTGCTA	CCTGGCCGAAAATACATTGTAAATGTC
2 FN1	ACAAAACAACCCTCCTTCAGGAA	AGAAAATCAAGAAAGTACACCTGTTGTCA
2 FN1	CTCTGCTCCCCATCCTCAGATAT	AGCTGTAGAGTCATTTGAGGGACTA
2 FN1	GTGCCAGTGGTTTCTTGTTGAA	CAGCACAGAACTCAACCTTCC
2 FN1	GTGACGGAGTTTGCAGTTTCA	TGTCTCAAAGAAAACAGTGCATCTTACT
2 FN1	GTAGTAAGAAGGAAAATGACAGCATGGA	AATTCCTTGCTGGTATCATGGCA
2 FN1	TTGATGATGTAGCCGGTAATCCTG	TCGGGAGAAAAGTGTGGGAGTA
2 FN1	CCCTTTCTGTACCTTTTGCCATCT	TCAGTGGGATAAGCAGCATGAC
2 FN1	GCACCTCATCATGTGACCCAT	CTGGTGATCATTTATCAAGTGCATTTCT
2 FN1	CCTTGGGAGAACATTGCAAAACA	GCCTTAAACCTGGAGTTGATTATACCA
2 FN1	CCAGTGACAGCATACACAGTGA	CCTCACACTCTGAACTAACCCTTTTT
2 FN1	GATAATGCATACCTGTCTCTTGGCA	TCCTCCCTATGGCCACTGT
2 FN1	AGAGTAGACCACACCACTGTCT	GCAGAAAGTAGTGAATTGTCTTCTCTCA
2 FN1	CACAAAACTTCAGCCCCAACTTT	CAGGCTCAGCAAATGGTTCA
2 FN1	CTTGCTTTGACTGACAGCCA	TCTCCCCCACCGTCTCAA
2 FN1	CCTATCTTTCTCCTTGTTACCTGCAA	GCCCATTGACATAATGTGATGGTTCTAT
2 FN1	GTATTACCCTTCTCTCTAGGAATCCAGA	CTACCAAGGCTGGATGATGGT
2 FN1	CTTCTCCCAGGCAAGTACAATCT	GGAGCTTGCACAATTCAAAATAACCTTT
2 FN1	TCCAATGAACAACGGTCATGTCT	ACCACCCAATGTTCAGCTCAC
2 FN1	CACCCGCACTCGATATCCA	CCTCTAACCAATGACAATCTCGACAG

2 FN1	CTGAACCTGTCTTGAGCGACAT	GGCCACTTCTGAATCTGTGACC
2 FN1	AAATATTTCTTGACCTGCTTCCCCAT	AGACACCTGGAGCAAGAAGGATA
2 FN1	CTGGAGCAGGTTTCCTCGAT	CTCGACCTGATGTATGTATGTGCTT
2 FN1	CACAAAGCTACTGGCTGTGATTTC	ATGACTGAAGGCTGACTTTCCC
2 FN1	CTTTCAGGTTATCCACAACAGAATCTGA	CGAGGGACCTGGAAGTTGTT
2 FN1	GTTTTCACTGGAATTCAGGTCTATGA	GAGTGTGTGTGTCTTGGTAATGGA
2 FN1	CCTGTGCAAACTCATCTAGGGAAA	GACATCACTTACAATGTGAACGACAC
2 FN1	CCTCTTCATGACGCTTGTGGAAT	AGTGGAAGTAGTTGTATGCTTTGAGAAG
2 FN1	GCTTGCAGGTCCATTCTCCTTT	CCCTCTCCTAACATTTTCGTTGTATCTTC
2 FN1	tgaacccCACTGATTTACCATTCTTT	GCTCTTAAGGACACTTTGACAAGCA
2 FN1	GACAACTCCCTGAGCTGGTC	GCTGTAGTGAAACTCCTTGGGTTT
2 FN1	CCCATGAGATGGTTGTCTGAGAGA	CATAAGGCATAGGCCAAGACCA
2 FN1	ATGGATAACAGAAAATGACAGTGATGGT	CACTTATGAGCGTCCTAAAGACTCC
2 FN1	TGCAGGTACAGTCCCAGATCAT	GGCTCACATGGACTTTCCTTTTC
2 FN1	GTATGAGGGAACACACTTGTGCT	TGACTTCATGCCTCTACTCTCTCA
2 FN1	CTGGCAAATAAAACTAGGGCATGA	CTACTCCTGCACCACAGAAGG
2 FN1	GAAAGAAAGAAGAACTCTAAGCTGGGT	CAATTGAGTGCTTCATGCCTTTAGATG
2 FN1	CACCAAAGATGTCCGTCCTGT	GCATCTCTGTTTACTGACACTCTTTTG
2 FN1	GGGAATCTTCTCTGTCAGCCTGTA	CCTATCTATAAGCAGCTGTCATGAGTC
2 FN1	GCCTGGCACATCCAGTCTTA	GCGTGCATCTGTCTTGTGTCTATAT
2 FN1	ATGCATGGAACTTGAGGAGACAA	CAGTTTCCCATTATGCCGTTGG
2 FN1	CAGACATTCGTTCCCACTCATCT	AGTCATTCGATTTTCTTCTTATGGTCCAA
2 FN1	AAGCCCGTTTACATTGTGGGTAT	GACTGAGACGATCACTGGCTTC
2 FN1	AGAGCATTAATAATCACTGCCCTGTT	GAGAAGTATGTGCATGGTGTCAGA
2 FN1	GCCATAGCAGTAGCACTGGTA	AGGCTGTTTATCTTTTCCTTCCATGT
2 FN1	GCTGGAACGGCATCAACTTG	CAGGCACTAATCTCTAGCTATGCT
2 FN1	TCAAGACATAAACTAACCAACAAGTTCA	CTCTGGTTCAGACTGCAGTAACC
2 FN1	AAACCAAGCAGTGGTTACGTACT	GTGGATGTTGCATTTGCTTTTCAC
2 FN1	GCTCAGGGCTGTATCACAGAGA	CTGGGAGCTCTATTCCACCTTAC
2 FN1	GTCTCAGTCACCTCGGTGTT	GCTTGGTTAAACTGAGGTTCTCCATA
2 FN1	GCTGACTCGGAGTCTCAGTGATAAATA	ACATTTAACGACAAGCAAAGCTG
2 FN1	CTCAGGCCGATGCTTGAATC	ACTACTAGTAACCTGATATACACTGCTTT
2 FN1	GCTCCCACTGTTGATTTATCTGATAGT	AAAAATATGTACGTCAATATTCACATGAAGAAA

2 IDH1	CCAACATGACTTACTTGATCCCCAT	AAGTTGAAACAAATGTGGAAATCACCAA
2 IDH1	CGTGCCACCCAGAATATTTCGT	ACGACCAAGTCACCAAGGATG
2 IDH1	GCCAACATTATGCTTCTTTATAGCTTCT	GCTCTATATGCCATCACTGCAGTT
2 IDH1	TGTAGATTCAGATTATATTTTTAGGCAGTCACA	CGGAACCCAAAAGGTGACATACC
2 IDH1	GCTTATGCTACAGTCATACATACCTTCA	CCTTTTTCCCCTCTACACAGTACAG
2 IDH1	CCAGGAACAACAAAATCAGTTGCT	CCCTATTTGTCTAGGTGTCTTCTAGGT
2 IDH1	GACTACAAAACTCCCCTTCCCAA	CATGGTGGCCCAAGCTATGAA
2 IDH1	CCAGATGAAGCCTCCCTCTGAT	GGACAAGCAGATGATATCCCTAACAAAA
2 IDH1	ccagcCAGATTATCCTTTCTGAGT	TCAGTGGCGGTTCTGTGG
2 IDH1	TCTCCTTGCATCTCTACCACAGA	TGAATTGTTCTTTTAGTAGTCACTACGTTGT
2 IDH1	CAGAAAGCACCGATGCTCTGA	CAAATGCTTTGGAAGAAGTCTCTATTGA
2 IDH1	CATGAAGCCAGCCTCAATTGTC	ACTAGGATGATTGGAGTGTTGTGTTTTT
2 IDH1	GACTTTGCACACAAAACACTGAG	CTACCGCATGTACCAGAAAGGA
2 IDH1	GGATTGGTGGACGTCTCCTG	CAACGCTTCATGCTCCACTACTTA
2 IDH1	CCAGAATCATAGGGATAGGGAGATACAT	CCTTTGTATCTGAGCACCAAAAACA
2 IDH1	ACGCCCATCATATTTCTTCAGAATAGTG	TCTCACTGGCTTCTCCTCTACAG
2 IDH1	ACCTGTAGACCTAGTTACCAAAAGACAA	TGCAACGTTCTGACTACTTGAATACAT
2 IDH1	CCTGAGCTAGTTTGATCTTCAAGTTTTC	ATTTCTAGGACTTTACCACTACCTGCTA
2 IDH1	TCATACCTTGCTTAATGGGTGTAGA	CCTATCATCATAGGTCGTCATGCTT
2 LRP1B	GACAAAGTTCAGTAAAATCTTGCACAGT	GCAACCAGAACATAGATTGTGCAG
2 LRP1B	CTGCAGTTCTTTTCATCTGAAGCAT	CCATCTTTGCATTTGTTTATGTACCTGT
2 LRP1B	TTAACATAAACACTGTAGAGCACATGGT	ACTGTGAGGATGGATTCGAAATAACAG
2 LRP1B	CTTACCTTTACAGCTTCTCCCATCTT	TCTCTACAGAACTGTTATCCAATTGCC
2 LRP1B	ACTGACAATTCAGCTGTTGGCA	TCTGGGAGAACAATCAAGACTATAGTGT
2 LRP1B	TGAAACCACCCTAATGCATTTTATGAGT	CCGACCCAATGGCAGTC
2 LRP1B	TGCGACTGCCATTGGGT	GAAGACAGTGATTCCTGTAAACTCCTAA
2 LRP1B	GGTGTGAAACAGAAAGATAAAAAGCCA	GAAAATGATACCATCTACTGGACAGACA
2 LRP1B	CTCTGCTAATTTTATTGAAGCCCATGT	CTGTTGCAATATGATCCTAACACCGATA
2 LRP1B	TGACATGTTAGGACACTTTCCAATGTT	GGGCTTGGCCTAATGGAC
2 LRP1B	CCTTTTCTCAAAGTGGTCCACAGTTA	GAATTCTTTTCTGGACAGACTGGGA
2 LRP1B	CACAGTTCCACATGTATATTCTTGCC	TTCAGTGATGAAGCCCAGATCAATT
2 LRP1B	CCTTCTTTAGTACAATTGATCTGGGCTT	TGTGTTCACTCTTGCTTTGATAATCAGT

2 LRP1B	GCATCTGCCACTGGAACATCT	ATGGTTCAGATTTCACTGCCTCAT
2 LRP1B	TGTATTCTTTCAAGAGTGCTGACCTTT	ACATGCAAACGGGTCTGTCA
2 LRP1B	CATCATTCTTGTGGCCCCTTC	TTTCTTTCCCTTCTGTTGGTCCAT
2 LRP1B	TGAAAATAATGCCCTCAGATGTCCA	GGATTGATGAATGCGGTGACAATT
2 LRP1B	TGGTTCTAAAGCGCTGCTTTCT	CCAGAGCCACAGACATGTACATT
2 LRP1B	TTTACCACCACAGTGATCTTCATCTG	ATGTACTCGAAGGAGCTTCTTTGTTT
2 LRP1B	AATTTTGGAAAAGTGTCAGAAGGCAAT	TGGTGATGGATCTGATGAACCTGA
2 LRP1B	GCTTTCCAACAGCCATGAGCTA	GTATCCAGATAACAAAAGCAGGAGGA
2 LRP1B	TGCCAGTATACTCACGACAGTCA	TCCATTATATATGCACTGGGTTCTTCAAC
2 LRP1B	TTACCCCCTGACTGGGTGA	AGTGGATTGGGTGTCACGTAATTT
2 LRP1B	GATTGTCCATTCATCTGTTAATCCTCCT	ACTCTAAGCTCAGTCAATGGAAATGAAA
2 LRP1B	CGTTTCATCAAATTCTGAGCTAATCCA	AAAATAACTACTTGCAGAGCCAGTACA
2 LRP1B	CCTCCTGCTTTTGTTATCTGGATACATT	TGGAAGTAAAATGGCAACTCTAAGCTC
2 LRP1B	TGAGCTTAGAGTTGCCATTTTACTTCC	AGCTTTCAATAACTAGGGCAAAACTGTA
2 LRP1B	CAGTTGCATTGTTCCACAAATGGA	TTTCAGGTCCCGAGGAGGT
2 LRP1B	aggagtgGATACAGGAAATCAGAAGAT	GAACCTGCATTGTGACTGCTG
2 LRP1B	CAGTGGCAGTAAGGCTGGTT	TCCAATAGGGAGACCCACCTG
2 LRP1B	TGCTATTGTGTTTCTCAATCCAGGAA	CCAAGCATTTCATTACCAGAGTTTACAT
2 LRP1B	AGAAATGAAGCTGTTGCTGGAATG	GTGTGTGACCAGAATTTTCAAGAAAGAA
2 LRP1B	CTTACCTTCTGCTATGCAGGTGTTAT	GGGTATGATGATGCTATTTTGAGCTCTT
2 LRP1B	ACAAAAGAGCTGATGTTTCACCATACA	CTTGTGCATGTCCCACTAACTTCT
2 LRP1B	CAAGTCCTATTATCAGCTGCCAGAT	TCCTGTTTTGTTGTTGTTGTTTCTTTCTT
2 LRP1B	GGTGTCACTAGAAACATCACATGCT	CCATTCTGATGAAGCACCTTTAAACC
2 LRP1B	CTGTACCTGCACTTTTACACTTTGG	AGGATATCGACAGTGTGGTACAGAA
2 LRP1B	CTCACTGCCCATTATCTTAAGACATACT	TCAATGGAGGAATAAATGTAGAAATTGGCA
2 LRP1B	CGCCCATCAGCACAACTAAATTC	GGTGTAGTGTACATTGAGGAGATGAC
2 LRP1B	GTACTCTACTGAACCATGGCCAAATT	CTTTCATAACTGCCTTTTTAAAATTCCAGGT
2 LRP1B	AACTGATTGAGGACAGAAGATACACAAC	TGTTTGTTTCCTAGATTCTTCTTGCTCTT
2 LRP1B	CACTGTTTTCTGCATATTGCTGCT	TCAGTGTATACCAGCAAAATGGAAATGT
2 LRP1B	CATATTTGCAGTCTTCATGGCCATC	TGTCTTTTGAGTGTGTAGAAGTGTTTCT
2 LRP1B	ACTAAGAAATGTGTTGCTTGTTGCTTT	CCCAAGCAATTTGTTTGTGACCAT
2 LRP1B	GCCATCTCCACAGTCGTCATC	GGGAAGATAACATATGTGCTCATCTAAC

2 LRP1B	GAAAAAGCAAATCCAACTATTACTAGAAAACAT	GCCAATGGGAATCATAGCTGTTG
2 LRP1B	ACAGCTATTGGTGTCATTGGCA	TCCTCAGTTTGGCTGTTTATGACAATTA
2 LRP1B	GAATGTTACTGAAAGATGTTAATGCATTTATGG	CAGACAGACTCTCACTGATTTACTCAT
2 LRP1B	CACCTGGATATCTGTGATGGCAT	GGTGATTGCACTAACATTGTTTGAAGA
2 LRP1B	TTCCCCAGTCCGACCAGAATATA	TGAAGCAAAAATAGGTGTGGACGTATAA
2 LRP1B	GTTTTCCCATCAGTCCAGTAGATGTAG	TGTATCTGTCAGCCCATGTAGTATTGA
2 LRP1B	TTTATGACTCATTGCAGCTCGACT	TGGGAGTAGTGGACTATCAAGGAAAAA
2 LRP1B	CTGCCTTGAATGACAGTGTGTCTAT	ATGGGATGAACCGAACAAGGATAATT
2 LRP1B	CTGGCTGCTCTGTCTTTGAATC	TGGCTCCATCAAACGTGCTT
2 LRP1B	TCAACCCCCAATCAATACAAAGCA	GATCAGTTCAGGGAATGGAACCATAAA
2 LRP1B	CCACCATCCAGGTTGCATCTA	CAGTGGTGCTAGGTGGATATTACAATTT
2 LRP1B	AATACTGAAAAGAAATCCAACAGACCTCA	GACAAATGCCCTGTATTGGAAAGG
2 LRP1B	CAACCTTCTCTGAGCCATCCAA	GTAATGTCTTTTTGCATAGCTCTGCAT
2 LRP1B	GCAACACACACAAAATAGTTACTTACCA	GCAATCATCTAAAGTGCAGCCAA
2 LRP1B	CAGAACTAAAAATTGCCATCCTGCTT	CCTCATGAATTCCAGTGTAAAAACAACA
2 LRP1B	GTGCTTGTGCTGCTCACATAC	AGGCTGTAGCAACCACTGTTC
2 LRP1B	GCCAGTGATCGGGAATACAGT	GTTGTGAGGGATGTCTTCATGGAAA
2 LRP1B	GACAATTCCTCTTCCAGGAACAACA	TGTAGTATCCTGGCTAATTGTAGGCTTA
2 LRP1B	GGTAGCTGCTGGCAAATGTTTTAT	CATCTGTCTACTCAGCAGCAGTTA
2 LRP1B	CAGCGACAAGTCCGAGTTTTG	ACTTGAATTTCACGCATGCTGTTTTT
2 LRP1B	ACTCTATACAACCAGGTAGCCTCAA	CTCACTGTGTGTTCTGCCAAC
2 LRP1B	CATTAGACCCGTCGCATATCCA	CCTTCTTCTAGATAACACAGACTGCAC
2 LRP1B	ACTCCAAGCTTATAGAAATGTGTGCAG	CCCTGTTGTGTGGGATAATTACGTAT
2 LRP1B	AGATGTTACTAGGTGTTATATGCCACAGA	CATGGCAAGTTATGTGATGGAGAAAATG
2 LRP1B	CATCAGAGTTGTCTCCGCAGT	TGATCTCTGGTTGTGTGTTTCTACTTTT
2 LRP1B	TGATTTTGTCTCAGACATTACAGCTAAAAGT	CAAGGCCTGGATCAACCAAGAT
2 LRP1B	CTTTCTCTGGGTGCACAGCTA	CCTAGGTAACATATATTGGACAGATCATGGT
2 LRP1B	AAAGATTCTATCCTCTGCCTCTAGCA	GTGATGGAATTCATGACTGTGTGGA
2 LRP1B	CACAGTTCTCTTCATCACTGCCA	CTGCAGTGTCCATTTGTATTTGAACA
2 LRP1B	ACTGAATAACAAACACTTTTAAAGTTGTAACAT	CGATGCATCTGAGGAACGTTTATACTG
2 LRP1B	CCAGTTCCGTTAATAAAAGCTCGTTTAA	GTTCTGAAATCAGAGGAGTGGATATTGA
2 LRP1B	GCCGTGATGAAGTTAAAGTATGGATTG	GCTTGCCAACTTCATAAGAGAAAAGAAT

2 LRP1B	TGAAAGCAAATCTCTCTCTTCAAAGTCC	GCTACCTAATGCAGCCAGACAA
2 LRP1B	CTTACCAATTTTAGCCTTGCAAGATCTG	AGATAAAGCTGCTTAGAAATGATGCCAA
2 LRP1B	CACCTCAATTGCAGTGGATCTCT	CCAAAGAGCAGCAAGTCTGATCAT
2 LRP1B	TTGTGCTGATATGATCAGACTTGCT	GGCTTTGTTATTCGCTGATCTGTTTTG
2 LRP1B	CTTACTTTCAACAATTACTTGTCTGTCTTCC	AGAATGTCATAGCCTTGGCTTTTGA
2 LRP1B	CCAAAGTGTGCATCACTGTAAAAGATT	ATTTAAATTCCCCAATAAGGCCATATGA
2 LRP1B	GGCTATGACATTCTTGAAATAACGTGGA	GCCCATGGATATTTGGCAGAAG
2 LRP1B	GCCTCAGGCAAGTAACTCCAT	AGGGACCAATGTTTGTGCCA
2 LRP1B	CATACATGGGCACAAAATAACCACTT	CTAATAGCAGGAGCCATGGAACA
2 LRP1B	CCCATATCTTGGGTCCAAAGCAAT	AGTCGACTGGATAGCAGGAAAC
2 LRP1B	TGAGAATTTCTGCACACTTACACGTT	TGAAGTTTCCCAGAGACTTGTCTTTAG
2 LRP1B	AGTTTTTACAAACCCAGCTTGAGGAT	ACTTGCTGTCGATTGGATTGGAA
2 LRP1B	TTTTCTGTGTCAGACCAATAGAGGTTTT	GCAATTGCTCACTTCTTATTTTAGGGA
2 LRP1B	GGTCCAGATTGCTGTCTATCCAGTATAT	GCACATGTTGGTTTTCCTTCTTTTGTAG
2 LRP1B	CTTGTCATTCTGCTTCATATTTTAGCGT	CTCTGACCCGCTCTCTTCAG
2 LRP1B	AATTTCAGTGATGTGTGTGGAATTTGG	ATTATTGAATGGAGGCTGCCATGA
2 LRP1B	ACATTATGTTTCTGCACCTAGTTGTGT	tTCCAGGAAGCATTGCCATCA
2 LRP1B	CCATTGGGAGTTAAAAGGCACAA	AGAGATTGAGGCAGTCTAATAGTTTACGA
2 LRP1B	TGACTTTGGAGGGAATTTGACATCTT	CACCCCTATTTGGGCTTCAGATT
2 LRP1B	ACCTTGTTGCTTTCGTGGATCATA	TCATGGAAATTATGTGTTCTGGACTGATT
2 LRP1B	CAATGTCACCTCACTTGTTATCAAATCT	GGTCAGTTCTGATTTTTCACAATGTGT
2 LRP1B	CAACACAACAAGGCTCTGTGAAAA	TCTGGACTTTCACACCAACACATTAT
2 LRP1B	CCTAACCCCAGGATGGTATAATTGGTA	GTGTGATGGAGAGGACGACTG
2 LRP1B	GATCGTAATAGGCATCACACCAGT	GATTGAGAAGGCCTGGATGGAT
2 LRP1B	GGCTTCATCAGAGTTGTCTCCA	GGCACTGATTACTCTGAATCTCTTTGTT
2 LRP1B	TGCAATGTCCATTGGTGTCCAA	GAAGGACCAAAATGTGAGGTTGAC
2 LRP1B	TCACAAAAATCTGCCGATTGAATCC	GGCTGGTTGTAATAACATGTATGCATTT
2 LRP1B	TGTACAAGTACAGGTTTGTCACATTGA	AATTAAGAAAACTCTACTGGACCGATGG
2 LRP1B	ATTGTGTTTCCATCGGTCCAGT	GGACAATTTTGGAGTTTTCTGAAAAGAA
2 LRP1B	CCCATGGCACCTTACACACTT	GCCACCACTATTGTGTGAATTCTGAAT
2 LRP1B	ATTCAACACTTCCATCATCCCCAAT	AAATGAAAGGGTCACCTTAATTATGACTGAA
2 LRP1B	TCCACAACAACACTTGTCATACTTCAA	TGTAATTCCAACGGTTCTGTATGTGTC

2 LRP1B	GAAGCTCCAGATCAATCAGGGT	GCACCTGTGATTCAATGATTTTGTGTAT
2 LRP1B	TCAACTGGCTATTTTGAAATTCCTTGAT	CAGTCTAGGAAAAATTGAAAGGTGTGAA
2 LRP1B	ACATGTCTCTGGGATCCATCGTA	TCATGAGATCTACTCTGACTGGGAAAAA
2 LRP1B	GTACTGACCACCACTTGAGCA	ATAGCTTGGTATTGCCATGTTTCTATT
2 LRP1B	AACACAGAGACAAAACCTTTTCAGGTA	GAGGATCCAGACTGTGCAGATG
2 LRP1B	GCAGTTGGCCTCGTCTGATG	CTCCAAAGCTACATTTTAATGGCAGTTT
2 LRP1B	CTTTGTGAGTTCCTTTTTGTGACCTT	AATGTGGATATGTTTTCAGTTGCAGTC
2 LRP1B	TTTATGTTACTGGACCAACCTTTGTGT	GGAACCAATCAGAGTGTTGTCATAGAAA
2 LRP1B	GACCAGTAGATGTAAGCCCCAAA	AGGAAAATAAATTGTACTGGTGTGATGCT
2 LRP1B	TGTGCGAGCATCACACCA	ACTATGAGGTCTGTTGGATTTCTTTTCA
2 LRP1B	GTTAGTGCCATAGGTCTAGAAATCTTGG	CTCCCAGAGAATCTGTAAACAAAATCAG
2 LRP1B	AGACCTGGCACAAATTCATTAGTGT	GATAAGAAACTGTGGTGGGCAGA
2 LRP1B	CACTTCAACACCATACAAAAGCACA	CCGATTTAATGGGACTGATATTCACTCA
2 LRP1B	GGATTCCCCAAGCATTCTCAATTTTAAT	GTTTTGCCCTCTTTATAGGTTAGACATCT
2 LRP1B	GCCCAGGAACTAGGAAAGCAATG	TGGTGAAAGGGACTGTCCAGAT
2 LRP1B	ACACATTGAACACTCATTACAGAGACAA	GGAGAACCCTATGCTATTGCAGTAAAT
2 LRP1B	CTAAGCCATTAACCTACCCTCTTTTAGG	CTCACTGGACCAGTCTGAGGTA
2 LRP1B	TGTGGAAAGCTCATCGCTTCC	TGGACACAGAGTAATCTTAATGGCATT
2 LRP1B	CAGCTGCCCTACGTTGATAGAG	TGGTGTGTTGTTTTGTTTCCTTGTAG
2 LRP1B	AGAAAGAGGTAATGCTGATGCCAAA	TGAAAGGAAAGAACTGCTTCCAAAGAA
2 LRP1B	GCCACTGTCTCTCTTATACCAATTTCTAT	ACAAATTACTCCAATCCGGTATATGCAA
2 LRP1B	TCGACAGTTTTGCCCATCCATATATAATT	CGATTAGGAGATGGACATTCTAGGAAGT
2 LRP1B	TGCTGCTTTCTTATTTCTCATCTTGACT	GCTTTGATGCCTATATCAGGAACTTCAT
2 LRP1B	ATGGAAATCTATTCCCACGGCAA	TTATCTCCGGCCCTGCATTTAG
2 LRP1B	GTAGACAAGGTTCAATGCTAAAAGCTAC	GATTGTCCTGATGGCTCTGATGAA
2 LRP1B	ACATGCAATACCACAGAGATAGCC	GATTGCGAAGATCAGTCAGATGAAGAT
2 LRP1B	TCCACACAAGAAACTGTCACAGTC	ACTGAAGACAATAATGGCCACTCAT
2 LRP1B	CAGCTGACAATGTTTTGTATAAAATCCCT	GATTGTGAGGATGGACGTGATGAAT
2 LRP1B	ACAGTGGAATTCATCACGTCCAT	CACAGTTCAAAACAAACACAAATGTGA
2 LRP1B	AATGCATCTTCCACTAGGACAACTAAAA	AGCTTACTTGTTATTTATCTCTTTCAGTTTAGG
2 LRP1B	GAGAGAGAGAGAGAAAATTTGTGTCTGA	CTGTGATGGAGAGAATGATTGTGGA

2 LRP1B	CCACAGTTGAGTTCATCAGAATTGTC	ACCATTCCACAATATCTATGCTTTCTCC
2 LRP1B	GAAGTCTTGGGAATCTTCATAACCCA	GTGAATCCATGTGGAGATGATGCATAT
2 LRP1B	AAATCCAGGCTTACAGCGACA	AGTTAACTCCCCACTTCAGGAATTAGA
2 LRP1B	AGCTGCAAGGACTTAAGTTTCACA	GTTTCTCCTCGCCTTACTCACTC
2 LRP1B	GCAATCGGCAATAATCCCGAGA	TGTAAATGACGCCGCTCGAT
2 LRP1B	GAAGACAAAAGCACTTCACAAACCTT	TGTCTTGTGTGTGTTGATTTTGCAG
2 LRP1B	TGCATACCCATTAACTGGATCCAC	GACCAATGATGGCCATAGGAAAAC
2 LRP1B	GATGAGACATTTCACCCTCTAAAAGAGT	CTGGATAATGTAGAGGGCATTGCT
2 LRP1B	AACTTGAGTTCTGGTTAAAGCTAGTCTG	CCTAATTGGCCGGCAGAAGAT
2 LRP1B	GAAATTTACCATAGCAGGTCTTCTTGTCT	GTGCAGCTAATGATGGCAAAGG
2 LRP1B	GGTTTCTCTCTCTGTGCCATCT	GCTGATGAATACATGATCCCCATAGAAAA
2 LRP1B	TGATTGATTAGACACATGTGAGAGCAG	TGAATAACCCCTGTCAACCTCAGA
2 LRP1B	CTAAAGCACGAGGGTTTACCAGA	GCAGCATCAGTTGTTTGTGTTTGTAAT
2 LRP1B	GGAGTCAAAGTTAAGACAAGACAGAGT	CCAAGAACCAGAAATGTATCCCAGTAA
2 LRP1B	GAAATGATTGCATTCAATGTACATATCTAACA	GGAGAATATGATTGTGCTGATGGTTCA
2 LRP1B	CAGTCATCTTGCCCATTACATCTTAAGT	CACAAATTTTCTCTCTCTCTCTCCTTCT
2 LRP1B	CCTTTAGTTTCTTCTGCTTCTAGTCTTCA	GTGCTTGTGCCGATAATCAACTTTT
2 LRP1B	GTGCAAGTTGTCCCATTTTCATCC	TCTGACACATTTGCTTTCTTCCTCTT
2 LRP1B	CCAGAAAAGGAGAATAATAGAACAGGGA	GCTGTCAGTTATGTGATGGCTACTG
2 LRP1B	TGGCATGTGCCACCATTGTAA	AGCTTTCAGCAATAATGTTGCCAATAA
2 LRP1B	AAATTATCCACGTAAACCACACCGA	TGATGGCATTCCTCACTGTAAAGATAAA
2 LRP1B	CCACAGTAGAGCAGTTTTTCATCTGA	TTGCCATTGCACAAAAGTATTGACTT
2 LRP1B	CCTAGAACTGATGGTACGCAAGT	CTTGTGAAAATGGAGGAAGATGCATT
2 LRP1B	CCATTCTGGCAGTAGTTGCTACA	AGTTAACTTGTGAAAATGGAGGAAGAT
2 LRP1B	TGCATCTTCCTCCATTTTCACAAGTTAA	GGGTTAATTATTTTATGGAAACACAAGATCC
2 LRP1B	TCAGATTGTCACAAACTGCCTTGAT	CTGTATCCAAGCTCGGTGGAAA
2 LRP1B	CAGTCATCGTCGCCATCACA	TGGACTGACTGTAGCTAGCATTATTCATA
2 LRP1B	TGAAGAGCAATATTTCCATCCAGAACA	GATCTCCAGTGTGATCGACTTGA
2 LRP1B	CTGAACCATCTCCGCAGTCA	AGAGTGTTTTGATTCTGTGAAGCACTAA
2 LRP1B	GTGCTATACAGTGAGCATTTGGTACT	CGAATTATGTCATGCACAGTCAATAACA
2 LRP1B	AGGATCCATATGTATAACTGAGGTTGGA	CCAGCAATGCATCAATACATACGG
2 LRP1B	CTGTACAGAGGCACTTGTAAGTC	GCTCATTTTTCTTTTCCCAGTGCAA

2 LRP1B	CGTCATCCTTCAGTTGGAATCCAG	AAATGCTGGGAGACAGAAAGACTT
2 LRP1B	GTGAAGTAAATGAACCAGAGGTAAGGT	GCAATTCCTGCCAACTAAACAATGG
2 LRP1B	TGGTAAACAAAGTTGAGAGCATCCA	GAAACAGCTGCATTTAGAAATCAATAGCA
2 LRP1B	CACTGTTTCTTACTTTTGACATTCATCCA	AGCTCTACCACCTCATCCATCA
2 LRP1B	GTCTGGTCCACAGTGTGTCTG	TTTTTCTTTTTCTTCCCCCACTAGATGT
2 LRP1B	GCAAGTCCTTCCACAGAACCC	GAGATTTTGTGGAGAAATGGTTGATGAA
2 LRP1B	CAGGTAGTTCTACAGCTCTAACACATAC	GTGTTGGTGTTTAGATGAAGAACCTTTT
2 LRP1B	GCCATCAGTGCTAATTTTCCTTATCTCAT	ACCAGATCAGCACTGCATTTGAA
2 LRP1B	GATACAGATTCAGCCTGAGCCA	GAGAAAAAGACTGTGAAGATGGTAGTGA
2 LRP1B	CTTACAATCAAGCCACTGTTTGCT	TGGCGATCATCAACAAATTTCTCATATTG
2 LRP1B	GCCTGGATTAAACTGAGTACTCCAAATA	GCTAATGACACTGATATCCTGGGTTT
2 LRP1B	CTTTCTGGGTGGTTAATCCATAAGTGA	CAATAAGTGGACAGGGCAGAATG
2 LRP1B	TGGTACCATTGCAACCTTTTTCA	TCTGCTTCTCTCTTCCTCATTTCAAATT
2 LRP1B	GCACTGGTTTTCTGAATCACACTGA	GTCATGAATACCTTTCCCATCCCTT
2 LRP1B	TCACTCCCATATAGAGACACAGCA	GTCTCTGGACTAGTTGAATGAGTTTTGA
2 LRP1B	ATTCAAGAACCACTGCTATCAAGGT	GGAAAATATTTGATTAATGGCACCTGCAA
2 LRP1B	CCACCTAACAGGCTGTCATCA	CTTTTGTTTCACCAGTACCCAATCC
2 LRP1B	ATCCGAATGGCATGAGAACCTTT	GTGATCCTGGTGAATTTCTTTGCC
2 LRP1B	GAGACACAAGTCACGTGATCGT	CATTTCATGTTTGTACTGCTCGTTCTG
2 LRP1B	TCGACACTCTCCATTCAATTCGTTT	GGATGTTCTCAAGACTGTCAAGACC
2 LRP1B	GAGTACCTACCTTATAACTGACCGGAA	AGGTCTTTGCGACAATAAGGATGAC
2 LRP1B	CTCATCTGAACCATCGCCACA	GGCATGCCCTTTATGTTCCATTT
2 LRP1B	GCCATATCTTCTGCCTCTTTTACCT	TGACCAGACAGATGAAATGGCATC
2 LRP1B	CATGTCTCACTCACAACTTACCACAA	TGATCATCATAGCCAGAACATGCC
2 LRP1B	GAGGAGATTTACGTGTATATCCAAGCA	CCCTCCCTCCCAGTCAATCA
2 LRP1B	GCATTTAAACTGATCATCAGGACAGCTA	GGCAATGAATGACAGACTCTATGACAT
2 MSH2	CTCACGCTTCCCCAAATTTCTT	GCGATTCTCCAATATACTGAAACTCCTC
2 MSH2	GCTAAACAGAAAGCCCTGGAACTT	ACTATGAAAACAAACTGACAAACCTCTCT
2 MSH2	TTCATGGCGTAGTAAGGTTTTCACT	CCACTGGTTAACAAGTCTTTGTCCT
2 MSH2	TTGCTGAATAAGTGTAAAACCCCTCA	GTATAATCATGTGGGTAACTGCAGGTTA
2 MSH2	TCTTGCACATTGTATAAACATTTAATGTAGGT	CTGTGATCAGAATTCCTCCTCTTTGAA
2 MSH2	ATTCAAAGAGGAGGAATTCTGATCACAG	TCCATTTCTGGCAATACAGCACTAT

2 MSH2	CAAAGAGGAGGAATTCTGATCACAGA	GTACCTGATTCTCCATTTCTGGCA
2 MSH2	AGTGCTGTATTGCCAGAAATGGA	CACAGTTTAGGTTTTGAGATAAATATGACAGA
2 MSH2	ACAGGCTATGTAGAACCAATGCAG	TTTCTCCAAAATGGCTGGTCGTA
2 MSH2	TCAGCTTTGCTCACGTGTCAA	TGCAATTTCATCTTGAACTTCAACACAA
2 MSH2	GGGTCGCGCATTTTCTTCAA	GTCGAAAAGGCGCACTGT
2 MSH2	GCTTCGTGCGCTTCTTTCAG	CCGGCCCCATGTACTTGAT
2 MSH2	CCCGGGAGGTGTTCAAGAC	CACTCTCTGAGGCGGGAAA
2 MSH2	TTTAAGGAGCAAAGAATCTGCAGAGT	GCCAAATACCAATCATTCTCCTTGGAT
2 MSH2	GATGGAAATGAAACAATTTGTCACTGTCT	TGTTTCACCTTGGACAGGAACTC
2 MSH2	TCAGGAGTTCCTGTCCAAGGT	ATTCCATTACTGGGATTTTTCACGTAGT
2 MSH2	ATGCCCTTTACTGAAATGTCAGAAGAA	CCTTCATTCCATTACTGGGATTTTTCAC
2 MSH2	CTTAGGCTTCTCCTGGCAATctc	GAATTCACACAGTCCTAGTTTCCTCT
2 MSH2	TGGAGTTGGGTATGTGGATTCCATA	TCCTCCGGGTAAAACACATTCC
2 MSH2	CTCCTCATCCAGATTGGACCAAA	CCTTTCCTAGGCCTGGAATCTC
2 MSH2	TGTTTCACGTAGTACACATTGCTTCT	TGACAATTTCTTTAACAATGGCATCCTG
2 MSH2	AACAGAATATGAAGAAGCCCAGGATG	CTTGCGAAAATAACAAAACACACTTCAA
2 MSH2	GCTGATTTAGTTGAGACTTACGTGCTT	GGGAATCGACGAAGTAAATCTTCTTGTA
2 MSH2	AAGATGCAGAATTGAGGCAGACTT	AGTTGATTTATACCCTGATAGAGTCGGT
2 MSH2	GACAAGCAGCAAACTTACAAGATTGTT	GGACAGCACATTGCCAAGTATATATTGT
2 MSH2	AACACCAAGGAAAATGAGGGACTT	AGTTCTAAAAACTTGATTACCGCAGACA
2 MSH2	TTTAGGTTGCAGTTTCATCACTGTCT	GCTCTGACTGCTGCAATATCCAATT
2 MSH2	TCACTGTCTGCGGTAATCAAGTTTT	ACCTGAAAAAGGTTAAGGGCTCTG
2 MSH2	TGAAATTGGATATTGCAGCAGTCAGA	GCTCCTTTATAAGCTTCTTCAGTATATGTCA
2 MSH2	TGTTTGTGGCATATCCTTCCCAAT	GAAGTTCCTCTTCCCAATTCATCTATGA
2 MSH2	TCAGGTCTGCAACCAAAGATTCATTAA	GCAAACATGCAAAAAGCACCAATC
2 MSH2	TGGGCTATATCAGAATACATTGCAACAA	GGTGAGTGCTGTGACATGTAGAT
2 MSH2	TTGGCCAATCAGATACCAACTGTTAATA	GGTAGTAAGTTTCCCATTACCAAGTTCT
2 MSH2	CTATGTCAGTGTAAACCTACGCGAT	CACCCCAGTTTGTCGAATATATGTTGA
2 MSH2	AGGCCCCAATATGGGAGGTAAA	GGGCTAAGATGCAGTCCACAAT
2 MSH2	ATGTGAGTCAGCAGAAGTGTCC	TCAAGGGACTAGGAGATGCACTTA
2 MSH2	GTCACTTTGTTCTGTTTGCAGGT	TCCAACCTCCAATGACCCATTC
2 MSH2	GGCTTGGACCCTGGCAA	CGACTTGCAAACCTGTTGGT
2 MSH2	GTACCTTTTGGATCAAATGATGCTTGT	TGGAGAAGTCAGAACGAAGATCAGT
2 MSH2	TCTTCGTTCTGACTTCTCCAAGTTTC	TGTCCACAAAGGTGCTACAATTAGAT

2 MSH6	CCAACGTACATGTGATTGTGAAAG	AACCCATCTGGGCCATTACAG
2 MSH6	CCTCATTCACAGGCTGGCTTATTAG	ACTGAATGAGAACTTAAGTGGGAAACAAA
2 MSH6	GGGAAGGGATGATGCACTATG	AACTCAAAGCTTCCAATGTAGTCAGT
2 MSH6	TGAGAGGGCACTTCTCTTGCTA	GCTTTTAGGACAAGCTCCCTTAATGAAT
2 MSH6	GCCAGGAGACTATTACGTTCCTCTATAA	TCTCTTGCTTTTCTATGTCCCTTTTGAA
2 MSH6	CTTGCTAATCTCCCAGAGGAAGTTA	CATCATCCCTTCCCCTTTTACTGTT
2 MSH6	GTTCAAAATCAAAGGAAGCCCAGA	CAAACTGCCAATTCAAGCCTCTTAA
2 MSH6	TGCAGATGAAGCCTTAAATAAAGACAAGA	CCCCATCACCCTAACATAAATAACAACT
2 MSH6	AGGTAACTGCCTTTAAGGAAACTTGAC	TTCTCGCGGATGAATGTTCCA
2 MSH6	TGGTTTACAACCACCCCTTTGA	acacaTGGCAGTAGTGACTCTTAC
2 MSH6	AATCTTTTATACCAATATGTGTAGCTCATGAT	GTGCTGTTGCATGCATGAGTATG
2 MSH6	CAGCATACTCATGCATGCAACAG	TGCGTGCTCTAAAAACATTCATATTGTT
2 MSH6	GAAAAAGAGGAAGATTCTTCTGGCCATA	GCCACTAGAGTCCTAAATCTCGAACA
2 MSH6	GGTCAGTTTTCAGATGATCGCCAT	AAAACTGGGAGCCGGGTATC
2 MSH6	AGTTCATTGTCCTGTTCTCTTCAGG	CCCAATGCCATCACTTAGCTTTT
2 MSH6	CTTTGAGAACTCTCCTTGAGGAAGAAT	AGGTAGAAGACACAACCACCTAGAG
2 MSH6	GAGAAAAGTGAATTGGCCCTCTCT	TGATAGGCTTTGGTGAAGATAGCAC
2 MSH6	CCAAACGATGAAGCCTCACTTT	CGTGATCCTTTAAGCTCTAAGAAGGG
2 MSH6	ATTCTGTTGCCGGAAGATACCC	TTGGTCCAGTAACAAGCACACA
2 MSH6	GCAGGAAAATGGCAAAGCCTAT	ACTGTGTTTGGAAAATGATCACCTAAGTA
2 MSH6	GCGGTAGATGCGGTGCTTT	CTGGCCGAGGCCTTGTT
2 MSH6	CGCTGAGTGATGCCAACA	CCGTTGAGGTTCTTCGCCTT
2 MSH6	TGCTTTTAGACGTGGATGTACTAACC	TTGCTATTGCCGTCCCATCAA
2 MSH6	CTTTAACAGGAAGAGGTACTGCAACAT	CGCACAGCAACATTTTGAGAATAATCTT
2 MSH6	GCAGTTGTTAAAGAACTTGCTGAGACTA	GCAAGAGAAGTGCCCTCTCAA
2 MSH6	ATCCCAAGCCCACGTTAGTG	AATTGAGGAAATCCTCAGGCACATAG
2 MSH6	TTTCCTTGCCTGGCAGGT	CTCAGAATCTGATATGACCCTTCGTTT
2 MSH6	ACACAGTCAGCACTACAAGATCTG	CTCTCTAGTAGGGTTCCTTCAGTAGAA
2 MSH6	GCTAATCTCATAAATGCTGAAGAACGG	CAGCACTACTTATCAAAGCCTGAGAA
2 MSH6	CCCCGATTTTGATGCATCTACACT	CACTGACTCCAATAAGAGCATCCA
2 MSH6	GCTCTGGAAGGATTCAAAGTAATGTGTAA	GGCTGTATCCCATCGGTTCA
2 MSH6	CAACTTGGAGATTTTTCTGAATGGAACA	GTCTTCTATGGCATCTAGACGATCATT
2 MSH6	GCGAAGTAGCCGCCAAATAAAA	ACTCTCACTATCCCCCACTCC

2 MSH6	CCCCACTCTGTAACCATTATGCTATT	TGAGTAGCCTCTCAAGATCTGGAAG
2 MSH6	CTCCGAAGTTGTAGAGCTTCTAAAGAA	TCTTCATACATTATAGCCCTGCTGTCT
2 MSH6	GGTCGTTTTCCTGATTTGACTGTAGAAT	GGAGGCTCTGTTCATTTTCTCTTATGTC
2 MSH6	GGGAAATTTTATGAGCTGTACCACA	GTCTGTTCCACTCGTGCTACT
2 MSH6	TCCCCTGAAGAGTCAGAACCA	TCAGCAACTTCTTCCATGATCCCTATA
2 MSH6	AGGAAGCAGTGATGAAATAAGCAGT	CGTTTCCTTCCTAGAGCTTTTCCTTTT
2 MSH6	GGTGACTGGAAATGGCTCTCTT	CGACTACTGTCATCACCACCTC
2 MSH6	CTCTGATTATGACCAAGCTCTTGCT	GGCAAATTGCGAGTGGTGAAAT
2 MSH6	CCTGGTGCAGAAGGGCTATAA	GTGTACCCTTGGTAATGATCCTACAG
2 MSH6	GTTACCAGCTGGAAATTCCTGAGA	GCAGTCCTTCAATGATACATCCCT
2 MSH6	GATAGAGTGGTGAGGAGGGAGAT	GCACACACCATATGCACGAG
2 MYCN	CCCCCAACCCGGTCATC	ggACTGGGCGGTGGAAC
2 MYCN	GGTTCCACCGCCCAGTc	GCGCTTGTTCACGGGAAA
2 MYCN	GACCACAAGGCCCTCAGTA	AGACATACGAGCACTAACAAAGGG
2 MYCN	TTCATTCAAATGGTTCTCACATGAGAGT	TTCTTGGGACGCACAGTGAT
2 MYCN	ACCAAGGCTGTCACCACATTC	ATAGTTGTGCTGCTGGTGGAT
2 MYCN	TGATCCTCAAACGATGCCTTCC	GGGCTCAAGCTCTTAGCCTTT
2 MYCN	CGCCAAGGTGGTCATTTTGAAA	ACTGTCCAGTTTTGAGAAGCGT
2 MYCN	CGTCCGCTCAAGAGTGTCAT	CGTGAGAAAGCTGGACCGAAG
2 MYCN	CGTCGCAGAAACCACAACATC	GTGGACATACTCAGTGGCCTT
2 MYCN	GCGGAAAGAAGCCCTCAGTC	GCCGCCGAAGTAGAAGTCATC
2 MYCN	CTGCTTCTACCCGGACGAA	CTCAAGCAGCATCTCCGTGA
2 MYCN	GGAGGACATCTGGAAGAAGTTTGA	CCACAGCTCGTTCTCAAGCA
2 MYCN	GAGCTGGGTCACGGAGATG	TCCACATGCAGTCCTGGAG
2 NCOA1	CTCTATCCTTCAATTTCCCCGTCTT	CCTGTAGTATCTTGCTTGGTCATAAAGG
2 NCOA1	CCTCCAGCTATTACGGGTGTAGAAT	GCAAAGCCTAAATCTCATAGAACAGAAAG
2 NCOA1	CTTCAGAGTTTGTGCTTTTTCACCAT	GGTGAATTAGCATCCTGCAGTTAAAG
2 NCOA1	GCAACACGACGAAATAGCCATAC	GATTTTGGCTGTGACACAGTGAA
2 NCOA1	CAGCGTTATGAAGTAATGCAGTGT	CCTCTCTTTTCCTTAAAGTCACAGAAGA
2 NCOA1	CAACCCTATAGGAGGCGTGA	GTGGAAACTCACTAGAGCATTCCT
2 NCOA1	GTGTAAGCATAGATACTTGATCAGAGAAGG	TGGCAGACAGTAACTCAGCTAGT
2 NCOA1	CTTTGTGTAACAGCACGGAAAAGA	TTGCATTTGTCTGGTTTTACACTCAAG
2 NCOA1	TGCCAACATTAGTGACATTGACAG	CACACAGCCATGGGTTTTCTTTT
2 NCOA1	CCTTGTTCATGATCAGTGGATGTAATCT	GCCATCATTGGGTTCATGTTCTGAA
2 NCOA1	CATGGCAGGTGGAAATACGAATG	TCAGAATTAACTGTGATGTGGTGTGG

2 NCOA1	GCTTTTCATTTTTGCTGTGCTCCTAT	GAGAGCTGGTTGCAGTAGAGG
2 NCOA1	ATGATCCCGCACTGAGACAC	GTACATTTGACAGCTCTGGGCTTA
2 NCOA1	TCTAACCAAACCTGCAACACTAAAACT	CAGTTGCTGCAAACTGGTTTAAGA
2 NCOA1	TGATACACCAAAATCGGCAAGCTA	ACTAATGACGTCTGGTTTTCTCACATT
2 NCOA1	GTGCTTTTTGATGCTTTTGTTTATTGAG	CCAGCAGCTCTCAGGGA
2 NCOA1	GGGAAGATTTAGTGAGGAAGTGCATTTA	AGATAAGGGTTAATGAAAATAATTGAGAGAGAA
2 NCOA1	TCTACGTTGTTTCAGGTGAAAGTGT	ACCATCCATCCTGTCTGTCTCA
2 NCOA1	GCACAGTTGCCAGGCTTATG	TTTAAAATGAGGTGTTGTCCCCAGAA
2 NCOA1	GTGTCAATTTACAGGAATGGTTCCC	GCTGCCAGGCCTTCATGT
2 NCOA1	TCAGAGTTCTCTTCTCCAGCAAAC	GGATTTATCACAGTATCAAGGATGCTCA
2 NCOA1	TGCTATGTCAGTCTGAAACTTTCTTGT	GCTGTTCAAGAAGAGCCTTCTCA
2 NCOA1	CCACAACAGTAGAAGGGAGAAATGA	GGGCTCCATCAGTTAAATAATGTTGACT
2 NCOA1	GGTAGATTTCTTACTGTTGTCCTTCCT	TGCCAAAAATAGACTAAGAGGAGTGTTC
2 NCOA1	TTGAAATTTGGTGGATCTATGTCTTCGT	GACTACTTGATGAGATGTCTGATTTCTGT
2 NCOA1	AGAGAAATCAACAACTGATGACGATGT	CACTTTCTGAGTCAATGAAGACACCTA
2 NCOA1	CCCCAAGCAAGTAGGCCTTAA	TCTGTCGGTGCTGTTGACTG
2 NCOA1	CAACGTCAGCGGGAACTGTA	CCCCATTTGAACTGGTAGTCCA
2 NCOA1	GAACAACCTCCCTCCCTCATC	ATGCTTCAGGATTTGCTGCTAGT
2 NCOA1	TTCTACCAGCCCGTTTTCACA	GCTGCATCTGAGGATTGATTCCAG
2 NCOA1	GAAACATAGGAGGACAGTTTGGCA	AGAGAGACCTAAGCCTGTATGGATTAC
2 NCOA1	CGGAAGCATGTTACTTATTGGCAGAA	GGCTGGGAATAAAGGTTGGGT
2 NCOA1	GCCACCTTTGATCATGGAAGAAA	CCATGCCAGGTGAAAAAGCAC
2 NCOA1	GACGATGCCTGTTCAAGTAACAC	aGTATCAACTGCTAAACAAGAGCACTT
2 NCOA1	AGAAGCTCTTTACTTGATAGCTTGCAAT	CCAGAATTAGTGTCATCTTGAGGAGAAA
2 NCOA1	CCCTAGGGAGCACAGTGG	CGGTTTATTCTGGTGGATACCATGTT
2 NCOA1	GGCATGACAAGTAGTGCCTGTAATAATA	GCTAGGTGAATTCTGTGAGCTCATC
2 NCOA1	CACATTGCCACCATCCAACAG	CTTGGTTTAAGGCAACATTGGCTA
2 NCOA1	CTTCTCTGCCAGTTCTCCAGTC	GAATCCAGAGGTTTGCCATCACT
2 NCOA1	GCTCACCCGGAAGTCAGATT	TTGCCAATCCAGAAGTAACCTGT
2 NCOA1	TGAAATGATTCAATCTGACAACAGCTCT	GCATGCCGTAACTGCTGTTC
2 NCOA1	GCACAGTTCATGTCTCCAAGGA	TTACTGGGATGTTTGAATAAGATCGGTT
2 NCOA1	CTAGTGCAGCTTTTGACAACAACT	TTTATGCCGTTCTGTCAATGAGCTA
2 NCOA1	GGAGGTTCTTGTCCCTCTTCTCA	TTTCCTTGTACCTGTCCAGTCAC

2 NCOA1	GGAGGGCCTTTAAAATGAGGTTCT	GCTAAGCATTGTCCCATCATTCAATATG
2 NCOA1	CCTCCAGCCCCTCCTATAGATT	CTGTCGATGATATGAATTCCCATGATGA
2 NCOA1	TCAAAGTCCAGACATGCAACCTT	AATTTCTGCTTTCTAGCCATCAAGAGTAT
2 NCOA1	TCCTCCACTGAGATAATGACTTCTTACC	GCTGTCACTGTATTATTTGTCCATGGAA
2 NCOA1	CCAGGAACAGGCGATCAGATT	CCACTGTAGACTGAAATGTAGTTTGGA
2 NCOA1	CTCTAGTGAGTTTCCACTTCAAGTAGT	GACCAGGCTGGTTCAGGTAAG
2 NCOA1	CTGACGTCCAGTGTACAGTGAA	GGTTATTCAGTCAGTAGCTGCTGAAG
2 NCOA1	CCAGCAGAAGAGCCTCCTT	GAAGAATGGCTGCAGATTGAGATATCA
2 NCOA1	TCTTTTGGTTTTGCAGGCTTTGG	GTATGCTGTAGACGCTCGTATTCATT
2 NCOA1	GCTACTTAGGTTACAATCAGGAGGAATT	atataaGGAGAAGATACAGAGAGCTAGTATTC
2 NCOA1	GACAGTGCATCTACTTCTGTGTCA	GCTCAGGTTTGGAGTTGATCTTAAATCT
2 NCOA1	CATCAGCTCCTACGCTATCTTTTAGAT	GGGTTGGGTTTGTATTACATGGA
2 NCOA1	TGAAAGTGGAAAAGAAAGAACAGATGGA	TCTGTGAAGCAAGCAGTTACCC
2 NFE2L2	AGCACCCTCCAATCCTTCCTA	GTATGCAACAGGACATTGAGCAAG
2 NFE2L2	AGGAATGGATAATAGCTCCTCCCAAA	CCCAGTCTTCATTGCTACTAATCAGG
2 NFE2L2	CCTCAAAAGCATTAAGAAAATGTGGACT	TGAAAATGACAAGCTGGTTGAGACTA
2 NFE2L2	GGCTTCTGGACTTGGAACCAT	AGAGATAAGCCTGAAGATAATGTGGGT
2 NFE2L2	CAACAGAAGTTTCAGGTGACTGAG	CCTGTTGGTGGAAGACTCATAAATCA
2 NFE2L2	GCATTTCACATCACAGTAGGAGCTT	CAAACAAGAGATGGCAATGTTTTCC
2 NFE2L2	TCTGGCTTCTTACTTTTGGGAACAA	TGACAAAAGCCTTCACCTACTGAAAAA
2 NFE2L2	CTTCGAGATATAAGGTGCTGAGTTGT	CTCAGAATTGCAGAAAAAGAAAACTGGA
2 NFE2L2	TGAAGGCTTTTGTCATTTTCTCCTTTTT	CTTGCATTAATTCGGGATATACGTAGGA
2 NFE2L2	TGAGCAGCCACTTTATTCTTACCC	TCTCCATATCCCATTCCCTGTAGAAAAA
2 NFE2L2	TTGAAGTCAACAACAGGGAGGTTAAT	CACACCAGAGAAAGAATTGCCTGTA
2 NFE2L2	TTAAACCTGCCATAACTTTCCCAAGA	AGTTACAACTAGATGAAGAGACAGGTGA
2 NFE2L2	GGCTGGCTGAATTGGGAGAAAT	GGAAAGAGTATGAGCTGGAAAAACAGAA
2 NFE2L2	TGGAGTTGTTCTTGTCTTTCCTTTTCAA	TTTGATTGACATACTTTGGAGGCAAGATA
2 NFE2L2	GTCAAATACTTCTCGACTTACTCCAAGAT	CCCACTTCCCACCATCAACAG
2 NFE2L2	GGTTTTCCGATGACCAGGACT	AGATAGTGCCCCTGGAAGTGT
2 NFE2L2	CTGGTGTTTTAGGACCATTCTGTTTG	TCAATGATTCTGACTCCGGCATTT
2 NFE2L2	CACACTGGGACTTGTGTTTAGTG	CCTCACCTGCTACTTTAAGCCAT
2 NFE2L2	GCCCATTTAGAAGTTCAGAGAGTGA	CAACCAGTTGACAGTGAACTCATTAAA

2 NFE2L2	TGTGTTGACTGTGGCATCTGAA	CTCAATGGAAAAAGAAGTAGGTAACTGT
2 NFE2L2	GAGATTCATTGACGGGACTTACATAGA	CCACATTCCCAAATCAGATGCTTT
2 NFE2L2	GCAGTCATCAAAGTACAAAGCATCTGA	ACATCAATTATTGAATATTTAGCTTGGCAATGG
2 NFE2L2	CCGCGGTTCCCTAGCTC	ACACACGGTCCACAGCTC
2 PAX3	ATGGTGTCAGTACTGCAGAAGG	TCTTACCAGCCCACATCTATTCCA
2 PAX3	CTTGACTCTTCCTCGGTACCTTG	CAACCGCCGTGCAAGATG
2 PAX3	CCCAGCTTGCTTCCTCCAT	CAGTAAGAAGCCTCTAATCTGTTTTAGCA
2 PAX3	CATGTGTGGCTTAATCTTGCCTCTA	GCTACAGTATGGACCCTGTCACA
2 PAX3	CTTTGTCCATACTGCCCATATTGGT	GAACCTACCACCACGGTGTC
2 PAX3	GCTCTTCATATGGTCTAGTCTCTGACTG	CCTCTTCCCAATCTGGCTGTTTT
2 PAX3	GGGAGAGGTTAATGGGCCTAGT	TGCCCGCCTGTTCTCTTAAAG
2 PAX3	ACTCATTGGAGAGCCCCAGAT	CAAGATCCTGTGCAGGTACCAG
2 PAX3	AGGACGTATGGAGCCAGTCT	CGGCGGTGTTTTTATCAACGG
2 PAX3	ATCTTGTGGCGGATGTGGTT	GCGAAATTACGTGCTGCTGTTC
2 PAX3	TCTGTCTAGAAACACGGGACTGA	GCACGATTCCTTCCAACCCA
2 PAX3	CAGTAGGCAGAGCTGCTGTC	AACTTTCTCTGCTGGCCTAAAAGAA
2 PAX3	CGCTGAGGCCCTCCCTTA	TGGATATAATTTCCGAGCGAAGCTG
2 PAX3	CAGATTCTTCATATCTAGGCTGCGA	AGCTGCGTGTGTTTCCTTACA
2 PAX3	TCCAAGTCACCCAGCAAGTG	AGAAGTAAATTCGGGAAAGGTGAAGAG
2 PAX3	TGTTTGGCCTTCTTCTCGCTTT	CCCTGCTTGTCTCAACCATGT
2 PAX3	AACTTCAAACAAATCAAACCAGTCAACA	AGTTGAAGTAGCTTTTATGCCCTCAG
2 PAX3	CTGGTCTTTCAGTTCCATGTCGT	TCCTGAGCGAGCGAGGTAA
2 PAX3	CAGCACTAAAGAATTGGGATGTTTTGA	ACAAAGGAGAGTGATTGATTTCTTCCT
2 PAX3	TTGTTTGTTTCCTGTCTGGACTGA	CTTCACAGCAGAACAGCTGGA
2 PAX3	TCTCAAAAGCACGCTCCAGTT	TGTTTTGTTCTTCTGTGCTTTTAGCC
2 PAX8	AACTTCCAGCTGCTTTGATGGA	CGCTCGAGTGCCCATTTGA
2 PAX8	CACACCTTCCGCCTGACA	CCCTGACCCCTTCCAACAC
2 PAX8	GGTCTGGTGAGTCGAGAGGTT	GGTCGGCTTGTTGGATCGT
2 PAX8	GGCATAGGCCTCTGGGTAGT	GGGTACCAGGTGATCAGGATAG
2 PAX8	TGCTCTGTGAGTCAATGCTTAGTC	GTGAGTCAGCTTGGAGTCAGT
2 PAX8	ACCAGAGGCTGCTTTCTCTCTTA	CAAGGTGGTGGAGAAGATTGGG
2 PAX8	AGGGTTCTGGCGTTTGTAGTC	GCTTATTGGGTAATTCTTTGGGATTCTCT
2 PAX8	CCCTGAGATCAGCTGGAGAAGT	CCAGCGCATCGTAGACCTG
2 PAX8	GATGTCGCAGGGCCTTACA	CCACCCAAACTCCTACCTGAC
2 PAX8	CGGGACCTCCCTGTCGTA	GCTCCACCCCTTCCTCTTTATC
2 PAX8	CAGATCCAAAAAGGCGGAGCTA	ACACCCCTCTCTCCTTGTTGT
2 PAX8	CTGACCACACCGCGTTCTTA	ATCGCATCTCATGCCCTTCTC
2 PAX8	CCCAGCTTTCAGGTAACCTTTGA	TGAATACTCTGGCAATGCCTATGG

2 PAX8	GCCTCGCTGTAGGAGGAGTAG	CCCCAACCATTCCTGAGCTTG
2 PAX8	ACACGAGCATGTGTGTGTATCC	TCATGGCCTTTCTTCTCTTCATCAC
2 PAX8	CTCTTTGCAGTGCTCCCCTT	TGACGACTCTTTGCTGTATTTTTCCA
2 PAX8	GAAGGGTTTCTGTCCCCATCAA	GAGTCACCCCAGTCGGATTC
2 PAX8	CCCATTGATGGAGTAGGTGGA	AGTCCCCAGCTCAACTGTAACTA
2 PAX8	GAGTCCTCCTGTTGCTCAGTC	TTCTGACCAGCAGTGTTGTTGTTA
2 PMS1	GCCCTAGGATTAACTTTTTCCCGTAA	ACGAGGATCAGACAGGTAAGTTGAT
2 PMS1	AGCAGATGACCAAAGATACAGTGG	TGAAACCCAAATGTAACTCTTGGAACA
2 PMS1	GAGGGTTCACTAAATGTTTTAACAGCAA	TGCTACTCCATAGAATGGGAGACA
2 PMS1	TCTTTCACTTGTGTTTCTTTCTAAGTGTGA	GCCTTGATACCCTCCCCGTTAT
2 PMS1	ACTATGGATTTGATAAAATTGAGGTGCGA	CCACGAAAACCGTAAGTTGTCAAATT
2 PMS1	GCACCTGTAATGGCAATGAAGTACTA	ACCTTACCTCAGCTATACAACAAATTGAC
2 PMS1	GGTTTTCGTGGAGAAGCCTTG	CCATACTTCACCAAATACTGGGTTAGT
2 PMS1	CAGCTTCAGGGTATATCATGGTAAAATGT	GCGACCATGAACACACTCTTTAATTT
2 PMS1	AGAATGAAGCACCAGTTTGGAAATG	CTCATGCTGTTTTATGACAGAACCAATT
2 PMS1	CAAATCTTTGATAATGAACCCATCGCAA	CTTGACCAAGATGTGAAGGTTTCTG
2 PMS1	GGCAGTGGCCACATACTTTCT	CCCAAAGCAATATATTTGTATTCACCGA
2 PMS1	CTGAAATTTTAAGCTGAGGATGAATGCAA	CATGAGAGCCATCTTGTGATCTGA
2 PMS1	CAGTTATTTGGCAGAAAAGCAGAGTA	TGTGAACAGCTTCATGAGTAAATCAGAA
2 PMS1	CTTGTTTTATAAGTTCCGTTAAGCACAC	GTATATTTCCCCTGCTCCACTCATC
2 PMS1	CAGGTCTTGAAAACTCTTCGGAAATT	TCATTAAGATTCATTTGTTCAGGGATTGGA
2 PMS1	ACTTAGCAATCGAGTAATCAAGAAACCC	ACTCAATGTCTTCCACAGTTCTTCAA
2 PMS1	TCCTAAGACTAGTTTAGAGGATGCAACA	TGGAAATAGCTGAACATTCAAGTCACA
2 PMS1	AAGATCTGTGACGTTCCTTCCAAAT	GCTCATGAGGAGATCTTGGATCTTT
2 PMS1	AACAGGTACAACTGTAACTGCTTTAAGA	AGGATACCAAAGCTCATGAGGAGAT
2 PMS1	CCTGTTGGTCAGGAATGCTTAACA	GAAGTGATGATCTGAGAACTTGAAAGGA
2 PMS1	CTGCGGCAACAGTTCGAC	GTATTTGGGTTCTCAAGGACACTCA
2 PMS1	TCTATTCTTAGATATGAAGAGAAGGCTACTAAA	CATGCGCTGGTGGGTTTTATC
2 PMS1	CACAAATGTCACTAAAAGATGGCAGAAA	TTTCAATTTGGGACTGAAGGAGTTCA
2 PMS1	TCAACCAAAACTTGATGAACTCCTTCA	AAACCTGAGATTGTGGATCAAGCA
2 PMS1	AGTTGACTTAGAAGAGAAGGATGAACCT	GCTCTGCAGGAAGTTTATGATTCTCA
2 PMS1	GAAGCCCTGCTATTTAAAAGACTTCT	CCCAACCTTCTACCTTCGATTTTAATCT

2 PMS1	TGTTATATATAGCCAGACCCGTGGA	TTCTGGTGTTGAAAGACTAGTGAAAGAG
2 PMS1	TTCCAAAGTGTGATGCAGACCA	GGAAGCAGTCCTTGTTACAATGTGA
2 PMS1	CTCAGTTGAATTTGCTGGGTTTTATTGT	AACATCAGCTGTAGGAACATCGATT
2 PMS1	GTTTTAATTGCTCTTGAAAATCTGATGACGA	GCACATCTGTTTCTGCTGTTTTACTAAG
2 PMS1	GACTTGTTATGGACCATTACCTAGTACA	TTGGAATGGAATGACTGAAGTATCAACA
2 PMS1	CACCAGATAAGTATTGGTGACTTTGGT	TCTGAGAGTTCTCCCATGATACATTACT
2 PMS1	CACTAAGAATGCATTTCAGGACATTTCA	GGTCTTTATTGCCATTTTCTGACTGG
2 REL	GCTATATTAATCTCACTGACCTCCTCCT	TGGAATGCTGCCTGCTGAT
2 REL	GGGAATGCGTTTTAGATACAAATGTGAA	TGAGATAGACACAGAAGAAGGGTCT
2 REL	GGCCTCCTGACTGACTGACT	CCTTCAGAAGAGCTCCTTTCCC
2 REL	TCTACTTTGTGACAAAGTTCAGAAAGGT	GCTTCCCAATCGTTCAACACAAAA
2 REL	CATAGAAGTTCGTTTTGTGTTGAACGAT	GGTCTCCGCAACTGCATTTT
2 REL	GCTATCACAGAACCCGTAACAGT	CAAGAAGATGTTATTAGCAGGACCACTT
2 REL	CTTTTCCTCAAATTCTTCCCTGCAA	AAATATTTCATCTCCTCCTCTGACACT
2 REL	TGACCCATATAAACCTCATCCTCATGAT	TGTCATGGAGAAGAAACAGAACATCC
2 REL	ATGTTACATTGGTTTCCTTGACATTTTTCTTT	CACAACTGCATCATGAGAAAACAAGT
2 REL	TTTTTCCTCCCACAGAACCAAACT	GTGCCATTGAGGCATGATGTG
2 REL	GGCCCATCTCAAGTGGATTGT	GGATACCTTGCGAATTAGAAGGAAGTG
2 REL	CCCACTGAGTAGTTTTTCAACAAGGA	CCATATAAATCTGCTGATGGCATGGA
2 REL	CATAGTCGGAATGGAAGCGTCA	GGGTTTTCAAGATTCATGCTCAGAAG
2 REL	GCATGGGAGAGACTGATAATCCAAGA	CAAATGCATCTGATTGTGAAACAAAAAC
2 REL	TTGGATGCTATTCAAGGTTATTGGCT	ACCACATTGAGGTCACAATCTTCAA
2 REL	AGTCCCTGAAAAACAGCTGAATGATA	AGTACTTACGGTTGTCATAAATTGGGTT
2 REL	cccggccAGTTTTTCAGATTTTAA	CCTTCTCCAATTGAACCGAGGA
2 REL	TTAATTTTCCTGAGAGACCAAGACCTG	TTGTGCAAAGTACTGGTCTTTCTGAT
2 REL	CAGAGCAATTGGAAGCACGTT	CACACATCGAATACCCAAATTTTGGA
2 REL	CAGGCGCCAATTCCAATACTACT	CATACTGCCAATACCTGAATACTGACTA
2 REL	CCAAACAGTCATGGTTTTGTTCAAGA	TGCTGCATCTATATAGGTGGTATCAAGA
2 SF3B1	AAAGGACAGTCATGAGTTGGTAATATTAATC	TGGAAGGCCGAGAGATCATTTC
2 SF3B1	GCCTTCCACTCTAGCATAGTAATCTTCA	TTCTTATGTATGTTTAATTCTGTACATGAGCAT
2 SF3B1	TCACGATGTTCTAAAATGAAGGAAGAGTT	GAGTGTTAAAATCGCTTTCCTTCTTGTT
2 SF3B1	ACGGCATAAATGTAGTCTTTTCCCATT	ATAGCTATTGTTGCAGAAACATGTTCA

2	SF3B1	GGCAGGGAGTACTGTAAAGGG	ACAGCTTGTTGACCCATTTGTTTTT
2	SF3B1	TCACTCAACATTTCACCCACACA	CTGGACGAGGTGATACACCAG
2	SF3B1	TCTGTTTTTACGAGCACTGGAAGTT	GCAACCCCAGGCTCAAAAATATG
2	SF3B1	GTGTGTGGCTAGGTGTAGGAT	TTTCTAATTATTTCTGTGTGGGTGTGTGA
2	SF3B1	TTCAACTAAACTTCTAAGATGTGGCAAGA	TCTTAAAAGCTGTGTGCAAAAGCAA
2	SF3B1	CAATCTTAATACCAGTGTGTCTCGCTT	GGATGAGTATGTCCGTAACACAACAG
2	SF3B1	AAAAGCAAATTCAGAAGCATGCCA	CCCAGAGCGTCTTGATCCTT
2	SF3B1	AGTGGGAAAGAATTACCATCTGCAA	GATCCATTTGCTGAGCACAGAC
2	SF3B1	AGGCTACAACAGCAAAAGCTCTA	ACCAACTCATGACTGTCCTTTCTTTG
2	SF3B1	CCCGGTCTGCAATCTTTGGA	ACATTTGTGCTTTATGGTGTTCTGATTTT
2	SF3B1	ATGTATGGTGTGTACTTGTGCAA	ACAAACTTGATGACTTAGTTCGTCCAT
2	SF3B1	GGAGAACAAACCTTATGCACATATGGA	GGAGCTGGTCCTTTGTTTAATCAGATT
2	SF3B1	TGTAGGAGACATCAGCAGAGGAA	AAAGAAACCACACCTATTACTCTGCTC
2	SF3B1	ACAATTATGTCCAATGAGACAGTTCTACC	GGATGAAACACCAGCTAGTCAGAT
2	SF3B1	TGTGCCAATTGGTGTCTTTCCA	AGATTCTATTGGTGAAACACCGACTC
2	SF3B1	CATCCCACCGTGATTTTCTTTTACTG	TCTTTTTAAAGATACTCCTGGGCATGG
2	SF3B1	CAATATTGCAACATTCTACATGGTCCAA	GATTTGGTTGTGAAGATTCGCTGAA
2	SF3B1	GGGCCATACATAGTTCAACAAGTGA	TTTTTCCCTTCCGTTGTAGAGACC
2	SF3B1	CTAGCCGTCTGTCTGTGTACAA	CCGGTGAATATTTCCTGTAAAACTAAGAC
2	SF3B1	GGGAAGAAGTAAGAATTTGATGCAAAAG	CAAACCATACTTGCCTCAGATCTGT
2	SF3B1	TGTTATTTAAACGCCACAAAACTGTACC	GTATTTCAACTGTGCAGTCATAAACCAA
2	SF3B1	AACTGCATTATTCAGACCATGCCT	GCCACAGTCAACACATTTGGTT
2	SF3B1	CACCCAATGGCCTTTGCAATAT	GGGAGCTGAATATGTATCTGCAAG
2	SF3B1	CTCAAAGCAAATCCTCATCCACTCT	TGAGTTAATCATGTTTTTAGAACTGAATTTGCA
2	SF3B1	TCAAGCACATATAAACTGTGAGATAATCAA	CACTTCTGGCAGCACAGGAT
2	SF3B1	CTTACCTGTCGGTAATTTCTTCTATCCA	GGTAAAACAGTGTTGTGGGACAGAT
2	SF3B1	CTGTGCTGCCAGAAGTGTTTAAAA	tCACGTAATCAGCAATGAGTATTCTCTTC
2	SF3B1	TCTTTCACAACCATTAAAACAGGAGACA	GTACTCTTTTTCCTGTTGCTGTTCTTTT
2	SF3B1	GCTAACAAAAACCTCCCAACTCCTA	CCTGAAGTATTGGGCAGCATTC
2	SF3B1	AATGGCCTTCAGTGCTCCAA	GCATCAAATTCTTACTTCTTCCCCAAAT
2	SF3B1	GAAGTGATGGGTTCCTGGGTT	CAGCTTGCAACAGGTAATGTTTTGAATA
2	SF3B1	TTTAAGGTGTGAAGTAGCTGTGCAT	TTTACAACTCCATCTACATTGGTTCCC

2	SF3B1	GGCTCAGCTCCTACGAAAGA	GTCTGTGTGTTCGAGTGGACAA
2	SF3B1	AGTTTATTTCTGCTAGTATCACTTACCAATAG	CATGCAAACTGAAGATCGAACTATGAAA
2	SF3B1	ATTTCCAGATGGCTGGTCATTAACA	ACTTCCTCCTCCAGCTGGTTAT
2	SF3B1	CGAGCTGGAGTTCGAATAGGAA	GATGTTGTCTCCAAGCAAATAAAAACC
2	SF3B1	AAAATTCTGTTAGAACCATGAAACATATCCA	GGATGCAGAATATGCCAACTACTATACT
2	SF3B1	TCCTCATCAGGAGACTGGAATTCT	aGGGTTTGGCTGCTTTCTTGA
2	SF3B1	GGATTTCTATGGGAACTCAGACATTCA	CTTGGTCAGAAGAAGCCAGGATAT
2	SF3B1	AAAGATCACCTGTTCTGTTGACTGT	GGTTTTCATTGCAGGATGACGA
2	SF3B1	atGTACTTTAGTAATTTAGATTTATGTCGCCT	GCAACTCCTTATGGTATCGAATCTTTTG
2	SF3B1	CCCTTCCATAAAGGCTTTAACACAGAAT	GGCATAGTTAAAACCTGTGTTTGGTT
2	SF3B1	CCAGATTAAACCAGATGGCTGCAA	AGATTTGCTGGATACGTGACATCA
2	SF3B1	CATCTTCAAGTTCAGTTGCAGCAAT	CACAGATTCGAGAAATTCAAGGCAAG
2	SF3B1	AGCTTCATCAAGAGCTGCCTT	AAAGAAATGCATTGTGTTGGGAGTTC
2	SF3B1	TGCATTCTAGAAAAATTTGCTTGACAACT	GGAGCAGCAGATATTGATCATAAACTTG
2	SF3B1	CTCTGTAGTCTGTTCTTGGAAAGCATAA	CCAGGATTGTGGATGATCTGAAAGA
2	SF3B1	CATTTTTCTGTACTGTTCGGCTTCA	CCTTGGAAAAGCAGTCTAAAAGGTTTT
2	SF3B1	AAAGAAATTACCTCTGCCTGATCCC	GCAGAAAAAGCTAAAGCTGGAGAACTA
2	SF3B1	TGCTGCTCCATTGACGACTTT	GTGATTGCGCTAATGGTAGAAAAATACA
2	SF3B1	TGGCCAAATTTGAAAATTGATACTGCTT	CCCCCATCTTAAAGAACAGACATGA
2	SF3B1	GCAATACGACCAACAAGATCAATACAAT	AGGAAAGTGAACAAAAGTTGCAATTCAA
2	SF3B1	GCTCTTCTGGACTAAGTGTTGATTCAT	TGATAAACTATTGGTAAGTGATACTAGCAGAAA
2	SF3B1	ATTTGGGCAAAGCCAATGCAA	GCCCACTTTCTGATGAGGAATTAGA
2	SF3B1	ACCTTATATCCTTCTGGGAACATAGCA	GCTTTTCTTGGTAGGTCACATAATGAGT
2	SF3B1	GCCAAGCCTGAAGCTGTTC	AGCAAATAATAGGATTGATCTTAACTGTCTTTT
2	SOX11	ggcggTGCCAAGACCTC	GCTGCCCAGCACGTAGT
2	SOX11	CAAGAAATGCGGCAAGCTCAA	AACACGCACTTGACCGTCT
2	SOX11	CGGGCGACGACTACGTG	CGTCCGGCTCCTGTTTGAT
2	SOX11	TGCCGAGGACTTTGCAACTT	CTCTCCGCTTCCAAGCTCTC
2	SOX11	CGTGTTTCTGGATGAGgacgac	GGCACTTTGGCGACGTT
2	SOX11	GCAGCTGCTGAGACGCTAC	GTGCTGCTTGGTGATGTTCTT
2	SOX11	CCTGCAGCGGATCATGGT	AGTCTGGGTCGCTCTCGT
2	SOX11	GAGGGCGAATTCATGGCTTG	CCATGATCTTCCTGCGTTCGAT
2	SOX11	GCCGCCTCTACTACAGCTT	CAGGTCGAACATCAGGTCGTC
2	SOX11	CGCGTTCATGGTATGGTCCAA	GGATCTTCTCGCTGTCCTTCAG
2	SOX11	AGCTTCAAGAACATCACCAAGCA	AGAAATTCAAGCTCAGGTCGAACA
2	SOX11	GGGCAAGCGCTGGAAAATG	tcTTCTCTGGGCTCTGGCT

2 SOX11	ACGCCGACGACCTGATG	CCCTCGCTGAACGAATCCAAAT
2 SOX11	CCGACTACCCCGACTACAAGTA	GCATTTCTTGCTGGAGCCCTT
2 SOX11	CCTGTCGCTGGTGGATAAGG	GAACACCAGGTCGGAGAAGTT
2 SOX11	ACTTCGAGTTCCCCGACTACT	caccaCTACAACTTCCTCCCAA
2 STK36	ATCTTTTCAGTAGTGTGATTAGGCGTT	CAGACCCCGCATTATTTCAATCTC
2 STK36	GAGCTGAGGAATTTGCAACGA	TGGGAAATTCCTGGAACCCTTG
2 STK36	CCGCCCTCTTTCACTTTTATCTCT	GATAGCGAAAGCTCCCCTTCC
2 STK36	GTTTTGTGGCACCGCTTCTC	GAAGAGAGATCAATGCACTCCCA
2 STK36	GGACTGACCCATTCACCACAT	GCTCAGAGTCATCTGGTTGGG
2 STK36	ACCTTTTTCTGGACCTGTTGGG	ACACGGAGAATGATAGGAGACACA
2 STK36	TTCCATCCTTCACATTGCCCTT	CAACAATGCAAAAGCACTTCTTATCC TA
2 STK36	TGACCTGCCTTGGTTTAACGG	CCATCCATCCCAAGATCCATCTGTA
2 STK36	CCAAGTGGACACAGGGACTAT	ATGGGCCTGTTCGTCCTTTAG
2 STK36	CACTGTCTCAGCCAGAGGTTG	ACTTCTGTGGAGACACCCTACTTTAT
2 STK36	CTACCCCCAGAACTTCAGGTC	AGCCTGGTCATTTCACATATTTTCCT
2 STK36	CTCTGAAAATATCAATCGTTGCCTCTT T	CTCCCGGCAGAAGGAATACAAG
2 STK36	TCCAGCTGCAGTGATTCTGTT	GCTTCTGGCCTGGTGCTTAC
2 STK36	GCATCTGTTTCTGGAGGCATTCTT	GAGGTCAAAGGTCACCCCTT
2 STK36	GTCTATTGGGACAGCTTGGTCAG	CTTTTCCCCTTGAATGCAGTCCTA
2 STK36	TCCCTGGATCTATAGCTCTTCACC	AAAGGTATCCCTCTTTGTGCAACA
2 STK36	GAGGGAGGAATGATAAAGGTTAAGTG A	GGTCGGCCAAGACACCTATAAG
2 STK36	TGAATCCTTTTACCTCCTGTTCTCTCA	GAAACGAGACGATGGTTCTAGGG
2 STK36	GTCAGCCAGCTGCTGTTTCTA	GTTCTGAGGCTTCATATCTCGGT
2 STK36	CTGGACATGGATGCTGACCTC	GCTGCTGGGAAGTAAATTAGAGGATA C
2 STK36	TTGTGAACACAGTGTCTGCCT	AGAAGCTCTTGGATAAAGGACAAGTG
2 STK36	CATACTGCCAGGGTCCTGTC	TGCTGTGTTGGAGCAAGTGT
2 STK36	GCATTCCCACCGCATCCTAC	GTGGAACTGGAAGCTTAGATGGAT
2 STK36	TGCGGGCACACACTTATAGG	CACAGCAAAGCTGGCACTG
2 STK36	GCTTGGAGACAAGGATCCTGTT	GCCCAGAGCTGATGCAACATT
2 STK36	ATTCAGTGTTGCCCCCTACC	GCTGCTCTTCAATTCTGAGGCTAA
2 STK36	GGCCACTCCTCAGGAATCAAG	CTTTGGCTAGAGTTAAAGATGTGCAA
2 STK36	CCAGTCTTCTGATGCCCTGAT	CATCCAAGACAACCTGCTGTG
2 STK36	TTTTACTCCAGCTTGCTGACGA	AAGTAAGTCAAGAGAACCTCCCTTCT
2 STK36	GCCGCTTACCAACCTCCTTTAA	GGTAGATGCTAGGGTGAAAAGGG
2 STK36	CTCCCTTAGGTTCCCTACTCCT	CAGAGGAAATGGCTCCAGGTA
2 STK36	TGCGAGAGCAGAGTGAGGATA	GGAACAACCAATTGTAGCTCGGA
2 STK36	CTGCCAGACTCCTTCCTTCTC	GAACAACCTCTGGCTGAGACA

2 STK36	CCTGTTCCATTGGGTCTCCATTT	AGATGCTTCAGGATGGACATGAG
2 STK36	CCTGTCCCAGCATGGAAGTATC	GTAACTTCTTTCCTGCCTTCTTCTTTCT
2 STK36	CTCTGCTCCGGTTTGGGATAT	CTCTGGAGACATATAGAGTGGTGTG
2 STK36	GGAAGGGAAAGAATGACTGATGGAAT	CTGGGAACCCTGATGACTCAC
2 STK36	GTCATAGGTTTGGAACCCGTTCT	AGTAAGAGACCCTAGGGCTCAC
2 STK36	TGGTGCTGACATCCATCAAAGG	CTGACCAGCTGAAAGATGCTTG
2 STK36	GCACCCCTCCCTTCTATGCTA	GAACAGAGATAGAGGGAGACAGATTTCT
2 STK36	GATTCCCCATGGTTTCTACTGGTT	GCCTTCAGTTGAATAGGCACAGG
2 STK36	CACCTGCTAGAGACCACTGAG	TCCACAGTGTCTTTCTCCCCATTA
2 STK36	TCGAGTGGGAATTCCTGGGTAA	CAGTGACTGATTCCCCAGAGAG
2 STK36	GCCAGTGAGAAACTATCCTTGCT	AAAGAGCTGGCAACCAAAGTTG
2 STK36	TTGGAGATCCTCAGGCTGGTAT	GTCTCCACATGCCATTTCTAGGAG
2 STK36	GCTGTTACAGTGCGAAGTACCC	GGCCCTGATCATCATGGTTCA
2 STK36	GGGTTCCAGGAATTTCCCAACC	GAGAAGTTGAAGGCAGAAAGCATAC
2 STK36	GTAATCATGTGGATAGGATTGAGCCTT	GTGATGCCAGGAGTTAAGAGGATT
2 TCF7L1	CATTCTTCTCTCCCAGCTTACCT	GGCTGTGACAAAAGGAAAAGCA
2 TCF7L1	GGACTCGGAGGTAAGGAAGCA	GTCCCAGCGGGCACATA
2 TCF7L1	cctggcacTTACTTGATTCCATAAATAAC	AGTCAGCGGATGCATGTGAT
2 TCF7L1	GTCTAATAAAGTTCCTGTCGTTCAGCA	CCTGAGTGGCAGACGACTTA
2 TCF7L1	CCTCACCTCGCCTTGGT	CGCTCCTCCGGGAGACA
2 TCF7L1	CCCGTACCTCTCCAACGG	TTGGGATCGGCGCAGAA
2 TCF7L1	GCGAACGACGAGCTGATCC	GCCGCGGTGCTTCCTTA
2 TCF7L1	TTGTGTGACACCTGACATGCT	GGCCCTCATCTCCTTCATATACAAC
2 TCF7L1	AAGAAGCCTCTGAATGCCTTCAT	AGGGAGAGGGCAGGTCTTAC
2 TCF7L1	GCGAGGGAACAGTCTGACATAT	AGAGAGGCTGTGGGTTCCTTA
2 TCF7L1	CCTGCATTGATGGCTCCGT	CTGCCCAGAGTGTGCACTTA
2 TCF7L1	GCCTCATCTCACCAACAGCTT	GGTGCTTTAAGGTGAAACAGACTTAC
2 TCF7L1	CTGCCCTGAGCTTTCCCAATA	GCTAAGACAAACATGCGGACAG
2 TCF7L1	GGACATGCCTGTGCCCTTAA	CCACCATGTGAGGAGAGAACC
2 TCF7L1	CGCCAAAATCATCCCTGTGTCT	GCAGGTACTGAACACATGGCT
2 TCF7L1	CTGGCCTCCAAGAGCAAGA	GGCTTGCTCCGAATGGGT
2 TCF7L1	CACAGCCTGGTCTCCAGTC	GAATCCAGGCTGCCGCTTA
2 TCF7L1	CCACTCCCTCTGCAGCTTT	CATCTGCCCAGAGGAGGAG
2 TCF7L1	CAGCTGGCTCTCCACTCT	AGAGCTGTGGGCATGGAC
2 TCF7L1	CCAGCCTCCCCTCCTGT	GGAGCTTAGTGGGCAGACTTG
2 TCF7L1	CAGCCTCTTTCCCTGGTCAC	TGCTGTTGTTACCAGCCACTTTA
2 UGT1A1	CTATCTCAAACACGCATGCCT	ACAATTCCATGTTCTCCAGAAGCATTAA
2 UGT1A1	GGCTCTAGGAATTTGAAGCCTACA	ATTTTGCCCAAAGCATCAGCAAT
2 UGT1A1	AAATTCCAGAGAAGAAAGCTATGGCA	GAGAATTAATCTGGAAGCTGGAAGTCT

2 UGT1A1	TTTCTTTACGTTCTGCTCTTTTTGCC	ACATCCAATCCGCCCAACATAC
2 UGT1A1	TGTAACTGCTGACATCCTCCCT	CATGCGCTTTGCATTGTCCAT
2 UGT1A1	GATGCCCTTGTTTGGTGATCAG	ATGCCATGACCAAAGTATTCTTCTGT
2 UGT1A1	CTGGCAGAAAGCAGCTTTGATG	CACGTAGGAGAATGGGTTGGG
2 UGT1A1	GAACCTCTGGCAGGAGCAAA	CAAGCATGCTCAGCCAGTG
2 UGT1A1	GAAGATACTGTTGATCCCAGTGGAT	GAATGGCACAGGGTACGTCT
2 UGT1A1	CTGGAATTTGAGGCTACCCAGT	GGAATAAACCACGTCGCACAGA
2 UGT1A1	AGACGGAGCATTTTACACCTTGA	AGACAAAAGCATAGCAGAGTCCTTT
2 UGT1A1	GCTCATTGCCTTTTCACAGAACTT	AAGTCACTTCTAAACAGCCAGACAG
2 UGT1A1	AGCGTGTGATCAAAACATACAAGAAAAT	GAAAGGGTCCGTCAGCATGA
2 UGT1A1	CAGGACCTATTGAGCTCTGCAT	TGTAAAAGTCCCACTCCAATACACAC
2 UGT1A1	AGGATTGTTCATACCACAGGTGTTC	GCCCTTGTGCCTCATCACAAA
2 UGT1A1	GCCGTGTTCTGGGTGGAG	CGGTAGCCATAAGCACAACATTTAAAG
2 UGT1A1	GCTGACAGTGGCCTTCATCAC	TGGAAATGACTAGGGAATGGTTCAAAATT
2 XPO1	cccagcTGGCACTTAACATTTAA	GCTGCAGCTCAGAGTTTTTATCAAAC
2 XPO1	TCACAACAGAAAAGATATGCTGGAGAAT	ACTATCATGAGTGCTGTCAGTCTGTA
2 XPO1	GCATATTCCATAAAACACAGCATCAACA	GTATGCGAACTGTAAAAAGGGAAACTT
2 XPO1	GGCTCACCCAACCAGATATTAACT	AATGGAATTGCATCTAATGTACACCTGA
2 XPO1	TGTTTCCAAGGTTGCATGTACAA	TTTAGCATGTGCAATGAATTCTCACAG
2 XPO1	TGGATCCAATAGTTGCCCTCCT	TGGAGAAGTGATGCCATTTATTGATGAA
2 XPO1	AGAACACGTCTTGTTAAATCCCATGA	GGGTACATGATTGGTGCACAA
2 XPO1	TCAAGTGTTCTTGTACTGTTTGATCTGT	TTGTGTGTGGTTTTCCTTATTAGGGT
2 XPO1	TTGAGGCTGAAGATCACAAATAATAGTGT	CCAGGATATGGCTTGTGATACTTTCAT
2 XPO1	CCTGAACCTGAACGAAATGCCT	CCATGATGGAGTCCAGGACAAAA
2 XPO1	TTTCAAGAATTAAGGCTATCCTGCAGAA	GGGACATATAACAGCTGAAATACCTCAA
2 XPO1	CAATGTGCATTCAAAAACAGCATCAAAT	CCCCTCTGTTGGATGCAGTTC
2 XPO1	GCTGGGACATTTCTCTGATAATCAATGA	TGCTACATCTCATAGACAGGAATAACCT
2 XPO1	AAAAGGCACTTTTAAGAGTTATTAGTAGGCA	TCAGTTTGTTTCTCTGCACCTTTCTT
2 XPO1	ACCTCCATAAGAGTTTCCCTGAGATTTAA	CGTACTCAAATGGAAAAGATGATGAACA
2 XPO1	GTGCAGAGAAACAAACTGAGATTTTGAA	AATGCGAACTGATCGTCCCTTTTA
2 XPO1	CATAAAAGTGGTCACATCTACCCCTAA	TCATTGTGTTTTGATTTGCAGTGCT
2 XPO1	GGATTAAAGATGCCAGGGACAGAC	GCAAGTTGAAGTTTATTGCGTTGACATT
2 XPO1	AGAGATTTACCATGCATGAATTCGAACA	AGGATTATGTGAACAGAAAAGAGGCAA

2 XPO1	CTTTGCCTCTTTTCTGTTCACATAATCC	GGACGAAAAACGATTTCTTGTTACTGTT
2 XPO1	AACCCAACAACTCTGTTACACTGT	GATTTCAGCCAAAAACTGGATATCAACT
2 XPO1	TCCATGGTATAAGCAATTCACCACATTA	ACCAAAACTTGATAGGCAGCTTCA
2 XPO1	GTTTGACTTAAGGCAGTAGAAGAGAAGT	GCTTAATGTATACAAGTGCCTCAGTGAA
2 XPO1	TCTAATTCATACCTATCCCTTGCATACCT	GACAGAGAAGCTTCACAATCAAGTG
2 XPO1	ATTTTTCCATGACCACTCTGTACCATT	TGCTCATTATTTGCATTTGAAACCATGT
2 XPO1	CCATTAGCTTGGATAGCTGCAGAAATAT	TCAAGCAGCTTGGTAGCATTTTG
2 XPO1	CTTTGCAGGCTCTCACATTTGTTTT	cccagccGTGGATAATTCTTATATGAT
2 XPO1	AGCAATTTAACATATTACTGATCATAGTTCCTT	CTAGTGGACAGATAACCCAAGTCAAATC
2 XPO1	TGCTTAGATTTGACTTGGGTTATCTGTC	AGATACTGAAACAAGAATGGCCCAAA
2 XPO1	CCTACTTGCTCCAACAATATCACTGAT	GTGATTTATCCTTACTCCTGCCTTTATCC
2 XPO1	AAAGCAATAAGCTCCATTAGAGCAA	CTTCCAAGGAACCAGTGCGA
2 XPO1	TCTTTATTTTCCTACCTTCGCACTGG	CTGTCCATTTGTGAATCCTGAGAGTT
2 XPO1	ccgaccAGGAACATTCTACGTT	AATCCTGGAAATCCAGTTAACAACCA
2 XPO1	GGATTCAGACCTCAAAACCACAGT	CGTTGCTTTCTGGAAGTCAACATT
2 XPO1	TGTCTCCTGGGAGGAACATCAA	GCCCTTCATTATATGTTGTTGGTATCTG
2 XPO1	GCCAAATGATTCCAGTATTCAAGACAAAT	CCTTAAGGTGTAACAAGTGAAAAGCA
2 XPO1	AACTACAAGTACATTTCCTAAAATGTATTTCCA	GAGAAGTTGTGAGAGAATTCATGAAGGA
2 XPO1	ATTGGCAGGCAAATGAATAAAAGAACAT	CAATTCTCATTGTTTCCCAGCATTCC
2 XPO1	GTTTAAACTGTGTAGGTGGAATAGCAA	TCTAGCACAACAAAGACACTGAAACAT
2 XPO1	TGGCTTATCTTTGATACGTCGTTCTAAAA	TGTGAGTGTAAGCCAATATGAAGAACAA
2 XPO1	TGCTTTAGTTGCATCATTGTCAGAGT	TCAGGCCAATTAATTTTATGGTTCCCT
2 XPO1	GAAAAGAATATATGTGGCTATCCGGTGA	CTTGTACATGCAACCTTGGAAACA
2 XPO1	GGAATCCAGTTCAGAAATCTGAGCAA	ACTGTGTCAGTTTGTAATGGTAAGTGT
2 XPO1	CAAATGCTAATACTAAAAATGAGATACCTTCCT	GACAAGAGTCGACACAATTTTGGAAT
2 XPO1	CAGATTTTAAAACCACCACTTGCTTACT	GCAAAGAATGGCTCAAGAAGTACTG
2 XPO1	GCATCAGGATGCTCCTTTAAATGTGT	GAATTTGTGCCTTGCTCAGTAGTTTT
2 XPO1	ATTTTATGCTCTCCCAATAAGCTCCAA	GCATGCTACAAAAGAGCTCAACAAT
2 XPO1	CGACAAATACCCACATGCTGTTTT	GCTGATGAAGAGAAACATAAACGTCAAAT
3 ATR	AGATGAGGTTCTAGTATTTCCCGAACA	GCTTCACTGTGGACTTACAAATGTAAAA
3 ATR	CAGGTATGTCAAGGAAGATACAGTTGT	GACCGTCATGGTGAAAATATTCTCTTTG

3 ATR	TCACAAATAAGATTCACGTAGATTTTGTGAAA	GGCCTATATTGCAGGGTTTGATGATA
3 ATR	AGTCATATAAAACTGAAGTTTACCATATCATCA	GAAATCCTCATTCCTCTACAATCAGTCA
3 ATR	TGTACGCATTCACCAGTCAAAGAAT	AGGCATTGTGTTTCTTTTAGGTACAGT
3 ATR	GGAACGGCAGTAAGCTGATCTA	CCAAGAATATAATACGTTGTTATGGTTGAATG
3 ATR	GGTACCCAGAATTGATGGAAGTGTAG	TCCACATTAAGCATGAGCACTCATTTTA
3 ATR	GCTTCTTCTACCAGCTTTTTAAGCATTT	ACAGACTGCTGAACCTTTGTAATCA
3 ATR	AGTAGTGTGAGAAACCTTTTGTTGAGG	CAGCATTCTCCAGGTGACAGTT
3 ATR	AGCTACCCAGTTTAGAGAATCTTCTTGA	AACATTTTAACATAGGCTCTATCTATCTTCAG
3 ATR	CTTACCTCTGCATCTACCTCAATTCC	GCTAAAAGCAGCTTTGTGCCATTTA
3 ATR	CCAGCTGGCACAAATTTAAGGAAA	ATTTAAATATGCTGCTGGAAAAACTCTG
3 ATR	CACACCGTCTTCAAACATGACA	CCTTGTAGAAATGGATACTGACCAATTGA
3 ATR	CAGCTTTGATAATGGCTCTTCATAGAGT	CAGAACTTTTTCAGCTTGGAGGACTA
3 ATR	AAGTGCTAGCTGGTTGTGCT	TCTTACTTTGGCAGATAGGTTGTGTTC
3 ATR	AATCCAAGTTCATTACAGGAAACCCA	GAATACAGTGGCCTAAAGAATCCTGTTA
3 ATR	ACAACAGCAATTCCTTCTAACATCTCAA	GAATTAAACATGTGGACATGAACCAAAAGA
3 ATR	GGAGGATTTTATAGAACAGATGGCAAGT	CTCCGTGATGTTGCTTGATTTCA
3 ATR	GGGAGGATTTCATGATATGCTGGA	cCACAGTCTGGTTTCTTAACAAAAGGA
3 ATR	GTGTGTGCTAGGCATTCAGATAGA	TTGGTCTGGAGTAAAGAAGCCAATTT
3 ATR	ACCATTCTGCAAAGTTACTACCCAATT	AGTCAACTGAAGGAGTTGCTGTAATTT
3 ATR	AACCTCAATAGGACAGAGAACTCTTTTG	TCAAGCTCGGTTGCTCTGTG
3 ATR	GCCCCCAATTCCCCTAAACATTC	GCAACAGACAGTGAAACAGTAGAAC
3 ATR	CCTTTCAAAAGCACTGTCACCAA	AAGCTTCCTAGCTCAGGAAAACATAC
3 ATR	GGAATCAGCGGAGGAGGATG	CGTTGGCGTGGTTGACT
3 ATR	AGGCTATAATTTACTCACCCTCTTTCCT	TCTCATTTGGTCTGTTCTTGTTCCAA
3 ATR	TTCAGATAATGAAGGGCACGTTCTAATT	CTGATCTTGCTGCCAAAGCA
3 ATR	TTCCTAAAGTTCGAATGAGAGCAGAAG	TCACCCAGACAAATGTACTTTTTGGT
3 ATR	AAGATTATGATAGTACCGGTTTATTGCACAA	TGAACAGATGCAAGGAAATCCTCAAT
3 ATR	TGCACAATTCTAGAAGCTTATCTGTTAGG	TCATCTTATCCCATGCGTGTGAA
3 ATR	TGGCTAAATACAAACTGAATGAAGGTCA	GAAAACCTCAGCAGTAATAGTGATGGAA
3 ATR	ACGACGCCTTTTGGGTGATA	AGTACTTGTTGGGCCCACTTT
3 ATR	CCATACTTTCCATTTTCAAAGCTGCAT	CCTAACATCTAATACATTCTTGCTGCCT
3 ATR	CAAGTTAGTGCTACTGGAAAATGCaaaa	GGTGAAGATGATGACCACACTGAG

3 ATR	ATCCTTGAATCGAAGGCCAGTT	GCTTTACTCTGAAGCACTACTTAACTCT
3 ATR	GCAACTCTGAAATAAAAGCAATCTGGTT	ATCATGATGTGTAAGCCAAAAGATGA
3 ATR	TCCATTAGTCTACAATCCTTTCTCAGGT	TTTAGGTGGAAATTCTTGCTTCTCTTCA
3 ATR	GTCTACAATCCTTTCTCAGGTCATCTTT	CATCATCTTTTAGGTGGAAATTCTTGCTT
3 ATR	ACAGCAAGCAAATAAAAATGGAGAATGG	AGCATATACACATGCCCAAATATCAAGA
3 ATR	CCCTGTTGTAAGAATCAAGGTATCCTT	CATTTGAGTTTACGTCAACCTAGCTCTA
3 ATR	ATACCCATATTATATAACTTAGTACCCACACT	TGGGATTATTGAATGGGTGAACAACA
3 ATR	GTTTGGTCAGAATAGGTCTCAAACCA	GAAAAGATGCAGAGTCTCGTAGAAGAG
3 ATR	GTCTCAAACCAGCAGTGTTGTTC	ACAGTGCTTAAGAAAAGATGCAGAGT
3 ATR	AGTTCTCTTCTACGAGACTCTGCAT	GCCTTCAATGACATTCTTTGGTTATGAA
3 ATR	GCTAGCAGCCAGAAAATGACCAA	CCATATCAGGGCCCGAGAGATA
3 ATR	TCCTTACCATCAGTTCAGGTGATATGA	AGCCTGTTGAGACAAGATTTCCAA
3 ATR	ACGCAGCAATAATTCATTATGCAATCC	GAGCTCCTGATGTACTAATAGCATGTT
3 ATR	ATTTTCCAATCCTGTCAGGTGACAT	CTGAAACTAGTGAGAGCAGAACAAATTG
3 ATR	AAGCTTGCAGCTGAAAGAGGTA	CGTGACATTTCTTGACTCTTCATTTGTT
3 ATR	GAACTGATAAAGGGAAGAGCTAATTGGT	TCAGGCACTGAAAGCTGAGAAAT
3 ATR	TTTCTGTTTGATTTGCTGTGTGGAC	GATCAGCATACCATAAATACCCAAGACA
3 ATR	TGAGTTGACACAGATCAGATGCAA	TGCCTTAGAATGGTTAGCTTTAGATGTC
3 ATR	GCACTTAGGCTTCAGGCAAAAA	CTGTTCAGAAACTGGACCTGGA
3 ATR	CACCACCTTATCAATGGTTGCC	AAGGACTGTCAACATAAATCCAAGAAGA
3 ATR	AGTCAACAGAGTTAACTGAAACTGCAT	GAGGGTAAGAACATGTTAATCCATGGT
3 ATR	CATAAATCGGCCCACTAGTAGCATAG	TGATGTTCACCAGGCACTAATTGT
3 ATR	GACATCCAAGTTATCACTACAGAAGGTT	CGTACTCACAATTTCTTTTGGCTTTACT
3 ATR	CCGGTCAATGAATTGACACAGAATTT	AACATTGACACTGAACACATTTGATGAG
3 ATR	TGAACAGATACAACCACAGATTCATACC	GCATTACCTTATACAGGAAGCTACTGAT
3 ATR	CAACCAAGATACATCTGGCATAGTAAGT	TTCTTTTTAGGCCAAGACCCATGT
3 ATR	GTAGTCGCTGCTCAATGTCAAGA	GGGTATTGGTCAGTAAAATGGTATTAACA
3 ATR	AAAATACTGAATTGCAAACCTCAAGTGAA	CTCACCAGAGAGAAATGGCTTTAAATAC
3 ATR	GAAAACGTTGGCAATTTCAGACAAC	GCTCTAGTTTTTGGTAGAATCCCTTCA
3 ATR	AAATATTACCTGTTAGCATGCACTCCA	GGTCCCCAAGCTATTATTGAAGTGATAA
3 ATR	GAGACGCCCTGGAACTTGTA	GCCAAAGTATTTCTAGCCTATCCTCAAC
3 ATR	AGCTGTCATCATCCACATTGCTT	CTGCTTTTTCACAATTGATCTCTCGAAT

3 ATR	CAACAAAAACTTCATCGTGAGAATGACA	GCCGCTCCGATCGTGTA
3 ATR	TCATTCCTCATTTGTACACGATCGG	CTTGGGCATGGGAATAACATACTTTAA
3 ATR	CTAGGAAATTAGACTGTCCAGCCAAA	CAGTTCATATATCAGTCAATGCCACGA
3 ATR	GCCATAGAGTTAACATTCGTGGCATT	TGAAAACGGTATGTGGTAGTTTGAATCT
3 ATR	CAACATAAACATAAAAGACTGGCCACA	CGAGCTGTAATGCACTTTGAATCATTTA
3 ATR	ACCTGTAAAAATCCAAGATGTTCCTGAA	GACTATCAGAGTGTAACCCGTTTTCT
3 ATR	AGCTAGGTTGACGTAAACTCAAATGTT	TGGAAGATCCAGACAAAGATGTTAGAGT
3 ATR	GCTTGATATTTCCACTAAAAGCCACTCT	GGTATTGTATACTCTTGTCCTTCAGCTT
3 ATR	TAATTACATTGGAGAAAGTAAGTTTCACATGTT	CAAAGTGACCATCTATCTTCTTCCACAT
3 ATR	CAACCCAGTAAGACATACACCAGAA	TGTGAAAGTGTGTTACTGTCTACACAT
3 ATR	TTCCAGAGTTCCTATTCAGGGCTAT	CATGAAAGCCTTGGCTTGCTG
3 ATR	ATAACAAGCAGTGGCATCCCT	CAAAGAAATTGTATGCTGCTATGCATGA
3 ATR	CTCCATCAGGTTCATGCATAGCA	AATTGCTGTTTCAATATAAACAGGTGGTTTT
3 ATR	AAGTGACAGTTTCCACATTACCATCA	ACCTCTTATACACATCCAGCCTAAAGA
3 ATR	GGTAGTGGAAGATAGCTGCAGTT	CACATAGAGCTTGGGACTGCT
3 ATR	TTGCTAGGGATTTAATTTTTGGACTACCA	AGCTCCTTTGCAGTTGATGAGTAT
3 ATR	tGATGACATTTCCCTGGCCATT	GCTAGAAAGGCTGGTCACCAC
3 ATR	GCCAAAGTAAGAGTTCTTGCCTTCTAAA	TGGTCATGAGCCGATTTTTAAGTCAAT
3 ATR	GGAGCTGATTGTAAATATCCCATGTGTT	ACTTTTTGAAGACTTGGTTTACCTCCAT
3 ATR	AGCATGACCCATCACATTTCTTCT	ACACAAGAAAATCTGTGAAGTCATCTGT
3 ATR	GTACCCCAAAAATAGCAGGACTCTT	TCATAACGAGACTTCTGCGGATTG
3 ATR	GGAGAGCATTGTAGGCTGTCTG	CTCTCACCAATGTGTTGTTACTTCCT
3 ATR	TGTAACAAATGACAGGAGGGAGTTG	AGTCAATCGTCAAGGATTTAGCAAATGA
3 ATR	TCTTTGAATGTTTGTAGCTAGATGCAGAA	CATGCAGTTACTGAGCTCTAGTGT
3 ATR	TGCAAACATGCAGTTCTCATACTCA	GCATTTTTACCTTGCCAAGTACTATGAC
3 ATR	TCTGTGACCATGGGCATCAATTT	GGGATTTCCTTAGGTAGGTATCCTATGT
3 ATR	GTAGAGATGAGGACTACAGCCCATA	TCTACATGATCCTCTTGTGGAATGGA
3 ATR	GGAATGCCCTTTCACTGGTTTACT	GTATTACCATTAGCTTCTCATCCTTCACTT
3 ATR	TGTATTGGAACTTGGGAACAATTCTGT	CAACTAACTATTAGGGATGCTGTGCAA
3 ATR	cgcacccATCCTAAAACTGCTTAT	CAGATGAATTAAACACGTACAGAGTGGA
3 ATR	CACCAAATCCCACTGTGACAATTT	CCAATGAAAAATCTCTGAATGTCACCTT
3 ATR	ACTCTTAAAAAGTACTGCTGTTGCCT	CATCTGTCTCTGGAGCAGCATAC

3 ATR	GCTGCAACCAGAGCTCTAATTTCT	TGTTGTTTTTAATTTTAGGGCCGCAA
3 ATR	AGTGCAAATGGTACCAAATCTCCTT	CCGTTTGTTTCTGGGATATTGGAGATA
3 ATR	CACACACATTCTTGTGAGCACTT	GCTCTCACTTCCATGGATTTATTCCC
3 ATR	GTCAACTTTAAACAGCCATCATCAGAAT	TTAATGACTTATTTTGTGCTTCTGTAGTAAACA
3 ATR	TGTACCTAAAAGAAACACAATGCCT	TCCTCCTATTTTTCATGAGTGGTTTCTG
3 ATR	CATGATGTAGGATCAGGGAATGTTCT	GCCAGTGTATGCTACCAAAGTCA
3 ATR	GATGCCTGGGCAGGAGAAAT	GATATCACTCTTCCTTGCAGGAGT
3 ATR	CATGTAACTTCCTGTAATTTTTCAAGGCT	GCAAGCCATTCCTTTTCCTACTGAA
3 ATR	CTCACCAAGTTTTACTGGACTAGGTATT	CTCTGAACACGGACATGTGGA
3 ATR	GGCTTTCAAGTTCCTACAGAAGAGG	CCCCAGAGATAAAGTCAAAGATGATTCT
3 ATR	CAGACAAGTTGACCAAGTATAGAAGCAA	GGCTTTGTTTTACCAGTACTTTTGTCC
3 ATR	ACTATATCCACTCTTACCTGAATGGCA	GTAGAAGATTCAAGCTTTGCCTATGGA
3 ATR	GGTAAGCTCTTGTTAGCTCCATCAATAA	CATGGAAAAAGCAGTACACCTAAGAAAA
3 ATR	GGGCCAATAATTATATTCGAGGTTACT	GTCTTTTTCGAAGAGCATGTGAAGTT
3 ATR	TCACGCATCAGCCTCATTGT	AGAAACCTTTGAAGTTCCAGAAATTGTG
3 ATR	ACCATATTATGAGTCAGGCGAAATGG	TGCTAAAAGAATCTGGACATGAAGTTCT
3 ATR	CAACAATAAACACAACCCTGCATACA	CGGGCTAGTTGTGTTAGTGGATTTTT
3 ATR	ACAAGAATTCTGCTGCTGCAATAAGATA	CCACAGGCACAATCACGATG
3 ATR	TGTCATCTTTTCTTTAAGCCTCCAATCT	GCCATTCAACATGAAAATGTCGATGT
3 ATR	TTCAAGCTTGTAAGAGCATGAATACGA	GGCCTTTGGAAATGGTTGTTAAAATAGT
3 BAP1	GAAATCTTCCACGAGCAGGGT	CCCGCGGGAAGATGAATAAGG
3 BAP1	GTCGCTCTCCAGCTCCAG	CTGGGCCCGTTGTCTGT
3 BAP1	CCTAGGAGGTAGGCAGAGACA	GGAGGCGTTCCACTTTGTCA
3 BAP1	CGGCCTGTGATAGGCACATAG	TTTTTGGGCCCTGACTCTGTT
3 BAP1	AAAATGATACTCCCCCTACTCCCA	CCTTATTTTCTTCCCCGTAGAGCAA
3 BAP1	GCATTGCCAATCGCATATCCT	TGTTCCTTCCGATTCCTGGAATG
3 BAP1	CCTCCTGGGTGCACCAA	ATGACAGCCTGCTGCGT
3 BAP1	GGTTGTAGCGTATGCAGTCAAC	AGTACAGACACGGCCTCTGA
3 BAP1	GAGTTGAAAGCACTGCCGAT	TCTTGGCTGAGAAGCTCAAAGAG
3 BAP1	GGAATTGAGAGGTCCTTCTGGGA	CGTTCCCTTGCTTCACATCTTCT
3 BAP1	CTTGACACCTGCGATGAGGAA	CCATCACCCCCTCCTCTTGT
3 BAP1	GCTCTGAGGTCCACAAGAGGT	CAGAGCTGATTCAGACCCACAA
3 BAP1	GGCAGCTGTGACTCTTGAGAC	AGGTCCTCAGCCCTTAGCTATTTAA
3 BAP1	CCAGATCAGGCAACTGGAGAAA	CCCCGATCAGAGGTGCAATG
3 BAP1	GGCATTCTCAGACACAGACTGA	CCCAGCAGTACTCAGATgatgag
3 BAP1	AGTTGGTGTTCTGCAcgtcat	CCGAGCAGCACTTGTTTGTA

3 BAP1	CCACCCCTAGAATCTGCCTATCT	GATGATACGTCCGTGATTGATGATGATA
3 BAP1	GTGGGCAAAGAACATGTTATTCACAA	CTTCTCCCCTTTGGCTGATCTG
3 BAP1	AGAGAGTATGTTCACGAATCAGAGACA	GATCAAGTATGAGGCCAGGCT
3 BAP1	TGACGGTTCACCTTCAGCAC	TGCCAGGATATCTGCCTCAAC
3 BAP1	GGACTCTCCAGCTGGGACTAT	GAAACGCTCTCGCCCCTA
3 BAP1	CAGTCCTCACTGGCGCTT	CACCTGCTCAAGGGTCTCTAC
3 BAP1	TCCTGGCACTGTCTTCCCTAA	CCCTACTGCTTTCCTTTCCTCATC
3 BAP1	GCCTTACCCTCTGCCAGGATTA	CCAGTGGAGAAGGAGGTCGT
3 BAP1	CACCATCCCCGTCTTCTCTC	AAAGTGTCCTGCACTCTGATGATT
3 BAP1	CAAGAGAATCAAGTAGAGAATCCTGCAA	ACAAACCCAAGCTAGTGGTGAAG
3 BAP1	GAACCCCATTGAGGCTGCT	GTGAGCTTTTCTTGGAGATCCTACT
3 BAP1	CTCAGCAGGGCATTCCAGTTA	GCTCTCAGTCTTCTTCTCTCCTACAG
3 BAP1	GGATACTCTCTGTCCCTCCCAAA	GATTCCTTGTCTTGGTCTCCACT
3 BAP1	GATGAAGGCACTGCAGCCTA	TGTGTATGGGTGACTATTCTTGGTTTC
3 BCL6	TCCCTGTCCTGCCCCAATAA	CTGCAGACCCACAGTGACAA
3 BCL6	GCAGCGGTCACACTTGTAGG	CCTTCCTCGTGCTGACTTTGG
3 BCL6	TGCAGTAAAAGGCCCACATGTT	GTCGAGACATCTTGACTGATGTTGT
3 BCL6	GCTCACGGCTCACAACAATG	GGACTAACAGGATTTTGGCCTCTTTT
3 BCL6	GCTTGAGATGGGAGCAAACAGT	TGAAGAGCCACCTGCGAATC
3 BCL6	CATGGTAAGGTTTCTCTCCTGTGT	CCAGAAGACCTCTCATGCCTT
3 BCL6	AAATCTGGCTCCGCAGGTT	TGGTAATAGGTGAGAAACCCTATCGTT
3 BCL6	AATTCGAGTGTGGGTTTTCAGGT	GAGGAGCAGGCTGCTTAGAAAT
3 BCL6	GCAACAAGAGTGGAAATGTGGGT	TGGACACTTGCCGGAAGTTTAT
3 BCL6	GTAGCAACTCACCTGGCCTTA	GAGATCAACCCTGAGGGATTCTG
3 BCL6	GTGTACATGAAGTCCAGGAGGATG	AAGGGATCACAGATTCGATATGTCTACT
3 BCL6	GCCTTCCTTCTCCCTGACAA	GTCCTGCGGCTCTCAGTC
3 BCL6	GTGGAGGCACATCTCTGCAT	CCAGGTGCTGAGGCTGTTTTTA
3 BCL6	AGACGAAAGCATCAACACTCCAT	TGCAGTGTGAGAAGTGTAACCTG
3 BCL6	GCTGGCTTTTGTGACGGAAATG	CTCGTAGGGAGAATCTTCCTCTCT
3 BCL6	CCGAGGAGGGTCTCTCTTCTTC	GCAATATCTATTCACCCAAGGAAACAATC
3 BCL6	CAGACCCAGACAGTCTCTGA	CTTTCCCCACGAGCCTACAC
3 BCL6	GCATATCACTTCGTGCCTCTTCT	CACTCCCATGTGATAGTGCCA
3 BCL6	GTCAAGGTTCTCAGGCTCCAT	CAAAGCCTGCAACTGGAAGAAATAC
3 BCL6	GCTGTTGAGCACGATGAACTT	GAGCCCCCAGAAATCTGACTG
3 BCL6	GGCTGTACTCACCAGGGACT	ACACCGCCAGCCTCTTATTC
3 BCL6	CTTACTGCTGCAGGACTCTGT	CTTGTGACAAGGCCAGCAAA
3 BCL6	ACAGGGAGGTGGCTGTACAT	CCCCAAGACATCATGGCCTATC
3 BCL6	GTTCTCCACCACCTCACGAC	GGCCGGTGTCTTTCCTCTTTTT

3	CRBN	GGCACTAGAAACTGGAAAAACTAAACC	TGTTTTCAGAGAAAGTTTCATTGTGCAA
3	CRBN	CAGCGAGGCCATGAAGTTAGAT	CCTAGGCCAAAATCATTTTTCAGATGTT
3	CRBN	GGCTCCAGGCAGGAAACTAAC	GCCCATGAACTTGATTATGGCTACC
3	CRBN	AGGTACTGGAGGAAAGTTTATGGAAAAC	AGTGTACTTCCCTTTGCTGTAAACAA
3	CRBN	TGACATTGTTTACAGCAAAGGGAAGTA	AGGAAAATGTGATATTCAGAGAGAGCAA
3	CRBN	TTTTGGCTTCTTTACTATCCTGGTCTTC	CATGTGGTTTTAGAAGTACCCCTCTTTT
3	CRBN	CCCCAACAGAGCATCCTAGTT	CTGCTTGTCTTCCTATTGATGATGTATTG
3	CRBN	ACTGCCAATTTTAAGGAGCTGAATTCT	AGTTCTCTGATGCTTATCTGACTCCTAA
3	CRBN	AGGCTCAGTAGAACAGAAAAAGTACAAG	ACAAAGGTTCAAAGTCCTTGAGCTA
3	CRBN	GTTATTTCTTACCCATCTGACTGTGTTCT	ACAGTTTGGAACAACAGCAGAGATA
3	CRBN	CAAAATCCTGTTCTTCTCGATAGGCATA	CTGTATTTATTGCTTTGCCTACTTTGCAT
3	CRBN	GGCAAATAGCCCATGTCCTCAT	GACACCAGTCTGCCGACA
3	CRBN	CAACTCTGCTTAGGACTCTGGAT	TGCATGAGACACTTACTGTGTATAAGG
3	CRBN	GGCCTATCAGATTCAAGTTGCAAG	CGTGTTCTCCATGTTGATTCTTTGTTTT
3	CRBN	CTTCAACACTGACCACTGCAAT	CTTCAGCTTTTTCACCCTCAAGAAG
3	CRBN	TCTGAATTAAATTCCGCACCATACTGA	AGGTGCTGATATGGAAGAATTTCATGG
3	CRBN	CGTCATCGTGCAAAGTCCTG	TTGCTCTTTAGTCTGTTGAGCTTACATT
3	CRBN	CACTCCCGACTACAGGGAAC	AGCCTCCTTTGCGGGTAAAC
3	CRBN	CCATACAAGGTGGCTGGGTA	CTGTCTCAAGAGAAGACCAATGTTCAT
3	CRBN	CCTTCTGGTATTTCTGCCACCATTTAT	CTTCCCGAATGTGTGTTGCC
3	CRBN	TCTAATTGAACTGCAGACATGGTTGAA	GTTTTGAGTTAGTCAATCTTAGAAATGAATACC
3	CRBN	AGAGGCAATAATTTCCAAAGCAGATCTT	CTTAACGCGATCTGCTCTGTTG
3	CRBN	CATCTTCAGTGTCTGGGATCGTG	ATTTCTTAGGTATGCCTGGACTGTTG
3	CRBN	GCTTGCACAGATCTTACACTGG	CCTCTAAAGTCTGTCTTCATGATAGGTT
3	CTNNB1	TCAGAATGTCTACCCAATACCAGTACTT	GTACAATAGCAGACACCATCTGAGG
3	CTNNB1	AGACACGCTATCATGCGTTCT	GCAGGAATGCCTCCAGACTTAAA
3	CTNNB1	GTGAGGGCTTACTGGCCATC	AACACAGAATCCACTGGTGAACT
3	CTNNB1	CCTTCTCTGAGTGGTAAAGGCAATC	GTTCTCAAAACTGCATTCTGACTTTCA
3	CTNNB1	AATTGGGAATGTTTGCACCACAG	AATAGCTTCTGCAGCTTCCTTGT
3	CTNNB1	GTCCTCTGTGAACTTGCTCAGG	ACACATCTGCTAAAGGCTTTGGTT
3	CTNNB1	GCCCTGTTTGTTAACCATGTTTCT	GAACACTTCACTTAAAACATACCTGCAC
3	CTNNB1	GCTGCAACTAAACAGGTAAATTCTGAGT	CTGCACAGGTGACCACATTTATATC

3	CTNNB1	CTTGTTCAGCTTCTGGGTTCAGAT	CTCAGTGATGTCTTCCCTGTCA
3	CTNNB1	TGCCTTCCTTCTTGCCTATTTTGTT	GATCTGGCAGCCCATCAACT
3	CTNNB1	CCACCCTGGTGCTGACTATC	GGATGATTTACAGGTCAGTATCAAACCA
3	CTNNB1	TGTGCGTACTGTCCTTCGG	CTTAACCACAACTGGTAGTCCATAGTG
3	CTNNB1	CCTCCAGGTGACAGCAATCAG	TGGCAGATTTACAAATAGCCTAAACCA
3	CTNNB1	CCCAGAATGCAGTTCGCCTT	CAACAGCTAGAGATGCTTCATTTTCAAT
3	CTNNB1	TTTGCTTTCTATTCTTCCTTGCTTTGTG	AACCGGCTCTTCTGATAAAAGTTTTAGA
3	CTNNB1	AGCTAATGACTAGGGCCTTATATCCTTT	CATGGCACCAGTTTACTCAGAATTTAC
3	CTNNB1	GGCTCTTCTCAGACATGTGATCAA	CAGATAGCACCTTCAGCACTCT
3	CTNNB1	CGAAAAACTACTGTGGACCACAAG	CTAATTAGAACTGCAGATGCTATACACA
3	CTNNB1	AATGCTTGGGTAAGAAAACATGTCA	GGTTGTGGAGAGTTGTAATGGCATA
3	CTNNB1	TCACCAGTGGATTCTGTGTTGTTT	AGTCTGTCGTAATAGCCAAGAATTTAACA
3	CTNNB1	GGTTGCCTTGCTCAACAAAACAAA	CAATGCTCCATGAAAACCACATAAAGAA
3	CTNNB1	GTTGTATGCCAGTTCTTCCTTCTGT	GATGTGCACGAACAAGCAACT
3	CTNNB1	GGTGCCATTCCACGACTAGTTC	GCATTCCTTTTAGATAGCCAGGTATCAC
3	CTNNB1	CTTAGGTAAATGCTGAACTGTGGATAGT	GCCCTCATCTAATGTCTCAGGGA
3	CTNNB1	AGAGGGTACGAGCTGCTATGT	CAAGTTTACAACTGCATGTTTCAGCA
3	CTNNB1	CGTTTGGCTGAACCATCACAGA	GCAGACTAAAAAGCTAGCTATGTCATTG
3	CTNNB1	CATACAACTGTTTTGAAAATCCAGCGT	CCTGACAAGTAAGCAGGGAGAGA
3	CTNNB1	ACTCTAGGAATGAAGGTGTGGGT	CTTGTCCTCAGACATTCGGAACA
3	CTNNB1	CGACATATGCAGCTGCTGTTT	ACCAGATAAATAACTGCTCACATTTCCC
3	CTNNB1	TTTCAATGGGTCATATCACAGATTCTT	GTAAGACTGTTGCTGCCAGTG
3	CTNNB1	TTTCAATGGGTCATATCACAGATTCTT	TTCCTCAGGATTGCCTTTACCAC
3	CTNNB1	AGACAGAAAAGCGGCTGTTAGT	GGTATCCACATCCTCTTCCTCAG
3	EPHA3	CCTTACATTTTGTTGCTGTTCTGCTT	CTGCTTTTCTGTGTAGCCAACTTTC
3	EPHA3	GATTTCAGTGGCCATTAAGACCCT	CGGAAACACAGGTCTTATGACTACTTT
3	EPHA3	GAGCGGAGCATGGTAACTTCT	AATCAGTTCCCCGAAGCTGTC
3	EPHA3	CCTTCTCAGCTGCTCTGTTCTC	GCTCTATTCGGAGGCACCTTC
3	EPHA3	CAAATCTAGCTACAATTGCGCCTT	AATTCCTTGGCAAACTCATGAACAG
3	EPHA3	CACATACATATGAAGACCCTACCCAAG	CTGAGCAATCGTGATTCACATACAGTA
3	EPHA3	GAAGACAGGCAAAGTTCTTACATCTCT	GGAACCATTCTCCATGTATTCTGTGA
3	EPHA3	CTAGGTAAGCCAGTTATGATTGTCACA	AGGGTGTCTCTAATGTTTTCAGCAATTTA

3 EPHA3	GTAGTAGAAAACAGAAGTGAGGCTCAT	AACATCTTTCCGGCCTCCTG
3 EPHA3	CCTCAGTTATCCTGGACTGGAGTT	TGAGTCCAAACTGTCGAGGGA
3 EPHA3	GAATATAAAACAGTGTGAGCCATGCAG	CTCAGCTCTGACACCCCATTAA
3 EPHA3	ACTAACTACACCTTTGAGATTGATGCC	TTTTCCACCCAGAGTCAAAACCA
3 EPHA3	TGTTCCCTTCTCCCTCTGTTCTTA	GTCCGATCTTTCTTAATCGTCAGGA
3 EPHA3	CTCTCTTTACAGCTCCATCACCTG	ACAGCCCAACCCTTAAACATTGT
3 EPHA3	TTTTGAGCAACATCAACAACAGACAAT	GAACTCACCCCATGTGATGGA
3 EPHA3	GGAGCTGGGCTGGATCTCTTA	GTCACCAGAAGTACAAGAGTTCTCTTT
3 EPHA3	TGAATCAATCAACTGTTCCATGTAGATGT	AGAATAATTGCTACTGCCGCTGAA
3 EPHA3	CCAAGTGGTCATGATCGCCAT	CAATCAGTGGCAGCAAACAAAG
3 EPHA3	AGAAATTCTGTCCGGTTTCAGATTATG	CGTGAACTTGCGGTAGGCTATA
3 EPHA3	AATCAGGTGGACATCACCAGAAG	TCCTGATTGGACATCTCCCAGTAT
3 EPHA3	AGGTGATGTCTTATGGAGAGAGACC	GCCACCCAGTTACATTATGTTCCA
3 EPHA3	TCTGCCTCACTCTCTGTTTCTCTT	ACATAAATCTTCTGAGCTGAGTTCCTG
3 EPHA3	ATCAGGTTTGAGTGGTTCACTGTT	TGTCAGGCTTGAGGCTACTGA
3 EPHA3	AAGTGCCCATTTACAGTGAAGAATCT	GGAACACTTGCCAATGGGTACA
3 EPHA3	GCAAGAGGCACAAATGTTACCA	CCCTCCTCTAGACTGCATTGAAATAAT
3 EPHA3	CAGTACAGAAGGCGAATGGCT	tgagttGAGACTGAGTGCATTTCATTTA
3 EPHA3	ATTGGCTGAGAACAAACTGGGT	GCTCTCGAAATTTCACCCCATGA
3 EPHA3	CCTGTACTACATGGAGTCTGATGATGA	ACAGGACCTACTTCTCTAATCTCAGTG
3 EPHA3	GGGACCGTATTCTGAAGCTCAA	ACCGTGTCTGGAAACATAGCC
3 EPHA3	GTTCCTGCCGATGTTAGTGTCA	CCAGACACCATTAAGCCAGTCAC
3 EPHA3	TCCTCTTATGTGTTCGCTTTCCTTG	AGCTAAAACGGTGAAGCAAAACAAAA
3 EPHA3	TCACTACCTTCCGCACAACAG	GCCAAGAAAGTTCAAAAGAATCCTTCA
3 EPHA3	GAGTTTGAAACTAGTCCAGACTGTATGT	TGCAATGTCCCAAGCAATATACCTTAAA
3 EPHA3	CTAAGTGACACTTCCTGAAAACTTCCT	CAAACTTGGGTCTGTTGTTCCTG
3 EPHA3	CTGGACTGCTGGCAGAAAGA	AGTGAATGCCAGATAACAAATTGAATGTG
3 EPHA3	CATCGTGCAAATTGTGCTAATTGAAAAT	CATTCTTTGATTGCGTTTCTAGAGCTTT
3 EPHA3	CCACAGAAGAAGATCATCAGTAGCATT	CCAGAATTATTGTCTGTCTGCAGGAT
3 EPHA3	TCATTTGCTTCTGTAAATTCTTGGAGGA	CCATCCAATGCCTTGTAGAAACCT
3 EPHA3	CATTTTGTTTGTAGCTTGTCGACCA	AAGTAATTATTCTCACACCTGCAGTTCA
3 EPHA3	CAGTTCTACTCAGGAAGATGGTTCAA	AAAACAAACATGGAGGCATGGG
3 EPHA3	CTTGAGTGCTTAGGACTAAAGTGTGTA	CATCCCCACTAGCTGAATGACA

3	EPHA3	CAGAAACACGATGCCCAGTTTAC	AAGATGTTCCGAGCAGCGA
3	EPHA3	AGTACCTGTCAGACATGGGCTAT	TTGTATAAGCAGCTTCTGGGTCATC
3	EPHA3	GTGTAAGGTTTCTGATTTCGGACTTTC	GACAACACGTTTTGGGTCATGAT
3	EPHB1	GCAGTCACTCAATCACACCTGTT	GTGTTGACAATCTCCGCAAACC
3	EPHB1	AGCTCATGCTGGACTGTTGG	CATGAAACCAGCCATATTTAGGGATCA
3	EPHB1	GATCCCAATGCCCTCTATGTCTTATT	TGCACACCAGGTTACTGTTGAC
3	EPHB1	CCTGGCTGCTAGGAACATTCTG	ATGCTTCTACTTGAGAATGCCAAGAA
3	EPHB1	CTCCCAGGAATGCCCTTAGTG	TTCGTTGGGATCCTCGTAAGTG
3	EPHB1	GGATGAAGATCTACATTGACCCCTT	AGATGTTTGGACACCAGGAGAAC
3	EPHB1	ACTGTCCATGCATCTCTGCAAT	GGAAGCTGTCCCTGTACTGGA
3	EPHB1	GGCTCAGCGCCATCAAAATG	ACCATCTGCAATAGCAGGGAAG
3	EPHB1	GCCTGCCTTCAATCAGAAACCT	ATGACTTCCCACATGACGATCC
3	EPHB1	CAGCGACGTTTGGAGCTATG	TGCCTTTGGGATCCCTTCAATG
3	EPHB1	TCTAGCCTCACTGGACCTTCTT	CCGGCACTTTTTGCAGATGAT
3	EPHB1	GCGGGATGATGTGACCTACAAC	GGCCTGGATGTCAAAGGTGTA
3	EPHB1	GTCTCCATCAGCAGCCTGT	GCACAGAAGCCTCCAGACTT
3	EPHB1	GATAGTGACCCTTTCCCTTGCTT	TGCCACTGAACTTGCCGTAG
3	EPHB1	GGTATATGTGGTACAGGTGCGT	CATTTTTGGCTAGTGCAGCATCT
3	EPHB1	TGCTGGACTGGTGAGCTCTTA	TGCGGATGGTGTTCAGGTTTT
3	EPHB1	GGAAGAAGTCAGTGGCTACGAT	GCTGCAGTCTCTCACAGTGAAG
3	EPHB1	CCCATCGCATCTACACAGAGAT	CCTCAGACCAGAAGGCTGACT
3	EPHB1	GACTCTGTCATTGCCACCAAGA	ATTCCGAGTAAGAGGCCCAAAG
3	EPHB1	ATGAAGGTAAACACAGAAGTCAGGAG	TCATAGTCTCTGGAAACACTGCAAAA
3	EPHB1	GCCTAGATGGAGGTACCTGGA	CACCGGGTCTCTGCTTTCTAC
3	EPHB1	AGAGTTCAAGCCGAATGCCATT	CCATGACAAGGTGATGCTCCTC
3	EPHB1	GCACCAAGTCAGTGCCACTAT	CTAGCAACCATCCATGGAACAGT
3	EPHB1	TGACCTTGAGGGCCACTGTAT	CTGAAAGACGAATGGTTTGATACTCAC
3	EPHB1	ACTAAAGTGACTTCTTTTCTGGCTCTTT	TCCTCTCCTTCCCCAAGAAACA
3	EPHB1	GGCGATGGCCCTGGATT	GATGCCTGCACCGCAAAG
3	EPHB1	TGGCTGCCACACTTCCATTAA	CGGTAATAACCGGTCCGACAG
3	EPHB1	GGCGTCTCCCATCTGCAC	TTGCACCATCAGGGAACCAA
3	EPHB1	GGGCACCATTGTTCTCCATTAGA	TGATGGCCACGTAGATTTCCC
3	EPHB1	GTTTGAAACTGCCAGGCAAGA	CCCTCCAGGCGAATGATGTTAG
3	EPHB1	ATGGGCCAGTTCGACCATC	GCATCCAAGTGCATCATCAAACC
3	EPHB1	GTGTCCCAGCATTGTGCAAAA	CCAATAGGCACCATCCATTCCC
3	EPHB1	GCCCATCAAACTCTACTGCAAC	GAAGCAGAGGCTCCAAAGCTTA
3	EPHB1	GGGAGGATTCTAATGCCTCTTTC	GCAAGTGAGAGTGGAGGACCTA
3	FANCD2	GCAACTCTTAGGTTGTGTACTAACTGT	CTGGTCTAGAGAGTGAAGCAAACTC
3	FANCD2	CCATTCTGTCGCTGGCTCA	GTTCCCTCCTTGCCAAAATAAATGTT
3	FANCD2	CTGGCAGGGCTTGTGTTCTTA	CCAGGGTCTTTTTGAGCAGAGG
3	FANCD2	ACGAGACTCACCCAACATGTG	TCACTAGCAGGATCCAAGGCTTA

3 FANCD2	GAGAAGGAAAACTATGGTAGGAAACTGG	AGAGTTATGCTGGCACAAGCAA
3 FANCD2	TCCAGGTTTTATTGGCTTGCACTA	CCTCTCGGAACCAGTTGAGAGT
3 FANCD2	CATATTTCTTACTCTCAACTGGTTCCGA	GGTTAGTGCTTGGTGAAGACTTCTA
3 FANCD2	ACTGGTATAAAGTTGAGTGGGCTAGA	CCTGAAACTGTCTTCATCCTCAATGTAA
3 FANCD2	AGAATTTGTTAGTGGCCTGGAGTC	ATGAAGTTGGCAAAACAGACTGAATTT
3 FANCD2	GAGGAAAAGCTGCTGTTTCATTGT	GATCGAATCTTATTTCTTAGCACCCTGT
3 FANCD2	GGTGTTTGACCTGGTGATGCTT	GAATGTACTCTGGAGCAGCTGT
3 FANCD2	ACAGTAAGGGAAGTATTTGGCTGTG	TCTGCAGTTATTTGGTGAGATGTGT
3 FANCD2	TCTTCCTTTGTATTGCCTGTAAACTCAA	CTCACTCTCATCTGCTGTGCT
3 FANCD2	AGATTAAGTCCCAAAATTCCCAGGAG	TGCTCCACCAACTTAGAACAATTAACAA
3 FANCD2	GCTAACCCCTCTTACCTTGACTTC	GGGACAACAATCTTGGCTATGTAGTTAT
3 FANCD2	TTGAGATCATCTCCTAACTCCCTATGTC	GTACATCAGGGTACTTTGAACATTCCAT
3 FANCD2	TTTGTTGCTGTGACTTCCCCATA	TCGTCACAAGTCCACAATGTAGAATAG
3 FANCD2	ACGTGGAGTAATACACCTATGAAGTGT	GACTTCATCCCATACCTAGTAGACCA
3 FANCD2	AGAGCTGGACATTGAGGTCTTCT	TCAGTGTCACAGTGTTCTTTGTGAA
3 FANCD2	CCGTATTTCCGGTTACTGAGACTTTG	TCCTCCCCACATACACCATGTATT
3 FANCD2	AGCATTAAAACAAGGAAAGCAAAGTGG	ACCTCTTCCATCAAATGTTCACAGAAA
3 FANCD2	TCCAGCATTTTCATCTTTCTTCATCATCT	ACAGAAGTCAGAATCTCCACGTGTA
3 FANCD2	TTTAGAATTCTCTGCAGCACCCAA	ACAGGGCCCAGTAAATCTCTCA
3 FANCD2	TCAGCTCTGCATTTCACACGTA	CAGGTTCTCTGGAGCAATACTGA
3 FANCD2	CCACCAAGATCATGCAGCTGA	ACTTAGATGATGACAGAGGGTTTATCCA
3 FANCD2	GGGCCTTTCAGTGAGATACCT	CATATCTTTGTTCTCTGCTCTTAGCTCT
3 FANCD2	GGTGATGGGTTTGGGTTGATTG	CAGCTTCATTCCCACTGCAGATA
3 FANCD2	TCTGGCACTGATGGTTGCATT	TGGGTCCCAAAAATTATATAAGCACAGA
3 FANCD2	AGCCAATGGAGTGACTGAATTTCAA	TGCTGAAATAGCAGTAACAGTATGAGG
3 FANCD2	GCCCAGCTCTGTTCAAACCAT	TGTTTCTGGAAAGCTGAGTCTTTTCTAC
3 FANCD2	TGATTATCAGCATAGGCTGGAAACTG	CCTTGTGTCCAACTGGAAGGTT
3 FANCD2	AGGAAGATGTTCTGAGCTTACTGGA	GCAACTGCAGATTAAGCCAACAG
3 FANCD2	ATGTTAGCTGCTAGCCTCATTGTT	GCTGCTGGCTTCATTCTGTTTG
3 FANCD2	TGGGAAGATGGAGTAAGAGAAGTGA	CCTCCAATCTAATAGACGACAACTTATCC
3 FANCD2	TTTAGGTTCGCCAGTTGGTGAT	TCTAGGTGTGATGGGATATAAGAGCATAC
3 FANCD2	GGACACATCAGTTTTCCTCTCATGAT	GCTGTCATTTTCTTCAACTTCATTAGCA

3 FANCD2	GCTAATGAAGTTGAAGAAAATGACAGCA	GAAAACCCATGATTCAGTCCTGAACTA
3 FANCD2	GGCAGGAACTCCGATCTTGT	AGGCTTCTGAAATGGTTTTCTCATATCT
3 FANCD2	GGTCAGAGCTGTATTATTCTCCTCTTTG	ACATCTGTCACTAATTGCCACTCATT
3 FANCD2	ACTTCTGTTTCCCGATTTTGCTCT	GCCTCACACATTGCTACAGGAAATAAA
3 FANCD2	GAATGAGGTCAAGTTCCCATATGTAAGA	GGCTCATTGGAAGAAACCAAACAT
3 FANCD2	GGTGACACAGGTTTGACTTGACT	GGGCAGATCTCTGTTGGAGATG
3 FANCD2	GGAAGCCGGAATATTGGATTCTCA	AGCAGCTTCTAAGTCTAGGCTTCT
3 FANCD2	GATCTTGCTTTTCATAGACATCTCTCA	TTTAACCGAGTGAGCACCTTCC
3 FANCD2	TGCCTTCTGCCAGGAAACAT	AGTGCTTTATTGCCTCACTACTGG
3 FANCD2	TGCATTTGTTTGTTTTTCTTGTCTCCTT	GCAGAAGCTGTTGATTTCTCCAAAATA
3 FANCD2	TGTGCTCTTTATCTCATCAGACTTTTGA	AAAATGTCCTGGGAAGCCCAA
3 FANCD2	ACCCACTATGAATGAGCAGAAAACC	CCTGTGTGAGGATGAGCTCTTTAC
3 FANCD2	TTTCAGGTCAGAAGCCTAGACTTAGA	CTTTCACTCCTGGTCCATCAACTAC
3 FANCD2	TTTAGCTGCTGAGAATCACGGT	TCTCTCCACACTTCCCACATACTTA
3 FANCD2	TTTTGTGCAGTATTAATGCTTGCTGTT	AAAGTCCATAAGCCTACCTTGCC
3 FANCD2	AATGGCTTGACAGAGTTGTGGAT	GGAAAAAGCTAATGGATGGATGGAAAAG
3 FANCD2	CCCAGAGCAGTAACCTAAAATGCTT	ATGATCACAGGCTGGCAGAG
3 FANCD2	TGCGTATTCCTGAGCTGCAA	CGGCAGGAGGTTTATGGCAAT
3 FANCD2	ACTGGAAGAATACGACACTCAGGAT	GTGGAATTCCCCATGAACATTCAAA
3 FANCD2	TCCTGGAACTAATCCTTTCCTCCAT	GTCCTATGCTTCTGCCTACACTAAC
3 FANCD2	ACCATTCTTCCTCTTTGCTCCAG	CAATTGGCTTTACCAGGGCTTTT
3 FANCD2	CAACCTGATCCAACATGAAAAGCTG	CTATTTTCACACTGCAACAGAAAGACTT
3 FANCD2	CCTCTGGTTCTGTTTTATACTGTTAGCT	TCTATGGCCTTCAGAATGCTCTCT
3 FANCD2	GCAGTATCTACCTGGAGCACAC	AGGGAAAAACTGGAGGAAAGAACTC
3 FANCD2	CGCATGCTTTTCCCGTCTTCTA	GTAATCCAAAGTCCACTTCTTGAAGTGA
3 FANCD2	TCTTCTACCTCTAGGCAGTTTCCAA	CTGAAAGTGTGTCAGAGGAGGATG
3 FANCD2	CAGATGGCAGCAAGACATCCT	AGCTGGGTTTCTGTTAATCTCACTG
3 FOXL2	GGCTCCTTGACTGTGCGA	GTGCGCCCCAACTCTTTG
3 FOXL2	GTCCCTGCATCCTCGCAT	GCCTGCAGTTCGCTTGT
3 FOXL2	GCTCTGCACGCGTGTGTA	TCCCTCACCCATGCCCTAT
3 FOXL2	CTTGCCGGGCTGGAAGT	CGCAAGGGCAACTACTGGA
3 FOXL2	GCGGCCACGAGTTGTTGA	CGCATGAAGAGGCCCTTCC
3 FOXL2	CCTTCTCGAACATGTCTTCGCA	TCGCGAAGTTCCCGTTCTAC
3 FOXL2	TTGCCAGCCCTTCTTATTCTTCTC	GCAGAAGCCCCCGTACTC
3 FOXL2	GCGATGAGCGCCACGTA	CTGCTGGCCCCAGAGAC

3 FOXP1	AGCACAATCCAAGCTAGGCTAAG	TCACAAGTGTTTTGTGCGAGTAGA
3 FOXP1	TCCATACTGCCCCTTTAACGTTT	CTGAATTTACTGCTGTTTTTGGAGTCTT
3 FOXP1	CCTGTGCCTCTGCTGCATATT	AGTCGACTGTGTTTAACTTTGAGTTTTT
3 FOXP1	AAAGGCAGTTTTGGACCCATCT	GCAACAATCACAGCAGTTTGGAT
3 FOXP1	GGAGACACATGTCGTGGTCAG	TTTGAAGAGGACGGAATTAGCTGTTAAT
3 FOXP1	CCCTTCAGAGAATGTCTGCACAA	GCCAGAACCCATCTACAGCAAG
3 FOXP1	TGGAGGTGGAGGAAGGACAATT	TTCCCGTGTCAGTGGCTATG
3 FOXP1	TCTGCCTTTGGATTTCCGTCTT	CCTGAGGTTGCCTACAAAGGG
3 FOXP1	GGGAGTGATAACTTGAGGTGTCAT	TTTAAGCAGGCTGATGGCAGT
3 FOXP1	GGGATGAGTCAACCATGCAAAG	CTTTTGGACAGTTGATGATGAAGAGTTT
3 FOXP1	TGAATATGGCGGCCACGTTT	CCTTTGCTGCTGCTCCTATCC
3 FOXP1	AAAGACCTGTTGGGTGGACAA	CTATACACTACCGCTTCCATGGG
3 FOXP1	CTAAGTTGCCCAGAGTGGGATTT	CCTTGTCTTACATACCCTGTGTCTTC
3 FOXP1	CCTCAAAACTCAAAGCTCCACCA	GATGACCCACCTGCATGTGA
3 FOXP1	CGGCTTTGGGTTCTGTAGACT	AGAGCCATGCATCTTTATTCAGAGAAAT
3 FOXP1	GTAGCTTCCTTATAGCACAACTGCAT	CTGGTTCACACGAATGTTTGCT
3 FOXP1	CGCGTTGCGTCGGAAGTA	ACGACTCTGCTGATTTGGTCATAAATT
3 FOXP1	GCATGCTTGCATACTAAACGGTTTTT	GCAGAACCAAGAATTTTATAAGAACGCA
3 FOXP1	GTAAATGGTGGTCTAACTTCTGCGT	TTGCCTCACGTAGAGTTGTAATCTG
3 FOXP1	CGTAGTGAAAATCCTCCAGACTGT	GACCATGACAGAGATTACGAAGATGAA
3 FOXP1	CACTCCATGTCCTCGTTTACTGG	CCCTCACTGAACCTCTGCATATTTTT
3 FOXP1	CCAAGACTCCAAAGCCCAGTA	GTTCAGCCATCCAGAATGGGT
3 FOXP1	GCCGCACTCTAGTAAGTGGTT	AACAACTGCTTTAACAGCTGCTTTT
3 FOXP1	GCCACTAGATAGTCCTCTGGGAT	CTTACCGTCCTCTCTTTCCTTTGTAG
3 FOXP1	GGACCTTCCATTCAATAATGAGTACAC	GAAGTACAGCCCAATGTAGAGTACAA
3 FOXP1	CTCTAACTGCTGTACAACCTGCAT	GCCTAGTTTATGCTTAAACATCCTGGTA
3 FOXP1	GCATGAAAGCAGTGGGAACCAT	GAGACTAATTTCAAGCCCTTGGGA
3 FOXP1	GGACCTGCAGGACTTCCAAC	GCGATCTCGCGATCAGTGTTT
3 FOXP1	AAAAGGCATTTTATGAGCAAAGCATGA	CCCCCATAGCCATCCTCTCTAT
3 FOXP1	GCCACTTGCATACACCATGTC	CCTCGTCTGGTGGAATGGTT
3 FOXP1	CCAAAGTCTTCACAGAGCTATGCC	GGCTTTTCAGCAGCAGCTTTTAC
3 FOXP1	GCTGCTGTAACTGCTGCATCT	CCATACTTTTAACCTTCCTTGGGAAGTG
3 FOXP1	TGGTTCTGCGCAATATCTGCT	GCAGGCGGTACTCAGACAAAT
3 FOXP1	GACGAAATGGGCACGTTGT	CATCGGAGGCTTCTCCACAG
3 FOXP1	GGGTCGTTGGAGTATGAGGTAAG	CGCATCCTCTGTGTTACTCCTG

3 FOXP1	TGGTAAACCAAAAATGCACTTTCC	AAATCTCCCAAGAGGAATGACAAACA
3 FOXP1	AAAGAATTGTTACCTGAAGAGCTGGT	CTGTGTAGTAACGCAAAGGATTTTCTTG
3 GATA2	GGCGCCTGGGTTCTCATC	AGCTGCTGCCTCCAGAC
3 GATA2	TGCGCGGGTTCCATGTA	TCTGCACCCAGACCCTGA
3 GATA2	CCAGCCCCTGGGTACAC	GTTTGCCCTGGCTGGAC
3 GATA2	GCAGAGAGGGCTGCTTTG	CCCTAGCTCTGCCTACCCT
3 GATA2	CAAAGCGTCTGCATTTGAAGGA	ACTACCTGTGCAATGCCTGTG
3 GATA2	TGCCCATTCATCTTGTGGTAGAG	CCCGAGAACTTGCCGGTTAA
3 GATA2	CTTGCCTGGCAGCACAAA	CACCTGTTGTGCAAATTGTCAGA
3 GATA2	CGCCATAAGGTGGTGGTTGT	CCTCCTTGACTGAGCTggtg
3 GATA2	CTCCCCTCTTCCACGAAGT	CCCACCTACCCCTCCTATGTG
3 GATA2	GAAGAGTCCGCTGCTGTAGTC	GTGTCACTGACGGAGAGCAT
3 GATA2	GGGACTGCCACTTTCCATCTTC	GCCACCCAAAGAAGTGTCTCC
3 GATA2	CCGTGGTGCTAGGGTCA	CCTGGAGGCCCACTCTCT
3 GATA2	GTCCTCGACGTCCATCTGT	CTGGACACATGGCACCTGT
3 GATA2	CAGGATGTGTCCGGAGTGG	AGATGTCCAACAAGTCCAAGAAGAG
3 GATA2	GCACTTTGACAGCTCCTCGAA	GGAATGTTGCTGGAGGAAGGAA
3 ITGA9	cccCCTGTGCCTCATGTTATATT	TCCGAGGTTGGAGATAGAGATGTTT
3 ITGA9	GGCTGTGAAGAACATCTCCCT	CCAGATGGAACAACTGCTCTTTTTAC
3 ITGA9	TCTCTCTCCTTCCTTCCCCCTA	CACCTCTAAGGGTTGCCAAAATCATAT
3 ITGA9	GTCCTTTCTGACATTTTGACCTCTCA	GACACGTCTCCTCCATGTAAGT
3 ITGA9	CATGGGTCAGGTCACAGAGAAG	AAAATAGTTTCCACCAGCTCCAAGT
3 ITGA9	GCTCCTCTCTTCTCCCACAGAA	GATGTGGTCGGCTTCATAGTAGAT
3 ITGA9	TGTGCTCATCGCTGGAAGAAC	GGGATGAGGAAAAATCAATCCATGCT
3 ITGA9	GGGATGACAGATTTCATGCTTTCCT	GAGGTCTCCAGGAGTGGGTA
3 ITGA9	CCCATCTGTTGGTGCTTTTTCT	GGTCCCGTCTGTGTCCCTTA
3 ITGA9	GTGGCTTTTTAACCCATAGCTGTTT	CAGTTGCCATATCACTCTCACACTAATT
3 ITGA9	GTGTTGGCCAGCGTTCATTC	ACTCCTGTGTTGTGGCAACC
3 ITGA9	GGTTCTACCACTCCTGCCTCTA	GATGCAGGGAGTTGGGTTTTTC
3 ITGA9	GAGAAGGGAAACTGCTCTTTCCA	ACCCCAACCACTAAACAAATCCTTAC
3 ITGA9	CTCTCAAGGCCCAGCCATTAT	CATGAGCTATTTCCACCACCCT
3 ITGA9	TGAAGGTGGATCCTGCCCTA	GACTGTCTTGACCCTCAAATTCCA
3 ITGA9	GTGTATGGGCTTCAGCAAGTTTC	CTCACCCTGAACAGCACAATTC
3 ITGA9	TGTTTATTGTTTCAGCGGAGGGT	CCTGATTCTTTTGGGCAATCTTTTGTC
3 ITGA9	CAGTTCTCCGCTGGAAAAAGG	TCTCTCAACTTTTGGGCCATTATTTCT
3 ITGA9	GGATTTATCCTGTTGGTCTAGTTCTTGT	CTGTCCGTCGTGACACTGAG
3 ITGA9	CGGGCTCCATCAACATCACAG	CCACTAGCCCACTCCAGCTA
3 ITGA9	ATTGCATGACAGGAAATGGAGGT	CCAGATGAGAGTCGATTAGGGAAAG
3 ITGA9	CAGGGTCATCTGTCAGCATCT	TGGCTCAAAGCTGAAAGCAATAGT

3 ITGA9	TGGATTTCCTCTGCCTGGAATTG	CTGTTAGGCAAGAAATTCCTGGTTTTT
3 ITGA9	TTCTGACTTTTCTTCTCAGGACTGTG	AAGTGCGGTTCATTAGGGCTTA
3 ITGA9	GAACATCACTCCTCAACCCAAGT	ACTTACCAACACACGGCCATC
3 ITGA9	CCGCGATGATGAGTGGATGG	cagccttcCTAACAGATCATTCATTCT
3 ITGA9	tttCCATTTTCTCCTCCATCCTGTTT	CCATCATTGTCCAGATCGTCCAG
3 ITGA9	GCGCACTTTGGAGAGAGCATT	CAGGAGCATGCCACTAGAGAT
3 ITGA9	GACTCCCAGGAGAGTGCTGATA	CTCACCTTTTTACCTGATGCTTGAAA
3 ITGA9	GACCGAAGATCAGGCACCTTAATT	GACAGCACTGCCATATAAACACA
3 ITGA9	GTGACTGTGGTCTTTTCCTGTGA	CTCATGTGCCTTTAAAGAAAAACAGCTT
3 ITGA9	ATTCTTCAGCACTTCTAACTCAGCTT	ATCAGGTCATGTGACTGGTGTG
3 ITGA9	AAAGGAATTGGCCACTGTGTACT	GCTCTGAAGGAAACACCGACATAC
3 ITGA9	TGTGAGGAATTTCTCCTTTGTTGTGA	GTTCAGCACTTTGATGGTTCCAG
3 ITGA9	TGCTCCAGGGTCATTTTATTGGG	TTTCTTACCTGTTCTCTCCCTGAGT
3 ITGA9	GAATGCCTGCTGATGCCAAAT	CTGGTCCCATAAATCTCTCATCTTTGAA
3 ITGA9	CCCGTGGTTGGGTGAATACTT	CCCAGGAGACTTCACTGAAGG
3 ITGA9	CACCAAAGGCAGATTCCAAATACAG	AAGAAGAGCAAGGTATAAGGGTACAGA
3 ITGA9	GCTACGCAGTTCTGGAGCATTT	GACATTTAAATCGCCCCCTCG
3 ITGA9	CCCTTCCCTTCTTTTTCAACCGT	CCCAGCTATTCCACAGATGTTTCTC
3 ITGA9	ACACTGAATGCTTTGTTGCAACTC	ATTTCCTCTGTTGATGTAGACAGTGAC
3 ITGA9	TGAGATCAGGGATGAGGGACAG	ATATTTTCATCTCCTCTGGGTAACATTG
3 ITGA9	GTTTTCACTGTTGCGTTGCCT	GACCTGCAATCACCAGACTCA
3 LPP	GCAGTATCTAACCAGAACTCTCTCTTC	GCAGGGCTGTCAACCAGAC
3 LPP	CACGAGTACTATAACGTTGCATCCT	GGAGGAAAGTTTCCAGAGATAGATGG
3 LPP	CTCTAGATGATTCCAGTGCCCTT	TCCCTAGGATGGCTTATAAACATGACT
3 LPP	TGTTAACATGTCTGTGTTGCTTCCA	AGATGATTGTGGGCTGCACTAA
3 LPP	GGAGGCCTTGAATAGAAAGCTCA	GTTGCAGGTCTTGCAGAGGAT
3 LPP	GAGATAACCAAGGCTGCTACCC	TCTTTTTCTTGTGTTCGTTCTTCTCAGT
3 LPP	GTTGGCAGTAATTTTTGCTTTCTTTCTT	AGGCTGTGGTTTCAATGTAGACTTC
3 LPP	CCCCCTCTAACAGCAACCAA	CTGACTTCACCTGCACATTAAAGG
3 LPP	CCAACCAGGGACGCTATTATGAA	GGATACATCCCAGGAGGGTTCT
3 LPP	ACCAGGGTACACTCCTCCTG	ACCCCTGTATTCTCCTCCTTATGT
3 LPP	CCTCCACTTCTTCAAGGCCTA	GTGTTGAAGGAGGTGGACACT
3 LPP	CTATAACACTCAGCCAGTTCCTGT	CTGGCCCTGCTGCATAGTAG

3 LPP	TTGTAAAGATGCCTGTCATGCAGTA	TCACGCAGGTGAAACAGTGAG
3 LPP	CACCGGGAAGGCCTATCATC	GTGACGAGAGAGTAGGTTGCC
3 LPP	ACTTTGGCTTTAGCAGGACTGAA	CAGATGATGCAGGTAAAACAATCCAC
3 LPP	GCCATGGATCAGGTCTTCCAC	GCTTGAGGAACAAATCTGGAACTTAC
3 LPP	CAGTGTTTCTTTTTCATTGCAGATTCCA	CTTTTTGGGTGGCTGTTGTGTAG
3 LPP	GGAACCCCAGCATTTCAGTGT	CCAAGTAACCAACTCTGAAATCCCA
3 LPP	GACTGCCTTGGTTGATGGTCTA	CAGGTGGATTTTCCATGTCATACAG
3 LPP	CTTGAGCACCTGACCAAAAAGATG	CCAGTGGAGCTGATAATAGTTCCAA
3 LTF	TTGTGATGCCAAAGACTCTGCT	GCCCCACTAGTTTCTCTTCTGTAATTAT
3 LTF	GCAGTGAATGGCTGAGGCTTT	AAATCAGTTTCTGAATCTCTTGCTCTGT
3 LTF	TTGACTGTTGACTTCACTTTAAGCAGA	GTAACCAGTGGAGTGGCTTGAG
3 LTF	GCAGGTCACGCTGCCTT	AGCCACCTGCGGTCTTC
3 LTF	TCACTATCCCCCAGCCATCTTA	AGCTGAACGAACTGCAAGGT
3 LTF	CCTGTGTGGCAGGACTTCAG	TCTTGTCCAGGCTAAGAACTTTCAATT
3 LTF	gctcaaAGGTCAGAGTCATGATCAA	CCCCTGATGGTTTCTCTTTTCACAG
3 LTF	CCGCACCTTTGGAACTCCTT	GACACTAGCCTTACCTGGAACTC
3 LTF	GCAGGACTTCTTGCCTTTCACA	GACAGCTCCACTGGAATCCAT
3 LTF	TGCATCAAACCTCCACGATGA	GCTACTTCACTGCCATCCAGAAC
3 LTF	GGGCTCACTCACTTTTCCTCAA	CAGAAAGATCTGCTGTTCAAGGAC
3 LTF	CCTCGAAAACCCAATGGCAGA	CCTCCCTATTTACCATTGACACCATAAT
3 LTF	GGGATGCTAGCTAGGGTCTAC	GTCTGAAACCAAAAACCTTCTGTTCAA
3 LTF	GCCAGACACTCAGTGTTGTCA	GCTACTCACTGTCTGCCCTTTT
3 LTF	CTCCCATGACCCAGAGGGAATA	GCTGGAGATCTGCTCTGTCTTTG
3 LTF	TTTTTACAGTTCCTGCCACTTCCT	TGATGTCTGGCTTGTCTTTATTCTTTCA
3 LTF	ACTTGGCCACTGACGAGAAG	ATAAAGGACAGTTCCCCAACCTG
3 LTF	GCACATTTGTTTTCCCCTGTCC	TCACACTCTGTGGTCCACTTCT
3 LTF	GTTCCCCCAGTCTTAATCCACA	CTGTGACCCTTGATGGTGGTT
3 LTF	CAGGCCTGCCTCGTATATGA	CTTTTTGAGGTCCTGAAGTCCCA
3 LTF	TCACCTAACATGAGCCCACAC	GCAAACGGAAGCCTGTGACT
3 LTF	CAAGATGGCAGCTTCTAGCCT	CGTGGATGATGCCACCTTCTT
3 LTF	CTTTTTCCATTTCTCTCTCCCTTCCA	AAAATGCTTCCAATGGCAAAGGAA
3 LTF	GGGCCACGCACTTTTCTCATA	TCAGTGGTTGAATCCTTACTCCTTG
3 LTF	ACAGGTCACATCTGGCTCCTTA	GAGAATAAGTGCGTGCCCAAC
3 LTF	GCCGTAGTATCTCTCGTTGCT	GACTCAGGTTTGAAGAGCTGACT
3 LTF	GGAGGACCGTGGGTGAAGATA	TTACTCTGCCCAGACAACACTC
3 LTF	CTTTGAACTTGTCCACTGGCTTC	GGGAACTATAGCTCGAGGTTACT
3 MAGI1	ATTCAGTTACACGAACACTAATCAAaga	AACCTAATCAAGGAAGCGGGAAA

3 MAGI1	AAGTAGCTTGAGAAAGTTGGAAGGAAA	TCAGTTTGCTAATGGGTTGCTCTTATAT
3 MAGI1	TGACCCACCATATTCTGGTACTGA	CTCACTTAGTTTTCTTTTGCTTTGGCA
3 MAGI1	GTGTCAGCGCTCAGATTTCAC	GAACCCGGTTCTAGAAGCCA
3 MAGI1	GGAATGATGCGGAGGGTAACTG	CTGTGGCAAGCTGAAAGTAGGA
3 MAGI1	CAGGTCCATTGAGGGCAACAT	CTAAGCAGTCGACCCCGAAG
3 MAGI1	GTGTTCCAGAACCAGTTGTTGAG	CTCCAGCAGACCATAAGGGAT
3 MAGI1	gctCAAGCTGCTTCTTCCGTT	CATTTTGCTGGTGGGATGAACT
3 MAGI1	GAACATCCATTTACTGCCAAGATCC	TTCCCTTTGCTATATTCCCACGTAAC
3 MAGI1	GTAAAACACATTTAGTGAAGTGAAGCCA	CCTCCCCTCCTTGTTGCATTTT
3 MAGI1	CATTTTGCATATCATTGTAGGACTTGGTT	CATGCAAATGTGTCTCTCCATCAC
3 MAGI1	ACAGCATGGCGGTAAAGGTT	CCCTGTGTTGATGTGGTATGTCTATTTTA
3 MAGI1	ACTTGGGAGTTACGAGATTCCACT	CCCTTGTGCCTCCTGTTATTCC
3 MAGI1	GGATTGCTTGGAGGGTGGTTT	GCCCTTGGTTTATTGCCTTGAGTATTATT
3 MAGI1	TGGAGGAGTGCAAACTAAAGAAACA	TCATTCCTGCTATTCCTTTGGAGAAG
3 MAGI1	CCCTCCCTCCGCTTCTC	AGTATAGCAGACAACCCAATGAACAC
3 MAGI1	CGAAGTCCCATTCCAGGTGTG	TCCAGTTACCCACCCGATCT
3 MAGI1	ACAAGATGAATGAAAACGGAGAGACA	ATGTACATTAAGCTTTGCAAAATTGTGA
3 MAGI1	CCCCATGTGGTGATGATTTGTGA	TGCTCTCGTTTATTTTCCTGGCA
3 MAGI1	GATGTCAGGCTGTACTTAACACAAATG	TGCTCTTCTTGCCCTAAATGTTCTTT
3 MAGI1	GACTTCTCCATTTTCAGTATAGGCCAT	ATTACCACCTGTGAATAGTAGCATCATC
3 MAGI1	GAACAAAACAACCCTACTTACCTCCT	GTGCCGAGGCCTGAAAGAA
3 MAGI1	CTTCTGAGAAGGGTCCGTGATG	TCTCTCTCTCACCTCTTCACATGAT
3 MAGI1	GGTCATCTGGATCAAAAGGCAATG	CCCACACTCTTGCCTTCTACAG
3 MAGI1	GATTGGAGTCTCACCACACAGA	CTTTGGCTTCACGGTGGTTG
3 MAGI1	CCTGCACGTTCTTCTTATTAACTTCCA	GGCCAATGGGCTTTGGTTTTAC
3 MAGI1	GGCTACGACTCTTTAAAATTTGCACAT	CTCGGTGCCTAAACAAGCAG
3 MAGI1	CACACTCTTCCAGTGGCTTCT	AGTGTGTAATTCCGTCATTGGG
3 MAGI1	CACCAGGACTGTCTGCGATA	GTTCTCATGGTTATCCTAATGACACTGT
3 MAGI1	GATCTGGAGAAACTCATCAGGTTCA	AGTGGTGACATTCCTTATGCGT
3 MAGI1	TGAGGTCTGAGTCAGCTTTCACT	GGCTTCGTCATCGTGTCCTC
3 MAGI1	GGCTATGGAGGAAGCCAAAGAA	TCACCAGCTAGCCACAGTAGTAA
3 MAGI1	CGTTCAGCACTCACCAAAGG	TCAGCACCGTGGTGCAG
3 MAGI1	CCGGATCTCCACGTCGTA	GCCTCGCTGACAGAAGAGAAG
3 MAGI1	TCACCGTGTTCAGCGAGTTC	TGATGTACGCGTGCATCTCTC
3 MAGI1	CATGCCATTGACTTTGCCTGTT	CCTTGAACTCTGCCGAGGTTATC
3 MAGI1	ACCTACCCCAGACTCCTCTCT	CAAGGGATTTGGCTTTAGCCTTC

3 MAGI1	GGCTCTGGCTCCTCTCAAGTA	GACTATAACTTTCTGACTGTGAAGGAGT
3 MAGI1	CATGTTATACTCTCGGCCTCCTC	GCCTTTTTGCCAAGAATGTTTAGGAA
3 MAGI1	GTCACTGTCGCCAATTTGCTT	GAGAAAAGAGACTGGATTTGGATTTAGGA
3 MAGI1	ACTCTGCTCGAGGTCCAAGA	TGATGGTTTGTTGTGCCTTTGTATTTT
3 MAGI1	AGCACAGAAACACCTAGTATTGATTGAG	tctctGACTGTTCTTGGTGCTG
3 MAGI1	CCTGGTTCATTTCCACCCAGAA	GTCCTGCAACTGTGTTCATCTTG
3 MAGI1	GGAAGGAGGCCTTTTCTGTGAAT	CAGTAATTGGAAAATCACACCAGCTT
3 MAGI1	CCACAGACCACCCTCCTTTTC	GGAGCTGCTTCTGGAGGT
3 MAGI1	CGCTCCGACGTACGGAA	GGGTGTAACTCATCATGTCCAAA
3 MAGI1	CCAGTGGTTCTTCTTCTGGATCAC	TGCCAAGTTTCTCTCTCCTGTTTT
3 MAGI1	AGACAGAGGCCAGGATGCTA	GGTGCTCCCCGAGTTCC
3 MAGI1	CTTGTTGCATAAGCTGGACCAC	GCAATGTTTCATCCATCGTTCAATTACT
3 MAGI1	TCAGTTTGATCTGGAGCTTGGG	CCCCTCTCACTTGCTTACTTTCA
3 MAGI1	AGAGGCTGGTAAGACACTGGT	GCAGAGTTCGTCTGTTTCTGAAG
3 MAGI1	TTAACAAAGGAAACCAGTCATGTCTGA	CTTCATTTCTTTATGCTCTGTCCCTCTT
3 MAGI1	GGTTGGAATGTGACTCAGCGT	CGGTCCCTGGACAAAAGGA
3 MAGI1	GCTGGCTCTGTCCTCTCTGT	GCGGAGACGGTCTCTGG
3 MITF	GATGTGCTAAATGCATACATGGCA	TGATGCAGATTAACACGCGATTGTA
3 MITF	AAGCTTCTGTATGTTTGGGAAATGAGA	GCTCTTGCTTCAGACTCTGTGG
3 MITF	ACGTGCACAGCGTGTATTTTTC	AACTCTCCTCTACAGAATTTCTGGAAAC
3 MITF	TGTCTCTCTTCTCTTACCCTTTTTCCT	CCCTGGAAACACCTCAAGATTTTCTAC
3 MITF	GGTGCAATTTAGGATACCCCCAA	CGTAGCAAGATGCGTGATGTCA
3 MITF	CCTCCGATAAGCTCCTCCAGTA	TTTATGGCTGGTGTCTGACTCAC
3 MITF	CCAGTTCATGCAACAGAGAGTG	ACAAAACACCTAGCAAATGGAAAATGG
3 MITF	CACAGCCAGTGCCAGAACTAA	TGGTCCCGGGATTATTTCCAGAATA
3 MITF	ATCCTAGAGTAGGATATAGGTTTTATCTGAAAA	GGCAGACCTTGGTTTCCATAAAGAT
3 MITF	CCTGTCTCGGGAAACTTGATTG	AGAAATCTGCAGTAAGAGGCACATTT
3 MITF	TGTTTTCTTTTCCCTCCATGGCTAT	GTTTGGACATGGCAAGCTCAG
3 MITF	AGCAAATAAACATGCCAACCAAGTC	AGAGGAGAGGAGACATGAATTACCTC
3 MITF	CTTACGCTTAACTCCAACTGTGAAAAA	GGACAAACTAGAGTCCACAGTTAAGTG
3 MITF	CTGTTTTCCTCCATTTTCATCGCA	GTTTCTTCTGTCGGTTTTCAAGTTCTTT
3 MITF	CTATATCCGAAAGTTGCAACGAGAAC	TGTCTTGAAGTCGGTAAAAAGCAGTA
3 MITF	CCGGGCTCTGTTCTCACTTT	CCCAGCTGTCACCACTCAC
3 MITF	TTCTCTTTGCCAGTCCATCTTCAA	TGAGTCAGAATAAATCTCACCTGATAGTGA
3 MITF	GTGCCACCTAAAACATTGTTATGCT	AAACAGTATGTCTATTACAATAACAAAAAGTGG

3 MITF	CTGGCCCTCTCCAAGTGAAAT	CACACCTCTTCTATTTTTGCTTTTCCTT
3 MITF	CCAAGTTATAGACTGTTTTTGCTTGTGT	CAGGCTGCCAAGTCATTCAGTA
3 MITF	AAATGCAGGCTCGAGCTCA	CCGTGAGATCGAGAGTTGTTGT
3 MITF	GCATCATGCAGACCTAACCTGT	GAGAAAGGGTGTCGTCCATCAG
3 MITF	ATGGGATCCAAACTGGAAGACATC	AATGCAGTGCAGGGAGGATT
3 MITF	GAATTCAGACCTATTCCGCTCCAT	CCTGTAAACTGAGGTACTTTGGTTATCA
3 MLH1	TCCTGGATTAAATCAAGAAAATGGGAATTCA	CCATAGGTAGAAATACTGGCTAAATCCT
3 MLH1	TTCACTACTAGTAAACTGCAGTCCTTTG	ACAGACAATGTCATCACAGGAGGATA
3 MLH1	GGCTCTGACATCTAGTGTGTGT	CCATCCCCCATAAACCAAGAACTTA
3 MLH1	GGCTTCTTTGCTTACTTGGTGTCT	CACGAAGGAGTGGTTATGCAAC
3 MLH1	GTTTTGCAGTTCTCCGGGAGAT	CTTGTTCACACACTCAGCTGATTTAC
3 MLH1	CTCACCCCTCAGGACAGTTTT	GTTGAGGAGTTTGGTGCTACATTAC
3 MLH1	CACAAAGTTAGTTTATGGGAAGGAACCT	ACCATTCATTTTGAAGGCTAGGGTTTTA
3 MLH1	GAGAACTGATAGAAATTGGATGTGAGGA	TGGATTTCCCATGTGGTTCTTTTTAACT
3 MLH1	GGGAATTCAGGCTTCATTTGGATG	ACTCAACAATGTATTCAGCAAGTCCTT
3 MLH1	GACAGAGGAAGATGGTCCCAAAG	CAACAGAAGTATAAGAATGGCTGTCACA
3 MLH1	AGTTTGCTGGTGGAGATAAGGTTATG	AGCATTTCCAAAGATGGAGCGA
3 MLH1	AATGCCTCAACCGTGGACAATAT	TCTTGAAAGGTTCCAAAATAATGTGATGGA
3 MLH1	TGAGGCACTATTGTTTGTATTTGGAGTT	CCTCCCTCTTTAACAATCACTTGAATACT
3 MLH1	GCCAGTTTAGATGCAAAATCCACA	AACATCCTGCTACTTTGAGGTTTTACTT
3 MLH1	TCCCCTTGGGATTAGTATCTATCTCTCT	AAGCTTCAACAATTTACTCTCCCATGTA
3 MLH1	TCAAATCTTCTGGCCACCACA	TCGTGCAGGAAGTGAACTTCAT
3 MLH1	GATGTTAATGTGCACCCCACAAA	GCCCCAGAGAAGTAGCTGGAT
3 MLH1	CTAGCTTCTTTCTTAGTACTGCTCCAT	GTGGATTTAACCATCTCCCCAGAG
3 MLH1	ttAATACAGACTTTGCTACCAGGACTTG	CCGGGAATCTGTACGAACCAT
3 MLH1	GTGATAAGGTCTATGCCCACCAG	CCCTGCCACTAGAAATATCTGTCTT
3 MLH1	CAGGCCATTGTCACAGAGGAT	GTTGTATCCCCCTCCAAGCTC
3 MLH1	CTGAAGTGGCTGCCAAAAATCA	GAGGTAGGCTGTACTTTTCCCAAAA
3 MLH1	AACTGTGCTGTTGGTAAATATAATTGGA	TTTCTCCTCGTGGCTATGTTGTAAAA
3 MLH1	TCTGTTCAGGTGGAGGACCTT	CCACTCCCAGATTTTGGACTGTA
3 MLH1	GCCAGGACACCAGTGTATGT	GCAAGGCTTTATAGACAATGTGTTCCA
3 MLH1	CCAAACTCCTGGAAGTGGACTG	ACATCCCACAGTGCATAAATAACCATAT
3 MLH1	CAAAATGGAAGCAGCAGTTCAGA	TCCATCAGCTGTTTTCGTTGTAATAGT

3 MLH1	CATAAGCCATGTGGCTCATGTT	TGGTGTTGAGACAGGATTACTCTGA
3 MLH1	AGAAGTTGCTTGCTCCTCCAA	GGTACAAGCTGCAGTCATTTCCT
3 MLH1	AAATGGTGGAAGATGATTCCCGAA	CTTGGCAGTTGAGGCCCTAT
3 MLH1	ACTATTTCTGTCTCATCCATGTTTCAGG	TGAGAACACCAAAATTGGCAAAATCAT
3 MLH1	AGTGAAGAACTGTTCTACCAGATACTCA	GTGGAGAGCTACTATTTTCAGAAACGA
3 MLH1	TGACAGCATTATTTCTTGTTCCCTTGT	AAATGCTTAGTATCTGCTTGATCACTGA
3 MLH1	CATCTAGACGTTTCCTTGGCTCTT	GGCTCGACTCCCTCCGTA
3 MLH1	ACAGTTTTAACGCCTAAAGTATCACA	CTTTACTGAGGCTTTCAAAACATTCCTT
3 MLH1	CCAGGTGAATTGGGACGAAGAA	CCAGTGTGCATCACCACTGTA
3 MYD88	CTCTGTAGGCCGACTGCT	TCCATGGGAGACAGGATGCT
3 MYD88	GAAGCCCTCTAGAACAACCCA	CTCCTGCTGCTGCTTCAAGATA
3 MYD88	CACAGAGGAGGATTGCCAAAAGTA	CATGGGAACAGTATTGGACCCTT
3 MYD88	CCTTCCCATGGAGCTCTGAC	TTCCAGTTGCCGGATCATCTC
3 MYD88	GCGACATCCAGTTTGTGCAG	CTTCTAGCCAACCTCTTTTCGATGA
3 MYD88	CTGTGTCTGGTCTATTGCTAGTGAG	CCCTGGGAATAGCTTCAGGAA
3 MYD88	GGGTTGAAGACTGGGCTTGT	CAGACAGTGATGAACCTCAGGA
3 MYD88	AATGAAGAAAGAGTTCCCCAGCA	GTACATGGACAGGCAGACAGATAC
3 MYD88	GGATATGCTGAACTAAGTTGCCACA	CCAGAGCAGGGTTGAGCTTAC
3 MYD88	GCGGCGCTTCTCGGAAA	GGGAGGATGTGGAGGAGACC
3 MYD88	CGCTGAGGCTCCAGGAC	TGTCCGCACGTTCAAGAACA
3 MYD88	GCTGCTCTCAACATGCGAGT	TCCGCTTGTGTCTCCAGTTG
3 MYD88	GGAGATGGACTTTGAGTACTTGGAGAT	CCCAGCTTGGTAAGCAGCTC
3 PBRM1	CTCCCGCAAAATGTGCCTAC	GAGCATAACATCCGCAATGACAAAT
3 PBRM1	CTATCATTCCCTCTTCACCAGCAT	GTTCTTGAAGCTCGAGAGCCA
3 PBRM1	CATAAATAGGTCACAAAGTCTTCTGCCT	CCCTTTTCTACCTCCCCCTCAA
3 PBRM1	ACTGGTTGAAAGCGGAAAAAGAGA	TGATGGCCTTGTTAGCATGGG
3 PBRM1	CAGGGTGAAGTGGCTGCAT	GGGTTGGTTGTTATTACTTAGCTCA
3 PBRM1	GTCTGAAGCTGAGGTAGAGAAGG	ACCTTTGCTGGAAAAGAAGATCCA
3 PBRM1	CTCGGCAAATTTAGCTTCTAGCAAC	GAACACTGCTTTCCGGCTTATTC
3 PBRM1	GAGAACACCTAGAAGACAGTGCAT	CACCGGCACTCAGCTATACC
3 PBRM1	GCAAATGTTTTGTGGTATAGCTGAGT	CAGCTTGTATGTCAATGGATACTCTTGA
3 PBRM1	TGTCTTCATCCGAAGGGTGACT	CATTACAATGACATGTGGCTGAAGG
3 PBRM1	TTGATGAAGACACAGTCGCCAA	TTTCTTCAGCCAGTTTTCTTTACAACAG
3 PBRM1	GGACCCCAGTATCATTTAAGCCA	GAGCTCTTCATTAGGGCACCAA
3 PBRM1	GCTTCGAACTCACCTTCGCT	TCTACACTGGCTTTCTGTTCACTTTT

3 PBRM1	TCACTTTACTTTGGTTTTGAGTCTGCT	AGAAGAAGATAGTGAGGTCATTGAACCT
3 PBRM1	CTTCTAGGAAAGATCATAGGCCAATCT	GTTTAACTTATTGAGCATGCCTGGTTTT
3 PBRM1	AAATAATGAAGTGTGAGGCTGCCTTA	AATGATGCACATATCCTGGAGAAGTTAC
3 PBRM1	GCAGGCAGAAACCATGTAATCC	GGTTCCAAGAGAAGAAGAGCTACCT
3 PBRM1	CATCATCAAAGTCCCCGCTGA	GTAGCAGGTGTCCAGCTGAAG
3 PBRM1	GAGACTCTGCACATGTTCTGAGA	CTAACTGTGCCATTGCATTCTGAATTAT
3 PBRM1	CCTTCAAGATAGCCATTGGGCAA	GTAAAGGCAGCCTTGGTGAAGA
3 PBRM1	CGGTAATACTTGCTAGTGGGATTTCTC	GCACTCTAATTTCAGCTACCAAGTTTCTA
3 PBRM1	CCCCTTGCTTCGAAAGACAGTT	GTGATTAAGGCCCAACACCCA
3 PBRM1	GAGCTCCCCGAAAGAGTAGTC	TTGATGCACCATAAGACCTTTCTGT
3 PBRM1	TTGCTATCAGTTAATGGAAGTGCTCTT	GCTTTGTGAGAGCCGCTACA
3 PBRM1	TTCATCTGCTTGTCGCTCTCAT	TTCTCTTTCAGGAAAGTGTGCTGT
3 PBRM1	ATCCACTAAGAAGGCATTTCGCT	ACATCCTTCCCTGTCTAATTGCTTTTT
3 PBRM1	GACAGCTTTGAGGAAGCATGTATTTC	GTTTGTAGCTCCCCCACCAAA
3 PBRM1	AGGTAGGCCTCTGAGTGAAGAA	CATGTGGTCCTGTGCTCTTTCA
3 PBRM1	AAAAGCACAAATACCTACCGAGAATACA	CCATCTTCATTCACCCAGAAGAAAC
3 PBRM1	GCAGGTTTCTTCCAGATTACTCAGAA	TCCTCCCCAAGAATTGAAAAAGTATGG
3 PBRM1	CCATAAAAATATGCAGCTCCATCTCGAA	CAGCCAGTGAAACTCCTCTGA
3 PBRM1	CTGTTGGATTTTCATCAAGTCAATGGG	TGCTCTGTCACTTGGAATGTTGAA
3 PBRM1	CCCCCACCCCCAGTAACTAAA	AGATTTGATGCTCCGGGACAC
3 PBRM1	GTTGTATGCTTGGCGAATGTTGAG	TCCATTTGTCGAAAGAACAGGAGAG
3 PBRM1	CTTTTCAGCCAGTGAGAGGGTAG	TCAGACCTTAGAACTGAGGGAAATTTTG
3 PBRM1	CAGGCTCAGCCACAACAAAATAAAAT	AACCTTTTTACCATTTGCCTTCAAAGAA
3 PBRM1	GTGATATGGGCATTTTAATTTGCTGGTAA	TCAGCTTTATGACACAGTTAGGAGTTG
3 PBRM1	GCTGCCCTTGGTTATTCCGA	TTCTTTCTTCATCTCATTCGTCCCTTT
3 PBRM1	TCTTCCTGTTTGGCTAAGGTTTTG	CGAGCTGAAGACAATTTTAACTTGGAAA
3 PBRM1	CTGTGTGGCTATTATCTGCATACCT	GTTCGCGTGGCCTCTGTAT
3 PBRM1	CTCTGAGTTGTCTGTATTCTTCTCATCA	ATTAAACTGTGGACCATGCCCAT
3 PBRM1	CGAGGGACAAACCTGACTGAG	CCAGGAATACTTTAAGTTATGCCCAGAA
3 PBRM1	ACAAAAACATCCTCATCTCGGAAGTT	CAGTTCAGCTTTTGTTTGGTTGGTT
3 PBRM1	cccagccTAGACATTTTCTTAAACC	GCATTAAGCTGTCCTTTTATCTGTTCCTT
3 PBRM1	ACACTTGCCTAGAATTTTACTAACTGGAA	AAATGGTTGTATGGCTGTTGGTTTTAC
3 PBRM1	GGAACTGAGATGTCCCCAGCTA	CAACTCCTGAGATCTGCTCTGTTTT

3	PBRM1	GATCACACATCTTACTAGAACACACTCA	TGTTTGAAAATGCCAAACGCTATAATGT
3	PBRM1	CTGAATTGGGCACATTATAGCGTTT	CATTGGACTTTCAGGAAGAATGTTTCAT
3	PBRM1	CTTCCAAGAGCCCATCCATCA	GATTTGTACCTTCGAACAAGAAATGAGT
3	PBRM1	GTCATCTGCTTCTCCTTTCTGAACAA	GAAACAGGTGCTTTAAAAGTAGCAACT
3	PBRM1	ATTTTATTCCTAGGGAATGAGTGAGACTTTG	CCACCTGGCTACACGAAAATTTCTA
3	PBRM1	GCAAGGAGAAGTCAAACAGTCATATCT	GTAGCTTTAAAAACAGCATGTACCATGT
3	PBRM1	AGGTTCCACATAGACGTAATCTCCA	CCCTTTGGTGAGTACCTTTTTCCTT
3	PBRM1	AGCATGCTACCAACTACAAATCAT	CATATGTTTGAAGTATTGGAACGAGCAA
3	PBRM1	GCTTTGAAAACATACCGATTCATCCTT	ACCACCCCTTACATTTGACATAATTAGG
3	PBRM1	TCAGATAGTAGTCAGGCAACTCAGAT	AGGAAGAGTGGCATTTCTCCTA
3	PBRM1	GCTGCATTGGAGTCATGTATTTTGATTTT	CAAATTACTTGGAATCGTCCCTTGTG
3	PBRM1	GACGGTAGCGATTATTTTCAACATTCTT	TTTGTGTCAGTCATGAGTCATCAGG
3	PBRM1	GCTGTAGCATCTTCCCTCATCAT	TGCTCTTGTTCTACACAAAGTCCTG
3	PBRM1	CCAGGTCTCTGCGTGTTTCAA	GCCAACAAGTACCAAGATATTGACTCT
3	PBRM1	ACATCATGACAAAGTCCTCAACCAT	GCCATATTTCTGAGGCTTCCCTCTA
3	PBRM1	CCAGGCTCATTATAAGTTTTGGCATT	TGATGAACTCTCATTAAAAGGAACTGCT
3	PBRM1	ACTGACATGTACCTGTTTTTAACTGCAT	CTCCACATTACCAGTCTTCTCCAG
3	PBRM1	GCTCCAGGATCTCCTTCAAGTAAG	aaaaaGTTGCTGTTTTGAATTAGCTCTACAA
3	PIK3CA	AAAATTCCATCATTTAATTGTAAACGTGTTACT	GCCGCCTTTGCACTGAATTTG
3	PIK3CA	GTGGTGCGAAATTCTCACACTATTATG	ACTCACATTTCTCCTTTGTTCTTGTCT
3	PIK3CA	CACACACTACATCAGTGGCTCAA	TTCTAACTCAGAGGAATACACAAACACC
3	PIK3CA	TGATGGCGTGATCCCCAAATT	ACAGGTTGCCTTACTGGTTACCTA
3	PIK3CA	TGGAGAAGTTAGACATGTCAACCTTTTG	ATTTACCTGGGCTACTTCATCTCTAGAA
3	PIK3CA	CCAAATTGCTTCTGTCTGTTAAATGGAA	TGGACTTTCTGAGAGAAAACAATTTAAGTGA
3	PIK3CA	ACATTTGAGCAAAGACCTGAAGGTAT	AGAGCCAAGCATCATTGAGAAAAGATTTA
3	PIK3CA	CGACAGCATGCCAATCTCTTCA	TGAAATACTCCAAAGCCTCTTGCTC
3	PIK3CA	TCGAAAGACCCTAGCCTTAGATAAAACT	CCAGAGTGAGCTTTCATTTTCTCAGTTA
3	PIK3CA	TTCGTTGTCAGTGATTGTTTTCATTGTT	CAGCACATGAACGTGTAAACAGG
3	PIK3CA	TTCTTTGTAGATATGATGCAGCCATTGA	AGAGAAAACCATTACTTGTCCATCGT
3	PIK3CA	GCTCGCCCCCTTAATCTCTT	GGAATAAGATGGCATTGTAAAACAGTCC
3	PIK3CA	GAAAGCCTTTATTCTCAACTGCCAAT	ATTCACGTAGGTTGCACAAAGAATTTTT

3 PIK3CA	CCTTTGGGTTATAAATAGTGCACTCAGA	AGGTCAACAGATTACTGTATAGTGCAAG
3 PIK3CA	ACAGAGTAACAGACTAGCTAGAGACAATG	AGCACTTACCTGTGACTCCATAGAA
3 PIK3CA	CGAGATCCTCTCTCTGAAATCACTGA	GTAAAAACATGCTGAGATCAGCCAAAT
3 PIK3CA	GGCAGTCAAACCTTCTCTCTTATGTA	CTCTTCAATCACTGACATATCTGGGAAC
3 PIK3CA	ACTGGTTCAGCAGTGTGGTAAA	ACTCAGTGATTTGCCTTACCAGTC
3 PIK3CA	CCCGAGAAGCAGGATTTAGCTATTC	GGTCCTAGAGTTCATAGTATCTATGCCA
3 PIK3CA	CGGCCATGCAGAAACTGAC	ATGCCAAAAGAAAAAGTGCCCAAT
3 PIK3CA	CTGAGATGCACAATAAAACAGTTAGCC	GTTTCATCCTTCTTCTCCTGTTTGAGA
3 PIK3CA	GTGGGATGTATTTGAAGCACCTGAATA	TCTCAAACACAAACTAGAGTCACACAC
3 PIK3CA	TTAAGGAACACTGTCCATTGGCA	GGGTTCAGCAAATCTTCTAATCCATGA
3 PIK3CA	GCTTTGAATCTTTGGCCAGTACC	AGAGAGAAGGTTTGACTGCCATAAAAA
3 PIK3CA	CTGTACAGTACTGAGGTTCTCATGT	GGAGACAGAAAGCCCTGTAGAG
3 PIK3CA	GTGATCTTCCAAATCTACAGAGTTCCC	AGTCCTGTACTTCTGGATCTTTAACCAT
3 PIK3CA	GCATGCCAGTGTGTGAATTTGAT	AGACATACATTGCTCTACTATGAGGTGA
3 PIK3CA	CTGTGGATCTTAGGGACCTCAAT	ACAGGTAGAAGACTGCACTATAGTAATGA
3 PIK3CA	GAGGTGAAAGTTGTAAATCTTTGTAACAC	TTTGCAGAGGACATAATTCGACACT
3 PIK3CA	AAGGCTTGAAGAGTGTCGAATTATGT	GCTCATTAATACTCTTCCTTACCATCCC
3 PIK3CA	GACATCATGTCAGAGTTACTGTTTCAGA	TGGCTTTCAGTAGTTTTCATGGTTCA
3 PIK3CA	TCATGGTGAAAGACGATGGACAA	TCTTCTTGTGATCCAAAAAGTGTCCAA
3 PIK3CA	GATTTTGGACACTTTTTGGATCACAAGA	GCTCGAGCTCACCTCTCAAATT
3 PIK3CA	GCCATTTGTTTTGACACAGGATTTCTTA	TGATTGTTTCTAATAGAGCAGCCAGAAC
3 PIK3CA	CAAAGAATCAGAACAATGCCTCCA	GAGGCATTCTAAAGTCACTATCATTCCA
3 PIK3CA	GATAGTGACTTTAGAATGCCTCCGT	CCCTTTCTGCTTCTTGAGTAACACTTA
3 PIK3CA	CCTCCATCAACTTCTTCAAGATGAATCT	CGGTTGCCTACTGGTTCAATTACTT
3 PIK3CA	CCTTCGGCTTTTTCAACCCTTTTTAA	AAGGGACAACAGTTAAGCTTTATGGT
3 PIK3CA	TTTACATAGGTGGAATGAATGGCTGAA	AGCGGTATAATCAGGAGTTTTTAAAGGT
3 PIK3CA	GTCTATCGAGTGTGTGCATATGTGTA	CTTCCTTACCTGGGATTGGAACA
3 PIK3CA	TCCCTTGAAAAATGAAAGAGAGATGGT	ACACAGTCATGGTTGATTTTCAGAGT
3 PIK3CA	GGTGATCTGGGTAATAGTTTCTCCAAAT	AGAGGATAGCAACATACTTCGAGTTTTT
3 PIK3CA	CCAGAACAAGTAATTGCTGAAGCAATC	GGAAGTATTCATCACATCCACACACT
3 PIK3CA	GGGAAGAAAAGTGTTTTGAAATGTGT	CAAACAAGTTTATATTTCCCCATGCCA

3	PIK3CB	GATATTTCCCCCAAGTGACACAGTA	GCATCTGATGGCTCCATACCTG
3	PIK3CB	CCAGTGGGCAAAAGGAAATCCA	GGCTGATGGTTGTTTTGCTATGTTTTAAT
3	PIK3CB	AATACTGGAGCGGATGGCAAAT	ACAGCATGTTTGGGAAAATAACAACC
3	PIK3CB	TTCCCTTAACCAAGACAATTTGGAAA	GATGACAATTGCATTGCTAGCATAGAA
3	PIK3CB	AAATATTACCTAGTCCACATGCCAAAAGT	AGCGTGGGTAAATACGATGGTTTT
3	PIK3CB	TCCAGTTCTCAATTGTCCTTTAAAGTCA	AGGACATGTGCATGTTTACACCTTAA
3	PIK3CB	GATTCACTGCGCAAAGCACA	GTTTAATCAAACTGAATGCCGTGAAGTT
3	PIK3CB	CTCCTTCCCTTTGGCTCTGTTT	GGGAGCAGGTGTTATTGATTGATATGAT
3	PIK3CB	TCACAGACTGTTCTTGCATATGAGTT	ACAGATTATTGAAAAGGCAGCTGAGATT
3	PIK3CB	CTGACACATTAGCACTATCACTGCTT	AGCATAGCAATGCCAGATCTTATGT
3	PIK3CB	GAGCATACAGTATTTGTTTGGCTGTT	ACCTCTGAAACAATTGCTGACATTCA
3	PIK3CB	AGCAGCCACATTGCTACTGTT	TTTTTCCTTCCAGGATGTTGCCTTA
3	PIK3CB	CCAACCAAGTACCATACACCCAAA	CTGCCGAGAGATTTTCCCACA
3	PIK3CB	TGACAGCAGTAATTTTGGCAGTGAT	CTGTATTGAAAGAAATCTTGGACAGGGA
3	PIK3CB	ATTTTCACACAGTTGAGACAAGGGA	TGAAAGTTCGCTGTGGTGTTTG
3	PIK3CB	GAGTCTAATTTTTCCCCTGGGTCA	ATGTGTGAATCAGACTGCTGTATATGAG
3	PIK3CB	ACAGAGTCTTCGTGTTTCATCTTCAA	GTTATGGAAGCAAGTTCACAATTACCC
3	PIK3CB	GCTGATATTCATGCTTTAAACGTTGTCT	CACATTCCTGCTGTCTCAGTACAA
3	PIK3CB	AGTATGCTTCAAGGATGACACCAAA	ACACAATGACCTTGCCATCATAAGTTTA
3	PIK3CB	GCAAGCGACAGACACTTCTAAAA	GGGAAAGCTCATCGTAGCTGT
3	PIK3CB	tCCTACCTGGCAGTTTTCAAAATGA	TGGATGGACTGGCTAAAACAAACAT
3	PIK3CB	GATGGTTCATGCTCTGGTGGAT	GTGTTTAGGTCTGCATGAATTTGATTCC
3	PIK3CB	TTCAGGATCCTTCAAGGAATCAAATTCA	CCAAGCATCCAACATCCAAGTTAGT
3	PIK3CB	GCCACTTGAATAAAACAGGCAGCTA	GGCATGGGAATCTCTTCATCACT
3	PIK3CB	CAGTCAACATCAGCGCAAAGAG	TCTAACACTGTGCTCATGTGGTTT
3	PIK3CB	AACAAATACATTAGGAGCGAAGGCT	GAAGAAGCACTCAAACAGTTTAAGCAAA
3	PIK3CB	CCCTGAGCGCCTCATCAAATT	GCAGACTTTGATATTTGCACATCTCTAC
3	PIK3CB	CATACCTGGAACTGAATTAGTGGATGA	AATTGGCAATCCAAAAACGTTTGACTA
3	PIK3CB	GCTAACTTCATCTTCCTTCCCATGA	CCATCTCCCTCCCCCTTCAG
3	PIK3CB	CAGAAGGAGAAGTGAGCAAAGGAAATA	AGAGGCTGCCATAAATCGAAATTCA
3	PIK3CB	TGGTGGTAATGGAAGAGGAAGATTAGA	CCTTCCCCTTTCTTGCCCCTATA

3	PIK3CB	ACCTGGTGGGCTCAAAGTAAAAT	GGTGCAAGTGTTAAAATATGAGCCTTTT
3	PIK3CB	GGAATCTAGAGAGGGCACAATCAAG	GGCACAGGTTGTTTGGTTACTCA
3	PIK3CB	TTTTGCTATGGGAAGACATTAGACTGAAA	GACAGGATATGTTGACACTCCAAATG
3	PIK3CB	CAAACCAGCTTCTTTCCAGAGTAAATC	AGTTACAGGGCATAAAAGGAAAAGCA
3	PIK3CB	ATTATTCCGTTATATAAAACAATCCTCAGAAGT	TGTTTTGCTGTTTATGCAGTTTTGGAT
3	PIK3CB	TTTCCTGATGGTCTGATATTTAGAGGGA	TGATCATATTTGGAATGAACCACTGGAA
3	PIK3CB	CGAGCCATTCTTGGTAAGTCACAAATAT	TGTTTTTGTTTTTGTCTGCTTCGTTACA
3	PIK3CB	GCAAAATGGCAACAAATGAAAAATTCT	TTTGTGGCTGGTATACAATAACAAGGT
3	PIK3CB	CACTCCAACTGAATCCTCACCAAAT	ACTGAAGTTGACTAATTTCCCCACTT
3	PIK3CB	AGAATCACAAAAGAATCTGGCTGAGT	CTTCTTATGTCCTTGGGATTGGTGA
3	PIK3CB	CTGAGCCCTTTTCTTTCTTATGAGACA	TCCAGACCAGTACGTTCGAGA
3	PIK3CB	TGACCATGATGTTGTCACTATGTCTG	CGCTTGGAGGCTGAAAACCTT
3	PIK3CB	GCAGGCAGCCTACAGCATAT	CCCCCATTATTTCTGAACCAGTTTCATT
3	PIK3CB	TTCAATCATTTCATGCATAGAGGTCCTT	AATCCAATGGGAACTGTTCAAACAAATC
3	PIK3CB	TGCAAAGCTGTTGCATTTTCAGTATATG	TCTAAGGTGATTTTCATGGGAGGAGAT
3	PIK3CB	TCAGCTAAGAAATGCTGGTCCAC	GAGCGAGTGCCTTTTATTCTTACCT
3	PIK3CB	CCTTGTTGAATGACATGGATGAAATCAT	GAACCCACCAGAAAAATGAAGGAAATTA
3	PPARG	AGTCATCCACGTTTTCCCTGTTTT	CCTCCACGGAGCGAAACTG
3	PPARG	TGGCCATCCGCATCTTTCAG	CAAGGAGGCCAGCATTGTGTA
3	PPARG	CTCAAATATGGAGTCCACGAGATCATT	CAAACTTGGGCTCCATAAAGTCAC
3	PPARG	GAGCCTGCGAAAGCCTTTTG	TCATCCCACCCTCTTTCATAGAAGAT
3	PPARG	TTCATCCTGTCATTCCTCTTCCTCTAT	CGGGAAGGACTTTATGTATGAGTCA
3	PPARG	GGCCCTGGCAAAACATTTGTA	CGCCTCCCCCAATGAAGAC
3	PPARG	CAAATACAGCATGAAGGTGTACTATGGT	GCTTATTGTAGAGCTGAGTCTTCTCA
3	PPARG	AGCCTGCATCTCCACCTTATTATTC	ACCTTGCATCCTTCACAAGCA
3	PPARG	CAGTGTGAATTACAGCAAACCCCTA	AATAGCCGTATCTGGAAGGAACTTTAC
3	PPARG	GTTGCTTGGTAGAGCTGCCTA	GTAGCAGGTTGTCTTGAATGTCTTCA
3	PPARG	GTTTGCTGAATGTGAAGCCCAT	GTGTTCCGTGACAATCTGTCTGA
3	PPARG	AAGCTGCTCCAGAAAATGACAGA	GGAAGAAGGGAAATGTTGGCAGT
3	PPARG	gctgcttcCATGTGTCATAAAGACTTA	TCTGAAACCGACAGTACTGACATTTATTT
3	PPARG	TGATCTTAACTGTCGGATCCACAAAAA	TCCACTGGTCTGGCAGCTATA
3	PPARG	CTCTTTCTGAAACTCTGTGAGATTGCT	GGTCTTCCATTACGGAGAGATCC

3 PPARG	CAACTTTGGGATCAGCTCCGT	AATGGAATGTCTTCGTAATGTGGAGTAG
3 PPARG	CACTACTGTTGACTTCTCCAGCATTT	GAGTCCAATTCTAGTCCTAGTAGTCTGA
3 RAF1	CTGAAATAAGTATCAACCTCACCCCATT	AGAGGTCGACATCCACACCT
3 RAF1	GGTGCTGACCATGTGGACATT	CCAATCATGGAATTTTCTTTCTCCTCCTA
3 RAF1	CCAGAGAGGCATCAGACCATCTA	CCTGGCAATTGTGACCCAGT
3 RAF1	CATGCAGGTGTTTGTAGAGGCT	AGTAGACCTCCCTGAGCACTT
3 RAF1	CTGAAGGTGAGGCTTAATAGACAAGA	TGACCAGCTTTCCTTTTCTGTTTCA
3 RAF1	CCGGTTGATCTTCGGTAGAGAGT	GACCCAGGTCCTCTACAAGATG
3 RAF1	ACAGTCCTTGTACTCCCCACT	TTGCACTGACTGCCAACTAATTTTG
3 RAF1	AGTCCTAACCCTCTAGCTGCTT	TGTCTACTCCTATGGCATCGTATTGTAT
3 RAF1	AATGAGTCTAGAATCCAGACCTGTCA	GCACTGTAGCACCAAAGTACCT
3 RAF1	GCTCCCCCGTCATCAGTTC	GGTGATAAATGCACTTTTGTCATATGGT
3 RAF1	TGTTACTCCAGTCCACACACATAGT	GCATAATTTACACCTGTGTTCTTGTTGT
3 RAF1	GCTGAAACTTGACTTCACACCAA	CAGTGAAGTGATGCTGTCCAC
3 RAF1	AGAGCCTGACCCAATCCGA	GGTTGATCCTTTGATGCCCTCATTAT
3 RAF1	ACCTGGGTCCCCTCCCTATA	GAAGAGGCTGGTAGCTGACTG
3 RAF1	GCCTCTCTTCCTTTACTTTCTTCACA	AGCTCAGCTGTTTTCTTTCCCTTAG
3 RAF1	GAAGCTAGAAGATCCTTACTAGTCTGAAGA	AGCTTGCATGACTGCCTTATGAA
3 RAF1	TGGAGAAGTCTGAACACTGCAC	TTATTTCTGTGCCCACCTTTCCA
3 RAF1	GCCTGTCTTTAAGTTGAACATGATCCT	GACAAGCAACACTATCCGTGTTTTC
3 RAF1	CTGTTCTTTGCTTGTTCGGCAA	CCAGCTGCATCTCTCCTACAAT
3 RAF1	GCTGATAGCCAAACTGCTGAACT	CCAGGTTTAAGAATTGTTTAAGCTGCAT
3 RAF1	AGAAATCTCTGTTATGCCTGGCAA	AAAGCACGCTTAGATTGGAATACTGA
3 RAF1	TGTGATAGAAAGCCTCCCTTCTGT	TCTACTCATGCCCCTCAAACTTATTTTT
3 RAF1	TGGCAGGAGGTACTGTTGTCTA	GTCTTGTCTATTAAGCCTCACCTTCAG
3 RAF1	CTGTTGGGCTCAGATTGTTGG	CGAAGTCACAGCGAATCAGGTA
3 RAF1	GTACAGAAACGCTTTAAGTTTGCACA	GAGTGGTTCTCAGCAGGTTGAA
3 RAF1	GAGGACAGAGCCAGTAGGTTG	ACCAGAGTCCTTAACAAGCATTGAG
3 RAF1	GGAGGGAATGCGAAGAAACTCTT	CGTCGTATGCGAGAGTCTGT
3 RAF1	AAAAATTACCTAACAGGCATCCTGGAA	GTCTCTGTAAGATTACTGCAAGCCTAAA
3 RAF1	CCTGAAGACAGGTGCAAAGTCA	TCTTCCATTGAGCTGCTCCAAC
3 SETD2	CTTGGATTTCTCTGGTGGCAGTA	AACAGAGTCAGCATCAGAGCAG
3 SETD2	CGTTCCTTCTTCATTTTCCCTCCTG	AGCTGACTTCTTTCCTATGCTCTTTG
3 SETD2	AGGAAATACATGTGTAAGCCACACT	AGCATGGCTGTTTTTGTCTCTTTCTA
3 SETD2	CCACCGTGGGCCTGTTA	AGCCGCCTCCGAAGATG

3 SETD2	AGTCCCGATCAGATTTAGAATAGGATGA	GCGCTATTCTCGGCCATACAC
3 SETD2	AGAACTCTCTCGTGCTCTGTTATCT	CCTATTCCAGGTCAGAACGATCTCATTA
3 SETD2	TGGTAGCGACGATCAGAGTCATAA	ACTCGGTATGTATCTTCCCGATGTAG
3 SETD2	GCCGTCGCTCTCTTTCTGAT	AGAGATTTTAAAAAGAGCTCAGCACCTT
3 SETD2	GAAGGTTTCCCTAGATCCTCACTTTTTA	TGAAGGCATCTTTCTTGGTTCAGAA
3 SETD2	AGAAGTCCGTACAGAATCTTCATCAGA	GGGAAGGATGAAGAAATTCCAGATAGTT
3 SETD2	CCCAATTCTCCTCTTCTTCACGA	TGATTACTGGCAAGGCAATGGT
3 SETD2	AGGCAGGCGATTATCTATTTGTTTTCTA	GAGTCAAGGAGACATTTGTATGAGGAAA
3 SETD2	GGACAGGCTTTACTTGCTATACTTTCAAA	CTTTGCCCATGGAAGAAACAAGTC
3 SETD2	GCCTTCAGATTGTGAGGATTTCTTCTTA	GAATGAGCAAGCAGATATTTCCTCAAAA
3 SETD2	TGGAATTTCTTCATCCTTCCCAATATGG	AGAAGCAGATACTAAGCAGGACACTA
3 SETD2	TGAGGAAATATCTGCTTGCTCATTCAAT	ACCAGTACCCTTACCAGTAGATGTAG
3 SETD2	ATTGGTGGTTCTTTCAGAGATCTAACTG	CTCTCATCACCAGCACCAGTAA
3 SETD2	GTCTACCTGATCTTGGATCCCAGTA	CTCAGAAACTTAGGTTGGGACTTCTC
3 SETD2	TGACTGCTCCGAGAAGAACAAG	GTGATTCAGAAGATACAGATTCGGATGA
3 SETD2	GAGACGGTTTCTTGGAATACTGCTA	CATGGAAGACAGTGATGGTGTAACT
3 SETD2	CTGTTATTGGTGTATGTGGCAAGG	CACCTACTCATGCAGCACCAT
3 SETD2	GACCACTACTGTCACACTTTAATGCATA	CCATATTGCCTGAAAGAAGAGGAAGAC
3 SETD2	GATACGTGGTAGAAGGCTTTTCTTGA	AATAAGTCTTGGCAACAGACCACTT
3 SETD2	GTTGATTCTGCTATCACTGCTGGTA	GCAGAAGAATCTTCCCCACCAAA
3 SETD2	TGTCCTCCTTCTCCTCTTTCATCTAAA	TCAGGAAAGGGATTTGCTTCCAG
3 SETD2	AGTCTACTATCTGGCCTGTTTTGGA	TGGGTCACCCAAATTCAGAGG
3 SETD2	ACCCATTATTACGCCTGTTCTCC	TCTGGATGCAGTGCTAAAGAGTAAAAA
3 SETD2	GAAGGTATTTTGGCTTTCACGGTTT	CCGGAAATTTCTTTTACACAGTCCAG
3 SETD2	CCTGCATGCTTTAAAAACTCTGAACTT	TCTTCAGAGTCTTCCACCAGGAATA
3 SETD2	TCAAGAGAGTTAGACTGTCCACCTT	CAGATCACTCAAAATTTGCATGTGAAGA
3 SETD2	GTGCTACCGATGCTCTGCTTATAT	ACTCTGAAGCTGAAAATATTGAGCCTT
3 SETD2	GTTCAGATTTAAGCCTGAGGAGCTT	CCACTGTGTTTTCTCCTCCATTTTTAG
3 SETD2	AGTGTTGAGCAAAAGCCGAGTA	TCTCATCGAGATATTAAGCGAATGCAG
3 SETD2	AGAAAACCTTCCAAAGACAAAGTAGACA	GGCAACAGATTTTCACAGCTCA
3 SETD2	TGTACCACCACTCCTTGTGGA	GCCAACTTGGAAGTCAGTCTGTTTATTA
3 SETD2	GTAAAACTCCCCTTATAGTGGCAACTT	CATTGTCTTACCTCCCAACTGGA

3 SETD2	CCTTCTGGATCTCGAGCTGTCT	AGAGATCACTGATCAAAAATGTGTGTGT
3 SETD2	TGGTGGGCTACCAAAAGCAA	AACGTGTTCTCTTTTCTCTGCCT
3 SETD2	GAAACAAGCAAGCTAAATGTGAACTGA	GTCTCCCTTCCCCCATGAAAG
3 SETD2	aaTCAAATCAGTATCAATGGCTCCTTCA	GAGTATGCACGAAACAAAAACATCCAT
3 SETD2	CATCATTCTTCAGGGCCATGAAATAGTA	TGCTCCTAGGTAGGAAGTGCTATTTT
3 SETD2	TGACTTGGGTACTTTTTAACTTGCTCaa	CCAGACAAGCAAACTCAAAATAAAGAGA
3 SETD2	GAGGGAGCTTCTTCGTTTCCTTT	GCAACTGAACGAGGAAGGGATG
3 SETD2	GTGTTTGATCTCTGAAGCCAACAG	CGGAGGAGTCCATAGATACATTTGTT
3 SETD2	CACATAGGCCTGCATGGGATAA	GGAAGTTGTTTGAGCAAGAGGTG
3 SETD2	CATGAACAGACCATAAAGCAGAAAAGAA	CTGTCAGTGTACAGCAGCAGTA
3 SETD2	GTTTCTGAGCCTCCCGTTGA	TTTGATTGTGTCTATGATTTAGGGAGCA
3 SETD2	GCTTGAGACTGTGCAGGAGAG	TGGCCCAGAGTGATGGTGTA
3 SETD2	CAACGCTGGAGTCTTGGTGTA	TCTCCTGCTCCTTATGATCATGCT
3 SETD2	GGTTCTGTAGAATGTCCCACCA	GCATCATCCCTTTGCTGGTTAC
3 SETD2	TTACTTCATTTCACATTCAAAATGATAGCTACA	CTTGGCTGAATGGAAATAGAGAAGAAGA
3 SETD2	CTTACGAAGGAAGGTCTTTGGCA	TTTCAGTTCTTCTCGGTGTCCAAAT
3 SETD2	CTGCATGCTGTTTTCTCTGAAACC	AAAGTTACTTTGGTTCTTTTGCTGCTT
3 SETD2	TCCCACAACTCTTTCATGTGTTTAATCT	CAAGCAAGGGAAAATGCCATGT
3 SETD2	AGATCAAAGTAACATGGCATTTTCCCT	GTGAGCTTCAGGACAGAAAGAAAGT
3 SETD2	TGGTTTTGATTCCAAATGCACATTCAT	GCTGCAAAACCAAAGATTCAGACA
3 SETD2	CTGGAGACCCAGCCTTTTCT	CCTTTCAAAACCTATACCCAAGTCTGAT
3 SETD2	GGGTTGCTATCGTTCAAAGTACAGTATA	TCACCACACCAAGATAAACTCATGTC
3 SETD2	CTACCTTTTGAACAAGGTGTCTGTAAAC	GACGAGGGTCATCATATTCTAAGCAT
3 SETD2	CCCTGCTCTACCTCCACTCTA	GGACACTTTAGCTGTGAATGAAAAGAAA
3 SETD2	GTAGATTTATAACGGGAAGCACTACTGT	ACTTAGAGATGGAAAGAAGAGGCAAGTA
3 SETD2	GGAATAATCCACAGTCATAACTGGCATA	GCATGCTTTCATCAAATGGATTTCAGAA
3 SETD2	GGGAGAACAACATCTTTTAATTGCTTCA	TTCCTCTTCTTACAGAGACCTAAGGAC
3 SETD2	CACTCTCTATTTCCTGCCTCCTTTTT	GTGCAAGCACCTGAAATAAGCA
3 SETD2	CACAGCTAAAGTGTCCTTAATGGAATTG	ACAAGTACCAGATTCCCTAACAGATGA
3 SETD2	TCCAAGTCTTTTTCTTTGCACCTACTAA	GGGCAGAATCTGATTTGGCA
3 SETD2	TCAGATTCTGCCCCAGGAGAT	TCCGAATTTATAACACATGATAGCCATGA
3 SETD2	CTGCCCCAGGAGATCCATTTATATTTAA	GCTAGATGAATTGCCTATTTTTAAGTCCGA
3 SETD2	TGCCCAATTCCACCCTTGAC	GCAGAATAGGTTTCTCACTGCACTT

3 SETD2	GGAGTATCACTTTGCTTTTCATTGCC	GACCAATGTTCAAAGGTGTTGCT
3 SETD2	GCCTTTGGGCAAAAATCGACTAGA	ACTGTGCAGTCCATTTGAAAGTAACTAT
3 SETD2	CCCACAGGATTTCCTCTCCCTA	GAGTGCAATGAGAATGTGAAACACAAA
3 SETD2	GCATGTACTTCTTAATGTACTCCTTGGT	CCCTCATATAATTTCTGCTCTGATTGGT
3 SETD2	TGTAAAGCCATCAACACAGAGTATCTC	ACCATTTCCACCCTCACTAATAACC
3 SETD2	ATGGTCAGAACAGCAATCGTGA	CCAGATAATAGATGCCACTCAAAAAGGA
3 SETD2	CTGTGATTCATGAAACGAGAGCAATT	GTTAGGTTTCGCAGCACAATCTTTTT
3 SETD2	CAAGTTGAAATGAACAAAAGATGCTTCCT	TGAAGAGGAGGGTGTGTCTGAT
3 SETD2	TGTTCTTGGCTCCTTTCACTCTC	ACCAACATAGAAGCTAGTAAGCTACCT
3 SETD2	GAGCACGCGATGTATTCTCACTA	AGACTTTGGAACACTTGCCCAT
3 SETD2	TCTTCATTCATTCATTCCCACCACAA	CTTATCCCGGCTAATGGTTAGAATTGAA
3 SETD2	GACAGGTAAGTTTCTGCTCCAAAGT	GGTATTTTTATTTGCTGTGGTAGGTGGA
3 SETD2	CGTCAGCTTCTGGTTCAGATGT	GGATGGCAAAGAGGATCTTGATCAAT
3 SETD2	CCTCTTCCTCTACAGGGACATTTTCTA	CAAGAAACTAATGTTTCGCAGACTGAAA
3 SETD2	TGCACTGTCCATGCTATTTTCACTTATA	CGTTGAGTGAAGGAGATGGGTATTC
3 SETD2	CTGAACAGCCTATTTCCCCATCA	GGACAACTGAGGGTTGGGTTTTT
3 SETD2	CTGAAGGAACCAGTTTGGTGGTA	TGATGATCTTTATATATGCGcacacaca
3 SOX2	AGCGCCCGCATGTACAA	GTCCGGGCTGTTTTTCTGG
3 SOX2	GACAGTTACGCGCACATGAA	GGGCGCTCACGTCGTAG
3 SOX2	CATGATGGAGACGGAGCTGAAG	GGACCACACCATGAAGGCATT
3 SOX2	CGTCAAGCGGCCCATGA	GCTTCTCCGTCTCCGACAA
3 SOX2	GCACCCGGGCCTCAATG	CGAGTAGGACATGCTGTAGGTG
3 SOX2	GGCGCCGAGTGGAAACTT	GGGCAGCGTGTACTTATCCTTC
3 SOX2	CCAGCTCGCAGACCTACAT	AGTGGGAGGAAGAGGTAACCA
3 SOX2	GGAAAACCAAGACGCTCATGAA	GTAGCTGCCGTTGCTCCA
3 SOX2	GTTCGGTGGTCAAGTCCGA	GTAGTGCTGGGACATGTGAAGT
3 SOX2	CCGGGACATGATCAGCATGTAT	CCGGCCCTCACATGTGT
3 SOX2	GGCCATTAACGGCACACTG	TCTTTTTGAGCGTACCGGGTTT
3 TGFBR2	GGGCCACCATCAGCTATATTG	CCAAATTCATCCTGGATTCTAGGACTT
3 TGFBR2	ACTGCAAGATACATGGCTCCAG	CAATGATGCTGGTCCACACCTA
3 TGFBR2	GCCTTTCTTCAGATTCATTCTCATGAC	GCACCGTTGTTGTCAGTGACTA
3 TGFBR2	TCTCTCTCCTCAGTTAATAACGACATGA	CTCACAGATGGAGGTGATGCTG
3 TGFBR2	CAGAAATCCTGCATGAGCAACTG	AGAGATACACTGACTGTGTGTACTATGA
3 TGFBR2	CAGGCATCTCACCATGCTCAT	TCGATCTCTCAACACGTTGTCC
3 TGFBR2	CCCCTGTGTCGAAAGCATGAA	ACTTGGTTGAATCTTACTGACCTTTGT
3 TGFBR2	CTCCCCTCGCTTCCAATGAA	GGAGAAGCAGCATCTTCCAGAATA

3	TGFBR2	CAAGCTCCCCTACCATGACTT	CCACACCCTTAAGAGAAGAAAACTCA
3	TGFBR2	CATCTTCTCAGACATCAATCTGAAGCA	GGTCCTCCCAGCTGATGAC
3	TGFBR2	GAGTACCTGACGCGGCAT	GTTAGGTCGTTCTTCACGAGGATA
3	TGFBR2	TCCTGACAGTACTTACCTACCACATC	GGTAGCAGTAGAAGATGATGATGACA
3	TGFBR2	AGGGACCTCAAGAGCTCCAA	GGGATCTAGCACTAGCTCTAACTTAC
3	TGFBR2	ACCACTGGGAGTTGCCATATC	GCTGATGTCAGAGCGGTCAT
3	TGFBR2	CACTGTGCCATCATCCTGGAA	TCTCAAACTGCTCTGAAGTGTTCTG
3	TGFBR2	TGAGGTCTATAAGGCCAAGCTGAA	GTCAGGAACTGGAGTATGTTCTCA
3	TGFBR2	CTTTGGATCTCTTTCCCGCTACA	AGGAATCTTCTCCTCCGAGCA
3	TGFBR2	TTCAGTGAGCTGGAGCATCTG	CTGCCTCTGTTCTTTGGTGAGA
3	TGFBR2	GGGCTCGGTCTATGACGA	CCACTCACCCGACTTCTGAAC
3	TGFBR2	GCGTATCGCCAGCACGAT	CGGGCCCGACTGTCAAG
3	TGFBR2	AGGACTGCCCATCCACTGA	CCATCGCCTCTTTAACAATTTAGCTATG
3	TNK2	GCCTTGGCCTTGCTCCAT	GCTGGTGGTTTCCCATTCTCTA
3	TNK2	CGAGTGGTCCTGAGGACAGA	GCGGCTCCGAGATGACC
3	TNK2	CAGGCGGGTGACGTTGA	CCACAACCCTTGTCCCTGAG
3	TNK2	GGTCCCATGCCTCTGCT	AGCCTCACCTGCCTCATTG
3	TNK2	GAGGCGCAGGTCCTTCT	CCCGGCTGCCTTCAAACA
3	TNK2	CTGAGGCAAAGCTCTGGGT	GCCCCTGTCCCCTCAAG
3	TNK2	GGAGCTGCCAGGTGGTA	CCACACGTCCAGCTGTCTC
3	TNK2	GGTGTCCTCGAGCCTTGA	CCTCCTCGGGTACCCATCC
3	TNK2	GGAGACAGCTGGACGTGT	GAGATCTTCCAGGCGCTACAG
3	TNK2	CAGTTGCCTCATGCACTCCT	CAGACCAACTACGCCTTTGTG
3	TNK2	GCGGGAGGAACAGGTTGTC	AGATCTGCTCCATCAACAGCAC
3	TNK2	TCAGGCACAAAGGCGTAGTT	CGCCCGCCTATGACGAC
3	TNK2	GGGTCTGCAGGGACTCTGT	CAATCTGCTGTTGGCTACCC
3	TNK2	CCCAAAGTCCCCGATCTTGA	CCGGCTACGTAAGCACCAG
3	TNK2	GCGTAGCGGCTCAGAGT	CAGGGTCCTCCAGCTCATT
3	TNK2	GGTAGCCCCTCAGCTTGAAC	GACTGTCCCCAGGACATCTAC
3	TNK2	AGCACTGGACCATGACGTT	GGGCGCTCTGTTTGTCGT
3	TNK2	GGGCCCAGGTATCTGGCTAT	GCTGCTTCTCTCCCTGACC
3	TNK2	CAGGCGCTGGTAAGCAGAT	GGCCCTGCACTTCTGGAG
3	TNK2	GCCTCTGACCTTAGGATGGAGA	CTCGCTCGACCACCGAAA
3	TNK2	CCCCGTAGAGGCGGATGA	AGTCCTGAGTCCTTGCAAATCC
3	TNK2	CGAGGTGTTGGGCTTCCT	TGCCACAAAGTGCTGGAGA
3	TNK2	CTCCAGGTTCCAGTCGAACA	CCCTCTTCCCCACCCTAGTC
3	TNK2	GGTCCCTGAGAGCCAGAGT	GCCCTAGTGGTGCTGACA
3	TNK2	CTTCCTGAGTAGCCGATGAGTT	GCTCTCTTGACCCAGCTCTCT
3	TNK2	CCGACCTGTGCCCTTCAA	ACGACCTTCCTCTTCCTCCAG
3	TNK2	ACGGCGAGATTCGACCTG	CAAACGCAAGTCGTGGATGA
3	TNK2	ccacctTACTCATCCACGACT	CAGGCAGCAGCCTCTCT
3	TNK2	GGCTGGGTGCTCACCAT	CCCAGGCTGCCTTGGAC
3	TNK2	TTGGTGGAGAAGTTGGCCTT	CGCTACCAGCGCTTCCT
3	TNK2	CGCAGGAAGCGCTGGTA	CTTTGCCTCAGACCCCAAGT
3	TNK2	AAGTAATAGTGGGTGCTGCTGAC	TCTCAAGCTCACCTGGGAAGA

3	TNK2	CAGATCTCAAAGTCATCCTCATCCT	GCCATGGACGCCTGCTC
3	TNK2	GGGCTCCTCACCGAAGT	CTGTGAGCGAGGACCAAGA
3	TNK2	CCTCTTGAAGTCGCTGGACAA	GACACTTGGCTTTCTCTCCCA
3	TNK2	GGCCAGGTCAGCATCCA	CTGTCGGCCCAGGACATC
3	TNK2	CTGTGTGGATGAAGCTGTTCTG	GTTCACCGTGCCATCTCTGT
3	TNK2	TGCAAGCAGGGAATGGAATTCA	CCAGCGACACCTGGATGTTC
3	TNK2	TGAACATTTCCCACAGTGTCACC	GGTGTTTGAGAGGGCTTCTCTT
3	TNK2	GCCCAGGCTCTGTACCTTC	TGTCCGCATGGCCTTTCTT
3	VHL	GGTTAACCAAACTGAATTATTTGTGCCA	CCTGTACTTACCACAACAACCTTATCTT
3	VHL	cACCGGTGTGGCTCTTTAAC	GCTGTCCGTCAACATTGAGAGA
3	VHL	GGTAGTTGTTGGCAAAGCCTCT	GTAGAGCGACCTGACGATGT
3	VHL	GCCTGAGAATTACAGGAGACTGG	CAAAAGCTGAGATGAAACAGTGTAAGTT
3	VHL	GAGCGCGTTCCATCCTCTA	CCTACCTCGGCCTCGTC
3	VHL	GAAGAGTACGGCCCTGAAGAA	GATTGCAGAAGATGACCTGGGA
3	VHL	GCTGCGCTCGGTGAACT	GTACCTCGGTAGCTGTGGATG
3	VHL	CCCGTATGGCTCAACTTCGAC	GGGCTTCAGACCGTGCTAT
3	XPC	CCAAGAAACTGACCCTGAGCTT	ACTTTTCTCCCACTTCATTTTTGCC
3	XPC	GATGAGAAAAATCAAGACCCGAGACA	TCAGCAGATGGTCCAGCAAAA
3	XPC	AGTCACCTTGGCCACTTTCTT	CACCTTATGTTCTGTGTTGTCACCTA
3	XPC	AACAAAGAAAGATGTTTCACCTTCAACC	ATCTTCCTTCCTGTGGTCTTTACAC
3	XPC	CCTCCGCGTCTGGACTC	GAACTGCGCAGCCAGAAATC
3	XPC	CTCTCACCCTCCTCCTCCTC	TGCGTCACTCGCGAAGT
3	XPC	GGTGAGCAAGTCAGCATTTGG	AGAAAATGACCTGGGCCTGTTT
3	XPC	TGATACTCCTCTGTCTGCCAGTAG	CTGAGGAACTGGATGCCTTTGT
3	XPC	GCAACCCTTGCCTAGGAAGAA	CTGAAGCGGCATCTCCTGAAATA
3	XPC	TCTCGGGATAGATGGCCTCA	AATAACATGCTCAACCTTGTCTGTCT
3	XPC	CCAGCCTCTGAGAGAAACACAAAT	TCTGCATGCTATTGGCCTGTC
3	XPC	GTAAAGCGGGCTGGGATGAT	CAGCAAAAATTCCTCCTGGTGT
3	XPC	CTTTCCATCCCCATCTCTGGA	GCTGAACCTGCCCAATCTACAC
3	XPC	GACACAGTCGATGTCCAGCTT	GCCAAATGCTGACTTGCTCAC
3	XPC	GCTGTCCCTCAGTCCTGCATA	GTTGACATCGCGTTGTGTCC
3	XPC	ATCTGTGGTCTAATTAGCTCAAAAGAAA	AGGAAGAAAGTGAAAATGATTGGGAAGA
3	XPC	AGCAGCAACTGGTGGGT	GCCTCAAAACCGAGAAGATGAAGAA
3	XPC	CCACCCTTCAGCTTCTGCTT	CCACCACTGCCACCTGTC
3	XPC	GACTGGGAGGCTCATCATCAC	GTGAGGCTTGGAGAAGTACCC
3	XPC	CCCCAGCTCCAGTTACCTTGTA	ACCTTTGTGTCCTGTGTTGGTT
3	XPC	GCATGTGACAGGAGCCTAGAA	CTGGGTGACGTGAGAGAAAGT
3	XPC	AGAAGAGATCGAGAGAAGGCTGT	ACTTCCTCCTTCCCAGCAGAA
3	XPC	CAAGCTCTTTGCACCGACAA	CCCCTTCATTCCCAGATTTTTCACTAT
3	XPC	GGGCTTTCCTCTGCTTTGGA	CTTATAAAGAGGAGAGTGGGAGTGATGA

3 XPC	CCACTGGAGAGCTCAAAATCAGAG	CCTCCAGCGAGGAAGATGAG
3 XPC	GTTGCCTTCTCCTGCTTGTCT	AAACCTTTGCTAAGGGCACCT
3 XPC	CTCCCTTGTTCCTCTTCCCTTTG	CCAAGGAAAGATTGACTGCGGAT
3 XPC	GAAGTTTCTGAGGAGCCTCCTG	TTCTCTAGCTGGTGACTTAACCCTATT
3 XPC	cacatggCTGCCATTATCATTAGTT	CAGACTACATTGGAAAGGAGATTTGCTA
3 XPC	TCCTCATCATCTCGAGCAGAGTAAA	ACACAAGGAATGCCTGCTTTCT
3 XPC	GAAAGAACCCACACTCCGTGAATA	TTGACCCGGCTGGTATTGTC
3 XPC	CTGGCAGCCAAGGCCTTA	GGGTCCGAGATGTCACACA
3 XPC	TCCAGACTGGGTCGTACCTC	GTTCTGTGAGCAGGAGGAAAAGT
3 XPC	CGTGCACACAGTCTACACATACC	GTAAGGACCCAAGCTTGCCA
3 XPC	TTTTACTGCTTGAAGAGCTTGAGGAT	CTCTGATGAGGATTCCGAACCTG
4 AFF1	AGTAAGTGTTGCACAGCCTGTAA	GTACCTTGGAGGTGCAGATGAG
4 AFF1	GAATGCCTAGACCCCAGAGAAG	TGATCCTTCAGCTCCTTCCCAT
4 AFF1	CAGAGATGTCTCCAAAGGTTCCTT	TGATTTGATTTCCTTCTCCAGTCTGATT
4 AFF1	AAGCAGAAAGAGACTGTGATAACAAGAA	CACCGTAGATTAGGTGGTTGCTAA
4 AFF1	ACTAAACAACGAAGCCGCTTGA	ACCTCTTAAGTGCAGGCTTGG
4 AFF1	ACAGTCCTCAAAGAAGGAAATGCTC	ATGGAAGAGTCTTTGTGGTTGCT
4 AFF1	CAAAAGTGCCAGCAGTACCAAG	CAATGCATCCCATCTTACAGAGTGA
4 AFF1	CATTTCACATTCATTCTTCTCTGTGTCC	GTTAGAACAAGCAGCTCAGATCTGT
4 AFF1	GGGACCGACATAAAAGAACTCACA	AAAAAGCATTTCACTTACGATGACGTT
4 AFF1	AAAAACTCACTCAAATTCTCAGCAAGG	TTCCCTTTAGAATGCAGCAGTCTT
4 AFF1	GACCTACCTTCTGGAACCTTGAA	GTGTATAGTGCACCAGGTCCA
4 AFF1	CAGCACAAATGTGTGCACCTT	AAAATAGTTCCCAAGGCATTGAATCAAC
4 AFF1	CGGGCTATTTAAGAGGGATGGTACT	CAAAGAAGGCACTGGAAAAGGATTTG
4 AFF1	GAAGGGTTCTTCCGGAGATACTG	AGAGCAAGAAGAAATCATCTGAAGTCTT
4 AFF1	TCAGATGAACAGACTAGCCACTTTG	AGTTGCAAAATTGAAAAAGACCAAACTT
4 AFF1	TGGACTGTAGTTCCAGACACGTAA	AGCACTTGACTGGGACCCTA
4 AFF1	TTTCCCCAATGCCTTCTCCTG	AAGGTCAAAGGCGGTAAGAACAT
4 AFF1	CTTCCTATGTCACCATCACATCCC	AATCCAAACACCCCAGTAATTACCA
4 AFF1	GTCACGTGCAGTTTCACTTCTG	CTTGCAATGCATGGAGAAGGT
4 AFF1	ACACTTCGAGAGTTCTTCCAAAGTC	AGGCAAAGTGACATATGTAAGCATACAA
4 AFF1	TTTTATTTTCTTTCTAGGACTCTCAGCATGT	TCAACATAGGAAGGCCTCATCTCT
4 AFF1	CCACCAGTACCATCACTCTTGAC	GTAAATGGATGTACGCCTACCTACT
4 AFF1	AACGTGGTTTGTTAAATGGTAGTTTTCC	AGGCGATGAGTGTGAGACTTAGT

4 AFF1	CGAAGAAGTGAAGGAGTTCCTTAGT	GCAGGAGTGTGAATGGACTGG
4 AFF1	CATCCAGCTCCTTCCACACTA	CAGCTTTTGGCATGGAGACTTG
4 AFF1	GCAGCCAAGAACTGAACCAATG	GTCAGCTCTTCGGTCACCTTT
4 AFF1	GGTTCGGCTCTAGTCATCACAAG	GGAAGAGTTTGCTGGTTGGAATGTA
4 AFF1	GCATAGTAAGTTGTATTTGTGTCTGCCT	GACAAAGGAAAATACGCACCATAAAACA
4 AFF1	GCCAACACAAGAGAAAATATTTGCTG	CTTTTGACCAAAGTACTGACTTGCTTTT
4 AFF1	CACAAGAGCTCCCTCCCT	GGCGGCAGCCACTAGTC
4 AFF1	ACTTTGCTCTTGTTCCCCTGAC	AGCAGCTTGGTGTCTCTCAAA
4 AFF1	CCGCAAAGACAGACTCCCATT	GCTGTTTATCTTCTGCTTTCCTCTGG
4 AFF1	CCTAGACCTGCTCTCTCGGATAC	AGTCTCCCCACACTCACCTTT
4 AFF1	CTCAAGCAAGTTGGCCAAAAAGA	AGGCTACAGCGAATCAAAGCA
4 AFF1	TCTTTAAATCACAGCCGTCATCAC	AAGCAGGTTTCTGTCGTCATTGTA
4 AFF1	TCTTTTGTGGCATCTGAAACAGTTTG	CAGGACTTCAATGTCTAGTGTTCAGTAAA
4 AFF1	TCACTCTTGGTCTTATCTTGCCCTA	TCTACCAAAGAGATAAAGGGCCAATACT
4 AFF1	GGGAGAAAGAAGAAATCCACCTTGT	TCTGAGTCTGAGGAACTGTCTGAG
4 AFF1	GGCTCCGCCAAATGGTGA	ACGAACGCGTTTGAAAAGTTTCT
4 AFF1	CAGAGTCAGAAAGCACCAGTGA	CTGCCATCTAAGGTGGCCAT
4 AFF1	CATTCATGGCCGCCTCCT	GCAATGCCTTACGCTTTTCACTAA
4 AFF1	CAGTTCCCCCTTTGTCACCTA	GGGTCTCTTTATCATGGACTTTCAC
4 AFF1	CAAATCTCCCAAGGACCTAGCA	ATCCATGGGCCGGACATAAG
4 AFF1	AAGTGTTGCAATGCAGCAGAAG	CATCTTCAGTTTGGTGAGCTTGG
4 AFF1	GACTTGAAAGTGCCTGCCAAAG	CGGACATTTTCATCTCAAATCCGT
4 AFF1	CCTCTGCAGGACAGTGTTGT	CTGACTTTGGTCAGCCAGTTG
4 AFF1	CAACAAACAAATGGCAGCTGGA	GGGAGGATCTTTGGATTCAGAATG
4 AFF1	CGACAGTGCCACGAGTCAG	TTCCAACGGTTTGCCTTTGTG
4 AFF1	AAGTCTCCGGCACAGCAG	CCTTGGGCTTGTCTTTGGAAGT
4 AFF1	CTATGGCTCCCGAGACCAG	TGGGCCTGAGAGAGCCTTAG
4 AFF1	CACACTTCTCCCTGGCCATAAT	CAAGACGGCTTCCAGGTACTTA
4 AFF1	GGACAGGGTTGGAAAGGCTTT	GCACAATGACTCGGCACTTAC
4 FBXW7	ACAAAGACTGTGAGGAAAGTTTCATTACAA	ACAGTGTCACGAACTCCAGTAGTAT
4 FBXW7	GGAGGAGAGTTGGTGAACGG	AGAACATGCTGGTGAACAAGATga
4 FBXW7	GTCACTCTCCTGGTCCATctc	ACCTGGAGGCCAAAATGATTCC
4 FBXW7	GTTTTCTTCCAACTGTCCTTGCTG	CAAGCCAGGTAGATGAAGAACAGAT
4 FBXW7	GTTCCTCCTCTACCACACGATTC	CAAGAAGTAGCAAGCTGGCTTTT
4 FBXW7	TTTGCAGCAATTAAGTGAGGCATTT	GGACCTCAGAGCAGCCAATG
4 FBXW7	TCACCTGAAACATTTTTAGCCATTCCT	GTACCACTGGGCTTGTACCAT
4 FBXW7	CAAAAGTTGTTGGTGTTGCTGAAC	TGAAATATGCCAGATCATCATTCTTTGTACT

4 FBXW7	CTAAAAGAGGCCAAGGTACTTCACT	CCTCTACCACATCAAACTGTGATGA
4 FBXW7	TGGGCAATGATGCTAATGCTAAATATCT	GAGTATTCGTCAACCAAGAGGAGTTTTA
4 FBXW7	ATCACATAGCATAGGAAGAAAACAGCTT	CTGTGACATACCTACCTGAAAAAGGTTT
4 FBXW7	CTGCTTGGCAGTCTCTGACAATAT	CGGTTTGATACTGAGCTGCATTTG
4 FBXW7	GGCAACAAAACTCCACAGTAAAGG	CCAATAATCTTCTCCGTTCTCTCTCATT
4 FBXW7	CAACCTTATGATTCATCAGGAGAGCAT	AGGGTTGTTAGTGGAGCATATGATTTT
4 FBXW7	TCTGGATCCCACACCTTTACCAT	TCTGCAGAGTTGTTAGCGGTT
4 FBXW7	AGCACACTGTCACTATTTCAGTAACTC	AGAGACTGGAGAATGTATACACACCTTA
4 FBXW7	GCACAGTGGAAGTATGCCCATA	CTTAAGTGTTTTTCCAGTGTCTGAGAAC
4 FBXW7	ACTCCACCTGTATGTCCCACTAAT	GGATGTAATATTTTCTGAAGAGCCAAACA
4 FBXW7	ACAATGTTTAAAGGTGGTAGCTGTTGA	ACTCATTGATAGTTGTGAACCAACACA
4 FBXW7	GGGTTCTATCACTTGCATCATATGTTTT	CCTTTATAAGCCTGTAATTTGGGACATCT
4 FBXW7	CCAGTTGCTACTTGCAATGATATACAC	TGATCACATGCTTACAGTTTTGTGGTAA
4 FBXW7	TTGTCATCAGAACCACTAACTATTCGG	TGCCTTCATTTTTCTCTTCACCAGTATT
4 FBXW7	ACAGTTTGCCAAGTGAAATAGTACACT	GCATACATCAGACAGCACAGAATTGAT
4 FBXW7	TTCTCCTCGCCTCCAGTTAGT	GGTGAAGGCAATTTACTCTTGAACTGTA
4 FBXW7	TTTCTACTTGTTTTCAGAATCACTCTGCT	GGAGAATTTTGGCTGAAGACAACCT
4 FBXW7	CTTACCCTCTTCTTTGCATTTCTCTCT	TTTTCTTAGTTGGCACTCTATGTGCT
4 FBXW7	GGTTCCAGGAATGAAAGCACATAG	TATCAAGTATCTCATCCTGTGGAGaataattaa
4 FBXW7	GTTAAACAGTCAACCGTACTAGTAACA	GAGGTCCGCTCTTTTTCTTTGG
4 FBXW7	CTGAGACTTTGCATGGTTTCTTTCC	AACCATGCTGACTCAAGATTTGATAGTT
4 FBXW7	GCACCACTGAGAACAAGGGAttt	GCTGGTGCTGGACTTTGATGT
4 FBXW7	CATCTTTTCTGCTCTTCACTTCATGTC	CGAAACCTAGTCACATTGGAGAGT
4 FBXW7	CTCTGATCCGCCACACAACT	TGTGACCTGTTTACAGTTCAACAAGA
4 FBXW7	CCATCATCTGAGCTGGTAATTACAAAGT	AACTAACTCATAGCCATTATTTCTAACCAGTAA
4 FBXW7	ATGAGGTTGACTCTTTTTGTGATGCTA	CTCTGGGAATGCAGATTCTACAGTTAA
4 FBXW7	CACTGTCCTGTTTTGATATCCCAGATT	CTTGATACATCAATCCGTGTTTGGG
4 FBXW7	AATGCAATTCCCTGTCTCCACAT	CCCTTTCCTACTAGGATTAAGGTCAGTA
4 FGFR3	CCTATCGCTCTGCTCTCTCTTTGTA	ACGCAGAGTGATGAGAAAACCC
4 FGFR3	GCCTGGCGGGCAATTCTATT	CGGCCGTAAGTCACAGGATTC
4 FGFR3	gCCCCTGAGCGTCATCT	TGTGCGTCACTGTACACCTTG
4 FGFR3	GCGACGTGGAGTTCCACTG	CACCTTGAGCACGGTAACGTA
4 FGFR3	TGGAGGTGAATGGCAGCAAG	CTCCAACCCCTAGACCCAAAT
4 FGFR3	CCAGCCTCGATCTGTACCTT	TCCCGCTCCGACACATTG

4 FGFR3	AGTCCTGGATCAGTGAGAGTGT	GGCATGGGACAGAGTCGTTA
4 FGFR3	GGCAGGTTGGGCATTGGT	GCCTGAACCCTGGACCCT
4 FGFR3	GACAGCCTGACCTCACCTT	GGTCGTGTGTGCAGTTGG
4 FGFR3	GGAGCTCTTCAAGCTGCTGAA	CCCGCCTTATTCGGGAACA
4 FGFR3	GACGTGCACAACCTCGACTA	CTCGGTCAAACAAGGCCTCA
4 FGFR3	CCTTCCCACACCCTCCCA	CGCCTCCACCCCTGAAG
4 FGFR3	GGCCTCAACGCCCATGT	GGAGCCCAGGCCTTTCTTG
4 FGFR3	GCTTCTTCCTGTTCATCCTGGTG	CAGCTCAGAACCTGGTATCTACTT
4 FGFR3	GGTGCCTGCCTCATGGTT	ACCAGCCCTGTGCCATC
4 FGFR3	GCCCACTGTCTGGGTCAA	GCACACTGAAGTGGCACAGTA
4 FGFR3	GCTGAATGCCTCCCACGA	GCACTCGGCTCCTTTCTGTAG
4 FGFR3	GGGCGAGTTTGCACACTCA	CCCTCCTCACCTTTCAGCATC
4 FGFR3	CTGTCACCGTAGCCGTGAA	CCTTGTCAGTGGCATCGTCTG
4 FGFR3	TGTCACCGTAGCCGTGAAG	ATCTCAGACACCAGGTCCGA
4 FGFR3	TCATTCAATGCTGGTGGAAGTCA	GGTGTGTTGGAGCTCATGGAC
4 FGFR3	GGTGTCCCTGGAGTCCAAC	GCCCTCAGCACCACTGA
4 FGFR3	GCTGCCTCCGCTCACTC	GCCGGATGCTGCCAAACT
4 FGFR3	CCTGCGTCGTGGAGAACA	GGGCAGATGACGCTCAGG
4 FGFR3	GGAGAGGTGGAGAGGCTTCA	CTCACGTTGGTCGTCTTCTTGTA
4 FGFR3	ACAGACGATGCCACTGACAA	GCCGCTACCGCACCTAC
4 FGFR3	GTCTGAGGAGCCCGTGTC	TGCAGGTGTCGAAGGAGTAGT
4 FGFR3	GGGTAACCTGCGGGAGTTT	CTTCTGGGAGGCCAAGTACTC
4 FGFR3	TGGTGTCCTGTGCCTACCA	GCAGGGCTGAAGCCTCTC
4 FGFR3	AGGTGGCTGTGCGAAGAG	CGGAGTCGTCCCCTGAG
4 FGFR3	CGCCTTTCGAGCAGTACTCC	GCTAGGGACCCCTCACATTGT
4 FGFR3	CGGGTCATGGCCTTCACAC	GAACTCCCTGCCGTTCTTCA
4 FGFR3	AACCCCACTCCCTCCATCT	GTGAGTGAGCGGAGGCA
4 FGFR3	CCTTCAGGCTGTTCCCGAATAAG	CGGTGGACGTCACGGTAAG
4 FGFR3	GGAGGACCTGGACCGTGT	CGCACAGCCACCTCTGT
4 IL2	AAGGCCTGATATGTTTTAAGTGGGAA	GGATCTGAAACAACATTCATGTGTGAA
4 IL2	AATGGTTGCTGTCTCATCAGCATA	CAGGCCTATAAGACTTCAATTGGGAAT
4 IL2	TGGTGAGTTTGGGATTCTTGTAATTCT	ACACAGCTACAACTGGAGCATTT
4 IL2	CATCTGTAAATCCAGCAGTAAATGCTC	GCCACAATGTACAGGATGCAACT
4 IL2	GACTTAGTGCAATGCAAGACAGG	TTGACACCCCCATAATATTTTTCCAGAA
4 IL2	TCCAGGAGAGCAAATAAAGTAATGCC	GGCCACAGAACTGAAACATCTTC
4 IL2	AGAGGTTTGAGTTCTTCTTCTAGACACT	AGTGACATTGAGAACAGAGAGAATGGT
4 IL2	ACTCTTTACCTCAGATGAGCTGCTA	AGAATTACAAGAATCCCAAACTCACCAG
4 IL2	TGTAAAACTTAAATGTGAGCATCCTGGT	GCTGGATTTACAGATGATTTTGAATGGA
4 KDR	GCTTCTTGGATGGAGGTGACAA	AGGCATTTAATGAAATCAGTGTTTTGCT
4 KDR	GGAACGGTGGTTTGGCTGAT	AAGTGAGTGAAAGAGACACAGGAAATT
4 KDR	GGGATTGGTAAGGATGACAGTGT	CGTGTGAGAGCAAATCCTTTGACTATA

4 KDR	TGACCTTCTATTATGAAAAATGCCTCCA	CGTTTTCTTCCTCTGTTAGGCATTGTA
4 KDR	GTTCCGGTTCCCATCCTTCAA	AGAACAAGATGAAGGGCAGCATTTATAT
4 KDR	TGATGCTAATTTCTCCAACCAACA	CTGAAAACTTTGGAAGACAGAACCAAAT
4 KDR	CTGAGTCTTACCCAAAAGATGGAGATA	CTGTGCCATCTCATTGTCTCTTAAAAAG
4 KDR	GGAGTCTGGATGGAAGGACAAA	TGAATCCTTGTCTTCTACCCCTGTAG
4 KDR	TCATAGCTCAGCTGTAAGAAATGCAA	CTACCAGTACGGCACCACTC
4 KDR	CATAGACCGTACATGTCAGCGTTT	AGCTGACTGACAATTCAAAAGTTGTTTT
4 KDR	ctgacTGCATGTGTGGCTAGTA	TTTGGAATTGACAAGACAGCAACTTG
4 KDR	CAACATTTTGACTGCTACTGTCCTG	CTTGTTGCTGTTGTTGTTGACTCT
4 KDR	TCCCTTCATTTTATAACATGGCCAGAG	AAACCCATTTTCCTTCCTGGACAT
4 KDR	ACCCAAACCTCCAGAGAAGAGTA	GTTGTTTATGGCCTGGGATTTGTT
4 KDR	AGAATCCAACATCTGAATACACCACATT	GAACATTGTGAACGACTGCCTTATG
4 KDR	GGAATTCCCATTTGCTGGCAT	GTTTATGACTGGAAGGATTGATGCTTTC
4 KDR	TAGATCTAGAATGAATCCTTACCTGCAA	GGTCTAACTCAGTGGCAACTACAT
4 KDR	TAGGAATCCGCAAAGTATTCTAAGCTA	GTCACCATCTCAATGTGGTCAAC
4 KDR	GCTTGGTACAGGCACCTAGAAG	TGTCCATACCTATGATTTCCCTCTTTGTA
4 KDR	AAATTATCTCACTTGTCAAGGCACAGA	ACTATAGATGGTGTAACCCGGAGTG
4 KDR	GCACAGGTGTACAATCCTTGGT	GCTTTGGAAGTTCAGTCAACTCTTTTT
4 KDR	CCATAGCATGCAGGAAGCACTA	AATCCCTGTGGATCTGAAACGG
4 KDR	TCTCCTAGAGGACAGTAATCTTTGCAT	CTTGCTCAAGACAGGAAGACCA
4 KDR	TCAAAGGATTTGCTCTCACACGAA	AGAAAAACCTTTTGTTGCTTTTGGAAGT
4 KDR	CTGACCACGCAATGTCTTTTCT	GAAATTGAATGCCACCATGTTCTCTAA
4 KDR	TTCCACCAGAGATTCCATGCC	TGCAGCTACTCAGACCCTCAA
4 KDR	CTCCATGATCAAAATGTCATTTGTGCTA	GCAGACAGATCTACGTTTGAGAACC
4 KDR	GGCCAAGCTTGTACCATGTGA	ACCATTCAATAACTATGGCCTCTTCATT
4 KDR	AGATAGCTGATTTCCCCTCAACCT	GGAAATACTGATCTCCATCCTCCTG
4 KDR	ACCATCCTTCCATTAAAGAGAGAGAGAT	GCTGTCTCAGTGACAAACCCATA
4 KDR	ACACTTCTCCATTCTTCACAAGGG	AGGCTGCGTTGGAAGTTATTTCTAA
4 KDR	AAAGTTCTGCAACCCAAGAGTGA	TGGAGAATCAGACGACAAGTATTGG
4 KDR	GCATGAGACTTCGATGCTTTCC	GCTGATACCAGAACCATTTCAAAAGTT
4 KDR	CCTTCTTCACTCTATGTTGTTATCTCCAA	CTATGCTGGCATGGTCTTCTGT
4 KDR	TCCTCTTACCTACAACGACAACTATGTA	ACCCAGAAAAGAGATTTGTTCCTGATG

4 KDR	TGCTGTCCCAGGAAATTCTGTTAC	AGTGATCATTGTACATGGCTCTCATTTT
4 KDR	GCCTTCTAATGAGGCTCCAAAACT	GCCTGTGAGTGTAAAAACATTTGAAGAT
4 KDR	CTTTTACTTCTGGTTCTTCTAACGGGAT	GGTAGCCGATCTGCAGTCAAATATAC
4 KDR	AAGAACCCCAGTCAGTTTTCATTAATCA	CAGGAAAAGACGAACTTGGAAATCAT
4 KDR	ACCGCCGTGCCTACTAGAATA	GCTTGGGATTTTGCTTTAGTGCTAA
4 KDR	AAAACTTAAGAGACGATTGGAGGAGATG	GGAGTCGTGTACATTACTGAGAACAAA
4 KDR	GACATGGAATCACCACAGTTTTGTT	AGTTTAGGAACCTGAAAAATGACCAACT
4 KDR	ACACAGGTCCTGAAGCTCTCTA	CTCTTATCGGAGAAGAACGTGGT
4 KDR	GCCAAGCCAAAGTCACAGATTTTA	GGTCACAACAGATGTGAAAGAGATCAG
4 KDR	CTGGTAAAGAGACGTGGGAAATGA	GCCTCTTCTGTAAGACACTCACAAT
4 KDR	AGTGTCATTTCCGATCACTTTTGGA	GGCACTTCATATTAATACCTCCCTGAAA
4 KDR	GAAAGTCTTCCAGGCAAGGAGAA	GATTGATGAAGAATTTTGTAGGCGATTGA
4 KDR	GGGCCCTCATTCTAGTTCCTTCT	GATACATAGGTGATGAGGATGTGCAA
4 KDR	CCTCCAGAGTGGGCTCCTTA	TCCCGAGTTCTGGGCATTTC
4 KDR	CTTAGACAAGGTCTTCCTTCCACTT	CTACCTCACCTGTTTCCTGTATGG
4 KDR	GGGTCACATACTTCCTCCTCCT	AACTGAAGCCATAACAACAGTCTTCT
4 KDR	GCCAAAGAGGCCCCTATCTCT	GCTTGTCTTAAATTGTACAGCAAGAACT
4 KDR	GTTGAAGTCAATCCCCACATTTAGTTC	GCTTATTTGCTCTCTCATGTTCTGTTTT
4 KDR	CTCTCATGTGATGTCCAGGAGTT	GACACCACCGTGTACTCCAG
4 KDR	TCAGCTTTAAAAGTTCTGCTTCCTCA	CCAACTCCTTTGTTTGCCATTTCTTC
4 KDR	GCTACTGGTGATGCTGTCCAA	TTGTATTCAAGGAGTTTGTTTGTTCCT
4 KIT	GTCAGTCCATATGTCCAGTTGCATA	TGCATCCCAGCAAGTCTTCAT
4 KIT	CCCTTGTCTTGCAGGTATGACATA	CACACCTGGAACTGGGAGAAG
4 KIT	CAGCACCATCACCACTTACCTT	GCATGCAGTACCATACAGGAACTTAC
4 KIT	GCCCTTCTACATGTCCCACTTG	TCTTGGTTGCTTGATAGGTAGGTACT
4 KIT	TTGCCAGTTGTGCTTTTTGCTAAA	CAGCTTGGACACGGCTTTAC
4 KIT	GATGGTACTCAAGTTATCACTCCACATT	CATCCACTTCACAGGTAGTCGAG
4 KIT	TTGACTCTGTTGTGCTTCTATTACAGG	TGAAGTTGTCTTTGGCAAGGATCAT
4 KIT	AAAGTAAGCTGTACACATTTGAGGAGAA	AGATAGCTTGCTTTGGACACAGAC
4 KIT	CTTCAAAGCTGTGCCTGTTGT	ATGAGAAGAATGAAGCGATTCACTCA
4 KIT	AGGTAAAATGATCCTTGCCAAAGACA	GCCTTCCTTGATCATCTTGTAGAACTT
4 KIT	CTGGAATGCCGGTCGATTCT	TCAACATCTGGGTTTCTGTCTCTAAAAA

4 KIT	TCCTAGAGTAAGCCAGGGCTTTT	CCATTGTGCTTGAATGCACTAGAATCTA
4 KIT	TGGAAAGCTAGTGGTTCAGAGTTCTA	AGCCTAAACATCCCCTTAAATTGGATT
4 KIT	CACAGAAGATGGAACTCAGTATTGGA	CCACGCGGACTATTAAGTCTGA
4 KIT	CACCATCCATCCATCCAGGAAAA	CGTGATCCATTCATTCTGCTTATTCTCAT
4 KIT	GACTTTTGAGATCCTGGATGAAACGA	CACTGCAGAAAGCCAAGCATTT
4 KIT	TGAAAGTTAATATGGAGAAGTTAATTGCTGCT	CACCGTGATGCCAGCTATTATATTTCT
4 KIT	CCTTTTCTGAAACCAGCAGACTAAACT	GTTGTGACATTTGCTGATCCAAAAGTAT
4 KIT	TGGAGTGTTCATGTGTTATGCCAATA	ACTGTCTAATAATTTCTTCTTTCACTACTGCC
4 KIT	AATGGTTTTCTTTTCTCCTCCAACCTAA	GGTACTCACGTTTCCTTTAACCACATAA
4 KIT	CAGCCTCAGGAAGGTTGTAGG	CTGTCGTAAGTCAGGATTTCTGGTT
4 KIT	GCCATTTCTGTTTTCCTGTAGCAA	CTCTGCATTATAAGCAGTGCCAAAAATA
4 KIT	tGTCATTGCCACTGTCTTTTCCT	ACCTGGTAAGAAAAGCTCAGCAA
4 KIT	GAGTTGGCCCTAGACTTAGAAGAC	GGCTCTAAAATGCTCTGTTCTCaaaaa
4 KIT	CCAAAGTTTGTGATTCCACATTTCTCTT	CACTCTGGAGAGAGAACAAATAAATGGT
4 KIT	CTGTGACCAGCCATTCCAACT	CACCCCTTGAGGGAATAATTGGT
4 KIT	CCTCTCACAGACCCAGAAGTG	CCTCCTGGTCCACAGAACAATG
4 KIT	GCCTACCATCGGCTCTGTCT	ACTAAGGTGGATCAACGAGAAGAGAA
4 KIT	ACCAGAGCTCGGATCCCAT	CAGGCTTCGCCGAGTAGTC
4 KIT	GATTGTTGAATATGAAGCATTCCCCAAA	CCAGGGCAAGGTCCATTTCTTAC
4 KIT	TTTCCATCAGTTAGTTGTGATCTTGACA	GCTGCAGTTTGCTAAGTTGGAGTAA
4 KIT	GACTATGGGCTTGTTTTCTCCAGAT	GGTGACCCAAACACTGATTCTG
4 KIT	AGGCATAGATTTCCAGGTAGAAACTG	ATTGACGTCAGAATTGGACACTAGG
4 KIT	CCGAAGGAGGCACTTACACATT	CTTGCAGCTGTTACAAACACGAG
4 KIT	CCCCACAGAAACCCATGTATGAAG	TGGAAAGCCCCTGTTTCATACTG
4 KIT	GGCCCACCCTGGTCATT	TGCAGAAGATTCTTATAAAGTGCAGCTT
4 KIT	GTTCAAAGCAGGAAGATCATGCAG	TCAGCCTTGATTGCAAACCCTTAT
4 KIT	GTCTAGCCAGAGACATCAAGAATGATT	TGCTAAAATGTGTGATATCCCTAGACA
4 KIT	GTGCATTATTGTGATGATTCTGACCT	TCTCCTCAACAACCTTCCACTGTA
4 KIT	CTATTTTTCCCTTTCTCCCCACAGA	TGACCAAAACTCAGCCTGTTTCT
4 LPHN3	GCTTAGGTATGTTTCTCAATGAGCACT	CATTTCGGCCATTTTGCTTTAAGGTAT
4 LPHN3	GCACTATCCAGCTGTCTGCAA	CTAATATTCCCGGCCAAAAAGAAATGAA
4 LPHN3	CCCCTGTAGCAGTCAGAGGA	GGTTACAAGAGCATGTAGTATGTGTCT

4 LPHN3	CAATGACAGGTTATTGGTCAACACAAG	TTACCTTAACTTCCACATGTGCCA
4 LPHN3	TCACATTTGAACAGTCATTCTTGAGGA	CAAGGAAGTGTCCAGTATGAATAAGTGA
4 LPHN3	TGTCCCATACCCAGTCCTTGA	AGTTTTACTTCTTGATCTGGGAAACACA
4 LPHN3	ATTGTCTACTTTTGTTCCTACCTGCAA	AATGGAACTCTCTGTACTTTTGCCA
4 LPHN3	CCTGCGAACACATTGCTGTAG	TCCAATCATAAGGGACAGTAACTGGTAT
4 LPHN3	ACCATGGATGTGAAGTCATTCTAAGAAAAA	GGAGAAGAACAGTTGCTGAGATCTG
4 LPHN3	TTGCTCCTGATGGAATTTGGGAT	TTCCAACAGCTATCATCAACAACAGA
4 LPHN3	TTTAGGAGAGATCAGAGTGGCCTT	GAACTCTTTGTTTATTGCTGCCGTAAT
4 LPHN3	TCATTCTGTTATTGTCAATTCCCCTGTT	GAACAACCAAGCTAATATGCCATTTTCT
4 LPHN3	TGATTTGTGTGAAAATTCACTGCATCAT	CGCTGAACTGTTTATGTCTCCAGTAATA
4 LPHN3	TTCTGTGTTTAATCTGCTGTCTTAATGGA	CATCACAACAGAGACATTCAACAACTT
4 LPHN3	CGAAAGCAGTCAGAGTCTTCCTT	AGGTTCAGAAGTTGCAAATAAAGCAATG
4 LPHN3	CAGTGTCGTATTGTTGATCTGATATGGT	TCAATCCTAATAGGCAGAGAAGAGCTA
4 LPHN3	CAGGTCATGGGTTATAGGTGCAA	CCCTGTAGAGAATTGAAAATGGTGAAGA
4 LPHN3	AGCACAGTCATCATGGCCTATC	GCTTTGCTATCATGAAATGTATGCCATT
4 LPHN3	GGGTTCACTAGAGACCTCTGTCAT	CACAAAAGCACTTTCCTCCACAG
4 LPHN3	CGGCCACCATGTTGCTTCATA	ACCTTCTGAATAACAAAGGCCACTTAC
4 LPHN3	CGCAACTTATGGCTTTTTCATTGTGT	GGATGGTGTTACGGTCACTCTG
4 LPHN3	AAAGCGAACACATGTAATTTTCCTTTGT	TGGACAGCGAAGCTCTATAGGATAG
4 LPHN3	CTGATTTGCATCTTCACATTTTGCTTTT	CTTCACTGGTATCAATGTAGGTTGCT
4 LPHN3	CGCAGAGAGCTATCCTGTGAGA	GCATCTGGCAGATAACATCGG
4 LPHN3	TGACCCTGCTCAGATGGAGAATAT	ACATGTAACACAGATTGATGTTGAAGGT
4 LPHN3	GTTAGAGTTGCATTCCATTTGGTGAAA	GTCGGACTCAAACAAATGTTCACTC
4 LPHN3	GTCCTGGACTACTAAAAGGAGTATACCA	ACTCAGTCAGGGTATCAGTTCTGT
4 LPHN3	AGATTTATTATATGCCCTGGACTCCCT	GTGCGCTCTTTGTTGAAGAACA
4 LPHN3	CAGGATTTGTAGTGTATGATGGAGCTT	CTATGTCAGATTTGCCTCCCCAT
4 LPHN3	CAATTACCATGATACCTCCCCTTACC	GCAGTATCCCATGTTCCTTCGAT
4 LPHN3	GTGTGTGTTCTATGGCTTGTTGG	CCGCTGGCAATGCTGTAACTAT
4 LPHN3	CCACTGAATGGTAACCATGGCA	GCTCATGGTTGTTCAGGTAAGAAG
4 LPHN3	TCTCCAGTCACCCCATAGAGAC	GCCTAGGTTTGGCATGCTTTT
4 LPHN3	TGGTGATGCCGAAGATGTTTACTAC	GTCCCATCTTTGTTTGGAGGAACT
4 LPHN3	CTGAAGGAACTCACTTCCAACTATATCC	GTGGTTAAACGAGGTGGCATC

4 LPHN3	GCGGCAGCAGTGATGGATTTAT	TGTTGGTTCCAATTTCTGTGTCATCT
4 LPHN3	CTGTGAACAAGCAATGTAGGTGTT	GTACTCCGGTTTCTTCTTCCTGAC
4 LPHN3	TGCTAATGTTGGTGTTCTTGTCTTTATGT	GGTTCATGGGACCACACTGATAA
4 LPHN3	GAAGATGATGCCATTGTCCTGGAT	CCTTCTGGTATAATGGTGTGGCT
4 LPHN3	GGAGTACCACCCCGTCAGT	ATGATTTCTCGGGCTTCCACA
4 LPHN3	AGTATACTCCACCGAGAACCAC	TCGGCATGCTGGTATAGAGAGA
4 LPHN3	CAAATCCCAGCTCTCGAAGAGA	GCAACACAACAGTTTTAACTTAGTGCTT
4 LPHN3	TGGCAGTTGACTAGTATCCCATGT	CCAGGAACATCCAGGTGAAGG
4 LPHN3	CTGCCCTGTTACATTTCTTCTTCTTG	CTGACACAGCCACAATGAGTG
4 LPHN3	CTTTTCCTTTCTTTTGACTTCAGATGCAA	ACACATTCATACTGCACTTCAAGGTA
4 LPHN3	TTTTATCTGGTCGGCTATGGGATG	ACCTTTGCTTCCAAGAAAGTCCAA
4 LPHN3	ACCCGTGTCCAGGAACCTATAAA	TGTCAATGCATGCAAAACACAGT
4 LPHN3	TCCCAGAACAACTCATGTGTTATAGAGA	AAGACAGCCTGATTCAGGTTTCA
4 LPHN3	TGAAACCTGAATCAGGCTGTCTTG	TTTAGCAAAGACAATACACCCATATTCT
4 LPHN3	GCAAACATGATCGTTGAAAGAAAGCA	TTGTCTGTTCAGCCAGCTCTC
4 LPHN3	GAAACAGCTGCCAACATTGCTA	AAACTCCGGGCAGCACTATC
4 LPHN3	GAACTTGACCCCAGGTGGAAAA	AAGGAGGAAAATTAAGCAGTGAAGTCT
4 LPHN3	AATTGAACCCTTACACCCTACGG	AGTAGCCTCATTGTCATCATCCTCATAT
4 LPHN3	TGGAATTCTGTATGTGGTCAAATCTGT	ACAGCTGCAATGTACTGGTATGAAT
4 LPHN3	GTTTGGTGGATGTACCCTTTCCTA	CAAAGAGTACCACAAAAGGTTGAACTT
4 LPHN3	TTCTCTCTCTCCAATAGAAGTGGAACA	TGAAGTTGAGCAGCAGAAGTAGtaaat
4 NFKB1	GGCCAAATAAAACTAGGTATGAGAGCA	TGAGAATGAAGGTGGATGATTGCTAAG
4 NFKB1	ACTGTGTTTTCATTCCAGTGTCTTACA	CTTCAGTTGTTTTCTTGAGATTTCTGCT
4 NFKB1	GACAGACACTAAGTTGTGCTGAGT	GCATTACTTACCATGCTTCATCCCA
4 NFKB1	TGTTTCATTTGGATCCTTCTTTGACTCA	GCATTTTACTAAGACTGAAAGCAGAACAA
4 NFKB1	TGTCAAGGTGTCAGAACACTGATG	ATTGGCATGCCTCTTTGCAAG
4 NFKB1	CCTCTTTCTAGAGAAGGCTATGCAG	CCAGGACAACCTATCATGCAAAAGT
4 NFKB1	CTGTCCCTTTGCTTGGACTCTA	AGATGTTGTCGTGCTCCACAG
4 NFKB1	AATGCTCAGGAGCAGAAGTCC	GCAGCTACTGACTTCCTTCTTTGA
4 NFKB1	AGTTTATTCCTGCATGAATTCCATGGT	GGCCTTCCCAAATATGGATCATCTTC
4 NFKB1	gttttAATACACAGCTTCAGAATGGCA	GCTAACTTACTAATGTTGCCTTTGCAT
4 NFKB1	AAGGGCAAAAGAACCTTCCACTAA	ACCGCCAGATCACCATCTTAC
4 NFKB1	TGTTATTTGTTGTTTTTCCCCTGTGAAC	TCACACCTCTTGAGATCAGAGATCA

4 NFKB1	ACTGGGACTCGAACACAAGAAC	GCCTGGATCACTTCAATTGCTTC
4 NFKB1	CCTGAGACAAATGGGCTACACC	AGCTGGAAAATATCTCCACTGTCTTTT
4 NFKB1	TGCAGCAAACTTGTCTCTTACCT	GTGTTTTTGTCATCACTTTTGTCACAAC
4 NFKB1	ACACTGAATCTAAAAAGGACCCTGAAG	CTTCTTTTGTTCCTGTTGCATACGTT
4 NFKB1	TGGGAATGGTGAGGTCACTCT	AGATATCTTTCTGCACCTAGGACTGA
4 NFKB1	GGGCAAGAGTTGTCCACAGAAT	GTCAGAGACTCGGTAAAGCTGAG
4 NFKB1	GTGGAGACATCCTTCCGCAAA	CACGGTGTGGGAAATTGTCAG
4 NFKB1	TGTCTATGGCATGTTAGAACAAGTGT	AAGGCATTATTAAGTATCCCCAGACCTA
4 NFKB1	CAAACTTAATTGGCTTAACGTTCACCTT	CAAATAGAAGGAGAGCTACCACAAACTT
4 NFKB1	GGCTACTCTGGCGCAGAAAT	GCCAGAGATGTTAACAGAGTAAAGTCAA
4 NFKB1	ACTGCTGACCAGTGAATCTCC	GCAGCTAGGTGCAAAACAGAGT
4 NFKB1	CTTCTGGACCGCTTGGGTA	ACACCAATGATGTGTGATTGTCTCTT
4 NFKB1	CCTGGGCATGAATGGACTGT	AGTGCTATCCGGAAGAAAAGCTG
4 NFKB1	CGTGGTGCGGCTCATGTTTA	GCCCCCTGTTGGAAGTGAAG
4 NFKB1	ACAAGTGAAGCCAGATTCCCTAGA	CCCTTATACACGCCTCTGTCAT
4 NFKB1	AGTATTTGAAACACTGGAAGCACGA	CAAGGCCAGGAAAATGATGCTTAC
4 NFKB1	TCCCTTCGATGTATAACGATTTCTGG	CTCCCTTTAAATGACAGTGTGGAGATAA
4 NFKB1	GGAGATTGCCTCAAGCTGCTA	ATTTTCCCAAGAGTCATCCAGGTC
4 NFKB1	GTGGAGAACTTTGAGCCTCTCTAT	ATTCCTGAACCTCTGACTCCCT
4 NFKB1	CCCATACCTTCAAATATTAGAGCAACCTA	GCTGTTACATAATTTGTCAGGTTGTTGA
4 NFKB1	ccatgtTGCTGGAGAGTCAGA	TGGGCATGCAGGTGGATATTTTT
4 NFKB1	GTTATTGTTCAGTTGGTCACAAATGGA	CCACAGCATCATATACCCCTACTTAC
4 NFKB1	AGTGGTCTTTCTGTGGCTAGTG	CGAAACTATCCGAAAAATTGGGCAT
4 NFKB1	GCAGAGGAAACGTCAGAAGCTC	TTGTACCTGGACCTGTACTTCCA
4 NFKB1	GCGGAGGCATGTTTGGTAGT	AATATACTCAATAATATCATAGCCACTCAACTC
4 NFKB1	AGCATAAGGAATGTGTTTAATGAGTAGCA	TCCTCCGAAGCTGGACAAAC
4 NFKB1	GCCATTGTCTTCAAAACTCCAAAGTAT	AACAACTGACTTACCTTTGATTTCAGGA
4 NFKB1	AGAATATTAAACCAGTTTTATTTTTCAGCATGT	GACTCCACCATTTTCTTCCTCTTCATAA
4 PDGFRA	TGTACAGTTAAATAATAGTAAGTTCTGAGTGTC	AGGATGGTCACTCTTCAGGAAGT
4 PDGFRA	AAATTCACCTGGACTTCCTGAAGAG	CCACTGTCAGCGCTCAGT
4 PDGFRA	GGTGGTCTGGATGAGCAGA	GTCCTCTCCTTCAGGGCCTAT
4 PDGFRA	CATTGTGCCTCTCTCTCTTGTCA	GCTTGTTCTCATTGGCTTCAAAGAT
4 PDGFRA	GCCAGTGGGATAGTTTTTCTGGATT	CGACAAGGTATAATGGCAGAATCATCA

4 PDGFRA	ACGGATTATTTAGTCATCGTGGAGGA	AGGGCCCTACAGTGAAGGT
4 PDGFRA	ACAGCAGACAGGGCTTTAATGG	CGCACCTTATGATTTTGCCTGTT
4 PDGFRA	CTCCACTCATTGCCATGACTCT	CCTCCACGGTACTCCTGTCTC
4 PDGFRA	TCAAACATCATCACGGAGATCCAC	TGTCCTGACTGTTGAGGAACTCA
4 PDGFRA	gtgaacgTTGTTGGACTCTACTGT	ACCCTCCAGCGAATTTCATACC
4 PDGFRA	CTGTCCTGGTCATTTATAGAAACCGA	GGAGGTTACCCCATGGAACTTAC
4 PDGFRA	CGCTTTCTAAAATGTCAGTTGTCCA	CCGCACCTCTACAACAAAATGTTT
4 PDGFRA	GCTGTCAACCTGCATGAAGTC	GCATACTTGGGCAGAGAGTTTCTTTAC
4 PDGFRA	AATGCCAAAGGCTTTCGTTTGT	CAATGGTCTCGTCCTCTCTCTTG
4 PDGFRA	GTTCCAGCAGTTCCACCTTCAT	GGAAGGAACCCCTCGAATCC
4 PDGFRA	GCACCCTGGGTAAGATTTCTCTT	CTGGATGTCGGAATATTTAGAAACCTCT
4 PDGFRA	AGTATGTCCCCATGCTAGAAAGGAA	CACCCAGAGTGAGTATAGACACTTTTAC
4 PDGFRA	ACAATTGGAACTTACTTAGCTACTGCTT	TTGAGCTACAATAGTATAATGGCCACTG
4 PDGFRA	CTGATCCGTGCTAAGGAAGAAGA	AGACACTCATCTACAGAGCTAGCATTAT
4 PDGFRA	CATACCCATCTCCTAACGGCTTT	AGCTCTTTCTTTGGCTTCTCTGG
4 PDGFRA	GATAGCTTCCTGAGCCACCAC	GCCTGTAAGATAATGATAGACAGCAACA
4 PDGFRA	CTGTGCCCACTCTTGAGTTCT	TTTCCACCCACAGATCCAAACA
4 PDGFRA	GGTTTAACTGTCTCCCTCCTTCC	GCCCAAATAAGCAGCAATGTCA
4 PDGFRA	ACCATGGATCAGCCAGTCTTG	GGCCAAAGTCACAGATCTTCACAAT
4 PDGFRA	GTCCTCCTGGCACAAGGAAAA	GAATCTCTAGAAGCAACACCTGACT
4 PDGFRA	CGTCTGGAGTTTTTGGGTGTT	CCAGGAAGGAGCACTTACGTT
4 PDGFRA	GAACTAGGCTCCAGGGTTGTT	CAAGGAACTCAGAGAGGACTGG
4 PDGFRA	CATGCCTCTGCAACCTGATGA	TGATAGGTGAAGCTCAACAAATCCAAT
4 PDGFRA	CAGATGATAACTCAGAAGGCCTTACTTT	GGGTCTAAAAAGGTCTGTGTTCCTT
4 PDGFRA	GAGAACAGGAAGTTGGTAGCTCAG	CCAGCAAGTTTACAATGTTCAAATGTG
4 PDGFRA	GTCTGAACTGAAGATAATGACTCACCTG	AGAGTGGAGGATTTAAGCCTGATTG
4 PDGFRA	CGGTTTTCTTCCCCTTTTGCTGTA	CTTCATTTCTTTGACCTCCCTGGT
4 PDGFRA	ACAGTGGAGATTACGAATGTGCTG	TCTCTGTACAGGGAAGAGTTTCCAT
4 PDGFRA	CCAAACTTTATAAGATCCTGGCTATCCT	GTTTCCCCTGACTTATACACGGTTTTAA
4 PDGFRA	AGCTGGATCTAGAAATGGAAGCTC	attGCACGTTTTGAGGGTACCTA
4 PDGFRA	GTCCTGGTGTTTTATTGTTTGGCTTT	CCCACAGACTGTGAACACAGG
4 PDGFRA	CTGTCCTTTCTGACTGCATCCTAT	GTACTGCCAGCTCACTTCACT
4 PDGFRA	TCATCCTTTTCTCTGAGATGCTTTGG	CAAGTGTACAACCCTGTGTGG
4 PDGFRA	TTTTGTGACGGTCTTGGAAGTGA	AGGAAGAGAAGAAGCTTGGTCCT
4 PDGFRA	CCCCTCCGGAGTGTTTTGAA	CCAGTTGAGCCATGGTGATCATC

4 PDGFRA	CCTTCATCCATTCTGGACTTGGT	GGGAGAAATATTCTGACCACGAAAGAAG
4 PDGFRA	TGGGACTTTGGTAATTCACCAGTTAC	CTGCATCGGGTCCACATAAATATATTCA
4 PHOX2B	CGCGATTACTTTAGGCCCTCAA	CGCCTTAGTGAAGAGCAGTATGTTC
4 PHOX2B	gccgcAGGATTCCAGATCA	GGCCCCATCACCTCCAT
4 PHOX2B	GAGGCTCCAGGACTTCGAATT	GGAGACTCACTACCCCGACA
4 PHOX2B	CAGCTCCTCCCGAGTGTAGA	GTCTCCTCTGTCATACTCTAGTTCCTT
4 PHOX2B	CGTGGTCCGTGAAGAGTTTGT	TGCCGTATGACCTGACCTTG
4 PHOX2B	GCTGAGAAAGCTGAAGGTCCTTA	GACTTCAGTTCCTGCAGCCA
4 PHOX2B	TCGGGTTATACTGGAAGCCACT	GCTGAGCCATCCAGAACCTTT
4 PHOX2B	GTGCTGTCCGGGTCAGT	GCCAGGTGTGGTTCCAGAA
4 PHOX2B	CTCCTGCTTGCGAAACTTGG	AGGGTGTTAAAACAAGCCGAAGTA
4 RHOH	CTTTTCCCTGGGATTCTGGACTT	CCTGTGTTCTCGTACACTGTGG
4 RHOH	CTTCCCGGAGGCCTACAAG	CAGAGTAGCACATCAGCACCA
4 RHOH	TGACGCCTTCAGAAGCATCC	GTACAGGGCAAGTTGCTCCTA
4 RHOH	CTTGAAGAACAAGTGGATTGGTGAAAT	GTAGCCCTTGGCTCTGACATC
4 RHOH	TCAATGCCATGGAAGGGAAGAAA	GAAGAGCCTCCTTCTGTTTCGT
4 RHOH	CGTCCGAACTGCCGTCAA	CACCAAGCTTTCCTGGCTTC
4 TET2	ACGTTTTCTTTGGGACCTGTAGT	AGAGACTTTGTATAAAGGCAGAACGTG
4 TET2	AAAACCTGAGGATGAGCAGCTT	GGCAAGTCTTGACTGGCTCTG
4 TET2	TTCGGCGAAAAGTCAGGATGTTA	GTTTTCAGTTTGTTTTGTACGTGATGG
4 TET2	CTCCCTGGAGAACAGCTCAAATAAAA	CCACACAACACATTTATCTACAAATGCT
4 TET2	AAGGCACCATATATTGTGTTTGGGA	TGCCATAAGAGTGGACAGGTTTTG
4 TET2	AGGAAGAGAAACTGGAGTCTCATTTG	AAGTTGTTACAATTGCTGCCAATGATT
4 TET2	TCTTTGCTTAATGGGTGTCGTATATCA	ctgcATGACTGGTCCTGAAAG
4 TET2	CCTGTCCACAGAACTTTTGCGA	GGTGGATCCAGAAGCAGAATAAGAG
4 TET2	ACCCTCAGACAGAGTCTGTCAA	CAGCTTGAGATGAGGTGGAATAGA
4 TET2	GAAGCACCAGCCCTATGAACT	CCATTGCATTGATATGATGGATATTGGG
4 TET2	CTTACCCTGGGCTTTTGAATCAGAATA	TGGTAGACTGAGCTTAGACAGAGG
4 TET2	CACCACCTTCCCAGAGTCCTA	GTTTTCTCAGAACACAATGGAACAGTC
4 TET2	GAATTCAACTAGAGGGCAGCCTT	CAGAGGTTCTGTCTGGCAAATG
4 TET2	AGTATTCAAAATCAAGCGAGTTCGAGA	CCTGTATTTTGCATGCACTTGATTTCA
4 TET2	CATAGTCAGATGCACAGGCCAAT	TCAATGATGCTCTTTTGCTGCAC
4 TET2	CGTAGAAATTCCCCTTATAGTCAGACCA	GTGATGCAAGTTTTGGGTCTTGTTT
4 TET2	GACTAAGTCCATTCCTGATACCATCAC	GCTTCCCTTCATACAGGGTATTCCATA
4 TET2	GACATACAGACTGCAGGGACAAT	GACCCTTCAGAATCTCTTGCTCTT

4 TET2	GACTACACATCCTGAACTTTTTGCAG	TGACTTCTGCTCCTGTTCTTGAAAG
4 TET2	ATCCCAAAGCAAGATCTTCTTCACA	GCACAGGAAAAACATTTGCATGGTT
4 TET2	AATCCACCTGCAAGCTGTGATAAT	CCCTGACATTTCAACTTTTACTTGCTTC
4 TET2	TTGCTCAGCAAAGGTACTTGATACAT	CTTCTGTAAGAGATGCCACCTTAGAG
4 TET2	CCAAGTGGCACTCTTTCAAAAGTTAT	TGATCTGAAGGAGCCCAGAGA
4 TET2	TGCCAGCAGTTGATGAGAAACA	GCAGTGATGCCTCATTACGTTTTAG
4 TET2	CCATAAGGCTCTTACTCTCAAATCACA	AGCTGTTCTTTTGGTTGGTGTCT
4 TET2	GAAGGACACTCAAAAGCATGCTG	CTTGCATCCAGGTTCCACCTTA
4 TET2	GAGCAGCAAACAACTTCTTCAGAAA	GGCACTTACCTACACATCTGCAAG
4 TET2	CCTGTCAAGACTCAATATGATTTCCCAT	CAAATTGCTGCCAGACTCAAGATTTAA
4 TET2	ACGCACAGTTAGTGAACCTTCTC	TTATCACTCAAATCGGAGACATTTGGT
4 TET2	TTTCACCAAGCGGAATCCCAT	GTGCTCCAGCTGTGTTGTTTTC
4 TET2	CTCCCAAGGCAAGCTTACAC	GTGAGGGTTTTTGGAACTGGAGA
4 TET2	TCTTCTATTATCTCAACAGAGCAAATTATTGAA	GTGCCTTTGCGTTAATTACCTTTCT
4 TET2	CCAAGGTACAGTGGACCAACA	GGACATAAGTTTTTCAGTTTGGGAATCT
4 TET2	GTGGCAGCTATTAGAGAAATCATGGA	GCAACCCTAGAATAGCTAAGCCTACTAA
4 TET2	TGGCACTAGATTTCATTTTCAACAAAGAG	ACTCTGGGATGGTTGTGTTTGTG
4 TET2	CCTTTTGCAACATAAGCCTCATAAACAG	TGATCCTTCTCTTTGCTGATCATTGTT
4 TET2	CATGGCGTTTATCCAGAATTAGCAAAT	CACAAGCATCGGTAACTTGACAAAAA
4 TET2	AGACTTTTCCTCACCCCCAAAG	TCCAGTCCCATTTGGACATTATGAG
4 TET2	CCTATAATATCAGCTGCACAGCCT	TTCTGGCAAACTTACATCCATTGTAGT
4 TET2	TCTCTTTTGGTTGTTCATGGAGCAT	GCGATTATACATCAGGAAGTAAACAAACC
4 TET2	GGTCTAAATACTAGTGAGTTTTCGGTGT	GAATGGACGGCCTTCCTTCA
4 TET2	AGATTGAATATGAACACAGAGCACCA	GCTGTCCTCAGCCCAACTTA
4 TET2	GGTGTTTGGGATGGAATGGTGAT	GTAGAGTTTGTCAGCCAGAGACAG
4 TET2	CATCCTGGTGTGGGAAGGAATC	TTTCCTATCAGTGGCCGCAAA
4 TET2	TTTGCTAATTGTATGTGTGTGTGTTTCTG	ACCAAAGGCTTTATCAAGTCACACT
4 TET2	AAAGTCACTTTTAGAGCCCTTAATGTGT	GGACCTGCTCCTAGATGGGTATAAA
4 TET2	CACAAATTAAGTGATGCTAATGGTCAGG	AATGAGAATTGACCCATGAGTTGGA
4 TET2	GATCTGTATAGGTATCCAAGCCAAGAC	ATTTGGTCTAATGGTACAACTGCTGAA
4 TET2	GCTTTCTGGATCCTGACATTGGG	TGGCTCATTCATGCTCTTATGCT
4 TET2	AACCAAAATATGCAGGGAGATGGT	GGATTGCTCAGATTGGGTGGTAATC

4 TET2	CAGGATCTCCCTCGTCTTTTACC	GGGCCATACTTTTCACACTCTTCC
4 TET2	ACTTCATGGGAGCCACCTCTA	AGCATGTCATTCTCCTTGTTTTGGA
4 TET2	GCTGAAAAAGCCCGTGAGAAAGA	GTCCTTTCGGCAAGAGACTTGAT
4 TET2	GCTCTCTTCATGCCCTGCATC	ACTAGTGCCAATGGCTGCTTTT
4 TET2	AGCCCACTTACCTGCGTTTC	GTCTTTTCAAGTGAGGTAACCAACAAA
4 TET2	AAGAAATCCAGGTGAAAGCAGTCA	TCCCCTCCTGCTCATTCAGAAT
4 TET2	GGCCTGAAAATCCAGAGCTTCA	AGTTCACCATGTGTGTGTTCCA
4 TET2	TGCTACAGTTTCTGCCTCTTCC	GACAACTCATTAGTAGCCTGACTGTT
4 TET2	TGATAATGCCAGTAAACTAGCTGCAA	TGGAACCTGAACAGAATTCTTCACC
4 TET2	GAAAACCACATCTCACATAAATGCCATT	TGGAGGCAGCTCAGAGTTAGA
4 TET2	GGAACCACAAAGCTAGCGTCT	GTGGCAGAAAAGGAATCCTTAGTGA
4 TET2	CAGATCAATTCCGCACAGACC	GGTTTCTGAAAGGAACAGGTATTTAGCA
4 TET2	CTTACTTCAAGCAAAGCTCAGTGT	CACCATTCAGAGTGCTTTTTCCTT
4 TET2	CAGGTTCCTCAGCTTCCTTCAG	GGCTGCATACATGTGTAGATGGAT
4 WHSC1	ATGTACATTTTCCCCATTCCCCAT	GTCAAGCAAACCTGGAACACAATTAC
4 WHSC1	TTGTTCTTTGCACCTCTCTCTCC	GGCTCTCAAATACAGTCAGAGGGTAT
4 WHSC1	AAAATTTTACCATGAGGCTTGTGTGAAA	GCATCTGAACCCACAGCAAG
4 WHSC1	CAGTAGATTTGACTAGTTTCATCCCAGA	CAAAAGCTACGAGGCTCTTCTCAA
4 WHSC1	GCCCCAGAAAGAGCTTGGATAT	GGTCTAAAAGTAGACTGAAGGGTCATCT
4 WHSC1	CAACGAAAAATCTGTCTGATGCATGT	TTCATACCAATGCCATGGTTATCGTTA
4 WHSC1	GGTGTGCAAGGGTATTCGGAA	ACACTCCGAATCAAAGCCACAA
4 WHSC1	ACCTGTGTTCATTCACATGAGG	AGAGCTTGTTCTGGCCGTTT
4 WHSC1	GAGACACAATCACTGACAAAACGG	CCTTCCCCTTTCACACTCTCA
4 WHSC1	GCAAGCCCACAGATGAGAATCC	CACCCTTTGCCATCTGTCTTGA
4 WHSC1	ATTATTATCGCTGTCTCTGAGGAGTCT	GCGTCCAGTAATAATCTTTAGACCCAAA
4 WHSC1	CCAGTACCCAGAGACCAAGATCA	CAGTGAACCGTAATTTCTCAAAAGCA
4 WHSC1	GAGATTGGAGAATTCCCTGTGTTTTTC	GTCTCACTCTCTATCTGAATATCTCCGT
4 WHSC1	atgaatcaTGTGCCTAGGAAAAGTAGTT	CTGACATGCTTGCAGCTTCTTC
4 WHSC1	GGAAATGGGCATTGTTCAAGCA	CTCCTGAGGATTCTGCCATTTCTC
4 WHSC1	GCATTGCTGCAGAGTCTTTGG	TCTGCAGAGCTACACAGTTTGG
4 WHSC1	GAAGAGTGCATTCCCATGAAGAGAA	TGGAGACTGTGGTCTTCTTCCT
4 WHSC1	CCCAGAAGAGGAGTAGGGTCTC	GATGTTCCTCAAATTCTGAAACTTGCTA
4 WHSC1	CTTTTTGTCATGGCCACATGCT	TCACAGTGGAGTTCCGCATTAC
4 WHSC1	TCATCCGCCTCCTTCATCTCTA	GGATGCTGCCTTCTCCTCAC

4 WHSC1	AGAAGGACAAAGGAATTCAGAAGATTCA	CGATTTCGCTTCTTCAGTGGTTT
4 WHSC1	CTGAATCTGGGCTGAGCCATA	CCTGCAGTCATTGCAGAACCA
4 WHSC1	CCTGAACATCGAGATGCCTGAC	AAGTACTGTAAAGCGAACACATGTGA
4 WHSC1	GTGGGAATAAAAATGCGACACACT	ACCTGAGCAGCTCTTCAATCTC
4 WHSC1	CACCCAGATGCTTCAGGTGAG	CCAAGCAGAGGACATCAGCATAAT
4 WHSC1	CCATAGTGTTCTAAGAACGGAAGCAT	CAGAACACTCGCGGTTCAC
4 WHSC1	GAAGACTCCGAGCTGCGA	CACCCGGGAAGTAAGATCTTTCA
4 WHSC1	CCCTTTATTCCAGCCGACAAG	TTTCAGCTTAATTTCTGGAGAGCCA
4 WHSC1	CCCCTAACACTACCCCTATCAAAAA	TGTTCCTTCTCGCCTTGTTTTCT
4 WHSC1	GGAGCTTGAAAGGTAGTTACCTGT	GCTCGCTCTCCTGTGTTTCTC
4 WHSC1	TCGTGAAATTAAGCTTCAGAGGGAAG	CATAGCCTGGGTGTGCCTAA
4 WHSC1	CTCTTTTCACTATGACTGGAGTCAGT	AAGTGGGCAGTGCAGATGAT
4 WHSC1	GGAACACAACTTGTTCAATGTGCTT	GGCTGGCAGCTGTGATTCATA
4 WHSC1	GCTCAGTGATCGCCTCCAA	GATATGTGGCTCCTGGGAGTG
4 WHSC1	CCCCAAAGGAAACTACTCTCGATT	AGGGTCCCCAGAGCTTGTA
4 WHSC1	GGTCTTCTGGATACACAGGTAGTGA	GGGTAACCCGACACTTTGGAC
4 WHSC1	TGGGTCAAGCCAGTACAGATA	CCCAGACAATCGAGGTTGTAGT
4 WHSC1	AAATACAACGTTGGTGATTTGGTGTG	AAGCCTGGAGATGAGAAAGATTAAGTG
4 WHSC1	CAGGGACGGAGCTGACTTTTA	AGTGCAAAATGCCAACACAGAG
4 WHSC1	TGTGTTCATTTGACCTGACAGTTGT	GAGGTGGCAAAATGAAGTCGAAG
4 WHSC1	GAGATCTCTGAAGTTCCTGGAGT	TCTTTTTGCCCTTTTCCTCTGATGA
4 WHSC1	ATTGTGACGTGTGTGGCAAAC	GTCTTGGTGCTTCTGACCGAT
4 WHSC1	GACGGCTTCCCTTGTGGTA	CTTCTGGCCTCCGGGAAAG
4 WHSC1	CTGCGGAGCTTTCCACCT	CAGCTCAGGGCAGGACTT
4 WHSC1	CCTACTGCTGTGAGCATGACTTA	CCCTGGATCCGGCCAAG
4 WHSC1	CTCTAGACCTCGACGACCCTT	GCAGAACTTGCGGTCACACA
4 WHSC1	CAGTCAGAGGACGAGTGCTT	ATCAGGAGCCTGCCACAC
4 WHSC1	CATGACAAAGGCCTGTGAAGGA	CAAATCCAACTTGACTGGTGTGG
4 WHSC1	ACTGGTAACAGCTTTTGTGGGA	ATTGTGATAGAAATGTCACTGAGTGTGT
4 WHSC1	CTCAGTCAGAAGAAAATGGACAAAAACC	ATGACCCCTTACACAACGTCTC
5 APC	GGCTTCAAGTTGTCTTTTTAATGATCCT	GTACCTGCTGTAAGTCTTCACTTTCA
5 APC	CAGTGAGGGACGGGCAATAG	ATTCCATAAGGCACTCAATACGCTT
5 APC	CTGTTTCTTACTAGGAATCAACCCTCAA	CCGGTAAGTAAGAGTGCCAACC
5 APC	CTGTAGATGGTGCACTTGCATTTTT	CCTGTGGTCCTCATTTGTAGCTAT
5 APC	TCATTTGATGAAGAGCATAGACATGCAA	GAAGTTGGTGGCCTTATATCCTAATTCA

5 APC	GACCGAGGTTGGCTCGAT	CCCGAGAACTGAGGGTGGTA
5 APC	CCCCTTTGCCCGCTTCT	GGGAGGAGACGTCAATGCC
5 APC	CATACAGACACTTCATTTGGAGTACCT	AGTGTGTGCTACTAGAACTCAAAACAC
5 APC	CGAATGGACCATGAAACAGCCA	CTTAGCAAAGTAGTCATGGCATTAGTGA
5 APC	GCAGATTCTGCTAATACCCTGCAAATAG	TTTGTGCCTGGCTGATTCTGA
5 APC	CATCTAGACCAGCTTCTCCCACTA	GCTTTGCTGGTCTTCCATCATT
5 APC	GCCTACCAATTATAGTGAACGTTACTCT	TGTTTCTGTGATGAAGGAATATCTGTGG
5 APC	CAGACTGCAGGGTTCTAGTTTATCT	GTGAACTGACAGAAGTACATCTGCTA
5 APC	CGTCATGTGGATCAGCCTATTGATTATA	TGTGGACGTATTCTCACTGCTTG
5 APC	CAAAGCAGTAAAACCGAACATATGTCTT	CCTTTTGAGGCTGACCACTTC
5 APC	CCATCCAAGTTCTGCACAGAGTA	ACATCCTATTTCATCTTCAGCTGATGAC
5 APC	AGTGTCAGTAGTAGTGATGGTTATGGT	TTGCACTATGTATTTTATGGGCTAGGTC
5 APC	CAAGGTCTTCAATGATAAGCTCCCA	GGAAAGGTCAACATCATCATCATCAAAA
5 APC	CACCTAATCTCAGTCCCACTATAGAGTAT	TCGAGGAAGGGATGATGAATGTTTG
5 APC	AGTTTTGCAGTTATGGTCAATACCCA	CTTGCCCATCTTTCATTCTGTGAAG
5 APC	TCAGGAGACCCCACTCATGTT	GCATGGTTTGTCCAGGGCTATC
5 APC	ACCTGGAAACGTGAGCACAG	TGGTTCCTGAAATAGAGTTCACATGTTT
5 APC	AGTTGAACTCTGGAAGGCAAAGTC	TGTGGTTGGAACTTGAGGTGTTT
5 APC	GAACTCCTTACTGTTTTTCACGAAATGA	GCTGATTGTTGGTTGGAGGTTAGT
5 APC	GCTAAAGTTACCAGCCACACAGA	GGGAAAAGTGGATTGTTTCTGAAGTATG
5 APC	CAGTGAAAAAGCAAAAAGTGAGGATGAA	AGCACCATTTGTAGCACCTGAG
5 APC	TCCTGTTTATACTGAGAGCACTGATGAT	GACACAAAGACTGGCTTACATTTTGATT
5 APC	CCAATAAATCGAGGTCAGCCTAAACC	GCAAACCGGAGTATTTTCAATAGCAAAA
5 APC	CCACAAATAGTACTTCTCAGACCGTTTC	GGATTGTTAATGGGACAGTCCTCA
5 APC	AAATCGAGTGGGTTCTAATCATGGAATT	TCTCTTCTTCTTCATGCTGTTCTTCTTC
5 APC	GCAGCAACTGATGAAAAGTTACAGAA	GGTTTACTTGGTTCTCCCTGTGA
5 APC	CATCACTTAATTGGTTTTTGGCTTTTGG	CAAAGTTCGCGACATATCATCCTT
5 APC	CACCTACTGCTGAAAAGAGAGAGA	AATGGCTCATCGAGGCTCAG
5 APC	TGTTGTCAATGCTTGGTACTCATGAT	GGGAATTTCCCAACAATACAGAGTCTT
5 APC	CCAGCTTTTACATGGCAATGACA	AGCGCGTATCTGTTCCAAAAGAT
5 APC	GCGTGAAATCCGAGTCCTTC	TTGAAACATGCACTACGATGTACACTAT
5 APC	GAGCTTTTAAGTGGTAGCCATAGTATGat	GCTTCTGTTGCTTGGGACTGT

5 APC	GCACCATGACTGACGTATTTGCT	GCCTTGCTTCATATTCCAATTGCC
5 APC	CTTACAAACAGATATGACCAGAAGGCA	GCTCTTCGCTGTTTTATCACTTAGAAAC
5 APC	CCTTGGGCTAAGAAAGCCTACA	CTGACCATTACCAGAAGTTGCCA
5 APC	GTCAAGGAGTGGGAGAAATCAACA	CTTCTGGAAATATGCATTCAGGACTAAGA
5 APC	CGTGCTTTGAGAGTGATCTGAATT	GTCCAGAAGAAGCCATAGCTTCA
5 APC	ACAAGGAAGTATTGAAGATGAAGCTATGG	TCGAGAGAAGCTGTACTTGGATCT
5 APC	AGCACTTTAGGTAGAGAAGTTTGCAAT	AGCGTAATACCAGTCTTTTTCCTTTTCT
5 APC	ATTGCTTCTTGCTGATCTTGACAAAG	TGTACTGCCAAGTTACTTACATTTTCAGT
5 APC	GATTAATTTGCAGGTTATTGCGAGTGTT	AGGTACCTTTTTAACTTCTAAAGCACAT
5 APC	GGAAGTGTGAAAGCATTGATGGAAT	ACTGAATAATACACAGGTAAGAAATTAGGAAA
5 APC	CCTTTCTTTAAAAACAAGCAGCCACTA	TCTAGCTCTTGTCGAAGATTTGAGTTC
5 APC	CAAGTTGAGGCACTGAAGATGGA	CCTCAAGTTTACAAGAGGGAATACTGAA
5 APC	GATTGTCTTTTTCCTCTTGCCCTTTT	CCATTCCAGCATATCGTCTTAGTGTAAT
5 APC	CAGAATTATTGCAAGTGGACTGTGAAAT	AACATACCTTGTTGGCTACATCTCC
5 APC	TTGTTACTGCATACACATTGTGACCTTA	GATTTCTTGCTGAGAGATTCCACAAAG
5 APC	TTGACAATAGTCAGTAATGCATGTGGAA	CCCATAGCAATCATTTTGTGCTTTGAA
5 APC	GCAACATGACTGTCCTTTCACCATAT	CTGGATTTTCTGTTGCTGGATGG
5 APC	AAGCTTCGTCTGATTCAGATTCCAT	CCAATGTACTTTTCTCCCCTGGTTTT
5 APC	ACAAGTAATAAAGGCCCACGAATTCT	GGCTGTTTCATTTGGCCTGA
5 APC	GTGGGTCAAGTAAAGCACCTTCTA	TGATGTCCTTGGAAGTTGAGATAATTTGT
5 APC	GCCAAATGAAACAGCCCCTT	GGTGGCTGTTTGACCTTCACTA
5 APC	AAGAGACTGAGCCCCCTGA	CAACAGGTCATCTTCAGAGTCAATACT
5 APC	GGCCATCTCAGATCCCAACT	TCCTCTCTTTTAAACAGATGTCACAAGG
5 APC	AGCATGCTCAAGAACCTCATTCA	GCTTCTAGGGCTTTTTGTTTCCTAACAT
5 APC	CGCCATTCTGGGTCTTACCT	CCAGAACAAAAACCCTCTAACAAGAATC
5 APC	GACACCTATAAACTTTTCCACAGCTACA	CTCCTTGAGCCTCATCTGTACTTC
5 APC	GCGGAATTGGTCTAGGCAACTA	CCCAGAACTTCTGTCTTCCTGAG
5 APC	ctCAAGAAACAGTTCTCTCAGTTCTCTT	CCACCCATATTTCTGGGACTATGTT
5 APC	AAAACAGAGGATGTTTGGGTGAGAAT	CCCACATTTTGTTTTGCCTGATTATCTT
5 APC	TTTTCTTGTTCATCCAGCCTGAGT	TTCTGCCTCTTTCTCTTGGTTTTCA
5 APC	CCTGGTAGAAATGGAATAAGTCCTCCTA	TGGATAAACCTGTTTGTTTGGTAAGGT
5 APC	CCTTCAAGACTCAAGGGTGATAATGAAA	ACCTTCCTGAATAGCTTTCCAATCAAAA

5 APC	CCTTAAGACTCCAGCCTCCAAAAG	GGGTCGAATCTCTAGATCCTGAT
5 APC	CATGGTCTATCCCCTGATTCAGAAAA	GAGAGATTCCTGATTTCAGGGAAAGG
5 APC	GCTCAAGCTTGCCATCTCTTC	GCTTGTGTCTCTGCTTACTACGAT
5 APC	GAGATACCATTCCTACAGAAGGCA	ACACGGAAAGGCTTGTGACTTT
5 APC	CAGTGTTTCAGAAAAGGCAAATCCAA	GTTTTATCTCAGTTCCTTTTTGGTCAGG
5 APC	AGGTAGACAGATGAGCCAACAGA	TTCTACCTTTTTATTGGCTCCATTACCA
5 APC	GAAGAAGTGTCAGCCATTCATACCT	GAACATGTCCTATTTGAATTTTCCGACTT
5 APC	AGAATCAGAGCAGCCTAAAGAATCAAA	TTTGCTTTACGTGATGACTTTGTTGG
5 APC	AGAAGAATGTATTATTTCTGCCATGCCA	ACCTGTTTTGTGATGGTAGAAGTTTGT
5 APC	AGACAATTTAAGTCCCAAGGCATCTC	GAGCTGGGTAACACTGTAGTATTCAA
5 APC	ATTAATTCTGCTATGCCCAAAGGGA	TGTGGTATAGGTTTTACTGGTGAAGTTG
5 APC	AGAAACCAACTTCACCAGTAAAACCT	GGAGCTTATCATTGAAGACCTTGGA
5 APC	GCCCATACACATTCAAACACTTACAATT	AATCGAGGGTTTCATTGACCTCTT
5 APC	AACTCCTTTATTCAGGTGGATGCC	GTCCCACTAGGTGAACTGTGTT
5 APC	GCCTCCAAAGGACTAAATCAGATGAATAA	GGCTTGGAGCTTCTTTGATGAAA
5 APC	GACCTGTATTAGTACGCCAGTCAAC	GGACTTAAAACTGGAGTTTGTGCCT
5 APC	AAACCAAGTCAGCTGCCTGT	GGGATTCGATTGTTAGATCACTTAGAGA
5 APC	ATTCAGTTCTAGCAGCTCAAGCA	CGCTTCTTTGTGTTGTTATTCACTGG
5 APC	CTGGAGTAAAACTGCGGTCAAAA	TCCATTTACAAACCCTCTTCTTGGAA
5 APC	CAGTCCTGTTCCTATGGGTTCAT	AACTGGAGTACACAAGGCAATGTTTA
5 APC	AGAAGATACTCCAATATGTTTTTCAAGATGTA	AGCTGACCTAGTTCCAATCTTTTCTTTT
5 APC	GCATTATAAGCCCCAGTGATCTTCCA	TGCAGCTTGCTTAGGTCCAC
5 APC	ATTTTAGACTGCTTAAAGCAATTGTTGTATAA	CTTCCATAAGAACGGAGGGACAT
5 APC	GGCTTTGACAAACTTGACTTTTGGA	GTGAAGCACAGGTTTTTATCAGTCATTG
5 APC	CCAGATAGCCCTGGACAAACC	CAGCATTTACTGCAGCTTGCTTAG
5 CSF1R	CGATTGTTAATTTTAGGGCCCATTGT	ACCAACAACGCTACCTTCCA
5 CSF1R	GCGATAGGTCCCCGTGTTT	CGGTGACCTTGCGATGTGT
5 CSF1R	CCATTCCACGCTGCCATTG	CGCATCCTAGACCTCACTTCTG
5 CSF1R	TGAAATGTGTGTGATGCCTCTTGT	GAGGATGCTGTCCTGAAGGTG
5 CSF1R	CTCACTCACACTTCAGCATCTTCA	CCAGGGACTTAAGGGACCTGT
5 CSF1R	cTCCCTCTCACCTGCAGAGA	ACAAGGTGACGGTGCAGAG
5 CSF1R	CTCTAAGGTCTCAACAGTCAGCAG	CCTGAAGCTCTGAGCCACATT
5 CSF1R	ACCAAACAGCTTTGTCCACCA	CCCAGAGAGCATCTTTGACTGT

5 CSF1R	GTCGCTCTGAACCGTGTAGAC	TGTCAAGGGCAATGTAAGTGCT
5 CSF1R	GAACCGTGTAGACACAGTCAAAGA	ATGACTCCAACTACATTGTCAAGGG
5 CSF1R	GCTCTCTGTCCCCACTCTTCA	CCATCCACAGGGCCAAGTTC
5 CSF1R	GCATTGATAGTCCTGGCTCTGAAT	CTACTGCCCTGTCTGCTCAC
5 CSF1R	CTTCCAGCACCGGGTCT	GTAACCCTGTGCCCTCCTCTA
5 CSF1R	GCCCTCCCAGCACTTACATTG	AAGACTAACCCTGCAGTGCTTT
5 CSF1R	TGTGATAGGAAGCTGTGGAGTGA	CTGGAAGATCATCGAGAGCTATGAG
5 CSF1R	GGTCGATGAAAGTATAACTGTTGCC	GTCAGAAACAAGGTCCAGGAACT
5 CSF1R	CCAAGCCTCACCCCACTC	TGGGCATCCTCTGTCCTATCTC
5 CSF1R	CACAGACCTGGGTGGCTA	TGCCCTGACGCCTCTTC
5 CSF1R	TCAAAACCCTTCTGCTCACACT	TGACCCTCAACCTCGATCAAGTA
5 CSF1R	TTGCCGGCATGTTGGAAATC	ACCTTGGTATAACATCTCCATTCCTTC T
5 CSF1R	CCCTGGGATCCCTTCGCTTA	CCTCTTACCTGATCTTGGCCTTTG
5 CSF1R	CCCCTTCTCCTTTTCCCTTGTT	CCCCAACCCTCTCTCCTATGTA
5 CSF1R	ACCTGGCCTTTTTCTTGTCCTTT	GCTATGGCGACCTGCTCAAC
5 CSF1R	TCAGCCTTCCTTCGCAGAAA	gtctCATGCTCCTGTTCATGGA
5 CSF1R	GTCTTCTCCATCACACCCCAA	AGCTAGTAGCTGAGAGCTCTGT
5 CSF1R	CCTTCAGCTAAGACAGCCAGT	ATGTTAACTTTGATGTCTTCCTCCAAC A
5 CSF1R	GGACTGACCTTGGTGTTGTTG	TCTCTCCCCCGGTCTCAG
5 CSF1R	CTTGCTGAAGCATACCCCATCT	GCCTGCAAGGTTTTAACTGGAC
5 CSF1R	GTCAGAAAAGGGTCCCAGGTAG	CAGATGCTTGTGTGTTCTGCTTT
5 CSF1R	CCATCCATGGAGGAGTTGAAGTT	CTCTAGTGAGCACCTGACCTG
5 CSF1R	GCTGGGCGATATCCCCTTG	CTGATGGATCTGGACTGACAGTT
5 CSF1R	CCCTGACACTTGGAGCAGTA	GAACATCGTCAACCTTCTGGGA
5 CSF1R	GCCCTTACCTCCATGGGTACA	TGTCTTTGGGACTGTGGTTCTTC
5 CSF1R	CCAGGTGAGGGAGGTGAGT	GCCTCCTCCTTTCTTCCTCAG
5 CSF1R	CCACCTCCCAATCTCCCAGTA	CTGGAGAGCTCTGACGTTTGA
5 CSF1R	CAGCACTCACATCGAAGGGT	CTCACCAACACCACCTGATTCT
5 CSF1R	ctgaTGGACTGACTGAGGAGGA	ATGTCCATCATGGCCTtgct
5 CSF1R	CTCACCTGCTTATACTTGTACAATagca	CTGCTCACTGCTTTGCTTCAT
5 CTNNA1	CCCTTGTCATCTGTTCCAGAATG	TCGAACAAGCTTTACACCTTCTTCATT A
5 CTNNA1	CCAACTTGGCCTGTTCCATCT	TTTGGTTATGCTGTCACCAGTGT
5 CTNNA1	TGAAAATTCCTAGGATGCCATCTTCTT T	CTTATCCACTTCAGGTTTTAGGGCTTT A
5 CTNNA1	GCAATGAACAAGACTTAGGAATCCAG TA	GGTACTTTCAGAAAACACACAGAATG AGT
5 CTNNA1	CACAAATGGCTATAGGCTATCATTAG GT	GCACTTTCATCCAGATTACCTGTCT
5 CTNNA1	AGACCAGGGACTTGCGTAGA	TGTGAAAATCCATCCTTTCTCTCAATA A
5 CTNNA1	TTCTCACCTTGAATAAAGAAGGGAAC AG	CATCAAGGCAGCCCCACTAA
5 CTNNA1	GGAGCGTCTGGAAAGCATCA	CTTACTTCAAAGAAAAGCTGTCAAAC TCA
5 CTNNA1	AGAAAGCCTCCTTCCTCATTCAAC	CTCTGAGGTGACTACGTGAATGAC

5 CTNNA1	TTCCTGATGCAAAAGTCCCAAAGTA	ATCTCTAGACTTTTAGGATCCCACTTGAA
5 CTNNA1	CTGTCCATGCAGGCAACATAAAC	GCCTGTGCTATAGAACCATGTTACAAT
5 CTNNA1	CGCACAGCTGGTGCAATTC	GAGCACTGGAAAGGGAGAGTTC
5 CTNNA1	ATGTTCAGTGGAATCTCATGTGATTT	CTCTCTTCTTATTAGAGGGCCCTTTAC
5 CTNNA1	ACACCAATAGTAAAGGGCCCTCT	CCACAAGCTCCTCCTTGAGAAAC
5 CTNNA1	ACAAGCAACTGAGAATTTCTTGGAGAA	AGGCCTGCCTCTCTTTACCTA
5 CTNNA1	AGTTAGGATAGAGCATGTGGTGTGA	TTGGGCCAGTTTACTCTGTGG
5 CTNNA1	GCACTGGCTTTAGCAGCAAAA	CCAAGAAGTCATCAATGGAAGTAATGTC
5 CTNNA1	GTGTTCTCACAGATGCTGTCGAT	CAGTGTTTGCCCATTAATACAACTTAGA
5 CTNNA1	AGCTCTTTGTGCTTCTGATCACA	TGATGTCATTGCCACTGTCGT
5 CTNNA1	GCTGGATGCTGAAGTGTCCAAAT	GGGAAGAAAGTAGCCCCTGT
5 CTNNA1	AGAGATGTGTCTGACCTGTGATCT	CCACGGAGCGCTTCCTTAC
5 CTNNA1	GGGCTTCACATACAATAATCCTTGTT	ACCTGGCAAGTCTGTCACTTAC
5 CTNNA1	CCTGCTGACCAGGGTATCTACT	AGGCGGGAAGCATCGATAAAC
5 CTNNA1	CCAGCCCATGGATGAGAATGA	GAAAGAAGCTGTTAAGACGTCTGAAATG
5 CTNNA1	GGTGTTAAGCCTGCTCTCTCTTC	CTAATGCTTACCCCAGAGACAACAA
5 CTNNA1	GAACATCTGCAGCAAGGTCAAG	GCAGGTGTTTATGCGGATAGGA
5 CTNNA1	CTACCGTAAGCTTCATTAGATTTAAGTGT	AAAGAATCTGAAACGTGGTCCATGA
5 CTNNA1	AATTTAATCATTAGCTCCGCAAAGCTG	CTTGGGCATACTCCTTAACTTCTTTCT
5 CTNNA1	TGATTGAAGCTGCAAAGAATGGAAATG	CAAGTTGCCTACAAGTCAATGAAACTG
5 CTNNA1	GCCCATATAAAGAGTGCTCCAATTTCT	CCTTATAGGCTGCGACATCAGG
5 CTNNA1	AGAAGAACGTTCCGATCCTCTATACT	CCTGGGCTGCATTGGAAATG
5 CTNNA1	CAACAGGGACCTGATATACAAGCA	GAAATTTCCACCCAAGCATAACTTAC
5 CTNNA1	GTGCTTCTTACCACCCCTGTC	TGGTATTTGGTAGAGGCGACGTA
5 CTNNA1	CAAGAACTTGATGAATGCTGTGGT	TCTCTTCACCAATGGCTTTTTCTCT
5 CTNNA1	CCTGCTGTGTCATGGAAGATGAA	GGCAGACTTAGATGCTGTCCATA
5 CTNNA1	GCCCTCAGCGAGTTCAAAG	ACTGGTTCAGTCTGCCCATTT
5 CTNNA1	TTTGAGTTCATTCTTGCTGAGTTTGTTTT	CAAAATCAGCAACCGGGTAACAG
5 CTNNA1	CGAGGCAACATGGTTCGG	ACTAAGGAAGGCCAGTAGCAATCT
5 FGFR4	GTTGGTGGGAAGTCCAGCTT	TCTCCCACCTCAGAAGCCATA
5 FGFR4	AGCTGGGAGGGACTGAGTTAG	CCCGACGCGTACAGGATG
5 FGFR4	CGAGGCCAGGTATACGGACAT	GTACCTGTCGGGCCAGAG
5 FGFR4	GCCACTGTGCAGAAGCTCTC	GTACCAGGGATGAGCTTGACTT
5 FGFR4	CCATTCCCCAACAGCTGTG	CACCGGGATGCCAGGATA
5 FGFR4	GGATCCTGCTATGGGAGATCTTCA	GGAGGAGGACTGGAAAGTGG

5 FGFR4	TGCTGACCTCTGCCCTCT	GACTGCCCTCCTTGTACCA
5 FGFR4	GCCTGTGCGTCTGTGCT	GGCCAGCATCCTCAGGTA
5 FGFR4	GCCTAGAGATTGCCAGCTTCC	TCACTCCCTGCTAGAGTCTCTTAC
5 FGFR4	CCTCATCAAACTCCCCACCAA	TTGAAGGTAGGCCTCTGGGA
5 FGFR4	GGCTGATGCGTGAGTGCT	CGCGGTCAGGCCAGATG
5 FGFR4	GGAGGTCTGAGGCTGGACTT	gggccACACTCACCTTTG
5 FGFR4	CGTTAGGGTGCAGAGCCAAA	CCTCACTGCTCCGAGGAC
5 FGFR4	GACCTGTGGGACTCTGCAC	GCAGTGCCTTGCCCTTTTT
5 FGFR4	GAAACCTGCGGGAGTTCCT	CTTCCGGGACTCCAGATACTG
5 FGFR4	TCTCCTTCCCAGTCCTGGTC	AGAGGGAGAAGTCGGGACATT
5 FGFR4	CCTGCCCCTCTTGGACCTTA	CAAGTCCTTGGAGACCTACTGAC
5 FGFR4	CCTAAAGGTAAAAGGTGCACCCT	GGACCTCCACCTCTGAGCTATT
5 FGFR4	CACCCCAGACTGCAGACATC	GCATCTCCTGGCCCTTCA
5 FGFR4	GTGGTGTGTGCTCAACTCCA	GCAATCTTCATCACATTGTCCTCAGT
5 FGFR4	AGTGTATCCACCGGGACCT	CATCCAGTTCTGCCCCATCTC
5 FGFR4	TGCAGTTCTCCCTGGAGTCA	AACTCCCATAGTGGGTCGAGA
5 FGFR4	GGCCTCGTGAGTCTAGATCTACC	TGGAGAAAGTCCAGCCTCAGA
5 FGFR4	GTGGTCAGGGTTGACTGTCT	TGTGCTTCAGCCACTGGATG
5 FGFR4	GTGCAAGGTGTACAGCGATG	AACCCAAGGTGGGAGAAGAATG
5 FGFR4	GGCTGACCAGCTCCGTT	GGAAGGAGCTGGATCCCAAT
5 FGFR4	CGATTCTGTCTTCAGCCACGA	GCTATCAAGGTCGAGCACTGT
5 FGFR4	TGTGGGCAGGATGAGGATCTA	TCTCTACCAGGCAGGTGTATGT
5 FGFR4	CACTGGAGTCTCGTGATGGA	AACTGAGCCCAAAATGGGTCA
5 FGFR4	CCGTCTGCTGCCCTTACA	CCTAACGGCCCGTGCAG
5 FGFR4	GTGGGAACACACGGTCATTCT	CCATCCTTAAGCCAGCGGAT
5 FGFR4	CGTCAAGTTCCGCTGTCCA	GGTCTTGGAACCCAGAGACTCA
5 FLT4	GCCTCCCTGTAGAATCTCGGA	CACCGTGGCCGTGAAAATG
5 FLT4	GCTGACCCCACACCTTTCAG	CACGCTCACCCCGTACAG
5 FLT4	AGTGACCTCGCCTCCTCTC	GCTGCCCCTGAAGTGGAT
5 FLT4	CATGAAAGCCCCCGCTGA	CAGCGACAGGGTCCTCTT
5 FLT4	GCCCTCGGTCTTCGAGAA	CCTCGAGCCAGCTTCGT
5 FLT4	GGCACAAGGACCCTGGTTT	GAACTCTCCAGCATCCTGACC
5 FLT4	GCTGGCTGACGTTGTGGAT	GGAGGTGTGCCTTCGGT
5 FLT4	CCTGAGGCTGGAGCTGTACT	CCTCCTCCCCCAGCATC
5 FLT4	CGGCTGTGACGCGAGTA	TGGTGAATGGTAGGTCAGGGA
5 FLT4	CCCAGAAACTCAATGAGCCCAA	AACGTGTCTGCCATGTACAAGT
5 FLT4	CCACCTTGTTGGAGACCACAC	GCGACCTCCTTGCAGAAAGAC
5 FLT4	CTGGATGCTCAGCTTCTGGTT	GTACAAAGACGAGAGGCTGCT
5 FLT4	CCCTCCCTACCAGACTTTTCCT	CGCAGAACTTGACCGACCTC
5 FLT4	AGTCGCTCACGTTCACCAG	CACAACCCCTAAGTCCAGCTT
5 FLT4	ACGCTTGCTGTCCCCAAAA	ACCTCCCCTTCCTGTTGTCA
5 FLT4	CCACGTTCCCTCTCCTCAATG	GGAACATTCTGCTGTCGGAAAG
5 FLT4	CCAAAGTCACAGATCTTCACCAC	GGAGTTTCGGGTGTGGGTATT
5 FLT4	CCCGCCTCCAAGTCTCA	CAGGCCCTACTGCAAGGT
5 FLT4	ACCTCGTGCAGCAGCAA	CCACCTGACCACTCCTGTC
5 FLT4	CCGTCTTCCCCTATGGTCTG	CAGGAGACGAGCTGGTGAAG
5 FLT4	GGGCTGTTACCACTGGAACTC	GACGGGTCAGTCCTGGATTTA
5 FLT4	GATGCACCCTTTTCCCGTCT	CACCTGGCTCCACTGTGTA

5	FLT4	CCGATGACGCAGAGGGATAA	AGCTTCTCGCAGGTGTCC
5	FLT4	CGATGTGTAGGGCCATGGT	AGTGGCTGGTGGTTTCTGG
5	FLT4	CATGGCAGTAATGGCCTCTCT	GGATGCCGTGAACCCCATC
5	FLT4	GTACCTTATTCTTTCCCTCCACAAACTC	TGTATCCCTGACCTGCCCTT
5	FLT4	TCCTGCGGATGCACGAA	GGAGTTCTGCAAGTACGGCAA
5	FLT4	GGGCCAAAGGCCATAGTAGA	GCAGCCCACGTGATCCT
5	FLT4	TGTGTGCTGTACAGTCACAGG	CACCGTGTGGGCTGAGTTTA
5	FLT4	CCAGTCAAAGGTGACACCTGAG	TCACTGTGTGCGTGTCAACA
5	FLT4	AGGCAGCTACTGCTAGAAGAGA	CCACGCTCAGCCTGAGTAT
5	FLT4	GCACTTCGCACACATAGTGG	CGCTTCTGCTCGACTGCAA
5	FLT4	GGCGAACAGATGCACGTTC	AAGCCATCCGAGGAGCTACTA
5	FLT4	CGTACTTGTAGCTGTCGGCTT	AGTGCCACAGGGCTTCTTC
5	FLT4	GCGGGCACCTTATTCTTTATCTTAGG	CATCGCTGTCTTCTTCTGGGT
5	FLT4	ATGTTACAGAAGATGAGGAGGAGGA	GCAGGTGCACGCTCTTACA
5	FLT4	GGGAGGGCAACATCGATACC	CCCACAGCCTGGCTTGTC
5	FLT4	GGCTTGTGCAGCCTCTCT	CACCACGCTGACCCTGT
5	FLT4	GAGGTGGTTGCCGATGTGA	GTAACCCTCCCCCTCCCAA
5	FLT4	CCCCACCCTTCATGTGAAGTA	CACCTTCTGATTTCTCCCCACAG
5	FLT4	TGTTGACACGCACACAGTGA	GCAGTGCGAGACCACCTG
5	FLT4	GGTTGGAAAGGAAGTCCTGGT	GCAATGATGGTGGCCTTGTC
5	FLT4	CTTCTGGTGGCCACGACTT	GAGGATGCGGGACGCTATC
5	FLT4	CGCTGGCGGAGGAGTTG	ACGATCCCAAACCCAGGTG
5	FLT4	GTGCAAGTTTTGAAAATGGAGGGATT	AGGAGCAATGCGAATACCTGTC
5	FLT4	CCACTGGCTGGCATCGTAG	ACAGTGGTATGGCCAAATCAGT
5	FLT4	GAAGTCTGCAGAGAGGGAAGAG	CGTGACTTTCTTCACAGACAACAG
5	FLT4	TCTTGTCCGATGCTGCTTAGTAG	GAGGAGGCCAGGTGTTTTACA
5	FLT4	AGCTCCCCATACTCGCTGT	CTAACACCACCTTCCCTGTCTTG
5	FLT4	TGGGAGGGTCGGATGCT	ACAGCTTGCTCCCTCTCCATA
5	FLT4	CTCTCCCGTCCCTGACCTA	ACAAGGATGGAAAGGCACTGTC
5	FLT4	CTCTGTCACCTCCTTGAGCA	CCTCCCTCCTCCCAACTGTATT
5	GDNF	TTGGTCCCTTTCTTTGCACTGTA	GACCCATCGCCTTTGATGATGA
5	GDNF	ACCAGGTTATCATCTAAAAACGACAGG	CTGAGACAACGTACGACAAAATATTGAA
5	GDNF	CACCAGCCTTCTATTTCTGGATAAGTTT	GGGTTGTGTCTTAACTGCAATACATT
5	GDNF	CCAGACCCAAGTCAGTGACATTTA	CCAGATAAACAAATGGCAGTGCTT
5	GDNF	GTAGGCCACACAGCCATCA	CCTTCGCGCTGAGCAGT
5	GDNF	ggAGGGAACGGTTCTTACAGT	GAAGTTATGGGATGTCGTGGCT
5	GDNF	CCTCGGGAGGCCTCTTAC	CCCAGGCTTAACGTGCATTC
5	GDNF	CCGATTCCGCTCTCTTCTAGG	TGTCTCATGTGCCATTTTCTCTTTTCT
5	GDNF	CTCCTCCCACCTCTTAAAGTTATGA	CACGGAGGATTAAAACTTTCAAGACAA
5	IL6ST	ATGACTGAAACTTTTTAAGATTAGAGGGTTGA	GGACCAACCCAAGTATTAAGAGTGTTAT
5	IL6ST	AGGCATCTTTGGTCCTATATTGAATGTT	AAGCCCAATCCGCCACATA
5	IL6ST	GCTGAAGTTGTAGCAGGAACTACTA	CAAGTAGAAAGATTTGAAACAGTTGGCA

5	IL6ST	CAGTCTGTGGTAAGTAACTTTTAGGCAT	GTGGATCTGGGCAAATGAAAATGTT
5	IL6ST	CCAAAAGCATCTGCTGCAGAAAC	GCATGAATCCAGTCCAGATATTTCACAT
5	IL6ST	CTCATTGACTGATGAAACTTGCTTTGA	CATGTAGATGGCGGTGATGGTA
5	IL6ST	AGTACTGTTGCCTGGGCAAA	TCCAGTATTCTACCGTGGTACACA
5	IL6ST	ACTTGGACTGACGGAACTTGG	AAGGACACAGCAGTGGTATTGG
5	IL6ST	GAACCTTACCTGTAGATTCAGTGGTG	ACAGCATGGAAACAAGATTGTGTCTTA
5	IL6ST	TGCAGTTCAATAGTCATGACAAAGTCT	AGAACAGCATCCAGTGTCACC
5	IL6ST	GAGTTGTACAGTTCTGTAGCCTTGA	GGAGGGCAAGAGTATTAAGAACAAAGTA
5	IL6ST	CCCTGTCCATACCTAACCACCAT	CAAATGATCGCTATCTAGCAACCCTA
5	IL6ST	CTGATTTGCCAACAAGATTTCTTACTGT	GACATTGCCTCCTTTTGAAGCC
5	IL6ST	TTCAAAGAAGTGAGTTTGGACTTACGA	AGGGAACTTAGCAGAGAGCAAATG
5	IL6ST	ACGAGCTTGTTTAAGGTATGCCTTTAT	TGCTTAATCTGCTATTCTTGCTTTAGGG
5	IL6ST	ACGACTACAGTGTCAAATAAACTCTCAAA	CCCACCTCATGCACTGTTG
5	IL6ST	CAGTAGAATAATCAACAGTGCATGAGGT	CTAGGTCTCATCAAACTCTTTGCAAGTA
5	IL6ST	AATCCATTCTGAACATCAACAGGAAGT	TCAGTTAGCAAATGTGTTCACTCTGTTA
5	IL6ST	CTTACAGGTCTCGCTTATTAAAGCAGAA	TGTCAACATTATTTCCCATCACACCT
5	IL6ST	AAAATCATCATGAATCCAAAGATGTACTTTTT	CACAGATGAAGGTGGGAAGGAT
5	IL6ST	GGGTAGTAAAAGTGAATTCTGGACCAT	GCATTGAATAGTTGAATGAAGCACAGT
5	IL6ST	CAGAAGCATTTTCTCTTACCCTTGGA	GTGTGGTTTTACATAGGCCATTTTTCTT
5	IL6ST	AAATTGGAAGTCGTTTCAACGGA	CCTGTATCACAGACTGGCAACAAG
5	IL6ST	GCGATGCACGGTACCATCTT	ACCCTGTAATGGATCTTAAAGCATTCC
5	IL6ST	CATTCCACCCAAAGCATGTTATCTT	CACATGTTTTGGAAATGACTGTAAAATATGGA
5	IL6ST	ATCACAAACTAAGAAGCAATGAGGCTAT	AGGAAGATGGTAAGGGATACTGGAG
5	IL6ST	CACTTGCTTCTTCACTCCAGTCA	AGGTTTTCTGTTGTTTGGTTTGCTT
5	IL6ST	GGCCTAGAAGATGACATGCATGA	ATGGCAATTTCACTGATGTAAGTGTTG
5	IL6ST	GGCTTTTTGTCATTTGCTTCTATTTCCA	CTCTGTGGCTTTAACTAATACTTGCC
5	IL6ST	AAGTGAAGTTTGTCTCCAAGTGTGT	CTAATTAATCCCTTTTAGTGCCTCCAGA
5	IL6ST	CGTTCACAATGCAACTCAAATTTTTAGG	TTTAGATCGCAAATGCCTTGGGTA
5	IL7R	TCCACTGCATACAGGAACTCCTA	ACCTTCACACATATATTGCTCTTTCCAA
5	IL7R	CGTGGAGGTAAAGTGCCTGAAT	TTTTGCAGGTTAGACTCTTTTCTCCAA
5	IL7R	TGGAAAGAGCAATATATGTGTGAAGGTT	TTTTCAGGTAGCCCAAAAGTGACA
5	IL7R	CTTGGCTGCCCTTTAGACAGA	GGTAAGCTACATCGTGCATTAAAACTTT

5 IL7R	ACATCACACTTGCAAAAGAAGTATGTA	CACTACTAGGCCAACAAAATAGAAAACA
5 IL7R	CATTACTATTTCATGTCTGCCACAGAGT	CTGGCTATAGCATGAGAATGAGTAGTC
5 IL7R	GACTTGGAAGATGCAGAACTGGAT	ACCATTAAAACCACCATCCCTCAC
5 IL7R	GCTACTGAATGCTCACCACAATCT	CCACACAATCACCCTCTTTATTAGTTGA
5 IL7R	CCTCTGCCATTCACTTCATCTATCAA	TTGCCAACACAACACTTTTTAAGCA
5 IL7R	ACTTCCCTTGTCTGTGGTTAGATAAGT	AACCATGCCAAAAGTTGTACCTAGAA
5 IL7R	CAATTCTAGGTACAACTTTTGGCATGG	TGACTCAGGTCTGTTAGGGAACT
5 IL7R	GCAACACCTCTTTTCCCATCCT	GATCAGGGATGGATCGAACTTTAATCTC
5 IL7R	TCCAACCGGCAGCAATGTAT	GAACAAAAACTCTACCACCATTCCTTAC
5 IL7R	CCCATTCTTACTTCCCTGGGATCA	GAGACTAGGAACTCTAGACTTCCCTTTT
5 IL7R	GTGTCTCTCTGGTGCCATCTTAA	ATTCTTCTAGTTGCTGAGGAAACGTATC
5 IL7R	AGATGAAGTGGAAGGTTTTCTGCAA	AGGCATGTGAGGGATGAATCTC
5 IL7R	TCATCACTCCAGAAAGCTTTGGAA	CCAAGGCTAAGCAGGAGGTC
5 IL7R	AATGGGCCTCATGTGTACCAG	ACATGGTGACATATGCTTCTTCTTGATT
5 LIFR	CCCCCACTCCAGAAGAATTAAGG	CTGCAAGTAGTGGAACAAATGTAGTTTT
5 LIFR	AACGGTTCCAAATATGTTATCTTCGGT	CAAACTGCCCCTTGATCCATCT
5 LIFR	TGTTCCACTACTTGCAGAAACAGAAAT	TGTAGGCTCAGACATAACATTTTGTTGT
5 LIFR	TTTGTATGGCCAATCAGTGCTGATA	CTTTCTAGGGATACCTGATTCTCAGACT
5 LIFR	GAAGAAAACAGCAAGAGTAAATGCAGAA	GTGGGATTAATTATTGCCATTCTCATCC
5 LIFR	GATACTTGTCACCACTCCAACAATGA	TCTGTACTTCACACTAAAAATGGCGATT
5 LIFR	ACACTTTCCTTGTGCTATCAATTTACTCA	GTTGAGGATACTTCTGCAGATTCGATAT
5 LIFR	CCACAGGAATGTCTTCCCATTTTACTA	CTGTATTCATACACTCCCCTTTAGCAAT
5 LIFR	TGGATTTGGAATATCAGGGTAGAAGGT	ATTTTAATCTCAGATGTGCATTAAAGGATTC
5 LIFR	CTAGAACACTACGCATGCAGGAT	GAGAATTGCTGATCTTCAAGGTAAAACAA
5 LIFR	TCGCAAGACCAGGTGGTAAC	ATTTGAAACGGGTTAGAGATTAGTGCT
5 LIFR	CTTACTGGCTTCTGTTGTTAAATGTTGT	CTTGTTGCTCTGGACAAGTTAAATCC
5 LIFR	CAGAAAGTTTCAGTAGAACAACGAATCC	tTTGTTTTCCAATAGCGGAATGTCAC
5 LIFR	AGAGAATCTTACCTGCTCTTGTACTGA	CAGTTTATCCCCATACTCCTACTTCATT
5 LIFR	ACAGCTGTTGAATTAATATCCTTCACTT	TCAGCACTAATGATGCCTCGTT
5 LIFR	GCAACAAAATTGCTTCGTCATCAAAAT	TGGACAATAAAAGAATGAGGACTGCTT
5 LIFR	AATGTTGATAACAGCCACTGGAAATTTG	CCTAACCTAGGACTGACTGCATTG

5 LIFR	GCCATCTGACATCTTTTCCCAGTTAT	TTTCTGTTAGATCCACCAGATACTCCT
5 LIFR	AAGCACCACAATACTAACAAGTGACA	AATTCCAGCTCTTTCACATGGTGA
5 LIFR	cccAGTCTTACGTGTTGCCTTT	ACCATGCCTTATGGACTGGAGA
5 LIFR	TCAGTGCTGTTTGAGGGAACTTT	CTCAAAATAGAACAAGTTGTTGGGATGG
5 LIFR	CATAATCAGTACCACGGCCTGT	TGCAGTTGACTTATTCAGCATATTCtgt
5 LIFR	GTAAGAGCTTATTGAGATGGCTGACT	TCATTTGGAAGTCCATGCTCCATT
5 LIFR	GGAGGAATCAAAAATTGTCGGGAATT	AAGATATAGCTGCAGAAGAGGACTTAGA
5 LIFR	GAGGTCTGTAACCCGCAGTTTTA	GTATCAGCCTCAAGCAAAACCAG
5 LIFR	CTTCTAGGTCAGCAGCAACAA	CACCTTCCAAAATAGCGAGTATGGA
5 LIFR	TCTCAAGCACTCACCATTTGGAATT	GAGGAAACACAGTCCCTTTCTGA
5 LIFR	CTTTGTGCTGAGGATCAGGGATT	TCACGTTTTGCAGGGTTTTATAACAATT
5 LIFR	ACAAAAGCCAGTCTGGGAAGTT	GGGCCTGATACTTGGAGAGA
5 LIFR	TCCATCAGAACTCCACTCTCTCC	AACTGCTTCACGTTTTATTGAGAATTCC
5 LIFR	AAATGTTCTTCACAGGGCTCCA	ACAACTCTGAATGGCAAAGATACACTT
5 LIFR	GGCATATCTGAGGCCCAACT	GGAAAGCAAAACATATTGCTACTTGGA
5 LIFR	GTTCACTGATACCACCTTAAGGCT	ACCGCTCAAATGTTATCTGGGAAATTAA
5 LIFR	AGCTCCATACTCTCTTTACGTAGAACT	AATTTGTCTGCTGATTTCTCAACCTCTA
5 LIFR	GATAACATTTGAGCGGTGTGGAAAA	GTTTCCTCTTTTTAAAGCCTTAATTCCAGAT
5 LIFR	AGCATTTTTCATTAAAGCATTTCACCACTT	CACAATCCGCTGGGTCGAT
5 LIFR	ACTAAAATTGTTGATTGTGATCGACCCA	GTTAGACTTAAAAGAGCTGAAGCACCTA
5 LIFR	GGTGCTTCAGCTCTTTTAAGTCTAACAT	TTATACAAATTAAGTATTACGAATTTGCCCGTT
5 LIFR	CCTACAGGGTCATTTTCTTGTTCTTCTT	CCTGAAAACCATGTGGTTGTGT
5 LIFR	CCTCAATGATGGGTGGACAATAGG	TGGAAATGAATCCTTGTACCCCAAATAAT
5 LIFR	TGATCGAGTTTCCAGAACCTCAAC	ATTGCAATGGCATTTAGTGCTATTTTT
5 LIFR	GCCAATTTATAATTTCTCATGTTGCCTTGA	ATCCTATAGATGAGTTTCGACCAGGTAT
5 LIFR	CCTTGATTTCTGCATCCATACAGGAA	TTAGCTTTGGAAATTTTAGAGTATGTTATGAAC
5 MTRR	CTGGCAAGGAAGAAGATTGAAATACAAG	CCCGTGGTAGAAACCACAACA
5 MTRR	AAACCGAAACAGCTCCTCTTGT	AGATCAGAAGAGAGAGTCCATTACCC
5 MTRR	AATGTGAAGCTCTGCATTATTACTCCAT	CCCATTGCAAAAGTAGGTGTATTCTG
5 MTRR	GGGTTCTCTAGGTCTCGGTGATT	AGTAAACAGAGAACACTGCCCTTAC
5 MTRR	CTAATAGCTCTACCCACAAATTGTGTCA	GCTATAATCGGCTGCCCCTT
5 MTRR	GCTACAGGAGCTGTGCAGTAAAC	GGTGCTTGTGAAAGCGACTACT

5 MTRR	CCCTACAGCTTAAACTATGTAACACGTA	TTATCTTCAAGCTGCAGTCTTTGGA
5 MTRR	CAGTGATTCTGAGGTACAAAGCCTA	GAGCCACACTCTCACCATCAG
5 MTRR	TCTTGCCTAGGTTTTCTTTCATACTTACC	GTTCCTGGACCCACCATTATGAT
5 MTRR	CTTACCAGATGACCCCTCAATCC	ACGGTTACTCAGGAATACATTTTCCC
5 MTRR	GTGTGGGTATTGTTGCATTGTTTCTT	TGCTGTGTAGCATATAGTAACAGAAACC
5 MTRR	GTTACATGCCTTGAAGTGATGAGGA	GGTAAAATCCACTGTAACGGCTCTAAC
5 MTRR	GTCCCCTTCGGAGCTTTCTAT	CGGAATCCGGGAACCGTA
5 MTRR	CTTCTATGTCATGAACCCCTTTCCC	ACCTTTTTAGGAATTGCTCGGATTTCA
5 MTRR	GGATTGGTTTGACCCATATGTGTAGT	CAGCCTGTACATACTCCCTTCC
5 MTRR	GTCTACTGCCACAACAGAGGTT	GAGTTAACAATGCTGAGGTTAAGGTACT
5 MTRR	AAAATGCCTGTTTGTAAGCAGTCATC	GGCCAGGGTTTTCATTGCTTCTA
5 MTRR	AAGCAAAGAGGTTGGAGTTGAAAAAC	GGTTTGGCACTAGTAAAGCTGACTTTTA
5 MTRR	GAACATCTTCCTAAACTTCAACCCAGA	TCCATAAAGCAGGTAAGTTTTCACTGAA
5 MTRR	ACTGAATGTTCAAATGCAGAGATGGAT	GCTCCAAAATTTCCATCTGGGTGT
5 MTRR	TCCACTTAGAGAGAAACTCCAAGAACA	CTCAGTGGTTGAGTACAATACTGGAATT
5 MTRR	AGCTTGACAACCTTTTAGTGATCCAT	ATGTTGTCTTGCACATACTTTGCTG
5 MTRR	TTCTCAAGAGATGCTCCTGTTGG	CTACCCGACTTAGGCACGAT
5 MTRR	GGAAATGAATACTGAGTAGCCAGGAA	ACTTGAAAAACTGGATCTGCTGAAGT
5 MTRR	AAGGAGGAAAGCCAAGTATCTGTG	CATCCTGACAATATTACTGTGCTGAGAA
5 MTRR	CAGACTGTCATTATCCTGTTCTGTGT	CTTATCTCCTCTTGTCCTCTGCTTG
5 MTRR	AGCCCTCAGAAAGCATTTTAGGT	AACCTCAGAATCCTTTCTTCCTGAATC
5 MTRR	CAAGTCGAGCTTCTGAGATTCGAT	GGGTAAACCAGGAATATTCAGAGAGG
5 MTRR	CGGTACCCCCACTCTCACAA	GCCCAAAGCAAACATCCATGATTA
5 NPM1	ACAGCTTTGTTTGCACTGTTGTT	CCCACGTGTAAATGTAGCCACTTA
5 NPM1	AACAGGTTCACTGGTTTGTTGATTTG	CCCTGAACCACACTTCAACCTTAA
5 NPM1	CTTTGAAATAACACCACCAGTGGTC	GCAACTGCACTAAAGAGGACAAACTAA
5 NPM1	TTTTTCAGGGACAAGAATCCTTCAAGA	AAACCAGACACGTGCAATTTGAG
5 NPM1	TTCTGACTTCTTGCTGCTTGAGT	ACCGCTTTCCAGATATACTTAAGAGTTTC
5 NPM1	AGAAGATGAAGAGGAGGAGGATGT	CAAGAGCTGTTCTATCACCAAACTAAAAG
5 NPM1	GTAAACCCTTTAGCCTTCCTGGTTA	CTTAATTTACATGTAGTGCCCAGGACT
5 NPM1	TGACCTTTTTGGTTACCCACACTT	ACTGGTGCTCATTTTCATCATTATCCA
5 NPM1	AGGCCGACAAAGATTATCACTTTAAGG	AAAGTCTCAAGCCACATGAATAGAAACT

5 NPM1	AGGTATCTCTCTCGGTGTATTTCTCTAC	ATCATGTCCCCAGAAAATCCAGTTAC
5 NPM1	GATGTCTATGAAGTGTTGTGGTTCCT	ACAGCCAGATATCAACTGTTACAGAAAT
5 NPM1	GTCCGCCTTCTCTCCTACCT	GGATTCCCGCCCCTCAC
5 NPM1	GGGTGTAAAATAGGTGGAACTCAAAAGT	GCCTTCGTAATTCATTGCCTCTG
5 NPM1	AAAGGATGAGTTGCACATTGTTGAAG	CCTTGACACAACATCCAAAGTATGTAAG
5 NSD1	TGTATACATTTGGATACCAGTGTCCT	ATTCGAGCTCTGCATTCTTCTTCAT
5 NSD1	GTGAATGAGTATGTGGGTGAGCTTATAG	CAGTGAAAACAGCATTTCCCATTACTT
5 NSD1	TGGGATCTTTTCTCTGAGAGGTT	TGAACCGAGCATAGTTTCCTTTGG
5 NSD1	CCCTGGGAAAAGCCTCATCAC	CTGGAAGTGTATTTTGCTCTGGTTT
5 NSD1	ACCGAATCATTGATGCTGGTC	TGTCAGCTGCAGAATCCTGAAAT
5 NSD1	GTCTCTTGGAAGAGGGCAAGAC	GGCAGCTTGTTTGTTCATGTGA
5 NSD1	GGTCAGGGACCAAATCCCAATC	TGACTGAGGGTGAGGAGCTT
5 NSD1	GACAAACCCTCTCCAGTGACC	GGAACTGAGGTTTTCTCCAGTGA
5 NSD1	GGATAAATCCATAGGTGCTGCCA	TGGGAGTCTCTGGGACAAAGA
5 NSD1	CCTACTGACAAACCCCATGCC	GATCCATCACCAAAGCAGCTCTATT
5 NSD1	CTGTGGACCAGAATACTCAGTCAAAA	ACTTGACTCCAACACTGGCATC
5 NSD1	CACACCACAGGCTGATGAGAA	GCTTTCCCAGATGTCTGAAGAGG
5 NSD1	ACTTGTGCCCAGTTTCTAAATCATCT	GCCAAGGTCCCCCTTCT
5 NSD1	CCCTGGATTTATGCCCAAGAAGG	ACTGCTGACACACACACTAATTTCT
5 NSD1	TCCTCTGTAAATGGTGTTGTTTTCACT	CAAAGCACTTCAAACTTACTACTGCATT
5 NSD1	TCCCCTGAACTCCAGGTAAAAGTAA	TCTGAGTTTCCTCAAAGATCTCCTCTA
5 NSD1	GCCAGTCACAGAGGATGAGAGTA	GCTAATGGCAAGAATGGCGATT
5 NSD1	GAGTAGACACGGTGCAGTCA	TGTTTGTGTCTCCTAGTGAAAAGGG
5 NSD1	CTGACTTGTTTTATGCGGTGTACTT	TGCGGTTGATGCATTCAGAGT
5 NSD1	GCAATGAAAAAGCAGCCCTTCTC	GATGAAGTTCCAGGTAATTCTAGCATGT
5 NSD1	CCAGATTCCAGTACCAGTACATTAGGAA	CAAGTTTCCCTTTAAGTGGCCTGTATAT
5 NSD1	TGATGAGAACCCCTGTGGGATAG	ACCTCTGGATATTGGCGCTTG
5 NSD1	CGCTGTCAAAACCAGTGCTTTTC	AAGCAACTGCAAAGAGGAGAAAGTATAT
5 NSD1	AACAATTTTGGCCTGTGGACTCTA	AAAGAATTCTCGTCCACCTGCT
5 NSD1	AGGTCGATCTAGTGCTCAGAACA	GCAACTCAGCATGTCCACCTC
5 NSD1	CCCTTGGCTCAGTCAGAACTT	TGCTCAAATACTGAGACCCCAAC
5 NSD1	TTTCCTAATGCCTTGCAGCCT	CCCACATCATGTCTCATCTTATCAATGT
5 NSD1	TCCTCGAGCTGTTCCTTCCA	TCTTATCCTTGCTGCTCACGTC

5 NSD1	AGTCTTCCCTTACATGGAGGGT	ACAGATCAGACACTCAGGTAACAGA
5 NSD1	TGATTCTGGTTCTCTTACCCTTACCT	CGAATTGATGTCTGCCTTCAAACA
5 NSD1	TGGCTGGAAAAGCAATCGTCA	TCTTTTTCTTACTTGCTTCTGTCACTCA
5 NSD1	CACTGCAAAGAAAGAATTTGAGACTTCA	CTTTTCCTCATCACCTGCTCCAATA
5 NSD1	TGGTTCAAAAGTGAAGCTCTGCTA	GACTCTGAGCTGTGTTCTATGGG
5 NSD1	GATGGAAGCAGTGACCTGGAT	CTTCTGTTTTGCTTTTACCCTAGTGTT
5 NSD1	AAAGTATTCTAGGTTTGCTGCCACA	GTGAACAAGAGTAGATGCCTTCAGA
5 NSD1	AGACGTCTCAGGTTAATCTCTCTGA	GTTCTCACTGGATATGCTTGATGACA
5 NSD1	ATGACTTGCAGTCTTGTGATCTGAAT	TTTCCTTCAGGGATATCAATGTTCAGG
5 NSD1	CCTGCTGCTTTTCATCGTGAATG	AGAGATGAGTGCTATTCTTCAGGCTTA
5 NSD1	TCAGATTAATTGCTGTGCTTTTGGATTC	GGCTCAGAAAAATTGGATTGACCAT
5 NSD1	AAGTGCTTAATGGCTAGACAAATAGCA	TCCCGGGATCGTGTTCTACA
5 NSD1	CCAAGGAGACTGTTGAGGAAGG	ACACAAGTGGGCAAACATTTCTCTA
5 NSD1	TGAAAAATTGGGTGAGCTGCTG	TTGGAGGAAGACCGTTCGATATCTA
5 NSD1	ACATTGCTTTTTCAGAAGGCTAATAGGA	CCCAGATGAGATCTCCAACTTCATACT
5 NSD1	CCTAAGAAAAAGTCTACGCCACTGA	GCAACTGCCTTTAACATGTATAATCACT
5 NSD1	TTTACTATGTGGTTTCCCATCTGGTTAC	GGCTTCCTAAGGCGTTTCTTCTC
5 NSD1	ATCATCTTTTAGCTGTGCGGTCA	GGACCAGTGAAAGTTTGCTGAAATT
5 NSD1	TCTTTTGCTTGTCCCTGATTTTCCT	GGGTAAAGTGATTAGGGCAGATGATACTA
5 NSD1	GCTGGGTCAAAGATCCTTGCAT	AGAAATGATTTCTTACCTTCTGAGCACA
5 NSD1	CATGTTAATGTTAGCTGGTGCTTTGT	CCAGTGGCAATATGATGAAAAGTATTTTTGT
5 NSD1	CATAGCCTTGGCCCATGTGATA	GATGCTGCTTACAAAAGGAGCTG
5 NSD1	CAGCCTCCTTCTGTGAGATGT	CCCAGGTTCCAGAGGATTGG
5 NSD1	TTGAGAAAGGCTGTGTGTCAGAT	CCTGCAGAAGCTCTTCCTGAG
5 NSD1	GCCTTTTTATATGAGCCAACAACTCA	GAGACCTAAAAGATTGCCCACTCTT
5 NSD1	TGTACTGAGCATGACCCCTGT	AGCAGGAGGTTTATCTGACATTTTGG
5 NSD1	GCCAGCAACTACCTGCACTT	GTCTGTCCAGCCACTATGGG
5 NSD1	ATCAACAGGAATGGCTGCTCA	GTCAGTTCTCTCAAGAGGTCTTTCAG
5 NSD1	GACTTGTCAGAGGCCATTGCTA	GCCTCTGGCTGGAAACCAAG
5 NSD1	CAGTGGGTTCAGACGATGTCA	CCACTCTGCTTACATACAAAACAGGT
5 NSD1	TTCTTTTGGCTTCTCAGGAATCCAT	CTGCATAACAGTGGGTGGGTA
5 NSD1	TTTACCATGAAGAGTGTGTCCAGAAG	CAATTAGACTGGTCCACATAAGAAATCCA

5 NSD1	CAAGGACAGCCCTTTCGGTA	GATCATGGAGGCCAAATCCTGTAG
5 NSD1	CTTGTTACATTCCACTGCGGAGA	AGGACTCAAGGAAGTGCAAACA
5 NSD1	ATTCAAGAGCTCAGACGCCAAT	GCAGAAAGCCATTCTTGATAGTCTTTG
5 NSD1	TTGGTACTAATGTGTTCACAGAATGCT	CATCCCCACAACTAAAACACTCATCT
5 NSD1	GGTGAAATCACAAAGGAGCGAGA	CGAGTAGCGGTACAAATGGATTTTG
5 NSD1	AGCTTCTGATTTCATCTCCCTTTTCC	TGACCCCTTGGGAACATCATACT
5 NSD1	TCCAAGTGTGACTCTTGATGCTG	CTTGCTATCGAAATGCACATCAGAA
5 NSD1	TGATGACTGCTCAAAACCTGGTC	TTCTGAGTGCCATCTCCCTTTTT
5 NSD1	TGATGCTCTCTCTCCAAAATTCAACC	TCTGCCATAACTGGATTTTCTTTGTGT
5 NSD1	CTGGGCCACGGTTAAATGTTT	GACAATGTTTATCAGCACTTTCTCCAAT
5 NSD1	CGAGTTGTCTGCTTCCCTACC	TTTACGGTTCACTGTGTTCTTTACCA
5 NSD1	GCTCTGATAATCCAAAAAGGACTAGTGT	GGCTATTTGCTATCCTGGAAAGTTCATT
5 NSD1	CGTTTAAACCAAAGGCGCACTA	TCAAGTATGCTTGCTGAAGGAGTTC
5 NSD1	AACATGTTTTATCCGAGTTGAAGGA	AGCAATGTACTGAATTTGTAGTCTGGTT
5 NSD1	TCTGCTTCTAGTCAGAATCACATACCT	CGACCAGGACTCCGGTAAGA
5 NSD1	CCAGTGTTGGACTTGCAGAAC	GCCAGTGATTTCAAACAGCCAT
5 NSD1	GAGGCTCCACACACAATTCAGA	CTCTTGTCGGACAGTAAGCCA
5 NSD1	ACTTAACAAGTGAAGATGGTGACCATTT	CCATTGAGTTCATCATTCTCACTGTTC
5 NSD1	GAGTCAGAACATGACCTGTTGCTTA	GCTTCAAATTGTATGTGGCCCTTTTTC
5 NSD1	CCCAGAGCTGGACTCTGTAAT	TGTTCATGCGCTTACGAGGTT
5 NSD1	CCCAAGTTCCGAAGTATAAAGTGCAA	GTTTTAGACCATCCACTTTCCCACT
5 NSD1	TCTCCTTTGGCCAGCATTTCTAAA	CCTCACCTAAGGATCTGTAAGAGAGT
5 NSD1	GGATATATCTGATACGCAGGCCTCT	GCAAGCCCAATAAAGAGTCACCATT
5 NSD1	CGGGATGAGTTTCCAGAGCATA	CAATGCTGGCACTGGATTTATCAC
5 NSD1	CGAGATGCTTTTTCAGCCCAAA	TTCTCGGTCTCCCTGCAGTA
5 NSD1	AGATTTTGAAGTGACTTGTGCTGTCT	GGTCCCCCAAGATTTTCTCCTC
5 NSD1	CCCCGTTTTCCTAATCCACAA	CGCTTCCTTGGCTTGTCAAATATCT
5 NSD1	CTATTTTCCTGTCATAGGCACTACCA	GAAGGTGAAAAGATCAGGAACTGAGTAA
5 NSD1	TGTTCTCTTGGGAGTTGGTATCCT	CAAGACATTCTAGGTTGTAGTTGAAGGT
5 NSD1	GTCTCTTATGCAGGCACTGAACTT	AAGCAGAAGTCTAGAAGGGTGGTA
5 PDGFRB	aaatgcaTGAGACTCCACTCACAT	CTCTTGTCTCTGCTCCCCAAC
5 PDGFRB	gcctcTCAGGACTGACCCA	CTCCAGAAGCCACGTTACGA
5 PDGFRB	AGCTCACAGACTCAATCACCTTC	TGTCCTAGACGGACGAACCT
5 PDGFRB	AATCAGCCTGCAAGCCAGAT	GTGCATTGTGATCGGGAATGAG
5 PDGFRB	TGTCCACTCGAAGTTGACCAC	GACCTAAGCCAATCTCTCTCTACCA

5 PDGFRB	AGGCATGAGTGGTCGAGGTA	CATCTTCAACAGCCTCTACACCA
5 PDGFRB	TGAAGATCTCCCAGAGCAGGAT	CTGAGGTCCTTCCTTGCACTC
5 PDGFRB	AGGGCTCCAAGGACTACTCA	GCTAATGGATCATCTCTGCACCAG
5 PDGFRB	gttatACTTGCCTCTGCTGAGCAT	CTGCCTGTCCCCTATGATCAC
5 PDGFRB	TCAAAGATACCAGAAAAGCCACGTT	CTCACGGAAATAACTGAGATCACCA
5 PDGFRB	GGTCTGTTACTCGGCATGGAA	cctgattTATTCACGGCTCCACTC
5 PDGFRB	GAGCCCCACACAGATTTCCTAT	CGAGACATCATGCGGGACTC
5 PDGFRB	CGCTGCCTTTGGAGATGTAATTC	TTTCCCCACAATTGTCCCCAA
5 PDGFRB	GCAAAATAATGATGTGCCAGTCTTCA	TCATGGCCTGCTCTCATCTCT
5 PDGFRB	TGGGAAAATACAACACTGCCCAT	GGCCAATGGCATGGAGTTTCT
5 PDGFRB	CACCACACGTACGTTCTTGGA	GTGACCCTCTGCTTCTCCTCTA
5 PDGFRB	AGGAGTGTGCTGTTGTGCAA	CTGAAGATCATGAGTCACCTTGGG
5 PDGFRB	CCCAACAGGTTGACCACGTT	GAGTCAGAATAGGCTCCTGTGG
5 PDGFRB	ATGTGAAGAGCATCAGCCTGTT	TGAAAGGAGACGTCAAATATGCAGAC
5 PDGFRB	GCCTCCTAGGATGCAACTCTA	CGAACATCATCTGGTCTGCCT
5 PDGFRB	CCATGTAGTTGGAGGACTCGAT	CTCCTGTGATGCTCCTTTACCC
5 PDGFRB	TGCTCACCTTTTGAGGTCTCTG	TCCTAACTCAGTGCTCTCTCCAT
5 PDGFRB	GGCGGCTGCAAAGAAAAATAACT	GCAGGTAGTGTTCGAGGCCTA
5 PDGFRB	TTGTCTTTGAACCACAGGACAGT	CCTGACGGTCACCCCTT
5 PDGFRB	CTCCTCAGGTATCCCAAAGATTCA	ACGGAGACCTGGTGGACTAC
5 PDGFRB	CCCCTACCCTGCCTCAACATA	GCCCTGACCCTTCTCCCTATC
5 PDGFRB	CTGCAGGAAGGTGTGTTTGTTG	CCACATCCAGCCCCTGATTA
5 PDGFRB	CTGATGCCTCCAGTTGACAAGA	CTCACACTGACCAACCTCACT
5 PDGFRB	AAAAGTATTCTCCCGTGTCTAGCC	AATGTCTCCAGCACCTTCGTT
5 PDGFRB	GCTGAACCCGAGCAGGTC	GAAGCCCCTGTTTCCTGATGT
5 PDGFRB	CATGGGTGGGTGGACAGT	CTTCCATGAGGATGCTGAGGTC
5 PDGFRB	CATTGATCTGTAGCTGGAAGGAGA	CCTTTCCTGCTCCCCAGGTA
5 PDGFRB	GGGAGCGTTGAAGGAATTGG	CATCCCCAGTGCCGAGTTA
5 PDGFRB	GTGTAGGTCCCCGAGTCTTC	GCTTGCAGGCTGATTTCTCC
5 PDGFRB	GTCCCCTACCTTATCTCCCATCT	GGTGGTGAGATAGCCCCTGT
5 PDGFRB	CCTACAGGAAGCTATCCTCTGCTT	TCCTCAACCATCTCCTGTGACA
5 PDGFRB	GCTCTGGCTCTGGTTCGT	tgCTGCTGGAATCCTCCTG
5 PDGFRB	CAGCCTGGCTGACAGGAA	GCTGGAACAGTTGCCGGAT
5 PDGFRB	acacacTGTGCACAATTTCCTTG	CCAATGAGGGTGACAACGACTATAT
5 PDGFRB	GGGCCCTCGTCAGCAAC	GTTCCATGGCCTCCGATCTC
5 PDGFRB	GCAGTATAGAGGACGGAGCTG	CAGAAGTACCAGCAGGTGGAT
5 PDGFRB	CCTGGGCATGCTAACTCCTTT	GGACACGCAGGAGGTCATC
5 PDGFRB	GGGACTCACAGTGTGGCA	GGAGAGCCAGCTGGAGACTAA
5 PDGFRB	TCCTCCTCCCAGTACGTCAC	AGTTTCTCCTCAGTTTCCCTGTCT
5 PIK3R1	GGAAGTAGTCGCTTACTCATTTCTCTT	GGTTTGAGAGAGCTTGAAGAAATGTTTT
5 PIK3R1	AGTATTGGCTTACGCTTCAGTATTTGTT	CCAAAATACATGTTTGTCTCCTTTCACT

5 PIK3R1	CCAGGGAATATAGCTGAAATTAGGGT	AATGGGTCAGAGAAGCCATATTTCC
5 PIK3R1	TTCTCTGACCCATTAACCTTCAGTTC	GGATCTTGTCTAAACATCGTAACTGGAT
5 PIK3R1	ATGCTATCACACACATTCACTTGAGT	ACCTGGTGCAGGCTGTC
5 PIK3R1	ATCTCAACTGAATGGAATGAACGACA	AAGCTGTGTTACTTCAAAGGAGGTTTAT
5 PIK3R1	GTCCTGGTAGTGTCTTGCAGT	GGTCTGGCACTGTTCTTCAAATATTTTT
5 PIK3R1	GGACAGCTATTGAAGCATTTAATGAAACC	GAGGATTTTGGTTATCTCTAGCACAAGA
5 PIK3R1	CCATTACAAAGAAAGCCGGACTCT	CCTGGTGGGTCCATTTCTATATAAAACAT
5 PIK3R1	GACAGATATAAATTGTGGCACAGACTTG	CAAAAAGGATCTTTTACAGTAGCTTTCGA
5 PIK3R1	GGTTCTCCAGAGAGCTGTGTTT	GCATTTCTAGCTCTGTGACACTCA
5 PIK3R1	TGCGTTCTCTTTTCAAAACTGTTTTTCA	AACAAAGCAAGTCATGCATTTTCCTAAA
5 PIK3R1	TGTGGTCTAATGCATTCAACTATCCAA	GCCCTCTAGCAGTCTGGTTAC
5 PIK3R1	TGGTCACTAAACCTTAAGATGAGCATTG	CCTAATTTCAGCTATATTCCCTGGCTTA
5 PIK3R1	ACCGTTCCTGATGTACCCAGAT	GATACTCAGCTGCCTGCTTCTT
5 PIK3R1	GACAGTAGAAGAAGATTGGAAGAAGACTT	CAAGTATTGGTCTCTCGTCTTTCTCA
5 PIK3R1	ACAGCATTAAACCAGACCTTATCCAG	CGAACACCTTTTTGAGTCAACCA
5 PIK3R1	GATGGCTCCTGCACTCTTCATTTA	GAAAAGTGCCATCTCGCTTCC
5 PIK3R1	CAAAGCTGAAAACCTGTTGCGA	GCCCCCAAAACTCAATGAATGAA
5 PIK3R1	CACATGTGAGTCAAATTGTTCAGAAAA	GCACATCGATCATTTCCAAGTCC
5 PIK3R1	TGTTTCCTAGATACACCCTCCGT	TTCAATGAGGTTCCCAGAAGAGAAAAA
5 PIK3R1	GCCCACTTGGTGGAAGAACAG	CACCGCTCAGATAAGGAACAGAG
5 PIK3R1	ATTAAAGATGCCCTGAAGAGTTGTGAA	CAGTTCTTTCAGAGAGCTGTACAAGT
5 PIK3R1	GGCTTTGCCGAGCCCTATA	GAACTTCAGGAGAAGGATCAAAGAGT
5 PIK3R1	TGTGAATAAAGGGTCCTTAGTAGCTCTT	GGTGTGGGAGGCGAGATTTTT
5 PIK3R1	TCCGGGAACTTACGTAGAATATATTGGA	AAAGAAAGGGAGTCATTAAGCAACCA
5 PIK3R1	TCAACTGTTGCATGGTAGCAGAT	GCTTCCTGTCCATCACTGAATCC
5 PIK3R1	TTCTAATGTAGTTGGGATTGCGAACA	GTACCATTCAGCATCTTGTAAGGACATAT
5 PIK3R1	TGTAGCCAACAACGGTATGAATAACA	GCAAGCTGGTGCTTTTCTTTCTTTTAAT
5 RAD50	CACAAGTTCATGTGTCTGACAAGGT	GCTCCACAAAATCTTCATCATGAGTGAT
5 RAD50	GTTACACAGGTTTTTCAAGGGACTG	CCTGTTCAACAAGCAGTTCTGATTTT
5 RAD50	AATCACCAGAGAACAGTAAGGGAGA	GTCCTACCCTGTTCAACAAGCA
5 RAD50	GCGTAACTTCCAGCTTCTGGTA	GCTAACACTGCATTTCACAATCTCTG
5 RAD50	AGGATTAAAAAGAACATCGATCAGTGCT	CACTTTCTGAGGACCTACATTTCTATGG

5 RAD50	AAAAGGTCGTCTACAGCTGCAA	TTTCACTGAATGGTCCACGCT
5 RAD50	GGCAACACAGCTAGAATTGGATG	CCATCAGTTGGTTGGCAGTTTTTG
5 RAD50	CACAAACTTGTGAGAGAGAGACAAGAA	TGCCAAAATGGAGTCCAACCA
5 RAD50	GAAAGACATTGAGAATTATATTCAAGATGGGA	TCGCATTCACTTAGTTGAGCTATTACTT
5 RAD50	AGATGGGAAAGACGACTATAAGAAGGtaa	TCTTTTCTTTGTGTTTCTCGCATTCAC
5 RAD50	AGAAAAAGATACAACCGTATTCAGAATACT	TTAGCTGAGTAATGAACTGGGAGTAAAC
5 RAD50	AAAATAGCCATGCTGGCTGGA	ACTTCTTGTAACTCAGCCTCTGTCT
5 RAD50	GCCCCGTTTGTCAGAGAGTTTTT	CATCACGCCGCTTTTCCTTTTT
5 RAD50	AAACTCAAGTCAACAGAATCAGAGCTAAA	AGGCATGAGATGGGTACCTTTTAC
5 RAD50	GGCAAAGCATAATTGATTTGAAGGAGAA	CAGGCATTATTGTACCCAAGAGTGT
5 RAD50	GCCTAAAGAACGACATAGAAGAACAAGAA	CAAGCCACCATGGAACAAGTTTTAT
5 RAD50	GTTGAAAGAAAAATTGCACAACAAGCA	GTCTCTATGAGTACAGAAGGACACAGA
5 RAD50	AAGAAATCTATGACTTTTCCACTTCAGGTT	TCTGTCGCCCTAATGCCAAATT
5 RAD50	CATCAGAAGTTGGAAGAGAACATAGACA	CGAAATTGTGGTTCTCGAAGTTCTTTC
5 RAD50	GGCGACAGAAAGGTTATGAAGAAGAAA	CACAAGTTCTGTTGTCCTCATAACAATC
5 RAD50	GGGATGCTGAGGAAAAGTATAGAGAAAT	ATTAACTGCAATAAGAAAATCCCCAGTCT
5 RAD50	ATGACTTTGCAGAAAAAGAGACTCTGA	TCCAACTGCTGTAATTCATACTTCACAT
5 RAD50	CCTAAGTAAGAAGCAGAATGAGCTGAAA	TATTCAAACAATATCTTACAGCTTTTATGAGCT
5 RAD50	ATGACAAAAGGCTACAGAGCATAGG	CAGCATCACCACTCGGTAGTTAT
5 RAD50	TCAGCTTCTGATAAAAGGCGGAATT	GCTCCCCAGGCTTTTGAGATAC
5 RAD50	CTCAGAACCAACACTGGTGCTTA	TTTGCTTTTCTGAGTACACACCATAGAT
5 RAD50	CAATGGAGAACTTATAGCTGTGCAAAG	CCATCAACTTACAGACCTAAAAGACTGA
5 RAD50	ACTGTGAAGTCTGACCCCTAAAGTA	TTGCTGGAACTTTTCCAATGTTGTTT
5 RAD50	AAGAGCAGGTAAGCCCTTTGG	GACGTGGTGCTATGAACATAAGTTTAAT
5 RAD50	CCTTGCTTCGGCCTCAGTTA	GGGCTGAAGAAAGTGATAATTTGCTTAT
5 RAD50	CGGAGTTTTGGAATAGAGGACAAAG	CCTGTAAACTGAAGGCGGCTA
5 RAD50	TGAAAAGATCATGTCAGGACTGCTT	CAAGGCAATGATGCCACAGT
5 RAD50	GGCTGAAACGTTCTGCCTCA	CCAGAGCATGTGCAAGAGATACTTAC
5 RAD50	GGGATAGGTGAAGGGCCTTTT	ACACTTAGAGCTCAGACTGACCTTT
5 RAD50	TGATTTTCATTTTCTGTAGGCATGGTGA	TGCTTCAAAGCCTTTCCTTCACTTA
5 RAD50	TGTCATCAAGAAGATTCTAATTGGCCTT	GCATCCAAATTGCAAACACAGTTCA
5 RAD50	GGTAAACTTCTGTGGTTCTCTTATAACGA	CCATTACCTTGGGATCGTGTACAAA

5 RAD50	GCATTTGTGGATTCCATAGACCGATAA	ATGTTGAATCTGTTCCTGCTGGT
5 RAD50	gctcTCCTGTTATGTGCCCTTA	aCCTTCTTATAGTCGTCTTTCCCATCT
5 RAD50	CCTTAGAAACACTTCGGCAGGT	CTGGGCTTCCTTACTTGTAATCTGAT
5 RAD50	GTCAGACACAAGGTCAGAAAGTAAAAGA	CTTTGAAGATGTTAACTGGGCTTCC
5 RAD50	ATGATTTGTTGGCAGAATTTGTCTTGT	GACAACTGCTCTTCCTTTCTTTTTAGTT
5 RAD50	GGAACTAGCTTCATCTGAGCAGAATAAA	GCCTGTCTAAATCACTTTCAAAATCCTG
5 RAD50	GACAAGCTGTTTGATGTTTGTGGTA	CCCATCCTTAACAGTTACCTGTGA
5 RAD50	ACAGATTTCATGTTAGTAACTTGGTTATTTTTG	ACCTGCATTTGACCCATTTCCTT
5 RAD50	TGTTGGATGCAAACAGTAATATTTGGA	CTGTTTTTCTCAGCCTTGCTTAACT
5 RAD50	TGACTAGGAACGTGAGTTAAGCAAG	ACTGCTCCATCTCCTGGTCAA
5 RAD50	CTTAGACAGGACCCTGCGTAAAC	GTGCAAGGTAGGCTATTTTAAGTACCA
5 RAD50	atatatatGAAGTACCAATGACTTCCTTTTCC	TGGTACTCACCTTGTCCACGATA
5 RAD50	TTTAAGCTTAGAACTTTAGTCAGTCTAGAATTT	AGGTTAATTCATCACTGTGCCTAGATTT
5 RAD50	TTTTACCTATAGGCTGACAAAGATGAACAAA	TGTCCCTGGTCTGATTAATTTCTTTTGA
5 RAD50	GTGATGAATTAACCTCACTGTTGGGATA	TCTATTACATCTCCAAATGTTGCAACTTACT
5 RAD50	AAATTATACGTGACCTGTGGCGAA	GGGTCCAGGGAGGTAATGCT
5 RAD50	CTTACTTGTCTTCATCTATCAGCCATGT	TTCTCCATTTGCTTCTTTCGGCTA
5 RAD50	GACAATGAAATTAAAGCCTTGGATAGCC	AATGTGGATGGCAAAATGGATTCAAA
5 SDHA	TGCCTTCTGCTTATCTTTTTCCTCTT	TCCCTCTCCACGACATCCTT
5 SDHA	GTGCTGGTTGTCTCATTACGG	AAATCACACGCACCTTCCTTCT
5 SDHA	GAGTGGCTGTGCTACATGTTTG	ACAGATTCTTCCCCAGCGTTT
5 SDHA	ggccTACTTCCCTCTCTCTGA	CTCCCCACAGGCGTACA
5 SDHA	CCTGCCCCTGATGGAACTT	ACCTTGTAGTCTTCCCTGGCA
5 SDHA	CCCTCACTGGGAGTCACTGT	TGTTCCCCAGAGCAGCATTG
5 SDHA	GTTGGTGTTTCCAGGGAGGAAT	GCCCATCACCTCGACCAC
5 SDHA	ACCAGGATGCCATCCACTACA	TGCCACATCCGCCTCAATT
5 SDHA	TTGCCGTTCTCTGCCGTAT	ATGGCTGTCTCTGAAATGCCA
5 SDHA	TGCACCACCTACCTCCAGA	CATCACCTGCCCCTTGTAGTT
5 SDHA	CCTGCCTGGCATTTCAGAGA	CACCATCACCTGCCCCTT
5 SDHA	CGGCATTCCCACCAACTACAA	TAGAAAACAATACAGTACTTTCTTGAAGGAAAC
5 SDHA	TGGCATAGTGGAACATGTGATTGA	GCTTGGTAACACATGCTGTATTAAACC
5 SDHA	GCCTTTCTGAGGCAGGGTTTA	CAGGTAGAAAGTGCGAGGCT
5 SDHA	GCGAATATCTTGACTCCTTTAAGGTGTT	CCTTTCCAAACTTGAGGCTCTGT
5 SDHA	GAAGATTTATCAGCGTGCATTTGGT	GCGGCCTTACCCTTCCATATA
5 SDHA	GGACTGGCCACTCGCTATTG	CCACGCTGCTGTTCTCTGTT
5 SDHA	CTTGGTATGGCTGCTTCTATGGAT	TCTTCAGGTGCTTTAGGTCTCCA

5 SDHA	TTTGTGCTTAACTTACCACTGACTCTT	ACAAAAGCTGATTCTGTCATCACCA
5 SDHA	CTGGGTACATAAGAAGGTGAACAGTTT	TGCCCTCTTGTTCCCATCAAC
5 SDHA	AACCCGAGGTTTTCACTTCACT	CAAACTGGTATAGATCCTTACCCCCTAA
5 SDHA	CTCACCAGATAGGAGGTCCAGAT	AACAAACTCTAGGTCCTGGCAAG
5 SDHA	GGCACGGCCATGATCAC	CATCACATGCTTACAAGACACACA
5 SDHA	TTTAGTCTCTGCGATATGATACCAGCTA	TCCTACCCTGTGGCAACAAC
5 SDHA	CATCGCATAAGAGCAAAGAACACT	CAAGGCCTGTGTCTAAACCGT
6 BAI3	GGGTTCATCTTCAAGGGTGGTT	GCTTCTCTGCTGAGTCCCATTC
6 BAI3	CCAGTGCTCAGTAACGTGCTC	AGTGCCCATGATATGCAGGAATC
6 BAI3	GGTAGATGACTGACATTGACCATGAAA	ACTTCTTCGCCCGTTCCTTC
6 BAI3	AGTGATAACAGGGCAGCAATGT	GAGGAATATGACCCTTAACCAATGACAAT
6 BAI3	GGGTCAATAGAGTTAATGCAGGTGA	GGAAACGTATGGTGTAGATACTTGCAAT
6 BAI3	GGGTGTGATGTATAGCTACGTAATGT	TGCAATGAATCAAACACAGCAAAAAGTA
6 BAI3	GGGATGAGAGCTATCTTGTTCTTTTT	CAGGCATTGAAGCAAAGCATACTTAC
6 BAI3	TTTGATTTTACTGCTCTGCTTTTGTTGT	GTGTGCATGACCTTGTTCTTGTTC
6 BAI3	TTTACATGTGGTCGAGGCCAAA	CATAACCAAATGCAGAATAGGCTCAC
6 BAI3	TGACAGATAAACGCTCCATATTGTTTCA	AGCAAGGAAATACCATTCCAACCAT
6 BAI3	CCTTGCCGCATGAAAGGCTA	CAGCTGTGCGAGGTTCAAAG
6 BAI3	CCAGGAACATATGCAGAATTTGCC	GGCTCACCTCTAAAGAACTCATTGATA
6 BAI3	CTATCAAGAAGTGAAACTGGATCAACGA	CCCACACCAGTGTCACatcaat
6 BAI3	CAGAGATTAACAATCCCCTGAAGAACA	GTCAAAACCCAACAGAATGAAGCC
6 BAI3	ACTGCATTTTTGCACTTTTTCTTCCT	TCATTTTGAGAAGAGCATGAAACGGTAT
6 BAI3	CAGATGAATGCCATCAGTTTGCAC	GATGAGATGATAGACAGGCAGAAGTTAA
6 BAI3	ACTTTTCTCTCTCTGGCTAGGTACAT	ACCTTATTATGTGTCTGAGTCTGTCCA
6 BAI3	CAATATCCTCATACTGGTTGGACAGA	TGTCAGAAGTTCTCCATTTTAGAATGAATGT
6 BAI3	ACACTACTGACATTCAAGTCCCTTTG	TTGGGTGGGTCATCCTACTGTA
6 BAI3	GTTGTTTGTATCACACGTTAGTGGTCTA	GTTGATTTTGATGTTTACCAGGACAACA
6 BAI3	GAAGGAGGACTACTCTATGCTTTTGTG	TTGCCACTCTAATTGAAAGAAAGTAGTCTT
6 BAI3	TGTGAGAAATGTCTACACACACAAGCTTT	CTTGTTCCTGAGGGACCCTTT
6 BAI3	GCGACCTCGATCTGTTCATGAAA	GCAAAAGATTATGAAAACATGCAGTGGT
6 BAI3	AACAAAACAATAAACTTGTACTGCCAA	GGCAATGTCTACATCCTTTTCAAAGTCT
6 BAI3	CGTATTTAAAAGCTAACTTGAAGCAGCA	AAACTACTCCACACTTGGCACAT

6 BAI3	TCATAGCGGTTTGACGCTCAA	GCATTTTCTCACATGCACTGTAAAAGAT
6 BAI3	CAGGGACCTGATTCCCTTCAAT	TCAAAATCCACCAATTGCAGCTTAC
6 BAI3	TCTTGCATGTCATAATGTCAGTTTGGTA	CGACCCTTGACCACAAGTAACC
6 BAI3	GTCCCAGTGGAGCACATGTT	ATCCACAGTTGTAGTTGCTGCT
6 BAI3	TGTACAGGAATTACAGTAGACGACTCT	AGTTTTACATCCCTGGGTGGAC
6 BAI3	GAACGAGTCTTTGGGAACGTG	AGCCGTGTTTTCCCTTTGTTACTTA
6 BAI3	TGTGAAGGAAATATGTATAAAACAAGCAGGA	CCTCAAAGTCAAGGTCTGAATATCGT
6 BAI3	GAGAAGAAAATCACGATATTCAGACCTTGA	ACCCTTACATGGCTGCATAGAGTA
6 BAI3	ATGACTCTCTTTCCCTCTCTTCTCAA	GACACTTCTCCCACTCTGCTG
6 BAI3	GCAAAGGATGCTTTGGAACTGA	AAATGTCAGGCTTCCCAAGTCTT
6 BAI3	ACTGCAAGATTTACCTTGATCAACACT	CCCTTCCGTCCTTTCATTGGAA
6 BAI3	GCCTCTGTTCTAACAGACATCAACT	CCTTTTGATGACACCGGAGTGA
6 BAI3	CTTCTCTCTGTTCTTTGTGACACAC	GTACTAAAGCTGGCTTTGCACCTA
6 BAI3	ACCACAAGATAAGAAATCTTCCATTCTG	ATCTGTCACTTACGTTTTGGAGTCAT
6 BAI3	GGGTACTTTGAATCCCTATTGTGTATTGT	CTGTTAACTAGCAGGCAGCTGAA
6 BAI3	TTTTGAGTGATAGGTTAGCTTTGCTGA	ACCACTGCCTACTATTAGGGTAACT
6 BAI3	GGAATCCTCTGGCACACCTTC	ACTAAGCATTGCATTTCAGGTCTATCAA
6 BAI3	TATCTTTGCCTAAGGAAGTACTGGAA	AGTTTTTGTATAGGACTGCGCCAA
6 BAI3	TCTGTATTTGTTCTTGGCGCAGT	CCCTTCATATTACCACAGCTTGTTTGA
6 BAI3	GCTAAATATGAATGCGTGACATTGCT	CCAACCAAGACTCTTTGATGATTTGTTA
6 BAI3	CATCCCAAAATGTATTGAGCAGTAGGA	GTTGTTTTGGGTTCAGGCCTTATTG
6 BAI3	GTCATTAATTCCAAAATCATCGTGGTCA	TGGTGTTATCAGTTGAAAGAATGACTGT
6 BAI3	CGAGTATTTCCAACTAATTTCCCAGGATT	CACATGATTCTGTTCTCCCATTTTCTG
6 BAI3	AGCTGGCTGTAATATTGAAAGGAGTT	CAAACATAACCAGGAGATAGGTGGAAAAT
6 BAI3	ACTTGGTTGGAGAGCTGCTTAAAAT	CCCTCTGTCTGCTCATTCAGG
6 BAI3	GGCTGTTCGTAACCTGCTGATTTATAT	CCAAGTGCAGTTTGTAAAGTTTTTAGGA
6 BAI3	AGGGAGTCATTTATGGATCGTATTCTGTA	AAACTGATAAGCCAGGAGTGAAAAGT
6 BAI3	CCAAAAAGGACCTTAGCTGCTCTA	CATTCTTGGAATTGCAGAGTTGCATTAT
6 BAI3	CGCTGATTTTGTTAAATAACGTGGTGTT	CTTCCCCTCTCTTTGGCACTTA
6 BAI3	ACTCCTGGCTTATCAGTTTGATCATTTT	GGGAAATTAGTTGGAAATACTCGACGTA
6 BAI3	CCAAGAATGCTTTCGTTTTTCTACAGT	GCTGACCTTGTTCAATACCAAAAACTC
6 BAI3	TGAAGATTCTGATGTTTGCTCTGTTCT	CAGAAGATCGCCTGCATAGAAATTTTT

6 BAI3	ATCTTTAATGTTTAGTAGAAAGCGAGACA	CTGCTCGGCAAGGACCAATA
6 BAI3	TGGTCCTTGCCGAGCAG	CTGGATGATGACTTTGGAAATGACATT
6 BAI3	CAAGACACTGTTGGATTTAACTCAGAGA	CCTTCACGAACTTGTCTCCCTTAC
6 BAI3	GCTAAGCTATTCAACATTGCCTGGA	CCCCTCTCAAATTATCATCCGTACATA
6 BAI3	TGAATTGCGGAGAACTGTGTACT	TCATTGAAGGCTGGTTATTTACAGAACT
6 BAI3	GAAAGTGACTATATTGTGATGCCCAGA	TGAATTCAGGGTTTACTTTGTAGTGCAA
6 BAI3	CGTAGTCAACCCAAATGGGTGTTA	GTTCTGAAATAACCCTAACCTTACCTGT
6 BAI3	CCTCAGAGCTGATTGCCAAGA	GATTTGAGCATGCCCATTAGGAAAC
6 BAI3	CCATCAATGCAGATTCTTCGAGTTC	ACTGAAGCCAAAGTCAATCAAAATGAAAA
6 DAXX	GGTACAACAATCTTCCCCGCTAA	CAGAAGCCCCTAGCTCCTCT
6 DAXX	CCGAACTACTGCTTCCTCTGG	ACCGCTAACAGCATCATCGT
6 DAXX	GCTGCTTCATCTTCGTCATCATCAT	AATGTGTGAGGTGAAGGGATGG
6 DAXX	CCCTCTTCCTCCTCCTCTTGTTA	GAGCAGGCATGGTCTCTTCTAC
6 DAXX	GAGACGCCTCCATTGAAGGAA	AAGCTTTGCCCCTGGATACC
6 DAXX	GGAAATGTCCGTCTCCACAGA	CCATCGTTACTGTCAGAAGAACCC
6 DAXX	GGAGAATGTTCCCTTGACATACCTG	ACGATGAGGAGAGTGATgaggaa
6 DAXX	ACATACCTGCTGCTGcttctt	CTGAGACAGATGACGAAGACGA
6 DAXX	GTTCTCCATTGCTTTCAGCATCTATG	AAGAGCCCCATGTCCTCACTA
6 DAXX	ttcctcATCACTCTCCTCATCGT	GGCAAGGGATTCCTTAGAGAAACTT
6 DAXX	TCCAGGTTCTTTTCATTGGAGATCTG	CCTAGTGTCCTCTCAGCAGTATTTTT
6 DAXX	GACAATGTCTCTCTGAAGGCTGT	GGATGAGGTCATCTCCAAATATGCAAT
6 DAXX	CCTCCTCACTTTTGTCTTGCAAC	GCTGAGTGCTCTGACTTTATGTCTTC
6 DAXX	TCCATCCCTTCCTCAGTCATCT	TTTGACTCTTTGTCTCTCACTTCCC
6 DAXX	ACACGCCCTCCCCTTCTTA	CTTCCCCCTTGGCTTCCTT
6 DAXX	CGTGGAGGAATCAGCAACTGG	TCCCGTACCCCTCCACATATTT
6 DAXX	GCCCAGCTCTCCCCAATA	GTGGGAGTGGGAAGGAAGG
6 DAXX	CATCCGCCTCATACCTGACAT	AGGATGCCTTCCGAGATGTG
6 DAXX	GAGACTGAGAGGCAGTGTTTTCA	GCCAAGCTCTATGTCTACATCAATGAG
6 DAXX	CGCTCCTGTAACCTGATGCC	GCATTGAGCGGCTCATCAAC
6 DAXX	GGCCTTGAGAACAGTGCAGA	GTGCCCACTCTCTGTTTTTGG
6 DAXX	CCCTAGAGAGGATGTTGCAGAAC	ttcccCTGAACCTTCTAGTTCCT
6 DAXX	CCCCATAGTCAGGGAAGGTATCAG	CCTCTTTGGGCGACTATGTGAG
6 DAXX	TGCTGTCTGCATCTTACAAAGTTCA	GATGGCTGACTGGTtctcct
6 DAXX	GGTCAGTGAAGAGCAGTCTTTCA	GCTGCAGGAAAAGGAGTTGGAT
6 DAXX	GTCTGGGTCATCCAATTCTGAGAG	TCTCCTTGGACCCCACAAATG

6 DEK	CTGCAGTTTACCTGTTTCTCCCA	GAAAAGTGCCAATGTTAAGAAAGCAGAT
6 DEK	ATTCTTCTTGGTGGTGCTGCT	GGGATGGGATAGCATTGAATCTCAT
6 DEK	GCAGAAATATTTAACTCTCCCCATGGAA	CAAATGTCCTGAAATTCTGTCAGATGAA
6 DEK	CTTCATCATCTGAAGACTCTTCCTTGT	GCAGCCATTGCCGAAATCTAAAA
6 DEK	TTCCTTGCCATTCCAGAACTGT	AAGAAATTGAGTTGATAAGTTTTCAAGAAGAG
6 DEK	agtgaatGCACGTAAGTATTTAGCTTCA	CTGGCCAGTGCTAACTTGG
6 DEK	TGCAAATCTGTTTCATTGTGACTTCTTC	AAGTGAGTCTGAGGATAGTTCAGATGAT
6 DEK	CACAAATTTAGGCACTTTCACCACAA	TTCTAGAAAAGAGTCTCATCGTGGA
6 DEK	TTTCTTTTCCCTCTTGCCTTCCA	TCCAAAACCATGCTCAGAAGAAGAATAA
6 DEK	ctggccCGAATGTGATTTAACAT	CCTCATTAAAGAAGAATGTGGGTCAGT
6 DEK	TCAAATGGAAAGCCACTGAACTGA	AAGATGTTGACTTTGAATTGGCTTTGAAT
6 DEK	ACGAATACTGACAGCAATGCTGTT	TGGAGAGATCAGGTGTAAATAGTGAACT
6 DEK	GGCTTTGGATGCATTAAGAAATTCAAGAT	ACACCTTGGTTTCTTCCATTTTCTGT
6 DEK	tcTCTGGGACCGGGCAT	CGAGACGTCCGGCTTTCTTC
6 DST	ATCAGGAAATCCAGGAAAATGTCTTTCT	GCCGACACCCTTTACAGTCAAATTAA
6 DST	CCCAGTATGTTTTGGTGCTGGAT	ACTGTGATGGTTCGTGTTGGAG
6 DST	TCATCAAGTGCCATCCATCCAC	GCAAATTGCACATCATCCTGTAGTTTA
6 DST	CAGCACGCTTTTTGACATCTTCT	AGATGAACAAAACTGGGCCACA
6 DST	GGATACTAACCTGTTGTCAATCTGCAC	GTTGCAGAAAGCTAAAGCCATCA
6 DST	CTGCTTTTTCTTTGTGCCAGTCAA	TGAAAAGAGACAGGTATTCCATGCC
6 DST	CCCAGGGCTCAATTCCAGTAAC	GACCCTGAGATACTGAAAAGCTGTTT
6 DST	GGCTTGTAATTTTCAACTTCTCAGCAA	GCAGAGCAGCTACTTCTAGAAAATCA
6 DST	CTCAATAGTTAACCGGGCTGGA	TGCATACTAGGGTTGCATTTTAGGTTTA
6 DST	AGGACAGAGAAAGAATAAGTAGCACTCT	GAGTGCAGTTTCAGAATGAAGCA
6 DST	GTTCTCACATTCAAGTATATACCCAGCAA	CCCAAGTAGGCTTCTGTTCCA
6 DST	GTCTTCCATAGGTGCACCCA	TGCAATGATGCAGTGGTTACAGAA
6 DST	GCCATTTTGTTGCAGTTTTGTTCATT	TCTTGGCCTCTTACTCAAGGACA
6 DST	CCAGAACACGGTATTCTAAAATGCCT	CCCCAGCCCTTGAATATGAAACTC
6 DST	GTTCTTCCTGCTGCTGCCTTA	GGTTAACCAATTCTGGGAAACATATGAA
6 DST	AGAGTTTAGTGTTCAGTTCCGCTATTT	TCCAAATGTCCTGAAAATGTGTCAGTAT
6 DST	GACTCCTGGAAAACAATGGAGATCA	GACAAGGCAGGTAGCTAAGTGTAAA
6 DST	CTCAACTTGAAATCGCTTTGCACA	CGATATCATGCACAATTCATACAACCAT

6 DST	TGTCAGCCATGGCCAAAGTT	CCTGACCAGTTAAACATTTCATCCTTT
6 DST	ACGCTCCTCATGGTTAAAATAGAATATATG	AGCCTGGAGGAGAGAATCATCAA
6 DST	AGTCTTGGTATTTACCTGTCTGTTGATG	CAAGCAGAGCTTTTGCTAAAGAAAGTAA
6 DST	CATTAATGGGTCTTGAACAGTGTGTTT	AGCAATTTAAGTCTGAGGCCTATCAAC
6 DST	TGCTTGATGATTCAGTCTTTCCATTTCTA	GTCACCTCCCCTCAGTAAGTAGT
6 DST	CCACAAATATGATTCATGGTCACCACA	GGAGCATGACCTGGATGATGTC
6 DST	GAGAGTCTTCCACCGTGCATT	TTGCAAGGTAAACAGCAAGATGTAAAC
6 DST	CTGAATAAGGCCTTGACCTAACCA	ACAACCTGATGCCTAAAGAAATGCTTA
6 DST	TCCATGCTATTTTGGTCTTGAATGCTA	ACGCTGGCAAAATGTTTTGGAAA
6 DST	CTGCTGCTTCCTTTGTTCTGTTT	GATGTATTAGCCCATCAGTCCACAG
6 DST	TTCCTGCTTTATTAACGGCTTCCA	TTTGAGGATGTTTCATAACCAGAAAGGA
6 DST	CAAAATAGGCAATCTCCTTTCTAAGCAAA	TCACAAGGTCCTGTTGGTATGG
6 DST	CTCCTGAGTAGTAAGGGAGGCATA	GAGGAGCTAAAAACCAAAGTAGAACTGT
6 DST	AGCTGCTGATTAATTGTCTCCGTTT	GTTACTGGACCGAGCCCAAG
6 DST	GGAGCTTTTCTGAGAGGTTCTCAA	CACTGCAAGCCAAAATGAAAGACT
6 DST	CCTTCAACCTGCTCTTCTCTGG	CAATGCCAATAAAACCTGCAAGATGAT
6 DST	GCTTCTGAAAACCGAGCAGACA	TGTTGGAACAGGATATTGCAGGTC
6 DST	GCTCTACTCAGAAATAAGGCCAAGT	GAAAGAAAAATGGGAATCGGTGGAA
6 DST	TTCATTCATGGCATTTATACTGCTCTGA	CACAGGTTGTGCAAGCTTATGT
6 DST	CACATACTTTCCTTTCATTGAGTTTGGT	CTGCCTTTGAAGCTAAAGAAGAAACATA
6 DST	GGAGAGGTTTCTTCTGTGGCTAAC	GATGATGAACTGGATAGCATGGCT
6 DST	CCCCCAAAACTAGCTCAACATTTT	GAAGGTCATTAGTCCTACTGGGAATG
6 DST	GGCCTTTCTGCATCAGACTCTTA	GCTTGGGACTGATGACCTAGAAAAAT
6 DST	ACACAGATGGGACCATAGCCT	GTGACCTCATTGAGTTATTCTGCTCTTT
6 DST	TTTCTGCATCTCTCCCCACTG	ACTAAGTCAGCAGGTTGATGAAAAGT
6 DST	GGTGGTGCCAATATGTAGACATCAAT	CAGCATGGGATTCTCTAAATAAAGCTTG
6 DST	CAAGTTTGTCAATCCGGTCTTTCC	ACTGGCTTACTTATCAGAATGGCTTTT
6 DST	GCCATTCTGATAAGTAAGCCAGTTGT	ACAGGAGGAGCTGGATATAGTTATTAACC
6 DST	ACATGCCGCAATGAGTTCAGA	GTGGTTTTGCTTAAAATTCTAAGGCTGT
6 DST	TCTTTCAGCGGCTTCCTTCTC	GTAGAGGAAGAACTTCCGAAGGTC
6 DST	ATTCATTTTCTGCAGCCTCCCT	ACAGTGCTTGAGAATAGCAAACTTACA

6 DST	ACCATTCTTTCCAACTCACTTATCTTTCC	ACAGAACAGGCTTGACACTTTCA
6 DST	TGTCATGTTCTTGCTGGAGACC	AGGTAGCAGAGAAACAGTTGAAAGAAA
6 DST	TTCTTGCTTCCAATTCAATTTTCTGCT	AGAAAGAGAGACAATGAGGAAGAACTCT
6 DST	GTCTTTTTCCATCTGCTTTATCAGCTT	CAATCAGTTGGAGGAAAACACCTT
6 DST	TGATCTTCCAGTGTTCGTCTGGTA	AGGGAACTTGAAACCATTGTGAGA
6 DST	GAGCTTGTTGTAGCTGAACTTTCTG	CCTGGCGAAAAGTCAAAATTTGGT
6 DST	ACATTGGTCTCAGTAAGTCAAATTGCT	TGTGGAAAAAGAACTTATGGTCAGTGTT
6 DST	TGAAACGCAATACAAAAGCATGACTAAC	ACTGTGGGAGGTTCCAGGAT
6 DST	GCTGCCTTTGTGTGTTTAGCATAT	ACAACAAAAACTGGAAGAATCCTCCAA
6 DST	GGAACTGGGTTAGATTATTGGAGGATTC	ATCATACCATACAGTCCTCATAGACATAGTC
6 DST	GTTGGTCACACTTTTGCTTAAATTCACT	GCACTGAAAATTCAGGCAGATGG
6 DST	CTCCTCAGTGTCCACCATCC	GTGCTCAGATTGCATTATTTTGTGAAAC
6 DST	GTGCTGCGTAGCTGATCTTTAAAA	AGCTTACTAGGCAGAATAATGAAACCAAA
6 DST	ATTCTGCCTGAATTCTCTGCATCATTA	CCAAAGGCTCAATGAAGAATTGGA
6 DST	AGCATCTCTGCACACTCATTACTTTT	ACAAATCTGCCAGAGAAAATCAGATCAT
6 DST	GCTCATGGCTTTTCTCAAATTGTTCTTTT	ACAAAGAGAAGTAGACAGAATCACAAG
6 DST	ACATAGATTTCCAGAGCCATGCAA	AGTGAATCTCTGAAACAGCTACAAGAAT
6 DST	CTACCTTTACATCTCCTGAGGTTTGTG	CTTGAGGACGACTTGCATTAGTCTT
6 DST	GCCTAATACAGCCTGTGATAAAAGTACA	GATGGGAAAAAGTGGTTCAACGG
6 DST	AGACAGGGATCCCCATGCTTTA	GAAGATAGCCAGGAATCCCAAGTC
6 DST	CCAGTTGTGTTATATCTGAGCCTGAAAA	AATGAATTACATGGGTCTTTGGTCTTCT
6 DST	ATCCAAAGATCTTCCTCTCTCTACCAA	AATGAAGTAAATTCTCATCGTGAGCAGA
6 DST	GGTTCCAGTTTTGTCCAGCTCTATTA	CAGGTTTATGACACCAGAAGCATTTTT
6 DST	AGCCCTATGCTTATTCTCAAGTTTTCAA	TGTCAAAGAAATTCAAGGAAATGGAGGA
6 DST	GGACCAAAGCCTTATCACTTGGTA	GAATGCCATTTGAAGATCTAGCAACA
6 DST	AGAAATTGGAAGCCTTTCAGTAAGACT	ATGTGGACTTCTTGCTGGACTC
6 DST	GATAAGTGTTTGCTCGCTGTGG	ATCTCCTACCCCTTTTCTCTTTTGATTG
6 DST	TGACATAGCAATAGTGCACCACAT	GAAGATAAGGCTTTGATTGAACAGAAACT
6 DST	AGCTGTTCACACTTTATCTTAGCTTCAT	CAAGGAAGTGCACAGGCATT
6 DST	TCTGTGTTTTTCACTACTTCAGCCA	ACTCTCTCTGTACTCACTCTGTCTTTT
6 DST	TGTCACTTTCACTTTGTATTTCTGAGGT	CCACCCCTGCAAAGGCAATA

6 DST	ACTGTTGTTTACTTTCTGAATCTCTGGT	CCCTCACAAATCAGGGTCTACAATTATT
6 DST	GAAAAAGATTCCTGCAAGTTAGCCAAA	ATCTTACAGCTCTGCCAGTGTG
6 DST	GTTCTCCTTTATGTGCTGCTCCA	GCCTTATTTCTCCCCAGTCAGTTAT
6 DST	TCTGCAGAATTTTAAACTCGCCTCT	AGGAAATCACCTGCCAGCAAA
6 DST	CACTATCTCTTTGAGGACTTGTCCAA	TTGACATTTCAGAAATCCAGTCCGT
6 DST	GACAGTTTCCACATCTGAGCAC	TGAGCTGTCTCTACCTTCTTTCCT
6 DST	CCAATTGGACGGTGAGTAAACC	AGCCGTTGTATGAAACTCTTAAACAGA
6 DST	CCAGATCTAGCAATCATTTCCTCTCC	TCTGCAGAGGTTGAGAAGATCAA
6 DST	GCTTTTCCATGTCTACTGACACATTCT	TTGTTTTGCCCCTAGTTCCATGA
6 DST	CAGGCTCTCAAGGATCTGATCTATCT	TAAAGAGGCTAAGATTTATTCTTAATTCTGACA
6 DST	TTCATAGGCATTCATCTAAGTAACGCA	GTTAGTCGTTTGAGGCAGAACTGA
6 DST	AACTCCTGTACTTTGTTTACATTCTGCT	ACCCACCACAACATTTAACAGTAGATTT
6 DST	GCCAAATATAGATAAGGGAAACAGAAGGT	CAGATTCCTGACTGGAGTGTCTTTT
6 DST	GTAAAATCACCAATTGCCCCCAA	CCTTCCTCATTACAATCCCATATTCCA
6 DST	CTGCTCGTCCCTTATCCAAGAC	TCATTTCTTTCATTTCAGACCTTCATGGA
6 DST	GCTGTTTTCTGGTCATTTCCTCCAT	ACATGGTTCTTTTATCCTTCTGCTTTTAGT
6 DST	GAGGAAAAGTCTTCATCCTGAGTCT	GGGTATGGCTGCTTTCCGA
6 DST	TGATGATGGCCGGGATCTTC	AACGATTGAGCGCCTCTTCTT
6 DST	CAGGTGTTTAAGTATTTCTCAGGAAGGA	GTGTCCTCCCAGATGATGAGG
6 DST	TCAATGAGAGTCCGGAGAGCAT	GGTGTGTGTGTGTGTGAACTCTTA
6 DST	ACTGGTCTGTTTCTAGGAGTTACAAGT	agaagaACTTCTGCAGTACAAAAGCA
6 DST	AATGGTTTACATTTCCTGTACACTCAGAA	CCAGCCTGTTCATGGAGACAT
6 DST	TCGATCAGATTCATCACCAAATCAATGT	TGGAGGAAGCCCTGTTATTTTCTG
6 DST	CCTGTAGGGCATCTGTGAATTGTC	GCTCTTCCCCTTGAGTACCACT
6 DST	ACAAGCACATTTTATAGAGTTGGAGCA	GGTGACATCAGGCTTGAGCAA
6 DST	GAAGCTGAGCAGAAGTCTGGT	CGGGAACGTGCTGACCTAATTAT
6 DST	CACCTCTGTTTTGCTGAAAGCTTTTA	TGAGGAAATATCAACCAAGAAGGAACAA
6 DST	GCTGTATAGTTTGAAGTGCTTCCGATAA	ACTTGGGTCTTGACTAAAGCTTGATTAA
6 DST	TCATCTTTGTTCAGGTAAAAGCTCCAA	CTACCGATTAGTGAGCGACACC
6 DST	CCCACTACCAGTCCACAGACA	CCATGGAGATTTTGAGACACAAGGATATT
6 DST	ATCTCCTCCACCTTCTGAGTGAT	GAAGTGAGCAGTGCTTTGCTG
6 DST	TGCCCAGATTTAACAAGGTCATCAAT	GACCCCTCTAGTGTAACGTGATATTTTT

6 DST	GCCCTCCATGGTACCAGTTC	GAAAAGTCTGTTGAAGCTCTTAAGTGTT
6 DST	TTTTTACCTGCATCTCATCAACCCA	GAGTAGAGCCAAATTCATTGCAAACT
6 DST	TTACCAACCTGGAAATAGAATTGAACGA	GTGATGGCAAGTCAGCAATTGG
6 DST	CAGGTTACCATTAACTTCATCCTCTTCT	CCTTTTGGACTGGTTGTCAAATGTTG
6 DST	GGGTTTTCGGATCTGCATCAACT	GCCTGACTCCATCTGTCACTC
6 DST	CCTGCCCTTTCTGAGTCTTTGT	GAATTCCTCCTGTGAACTTTCCCTT
6 DST	TCCTGATGGGAAACCAGGTGTATAA	GTTTAAACTCAGGATTTGCACAGAC
6 DST	CCTGAGGTCAGACTAGGGTGTAAG	CAGGGTTGCAAAACTGCGT
6 DST	AACATTGTGCTAGGTAAACTGTGTCA	AAAGATGCAGAGAAGGCTGGAAA
6 DST	CCTTTTGCTTAGAGAAGGGAGGT	TTTCTTACCGCTCACAACTGTCA
6 DST	GGTCTCATTTTCCCATGGCCATA	CGACTCCATCACTACCATTAAGCAC
6 DST	CGCCCGGATGATTGTTATCCA	GCCAATGCCAACAGTTAATTGAAGTC
6 DST	GCATCACCCCTTGCACAGTT	CCCTCAGACAAAAAGAAAGTTGACAT
6 DST	GATAACAATGATTCACCTTAGTGTCTTCCA	GTCAAGAACAGTTGGATGCTTTCC
6 DST	TTTCCTAAATCCTCTGCACCAAAAGA	AAGCTGTAACTTCTTGTCAAGAACAGT
6 DST	GATTTAACAAGAACTTGGAAAGCATCCA	TGGTTTCTTAACATTGTGTGACATGAGA
6 DST	TGTGTGGAAATCAAAATCAGCTTTTTCT	CAATAGAAGAGGCTCTCCAAGTAGGTAT
6 DST	AGTTTTGTGGCAATGAGGACATCTAT	TGGTGGAAGCTGTGAATGCAAATATTA
6 DST	AGTTACTGCTCATCTCTAGCTCCTTT	AGTTCATGGACCTAAGGACTCGATA
6 DST	TGAGAGTGACCAGGGCAGTA	GCTGTCTGAAGGCCTAGTTAATGTAATT
6 DST	CAAACATCGGATTCCCATTTCCTTATT	GCCGAAGCTTTGCATAGAGG
6 DST	TGGGCAAACCCCTCATCAAC	TCTGTACTAAAAGCCTCCCGTAGAA
6 DST	CAGGGTCTTCATGTAAAAAGCAAAACA	CCAAGTGTTGAAAGCCATTTAGAAAATC
6 DST	CCTCACTGATTTTAGCTTCTTTGAGACT	GTACAACTGGACCGCACTGA
6 DST	GCTTTCAACACTTGGCAAATCTGAG	TGTTCAGGATCTTTTGAATTGGGTTGAT
6 DST	GTCCAGTTGTACCTTCAGACAGTAAA	ATGAAATTTGTTCAGGATCTTTTGAATTGG
6 DST	AGTAAGTGTTCTTTCTTTTATGCCCCTT	GGGCCATGGTAGATTCACAACA
6 DST	TCGGCGTTTCACTGGAGATTTT	TGAAACTCTAGTACTTTGCTTCAATTCAT
6 DST	CCTGGATTATTGGCAACATCTTGGA	ACTGGTTAAAGAAGAGTACTTGAAAGCA
6 DST	CCACACTCATATTCAGTAAATGTCTCTT	GCCTTTAAACTCTACTGCTCTTCAGG
6 DST	CTGAAGAGCAGTAGAGTTTAAAGGCA	TGTCCAAGTCTGTTAATGAACGAAATGA
6 DST	GATGCCTTCTAGTTGCCTACTAAGTT	GCTATGAACCAACAGTTGGAAACAG

6 DST	CGGGTCAAGGTTATCTGGAGTTTT	CCTTCATGTGTTTTTGAAAAGGGATAGG
6 DST	CCTGCTGTATCTCCTGCTTCATTTTT	TGACCAAGCCATTGTTAAAAGCAC
6 DST	CTTCTCAGCAGGCTTTGATACTGT	AAGACCCTTCTTTACGTGGGATTG
6 DST	AGCTGCCAGTTGCTCTTTCA	GCGTTTGTTTACCCTTGTTACCTTAG
6 DST	GGGAGACAGCATGGGAAAGATT	CCTGTTGAACTAATTGTGTCTTTCTTCC
6 DST	TTAAAACTGCAAGGGAGTCTTCGAT	GTGGGCGAATGTGTCTTTCAG
6 DST	aaaaGCCTGGTGTCTGAAAAGAGA	CAGGCTGAACAGACCCTAAATGTAG
6 DST	CTCACCTCACACTAGACTGGCTA	CATGAAAGTGTGTTCAGAATCACAGAAC
6 DST	CCCAACATATTTATAGCTCTCACCTCCT	TCGAAACATTAGACTTCGGTTAGAGAAC
6 DST	AAGATGAGACTTGGCCGAGAAG	AGGCCCTTAAACATCACATACTAAACAA
6 DST	CTGTCTAATCAGCCGATCTTCACA	GAAGCTTCTAAGAGATAGAAAACAAGGT
6 DST	AAAAGGGACTTTAATCCTCAGATGACC	GCAAGCTTCGACTTCCAGGATATTT
6 DST	GAGTGGAAGCCTTTCCCTGAT	GTCAGTGGAATATCATGGTACAGGAG
6 DST	GTAGATTCTAGGTAAAGCCCTGCAA	GGGCTTAGGCACACTGTGAC
6 DST	CCAGAAGCTTAGCTTCCACCAA	GCATCTGGTGGACTCAAGAAAGG
6 DST	CTGGTTCTAAGCCCTTCTTAAGGAAAT	CCTGTTACAGATAGCTAAGAACAAGCA
6 DST	CCATTCTTCAGCACTGGGAACT	TGTTTGGATTGAGAATGGATAAAGTGACA
6 DST	GTACAGCAATTCATCGAAATCATCATAAACA	GGCTATGGAAGCAGCAGTCT
6 DST	aataCCCGAAGTTCAGACTGCTG	AGCAGGCCTTATGTAATGCTAAGATTT
6 DST	ATCAGTTCAACTTCATCTTCCCCAA	TTGAGCTCCAGGGAGGATAAAGA
6 DST	GAGAGAAGTCTAATTTACTCTGCACCAT	GCTTAGGCCTTAGAAGATGACATCA
6 DST	GCCTGGTGTAAATGTTTATTGTGATTGA	GCAAACTAAATCCATTTAACATGTCATGCt
6 DST	GACACATATGAAAAGGCATGCACAT	TGGCATGAGTCTCACATAAACATGAA
6 DST	GATGAGATAATGCCAGGATACTACACTC	GCAGTGTGTTTTCAGAGGGAGTAAAA
6 DST	ACAGGTTCACACTGGACCTACA	GGAAATCGCAAATGATCCAGACAAAATA
6 DST	ACCTTATGTTGTGCAAGTTGTGTTTT	CTAGTTCCATGAAGCTTGGAGTAAACT
6 DST	TTTCTGACTCTTCTAGCCACTCCATA	CTGTCTTAAAACTGTGCTAGATCTCAAGT
6 DST	ACAGGAAGCAAGGACATGAATATCTTAC	CAGGTTGCTGTAGAGAAACTGAAGAA
6 DST	AAGTAAAGATCCCCTGGCATCTAAAAG	CCTGCTGAGTATAATTGAAAAGCGTTT
6 DST	GTGCATGATATCGTGGCAGAAC	AGAGATGGCGATGGATATATTGACTACT
6 DST	GGTGAAGGGCTGCTACAAATTCAT	TGCAATTGAGAATGTCTTGAGAAAGAAC

6 DST	TTCAATATAACTACTGTTTGCCTCTAAGATTTT	GGAACAGCCACAAATACTGAGGAAT
6 DST	CCTCAGTATTTGTGGCTGTTCCT	GGGTTTGATTTAATGCCATCTACTGTTA
6 DST	ACAAAACCACACAATAAAAACTGAACCT	GAAAAGAGGCTGGTGAATTGTGAAC
6 DST	AAGTTTAGATGCTTGGGTTCCTATGG	TCCCTAAATGACCTTTTGGCACATT
6 DST	GAGGAATGAGGGCAAATGACAATG	ACAGACACAAATTGAAACCAAAAATCCT
6 DST	ATTTGCTCACCAGTAAGTTTACCCT	GAAACTGCCATCCTAACCGAAGT
6 DST	CACCCAGCTCTCATTTACATGGT	CAGCCCGAGAACTCATAGAAGG
6 DST	AGGAGGAGTCATCCCGACTG	AGTATGTGTTGCTGTTTTGGTTTTGTAA
6 DST	TGTTTTGAGAAGTACAGCAGTGAAAGAA	GATAAAGAAGTCATCCCCCAGGAG
6 DST	AATGAGTGCTTTCACCTCTTCGAT	CCTGGGCAAAGCAACATCAG
6 DST	GCCAGAGCACTTGCTAATCTCT	CAGAAGCATTATGGGTCTAGCTCA
6 DST	ACCTAAAATGCAACCCTAGTATGCAA	GTGCGACTAATGAACTTATTTGGTTGAA
6 DST	CTCTCACTCCAGTCATAAGCAACTT	AGAATACATCCAGGAATCAAGAACGG
6 DST	GCACGACTTACAAAATTATGGAGTGTAT	AGTCAGTATGCAAAACTCTTGGTAAGTT
6 DST	GAATTAGTGGGAAATATCAAGAGTGCCA	AGATCAAGCAGCATTCACAAGCTA
6 DST	ACCATTGATTCCTGAACAAGGTCTTC	ACTTTAACTTCCAGGTTTCATCTTTCCTT
6 DST	TCTTGTCAGCTATAACTGCTTCTTCTTC	AACATTTAACCATAGGTGGCTATTGTTT
6 DST	GCTGTGTTGTTCAGTAATTCTACCATAC	GTAGAGACTCTCTGGCCATGGATA
6 DST	TCCAGGTTGTTTTGGCATTTGTC	GCAGCCTTACAGTTACAGCTTAATACAAT
6 DST	CCTGAAGCTTGGTTTCTAAGAAAGAAC	GCTAAAAGACTTGCACAGGACCTT
6 DST	ACCTGCTTATTAAATGTATCCCAATTGGT	CTTTGACCGCTCAGTCTCATATGTAT
6 DST	AGATTTTCACCTTCTGCAATGGTTTTT	GGCTGGATACAAAGAAAGAAGAGCA
6 DST	GTTTGGCAGATATTGGATGAGATTTGTT	TCTAGAGAATCATGTCCAGCACCT
6 DST	TTGCACAGGCCGACTGAA	AGTCAATGCTAAAAGCCCTTGCT
6 DST	TGAGTCTGAGGCCTCTACCAC	TGTGCAAAGGAGCAGCTAGAT
6 DST	GGATCCCAGCGAATCATGGAT	GGTGGACATGGTCACTGAACA
6 DST	GTAACACAAGTTTACCAGACCCACA	GAGAATTGATAAAGACCAGGATGGGAAA
6 DST	CATCAATAAATTCCTGCCGCGTTAT	TCCTTTTAGCTGAGGGAATTTGCT
6 DST	TTTTGCGCCAGATATCAAAATCAAAGTT	TGAAATGGGAAGGATATGTGTACTACCT
6 DST	GTATCGCCAGGGCCCATAT	CATCTAAACCGCTGGGAGGTT
6 DST	CTCCAGACAGAATTTCTTACTGTGAAGT	CAGAAGGAAATGGACCAACACTTTG
6 DST	CTCCTTGGCTGTTTCCGGTA	CCCAACTTAAAACTGCCTATTACTTCAG
6 DST	ATGCTGACCACATGATGATTTGATTTG	CCCACATAAAAATGGTACCTGTGTTTT

6 DST	ATTAGGTAGCTGATAAGGTCTCAGAACA	AGAAGATAAGAGACCAAGCCCTGA
6 DST	CTGAATATTCTTCTCAGCTACAGCATGT	TGAATCAAACCACAGGTAAAGCTGAA
6 DST	AGGTTTTCAACCTCACGCTTCT	CCAAGAAAATGCTAAGCTTTGTGAAACA
6 DST	AGAGGTCTACAGAGTAACCAAACTGT	GACTGGACGTTCTCTGAAGGAG
6 DST	TGATACCTAACAGGTGGTCTAGAATTGT	GGGCTCCACTCGAGAAAGAGA
6 DST	ATCACTAGCTTCTTGCATGGCTT	CCTTAAGTTGGAGCTGGAAAGGA
6 DST	GTCATCAGCCAGGGAGGTTTT	CCTCTTACCTGTTTATTAGTCCCAAGA
6 DST	CTGTTGAAGCTGTCTTTCAAGTTCTTT	ATGGAGTCCAAAGTCATCACTGAAAAT
6 DST	ACAAAGTCAAGCCTAATGCCTGAAATAT	CCATAGAGGAGCAGATGACCCA
6 DST	CTCAGAGTAACACTGCTGGCTTT	ATCCCCTCTGTTGAGATGGACT
6 DST	GAATACCCTTTCTCCGCCTGAT	TGAACAAGACATGAGAGAGCTGAC
6 DST	ATTCTTCCTGAAACAGAACCATCTTTCT	GCGGAACTGAATGCTTCCAAA
6 DST	TCTTCCAATGGCTGTGGTTCTT	CACAGCCAAAGACTGTACCTTC
6 DST	CAAGCTGGAGGGCATTAAGTTCATAT	GCTACATCATGCAGGGCAGTAAC
6 DST	CCTTCACTGTCATCTCAAAATCTGGTTT	AAACTTGAGGATGAGCTGATAGCC
6 DST	CACTGAGTGAAATGACTTGCATGG	TGTCCGAAATAGAAATGAAACAGAGCAA
6 DST	CTGCATATTTTTGACACTCGTCCATT	GCTGTACTGTGAAGTGATGTAGATATGT
6 DST	CATATATTTAGGGAGTGGTGCAACGA	GTCAACTCCTTGTAAAGCAGCAG
6 DST	CACGGGAAAGTCTGAGCACT	CCTTTTCATTTGATTTGCCATTGTTCCA
6 DST	ACACAATCCCACACCAATGATTCA	CGTAGCAGTGATTCTGTGTTTAGTAACA
6 DST	GACTGGTTCGAGTGCTGGTAG	CACCGTTAGAAGTAGAGCTTTTTCCA
6 DST	CGTCTTGGGTATGCCTTGCTTCTA	TGGCACAGTTGAGTTAAAGAAATATCAGT
6 DST	GAAAACCAAATTCTACCTCTTGCTTGG	AGGGATATGTGATCTTCTTACCCAAACT
6 DST	GGTGACCAATTAGGCGGTTTTC	GCGATTTTGTGACCACCATACATTTAA
6 DST	TGTAAGTGGAAGACATAGAATAGACTTGGT	GAACGACTTAAAGATGATTTGGGAACAA
6 DST	GCTTGACTGAAAAACTCCTCACACTTAT	ATCACAGGTGATGGCCTGATAC
6 DST	GTGGAGAGTATTACGAAGAATCAACCA	AGTGTCATCTGTGAAGACAAACTGAT
6 DST	ACTGAAGAGCATTTCCAGCAAGA	TGGTGAGCTCTTTGACTTCAAAGG
6 DST	CCAAGAAATCTGATGACACTAGAAATGGA	CTAAGTGAGCAGAAACCTGTTGGA
6 DST	CAATTTCAATGGCTTTAGGGTCTCC	GCATAAACTGGAGGGTGCTCTAT
6 DST	GTTGGAACTGACCCAAGGCTA	GGCTTCACTGGATACGTTTATGGA
6 DST	TTCATGTTCTTTTGCAGTTTCTCTTTTT	CCCCGTTACTGCTTGAAGATTATGAT

6 DST	GTTTCTTGCCTCTTGTTCCAAAACT	CGGTGGCAAGGTTGATACTTCTG
6 DST	CTGCACTTCTCTGTGGGTTG	ACCAAATTGAAGAGGCAGAAGAGTTT
6 DST	CAGTTTTGTAGACAGAGGCCACT	AGCCCGCAGATAAAGTGAAGATTTT
6 DST	ACCTTTGTGAGAAATAACGTCTTCTGA	GCAGGAAGAATTAGAAAAGTTTGATGCT
6 DST	GCTATCCAAGAGACTGAGCTGTTTC	GTTTTGTCTCAAGCTTCTCCAGAGAT
6 DST	CCGTAGATTTTCGGTCATCCAACA	TCAGATCCTTTCATATGTGCATACATAC
6 DST	CCAGTGCTCAAACTCGGTATAGT	AGGGTCAGTATCTCTCTCCTGAAG
6 DST	ATTGGAAATGTTCCTCTCCCCAAA	GTTTTTACATGAGCCATCCAGCAA
6 DST	GGGCAGTAATCCGATCAACCAAAT	CATCACACTTACAGAACTTGCTGATTC
6 DST	GCTTGTTATTGGGATTAGGGAAAACTCT	GTAAACATATCTTGGAAGCCCAGATTG
6 DST	CTAATCCAAAATGCAGAAATCAGAGCTT	CTGCACTCCACACACGAAGA
6 DST	CCTCTCACAGGGTCAATGACAC	GAATTAGGCTTCTTGAGGCAGAGA
6 DST	GGACTAACCGGCTCAGCAAA	GCATTCTTCTCATATGCTGACTGATACT
6 DST	ACTTTGTCCAGCCAGGTACAC	GAAGCCATTAAAGCAAGGTACAAAGA
6 DST	CCACATCAGTGCTCAGTTTAGTAATG	CCATAATATCTGAAGAAACCCTCTCCCT
6 DST	GAATAAGAATATCCCACAGCTGCCT	ACAAAATGGTGGCTTTGGCATT
6 DST	TGTTCCCTGCTCTATAGCCTCATTAATA	GCTTTCCAGAGAAACCTGATTGAGAAA
6 DST	TGTTGCAGCCTGAGCTTCTAAA	TTTCTGACGAAAGCCACCTCAA
6 DST	GCTGCCCTGAAAGTTCAAGATATATACT	AGTCCCAATGCTTTCTGTTTCCA
6 DST	ACATAGGAAAGGAAGATGAGGAGCAT	GCAGGTTGAAACTACTCAGAGAAAGATTC
6 DST	GGTTTTACTATTTTCAGGCTGATTTTCCT	CGCTGGACACCGTGACTATTC
6 DST	CCTAACATTTACCCAAGGAATCTGGAT	GAAGATCCTGGAATTGATCCTTCAGTAG
6 DST	CTGCTGCTTCTTGCTGTTGTTTTA	GGGAAGCCAAACTACTGGATGTG
6 DST	CCAGAACTTTTCTGCTAGCTCCAT	GCCTTGTGGATATTGTTTTGCTTCATAG
6 EPHA7	GCTCAATTTGCTTCATTTCTCCTGTT	ATTTTGGCCTGTCCCGAGTTATAG
6 EPHA7	TGTATAGACAGCTTCTGGATCATCCT	GCTGGAATGAGATATTTGGCTGATATGG
6 EPHA7	TGCAAGGTCCCTGTGAACATATC	AGTTAACTGGGCTTTTATTTTTGTCTTC
6 EPHA7	TGAGGACTTTGTTAATGCCAATGCT	TGAGATTGTCACAAATTTGCTTTCTTCAA
6 EPHA7	CAATATCTACCTCTTGTAACAACCCCTT	CAGTAGCCATAAAAACCCTGAAAGTT
6 EPHA7	AGTCTCTCCTTTGTTTTTCTGTGTAACC	TGCCTGCTTGTTCTGTTACTTCA
6 EPHA7	GCCTCACTGCTTCCAACAGTT	CCTCCTGTATTAAAATTGAGCGTGTGAT
6 EPHA7	CTACAATAGCCTTACCTGCACCA	TCATCACAGTCATGCTTTCTTACATCAT

6 EPHA7	GCTTACTCAATAGTCATCCTGGCT	ACACTCCTGATTTCACTACCTTTTGTTC
6 EPHA7	CTTAATAGCTTGTAGCCATTCTCCAACT	TCTTTGGTATTTCAGGATAATGTTCGCT
6 EPHA7	CCTTGCAAGTTCCCAGTACTCC	ACATACCAGGTGTGCCAAGTC
6 EPHA7	GTTGTTTTGGTTGGGCTCCAT	AGTACTTGCACTTTCAAAAGCAAAATGT
6 EPHA7	TGAATTGCGCTTCTGGTACAGA	CCATTGGAAAATGTATCTGCAAAGCA
6 EPHA7	TGTCTCCTTTTTGCTGGTAGCC	AGTGACTGGTTCAGAATTTTCCTCTT
6 EPHA7	ATGTCCCTCGAACCTCGACTA	CTTGCATAGCTTTGGTTTCTGTCAAA
6 EPHA7	GGACCAGCACTTCTTGTAGTACAC	CCTTGGTGAAAGAAAGATGAAGCTTAAC
6 EPHA7	GGTCCAATCTCTCTCACCTCAGT	CTGGCAGGAATATAAGAGAAAACCTCT
6 EPHA7	CTGGCTTGTGCAGGTAGACAT	AAACTCGGTACCCTTCATGGATTATTT
6 EPHA7	CTGCAGCAATGGTGTCTATTTTTACAT	CCTGAGGGATTGTAACAGTCTTCCT
6 EPHA7	GGAGCAGCCAGATGTAGCATA	ACAAACAGCATTTAAAAATCCACGACTC
6 EPHA7	AGTAGGAATCCAAACCAAAGGCATAA	GAAACCAGGAACAGTGTATGTTTTCC
6 EPHA7	GCAGCAGTAAAAGCCCGAATCT	GTCAAATTGTCTGGTTGCAATCACAA
6 EPHA7	ACAAGGCTCTCATTTTACATTGCAAAAA	TTCATGGAAAATGGAGCCCTAGATG
6 EPHA7	ATCACTTTGGTACTTACCCTGAGAAATG	CCTCCAGCTCAGGTTACGACT
6 EPHA7	AGACACCTCACTTAGGTAGTCACAT	GCTAGCCCACGCTAATTATACTTTTG
6 EPHA7	TCAGAAACTCCATTTACAGCTTCAACTT	AGAATATTGTGTAAGCGGTGCAGTT
6 EPHA7	GTTACTCCCACAGGGAACACATT	CATCTGCACCACAGAACCTCAT
6 EPHA7	CTTACTGTGGTTTGGTTGATGTTGAAA	GCCCCACACAGATCCTtttgt
6 EPHA7	CTTAATGAGCCTTTACCTACATGTTCCA	TCATGTGGGAAGTTATGTCTTATGGAGA
6 EPHA7	TTGATTTGACATGTCCCAATAAGGTCTT	ATTCCAGTAAGGTGGACAGCAC
6 EPHA7	ATTTCCGGTACTGGATGGCTTC	GTAGACCTGATGCTGCTGAACT
6 EPHA7	ATGACATGAGACACACAGCACAT	CGCAAGTGAGTGGAGTAATGAAGG
6 EPHA7	ACTCCGCTGCAGTACTCTCT	ACCTTAATGAAACTGGCACAGCAT
6 EPHA7	AGTGTGAATAATTACCCTGGAGGGA	CAGTACTACTGCTGGATTCTAAAGCA
6 EPHA7	GGAAATCCACTCCAACTCTGTTTGTT	TATGAAAAGATTCACCTTAAAAATGTCTACATG
6 EPHA7	ACAACTTTGTGTTCTACCTTCTCCC	GCTACAGCTGTCTCCAGTGAAC
6 EPHA7	CAACCACAGCAATGATAATAACAGGATT	ACTCTAATTTTATTTAACCTTTCAAGTTCTTGC
6 EPHA7	TGTCACAACAGTACAAATGAGTGGAA	CCAGATGTGAATGTGAAGATGGGT
6 EPHA7	GGTCAGATGGAGCCCTGTAAT	GCTCTCATGTCTCAATTTTTGCTTTCTT
6 EPHA7	CCAGATTCCATTCCCTTAGGCATT	CCAGCTGGCCTTCACCAG

6 EPHA7	TCCTTTTGCCAACAATCCAACATTAG	CATTTTGCCTACCAGGAAATTTGGTT
6 EPHA7	TGCCAGTTCCATGTAAATGTAGCA	GACAGTTTAGTGCCACATTTATTCTTTGT
6 EPHA7	GAAGGACCCAGGACATCACTTG	AGCATTCAGACTATGAGAGCACAAA
6 ESR1	CCTAGACCTCATCCTCTTTGAGCTT	TCAGGTGGATCAAAGTGTCTGTG
6 ESR1	CACCGAGTCCTGGACAAGAT	CTTTATGTCTCTCCTGTAGGAAGCC
6 ESR1	ctcaccTGTGCTTGAAAGTATTTCTT	TTCTCCCTCCTCTTCGGTCTTT
6 ESR1	CACCTGTGTTTTCAGGGATACGA	CAGGCTGTTCTTCTTAGAGCGT
6 ESR1	CCAAGCCCGCTCATGATCAA	TGAAGGGTCTGGTAGGATCATACTC
6 ESR1	TGAGCCCCCGATACTCTATTCC	GCTGCGCTTCGCATTCTTAC
6 ESR1	CCTTGTCAGTTCAAATCCCTGTTG	CACATTCTAGAAGGTGGACCTGA
6 ESR1	CTTTGTGGATTTGACCCTCCATGA	CTAAGCTACAGCCAGGTCACTT
6 ESR1	GAGCAACATAGTAAGGCTGAGGAA	TTTTTATCAATGGTGCACTGGTTGG
6 ESR1	CATAACGACTATATGTGTCCAGCCA	CCCCTGGGAGAGATGTACCTA
6 ESR1	GCCCTACTACCTGGAGAACGA	GCTCCCGCAGCTCCCTA
6 ESR1	GGCTCGGGTTGGCTCTAAA	GCTAGTGGGCGCATGTAGG
6 ESR1	CCTCGGGCTGTGCTCTTTTT	CCTTGGATCTGATGCAGTAGGG
6 ESR1	TCTATGACCTGCTGCTGGAGAT	TGCCTCCCCCGTGATGTAATA
6 ESR1	ACACCAAAGCATCTGGGATGG	CTTGCTGCTGTCCAGGTACA
6 ESR1	CCGTCCGCAGCTCAAGAT	CGTAGACCTGCGCGTTG
6 ESR1	CGCCGCCTACGAGTTCAA	GCGGGTGCAGTAGCATCAG
6 ESR1	CTCAACAGCGTGTCTCCGA	CCTCGCGCACCGTGTAG
6 ESR1	ACTTCATCGCATTCCTTGCAAAAG	CATGATGAGGGTAAAATGCAGCAG
6 ESR1	TGAATGTGAACCCTTTCATGTCTTGT	CCAGCAGCATGTCGAAGATCTC
6 ESR1	GAAAATGTGTAGAGGGCATGGTG	TCATCCAGCATTGAGTTATCTTGTGTT
6 ESR1	AAAATGTCAGGATAAAGTGGATCTGCT	AGCCATACTTCCCTTGTCATTGG
6 ESR1	GGCAGAGAAAGATTGGCCAGTA	ACAGGACCAGACTCCATAATGGTAG
6 ESR1	GTGTGCAATGACTATGCTTCAGG	TGGCTCTTAGGATCTGCTCATAGG
6 FOXO3	CACGGCTGACTGATATGGCA	TGGTGGTATACGGGAAGCTAGAG
6 FOXO3	AATGATGGGCTGACTGAAAACCT	GTCCGAACACCGTGCTG
6 FOXO3	CTCACTTAGCCACAGCGATGT	TGGAGCAAGTTCTGATTGACCAA
6 FOXO3	CCAGCCTAACCAGGGAAGTT	GACTGGCTGACAGGAGACTG
6 FOXO3	AACTCATGGATGCTGATGGGTT	CGGCACTGACCTGCTTTG
6 FOXO3	TGTCCTACGCGGACCTGA	GTGGGTACGCACCTTCCA
6 FOXO3	GCGTGCCCTACTTCAAGGATAA	AGCGCACTCCGACGAATC
6 FOXO3	GGTGCTCGGTTTTGGACCAT	GGGCTTTTCCGCTCTTCC
6 FOXO3	CCCATCATGGCAAGCACAGA	CAGTCAGCCCATCATTCAGATTCAT
6 FOXP4	GTCTCTCTTCCTCCTCCAGGTA	ACTTGCAGTTGTCCCTGTGG

6 FOXP4	TCTCTGGGCAAGCCGATG	CAAGGCGCACCAGTTCCTA
6 FOXP4	CCAGGTAGAAAGCCGAGTGT	CCTGCTTGACGCTGTCCTC
6 FOXP4	GCCCCAGCTGTGGAAGG	CGTTGGGCAGGGTATGGT
6 FOXP4	GCCACCGCTACCTCGTTT	AGGTCTACCCCCAGGCA
6 FOXP4	CTCTGCCACGCTGAGAGT	CGACAGGATCTGCTGCATC
6 FOXP4	AGATGCTTACCCCGCAACA	GCCCTTGGTGAGGTGTAAACAG
6 FOXP4	CTCCCAGAGCCTTCCACAG	CACGAGACCATCTGGGAATGAG
6 FOXP4	GCTCCTCCTCATTCTCCAAGGT	GAACTTGTCACTGCTTCTCCGA
6 FOXP4	CTGCCTCCCTGCATGGT	GAGACAGGCAGAGGATGAGGTA
6 FOXP4	GCCAGATGGCATGCCTGAA	TCTGCAGGTTGAGCAGGTG
6 FOXP4	GCTCCTGCAAATGCAACAGTT	CGTACAGGCAGGGCTGTG
6 FOXP4	GACACACAGGCAGCTCTCT	CCAGGGTTCAGCATGCCA
6 FOXP4	AGCTTCCCCCTCCTCAACA	GTCCGTGCTGTGTGCTC
6 FOXP4	TCCCTGATCGGCCACCT	GGGTGACACCTACTTGATAAACTG
6 FOXP4	CTGTGAGACCCTGTGTGAAGAC	AGAACCCTCTGGAGTGAGGAC
6 FOXP4	GGGATAGCAGTACCCTGCCA	GCCCTGGTACAGCCCCTTA
6 FOXP4	GTGAGGCTGTTTTTCCCATCTG	CATTCCCCACCCTCCTCCTA
6 FOXP4	CCAGGCCATTGCTGACTCA	CTCTCTTCCTCACCTGCACA
6 FOXP4	GACAGCAAACAGTCTGCCTCT	CCCACTTTTTGGTGTTGGAACTG
6 FOXP4	CTTAGTGAGAGTAAAGAGATGCGAATGA	GCATTAAGTGCTCCATAGCTGAGG
6 FOXP4	ACCCTGGTGAAGAACATGATCTCT	GGGTCTCTTGTATGAGAAAGCAAGG
6 FOXP4	AAACCGGAGCCAACTTTCTCT	GTCCCTCCCCAGCATCTTC
6 FOXP4	CCACTGTGCCCCAGAGTT	GTCTTCCTCTGCCTCTGCTG
6 FOXP4	CCACCAGGTGCAGGTGAAG	CCCTGCCGGTCACTACA
6 FOXP4	GCTGACGGTCCCTTTGCTT	GCAGTGTTTCTGCGGAAATAGG
6 FOXP4	GATCTATAACTGGTTCACCAGGATGTTC	GCAGGACTAATGGTTTAGGGAACT
6 FOXP4	CTCACCTCCTGCTTTGCCA	CGAGGCTCCAGGGTCTGT
6 FOXP4	GCCTCTGAGCTCCTCTTCAC	TCCAGCTCTGGACCCACATA
6 HSP90AB1	GCTTGACAATGCCTGTTTTCTCT	CATATTGGCTGTCCAGCCGTA
6 HSP90AB1	CAATAGACTTGTGTCTTCACCTTGCT	CCGCAGCGTCTCCACAAT
6 HSP90AB1	CTGGAGATCAACCCTGACCAC	ATGCGATAGATGCGGTTGGA
6 HSP90AB1	CTTCTGGCTTTTCCCTTGAGGAT	AATTAAGCCATGTGAGACTTGACCA
6 HSP90AB1	TGAACTCACTGTCTAAGGTCCTAACAA	GGGCAATTTCTGCCTGAAAGG
6 HSP90AB1	GAGGAGGAGGTGGAGACTTTTG	AAATGTGGAAACCAGAGCACCTA
6 HSP90AB1	AAGGTCCTCTTTTGAAATGTACCACTT	GCGGACATAGAGTTTGATGTTGTTCTT
6 HSP90AB1	CCTTTGACCTTTTTGAGAACAAGAAGAA	GCCTAAAACCTCCCAAGACTTCA
6 HSP90AB1	ACTAATTGCTGGTCTCAACTGCAT	CTCTTCTAGGTACTCTGTCTGATCTTCTT
6 HSP90AB1	GGGTACCAAAGTGATCCTCCATCTTA	ACATCCTGCACGTTAAGTGTAAAAGA
6 HSP90AB1	TTGGTCAAGTCTCACATGGCTTAAT	GCACAAGTTTTCCAACTATGAACTCCTA
6 HSP90AB1	GTTTTTCCTCTTCCCACCCTTCA	CGAGAAACATACTCTGACAGAGATGTC
6 HSP90AB1	TGCTGTTTGTATTTCCAGGCCTT	CCAAAGTCAGGGTACGTTCCT
6 HSP90AB1	CCTCCCAGTCTGGAGATGAGAT	CCACTCCTCCAAAAAGGCTTCAA

6 HSP90AB1	AAAATTGACATCATCCCCAACCCT	ATAAGTATGAATGCCTACAGAACTGCAA
6 HSP90AB1	TGATCAGGAAGAACTAAACAAGACCAAG	CAACATGCAAAGGCTTCTCACA
6 HSP90AB1	CTATTACATCACTGGTGCGTTGACT	GCTCCACAAAAGCTGAGTTGG
6 HSP90AB1	CATTTAATTACCCTACAGGTGAGAGCAA	CCCTCCTTGGTAACTGAGACC
6 HSP90AB1	CAAGGAATTTGATGGGAAGAGCCT	CAGAATGGCTTACCTTCTCAACCTT
6 HSP90AB1	TCTGCAAGCTCATGAAAGAAATCTTAGA	AGGCAAGAAGGCATGAAACAGTA
6 HSP90AB1	CAGGTATTGCAGTTCTGTAGGCA	CCACTTTCTCTGCCACCAAGT
6 HSP90AB1	TGGTGTTGGCTTTTATTCTGCCT	CCAACTACCTTGTAACAGAAAAGCTAAC
6 HSP90AB1	GGTGTAAGGGTTCAGGAGGCTAT	GCATTTCTCGGGAGATGTTCAGG
6 HSP90AB1	GCAGATTTTATCCGTGGTGTGGTT	CTTGTAATTCTCCTTGTCTTCTGCCA
6 ICK	CCTCCCGAAGGTTCATGCATT	CAGAATGGAAGCATTTTTCTAAAAGCAAAG
6 ICK	GAAGGGAAGCCCCCTAGTATCT	CAGCCAAGCAGCACTATTTGA
6 ICK	CAGGCAAGTATCGAGAGTGCT	CCAAGCCATGCTCTCTTCATTCA
6 ICK	GAAAATGGAAGAAAACCTGACTAGCTG	CCACAGTGTGTACCCAATAACTTAAAGA
6 ICK	ACTGCTAGCATTGGGAATCAAGG	CAATTGGCTGAGCAAGATTTTCTATCT
6 ICK	ATTATCATGGCAGTGAAGGATCAAACT	CGGCCGTGAGATGAGTGTC
6 ICK	GGGTCAGAAGAGGAATAAACAGACA	GCATCTCACGTACCCCTACAAAG
6 ICK	TGATCTGTCCTGGAGACCTCTG	ACAGAAAGGCATCCTGGAAAAGG
6 ICK	AGGTGGGACTGGCTTAATATAAGGA	GGCTAGACTTTTCTCCTAAAGCCTTATT
6 ICK	CCCACTGGCCTTTGTTTTGTATC	AACTACAGCTCCCCCATTGAC
6 ICK	GAGGGTGTAAACTTCTGCCATGAT	ATTTAATGTCTGAGTGGTGGAAGCA
6 ICK	TTCTCTTAGTGAAAAGTTAACATTTGTGACT	GGTTTGGCCCGAGAAATACGA
6 ICK	TTTGATCGTATTTCTCGGGCCAA	TTTGGATTTCTTCTCCTCTACTGTCTCT
6 ICK	TGATGACCAAACTGTACCTCTCTTTAATG	AGAAGCTCAACCATGCCAATGTA
6 ICK	CTGTACCTCTCTTTAATGAGCTGGTAAA	TGTTTTGTTTTGTGTTTATTCCTTGCCT
6 ICK	AGATTCAGTCTGTTTACAAATGTGAGCA	GTCTTATTTCCAGGTCAACAAAGGATTC
6 ICK	CATCCAAGTCAGCCCAATCATCT	TTCTGTTAATGCCAAGTATTGCTTTCC
6 ICK	GGGAAGGGTCTGGAATAGGT	GTGTCCTGGGTACATGTCTTTGT
6 ICK	AGTCAGCTGAGGGAAAGTATCCT	CATTCTTCCACACCCAGCCTA
6 ICK	GGTATCAACCCAGGAGTGCTTC	GCTTACATTGGCCCCATATCAGT
6 ICK	CCTTCCTGCATCCAGGGAATTT	CTCTCGAATCCAGGCAAGGAATT
6 ICK	AGAACTAGACCATGGATTAGGTGGAATA	AGAGTTTTTGACAAATTGAGTTCTGCATT
6 ICK	ACTTGATAACAACATTTCACTGCTCTTCA	AACTTTGTTTTCCTCTGCCTCATACA

6 IGF2R	TCTTGGTTCTCCCAAACACATTTGT	TGAACTCCAGCGCACACTTA
6 IGF2R	GTGTGAGCTCGCCGATGAT	TGCTCATGTAAACCAACCCCTTC
6 IGF2R	TTCACAGCTGCTTACAGCGA	GCCATTAGCATGCAAAGAGACTGA
6 IGF2R	AAAATCTGCATTAAGCTGCATGAAACA	CGCTCAGAGAACAAGTTTACTTTCCA
6 IGF2R	GTATGCCTGCCACAGAGATTACC	TCGATCCCCTTCTTCCTAAGCA
6 IGF2R	TGTTTAAAGAGAATACGACCAAGCCTAAC	GGCCAGAGACTTGCAGACATT
6 IGF2R	CCCTGCAGTGATTCTGAGGAC	GGAGCTTGTGATGGAGGAAAGTT
6 IGF2R	GGGACAACTACCTGTATGAGATCC	TTTGTGGGAAGCCACGGATTA
6 IGF2R	AGCTGCAATAGTGGTTCTCTCTTC	AGCAGACAGACTCCTCCGT
6 IGF2R	CCAGAACAAGGAGGCTGTAAGG	ACGTAACTTAAAACGACCGCTTCA
6 IGF2R	ACTGGATTACAGGCACCAGGA	AGCTTCTTTCTGCTGCTATGTCTAATTT
6 IGF2R	GTGGAGCATGTTTTATTTGGTAGCTT	ACATGTTCCCCTGAGTTGTCC
6 IGF2R	CTTGGAGGAAACTGGTATGCCAT	GGAGACAAGCAGTGAAAACAGATTCA
6 IGF2R	TTTGGTTCTATCAAGTTCCATGTTACTGT	CACACGTACCTGGTCTGTATCC
6 IGF2R	CCTGTCCCATTCAGACAACGA	ACACCCATTGTCAATGCAACATAGT
6 IGF2R	GGCTGCGCCATGAATACTGT	CAGAGTCGATATCTTGATGCAGGAAT
6 IGF2R	GTTTACTCAGGGCCCCTCAA	GACCGGCCTCTCAGTTCTTAG
6 IGF2R	TCTAGTTTGTGGCAGTTTTAAGCAAATG	TCCACACTTCCACAGATGTTGATTTTAT
6 IGF2R	GGGAAGCTGTTGATACCAAAAATAATGT	TTGCCAATCCAGTAATTTCAGGTAGTT
6 IGF2R	GGTTTGAATGCGCCCCTTTTT	ACTGGAAATGCATCATATCGCTCTTAC
6 IGF2R	TGCCCAAACCACTTATTCTGTTCT	ACTTGTCACCGTTGACATACATGA
6 IGF2R	CGGCGAATGGATCTTTGAGCA	ACATCTCATTGCTGGTCTGAACAC
6 IGF2R	GGGATTGAGTGACGAAGTGGTT	TGTTTCCGTCTGACTGCCATC
6 IGF2R	CAGAGCAGAATTGGGAAGCTGT	AGCATCACCTAGATCTTCCATCCA
6 IGF2R	CTTCCTTAGGGACTGCTGCTG	CTTCGCCCTGCTCTCTATGATG
6 IGF2R	GCACATGGAGACAGAAATGTCACA	CTGATGAACATGGGCCTGGA
6 IGF2R	ATGGGAAGAGCTACGAGGAGT	CAGTGTGAATCGAGCCTTGACA
6 IGF2R	ACCTGTCTGTGCTTTGTTGTAGAA	GGTTAACGACTGGCACCAC
6 IGF2R	CCTGTTCACTCTCTTTTCTCTCCAA	CAAGTGCCTGTGTGGACTCA
6 IGF2R	TGTGATTGTGTTTTCTCCGCCTT	TCTTATAGCTCAGGCCGGATTTG
6 IGF2R	CAAGGATGGGTCCCCTTGTC	AAGAGAGTGCATGTCTGCTTGT
6 IGF2R	CCAATAGGCCCATGCTCATCTC	GGGCAAGTGAACCTGACGAA
6 IGF2R	CCTTTTACCTGCCCCTTTGTG	CCTTACCTATGACACCCAGCTTCT
6 IGF2R	GTCCTGGGACTCGTTCACAC	GACAGGCAATCATATCTGGAGGTT
6 IGF2R	AGCCTCTTCTTGTTAATTTCCCTGTT	TGGCAAACTGCAGCTTGTTTTT

6 IGF2R	GTCTGTGGAGAAACTGAAATACAGTTCT	gaaaaTCGCACAGAGGTTGTTGAC
6 IGF2R	GGGAAGTGACTGTAGCCTGTG	AGGCTGAAGCTCTTGCCATTTATAG
6 IGF2R	TGAATGGGACCATCACCAACC	GCGGCATTGCTGGTTTAATTTTGAA
6 IGF2R	TTGCCCTTCACTTCTTCCATGTT	CTGAGGAAAGCTTCCCACAGA
6 IGF2R	GCTGGCGAATACACTTATTACTTCC	AGGAAAACGACAAACAATGGTACCT
6 IGF2R	CGCAGGGATTTCACAAAGTGG	CACTCATGTTTCTGACTCAAGGGAAA
6 IGF2R	ACCTTGGGAATGGTTAATTTCCTGAA	AGCGAGGTGTAGTTGAAATGCT
6 IGF2R	GTACTCCTTGCTTAGCGGACA	GTCTAGCCTTGGCTCACTAACC
6 IGF2R	GACTCAAAGCAGTCTTTCCTACTTAACA	GGGTTGGTATCGGAGGCATC
6 IGF2R	TGAGGCTTATGATGAGAGTGAGGAT	CTCCACCCTTGGATTTGTCATACTTA
6 IGF2R	ACTCTTTTCTCAATGTGGCTCTCC	ACTGTGCGCCACATTAGTGAT
6 IGF2R	GTTTTGCATTCTCACTTTTATATATGTGCCT	ACAACACATCCATGGACTTACTTGAAT
6 IGF2R	GCCCACCTTTCGATCTGACTG	tctaaTCCCCTGCACCCAAGA
6 IGF2R	TGGGTCTAAGGGTACGTGTGAT	TGGTGTCCTCTTACCAGGCA
6 IGF2R	ACACTACGAGACCCAGGTTCA	GCTGGAGATTAAAAACGCCAACA
6 IGF2R	CGCCTCCTCAGGGCTTATTTA	CGTCCAAGGTTTCCTGACTGTG
6 IGF2R	cgcctgCCTGAGGCTTTTTAT	CGGAGGATGCGGTCTTATTTCC
6 IGF2R	GTACGACCTGACTGGCCTAAG	AATGAACAGACGCCAATCAAGAGA
6 IGF2R	CTCCAAAGGTTATCCGTGTGGT	GAATTCCCTCCAGTATGCTCACT
6 IGF2R	GGGAAAAGCCTAACAAGACTGGTT	TGTCTGCCATCGCTGGTG
6 IGF2R	CCTTCTGGAATACGTGAATGGGT	ACATAAGGCAACGCTCAGTCAA
6 IGF2R	GGGAGTCACTAAAGGCAACTC	TTTCAGGACAATTATGCCATCTCTCC
6 IGF2R	AAGTCACTTCTTTGTCTGCGTGA	ACCACAATCATCACCATCTGAATAAGAG
6 IGF2R	CCATGAAAGAGAAAGGAAACATTCAACT	GATATGAAGAATGCAGGACCTCTGG
6 IGF2R	GGTAAGGGACGGACCTCAGT	GCAGAAGGTGCAGGAACAGTC
6 IGF2R	GACGTGGTGTGTCACTGCTA	CTCACTTCATCAGGACAGACGAC
6 IGF2R	TGTGTTCGAATGGGAGACTCCT	CCCAGGGTGAACGCATTAC
6 IGF2R	TCCTGTTCAGTTTCTTCCTTGCT	TTAACACAGGCGTATTCCGTGT
6 IGF2R	CCATGCCCTCTCTACACTGGA	TCAAGAGACTCGCCACAGAAAATTAC
6 IGF2R	TGCACCTACTTCTTCACATGGG	TGTTCCATGAGCATGTGGACTAAG
6 IGF2R	ACAGGAAACAAATCAGGCTGCT	GGGACAGGCCCAGTCCTA
6 IGF2R	GGAAGAACCTCTCCCTTTCAAACT	TCTTTGTCTCAACTTTATAGGCACCATT
6 IGF2R	GGGTTTTCTTTTGACTTATCACCTCTCA	GAAGCTACTTGCCTTTTTGCCA
6 IGF2R	CCTGTCAGCCAGACTCAGGA	GCAATGACATTAAGTGAGAACACACCTA
6 IGF2R	GTTTGTATGGCTCTTACCGTCTGA	GGAGGCTTTTCTCAATTCCGACT
6 IGF2R	CTCAAGAATTGGAAGCCAGCAA	ACCAGGGCCCTTTTTAGCTAC

6 IGF2R	GACCCGAACCAAACCTTGTTT	CAGTAGAAGAAGATGGCTGTGGAG
6 IGF2R	GGGACACTTGCCATAAGGTTTATCAG	CTAATATGATCCCAGCAGCCTGAG
6 IGF2R	GGACACCATAGCATGATAGTCCCTAA	CATGCTTCCTCAACTCTTCATCAAAC
6 IGF2R	CTTCCAGGTGCCATGCTATGT	CCTCAGCCCCACAAAGATTCAT
6 IGF2R	CATTTCCCACTGTCCCTTCCAA	CATAAAAGCAACCGCCTTCCC
6 IGF2R	cctacaCTCCCCAGCAAACA	TGGACGAATTTGCACTCCAACT
6 IGF2R	GTGCACCAGTGTTGAGAACTTC	AGTGGTTCCAAGTCACATTAAAGCA
6 IGF2R	AAAAGTTGTCTGCCCTCCAAAGA	CTGAAGTTCACATGCTGAGGGT
6 IGF2R	GGGAAAGTCAGAGCTGCTCTT	CGTTCTGACAGCCCCTTGTG
6 IGF2R	GGCTTTGACAGCGAGAATCC	CCCTGCCACCCGCTTAC
6 IGF2R	CACGTGGTCTCTGCTGTTGA	AGCTGAGGGCTTTCACTGAC
6 IGF2R	GAGAACGGCCATATTACCACCAA	CCTGAAGGGCATTGCTCTGT
6 IGF2R	GCTCCCACCCAGTGAGAAAC	GTCGTCATGGAAGGACACCA
6 IGF2R	AAGACAGTGAGCTCCACCAAG	ATCATCGAGTGGAAGTCTTATTTGGTT
6 IGF2R	CCTCAGGTCTTTGCTGAGAGAAA	GCTCTCGGTGTGTGTCTTTCA
6 IGF2R	GGCGGCACACCCTATAACAA	GCGCGATGAGGAACAAACATTC
6 IGF2R	ATGAAGGGAGATGGGCAGACTA	ACCGGAAGTTGTAGGTGGAGTTAT
6 IGF2R	CTGCCGTGGATTAGGAAGAGG	CATCTGTACCAGGCCAAAAACA
6 IGF2R	GCAAACTTTTTAGACCGTAAGGAGCTA	TGGAAGAAGATGGTGGAAGAAACAG
6 IGF2R	GTGCGGAAAGGATAAGACCAAGT	CTAAGCTTCCGGATGTCATACTCA
6 IGF2R	CCTTCTTGCTGCTGCTTTCTTTA	CACTCAAAGTAGTGCACACATTCTG
6 IGF2R	GGGAACTCCTGAATTTGTAACTGCAA	TTCCCCAGAGTATACTTCATAAGCATGTA
6 IRF4	CTCTCCTGCACTCCTTTAGCA	GGTCCAAACGTCATGGGACATT
6 IRF4	CACCCGGAAATCCCGTACC	GGCCACATTAGCAATTAGCTCTTCTATT
6 IRF4	AGGTCAGTTCCTGTTTTTACGTTGT	CGAAGGGTAAGGCGTTGTCAT
6 IRF4	GACCCGCAGATGTCCATGA	CATAGCAAATGGCAAGTTCTGCT
6 IRF4	GACTTTTCGTTCTCTTCATTCTTTCCC	GCTCAACCAGTTCCTCAAAGTCA
6 IRF4	GTGCGCTTTGAACAAGAGCAA	GTTAGGTGACCCAAATTCAGGAGAG
6 IRF4	ATGGTCTCTTTCTTGTGGGCTT	GCCACTATGGTAGGAGTGTAACTGA
6 IRF4	GCTGAAGGGCAGCTCTTCT	GCCACTGGCGGAGCTTC
6 IRF4	GCGGAGAGTTCGGCATGA	GGAAGATGCTCTTCTCCTCGTT
6 IRF4	CAGTGGCTGATCGACCAGAT	CGTCCTCCTCGCGGTTG
6 IRF4	TGCGTCGGACTCTCTGTCTA	CAAACCCTGCACTCACCTGA
6 IRF4	CATAAGGTCTGCCGAAGCCTT	GGGAGCAAAGCAAGTGTCTAAAGT
6 IRF4	AACTTTGGGCTTTACGTTACTGTCT	AATGTCCACTGTTTTGTTGAGCAAAAT
6 IRF4	ACCTCTGCTAGCCAGACAACTATATT	ATTCTTGAATAGAGGAATGGCGGATAG
6 IRF4	CTTGGCTCTGTGGAGTCGTT	GCTCCTTCACGAGGATTTCCC

6 IRF4	GCTGCACATCTGCCTGTACTA	CAGCTTCTCAATGTTTTTCCTCTGG
6 IRF4	TTCCCCTACCCAGAGGACAAT	AGTAGATCCTGCTCTGGCACA
6 IRF4	ACGGGCTCTATGCGAAAAGAC	ATTCTAACAGATAACGAGGTGCCTTAC
6 MAP3K7	AGCTACATAAACTACACACACACATCTG	CTTAATTTCTTAACAGCTTCGGCAGT
6 MAP3K7	GCAGGGTCCCGCGAATTAG	CCCAGGTCCTCAACTTTGAAGAG
6 MAP3K7	ACCTCGATCTCCTTGTAGTCGAT	GGGTCTCACCCGGATTGT
6 MAP3K7	GGGTGGTGAGAGTGAGAGAGAA	TTTACAGTGTTCCCAAGGAGTGG
6 MAP3K7	GGGTTGCATGCTGTGAAGATAAG	GCTTGCATTCACATTGTGTCTTTTGTTA
6 MAP3K7	TCACAGAGTATCTTAAGTGCAGAACTACA	AAGAGGAGCCTTTGGAGTTGTTT
6 MAP3K7	CATCTTTTGCTCTCCACTTAGCTTTG	ATTGGGCTGATCTATTTGATTCCGATT
6 MAP3K7	AAGGAGGCCAACTAAAAGGATAAATT	CCCAATGGCTTATCTTACACTGGAT
6 MAP3K7	GAAAGTACTCCTTTACCTGTAGTTGGTG	ATTCATGCTTTCTTGCTGTTCGTTTAT
6 MAP3K7	ATTATTCCGGTTCCACAGCTATTAGAC	GTTTGTGGTTGTTTGCATGTATACTGTA
6 MAP3K7	GCATGTTTCAGGAAGAAAATTCAGGAT	AAGCGCTTAGAATCAAAATTGTTGAAAA
6 MAP3K7	CAAACCTGTTGCTTTGCCTGAT	CAGCCATTAGTCATAAATGTTTCTGCTT
6 MAP3K7	CCAAAGCCCCTCAAAAGAAGGATT	GCCCATTGAGAGCCTGATGAC
6 MAP3K7	GGAAGGATCTTTAGACCAACAACGA	GGAAATACGTACAAAGAGTTTGCATCAT
6 MAP3K7	TCTAAACAACAGTATAATGCCTGCCTTT	CATGGACCCCTGATGATTCCAC
6 MAP3K7	GTATGAAGAAACAGACTGGTCACCT	TGTTTCTCCTGTGACTTCTTCCTGTA
6 MAP3K7	TTTGGGACTCTGACGGCAAAT	TCTTGTGATGGAATATGCTGAAGGG
6 MAP3K7	GATAAAGACAGGTCTAATGACACTCACC	gccctAAGATGATGGAGCTTCT
6 MAP3K7	ATATTTCAAAATGTAACGGTCCCAGAGA	TCGCCTGGTACAGGAACATAAAAAG
6 MAP3K7	GCTGGTAGTAAGTAGAAAGGCTTTTGTT	TGAATTTTTCCATCATGTGTAATATTATGTGGT
6 MAP3K7	CATATGAATTCACAAGGAGTGAGGGA	TGACATTCAGACACACATGACCAA
6 MAP3K7	CAAGCAGCACTCCCCTTGTTA	TGTTGGCCTTGTAACTTTTGATGTTTTT
6 MAP3K7	GAATGTTGTCTTACCTGACCAGGTT	CACGCTTTTTGTCTGTTTGACTAGATTT
6 MAP3K7	ACTACCATGGCCAAAAAGGCTTA	GGAGCAGTGTGGAGAGCTTG
6 MAP3K7	CTCTTGCCCTCAGAGGTTGG	CCCATTTGCCATCTTCCTCCTTTTA
6 MAP3K7	CTAATTCTAAAAACTACGTTTTCATTGCACCT	GGCAGTGTTTGAACAGCATTGTAAAAT
6 MAP3K7	CTGTTTGAACTTTCATATATTCTTGTGCCA	CTCTGTCTTGACTCTCATCTTAGCTAGA
6 MAP3K7	GGGACAGGAGTAGTCTTCCAA	ATAACGCGTCGGAAACCCT
6 MAP3K7	CTGGGCCACCAATCTCATCAA	CCATTGTTTCTGTAAGTCATGTGGAAAA
6 MYB	GATTCGTTCCCTCATTCACTTTAACTTG	CTAACTTACCGGGAGATAATTGGCAATA

6 MYB	CAGAATGGAACAGATGACTGGAAAGT	GGTAGGGCATTTCTCCCACTTCTTAA
6 MYB	CCTTCCTGGTGTCAACCACTT	CTGCTCCTCCATCTTTCCACAG
6 MYB	CAGTACCTGGGAACCTGCATC	GTCAACTTGAAGTGTTCTGAAAACCA T
6 MYB	CCACTCTTCCGTTTAGCCTCA	CTTCCAAAGGGTTGCGTGTTTA
6 MYB	AAAACAACTAATAGGCCCAAGGGAA	GACCTTCCGACGCATTGTAGAA
6 MYB	CTGATAATGCTATCAAGAACCACTGG AA	GGCAGGGAGTTGAGCTGTAG
6 MYB	GTCATTTGATGGGTTTTGCTCAGG	CCTGGAGCTAAACTAACCGTCTCT
6 MYB	CTCTTGTTTCAGCCCACGTCTA	AAGTGACGCTTTCCAGACTTG
6 MYB	ATGACTATGATGGGCTGCTTCC	GCTTGAGCACCTGTAGTTTAGTTGAA AT
6 MYB	ACAGTGTTAGAAAAATGTGGCTCTCA T	CAGGGACCTGTTTTTAGGTACTGTAA A
6 MYB	AGAAGATGAAGACAATGTTCTCAAAG CA	ccaAAGATGACAGTTTTTCAGTGAATG A
6 MYB	CAGGTAAAGCCATGTAGGGACA	GCATGTTTGAATGGTGTAGGAGTTC
6 MYB	AAAAGGTCAATCTTAGAAAGCTCTCC AA	CATCAGAGAGAACTCCTAATGGGACT AAT
6 MYB	GTCCCCTGAAGATGCTAGTAAGTTCT A	CAGATCTTCTACTAGATGAGAGGGTG TC
6 MYB	TTCTCCCTTTCTTCTGTCCTCTCTTTA	GTGGTGTTCTCCCAAACAGGA
6 MYB	CATGGAGACAGTGCACCTGTT	AGTGTTTCTGCAAATTCTAAGAGGTT CT
6 MYB	CAGGGCACCATTCTGGATAATGTTA	GCTTGGCTATAGAAAATGGCAATGAT TT
6 MYB	AGTGGGTCAGGAAAACATTCTTGTT	GGGTGGAAGTTAAAGAAGGCAT
6 MYB	CCCCCTCATTGGTCACAAATTG	AGTGGATCAGACATCTGGGAACTATA TT
6 MYB	AGGAAGTTCTAGAAACAACAAAGCA CT	GGCTGGTGCACACGTACTTA
6 MYB	GCGGCACCTCATGACCATAT	CTCGGTTGACATTAGGAGCAATTCTA A
6 MYB	CCTGAGAAGGAAAAGCGAATAAAGG AA	AAGCTGCTATCCCCTCATTCAAG
6 MYB	TCCCATATTTCCAGTGAATGAAAGCA	ACGTTTCGGACCGTATTTCTGT
6 MYB	GTGTTTATCTGAAGGTGATAGAGCTT GT	TTCTGTCCAGGAGGTTTTCTTAACTTC
6 MYB	GGTGGCATAACCACTTGAATCCA	CTCCCTCATTCTTCCCGATCAAT
6 MYB	TGTGCCTCCCACATTGTTTCA	GTTCACCCTTGGGCTAGGTTC
6 MYB	CAATGGGACTAAACCTGCAGGA	CTACAGTCTCCAGTGGCTAAGG
6 MYB	TTCCACTGGTCATCCTTCGAAAAA	TGAGTAACAGCTGTGCAGAAATTGT
6 MYB	CCTGAAGCATATGTAGCCCTGA	GTGGTGCTTTGAACAGTGAATGAT
6 MYB	TTTTCACTGCATATGGTCTATGTGTGA T	CCCTTGATGAGCTCAGGGTTTAG
6 MYB	CAGCACCGATGGCAGAAAGTA	CCATTTTACCTGCTTCCTCAAATAGGA
6 NOTCH4	GAGAAGGCAGATTTGTGGTCACT	GTGTGACATCTAATCTCCCCCATAATT
6 NOTCH4	CCAGCCCACCCTTGTCTTT	GATTCCAAGGCAGCCTGTG

6 NOTCH4	CCTGGACTCACATGGGTTCAC	CCCCACTTATCTTTGTATATCCCTGTT
6 NOTCH4	GACTCTCTGATTCTAATAGGGTCAAAGG	CTCAGGCAGCCATGCTAACT
6 NOTCH4	GGGCTTCCATCTCAGATTCCT	GGATGAAAGGGCAAATCAACCTT
6 NOTCH4	GGGAGGGTCTGGAAGATGTTA	CCAGCCCAAGCAGATATGTAAGG
6 NOTCH4	AGTTGGCCTTGTCTTTCTGGT	TGTGGAGGAGCTGTTTTCTTTTTGA
6 NOTCH4	TGCCCCAACCCAAATGGAATA	GCTGTCAAACAGAGGTGGATGAG
6 NOTCH4	TCAGCACGCCTGACTTCAAT	CTATCATGCCCTGTCTCTGTCTTG
6 NOTCH4	CCGGAGAGCCCCTGTGA	GGAGGGTCCACGTTGTGA
6 NOTCH4	CATTCGAACAGCCCCTAGGAG	CCATGCCTGTGAACGTGATGT
6 NOTCH4	GGTCCTGGAAGCACTCGTT	CCAGCTTCGGGACTTCTGTTC
6 NOTCH4	TCCATTAACACATGGGTTGGCT	AGAGTGAGCACTGGGTAACCT
6 NOTCH4	TGGAATTCCTCCCTACCATGTATTCT	TGGGAGCTTGCGGTTCTG
6 NOTCH4	GCGGGATCGGAATGTTGGA	CCGTAGGTTTTCTGCAGGCAT
6 NOTCH4	CACTCCGTATCTTCCTCGCATTAT	CTTGGTCCGTAGACTTGGCT
6 NOTCH4	GGCTCCGGCAATGAGAATAGG	GCACGGACGGTGTCAGT
6 NOTCH4	CACAAGCATACCCTCCTTAGTTCTT	CTAGCCCTGGAGGCTACTACT
6 NOTCH4	ACACAGTAGTCAGTGCTGGTTTG	CCCCCACCTGTTTCCTTCAG
6 NOTCH4	actccTGCAAGGCACACTC	GCACCCAGGGCTTCCATT
6 NOTCH4	TCCATTGCCTGTTGCTAGCAT	GGCTTCTACTCCGCTTCCTTG
6 NOTCH4	CCACTGCAGCCATCGCA	GGGACCAATACAGAGGTCTCTGA
6 NOTCH4	CTCGCCCCATTCCCTGTA	CGATTCTCCCGGCCAACC
6 NOTCH4	GGTTGGCTCCAGCCTCAA	GCATGGTTGGGATGTCCTGA
6 NOTCH4	CCTCCATCCAGCAGAGGTTC	GTGTCCTTTAGATTGACCCATTACTCT
6 NOTCH4	TCATGGATGTGGCTTAAACAACTCA	GCTGATCCATGACCCTGTCA
6 NOTCH4	GCATTCCTAGTGGGTTCAGGACTA	CCCGTTGTGAGGCTGATCAC
6 NOTCH4	GCTGGGAGAGGCACTCATT	TGGACTCCCAGCTCTTCTCT
6 NOTCH4	CCAGCACAGGGTGTATATGGTT	TGTGTGACGTGGGTTGGAC
6 NOTCH4	CCTAGCTCTGCCTCACACTCT	CCAGAATCTCAGGGCCAAAAGAA
6 NOTCH4	TTTTGTGGGAAGCCCTCTGT	GGGTAACAACCTCCCACGTC
6 NOTCH4	GGTCTTTGGGCCCTGCT	CCCTCACAGCCTCCTCTCT
6 NOTCH4	CTCTATAGCATACATCACCCCTTCCT	GTAGCAGACAAACTGCAGTGGA
6 NOTCH4	CCAGCATCAAGGGTGTGGTC	ACTCGAGTTGGCAGTCCAA
6 NOTCH4	GAGCCTGGGTTTCTCCTCATTC	TCTTCTCGTCCTCCAGCTCAT
6 NOTCH4	GGCCGTCGAGTGAAACCA	ctgccAACCAGCTTCCCT
6 NOTCH4	TGGCCAGAGAGGCATCTGTA	CTGAGCCACAGCCCTCAT
6 NOTCH4	CGCATGGGTGGAGACTATCT	CACTCTCTGGCCAATGCCTT
6 NOTCH4	CAGGCAGACACTGGCAGTAG	ACCATGGAACCTGTACTCCCA
6 NOTCH4	CACAGTGGAATCCTCCAGGTT	TGGTTCCCTGTCTAATCCTCTTTGT
6 NOTCH4	GGACATGGGTCACTCAGGCA	CTGCAAGTGTCTCCCAGGTAA

6 NOTCH4	CTGTCCCCCACAGTGTGT	TCCTCTCCCCACCCACTTC
6 NOTCH4	GTTTCTCAGGCTCCAGTTCTATCTTC	CCTCCCTCCTTCTGTTCCAAAA
6 NOTCH4	GAGGCCTGGATGTGGCA	CCCAGCTCTCCCTCTCCAT
6 NOTCH4	GCACAAGAAGCTGGGTGTCA	CCTGGCTTCCTGGGTGAG
6 NOTCH4	TGTGGTTTTGATTCTCAGATGGTTGT	TCTCTGCAACCTTCCACTGTC
6 NOTCH4	CGTTGGTTACCTTGGCTCAGT	CACCCCTCAGACTCTGATGGA
6 NOTCH4	CAGGGCTTCTGGGCACATAA	GGCCATTCAGGAGAAGGTTTTGA
6 NOTCH4	AGCAATCTGCCCTTTTCTGTCTT	CTCACAGGCTCCACACTCTG
6 NOTCH4	CGAATAGCCAGGCTGACACA	GAGGACTGCTAGGAGCTTCAA
6 NOTCH4	CCTCCTAAGGGAGCTGGGT	AGGGTCCCTGCCTGAAGA
6 NOTCH4	GGGCACGCTTACTGACAC	GACGTCGCTCACCAACGTA
6 NOTCH4	GTCAGCAGATCCCAGTGGT	GGCCCTGGCTCTTCTGTA
6 NOTCH4	TCAGTGAAGGTTGATTTGCCCTT	TCTACCCACCCATTCCTTTCCA
6 NOTCH4	GGTGGGAAGCCTGGCTTAG	CTTGATGGAGAGCGGGAAGA
6 NOTCH4	CGTCCTTCCTCCTCCTCTCA	CTCGGTGCTGGCTTCTCTT
6 NOTCH4	GTCACACTGTGGGTGGCA	GGGTGTGAGGGCAGAAGT
6 NOTCH4	CATCGCAGGCCCCATCT	CCTCCCTCCCCATGCCTATAC
6 NOTCH4	GTCTCTGAGCAGCTGCCATT	CATCACTGCCACCACGGA
6 NOTCH4	TCCTGATACCCTCTACCCCCATA	GGTAGCTTTCACTGCGTGTGT
6 NOTCH4	CCAGGTTCTCCTCACAGCTTG	CTGGGACTGCTCCGAAGATG
6 NOTCH4	CCTGGGTCTCACACTCATCCA	GCCACATCCATGACACCCAT
6 NOTCH4	TTTCCTCCCTCTGCCCTCTTAA	GCTGAAGAAAAGCTAGGAGGAACTC
6 NOTCH4	GGCTGCTCTCTCCTGATAGGT	CCCTAGACCAGCAGCTGTTTG
6 NOTCH4	CTACCCTCAGAGTCAGGGACA	TGTGAGAAAGGCTGCAACACT
6 NOTCH4	ATCTTCAGGCCTGCAGTCAC	TCTAGGGTGAAGAAAAGAGTCAGGTT
6 PIM1	CACTGAGTCCCCGTGCTT	GTTGTCGGAGACGCGGAT
6 PIM1	CTTCGGCTCGGTCTACTCAG	CCAGGGTCTCCGTCAGGTT
6 PIM1	TCTCTTCTATTCCCTTGGCTCACA	TGCTCGAAAGGAATATCTCCACAC
6 PIM1	GGGATCCTGCTGTATGATATGGT	GAAGACTAATAGCCCCATTAAGACCA AT
6 PIM1	gcACGGGCGTGCTTTAG	CCCCACCCACTCATCCTG
6 PIM1	GGGAAGACCTTTGCAGTGTAAA	GGATGGTTCTGGATTTCTTCGAAGG
6 PIM1	CCTGAGACCATCAGATAGGCCAA	CCTCGGGCATCTGACAAGAG
6 PIM1	cagcaACCACTAGCCTCCT	GCGAGTCGGAGGACAACTG
6 PIM1	cccagGCATAGCCTTCGG	AGGTGGGCAAGCGAGTT
6 PIM1	CCCGACTCCCGCCCTAA	CCAGTCCAGGAGCCTAATGAC
6 PIM1	AAGAAGGTGAGCTCGGGTTTC	CTCCCCTTTCCGTGATGAAGT
6 PIM1	GAGCCGGTGCAAGATCTCTT	TGAGGTCGATAAGGATGTTTTCGTC
6 PIM1	CTCCACCGCGACATCAAG	GCCTGGCTCACCATCGAA
6 PKHD1	CCAGAAAGTTTCCCTCCTCCATC	TGCTTCTGCTGGACACTAACAC
6 PKHD1	AACATACCTTCCTCCAGCCTTAGA	ATAAGGATTTCCACTTTCTGTCCTCAG
6 PKHD1	TGTGCAGTAAGTTGAGGATGCTT	caaccCAGCTCTCTGTTTTCCA
6 PKHD1	CATAAGAAATGTGCACTTGGTAAAAC CC	GGTAGTGGTTTGAATCTGACCTTCA

6 PKHD1	GCAACCCCAATCACCCCTTTA	CTTTGTTTCTGACGTGCCACAG
6 PKHD1	GTAACCCCTAGCAGGAAAGTTTGAG	CAGATCAACCTTCTCCCTCTGG
6 PKHD1	CACATAGAACAGGCCCGTCT	CAGTTCTGCTCCATGCTGTTTTT
6 PKHD1	GGACAGCACCTCGTTCAAATC	GGGTGGTCTTTATGCTCATGATATAAAT
6 PKHD1	CTTAGGGTGGCCCATTCACT	CATCTCAGGTCTCTGATGAAAACCTT
6 PKHD1	TGTCCTACACAAGAATGCAGAAATTCA	GTCTTTCATACAGGGCTGCACA
6 PKHD1	GCAGCAGGATTCACTCCAGT	AGCTGTCTGATTATCTGAAACACAATGT
6 PKHD1	CCTCTACTGAAGGAGTTCCTCAC	TTAGCTACAGTGTAGGTTGCTCTAAA
6 PKHD1	CTCCAAGAGAGTTGTAGCCCTT	GGGCAGAGTCAATGTTTCCATCA
6 PKHD1	ACCTGATAAAGAGGCCCGAGAA	CAGACACACCTATGTGCAGTGT
6 PKHD1	TGACACACTCTAAAAGATAGGCTGAATG	CCTAGTCCAGCCTTCAGATGGA
6 PKHD1	ACTGGAAGCTCATTTCCCACTTC	TTGCTACCATAGGGATATGTGTAATCCT
6 PKHD1	CGCCATGGCAACTGTCAAATC	GTTCTTTCCTAATGGTGACTGTTTCCT
6 PKHD1	GGGCCTTATTTATCATCTGTTCTGTCT	TGATGGTGTCTAACACAACCTTTGTT
6 PKHD1	AATGGCCACACAGTTGATGAGAT	ATGTGTCCAGTTTTCTTATTTTGCTTTCA
6 PKHD1	ACCACTCACCTAGGTTTGCAAC	GACATGCTCACTTGTGGTACTATTTTC
6 PKHD1	GGCCCAGCACATGTAATTTTGT	CTTGGAAAGTGATGCAAATACTTCAGTT
6 PKHD1	CAAAATGACCAAGTAATAAGCTGTCAGT	GACCTTTGCTGTAACATGCTGTATTT
6 PKHD1	GGTCTTCACCAGTACATTTCATTCTCA	CCTCCACCAGTTTCTGTATTTCCT
6 PKHD1	GCAGTCCATTCTGCCTCTGTTTT	AGAGTTGCTTTTTGACTGTCTTCACA
6 PKHD1	TCAACCACAGCAATGCCATCTA	GCAGGAGTATTACCATACAAGGAAATCT
6 PKHD1	GCAGCTTCTCCCTCTCATTAGTG	AGATTTGGAGTGATGTCCTCAGTTCTAT
6 PKHD1	CACAGCTCTACTTCATTTCCTCTGAT	GGATCCGAAGACAAGCCCTTC
6 PKHD1	TGTGATCTGAGCTCTGCCTTG	CCTCCCAGGGACTGACAATTTT
6 PKHD1	CTGTGCAGATAAAGTGGTAACAGA	CTCCCTCCTCCTATTCCCATTTCTA
6 PKHD1	CAGATGCTCGCCTCCGTTAA	CTTTCCGTATTCATACTTTAGGAGGGA
6 PKHD1	GCCTATTCCTGAGATGCTGAGGAT	GTTTCACTAACACATGCCCTACCT
6 PKHD1	CATCCCTCATGCCATACAGACAT	TTCCAGAAACTGGGAGCCTTG
6 PKHD1	CCCCAAGGCTCCCAGTT	ACTGTTGCTGGACTTAGAAAGTTAAAGT
6 PKHD1	AATTCCTTCAAAACACATTCTACTGACCT	TGCTCCAATCAAAACTGAAAATGCTTT
6 PKHD1	GGCACACATAGAGAAATTAGTAGTCAGT	CTAATGCTACAGTGTCTGCCTTCA
6 PKHD1	GTGGCATCTTTACTCACCATCCA	GTGTTTGGAGCGGGATTTTCTC
6 PKHD1	ACACAGCAGCTGAGACATTCC	GAAGAATGGGTAGTGAGGAAGCA
6 PKHD1	CCTCAAGGCCAACAAGCATTCT	GAATCAGTCTCTGTGGTGGGAT

6 PKHD1	CAGGAGGTGACACTGTAGACC	TTCCAACTGGGATTTTCCACACA
6 PKHD1	GTTTAGGGTTTGAAGAATTGCCAAGT	ACCAAATGCAGAGAACAGTGGAATT
6 PKHD1	TCTCTGCTGTTATTGGGTGCAT	CGATATTTGTGGCTGGTGGTTTATATGA
6 PKHD1	CAAGCATGTGCATTGGCAAGAT	TGGGAGTATTGCACAATTATCTCTTGTC
6 PKHD1	GTACAGTTGTCAAGTCCACTTTCCT	GACATCAACCTCCATGGCAAC
6 PKHD1	GCACAGGAACATCACCCAATCTC	GCTCCGCTTCTTTCCTTCACTC
6 PKHD1	CTGGTACAAGAATGTGCAATGTTCT	TTCCAGCAACCTTATCATACATGGG
6 PKHD1	AGTTCCCAGTCTCTTGCAGGTA	CCCCCACCAGTTGATGAATGG
6 PKHD1	GTCTCTCTGATCCTGCCACAAC	CAGCTGGCCATGGCATAGAT
6 PKHD1	GGCTAACTTTCCTTCTGGACACT	GTCAGAAATGCTAAGTATGCAAAATGTGT
6 PKHD1	GACAGTATAGGCCTGACCCTCTAA	TTGAATTCAGTAATGTGTCAGCAGGAT
6 PKHD1	TGGTGCAGAGTAGATGATATGATCCA	GGGAAGAGGAAAGTACCTGATGA
6 PKHD1	ACTTAAGAGACCTAACATTTTGCCATCA	GGACATGCACGGAGATGAGATAT
6 PKHD1	CATGAATTGCCTCCCAGGAGAT	CACCCAAAGAAGTAGTGTTGCAG
6 PKHD1	GATAACAGTCACATAAGAGCCACTCA	GGCTTACTCTTTCTTATCTCTCTCTGTCA
6 PKHD1	CCAACTGCTTACAATTTATTGCCATCA	TACATGTATATGGCTGGATTATCACTGG
6 PKHD1	AAAGTTTCCAATCTTCCAGTGATAATCCA	ATGACCTGCTTTGCCATATTGAGTA
6 PKHD1	GAAAAAGCCCTAAGTTACTCTCATTCCT	GGCTTTCATTCCTCTGAAGGATTTTTCT
6 PKHD1	TCACCAGCTACATGGCCTCTA	CTGCTCTTTCCAGTACCTCCAAG
6 PKHD1	AATGGACACAGGGAGTTGACC	GCTTGGGAGCACTTCACATATACTTTAT
6 PKHD1	GCACACAATACACACACATGCatt	ATCCCTTAGAAAAGGAACAAAAGCTTCT
6 PKHD1	GGAGAATCAGAAGCTTTTGTTCCTTTTC	GACTCCTCCATATAGTCTACTTCACAGT
6 PKHD1	GCAGCAAATCCATGCCACTA	CCGATGACTTCACATCCAGGT
6 PKHD1	GTGAAGTCACTGGCATTGAGGT	GAGGATGAAACTCTGTAAGGTGGATTAA
6 PKHD1	TTCCTGATCATCCAATGGCATGT	CATTTCAGTTGGTCAGAGGAACCA
6 PKHD1	ATGAGAAGACAGTGAATACCTTAGTCCT	GGTTACTCTTGCTTGACTCTATGTTCT
6 PKHD1	AGCTATTCCCACTATAAGGGAGAAAGG	AGCTTCTACAACTTGGCAGTCTTG
6 PKHD1	GTGCCAGTTTGACCCAGAGAT	AGAAGACATGAAGACATTGTTCGAGATT
6 PKHD1	TGCTGTGAGAAAACAAGTGACAGT	TGTATACCCTAAATTTCAGCCACCTTG
6 PKHD1	GCCAGTGACATTATCCCAAGGTG	AGATTCCATCTACCTTCATTCTCCCTAA
6 PKHD1	GTGAGGGCCAAGTGAATGGAA	ACTCTGGTTCAGTCAGCTTCCT
6 PKHD1	AAATCAGCCCTCATTTGGATGTGA	CATCGAGTCATCACTGCTCAACA
6 PKHD1	TGGCACCAATAGATTCATTCAGCAATA	AACTCCTCAAGTTTTGCGCTTTTT
6 PKHD1	TCAGAACATTCTCTAGTACCCCAGTC	GAAATTGGTGATTCGCCAACAGT

6 PKHD1	CTCGCCAGCCCATCATGATTA	TTTAATTCGCTTTAGGCATTCCATGTG
6 PKHD1	GAGAAAGAAACATGAGAAAGTCCTAGGT	CGTTCAGTCCTTCCCAGAAGAG
6 PKHD1	GGAGGTACTTTTGTTCCCCAATAGA	GGTTTTGTTGATGTCTTTAGCAGTGTAA
6 PKHD1	GGATGAAATCATTCCAGTGCTCCTT	CCTACTGTGACTTGCACTAGTCATTA
6 PKHD1	CACTCCCTTCAACTGGACCTG	CTACATGCTTTAGGTTCTCTGGACTT
6 PKHD1	GCCTACGTTGACCAACTCTTCTA	GATTGGCCAAAACCAAATCAGGTT
6 PKHD1	GAAGTAGAGCAGGGAATATTGGCAT	GATGCAAGGATTCATCTGCAAACAG
6 PKHD1	CAAGAATTAGGACCACTTGGTCAGT	GATGGCAAGAGTGATGGATTACAAATAA
6 PKHD1	GGCCAGGCATCTCGTGAATA	CCCATTGAAATACGCTCAGGTGT
6 PKHD1	CCACTCCCCTCCCTCATTTTT	CACCATCATTTAGTCTTGTCTGTCTTGA
6 PKHD1	GGGCTATAAACCAAATTGCTTCAGA	CATCCAAACGCCGAGAATCAC
6 PKHD1	TCCCACCACAGTGTCTTCTTTTT	TGCTGATGGTCCCACTTACAAC
6 PKHD1	TGTCCAGACCCCAATACAGTTAAGA	GTCATCATCAGTGGAACAGGTGTT
6 PKHD1	TCTGACTATGTGCTCTCAAAACATTCAT	GCCTTACCCTGACATCCTCCTA
6 PKHD1	TCCATCGGTTTGGCACCTTT	ACTTTTCCTCGGGCTGCTTTAG
6 PKHD1	CCCACTCGACTCCCACATCT	GGTTCCTTACTAAATGAGCACTATTGTGT
6 PKHD1	GTCTTTAACCTCTAACTTCCAAGGGAA	GTCTCCACAGAATTTCCGTCTCTATC
6 PKHD1	ACAGCTTCGGGTGTTAATGGT	GATCAGTGCTAAACAGGATCTTTTCCTA
6 PKHD1	GGATAGTACCTGAGTGTGTCTGGTA	CCTTTCAGGCAGCCGACTAAA
6 PKHD1	GTGAGTGAATGCTGACCCCATT	CGAGGCTGATGAACTATACGGAT
6 PKHD1	GACGTGGACTTCCACATCCAA	GAATAGATGGTTGAGCCCACAAAC
6 PKHD1	CGCTGCCATTTGGACTAAATTGT	CAGCCAATTACCGTCAAGATTACTGA
6 PKHD1	GCCCCAGCGTTTCCGTATC	CATTTAACCTCCCCTCTCGTTTCA
6 PKHD1	GCTCCTGCTACATGGGACTTT	GAATCCTGATGTGGTCACCACT
6 PKHD1	TGGTGCTTCTCCCGTAGGTA	GCAGAAGACTCCCAAGTTGGA
6 PKHD1	GTAGTACATGGCTCCACCCAAC	GCCTGGAAGCTGCATAGTATTGAT
6 PKHD1	AGAACAAATGAGAATGCAGCATACCA	ACAACCACTGTCAATTATGTACGTGAT
6 PKHD1	GCCACGAGGGTTAGACAATGT	AGGAATGTATTGGATTCAGAATGAGCAA
6 PKHD1	AGAGAAGCTCACAAAAATTTGTCTTTGG	AGCTGTCTGGTGTGCTGTT
6 PKHD1	ACTTCTGCTTTTGCTTCTTTTAAGCC	ATTTGCTGTCTTGCCTGTGACTA
6 PKHD1	GCTTCTTTTAAGCCAACAGCACA	CCCGCCACAGGAGTCAATT
6 PKHD1	CTTGCAATTGCTTTTGTCCGGAT	GTCATTCTTACCTCAGTTTGTCTTTTCC
6 PKHD1	ACCTTCCTTATCTGTCTCCTAGCC	TGCCAGTTCTCCATTTGGGTTTT

6 PKHD1	CCCAAATATAAGACCATTAGTGCCTGA	CAATCGGCTTTCAAAACATGGTAAAGAA
6 PKHD1	AAAGGTTGTCCTTCCTGTGACC	GCCCTGTAATGTTTATGCGATCTAC
6 PKHD1	AGACTCCAGTCACAGGTGGTA	AACTGAAGGACTTATTTGGTGTCCATAG
6 PKHD1	GGGCATAATGTAGAAGTGTCTGGAAAC	GATGATCAACTTCCTTGCAATGTAACTT
6 PKHD1	CCAGAAGTGAAAGGAGCTACCAA	GGGAGTACCACGTCAGAGG
6 PKHD1	ACACAACGTGGCTTGCATTAAAAA	GCCTTGTCCACAAACACCAGT
6 PKHD1	AGTTAAGAAGCAACCCCCTCAC	AGTCTGGAGGCTGGCATCTAT
6 PKHD1	CGAGGTCCCCAGCTAGGTTA	GAGCCTCATGAAGCAGAGGTC
6 PKHD1	CCACCCTCTGATGCAGTCATAG	CAGGAGCTGCAAACATTGACATTTT
6 PKHD1	GGTTCAGCCTGTCTGTGATTCAT	CTCTGCCCCTGAATCAGCTTTAA
6 PKHD1	CTAATCAGCACAGTGGTCAGAGA	GTCACAGTCCTGGTCAATGGG
6 PKHD1	CCCACACAGGGTGACATTCCTATA	TGGACTTTGGTATCACATAGGAGTCA
6 PKHD1	CATGTTTCAGGGAGGGACCATT	GAGGTGAAAATACCACTAGCATCCTA
6 PKHD1	CCCTGACACTCGCTGGTTAG	TGATTTTGAGTTTGGGAGACCACA
6 PKHD1	GCCCTTGACTTCTTTCACAGTGAG	CCAATGCCATTGTTATGCTGTTACA
6 PKHD1	CATCTGCTTCTGACGTACTTGGA	CATGTGGGAGGAAGTAACCTCT
6 PKHD1	GGGTAAAGGCCTTGTTATAACCAA	GGACAGAACACCACGTCAGTC
6 PKHD1	GGCTAACCTGGCAGAGAATGG	CATGTGGTGGGACCATACTTACT
6 PKHD1	GGCAGGTCTGCTGGTCAATATA	AGAAATGGGTATGCTTGTTCTGGTAAT
6 PKHD1	TCCCCAGAAGGATGACTGAGTT	GGTTCTTCGCCCGTGGTC
6 PKHD1	GTGAAGCCTTTCCCCACCAA	TCGGTCCTGTGACATTGTGAAC
6 PKHD1	GCTTCAGAGGGTCAGACATACTGT	CTGTTGGAATGCATCGGATCTTG
6 PKHD1	ACCAGAGGGTCTCACCAACAT	GTTTCTCTGGTGACTCCCAGTTC
6 PKHD1	CGTTTTGTTCACTGTAACCTGCAA	TTGATGATAGTGAGAGGTCTCTCAAA
6 PKHD1	AGAAAGTAAGCAAGATGAGAGAGATAGGT	CCTGAGTTAATGTGTGTTTCTTTGTGTT
6 PKHD1	TTTCAGAGGACATTGATTGCCCTT	CCAGAGCATTTACATATGTCTCTTCCTT
6 PKHD1	GCTTATGTTTCTGCTCAGAGTCACAATA	GGAAAGATGGACGCATTGTGAAT
6 PKHD1	AGAATAGTCCCCTCTGATCACAGTC	TGGAGTTCTCTTCCCTTAAGTCAGT
6 PKHD1	AGAAATACTGGGAACATTCTGCCTTT	TGAGTAAGCAAAGTGGCAGCTT
6 PKHD1	TCTATAAGCCTCGACAGCCAGAAT	CCCTTTCAGCTTCTTGTTTGGTC
6 PKHD1	GGAGGCTTTCTTCTCTTGGGAAA	CAAGAGCAGTTGCTCAGATACCA
6 PKHD1	CAACCCGACCAAAGCTTGAATT	CATTTCCTCATGCAGCAATTTTGGA
6 PKHD1	CATAGCAGCAGCAGCTGATTTT	CCTGGTACTACTGGCATCACATC
6 PKHD1	AGACAGAGACCCATCCAAGTCT	AATTTAGTTGCTATTTGTGCCATTGTGT
6 PKHD1	TGTATGAAATGGCACTGCCTAGATAAAA	CTGTGGCCTGTATTCACCTCAG

6 PKHD1	CCTGAGGCCACTGATTTGGTT	CCACCAGCTCTTCTTTTGACTG
6 PKHD1	CGGCTTCACTTTGTCCTGAATG	AGCGTGGAGATAGAGAACATTACTCT
6 PKHD1	TGCCAAAAGACCAATAGTATTGTCTACC	TCAAGTCTTCATGGCCTTCATCTCTATA
6 PKHD1	AGTCTTTCCATTTGGTCCATGCT	GCCTTCAATGTCTGACTAAAGTACTTCTG
6 PKHD1	GTTATATTAACAGTGGTCACTCACCCA	GGATCTTACTCGTGTGCCATCA
6 PKHD1	GCTTGAATTGCTTGTAGCGACAT	GGTGATCCCATTTGAATATGCTGTTTTT
6 PKHD1	AAGCAACCTGCTTGATGATACCA	CCTTAGACTTCCCTGTGGACAGA
6 PKHD1	CAACTATGGCTGGCAATTGAATCA	CCTGTGACGTCTTTCCTGTTTTCTT
6 PKHD1	TGCCACACTCAGAACATTGCT	GAGAACCCCAGAATAAACACGTAATTC
6 PKHD1	CATGTCACCACAGGCAAATCC	GCCTGAGATTTCTTTCCCTTGTGTTA
6 PKHD1	ctgATTGAGAAAGAACTTTATGCCCTCT	CCAATCCTGAAATGTTGACACCATCT
6 PKHD1	GTGGGACTGCAGATATAGATGCC	GTGGATCCCCTGGATTTGTTGAA
6 PKHD1	ACAGTTGGGATTTCTGGGTGTC	GGACTGTTGACCCGAAATATACAAATTC
6 PKHD1	CCCCTACATGATACGTCAGGCT	CCAGCTAGTGATTTTTGAAACAGGTTTT
6 PKHD1	TGACAAGGTGGAATTTGTAGAACTACTG	ATGCAAGTTGTGGCTTTTGGTATTT
6 PKHD1	GCAGATCACAGCAATCACTCCTT	CTCGACACCTTGTCCTTACTCC
6 PKHD1	TGATCTAGCCGATAGCCCTTCT	GCCTGCCTTTCTATTTTATCTGAACTG
6 PKHD1	ACTCAGTGTCCAAATCCAGGTTT	GTCTAACATCCTTCTCCGGCTTG
6 POU5F1	CCCACATCGGCCTGTGTAT	GGGAGATTGATAACTGGTGTGTTTATGT
6 POU5F1	GCAGAGAGACATGGCACTCA	AGTATCGAGAACCGAGTGAGAGG
6 POU5F1	GCAGGAACAAATTCTCCAGGTTG	CAGACCCCTGTGATGCTGTTA
6 POU5F1	ATTTCACTCCATCCCACTGAGA	GGTGGAGGAAGCTGACAACA
6 POU5F1	CACCCTTACCTCCTGAAGATTTTCAT	CTCACTTTGCTTCTCTTTTGACTGG
6 POU5F1	CCTGCTCCTCTCCTGGGT	CGTGAAGCTGGAGAAGGAGAA
6 POU5F1	CTTGCCTCCTCCGGGTTT	GGGCTAGTGCCCCAAGG
6 POU5F1	AGGAGGGCCTTGGAAGCTTA	GCAAGCCCTCATTTCACCAG
6 POU5F1	ACACTGGTCCCCCTGAGA	AGCTCATTGTCTAATGTCATTCTCCTTT
6 POU5F1	CTCTTCATGGGTGAGGGTAGTCT	AGAAGAGGATCACCCTGGGAT
6 PRDM1	TGCATCAACACTTGAGTCTTGGA	GCCCTTGTTGCAAGTCTGACA
6 PRDM1	GGTTTTCTCAAGAAGGAGGAGCA	CTGTACTCTCTCTTTGGGACATTCT
6 PRDM1	AGTGGAGAACGGCCTTTCAAA	GTCAGAAGGTCTACCCAGAAAATACTG
6 PRDM1	ACTGAGAAAAATGAACTCTGCCCAA	GGCTCCCACGTCTTCTAAAGTC
6 PRDM1	CTCACATCAGAAAAGGACCTCGAT	GGGAGCGAGTGATGTACGTG
6 PRDM1	CCTACGGGATCGAGAGACC	AGTCCCGGTGCTCTTGAGA
6 PRDM1	GGAATACGGTGTCCCCTGT	GGGTAGTGAGCGTTGTACGA

6 PRDM1	GCATCGCCCATTTGCCATTC	GGTAAGGCACACTTTTGTCAGTTAC
6 PRDM1	CCTCCCGTTGGCAACTCTTAA	GCACACCTTGCATTGGTATGGT
6 PRDM1	GCGACTCCATTCTGGAGAGAA	CAGGTGAACCTTGAGGCTACAG
6 PRDM1	GTGCCACAAGAACTACATCCATCT	ACTGATGTCAAACTTCTCGATTTCTTCA
6 PRDM1	CTTGGAAGATCTGACCCGAATCAA	ACACTTTCAGGCCAGTTTCTTCT
6 PRDM1	GGCCGTGGTCAGAAAAGAGAA	AGGTACCAGAGGTAGTGGGTTG
6 PRDM1	AAGGCCTTTCCTTTCTCTTCCA	TGGTCGTTCACAATGTATGTACACT
6 PRDM1	GACAGAGGCTGAGTTTGAAGAGA	TCTTCTTGTCAATAAACCAGAGGCTTAC
6 PRDM1	GAACGAGACTTTTCTCAGATGTTGGA	GTTTCTACTGCGACATTAGCCaaaaa
6 PRDM1	CATTGTTAGTTGTATTACTACTTGACAGTCC	GTGCCCTTTGGTATGTATTCTTTACTCA
6 PRDM1	CCTGTTTAGGTTATTGGAGTGATGAGT	CCAAAAATATTTCCTGTTGGCGTTCTTA
6 PRDM1	TGAAATCTACACCAATGACACAGTTCC	GTGAGCCACCCAGCTCTTTTA
6 PRDM1	CCTCCCGCCAGCTTTCATC	GAGGCCAAAGTTGTTGATGCC
6 PRDM1	CGCTGTGAGCAGCATGAAT	CCTCCGGGCTCCATCTGA
6 PRDM1	CATGCTCAACCCCACTTCTCT	GCTACAGGCCTTGTCCTTCAT
6 PRDM1	CAAAGCTACCTCAGCAGCGA	TCGTACTTGATCTTGCCGTTCTG
6 PRDM1	CCACAGTGCCTTCTCCTTTACC	AGATTCATGGCTTCGTCGCT
6 PRDM1	CTTCCCTACCCGCTGAAGAAG	AAAGCAAGCTAAATACAAACACAAGCAT
6 PRDM1	TCTACCCCTCATGAAGTTGCCT	GGATTATGTACAAGCCACTTCCTACA
6 PRDM1	GTACCGGCTGTGTTTATTCTGAGA	GGAGAGTGTGCTGGATTCACA
6 PRDM1	AGAAAAGCAACTGGATGCGCTA	TGCAAAGTCCCGACAATACCA
6 PRDM1	GCCAACCAGGAACTTCTTGTG	CAGAACCGACATTACTGGCATTTTT
6 ROS1	CAGGCAATATTTCATGTGGGAGATTAGA	GTTGGAATATTTCTGGTTGTTACAATCCC
6 ROS1	CTTACCAAAGGTCAGTGGGATTGT	CTCTGTGTGCTTAGGTAGAGCTG
6 ROS1	AGCCATGCTGGTTACATTTTCCT	CCTCAATTCCCAGGTGGACCTAT
6 ROS1	CTGATCAGCCTTAAGTTATAACCCAAA	GCTCATTATGTCACCATTATCTCTTCCA
6 ROS1	acacacGCCTCTAAATTATTATTTGCAC	GCCACGGAGAACACTACTGAA
6 ROS1	CTTTAGGAGTCTGCTTTAAGTACTCACA	TGGAAAGCACCAGACTAAATGGG
6 ROS1	ACTTGGCATGGTACAGAGCAAAT	GGCTGTCAGTGTTGGAGAGTTA
6 ROS1	CTGTGTCTGTAGTACAAGGGAACTTT	CACGTGAAGCTATTGGTGAATGAC
6 ROS1	TGTGCACATGTTTTTGTTTGGG	CTCATTCCAACCTTGATGTGTTAAACT
6 ROS1	CACCAACTGAATCCACCACCAT	AGCCATTAACTGGCCATACAATTATGTT
6 ROS1	GTCTCCCTCCTGTTTGCACAT	AGAAACTACTGTAAACCTGGTGTTTGT
6 ROS1	CGAGCATAGCAGGTACTGTGA	AGGAATCAATGTTCTTGTAATGTGACTGA
6 ROS1	AGCCATTGCTCCACTTAACTCAAAT	TTGGCTACCAGATGTTCTACTACAGTA

6 ROS1	CGGTGCAAGGTGTGACTGATAA	GTTGCTCTGCATTAGAAACCAATATTTGA
6 ROS1	GTGCCAATATCCGTAATAACCCTGTA	ACAGTTTTATGGTCCTTTACTCACCTTG
6 ROS1	GGTCTGGGTTGAGGTACAAAGG	TGGAAGCCACAATATGACATTACGAT
6 ROS1	ACTCCAGAGAAGTTTGCAGATTTCC	ACTCTCTTGCGCTGATGGAC
6 ROS1	GAATCTTCACCTCTCTGGGATATTTCAC	GCTTTTCCAAGATTCACTGGTTTTTGAT
6 ROS1	GTGCCTTGAAATCCAGTCAATTGTAATA	GGGATTACAAATGAGAAGTGCAGTAAAC
6 ROS1	CTAAAGGGACAGCCTTGGAGAA	AGGTGAAGGCCCTAAGGAGTAG
6 ROS1	TGCATAACTGAACTGTAAGCCAAGATAT	AGCAGGAAAAACCCTTGTTAGCTT
6 ROS1	TGTGTCAAGGAGTTCGAAGATTCAC	ATTGAAAATCTTCCTGCCTTCCCT
6 ROS1	AGACGCAGAGTCAGTTTTTCCC	AATACCAACACAAACTTGTACACCCTAT
6 ROS1	GCAGGCAAGAACCTTTTGGTAA	AATTTCCAAATGGAAGGCTCACTCT
6 ROS1	CCACCAGACAGTCTAGTAACAAGG	AAGCCAAATGGACCTAAAGAATCAGT
6 ROS1	TGTGAGATTGCCAACTGATAACGG	TTCAGTGCTATTGCAGCATCCT
6 ROS1	AACTGAAACACAGAGTTTTCTGAGTATTGA	GGTCCTGCTTATGTCTGTAATATCACAA
6 ROS1	CTTTCTGGAAGTGAGGTGCTATTTTCT	CCATGTTAAAACTTCATGCAGCCA
6 ROS1	CAGGACCTTGGCTGCATGA	CACTTGCTATAAGCCTATACATTTTTGTGT
6 ROS1	CAAGTACTTTGCAAACACACATACCT	TCCCTAAAGCTGGAGTCCCAA
6 ROS1	TTCTAGTAATTTGGGAATGCCTGGTTT	cccgcctcTGAATATTTCTTTAATGTTG
6 ROS1	GTCAAGATGATACAGGTCAGTGTGG	CAAGTTTTCAAATCCTGTGGAATGGT
6 ROS1	CAACACCATACCTTAGAATGAGCACTA	GGTTATTCCAGATTCTGTTCAAGAGTCT
6 ROS1	CCATTCCACAGGATTTGAAAACTTGAA	TTTCTATGATTTCTATATTGGTCAATAATCCCC
6 ROS1	AAGATGCATAGATAGGCTCATCTGGTA	CACCCTCTATTAGTGCACCTCAAA
6 ROS1	CCCATTGTATGAATCAGCCACAATTT	CAGGGATACTTGTTGGCTGAATTTAATG
6 ROS1	TCACTAGAGTGGTGCAGCCTA	GAAGCTTATCAGCAAAACCAGTATCAAG
6 ROS1	GTTCTAGATGCATGAAAAAGCCCTTT	CTTATTCCTTGGTTCCAGAGAACACTA
6 ROS1	TGGAGCCTTCCAATTAAATTGCAAAC	GCTCTCAAAAGGTTAAGCAATTGTTCTT
6 ROS1	ATGCAAATGGGAATCCTCTTACACA	TCACTCACCATTGTCTGTTATTGTCTC
6 ROS1	CACCCTCATTCCTGTGTAGCTAA	TGTGAAACACGTCTCTCCAAATTGT
6 ROS1	TGATCATTGTCCCTTCATTATCAACACA	GGTGTTTACTACCTCCTTCTTCATTTCA
6 ROS1	GGGCACTCAGCTAGTCCTCTT	CTGCAGAGTGCTATGAAATACAAGG
6 ROS1	TGCCTACCCCAAAATGCTTTCT	AGCATTTATAAGTCCAGAGATGAAGCAAA
6 ROS1	CAAAGCTTTCATTTATGACTCCACTGTT	GGTGGAATTTAATGACCCAGTGCT

6 ROS1	GGTCTTTGGTCGGGTTCTTGAG	AGGAAAACTGGCAATAACTCAGATTCTT
6 ROS1	TTTGGAGAACTTGCTCTCACAGAAA	CAGAACTGGACCTATGAGGTGAAAG
6 ROS1	CAGGAGGGTCTTGGGTGGATA	GAGAATCCTTGGTTTGCCCTTG
6 ROS1	CCTTGTTGCCACCACTTCTCA	GCAGTGTACAGTTTTAAATAGCTGCCTA
6 ROS1	GACTTACCAGATTAGTTACACACGACTT	CATCTAAAAATCTGCCATTGGTGAAGTG
6 ROS1	GCTAAGTAGCTGCTTCTGGTTGA	TCTTGTTCAATGGAAGCCTCCTG
6 ROS1	ACTCACTGGCTCCTATGGCA	AGTCACATAGAAGAATTTGGGTTTGGT
6 ROS1	AGTCAAGCGGACTTAAAACTTCTTACC	GTCTGCTATACTGATTCCTGACTTGTTT
6 ROS1	TGAAATACCTCAGAGCTAGAGCGA	TGTGGACGTTTTCAGCAGAAAGA
6 ROS1	CATCTCATTATCAGCTGTGTAGCAAAC	CTCACCTCATAACTCTTCTTGGTAACAA
6 ROS1	GTCCACACAACTTGATTTTGATCCATAT	TGCCAGATATTAAATCGAGACTTTGCT
6 ROS1	AACTCTCTATTTCCCAAACAACGCT	GAACCACATGCAGACAAAGATTTCTG
6 ROS1	GCAGTAAGCCACTTGTTTTTCTTGG	ACCGAGAAGGGTTAAACTATATGGTACT
6 ROS1	CCTTGGCCACATTCTGTAGCA	TTTCACAGGTGAAGATGGCGAT
6 ROS1	GTATGTTGGAGTAGGGCTGGT	GATAAAGCGTTTCTGTCTCTAGCTTCA
6 ROS1	TCCACAGTGTCTGAAGCTAGAGA	AGCTTTGATATTATTCTTGTTAAAAATCTGCTT
6 ROS1	GGATGAGCCAAAGATGACCAAGTT	TCCCCTTTGCTGATGTGAAAAGTT
6 ROS1	ACAAGAAAGTCATTGTTTTCACAAGCAA	CGTTAAAGGACTTTGCAATCAAGCC
6 ROS1	GAAGTAAATGATTCGCTTGGCTTGT	TGAGGTTTCTTTTGTTCCTGTGTCA
6 ROS1	TCACTATTCCTCTTTAACTTCTCGGACT	GCTGCCAACATGTCTGATGTATCT
6 ROS1	CGGACTAACCAGTTCATCCATGAT	CCAGCAAATTGTTTATTTCTCTGAAGGA
6 ROS1	ACATCTTGTGAAAAGACAAAGACCACTA	CAAACCTCACATGGTATGGCATC
6 ROS1	CCTTACTGTTGCCCACCCTTT	GGGTGACAGTGCTTATAAACGAAGA
6 ROS1	GCAGCTCAGCCAACTCTTTG	CACTTCAAATGCACTGTTAACATTTCCT
6 ROS1	CCACAACAAGCCCATAACTTACC	ACAGGAATTTCAGGACAGTATAGCTCT
6 ROS1	GTCATTGAAAGGGTATTGAATCTAAACAC	GCCCCAAAACATCTCTGTCACTT
6 ROS1	CAGATTTTAACCCCTGTACCTGTTTCA	TTTAGTTCTTGGCTAGTGAACAACACT
6 ROS1	CATCCAGAATGCCATGCTTTAACT	GAAAGGCGACGTTTTTGTGTG
6 ROS1	GAGATATCCGTCCCATTCAGCAG	TGTTGAGGTGCAGATGTGATTTGT
6 ROS1	TGTGCACACCATACCTCCA	TGTGTCCAGACCGTCCTATGT
6 ROS1	CTAGGTATGTGTGCCATTTACACTGT	CTCCCTCAGTGATGTCTTTTCAACT
6 ROS1	CTGTCATTCAAGTGAACAGAGGACTT	AAACAGCTAGTGAACCACCATTTATCA
6 ROS1	TCGGAAAATGTACTCAGTGAAGGG	CATCTTCTCTGTTGAGCCATCCAT

6 ROS1	CATCTGGGACTCATGCCTTCA	GGTGGAATTTAGGTGGAACAAACCT
6 ROS1	GTATATAGCATCCAACCGAAGGCA	CTACCTGTCTGCAAATTGTGTTGTTT
6 ROS1	ATTTTGTTAACACCCCATTTTCATGCTT	CTCTACCTGTGCCTCTACTTTTCAG
6 ROS1	GAAGGTATAGTGGAGTAGGGTAAGCA	GAAATAGGTCAGAAAACCAGTGTCTCT
6 ROS1	attctgtCAGCATTACTCTGTGTCC	GGACTCGCCAGAGACATCTATAAAAAT
6 ROS1	CTGATTAAATCTGGCTGGTTCCAAAAC	TCTGAAATTTCCCAGAATGCACTGA
6 ROS1	AGAACAGCCGACCACTATAGTACA	ACCACTCCAGAAACACACACTAATT
6 ROS1	GGCCTTCCCCTCTCTTTCTATAGTAATC	GGCAGCTAGAAATTGCCTTGTT
6 ROS1	TGGACTGGTATAGTCTTTCACGGA	ACCTACTAGGGCTACTTCTTTGATTTCT
6 ROS1	CTAAGGTTTTACCACTGCAGCCT	GGAGGGAGGAGACCTTCTTACT
6 ROS1	CCATCCGGGCTTTACGCAAATA	ATATCATCCAGCCTGTGTCTTTCC
6 ROS1	AGCTAGTGTGTAGACAGACATGGT	TGGAAGTCCAAAAACCTGAAAGGAATAT
6 ROS1	CCAAACCCTAGATTATTTGCAGCTACTA	ACAGAACCAGAATTTAAGGTGGAAGATG
6 ROS1	AACACTACTGCAGGATCCATTAAATGT	GCGTTCAGTCACTAAGAAGATCTATGAA
6 ROS1	ACAAGTTTTCTTCTCAATCAATTGCCTTT	CCTCAGTGATGGGCTCCTGTAT
6 ROS1	GTGAATACATTGACTGTCTTGAACCAAC	GACCTGTTCACAAAGTTATGCTTCAAA
6 RPS6KA2	GCTCTGTCCAGGTGTCCTTTG	TGAAGGAGATAGACATCAGCCATCAT
6 RPS6KA2	GGCGGTGTAAATAAGAGGGCTT	AAGCATCTGTTTTCTCTCCCTTTCTT
6 RPS6KA2	CCTTCTCAAAGCCCTCCTTCAC	CCCAGCTCGTGGTAAACTCA
6 RPS6KA2	TGAACTCCCTGACGGGTCT	CAGGAAGTCGCGCTCCAA
6 RPS6KA2	AGAGCCTGTCTTTGCGGATAG	GTCTGCGACTTCGGCTTTG
6 RPS6KA2	CGTGTAGCAGGGTGTCATGAG	GCCTTCTGTCTTGCAGGTTGTT
6 RPS6KA2	ACTCGGCTTCAGGTCTCGAT	GCAGGACCTGCTAGGAAGAC
6 RPS6KA2	GGTGATTAAAGTCCACCCATCCT	CACAAAGTCCCAGTTCACCCA
6 RPS6KA2	CAAACACAGTTACCTGCACGAT	CATCTGAACTCTGCCCCTGTC
6 RPS6KA2	CTTCGGAGTGATTGCAAGGTATCA	CGATGCAAGTGCTCAAACACC
6 RPS6KA2	GGTACTCTCTGTTGACCACCCA	GCTCTCTGACCCAGCACAAAT
6 RPS6KA2	CCCTGGCTTCCTCAGAAATCAT	GGCCAGACGATACCCCTGA
6 RPS6KA2	CGATCCGCGCCAGAATCTC	GGGAGAAGCAAAGTGCTATTCCT
6 RPS6KA2	GTTGCCCTGGGTCCTCTTTA	CTGCCTCATGAAAGCCTTTATCTTTTT
6 RPS6KA2	GGGCCTGAGGTGTTCTGTTTAG	GCTCTGGTTTCTCCCACGTT
6 RPS6KA2	CTACCAACATGGGCCAGGAAAATA	CCTTGGCTTTAGACCATCTCCAC
6 RPS6KA2	TCTGTAGATGATCCCCAGGCT	GAGTTCTTTAAAACACACCAGCCATAAA
6 RPS6KA2	TCTGTGAGCCCACGAGGAT	GCCGCCACCTACTTTGCT
6 RPS6KA2	GTATGTTCCCTCCACCCACTTC	GTTCTGATGATCATTTCTGTGTTGTTGAC
6 RPS6KA2	ATTGGCTGCAGAGTTCGGAT	CTACTATCAATACTCGACCAGCAGAAAA

6	RPS6KA2	CCCTCGAGTCCCAGACAGAA	TCAAAATGCGGGTCTCTGTCC
6	RPS6KA2	ACGGAAGGAATTCCAGGACAATC	TGACGTCCTGTGCACCATC
6	RPS6KA2	GGAGGTAGTCCATGGTCTTGGT	GCAGGTCTATGATGATGGCAAGTT
6	RPS6KA2	TCAGCTCCATTACCAGGTACACA	CCTCTGACTTCCCTCTCCTGTC
6	RPS6KA2	TTGTGTCAGTTGAAGAAACAGAAGGA	CCATGTGCTGTGTTTGTTCCAG
6	RPS6KA2	TGTCTCCTCCTGACACCTGTTT	TGTCCTGTCGTTCCCTCCTA
6	RPS6KA2	TTTCGGGACCTCTGAACCAAC	GCGGGACGATCGAGTACAT
6	RPS6KA2	CTCAAATAACGGAGTAGAAATTGCAGTT	CTGATGTTCCGTTTTCTCTTGCTAAG
6	RPS6KA2	CACCTTCTGGGCCCAATCTC	TGGCAATGCTAACCATCTTTAACATCTA
6	RPS6KA2	GTGTCCTCGCCGGTTCA	TGAGTGTGGACCCCCGAA
6	RPS6KA2	GGTCTGTTTTCAGTGACCTTGGTT	GCGAGCTCTCTTCAAACGGA
6	RPS6KA2	GGAATCTTACCCAGCCGGTT	GGCCCTCTTTATAGCTTGTCCTT
6	RPS6KA2	CAGAGGCAGCAGCTCTTGATA	TGAACTCACTGTGCTCTGTGTTG
6	RPS6KA2	CCCACATACCACACGTGCTC	GCTACGAGATCAAGGAGGACATC
6	RPS6KA2	CGCTTGCACACTGAGTAGGAG	CCACATTCTGTGTGCGTGTAG
6	RPS6KA2	ACCGATAGAGGGAACCACTGT	GCCCTGTCTTGCTTTCCTTGTATTAA
6	SGK1	ACTGACTCCCTTACAGTTCTCCA	CTAGTGGCGAGCTGGATTCTAAAT
6	SGK1	TGCCCTTTGGGCTTCTCTATACT	GCACCTGTCTAAATTAATCTTGGGTTTC
6	SGK1	GCCCCATCAAGCACATCTCA	CTCCCTGGATGAGGATAGAAAAATGATT
6	SGK1	AACCTTGCCTTTGTGTAGAGCTT	TGTAATCATGCCAACATCCTGACC
6	SGK1	GAAGGTTCTTGGATCGGGCTT	AGCCTTGGAATCCTTTCTCTAGAACTAT
6	SGK1	TGCATTCAATAAGGGCAGGGA	CCTCTCCAGCTGAAACCAAATATTACAA
6	SGK1	CAGGAGGTGTCTTGCGGAAT	GTGTGCCATATGAAACTTCCAATTAAGT
6	SGK1	TCTGGTAACATTCTCCCCACCA	TTTCCAGACTGCTGACAAATTGTACT
6	SGK1	TCTCCACCATTAATGTAGTCTAGGACAA	ACATGCCACTAAATCTGAATCGTCAT
6	SGK1	TGAGAATGTTTACCGCTGGCA	CGTTTCTTTATTTCTCCTCCTTCATCCA
6	SGK1	AGGCTAACTAAAACATTCGGAAACACA	CCCAACTCCATTGGCAAGTC
6	SGK1	CGCATAGGAAAAGCCTAGGAAAG	TGTTTTTCCCTTCCCGTCACA
6	SGK1	CCTGAAGAGCTCTCAGGCTTA	TTTGCAACCCAGAATGACTTTGTTC
6	SGK1	GCATGATTGTACATCTCTCTCTTGGA	AATATTTTGCTAGATTCACAGGGACACA
6	SGK1	AGAGTCCGAAGTCAGTAAGGACAA	CAGTGAACCTAAAATGCCTCTAATACCT
6	SGK1	GCAATATTGTAGGGAGCAGGCA	CTGTTTCCCTGGTCTGTTTTTCAG
6	SGK1	CCTCAGGCTTACTTCAGGCAAA	TGAATTCCCCACGGTGGAAA
6	SGK1	AAAAATTTCCACTTTGCGTCTCCTG	TTGAGCGCTAACGTCTTTCTGT
6	SGK1	TCATTTTTCTATCCTCATCCAGGGAGA	GCTAAACCATCTGACTTTCACTTCTTGA

6 SGK1	AAACTGCCCTTTCCGATCACTT	AGATGTCTTCCTTTGAAGCAATGGTATT
6 SGK1	CAAATCCTTTGAGGCATCTGCAAAA	GTAGCAGGGAGGCTTATTCCAG
6 SGK1	TGGGTATACTCTGGCAGAAATACAAGA	TCAAACATGCCTGGGTGAACA
6 SGK1	TGGAGGTAGAGCCCAGTTATGG	CCCTTCCTGTACTTAAATTGCCTTGATAT
6 SGK1	CAGTCCGCTCAGCAGGAA	CCGGTCGCTCTCGTCTg
6 SGK1	GAGAACCCCTCTTTGGAAAGCA	TGGATCAAGAGCCCAATGGTC
6 SGK1	GACTCTGATGCTTGTCCACACT	TTCTCCTTTCATGAGCTCCCATC
6 SYNE1	GATCCCTTTTCTGCTGCTGTGTA	CTCAGCACTGACATCCAGACC
6 SYNE1	GAAACACAATGAAGAGAAAATGGAAGCA	GTTTGTCCTTTCCCTTGAGCAG
6 SYNE1	AGCTTTTTGATCTGGAGCTCGAT	GAGTTGAGGATGAAGCAGAACCT
6 SYNE1	GTTAAACTGCTGCCACTTCTGG	CTGCCAACCTATGTTTAAAGCATTTCAT
6 SYNE1	GGGAAACTGTTCTCTTCCATTCTC	GAGGATAAACCTGTTGCCACCA
6 SYNE1	GCCTGCAGCTCCTGTATGTTAC	TTTCAAAGGAAGCTGTGGTACAATATGA
6 SYNE1	AGCTAGAGAGAGGTAGGAAACACTTT	CCTCGTTAAAAGCAGGGAACAG
6 SYNE1	CAACATCGCTCTCCTCCTTCTC	AGTGGAGCCACTTTGGTATCATTAC
6 SYNE1	CCCCTAGTGAGCTACGCTGT	CTAACTGGGATCGCTATGGCAAT
6 SYNE1	GCTTGCAGACTAGCCACTGT	GAAGAAGCTGAGAATGTGATGAAATTCA
6 SYNE1	ACTGAGCGGTGGTTTCATTCA	TGATGGATACTATTGCACACCAATGT
6 SYNE1	cCACCCGCACTAGCCTTATTAA	GCTGGCTAAGTAACACCAATAAAATGTCT
6 SYNE1	TGAGTAAAGCCACCTTCTGTACCT	GAACTCTGTGAGTGGTTGACTCAA
6 SYNE1	ATTGTTTCCAGTCTGTGAAGTTCCTT	AGCCTTAATGAATACCAGCCCAAATTAT
6 SYNE1	CAGAAGTCGTTTCCCATCATCTAATACT	AGGTAATGGGAACATTGTAGGTACCA
6 SYNE1	TCCATTCTGAGAAACTTTGCTTTCCAT	GGTCTTCTCTCTGATAATCATTTTGCCT
6 SYNE1	GATGTACCCCTGCAGCTGAAT	GGCTGAAGATGGACTCAAAGAATTTG
6 SYNE1	GCCTCTTTAATTCAATGATTCCTGCAT	ATGTTTTACATGGAGAGGAGGAACAC
6 SYNE1	ATTTTGGTGACCCATGGGAGAAT	GCTCGATTTGTGCCGTCAGT
6 SYNE1	CCTTTGCAAGCACAGGTTGTTAG	CCGTGAACACCATAATAGAAAAGGTGAA
6 SYNE1	cAACCCAATCCCACACGACTTA	CAGCGATCATCGAGGAGGA
6 SYNE1	GGCGATTCCTCCTTCTTTGTTAACTTA	AGGTCTTATGTCTGTGTTGCTCTCTATAT
6 SYNE1	ACCGTCGGAGCTCATCTAGT	GCAACAACAATAGCAAAATCACATGAGA
6 SYNE1	AATAAGGCTGTGATATAAATAGTGTGTAAAGT	CAGGAAAGGAGCTTACAGAAATTTGG
6 SYNE1	GGTGGGTGACACTCTTTTAATAATTGGT	AGATCCTGTTATGTCATCTATGGGTCTT
6 SYNE1	CATAAGACAATCGCTAAGGTTACTGGT	CATCCGCATCCCAGGAGTTC

6 SYNE1	GGCTCCAATCCCGGTTCATA	TCAATACTGTGCTTTGCACTCACA
6 SYNE1	AACCCTTTTACGGAGTCTTTCATCTC	AGAAGGGTGAAGCTCTTTTGGAAC
6 SYNE1	TTTAAAGTGACGTCCTGCACCA	GTATGTATTCATCTTGTCCACGGAGAT
6 SYNE1	AAAGACTTTTCTCATTAATGTAATTTGAAGCA	AGATTTGACAAGAGCACAGATGGT
6 SYNE1	GTTCCTCCCTCTGAATCTGCAA	CCACCCCTCTGTGGTCAT
6 SYNE1	GGACCCACTGGATCCTTACC	GCTTCTTTGAATTCCAAGTGCAAGAT
6 SYNE1	GTGCTTGGCTTTCATCGTCAG	CTGCAGGAAGATGAACGCTACTA
6 SYNE1	ATGCTGCAGACGAACTGTTCT	CGTTTTCAGATACAGCAAACCGAAA
6 SYNE1	TGGGAGTTTTGCTGCGAATGATAT	TGTCCAGTAAAGATTCTTATGGTTGCC
6 SYNE1	GGAAACAATTTGACAAGTATGATCACACA	CTGCACTTATTCCTCTTTGCTTTTACAG
6 SYNE1	GCAGCACAGTCTAACTCAAGGG	ATGAAAGCAGCAATCGCCTCTA
6 SYNE1	CTCCTCTTTGCTGGCAGATCA	CCTGGACACTATCTAAAACCTTGCAG
6 SYNE1	TTAACTTTAGTTCACATCAGCACTGCTA	CAGTGGTTGGTTAATGCAAAAATCAC
6 SYNE1	GAGGTATATCAAAACACTTGGCGGTA	CCATTAAATTTTTGCGTCACCTTTTCAG
6 SYNE1	TTCTGTTTTCCCAAGCCTGTCA	CCAGCTAGAACTCCAGGATCTACAG
6 SYNE1	CTCTTGGATGGCTCTGTATCGTTC	TGCATGAATGGCCACTTTATTTTTCTT
6 SYNE1	GAACAAGCCAAGAGCTATTTTCTTTCAA	TTCGGTGAAAACCACAGCAAAAG
6 SYNE1	CCGTTTGCTCTGTTTGTTGAATGT	TTTCACTCTGATAACTGCCTTTTCCA
6 SYNE1	CTATATCCCCAAGTAAAACTGACTCCAA	CAGAAGTTAGCTAAAGACCTCACAACT
6 SYNE1	TGTCTTCGCTTTCAGCTTAGTTAGAAT	GGCTTGTTTTGCTGTTCTTACTGATTTT
6 SYNE1	GGGAAAACAAGGTAGCTCCTGAA	ATACAAGGACTACTCCAAACAGTGTG
6 SYNE1	CTACAAACAAAAGTGCCACTGTGA	GCAAAAACTGGCAAATGTGTTTGA
6 SYNE1	TTTGTTCTGCTACGGGCTGT	TGAGTTTTCCCGTTGCTTTCCT
6 SYNE1	CTGCTGCTGAACTGTCGAAG	TGCTCATGAGTGTTGGTTATTATTTCCA
6 SYNE1	ACAGCCTCTCCCCATAATTCCTT	GCCAATGTGCAGACCACCT
6 SYNE1	TGTTGGTGTCACACAGCCTATT	TGATGGATGCCCAATACAAGATAATTACA
6 SYNE1	TGCACGTTTTGTTTAAGTTTCGCTT	TGGTTTCACTTTTGCAGGCAATG
6 SYNE1	GGTTAACCCATCTGACGTTCAA	TCTCTGCCTATATCAGTGCTGATGAT
6 SYNE1	TTTGGTAATGAGGTGTGCTGTCTT	CGGAAGTTAAGAGGAAATGTGTCATGTAT
6 SYNE1	CAAATTAGCATTCAGCAGTTGTCAGT	CTGCAAAGCATGTGGTTCTGG
6 SYNE1	CTCTACGCGTTCATTAAGAATGGAGAT	GTCCGTGGGTATTCAAGTTTGTTTTT
6 SYNE1	TCTGTCCTGAATGGCCTTCAC	GCCACCAGAACCTACTTAGAATGAC
6 SYNE1	GCAGCTCCGGAGTTTCTCTTT	AGAGAGTCTCTTGATAAGCTTTCCCA

6 SYNE1	CGTGGCCTTCTTCACTCAGAA	GCTATGAGCCTGTGTCTGGTTA
6 SYNE1	GGGTTTTCATGTAAATCTGGACAGT	CGTGCTACAAATCAGCAGTCAG
6 SYNE1	TCTTATCACTACGCTTACTTTCCACATC	CCAACTCCTGGAAAACTAATGAGACAT
6 SYNE1	TGAGCGAAATAGTTACAGTGACTTTCC	GCTGTTCGAAAGCTCCACAAAC
6 SYNE1	CTGTCCTGACTCACCTTCCATT	CCCACTTTCCCCTTCCATGTC
6 SYNE1	TGATGGGTAAAGAGAATGAGGAGACTT	AAATGGTGGAGACTCAGATCAATTCTG
6 SYNE1	TCGTTTCCTGAACCCAACATTTCA	TCCTTTCACTTCACTTCTGTTTTATCCC
6 SYNE1	GCAGACCAGATATAGGTCAACCTAAG	ACCCCCTGTTCATTGCAAACA
6 SYNE1	TGTCTCTTCACATTTCGCAATCATTAGA	GTAAACGGAAGGTGACCACCA
6 SYNE1	GCCTTCTTAGCATTTCCTTGGATCT	TGCTGGCTGTTAACTCACAAACA
6 SYNE1	CACTAGTGTTGGGTCTCATGCT	CAAGCCTTCGAGGAGGTTTCA
6 SYNE1	GGATTTTTGTACGAGAACATGAGCAA	GAAGCTCATGGAAGACCCAGAC
6 SYNE1	TGATAACACAGCCTTACCTGGGA	GCTGCCTTTGTTTGCCTACAG
6 SYNE1	CCTGCTAGTGTAGTCCTTCCACTT	AAAGGTTACAGCTCATCGAGGAAC
6 SYNE1	GCACTGGGAGTTTCACACAGA	GTGGCTTCTCACAGGGATTATAAAAAC
6 SYNE1	CTGAAGGTCTGGCGGTGTT	GCCAAATATAGCATGTCAGTGTTTTTGA
6 SYNE1	GCTATTTTGGCTGGCGAAGTT	GGAAGTGGGAGCTAGAGCTCA
6 SYNE1	TGGCTCTCGTCCAGTATCTCC	TGGCTTATTCGAATGGCTAAGATTGAAT
6 SYNE1	GCTTAAGTCAGCTATTTCCCACGAA	TGGAAATCAGCTCCTTCGACTAAAAA
6 SYNE1	CTATCAATGCGCGACAGCTC	ACCCTAATTTGAACATCGCTACTTGT
6 SYNE1	ACTGTGTCTCAAGACTGCATTCAA	CATGTAGCCTCAAACAGATGGTTC
6 SYNE1	CCATCTCTGGTTGCCTTCGT	CTGATATGCTTCTCTGTGGCCTTAG
6 SYNE1	CTGGGACTCAGATTTGAACACACT	GAGTTCTTGATTATGAAACCTTTGCCAA
6 SYNE1	AGGCCTCCAAAGCTTCTAAACTC	CCACTTGCCCTTTTACCAGACTTTATTA
6 SYNE1	TCTTCACAAAAGAGCCACAAATCCT	TCTGGTCTGGCTCACAGAGAT
6 SYNE1	TGTTCAATATTAGTGAGCTGCAGATCC	AATTCGCTGTGCCATCTCCATAT
6 SYNE1	CCTCAGACAGAGGCAGTAGAAC	GGTAAATTGTCACTTGACCAAGCATT
6 SYNE1	GTCACTCTATCCCAAGATTGTTTTACC	GCCATACTGTTGCTGTTTTTCCT
6 SYNE1	TTTGGCTGATGAAAGGCAGACT	CAAAGCATCTGAGATTGAATACAAGCTG
6 SYNE1	ACCGGTCGTTGACCTTTCC	GCTTTCTGACCTCTGTGATTGCTATTTA
6 SYNE1	CTCTGCATGCATCTGTCAATGAGTA	AATTCTCAGATAAATGCAAAGCACTGAC
6 SYNE1	TCCTGGTATTCTGCTATCCACTGT	CAGAGAGGTTAAGAAAGGAGATTCATGAT
6 SYNE1	AGTTCCTTCAACTGCTCCATGTG	TTTGCTCCTACTCACTCTCTTTTTCAG

6 SYNE1	GCAATGAGAAGTTTAAGAAGATGCACTT	TCCCTTTGTTTTTCATGTCAGTCCAT
6 SYNE1	GTACTAACCGGTGATCCTCTGTG	CAGGTTCTAAACTGACTACCTACCTCTT
6 SYNE1	TCTGTAAATGAAATGCAACCCTGCTA	GGTTTGCTTCTTATGCTGGAGAACA
6 SYNE1	GGGACAATTTCATTTTTCCTTCTGTCAA	TGGTGGCAGTTGAAAGACATTTAAGT
6 SYNE1	CCCTTCTGTAGTTATCAGGTGAGTAGT	CAGTTGGATGAGAGATGGAGAGATT
6 SYNE1	ACCAACCTTTTGTTAATGATCTGTGGTA	AGCAAACTTTTGCAATTGAAGGAATCA
6 SYNE1	CATCTTTGGAGGCTACTGAGAACAA	CCTGTTAGGATGAGATCCGTAATGTCT
6 SYNE1	GCCTCACTCAGAACTGTCTGTTTA	CTGAGAATGAAGCCCAAAAGCAG
6 SYNE1	TGGATAATTTTGCCAGCTCATCTCC	ACTGTGTTCATCAAAAGTGTTCTCTCAT
6 SYNE1	GTGCGGTGGAAATGCCTAAG	GGAGATGGCAGAAAAGCTTCGT
6 SYNE1	GGTAGGACACTTCAACCAACCATTT	CAACCTGAAATTGACACTCCTCAGTAAT
6 SYNE1	CGAATCACTCCCTGCCATTG	GTATACATTGGCTGATTGCGTAACTG
6 SYNE1	AAAGGTAGGCGCCTCTGTTAAA	CAGGTCTGTTAACGGGATTCCAG
6 SYNE1	GTCCTCTAATTGATCAGGTGGCA	GCTTCTTAGCCCTCTGTGAAACAT
6 SYNE1	CACAGAATGCTTCCATGTGGTTG	ACCCTGCTGACCAAAGAGAAAG
6 SYNE1	CAGCTGTAACTTTGGCCTGGAT	GGAGTTTTTGTCAGAAAGTGAAAATGGA
6 SYNE1	GTGAGGAGGCCACTTTACAGTT	CTGGTATCCGAGTCTTGTGAGG
6 SYNE1	AGTGAGCGTGGACTGCAAG	AATGAATACATGAGTGTGATTCCACAGT
6 SYNE1	TGAGGCCCCACTACAGTTCA	TGATGGGCAACGTCTTCTAGAAC
6 SYNE1	CCTACCTGTCATCAACTTGACCTT	TGGAAAGTTATCAGAGAACCGATCACTA
6 SYNE1	CTAATAGTGTGTCCCTGGAACCAA	CACCTGTGGCTAATACAGATGCT
6 SYNE1	TGGGAGGTACCTGGTTAACAGA	TGGGCTACATTTTACGTGGCA
6 SYNE1	GTTTCTGCCTTACACGATTTGGATTT	CAGCAGTACAGAAATTCTTGGAACAG
6 SYNE1	GGCTTACCCAGTTGGCCATT	GATCTCGGGAAACCTGGAGAAATT
6 SYNE1	ACAATGGGAGGAAGTAGTACAACGA	CGAACTCGCTGAAATCCAAGAGA
6 SYNE1	GCATGCTGGCTGATTTCCATT	AAGGCAGCTACATCCTGACTG
6 SYNE1	GAGAGAAGGAGTTGCTTGCCA	CCTTATGGGAAGGAAACGATGAAAGA
6 SYNE1	TGAACTGGATGCTACGCACTC	GGGCCGGTTGTTTATGTCTAAA
6 SYNE1	CCCCTAACTTCCGGCTCCTA	GAGAGATGTGCAGCAGCAGAT
6 SYNE1	CTCGTGGCCTCGGTCAG	TGTTGATGTCCCTACTTAGCTGTTTAGA
6 SYNE1	GCACTTACCTCACTTAGTAATATATTTAATGCT	GTGGAAAAGTTTCTGGCTTGATTAAAGA
6 SYNE1	ACCAATAAGGTTCATGCTCACCAATTAT	TGAACAGGAGATACTGGATGCTTTG
6 SYNE1	CTCTGTTCTAGGCTCTGCTTTTTG	TTCATTGAATCTACCATGTTGGTTCTT
6 SYNE1	ACTGCAGTCTACGTTGCAACA	GCTATCTTCTAACCATTCCTCCTTTTCT

6 SYNE1	CAATGTGGCGGCTTGTAACA	TCATGTTGTGTTTCTAGACGTGGT
6 SYNE1	CTGCTGGTCCTCATCCAGAT	GCCCAACGCAAACATCTCTGATA
6 SYNE1	GGGATTGCACTTAATGTGTATCAACT	TGAGACAACATTGCGTGATCTTCA
6 SYNE1	TTGTTTTAGGATAAAGGAGGCCTTACC	GTATCAGAGAGACCTAAAGGCATTGAA
6 SYNE1	CTTGTTCTTGCCCCAACCAAAC	AGTGAATTACAGCACACTGATGCTTTAT
6 SYNE1	ACTCCTCCAAGTTGGTGACTGA	AGCATCTGAATTTAGATGACAAGGAGTT
6 SYNE1	TTGCTCTAAATGACTCGTCTGTTCTT	CTGATGCAGAGAGTACAGCTGTC
6 SYNE1	ACCTCCTGCCCAAAGCTCTA	CTGAGTTCACCCAGGCTGAC
6 SYNE1	GCCCATTCATCTGCGACAAG	TTTTGTTCTTTTCAGGAGCTCCAGA
6 SYNE1	TGATGGAGAGGATGATGGCTTTG	ccatcctGTTAAGGCCAGTTTAAATT
6 SYNE1	ACAATGTGACTTGACACGGCTAT	CTGAAGATTGATGTGGAGAAGAATAGGTT
6 SYNE1	TTCTGCATTCTTCTACAGAGGCTAAG	TGCCACTTCAATTGGGAATGTACATAAT
6 SYNE1	AGCTGAGTCACTGTCATTGCAT	CCTATGTAGCCCAGTTTCTGAAACATTA
6 SYNE1	GCTTGCATTGTGGATGTCAGGA	GGTGTGATAGCTAGATACCAAACTTGT
6 SYNE1	TTTGACTGGATTATGGCCCCAA	CCCTGGAGAAACTACGAACTTTCAA
6 SYNE1	TTCTGGTCAACAATATGCCACCA	CGTTCCACAGATGGCAGAAAAC
6 SYNE1	GGAGAGACTGCGAAAGCTTCTTT	CCCCAGTTTCCTACATCAAAAACATCTA
6 SYNE1	GGTGGTTATCCAGGTTGCTGAT	CCATGGCTTCTGTTTCTCTCCTA
6 SYNE1	CTGTAGCTTTGACAAAGATCAGGAATCT	CAAATTGAGGCTAATGGAGCAAAAGT
6 SYNE1	TGTTTTGAGCCATTCATCTACTTGCT	TGATCCCATCAGGTATCCCACA
6 SYNE1	CTCTAAAGCCCGGTCCTCTG	ACATGCTAACCCATGCAAGTGT
6 SYNE1	ACTCCAACTACCAGCTGGCTA	ACGTTTTGAAGATTGGCTGAAGTCT
6 SYNE1	GGAAAAGCAGCTGTCCTTTCTGA	AGCGAAAACAGTCTCCAATAATCTGAAT
6 SYNE1	GCCAGGAGGACAGATATTCCAT	CAGAGCTGGCCAAGCCAATA
6 SYNE1	TGACTTTCCTTTAAGCTGGCAAACT	CCTGGAGTGGGATCACGAC
6 SYNE1	TCTTCCGAGTTACAGGAATCGTAGA	CTCTCGCTGAAGTGGGACAT
6 SYNE1	CAGGTCCCGACTGAGGTCATA	TGCAGACAGCCTGCTTTCTC
6 SYNE1	GAGAGATTGGAGGAAGGCTGT	TGACAGGGTTGCCTTGACTT
6 SYNE1	GGTTTCTTGTCCTCACTCTGCAT	GAGAAAGGCAGTCAAAGTGTTCAAAA
6 SYNE1	GGTTTGAGAGCATGCTTTCCAAT	CCCTACAAGATCTGGCTGACC
6 SYNE1	CACGTTGCTCAAGGTCACAAAC	TCTTTCTTTCTCTCTCACTCTGTCTTGTA
6 SYNE1	TCTTCTCCTGACCAGTTTCTAGCA	GAGTCTTGTGGTCTCTGCTTCTTAG
6 SYNE1	AAGAAGCCCATGATTCATCAGCA	GATCCCCGAGGATGTGGTT
6 SYNE1	AGAGCAGCATGACAGAGAGGTA	GAAATTGCATTTTCAGCATTTGTTGTCT
6 SYNE1	GATCACATCAACACAAAACAGGCAA	CACCTGAAGTTGACAGGGAGAT

6 SYNE1	CAGGTGACCTCTAAGCGGTTT	AGAAGATGAATTGAATTCTCACGAGCA
6 SYNE1	CCAACATAGTTCATGCTCGTGAGA	GCTGTCTAAATGAGTGCTTTATGGACT
6 SYNE1	CGCCAGAGCAGGTTGTAACTA	CCGTTCAACAGGTCCACGAG
6 SYNE1	CCGTTGTATCGTGTTTGCTGTTTC	GCTCTTTGACTGGCATATACAGCTT
6 SYNE1	TGAAGAACTTTGTATGTTTCTGTAGCTGA	GTCTTGCTATGTATCTTTCCCACTGTTA
6 SYNE1	CTCTCTCAAGCCAGTGGTTGT	CAAGCTGAGAAGATCTTGGATACTGAAA
6 SYNE1	AGTAACTGCTGTGCTTCAAACAGAT	AGGTTGAAAAGATGCTAAGCAATTTTGG
6 SYNE1	AACCAGTCACAACAGTGCCTTA	CCCTCCAGTCCATCTCTACGAAG
6 SYNE1	TTTCAGCTCAATGGCCTCCAT	GCATTTGGTAATATCCCTTTTCTGGTGT
6 SYNE1	CTCAGAGAAGAAAGTTTAGGACTTGTCA	GTCCAGGCTGAGAACCTTGTAA
6 SYNE1	AGCGGTATCTCTGAAGCTTCCT	TGCACTTTATTTGAGGGCAGAGATAA
6 SYNE1	TCAAAAGTACTCAAGAAGGAATGAAGCA	GAACTTCACCGGTGGTCAGAT
6 SYNE1	GTGGCTGATGAATCTCCAGACAT	ACCACTGTTAAGGTAGTCTTTTCTTTGT
6 SYNE1	CTGTAAGCAACACCCTGGTCTT	TCACAGTTCTTGAGCGTGAGG
6 SYNE1	TTGTTTAAAAAGGGCACTCGATTGTG	CAGCAGCTGTTGATTCCGTTG
6 SYNE1	AGGGTACACGTAATCCCATTTTTGTC	GGATAGTATGGAAGCCAACCTGAAT
6 SYNE1	GACGTCATCTGCTTTTCTAATTCCTC	GGCACTTAATGGTCTTTCATGGAATTTT
6 SYNE1	AAAACATAAGTATGCCCACACCTTCT	CCACTTTTTACAGCCTTGTGAACAA
6 SYNE1	CGCATGCTGCCTTTCTGATTC	GACTCCTAAGGATTCTCAATGTGGATTT
6 SYNE1	CACAGCATGGATTCGCTTCATC	GAAAGTGAACAATGTTGGTTGCTT
6 SYNE1	AGGAGACACTCCATTCTGACAAGT	CGTCATATCAAGGAACTGGAGAAGTTAT
6 SYNE1	CTGCTGACTACTTGACACGTCTA	GCCAACTACTGGTGAATGCTGA
6 SYNE1	TTTGGCTTCTAAACAGTCTGTTCCT	GCACTTTTGGAGCTTTTGCCAATTAT
6 SYNE1	TGTGTTGCTAAATCCATGGCTCAT	AATTAAAGTACCGTCTGCTCTCACTG
6 SYNE1	AGCTTTGACTCTGCAAGAACCA	ACAGGTTAAGTAAGCATCAGTTCTGTT
6 SYNE1	AAATGGCCCAAAACTCTGACCT	GCTCAATGGCACGATTACCAGAA
6 SYNE1	GTGCTTCATGATGTTGACATATTCCTTT	CTTGAGTACCAAGGCCACTCTG
6 SYNE1	GTGGACTCAGCGCAGTGT	GGAGAAGTTGAATGATCAGCTGGA
6 SYNE1	ATCAATTCAATAACCTCTTCCCTTGCT	ACACCAGAGCTTCGTGTAGAAATAC
6 SYNE1	GGAGCAACCAAGACATCTCTTGT	AGCAGCATGGCTCCAGATTTAG
6 SYNE1	ACCTATATCCAAGCTCATTTAGACGGT	AGAAATTCACTGCGCACTTTGAAA
6 SYNE1	GCCATGACAAGGAATTCTTTTTCTGT	CAGCTATTCAATCGGATCAACTCTCTTT
6 SYNE1	AGGGCACACAAGTGTTGACTC	GAGTGGGCATATTTTCACCTCTTCTA

6 SYNE1	ATGCTAAGGCCCCTCAGAAC	TCAATAGCTTGTGCCGCAAGTA
6 SYNE1	GCATCTGGGATAACTACTCCCCAT	ACAGCAGCAATACGAGGACATC
6 SYNE1	GTTTCTTTATCAGACCAGTCATTGCAC	AAAGAACTTCAAAGTCAACAAAGCAACA
6 SYNE1	TCTCTCCTTCGCCCTCCTTAG	TGCATTCTTCTCGCACTCTTCTT
6 SYNE1	GTGGATGGAGACGGGAAATTTTG	GATGCGATTCACATGCTGGATT
6 SYNE1	ACTGATTGTGGGTTAAGATGGTATTTGAA	TCTCTTGGTGTTGGCTTCGT
6 SYNE1	GGATGTCGGGTGGCAGTATG	CAGCTCGAAAACCATTTCTGTCATTT
6 SYNE1	GACGAGGAAAAGATTGGCTCAGA	ATCAGATCGCACCGGTGAC
6 SYNE1	TGAATATTGGGTACCAAGGAGCCATA	CACATTAAGAAAATGGGAGCGATTTGAA
6 SYNE1	TTCATGACTGGAACCTGTTTGAAAAAG	TGAAAGTTGACAGTTTTGCAAACGA
6 SYNE1	GGAGCTTTGCTTCTAGAACTTTGCT	CCATTCAATGTTTTGGATTTGCTTCTGat
6 SYNE1	gccTGACTTCTTTTCTACTGGTGTA	ACTCAGCAATCTGTGACAGTCC
6 SYNE1	ACTCAGGGAAAAATGAATTTACGTGCTA	ACTCAGGAGCCAGAGGATGAG
6 SYNE1	GAAATACCTCTGCAAGAGATCCACT	ACCCTGGACCCACTCATCAA
6 SYNE1	GGACCATTTCCAGCTCTTGTG	AGGTGAGTAGAAGATAGGGATGAAATGTT
6 SYNE1	GTTCTAGGTCTTCTTTGCCGGTT	TGTTACAGCTGGCAGGCAAT
6 SYNE1	TCTGCGCTCTCCACGTCTA	GGAGAGTTGATTATGTTTGCAATTGCT
6 SYNE1	AGGAAGTTTGCAGCACAGAAGATA	AGCTTTGCAGTCCAAAAGGAAAC
6 SYNE1	CCATCAGCTCTGTATGGAACTGG	AGTTGCTGACTTCTTTCTTCCTGAA
6 SYNE1	CTCCCATGGGCCAACTGAAA	TGGGACATGGAGTTGTTAAAAGAATCT
6 SYNE1	TTGCAACTCTCAAGGCCTATAAACAT	CGAATTGGAGATCAGGCTTCTGTTC
6 SYNE1	ACCTGACAGTCTTTCAGTGCATTTT	TGTGTTTGTTCAGATCCAGCTGT
6 SYNE1	GACCAAGATTCCAATAAGCCTTCCA	TTCTTAATTTGGAGAAAATACTGGCTAGAAT
6 SYNE1	AGTCTTGTCCTGGGACCGAA	CATCTGCCATGGACATGCAAATC
6 SYNE1	AGCAGGTCACCTTAATTTGGCT	TGGGATCATTTTGGCAGTAATTTTGAGA
6 SYNE1	AAAACCACTTTACCTTATGCCGAGT	GATTAATTTTCTTCAGTCTGTGGTTGCT
6 SYNE1	GGCATGATTTTAATTCTCCCACACA	CAGACAGAACAAAAGTTAGCAGTAGAGA
6 SYNE1	CAGCTCATCAAATTGCTGGTGTTC	TGCCCTTGGGTAAGCTTAATTCC
6 SYNE1	TCTCAGTTATCCAGACGGACAGAG	TGGTGATTTCAAGGAAAATGTGTTGTTG
6 SYNE1	CCAAAAGGTGCTGATAATTTCCTGAAA	CATTGTTCCTTGCCCTTTGTTCT
6 SYNE1	CTTTGCCTTAATAAACCCGTGCT	GACCTGAAGCAGCAATTACTGTTG
6 SYNE1	AGACATACTTGCTTGACTTCCATAAACA	CGAAATTTACCTCATTGGATTCAGCAG
6 SYNE1	CAGCATCGTTCATGGCAGTATG	TCCTACTTTAGTCACTTTCATATCAGTCACT

6 SYNE1	ACATGGTAAGAGGTAAAGGACCTGT	GGAAGAAAGGTTCAATACGGAAAACTTG
6 SYNE1	CTCAGGAATGTGCTGTTTACTCTCT	ACAAGCATGTAAGAATTTCTCTAAGGCA
6 SYNE1	AACAGTGTTGAGTTTAGGAGGCATT	TCTCCAGAAGTTGGACGTCTCA
6 SYNE1	GAGAGAGCTGCTCCTTGAGAC	TTATTCTTCCCCTATGGCTTTGAACTT
6 SYNE1	CTTTTAGTAATAGCTTCCCCAGTGCTT	GGGAGAATCTGTCCTTCAGAATACTTCT
6 SYNE1	TGAATAGTGGGAATGCCTTCTGG	TGACAAAGGACTTCTGTATGTGTTTTCA
6 SYNE1	GTCTAAGGATTGACAGACACTCAGG	CATTGCACAGAACATGGTTTCACA
6 SYNE1	GGGCAAATTAATTCCCACCTTAACT	GGAAGAGATCAAGCACCTGAAGTC
6 SYNE1	CTTGCAGGTAAATCCAGAGCTCA	GCTGGAAAGTTCCATCTCTGATTCATTA
6 SYNE1	TGAACAGTGGATACTCTGGGTGA	ACTGATGCCGAGTGTGACTCTA
6 SYNE1	GGTTTCTCGTAGCCTGCTGTA	GTTGAATAAAGCCTGTTACGTTCCC
6 SYNE1	AAATTTTACATCACAGCCCTCTAAGTGT	CTAAAGTCTTGGCTCATGGAACTATTG
6 SYNE1	AGCTTTCCTCCAAGGTTGTTTGT	CTCGAGCAGAGTTGGAGAAGG
6 SYNE1	AGCTGGCTTGCAGAATTCCAT	AGCTCAAGAAACTGAGGAGTTTACATTT
6 SYNE1	CTCCTGAGCAATCCGCAGTA	GAGAAAGCAGCATCACATTATGATTGTT
6 SYNE1	TTCTTTACCTGACCTTGGCAACA	ATGAGTCTTTCAAGGACACAGCTC
6 SYNE1	TCATTAAACTGTGTTTTCAGCTCCTCTT	CTCCAGTCCATCCTGTCTGAG
6 SYNE1	CATCCTTGAATAACGCTTTGTCCAAA	GTGAAAACAGACATGGAGAGCAC
6 SYNE1	CAGGTGATTATCTTACATCCAGCCATT	TGCCAAGAACAAAATGAATTACAAACAGA
6 SYNE1	GATAACAAGTGTTTTCCTTTGCTGGTAA	ATGGTTTTAAACTCATGACTTATCACTGACT
6 SYNE1	AGGGCTCTCGTGTGATCTTCT	CCAATGGAATGACTATGTAGAGAGGAA
6 SYNE1	ATCCACTGCTCCAACTGGTTT	CTTTGTGATTGGTTTTCAGTAGAGATGTC
6 SYNE1	AGCATAACCACCAATAGAAAACAGGAA	GGAACAGTTCAGTGCCCTTCTG
6 SYNE1	GTTAACTGCTGAGCCCAGGTT	AGACAGTGTATTCCAAACGCAGAG
6 SYNE1	GCTGACCAGGTTCTCCAAACAG	CTGGCTCCCGAAGTGAAACA
6 SYNE1	ACCCACTGGCAGTTGTGTTC	CTCTTCAGTTACCATGGTGATATTCCA
6 SYNE1	ACATCAGTATCACACTCACACACAG	CTACGAGCCTCAGCTCAACAG
6 SYNE1	TGCACTTACCCACAAATACAATGTGA	CATGCACAGTGTCTGGAAGGAA
6 SYNE1	TGCTGAGATAAATGTCCCAGGATTG	TCAGTTTGTTACCACTGGAGAATCTG
6 SYNE1	AGCTGCTGAGCTTTCTCTTTCAG	ATTTCCATGTTCTCTGGGCCAA
6 SYNE1	GCCCATGGACAGTGGAAACTA	GAGAAAACCGGAAATGATGCCA
6 SYNE1	TTTGTGTCAACTTGGCTTTAATTCGAG	CTCAGCCTACATTGTCATTTTTCTTGTT
6 SYNE1	GGTGGTCATTGACCTGCTCA	GATCTCTCCCTTGTCTCCTCTCTT

6 SYNE1	GCAGATGTGCTAAGAGAGGAGGA	GCTGTCAACTTAGACTGTGCTTTTT
6 SYNE1	TGCTTATGACCCGATCCTCCT	GCTTGCCTGCCTTGTACCAAT
6 SYNE1	CACAGCTGTAGTCTTCCTCTGA	CAGGTCCACAAAAGGTGGCT
6 SYNE1	GCTCAGAAAGGGAGGAATCGG	CCCTTTAGCTTCCAGGAAGGT
6 SYNE1	CCTTCCTCACACTTCCTTGAACT	TGTTTCTGCTCTCTGCTTCCTTT
6 SYNE1	TGCAAAAGATGGGAAACCTGGA	GCGGATGCCTTTCCATTGATTTTATT
6 SYNE1	acTGGTGAAGCCATCCCAAAG	GAAGACTACAACAGTGAGCTCCAA
6 SYNE1	AGCAGCCACTTTTCGACCTC	GTGAGTTGCACTGGGTTTTTGTAATA
6 SYNE1	TTTCAGGAGTTTACTACCTGCTTAATGG	TGTCTCAGATGTACCAGAGTTTGGA
6 SYNE1	GGCTGACGTGCTTCTGAAGTT	GGGAAAATCCATTTAGATGTACTGGT
6 SYNE1	ATTGCACAGTTATAATCCCTCAGCA	GTACCAAGCAGAAACTACTACAGAATGT
6 SYNE1	CTTGCTCTTGTTCCTGTTCCAGA	CCTGATGTGTTATCCCAGGAGTTG
6 SYNE1	GATGATTTCTCCCCTTCCATCCC	GCCAGGTCTGGTACACACAAT
6 SYNE1	AATAAACTTGCTGATCTTTCCTCTCACA	CTGTGTTGTTACCCTGTCTGCT
6 SYNE1	TCTTATGGGCTTCCGTTGCAAA	GCTTTCACGATGTTCCCTCCTC
6 SYNE1	CAAGTGGCCGTGACAGTTTC	GATGCTCTGGCAGTGTTGAAAAA
6 SYNE1	GGAAGCAGTCCTCAGCCTTTC	GAATCAATAACTCTCTCTTCCCTTCCA
6 SYNE1	TCTCTAAGCCTTTAAAAACACACTTGGT	CCAATGAAGAGTTTCAAGCATTTCTGAA
6 SYNE1	AGCCTGCTTATCTTTAAGGCATTGT	AGAGCAAACTTGATGCAGTGACT
6 SYNE1	CCTCTTTCTGGTCTTGGAATGCT	CCATCTGAAAATCGGGAGCTCTG
6 SYNE1	AATGTGCATACAAAGTTTGTCCTTCTTG	ACTTCAGCTCACAATTGTAATGCATAGT
6 SYNE1	CTCTGCAGGAAGTCCTTTGCT	CCTCAATGATGCTCTTCAAAATGCTAA
6 SYNE1	ACGGCAGCAGATTCTAATGCT	GCAGAATTCCTCCAGAGCCAT
6 SYNE1	CCTCAGCTGGCGGAGAATG	CTGAGGACCTCCACCTCCT
6 SYNE1	CCCATCAAAATGTCAAGTGCTTTGTAA	TGGAGTCTCAGCCACCTCTAC
6 SYNE1	GTTCTTCAACGTCATCCAACCAA	cCCATCCTGATATTTTTCCTGGATTTTC
6 SYNE1	AGCTGGCTTTCTGTGAGCAATAT	GGACTTGCTTTGATTCAGAACAAGAA
6 SYNE1	CATGACAATGCTAGAGACGTCTTCT	CACCCCAGAGTTCCATAATAGAACAA
6 SYNE1	ACCTGTTTACAAATAAGCAGAGTGGAAA	AAGGAGCTGATTTCTGCGGAT
6 SYNE1	GAAGCATATTCCTTCCGAGCAAG	CAGACCATATTACCATCGCTGAATGA
6 SYNE1	ACTTCCTGCCAGGGAGTGATA	AGAGGGTTAGCTTATTAGAAGACACCA
6 SYNE1	CTCAGCGAGGCTGTGTTCTAA	ACATCCAAGAGGAAATCAGAAAAATCCA
6 SYNE1	TCAGCGTAAAGCAGGTACACA	GAGAGCCCTGCATGATAAGCA
6 SYNE1	GCTGGCTTGTGCACTCTTACAT	CAAAGCCTTGTCCGAGAAAACC
6 SYNE1	TCACTGAGGTAGGAATGATCGACT	CCTAATGAATGAGAGTTCTGAGCTTCA

6 SYNE1	GAATGTTTTGGTATTTAGCCAGTTGTGT	CGGTTACAAGAACTAGAGAAGAATTTGGT
6 SYNE1	TCGTGTTCTTGGTAAGCACTGG	CGGTGGATGAACTGAACCAGAAA
6 SYNE1	GAACCCTCACCTGCATGTTGA	TGGCTTTATCAGAACTGTCAGTCC
6 SYNE1	CAGCTTTCTGGCCTCCAATAGA	TGCTTTCACTATCGCCGAAACA
6 SYNE1	CCAGTGGCAAGCTTTATCAAAATCT	GCAGGAAAAGAGTGAAGTGCTG
6 SYNE1	GGAACACTGCCATGAGAAACTG	TTGGTCTCAATGAGCGACAGG
6 SYNE1	GGCAATATTTCAGGATGATGTCTCCA	GCTCCTTAGAAGCTGTGCAAGT
6 SYNE1	CTTACCTGAAGATTGTCCACCTGA	TGCTGAAATCAGTGCTTGACCAA
6 SYNE1	CAGTCTTGGGATCCCCAGTTC	GCCTTTCATTCAGTTATTCATGCCA
6 SYNE1	CCAGCAGCAGGTTCTCATTGT	CTGTGGGAAACAGTCTTTGCAG
6 SYNE1	GACTGGACCTCTTCCTGATGAATT	CGAATGGTGCAGAGTATAGACTTCC
6 SYNE1	GGCCACTTTGTGACTACTCCA	CTGAAACCCTGATGAAGTTCTTAGATGT
6 SYNE1	TCTGCAATTCCTGAAGCTTCCC	AGATGATGTGAGCAAACAAGTCAAAAC
6 SYNE1	GTCCACCAATTCCGGTCGAA	TGCTTGTATCTATTCAGAATGTGACATCA
6 SYNE1	CTGTGCTGCGAAAACCTTCTTT	GTGAACTTGAAGCCCAATTCTTGAG
6 SYNE1	ATCAGAGAGAGCCTTCTGGATGA	GCAGAGCCCTCCTCCAGATA
6 SYNE1	TCGGTGGGAACTTTGCTCATC	ACAAGTTCTATAAAGTCCAGGAAGCAATT
6 SYNE1	GGGAGCGACTCATCTCAGTCT	CACCTTGACCCCTTGGCTTAT
6 SYNE1	CCATGAGAAGGTTCTGAGCCA	AGGACCTCATTAAAAGGAAAATCCAAGT
6 SYNE1	TGGATCTGATGCCTGGCTTTC	CCTGCAGAGGAGAAGCTCAAAAT
6 SYNE1	GCCACCATGATCTCCCTCTGA	TCAACATTAACTCCACCGATATAGCTG
6 SYNE1	CCATCAGCTATATCGGTGGAGTT	TTACTCCCATGTACTATTACATAGGCA
6 SYNE1	TGCTTAATTACTTCAGGCACCGA	AATACCACGACACGTTCGTTCT
6 SYNE1	CCCAAGGCCGACTGCTG	AGGTTAAAAAGGCCACTGAAATGATTG
6 SYNE1	CCAGGTAACTTATCTTGCAGCTGAT	GTGGCTTTTATGGGAGTTACCCTATT
6 SYNE1	TCACACAAGAGGACTGACCCTAT	AAGAACAGAAGGAGAAGTACTTAGGTCT
6 SYNE1	AAGGGAGAGTTCAGAAGGTAATATGGTAT	GCCTTGTCTATAACAGTCCCCTTAG
6 SYNE1	TTCCTCTAGCAAATAATGACCCTGAAC	TCAGATTCTCTGAGTTCTCATCTTGGAT
6 SYNE1	TGATCCCCTTTAATTGTGTCTCATTTGT	ACTTAATTGTTAAATTTCCATGAGTGAATTCAA
6 SYNE1	TGGGTAACTGAGAGCAGAGGT	CACAAAAGTGGAAGAATCGTTGATGAA
6 SYNE1	AGCTTGGTGAACGGAAAGGTT	CCTATTCTGATTGGTATGGCTCTACTCAT
6 SYNE1	CACAAGTCTCATTTTGGGCACAG	CTGAAGTAGCAAAAGGAACCCTGAA
6 SYNE1	GTGTACTTTGAGCCGTGAAATCC	ACGTGAACAGAATTTCTACTGAATGAGT

6 SYNE1	CTTGGTAGCCACATTCTTGAAGTTTTT	GCCTTTACTTCACCATTCAATTTTCTCT
6 SYNE1	TTCATGAATTGCCTTGTAACCTATGG	ATTTTAACACTTGGGACTGGTTCTCT
6 SYNE1	CAGTATCATTGATTCGCAACAGTGAC	GAACTTTCACAAAATGGATCAACTCTCA
6 SYNE1	GTAGTTTCCTTTACCTTGGCCAGA	TTCACGATTCCTTCCAGATTTGTGT
6 SYNE1	CCAGATCTACTAAAGCAGAATACCAACAT	CTTAGAAAATGCCAGCAGTGTGATTG
6 SYNE1	CTCCTGATCTTTTATGGTAGTTCTGGTT	AGGGTCAGTGCTGTGGACTTA
6 SYNE1	GCTGATTTCAGGTTCTGATATTCTCTCA	AGTCCAATTGCAGGCAACTCT
6 SYNE1	GGAGACCTCAGTACTGCTCCAT	AGAGGGTGATTCAGACTCAGTTAGAG
6 SYNE1	AGAATAACATCACAGTCAAGAAACCACA	AGGAGTTGCGACAGATGATCAAAAT
6 SYNE1	GAAAAACAACAACCAGGTCTGTAACA	TGCTAGACTCAGTAGTGAATCAACGA
6 SYNE1	CAGCCCACACTTCTTTAAGGGT	GCCTACTGATGAGTATTCTTTCTTCCTT
6 SYNE1	ATCTTGGAGGTTCTGCAAACGA	TGGAAGCAATGACTGAGAAATTACAGT
6 SYNE1	GCTGAAGTCTTTCTTTCATCTGATGACA	CCAAGCTTTTCCAGAGAATGGTGATAAT
6 SYNE1	AAGGATAACCTGCCCAAAAGACAA	ACTGAAAAATGCCCTTTTATCACCTCTA
6 SYNE1	TGTACAGTACACGCTAGTGAGGT	CCTGCCCGTCAATGTCATGAATAT
6 TNFAIP3	CAGGCAGCTATAGAGGAGTCGTA	GTCTTCTGGAGTTCTCTCCCGTAT
6 TNFAIP3	GCAATATGCGGAAAGCTGTGAAG	GTCGATGAGGGCTTTGTGGAT
6 TNFAIP3	GTTTTGTCCTCAGTTTCGGGAGAT	GAGCTATCACCCAGGCAAAAGAA
6 TNFAIP3	TGATCTGCCTGTTCTTTCCACTC	AGGGCCCATCCTCTGGTT
6 TNFAIP3	GAGCTCTGCATGGAGTGTCA	GAAAGCATTCGTTGCAGTAGCC
6 TNFAIP3	TGATCATTTTGGCAATGCCAAGTG	TCAGAGGATAGCACCATGATGACT
6 TNFAIP3	GGGTGACCCCTATGTGGTACTA	TGAAACACTTCTGGCAGTATCCTTC
6 TNFAIP3	ACCCTTGGAAGCACCATGTTT	CTCCAGCAAAAAGCATCGAACA
6 TNFAIP3	ATGAGATCTACTTACCTATGGCCTTGT	CCTTCATCTCATTTCAGGATCTGTCAAA
6 TNFAIP3	GAGCTGTTCCACTTGTTAACAGAGA	ATGAGATGAGTTGTGCCATGGT
6 TNFAIP3	GGTGATAGAAATCCCCGTCCAAG	CAAATCACTCTACTGTTGAGCTTCAG
6 TNFAIP3	TTTCCTTTTGGTCTTCAGGTTGGA	CGGAAGGTTCCATGGGATTCT
6 TNFAIP3	GAGTACAAGAAATGGCAGGAAAACAG	GCATAAAGGCTGGGTGTTCACA
6 TNFAIP3	ACACTGAATGTGCAGCACAAC	TCCTTTTGAAGCAAGTACTGCAGAT
6 TNFAIP3	CCAACTGCCCCTTCTTCATGT	CTCATAGGCTTCTCCCCGAGA
6 TNFAIP3	AAACTCCCAAAGCTGAACTCCAA	TGGTCTCACTGAACAGAAAAGGG
6 TNFAIP3	GGAACCCTGAGGAGTCCACT	TGTGGCAACGTTCACAAAATCC
6 TNFAIP3	AGGATGTTACCAGGACATTTAATGGG	TTTCCAGCTCTGTGGCAAGAAT

6 TNFAIP3	CACCAGCGTTCCAAGTCAGAT	GCTTTTCTGCACTTGCTCGTC
6 TNFAIP3	CTGTCTCAAGCTGCACGGA	CAGCCGCTGTGTTAGGAAGT
6 TNFAIP3	CATGCAGATAACTTGACTTTCCTTCTCT	GGAATTTAAAGTTGCGTGTGTCTGTTT
6 TNFAIP3	CTGTTCAGCACGCTCAAGG	CACCATGGAGCTCTGTTAGTAGATAATT
6 TNFAIP3	GGGAGTACAGGATACATTCAAGCTTT	GGATGTTGCAAAGGACAAATATGTGT
6 TNFAIP3	GAAAGTCACCTAAGGGCCTCAT	GCTGTCATAGCCGAGAACAATGG
6 TNFAIP3	GCCCAGGAATGCTACAGATACC	AAAACCCTGATGTTTCAGTGTCTAGTT
6 TNFAIP3	GACTTCAGTACAACTCACTGGAAGAAAT	GCTGAAAGCATTTAAGTACAGATCCGT
7 AKAP9	GACAGCAAATGGAAAAAGATAGGCA	CGCTGTCCTTCAAGGTCTAACTTATAAA
7 AKAP9	TAGAGAAATATGCTCCAGTGGTAAGT	TGTAAGATAAATTCCCTGACCCCAGT
7 AKAP9	TGCTTTATGGGATAAGGAATAGAATTGCTT	CATCCATGCTTGACACTGAAATCT
7 AKAP9	TCATGATGAGATTTCAGTGTCAAGCA	TGTCTCTCCATTTGCCGCATAT
7 AKAP9	GAACCAGGGCCTGTTGGT	GCTTCCAGCTCCTCTAAAAGCA
7 AKAP9	ACCTGGTTTCAGAGAGAGAGAGG	CAAATCCTACTGAATTGGTGATCCATTG
7 AKAP9	GGGTCAGGGAATTTATCTTACACACA	AACATAGAAACAACACTGGCTGAGTTA
7 AKAP9	GCTCCAGTCCAAAGTGGAAGAT	CTCGTTCTAGTTCTTGCTTCTGGAA
7 AKAP9	CGACTACAAGGAATCATGCAGGA	GGGCTGCAGTGCTTTCCTTAA
7 AKAP9	AACGGAATTGTTAAGGCAAGCAC	TCTGGTTCAAGTTGGTGTTTGGAA
7 AKAP9	GGGAGCTTCCAGTTGAAACAT	AAATAAGAACATTCTTCAGACACTGCCT
7 AKAP9	TTCTCATGAATTTTTAAACTCTTGTAGTCAGTA	CAAATGCGTTGGGCTTCCAT
7 AKAP9	AGAATGATTTAAGGCTACAGATGGAAGC	TCCTTGATCTTTTCCTCTTGAGCAAA
7 AKAP9	TGGATCAGGTTCGTGAATATATGGAAAAT	ACCTGTTTCTTGTGTTTTCATAGTCTGT
7 AKAP9	TCAAGGAACTTCAGAAAATACACCAGTT	CAAATCACCTTCCTCTGTGGGT
7 AKAP9	GTTTCTTGTGTTTCCGCTGTCA	CTCTTCTTGAAGTTTTTCAGCCTTTTCT
7 AKAP9	GTGGCTACCAAAGCAGAACTTG	TCTAGCTTTTAAGTTATGGAGCTCATTTGTT
7 AKAP9	TGAAGAAGTTGTCATAATTCTGGCTCTT	TGAATAGAATGGTCCTCTCCATCACT
7 AKAP9	GCTTTCTCTCACTGAGTCTCCCTAT	CATAGTATTCTTTGGTTACCTCGGCTT
7 AKAP9	ttGCCTCTTATATTTCAGGTTGAACAGT	TTCTCTTACTCTGAACTCCAGTTTCTCT
7 AKAP9	CAGCTTCAAAGGGATATACAAGAAAGGA	AACACTTATTAAGTTAGCCCAAATAAAATGCC
7 AKAP9	TGCTTCGTCAACATAGCTTTATGGA	CGTCCCAACTTGGCTTTCTCT
7 AKAP9	CTCCAGTGGGCTTTGGAGAA	TTGACATTTTAGATGGAGGAAAGCCTTA
7 AKAP9	ATGTAGTTTTCTGGTGAATTTGGAGTGA	AAGGCTCAAAGGGAATATAAACCAAGAT

7 AKAP9	TGAAAGATGCAGACTTTTACTGTGAAGT	TTTCCATCTCACAGACCTTGCTTAG
7 AKAP9	ACAAACTCTTGGTACTTCAAACACGA	TTCCCAATCTCCTTTTTCATGTTCTCTT
7 AKAP9	AACCGTGAGGCCATGCTT	CCCCAGATATTCTAGACAGTTCAGTTT
7 AKAP9	GTGTCAATGAGTATAGCATTTGCTCAAC	AGTCTGAAAACCAATTTGATCCTGTGAT
7 AKAP9	CTGTGTGTCAGCAAGAACAACATTATTT	TCCTTTCCATGTTCTCCTAACTCTTTACT
7 AKAP9	AGACTTTTGAGACAGTGGATGTGAAATT	TTTCTTCTGAAATAGTCAGCACACAGT
7 AKAP9	AGAATCAAAGGACTGTGTGCTGA	CCAAACATTAAGCAGCACTATTGCTAAT
7 AKAP9	gaaGTACACATGCTCCTAGGCTTT	CAGTAGTTCACCTCGCCAGT
7 AKAP9	CTTCTCAGACTGGCGAGGT	CCTGTTCTTGTATTCTCTGTTCCAAACA
7 AKAP9	GGTACTACAGATGCAGTTGGTTTACTAA	ATATTCATAGTGGCTCCTATTAAAATCTTAACC
7 AKAP9	CTGTCACAGAGACTGTCTGATCTTT	TGCCCTGTTGTGGTTAATAATGATTTCT
7 AKAP9	GCAAGAGAAAGAACAAGTTTCATTGAGA	CTTGTCATGGTCACAACTCCATCTAATA
7 AKAP9	CAGTCATGTGATACTCAAGTAAGCTCTT	TTGCTTACTTTCTTCTCCAACAGTCATAT
7 AKAP9	GGTGCTGAAGGATCAGTTTCTAAAGTAA	AGTTGCTCTAAGTGATGATTCCTTTGT
7 AKAP9	AACAAAGGAATCATCACTTAGAGCAACT	TGAAGGTGAAACCTTAGTGTGAAATGT
7 AKAP9	AGACCTTACAATTTCCATGATTTCCAGT	CCACATGTTAATGTCATTTCTTGGCATA
7 AKAP9	GAATCCATTCCCTCTTATTCTGGAAGTG	AGCATAAGTTCTTCATTTTCAGGGTCT
7 AKAP9	GAGTGGTTTTGCTGGAACTGAAAT	GGTTACCTGACTGCTAGTTTCACT
7 AKAP9	GATACATTATGTGTGTACAAGTGACCCA	AAGCTGAGAACATGCTAAAGAACCA
7 AKAP9	GCAGTTGAAAAACTCCTAGAAGCCATA	TGCCAAACAGCTATCCATGAAAATATCA
7 AKAP9	GCACTCCAGCTGATTTCAATCC	TGCCAAGATGTGGTTAAACTCAGA
7 AKAP9	GGGACAGTCGAAGAACTTCAG	GCTGCCCATACATCTCATCCA
7 AKAP9	GTTAGAACAACTCCGGGCAGA	CATTTCTCCCTTATGCCGTGTTTT
7 AKAP9	CACATGGCACAGATGGAGGAAA	TTCCTTTTGAGAGTTAGTATCTTGCAAT
7 AKAP9	CTCTTTGTTTAGCTTGAACATGCGA	CTCATGAAGGCGCTCTCGAA
7 AKAP9	TCCCTTAAGTGCCAAGAGGAAC	AAACCTGGCTGTGCATCAAAAAT
7 AKAP9	CATTTTGACCGCCTTGCATCT	TCTCTTTGTCTTCTGCATTTACTTCACA
7 AKAP9	GTGTGGTGTAGATTTTGCATGAACA	GTACCATCAATATTCTTTTTCACACAACTTCC
7 AKAP9	AGAAACAGGATTGCTGCTTCTCa	CTAGGGACAATTCTGGTTTAGCTTCT
7 AKAP9	ACCGAAAACACTTTGGAGCTGTA	TCAAACCACGCTGTGTCATTTAAAAAT
7 AKAP9	TGTTTAGCTCAGACTTTTCTTCAGAGAC	AGCCTCTGCCCTTTTCAAAGAT
7 AKAP9	AGTCTTTTGAGCAGTTACCTATTTTGTAGAAA	GCCCACCAGAAGAATGATAAAATGAGT

7	AKAP9	GGTTACTTTAGGTGCAGAAAAGACTG	ATTCCAGGCAGGTTTTCTCATATTCTT
7	AKAP9	GGAACAGAGAAAAATTGACTCTCCAGAA	CTGTGATGTTTAGATGGGAGATGTCT
7	AKAP9	CCTCATATCCTCTTTGCAGCAACA	GCAGATGAATACCTTGGCGGAA
7	AKAP9	GCTTCAAGCTGTTAGTGAGTCCA	GCTACATCCTATCTCTTTCCAGCAA
7	AKAP9	ACAGCTTTCTAATTGCTGGATTATGTAT	GTCTTCCAAATTGGTCATCTGAGAATG
7	AKAP9	CCATTCTCAGATGACCAATTTGGAAGA	ggtcacTACGTTAGCTCACATTTTAAAGT
7	AKAP9	AAAGTGTAAGAATTATGCCAATGTGTTTTGA	GCTGGGTTATAATGCCATCTCTTTGT
7	AKAP9	ACAAGAATTTGAAGCTGCCATTAAACAA	CTCTGTTCTGTCAACTCTAAAAATTCTCTCA
7	AKAP9	CCAGCTCACTGCTAATTTACAACAA	GCATTAGTAATACTTACTTGCTGAAATTGAATC
7	AKAP9	TGAATCTCTCTCCCTAAGGAATATTGCT	CCAACTCACTTTCCATCATCTCTAGAT
7	AKAP9	AGCACAAGACAGTCCGACTC	GAGTAGCAAATCAAAAGCCACAGAAAAT
7	AKAP9	AGATGGGCTGTGAAATTTTATCTTGGA	TGCTATTTTGACTTCACCTTGATTCAGT
7	AKAP9	ATCATCTCTTTGAGGTACTGCCCTA	TTGGGTTTCAAGATCCCTTATCAGTTC
7	AKAP9	GAACTGATAAGGGATCTTGAAACCCA	AGCATTCATTGATGTCTTCTGTCCAAT
7	AKAP9	TGAAAAACTTCAACAGGAATTGGCAAAT	TTCTTCATTAGCGGTTTCTACTTGCT
7	AKAP9	CTGAAAAGCTGGCCTTGGAAC	TTCCACACTTTCACGTTCTCTCTTT
7	AKAP9	CATCTGAGCAAAGACAAACCTGAAC	AAGTTGTAGCAACCTTGTTTCCAAATT
7	AKAP9	TTTTGAAGAAAATGGCAAAGGTTCCAT	GCCTTGTTCTTGCATGTCTTTTATTTGT
7	AKAP9	ATCAGCTAACACAGGAATTATTCAGCTT	GAGCATCCTCTGTAAGGACTACTTCTA
7	AKAP9	CAAAGGACTTAGAACTTACCCAGTGTTA	CTCAAGTTGGATTTGACTGACAAGAG
7	AKAP9	GAAAATAGTTGAAGAAAAAGTGGCTGCT	TGACCCCAACTCATCTTCTCTTAGT
7	AKAP9	CAGTTCAGGAATATGCAAAATTCTGTCA	GCATTTCAACAACCTGGCTTTCTAATTC
7	AKAP9	GGGTCAGATATATCAGCATTAACCTTGA	TCTGTAGTTCTAGCAGCTTCTTTTCTTTT
7	AKAP9	CAGGTTGTTGAAATGCATACTAGTTTGA	AACTTCAACATCTTGAGGGCTTCT
7	AKAP9	AGAAGAATGACATGCTGTTTCCAAGT	ACATCTTCAGGTTTTCTGTCTAATTCCTT
7	AKAP9	CCAAGGAGAAGTTGAAGAACAAACATTT	AGGACAAAGTCTATTACATTGAAACAACCA
7	AKAP9	TGTGCTTAAGTGACCTTACACCATTT	TTCCAGCTTCATAACCATGTTCCTT
7	AKAP9	CCCCTAGAGAGCAGGGTGAAT	CCAGTAACAGCAGCAGGTATTTCT
7	AKAP9	GTCAACTGACTGAAGAGAAGAATGACTT	CAGCAGAAATGATAAGCATGCACTAATT
7	AKAP9	TTTTCGAAAGGCTCTCATTTACCAGA	CCGACCGAAACCTGGTGAAG
7	AKAP9	GTGATCACCAATCGCCCAAAG	ACCAGCAGAGAATAGAGGGAGAATT

7	AKAP9	ATGTTCCATGATCATCAGTCCTTTGT	TGGAATACATCTCTCTCATGTTCTCTGT
7	AKAP9	GTCATTAAACTTTAGGAGCAAGCCATTG	AGTTGGGCAATATCAGTGAAGTTCTT
7	AKAP9	GGTAGTAAACACCCCCAATTGAGAA	TTCACCTTGCTGGCCAGT
7	AKAP9	CAGGTATGCACTCCAGAAAGCTA	GGCAGATGACTGGCTTTTACTAGAT
7	AKAP9	GCCATGTCCTTGGGATTCTAGATAG	TTGAAATGATTGTTTCCACAACACAGAA
7	AKAP9	GAATTACATTCCTCCCACACCCTTT	CAATTTCTCTTTTGCCTCTGCGA
7	AKAP9	GTCCCACCTCCGTCCAA	GTTGCCTCGGGCTCTCC
7	AKAP9	GACTTAAGCCAAGTTAGGGATCACC	GGGATGCAATGCTCTTACTCAGTTTTA
7	AKAP9	TGCATGTTTGAGCCACTTCCTA	CTTCTTCTGAGGAACATTCACTCTGTAA
7	AKAP9	GCAATCAGACTCCACAAATTCTTGTTAA	CTGAAATATGTCTGGATTCCATGTCCAA
7	AKAP9	GCAAGAACTACATGCTGCTGAAATT	CAGACACTGTATCACTGCCTTCA
7	AKAP9	AGTTACTGAAAGAGGAATGTGGTACCT	TGAGTTGCATTTTGGTTTGTCATCT
7	AKAP9	AGAGACTCCCAAAGCTGCTTTT	CTCTGCCCATCCGACTGAG
7	AKAP9	CTTGCCCAGTTTCGACAAAGAA	TTGATCAATATTCAAATCATGGTGTGCT
7	AKAP9	ACGTCAAGCAGTAAACATGATGTGT	CTCATGACTGGTTATTTCTCCACTATGT
7	AKAP9	AGTTCTCAGAGAGTAGAATCAACTGTGA	GTTATGACTACTTTCCATCGAGAACCA
7	AKAP9	TGTATTATAGGCGTCATTGATGGCTATG	CTCCAATTCTTGCAACCTGTTACTTG
7	AKAP9	GTCTTGAGCAGGAGTTGTTATGTG	CAGAGGACTGTTTCATACAAATTCTGA
7	AKAP9	TTGATTTTCTCGTACCAGGATCTGAAG	CTGGGCATCATATAGCATTCTCTGT
7	AKAP9	ACTACTGAACGAATCCCAGCAAAAA	CCGCTCCCTATCTAGGGTACT
7	AKAP9	AGAAAGTTCGAATTCGGGAAATGAGT	CGACTGTGTTTTTCCTCTAGCTGT
7	AKAP9	GACCTACTGAAAGAGCTGCAGAA	CTGCAGTGTTTTCCTGTGAACC
7	AKAP9	GATTTGATTGATTACAGCACGGGAAA	CTTACCACTGGAGCATATTTCTCTAGTC
7	AKAP9	CATTCTGATGCTTACCAGACTAGAGAAA	ACCCCAGTCTGATCCAGAATCTAAAATA
7	AKAP9	TGTCTGTTCCCTAATACAGTCACTTCA	CTTGTTCTTCAAGCTGCTGTTGAT
7	AKAP9	AGCCAAACAACAGATCCTCACTC	GCTTCTACAAGTTAGCTGACTTCCTTA
7	AKAP9	AGCAGAAGCAGTCTCAAATGCT	AGTTTGTGCAAGCTCACTCTTCA
7	AKAP9	TTGTACGTTATAGGCTCTGATTTCTTTTGT	GCATCAGATTCCTTTATGGCAAGTC
7	AKAP9	TGTTCCTAGGATGACATGGAGAAACT	TTGGTCAATTTCTACCTCTTTTTCCTGT
7	AKAP9	GCCATGTCTACTCAAGACCAACAT	TGTTGTAATTTTAAGTTGCTGCTGGAG
7	AKAP9	GTGGGTCTGATACAGGTAGCAA	GCACAAAAGAACCATTGATTTACAAAAGG
7	AKAP9	CTGAGAAAATGGTGGTTGCTGAAC	GTTTTTAATAAGGAAGCCTGGCACAC

7 AKAP9	CCCTAGGAATAAAAGACATACACGTGAA	AATTTGTAGGCTTTCCTGTTCTCATCA
7 AKAP9	ACAGGTAAATGGTTGCAGTTTTGTG	CAGAAAGAAGTACATGCAGGAAAAAGTT
7 AKAP9	GGAGAAATGGAGAATGCTTTAAGGTCAT	GTCCTAGTTCTTCCTTGAGTTTTTCCT
7 AKAP9	ACTCTCAAAAGGAAAAACTCAAGGAAGA	TCTAGCTCTCTGAATCTGTTCCCTT
7 AKAP9	AGCTTTCCAAACTGAAGGAAGATTTAGA	TCAAATTGTCCTTTTCAAACTGCATGG
7 AKAP9	AGCTAAAAGATTTACAGCAGTCTCTTGT	GAACTTCTTGTTCAAGTGTACCCTTTTC
7 AKAP9	AGAAAAGGGTACACTTGAACAAGAAGT	CAACATGTCTTCAAGGGTTTTCTTTTCA
7 AKAP9	GCACAACTTGAAGCAGAGAATAGCA	GTCTTTGGATTTGGACTTAATGGAGTCT
7 AKAP9	GAAGACCTTGTTGAAGAATTGAGCTTTT	GCAGAAACAATCTCAGCTTTCAAATCTT
7 AKAP9	TGAAGCACATAAGTCCCTTAGTACAGT	CATTCTGTCTAACACAGCATTCTTTTCT
7 AKAP9	CTCCTGTTAGCCAAGAAGAAAGATTGAT	GTGTTCCTTTGCTTTTCAATCTCTTCATT
7 AKAP9	GGACCTCAAACAACAATGTATTCAGCTA	TCATCTTTTACTTTGAGAAGGCAAGCA
7 AKAP9	AGAAACATTGGAAATGGGTGAGGT	TCACCATGTTTCTGTTTCAATTGTTCAG
7 AKAP9	GCATCTGAATCCAGAAAGGAACTAGAAT	TTCTTCGTGACTAAATAGAAGCTGTGTT
7 AKAP9	GCTGAATTAGAGAGGCTGAGAACAC	TTTCATTCTGTAAACCATCTATCTGCTGTT
7 AKAP9	GCCTTTCATGAACGTAAAATTTGGACAT	ATCACGTACTTTTTCAGCTTGACATTG
7 AKAP9	CAATGTCAAGCTGAAAAAGTACGTGA	ATGCTTGGTTTTGCTGTCTTAAATCC
7 AKAP9	CAGTAGGTTTATAGAGCTGGAACAAGAAA	AGTATACTAGCAAAGAACATTCCACTGA
7 AKAP9	AGTGGTATTTTATGTGACTTTTCTATCATATGT	CAGTCATCTGCTGTGGTTGAAATT
7 AKAP9	GAAATTTCAACCACAGCAGATGAC	GTGACAAGAAATCAAGTAGCAACAATCA
7 AKAP9	TGTATTGCCTTTACATTGCTCTTCACT	TGCCTGAATTTCAGATAACAAACTCCTT
7 AKAP9	GGAATGCCTCCAGAAAGCAGATAG	TGTAACCATATGAGCTTGTCATACAACA
7 AKAP9	CAATAATTGTGTTCCCTTAAGATAGACTGTGA	GCGTAGTTTGATTCTTTTGTTTCTCCTT
7 AKAP9	TGGAGCTTAACCAGTGATAGAACTAGAA	AGCTACACATTGAAGCTTTTGTCTGA
7 AKAP9	ACCGGCTGTAATCATGAATTAGAAATGA	CTACTTTCCCAAAGATCAGGCAGAA
7 BRAF	TCAACTCAGGTAAAATGTCAGTTCCAAA	TGCATGTGGAAGTGTTGGAGAA
7 BRAF	CAAAGTTGTGTGTTGTAAGTGGAACA	CCAAGAAAGGCTTGTGTCTACATTTTT
7 BRAF	GCTACTCTCCTGAACTCTCTCACTC	TGCATCAGAACCCTCCTTGAATC
7 BRAF	CCTGCCTGGATGGGTGTTTT	GTAGATTCTCGCCTCTATTGAGCTG
7 BRAF	CGGGCCAGCAGCTCAATA	GGACTCTTAAAGATTTATACCACCCAGAT
7 BRAF	ACATTTGGCTGTGACTTCTAAGAAGAAA	GCTCACTAACTAACGTGAAAGCCTTAC

7 BRAF	GCTGAGGTCCTGGAGATTTCT	CCCCTGATAAATTAACATACTTGCTCCT
7 BRAF	TCCTGTATGACATGGATGCCTCT	GTTAGAGTCTTCCTGCCCAACAA
7 BRAF	TGACTCACCACTGTCCTCTGT	CATCTTCTTCCTCTTCTAGCCTTTCAG
7 BRAF	TGAAAAACTGAAAGAGATGAAGGTAGCA	GATGCACTCCAACAAAGAGAACAAC
7 BRAF	CGTTCCCCAGAGATTCCAATAACT	TTAATGTCAGGACAAAGTCCGGATT
7 BRAF	TCCCAAATCTATTCCTAATCCCACCT	AGGCCCTATTGGACAAATTTGGT
7 BRAF	TGGATTATGCTCCCCACCAAATT	GCAGTTACTGTGATGTAGTTGTCTATGTT
7 BRAF	GGTAAAATAAACACCAAGACGTGGTAA	CACCAGAAGTCATCAGAATGCAAGATA
7 BRAF	ACATCTGACTGAAAGCTGTATGGATTTT	GCATTGCTCTAGGAATTATAGTAGGTTGT
7 BRAF	CCTTCAATGACTTTCTAGTAACTCAGCA	GAGTGGGTCCCATCAGTTTGA
7 BRAF	CCACAAAATGGATCCAGACAACTGT	GCTTGCTCTGATAGGAAAATGAGATCTA
7 BRAF	CTGTTCTTTTGGATAGCATGAAGCTTT	CAGATAATTTTTATGGTGGGACGAGGAT
7 BRAF	AGGCAAGTATAAAGGAAATAAGCAGCA	CTACTCTCTGATTATATGCTTGCTTGGA
7 BRAF	GATCCAAAAGAAAGCGGTTCAAGT	GAAGAGGCGTCCTTAGCAGAG
7 BRAF	GATGATCCAGATGTTAGGGCAGT	GGTTTTGCACAAGTTAGGTTTGTTTTG
7 BRAF	AAGACAAAATGCAGAAGAAAAAGTCAGG	AGTCCTTTAGCCCTACTCAGGTTAA
7 BRAF	GTTCTAGCAATGCTGGATACTTACATCA	CCTTCAAAATCCATTCCAATTCCACAG
7 BRAF	GCCCAAATTGATTTCGATGATCTTCA	ACCTTTATATCGTTACTCTGAATCTTATCTTCC
7 BRAF	CGTATGGAAGAAAAACCCTCACAGA	TGGTTATAAATTTCACCAGCGTTGTAGT
7 BRAF	ATTAACACACATCAGTGGAACTTCTGT	AAACGTTTTTCACCTTAGCATTTTGT
7 BRAF	GCAATCCAAAAGAATAGCAGCCAAA	CACCATCTCCATATCATTGAGACCAAAT
7 BRAF	GCAGTCTGTCGTGCAATATCTATAAGTTT	CAGGAAAACACGACATGTGAATATCC
7 BRAF	CTTTGTGGAATAGCCCATGAAGAGTA	CGACAGACTACTTTGGTTCTCTTTTGT
7 BRAF	CGCCGCCTCTTTCCAAAATAAA	CCAGGCTCTGTTCAACGG
7 BRAF	CACTCACCTCCTCCGGAATG	GCCCCGGCTCTCGGTTATA
7 BRAF	AACAAAATTTCACGTCACATACAAACCA	CCTGCAAGGTGTGGAGTTACAG
7 BRAF	GTGCTTTCTTTAGACTGTCTCGGA	GTTGTATCTGACCTAGTAACCCATTTGA
7 BRAF	CTGGAAAAGAGTAATTCACACAAGCTC	TGACACTTGGAGTAACAATTGCCTTTTA
7 BRAF	TGCGAACAGTGAATATTTCCTTTGA	GGATCTGGATCATTTGGAACAGTCTAC
7 BRAF	CATACTTACCATGCCACTTTCCCTT	TTTTCTGTTTGGCTTGACTTGACTTT
7 CARD11	GGGATAACAGATGCCGAGTACC	TTCAGATCCCAAGCACTGCAA
7 CARD11	GGAGAATTGAGCCCTGGTGAC	TCAAGGTGAAGGACGACAACTAC
7 CARD11	GCGTAGCGCATGGCTAAGTT	AGAAGAAGCAGATGACGCTGAC

7 CARD11	GTACCGCTCCTGGAAGGTTAG	GCCTCACGCACTTCCTGATG
7 CARD11	CTGCAGCTTGATGACCTCGT	TGCGTCTGGAACCTCCTTTTT
7 CARD11	CCCTCCTCTTAGAGTCCAGATGT	GAGCCTAGAATTTTATTACCCAGAACTGT
7 CARD11	CCGGCTCACAGACCCTCT	TTCCACCTCGGAGGAGTCA
7 CARD11	CAGGAAGTACTTGCTGTCTTCAGG	CCACCACTAACGCCAGCTTT
7 CARD11	ACAGGGTGCTTGGCAGAC	GTGCTTGGCCCCTGAATTT
7 CARD11	CTCTTTCCCAGTCACCAGTTTGT	CAGAGTGATCTGGTCTTTCCCCTA
7 CARD11	GTGGGTGGTGGGCATGAA	GACGCCAGCCCTAGCTC
7 CARD11	GGCGTCCGTGCCTTCTT	CCATGAGGCTCTGActtccc
7 CARD11	GAGGTCCTGCTGACACACA	GCAGGAGCTGGTCAACAGG
7 CARD11	CCTCCTCCTGCAGGTTGTAGAT	CTGACCCTCTGAAACCTCCTC
7 CARD11	GGCAAAAGCCCAGTCCTC	GTAACGTGTGTGTGTCTGTGTCT
7 CARD11	tgattCGGGTGTCTCTTCTCAGT	TCTCTCGCGAGCAAGCTTC
7 CARD11	GGAGAGCCTTACCTGAAGGAG	CAGGGCCTGACTGATTGATAAATTCA
7 CARD11	CCCTCTGTGACCCGTCCTA	CACTCACATGCATGCTTTCCC
7 CARD11	CCGCAAAGAGGTCCTAAGGTT	TGTAACCGGCACATGCTCAG
7 CARD11	TGAGCTTGGCAGGGTTGATATAG	ccAGTGAGACTGCTTTATTAAGCTTGTT
7 CARD11	aattctGGTTCTGGCTCTAGGAGA	GGAGCAGGTCAACCTCATGT
7 CARD11	ACCTTTCCAGAGAGAACTTCCTGA	GCCACCCCTGGTACTGATG
7 CARD11	TCCCGTACTTGGTGAGGGTTTA	GGTGTGTGAGAAGAACATCAAGAGG
7 CARD11	TCTGGGAGCCCTACCTGAA	CTGACCAAAGCCGCTGTG
7 CARD11	GGGAAGTCGTCCTGGTTCAT	CCGTGCTCTTCACACCCA
7 CARD11	CTCTGCACCAGCGTCTTG	TCTGACCGCCACCCTCT
7 CARD11	GGCTCCTAAATGTCACCACTGA	CTTCGTGCACTCGGTCAAG
7 CARD11	CGGCTTTCTCGGCCAGA	GCCTTCGGTCACCTCTGT
7 CARD11	GCTGTCGCCATTCAGCGT	TGTGTCCCAGTAGGGCTTTTC
7 CARD11	CAGGCGGTCCCCATGTA	CCCTCCCTCTTCTCCTCCAG
7 CARD11	CGGTGGAGGGAGGTCTCA	CGCTGTCTCTGCTCTCCAG
7 CARD11	CCCTGGCAGGTTCATCGTT	ACTGGAGCGGGAGAATGAAATG
7 CARD11	AGCTCCTGGTTTTTGGTCTTCAG	CGGTTGAATAAGATGGAGGAGGAA
7 CARD11	AGAGACTGATTTCTCTCCAGCTTACA	CGTTGCCTTTTCTGAGTGTTTCTT
7 CARD11	GGTCCTCCATAGACTCAGGGAAA	GTGACGATGTTGTGCACGTC
7 CARD11	CTGTCCTGGTACATGGTGTCA	GAGGACGGCCTGATCACATC
7 CARD11	GCCGGATGTAGAACGAGTCC	GTGGTGAGGAAAGCTGCTGTA
7 CARD11	CGAGAGAGACAGAGTGACAGATG	TTTGATTTGTGTCTTGTGTCTTGTTTCTT
7 CARD11	CTGCAGGTGGTGCCTGT	GCATATTGATTTCTCTGTGTGGTTCA
7 CARD11	TGAGGATCATCACGGAGGGT	GATGAGTTCCTCAGAAGGCAGAAG
7 CARD11	GGGAGTAGATGATGGTCTCCGT	GATCGGACATCGAGTTCTGTAGAG
7 CARD11	CTGGCTTCAGGTGTGGGT	CATCGTCAACCTGGAGAGCAA
7 CARD11	TGGTCCAGGTTGTTGCTGTC	GACACAGTACTCGCAGTGCTT
7 CARD11	GCTTCCTGTACTTGTCCTTTTCGATT	GGGTTGGGATGATGCCTGTC

7 CARD11	CAGGGTGCCTGCCTCATAG	CGGTGGAACCTGACATGTGG
7 CARD11	GCGGAGCAGCTCCTCTA	CTGCGTGTCTCCTCCCA
7 CDK6	CTCCGCAGGATCAGCTTAAGG	CCAGCCAAAAGAATATCTGCCTACA
7 CDK6	CTGGAAGTATGGGTGAGACAGG	AGATGCGCTTAACATATGACAGTGAA
7 CDK6	GTGGCATGTGATGCCTATAGCA	CCAACCAATTGAGAAGTTTGTAACAG A
7 CDK6	AGTAGGTCTTTGCCTAGTTCATCGATA	CTTATGTCCATGTGTGGTTCTTTTGTA G
7 CDK6	TCAAGGAAAGCAGAGTGAAACAAAA TG	CTTGGATCCGCTTGCCATTG
7 CDK6	AGGGACAGACTGTCATTCAAAATAGG	TCGCGATCTAAAACCACAGAACATT
7 CDK6	GTTTTATTTGTCCGCTGCTGGT	CATAGCAAATGCTCAACTTCCGTTT
7 CDK6	CCTTTCTGGGCCTGAGGATT	CATGCCGCTCTCCACCAT
7 CDK6	GGTCTCCAGGTGCCTCAG	CGAATGCGTGGCGGAGAT
7 CDK6	GGCCTTGAACACCTTCCCATA	GGCCGCCAGCTAGTTGA
7 CDK6	GAAAGAAAGCCATTGCTTAACAGACT	GATCAAGACTTGACCACTTACTTGGA
7 CDK6	CACTCCAGGCTCTGGAACTTTA	TGCCACCTACTTAACCTCTTTGTTTT
7 CDK6	GATATGCATGTCAGAGGAAAGTCACT	TTCTTTTAGGCCTCTTTTTCGTGGA
7 CDK6	TTGATCAACATCTGAACTTCCACGA	TTTGCTTCCTGTGAAACAATGATAAT GG
7 EGFR	CCATGGAATCTGTCAGCAACCT	GTGCGCTTCCGAACGATG
7 EGFR	GATCGGCCTCTTCATGCGA	AACTGCTAATGGCCCGTTCT
7 EGFR	CATGCATCCTTCATGGGAATTTAAAG G	TTGCTGAGAAAGTCACTGCTGA
7 EGFR	CATCCAGTGGCGGGACATAG	CTGTAGAGCTGTCCCCCATAG
7 EGFR	ACGTCTTCCTTCTCTCTCTGTCA	CTGAGGTTCAGAGCCATGGAC
7 EGFR	GTGTGGCGCTGAGTGTACTTA	CACATCCATCTGGTACGTGGT
7 EGFR	CCCACTCATGCTCTACAACCC	GACAAGGATGCCTGACCAGTTA
7 EGFR	GAGAAAAGAAAGAGACATGCATGAA CATT	GTACTTCCAGACCAGGGTGTT
7 EGFR	GGCAGGAGTCATGGGAGAAAAC	AAATGTTCTGTTCTCCTTCACTTTCCA
7 EGFR	GGCTGACGGGTTTCCTCTTC	AATGGACGTGGATAGCAGCAA
7 EGFR	ACCACCTGCATTCAGGAAAAGT	GCTGTAAATTCTGGCTTATAAGGTGTT C
7 EGFR	CTGACTGCTGTGACCCACTC	TTGCACTTGTCCACGCATTC
7 EGFR	CGTCTCTTGCCGGAATGTCAG	ACAGAATGCCTGTAAAGCTATAACAA CA
7 EGFR	TTCTCTTTCACTTCCTACAGATGCAC	TCAGAAATGCAGGAAAGCATCTTCA
7 EGFR	AGTAATGATGACTAAAGCAAGGGATT GT	GGGATTCCGTCATATGGCTTGG
7 EGFR	TTTGGGAGTTGATGACCTTTGGAT	GACAGACCCACCAGTCACTC
7 EGFR	TCTTCAGCTCACAGGGAACCT	CATCTTACCAGGCAGTCGCT
7 EGFR	CCAGTGACTGCTGCCACAA	CTAATTGACAGCTCCCCCACA
7 EGFR	AGTTCCTCAAAAGAGAAATCACGCA	GCTAAGGCATAGGAATTTTCGTAGTA CA
7 EGFR	GAAAACCTGCAGATCATCAGAGGAA ATA	CTGCTGTCTGCATTTATGAACCC
7 EGFR	AAAAGAAACTCCTACGTGGTGTGT	GGCTTTGGCTGTGGTCAACTTA

7 EGFR	CCTGGACCTTGAGGGATTGTTT	CCACCTCACAGTTATTGAACATCCTC
7 EGFR	TTTGAAGATCATTTTCTCAGCCTCCA	GCCTTTCTCCACTTAGATTTTCTCCAAT
7 EGFR	TCATTTCTTCCAGTGTTCTAATTGCACT	TGGAAGCACAGACTGCAATTTGTA
7 EGFR	CAGCATGACCTACCATCATTGGAA	CCCAAGGATGTTATGTTCAGGCT
7 EGFR	CACCCTGTTGTTTGTTTCAGTGAC	GGAACAGGAAATATGTCGAAAAGTTCTC
7 EGFR	GGTCAGTTTTCTCTTGCAGTCGT	CCGGAGGTCCCAAACAGTTTTT
7 EGFR	TCAGGAAACAAAAATTTGTGCTATGCAA	GGACCCATTAGAACCAACTCCAT
7 EGFR	CCACCGTCATCACCTTCCTTT	CCCTTCGCACTTCTTACACTTG
7 EGFR	GACAGCTATGAGATGGAGGAAGAC	GTTCCTTTGGAGGTGGCATGA
7 EGFR	CTTAACTCAGGCCCGGGAAA	GTCTGAGGCTGTTCACTGACTTAC
7 EGFR	GGCAATAGACCCCTGCTCCTA	CGTCGTCCATGTCTTCTTCATCC
7 EGFR	CAACTTCTACCGTGCCCTGAT	AGTAGACACAGCTTGAGAGAGAGAG
7 EGFR	CCCAAATTAAGAAGAGCAGTGTAGAGAAC	GGCAGAATAACATGGCTTTGAGACT
7 EGFR	CTTTCTCCAGGCCAGGAAATGA	GCTGAAACTCTGACGGCAGAA
7 EGFR	AGTCATGCTTAGGATGGATCCCTT	ACAGACACGTGAAGGCATGAG
7 EGFR	TGCCTGTGGATCCCTAGCTAT	CGTAGCATTTATGGAGAGTGAGTCTTTAA
7 EGFR	GTGTGTAACGGAATAGGTATTGGTGAAT	AAGCAACTGAACCTGTGACTCA
7 EGFR	ACCTCCATCAGTGGCGATCT	CAGAGGAGGAGTATGTGTGAAGGA
7 EGFR	TGAGGTGACCCTTGTCTCTGT	CCAGCACTTTGATCTTTTTGAATTCAGT
7 EGFR	AGCTCTCTTGAGGATCTTGAAGGAA	GGAAATATACAGCTTGCAAGGACTCT
7 EGFR	GAGGACATTCACAGGGTTCAGAA	GTTCAGAGGCTGATTGTGATAGACA
7 EGFR	GCTGGCTCTGTGCAGAATCC	TGTCGAATGTGCTGTTGACACA
7 EGFR	ACCCCGAGTATCTCAACACTGT	CCATTTGGCTTGGCTTCCTTG
7 EGFR	GACTACCAGCAGGACTTCTTTCC	TTTTAGGGCTCATACTATCCTCCGT
7 EGFR	ccctgACCGGAGTAACCTT	TGTTTCCAGACAAGCCACTCAC
7 EGFR	CCCCTGACTCCGTCCAGTATT	cgAGACACGCCCTTACCTT
7 EGFR	CCAACAGAGGGAAACTAATAGTTGTCT	GCCTCAGTACAAACTCATTAGCATCA
7 EGFR	TGAATTCGGATGCAGAGCTTCTT	CCAAAATCTGTGATCTTGACATGCTG
7 EGFR	CAGGAACGTACTGGTGAAAACAC	CAGGAAAATGCTGGCTGACCTA
7 EGFR	CCACACTGACGTGCCTCTC	GTCTTTGTGTTCCCGGACATAGT
7 EGFR	AGCTCATCACGCAGCTCAT	ATGTGAGGATCCTGGCTCCTTA
7 EGFR	TGATTGGGTTTTGGCATTTTCAATACA	GGAGACTAAAGTCAGACAGTGAAGATTT
7 EPHB4	GGAATCCCACCTTGGCTGTG	CAAGGAGACCTTCACCGTCTT
7 EPHB4	TGTCCGCATCGCTCTCATAGTA	GCTTCGCACAGGTTGGGT
7 EPHB4	CGTGGCGTACACGTGGA	CCCTTGCTGACCTCCTTCT
7 EPHB4	GGTCCACCTCAGAGGTCTGT	CCCAGCCAATAGCCACTCTAA

7 EPHB4	AGACGGCTGATCCAATGGTG	CATGCTTTTGATGAATGCTTTCTCTCT
7 EPHB4	AGCAGTGATGACTCTCTGGGA	CCTGGAGTTACGGGATTGTGA
7 EPHB4	CCAAATGACATCACCTCCCACA	CCAGCTCACCTTTTCTCCTTTTTCT
7 EPHB4	GGGAAGCTCCAGCTCTCA	GGTAGCTGCGTGGTGGAT
7 EPHB4	CCATCCTCACGGCAGTAGAG	CCCTGCACCTCTTCTACAAAAAGT
7 EPHB4	AATCGAGTCAGGTTCACAGTCAG	CCACCGGGAAGGTGAATGT
7 EPHB4	CCCAGACGCAGCGTCTT	GGTGAGCTGGGAAGAACACA
7 EPHB4	CACCTGCACCAATCACCTCTT	CCGAGTCCAGTGGGAGTTAATTC
7 EPHB4	CTCCGAGGCCCAGATGTTG	AGTTCGGATCCTACCCGAGT
7 EPHB4	CCTTCCCCAGGCTCACC	AGCGTGCGGTTCCTGAA
7 EPHB4	CTCTGCCCGGTTTTCTGAC	TTGCAAGGCAGGCTAGTGAT
7 EPHB4	CCTAAGAAGCTCACACCCAGT	GCTCGCAACATCCTAGTCAACAG
7 EPHB4	AGACACTTTGCAGACGAGGTT	CCACCCTATCTGCTCTCCAG
7 EPHB4	TCTGGGTGTCAGAGCCCTTA	CCTGATCAGCAGCCTTTCCTTC
7 EPHB4	CCAGCCCCCAAGTCTCA	GCCCTGGGTGGTGGTTC
7 EPHB4	AAAGGTATAGGTGAAGTCAGGACGTA	GGAGTCTGGTGGCCGAGA
7 EPHB4	TCCTCTCGGCCACCAGA	GCTGCTGGTGCCTCCATT
7 EPHB4	GGAAGGAGGGCCCATTCTC	CCTCCCTCCACCAGCTCAT
7 EPHB4	GGTCTTTCTGCCAACAGTCCAG	CCTGGGCCTGTTAGCATCTTT
7 EPHB4	CACTGTCTGTCCTGGCTTCTTA	ACAGCATGCCCGTCATGAT
7 EPHB4	CGTTCTCCATGAACTCTGTGAGA	TGTGGCAATCAAGACCCTGAAG
7 EPHB4	GGCCTCGCTCAGAAACTCAC	GATGACCTCTGCGTCCTTGTT
7 EPHB4	GGTGTCCCAGCCAGGAGA	CCTCACCCAGCAGCTTGA
7 EPHB4	CCTCGCCAAACTCACCTTCAAA	TCGATGTCTCCTACGTCAAGATTG
7 EPHB4	TGACCTCGTAGTCCAGCACA	GGACAGAGCTACCATGCACAAG
7 EPHB4	CCCTCTCCTCCCTCAGTTTC	GACTCTGTCTCTCTGTCTGTCTCA
7 EPHB4	CCAAGCCCCTATTCCCATCAAA	TTTTGTTCCTTTCCCATCGGCTA
7 EPHB4	TGGACACTGGCCAAGATTTTCT	GCTTTTCTGATTGGCTTACAATCCT
7 EPHB4	CCACTCTGCCCCGGAAAA	GTCACTCTGGCGGGACAC
7 EPHB4	CAGCTCCTGCCACTGCTTA	CTCACACCCTCTCCTGGAC
7 EPHB4	CCAAAAGCTGAGTAGTGAGGCT	AGAAGTGTCCGTTCATTGGTGTT
7 EPHB6	GTGCCTGGGCACATGAAC	GGATGGAGCCGATCACCA
7 EPHB6	AGCTTCCGGAAAGACTCTCCT	GGTTTGGCTGTGGAGTTAATGGT
7 EPHB6	CCACTGTGTGCCCCTCTTATTTC	GCCAAGGGATTCAGGTTCAGAAT
7 EPHB6	CCCTCAGGGCCCAAGTT	CTCCATAACTCATCACTTCCCACAT
7 EPHB6	GATGTCTGGAGCTTTGGGATACTC	TGTTCCAACCTGGGAGGGAT
7 EPHB6	GGCTAAGATGAAGAGGAGACCTTGA	GGAGAAGTTGTCCTGGTAGCAC
7 EPHB6	GCTTTCAGCCATTGGACTGGA	TTCTCCGAGATCCCCAATACGA
7 EPHB6	ACAGACCGACTAAAGAGCAGTCT	CCCACCCATTTCCTCTCCTCA
7 EPHB6	CCCACCCCAACCTACACCTAT	CTGGAACTGACCCAGCACT
7 EPHB6	GCCTGCAGATGACCTTCCT	CACCCAGGCTGGACTGAC

7 EPHB6	CACTCTCCTCTGCCTCCTCA	CCTCTGGTGGGTCGGAACT
7 EPHB6	CTGCCTGGAGGGCTTCTAC	GGAAAAACAAGTCACTCCAAATGCAA
7 EPHB6	GGTGGACGCCCTGAAGATG	TCTCTATGGCAGGTCTGGGTT
7 EPHB6	GCCTTTCACAACACGCTCAT	TCCCAATGACCTCCTCAATCTTGATA
7 EPHB6	CGGGAAGTCGATCCTGCTTA	TGTGTTTGGATGGGACTCTGG
7 EPHB6	AAGAGATATGTCCCCTTCCCTATAACC	ATGCCTCAAAGGTCCGAGTC
7 EPHB6	GTTCTGGACGACCAGCGA	ACAGAGAAGTGGAGTCGAATGTG
7 EPHB6	CAATTGGTTGCAGACACACTTTGT	CTCCTCAGCCTGACGGTAG
7 EPHB6	CCGGGAGACCTTCACCCTTA	ggaggGAAAGCTCTCGTCTG
7 EPHB6	TGGACCAAGGTGGACACAATT	CCAAAGCTCCGCTCTTTGAC
7 EPHB6	CCAGGGACTCCTATCCCCATA	GGATGTTCCCATTGGTCTGGTC
7 EPHB6	CAACAGCATCACGGTGTCC	TGTGAGGCCTATGAGCTCCTA
7 EPHB6	GAGAAGCTGGCCCAGATTTGA	CTGAGTCACGGGTATCGTCATT
7 EPHB6	GGCCCTTGGACTGCCATAT	GCCTTCACACTCCTTGCAC
7 EPHB6	GGACCTGCTCTTCAATGTCGT	ACTAACACTCGGCTCTCAGTCA
7 EPHB6	GCAGGGATGAGGTCCACTTC	TGAGCTCAGACACCCCATTA
7 EPHB6	CATCTTAGAGGTGCAGGCTGT	CCAGGCTGGAGATCAGGAGTAG
7 EPHB6	CCACCCTCTTACCCAACAAAGT	GCCAAGTGTCCAACATAAGTAGATGTAA
7 EPHB6	GGTACTAAATGCAATAGAGCAGGAGTTC	GGTATCTGGCTTGCGGATCAT
7 EPHB6	CTGGTGGCTGCATTTGACAAG	TCTCCTCAGTTTTCTTTAGGTTTGCAT
7 EPHB6	GGGCTGGACTGCAACTGAAC	CCTGCGTCTCTGGAAAGGAA
7 EPHB6	CCGTGCTCCGATCCTTTG	CACTTGCCCTCCCCGTT
7 EPHB6	GAGCCAGAGGAGGATGGAGTA	GCAAGTGCAAGACGAGTGG
7 EPHB6	GCTGTTGTCTGCTGTGCAGTA	GATGGACGAAGGCAAAGCTG
7 EPHB6	CAGGGAGTGAGTGGCTGTTA	GGAAATAGACTTTGCCCCCGTAG
7 EPHB6	GGCTGCTGCCATGCAGTA	CTGTGTCCCCAAGGCTGT
7 EPHB6	CTGGCCACATCTATGGTTTCCA	CCTGGGCCTCTCCTCACTAC
7 ETV1	CTAACCTCTTCAGGCTCAATCAGT	GAATGTATCGGGAAGGACCCA
7 ETV1	TGATCCTCGCCGTTGGTATG	CCCATAAAGTGGTTGATTGTGGTTTAA
7 ETV1	TTCTAGAAAACTGGTATCTGCAGGT	ATCATGCATCTCCAAACTCAACTCA
7 ETV1	CCGGTCAGGTTTCGGTGTA	CTCTTCTTTTGTCCCCTGGGTAG
7 ETV1	CTTTTAGCAGGGTGGAGAGTGT	CTTTCCCTTTGTAGAGTCAGCGT
7 ETV1	AATAGCAAAGGTCAATTTCAGCAAACAT	TCTTCTTTCCACCTTGTTCACAACA
7 ETV1	AGCATTAGATACACACCTAACGTTCTG	AGAGCAGCGCATATCATTATTTGGT
7 ETV1	TCGCTTCAGCAATGCAATGATATG	AAAAACAGGCCAGCTATGAACTATGATA
7 ETV1	GCGGAGTGAACGGCTAAGTT	GGACATTTTTGTGGGTTTGGAAGtaatt
7 ETV1	CCCCGACGGAGTCAAGTTT	ACTGAGCTGAGATTTGCGAAGAG
7 ETV1	CATGACACATGAGGTGGCTCTT	ATCTAAGTCAATTACAGGAAACATGGCT
7 ETV1	TTGGGATGTCTGATTATTCTGAACGG	CTCCAGACTCTTTTTGACCAATATTGTT

7 ETV1	ATGTGACAAGGGAGGTGAAAAAGT	GAGTACCACGACCCAGTGT
7 ETV1	CCAACCATGGTGTTGTGTTCAT	CTGAACCCTGTAACTCCTTTCCTC
7 ETV1	CTTGGCATCGTCGGCAAAG	CACTAAGAAATGGAAAACCGTATCCG TA
7 ETV1	ACTGTTTTCAGAAGAAGCGAAGGT	ATGTATTCCTTTTTCATCCACAGGCT
7 ETV1	CTACATACATACACCTGGTGGCT	AAAGAACCCCACAGTCCATGTT
7 ETV1	TGCAGGCAGAGCTGATTTCT	AACACATTAATCTCCATAATTCCTAC AGCAT
7 ETV1	GCAAAAACGCCCTGCTTGAC	TGAAGACAGACATGGAACGTCAC
7 ETV1	CACTGTGTCCTCCTCGTTGAT	GAGGATGGGTTTTTCTCTTCCATGTTA
7 ETV1	AGGTGCTGGTACAATGTAAGACAAG	ACCTGACAATGATGAGCAGTTTGT
7 ETV1	CAACTTTCAGCCTGATAGTCTGGT	AGGTTTGATCTCAGCCCAAGAAG
7 EZH2	AGATGGACACCCTGAGGTCAAT	AGGATACAGCCTGTGCACATC
7 EZH2	GCAATGAGCTCACAGAAGTCAG	AATGAACAATTTCTCCTTTCCTCTCCT T
7 EZH2	GCATGTTCTTTTTCTAAATTGCCCACA	GGACATCTCCTTCCTGTTGTTTCAG
7 EZH2	TGCCTGCAGTGTCTATCTATGTTG	GCTCTGTGACTGTGCCTCTTG
7 EZH2	CTAAGGCAATCCTGACATTTGCATC	AGTGTGACCCTGACCTCTGT
7 EZH2	TCAGCGGCTCCACAAGTAAG	AGAACCTCCAAACCTCTCTCTGAA
7 EZH2	TGTTTGGACAACGAGTACAGTTTTAT CT	GTTTAGAGTCCTCATTGGCACTTACTA TG
7 EZH2	ACCTAGCAATGGCACAGAAATTGT	GACCAGTGCTTACATTTGGTTTTGTAG
7 EZH2	GCCACCTGAATACAGGTTATCAGT	CATCTATTGCTGGCACCATCTGA
7 EZH2	TGAATTCATTTTTCTGCACAGGATCTT T	TTCTGTAGTCTACTTTGTCCCCAGT
7 EZH2	ACATCCTTAATCCTCACAACACGAAC	GGGAGAGAACAATGATAAAGAAGAA GAA
7 EZH2	GAGGAGCTCGAAGTTTCATCTTTCTT	CGGCTTCCCAATAACAGTAGCAG
7 EZH2	TCCTTTGATTCCAGCACATTAATGGT	CCCTGATGTTGACATTTTTCATTTCGT
7 EZH2	GCCTCCCAGCTCTGAAACATA	AGGCTTGATCACCTTTATCCAAAAGA AT
7 EZH2	TCCATCATCACAGGACTGAAAAGG	TTCACTGGGCTGTGCTTACTTT
7 EZH2	AGCATGGGTGCAGACAACATTA	GCTCTAGACAACAAACCTTGTGGA
7 EZH2	CCAAATGCTGGTAACACTGTGG	AGTGGAACTGGAAGAGTGAAAAAGT AAG
7 EZH2	GTGAGTTACATACCATACAGGGCAAG	CCGCCCACCTCGGAAATT
7 EZH2	ATAGCACTCTCCAAGCTGCTTT	GCTTACACTCCTTTCATACGCTTTTC
7 EZH2	AGCAGTCATATTTAAAACATCGCCTA CA	ATGTCAGATATAAAGAACTCACCGAA CAG
7 EZH2	GCCTGGGAGCTGCTGTT	AGCCATTCCTTTATGTTTTAGGCAAGA T
7 EZH2	TGAAAGAAAGCTGTAATGGCTACACA	GGTGAATGCCCTTGGTCAATataatgat

7 EZH2	CTTTCTTCAGGATCGTCTCcatcatc	ACTTTTGACATTATTGCTTCTCCTGTGT
7 EZH2	GTGTCTCTCAATTCTTTAGCCCCTTT	GGGAGATGAAGTTTTAGATCAGGATGG
7 EZH2	CATCCTGATCTAAAACTTCATCTCCCA	GAGAACTGGGTAAAGACATGTACACA
7 EZH2	CCTTTACTTCATCAGCTCGTCTGA	GCTTTTAGAATAATCATGGGCCAGACT
7 EZH2	GGTCCCTTCTCAGATTTCTTCCC	GTTAGTTTGCTGCGGATTAAAACACA
7 EZH2	TTATACTGTCTTGATTCACCTTGACAATAAA	ATGCAGTTGCTTCAGTACCCATAA
7 EZH2	GGGAGACCAAGAATACATTATGGGT	TGATGGCTACAGCTTAAGGTTGTC
7 EZH2	GCAATTGCATCAAAGCAACAAATAC	GGCTCCTCTAACCATGTTTACAACTAT
7 EZH2	CCGTGGATGATCACAGGGTTG	CCACAGGTAGTAGGGAAGAATGTACT
7 EZH2	GTCCAGAGTTCACAATCCAGTAGAA	ACACCAGTGTGTCTCTTTGCAG
7 EZH2	GCTAATAATGAGAGGAATGGAAAGATGCT	GGATTTGTAGCTTCCCGCAGAAA
7 GRM8	AATAGCACAGACTGAAGCATCTTTAGAG	ATTTGGTTAGCTTTCATCCCCATCTT
7 GRM8	GCTGACTGGGCTGTACCAAAA	ATGGTCACTTGTACTGTTTATGCCAT
7 GRM8	TCTCTGGGACACCTCTCGTTTTA	CCCCACATCATCATTGACTATGGAG
7 GRM8	TGGATCTAGTGTCCGCTGCT	CAGCGCCCAAGTTCATTAGTC
7 GRM8	CATGCCAAGTCCTAGGAAGACC	GATCGTGACCTTTGTCCGCTAT
7 GRM8	GTGATCACCAGCTGAGATGCT	CAGATACAATCATATGCTCCTTCCGA
7 GRM8	CCCTCACGATAGGTGTGTCATT	TGCCAGCTTATCCCCATCATC
7 GRM8	AGGGAGAATGCCACTCCAATTT	TGTGAAGGTTACAACTACCAGGTG
7 GRM8	CAAAGTTCACAGGACAGCTCATC	CATGCAGTGGGCTCATAGAGAA
7 GRM8	CAGACGCCGGGTGAGTATG	ATTACAAACCCGTGGCTAGGATTAG
7 GRM8	CCACTCTGCCTGGGTATCTTCTA	TGGCCTTTGTCCACGAATGAG
7 GRM8	CAAGTAGCTCTTTCCCATCAATGGTA	GAATTGCTCGGGATTCATCTTATGAAC
7 GRM8	TCAATTACAAATTGGACCTTTCCTTCCT	TCGTGTCTCATTAAGTGTTCCTCAAAT
7 GRM8	CCTACAGATAAGAGAGCACTTGGAGAT	TGGAATTATGTTTCGACACTGGCT
7 GRM8	CTCTCACCATAGTTCCCCTCAGA	CAGAGCTAAGTGATAACACCAGGTAT
7 GRM8	CGGAACCACTCGAGAGAAAAAGTC	ATCATTCTTCAAGATTCGTCATGGGT
7 GRM8	GAGTGAATTTTTGCGGTCTCATGTT	GTCCTCATTTATCTTAACAGCTTCCTCT
7 GRM8	CACAAATCTGACCCTACATACAGAAGAA	GGTGTTTGCATTGCTCAGTCA
7 GRM8	GCTCTTGACCATCGGAAACTCT	AAGAGGAGCTTCAAGGCTGTG
7 GRM8	TTGCCTTTGAGTTCAGATCCAACT	GCATTTTCTCTGGATTGGCTCAGA
7 GRM8	GAGCCAATCCAGAGAAAATGCC	TGACCTACTGAGTGTATACAACTTCCT
7 GRM8	GGATCAGTTTGCTTTGCATGGTG	CAACAACACTTACTGTCTCCATGAGT

7 GRM8	GCCCAGAGATACTGAAGCACTTAA	GCAGAGAAGCTGGAACAATTTTAGAAAA
7 GRM8	GCATCCCTCCTGGAGAGCTA	CCAGTATCAAATAACCAACAAAAGCACA
7 GRM8	AGTGGCCGATGACTTTGTACTC	CCCAGTGGGCTATCTTACTAAAACTT
7 GRM8	CTTCGAATGGTTTTAAATGTGATGGTGT	TCTTGAAACCAACAGTAAGTCATCTGT
7 GRM8	TTGGCAAGAGTTCGGCTTCT	TGGCTTTCTCTCATTTGTTATGTGTTTTG
7 GRM8	ACAATGGTCAGAAAGACAATGCCTTTA	TGAAGTGTGCTAATGGAGATCCAC
7 GRM8	CTTGTCGGGCTTGGTGAAAATG	CGTGCTCTAGGGACACCTATG
7 GRM8	ACGAATGTTAGAGACTGCTCCAAAG	TGGAGGCCATGCTTTATGCAAT
7 GRM8	TCAGGGTCCTTGTTAATCTGGTCA	CCAGGAGTATGCCCATTCCATA
7 GRM8	CCCCAAAATAATGTCCCCATCCA	GGATTGCAATACCACCTGTGGAG
7 IKZF1	CCTGGCTCTTGTAGGCACTTA	GAGATCTCTCTGATCCTATCTTGCAC
7 IKZF1	ACAGTGAAATGGCAGAAGACCT	ACTTGCATCTAAAACACCCACATAGTTT
7 IKZF1	TGTATTGCATGCATTCCCCTTACA	TGCTTTCCAAGTAGTTGTGGCA
7 IKZF1	CAGCGAAGCTCTTTAGAGGAACA	CTCTGCTCCTAAGGCTGCAT
7 IKZF1	ACACTGAGTGGCCTCCTGTA	CCGAGGCATCAAGCATTCGTAA
7 IKZF1	GGGAAGAATGTGCGGAGGAT	CCACAGATATCACACTTTAGTTTTCCGT
7 IKZF1	GTTGGAGGCATTCGACTTCCTA	GAAAAACTGAGCCAGGCCTTAC
7 IKZF1	CTCGTAGCATCGTCCTCATGT	TGGCATTTGAAGGGCTTCTCC
7 IKZF1	CGGCACATCAAGCTGCATTC	CGGGCAGAGTGGAGGAATC
7 IKZF1	ACTCACTGTCGCCTGCTTTC	GATGGCGTTGTTGATGGCTT
7 IKZF1	CGAAATGATGAAGTCCCACGTGA	GGCTTGTGCAGCTGGTACAT
7 IKZF1	AGGTGGTCCCGGTCATCA	AGTCTTGGCAGCTGTTGCT
7 IKZF1	CTCTCCAAGGCCAAGTTGGT	TTGAGCGACAGCCCGTT
7 IKZF1	CTCATCTACCTGACCAACCACATC	GCACTTGTACACCTTCATCTGCTC
7 IKZF1	CGCCCGAGACTCACACTTC	CCGCTGGTGACCCACTTAC
7 IKZF1	GGGTTCTTTATCTCTCTCTCTTTCTCA	CCACACAGCTATCTCATAAGGTCTCA
7 IKZF1	CTCCGAGAACTCGCAGGAC	GCTGTGGTAGCCGCACAT
7 IKZF1	CCGTGATCCTTTTGAGTGCAAC	GGCAGTCCTTGTGCTTTTCCT
7 MET	AGTTCTATGTTGTCCTTGTAGGTTTTCC	GCTCTCATTTCCAAGGAGAACTCTAGT
7 MET	GGGACTTTGGATTTCGGAGGAATAATA	AACAAAACCTATTTTCCAAGCACACC
7 MET	CTCTTCCTGTTTCAGTCCCCAT	GGGACATGTCTGTCAGAGGATA
7 MET	GTTCATGCCGACAAGTGCAG	CCTGGACCAGCTCTGGATTTAG
7 MET	GTCCACATTGACCTCAGTGCT	GGCCAAAGATAAAATGCTTACTGGAAAA
7 MET	AGCCACGGGTAATAATTTTTGTCCTT	TCCAAAGCCATCCACTTCACTG
7 MET	GTGTACACAACAAAACAGGTGCAAA	AGAGGAGAAACTCAGAGATAACCAATACA
7 MET	ACAATACAGTACATTCTCCTATGTGGT	CACCAGCCATAGGACCGTATTTC

7 MET	GTTTTGTTTTTATCTCCCCTCCAGGAT	CCACCAATTGAAATGTGTCTAGAATTCC
7 MET	CCTAAACAGTGGGAATTCTAGACACA	ACACACACACAAAACAATCTGCTTAATT
7 MET	GTCTGGATTCACATTAACTCTATGACCA	ACTTCGCTGAATTGACCCATGAA
7 MET	CTTTGCAGCGCGTTGACTTA	ACAGCTACTCTCAGAAAGCACTTAC
7 MET	TGTTCAGTGTGTCAAACAGTATTCTTGA	CATAGACAATGGGATCTTCACGGTAA
7 MET	AACCGAGAGACAAGCATCTTCAG	CCAGAGATAAAACTGCAATTCCTCTTGA
7 MET	TCCTTTGCCATTGTTAGCATTCCT	CATAAAACGGCTTCAGAATGTAAGTGTA
7 MET	CTGAAGCAGTTAAAGGTGAAGTGTTAAA	ACATCATGAGAGGAATGCAGGAATC
7 MET	CTAGATACCCCTCTGGAAGCTCTT	CGATCGCACACATTTGTCGTG
7 MET	CCTTTGTTCAGTGTGGCTGGT	aaaACATGTATGCCAGCTGTTAGAGA
7 MET	TGTTACGCAGTGCTAACCAAGTT	GCAAACCACAAAAGTATACTCCATGGT
7 MET	TGTTTCCAAAGAACAGTTACCCATGA	ACCACTGGCAAAGCAAAATAGAAAAC
7 MET	TGGAAAGAACCTCTCAACATTGTCA	GAGTAGAACCATTGCTCAAAGGACTTA
7 MET	TGGAGTGTATTCTCACAGAAAAGAGA	ACACCCCGAAAAGAATGTCATCA
7 MET	GACAAATAGGAGCCAGCCTGAA	GCTGGAGACATCTCACATTGTTTTTG
7 MET	GGCCCATGATAGCCGTCTTT	CTTCGGGCACTTACAAGCCTAT
7 MET	ACGACTTCTTCAACAAGATCGTCAA	GCCCTGGATATTCTTTTTGAGACTGATA
7 MET	GCAAGAGTACACACTCCTCATTTGG	TCACAACCCACTGAGGTATATGTATAGG
7 MET	CCCAAAGTGCTACAACCTGTGT	CCTGTGAAATTCTGATCTGGTTGAACTAT
7 MET	TTTCTTCAACCGTCCTTGGAAAAGTA	CTTTCTCTTTTTCAGCCACAGGAA
7 MET	AACAGCACTGTTATTACTACTTGGGTTT	CACAAGAATCGACGACAATCTTAAACTG
7 MET	TGTATGGTCACATCTCTCACCTCA	TCTCCCTTGCAACAAGTAAACAGTTAT
7 MET	CCTTATCCTGACGTAAACACCTTTGAT	GGCAGGCATTTCTGTAAAAGTAAAGAAC
7 MET	CCCACAAGCCCTGCTAATCTG	AGTCCAGGAGAAAATTCACATGAGG
7 MET	TTTCTCGATCAGGACCATCAACC	TGGGAAAAACCACGTCTATGGAAATT
7 MET	GCTTTTCTAACTCTCTTTGACTGCAGA	GGATTGTGGCACAGAGATTCTGATAC
7 MET	ACAACCTAACCAGAAAATTCCTTGGAT	CTTCCTTACCACATAGGAGAATGTACTG
7 MET	GAAGTTAATGTCTCCACCACTGGATTT	CTTCACTTCGCAGGCAGATTC
7 MET	CCAATGTCCTCTCGCTCCTG	ATGGCTGGCTTACAGCTAGTTT
7 MET	GGGTGGTAAATTATAAAGTTGCTATGGA	CAGGGAAGGAGTGGTACAACAG
7 MET	CATGTCAACATCGCTCTAATTCAGAGAT	GCTTTTCAAAAGGCTTAAACACAGGATT
7 MET	TTTTCATGTTAGATGGGATCCTTTCCAA	AGTTTGAGCTGATGATTTAAGACAGTGT

7 MET	CCTTGAACCTGTTTTGGCAGAT	AGTTGGGAAGCTGATACTTCATATTCAC
7 MET	CGACATGTCTTTCCCCACAATCA	GTTGATGAACCGGTCCTTTACAGAT
7 MET	AGTAGTCTTTCTGTACCTCTTACGTTCT	TGATATCCGGGACACCAGTTCA
7 MET	GCACTAGCAAAGTCCGAGATGAAT	CAACCTTCTGAAGGTCTTCCTCATTT
7 MET	CCTGGGAGCCAAAGTCCTTTC	CGTTTCCTTTAGCCTTCTCACTGAT
7 MET	CGAAATGCGCCCATCCTTTTC	AGGATACGGAGCGACACATTTTAC
7 MET	CTTGGTGCCACTAACTACATTTATGTTTT	GAGCCATGTTGATGTTATCTTTCCAAAC
7 MET	CCCAGATCATCCATTGCATTCGAT	TGCTTTCAAAGGCATGGACATACTT
7 MET	AGCAAAGCCAATTTATCAGGAGGT	CCTCCGACTGTATGTCAGCAGTA
7 MET	CCATGTGAACGCTACTTATGTGAAC	GACTGTTGCTTTGACATAGTACTAGCA
7 MET	CTGAGTTCAGAGATTCTTACCCCATT	GGAACAGAACCTGATTATTCTTGTGTGA
7 MET	AGGGAAACTCTAGATGCTCAGACTTT	ACACTTCCTTCTTTGTGGATCTCTTTTT
7 MLL3	AAACCAAAGTGGTATGAGAAAGCTCT	AAGACTCCTGCTGTGTGATGAC
7 MLL3	ATTCTTAACATCCAGTAGGGCAAAACA	CTGGTCCCCAGACATTTCAGA
7 MLL3	CAAGCCTCACCCCAGGTAATA	TGTCACAGAATGTTGACTTTTCCCA
7 MLL3	aaTGCATATGAGGCCAAACAAAAACA	CTTAGTTCATCCTCGGCTCCAA
7 MLL3	TCTACATACCGTGAGTTCCAGATTTTG	GTCAGAAGATGGAGCTCAGCTTT
7 MLL3	GAAGGATCCAAGAAACCTGTGTTCA	TGTGCTTATCTTCATCTAGGAAGCTTTC
7 MLL3	TGAGCTCCATCTTCTGACAGATTATCTA	TCTGCTTCCTTGTCAAAAACCATGA
7 MLL3	GAAGCTGTAAGATGGTAGAGCAAATGA	TTACCAACAGCAGGATAACTTATGTCC
7 MLL3	CTGGATGATAACACTTCCCACAGAA	AATTGCAGAATATGTATAGAGTGTGGCA
7 MLL3	GTGGTGCCACTGAGAACTAGAC	AAAAGCTTAATGTATAATCCACAAATGATTCAT
7 MLL3	cccagcTCTGTTTCCCTCTTCTA	GACCTAGATATGCTGTTTTCCCAAAAG
7 MLL3	AGGATAGGGTCCACGCTGAT	CCGCAGTGCCTCATCCAG
7 MLL3	GGGAGACAGTGCACATCCAAA	TTCTTTGTCATGGTTTCTTTATGTTGCC
7 MLL3	TCTGGTTGCCAGTGTTGAAGAG	CCATTAATTACCACAAATCCCAGTGATT
7 MLL3	ACTCAGGTCTTTTAGAAAGCCTAGATGT	AATGTCAATCAGAGTTCACGACCAT
7 MLL3	CATTTTGTCTAGTTCAGGACTGAGGAT	CCTTACTCTTGTAGATATTGGTCCTGTCA
7 MLL3	GAGTAAGTAGCACTGCACAGCAT	AGCACTCGCTATGCCAATAGG
7 MLL3	TCTCCTCAATGGAGCACAGGTA	CAATTGGTCAGCTGCTTCCAC
7 MLL3	TGGCGTAGTGTAAAGGGAAATAATAAGG	AGTGAGCAGGTATCCAGATCTGT
7 MLL3	GAATGGAATGCTTGCATCTGCT	TTGCAGTCTTCAGGAGGGTCTA

7 MLL3	GAGGCTCCTCCTTGACCAATG	GCAGACTTTGATGTGTGTGTTTATGTTT
7 MLL3	GCACCTCATCACGCTGAACA	ATTTCGATGCACCAACATTTATCACTTC
7 MLL3	ATTGTGCTTTAATGGCGCAAGT	TGGTCCACGGAGGTCTATGAG
7 MLL3	CAAGTGGTCACACTGAATCAGGAA	CCTGTGGCATGTGTGAGAAAAA
7 MLL3	GGGAAAAGCTGGAGCATTTCAGA	CTTGTGAGTGGTTATTCTTCATTTTGCT
7 MLL3	CCATTTCACAATACACAACTGGCA	GGCCAATGGTTTTGCAACAACT
7 MLL3	GCCAGACAGCAGGGTTTGTAA	CCCGTTCTGAACCTAAAATGAGTG
7 MLL3	CGGCTTTTCCAGCAAGTTCTTC	TCATTGCATATTCGCTCAGTAGCATA
7 MLL3	CTTAACACAAACCTCTTGACATGGG	TTGTGTCTGCTTTGCTTCTTTTCAG
7 MLL3	ACATACAGCAATCGCAAAAATGTCTTT	TCCCTCAGAAAGTCTTGACACAGATA
7 MLL3	ACATACTGCTTACCAGCAATAAGAAGAC	GAGGGAACAGTGTTACAGAAATTTGAC
7 MLL3	CCACAATAAACGCAACATACAATAATATCA	GCAATTGCACAATCCTCTTATCCT
7 MLL3	ATAGAAAATAAGGCTTGTACCTGGCAT	GCCTCTCATGAATGGCCTTATTGG
7 MLL3	ATGACAGATTTCATTCTGCCTTCCA	GGTCATTGAGTACATCGGGACTATC
7 MLL3	GAAGCGCCATCTTTGCGA	TCCATGTGTCTTTCCTGGTGACTA
7 MLL3	CCTGTTGGCTACTTCGTTTCGAAT	CCGAGGGTGAAACCTTTGTATGAA
7 MLL3	ATGTGGGAGATGAGGACTGGAT	gctTTACTTCAACTCCTTGTTACTTGTGA
7 MLL3	CAAATGCTACATGCAGTGTACAAACA	GGGAAAACTGCAGTGGAAGA
7 MLL3	CCATCCATGCTGTCCTCATCT	TGTATACCTTCAAATTGTGCCTTATGTTGA
7 MLL3	CCTCGACTATCATCCATTTCACCA	AGAAGAAAACGGTCAAAACCAAAATTGA
7 MLL3	CCATTTCACCATCCCTTGAATGCT	GGTGTGTGTTTGACTGAATCAGG
7 MLL3	TCCCATGTCAAGTTCTGGATCTGTA	ATGGCAAGGTAGTTGAATTGGATACTT
7 MLL3	CCAGGTTGGGATCATTAGTTTCCAAATT	CTTGATTCTGATGACCCTTCTGTGA
7 MLL3	CTCAAGGTCTTTAACATCCAGTTCCT	AGAAGCTCCTTTGTCAACATCTGT
7 MLL3	AGCAAAATATTACTTGTAAATAGGCAGGGA	GTGAGCAGTTACCAGATACTTTAGTTGA
7 MLL3	GCAGGGAAAGTTTCTTCAAGCTTATT	TTTATAAATGTGACTGTTGTTTCAGGCT
7 MLL3	CCAACATTTAAAGTCGAAACTGCTTACT	GAATAACGCCTGGATTTATCTTGCC
7 MLL3	ATTATCAGACGTTGTTTCAGACGGT	CCTGTGCACCCAGATTTGGAA
7 MLL3	CTTCTTGTTAGAAGGTTGGTTTCTCCAT	AGCTACTTTTCTCCTTCTCTGACTTACA
7 MLL3	CCCGATGGCCTAATGTCTGAATTAT	GAAAAGAACCAATCGAGAAACTGCTG
7 MLL3	GAGTGGATGCCTGAATGACATTTG	CTGCTCACTCAGACCTAAATGATGG
7 MLL3	GATTTCATGAATGTCTAGATGCCCAGT	CCAAAATGGTGGCCCTTAAAGGTATAA

7 MLL3	CCAGATTGTTTTGTGCCATCACTTTA	ATATTTGAATTACTTCTTAGTGTACGTTGAACT
7 MLL3	CTTGTTGAGATGGTGGCACTTG	CAGCGTTGTAGAGGCATCTTCTAA
7 MLL3	TCACAAGGATGCAAAGAAGTCTTTTCT	GGTGGAATCCAAATGTGAAACTGAAAAA
7 MLL3	TGAGCAAGGAGTGTCAACATTATCTTTA	GGTTCTCTCTGATAAACATTCACCACAG
7 MLL3	AGTACTTCCGTTTTTACCTCATTGGT	GAGGAACTAGACCTTCCAATTGATGAT
7 MLL3	GAACCAGAGTTTTGTTTTCTTGTTCCTT	GGTCAGGAGAGTTTGATATCATTGCATA
7 MLL3	CTGCTCTAAATGACTCACCCTCA	AGATGGAGGAAGTGAAACCAAGAAAC
7 MLL3	CTGAACAACATGGCAGATGCAA	ACAGTTTTCTGACATGGTGGCT
7 MLL3	TGAGTCCGTTTGCTTCGCT	GCACTGCATGAACTAAAAACCAAATATG
7 MLL3	TGACCCTTTATCCTCAGTGATCCT	CCCAGATCAGGTCTTATATCTGTAGCAA
7 MLL3	GCAGCTGTAGGATGCAGAGTAA	GTTCAGAATCAAGAAGGAATGACATCAAA
7 MLL3	CAAAATATAAAGTGCCTGGCTCAGT	CCCTTTACACTACGCCAATTTACTAATGT
7 MLL3	GAAGCAGGTTTGACAAGTGGTAAG	CCTTCCTCAGACTGCTGTGA
7 MLL3	TTGCCATCCACACCAAAGCTA	CACTCCAAAAATTGGCATGGGTAA
7 MLL3	AATACCACATATGGTGCTCTTTCTTGT	ATTAGCATGGCTCTGAGAAAGTGTTAT
7 MLL3	AATAGCTGGTTTACCCATGCCAA	CAGACTTGCCTTCGCATGAC
7 MLL3	TGCCAATTTTTGGAGTGACTGAGAT	AGTCATCATTTTCATCATCAGCAGACA
7 MLL3	TGCCTCCTTGGTATGAAACATCTTT	GTGTCCCCACATGAGGAAAGTAT
7 MLL3	CCAACTGTTCCTCAGGACATAATGAA	CCAGATCACTGTGCAGCAAGAA
7 MLL3	TTGAGAGCTTTTACACTACAGCAATCAT	GTGAACGTGGACAAAGCTGTTG
7 MLL3	ACTTACTTCTGTGCTCCCTGAGA	TGCTCATTTGCTCCTTTTCATTTTTCC
7 MLL3	TGGTGTCTCTCTCTGATGTTGGAA	AGTGGTGTTGTCATTGTAAAGTGGT
7 MLL3	TCCAATAGAAATATCAAGACCAAAGGGTTAC	TGATCAGAAAAGATTCCTCAGGCTCTA
7 MLL3	TGCAAGGTAACTGCTCGGAAAT	GTGTACTGTGGAAATGTAGGGAGAAATTA
7 MLL3	CCGCACACCACTTCCAAGAATA	CACCTTGTGTCATCAGTCAGTTTTAATT
7 MLL3	GTCAGGTTCCTCGCTGTCTC	GGTCGACATTGATGACTTACTGACT
7 MLL3	CATCCAGGTAAACTGGGCATTCT	CCTAATTCCAGGTGCCACTCC
7 MLL3	CACCATGGGAAAGGTGGGTT	GATTCGTAAACAACAGAAAGAACATGCT
7 MLL3	TGCTGTTTGATCCGATAATCTTCAATCA	GCCAAACTGCCCTCTGTGTA
7 MLL3	TGAGATAAAGCAGAAATGTGCAAGGA	AGGATGATGAAGAAAACTCTATGCACAA
7 MLL3	CATGAGTTTGCTGGGAAACAGA	TTGATACTTGTGTACCCTGTTTTGACA
7 MLL3	cacagaaGTCACTCGTTACACATTG	AGAGGGTAGAGAAGGACATTGTCTT

7 MLL3	TCCTTCCACTCCCTTAGTTTCATCAT	CAACAACCCTACTTTTTATTCCCCATT
7 MLL3	CGCTTGTGCAGTTGATGAATAAAGAATAA	TTGACTTGTTGAGATTCCCACACA
7 MLL3	AGGTATTGCACATGTGAAAATGTTGG	TCTGGGTGAAATGGCAACTGTT
7 MLL3	GCTTACCAGGGAATTCTTCCTTTAAGTT	GAAGGAGAAAATGACACAATGTCGAATG
7 MLL3	CTCCCACTTAAGCGTGCTTCT	TTCCCAGGGATAAAAATTCAGCCTT
7 MLL3	TGTTAGGATCACTTGCCATTGGATTAA	GGCTTTTGCTATAAATATGTTACTTTTAACTGT
7 MLL3	GCTTCCAGAATTAAGAGGCAGTCATT	AGATGATCAAGCTGGTAAACTGTGG
7 MLL3	GCCCAACCAGACTGAGTTCATC	TTTTCTGCTTGTTTTCACTTTCACAGTT
7 MLL3	GCACGTTTTAATGGAGTAACCGCTA	AGCGAAGGAAGATGCAAACTGT
7 MLL3	CAAAGTCACTAATGAACAACACACCAA	CAAGAAAGCTGGGCGTGAAT
7 MLL3	GTTGTTCTGCATCTTCCTCTGGAA	CGTAAGCAGTATGAAGAGTGGCT
7 MLL3	TCAGTTTGATGAAATGAACAGCTTCCT	GTTCTCTGGTGTCTCCCAACTTC
7 MLL3	GATTTGGCATGAGGACTGGTCT	CATATGCTCATCCTCCTGGAACA
7 MLL3	CATTTGAAGCAGCTGTTGGGT	ATTATGTGGTCATGGTGAAACTACTTC
7 MLL3	GCAGGCCTTTCATAAGGGTCA	GACTCATATGCACGACCCTTGT
7 MLL3	TTACTCCTGAAGTTGGCACAGG	CTCAGTCTGACTCTTTTGGAACAAGT
7 MLL3	GGGCTTGAACTGGTGTACCTA	AGGATGGCAGTATAACACATCAGATTTC
7 MLL3	AATAATGCAAAGACCTCCCTTCTCAAA	GCTCTCGTATTGATTCGGAGCTTTTTAA
7 MLL3	GGTCGTGAGTTAGAGGACCCA	AGTACCCCTACGTCTACATCTTCAG
7 MLL3	CTGTTCATGTTCTGATTCTCTTTGCTTTA	GCCAGAGATAACAGAGCTGCTT
7 MLL3	AAGGGACAGAAATTCCAGGTCTTG	CCCCGGCCTACTACTGTTG
7 MLL3	AGCAACATCATGGGCAGTTTG	CCTGATACATGTTCCCAGACACCTA
7 MLL3	CTATCAAGAGGTGCAGGTGTCA	AGAACAAACTGCAAAAGGCCCTA
7 MLL3	GGGCTGCTGACTATATGGGT	CTCCACGACCTGTTGTAGATTCTTATT
7 MLL3	CATCATACACCTCTCGCTCACA	AGGTCCTGCTGATGACCCA
7 MLL3	ACTTCAGAAATATCAGCTAATGGGTCATC	TGCCTCACTCAGTATCTGCTGTA
7 MLL3	GCTTGTGGCTTTACAAACACATCAT	GGATACAGAGTCCCTTGACACC
7 MLL3	CGGCTAAATGTGTCTGAAAGACCA	AGGGTTTTACTAGGTCCTCAATGACA
7 MLL3	CCTAGCTGTTCCTGAAGATTGGG	CCCACATCCAGCAGTGAATGAA
7 MLL3	GATCACTGGTTCCAGCTGCTA	TTCCACGACAAATAATGACCCCTATG
7 MLL3	ACATATTTCCATTGCCAGGCTGA	AGCTAAAATTGAAGCCACACAGAAAC
7 MLL3	CCCTTGAAGGATGGGCAAAAGA	GGCATCAAGGCGATTGTCTGT
7 MLL3	gctgctCATTTTTCACCTGTTCAA	CCATGATGGTAGTTTTCTTCCTTAACCT

7 MLL3	CCTAGGTGTGTCTGGAGGTTTTG	AGAAGAAATTCTGCTGCACCAGT
7 MLL3	ACCGATGATAAAGGTGTACAGTTTTCC	CCTCACCGCAAGTGTTTTCAC
7 MLL3	aaaGCTGATGGTGATGATGTACAGA	TCAACCAGTTATGAAATCAGTACCAACC
7 MLL3	ACCCTTTGTCACACGTATCACAC	GTGCAGTTCCCAGGACTGATT
7 MLL3	GACTATACGCCAGCATGTTACC	GCTGAAGCAGCCTAATGTACCATT
7 MLL3	TCACCATTGCTTGTTGGAGGA	CAGATGTCCCATCCATGGGTTT
7 MLL3	TCTTCACAGCATTTGATAGTGGCT	GCAGGTTCAATTTGTTTTACTTGTGAT
7 MLL3	GGTTGATTCTGTGGCTACTGACC	TCTGGGTGAATAATACAGCATTTGCAT
7 MLL3	ATTTATTAAGGCACCAGCCTGAGT	GGAAATGTTGCTTTTGTCATGAAGAAG
7 MLL3	TGGTCCATCTGTCAATCCATCAC	GAAGTGGAGCATTCATATTGTAATCCCT
7 MLL3	ACAAGGTGGTTTAAATGTCCCCTT	CAGATGAGCTGAAGGTGACAGT
7 MLL3	CTTAGCCGAGGCTTCAGCTT	TCAGTACAGCAACAACATCTCCAC
7 MLL3	AAGCATATCGCATGCCACTCTAAT	GCCTCTACTTCTACAGGATCTTTTGG
7 MLL3	GGGAATGAAGTTTCCATGTCTCAGA	ACTGGGCCAAGCATATATTGAACTG
7 MLL3	CCGTCAGGAGCCCTATGTCT	TCCACAGCTAAGAAGATCAGTATCTGTA
7 MLL3	CTGCTGTTCTTGCCTTTCTTGAT	GGCCTCAAACAAGTCAGTCTGG
7 MLL3	GGGTCCAGACATGCTACTGGTA	CAGTAGTCTCTAGGGTAAACCATGTTTT
7 MLL3	TGTGAGTTATTTAAAGGCCTAGGCATATC	GATTTGGATTTCCAGGAGGTAGTCAT
7 MLL3	GCTCTTGACTCGGCATGGTA	ATTTGGCAACATTTTGTATGTACAGTTT
7 MLL3	GTTTACCTGCACACCCTGAGAA	TCCCCATCCAATCATGTGTCAAG
7 MLL3	AGATTGCTAGACTCCACCCTGT	TGAACGCACCTTATAGTAAACAGTTTGT
7 MLL3	CGGTGCTATGAAAGGAGGCAAA	CTCCAGTTCTCTCAAGTTTACTTGCTAA
7 MLL3	GGCAAAACTTGCTTGTGTGTGTATA	CAGCTGTACTTAGTCCTGCGA
7 MLL3	GCAATGGAGTTGGCTGAGTGT	CAGGACCCTACTAGTCTTTTAATCTTGTT
7 MLL3	GTACTGCGATGACTTGGAGTGA	ACTCAGAGGTATCTTCAGTTTTCCTTGTA
7 MLL3	GATACCACTGTTTCAGGTTCCTCTAA	AGACCTGAAAATGTCTTCTGAAGTGAAG
7 MLL3	ATGCTATTCCTAAAGACATTATGCCACAT	CAGCCTCCCTCCCAACAC
7 MLL3	CTTCCTGATTGTTATGAGGTGGTGTT	GCTTTTCCAGTTACCAAAACTCTAGGA
7 MLL3	AGGGCAGCTGAAATGGTTTAGG	CCAGTTGTGTATTGTGAAATGGCAAT
7 MLL3	CCAATCCAGCTGCATTCATTCTC	GCAGAAATCAAAGAACAATCTGCAGAA
7 MLL3	TGGTGATACAATGGCACAACATTACA	CTCAGCTCAAAGAAGAGTATATCTGCAT
7 MLL3	TCAGCTCCCAGGTGTTTACAATAC	TCAGCAATGCCTTCTCTTTACCTAGATA
7 MLL3	CAGTTGCTGGTACTAATCTGCTGTAA	GGCAGTATTTGTTCAGAAGATGACTGTA

7 MLL3	CTGGGTTCTGCTTCTCAACTAGTTTATTA	CTGCTGCCAAAGGAGACTCA
7 MLL3	GCTGCCATATTGGGAGTGGAT	TCAGATATAACTGCCCCACCGA
7 MLL3	CAACAAGTGTTTCAGAAGTTCATTCCC	GCCTAAATTGGAGGAACAGAATGGT
7 MLL3	GGTAGTTTCTGAGATGCCTGGAG	CCAATCGCTCATTCAGTTGTATTCTGAT
7 MLL3	CTACAGCGTTTCCTTCTACCTTACT	CAAATCAACAGACGTATGCAAATTCAGA
7 MLL3	TGGTGGATTCTGCATCAtcatctc	AGTAGCTAATAGCAGTCTCCCATGT
7 MLL3	CCTTCGCTCATTAGTCTGCATGA	CCTTCTACAGTTCCGACTTACCTTG
7 MLL3	GTGTCCATGGGTTATAGTAGAATCTTGG	TTTTCTTCTGTGAAGCAGGGACAT
7 MLL3	GTTTCCATGGAGAGCTTGTCTACT	CAAGAGTCGGTGGAACCAGTC
7 MLL3	AGTGACTGCATCTTGGCTTTTACT	GTATTCTCAGAGCAGGCAGCTA
7 MLL3	GGGTCCTAGAGGTTGCATAAAATCA	AGGTAGATAGACAAAGAGCTTTGCAG
7 MLL3	AGCTGGTCCCAGAAAGATTTCC	AATTAATTCACCCTCCACCCAAACTT
7 MLL3	CATCTTTATTAGCTGCCTGCTCTGA	GTGATGATTTGCCCTTACTCATACAAAA
7 MLL3	GCTGCTCCATTTCCATCCTCTG	ACCACCACCTCGGGTAGAATT
7 MLL3	ACTTTCACTAAAGGGATTGTTGTCATCA	CTCCTAGAATTCAGCCCCCAATT
7 MLL3	GCTGGTGTACAAGTTTTTATTGGTAACT	TCTGGCCATACTAGCCCTGTT
7 MLL3	CCGCTGAGCTAGCAAGGAAT	CTTTCTTACCCTGTGTCTTTGCC
7 PIK3CG	CTCTGCCCTTCACTCAACAAAGA	GTGCTTTGCTGAATTTTGGCAATT
7 PIK3CG	TCCCTGATTCAACGACATAGAGTACA	ATTCTTGGCAACTTAAAATTTCACGCT
7 PIK3CG	GATTGTGAAAGACGCCACGAC	AGCCATTAGACTGGGAAAAAGCA
7 PIK3CG	TGCTCTCTGAAAATGGTTTCCAGAAT	GAACAAAGGTTGCCACACAGTAG
7 PIK3CG	CAGCAGTGGAGAGATTTGTTTATTCCT	TTCTGCACTAAATAAACTCACCTGTCTC
7 PIK3CG	GGGAATATGTGACTGCTTCCTTACA	CGCAGATCATCACCATGTTTAAAGATAA
7 PIK3CG	CAGCCCTATCAAATGAAACAATTGGAA	CAGTAGGAGAAAGCATGTTTTTGATGT
7 PIK3CG	CTGGAACTCACATAACTCTTGTGTACT	ACATGGAGACATCCAAGTGATACCTA
7 PIK3CG	CTTACCATCCACGGCAAGGA	CATGCTGGATGTTTGCCTCTAC
7 PIK3CG	GGAACACCGACCTCACAGTTTTT	TGGGCAAGTCTTTGATTTTGATACTGA
7 PIK3CG	CCTGCGCCGTGGAGAATAC	GGGTGGCAGTAATTGTCCAGA
7 PIK3CG	TGTGGAATGTGTGGCTTGAGT	GAGAAGCTGAACTTTGCCCTTG
7 PIK3CG	GAGAACTCAATGTCCATCTCCATTCT	AGGGTTAAGTGGATCAGTGGCTA
7 PIK3CG	CAAGCAATTGGAGGCGATCA	GTCCCCATTTCACTGAACTAAATAGCTT
7 PIK3CG	AGAGTCCCCCAGTTCTGAGTC	TTGGTCTTCTCCCTTCCCAGATAT
7 PIK3CG	CCTTAAGCACCCAAAAGCATATCCT	GAGCTGCATTGTTAACCCAACA

7 PIK3CG	TCTGGGATCAAAGTGCTTTGGA	CCATTGATAACACCAACATCCCCTA
7 PIK3CG	ACAAGATAGCTCATTTTTAGGCAACTCA	CTCTCTCTTTATTAATGCCCAGGAAACT
7 PIK3CG	GGGCACATTCTTGGGAATTACAAA	GGCGATGAAGGAGGTGACTTAC
7 PIK3CG	AGCGTGAAATTTTAAGTTGCCAAGAATT	CTGGACTGGGCTATCTCACTTC
7 PIK3CG	ATTGGTCACTTTTTGTTTTGGTTCTTGA	ATCTCGATTACTTGGACTTGTTGGG
7 PIK3CG	AGCCATGCTGCACGACTTTA	GAGAACACAGAAAATATAGCCACCGT
7 PIK3CG	TGTGTTACTAGATATGATTGCTATTTTTAAGGG	TCCCAAATAGACTCCATGATTCGTAGAA
7 PIK3CG	CTGTGTTTCGATGCCCAGA	TTTTCAAACGAGCTGAGAGGTCTATT
7 PIK3CG	GGGACCTTGATGGTTTCTTCTCA	AGGGCTAGATAAGCCTTAACACAGA
7 PIK3CG	AGCATTTTCTTCTTCTTTATCCAGGACA	CCCGGATATATTCAATGTCTTCTTTGCT
7 PIK3CG	GACAGGAATGCCCCAGTTAACA	CAATTAAACTGCACAGTCCATCCTTT
7 PIK3CG	TGATCAGATCGAAGTTTGCAGAGAC	TCCAAGTTCGATGATTGCTATGCTAATT
7 PIK3CG	CGCTCCCAGGTCGCATA	CATTTGCGCTGGCTGGTG
7 PIK3CG	AGGAGTCCCAAGCCTTCCA	GGTGCATGGCGTAGAGCTT
7 PIK3CG	CTCATCCCCATCGAGTTCGT	CAGCCGGTGGTAGAAGTCC
7 PIK3CG	GGCAACGTGGAGCAGATGAA	CAGTCCAGAGTCTGCACCAC
7 PIK3CG	CGACGATGAGCTGGAGTTCAC	GAATGACGATGAAGATGCAGTTGTT
7 PIK3CG	GTACGAGATCTACGACAAGTACCAG	TCAGTGACGTCATAGCCAATCAG
7 PIK3CG	AGTACCTGTGGAAGAAGATTGCC	TCGCTTTGGCTTTCGGGAATAT
7 PIK3CG	TGGCCAAGAAGAAATCTCTGATGG	CGTGTCCAGTACCACGTGAA
7 PIK3CG	ACTGCCTCAAGAACGGAGAAG	CACGGTGAACACACTCTCGT
7 PMS2	GGGTCAAGTGAGTGGATAAAAATATTGT	TTCAGCTCTGAAACATCACACATCT
7 PMS2	GAAACCCGCTATAATCACTAGAGCAAT	GCATCCGTGTAAGTTGCACCA
7 PMS2	TCGTTTTCCTTGTCCAAGCTGAT	TGTTGTAACTTGAGCTGTGTAATTCCT
7 PMS2	TCCCGAGCTCCACGTAAAC	TTGGAAGGAGTTCAACAGACAGAC
7 PMS2	CCGCCGGTTGATAAAGAAAAACT	TCTAATCCCTTTCACTCTGGAATCCT
7 PMS2	TCAATTGTAGTTCTCTTGCCAGCAAT	GCAAAGCCTCATTCCTTTTGTTCAG
7 PMS2	CTTCACACACGGAGTCACTAGG	TTTTTGCGCTTGTAATGTCAATAGCTT
7 PMS2	AGTAAATCTTTTGCTCATGTGCATTAACC	GCGTGCAGCAGTTATTTTCCA
7 PMS2	CCTTATGGCGCACAGGTAGTG	CGAAGGTTGGAACTCGACTGA
7 PMS2	GGTTTTCTGGATAATTTTCCCATTGTGA	AGTGCCCAACATCATGGGT
7 PMS2	AGCTTTAGAAGCTGTTTGTACACTGTAT	TGTTGGCAGTTTTAAAGACCTCTTTGA
7 PMS2	GTCACTAGTTGTACTGAAATGCCAATG	GGCTGGGAACATTTGTCATTTATTCTT

7 PMS2	TTTTTGCATTTCCCAAGACAGTGTT	AAGCTTAAGGACTATGGAGTGGATCTTA
7 PMS2	CTTCTTCTACCCCACATCCATTGTC	AAAAACTGATAGCATGGGTCCGt
7 PMS2	GGAATGCCGTGGGTCTCAAA	GAGCCCTGGAGGGAACTTTC
7 PMS2	TTTTCTTAGTTCATCTTCGGCTGCT	CCCCTGGACTTTTCTATGAGTTCTTTA
7 PMS2	GGGAGCTGGCCGCATAC	CCAGTGACCCTACGGACAGA
7 PMS2	GTGCCCCGAGTCCTTCT	GTATGCTGTCTTCTAGCACTTCAGG
7 PMS2	CCTTTGTCAGAGATGGCACCT	AAAAGACGTGTCCATTTCCAGACT
7 PMS2	CTTTGCTGTGCTTCATGATGTAACTG	ACTCAGGACATGTCAGCCTCT
7 PMS2	GCACCTGAAGTGCTAGAAGACA	CCCCTTCATTAAGGACTGGAGAAG
7 PMS2	GGGCACAACTTTCTTATTAATTTTCACA	CCTCAGCCAACTAATCTCGCAA
7 PMS2	CAGTCTGGAAATGGACACGTCTT	TCTCACCATTTCAGGATAGTCCCT
7 PMS2	AGAGGCTGACATGTCCTGAGTA	ACCAGGAAGATACCGGATGTAAATTTC
7 PMS2	AGATTAGTTGGCTGAGGCAAAACT	GTCACTGCAGCAGCGAGTA
7 POT1	GCTGGTAAGTGTAGAGGCAGACT	GCCTTTTCCTTTTTATAGGCTCTGGAT
7 POT1	CTTTACAGACATGTAAGTGGTGCTAACT	CGTCTTGGACATTACTAAAATTGTGTGA
7 POT1	GTCAAAATACTGCATTGGCTGAACA	GAACTTTGGGAGCCCCTATCAT
7 POT1	ACTTGCTTGAAGTGCGAGGT	CGTAGGTTGTGCATCAGTAAGCTATTTT
7 POT1	GATTATCCTTACTTGGAAAGCTGTCGT	ttctggaTTTTGTGGGTAGAGCTAAATT
7 POT1	AGACATTCATTTGAAAGCGGGAGA	CCCCAGATGTCAAGCTACAAAATACAT
7 POT1	GTCCTTTTTGATTTTTAGTGGTCCAGAT	AGTTCCACATGAGGGCGATTT
7 POT1	GGTTTTAGTTGCACCATCCTGAAA	GATAACTTGTTAAATGGAAATCTTCACGCT
7 POT1	AATTAGTGCTAACTAGTTTCAGTGAACA	GCCTGCTCTTTAGTGGAAACTATGAA
7 POT1	CTGTGAAAGCGAACAATATCTCCATTTT	GTGTTTTCCAGATTATTGCTCAGTTGT
7 POT1	AGTTAGTTTTACATTTGTCTGGTCCACA	GGAAGAATAAGGTTTGGTGTTTTGAAGT
7 POT1	TATGCATCAGTGTTGTTTGGCAAT	GCCCCCATATCTAAGCAAAGGA
7 POT1	ACTCCACCAGTTTTAATACCTACCAGT	CTCTTTCTTTAGGTTCCAGCAACAAATT
7 POT1	GACAATTGTACCACCCTTAAGTTGATTC	AGGATAAAATAGTTACATGGATTTGCTGCT
7 POT1	GGAAAATACTCACAGCAAATGACATTTAGG	CACCACTATGTGCCATTTTGAAACAA
7 POT1	CGGATGCGGTATTGTTGAGGA	ATTTGGTTCAGGAGGATGCATGT
7 POT1	TTGTTCCTTGTATAAGAAATGGTGCTGA	ACAGGAGGTACACTCAGTGAAATTTG
7 POT1	GCCAGATCTCACAGGAATTACACTAT	CCTCACACGTTATTTAATAGGACTGTACTT
7 POT1	CAAAGAAGGAACAGGTATACTTTCAGGT	CCCTGGTTGATAAAACATCGTGGA

7 POT1	GAACTATCAGAAGCCCCAGGAA	TCATGGAGGTACCAGTTACGGT
7 POT1	ACATCAGAGTTACTTTCTGGCAAGAC	AGCCTTCATACCAAACTTCAATCAATGA
7 POT1	TGTGTGTGGCATATACAGGTATAGGT	GCATGTAATCACATTGGAGGTTATCTGT
7 POT1	TTTTCCTACTATACATCACCTTCAGAGATCTT	ACCAGGACACCATTTCCATCTTG
7 POT1	CAGATTTTGTAGCCGATGGATGTG	ACTACTCTACTCTCTTATGGCAGGTATG
7 POT1	CAAGATGGAAATGGTGTCCTGGT	AGTGCCAATATTCAGAGGCATAAGTTT
7 POT1	TCTCAAGTAAAAGAAGTGTGGGATTGTT	CTCTCATGAAACATTTTTAAGGACAAATTCTTC
7 POT1	ACTTACAGAATCCATGAGATAGGCTTCT	GGAATAGCAGACTCATATGGTTTGATCT
7 POT1	GGTCAGGAAAAGAAGCTCAAACAG	CGTGGTTGGAATGCTTCATCA
7 POT1	TCTGTTCCATTTGTGACATTGTATGACT	TTTCTAGCTCAGTGGTAAGCTTAGGTA
7 SAMD9	ATGTTGGCCAAGATGAAATTTAGCTTTT	GTTTTCTGGGCTCTTGGAATATCTTATCA
7 SAMD9	CATAGTGCTTATAGCATCCTCTTGACTT	CACAGGTCTTGGATCAAAGTTCAGT
7 SAMD9	CTCCTGCATCTCTCTACTTGAAGTG	TCCTGCTAAAACCCAGGAACAAT
7 SAMD9	GAGTTTTGGCCTCTTCATTTTGCTTAAT	GGGATCCAAACAATGAATATAAATTAGCC
7 SAMD9	ATTCATTGTTTGGATCCCCTGGAATA	CAAGGAGAGATAGAAGTTGGGCTTT
7 SAMD9	GGGTCATCCACAGAGGACAGTAAT	GCAGGTTAGACCTTGACAGTGAAAAATA
7 SAMD9	CCAGGAACTTGGATCAAAGGGTTTA	GCAAACCTTCACTTTCCAAGTGT
7 SAMD9	TGAGCTGGAGAATTTGGATTGTGT	CAGAACATGCCTCAAGTGCAT
7 SAMD9	TCTTCACTTTGCTGTTGAGATTCTTTGA	AGATACACTGGGTCAAGTCTACAAAAGTA
7 SAMD9	TCATTTGGTGTGGTTTTCTGTTCTACATA	CACCCAGATCAAACAAAACACTTAGATT
7 SAMD9	GATCAAACTCCAATACAGCAAACCATTT	AAGAGAGGGACCAAAGTTGGTTAAATT
7 SAMD9	CTGGGTGGCATTTATTTGTTACAAGAAT	GATTTTAAAACACTGGCAGAGTCCAG
7 SAMD9	GCTCTGAATTTTTCTTCTGCTGCTTTT	CTCACTATTTGTGCGAGATGGGA
7 SAMD9	CCGTTTTCCTCTATCCACCATCTTATTT	AGCGTTGGCAAGACATTTCTACATTA
7 SAMD9	GAGCATTGCCAAAGTCCTTCTTTT	GGTTTTCCCCATTTATTGAAGCATTACA
7 SAMD9	CTTCAACTGCTTCATTTCCTTCATCTTT	GTTCTTCGATACTGGTATGGGAAAAAGT
7 SAMD9	GGACTTTTTCCTCCTTCCACACAT	GGGCAATATAAACATATGCATCGTACAA
7 SAMD9	GGTACTGAGATCAAATTCTTGGAGGA	AATACCCATCACTCCCGCTTTTT
7 SAMD9	CCAAGAAAGAAATATGCAATTGGTTGCT	GCCAGAAAATCAACAACTAGATCAACAT
7 SAMD9	CTTGACATCCTCTTCTGTGGCT	CATCCATAGACCTGACATGTGTATCAT
7 SAMD9	CTTGAGCATACTCTTTCATTTGTTCAGA	CCTCCAGATTAGTAAAGCCAGTTGAAAA
7 SAMD9	TCAGAAGAGAAGTAGAAGTTCCACCA	AAATGAATGTGAGGGTACACTGTTAGAG

7 SAMD9	CAAGACTTCTCGAAGCTGATCTTTTAGT	AATGCACTGTCAAAATCCAGTCAAAA
7 SAMD9	CTTCTGCCACTTCTAAGTTGACCTA	ATTCCTTGTGGCAGAGTGGAG
7 SAMD9	GCTGTAGACTAAAATCCAACTTGTAACGA	CGTCTGCAATGAGTACAACTGCTAA
7 SAMD9	AGTCTTCCTCTTTTGATGCCTTGAATT	TCTATTGGTTTATCGACTGTCCTTCTGA
7 SAMD9	CAGAGCAGTCATGATATCTTCTTCCTTT	GAAATTTCAAGCCAATGTATTTCTGCTT
7 SAMD9	GAGCTCAACTTTTAGTGACTTAGAACCT	GCTCCTAAAGACCAAACTGTGTCTC
7 SAMD9	TGCCATTGATCTCTTCAAGGCTTA	CTACCAGGATCTCAAAGGAATGGAAAAT
7 SAMD9	TGGGTGCACACAAATACACAGTAT	AAGAGGGAAGTTTTTGGTGGTATTTCT
7 SAMD9	GCTTTGAAGTTTCTCTACGTTCCTTTT	CACATGGACCAGCTATTCAAATAGAAGA
7 SAMD9	GCTGTTTTCCGCAATTCTTTGAATAGT	GGAAATTTTGACTGAACAAGACGTGA
7 SAMD9	TTTTAACCACTTCAAGACTGCTCCAT	CATATAGGTATCAGAATGGCAAAGCA
7 SAMD9	GCTCAAAAGCTCTCTGTTCTTTGG	CTTCTGCAGTACTCTATTCAAACAGCTA
7 SAMD9	ATCACCAGAGGTTTTTCATATCGAATGT	GAGAACAGGTAACCAGTTTAATCACCTA
7 SAMD9	TTTTCGTAATGTCCTTAGAGCTGGTC	TTCCACAGTTCTCTGAATGCCAAT
7 SAMD9	GTATTCCTGACGGTTCATTGCC	GCACATTCTCTGGGAACTAAGGA
7 SAMD9	GTGAGTAGGAGTGTGTGCATATCTT	ATTCACTCTTTGATTGCAGAGTTCTCA
7 SAMD9	GTCCCATCTCGCACAAATAGTGA	TCTGACAGATTTGTTATTGAAGTGGACAT
7 SAMD9	AGGTGATAGCTTTTCTTCAATTCTTCCA	TGGGCACCTACTCTACAATTCTGATA
7 SAMD9	ATTGGCATTCAGAGAACTGTGGAA	ACAAGTATTTTGAAGACCATCAAGTCCA
7 SAMD9	TCAGCACAGCACATCTGAATTTCT	GCAGATTCTTCTAAACCAACAAGTACCA
7 SAMD9	CCCACATTCGATGACCTCTGTTTT	GATACCACCATTTCACTATCACAGTGT
7 SAMD9	CTCGAATGCACTTCTTTGCTTGT	TTGGAGTCAAAGACAAACCCCAT
7 SAMD9	CAGCCTGGATGATGATACAGATGAATAAT	CTATCGAGGTGGCAAAGTGTCA
7 SAMD9	AAGCCTTCTTGTTTCCAATTCCTAAGA	CGGAATATCCTGAAAGGGCAGAATA
7 SAMD9	GAGCTTTGCTTCCTTGGTGAAAA	CAAAGAACAGAGAGCTTTTGAGCTTAA
7 SAMD9	CAGGATATTCCGGACCACATTTTCTAT	TGCCGTAATACAGCAACTCTCTC
7 SAMD9	GTGACTTTGATGCCAACAATTTTCC	GAATTCAAAGCCTTCACAAATACAGCA
7 SBDS	CTATGCTGCAGCTGTTACCCA	CTTTCTCTCCTGCCCTCACAAG
7 SBDS	CGTCGGAAACGGAAACACTT	CGGGCTGCTTCCGAGAAATT
7 SBDS	TTTCAGATTGAGTACTTCCAAAGAACCTT	GTCTTGCTCATCCTCATGTGGTAAT
7 SBDS	TTTCTTTCAGCTTCTTGCCTTCATTG	AAATTGCATTCTCGATTTTGTCTTGGT
7 SBDS	CATTTCCTCCCCGGCCAA	CTTCGAAATCGCCTGCTACAA
7 SBDS	CAGCCGACGACCTTGTTTT	CGGGTCAGCCCTGGTTC

7 SBDS	ggccCCAGACCCATTATTTAATGA	TCCACTATTCGGTGAAAACCAACAA
7 SBDS	CACTCACCTGCTGTTTTGTACTC	ACTATTGTGGCAGACAAATGTGTGAA
7 SBDS	TGTACTCTTGTTGGTTTTCACCGA	ACACACACAACTGGAGCAGATG
7 SBDS	CCAGTTGTGTGTGTCTTTCTTTATCTGA	AGGATGAAATTTAATTTTCTCTCCATCCAGT
7 SMO	CAGGGAAGGGTCATGATCAGAA	CATTGACGTAGAAGAGAATAACAGCAG
7 SMO	GCGGAACTCGAATCGCTACC	TGGGTCCCCAAGACACCTA
7 SMO	CCAGGTAGAGGGAGTACAGAGT	CAGGGCTTTGAAGGAAGTGTG
7 SMO	TTGGTTTGTGGTCCTCACCTATG	TCCCGTTCCTACCAGTCACTAT
7 SMO	GCTGTGGCGCAGGTATAGTG	CGGTAGTTCTTGTAGCCCACAAA
7 SMO	CTGCGTCTGTGGGTCAGA	CCTGGCAGAGGGTACGG
7 SMO	GTGAGAATGACCGGGTGGA	CCTGGACCTTGTCTCACAGA
7 SMO	GGACTCTGTGAGTGGGATTTGT	CCCAGTATATTTTGTTGCCCAACTG
7 SMO	GGGCTTGGTAACGTCCTGT	CTCCATGCCCCTCACTCA
7 SMO	TCTGAGGTCCCCCTTCTGTT	GGAGGGTGCAGTGTTGGG
7 SMO	GGCCCCAGAACTAACAGGTTA	GGGAGATCTCTGCCTCAACCA
7 SMO	AGGAACAAGCCAACCTGTGG	AGGCAGTCGAGGAATGGTACT
7 SMO	CGCCCCCTGAGCTTCAC	GCTCTGGGCAGAATGGGTT
7 SMO	GCGTGGACCCTGGTCTC	GAGTCTGCATCCATGAGTTCTGT
7 SMO	CCTATTCACTCCCGCACCAAC	TTCTCTGGAAGACGGGATCCT
7 SMO	CTTCTTCACGCTCCTTCCCTAT	CATGCTGAAGGACAGCTCCT
7 SMO	AGATGATTGCCAAGGCCTTCTC	GCTGGGAGAGACTAGCACAT
7 SMO	GCTGCGCTACAACGTGT	GCTCCGCCGCACTTACC
7 SMO	CGGCAAGCTCGTGCTCT	TCCCCGCCCTCTCCAAA
7 SMO	CCTGTCTACGTTCCCTCACTGT	CTTGGTCCAGACCCAGGTG
7 SMO	GGAACTGGCATCGCCATGA	GGACCCGACAAAACCTAAAGATGG
7 SMO	CCTGCCATGCTACCTAGATACC	GCACTGGCCTGAACTGTTGAA
7 SMO	CCAGAATGAGGTGCAGAACATCAA	GATGTAGCTGTGCATGTCCTG
7 SMO	CTCTTCACAGAGGCTGAGCA	CCCTTCCCTCTGGCTCTCTT
7 SMO	CTTCTCCTTTCCTTCCTTCCATTCC	ACTCTCATACCTGGAGTTGCCA
7 SMO	CTGCCCCAGGATATTTCTGTCA	GCCCCACTCTTCAGATCCTCT
7 TRIM24	TCTTCTCTCTCATACTTACAGCTCTGG	TTTTCCGGTGGTCCACTGTTT
7 TRIM24	CTGGAGCCAATTCGAATAAAACAAGA	CATAGGAATAGCATTGCAAGCATTCATA
7 TRIM24	TGTTTACCTGGAAATGACCATCAAAGT	AAATGCATGACATGCTCCAATTGTT
7 TRIM24	AGGAAGTGGCTGGACTCTCTA	GCTCCATTGTTTTACTGTGCATTTTTAC
7 TRIM24	TGAATGTATGGTTTCCCCCTCCT	CATGTTGAACACTGGCAAAAGAAATCTA
7 TRIM24	ACTGCCTTATGTTTTTCCCCGTT	CTATCCCATGGGCAGTCACTTAC
7 TRIM24	GCCCGGCTCAACCTGTT	GCGGGCAGCATGAGGTAG
7 TRIM24	GCCTGCACTCTTTCTGCCA	CGGCCGGTTCTCACCTT
7 TRIM24	TTAAAAGTAGCTTTTAACTTGCAAAATCAGAAT	TGGCTGGCCATGATCTATATTAGTATCT
7 TRIM24	GACTGTTCAAGTACTATTATGCTGGACA	AAGAAGCCCACTTTACCTGCAA

7	TRIM24	GCACAGAGAAAAGCAATGAGAACATT	AGTCTTTTCTTGATGGTTGACAAATCCA
7	TRIM24	CTGTTTTTGTATATTTACTGGGCGCTT	GATCACAGTGAAATTGGATGGTGTTG
7	TRIM24	TCCAATGGATTTGTCAACCATCAAGA	TTTTCACACTGCTGTTCCTTCCT
7	TRIM24	GATGCATCCCCAGTGACCAA	TTTTACAGACAAATGCCACTGAGAAATG
7	TRIM24	TGACTATGCATGAACACTTTTCAAAACAG	CCAGCATTGGCTACTTCTGAATCA
7	TRIM24	CTCCACAGCCTGATTCAGAAGT	GGGCTGTACAAAGTCATCATCTG
7	TRIM24	CAAACCAGAATTCAGGAATGAATCAGAA	ACAAGCTAGTGGTGCTGCAT
7	TRIM24	GTATCATGTACGCATAGCTCGAGA	CCCTCTGATGAGCTCTGATACAC
7	TRIM24	GTGTTGAATGGCTCTGCAAGAC	TCAAAACAAGTCAATACAGGAAGGAAGA
7	TRIM24	TCTTCTCTTTGTCTCCCATTGTTTCTG	TCTCGACATGTCAGTTTGTCACAT
7	TRIM24	AGCTGAAGCTGTACTGTGAGAC	GCAGCAGTCCTTTAGTCCTCTAA
7	TRIM24	TCTCCTGAAGATTCCTTTGAAGCA	GCTATTTAAGAGCAGGGAGGTGAG
7	TRIM24	CTACAGGCCAATTATCCAAGAAGCATA	GGATCCAACCATTCAGACTTTCCAT
7	TRIM24	GACTTCACCAGGACAATTCCTCAA	GGAGTTCCCCTCCGTTTTGAC
7	TRIM24	AGCTTGTTGATAGCCTTTGTTCTTCT	CTTTCCATCATATCCTGCTGCACTA
7	TRIM24	AATGAGGACTGGTGTGCAGTTT	AAGAATGTGGGAAGGGAAGTACTATCT
7	TRIM24	CCTTCCAGCCCCACGATTAC	ccAACAGTTTTCAACAGAGGCTAGTATA
7	TRIM24	TGATCTGTGGCAGAGTTCTATTTTTCTG	AGAACTGGTCCATTGGGTTTGG
7	TRIM24	CCACGTTTGATAAACTTTCAGAATCACA	TGTTGAGCCAAGAAAGCCATCT
7	TRIM24	GCAATAAAGCCAAACCCCCTAC	TCTTGGCTCTTTATAACCTGTTGCTTAA
7	TRIM24	AGTCTGACACTTTGGGACCTCT	AGGCTCATTTCATGGCAGTAAAGAAA
7	TRIM24	CTTAGAAGTGTGAGCGCCTACTTTTA	CAGAAAAGCATTCCACTGTGTAAACAT
7	TRIM24	GGACATTGAAAAAGAACAAAGTCACAGT	CTGCACATTCTTGGCTGCAAA
7	TRIM24	TTTCCCAGTTGGAGTCATTCGTT	GGCAGGCAAAAGGTTCAAGATG
7	TRIM24	ttCTTGGCAATTTTGAAGTTCTGCAA	AATTCTGTTCCACGACAGGATTC
7	TRIM24	AGCCAGCCACAAATGCCTAA	AGCCATTACCTGTTGCTGCATA
7	TRIM24	GCTCAATTACGGCTCCAGCA	GGACAGGCCTGCTTCCAAATTTA
7	TRIM24	GGAAAGGGAAATAGTGAAGAGAGGAA	CTGGGAGCATCACAATCATATTCAAC
7	TRIM24	CTGCCGAGACTTATCTAAACCAGAA	GGTACCACAGTACAATATTGCACTACAA
7	TRRAP	CCTGGGTTGAGTTCCAACTTGA	TCAAAGCTGTACAAGAAAGCAGGAT
7	TRRAP	TTTTGTAGGTTCTGCAGCATATCTTGA	CACCCACACACATAGTGATACCA
7	TRRAP	GCACAAGAATCCTATGAAAAGGCA	GATTCATGCACACGTCAAGCA
7	TRRAP	GTTCATTCCTAGTGCTGGGTGTA	GAAGATGTTCGTTGGTTGGTATTCTATGA

7 TRRAP	TGCGGAAGCTCGTACTTGAAATAAT	AAAGTTTTACATACGGCTATACGACAAGA
7 TRRAP	AGGGCTGTGTTTGTCAGTTGT	CAAACGTGCTCACCAACTTCTT
7 TRRAP	CCCCCACACTCTCAATTTTGTG	CGTGAACTGGCCTTTCAGCTT
7 TRRAP	TGCACAAGACCCTGTCTTTCAG	AAGGATCCAATCAGCTTCAACACTAA
7 TRRAP	TTTTGGAAAAGTAGCATGTCTTGACAC	GCGTGAGCCTTGAGGAGACTA
7 TRRAP	AGTGATCTTTTTCCTGTTTGACACTT	GGCAGATGGCCAGGTATCC
7 TRRAP	GGCCTGGAAGGTGAACATGTA	GCACGCACCTGTAGGAGAG
7 TRRAP	GTGTTGCTTTCACTCTGTTTTTCGT	GCATAAAGACGGAGATCAGCCT
7 TRRAP	CGTCTCTTCCCTGTTTTAGGTTTTTCA	CCCCTCCTCCACAATGATCTT
7 TRRAP	GTGTGTTTTTAAGCCGGATTCAGAT	CCCATACAGAGGCACATGATCTT
7 TRRAP	GTAGTGTCCCACGTGCACA	GACAAGCAGAAGGAGTTTCATCTCT
7 TRRAP	CAGATGCTGACCCACTGGAC	GTAAAGGTTCAAAGTGAATGCCTGAA
7 TRRAP	GCAACAACCCCAGCTACATAGA	TGTTCAACAGAGTCTGAGGAAAGG
7 TRRAP	GTGCTCTATTTTAAGTATGCAGCTGATCT	AGTTGGCCACACGGATGTATG
7 TRRAP	TCCAGATAGCAGGAAATGGACAGA	TCTCCACCATATAAGGGACCGTAG
7 TRRAP	TCCAAACTACGGTCCCTTATATGGT	GCTATAAGCACATCCTTGGCTACATTAT
7 TRRAP	TCGAGACACGGTTCCCTAATTATCT	GCCTCTCTTGGAAGTTCTCACTC
7 TRRAP	TTGCGACGTGTGTGGGT	GGCTGAGCTTTTGGGACAGA
7 TRRAP	GCCTGCCCCGTTTCTCTT	CCGGTCATAGTAACGGGAGATG
7 TRRAP	GGCATCGAGCATGACAACC	TCATCTCCTAAACACCCCTGTCT
7 TRRAP	GCATCGCCCTGCTTTCATTCTA	CACGATCTCTTCGGCTCTCTC
7 TRRAP	GTGCGCAACGCCTTTAAAAGA	CTGCTTCCTCACAGTGGAGTTT
7 TRRAP	GGTTCGAGAAGTCACCTCTCC	TTGAAATTACCTTGGTTCTGGTGACA
7 TRRAP	TTTGCCTTACTGTTTAGGAATGACCTT	GTCATCATCTTCACAAGCAAAATGGAC
7 TRRAP	ACACCTACACTCCGGGAGAA	GGGAGCACATTAAGGACCAATATATG
7 TRRAP	CTGGTTGAACTCATGGACCTGA	AAAGGATCAACTCACGTGTATTCACA
7 TRRAP	GCGATAATCATGTCCCCAATGGA	TGTTCCTTCCCAGGTTGGTTG
7 TRRAP	GAGAAGTCCAGATGCCCTGAAT	GCTATCGGCCAGGTTGATGAC
7 TRRAP	GCACAAATCAACGCAGGCTTA	ccctggTAATGATGCTGCCT
7 TRRAP	GCTCCCGTCCATCACCAAC	GCCTCACCTCCTCTTTGGA
7 TRRAP	TCTTCCTATAACGAAAGTGTCTGCTTTTT	GCAACCCAACTGTTCTAAAGTCTTTT
7 TRRAP	GATGCAGCATTCCCACATGTTT	CCAGCTGCTCACAGCTCTTAC
7 TRRAP	GTGTGTTAGAACTGGCCCAGAT	CCTCCAAGATTTTGATCCACTTTTTCAA
7 TRRAP	AGCGTTCCAGGATCCATGAAG	TCTGTGCCGAGAAATTGCTCA
7 TRRAP	CATAGAGGAAAAGTGCCGGTTCT	CCGTGGGTTAGTTCCACCTG
7 TRRAP	TCTGCAGTTTGAGGGACAAGAC	AAATAGCAATTGCATCAATGAGAACCAA

7	TRRAP	TTGGTTTTGTTAGTGATCTGTGCAATG	CTCCTGGCATTTAACCAACCAGATAT
7	TRRAP	GGCTCGAAAGGAATGGATCCT	AGACACTCCTCAACCCTTCCTTA
7	TRRAP	CTTGAAGCCTTTGACCTTTTGGTT	GAAACTTGTCGTGCCTGTTGG
7	TRRAP	GAAACCAGCTGCACATGCTAA	CCTGATTTAGGCTTTGAACTGTCCAT
7	TRRAP	CCGGCTGTAACTCGGATGAA	CATCAGCAGCTTGTCGAACC
7	TRRAP	CAAGTAGCACAAATTTGCTTTTGGCTAA	AGAATACTGAGGAGATGACGTGACA
7	TRRAP	TTTTGCACAATTAGAATGTCACGTCATC	GCACCCTTGTCTTCTCATGTTATTTTT
7	TRRAP	CCCAAGTCCGAACTCAAGCTG	CATAAGGGTCAGAGTGATCCACAA
7	TRRAP	CTGTCTTCTTCTTTTGACCCTCCA	AAGAACAGGGCACGTTTCTGA
7	TRRAP	AGGAAGAGATTCCCAAAAATTACAGCAT	CAAAGTTCCCTAGAAAACCAGACCAA
7	TRRAP	CATCTTCATGTGGAGGCAGCA	CTGTCATTCCCTACCCCAAAATAGAA
7	TRRAP	GTGTCTTTACTGTCTTGTTAACCCCAA	CATCTCCCGCACTCGGAAAT
7	TRRAP	TCCTCCCCATCCTCAGGAA	gGTAAACTTTTCTTCACATTGCAAGTCA
7	TRRAP	CCTTGTTGTGAAGTGCTTTGAAAGA	TGATGCATGAACCCCAAGCAT
7	TRRAP	CATGATCCCAGTTCAAATAACGCT	AATCTTCTGGAAGCAATCCACGAT
7	TRRAP	CGGATTCATACTATTCCAACTGTTCCT	CCTGGCCTCATTCTTCAAGTGT
7	TRRAP	AGTTCACAACTGGAAATGAGTCCTT	CAGCTTGCTGTTGCGGTTC
7	TRRAP	CCACCACATCCATGACAACAACA	GTGTATGGCGAATTTGGCGATAAT
7	TRRAP	AGTACAGCGGACACTTGCTC	GCCAAGTGCACCATGTGAGAT
7	TRRAP	CAGTATTTGGCCTGTTGAAGAAGAAT	CCCAGGCAGAGCTGCTTC
7	TRRAP	CAGAACTTGGAGCCGTGGAA	TCTCCTTGCTTCTCGAAGGGA
7	TRRAP	GTGGAAGCAGCTCTGCCT	GTCCTTTTCTCCTTGCTTCTCGAA
7	TRRAP	CCGTGCCTCCCTTCGAG	TGTTATGCCCCACGTGATTGT
7	TRRAP	ACCTTGGTGTGTGGTGTCAAG	AACTGAGCTTCTGGAAAAGAGAAAGT
7	TRRAP	CCCATGTTCCGGCGTGAT	CTTGATGACGACTTCAGACAGGT
7	TRRAP	CAGTGTCACCATCGAGCAGAG	ACAGGCGCTTGGTTTTACTGA
7	TRRAP	CTGAAGTCGTCATCAAGTGGGA	CTCCACTGGAATTTGGGTCCAT
7	TRRAP	GTGGGCCCAAGAGCCATAA	GCATTGGTGGCCTTCTCGT
7	TRRAP	CATCTATGAAGGGCTCACCAACT	CTCAAAGCTCTGCCCAACAAC
7	TRRAP	GCCTAAATGACATGTGCTTTGGTT	GTACTTCAGGACGCAGGGT
7	TRRAP	GCTTTGTGGAAGCCATGTCC	GTTGTTTGCTTCGGCTTAATCTGA
7	TRRAP	GGCTTTTGAAAAGGGTCTGAGTCT	CAGGGAAGAGGTAATTAACAAACTGAGG
7	TRRAP	GTGAGTTTGATTAGCTTAAAACGGCTTT	AGCAGGGTGATGAACCTGTTG
7	TRRAP	CAGACCTCTGCGGGATGTG	CTACCTTGATGGCCTGGAACT
7	TRRAP	CCAACCCCAACAGGTTCATCA	GGAACCGTGTCTCGATTAAACCC

7 TRRAP	AAGTTCAGTGGGTAACCAGTCC	GCCAATTTTCTCTGAACCAGACCAT
7 TRRAP	TGGAAGGCATCGTCGATCAG	GCAACTAAAGCTCAAGGGAAGAGA
7 TRRAP	TGATGGAAAGTGTGTGTGCAGAA	GTGTGATCGGTGGCCTGA
7 TRRAP	CAGTTCAGGCCACCGATCA	GAAAACAGTATGTGTTCAGCCAACTTT
7 TRRAP	AAAAGTCTCTTGCTTGAGGGCTT	GGTGGCACACCAATGCAGTA
7 TRRAP	GCAGATGCCGTCGACAAG	TGTTTTGTGCACGTGAACAAGG
7 TRRAP	GTCCATCGTCTTGCCTGTCT	CATGGAGTTGTCAAAAACCTCGAAA
7 TRRAP	GCCACTCATCAGGGCAAAGTT	GATGTGAGCCGAGTCTTCCTAC
7 TRRAP	ATCTTCCTAAAGGAGAACGGAAACTTG	TCATCAGCAAAGGTCGCATATGT
7 TRRAP	ACCATAGAAGTCGATCAAATCCACAC	AATGCATTTGGCTTACCATCATCT
7 TRRAP	TGCAAAGCACCTGGTGGTAAT	GTCTCTTTGGGTTGTAACTGCTTG
7 TRRAP	CCAGAAGCTCAGTTCATTCCCAA	CCCACCACTATGACCTCTAATACTAAGA
7 TRRAP	GCCTAGCCTTGCCAACATAGTT	GCCGGGATCAGATGAAAAAGGTTTATA
7 TRRAP	CAGGAAATGAAGATTTGCTCAGCAAT	CAGCCTTCAGTATGGCCCTTA
7 TRRAP	TTATGTAGAACCGCTACTTTGAGAACC	GGTAGAAATAACTCAGCGAAACACTTAC
7 TRRAP	TGAGTGGTGCCTCCTCCTT	TGCTCCACCACGTTAAGGTC
7 TRRAP	CAGCCCAGGCTCTTCACAAT	CTGACCAGTCTTCGCGTACA
7 TRRAP	GGGAAGGGATTAATTTTTCACATGTGT	GCTTGAGGTACAGGTTGGAGAG
7 TRRAP	CAGAAATGGGCTCCAACGTG	aaaaaCCCTGAACAGAAGCACTACT
7 TRRAP	AATGACTCATAGGACTTACTGCCTAGA	CTTCAGTGCATAAAATTCGGCTGT
7 TRRAP	TCACAAAAGAGATGACAGCCGAATT	AGCTTCACATTGGCTTCAGGTTATAAATA
7 TRRAP	GCCATCTCAGCCTGTTGTCTTT	TGTCTGTCAGAGAGGATCTTCCAC
7 TRRAP	GGTCCAGCTTTTCCCCAGATT	ACTTGTTCGAGACTGTGTTCTTTTATGA
7 TRRAP	CGTGTTGTGGTTTGTTCATTTTCCT	GTTCTGGAGAACCCAAGTGAGA
7 TRRAP	GTGTGGTGTCTATTAAGTTTCTCATGGA	TTCAGGAAACCACCATTTCAAAAACAG
7 TRRAP	CCACCAGTGGAATGCACCTA	CCTTCCCAGAAACAAACAAGTCACT
7 TRRAP	GCTGTCGGATTACAGTGACACTT	CCCGTAGCAGCAAGAAGTAGTTG
7 TRRAP	CAGACTGCCAAGGAACCCTA	ACAAGACTAAAAGGCAACCAAAGTCA
7 TRRAP	CTGATGCTTGTTTTGCCGTTCA	GCTAGTAAAAAGACTGACAAAGCAGGA
7 TRRAP	GAGTGGCCTGTGTTGTCCAT	GTCATGCAGGCGGCAATTC
7 TRRAP	AATGAGCACTAGTCGAGGTCTCT	GACATGGTCTGGATGCTGCT
7 TRRAP	CATCCTCAGTAGCTTCAAACCTCT	CCACCGTCCACTTTGAAAGAAAA
7 TRRAP	AGAACATCGACGATGAGTCCCT	TGCAGGATGGATGATACTCTCAGA
7 TRRAP	CTTCTTCTCAAGACAAGGACTGGTA	CGTCGTTCATGACGAGGTATGG

7 TRRAP	GGGACACAATGGCAAGATCTAC	CCTCTTGGTGGTCTCCTTTCT
7 TRRAP	GCTGAACCCCTGTTTGGAGAAG	ACCCTCAAAGGCTAACGTGAG
7 TRRAP	AGTTTTGATCCGAACCTCGTGAA	GACTCCTTCAGCATCTTCCTGT
7 TRRAP	CGGTAAGTTTGGCGGCAGTA	GAAACGACATTGTCACAGAGCTT
7 TRRAP	TGTGGAAGATTGCTGTGTTCTGTTAT	CGTAAAGGTACGAGTGACGGAA
7 TRRAP	CAAAGCTGCCCTGTTATAAAAGCC	TTTTCTATAGACCTCCTGTGGTCAGT
7 TRRAP	TCTTTATCCAGAGCTCAGCAGAGA	GTAGCGTTCCCGCTGTTCTAT
7 TRRAP	ACCCTGTACCTGACCCTGAAA	GATTGGCCACGGCTATGAGT
7 TRRAP	AATGTGATGATTCTTCGGTATGCCA	GTGTTCATGATCAAGGGCACAA
7 TRRAP	CATCCACAATGTTGTTGCTGAGT	TTTTACTTGGAATCCAGGCACTGT
7 TRRAP	AGTGAGTGAGATGGGAGTGTCA	GGATGGTGAACATCTTCCGGAAC
7 TRRAP	AATGCCACGGACTACTGGAC	ATCTGGCTGTTCAAAGCAGGTTA
7 TRRAP	aCCATACACGTTTTGGTTTTCTGTCT	TGCCGCTCCTTCACAAAGAT
7 TRRAP	GGGCGACTACCTGGAGAAC	GCCTCTGGTCAAAGCTCGTTT
7 TRRAP	GGGCTATTTTAAATGCCACAATGGT	CCCAAGAGACTAATGAGCAATACACA<br>TA
7 TRRAP	GCTGAAGTTCCTAAAGTGTCTCCT	CGATGGTGGTCAGAAACTCAGAAA
7 TRRAP	TTTCGACTCACGCCCAACA	GATAGGACGACGTGGAGACC
7 TRRAP	GGAATTGTCTTGATTTCTCTCCCTGAA	cacCTTTCTCATACAGGCTTCTCC
7 TRRAP	TCCACCAATAGTGAGCTCCAAGA	GCATAAAGCCTTTTAGGCATACACTG<br>T
7 TRRAP	ACTGTAGTTTCTTTGTGTCTTGGTTGT	GTTGTCCTCCAGGCTCATCAT
7 TRRAP	CGTGGGAAGTGATCAAATGCTTC	TGCAGCCCCAATTCCTAAAACA
7 TRRAP	GGGCAGTTCTTTTGTGTGTTTCA	GGTCCTTCATGTGCTGCTTGT
7 TRRAP	CCCTAGGGCTGAACATGCTT	CCTTGGCTGACCAATGTCTGAG
7 TRRAP	CTTGGTGTCTGCACTCAATGG	CAGCCAGCTAACAACCTCACT
7 TRRAP	GAGACCCGTGGGTTTGTTCT	GAGGATTTTGGCATCTGGTGATTT
7 TRRAP	CATCCTGACATCCCTCATCGAA	GGGAGATCACATGGCACACC
7 TRRAP	TGTTTGGGTCTGTGTGGAAATCA	GCAGCAATGAAGTCAGCATACAAC
7 TRRAP	AGGCAACATAAGCTTTACAACAAGGA	acctgaCATAAAAGTCAATGACCTTACC
7 TRRAP	GTGTGCTGGTGATTGAGCTTTT	TCCTTCATGGCAGTCCAGTTG
7 TRRAP	CACATCAACCCCTACCTCGT	CCAAAATGCTTGTTCTAAGGGAAATC<br>T
7 TRRAP	TTTCTTAACACTCTCCTATCCCTGTAA<br>CT	AGTCTCTGCTGGACAATTTGAAAGTA
7 TRRAP	GATGGTGAAAGGAATGCTCCAGT	GACAAGACAGGCTGGACGTA
7 TRRAP	GCGATTCTCTTCCTGCCTGAA	AGGGCACTTGGATCCCGTA
7 TRRAP	CCAAATGATTTCAAATGACAGCACAG<br>T	CCACATATCTTCCTCTTGTAACAGGG<br>A
7 TRRAP	GATTCCCTTGCGGAGCTTTAC	GACCTCTCATTACACACCCAAGAC
7 TRRAP	ATTTGAAAAGAAGTCCTAGTGGAGTC<br>ATC	AGGGCCTGCTCAAAAGTCTTC

7 TRRAP	CATCGCTACAAAGCCCAGGA	CGACTGCCACCATCGTATAGT
7 TRRAP	CTTTGTCGCCAGCTTGATCC	GAAAATCAATCAGCCAAACTCAGCTT
7 TRRAP	CCTGTGGGCTGTTCTTGGTTAA	GCTGGCTGTCCATGTTCTCTG
7 TRRAP	GGACACGTCCTCTCCTCTCTC	AGGGTGTTCACCTTGCTTTCC
7 TRRAP	CACAACCTCGCCCAGTTC	CGGAGCGCCCTTCACATT
7 XRCC2	GCGTAGTACCCTGCAAAAGACT	AAGCACAGGATGTTTTTCTCCAAAC
7 XRCC2	TGGTTGCTGCTTTGAGAATCATCTT	AGAAAGCCTCGAGCTCATCAG
7 XRCC2	CGAGAGGCATGAGAAGGTTCTT	GTCAATGGAGGAGAAAGTGTGAACT
7 XRCC2	CTTGTTCCCATCTCCCTCACTC	GTAGAGTCTGCGCAGTTGGT
7 XRCC2	TGAGGAGTATGTGTATACATGTGAGCTT	AGCTCCTTGCCCGACTTG
7 XRCC2	AACATTTCCTCAGAGTAGACTCCTGTA	ACCCACTTACTTCTTACACTTTACTCACT
7 XRCC2	GAGATGGGTGACTACAAAACATACTTTCT	GATATGCTCCGGCTAGTTACAATTCT
7 XRCC2	GCTTTGGGATAGTCTGTGCTCA	GCTTTATCACCTAACAGCACGATGTAT
7 XRCC2	GCCACCTTCTGATTTGGGAAGT	GAGCTACTGCATTTTGACTGAGATTTTT
8 CSMD3	AATGGGCCATACCAAATTATTCTCCT	CCAGAGAGAATAGAAAGCAGCTCAAATA
8 CSMD3	GGCCATGAAAATCAAAACCACAACT	TCTTAGCAATTGGTTTGGATTCCTGT
8 CSMD3	GATCCATCACTCCGAAAAGCCA	CCTCTATATCTATGATGGACCAGACAGT
8 CSMD3	GGGCTATTACTGTCTGGTCCATCATA	TGGTTAACAGCCTAATAATAATTATCCTACTTC
8 CSMD3	GAGTTTGTGGTTCAGGACAGGAA	GTTTGATGAGACACTGATATTCAATTTTAGCA
8 CSMD3	CAATAACAATAGTCCTGACTTACCATACAGT	CCATTTATTCTCCTGGGTATCCTGATGA
8 CSMD3	GGTACTCTTACAAGCCAAAAACAATCTT	TGCCTTTTTCTCCACAGTCAGTT
8 CSMD3	AAAAGTAGGGCAAACAGAATGAGGAA	TGCCCCAGTTTCTATTTAGTAGCAG
8 CSMD3	GGAACGACTGTTGTCTGTTGTAAATAGA	TCAGACTGAACTGAATTATGATGTTCTGG
8 CSMD3	AGAAGATTTGGCCCATCATGAACTT	GCAGTCCAAAGCATTATTTCCATATATAGTGT
8 CSMD3	AAACAAGGGAACCAATATACATGATCACT	ATTTCAGGCTGACCACTCAATGT
8 CSMD3	AGTGATGTTAAAGCCACGTCCTG	TCCCAGTTTGATTTCCTTGCTGTTAA
8 CSMD3	TGCTTCAACAACATCAATGCAAGG	CTGATTGCACTTGGACCATTGTAG
8 CSMD3	GGGATTCTGGAGAGTCACCATCT	TCCTGATTACCCAGAAGGGTATGG
8 CSMD3	GAAATTGTGTCCCCAGGCTCT	AAGATGCTTGTGGAGGAACAATGA
8 CSMD3	TGGATATGATGCCACTGGATCCT	GCACTTGGTATCACATTTGAACTGTT
8 CSMD3	ATCAGAGATTATCGTCCAGATGCAATTT	TGTATTTCATAATCTTCCTGTTTCACCAGTT
8 CSMD3	TGTAGTGAAAAATAAAGCATGTCTGGATGA	GCACTGTTCTATCACCAAACTATCCAA
8 CSMD3	GAGTCGAATCTTACTTACCAAACTCCTT	TTTCCTAGCGCCCTGTGG

8 CSMD3	GTACTGATCTGTTTTTGCTCATGCAA	GGACTGTTGATGTAACCCATGGA
8 CSMD3	GAGGCAAATTACCTTTTCCATGGG	TGCTGATCACATGGATCTGTTTCTTT
8 CSMD3	CTAATTAGGCATTGAAGGAAGCTTAACA	GGGAATGGTACCTGGAGTGGT
8 CSMD3	ACGTAAGCACTGCGGTACTTC	GGAAGGCTGGAAGGAACAAATTTC
8 CSMD3	GATGTAGCTAATACTAAAGCCCCAGTC	GCTCAGGATATTTCAACAGCTGTGT
8 CSMD3	GCAAATAGTTCTGCCAGCAGTATG	CTGCACAGAAAAGGAATAAACACTTTGT
8 CSMD3	GGGTAGATTTATTGTCCATGTGCAATC	GGATTTCTGTTATTTTGCAGCTCAGT
8 CSMD3	GAAGTCTGACATAGCACCACCA	ACTCAGAGCCTTGAAAACGTGAA
8 CSMD3	GCATATGAACCAAAGACGCATTCT	CCTCTTGTGAAAACCCAGGTGT
8 CSMD3	AAATCCGAATGTATTGTTCTGAGATCCA	GATCCTTTGTACCAAACCCCAGAT
8 CSMD3	AAACTTGTTGCTGTTGTCTTTCCC	GTTTCTTTATCTTGCATAAGTCCATGCT
8 CSMD3	GAGCAAAGGAAAGCAATAGTGCAA	CCTGGCTTCTTCTTAGCAGGTG
8 CSMD3	TCGGATCTACACACTCTATGTTCTGTT	CTCTTTTCTTAATATAGCCCACAGCTGTA
8 CSMD3	GATGTCTGGGATAATAACAGAAGTTCGT	GCCTGATGGAGCTACTTATGTATTTCAA
8 CSMD3	CCAAATTGTCCATAATCTTTGCCTTGAA	CTGGGAAAATATAGCTATGGCCAGT
8 CSMD3	TCGATATAAAGTGTTAGTTCTATGAGGACAGA	GGACTATCCTCTGCCTTCCTGTAT
8 CSMD3	CCTACCAATACAGGAAGGCAGAG	CCACTTTGCTGGTAGCACCAT
8 CSMD3	GTGTAGCCTGGATTGCATCCATAA	TTTGTCCCAGGTTTTGAACTCTCA
8 CSMD3	GGAATGCCAGGATCTTCACAGT	ATGGTAAGGGCAATATGACTAAGAATAAG
8 CSMD3	GGAACTGATTCAAACAATTCGATAACCAA	GCCTCCATCGAATGTCTTCCTAATG
8 CSMD3	GGTCTTTCATTTCTCCAACTCCAAGTA	GGAACATGGAAGATGGCGAATTG
8 CSMD3	ATGATAAACATTCCTATGGTTTCCATGACA	TGGTATTCTATGACTGCAATCCTGGATA
8 CSMD3	GTTTTGTATTCATAATGGGAGCCATTCA	GGTCAGTGGAGATGTAAAGAATGGAAT
8 CSMD3	ACATTCTGGTAAAGGTTTGTCCCATT	AACATGGAAATTTTACTTACGGCACTG
8 CSMD3	CCAGGATTGCAGTCATAGAATACCA	TTTGTCATTTGTTTAGTGGTCAACTGTTC
8 CSMD3	GGAATTCCAGGATCAGAACAGTTG	ATTGGAATCTGGTTGTTTGAATAAATTTACTT
8 CSMD3	TTCAAATACACTGGCTGATAACAACTCT	GATCCGCTACAGCTGTGTAACT
8 CSMD3	GGGTGGCCATCAAGGATGTAT	AATTGCAGAGTAGCTCTTGTGGAA
8 CSMD3	CTTTGGGTGGAACACCAGGAT	CCTTTCAGTGACTCTTCTCCAAAGAAA
8 CSMD3	ACCCTTGGTTACCATCATACACCT	GATATCCTGACAGTTACCCAAATCTTCA
8 CSMD3	CTGAAATGCTCCATGCACACATT	TGAGGCTCTAAAAGACTGGCATTATTT

8	CSMD3	TGACAGTCCTGGTAGGATAATGTGT	GGATGGAAAAGTAATGTGGAGTGGA
8	CSMD3	ACACTTACCTCCACATTTTGGAATCAG	CAATCAATGCACGGCGGTTT
8	CSMD3	TGAGCACAATCTTTCCTTAAAGATTATATTACA	TTGCTGCTTGGAGTGATCACA
8	CSMD3	TGTAATTGTTTCTGTTCCCTGGGTT	GAATGCCCTGATCCTGGAATACC
8	CSMD3	ACTCCAAGCAGCAAAAACTTCAG	CCAGCAGAAGTGAAGCCTAGTTATTATG
8	CSMD3	CCTAATTGAAAGTTGTCCCCAAACC	AGAAGGATCTCTCTCTGGCTAATGTT
8	CSMD3	TGAACAATATACAAGTATGTCTGAGTTCACC	TCCACTCAACTATGAAAACAACCATGAA
8	CSMD3	TCTGGCTGAAATATTGATTCCCTTTCC	GCATCTGCAACGAATAATGAAGGAATTT
8	CSMD3	TCATGGTTGTTTTCATAGTTGAGTGGAT	TGCATAACACTTCCATACATGATATTGCT
8	CSMD3	GGGTTGGTTAGGCACTTCAG	CCTGGCTATCCTTTTCCATATGACAA
8	CSMD3	TCCACATGCAACGCAGGTTA	TGAAAAGATTGGCATTAAATTGTGGCTT
8	CSMD3	CATACCTCTACAAGTTGGCATTACTCC	TGGAGATGATTATGTGGTTGGACAAAA
8	CSMD3	TGGCTGGCACATGTAAGAAACA	CCATCTGCTCTTCTAATCCAATACTGAC
8	CSMD3	GCATTCAAAGCACAGCAATTGAC	GGTGATTGAAGCTGAACCTGGA
8	CSMD3	TGATAGAGTGTCCAGGTTCAGCTT	ATTTAATTTTTCAGCCCCATGTGGTG
8	CSMD3	GGCCATCCTGGTGAGAGAATC	AACTATTTAATAAGGATATGCAGAGACTTGTT
8	CSMD3	GTGCACTGTTTTGTGTGTCGAT	CTTTTGAAAATCCCATGTATGACACCA
8	CSMD3	CCCTTCCACTGACTTTGCGT	CTGTAGTCTGCTGTTTCTGGAAGAA
8	CSMD3	AGTACCCGATAGGCTAAGGCAA	AGGTGCATGTTTCTGTTTACTGCTA
8	CSMD3	CTGTTTTATTTCATAGTTGCAGGCAGAA	CTAATGGCAGTTGGACCGGAA
8	CSMD3	TGCTTGAGATTACCTTTGCACTCT	GCTCTAGACAGGAAAGCAATTTCAGAA
8	CSMD3	AGGATGTAACCAGTATCACAAGCATAAC	GAATTGGTTAACTTGAACTGTACTGCAT
8	CSMD3	TGTCAGTCAAAACCACTTGCTAAACT	CCTTATGACAACAATCTGAATTGTGTGT
8	CSMD3	TGCATTGCTAGTACGACACTTGT	AGATGATGGAAGACCTGGATGGA
8	CSMD3	CCTCTGGCACTGTGATCTTCC	ACTCCCTCTCTTGGACTGCTTTAT
8	CSMD3	ATGACAACTTGGCAAGGCTCTAT	GGCACCAGACTTGGAATGGATTATAAATT
8	CSMD3	TGAAGAACATAACCAGCATCACAGTAAT	GTTTTACAGCAAAACTGCGAGAGT
8	CSMD3	TGCATGTCAAATGAGTTATTACCATCACA	TCCATTGGCTCTACTGTTTCATTTAGTT
8	CSMD3	CATGACTCAACCTGTATCCTGAATCA	CCACCAAGCTTACTCTTTATTTTCTTCC
8	CSMD3	GGCTGGGCTGCCAGAAG	TCTGTTGCCACTGCGTGT
8	CSMD3	CCCCATTTCGAGTCCCATTCA	ACACAGGGATATATGAAATTGATTTTGGTAGTT
8	CSMD3	AGTAACTGATGCATGGCAGTTCT	GCTTCTCCGGGTTCCTGTTG

8	CSMD3	TGTGTTTCTAGACTACAGGGTCACT	TGTTTTGTTGAGTAGGAGAATCACTTCC
8	CSMD3	CCTGAACTCAGTGGAAGTGATTCT	GAACATAGATTGACATTTTCAAATGACAGTGT
8	CSMD3	ACAGAATGAAACTTTAAAATCTGATGAAGCTTA	ACATGTATGCCAGGACCTGTAAGA
8	CSMD3	CATCTTCTTACAGGTCCTGGCATA	AAAATAAAGCCATGTACTTAAGCTTGTTTTT
8	CSMD3	TTCATCTTGCCTAACAAAAACTAGAATTATTCC	TCTTCAGTGAGGATATGTCAACAGGAT
8	CSMD3	GAGCTGACCAGACCAATTGTG	TCAGCGGGTCATTGTGGAATT
8	CSMD3	CTTGACCATTCACAATCAGTTCTGG	CTTTAAAGGTTTGCTCATGTTCCACAT
8	CSMD3	CCAACACATATTGTCCAATTTTCCAAGT	AGTCCTGTCTGGCGAGAAGTATA
8	CSMD3	CAGGAATAGTGAACAGTAGACCCAAAAG	TGCTGGTATAGGTTGTTCGATAGGA
8	CSMD3	GCTTTGGGCTGGGTGTTC	AGAATAAAATGGACAGTGCATCACACt
8	CSMD3	TGTTAGATTACATTAAGGGCAGTGGTTT	GGTTAACAGGATTCCATCTGCCA
8	CSMD3	AATGTATAGCATTGTTTCCAGTTAAACATTT	GAACGACTGCAGATGGATGGA
8	CSMD3	ACCATTGAATACCTTGACAAACTGGA	GTTGCTTATATGAAACCTCCCCTCTT
8	CSMD3	ATTATTTAATATACATGCAGCACCCCAGT	CGGACTGACACTTAGTAGTACTTCAAAT
8	CSMD3	AGCCTTCATCTGTCCCTTCAGTAT	AGATAAAACGACTCATCTACTAGGTGCT
8	CSMD3	AGTAAAAGCACCTAGTAGATGAGTCGT	GCCCTTTGGAAATGTACTGATCTGATT
8	CSMD3	TCTCCTTTCCTGGACAAGCTACA	GCCAGGAAGCTCGCCTA
8	CSMD3	TGCGTAACATTAGGCGAGCTT	CCTTGACTTGCCCTTGTTTTATTTCTT
8	CSMD3	aCTTGTTCTTAACCTTTTTGAATGGCAC	AGGAACAACAATACCCCATCTTTTGAAT
8	CSMD3	GCAGCAGAAACACTGATGTCTGA	ACTTATTATGTGGTCATTGTCTTGCCTT
8	CSMD3	GGCTGTAACTAAATGGGCACAAGT	CAGCAGATACACAAAGTACCAGGAG
8	CSMD3	CTATCTGTTCAGCATGTCTTGGTCTT	CCAGTGTTACAGCTGTCACCA
8	CSMD3	CTGTTCCTCGGAAAGTCTATGGA	TCTAGTGCTACTGTGTCCCACA
8	CSMD3	AAGATGATAGTGAGATCTGTCCTGAAGT	TCCAGTGCTTATTTCCCTCAGTG
8	CSMD3	GATGAATAATCCCCACTGAGGGAAA	GATTGGTGGTGAAGCAATTTAACCATAT
8	CSMD3	AAGTCATGAAGGTATATTTCGCTGCT	CCCATGACCTTAACAGTTACTAGTTTCA
8	CSMD3	GCGTTAACTCTCCCAGTGGAA	CACAGTTCCTGATGATGTATTTGCC
8	CSMD3	AATGTAGCATGTGTTCTGCAAAACT	TCAAACGGACGAAAGTGTTGGAT
8	CSMD3	CCTTTGTAGTTAACCTTGAAACCAACAG	CTCTCCCTTCTTTGTCCATCTCTTTTTA
8	CSMD3	TCATGCCACCAAATGGGAATATACTTAG	TGGTAACATTCTCATGCAATATTGGGTA
8	CSMD3	CTTTGTTGGCCCTTGCATAAGA	TTTCTGTGTTTCCTTCCCTGTCC
8	CSMD3	AGTTTACCACTCTCTCTTTCCAGA	GGTTCCTTTGCTGGAAACCTGTA
8	CSMD3	GAGGCTGTCTGTCGGTCAAG	CATCTTCCAGATCATCCGGGAAAG

8 CSMD3	TGCTTCCGAATATTTCCATTCTCAGAAA	GTGCTGTGTGCAGAAAGTCTTC
8 CSMD3	GCATCCCATGCATGATACCCATAG	CCAGAATCCCCACCTCATGGATATA
8 CSMD3	GCCCACCTGTCTGACTGATAA	AGCACTAGTCGCTGTATAGGTGT
8 CSMD3	ACTACCAAGTAAGAGTTGTAGATAGCTCTTT	GCTTTGCTACAGAACATAATTGGGATTC
8 CSMD3	TCTCCCCCATCATAAAAGTCCAGA	GGGATTGGTGGACTATAGTTATGTGTAT
8 CSMD3	CCTTGGTATACAATATGAAACCCTTGTTTG	ACTGTTATTGGCCGGCTTAGTG
8 CSMD3	GGTGCTGAATAAGGAAGATGGTATTTGA	CTTCAGGTGTACATCTCCAGTTTGTAAA
8 CSMD3	GAGGATCCACTTCGTACTTCCAAATAA	AGTACATTGATGTGATTGTGTGTGTGT
8 CSMD3	GAGATGCCTCTTTTCAAGGGTGA	GGGCTCTGCTGTAAGGGAATG
8 CSMD3	CACTCCAAAGACCTGAGGAAAGG	ACACCTCCAAATGGAAATAAGATTGGAA
8 CSMD3	GCTGTTGAGCCATATGAAGTTTGAG	CAGTTTTGTTACAAGTGCAGCTTGA
8 CSMD3	TCTTCTCTCCAGCTATTTTTCACTGTTTT	GGGCTCAATTGCGTTTCATTTCA
8 CSMD3	TGAAACGCAATTGAGCCCTGA	TGGAAGACCATCATGACTACTTACTGA
8 CSMD3	GGGTAAAACTGCCATTCTCTGTGA	ACTCTCTGTGTTTTACTGATCTACCTGTA
8 CSMD3	CCCAGGAAATATTTTCTCTCCAGTCTT	CACATGGGTAATAATAGCAGAAGAACGA
8 CSMD3	GTTTGTAGGATGAGGATGTCCATCAT	CACTATAGAAAGCCCTGGTTTTCCA
8 CSMD3	CCATGTGCAGTTTGCACCATTT	GTCTGTTTATTGTCTTTTGTTGCAGGAT
8 CSMD3	GGCTTTCTATAGTGCCATTAAGTCCTT	TTGTTAACTTTGGTCTTGGAAATGCAATAG
8 CSMD3	ATCACAGATCATACAAATTTATATACTCACCAA	GCAGTCGGTTCATCGGTTCT
8 CSMD3	ACTGTTTCACACCTAATTGCTATGGAT	ACAGCTGTTCCTAGAACAAGTTCTAC
8 CSMD3	TCAGGCACAGAACTGCATTGT	ATTCAAGTTCTGATAAAAAGTGATGATTTCTCA
8 CSMD3	ATCAAAAGAGGCAAAGGTATTCCATAAGT	CCACCACCAATTATCAGCAACAAAA
8 CSMD3	ACAAAATGCAGTCTGAGCCAGT	CAATTTTGTGAAAACGTTTACTGCTGAA
8 CSMD3	AGTGATGAAATTCCCTTGACACACA	GAACGCGAGTTACCTATTTTTGTAATGA
8 CSMD3	CTGTAGTGAGTTCTTTGGATGACAATC	ACGATTTTGCTTGTGTGTATGTTTTTCT
8 CSMD3	ACAGTTACTGTGCATAATCAGCCTTTA	GGCAAAAGCTTTATATACCAGTCAGAGG
8 CSMD3	TGAAAGGAAAATTGCAGCTGAATGAAA	CCAAACTGTGTACTCTGCTGTATACTAAA
8 CSMD3	CTATCTGGAAGCAAAGCATTTGGT	GCGATGGATTTTCTAATCGTGATGTTTT
8 CSMD3	CCCAAACTGGCATTCAAACCTT	GGTATCATATGCCTGCAACCCTT
8 CSMD3	CAGTTTGATGCCACTAAGACTTCATC	GCTTCCAATTTATGCCCAGATCCA
8 CSMD3	CTGGGCATAAATTGGAAGCTGTTTT	AATGCTTAAATGACAGTGATTATAGTGACA
8 CSMD3	GCATGCTATTGGAAGAGGAAGACA	GGAGTCAGTCGTAATTGGAATCATCC

8 CSMD3	GCATACCTTCACACCTTGGAAGT	CCACGCCCGTTTCGAAATG
8 CSMD3	GTTTGACCCACAGTAAAATCATTACCAA	AGTTGCAAAATTGCTGTAGTACCCT
8 CSMD3	CAGTAGCAAATGAAATAGGAACTCTCAGA	TCACCTCAGATCGGTCAGTTCA
8 CSMD3	CTGTAGACTGATTCCAAAGCGGTATT	CGGTTCAAAACTACTTCCACAATCAATC
8 CSMD3	ACATCACCTTCCTATTTTGCATTGAAGA	CTGAAGGAATTCATAGCACCCTCAATAT
8 CSMD3	CCGTGTCAAACTGGATGGTTACT	CATCACATGATATACTCCGAGTCTGG
8 CSMD3	GGTGCTATGAATTCCTTCAGGAATAAGA	TTCTCCCACAGCTTGCAGTT
8 CSMD3	GTACAAATGCTAAAGCAAAATGGCCTAA	TGTGACTGGACTATCACCGTCA
8 CSMD3	CAGATTATAAAGAACGAAGGGTGTAACCA	GGGTTATCGACTGGAAGGAACATC
8 CSMD3	GCCAAGGAGATAACATAGTCTGCAT	TGTGGAGGCAATTTAACAGGATCTTC
8 CSMD3	GGAAGTTTGGTGAAAGAATAAAGCCT	GTCTGTACAAAGCCTCATAAGCTCTT
8 CSMD3	CACCACCAAGACAGATGATCTCT	ACCTTGTGAAGATCCTGGCATTC
8 CSMD3	GAACCCGATTCGACTACCATATTGA	AACCACTATCATTTTCTTTGTCCTTTCCT
8 CSMD3	GCCAGCCTCTCTCCATCTACA	CCGCCTAGACTTCATCCTGAT
8 CSMD3	ATGCATATAAATTCTTACCCATGATAGCATT	TGCTATAGGTAGGAAGTGTTGTACAGTT
8 CSMD3	CCCTTGGAGAAGGTGTCCTTTTT	TCTTCCAACTTTACCATACGGAATTTCA
8 CSMD3	GAGGTTCCAAAACGTAAATCCACTTTTA	CAGGAATTAGGAGCTCGCGAA
8 CSMD3	GAAAGGCAGTACAAACAGTTAAAACCT	CAGTGCACAGCCAATGGAAC
8 CSMD3	TGTACAGTTAGGTAAAGTTCCAGACCAT	CGAATTGATGGCACAACATTTTCTAGTT
8 CSMD3	TCCAGAAAGGATGTATCCCTCCAT	AACACTTTGTTTTATAGCTGTGCAGTG
8 CSMD3	GGGAATATTGCGCCAAAATCCTACT	AGCAATGCACAATGTGTCTGG
8 CSMD3	CCTTATTTGTATTCACTGCTGTGATGAC	TCTTTTAGTGAAAACGTGTGGCTCTA
8 CSMD3	TTGAGCTAGAGCAGTAGAAATAACATGAAAA	GTAGTTCTGTAGCCATTGCTATTCTTGT
8 EXT1	CAGTGAGTGAAGCAAGGGAAGA	GGACATTCTCATGAACTTCCTGGTG
8 EXT1	GAGGCAATTTTGTCACAGCAGA	TGATAATGGCCCCCTGTGAAAC
8 EXT1	CCAAGAGCCAAGTGGTCTCA	TCCTATGTGTTCACCTTGTGTTACAG
8 EXT1	AAGGCCTTTAGTTCTGTATGACATCT	ACATCCTGGAGGATTGTTCGTACT
8 EXT1	TCTCCCAGATAAGATGAATACTGTGGT	TCATCCTCACTGTGTGCTATGTTTTATT
8 EXT1	GCTGATGTGTTGAAGGCCACA	TCACATTCACGAAGTCCCCTTTT
8 EXT1	CAATGCTGTTAACAAGATTTGGCCTTA	ACATCAAAGTGGACGAACGACT
8 EXT1	CTCCTGTCAACACCATGGAGT	TGCTTGTTTGGCTTGTGTTCTG
8 EXT1	CTCCTCAGGCATGGGTTCTTA	CTGCCTACTTCTACTTCCTCCCA
8 EXT1	CTCTGTCAACTTCCCGCTCA	GCTTCATTAGGCTCTACTCTCCTTTTTA
8 EXT1	AAGGCTGACTCCCAAAGACAC	GATCAGACACCAGGAATGCCTTA

8 EXT1	CACTTTGGATCTCAAATTGTGCACAT	AGAAAATCGCCGAAAGTTACCAAAAC
8 EXT1	CCTCCCCGTTATGGACGTGATA	CTAAGGATCATCCCAGGACAGGA
8 EXT1	CCTCGATGGCCGCTAGAAT	CAGCATCTACAAAGGCAAGAAGTG
8 EXT1	TGAACTTCAAAAACCCCCTCTCC	GGCACTTGGCCTGACTACAC
8 EXT1	ATGTCAAACCCCACGTCCTC	AGAGACCAGTTGTCACCTCAGT
8 EXT1	GTGAAATCGAAGCAGGACTCCAT	CCCTTCGTTCCTTGGGATCAATT
8 EXT1	CACGCTGGAATCCTCGTTTTC	GTTTTATTTCGGAGGCTTGCAGT
8 EXT1	TGGCTCCTCGATGCCCTAA	GGAAACTTGGGTGATTCTTGTGTTTAT
8 EXT1	GCTCCACAGTGGTTCCACATAT	CTAATGAGCCCCATCCCTTTCAG
8 EXT1	GTCGAGCCTCATCTGAGAGTG	CTCACCTTGCACTTCTCTCATCAT
8 EXT1	CTTGACCCCCATCCCTTCTT	TTGGCTACATGCCGCTGAT
8 EXT1	GCTTCGGTCCTCAGCCCTAT	ATTTGCTGTTTACTCCTCTTCTCTTTCT
8 FGFR1	CCCACTCCTTGCTTCTCAGAT	TGGTGACAGAGGACAATGTGATG
8 FGFR1	CGAGGCCAAAGTCTGCTATCTT	ACAAGTCGGCTAGTTGCATGG
8 FGFR1	AGGAGGTTCACCTTCCTCTGAAA	GCCTCTATGCTTGCGTAACCAG
8 FGFR1	CGGAGAAGTAGGTGGTGTCACT	ACGATGTGCAGAGCATCAACT
8 FGFR1	CCACCTCCTCCCCTGTGAT	GGGAGCCCCTGTGGAAG
8 FGFR1	GGACCAGGAAGGACTCCACTT	GTGTGGCCAGCGTAATtcc
8 FGFR1	CCTCTTAAACCCAATGCCCAGA	GTGCCCCTTCTTCTTCCCATAG
8 FGFR1	CTGTTCCCACCCTGGCATTA	GGCTGGAATACTGCTACAACCC
8 FGFR1	GCTGCTCCTCTGGGTTGTG	CCCATGGCCTCTTCTCCTG
8 FGFR1	CCTCCCCTGTTCCCATTACTCTA	GAAGTTCAAATGCCCTTCCAGTG
8 FGFR1	CATTTTTCAACCAGCGCAGTGT	TGCCTGTCTCTCTTGGCTTTC
8 FGFR1	CCTCTCTTAACCCCCTTCCCTA	TGATGAAGATGATCGGGAAGCATAAG
8 FGFR1	CCCCCAGCAGGTTGATGATATT	CAAGTAAATGAGTCTCAACGTGTTCTTT
8 FGFR1	CCTTCAAAAAGTTGGGAGTCAAAGTAT	TCCAGTGCATCCATGAACTCTG
8 FGFR1	CGTGATGGCCGAACCAGAA	CATCAGATACACAGACATGTGCCT
8 FGFR1	AGTAGACTGGCCCACGAAGA	GTAGCAACGTGGAGTTCATGTG
8 FGFR1	GCGGGTCACTGTACACCTTA	CCTCTTTAGCCATGGCAAGGTC
8 FGFR1	CAGGGACGTCTCCTGGAGAT	GAACTTTTCAAGCTGCTGAAGGA
8 FGFR1	GCAGTTACTGGGCTTGTCCAT	GAGGCGGAGAAGCTCTAACA
8 FGFR1	GAGCTCTGGCTCTGGCA	AGACGTCCCTGACCTTACAGT
8 FGFR1	GAGTGTGCAAATCCCCCATCTA	GGGTCGGTCATCGTCTACAAG
8 FGFR1	TCACTCTTCTTGGTACCACTCTTCAT	CTAACACCCTGTTCGCACTGA
8 FGFR1	GCTATCCTGACTCTGCCCCTAA	CAGCATCAACCACACATACCAG
8 FGFR1	CCTCTTACCCACGACATCCAG	CCCTCAATGTATCCCTTTGGCATT
8 FGFR1	GGCAGAAAGAGGACTCCTCAGT	CGACTGCCTGTGAAGTGGATG
8 FGFR1	TGGGTGTAGATCCGGTCAAATAATG	TTTCATCTGAGAAGCAAGGAGTGG
8 FGFR1	ACAGCTGACGGTGGAGTCT	CCGGAGCTCTACGTGCT
8 FGFR1	GGCTCATGAGAGAAGACGGAAT	CTTCCTCTCGCCCATCACAG

8 FGFR1	GCACCTTACCTTGTTCAGGCAA	AGGAGGATCGAGCTCACTGT
8 FGFR1	CTGTTTGCTTGGAATGGGACA	GTGGTGTTGGCAGAGGCTAT
8 FGFR1	GGTTGGGTTTGTCCTTGTCCA	GGTGCCTCTCAAGGTTTGAACT
8 FGFR1	ACGTATTTTGGCTTTGAAATGGAACTG	CTCTCCACCCAAACCCTTGTAG
8 FGFR1	CAAGCCTGGAAATGCATGCTC	GTAACTCTATCGGACTCTCCCATCA
8 FGFR1	AGAACGGTCAACCATGCAGAG	CCATTTTGCTTCCGTTGTCTCTTCTA
8 FGFR1	CATGCAGAGGTCCTCGGT	GGATCAGTACTCCAGTGAGAAGACA
8 FGFR1	ACCACATCACCTGCAACCAT	CTGGAAGTGCCTCCTCTTCTG
8 FGFR1	GCTCCACATCTCCATGGATACTC	GCCTTAGTAAGTCCACACTTTAAGCA
8 GPR124	GGTCCTGGTGTCTCTGAGGA	GTGGAAGAGGCGGCCATT
8 GPR124	GCAACTGCTCGTCTTCCGA	GGGAATTCAGTCCCTTACTGGTT
8 GPR124	GCCACCCCCGTCATCTT	ACCCAACATCCTGTGTCAGT
8 GPR124	GCACCTTGTCCTGTCTGTGTC	GGGTGACCTCTTGCCATCAC
8 GPR124	GCAGGAAGTCAAGTCCCTGAGA	CTGCACTGTGCTGATGATGTTG
8 GPR124	TCTGCAGGGACCTGAGGAA	GTGGGCATGGAGCAGGA
8 GPR124	GGACCCCCAATCCCTCATTC	AAGGCATCTGGTGTTAGGTAGGTA
8 GPR124	CCTCACGCCTGGTGTCT	GGTTGTGGTACCAGCGGATG
8 GPR124	GCTACCTGGGCAACGACAC	GTCCCCTAACCCCATGCT
8 GPR124	CCACCCATGTGTTCCTCACAAG	CCACTTCTCACCCCATCACTTA
8 GPR124	CCTCATCACTGCTTCTGTGTCT	GGGTTCTGTGCCAGAGGA
8 GPR124	CGCCGGGCTACGCTTAC	GCTCCCAGTAGCAAGAAGGGA
8 GPR124	GCCCCCTCCTGAGTGACT	GCCCACATGGCCAAGTACA
8 GPR124	GCTGGTGACCACGCACTT	CGAGGCTCTCACGTCCCT
8 GPR124	CTTCGTCTTCACTCACCACTGT	GGGAACCGTCCTCTgcg
8 GPR124	CAGCTACCTGGGCAGCA	GCCGCCCTTGAGACTGT
8 GPR124	CAGCCGCGACAGTCTCA	TCAGGGTGACGTCGTCGTA
8 GPR124	CGCTCGTACCCGCTCAAC	GACGGTAGTTTCGCTCTTCCA
8 GPR124	GCTGCATGAAGACCGGACTC	TGCTGACTACGGAGACACCTT
8 GPR124	TCACCGTTCTAAAGTCGGGAGA	GCACTGAGGTGAAGGGATACTG
8 GPR124	GGCATCACAGCCTACCAGTC	GCACCCTGGTGATGTCGTT
8 GPR124	ACTACTCCCACTGTCTCTACACC	GCCCGTTAGGTTGGCTTCTC
8 GPR124	CAGGCCCATTGAGAGAGATGT	CCTCCCTCCCTCCTCTGG
8 GPR124	CCTGACCTGCACAGCCTT	CCCACCTTGATGTGGAAGGA
8 GPR124	GGAGGCCCAATGTTTCTCTGT	CCCCCTAGAGCACTGATGTCT
8 GPR124	GGTGTCCACATTCCTCACGTC	GTTGAGGATGTAGGTGATGATGGT
8 GPR124	GCTGCTGCTCTGCCTCTT	AGCATAGGACGGGAAGTGGA
8 GPR124	GGCCCAAGGGTGACTCA	GGCAGAGGTCTCCAGCAC
8 GPR124	CAGCAAGAAGGTGGAGATCGT	GAAACTCTGGCCACCTGTGAT
8 GPR124	GGGCGATGGGTTGATGG	TGCAGCTGCGGATGGATAG
8 GPR124	GGGCGATGGGTTGATGGG	CACTTGCAGCTGCGGAT
8 GPR124	GGCTGCCCGCTATCCAT	GGGTACTCACAGGGTAACGGT
8 GPR124	CGGCCTTCTGCCTAACGG	AGGCCTGTCCAGAAATCTCTGA
8 GPR124	ATGATCCTCTCTCTTCCCCCAAT	CTGAGCCACATAGACTACATCCATC
8 GPR124	GGCCGCTAGCTTTTCAGACAT	GAGGCTTGAGCAAATGAGGGA
8 GPR124	CGGCCATTAATTCGAGACTGTTTC	GAGCCCCTTCTCCTGCTTAC

8	GPR124	ACACCCTAAGACCCCATCTCAG	TGGGTCCTTGAGCTAGGAAGA
8	GPR124	GGGAGGGTCCAGTCGTAGT	CCCTCCAGGTGAGCTCCTTAT
8	GPR124	CTGCACTACTCCTCCCTATCCA	AGTCCCTCCCCAATTGCAAAG
8	GPR124	GGAACTCAGGAGCTCGGAAA	CTCGTCCACCAGCATCAGG
8	GPR124	CCTCCCTGACACCCTGTGT	CAGGTTCGGCTCCCTCAG
8	GPR124	GTTTCGCTGCGGCACTG	CATGAGCACGGCCACATT
8	GPR124	CTCCAGCCAGCCCAATGT	CTTTCTGTATTTCTCCATGGTCCCAA
8	GPR124	GGTGGACATGGCCAGCAA	CCTCTGCTGACCCCCATTAC
8	GPR124	GACGGTTCCCCGGTGTT	GCTCCGGCTCTCCTTcc
8	GPR124	CCAGAGTCAGGTGTGCGA	CTTGGCCCGGCTCTTGT
8	GPR124	CTCTGCTCTGCTCCGTGAC	GTAAGCACAGAGCGTGTGTTC
8	GPR124	GCCCAGAATCGCTCCCT	AGGGTCCCTGGGAGATGTG
8	HOOK3	AGTGGCTGGGTATTCATTTTCTTATGTT	CATAGGCATCATTTCCAGCAGAGA
8	HOOK3	TTTCAGCTGATGAGTAAAGAATCTCCTG	ACATCTGATGCTTATCCTTTTCAACCTT
8	HOOK3	TTAAAACGTGAAGCTAACCCTCCA	aaaTCATCTGGTTTGTACTCGCAAAAC
8	HOOK3	GGGAAGATGTTCAGCGTAGAGT	GGAGGGTCCCGGTAGGT
8	HOOK3	TTTGTAAGAGTGCTTAGCTGGATTTGA	TCCGAACCTTCTTGGTTAAGCTTT
8	HOOK3	TTCGTCTTCAGCATGAGAATAAGATGTT	GATGCGGCCACCTATAATACTCTAC
8	HOOK3	CAGCACCAGAAATACAAGCTCTTAAAA	GAACACAATTGTAAAATATAACAAGGCTCCC
8	HOOK3	TTTGATCTGTCTTGCCTTTTACCCA	CCGATAGTAACACTTCTAAGCAGAATGA
8	HOOK3	GAAGTATGAGCCTTGATTCCCTTGA	ACTATAAGCATGAAGCTCCAACATACC
8	HOOK3	GTTTTGGGAGCCTTGTTATATTTTACAAT	TTTCTCTTCCATCTCTCTCTGACTCTTT
8	HOOK3	GAGAAAACAAAGAGTCAGAGAGAGATGG	CTTTCAGATTCATTTGGTAGAGTGACTTCT
8	HOOK3	AGCAGTAGTAGGTGATGTTTACCTGT	GCAGTATACAGCAAGCCCACTTAAAA
8	HOOK3	TGGTAATTCACAAGTGATTGAGCTGAT	CCATTAATACCTGATTCTCTGCCAACAA
8	HOOK3	AGCATTGCAGGAAGAGAAAAGTAGT	TGTCTTTTACCTGAATGTTTCTTCTTGGA
8	HOOK3	TTTCTACAGAGGTAACCACAGAATCTCT	GAAGAACCTGGGCCATCACAA
8	HOOK3	ACCGTGGAAGATTTAACGAATGGG	TGCCGCCTACAACTCTTACTCTA
8	HOOK3	CACCCTTCCCTCTTCAGGAAA	GCTGACAAAGCTTCATTTAGTTCCTC
8	HOOK3	TGTTTCTAACCAGCTGAAGAAAACTACA	GAGGTGGTGCACAGAACAAATC
8	HOOK3	CTGTGTCTCATCTGCAGCCT	GGTAGAGTGAGATGCCTACAAAATTCAT
8	HOOK3	AGGCATACGTAACTCACCACTTG	CTGATGGACCATCGTTGGTACATATTAA
8	HOOK3	ACTGCTGGATCATATGCATTTGAGT	ATCTGCTTTCTTTGATTCTTCGGATAA
8	HOOK3	ATCCGAAGAATCAAAGAAAGCAGATAA	CAGTGCTCAAAACCAAAATGCCTAATAT
8	HOOK3	ACTCTGTCCTTGGCAATGAGTT	TGCTCCCCAATAAGGTTCACATC
8	HOOK3	ACAGCAAATTAATGACTTTACCCTTCCT	GAAACAAATTACCTTGCTTCTGTTCACA

8	HOOK3	CTCATCTTAGGCTGTGCTGTGAA	GGGTGACCTTAAACAGATGTTACTG
8	HOOK3	TTGCAGTTAAGTGATTATATGGGATGAG	ACCTTTAGCCTCCAATTATCTCCTACTT
8	HOOK3	GCAGAGATCCTGCATATTTTGATGAAAA	CTTCTAAAGCTCACTGGACACATC
8	HOOK3	CGAAAGTTGTGTAACTGTGTTTTCCA	AGTGTAAGTGTGAACACCACAATCT
8	HOOK3	CACTGAAACAACTTTACAAGTGTGGAT	CTAAGAGTTTAACCTGCCGCCTTA
8	HOOK3	AAAAAGAAGCTAGAAGACCTTGGTGATT	TGTCTCTTGTAGGTTTCAAGTTGACTTC
8	HOOK3	GCAGAATACTGTCAGTCTAGAGGAAGAG	TTTTGGTAAGCTACATGTCATAACTGAGAG
8	HOOK3	TTCTAAGGAGAATGAGGAGGCAGTA	CAAAGTGGTCAGTTCATCATTCTGTTG
8	HOOK3	GTTAGAAAAGGAGATCTCTGAACTTCGG	TTTCTAACACCAGAGCCAAACTGT
8	HOOK3	GATACACTGTAACAAATTCTGCATGATGAA	TGTGCTTGCCAGTCTATCTTCAG
8	HOOK3	GAATGACCCTGCATAAAAAGGCA	GTGATTGAGCGGCACAACTTC
8	IKBKB	GAAATGTGTTGGTGGCTGTTGTT	ACTGCACAAGGCCTGCTATG
8	IKBKB	TTCTGCTTTCTCCAGCCCAAA	GACAACAGTCTTCTCATCCTCATTCA
8	IKBKB	GTGGAAGAGGTGGTGAGCTTAA	CAGTGCACAGAGGTGGAAAG
8	IKBKB	CGTGACGTCAGAGCAGGAA	TCACCTGTCGGCAGGGA
8	IKBKB	tgtgactgCACCTAACAAGATTCATAT	GTATTCGTGTAATGCCGCTGTG
8	IKBKB	GTGGTTATTCTTTGACAATTGCTTGTGT	CAGCCGGTTGCAATCTTCC
8	IKBKB	CACAGCATCCAGACCCTGAA	TGTTGCTCATAACGGTTGCTTCT
8	IKBKB	GGGTCTGCTCTCATCGGTTTT	TCCTCCTTGGCAGTACTCCAT
8	IKBKB	GAACTTGGCGCCCAATGAC	GCTTCCTGATGATGACAGAAGTGAA
8	IKBKB	CTTTAGTGAAGAACTGGTGGCTGA	TTGCCATAGAAAGAAAGGACATGGAAT
8	IKBKB	CATGCGGCATTTATCTTTTGCAG	CCGATGGATGATTCTGTTTTCATGAAG
8	IKBKB	CTCAGCCTCTGCGCTTAGATAC	GCAGAGTGTGCTCCTTTCCTC
8	IKBKB	GGGAAAGCTGTGGAACTTCTT	GAGTCTCATAGGTGATTTTACTGTTGTCA
8	IKBKB	CATTGGACATGGATCTTGTTTTTCTCTT	GCCTTGTCCCACATATTATAACGGATTA
8	IKBKB	GAACTATGCTACCCTCCCTCTCT	CATCTCCCAGTCCTTCCTCCA
8	IKBKB	GTGCCCTCTAGGATGAAGCC	GTGCCGAAGCTCCAGTAGTC
8	IKBKB	GCAGAAGTACACAGTGACCGT	AAACAGGGTACGTGAAGAGAAAGG
8	IKBKB	GGTCCCCACTGTGCTGTTT	CTCCAGCTTTCCCTTGGGT
8	IKBKB	GCATGCGCCTACCACATCA	CCCCCAGCCAGTACTCAC
8	IKBKB	ACACACTATGCTCCTCTCCACA	GCTAACTTGGCTCCACACTCA
8	IKBKB	gccATATTTGACCTCAAGTCTAGACA	CCATAAATGAGATAAGGAGAAGCCCAAG
8	IKBKB	GACCTAGGCAAATAGATGGGCATT	TCCTGTTGGATTCTGGCCTTC
8	IKBKB	GAATCCTCGTAGAAGGACATTGTGT	CAGGCAAGGCCCACTTCTTA
8	IKBKB	AGGATGAGAGTCTGCAGAGCTT	GGATGGTGTGTACGAAGCCA

8 IKBKB	ATGGGCTGGTAAGAGACATGTG	CTGAATTCCGGGACTGGGATTTA
8 IKBKB	TTGCTTATTTCTGTTTCAGTTTTGTGGT	CCGAAACCCAAAATGAGTCTCCTA
8 IKBKB	GCCAGCCAGTTGTCTCCTAAG	CTGGCTAAGTCGAGAGGCATT
8 IKBKB	GTCAGTGGAAGCCCGGATAG	TGCCCTTGATGGTCACACTTG
8 IKBKB	TCTCCATCCCTTTGAGCTCCAT	ACATTTCCAAATCCCCCTGTCC
8 IKBKB	GGGAAATGAAAGAGCGCCTTG	CCCAGCAGGATCAGAGAAAGTG
8 IKBKB	GGGTTTTGTTATTGTACAGGAAATGG	ACATGGAAGCCATGGAATTCTTCA
8 IKBKB	ACAACAGCTGCCTCTCCAAAA	CTCATTGCAAAGTGCACACTCA
8 KAT6A	AGGTCCATTTTTCTCTGGTTTGTCA	CCATCACAGCTACATGAACGCT
8 KAT6A	TGGTTCATGTAGGCAGGGTTACTA	GTTCAGCGTGGCATGAACAT
8 KAT6A	CTCATGTAAGGTCCGTTGAGTGA	GCCTATACCCAGCAGCCTATG
8 KAT6A	CGGGAGTAGGCATCAGATTAACC	CATTTCCATCCGCTCCAAGTCT
8 KAT6A	GGCCTGTGTACATCATGTTCCC	AGCCCATGATGAACAGCAGTT
8 KAT6A	GATGGGCTACGGCCATACAG	CTCACATCTCCTCTGCTTCAGTG
8 KAT6A	GAAACACCCATATTAGAAGGCAGAGA	TGTTCTCCCTCGTATTAGGCCAT
8 KAT6A	CAATGTTGGTGGCAGACATGTT	TGCAACCAGTGTTTCTCTGTCC
8 KAT6A	CAGCTGAGCCAGTCCTGTATT	GGCAGCTACTCTCAACCATCAG
8 KAT6A	AATGTGCTTTCACTAACATCCTCCTT	GATTCATGTGACCGAGGTTTTCAC
8 KAT6A	GGCGGATCACAACACTCCAT	TCATGATCGTAACATGTTTCTGCTTCA
8 KAT6A	CCTATCCTTTTCTCCCCTCACCTT	TGGATATGTCAAATATGTCGACCTAGGA
8 KAT6A	TTTATCTGTGCTGCCTTCTTTTGTAGA	AACCTTTCAGTGTATTTATGTCTGTCACA
8 KAT6A	acacaGAGAAGGTCCACTGGAA	CTTGACTTGTGTTTTCTCCACCAG
8 KAT6A	CATCTTGTTGTGTCCCCGAGT	TCTATTACGATGTGGAGCCATTTCTTTT
8 KAT6A	CCCTTGACATCATTCTGTGTTAGTACATA	GAGACCCTCTACTGACTGTTTTGTT
8 KAT6A	cacctcAGGCTCCTTGGTTT	ATGCCCCTACCCgagga
8 KAT6A	ACTTCACTGCTGGCTGCAt	TCGTTTCCATTCCTAAAGCTGGAC
8 KAT6A	CATCTGCTGAGTGACCACACA	GCACCACTGAAAACTATGAGAACCC
8 KAT6A	CCCATCGTGGAGTCGTAACT	CCCCATGATGGATGTGCCTTC
8 KAT6A	CTGAAGACAATAAACCACAGTAAGTGAGT	TCGTTTGTCTTCGCAGGATGT
8 KAT6A	CAAAACAGAAGGTCAAGGGTCTCT	ATCCACAAGTCCGCTGTCC
8 KAT6A	GAGGAGTACCAGGTGTGAATTTCATA	TCTTTGTTATCTCAGTGTGTCTAATTTAGC
8 KAT6A	ACCTGCTGAGAGTGGTCTGAT	CTGCCCTTGAGAGTGGCTAC
8 KAT6A	GGTGGCCCTACTTATGAAATGGA	GCACATGAAGAAATGTGGTTGGTT
8 KAT6A	TTAGAAGAGGACTGACACCTGAGT	GCCAAGATTAGAACCCACGTTTG
8 KAT6A	GGTATACAAAAAGATGTGATACTCACTGGT	TGACAAGCAGATCAGCATTAAGAAGTT
8 KAT6A	CTGTAAATCTCATTGGCAGGAGGAT	ATCGTTTACAGGCTGCCCAA

8 KAT6A	CACTCTCCTGGATCTTGGGTTTAC	CTGGAAGAAAAGACCTGGTCGAA
8 KAT6A	TCTTCCTCCTCTTCTTCATCGATCT	CACAATAGCAGTGTAGTCACAGAAACTA
8 KAT6A	GTTCATCTAACACTTCAGTGGTCTCA	GAAAAGATAGTGTTCATAACTGTAAATACTTGC
8 KAT6A	GATCCTTGTTCTGGGCTGATCT	CGAGGACCCTCAGATGTCCAT
8 KAT6A	GCAGATTCCAGTCAACTTGCTT	TCTCCAGAGAAACCGTTATCTGATCT
8 KAT6A	CCCGACTCAACTTAAATCCTGGTT	GAAAACGTGATGTGAAGAATTCTCCT
8 KAT6A	CAATATGCCATGTAGGAAAGACGAC	GAGTTGCTGACCTAGTTCTGATTTGA
8 KAT6A	GGTGTGGATGTATCTGGCTCAAG	TGCTAGTCTTGGAAGAAGGAACTAGA
8 KAT6A	GCTTGCCTTCGTAACACATTTTAGA	GTGTTTTTCTTCACAGCCGGTT
8 KAT6A	ATACTCTTTCAGAACTGTAAGCTAAAACG	CTCGCCACCAATTCTCACAAAG
8 KAT6A	TCCGAGATGGCTGGCTATTTG	GGTGGGAAAGTCTGTGTCTCAT
8 KAT6A	GACGCATGACAGTCTTCAACC	GTACAGGCAGTGCAGTCTTTGA
8 KAT6A	GCCATAACTTCTGGTTTCTTTTCACTTT	TTCTCTCAATACTTAAAAGGTCAGAATAGGT
8 KAT6A	GAAATGCGTTACCTTTCGCTTCA	AGATTAACAGAAGGAAGTGAGAGGCT
8 KAT6A	CATGCTCACTGCTTTCTTCTTGAG	CCGTGGTGTCAGAGCAGATG
8 KAT6A	TGTCACCCTCACTGTAGCGA	GGAAACCTGACCTTCCCAAGAG
8 KAT6A	TAATTAGTGTTATCCCGAATAATACATGTACAG	TGGACTACTCTGAGCAATATCGAATCA
8 KAT6A	TCAACCCCCTCACTGAGTCTT	AGAGACGTCTTCAGCTCCTCA
8 KAT6A	TGATTTCCTGTTGCCCCTCTTTC	GTCTCCTTGAAGTTCAACAAGAAAACC
8 KAT6A	GTAAGGCCATCAATGAGCCCTT	AGCCAATCAGCATCATCATCATCA
8 KAT6A	GATTTCTCCCCACATTCTCCATATTGTT	AGTCCTTCCTCATGATAGTCTTCCTG
8 KAT6A	CCATCTATCCGCTCTAAATATCCTTCTTC	TTTAGATCAAAAGGTCCCTTCAGCAA
8 KAT6A	AGAGACTATTCATGCCCTTACACTGA	GCTGAATTTGCGACCTGTAGATG
8 KAT6A	CCAGCGCAAACATTCTGGATCTA	GGGTCTCATCTCCACATTCATTTTTATG
8 KAT6A	TTACTGCTTAACCCACAATGTAATTAATCT	GCCAGGAAATCATGACTGAGAAAGATAT
8 KAT6A	AGTGCTTGTTCTTGGATATCACGAAATA	GGGACAGTTAACATGTGAGCTCTT
8 KAT6A	CAGCTCTATAAACCCCGAAGCATA	GCCACGGCAGACTCCTTAAAG
8 KAT6A	TCCATCTTTAACACTCAACTCCAATTGT	AGTGGATTTTGGAGGCCATCAAA
8 KAT6A	ACGCTGTTTCTGCTTTTTCACTTT	CATCAGGATAAGGGATAACGACTCTATG
8 KAT6A	GTTGAGCCGATAAAGAGGTCCAT	CTCAACTTTGAAAAGCATTGAACGTTT
8 KAT6A	CAGACACATCCTTCTGACCTTTCAAA	CCTGGGCGAATAGCACTTCC
8 KAT6A	AATTTTCCATGGTTCCGAGGCTTA	TGGCTTGGATCGTAAAACTGTTTTAGA
8 KAT6A	GCTTGGCTAGGCTGAAGGTG	cccgcTGTCACAGTGTAGT

8 KAT6A	TCTTCGTGGTCGTCCTCAGA	AGCCTGGTGTTCAAGAGTCTTTTT
8 KAT6A	ggCTGCTGCTGCTGGTTA	GTCCTCCAGCAGCCTCAC
8 KAT6A	CTTTTCCCTACTCTTCTGCATATTAGCA	CTGCAGAGACTGCCCAGA
8 KAT6A	CAGCGTCGTGGTCGTCAT	AATAGTCCTGAGACCGAAACCAAG
8 KAT6A	CGTCATTCTGGGCAGTCTCT	GCAGACAGCAGCAATAGTCCT
8 MYC	GCCACAGCATACATCCTGTCC	TTCCTTACTTTTCCTTACGCACAAGA
8 MYC	CACAAACTTGAACAGCTACGGAAC	GGTTGTGAGGTTGCATTTGATCAT
8 MYC	GGGAGGCTATTCTGCCCATTTG	GCAAGTGGACTTCGGTGCTTA
8 MYC	GCACCAAGACCCCTTTAACTCA	GTAGAAATACGGCTGCACCGA
8 MYC	GCCAAGCTCGTCTCAGAGAA	GGGTCGATGCACTCTGAGG
8 MYC	CCAGCTTGTACCTGCAGGAT	GAGGACTCCGTCGAGGAGA
8 MYC	CTCTCCGTCCTCGGATTCTCT	GCCAATGAAAATGGGAAAGGTATCCA
8 MYC	AGGAACTATGACCTCGACTACGA	GCAGCAGCTCGAATTTCTTCC
8 MYC	CGCCCAGCGAGGATATCT	GTCGTTGTCTCCCCGAAGG
8 MYC	CCTCCTACGTTGCGGTCAC	TCGCAGATGAAACTCTGGTTCAC
8 MYC	AGCTGCTGGGAGGAGACAT	GGGCTGCCGCTGTCTTT
8 MYC	TTTGTCCAGAGACCTTTCTAACGTATTC	GCCTCTTTTCCACAGAAACAACATC
8 MYC	GGAGGAACAAGAAGATGAGGAAGAAATC	AGTTGTGCTGATGTGTGGAGAC
8 MYC	CCCACTGGTCCTCAAGAGGT	GCTGGTGCATTTTCGGTTGTTG
8 MYC	TGTCAGAGTCCTGAGACAGATCA	CGTTTTAGCTCGTTCCTCCTCT
8 MYC	CGAACACACAACGTCTTGGA	CTTTTGCTCCTCTGCTTGGAC
8 NBN	GGATTTGGCTGAAACAAAGCTGT	CAGAATGGCTTTTCCCGAACTTT
8 NBN	AGGCACAATCATGAAGTAAGCCATA	ACGTGAACTCAAGGAAGACTCAC
8 NBN	CAAAAGTAATACCATCCCCCGACTT	TCAAACAGATGAAATCCCTGTATTGACA
8 NBN	CGGGAAAAGCCATTCTGCATTTTT	TCTGAGAAGTGAATGTACTGAGTGGTA
8 NBN	CAGCAGCAGAAGCATACTTAATCAGA	GCTTCTCAGCAGCAGCAG
8 NBN	CTGAAAGTAGTTTCTGATGGAGTTGGT	GGAGTCCTGCAAAACAAGCTCTAATAAT
8 NBN	GTTTGGGATTCTCATCTTAGCCAAAGTA	GGCCAAAAGAAATCAAAGTCTCCAA
8 NBN	GTGCATCTTGTGAAAGCATTCTGAATT	ACGATCTTTGTTTCTCTATTAAAGTTGCTGT
8 NBN	GTGATTTCAACCCCCTTACTGGA	GTTGAGTACGTTGTTGGAAGGAAAA
8 NBN	GATCATTTTCAATCAGAATGGCACAGT	TGTTCGTGTACATGTGTATGTGTCTATC
8 NBN	TGTTTTCTTTCCTGCCGTCCT	TGTCAGATAGTCACTCCGTTTACAATTT
8 NBN	TCAAAGGTACATGAGAAAGGTGAATCAA	CACCACTGAGCAAATGTTTGAAATTATGT
8 NBN	CCTTTGGAGCATATCCATTATTGACTGA	GGAAGCTAGGTTGATAACAGAAGAGAAT
8 NBN	TGTTATTCCTGTATCAACAACACACGTT	CATAAGAAATTGAGTTCCGCAGTTGTC

8 NBN	CTCTTCTGTTATCAACCTAGCTTCCC	AGTAGCACCAAGTCTTCTTGAATTGT
8 NBN	ACACAGCAACTATTACATCCTGAAACA	CCTGAAAGCAGTTGAGTCCAAGA
8 NBN	ACGTCCACAAATGAGTGCACAT	TGGATGTAAACAGCCTCTTTGTAGTTT
8 NBN	TTCCCTGGTATCCCATTCTTCCA	CTACAACATACGTAGCTGACACAGAA
8 NBN	CTTTACCATGTATCTGCTTGCTCTGA	CAGTTGTGATTCTTTCTTTCTACTTGTGT
8 NBN	GCAGTCGCTACCGGGAAAAT	CGTATCCGCGCTCGTCTA
8 NBN	ACCCCTAGCAAGTATATGAGTAACGTA	CTATTCCTGAAGCAGAAATTGGATTGG
8 NBN	CCGCCAATCCAATTTCTGCTT	AAAGAGGTTGCTTTATCTTGACATTATCTGA
8 NBN	GGTAACTTTCACTGATACCATGACAAGG	CTCTTCTTGTTTAGATGTCTCTGGGAAA
8 NBN	TCCTCCAAGTTGCAATATAGCTTGATTT	AATTGCCATCTCTGCAACTCTGA
8 NBN	GAATTTGCTTGAAGGCCACCAT	AAAACTTCCACACATCATTGGAGGA
8 NBN	TCGAGCATGATGAGCTATTAGATCTGA	CACTTATGCATGATTTACCATCTTTGCTT
8 NBN	CTTACTAGGAAGTTTTTCCATGGCTTCT	CCTATTTGCCAAAGTGTGACTACAGTT
8 NBN	CACTGACCTCTTGTGATACAGTTGA	AGGATGATAGTGAGATGCTTCCAAAAA
8 NBN	CTCTTGTGATACAGTTGAAATACCTACCT	CAAACTTCAGGATGATAGTGAGATGCT
8 NBN	GATGCTCATACTGTCAATACAAAATCGAA	GCCAAGGATGGATATAGAAACAAATGAC
8 NBN	CTACTTTCTGGTACTGCTTCATCACT	GACACAAAACCAGAGTTAGAAATTGATGT
8 NBN	TGACATCTTCCTCCTGTTTTTGAACTTT	CATGCTGCAGAAAAGCTAAGATCAAATA
8 NBN	GGCCACATCATCCATTTCCCTTT	GTGGAAAAATAAGGAGCAGCATCTATCT
8 NBN	TTGTGTCCACAGGCTCATTCTC	GTCTTCATGCAAATCAGCAAGAATAGAA
8 NBN	AGCAGGTTGTGTTTGTTCTAAAAGAGA	GCCAAACTAAATGGTTACTTAGCTGTGT
8 NCOA2	AGTACTAGCATCCTTACAGTCTTCTAAA	GAAAGACCAGTTCTTCCCTCATCA
8 NCOA2	AGATCCTGGCGAGTAGTAAAACTTTC	GCTGTCATAAATACACTTGGGTTTGTT
8 NCOA2	GTCACTCTCCTCCCCATCTGA	AATTTGTCAAAAACCTGCTGCCA
8 NCOA2	ACCCAGCAGACATTTACCTATAGACTT	TGTAGTGAACCTGGAAGGCAAC
8 NCOA2	GTCACATTCTCTGACACAAACACAAC	GGATCATTCCCTTCTTAACAATTGCAT
8 NCOA2	AGAAGCACTCATGTGGCTTTAACA	GGATGGGAGAAAATACCTCTGACC
8 NCOA2	CTTTCTTGTCTCTGCCCTGGAG	CAGACACTTCATCATCAGCTCTCTT
8 NCOA2	CCCAGGTGCCATCGAAGAAC	GCAAGATCCAAACTTTCACACCAT
8 NCOA2	GCATAACTAGGCCGCTGTCC	AGCAGTAGATCCAGAACAGTTCTCA
8 NCOA2	CAGCATGATGTTGGAATCCTGACT	GAGAAAACAGCCTTATATTGACTCTGCT
8 NCOA2	TTTGGGTGACCACTAATTAAAGTGTGA	CATGATAACCAGGAAGCTCATCAGAAAT

8 NCOA2	GCGAAGCACTGCATAGTTTCAT	CTGGATTACTTTTGCCTTGCTCAG
8 NCOA2	GGATGTCCTTCAGCTGTCACA	CCGAATGGCACATACACAGAGT
8 NCOA2	AATTTCTTGGCATAACCAGCCATTTC	GTTGACAAGCAAGCCATCATCAAT
8 NCOA2	CCTGAGACTGTTGCATCATGGG	ACAGTCTCTTTTGGATTTCTGTTTCTG T
8 NCOA2	AGCTGTGAGTTGCATGAGGTC	GAGGAGATTTTGGATGATTTGCAGAA T
8 NCOA2	TGGGAAAAGCTGTGGTAATTGACT	GGGCTTGTGGTGTCTACTCTTAGA
8 NCOA2	AAAGCCGTGATATGAAGCCACT	ATGTGTCCATGGCGACCAA
8 NCOA2	TCATCTGGTTCATGCTGCTCAT	ACCAGCAAGCTGTAACATTCATTCA
8 NCOA2	AGCACTAAAATGAAGATGTGCTACCT	GCACTGCCATTTAATTCACACTTATGT T
8 NCOA2	CCCACCAATAAAGTAACAGTGCATTT	AACAGTAGTCCCTACTCAGTGATACC
8 NCOA2	GATTACCCATCATTCCTGGCTGA	TTATTGCCAAACCAGAATTTACCACT TG
8 NCOA2	GTTGGGCTTTGCAATGTGATGT	CAGACATTGTCCCTAAACACTACTGT TA
8 NCOA2	CTGGTTGACAATATATCTATGCCACTT TTG	AGCTGCCTGGAATGGATATGATTAAG
8 NCOA2	TTTCTTACCCGTGTTGTGTCTCC	TTTTCCAGTGCACTCTTGGACAT
8 NCOA2	AGAGCATAAAAGTTAAAAGCAAACA GCA	GCTACCACCAGTGCCATGAAC
8 NCOA2	CGAATCATACCTCCTTGGACTGG	CAGTTACTGCATCTCTCAGTTTTCTTT G
8 NCOA2	TCATTCCCTGGTTAGCCAGAT	GCACAGCAGTTTCCATTTCCTC
8 NCOA2	GGATCTAAGTCCAGTACCGTAGTTTG	AACTTTGATGATGAGAGGACAAGGG
8 NCOA2	CACCATGCTTGGTGTCATATTCAA	CATGTGTTGCCCCGGATTTC
8 NCOA2	GCAGACCGTGGAGTACCTGTA	GTATCTGGCCTTGCGGAATTTTG
8 NCOA2	TCTATCAATCTCCTCCAGGCCAT	GCCCAAGGTGACATTTCTGTCTTTA
8 NCOA2	CAGATGAGACAGCTCACGAGAA	GCAGCATGAAGGAGAATCTGTG
8 NCOA2	TCATGATGATGCCTCTTAGCATAGGA	CATGTAGGTATAATGCCTGCTGTTACT T
8 NCOA2	TCTTAGAACTAGGATTACCTGTGAAG CA	AGACAAGGATTGGCATTCAGTCAAAT
8 NCOA2	GCCATCAGACAAGGAAAAACGATAG	TGCTGGTGGGAAACTATTATGAAACT T
8 NCOA2	CTACCCTAGGTGCTCAGGACT	CCCTTTCTAGGGCCATCTGAATTAGA
8 NCOA2	GCTTGTTGTTGGCTATACTGAGGT	CTGCTTCTGTGTTTTCCCTTTTTCA
8 NCOA2	ATGCCCCTATGGAGAAAATCCTTG	CCAAACTTGAGAGACTGGACAGTAAG
8 NCOA2	CAGGAGTTTGGTCTGCCCTTT	GGTACAACTGGACAAGCAGAGA
8 NCOA2	GTCATTTGTTTCCTTTTGCTCTCCA	AGTTAATATGAATCCTCCCCCACTCA
8 NCOA2	TTTTGTGTTACTGGCAGGATCTGT	AGCTCCTGGATCAGAAGTGACTAT
8 NCOA2	GGGCTGTTCATTTTGAGTGCATA	GGACATGACCCTCAGTAGCAATATAA ATT
8 NCOA2	GGCTCACCGGCTCTTGTTTA	TCAAGGAGAAGCATAAAATTTTGCAC AG
8 NCOA2	TTTTGAGTCCAAGCTTCCCATCTT	CCATAGTTATACCAACAGCTCCCT

8 NCOA2	CCATTTGTTCCTTTGGGCCATT	GTGTGTGTGATGAATCCGGATCT
8 NCOA2	TGAGGGCCTGAAGTGCATTG	CACCAAGGCATCGCATGAG
8 NCOA2	TTCCCCATCGTTTGTCCAGTC	GTTCTTTTAAGGTGAAGAGGCCATATCA
8 NCOA2	GAACTGCTGTCCTGCAAGAGT	GCCAGCTCTTTGTCGGATACA
8 NCOA2	GGAAAACTGACTGGGTGGGATTC	CAACCACTCCTCAGGGTAGTAAC
8 NCOA2	CAAGCTACCTGTGGAGTCTTTGTT	GCAGAGCAGACTGCATGACA
8 NCOA2	GAAACAAATAGACACAGCTCTCCAGA	GTTTTTGTGGTGGGCTTTATGATGTT
8 NCOA2	GATTAAAGGGACTCTGAAGGGACTT	AAACCTGACAAATGTGCAATCTTAAAAGA
8 NCOA2	GATCTGACGAATTTGCTTCACAGTT	CCCAAAAGGAACACTGAAAAACGTA
8 NCOA2	CACGATTACGTTTTTCAGTGTTCCTT	TCTGTGTTTATATAGGTAGCTTTTGTTTGCT
8 NCOA2	CTAGATGACAGTACAGGGCTAGTG	TTGACTCTGAGGCCTGGAGTA
8 NCOA2	GTATGTTGCCTTACCTGTGTTGG	AGGGCAGTTACTTGGGAAACTTG
8 PLAG1	CATCTAGGGCACAGCTACACAT	ACACTATAGGGCTTGGGTCTCTG
8 PLAG1	TGAAAGCTGCTGACAGTGAGT	GGAATGAGCTATTCCCAGGAAGAAG
8 PLAG1	GGCACAGACACATTGCAGGTAA	GTGGTGCACACTGGAAGAAAG
8 PLAG1	TGCACAATACTGACAGAGGAAGTC	GAGTGCTTCTGGAGCACCTTA
8 PLAG1	ACGACTTGCCTGCATGAGATT	GAATGTGGCAAGAACTACAATACCAAG
8 PLAG1	GGCCAAGTGACGTTTAAATCCAAG	TGTGAGAAAATGTTTCACCGGAAAGA
8 PLAG1	CAATGGCTTCAAACAGCCAACAT	GCCCTACAAGTGCATACAACAAGA
8 PLAG1	GAAACAAAGGCCTTGGTGCAG	CGTGGTGAAACCAAACCAAGAAAA
8 PLAG1	CACACAGTTGGCAAGGAAAGTT	TCCAACAAGAAGGCCTGGTTTAG
8 PLAG1	GAGCTGGGAAACAGAAGAATGTG	CTATCTCCATCAGTGACCCCCTAA
8 PLAG1	GAGAAAAATCCAATGCTGGTGTGT	CCCTCTTCATCCCAAGATTCTCA
8 PLAG1	TGTGTATGGAGGTGATTCTTCAGATGA	TTCCATTTGAAAAGTGAATACATGCCATT
8 PLAG1	CCTAGCTTAGATGATGACGATGCT	CCCTCTCACCACCTTTCTTTCA
8 PLAG1	GCATATGAGGTAGAACTGAACGGATATT	GCAGTTAAACCTCTACAACACTCCAT
8 PLAG1	CGAGCTCTGCATGGACTGAA	ACCAGTGGATTTCCTTGACCCAT
8 PRKDC	GCAGAAATAAGCAAATTCCCCAATGA	AAATGGGCCAGAAGATCGCA
8 PRKDC	GCCTCAGGGCTTGTACTCAT	GGGCGCTAATCGTACTGAAA
8 PRKDC	TTAATTTCAACAGAGTAAGGTGCGATCT	GAGTGACTCCTCTTACCCATATTTACAAT
8 PRKDC	CTATTTCTGGAGGTTGTTGAAAGTCCT	GGGATTGGAGACAGACATCTGA
8 PRKDC	TTCTCGTTTTCTAAAAGACGTGACTGT	CTTTGCCTCAGAGAGATCATCAAGATTA
8 PRKDC	TCCATGGCCACCATAAAGTTGT	CTCTTTTGTAGGCGGGCCTT
8 PRKDC	TTGACTGAAAGGGACCACGTC	GCTGTGTGTATAGTCACCCTCTAATACT

8 PRKDC	GCATGCAAAGGGAATTTAATTCTGTGT	GGACAACTACGTGTCTCTATTTGAAGT
8 PRKDC	GACCTGCCACTGGAGAAGT	TGTTACCTTGAAGGACCTTCTTTTGAA
8 PRKDC	CTTCTCCTCTTGGGACATGGTG	AAGAGCAGTTTGGGTTTACTCGTTAA
8 PRKDC	GTGGGCACACCACTTTAACAAG	TCTGTTCTGTGTTATGTCGTCTGATTTT
8 PRKDC	CAGGCTGTGCCAGTCCATA	TGACAGGATTGGAGCAATGATGT
8 PRKDC	GGGTTTTTGCTAGTTCAGCTCTT	GGCTTCTGGCACATAAAACATATAAGTT
8 PRKDC	tggccCAGAAACATTTGTAGCAT	TGACCTGCCTCCCTATGACC
8 PRKDC	CTACTCTGGATGCCACACTGT	CAGTCTCAATTTTCTCCTTTGTGCTT
8 PRKDC	CCAAGAGCAGGGCTAGTGTTC	GCTGCCAAAATTTCATGGAGTGTT
8 PRKDC	CCACCTCCAGACAGAGTGTTTTC	CCCTCCTCTTGCAGACAGGTAT
8 PRKDC	AGAAAGAACACAGCATTCATGAACCT	GAGGACAAGTTTATTGTGTGCTTGAAC
8 PRKDC	ACCTATTGGGTGGTTTACAACTTCTC	AGGAACTCCGCGGTTCAATAATT
8 PRKDC	ACTAACCTTTTTCATGCAGTCCACAT	GAGGCAGTCTGGAGGAACTTAG
8 PRKDC	CGATGAGCTGCTCCAGAACAC	TAAGCCATAATTCGTGCAGTTTCTTAT
8 PRKDC	CACAAGTGTTAGAATCTGATCTCTGCAT	GATGAGACCAAGAATAACTGGGAAGT
8 PRKDC	CCCGAGAAAGAGCTGACACTTC	GTTTCTATTTGACAATATAGTTTGTTGCCTCTA
8 PRKDC	CCTCAGCCATGTTTCCTCACAT	CACGGAGACCTTCCTGACATT
8 PRKDC	ATGAGGCTGCTGTGCTTGAT	AGCTCATGCACGACTAGTTTCC
8 PRKDC	AAGTCATGCTAACAGAGTGTCAAGAG	CCGACAGAAAATATGTTACGCTAAGAGAA
8 PRKDC	AGATTTACCTCCCAAATGTCAGATGATG	CTGTGCTGAAAACAGTCTCTTTGTT
8 PRKDC	TGGATAAAAGCGGTCAACTTACCC	TGAAGGAGCTGCATAAAGAGTCAAAA
8 PRKDC	CTGGATTGGCACCTGCTAACT	GCCGGGAAAATCATGTTCTTTTCTTCTA
8 PRKDC	CCAGCCAATCGTCTCTGGT	CCAATTAGAAACTTCTGTCACTTGTGT
8 PRKDC	GAACGTTCATCAGGACAGGGA	TCCTAAGCAAGGTTCTGGTTTGTTTTA
8 PRKDC	AAAGCCAAAACCCCTTCTAACAGA	CACCGTTGTGGTCCGGATT
8 PRKDC	GCAGAGTCGTGGTAAACTCCAT	GAAAGCATTGCCATGCATGACATTA
8 PRKDC	CCAAAGCACTTTTCTGCTGCTA	TTAATCTTAGTGGAGTTGAAAGGAAGCA
8 PRKDC	GTAGTGTCCACGTGTGTGCTTTA	TTCCTTCAAGGATACTTCTACTGGTCA
8 PRKDC	CTACCTTGCCACAAACTCCTTATTCTTA	CACTCTGTGGAAGAAATCACTGATAAC
8 PRKDC	GGATAAACAATAGCCTGCGGGTA	CAGCATTAAGTTTCAGCTCTCTGTTTT
8 PRKDC	ACATTTAATTGTCCCAGCCCAGT	TGACATTTTGCTGTTTTCTCAAGCA
8 PRKDC	TCCTCCTATCATTTCATGTAATTAAATTTCACT	TCGAGTGCTGGAAGGATTGTTTATC
8 PRKDC	ACAATCCTTCCAGCACTCGAC	GTCCCTAAAGATGAAGTGTTAGCAAATC

8 PRKDC	GTCTAAACACAGCTCTTTTGGATGAAA	TCCAGAATGGAATGCCGCATAT
8 PRKDC	CCTTTAAACAATCACGTGAAACCAAGA	TGGAGCAGTTTTATGGAATCATCAGAAAT
8 PRKDC	CGGATAGCAATAGATAACTCCTTGTTGT	GCAGGTTTCTAATATGGTGGCGAA
8 PRKDC	ATTTCTGCATTTTTCGCCACCAT	AGCAATTGCGTGTAACTATAACAGTACA
8 PRKDC	GGTTTCTTTGTGAGCACTTGATAAGT	TGAGGATCTTCACCATTCAGCTATTTTT
8 PRKDC	GACTTTAAAACTGCATAGGCCACAATC	CATCAGTGGTTTCTACAAATTGCTTTCT
8 PRKDC	CTGCAAGAAAGACTCACCTCGAAATA	TCTCCCTGAGAAACAAGCAGAATTTT
8 PRKDC	TGAAAATGAGTACACCCATGGTTCAA	GCTTAGTTGTTCGATTATGTCCCTTACA
8 PRKDC	TGATGATCTCTCTGAGGCAAAGCTA	CGTGTGAATATAAAGATTGGCTGACAAAA
8 PRKDC	CTCCAACATCATGTTTTCCTGACAT	CTTGGTGATCTTTGCCTTTGAAATCA
8 PRKDC	GGCAAAGAGCCAGTCAAAGCTA	ACAAATTTTCTGCTCGAAATGACCAG
8 PRKDC	GGGTTTGGATAATCTGGGCTCAT	GGAGCCATGAAACTAGGTTACCTT
8 PRKDC	GCAACCGGTCCAAGGTATTTG	GCACATTCTTTCCCATTTGCCATT
8 PRKDC	CTCTATACTCCGAGGGTTGTCAATG	CATACAGCTCCCAAGACCCTAGA
8 PRKDC	CACCCGTCTCCGAAAACGA	AGATGGTCCTTCCTATATGTCTTCCC
8 PRKDC	GGGTACTGTCTGCCAAATATGACA	TCACTGGCTTGATGAAAACCTTAGATT
8 PRKDC	CAACCCATACATTAAGAAACATGCACT	CAAATTGCAGTCTGTACAGGCTTTA
8 PRKDC	AAGCTGATGAACTCCTGAATTTCTGT	CACAGCTTGATTGATTTATAACATGGCA
8 PRKDC	ACCTGGATACATCCAAATAAAAGCACAA	AGAGTAGTACAAATGCTTGGATCTCTA
8 PRKDC	TTGTCCTCCTAGAGATCCAAGCA	CAAGTGGGTGGCAAAGAGTTTT
8 PRKDC	AACACAACCACATCTATGGAAGCA	GGGTGAAGTTCATCCTAGTGAGATGATA
8 PRKDC	GCGGAACAGGTTTTCTGCATTATT	GTAGTTTGAGGCCTTCACTTGTCTA
8 PRKDC	TGATTAGATCCTATCCTTCCCCAAACA	CTAAATGTAAAATTCCAGCCCTGGAC
8 PRKDC	AGAAGGTCCAGGGCTGGAA	GAGAAGTAGTTTCAGGTATTGCTGAGT
8 PRKDC	TGCTTCACAAGTACTACCTGTTTTTGA	TCTACAGCCAGTATAGACAGTGAGAAC
8 PRKDC	ACCTGATAAAATGGTTCACTCCAGATTT	CCGAGAAGGATTTTTGGGAACTTG
8 PRKDC	GGAGCAGCCTGGCAGTTA	GTGAAGGGTGGCGAGGAC
8 PRKDC	GGTGGTTGTAACAGTCAAGGGATG	CGTGTTCTCATTGGTATTCTTTCCAACT
8 PRKDC	GATCCCATTCATGACCTGGAAGAG	CCCTGAGATGACATTCTGATTTTTGAAG
8 PRKDC	GGGACACTGTCACAAAAGTGAAAA	ACCAATGAAAGAAACGGGCCTTAT
8 PRKDC	TGCGTGTACCATGATGCTGTA	CATTTGTGCTCGGTACTTTGAGAG
8 PRKDC	TCAAGGGTCATAACAAATCAAAACAGAGT	CAGGCTTTGGTGAATAATATGTCCTTTG

8 PRKDC	GCAGCGGCATACACTTCTTTATATCTTA	GGCTGTCAATATGACATGAGAAGGAAAA
8 PRKDC	CCTACAATAACAATAGTGCACACCGT	AGCATACAACTGCGCCATATCT
8 PRKDC	ACTCATTGAAGACACAGCAGATGAC	TGATTGCCATGTTGTGTGAATTGATTT
8 PRKDC	AACCCATCGATTTGAATCCTTTCTCA	CCCAATAGGTTCCTATGGAAAGAAAGAA
8 PRKDC	TCTCTGGCTTCTTTCCTAATTTCAATGT	ttGCCAGATTGCTACTAAAACAGAAAAAG
8 PRKDC	ATTTTGGTATTATCCACTGAAGACTGCAT	GGCAGCGTCATCCACTTCTC
8 PRKDC	TGTTGATCGTTTCTGAGAACAAGCTATA	GATGTGCCCCAGTTATGGTACT
8 PRKDC	CTGAGCACACAGCAGTGTCTA	GCTTTGAGAGAATTCTTCAGCACAATT
8 PRKDC	CACATCAATGGCATCCACCAC	GGCCTTCTGGAAAGCGTGTA
8 PRKDC	GGGTCATCCTTCCTGAACATTTCA	GCACTCATAAAGTTCCTCTTCAAAGCA
8 PRKDC	AGGAGTTCAAAAGTTGTGTCAAAGATACA	AGCAAAAGGGCCAGTTCTCA
8 PRKDC	ACTTACCCACAGTACTAATGCAATTCC	AGATAGACAGTTTCCCACAGTACAGT
8 PRKDC	GACCTGAGCTCTGCAGTAATTTAGT	CCACAAGAGGAGTGAAAGGTTACAG
8 PRKDC	ACACACCAGCTGCATTTTTGG	CCTCTAACTCACGGTTTGCTGATTATTT
8 PRKDC	CACTGACTTCAAGGGTGCTCT	CCTGACCTCTCTGATTGCATGTC
8 PRKDC	ACCATCATCTGGTCAGAGCTCA	GGCCCTGAGTCTGAATCTGAAG
8 PRKDC	CCAGTTCTGACTTCCCCTGAA	GTCTCCTATGGGTGGCAGTTTAA
8 PRKDC	AATCAGGCATTGCTGGTTAGACTT	CCTGTTCTGGCAGGATGTCTG
8 PRKDC	CAGAAGTGAGGACAACCCCTT	TCCATAGATGTGGTTGTGTTTTTCTCTT
8 PRKDC	CAAGAGCACCTGACCTCACA	TTGCCTGGCCAGCCTAC
8 PRKDC	CCTCCTCTAGCAGGCGGAT	CTCCTTGTGAAGACATGGTCCAA
8 PRKDC	TCCCAGTATCACTATTCCAGAAACCA	GACTTCAAAGTGGAGTTCCTGAGAA
8 PRKDC	ACCGGGAATCTCCAGCTCAT	GGTTCTAAACTACTGAGAATGAAGCTCA
8 PRKDC	AGGAGTCACTCCAGGAATATACAAGAA	CTCTTGGCACTCTCTTAACTTGTTTACT
8 PRKDC	CATGTTGGTAATGTCGTTGAAGTCAC	TCCAAAGTCCAAACTAAACATCAGTCTT
8 PRKDC	GCAAAATCCTGTGACACAGCA	ACCCCAGTGACAGGATGGAA
8 PRKDC	TGATATCTTCTTCCTGCTCTTGCAC	AGCTTCCTATTCCTGAGTCTGTGA
8 PRKDC	ACAATGTAATGCTTTCTATCTGCAGACT	GAACCAGAGAGTGGGCTTTCA
8 PRKDC	AGGCACTTCACTTGAGTCTCTTC	ACATGCTTTGTGTTTTCTGTGTTTGTT
8 PRKDC	ACCCAACTTATATGTTTTATGTGCCAGAA	AGTAAGTTGGATCAAGGAGGAGTGA
8 PRKDC	ACCTTAAAGAGCAGTTCAGGATTAGAGA	tatTGATTTTTGGAAGATATTTGATTGCCTAAA
8 PRKDC	TCCCATTTCAGCATGCTCAATACA	GTCTGCCTGTAAACAGCTTCAC
8 PRKDC	AATATATTATGCAGAAGCCCAGCTCT	AGCTCACCTAATTTACTGTTTTGTCACA
8 PRKDC	ACATGTAAATGCATCTCACCTGTATCTG	CCCGCACTACATTGATGTACTAATTTGT

8 PRKDC	acccggccTATTATTTCATTTCTTACATC	ACTTGCATCCAGCTAAACCTAAAGATTT
8 PRKDC	CCACCAGGTTAATGAAAGCCGAA	AGTGATTCGTGATTGAATGGTACAATGT
8 PRKDC	ATTTCCTTCTGTAATACACGAGCCAT	CAAATCACCAAGAAGATGGGCTACTATA
8 PRKDC	CACGAGCCATGGAAAACTTGATTAATTT	CCTTTTTGAAGCTAAATGAATCTACCTTTGA
8 PRKDC	GCCCATCTTCTTGGTGATTTGAGTAT	GCATTTTTGACTGTTAAAATTCTGTCTGTCT
8 PRKDC	ACCTTTAAATGTAGATCCAGCTTTGTGT	AAATGTGTCCTGTTTTTCTTTTCCTTCC
8 PRKDC	TGGAATGGCTGAAACATGTTCTCT	GATGTGTTCCTGCCTCGAGT
8 PRKDC	GCTGTGAGCGCTAATTCTGTG	ACAGTCACGTCCTCAGATGAGAT
8 PRKDC	CCAGGCCACATAGCTCTTCATC	AGCTGGCATTAGAAATGTTTTGATTTGT
8 PRKDC	CACCAGAATAAGGCAAGGGTGA	CCCAGCCCACCCTCTTG
8 PRKDC	CTGAATGGCCCCCGAAGTA	AAAGGCAACAGATCCCCTAATTTGT
8 PRKDC	CCTTGAGAACATCTTTCAGCCACAA	CAGTGAAACAAGTTGAATGCGTAGTAAAA
8 PRKDC	CCTTACTTCTCTCACAAAAGCCCAA	CCAGTGGCAGGGCAGATAAG
8 PRKDC	TGTGTCAGTGTGAAGTCATGCT	CCATTGATTCTGATTGGCGTTTCC
8 PRKDC	GTACTTCGGAAACGCCAATCAG	TTCAGCTGCATATGAAGTTATGGTACAA
8 PRKDC	CAAACATATTTTGAACACACTGGGCTTA	GCCTGGATGGATACCTGAAGACT
8 PRKDC	CTTCTAAACATTACCTGACAAGGCTGA	CCTGAATGCTCTAGAAGAATGGTCAA
8 PRKDC	GAGACACCTGCAAGTGACATATGTA	GAGACAGTGTCTGCCTTCTCT
8 PRKDC	AGGCTGCATTACATGTCTGTCAATATAAA	TCCTGCGTTTCAGTTTAACTTGTTTG
8 PRKDC	CAGCTGCTTACAACTGAGGTCT	GCATTTCAGTGTTGGCTTCCTTTTA
8 PRKDC	CAAAGCAAGAATACTTTTCTGGGTCTT	GCTAGGAACTACTGGATTGGGATACA
8 PRKDC	TCGAAAACCTCAGCAACATTCCT	tttCAGTGCATCAGGGTTTAATCAGAATA
8 PRKDC	GGAAGGACCACTGGTTTAGAACA	AATTGAGAGGTAAAGTTGGGTTCCAA
8 PRKDC	CCATCCCAGGTTGTCCTTCATTA	CAACAGTGTTGTGATGCCATTGAT
8 PRKDC	GCTTCTTTTCAATGATGCGGCATA	AAAATTACCTTTTCAGTTAAAAATTAGGCCAAC
8 PRKDC	GGGTTCAGAAGTTCTCGGAGT	GCCTGTCATACTTTGAAAGTGCAAT
8 PRKDC	ACCATCAGTATTTGAAAGTCTGCACT	CGATTTGTAACAGGCTTCTTGGTATTTC
8 PRKDC	CTGGTTTGCAGGTGACATTGG	GACCAGGAGAAGCTCAGTTTGAT
8 PRKDC	ACTCCAAGTCACCTTTCAAAACCA	AGTCCAGTAAACACCAAATCGCT
8 PRKDC	TTTTCTCCAGTTCTCCTCTACAAATGTG	CCGTTTTGAAGATTGTTGAGAAATTGGA
8 PRKDC	CCCAACAGTCTGTATTTCAAGTGTAAGA	GCAGATGAAGCATTTTTCTCTGTGAAT
8 PRKDC	GTAAATGATTCAGACTTTCACTGGAGGA	AAACCGGCACATACATCTGTGTA

8 PRKDC	GCAACGCCTTTTCTGGCATAC	AACCCGAGGTGAAAATGAACCA
8 PRKDC	GCAAGGCTATAAAGTCGCTTGAAA	GACCCTGTTGACAGTACTTTAAGAGAT
8 PRKDC	TTCTCGAATACACCGACCACAAAA	CAAGGGTATTCAGTTGTCAGTGGTTATA
8 PRKDC	ttCAGCAGAAAACAAAAGTGTTGAAGT	TTTACCAGTGAGATAGGAACAAAGCAAA
8 PRKDC	CTTCTGCTAATAATGCACTCTGAGTGA	AGTGTTCATTGATTTGGCCTTTTTGATT
8 PRKDC	GGCCTGAAATTCCAGGACAACA	GACGTCCAGGTTATGGCTCA
8 PRKDC	GCCTGAACACTGCACATTGAA	TCATAGCGAATGCTCTCAGCAG
8 PRKDC	TGTCCTCCTCGATTTCAGCAAG	CCAACAGATGAGAACAACGTGTCA
8 PRKDC	ATCAGATTCACACAAACATCAGGAAGA	TGGCTGACGAAGTGTTTTTATCAAAC
8 PRKDC	GCAACCTCCATCAAATTTTAAAGACACA	GCAACACTGGAAGAAGTGTGATTC
8 PRKDC	GGGAATCTTTGGCCCACCAT	CTTCACTCACAACGAAATTGTCTCAG
8 PRKDC	gCTCTGCTGACCACTGAATTAGAC	CCCACCCATGTACCAGCTCTA
8 PRKDC	GCACAGGAAACGTCCGCTTA	GCTGACCTCCTGGTCTGTTTT
8 PRKDC	CCATGTTAACTGCAATGATACCCAATT	GTCGACACAAATCCATTGAACTCTTTTAT
8 PRKDC	CATACCTGGCAATAAAGGAACGAATTT	AGGGACTAATGACAGTATGCTGTCT
8 PRKDC	TGCTGACATAATGGAACACTAGCTTT	CCTCCCTCCTGGAGCTGT
8 PRKDC	GCGTCATGTAAGCATCAATCACC	GCTTCAGTCATGCTCATGGGTTT
8 PRKDC	TCAAGAGTCTCAACACGGGAAAC	GTTGCTTCAAGACTTCAATCGTTTTCTT
8 PRKDC	GTATTACCTGCATAAAACTCTGAATGCC	GACCAGTCCCTGCTGACATTTATT
8 PRKDC	GGTGGAAAGAAAGAGAAGGTGGT	GAGGGACCCAATAATTGCAAAACAG
8 PRKDC	TTTCAAAATTCCAGAAAACAAGCTGCTA	GATGTGTTTGCAACAGTTTGTATTTTCC
8 PRKDC	TCCCCGTGCATAGCTTTGTC	TCAAATTGGCCTTCTGTAGTTACAAGT
8 PRKDC	GGGACTCACAACAGCGATGAA	AGGTGGCAGTTAAAATGAAGCAGT
8 PRKDC	CAAGAGGCCAAAAGTTCATCTTTGT	CAATAAAGGCTACGTGCCAATTTTTGTA
8 PRKDC	GGATTTCCCAGTTTGCCATGGA	TGTCTTACTTTGTCCTTGTTTTCCCATAA
8 PRKDC	TGTAACGCACACAGAACACTGA	GCTTTATTGTTTTCTTAGGTGAGGTTTCC
8 PRKDC	CCACAAAACTGAAATGCGTATGGT	ATGCATTGGAATTATCTCAAAGCCCTA
8 PRKDC	ACAACTAATCGATGGGAGTAAGAGGA	GGCAACTGTATGAGCCACTAGTTATG
8 PRKDC	GTTCCCAACAGTGCCTACACTAA	GTGACGACCGTGTTTATCAGATG
8 PRKDC	CTTGTTGTTAGTGAACCAGTGAATCAG	ATGGAACCCTTTACAGCGTAACAT
8 PRKDC	AACAGACTGGAGGAAGCTTGG	TGCAAGGTTATAAACGCAAAAGATGTTG
8 PRKDC	TGAATGAGCTCAACGTACATGAAGT	TCACATGGCTTAACATGACACCTTAAAT
8 PRKDC	cacacCAGCAATGTAGCAACA	CCACTATTCTTTCATGGACAGGCTT

8 PRKDC	GCATCAATCCTTACTGTTGGAGTGG	CTCTTGTAGGTCTTTCGCCCTTA
8 PRKDC	GAGGGAAGCTCTTGGTCACTTT	GCTTAACCTACATTTCCACTTTTGTGT
8 PRKDC	GGGCTAAGCCAGTGCTTC	GCTTACCTCGCTTTTGGCTTT
8 PRKDC	AAAGCCTCCCTGTAAATAACTGTGT	GAAGAGATATGCTGTGCCCTCA
8 PRKDC	CCTGAGGGCACAGCATATCT	GCAATTCGTCCTCAGGTAGGATTAAT AT
8 PRKDC	CTCCAGAACGACTCGGGAA	GGCTGCAGGAGACCTTGT
8 PRKDC	CTGCACACACTAACGCGTTT	TGAAGCCAGATTGAAGTTTCCTAGAT TAC
8 PRKDC	CTCCTCTGGATACCGTTCTATAATCTG AA	TGCTTTCTTCTTTGGTGTCCTTGATAG
8 PRKDC	CAGGTAAGATGAGTGGGAAAAGCA	GTGCATGATGATGTGCTGGAG
8 PRKDC	GATTGAGCTCGTCCATCTCCAG	TTATATGCTGTTGCCCCAGAAAGA
8 RECQL4	GCCCACCCCAGTTCACATA	GCTGACACCTGAGGCACTG
8 RECQL4	ATCAATGCAGGCAAAAGCAACT	GCCTCTGATCTTGCTGCCTTC
8 RECQL4	CCGAATCTGAAGGCAGCAAGAT	TGCATACACTCGGGCATGAC
8 RECQL4	AAGGCCTGTTGCTTGGAACATA	CAGTCACAACCAAGGAGTCTTGT
8 RECQL4	GATCGAACTGCTCGTTCAGGAA	GCCCAGTGACCCTCCTATGT
8 RECQL4	CCTGCAGGGTCCCCAGA	ACCTGGCTGTGGCTGAAG
8 RECQL4	TGCAGGTTGGTGGGAACTG	GGCGTGCACTGCTTCCT
8 RECQL4	TGTGGCTGTGGCTGTGAG	CGTCTGCAAGGTGAGCCATA
8 RECQL4	TGCCGGAAAGCATGTCAGAT	GCTCCGTGGAGTTTGACATGG
8 RECQL4	CATGGAGTCCACCAGCTTGA	TGTTGGCCACATGTCCCTTTT
8 RECQL4	TCTACCCGACATCCCCCAAT	GTGTCTTGGCTCCACCGTAG
8 RECQL4	GATGAGCTGCCTGGCCTTA	CAGGCTCAAGGACCTGCTC
8 RECQL4	TGCCCTTCCTCTTCCTCAAAGTA	CCGCAGTGATCAGCTCTGAC
8 RECQL4	GATTCTCCAACCTCGTCTCCAA	CCAGCCATCGAGACTTTGCT
8 RECQL4	TGCAGCTCCAGGTAGCAC	GCTTACCGTACAGGCTTTGGA
8 RECQL4	GGACCTAGCGTGGACTCACT	CATCTGAATCGGGCTGCGA
8 RECQL4	CCTGGCGTAGACTGTGGAC	GCTTCTGCCGCCACAAC
8 RECQL4	TCCTACGGTGGAGCCAAGA	GTTCTCCAGCAGGGCTGT
8 RECQL4	CACCGATGCCGTGGAAGAT	GAGCTCAGAGGAAGCCTGATG
8 RECQL4	CCGTGGTACGCTTCAGAGT	CCTTTACAGGACGACGTGGAG
8 RECQL4	AGAGTGCGGTATTCCCGGTA	CTGACGCGTTCCCTTTACAG
8 RECQL4	CTCGAGCACTGGCAGTGT	TGGACCGGCCAGATGTG
8 RECQL4	CCTGCACGTAGCTCTCGAA	CATGTGCAGCCGGGAAC
8 RECQL4	GCATGAAGGCTCGCTGTACC	ACATGGTCCCATCCCACTGA
8 RECQL4	CCCGGCCCTTCTTCACTT	CTCCGCAGCTCCGAGTC
8 RECQL4	AGGAAGTCACATATCTGGTCCTTCT	CCTGCATCTGACATGCTTTCC
8 RECQL4	ACTGCGGGAGGGTGGAT	GGGACTTGACCGCTGAGG
8 RECQL4	GGAGTCAGCAGCAGGGTT	CCGGCTGAGGTGTTCCA
8 RECQL4	AGGATCCGCATGACTGCAC	GGGTGACTCAAGTCATGGTGAT
8 RECQL4	CAGTGGGATGGGACCATGTG	ACCTCGATTCCATTATCATTTACTGCA A
8 RECQL4	GTGCAGGCAGGTTCGGA	GCTCCTCATCAGGCACTGT
8 RECQL4	GACAGATTCCCGTTGCTTCCT	CCTGAGGGACTAGGATGGACAT
8 RECQL4	GGTCACTGGGCGGGAAATAC	GCAATTACGTACGGCTCAACAT

8 RECQL4	CCGACTCACCAGGGATCAGAA	CTCCCTAGATCCTGGCTGGTTA
8 RECQL4	TCTGGGACCTCACTGTGACAT	CTGTCCCCTCCTTTGCAGAA
8 RECQL4	CGCACGTAGTGTTTCTGCTT	CATCGAACCCCAGGTACCAC
8 RECQL4	CCTTCCCAGCCCTAGCTT	GCTGGACCCCCATCGGA
8 RECQL4	AGGGTCTTCCTCAACTGCTACA	GCCTTCCAAGAAGTCAGCATCC
8 RECQL4	GTGGAGGCTCATCACTGACTTT	CCTCCTGCTGTTCTGTTGTTCC
8 RECQL4	CTCGTTCCATCTCCGCTTCTC	GATCTAGGCTCAGAGGAATCACAAC
8 RECQL4	TGCTGGTTCTTGGCTGTGT	CTGGTGCTGCCTACAGGT
8 RECQL4	GCTGGTAGCACAGGGACTTG	CAGTCATGCGGATCCTGTCT
8 RECQL4	CTGTGGCTTACCCCAGGTT	GGCCTGTGCCCAAGTACC
8 RECQL4	GACACAGATGTTGATCACCATGACT	GGTTCCTTCACCACAACCTGT
8 RECQL4	CTTGGTGGCTAAGCTGCTCA	GCTGTGAAGAGGCTGGTACA
8 RECQL4	GTGCAGGCTGGGAACAC	CACCCCACCCTCATGAAAGTT
8 RECQL4	GTAGAGTGGCAGCACGGT	TTGTGGCCAGTGGTTGTCTT
8 RUNX1T1	CTCTTCCTGCCCACATCAGAT	TGTGTGGTACGCTGGATTCAAG
8 RUNX1T1	GAGCCTCGCGGTCGAATAAT	TTTATTTGCAGCTCGGAGCCT
8 RUNX1T1	CTGAAACTCAAAAGCCTGAAATGACT	TGGATGTGAAGACGCAATCTAGG
8 RUNX1T1	TGGCATTGTTGGAGGAGTCAG	TGAAAAACTGAGGTGCTTAAGGAGTAAA
8 RUNX1T1	cctcccCTGTTCACGGAGAT	GCTTTTACTTCTGATAAGCTCCAATGTG
8 RUNX1T1	CCGCGGCTTTGTATTCATTAAACA	CTGATGCACGTTGGCTTCTC
8 RUNX1T1	GGCTTCAGCGTCCAGTCAAT	GCTATCTCCCATCACCTCTGCT
8 RUNX1T1	TCGGTGAGTCCTGTCTGGAT	AACATGTTATCTGTCGCTCCTCTTAG
8 RUNX1T1	GGTTTCGCGTTGGTTGTGTT	GCAGTCAGCTCCTCTGTCAC
8 RUNX1T1	TGCTGGTGGTGTGTCCATC	GCCCGATACTGTGGCTCATTTT
8 RUNX1T1	GCTTCTCCCAGTCTTTGTGCTG	TAACAGTGTATTAACTTGGGATCTTGCT
8 RUNX1T1	AACTATCTTGTTTATTTGGAGCTTCCCA	GAAAGCCCACGACATGATCAC
8 RUNX1T1	CCATCTTGGCCCTCTCTGTT	CTTGTCTGGTTTGGCCTGACT
8 RUNX1T1	CAAAATACTTGCGAGAACGGTGT	TGACATTTCACCCGAGATAGGAGAA
8 RUNX1T1	GAACGAGGGTGCGAACTCT	GCAATGGGCCTTCCTCTTCTTC
8 RUNX1T1	TGTTGATTAGCCAGAGAGGAGGA	ACTTCCTTCTGATTTTGTGCTCTTGA
8 RUNX1T1	TCACATCACTACATTAACAACTGCACA	ACCCCCTTCCCATCCTTTTCATA
8 RUNX1T1	CTCATCTGAAGAAAAGTTACCTAGTGCAA	CCGTACTAAGGCGGTGTCAAG
8 RUNX1T1	TGACATAGGCCAGCGGTTTAATT	GCACCACCTCACCTGTTGAC
8 RUNX1T1	ATTCAATTCTTCCCGGTCTGCTT	TGCTCTTCCCTATTCTCCCCTTT
8 RUNX1T1	CACATCGAGAAGCAGCTCTGA	TCATTCCTGTCATTCCCTTGCAG
8 RUNX1T1	AAAAGCACACAGTTATGACCTAGCA	TTCCATAGAACTCCACTTTGACAATTGA
8 RUNX1T1	GCTTCTTGCAGTTTGGAATGAAATTCT	TGGTTTCTGTCAGTATGTTTGCTTGTTA
8 RUNX1T1	ATTTGTCTGTTCCTTACCTTGACAATAT	AGGAAATTAGCAGAGCGATTGGT
8 RUNX1T1	GAAATAATGGTGCAAGTCTTCAAGCA	CTCCACCTCAGCATTACCGT

8 RUNX1T1	GGGCAATGGCCATATCATCCAA	CAGAGAGCCTTTGCACTCAGAA
8 RUNX1T1	CATGGTCGCTTGCTTGGATG	AGTGACTGAACTGTGCTGGTTT
8 RUNX1T1	GATAGCAGGAAAAACAATGCCTTCA	CTAACAGACAGAGAATGGGCAGAAG
8 RUNX1T1	GTCTTACATGGTCAAGATGTTTCCACT	GCTTTAGGGTTATTCAGCCTTCTCTAT T
8 UBR5	ATTCTTTTACCTCAAAACCACCCAAT	GTATTCATGGAAGATGTTGGAGCAGA
8 UBR5	TGCTATTCTCCTTACTCCAAATGTTGA C	TCAGCAGACAACATTGGATACACC
8 UBR5	GCGTATATCACACAAAGTATGACCCA AT	GTGAAAGTAACCACAGCAACCAAG
8 UBR5	TGCACAAAATACATACTGATCCAGGT	CACCCCTTCCCTTAAACTTCTTACA
8 UBR5	AATGGATACCTTTTGCGTTCTAGTTGA	TGTCTCTAATGCGGTCTCATAATGATG
8 UBR5	CAAAACTGGAAGAACATCAGAATGCT	TTCCTTTCTGTTCTTATAGACGTGCC
8 UBR5	TTCTGCGCCCACTAGCATTAT	CTGTTTTAAACCATAGTGAGTTTGTGC T
8 UBR5	AATTGATAAGCTAACCAGAGCCAGAC	TCCTTGGTCAGAATGAGAGGAA
8 UBR5	TCCAGCAGTGAAAATGGCTACA	CTGGAAATTGGGTACGATACTGTATC TT
8 UBR5	TTCTGCTTTTCCTGTAGCAAGATCA	ACAAGTGTGGCCAGAGAAGTTAAAAT AT
8 UBR5	TTTAAGATATGATGGCACCCACATAC TG	TCTCCTTCTAGCAAGTGAGGATTCT
8 UBR5	CCTCATCCACTCTTGCTCTCA	AGACAAGAGGTTTTAGAGAGTAGTCA AGT
8 UBR5	TGGAAGAGATACAATGATGCACAATT CT	CCCCTTTGAATGTATTGGAACAGG
8 UBR5	ACCTTTACCTCTAGTGACAAATCTCTT GA	ATTCCATTCTGCCTGATAAGCATGT
8 UBR5	CAGCTGTCTGCATGAGACCTA	CTGAAAGTAAGCAGGAGCCAGT
8 UBR5	CTGACATTAAGTGTTCTACGAGATCC AT	CATCAGCTGGAGCTCGAGATTC
8 UBR5	GATCCCGTCCTGTCCGAATAA	CTCCAATATTTAGAAGCCGCTGGA
8 UBR5	TTCACAGCAAAGAAGAGTACTTGAAA	AGCAAAGTGGCACAATTCGG
8 UBR5	AGGCAATGAGTGAAACAGTCCAG	TCATTCTGTCTTTCGTTTCTCCTCAAG
8 UBR5	CTTTCCATACAGCTAGCCTGTGA	CAGATGCCATGTTTTCTGAAGACATT AG
8 UBR5	CGACGAAAAGAAGGGTAACCAAAAT AG	GCTGCAGTGATGTTTGTTTCTGTTTAT A
8 UBR5	TGTGGAATAAAGTTGCTAGAACAAAA AG	ATTCTGAGTGTGGAAATGGCTTCA
8 UBR5	TGCACTCACACATATAGAAACCATGA TT	AATCTAGAGTGTATCCAAAATGCCAA CA
8 UBR5	GGATGCCTTACTTTTTCTTTTTGGATG A	ACAAATATACACCTGGACGTAGAGAA GA
8 UBR5	GCCATTTCCACACTCAGAATAACAAA A	TCTACTAGGTACACTAGTGAAAGAAC TCC
8 UBR5	GTACTTACTTGTGTGGGTGCCTT	GGCTGTACACAGAGTAAAAGTCACAT T
8 UBR5	CCCTCTCCTGGCTCATCCTTA	TGTTTTAATAGGTTGATCGGGATCGA G
8 UBR5	ACAAGGAACTGGTGTCAAAGCA	GGGATTCCTAAAGTTGGTGTCTTAAT GG

8 UBR5	TCGATACAGCAGCAGTATGTCAATAT	TCCAGCAGCTGTGAAATTGACTTA
8 UBR5	AGCTGTCATTCATATTCCAAACTGACT	TTGATGGTGGTTTTCTTTTGTTTGCT
8 UBR5	TCATACCTGTAAGTTTACTGCATCTTGG	AACGCCAGCAGTTACCTAACA
8 UBR5	CCTGGCTAAACTGCTTGCAGA	GCTCATTGTGGTTGTCCTTTTTCC
8 UBR5	CATTCAGACTGAGAGCACTCACA	GACTCTCCATGGTTTCTCTCAGG
8 UBR5	AGCCTGCCTAGAGTCTCAGAA	GTTGGTCTTCAAAAATTTACCTCTTGCT
8 UBR5	AGCCCTGAAGAAAGGACTCAGAT	GAAGTGGTTTTTGTGGAAGATGTCAAG
8 UBR5	CAAACCTTGCCAACAGGAACATT	TGGTACTTACCATTTTGTTGTTGTCTAAGA
8 UBR5	CCTTCGGTGTTCCACCAGTTT	CCTGCAGGATTGTAGGTTACTTAGAATT
8 UBR5	CTCACGGAGTCAGTTCCAATATTCA	CTAACTGTCAGAACAGCTCTGGTC
8 UBR5	GGAGAGAAGAAGGGTCAGCATCT	GAATTTGCCTATACTTCCAGGTGCTA
8 UBR5	CAACATAGGCACCATCTACTTTTAGCA	TGATCATTGAAGAAACATACAAAGAATTGG
8 UBR5	AGGACTGGCTGTTTTTCGGTTA	CTCCTCACGACAACATTCTGTTTTG
8 UBR5	CATACACAAGCATTCACCGATAAATTCC	GAGCACACTGCTCAGACTTACT
8 UBR5	CTATCCGCTCTCCTTGAAGTTCAG	CACAATCCTTTTGAAAAGATGGCTTGAT
8 UBR5	tctctccTCGGTTCCTTAATCTCTG	AGGGAAGGATAATCTTTAGCTGTCATGT
8 UBR5	AATACTAAGTTTTCAGACTTGCCTGCT	TGATGAGCCATTAGAAAGAACCACAA
8 UBR5	CACCATTGGCATGGGAGCTAT	AAAATTTTAGGAGCAAGCAGTGTTCC
8 UBR5	CATATCAGGCTGCCGCCTA	TGTACACGTGGTTTGTTAATGTTTTGTT
8 UBR5	ACTCCCTTTAAAGACCTTGCTATAGTTCT	GCCACAGATGTGATGGAAATCGAAATAT
8 UBR5	GCAAACTGATACACAAGCATGCAAA	GCCTGTCGACAATATCTAATGAATCTTGA
8 UBR5	ATGAATGTCTGCAGCATCTGTAGATT	TTTTCCCCTAATTTGTCTGAGCACA
8 UBR5	CAACCATTACAAACTTAAAAGGCCATCA	AGCTTCTGCAGTTCAAGCGT
8 UBR5	GGTGTTTTGTGAATTAAAATGCCCAGTA	GGATCTTCCACCTATTAGTAGTCTTGGA
8 UBR5	GCCATGATGATAACAGCAGCTTTCT	GTACCATCTACCCAATGGCCAA
8 UBR5	TGGTACCATTTCCATCTCGAAGAATA	TTAGCACATTTGAATACTAGGTTTCTTATTACT
8 UBR5	TGGAATATAGGATATGCCAGCTGAAGA	CTTTTGTGTCATGCAGGTTGTCA
8 UBR5	CTACTGCTGGCACTAAGACTTGA	GTTCTACAGGACTGGAATGCCTT
8 UBR5	CTGCGACCCAAACATAATCATAGATTTC	GACACTTGTGGGAAGCAATATGTTTTAT
8 UBR5	GCTCTAAATCATGATCTGGCATATCTGA	ACCTCGAATCAGGGAAGATCG
8 UBR5	CCTTTCATGACCTGCTCTTTTTGTT	TTTGGGTAGACATGAAGCTGGAG
8 UBR5	CCTGGTGGTCACTGGATGAAG	TGTGTAGATATGGTAGTGAGCTCTGATTT

8 UBR5	CCAAAGCTGAAAACTGTAAGTCTAGTGA	CAACCCTGATGACTCTCCTTTATATGTTT
8 UBR5	GTCCATGTAAAACTGCAAGTGTCATTAC	GTCCTTTCCTGAAAGATGAACTGCTA
8 UBR5	CATGTACACCACCTGAGTGCTTA	CATGCAGGGCAGTGAAGAATTATTT
8 UBR5	TGGTGGTAGTGGTTCCACTGA	CAACGTAGCAGAGTCACTGATTG
8 UBR5	GCAATCCCCATTCTGACAGGAA	TCTAGCAACTTTATTCCACAGCCAA
8 UBR5	GGAATACACGCTTGCATTTTCCAA	CATCCAAAAAGAAAAAGTAAGGCATCCT
8 UBR5	TGCTGGGACAAGTTGTCCTTAATAAAT	GCCAAATGCTAGAAAGGAGGATCTT
8 UBR5	ATGAAGAATAAAGACCCTGACTTGGAC	GCCACATCTGTTGCAGGTGAA
8 UBR5	TTTCTGTGATGCTTTAAGTTGAGGGTTA	GAGTTTGCAGGATTGGTTTTTCAGT
8 UBR5	CCAATTCCAATCTGTCTGGCTGT	GGGCACAGAATCTCTTTGTTGAATAAAT
8 UBR5	TGAAGAATTTCATACCGTCTGCCTTC	AGCTCAATTACGTTATGGTTCTGCAT
8 UBR5	CAGGATCACCAGCAGATGCTA	CTGCTGTATCGAATTTAGCTCTATTTGGA
8 UBR5	ACGCTGTACTGAACAATAAAAACAAAGG	CGTGCTGCCAGAAGAAACTGA
8 UBR5	AGATGGCCCTGGTTTTGAACTC	GGTTCTTCCAACAAAAATGTCTTATGCT
8 UBR5	GTTCATTACATTTTTCAGATTGGCAGCA	CCATTGAAGCTCCAGAAAGGGAAA
8 UBR5	TTACCACACATTAAGACACAAATAGCCT	CAGTCACTCCACAGAATGTATATGAGT
8 UBR5	ACCAACATTCTGTGTTCTGCGTATT	TGACAAAATTGCCTTTAAATAGCACTGG
8 UBR5	CGTGACACATACACGTTACTGGA	GTATTTCTTCAATGCCGAACATCACT
8 UBR5	CCATCACTTACCTGGGTACCAAC	GGCTGTTTTACTGCTTATTTGACCAATT
8 UBR5	CCAGTTAAGAGGTAACTAAGTACACAGT	GAAGACAGCTTTCCATCGACACTA
8 UBR5	TCCCTTCAGAGGCTGGTCTAAA	ACTGAACAACCTATATGCTCTGGTTTTC
8 UBR5	CCAAATACCTTATCAGGCCACCA	GGCTTGATGGAGCCTCATTTGATAAT
8 UBR5	CCTTGCTGGTAGAACCCCTTTC	GGTGTGACCCAATTGCATACC
8 UBR5	AGTAAAAGCTAAGCTAAGCTAAATCTTCACAT	CGAAAGGCGAATGCTCATTTTATATTGA
8 UBR5	GGAGAACCACACTGTCACATAACAATT	GTGTCTTCTTCATTGTTGTTTTGTACCA
8 UBR5	GCAAACGCATATAGTAGTCTCAAGCTA	AAGAACAGGATGATATTGTTTCAGCAGA
8 UBR5	ACCACAATTAAAATCACCTCTTCCACA	CCCACTCATAAGCTTCTGATTCTTTACA
8 UBR5	CCAAACCCTATAAACAGGCCTTAAGA	GCGGTGAAGTCAATGTGCAAA
8 UBR5	TCATTGAAAGAGGTAAAACTGATCAGCA	AAAGGTCTACTAGATGTGCTTCCAAAAA
8 UBR5	GCCTAAAATCTTCTGCCGTTAAATCTTCT	GGCTGCTAAAATTATGTCTTGTGCTT
8 UBR5	TTTCTCAAATAACCTGCATACCGTCTT	GGATTTAGGTTGAGGTGGTGGAG
8 UBR5	CCCGTGTTCTTCCTGTTCATCAT	CAAATTCTCTATAGTTAGTGGTCTTCGCA

8 UBR5	ACAGTTTCTTCAGAAATCCTGCTTTCAT	TGTTTTTAGGGAAAATGGAGCTGATAGT
8 UBR5	AGATCCAGGATACTATCAGCTCCATTT	CAGGTGCATTGGATATTTATTGACTCC
8 UBR5	TAAGGCTTGAGACACTTTGTATTTATGT	GTCTGCAAATAGCATAAGAGCAACTG
8 UBR5	TGCAGACAGGAGGACTATCTTTTCA	GCCTTACAGAAATGCCCAGGTATTTTAT
8 UBR5	CACCAATTCATGTACACGACAGGTA	AAGTAATGGCCCAGGTAATCGG
8 UBR5	CACATTACCTTCGGCTACTTGATGAT	AGCGTTCTGAAAGAAATACATTTGCAG
8 UBR5	CCTCAACAGCAGAAAGCCTTT	TAGAGATTAGTGTTTAGTGGACTATACATACTC
8 UBR5	ATTCTTGGTCTTAATGGCGAGTAACAA	CCTCAATCACAATAAGACCACCAGA
8 UBR5	GTATTTGCAGTAGGAAGATGTTGGTCA	GGCTTTGTGTATATACTGCCAGAGTTAA
8 UBR5	GGGTATGACAGCATTAACTTCCAT	ATTCCTCTAAACAGATTCTCAAACAGAAA
8 UBR5	CGTGGATGATGTAATGAAGGATTCTTTG	GGCTGTCAGCAGTAAAGGAGAA
8 UBR5	GACAGCCAGAAGTTCAGAATACAGA	TGATGAAGTGATTAAAGTGACAGTGTTCA
8 UBR5	GATCCTCCTTTCTAGCATTTGGCT	TTTGAAGTGCCTCTGGCTGAA
8 UBR5	CCTGCAACAGATGTGGCTGAT	GAAACTGGCCAAAACCATCCATT
8 UBR5	CATGGAGTCTGAACGTCGGAAA	GTTAAAGGTCTGTTACCCTAATCCAGT
8 UBR5	cacccagcGTACATACATTTTAAAACA	ATTTTATACTCCAAGGCCTGGCAA
8 UBR5	GTTCTTTTACCTGCCAATGTTTCTGAAA	CAGATGATGGTGATGACAATGCC
8 UBR5	TCCTCTTTTCCCAGGTTGGTAAAAC	GGGCTTACTCTCTGATATTCTCTGTTA
8 UBR5	GGACAGAAGAGAATCTATCCAAACTGAA	GGGATGCAAGAGGGATGACC
8 UBR5	CGGCCACTTACAGCTGACATAA	TTGTTTTATCATGAACATTTATAATCAGGAAGG
8 UBR5	AAGTAGATTAGCAGATTTTCTGTGCACT	CCTCTGACTTTTGCTCCTGTTCATATTA
8 UBR5	GCTCTCCCCTGCAAAACAGAAT	TTGATCTACTTTATCGCCTGCTCAC
8 UBR5	TGGCAGAGTAACCAGATTAGTAGCA	CTCAAACGGACATCACCAACAG
8 UBR5	ACATTTCTCCCAACAATCACAGTAGG	TCTCTGGTTTCAGTGAGTTATACCTTCA
8 UBR5	TGTACACACACGTTCATCTCTTTCA	GCATTTGCAATTGACCTGTGTAAAGAAG
8 UBR5	GCTTTACCTGTCCTCCACCTT	TTTTTAGGTCAATTGGCATGATTTTGCT
8 UBR5	TGAGAAAAACATGACGGTGATCCA	ACAAGGCAAATCCAGAAGTGTCATTATT
8 UBR5	TCACATCAAGATTTGTTCTCTGAAGTTCT	cacatccTGTCCTAGAAAGCTGAT
8 UBR5	AAGCAAAGAACAGTCTCCTTGAGAT	CAACCGGTACTGGTCAAGGAC
8 UBR5	CCTGGAATAGTGGAGGTGCTTG	TTAAGGCCAGTTAAGAGAACATGTTAGG
8 UBR5	GACCCGCCAGCCCTATT	GCGGCTGTGGGTTTTGG
8 UBR5	CGTAAGTAACCTCATGCTTTCTGAAGT	TCACTGTGTTGTTGTACGGAATGT

8 WRN	TGAAGTCAAATAATGAAGTCCCAAGTGA	GGCTTTTTGTACTTGTTTGTCCTCAA
8 WRN	GTCTTGGGTAGAATCATCTTGTCTCATT	GGAAGAGAAAGAAAGACTTTTAGAATGTCCA
8 WRN	GGGATGAAATAGAAAGTCAATGAGGAGAA	ACCTGAGTCTCCTGCTCTTGT
8 WRN	ACAGTTCCTCACAGCCTGTTATTTC	TGCTGGCAAAATACAGAATTTTAAGCAT
8 WRN	GATGTTTTTAATCGACAGGCACCTT	CATACTGTCTCCTACATCTGCTAGAATG
8 WRN	AGGCACCTTCTTACTGAGATACGTAAT	AGCCAAATTGTTGTCTTAAAAGAGCATC
8 WRN	GAAGTAGGACATAAATCCATCATACTTGACA	CCTTCAATTCCTACACCTGCCTTTT
8 WRN	CCCAGGGATTAAAAATGTTGCTTGAA	CAACATCTGTCAACTCCACAAAATTCT
8 WRN	CAGTGGAAACTTCTACGTGACTTTGATA	AAATCTAACATTCAGATAGAAAGTACATTCTGT
8 WRN	TGGACTCTGCAAATAGGACATTTCAA	CACCCAAAAGAATAGTCAATGAACAACA
8 WRN	GGATGGGTGTGTATTCAGGAACT	AACCACACAGGTAATCGTCTCTTTC
8 WRN	TCCTACAGACTTCATCTGCAGAGA	CATAATTGTTCTGGTAATTGCCAGCTT
8 WRN	CCTTCCACCTTCCTTTCTACAGAAT	CCATGTGGATAAGGTACGTGTCA
8 WRN	TGTTAGTTCCTGAAAACATTGACACGTA	AGAACCGGGAAAACATCTCCTTTT
8 WRN	GACTTCAACCTTCATGTGATGTCAAC	GGATATGCAAATTGAACAAACTGACACT
8 WRN	AAGGTATCTTGATTTTAAGTTGTAATTCCTTGA	ACCTGAACAGTATTCTGGAGTTACGTAT
8 WRN	tgttttACAGAGGTAAATACCGGATTGT	CCAGTGTCCATCCATAGGTTTAACTT
8 WRN	AGTGGCTAAATGAATATCTCTGCTTTGT	GGTTTTCCAATTTACTGAAAGCATGAGG
8 WRN	GTGATGGATCTGGCTAAGCATCTT	TCAAGCAAATTTCTATGAGTCAGCCTTA
8 WRN	GTTATTTGATGTGAACTTTGTGCCTGTT	GACAGGAAAGAGCAATCACTAGCA
8 WRN	GTTAAAAAGCTTCACAGTTTGTCCTTGT	GGCCTCAGTTCAGTCTCAATGTTAG
8 WRN	TCACTGGATCCATTGTGTATAGTTACGA	CACACCTCACCTGTAAAACTCAGTATAT
8 WRN	ACTGAGGAGGATGATAATTGGGTCTA	CCCATGTTTCATCTTCAACGTGAATTAA
8 WRN	GGGAGTACAACAGAAACAAATTAGAGAAC	GTTGTCTTCTACTCCATCTTCAAATCCA
8 WRN	CTTGGAAATAAAGTGGAACGAAAAGAAGA	TCCTGAGACTGCTGTTCCAAAATT
8 WRN	GGAAAGAGCTTGTTTGATGTCGTTAGATA	GTGCTTCCTCTTTATTTAGTACCTCAGT
8 WRN	TCTGTAGACAGACCTCTTTTCAAGTACA	TCCCTGTAACATGAAAGTCACACTTAC
8 WRN	TCCAAAGAATCAATAGACAAGTCTGTGTTT	CCAAATAAACTGTGCCTGCGATAT
8 WRN	CTCAGCGTCTTGCCGATCA	AGAAACTTCTACCAAGAATCCCTCAGT
8 WRN	GGCTTTTTCCCGTCAGCTGAT	AGGATCTTTTAATGTGGAGTGGGAAAC
8 WRN	CTCACTTTAGCATGAGTCTATCAGATGG	AGTAACATTTGCTCTCAGAAACACACA

8 WRN	CTTGGCAAAGTTGCACTAATTCAGT	AGAACGGCCAGCTAATAAAAGAACTTA
8 WRN	TCACACTGAGCATTTACTACCTGAATG	CAGCTTTCACCGCTTGGGATA
8 WRN	CTCATGACAATTGGCATGCACT	CAAGTCATTAAGGGTGCAGAGCTA
8 WRN	TGTTTAAATGCAGTCTAACTTGGAGAAGT	CCCAACATCTGACAGTTCTTTCTCTTTA
8 WRN	CATCTTTCTGAGAATGGAGTATCATGATTCA	TGGTACTTGATTATAACAATGCTCTTTGGT
8 WRN	AACTGTATCTTCGGGCACCAAA	ATGCCATTTTCTCTTTAATTGGAAAGG
8 WRN	ACCTGAAAACAGGAACTGATTTTACTGT	CTTGTCTTTCAGGAGCTGTTTACCT
8 WRN	ACCCATGGTAGCTGTCACTGTAT	GCCAAAGTACATCTTGAGGCAAGT
8 WRN	CCTTAACAGTCTGGTTAAACACCTCTT	ACATAAGCATCAGTGGCTGCAT
8 WRN	TCTCACTGAGGACCAGAAACTGT	AAATATTTTGGTAGAGTTTCTGCCACTA
8 WRN	AGCACCCAATGAAGAGCAAGTT	CAGGTTATTTGCCTTTTCCCTTGTT
8 WRN	TTGGTGAAAAAGATACGACACTGTCA	GTAGGAAGACCCAGATTTCTTTCCATT
8 WRN	CGGTAGAACCAACTCATTCTAAATGCTT	TGGTAGGTATCACAGGTGAGTCAT
8 WRN	ACTTTGTGCCAGGGACTTAAGTT	GACTTGGCGAATGTCAGCTTTATT
8 WRN	ACCATAGCTTTTGGAATGGGCATT	GTGACAAGAACTTTGAAGTCCATCAC
8 WRN	CAGGAGATTGGTAGAGCTGGTC	GGATACTGGAAATAGCCTCAAACACTTT
8 WRN	GTGTAGGAGTCTGCCTGTTTGA	TCTTTGGACACAATTCTTCATTAGCTTGA
8 WRN	ACAGAATCTCAGAGCCTCATCCT	ACTTGTGAGAGGCCTATAAACTGGTATA
8 WRN	CCAAACGGGTCTGAAGCATGT	TCCACAGGATAGATTCAGTTTCCTAAGT
8 WRN	ttCAAGGATATGGAAAGAGTTTGTGCT	AAAGAGGGTGATATTGTGGCAATGATAA
8 WRN	GAAAAATGACACAACAAGTTACAGGTGA	AGCAACGGAGAGCAATGATGATT
8 WRN	TTTACAGGTATCACGCTCATTGCT	GACATCAGACTTGGCAAAGCTTAC
8 WRN	TCCAACATCCCAGCTTGCTT	ACATAAATTACTCACAACTTcctcagca
8 WRN	ACATGTTGGGAATGAATGAGCTCT	CCAGTACAGGTGATCTGAGGATTTC
8 WRN	GAAGACATTGTACGTTGCTTAAATCTGA	GTTTTGACAAGAAATGGCTGCAGA
8 WRN	CCTTCAGGATCTGCAGCCATT	CCAAAACTAGGTGGAATTTGAAATATGTT
8 WRN	CATGCCTTTGCTAAGCTTTCTTCAA	CTAAGATGTCCACAGCAGACAAAAG
8 WRN	GGACTTTGGTCCACAAGCATTTAAG	ACTGCAAACCTCTGAAGAAAAGTGTAAT
8 WRN	GAGAGAATCATTAGGAAGCTGAACATCA	CCTTCAGAAACACCATCAATCCTTTT
8 WRN	AGACCAACTACGGTTGAAAACGTA	AGCTCCTGCAAACCACAGTATTTT
8 WRN	AGGAAAAATGTGGTATCTGAAGCTCTAAAA	GCATGTTTCTGCCTAGCTTCTAC

8 WRN	CAATAAAATGGATGTTCCCCCAGCTA	GACAGTGGAAGCTATGGTCATATTAT CA
8 WRN	TCCATGTTTGGGTGCTTTGTGA	GCAAACCTTTGCACAGTATCATCC
8 WRN	GAGATTTTGGATGATACTGTGCAAAG G	CAGGACTGCTCAGACATTCAAGAA
8 WRN	AGAGATAATGTTGCTGTCATGGCAA	CATCTGTTCTTTGTTCACTCTCTTCTA T
9 ABL1	CTGGCGCGGGAAAATGT	CGGCTACTCAGCCCAACC
9 ABL1	AAGCTGATATGTCTGATTTGGTTCCTT	TGGTACCAGGAGTGTTTCTCCA
9 ABL1	ACATCACGCCAGTCAACAGTC	TGGTACACCCTCCCTTCGTATC
9 ABL1	CCAGAGGTCCATCTCGCTGA	CTTCTTGGTTGAGCTTTCTTGCAAT
9 ABL1	CCCCTTTCTCTTCCAGAAGCC	GTTATCTCCACTGGCCACAAAATCAT A
9 ABL1	CCCAACCTTTTCGTTGCACTG	TCAAACCGCAATTCCCAGATTTCTAT
9 ABL1	CAGGACGAGTATGCGCTGAA	AGGGTGTTTGATCTCTTTCATGACTG
9 ABL1	GTGGAAGAGTTCTTGAAAGAAGCTG	CAACGAGGTTTTGTGCAGTGA
9 ABL1	AGCAGTGGTGGATTTGTGAAGT	GCATGGGCTGTGTAGGTGTC
9 ABL1	GCCTGAGCAGGTTGATGACAG	CCTGTAGTCAGGAAGGACCACTT
9 ABL1	CAGTACTGATGGCTGCTGGAT	CAGTGGTGTCTCTGTGCTCTG
9 ABL1	AGGACCTCCAGGAGAGCTG	AGCTTCCCGTTGGCCTATG
9 ABL1	CCTTCTGAGGTCTGCTGCAAA	GGGACATGCCATAGGTAGCAAT
9 ABL1	GCATTTGGAGTATTGCTTTGGGAA	AACAGGCTGAGGAAGGCTTAC
9 ABL1	GGACTGAGGAGCAGAGTCAGAA	CGTAGGTCATGAACTCAGTGATGA
9 ABL1	CGGGAGCCCCCGTTCTATA	CCCCTACCTGTGGATGAAGTTTT
9 ABL1	GCCATGGAGTACCTGGAGAAGA	CCAGCACTGAGGTTAGAAGCTG
9 ABL1	ATTTTGCTGTGTAGTGAATTAAGGCT	TCGGCCACCGTTGAATGAT
9 ABL1	CCTGGCCGAGTTGGTTCATC	TGATGTCCGTGCGTTCCATC
9 ABL1	GTGTCCCCCAACTACGACAAG	CAGCCTACCTTCAAGGTCTTCA
9 ABL1	GGAAGAAATACAGCCTGACGGT	GGAGGTAGACTTCCAGGCAGAT
9 ABL1	GCCGAGGAGTCCAGAGTGA	GGGCTTTCCTCCAGCCTT
9 ABL1	CCTCCTGCGTTCCCCAT	GCCGGCTTCTCACTTTTGTG
9 ABL1	GGGAAATTGTCCAGGCTCAAAC	CTTGGCAGCGTCACTGTTC
9 ABL1	TGCCTCGCCATCTCTAGGAA	TCCAGTTTGTTGATGGCCTCTC
9 ABL1	gagttGTCAGCTCTTCCCCTT	GCGCTGAACAAGTTGGTCTT
9 ABL1	AAAAGCCACGAGTCTGGTTGAT	GGATGCTGATGGCAACGTG
9 ABL1	AGACAGTTTGACTCGTCCACATTT	AAGACCTCATCAGCAGCTTCC
9 ABL1	CCCAGGCTGGTGAAAAAGAATGA	CTTTCCAGCTTCTTCCTTGTGG
9 ABL1	CCACTCCAAAGCCACAGTCC	AGAGACACTCGGGTTGATATGAGA
9 ABL1	CGTCTTCCACCGCCTTCA	CTGGCCATCTGCTCGGA
9 ABL1	CGCCCAACCTGACTCCAAAA	TGAGCCTGCTTTGCTGGT
9 ABL1	TGGAAAGGGCAGTGCCTTAG	CGACTCAGAGGAGTGCTTGTG
9 ABL1	CGCCTTCTCCCCAAAGACAAA	CTGATGTCTCGGCCCTCTTC
9 ABL1	CAAACGCAGCAGCTCCTTC	GCCCCATTGCTGGGCTTT

9	ABL1	CAAATGAGGAACAAGTTTGCCTTCC	GCTGCTACCTCTGCACTATGTC
9	ABL1	CTTTCACCCCCTTGGACACA	CTGGTCAGCGTGCTGGA
9	ABL1	TCTCCCCACCTGTGGAAGAA	TGACTGACCTCCACTCCGT
9	ABL1	CTCAGTTCGGTGAAGGAAATCAGT	TCACTAGTCCCTTGTCAGCCA
9	ABL1	CTTGCCTGCCCTGCATTTTATC	GAGGAAAACCATGAGCAGAAATTTTGAA
9	ABL1	GCTGACCGTTCTGGAAGATCTTG	GATTCCTTCTTCCCTGCTCCTTT
9	ABL1	GATCTCATGGATGATCTGACTTGGG	CTTTCACCCCACTGCAGGTA
9	BRD3	CAGCGCCCCAATCCTTACT	AATTATTATTGGAGTGCAAGCGAATG
9	BRD3	AACATGGTGTTGAAGTCCTGCATA	AATCTTCTGGTAATGCCTCTGAAAATCT
9	BRD3	ACATTCACCTGGGCGCTTA	TGCTGATGTCCGGCTGATG
9	BRD3	GGGATTGTATTTGTAGCAATTCGAGAA	GTGTGGCAGAGAGTCCTGATG
9	BRD3	GGGAAACAGCTCAGATGGCTAA	GGAAGTTGAATTATTACCCCCTGCTC
9	BRD3	CTTCCGACCTTTGCCCTTTG	CCTGGACTCTCTGTGTCTTCAG
9	BRD3	GCAAGCGAGACCCTGCTC	GGAGCTGCACGACTACCA
9	BRD3	CCATCGGGTGCTTGATGATGT	GCAAGAAGGGCAAGCTGTC
9	BRD3	CTGAGGATGCTGTCGCAGTA	CCCGCCGTTGTCAGACC
9	BRD3	CCACCACTTTGGCCTGCTT	GCGACATGCGTCTTCGG
9	BRD3	GCTCCACGCAGGCAACTTA	CCGACGAGATAGAAATTGACTTTGAGAC
9	BRD3	CGCAAAGTGGTGGGTTTCAGA	TGCCCATGAGCTACGATGAAAAG
9	BRD3	CCGGTTGATGTCCAGGCTAAG	GCAGGTAGACGGAGACTCAACA
9	BRD3	GCTCGATGCTCAAACCCCTA	GCTAGCTCAGGAAAAGAAGAAGGA
9	BRD3	TGACATCCTGCAGACGCTTTT	CATCGGTCTGCTCTGCAGT
9	BRD3	CCAGCAATCCCGCCTCA	CTGAGAGCAGCCGTAGCA
9	BRD3	TGAGTCCGAAGAGCTCTCCT	CGCAGGACGTGTTTGAGATG
9	BRD3	CATCTGGCATCTTGGCAAACC	TCCTAGCCCCACCTTCGTAA
9	BRD3	GCAGCAGAACGTGGCTCTT	CAAGACCAACCAGCTGCAGTA
9	BRD3	TCTTCACCACCACATTCTGCAT	TGTGACAGAAAGTGAGTGAGCTG
9	BRD3	CATCCATCTGTCCTGTTCTGTCC	GCATCTCCCTGCCCATGTC
9	BRD3	CCCACCCATCCGGACTCA	CACCCCTGTACCAACCATCA
9	BRD3	CCGACGTGACGTTTGCAG	TGTTGAACATCCCTTCCTTGTCTTG
9	BRD3	CAATCCCAGCACTGCTGCTA	CAGAAGAAGGCTCCTGCCA
9	BRD3	CTTGGCAGGAGCCTTCTTCT	TCTCAGGCCCCAGTAAACAAAC
9	BRD3	TCTTCTGCTGAGCCTGCTTG	CTGTCTCAGGCCCCAGTAAA
9	CDKN2A	CAGTTGTGGCCCTGTAGGAC	AGGTTTCTAACGCCTGTTTTCTTTCT
9	CDKN2A	CCCCTTCAGATCTTCTCAGCA	GCTTCGATTCTCCGGAAAAAGG
9	CDKN2A	CCTTCTGAAAACTCCCCAGGAA	CACCGAATAGTTACGGTCGGA
9	CDKN2A	CCGCTGCAGACCCTCTAC	GGAGCAGCATGGAGCCTT
9	CDKN2A	CTGGAAAATGAATGCTCTGAGCTT	CTGAGGAGCTGGGCCATC
9	CDKN2A	GCAGGTACCGTGCGACAT	GACCCCGCCACTCTCAC
9	CDKN2A	CACGGGTCGGGTGAGAG	ACACAAGCTTCCTTTCCGTCAT

9	CDKN2A	CCAGCCAGCTTGCGATAAC	GAGCACATGAATAAATGAGCATCCATT
9	CDKN2A	CGGACTTTTCGAGGGCCTT	GCCGCGAGTGAGGGTTT
9	CDKN2A	GCGGGATGTGAACCACGA	GCTCACCTCTGGTGCCAAA
9	CDKN2B	CAGACAGGCTTGCAGGCTTA	CGACGTTGCAGGGTACCT
9	CDKN2B	GGGAACCTGGCGTCAGT	CTTCCTGGACACGCTGGT
9	CDKN2B	CGCTCCTCGGCCAAGTC	CAGACCCTGCCACTCTCA
9	CDKN2B	CGGGCAGCATCATGCAC	GCCGGCATCTCCCATACC
9	CDKN2B	GCCCCAGCTACCTGGAT	GGCAGCGATGAGGGTCTG
9	CDKN2B	GTCGCACCTTCTCCACTAGTC	TTTACGGCCAACGGTGGATTAT
9	CDKN2B	CCAACGGAGACTCCTGTACAA	CCAACGGAGTCAACCGTTTC
9	FANCC	GCGTCTTATTCTCTGGGATGAATGA	GTTCTGATCTGACTTTGCATTGTTCAG
9	FANCC	AAGGTTCCAATTGCTCTTTTGTTTACATC	AATTGTCTGAGAAGGATCGAATGCTTTA
9	FANCC	CAGTACGTACCAGCGATGAATCTTTTA	AGGAAGAAGATTTCTCTCCCCATGT
9	FANCC	CCAGAGGCAGACTACAGCTGA	GTGACCATGATGAGAAGTCTCACAAAT
9	FANCC	AACAGTGAAGGGTATGTTTGAAAAAGT	TGGACAATCAAAACTTAACTCCTGGAT
9	FANCC	CATTTACTCTTTTTGCTGATGGCACAT	GCACTCAGATTTGATAAAGAAGTTGCTC
9	FANCC	GCATACCCAAGACCTTGAGTGAAAA	GCATTGTACATAAAAGGCACTTGCAT
9	FANCC	CAGCCAGAGACTACCACAACATT	ACATTCTCATGGTCTTCTCCTTTTACAG
9	FANCC	GCCTGGAGCAGAAATGAGTACTA	ACACAGACCTCAGAGCTCCT
9	FANCC	GTGCCTGATCAGCTGTTGTG	TTCCTAACCTCTCCCCTGTGAA
9	FANCC	GCAGATGAGGATCTAGGGAAACC	GCCCTCTGACGTATCTCTCTCC
9	FANCC	TTGACAATGCTCTTCCCAGGAAAT	TTGGATTTTCCATCCTGTGGCA
9	FANCC	AGCCATTTTGAGAGGACACGTT	TTGCATAATGCCTTTACTGACCAAAATT
9	FANCC	ACATTTTGAAACCTGAGAAGAAGGATGT	TGAGTTAACCTGCAACTGATTTTGTTTT
9	FANCC	ctgctgTCGTACAGTCTTTCCA	GGAGGCTCTCCTCATCTGTCA
9	FANCC	GATTTCCTGAGGTTCACGTCCA	TGCATGGCTCTTAGATTTGAGTGATT
9	FANCC	CGAGGGCACTTACTCCACAAA	GATCAGAAAAACTGGCCCGAGA
9	FANCC	TTGAGTTCGCAGCTCTTTAAGGA	TTTTAATTTGCCTTCTCTTCTGTCCTGA
9	FANCC	GCAGATATGGCAGCTGAGCAATA	GTGATCTCCAGCCTTGTGGT
9	FANCC	GTCTTCGTTTTGCAGGAGAATGG	CTTGGCTTCCATGACACCTGA
9	FANCC	ATTCTGTCTTGGTGAAATCTGGTAGAG	TGGGATCAGGCTTCCACTTTG
9	FANCC	ACAGGTGTCTTGCTGGGTTTC	GACATCACCTTTTCGCTTTTTCCAA
9	FANCC	CCCCAAACACATGCAGTGG	TCGGAGGATGGGCTGAGAT
9	FANCC	CGACATCAGTAATTGCTCTGCCA	ACCCAAAGGAAGAAGAATTTAGGTTGT
9	FANCG	CCTCCCATCTCCCTGCAATAAC	CTGACTGTCACCTGCAACTTCA
9	FANCG	CCAAGCTTGCCCTCAGGATAA	GGATTTTATTTGGAGGATCTTCCATGGG

9 FANCG	GTAGAAGAGATGAGTCAGGTTGCT	TGGAGCCTCTAAGGATCTGCT
9 FANCG	GAGGCATCAGAAGTGGGAAGAG	CCGCAGTTTCTCATTGAGGTAGAAT
9 FANCG	GGGACTCCAAGTTTTCAGAAGTAAC	TCTAGCCAGGATAGATGGAGATAGGA
9 FANCG	GGTCAGGTGGTGGCAGTAGTA	AGGAAGTATGGCTCCCAAAGATTATA GA
9 FANCG	TCCACACCCCCTCTAGGAC	GCTGTGGGAAGATGCCAGAAAA
9 FANCG	GCAGTATGGCAGTTCCTTGGTT	CCTGAGGTGTTTTTGGAGGCA
9 FANCG	CCTGCCTGGATCAGTGCTAC	GGTGGCTCATGCCTTTCAAGA
9 FANCG	CACTCTTACACTTACGCCCAAGTA	GTCGTGGACTGGAATGGGTAG
9 FANCG	GTCCTGTAAGGCTTTGGTATCCTG	AGGCTGGGAAGACACTTCTTG
9 FANCG	TGGTTTCCCCAATCCACCCTA	GGTCTCCAGGAGCTGATCACA
9 FANCG	GCTTAGTGCCTTGTCTGGGT	CCAGACTCATGCCCTCTCTCTT
9 FANCG	TCCCTTTCTCTTAAGTCTCCTGGT	CCCTGTTCTGTGTCCTACTTCAG
9 FANCG	CAGGGATCTTGAGGCTGCAAA	CTAAGTCCGCTTCTGGTCTCC
9 FANCG	CGGATGTTTCTCTGGCTATGGA	TGACAACCCCTCCCTCATGT
9 FANCG	GCCTAAGGGTGAAAGATTGGCA	GCTGGAGGCCCAAACTAAGG
9 FANCG	CCTCACCACAGAGCATCTTCAT	TGTCAAGTTCTAAGACTCTGTACTCTG G
9 FANCG	GACCCAGCTCAAGCTCTTCAA	GGCATCATCCTTCTTTCTCTCCAG
9 FANCG	CAGCTTCAGGTCACTTTCCCTAT	TGTGGGACTCTGTCCTTCGT
9 FANCG	CGGTCAGCACTCAACCAGAG	ACTCTACAGTGCTGGAGACACA
9 FANCG	CCTGAGCCCCTGTTCCAA	AGAGGAAAGCCAGAGTGTGTG
9 FANCG	CCACATCTTCACCTGGCAGTT	GGAAATCCACAGAGAGCACTGTT
9 FANCG	GGGACACCAACCCGTCTAC	GGCTTATGGGCTTTTACTCCTCA
9 FANCG	TTTCAGGGCTGCAACCAAGTA	GGGTCCTGTCACCGTAAGATG
9 GNAQ	CGGGTGTCTAGGAGGCACA	CCTGAACCCAGACAGTGACAAAATTA T
9 GNAQ	CGCACGTGAAGTGGGAGTAG	AAAGTGTCCCTCTTGTCTCTTGG
9 GNAQ	AAGCCTATCTTGTTTTGAAGCCTACA	GATCATCGAATACCCCTTTGACTTAC AA
9 GNAQ	GACTCAGTTACTACCTGAAAATGACA CT	ATTCTCATTGTGTCTTCCCTCCTCTA
9 GNAQ	GCCCCGGACGGTACTCA	GAGTCCATCATGGCGTGCT
9 GNAQ	GACGGTACTCACCGAGCAG	CGGAGGCACTTTGGAAGAATG
9 GNAQ	TTTGGAGACAAAACCTATTCACAGCT A	GTTCCAGAACTCCTCGGTTATTCT
9 GNAQ	AGTCGACTAGATGGGAATACATGAT	TTTTTCCAGAACCGAATGGAGGAA
9 GNAQ	ATAATCCATTGCCTGTCTAAAGAACA CT	TTGCAGAATGGTCGATGTAGGG
9 GNAQ	AACATTCAGCTGACTGCTCCA	ATGGGTCAGGATACTCTGATGAAGA
9 GNAQ	AGCTTGGTGAAGCCCCTTTTA	GGTCTGATGAGCTGCTATTGTTCAT
9 GNAQ	GGGCCACCTGGAAAGATACTC	TGCACAATTAGTTCGAGAAGTTGATG T
9 GNAQ	GGATTCTCAAAAGCAGACACCTTCT	CCAGTATATGAAGGAAGGTGTTACCT GA
9 GNAQ	TCCATTTTCTTCTCTCTGACCTTTGG	ATCATCGTCATTCAAGAGAATATTTT CCCT

9 JAK2	AAGAAGGTTGGTGTGGCATTACA	GGCTCTGAAAGAAGGCCTGA
9 JAK2	AACCAGATTTCAGGCCTTCTTTCA	GGCCCAAATGACATCAAGAAAATGTT
9 JAK2	CTCACGATTATTTTGGTCAACTTGAATGT	GGTCTTCAAAGGCACCAGAAAA
9 JAK2	TGCTCCAGTACTTGTGGACTGA	CTAATGCCAGGATCACTAAGTTTGATGA
9 JAK2	ACAGGAAGACAGGAAATCCTCCTT	ACTGCCCAAGTAAAGCTTAGTAACATTA
9 JAK2	TCTTGTTCCTACTTCGTTCTCCATCT	GCAGTTGACCGTAGTCTCCTACT
9 JAK2	CAAAGATTTTAAAGGCGTACGAAGAGA	CATATGAGAGCACATCTTTAAACAGCAT
9 JAK2	TCTTTGGAGCAATTCATACTTTCAGTGT	TTCTGATTTTGTGAAACACCATTTGGTT
9 JAK2	CAGAGGCCTACTCATATGAACCAAAT	TGTCACATGAATGTAAATCAAGAAAACAGATG
9 JAK2	AATTTTTGGTTTTAGTGGCGGCAT	GTTTGATCGTTTTCTTTGGCTATTCTCA
9 JAK2	CTTGGGATGGCAGTGTTAGATATGA	TCAAAAGGCATGGGTAAACACAGAA
9 JAK2	TCTTCAGGAGAGAATACCATGGGT	GAGAATCCAGAGCACTTAGAGGTTT
9 JAK2	GGAAATCTGCAGTGGAGGAGAT	ATGCCACACCAACCTTCTTTTTAAAATT
9 JAK2	ACTGTGATGTCCATTGTGACTATCC	AGACTGCTGAAGTTCTTCTTTGTCC
9 JAK2	GGACAAAGAAGAACTTCAGCAGTC	GCCACATAAACAATCCATGACCAGTAAT
9 JAK2	TGGACAACAGTCAAACAACAATTCTTTG	AGGCATTAGAAAGCCTGTAGTTTTACTT
9 JAK2	TTTGCAGGTAAAATCAAGAGTCCACATA	CTCCAAACATCTGAGGCCACA
9 JAK2	GTATGCTCCAGAATCACTGACAGA	CACTGACCGCTGGTGGA
9 JAK2	CATGGAGTTGACTTTCTAAAAGGTGCTA	GGTAATGATGTGCATCTGCAGTTAATCT
9 JAK2	CTTTGTCTTTCGTGTCATTAATTGATGGA	CGAAATTGGGCCATGACAGTTG
9 JAK2	CCTCCAGCCGTGCTTGAAAATATA	TACATGTATCACTCTGTTAAATCTTCTTTGT
9 JAK2	CCATAGGGTATGGAGTATCTTGGTACA	GGGACTTTCACCAGGTTCTTTTACTT
9 JAK2	CGAGAAATATATTGGTGGAGAACGAGAA	CTCACCAGAATATGGGACTTTCACC
9 JAK2	GCCAATTTAATTTCTTTACCTATAATGGTCACA	CCAAATTTTACAAACTCCTGAACCAGAA
9 JAK2	TCTGGTTCAGGAGTTTGTAAAATTTGGA	ACTTACTAGAAAATGCATGGCCCAT
9 JAK2	CTTGAAGTTGCTAAACAGTTGGCAT	CAGACTATTTTACATGAATTGGCATCCA
9 JAK2	TGGCAGAGTAAAACATTATTTCCACCTT	GTTACCTTGCATATCTGAGATGTGTACT
9 JAK2	ACATAAAACTTCTGCAGTACACATCTCA	TGGTCTTAACCCTATATATTCCCTTTATTATCT
9 JAK2	CCACTGTGTTGTAAGGCCTACT	GATAGTCTTGGATCTTTGCTCGAATACA
9 JAK2	CCCTAGCTACAAGACATTCTTACCAAAA	CTTAAGTTTCAAGTTTCTGGCAGTGG
9 JAK2	GCAGATTTATTCAGCAATTCAGCCAATG	ACCACTTCCAGGTTCTTTTACTTCAA

9 JAK2	CAGTCTGCCTTCTACACAGAGAAAT	CTGTTCTGTCAGTGTCTCACTTTCTTTA
9 JAK2	GAATTCAGTGGTCAAGAGGGAAACA	TCAAATGGGAGAAGTGCAATACCAT
9 JAK2	CCCATTGACTGGAGGAAATTGAGAAA	GAACACGATCATCTGTCCTTGTTTG
9 JAK2	GGAATTTATGCGTATGATTGGCAATGA	AGAAAATGCATGACTACCCTGGATTAAA
9 JAK2	TGTTTTCTCTTACAGGCAAATGTTCTGA	GTAAAGATACACCTGAAGAACTGGATCT
9 JAK2	CTGGAAATGCCAATTCTATGAAGCAAAT	CCACAAGCTTTAGAAGCAGCAATAC
9 JAK2	GGGAGTATGTTGCAGAAGAAATCT	ACGCCAGCCATACAAGCattaa
9 JAK2	aGACAGTCTGCTAATTCCAGCTACT	AGAGGGTCATACCGGCACA
9 JAK2	GGGTAATTTTGGGAGTGTGGAGA	TGCTGTAGGGATTTCAGGATTTCAATT
9 JAK2	GCACCTAAGAGACTTTGAAAGGGA	ACAGGATGCCAAACACATACCTT
9 JAK2	TGTATTTGAACTATTTGGAAGCTGACCA	GCAAAGAGGTAAGACATGACAAAGTCA
9 JAK2	GTGCTGAAGCTCCTCTTCTTGA	AGACAGAACTGCAATTTTCCCATATGTA
9 JAK2	GACTTTTGATTGTTTTAGATGACACTTGGT	CCAGTCTGATTACCTGCTTTCTTCA
9 JAK2	TCTTTACCTTTAGGATGGATTTTGCCAT	GTGACATACTAACCTCGACAGCAA
9 JAK2	TCCTTTCTCTGCTTCTTTTCTAGGTATCA	GCCTGGTTGACTCATCTATATGGAAGA
9 JAK2	AGAAAGGATCTGGTATCCACCCAA	CCATTTTGCCTTTTAGCATTAAGTGAGT
9 JAK2	AATGGCTCTGTAAATTCTACCCGTTT	GCTTATGGATAGTTACAACTCGGCTT
9 JAK2	TCCACCAATTAAAAGATGGCCCTT	CATCCAGCCATGTTATCCCTTATTTGA
9 JAK2	GGGATCTAGCTCTTCGAGTGGA	TCTTTGCTGGCTAGCATCATGATTTAT
9 NOTCH1	CATCCAGGTTGATCTCGCAGTT	GCGACAACGCCTACCTCTG
9 NOTCH1	TGGTCCCCTTCAGGCAGAA	CCACCTTCACCTGCCTCT
9 NOTCH1	CAGTGGTGGCCCGTGTA	CCGCCAGTCCTAAGTCTTCC
9 NOTCH1	GGCTCTGCCCTCGACAAAG	TGAGACTGACCTCTCTTCTCCTG
9 NOTCH1	CCAGGCCACATCCAAGTTCAG	GTCTCTGCAGTGCTGGAAGT
9 NOTCH1	TTGCACTGGCTGTCACAGT	GCGGGCAACAAGGTCTG
9 NOTCH1	CGCGTGGTTGTTGCACTG	CCAAATTCAACGGGCTCTTGTG
9 NOTCH1	CGAAGCTGTAGTCCAGGATGT	CCCCGAATGCCAGTTCCC
9 NOTCH1	AGGTCCCCTGGTTGTAGCA	GTGAGAATGACGCTCGTACCT
9 NOTCH1	CCTCACACAGGAAAATGGGAGT	ACCTCCCCAACACCTACAAGT
9 NOTCH1	CGGCCCTTACCCTGAGTG	AGGGCCCCTCGTTCTGT
9 NOTCH1	CCAGTGGCTGCACGTCT	ACTTACAGATGCAGCAGCAGAA
9 NOTCH1	CTTTGCTGCTGCTGGATGTTT	GCTGCACAGTAGCCTTGCT
9 NOTCH1	TGGTAGCTCATCATCTGGGACA	GCATGGTGCCGAACCAA
9 NOTCH1	CCCCGCAGAGGGTTGTA	TCTCTCCCACCTGCCTGT
9 NOTCH1	CTCCCAGGGCCACGTAA	CGCAGATGCCAACATCCAG
9 NOTCH1	GTGCGGCCCATGTTGTC	CGTCATCTCCGACTTCATCTACCA
9 NOTCH1	GTGCGGTCTGTCTGGTTGT	CAGACTGAGCACCCGTCT
9 NOTCH1	CAGGTTGTACTCGTCCAGCA	GGAGGAGACACCCCTGTTTCT

9 NOTCH1	CACCTTGGCGGTCTCGTA	CTTCCTCTGGTGATGGAACCTTG
9 NOTCH1	GCCACAACCCTTACCCTAGGA	CTACACAGGCAGCTACTGTGA
9 NOTCH1	GTGAGCACTCGTCCACCA	CTGGGCCTCACCTGTCTAC
9 NOTCH1	CCTCCTTCGGGCACCTCT	GCTTCACGGGCAGCTACT
9 NOTCH1	GCACTCATTGACATCGTGCT	CACCAATGCCCTCCACTCA
9 NOTCH1	CCAGGGTTTAGGACTGATGTGT	GGACGGCATCAATGGCTTCA
9 NOTCH1	CTCATTGACCTCAGACAGGCA	CCTGCCCTTAGGGAGCAT
9 NOTCH1	CCGCCCCTCCCACATAG	CAGGTCCCAACTGCCAGA
9 NOTCH1	CACACTCATCGATGTTGATGTTACAC	GGTTTCTGACACCTGGAAGGAT
9 NOTCH1	GACGCACACTCGTTGATGTTG	TCTGGCTCTTGGAGATGAGGAG
9 NOTCH1	TCAATGCAGCCAGCGCTAA	GGGATCCACTGTGAGAACAACAC
9 NOTCH1	CACACCTCTCTGTGCAGTCA	GCACTTTCTGTGAGGAGGACATC
9 NOTCH1	GGTCACTGGCACACTCGTT	CTCACACTCACCCTTCCGT
9 NOTCH1	GCCCACAGAGGACCTTGATG	CCAGTACCGCTGCGAGTG
9 NOTCH1	TGGGCACGTCGCAGTAAA	CCAGCTGACCCCAATCTGTC
9 NOTCH1	GGCTGCTGGCACCCTTA	CGTGGTTCTGCCTGACCTG
9 NOTCH1	GGTGGTCTGTCTGGTCGTC	AGGACCTGGAGACCAAGAAGTT
9 NOTCH1	TGGGTAGCAACTGGCACAAA	GCAGCCTCAACATCCCCTAC
9 NOTCH1	CTGCACGGCCTCGATCTT	CAGCCCCTCTCTGATTGTCC
9 NOTCH1	ACCTCTGAGCACAGTGCAG	ACGTGCCACCCCTTCAG
9 NOTCH1	CCTGGATCCCGCCAAGTA	CGACCTGCTCACGCTGAC
9 NOTCH1	GGCAGCGGCACTTGTACT	ACATGCCACGTGGTGGAC
9 NOTCH1	CATAGTCTGCCACGCCTCTG	AACTAACTGCCCTGGCACAT
9 NOTCH1	GAAGCAACCCACAGATGTTCC	TCAACGAGTGCGTCTCGAA
9 NOTCH1	CAGGTGGCGTCGTTCTG	CAGCCTCTCACCCGTGT
9 NOTCH1	CCCTGTGCTGGCACCTA	ATCAGCAACCCCTGTAACGAG
9 NOTCH1	GGTTGGTGTCGCAGTTGGA	CGTGGGTGGTGTGCCAT
9 NOTCH1	GCCTCCCTGGGTGCTTATG	GCTACAGTGGGCGCAACT
9 NOTCH1	GCAGTCGTCGATGTCGGT	ACATCAACGAGTGCGTTCTGA
9 NOTCH1	GTGGGTGTTCTGGCAGGAT	GGTCGGGAGAGGCACTGTA
9 NOTCH1	GCCTGCGTGAAAGAAGCAGAT	GGAGGACCTCATCAACTCACAC
9 NOTCH1	CCAGGTCATCTACGGCGTTG	CCTGAGCCTCTCCCTGTT
9 NOTCH1	CATCCCGCCTTCCCAACT	GACGTCACCCACGAGTGT
9 NOTCH1	GCACTACCTGGCAGGCA	ACAACGAGGTCGGCTCCTA
9 NOTCH1	GCTGCAGGGCACGTAGG	TCAACGAGTGTGGCCAGAAG
9 NOTCH1	GTAGGAGCCGACCTCGTTG	CTTCGAGGCCTCCTACATCTG
9 NOTCH1	GCCACACTCGTTGACATCCT	GACCCTCTTGTCCCCTTGTC
9 NOTCH1	GCAGACTCCCGGTGAGGAT	GCATGGCCAGCTCTGGT
9 NOTCH1	CACTTTGAAGCCCTCAGGGA	CGCAGCTGCACTTCATGT
9 NOTCH1	CTTGCCTTTGCAGCCATTGAT	GCGCGTCAATGACTTCCACT
9 NOTCH1	CACCGGTGTGACCAGCA	GTGGGTGAGCGCTGTGA
9 NOTCH1	GGACAGGCACTCGTTGACATC	TCAACGTGGACGACTGCAAT
9 NOTCH1	CAGGTGCCGTTGTTAAAGCA	GTCTTCCGGGACGGACAC
9 NOTCH1	GTCAGTTTCACTGCCCTGAGT	CCAGTACGATGTGGACGAGT
9 NOTCH1	CACTTGGCACCATTCTTGCAG	CTGAAGGGCCATAGTGCTGTT
9 NOTCH1	CCAGCTCCTCTTCAAAGACTCATC	ACTATGAGAGCTTCTCCTGTGTCT
9 NOTCH1	CCAGCCTCACCTTGCCA	GGGTCACGTGTGTCTCCTT
9 NOTCH1	CATGTGCCGCCGTTGAG	CGAGGGCTGGGTGTGAG

9	NOTCH1	TGTGTTTTAAAAAGGCTCCTCTGGT	CAGATCGCCCGCATTCC
9	NOTCH1	GCGCGCCGTTTACTTGAA	CTGACCAGTGGTCCAGCTC
9	NOTCH1	CCAGTCGGAGACGTTGGAAT	CCCTCGCAGCACAGCTAC
9	NOTCH1	GTTGTCCACAGGCGAGGA	CCTGGCGGTGCACACTA
9	NOTCH1	TGAAATTCAGGGCCCCTCC	GCATCGGGCACCTGAAC
9	NOTCH1	GGTGGGCCAGTCTCAAAGG	CAGCAGTCTCCGTCCGT
9	NOTCH1	CATCCCAGGCAGGTGGTT	AGCTCCGGCATGCTCTC
9	NOTCH1	CACGTCTGACAGGTAGCCAT	GGCCTGTGGAAGCAAGGA
9	NOTCH1	CCGTCCTGGGACTTCTTCCT	CGCCCAACGGCTACCTG
9	NOTCH1	GGGCTTGCGGACCTTCTT	GCGCATGCATCACGACATC
9	NOTCH1	GTGTGCGTCACGCTTGAA	GGACGGGCTGGACTGTG
9	NOTCH1	GCTGAAGTGGTCCTTGCAGTA	ATGCAGTTCTAAGGCTCTGCTC
9	NOTCH1	CAGCGGCCATTGTGCAG	CTGGAGGCAGGGTTCGTTT
9	NOTCH1	GTGCAGTTTAGTAAGTGGGTAGCA	TGAGGAAAATATCGACGATTGTCCAG
9	NOTCH1	CCCCGTTCTTGCAGTTGTTTC	CTTGTGTCCAGAGCAGTGTGT
9	NOTCH1	ACGGGCTCCTCGAACTACAT	GGACGACAACCAGAATGAGTGG
9	NOTCH1	CGACTCACCCGGAACTTCTTG	TCCTAGGGAGCTCGCTCAG
9	NOTCH1	GGCCCTCTGCACTGAGAAAC	GTGGGACCAACTGTGACATCA
9	NOTCH1	GATTCAGCCCTCACGTCTCC	AACCTACCCCATCTGCTTCTTTC
9	NOTCH1	GGTTGGATTCACACTCATTGTTGT	CCCATAGGGCATTGCAGACC
9	NOTCH1	GGGCCTCAAGGCACTCA	GTCAACGGCTGGACTGGT
9	NOTCH1	TGTTCTCGCTGCAGTCCTC	GGCCGACAGCTCCTGTTC
9	NOTCH1	CTGCACCCCTGCACCTA	CTCACTGCCCTGCTCTTACC
9	NOTCH1	CCAGCCTCTCGGGTACAT	CCACAGCCCCCTGTACGA
9	NUP214	CGACAGCCTCCGATCTTTCAG	AGTTAAAAGACTGGGAAGGATGCAAT TA
9	NUP214	TGTGACATCCTCTGCAACCAC	TGAAAAACTAGTCTTGCCAGCACTT
9	NUP214	CATCAGTTTTGGTGGGACATCTCT	AACTCAGGAGGCTACCAAATGAC
9	NUP214	CCTTCCAACATCATTCCCCACATT	CTCACTGTCACCTGGCTTCTC
9	NUP214	AGAGGCCACTTCATCAGCTTTG	CTGGGCAAGAACAGGTTCTTTT
9	NUP214	TCCGCAAACTTCTGACTCTGTTAA	AGGAGGAAGCAGGTGGTACT
9	NUP214	AGGTTGTGGCTTTATGCTTAGAACA	ACCATATTCACTGGACATCATGCAC
9	NUP214	AGCTGTGATAACCTCACACTCTCT	CAAGGAAATCTGCTTAGGGTTCTCA
9	NUP214	CCTATTTCTGTTTGTTTGCCTCTGTT	CCTCTCCGAACACTTTGCCT
9	NUP214	CCAGCCTTTACAAGCCCTCT	GGTCAAATTTCTTTTCAGAGCAAGAG T
9	NUP214	AGACAGACCTTGGTCTCAGTAATACA	GATTCTCACCTTCTTTAGCGCTCT
9	NUP214	CTCGTTTTGATTCTTCACAACAGGATT T	ACCAGACCATATTTGTTGGACACA
9	NUP214	GGAACGCTCGAGTCTGCTT	TCCTCTCTCTACTTTGCAACATAAACC
9	NUP214	CTTTTGATGACATTGCTCATGCTAGTG	CTCTGGGAGCTACTAACAGGAGT
9	NUP214	GTTTACTGCTGCAGCTACCTCT	TGAGGGCAAGACTGAGGACTT
9	NUP214	CCACCTAGTTCCGTGCCATTG	TGTCCAACTATGTTAGCTACGACTCTT A

9 NUP214	CTGGGAGATTCCTTTACTTGAACCAT	CCTCTTCCTCTGTTGGTTGATGA
9 NUP214	CCCTAGCCAACAATCGGGAAA	TTTATTGAGTGCAAGTCCTCTCCAC
9 NUP214	AGGGCAATATTGTGCCTTGGT	AGTAATGCTACTCAACACAGAGTCTGTA
9 NUP214	CTTGCCCAAAGTACCAGGTAATTG	CCAAGAAGTTTCTCAGTTGTGCCT
9 NUP214	GCCAAACTGTCCCCCATGAAA	cCCCACCACACCCTATCACTAT
9 NUP214	ACTGGAACAGTGTTTCTGTCTTTTTGTA	GTTCATTACTGACCCTGATGGAGAAAA
9 NUP214	GCAGTTCAGCAAGCCTTTCTCA	GGTAAGAACTCTGGGTTCATACACATTT
9 NUP214	CCTTTGCTGGAATTAATATGGTGTTCTC	TGAAACACACTGCAGTCAGAACATA
9 NUP214	CTGCAGACCCAGGTTAGCTTA	AAGAGATTTACCTGATCACTTTGTCGAG
9 NUP214	CCACTGACCTCAGTCTGTTTCT	GGTCCCAAAACCAGAAGTCTGT
9 NUP214	CACTTTCGGATCACTGTCCCA	AAGAGGGACCCATTTTGCTTGT
9 NUP214	GGAGAAAGGACATTTGTGATAGGAGAA	GATTTCTGTACCATTGAGGGCACT
9 NUP214	ACTTCTTTTTCAAGCAGGACGATCT	CACAAACATTGCAAGCTGAATTTAAACA
9 NUP214	ACCTTCCTTGATCCAAACAGAAAGAT	CCAGAGTCACGATTTCTTTCATTTTGTT
9 NUP214	CTGGTGTTGAGGAAGCCAGA	AGGTAGCAATTGTTACATGAGCTTGT
9 NUP214	CAAGGAGATGGAACAGGTAGCA	CCTCTGACAGGATGATCTGACTCATAAA
9 NUP214	AAAAAGTCATTCCTTGTCCTCCGT	CTCACTGAGCAGGTACATTACATGA
9 NUP214	GATTCTTTTGCTGTGGGATCTGAGA	CTGCCATAACAGGGCTCCATAAA
9 NUP214	GAAAAGCACCCAGAGATATTTGTGAAC	AGGAATGCCCTGTCCAGACT
9 NUP214	GGACAGAAGGTTGGCTTGAGAA	aaaTCAGTAAGTTACCTGCTTGGCT
9 NUP214	GGGACCACCTTTGTCTTTCGAG	CACACACTTCCAACCTTATTTAATGCTT
9 NUP214	TTGCAGTTTTTAAACCTTTTCCTTCCAT	CCAAGTCTGTCAGCTACTTTCAGAAA
9 NUP214	CCTCTGACCTCCCTCACATCTT	CCTTGGGATGCCACACTTCC
9 NUP214	GCACTTTCAGCTCTGGAGGA	CCCTAGAGATGTCTCCTTCCAGATAT
9 NUP214	GCTTTTGGGCTTGCTTTGAGT	TTTACAGGACGTCCGTGCAT
9 NUP214	TCCCAGTCTCTGGAGAGTGAAG	CCAGGTGAAGAACCATGAACCAG
9 NUP214	GCAGCACCTTTTGCTAAATCTCAC	TGGTGTGACAGTATAAGACCCAGTTA
9 NUP214	AGTCAATGAAATCAGGGCCTTTGA	CGAAGACGTAGGTACCAATCCAC
9 NUP214	GCCATTTCACGATGACCTCTCAT	GCATCCTGACTGGGTTTTCCTC
9 NUP214	TGTCTTTCAGTTCTGGATGTGCT	AACAATGAAATTTCTTCCACAGGCATAC
9 NUP214	GGAGGTTTCTTCAGTGGCCTTG	AACACCCTAAGAATGACAATGGCATTA
9 NUP214	ACCTTTTGTATTTTCCTGTTCTCTCACC	GGAAGGACTAGGTGCACCAT
9 NUP214	CTTGCCACGAAAACCGTGAAA	CCTGTGACTCAGACAGCTCATA
9 NUP214	GCTTTCTATTATGTGAACCTCCACTGTT	GTGATGAAGGTGGCAGAGAGG

9 NUP214	CCCACAGAAACTGGATGCTTCT	CCAGTGACCGTAGCAGATGAC
9 NUP214	TGTTCTCCTTTGGTTCTTCATCTTTGAA	GCTAGGGAGGCTTTAGAAGGG
9 NUP214	GCCCATCAACCTTCTCTTTTGTT	GCTATATTTGGAGCAGACACACTTGG
9 NUP214	CCTACCCTGGAAAGCACACC	TCTACCCAACTCACAGAAGAATCAAAC
9 NUP214	CAGGTTTTGGGTCTAGTAATACTGGTT	CAGTGAAGAAAACTGAATGGGTTTGAT
9 NUP214	ACATCTTCCCCCAAACTATCAGAAAAAC	TCTTTTGTTCCAGATGCTGATCCC
9 NUP214	TGTGTGTGATAGATCTTATTGGTTTGCT	GAACTTACAGGAAGATACTGGACCAC
9 NUP214	TTCAGATGAGTCCCTTAAACCCTCT	GACAAAGCACACCATCTGTTGAA
9 NUP214	CCTGCTCCAGTTCTCATGTTACT	CCCTTACTGATAAAGCAGGCAGA
9 NUP214	CCTGATGCCAGGACGGA	GTGGTGCTGGAGGCTATGG
9 NUP214	GGGTCTTTGGACAGACAACCTT	GCACCAAAGACACTTCCACTG
9 NUP214	GCCGTCGAAACATCAAGTACC	TGTTGTTGGTGAGCTGGTTGA
9 NUP214	GCTTCCTCCACTCCCACATC	CAGAGCCTCCCTGTCCAAA
9 NUP214	GTCATCTTTTGCTAAATCCAACCCATT	TGTGATTCCTGAGGGAGGTGA
9 NUP214	CTGCCAGTTCTAGCTCAGCTT	GCTGGCTGTGCTACCAGTC
9 NUP214	CCTTCTTATGAGGCCATTCCTGAAAG	CCAGCTTACTAGAGGTGGTGGA
9 NUP214	CTCCTGCTTTTGGGCAGAGT	AGAGGGAAACAAGTAAAAAGCTTCCA
9 NUP214	CACCGCACCAAGTCTGTTTG	CGACTGCCCAAAGACTGAGG
9 NUP214	GCACCTTCTGGAACTGCTCTT	CGCAGTCCTGAAAAACTTCCC
9 NUP214	CAAGTTCTTTGGCTGGAGAGACT	TCTTGGTTGGCTGGGTACTAGTTA
9 NUP214	CAACCAATAAGGCTTCATCCACAAG	CTGCTGGTGGTGCTACTGAG
9 NUP214	AAGTGAGTCATCTCTGTTCTCCTATT	GGCAATTCAGCTCGACTAGAATCC
9 NUP214	GATTAATTGGGAATCTTGGCTACTGGA	GCACTAACTACCAGGCTACACTTAC
9 NUP214	GGGAGGAAGTTTGCTGTCGAG	CCCGGTTAGTCTCTGACCTTCAT
9 NUP214	CGAGATGGATGCCATGATTCCC	TGTGAACGCGGTAAACTGTT
9 NUP214	TTACAAAGAATGGTTAGTGGTTTAACATAAACA	AAGCTTTGTCACCAGATGATAACTGA
9 NUP214	CCTTAAGCCATCTGGGCCTAC	GACTCCTGTTTCCTATGGCAACT
9 NUP214	CCAGCAGTCTGAGCAGTTAGTAA	CACTTGGAAACAGGCTTTGGAA
9 NUP214	GTTGGAAGAGTTAAAAGCCCGAAC	TGCAATTCAACTCAGGAAAAGTATCCAT
9 NUP214	CTGAGTCCTGACCTTTGCACA	GTTGGTCCCAGGGATCGAAAG
9 NUP214	CTGATTTGAGATACCTTGTGTATTCAGT	GCCTCCTGCATCTTTCAAAAGCTTA
9 NUP214	CAAAAACGCCCATTTGCCTATCA	TGACTTGCAGGACAGCAATACT
9 NUP214	GCAGTTTGTCTGGCTGATGGT	TGCTACAGTGTGAAAGCCTTTATTACTC
9 PAX5	TGTCCCGGAGTTTGCACATC	TTTCCCTGTCCATTCCATCAAGTC
9 PAX5	CCATCCCTCCAAATCCCCAAC	GAATATAAACGCCAAAATCCCACCAT
9 PAX5	TCCCTGATCTCCCAGGCAAA	GGTTCCTCATGGCTAAGCTTCT
9 PAX5	GCTGCCTGCTGTGGAGAC	GCTCACCCTGTGATTTGTTGCT

9 PAX5	CCTCAATAGGTGCCATCAGTGT	GGATCTGACCTCGGTGCTCT
9 PAX5	GGGTAGCTGATGGCCCAAG	TGTTTCTCTCTCCACCCACCATA
9 PAX5	GCTCCTCTGCAGGTAAGGG	CGGCATCCTGGGCATCA
9 PAX5	GCCTCTCTTACCTTCGTCTCTCTT	CCTGACCGCCCGTCTTTC
9 PAX5	GATGCCTCTGCCTTCAGGAA	TGGACCGCGTGTTTGAGAG
9 PAX5	GGTGAAGATGTCTGAGTAGTGCTG	CCTTCCTCTCTCCTGCTCATCAA
9 PAX5	ACACCAAGAAGCCACTCTTCC	GGACGACATGAAGGCCAATCT
9 PAX5	CACACTGCTCCCGATGTCA	ACGTGGAGTTGGCATTTGACT
9 PAX5	GTGTGCTGAAGTGTTTTATGCAGAA	TCATCCGGACAAAAGTACAGCAG
9 PAX5	CCTATGCTGTGACTGGAAGCTG	CTTGGGTATGAGTTTTCTGGTTGTCT
9 PAX5	CTGCTGGGTCATGTTTTAGGTCTT	CCGCCAGAGGATAGTGGAAC
9 PAX5	GCCTGACACCTTGATGAGCAA	ACTGGTTCTAACTACCCTTTCCCT
9 PSIP1	ACACACAAGACCCATTAAAACCTATTCC	GTTTTTCCATTGGATTCCTTCGCAA
9 PSIP1	CTATTACGTTACGATTTCCCCCTTGAAT	CGAAGTTCCTGATGGAGCTGTAA
9 PSIP1	GGTAGTTTGTTTGTGGGTGGCT	CCTTTTGGATGTTGTGCTCTTGTTTATT
9 PSIP1	CTCTGGAGAGGAGGGTAGCA	GTCTCGCCCCCGAAACA
9 PSIP1	GCAGAGATATGTGCTTGTTTAAAGTCAA	TTTCCATGTGTATATTAGCTACAGAAGTCAAG
9 PSIP1	AGCAGTCCTGGCAAATGGTTTA	GTGCTGAATAAATCTCTTGCTGAACAAA
9 PSIP1	GTTTTATTCGCTTCCTCATGCTGTC	TGTTTTCTCAGATACGGCGATTCA
9 PSIP1	CTTCTTGATCATCTCCTTCTTCTTCAGA	ACCTAAAAAGCAGCCTAAGAAGGATG
9 PSIP1	GGAGGTTGAAGTAACCCCTGTT	GGAAAAACAACCTAAAAAGCAGCCTAAG
9 PSIP1	GCTAGGATAGTGATTATTCCCCAGGAT	CAGAAAATAAGGAAAAGTATGGCAAACCA
9 PSIP1	CAGCAACACTTTAAATGGACTGGAA	GACAACAGCAACAGCATCTGTTAA
9 PSIP1	TCGTCCTCTTTTAGGACTCACTTTTAGA	CCATCGAATTAAACTTTGAGTGCCTACT
9 PSIP1	CCATGCTAGCCTTTAAAAAGAACCC	GACTGCTCACAGAAGGAATATGCT
9 PSIP1	GCTTCTTTCTCATGTTGGCCTTTC	TGCTTTTCCCCCAAGATTATAATTCCAT
9 PSIP1	ATTACTTACTAATTAATCATGGCCAACTCATTT	CAAAGGAAGATACCGACCATGAAGA
9 PSIP1	AGAACATACCTCATTGCTGGCTTTT	ATTAATAGAGTTGATTGCTCACAGGCA
9 PSIP1	GTATCTTAGAAAGTGAAATAACTAACCTCGCT	CATAGGGATTGTGGGCATTTAGTCA
9 PSIP1	ATGAAAACCATTACAAACTTCTCAAGTG	TTGTACCTTTCCCCCTTTTCTTCAG
9 PSIP1	GATCTTCATCTCTTGTTTGCTCCACT	AGCTTGTGTGAAATGCATTATGCAA
9 PSIP1	AGGTTTCATTGTATCTACTCTCCCTCAA	CAGTTGACATAACTACTCCAAAAGCTG
9 PSIP1	CTTTCTCTTTCTCCCCCTTCTGG	AATGCTTTCAGTTAAACGTGCTTGTAA
9 PSIP1	TGAATCGCCGTATCTGAGAAAAC	ATGCAACAAGCTCAGAAACACAC

9 PSIP1	TGAGCTTGTTGCATTGTGACCT	ACTGGTCATTTTGCACTACCTCTTTT
9 PTCH1	GCTATGCTGAAAGGAATTTGACTTCCA	CTTCTGGGAGCAGTACATCGG
9 PTCH1	CCAACACCACGCTGATGAAC	TCAGACTTTGTGGAGGCAATTGA
9 PTCH1	TGCTGCAGATGGTCCTTACTTTT	CTAACCTGTGCCCTTCTCTGTC
9 PTCH1	CCTGGTTCTGCAGAGTCACTT	CCGACTCGGAGTATAGTTCCCA
9 PTCH1	GAGGCCTGACACTGTCGT	CATGGAATCCCCTTAAATAGGTGTCT
9 PTCH1	AGGCGGTTCAAGCCGTT	GGAACAGAGGCCCCTGAAAAATA
9 PTCH1	AAAGTAGAAGCAATCTGATGAACTCCAA	CCTGTACGAAGTGGACACTCTC
9 PTCH1	AGCATAGTGCTTCTCAGCAAAAGA	GTGACACAGGACACCCTCAG
9 PTCH1	GTGCTCTCTGGGCTCTGG	CCACAGCTTTGCCCATGAA
9 PTCH1	GGACTGCATGGTAATCTGCGT	CAACATGTTTCCCCTCCTTTCAG
9 PTCH1	CACTGAGTCCTAGAGAAGTCACAGA	CATGGCCTCTTCTTTTTAACTTTGACA
9 PTCH1	GCGATCCCAAAGAGTTAGAGGA	GCCGGACCGGGACTATCT
9 PTCH1	AGTCTGAAATGCACCTTGGAAATCT	GCAGCGGCTGTATCGGT
9 PTCH1	GGCCGGTGCAGATAGTCC	ATGGCCTCGGCTGGTAAC
9 PTCH1	AAGGAACCTGTTGAAGCTGAACA	CTTACTTTACGACCTACACAGGAGTTTC
9 PTCH1	TTCCAACATGACATACTTCACGTTACT	CGGACATTGTACCTCGGGAAA
9 PTCH1	GTGCAGCAATAAAGTCATATTCTCTGG	CTGACCTTGTGCCTCTTCTGTT
9 PTCH1	GGAAAAGGCTGCAAGACCCTT	GACTGGGCCTGTGCTCATT
9 PTCH1	GCAGCGTTAAAGGAAATTCCGAT	GTTCTGTGCCCCCATTGTTC
9 PTCH1	GGGACATCCCCGTGTCACTA	TGTTCCCGTTTCCTCTTGATCTC
9 PTCH1	AGAAGAGGCCATGCGTTAGG	CTGCTTTTCATTTTATTAGGCAGTGGAA
9 PTCH1	GACCTCCCGATAAAACGCTACTT	TCATTAATCCCAGCGCTTTCTACATC
9 PTCH1	TGACCCAAGCCGTCAGGTA	CCCCCAACCCCATTCTCAAAG
9 PTCH1	AGCCAGAGGAAATGGGTTGTTT	GAGCTGTTCGGCATGATGG
9 PTCH1	GCACTGAGCTTGATTCCGATGAG	TGATCTGAACCGAGGACACCTT
9 PTCH1	GACTAAAACAACCAAACCAAACTCCA	GTCGCACAGAACTCCACTCAA
9 PTCH1	GGTGGTGAAGGAAAGCACCTT	GCTGTGTCACCTGAGATCTGTG
9 PTCH1	GGGTCACACGCTGTCAAG	AATTACAAGAATGGATCAGACGATGGAG
9 PTCH1	CCAGGAGTTTGTAGGCAAGGA	GCTGCGAGTTATAATGTGTTACAATCAT
9 PTCH1	GAAAATGAAGAATTGCATAACCAGCGA	GGGTACGAGTATGTCTCACACAT
9 PTCH1	CGCCTTACCTGCTGCTCATTA	CAACTCATGATACAGACCCCTAAAGAAG
9 PTCH1	GCTTTGTCCTCGTTCCAGTTG	ACTGATGATGTGCCTTCCCTTG
9 PTCH1	GTGGTCAGGACATTAGCACCTT	CATGAGTTTGCAGTGATTTTGCTATTCTA
9 PTCH1	TCCCTCGTCTCCCCCTTG	CTGCACTCCGCCGAAAG
9 PTCH1	gtcttTACAAAAGGAACGGAAAGTGT	CTGACAGGTCCTGCCTATGG

9 PTCH1	CTCTTTGCCTGGCTCTAGGT	TTTCCACGTCCGGTGTGAG
9 PTCH1	TCCACCTTCGAATCCCTCCT	GCTTCTGCCTCCGTGACT
9 PTCH1	GGGTAGCCTGGGCAGAGT	TTCTCACAACCCTCGGAACC
9 PTCH1	CCATGGCAGTGGACGCT	CCCCCAGAGAAGGCTTGTG
9 PTCH1	CTTCAGTAGAAATTTCAAAAGCGTCTCT	ATCCCGAATCCAGGCATCAC
9 PTCH1	GTGTTTTGCTCTCCACCCTTCT	GAAATGCTGAATAAGGCTGAGGTTG
9 PTCH1	GCGGTCCATGTAACCATGAC	TCTTTTCATGGTCTCGTCTCCTAATTTC
9 PTCH1	CTGAAGTGCCTTAGCAGAGACC	ATCAGCAATGTCACAGCCTTCT
9 PTCH1	GGGATTAACGCGGCCATGA	CACGTATCTGCTCACACAGTCT
9 PTCH1	cgcTCTTACCTTCCACCCA	GAGAGCGAAGTTTCAGAGACTCTTATT
9 PTCH1	CCAAGAACTTGCCGCAGTTTTT	ACTCCTCCCTTCTGCTTCGT
9 PTCH1	GAGGGAAGTGGCTTTTGAGGAA	TCATGGCTTATCCAGAAAGTATATGCAC
9 PTCH1	CACAATCAACTCCTCCTGCCA	gtgacCTGCCTACTAATTCCCATAATT
9 PTCH1	GGAAGCTGTGATGTCCCCAAA	CACCATCCTCTGTTTTTGCTGTAG
9 PTCH1	TTACCCACATTCCTTTATAAGTCCACAG	TCCTGCAATTCTCAGCATGGATTTA
9 PTCH1	TCCTGTCCTCGCGTCGATA	GCATGTTGGTGACCTCTGAATTTTT
9 PTCH1	ccggccCAATCACAATGATTTC	GTTTGCACCCGTCCTGGAT
9 PTCH1	ACAATGAAGTCGAACTCAGATCCC	GCAACCTGATCTTGTGAACATCCT
9 PTCH1	TGTCTCGGGTTCGAGGGT	CAGGGTTGACTGAGTCTTTGGT
9 PTPRD	CCTTAGGGAAAGGTTAGATTCTGAACA	GGGACTTTTGGAGAATGATATGGGAAC
9 PTPRD	ACAACTGTGGCACTCCGTT	CCTGGTTGAATAACACTCCTTGTTTTC
9 PTPRD	CCCTGTATGGCTCAGAAGAGACT	GCTTCTCCCCATTCATTGTCTCC
9 PTPRD	GCCAGGGCATTCTCTGCTAAA	CAAACCCCCTACATCCCTCAG
9 PTPRD	GGAATGACGCTGAAAACAGGAC	TCCTTCTTCTCGTACTAATGCTTCTTCT
9 PTPRD	TGAAACAAAGTCTTCATTTCTCCACAGA	CTCTGGGAACACAATTCCACCATA
9 PTPRD	CAGCTTGGTGAGCATCACAAC	GTGTGAGCTTTTGCATGTTCTCTAAAT
9 PTPRD	CCCTATTTCCCTCCTCAACCAA	ACAATGTAGCTGACAGCCAAATCA
9 PTPRD	GGGCACTAAGTTGCCAATAGTAG	CGAATGTTGAGTTCGACCACCAT
9 PTPRD	CAGGTTCCTTCCACTGTACCAAA	GGATTATGAATTCAGGGTTGTTGCTG
9 PTPRD	AGACATGGCCAACAATGACACA	CGTTTTCCTCCTTTCAACTAACCTAACTT
9 PTPRD	CCCCCGCCCAATGTTATTGA	AGCATAAACCTAAAAACTCTGAGGAACT
9 PTPRD	GCCACCCCATCAATTTCTTTGTAA	ATCCAAGCCTTACCCAAACCTC
9 PTPRD	TTCTTTCCTTCAGCCCTAAGCC	GGCAAAGACTGCACCAGATGTA
9 PTPRD	CCCTCCCCTCTTCAACAACTCT	GGCCAACTAACATTTAGCCTTTCTTTTT
9 PTPRD	GAAGGCAGGCTTGGTACGTAA	GGGAAAATGGTAGAAGAAGTGGATGG

9 PTPRD	GTTGACAATTAACTTCTGTGTGGCT	ACATTTTCCAAAAGCTAGTGCACATTG
9 PTPRD	CTATTCCAGAATGTCAAGAAGTTGCAG	CCTTTTCTAGCTTTCTTACGTAGAGTCA
9 PTPRD	CATCGGGAGGGTTACAGGTTT	GCTTGGAAAACAGAATTTCAGCTGATAT
9 PTPRD	ACTGAGTTTCCCATTTAAGCACCAT	GCCTCAAATAATGTGGGAGAAATAAGTGT
9 PTPRD	GCAAAACTGTGAGTCTGGTGGAT	CCTGTGACAGCTATTGTTTCCTCTAATA
9 PTPRD	AATTTGTTAGTTCATGCATCCAAGCAAA	CCTCCCAACCTTCTTATTCTCAATCAG
9 PTPRD	CCACAAATCCCCAAATAACCACATT	GCATTATGGTGGATTTACAAACAAGCA
9 PTPRD	GAAGACATATTCTTGACCACTTTGGAGT	GGAGATATCTAGGAAGCGCAGAAG
9 PTPRD	CCAAGATGGGTATTGGAGGATGG	CCTGCTTCCTCACCTAAAACATCTTT
9 PTPRD	ATTCAACTTCTCTCCCATAACGGATG	TTGGGTTGTTCCCAGTTCTGAATTAA
9 PTPRD	AATTGAGCTGTACAACTTCTGGGAAA	CAATAAGGAGATCCCTTCACACCAC
9 PTPRD	GTAGAAACAGTAACAAGACCCTACCTG	CTTTGTGCAGCCAGTGGTAAT
9 PTPRD	TCCACAGGTAAGAAATCTTTAAACCAAGT	GGGCTTCCCTACCATTGACAT
9 PTPRD	CTCAGTTCTACAGGGTCTGTTGG	AGGTGCAATTCTCTGCTTTTCTATCT
9 PTPRD	GTACGCTCAACCACCTTCAACT	GTTCTCAACAAGCACTGAGTAAGGTA
9 PTPRD	CTGCAAACAAAACCACTCATTATGGT	GGAGGCAGTGATTCCAAGCTG
9 PTPRD	CCCCTGAGCCTGAATGAAGAA	GAGTGAAGAGTCTGACCAAGGAAAAT
9 PTPRD	TGTTGGTGGCAACACACTCAT	CTTTGCAGGGCTTTTCATATTGTTAAGA
9 PTPRD	TCCTCAATAGCTCTCTGACCAAGA	AACTATGAACTGGTCTACAAAGATGGG
9 PTPRD	AGTCTTACCTCCTCTCCATGCTC	TTCAATCCATTGCCTCCCTGTT
9 PTPRD	TCATTCTTCACTGTTGCCATTGAGAT	GAGCTGGCCACATATTGTGTTC
9 PTPRD	GCAAACCTTGTAAAGTGCAAATGTTC	GCCCTGAATGTGTCACTTGTTTCT
9 PTPRD	GTCTGCTTGCTGTGCAAATAACTT	CTGTCTTGGGAGATTCCAGAGAATTA
9 PTPRD	TTGAAAGGCATGGCGGAGTTA	GGAAAGGACAGGTCATCTGTTTTAAGAA
9 PTPRD	CCTATTGAAAGACAGCAGATCAAGAC	GAACAGCTGGAAAAATGGACTGAA
9 PTPRD	GCTGTCACAGTGATCCGGTA	CCAGTGGAAAAACAGAATGGCATT
9 PTPRD	CAGTGTACTTGATGGAGTATTCAGTGAT	CCTGTCCTTTCACTTAAATCCTCCTTTA
9 PTPRD	AGGTATCAGTGATGTCTGCATTTCC	ACTTGGGAACATTCAAACTTGGAAGTAA
9 PTPRD	GATTTCTCTTTGGGACAACAGTCAAG	CCACAGTATATCCTAAGGGAATTCAAGG
9 PTPRD	CATTCGCGTATCTATTCTTTGGTTTGT	CTGGTGGTTTTCAGTATTGATTTTGTGT
9 PTPRD	CAACTTACCCTGGCATCTGTGA	GTCACCAATACTGGCCAGCA
9 PTPRD	CTCCATATCCTTGCGGCCAA	GTCTTACCACTCCATCCCATGTG

9	PTPRD	AAAACAGACACTGAGAAAGGCCTAA	CACCATCATTTCCTTTGCAGGTTC
9	PTPRD	ACCAGGGTAACCGGAATCATCT	AGCCACAATGTATAGTGAATTCTGTAAA
9	PTPRD	CACCTAAGGACAGCAGAACAATGA	TGTAGGTCCTGTCCTTGCAGT
9	PTPRD	GGATGGGCTTTCTAGAGGCATT	GCTATGTCAACACTGGGTGTCAT
9	PTPRD	GCAATGACAATGCAGATGATAAAGACC	GAAATAGACTCTGCTTTTCCTCAGAAGA
9	PTPRD	GAGTAAGGGCTGGTTGCATACA	CTGTTTTGCTAATATTGCATCACACACT
9	PTPRD	AAAGGTTAAAGTAAAGCCACGACTCA	GCCAGCTAGAAACTTGTATGCCTA
9	PTPRD	ACAGTGATCTGTGCTATTGCTTCA	GTCACCAATGCCTTATGTAAAGTGG
9	PTPRD	CAGATCTTCTGCCCCCAACAT	GTTATCATTTCCCTTGTGTTTTGTTCCA
9	PTPRD	CGTTTCTATTTGTGTCAGCTTCTGAATG	CCATGTAACTTTAATGAGAGCCCAGA
9	PTPRD	TGGTCTTCTGTTTGAACCATATAGTTCC	TTGGGCTTTTCTTCTCTATTTTCCTCAT
9	PTPRD	AAACACCACCACTTATCACTGCT	GGATTTATTGACTTCATCGGCCAAGT
9	PTPRD	TGGCCAAACTGTTCTTTTGTTTTATGG	CGGGTATGTGGCTTTCCTTCTAAAG
9	PTPRD	CTCTCGGTCACTACAGGAGTTC	AATCTTCAGTAGATACATAATCCTGTCATTTTC
9	PTPRD	AGCAAGCAAGTAACGTAGAAACCA	GTTGCCTCTTTCATCTGCCAAG
9	PTPRD	TTTAGGTCTTGGGTCTCCCGTA	GGAGAGCAGCGGTAGTAGGATAA
9	PTPRD	AGGGCACATGTAAATATCAAGGCTTT	CATATGAATCCACAAGGGTATGCCT
9	PTPRD	GTAATCAGATCCTTCTACTCCACGGATA	CCTATTCAACAGCGTCTAGCCA
9	PTPRD	GAAGATTGGCACTGATAAACCTTGAG	GGGTACACTGCATTCAAACTGT
9	PTPRD	AGCCTGCTAGAAGCTACCTATACC	CTCACCGTCACAGCCTACAC
9	PTPRD	CGAGCACCATCTCCTTTGGTT	CCAGAGCATTAGTAGCATGTTTTGAAT
9	PTPRD	GCAGGTTTGCTTATTGGTGAAGG	CTGGGTGCTTCTACTGCAGAAATA
9	PTPRD	CTTGACTGCATGGTTCTAGCTGA	ATGACCACATTCTCTTATTGCTTCCA
9	PTPRD	GGCCGTAAGCAGACAAATCCTA	CTTATTGTTCCAGCTGACACCAC
9	PTPRD	CTGGTTTTAAGCCAGTGAGTGTCATA	CACTTCAACCTCCGTCCAGT
9	PTPRD	GGACAGGTGGTTGCCAAGATA	GAGCATCATACGTCTTCAGGCT
9	PTPRD	CAGAGGTACATAATTTCAGAGGCTTACC	GTTAAAGTCTCATGGCGCTCAC
9	PTPRD	agtttgATGCTTTCCCCACAGTA	TCCAGACTGTCAAAATGTTAAGAACACA
9	PTPRD	CTTATCTGGCCATGCTGTTTATTGG	GCTCTGATAGGGTGGGACAAG
9	PTPRD	CCCACTTTGTTTCTGGCTGAG	CTCTTCAGGGCTACCGTCTAAAAT
9	PTPRD	TGTCTGTACCATAGCTGGTCGT	ACCCAGTAACATCGCCTTTGT
9	RALGDS	CTGAAGCTTCTGAGCCCCAA	CACTGCCCCTCTGCTCATAG
9	RALGDS	GCTCAGGGAAGGTCTCTCCTC	TGCTCATCCTCCTACCCCAAT
9	RALGDS	CCCACTGTCCCCATTTGCT	CTCTGGCCCCAGGAGTTC

9 RALGDS	AGCTTGATCTGGGCGATCAC	GGAAGGCCTGGATGGGAGTA
9 RALGDS	CATTTGCCGCCAGTGGA	GTCTCAGGGCTCTGCAACT
9 RALGDS	CCCACCTGCTGGTTGTAGAG	GGGAATCAGCCTCACAGTCAT
9 RALGDS	GAGCTGATGCCGGAGGTC	AGGAAGCCCTGGATCCTGAA
9 RALGDS	AACATCAGCTCTGGATCAGCAA	GATCTGGTGGCAGAGCAGTTTA
9 RALGDS	GCTCACCGCATCCATCAGTG	CAGTTCCATCATTACAGCCTTCCT
9 RALGDS	AACCTGAGAACAGATGAAGCCAAA	CCACTGTCACCCAGTTCAACAG
9 RALGDS	TCTCTGCAACCACAGGTGAAG	AGCCAGCTGTGGGACTAGAA
9 RALGDS	TGATGACACAGTTGGCCACA	GGCAGGACCAGGTGACATTTAG
9 RALGDS	TCTAGAAGCTGTGTTCAGGATCCA	AGGAGATCAACATCAGCTTCGTC
9 RALGDS	CACCTTCTTTTCCTGGCCATCA	CCAAGTCCTGTGACCAGCTC
9 RALGDS	TCCCCGCTGCTGAGGTA	GGGTTGGGATGTGTCCTTG
9 RALGDS	GCCGCCACTCCACCTTG	CTCTTTCCTTCTTTGCCCTTCTGA
9 RALGDS	CCCTCCTCATTCACCCTCTG	CGGACTTTCCCTGCCTCAAG
9 RALGDS	GCATGTTGAGCTGCACGTA	CAGGCCTCTGACTCTGCTG
9 RALGDS	GTGCAGTGGAGGGCTCAT	TGGTGCCAGCCTTCCAG
9 RALGDS	ACAGGAAGATGGTGACGTAGGA	CTGACTGTCTGTCCCTCCTCT
9 RALGDS	CGCCCTCTGGTCTTCTGACTTA	CTTTTCCCTGATGGTCCATCCC
9 RALGDS	ccccaTGTACTCTCCCACTGT	ggtagATGTGACGCCCTCA
9 RALGDS	GTGAAAAAGTCCTGGGAGGGAT	ATCGGTGAGGAGCTGATCAAC
9 RALGDS	GCAGGGAGATGGAGTAGATGACTC	AGTCTGTGCCCTATCTGGGAAA
9 RALGDS	CTTGACCTTCACTCCCTTGGT	CATCCCTGATCACCCCTCTG
9 RALGDS	CCACTCTGGTCCATAAGTGCTT	GTCCTCAAGAAGCGGACCTT
9 RALGDS	GGTCTAGCTGCGTGAAGCTG	ATGGTGCAGCGCATGTG
9 RALGDS	TGAAGCTGTGCAGCACCA	cgcTGCGAGCCTTCCAT
9 RALGDS	GGAGCTCCTACAGAGGCAT	GTGCCGGATCCTCAAGAACTT
9 RALGDS	GAGGATGGCATACAGTGACGAG	CCTGGGTGTCTGTCTCATTAGGA
9 RALGDS	AGGCACCGTGGCTAGAGA	CCTGTCCTCTACCCTGGCA
9 RALGDS	GTCCCATGTCCCATGAATCTAAGG	ACTTTCTGACGGCCATTGGG
9 RALGDS	acGCGGAGCTGCGTAAG	CCACCGAGGCTGAGGATG
9 SYK	GTGAGGCATTTTGGAAAGTTTCTTGT	GCATGACATTTGCTTCTGCTAATAACT
9 SYK	CAATGACCCCGCTCTTAAAGATG	ACAATACCTGTTCTGCTGCAAATACT T
9 SYK	GCAGAACTTGGTCCCCTCAAT	GCTTCCCAAGGAAATCAGGCT
9 SYK	GGTCCTCACCAAAGTTCTCTGT	CCAGGTAATCTTCTGCCTCCTC
9 SYK	GCCCTTCTTTTTCGGCAACATC	GGTGTAGTGGTGTGCCTTCC
9 SYK	CATGAGTGATGGGCTTTATTTGCT	GCGTAGGTGCCATTCAGCTC
9 SYK	GCACACCACTACACCATCGAG	CCGGTTGAAGGGCTTCTTGAG
9 SYK	CTACCACTCCCAGGAGTCTGAT	GCAGGTTCCATGTCTGCTTCA
9 SYK	gtctTGGAGTGGCTGTTTTGTTTT	CATCCGCTCTCCTTTCTCTAACA
9 SYK	TGAAGGAAAACCTCATCAGGGAATAT G	GTTGCATGTAGCCAGGAAGTCT
9 SYK	GAAAGGAAGTGAAGTCACCGCTA	TCACAGTCAGTGTCAGGTACTCA
9 SYK	cccggccCACAATTTTATTCTTAA	TTGCTGGAGAACTTGTAGTAGTTGAT G
9 SYK	AAGTGGTACGCTCCGGAATG	AGAAAATGAAGCAGGACTGGCT

9 SYK	GCACATCCTGATCTCATTGATTGATCTG	CATTCAGTGGTATTCCTAGTGACACTT
9 SYK	CGGTATGGTTTACTCTGCTTTGCT	TCCTATCAGGACAATTTGCTCAGATTC
9 SYK	TCCATGGAAAAATCTCTCGGGAA	GCCAGTCACAAGGAAGCTGAATTAT
9 SYK	GATGATGCAGTTCCATCCTCTCT	AGCTTCCCTGTCTTGTCTTTGTC
9 SYK	GTCGTGGAAGGGATCCTGTATTT	CACTTAGTTGGACAGCAAGCAG
9 SYK	AGGTGCTGCACTATCGCATC	TGCCCTGTACTCTACCCACAA
9 SYK	TTTTCACAAGCACATTGACAAACAAGA	CCTGTGATTGCTCCTGTGATCAAA
9 SYK	GTTTACCTGGACCGAAAGCTG	CATGTCTAGAACCTGAGCTCATGTG
9 SYK	TTTTGTGGATGAAGAAAAACAACCTGTT	GTCCCTTGAATGAAAGGAAGTCTCA
9 SYK	GCCCCAGGTCGTATGTTTCTT	AGTTCTTTGTCTTCCAGCGTCAG
9 SYK	CGTGAGGAGCATGGTTGTTTG	GCCAGATCTCTGTGCACAAAATTG
9 SYK	GGCATGAAGTACTTGGAGGAGAG	CTGCTGCTCTGAATATTAGCTAAGTTGT
9 SYK	GCTCTCAGTCACTATTTGTCTATTTCCT	ACTCCAGTGTCTTGGGAACTCA
9 TAF1L	CCGCATCTCTTCCCGATGTTT	TCGCACATTTCGAGATGAAGAGG
9 TAF1L	GTGATGAAATGGCCAAGAATCTGG	TGCTGGATCTCTGTGATGAAAAACTTAA
9 TAF1L	GATCTTTGCCAGGTTTCCGTTTAT	TCAGGTGGTGGAGAGTTGTTTTT
9 TAF1L	TCTCCATCTTTGCCTGTGAGAT	TGAAAAAGTACTCATTTGGTGCACTCT
9 TAF1L	TGTCTCACAGCGAACATACTCTTTC	CTACGGCGAATGCTACTGGTA
9 TAF1L	AAGCGAGCTAATTTGTCTTCTTTCTCT	CATCTGGAGCTAATTGTGAAAAACAGT
9 TAF1L	GCAAAGGTTGGACTGAATGGG	CCTTTGGAGGGAATATTATCCAGCATT
9 TAF1L	GTCAATGAATGTTTTGGCCCATTGT	CTCGGCCAATGGACCTACAAA
9 TAF1L	TGATTGTTTCCTGATGCTGCTGA	ATTGAGAACATGTTGCAGAACAAGAAAA
9 TAF1L	TCCTTCTCATCAGGAATTTCCAAAATGA	ATTGACAATGAGGATCTGGTGTATGG
9 TAF1L	GGGCTGCCATAATTCCATAGCA	ACAGGTGTCATCAGGGAGGA
9 TAF1L	CATCCCAAATGATATTGTCCTCCCA	TGGCTTACAATGTTCAGCAAGGT
9 TAF1L	GCTCCTCCCATTCACGTGAC	GTCAGCGTATCTTTGACCTACAGA
9 TAF1L	CTGAGACATGTTCTGCTGTGGT	GGCACTTGATCCCAATGATGAGAA
9 TAF1L	GTAGAGGCATTTACGCACATTTTCA	GGTGACGCTGTCATCCATCC
9 TAF1L	CATCATCCAGAGTGGGTGCAAA	CACAGCTGCATTGGGAGGAT
9 TAF1L	AAAGGGTGTGTATTTGGAAGATCTCTC	TTGGAACCACTGTTCATTGTGACTAT
9 TAF1L	CCTCAGTTGATGACAGAACCTTGT	GAACAGGCTCATTCTGGAGAGG
9 TAF1L	CTTGATCCACGGGCAAATTTACTC	GGTGTGCCTGAGGAAGAGATTAAAAA
9 TAF1L	GGTGGATGGACTTATGAGGTATATTCAA	GTCTTCGGGAAACAGCTAATTGAGAAT

9 TAF1L	CCTCCCCATCCCAGATGATAGA	TGAGAAAGACACAACATGAACCTGT
9 TAF1L	CTCAAGTTTCCTAAATTCCTCCATCATTC	GAGTGGCTGAGTGGCGTTAT
9 TAF1L	ATTTTCTGCGAACCTCATGCAC	ATGTGCCACCTTCGAAACCT
9 TAF1L	CTGCTCTTCTGTCATGGCAAC	GAAAATGAAAGAGCGTCCTGACCTA
9 TAF1L	CACATGCCCCACATTTCAGTTT	GGAGGATTCAAGAGCAACTGAGG
9 TAF1L	CTCCTTTTCCTGGTTCCGCTTAA	CGCATACGGACTACAAAAGATGAGA
9 TAF1L	CCTCATCATCATACCCTTCAACATCC	GCCTCTGTATTTCAAGATGAGAGCAATT
9 TAF1L	CATTGTGCGCACCACATCAAT	CAGAAGGATGATAAAGAGCCACAGG
9 TAF1L	GCTCCTCACAGCTCCCATAAC	TGGAATAAGACCCAAACAACCCTT
9 TAF1L	GTTCCTGTCACTGTCTTCTTCACT	GCCATGAAGGGCAAGTGTCTC
9 TAF1L	TTTGAATCCATCCCTGTGCGT	TTTATTTACCGCCTTTTCTGGAAGAGT
9 TAF1L	GCAGTGGAAATATCCAAGACAGACA	GAATTAGAAAGCCTGGACCCAATGA
9 TAF1L	CACCCAGCATATCATACCACAGT	GCTGATGATGAAATCACGATGATGG
9 TAF1L	TCTGAATGGGAAGGAAAGGCTTTTT	CCTAACTCCAGAAGGGCCAATATG
9 TAF1L	CATCCTCAGATTCTGCCTGTGT	GACCAAGATGCTATTACCTGTGTGT
9 TAF1L	GAATCCTTCACCACACCCTGT	CCCAGAAGAAGAAAATGAGGAAGATTTC
9 TAF1L	GGTATCATACATATCAGGAGGCTGAGA	TTGTGAACATCTGTTACCAGACAAT
9 TAF1L	CACTGATGTTGCCGAAAAGGAT	CTGTTGTTTTATTTCCGGTTTATGCGA
9 TAF1L	GGCGGTAAATAAAAACCTGTAGAAAGTC	ACAAGACAGGGTTACTATATTCGGGAA
9 TAF1L	GGGAAAATTTGGACTCCACAGGAA	AGCAGATCCAGGAGGTGGAAT
9 TAF1L	GCAAGATGATGTCTTCTCCACTTTCAG	CCTTTACCACTCGGATTATGATGAAGAT
9 TAF1L	TGTTCTCCTGAAGCATGCTGAAG	ATGCTGGGAGTGACGAAGAAG
9 TAF1L	GCTGTCCGAGTCTGACATGAT	CCCCCGCCTTTATTCAGCTT
9 TAF1L	TGGCCAACCACAAAAATATCCACTAA	CCAATTACTGCAGGCACTTGAGA
9 TAF1L	CTCAAGTTGAGTCAAATGCTCATCATAC	AAGTATCAGAGTCGGGAGAGTTTTCTA
9 TAF1L	CAGCGTGAACTTCATCATCAATCTTC	TGTGCTTATTATAGCATGATAGCTGCAA
9 TAF1L	ACTGGAGCACGAAAAAGGTTGT	GCATGGCAACCAAGATAAAGAACTATT
9 TAF1L	ACTGTTGGCAAGAATAAGGTTTACATCA	AGTTCCAGATTCTTGGCCATTTCAT
9 TAF1L	GCCAGCATCCTTCAGTCGTT	CTAAAGCTCTGCGCTGACTTC
9 TAF1L	CTGGCTGACTTCTGATTCTACTGAG	CAGAATTTCGACCTGGAAAAGTGTT
9 TAF1L	CAGCTGGATAGCAGAGAAAGGATT	TGAAGAGGAAGGAACTGTACAACAAC
9 TAF1L	CAATGTCTTCACAATCAGCATCATAGTC	ATGAAGGGTGGATTAGGAGTACAGAA
9 TAF1L	CCTCATACAGGACACTGGCTTC	GCTGGGTGAGGAAGACTCTGAT

9 TAF1L	TTGGAGATGTTCTTACGTATGGTCTCTA	TCACCCAGAAAATGATGGCAGTT
9 TAF1L	GGTCCAAAAAGATGTAGGAAGCGT	AGATGGGACCTCAGGAAGCA
9 TAF1L	CTCATTGATGTCTGAATAGTCTACAGCA	TTGGATGATGAGTGTAAGAAGCACTT
9 TAF1L	CCGTGAGTTCAGTGATTAGGCT	CATCTGGAGGTGGCCCATTTA
9 TLR4	AGTTGGGAGACCATGCAGTAAAG	GGATTAAAGCTCAGGTCCAGGTT
9 TLR4	CAACCTCCCCTTCTCAACCAAG	CTTGTGCAGTATGTAAAAGTGGATTCAT
9 TLR4	TCACAGAAGCAGTGAGGATGATG	CAGAAGTGAGGGAAAGTTCAGAGG
9 TLR4	CTTGTATTCAAGGTCTGGCTGGTTTA	TGCTAATCGGAATTCTTCAATGGTCAA
9 TLR4	CTCTAGAGGGCCTGTGCAAT	TTTACCCTTTCAATAGTCACACTCACC
9 TLR4	TGTTTCTTCATTTTCCCTGGTGAGT	TTCCCACCTTTGTTGGAAGTGAA
9 TLR4	TGAAACTCAAATCTCTCAAAAGGCTTACT	GCTGGTTGTCCCAAAATCACTTT
9 TLR4	CTTGAGTTTCAAAGGTTGCTGTTCTC	GAATGCTGGAAATCCAGATGTTCTAGT
9 TLR4	AGCATTTTCCAAGTAGTCTAGCTTTCTT	ATGCCCTGCTTATCTGAAGGTG
9 TLR4	GAAGTTGAACGAATGGAATGTGCAA	ACTTATAGACCAGAACTGCTACAACAGA
9 TLR4	GGTCCTCAGTGTGCTTGTAGTA	GCTCATTCCTTACCCAGTCCTCA
9 TLR4	CCTTTGTTATCTACTCAAGCCAGGA	CTTTCGGCTTTTATGGAAACCTTCA
9 TLR4	CATTGCTGCCAACATCATCCA	CTCCACCTTCTGCAGGACAAT
9 TLR4	TTGATGTCTTTGCCTATGCACA	GCTCTGATATGCCCCATCTTCAATT
9 TLR4	TTTATTCCTGTAGGTGTGAAATCCAGAC	GCCACCAGCTTCTGTAAACTTGATA
9 TLR4	AGTTCAAACTTCTTGGGCTTAGAACA	ATTGAAGATGCCATTGAAAGCAACTC
9 TLR4	CCTGGGAGCCTTTTCTGGAC	GATTGTGAGCCACATTAAGTTCTTTCAA
9 TLR4	CCTTGACATTTCTCATACTCACACCA	GAGGTCCAGGAAGGTCAAGTTTC
9 TLR4	TTCCCCATTGGACATCTCAAAACT	CGCAAGTCTGTGCAATAAATACTTTGAA
9 TLR4	GCAGTCGTGCTGGTATCATCTT	CATCCAGCAGGGCTTTTCTGA
9 TLR4	TTGGACCTTTCCAGCAACAAGA	GCACCTGGTTGGATAAAGTTCATAGG
9 TLR4	TCTCTCTTTAGACCTGTCCCTGAAC	CCAGAACCAAACGATGGACTTC
9 TLR4	GGCACATCTTCTGGAGACGAC	GCAAGAAATGCCTCAGGAGGTTT
9 TLR4	CCTTCCAGATATCTTCACAGAGCTGA	GGAGTTCAGACACTTATAAGGAAACGTA
9 TLR4	GCCACAACAACTTCTTTTCATTGGA	AAGTACAAGCAAAGTCATTCTGAGTAAGA
9 TSC1	CAGACGAGCTGGATCGCA	GTTCATATATGTTCTGCCCTTGTCTCT
9 TSC1	TGGGAACAATGTCATCAGTGG	ATCCTGACCACCTTGCAAGAG
9 TSC1	TCAGCCATTACCTTGTCATGTGG	AGTATCATCCATTGCCCTTTTCTTGATT
9 TSC1	GTCTACATGTCCATTCCTTACAGCAT	GACATTTTTGGCCGTCTGTCA

9 TSC1	TGGTTTCTTCAGGCACCATGA	CGTTTCCTGTTTGACCTTTTCTCC
9 TSC1	tcaaAATTTCCCTGTCTGCCGTTA	TCCTTTTTGCGTTCTCATTACAGTATGA
9 TSC1	ACTTCTTCAAAAGTCTCCAGGTTTTCTT	CTCTTCTCAACGGGTTCCTTTTCTA
9 TSC1	GGGTCAGGTTTTATCAACTCATAGCA	GACTTCAGTTGTCTTTGTTTCTCTTCAG
9 TSC1	TCCAGAGACAAAGTTGCAAAACAGATAA	CAGAAGCCTCATATGAAGATGGCTAT
9 TSC1	GCTGAGATTTGGTGAGACACAGA	GTTTGCATTTCTTGACTTTCATTGCATT
9 TSC1	GCAGATTCAGGTCTGCCTCAT	ACTTCATGTCCACGTCTCTTTGG
9 TSC1	AGAGTGCCCCAGTCCCTTA	GCCCATCATTTGTCATCAGGAAG
9 TSC1	TTGCTTTCTTTAACAGCTCCTCAGT	ACCAGTATCTTCACTCCCAGTCC
9 TSC1	TCGTCGGAGGTGGAATTTTACAA	CTTTACACTCCTCCCTGGACAAG
9 TSC1	AGGCTTGCTTTGGTGTGTCA	CCTCGAGGAGGCTTTGACTCT
9 TSC1	GGAGACTGTCTCGGTAAAAGGG	ATACCGACTGCCATTTCTTTTGTTT
9 TSC1	TGCTTCCAAGTGGACTGATTCTGTA	ACTGGATCCAAGGACCATGAAC
9 TSC1	CTAGTTTCTATACCTTCGAGGGTCCA	gttccacaAACATTCAGCCCTTTATAAT
9 TSC1	ACTGCCGCTCCGTCTTTTAG	AACTGCAGAACAAGCACTCAGA
9 TSC1	CCCTGGCATACCTTTGTGGTA	TCGGATTTTTCACTTTGCTCATGTTTTT
9 TSC1	GTGGCTCTAAAGTCAATCTCTTCTTTCT	AGAATGGCCCAACAAGCAAATG
9 TSC1	GGAGTCCAGCATGGCAAGAAG	GGGCCATTTAGTGACTGTTCAT
9 TSC1	TGCAAGTTAACACTGATTGACCATCAT	AAAGACAGAACTGGGCAAAGACTT
9 TSC1	GGGCTGGATTTGGAGCTAAAGTA	GCTGCAAATAAAAGTCCAGATACGTTTT
9 TSC1	CTGGAGAACATGGCTTCTGTTTTT	ccTTCTCAGTCCTTCTTACATTGTCTTT
9 TSC1	CTGCAGCTGTCCTCTGAAAGATA	GAAAGACCACCTTCTTTTGGAACAG
9 TSC1	GCCTGGAGTTTGACATCCTCTAGATAT	CGCCTATCGGAAAGAGCTAGAA
9 TSC1	GAGAGCAGGCACACTAGTTGA	CCCCTGTGTTCTTCTCTTCCATTTTAG
9 TSC1	CAGCCTGTCTAGTCAGCAGTAA	GGAGAAAGATGGCCTCCTGAAAAA
9 TSC1	CTGCTTCTGCTTTTTCTTCTTCAAGT	CTATATTCTGGCTGGTCTGTATCTTTCA
9 TSC1	AGCTATCATGCTGACCCAAAACA	CCAGTTACTCTATGAGCGTTTTAAGAGG
9 TSC1	CTTTGATCACCTTGCGGAGGA	CACCCTCCCTCTGCTTTACAAT
9 TSC1	AGCTTCCTTGCTTTAAGTTGCCT	CATCTTGGAAGCATAAGCTCTCTCA
9 TSC1	TTAGTAAAGAAGGCAAAAGAGGTGCT	GACTGCCCTTGTTCTTTTACATTTTCA
9 TSC1	TGCCTGGTGCTGCAGTTTATA	TGGAAGGTTAGTCTGCAGAAAGAAC
9 TSC1	TCCTGGAGCTGATTGTATCTAGCTT	GTTGGAAGACAGCTAAAATGATGACATT
9 TSC1	CATATAACCCAATTAGAAGAGGCAAGCA	ATGTGCAACATTTTTCGTCTTGTGATAT

9 TSC1	GGGAATCTTGGCTTCCACACC	GGGCTCACTTCCCAGTTCAAAA
9 TSC1	CGAAATAACTCTCGAGCCTTCATACC	gcGAGCTTTCTACCCCAGAGA
9 TSC1	CCCACCGACTGCTGAATGG	GGCACAATGAAGAGGCATCTG
9 TSC1	TCTTGGTCTCACCGTTGTGG	CAGTGTGAGTGAGTGTCAGTGTAAT
9 TSC1	CAGGGATTTGCAATAAGTGTCAAAAA	CACACCCTTACAGTAAAGTCTTTGGT
9 TSC1	CCAACCTAAGACATACATACCAGTTGT	CCACTGCTGATGTACTTTATTAACTTCC
9 XPA	GCTTGCACGAGCCAGTCT	GGCGAGTATCGAGCGGAA
9 XPA	CCTGGCGCAGCATCAGT	AGCTGGGAGCTAGGTCCTC
9 XPA	ACAGGAAGAATCTAGCTAAAGGCTTTTT	GTCAAGAAGCATTAGAAGAAGCAAAGG
9 XPA	CTCGGTTTTCCTGTCGGACTT	TCTTTGGTACCTTTGGATTTGACAGTTAT
9 XPA	ACTTGTTTTGCCCTAAACCTACACA	CGAAGAATGTGGGAAAGAATTTATGGAT
9 XPA	GCAGTTATCACAAGTTGGCAAATCAAA	AGTTCATTTTCCTTTTCTTAGGACCTGT
9 XPA	TCTTTCCCACATTCTTCGCATATTACA	TTGTCTTTTACTTAAATGTATTTATGTCAGGCA
9 XPA	CACACTCTGTAAGCAAAAGCCAA	GAAGAAGAATCCACATCATTCACAATGG
9 XPA	GTAGAGTTTCATATCACCCCATTGTGA	AGAGATGCTGATGATAAACACAAGCTTA
9 XPA	GATATTCTTGTTTTGCCTCTGTTTTGGTT	GCTGTGTGTGCCCCTAAGTT
9 XPA	GTTATGGCATTATTTAGCATCACTTTGCAT	CACAGGAGGAGGCTTCATTTTAGAA
9 XPA	CCTGGTTGATGAACAACTTTTCCAATTT	ACAGGCTTACCTACTTCAATTGTTGAC
9 XPA	GGTTTCATTCATCTATGAAGATGTTGCTT	GACTTGTACTATGTGTGGCCATGA
9 XPA	TTCATATGTCAGTTCATGGCCACA	GAATTGCGGCGAGCAGTAAG
9 XPA	ACAATCGTCTCCCTTTTCCACAC	ATGTACATGGCTGAAAGCTTGAT
10 BLNK	AGGTAGAAACTTCTCATCCAGGTAACA	ACAGGCAGCTTCAAAAGATGGT
10 BLNK	GGGAAGCCTTTGGCACAGATTTA	GCAAGACACTTCCCAGTAAGCC
10 BLNK	GTGAGCCTTGCTTTCTCTGAAG	CTTTCTGGATCTCAATGGCTTCCA
10 BLNK	CACCCCGCAATCTTGGACA	GAACGCTGATGACAGCTACGA
10 BLNK	CCTGGTTTCCTGCTCTACTGGA	GCTGAGCACAGTGTCTGCAT
10 BLNK	CTTTTAGAGGACATGCTCATCCTTCA	CTCTGACATTTATTTCCACATTCTGCT
10 BLNK	tccagTGAAACTGCATCATCTCAA	CCTGTGATCGAAAGTCTGCTG
10 BLNK	GTTTGATCTGTGCAATGCCTCTT	AATTAGAGTTAAGTAGGGACCATCCCAT
10 BLNK	GTATCCTTTCTCCTCATCTCTAGCATCT	AGCAACAAAACAATATGCCTTGGG
10 BLNK	CTGACCTCTTCACCATTTTTCTTTCTG	TTCGGAAAAGCTCTGGCCAT
10 BLNK	TGGAATCATGGCCAGAGCTTT	CCTTTGGAATGTAATATAGGTGTTCTTCACA
10 BLNK	GGCTGACCACCAGCATGTAAT	ACACTTGTGCCTTCTGCTTCTC
10 BLNK	CATCCTCACTCCCCAGATCTCA	GGACTGAGTAGTGTATATGTTTGTGCAG

10 BLNK	CTGGCCTTCTGTTCCCAATATTAAGA	GATCAACCAAGCCAAATTCCTCAAC
10 BLNK	TTTACCTGAAGCTGTTCCTGGAG	TTTCCCTGAGTGTGAATTTTCCTCAA
10 BLNK	gcttcgTTGTACTTACTTGGCAGA	CAGCATCATCAGTGTAAACAAAAAGTCA
10 BLNK	TTCAGGTGCTTAAGGCGACAA	CCCTCCCATACAGTCAACTATGAATGTA
10 BLNK	GACATGCTCTTTCCTGCAGTTTC	AATTGAAGGACTTTTCTGATCCCTTTCA
10 BLNK	GCTGTGTTCACCATAGCTGGAA	CGTTGTGTTTTAACAAAGCTCATCTCT
10 BLNK	GGGAAAGTGTCTGAAGCAATGG	TGCTGAAATCATCAGGAATCATCAACAT
10 BLNK	CTGACTGTCAATAAGAACCAAAGGACT	AGGAGTGGAGCTCAAATTTAACACAAA
10 BLNK	GAGGATAGCGTATTTCCCTTTCCA	CCTTTTGTCTCATGGTTGTTATTTCCAG
10 BLNK	TGTTGGATTTTTACCTAGCACTGCAA	GAGGGTCAAGTCACAGACAAGAAG
10 BLNK	GGAAACACTGGTGACTGCACA	GGTTCTTCTCAGCTGATGTTGCT
10 BLNK	CAGTATTTCTGAGATGCCATAAGAGCA	CTGGACAGTTATTCGTGTCTCTTACAAT
10 BMPR1A	ACAAAGTATTAAAGGCCATCTGTACCTG	TTCTCCCTGGTCATCTTCTTCTATGAT
10 BMPR1A	ttttAGAACTAATGGACATTGCTTTGCC	TCATATGGCCCCTCCCTTCTTT
10 BMPR1A	TGGATAGGATTCTTTCTGAGGGAAGG	CTTCATCCTGTTCCAAATCACGATTG
10 BMPR1A	GCATCTCAAGCAGACGTCGTTA	ACACCATTCATGTCTATAGGAACGTTT
10 BMPR1A	ATTTTATTTTTGGCCCTCAACTTGGAC	GATGTAGGGCTGGAAGTGGTTT
10 BMPR1A	GCTGGACGAAAGCCTGAACAA	CAGAAACTACTCAAACTCCCACCT
10 BMPR1A	AAAGTCGGAGCATGCTTCTCAA	CAGCCATAGAAATGAGCAAAACCAG
10 BMPR1A	TTTTTGATGGCAGCATTCGATGG	ACAGCGGTTGACATCTAATATTTGACA
10 BMPR1A	TTCTTGTGTAAGAATCCGTTTTAGTTCCA	GGATCACTCGGTACCATGTTGTAATAT
10 BMPR1A	GATCGTGGAAGAATACCAATTGCC	GTGGCATTTACGAATCACTTCTTCA
10 BMPR1A	CGGTGGAACAGTGATGAAGTGA	CCAGCATTCTGACATTAGCTTCAAAAC
10 BMPR1A	AACTCATCAACTGGACAGGTTTCATAG	AGACCACAGGCAGCTGAATAAG
10 BMPR1A	TTCAGCTGCCTGTGGTCTG	CAAAGAACCACTCACCTGTTGAATTTAA
10 BMPR1A	ACAGAAATTTATGGCACCCAAGGAA	GGCAAAGAACCACTCACCTG
10 BMPR1A	CTGGGCCTTGCTGTTAAATTCAA	AACAAGCATATGCACATTTTTAGAATTCCAT
10 BMPR1A	AATTGTCACGAAACAATGAGCTTTTCA	TGCTAAGGTTACTCCATTTTCTGACTTT
10 BMPR1A	GATGAAATCAGACTCCGACCAGAA	CCTAACTCTTAAGAAGGGCTGCATAAAA
10 BMPR1A	GACTACCCCTTTGCCAGTCTT	ACTTTTTCGCCACGCCATTTG
10 BMPR1A	GGTAAAGGCCGATATGGAGAAGT	AAATTGACTGAATCAGTGTGTACCCA
10 BMPR1A	AGCAAGACCAATTATTAAAGGTGACAGT	ACACTGATTTACCTTGAACACGAGAAA

10	BMPR1A	CAGATTATTGGGAGCCTATTTGTTCATCA	ACAGGGATGAGTTGAAAGAACAGAAG
10	BMPR1A	GGCCCCTTTCACTCACTGAAAT	GGTTACATAAATTGGTCCGACAACATTC
10	BMPR1A	CCAGCTACGCCGGACAATA	AAGATATTTGAGAAGCATGCTCCGA
10	BMPR1A	CTCTTTACTTTTCAGTGTCTACGAGCA	TCTAGAGTTTCTCCTCCGATGGTTTAA
10	BMPR1A	CTTGGTGTCAGGCGAATTAAAGTTT	CCAGGAACCTGTACCTTTAATGTCTG
10	CYP2C19	GTGACTTCATTTGCTGTTAACTGTATCTC	GAAATGGCCTCTTCCAGAAAACTC
10	CYP2C19	TGAAGGAAGCCCTGATTGATCTTG	CCGAGAAGCTCTGCTAGTCTG
10	CYP2C19	CAGCTCAGTTCACCTATGTCTCTTG	CCTGCTGAGAAAGGCATGAAGTAG
10	CYP2C19	TCACTTTCTGGATGAAGGTGGAAATTTT	AGAAGGCACATGTAAGTTCCAACT
10	CYP2C19	TTTGCTTTTAAGGGAATTCATAGGTAAGA	CCAGGATGAAAGTGGGATCACA
10	CYP2C19	CAATGTGATCTGCTCCATTATTTTCCAG	TGTAAGTGGTTTCTCAGGAAGCAAA
10	CYP2C19	TGAGGAGTAACTTCTCCCTATGTTTGT	GCAAATCCATTGACAACAGGAGTT
10	CYP2C19	GATTGACCCAAAGGACCTTGACA	ATCACAGATAGTGAAATTTGGACCAGAG
10	CYP2C19	TTTATCCATCAAAGAGGCACACACA	ATTGAAAGGAGAAGCAAACATGAGAGA
10	CYP2C19	CCTTTTGTGGTCCTTGTGCTCT	ACTTACATTGGTTAAGGATTTGCTGACA
10	CYP2C19	TCCCAGTGATTGGAAATATCCTACAGAT	ATCTCTTGTAACATTGTACCTCTAGGGA
10	CYP2C19	AAGCAAAACCAACAGTCTGAATTCAC	TCTGTGATCATACCTGTGACCTCT
10	CYP2C19	AACCCTGAGATATGCTCTCCTTCT	GCTGCGGGCACAGAAATTTA
10	CYP2C19	GATGGAAAACAGACTAGCAGAGCTT	GAGAAACGCCGGATCTCCTT
10	CYP2C19	GAATCGTTTTCAGCAATGGAAAGAGAT	GTATGTTCACCCACCCTTGGTT
10	CYP2C19	GCCTTGTGGAGGAGTTGAGAAA	ACAACCAGGACTCCAAATAAAAGATCTG
10	CYP2C19	GTAAAAGAACACCAAGAATCGATGGAC	GTCAATGAATCACAAATACGCAAGCA
10	CYP2C19	TCCATTTCTCTCCTTTTCCATCAGTTC	GGGATGAGGTCGATGTATCTCT
10	CYP2C19	GCCACATGCCCTACACAGAT	GAGGAATAAAAGAACATGGAGTTGCAG
10	CYP2C19	ATTGTTTTCTCTTAGATATGCAATAATTTTCCC	TCCATCGATTCTTGGTGTTCTTTTACTT
10	CYP2C19	AATTGTTTCCAATCATTTAGCTTCACCC	GGTGCTTACAATCCTGATGTTTTCATTC
10	FAS	CCCGCTCAGTACGGAGTTG	GCCTATCCCCGGGACTAAGA
10	FAS	GCACAGCAGATACTGCCAATTT	AATCCCTTGGAGTTGATGTCAGTC
10	FAS	GTCCAAAAGTGTTAATGCCCAAGT	TTCACTGTAATCTCTGGATGTTTTGTGT
10	FAS	CCCGTGTCCTGTTCAAACACT	TCATCACACAATCTACATCTTCTGCATT
10	FAS	CAGACAAAGCCCATTTTTCTTCCA	ACCACAGTAGGCCCCAATTTC
10	FAS	GCCAGGCTTTTGAATTTCTCCTGTA	GTGATTGGTTTTTCTTCACATCTTTCCA

10 FAS	GGCTGAGACCTGAGTTGATAAAATTTCT	GGAAAGCTGATACCTATTTCAATACCTACA
10 FAS	AGACCTTTAGGACTTAGCTATATTCTGAA	GACTTAGTGTCATGACTCCAGCAA
10 FAS	CCACTATTGCTGGAGTCATGACA	ACGAAGCAGTTGAACTTTCTGTTCT
10 FAS	TCAAGAATGACAATGTCCAAGACACA	CTGCAAGAGTACAAAGATTGGCTTTT
10 FAS	GAAAGAAAGAAGCGTATGACACATTGAT	CCAAGCTTTGGATTTCATTTCTGAAGTT
10 FAS	CTCAAGGACATTACTAGTGACTCAGAAA	CCAAGCAGTATTTACAGCCAGCTA
10 FAS	GCCCACCATTTTCATAGTCTGCTT	GGTGCAAGGGTCACAGTGT
10 FAS	CCAACCCCATGGAAAGATGTGA	GTGGAATTGGCAAAAGAAGAAGACA
10 FAS	CTACAGGATCCAGATCTAACTTGGG	AGAATGAGGCAAATCTTTGTGAACTACT
10 FGFR2	CATTTACAATGCAAGGGTAGCTGATT	CCCTCCTTCAGTTTAGTTGAGGAT
10 FGFR2	CTTACCTTCTGGCTCTAATGTGGT	GGGATTAACGTCCACATGGAGATATG
10 FGFR2	CACATGCCACAAAAGGAACTTTCT	GAAGGTTGCTTTGGGCAAGTG
10 FGFR2	TGTCTTTGTCAATTCCCACTGCTT	GTTCTGGAATTTCCCTCACTACACC
10 FGFR2	cctgaccAACTTTTCCCAGTTTCT	ACTTTCTTCTTCTGCCAAAATTGTTGTT
10 FGFR2	ACCCCAGTTGTGGGTACCTTTA	GGTCGGAGGAGACGTAGAGTT
10 FGFR2	CACAAGCTGGCTGGGTCAT	CATCCTGTGCCGAATGAAGAAC
10 FGFR2	GCATCACTGTAAACCTTGCAGACA	CTTTCTTCCCTCTCTCCACCAG
10 FGFR2	GAAGTCTGGCTTCTTGGTCGT	CCTCCTCCTGTGATCTGCAATCTA
10 FGFR2	AGCTGTGTTAATTTTATAGCAGTCAACCA	CGTGCTTGGCGGGTAATTCTATT
10 FGFR2	CCATGCAGAGTGAAAGGATATCCC	GCCTTTTCTTTTGCTTCCCTTGTT
10 FGFR2	TGGTCTCCCTGCTCAGTGTA	TCTTGTTCTTCAGGAGATGATTCTGTTTT
10 FGFR2	TTGGGCGAATGCAGTTTTTCC	ACTGATTTGTGAATATGCCTACTGTTCA
10 FGFR2	GTGAAATGCAGCAGCCACTAAA	CAGTCAAGTGGATGGCTCCAG
10 FGFR2	TGAGTGTATACTCTATCAAACAGGGCTT	AGCCCTATTGAGCCTGCTAAGATA
10 FGFR2	CATGGTTCGTAAGGCATGGG	TGACCCAAGATGAAATAAAACGTCTCT
10 FGFR2	TCCAATATCCCCATTTATAGCTGAGTCT	CGCTGGTGAGGATAACAACAC
10 FGFR2	TGTCTGCCGTTGAAGAGAGG	CCTTCTCAGATGGAGCCAGGATTA
10 FGFR2	GGAGCCGGGCAGTTACTTA	CACATCCATGGAATATGTTCTTTTGCAT
10 FGFR2	CCAGAGAGCTTCAGCCATTCTT	CACAGAATGGATAAGCCAGCCA
10 FGFR2	CCTTACAGTTCGTTGGTGCAGT	GCCGTGCTTTCTCCTTTTGTTG
10 FGFR2	GCTTAATTCTACCTTGTAGCCTCCAAT	CGGCCAACACTGTCAAGTTTC
10 FGFR2	GCATGGTTGGCATTGGGTTC	GGGAAGCTGTCCATCAGTATACA
10 FGFR2	GAGCATGTCCAAATTGCCTGTT	TTCCTGAGGAGCAGATGACCT

10 FGFR2	TGCATGACACCAAGTCCTTGA	GGAGCTTCTCTTCTTCCTCAACAG
10 FGFR2	AGAACGCACGTCCACCTT	TGAAAACTTGTAATCTTCCCCAGGAG
10 FGFR2	CACTGCTCCAGAAACCTTCTTCT	CCCAGAGCTTGTTGTCTCCAC
10 FGFR2	GCTGTATGCCTGGCATCCAAT	CTTGTACTGCCAGTAGGACTGTAG
10 FGFR2	ACCATGAAGTACCAAGTTTCACTGT	TGAAAGATGCCGCCGTGAT
10 FGFR2	CACCCCATCCTTAGTCCAACTG	CCATCTGTCTCCGTCTTCTCTCT
10 FGFR2	AAAAGAATAAACAAGACCACAGACTCCA	GAAGACTTGGATCGAATTCTCACTCT
10 FGFR2	AGGAAGTTCTTACCTCATTGGTTGTG	CCACGTACCCAGTGCATATGAAATTAA
10 FGFR2	GGCCTTTCTTCCTGGAACATTCT	CAGCCAGAAATGTTTTGGTAACAGAAAA
10 FGFR2	GTCCAAAGTCTGCTATTTTCATCACATTG	TTTGTTCTGGCGGTGTTTTGAAAT
10 FGFR2	CTGAAACTTATGGGAGAAACAGGACTT	CCATCAATCACACGTACCACCTG
10 FGFR2	GGCAAACTCACCCACAACATC	CCGCCTTTGCTTTGATCTTTTCA
10 FGFR2	CCTATAAGCTGCCTGCAGTCT	TCCAATTATATAGGGCAGGCCAAC
10 FGFR2	CTGTTTTGGCAGGACAGTGAG	CCGTTATATTGTTCTCCTGTGTCTGTT
10 GATA3	CACCGAAAGCAAATCATTCAACGA	GGTGGTGGCTCACCCAG
10 GATA3	GGGCCTCAGCCACTCCTA	AGGGTACCTCTGCACCGT
10 GATA3	CGAGAGCCTGTGCATTTCAGA	CCTCCAGTGAGTCATGCACTTTT
10 GATA3	AAATGTCTAGCAAATCCAAAAAGTGCAA	GGCTGAAGGGCGAGATGTG
10 GATA3	CCAGACACATGTCCTCCCTGA	GCATCGAGCAGGGCTCTAAC
10 GATA3	CCTGTCCTTTGGACCACACC	ATCGCGTTTAGGCTTCATGATACT
10 GATA3	CCCTAAGTGGCTTATCTGTGCTTT	CTTCGCTTGGGCTTAATGAGG
10 GATA3	CTATCACAAAATGAACGGACAGAACC	GCTGACACGATTGGAGGCTAT
10 GATA3	GTTTTGATTTCACCCTCTCCTCTCT	GTAGTAGAGCCCACAGGCATTG
10 GATA3	CTCTGGAGGAGGAATGCCAATG	TTTGCTGGTCACCATCAGGAA
10 GATA3	CCGCCCTACTACGGAAACTC	GGGCTTTCGCTTGACCTCTC
10 GATA3	TCACCCTCCTTCTCTCTCCTG	CGTGGTGGATGGACGTCTTG
10 GATA3	CTGGAATCTCAGCCCCTTCTC	GGTGGACAGCGATGGGT
10 GATA3	CCGCCGAAGGACGTCTC	TGGGACGACTCCAGCTTCA
10 GATA3	GAGAAAGAGTGCCTCAAGTACCA	CTGTACTCGGGCACGTAGG
10 GATA3	CCACCCCATCACCACCTAC	AGGGAAGAGCTGGCTCCTA
10 KAT6B	CAAAGGCACCATTCCCATCAAG	CACTGCATGTCTTGCATTCGAT
10 KAT6B	CCATCCTGTTTGAAATTTTGTCCTGAAT	ACACCTTAACACTGGAAATTAGCGA
10 KAT6B	CTCATGAGCTCTTATGTGTTATGTTTGGA	GCCTCTCTTCATCTGAGTTGTCA
10 KAT6B	GACAGAAGTACTGAATGAGCCCTT	TGTCTTTTCTTCCCAGGCTGATG
10 KAT6B	TGTCATAGCCCCGTGCTG	CAACTTACCACTACTGCCACAATCT
10 KAT6B	CCAGTCCTGAGAAAAGCATTCCAG	ACCCAGTCAGATACAGACATTCTCAA

10 KAT6B	ATCCCATTCCAATATGTAGCTTCTGTTT	AGCATGAAGCTATGATAATGGGAAGTC
10 KAT6B	GACATCACGGCTACATGAACAC	TCAAAGGTATTTGTGCAGATCAATCCA
10 KAT6B	GGCTCTGACATACTTGATCTGTTTCT	ATCAGCTTCCTGGATCATGAAAGTAAAT
10 KAT6B	GTTCAGCATACAGGCATCCTTGA	GCTTTCTCATGAAGTAGTTTTCTTCCCT
10 KAT6B	GGATGTGGATTTGCCAAGTCTG	TCTCAACCTACAACAATCGTTGCT
10 KAT6B	ACCCATTGGACGACCGAAAAATAAA	ACCCCTAAAGGAATCTGAGAGAATAACA
10 KAT6B	TGCTAGCATATGTCCGTATGTGA	caAGTTCATTGGCTCTGGAACAG
10 KAT6B	AGCCACATGGAAAAGCTGAAAAC	AGACAATCATTACCTCTTTCTCAGCTTC
10 KAT6B	ACCATATAGAAGAAACAATTTTATATGGTGCA	CCAGGAAGAGCTTGGCTAACAA
10 KAT6B	CTGATACTATTCTCTAAATTTCTGCCTAGGT	CAGATGAAGGTGTGGTACAGACT
10 KAT6B	GCTAGGTTGATGGGAATATGAGCAAAATT	CAACCAGATGACAGCCCTTTTC
10 KAT6B	ACAGATTACCAAAGCTTTACCTGTGT	CGCTTACCTCAAATACTGAAAGGTCTT
10 KAT6B	GTAACCAGTGATGAAGGATCCATGAAT	GGGTCTGTAACAGCCAATCTG
10 KAT6B	TGTATTATGATGTCGAGCCATTCCTTTT	CCCTGTTCAAAGAATTGGGCTCT
10 KAT6B	TCCAGCAAATGAAATTTACCGAAGGA	ACCTTCTAAACACAAATCAGCACATAGT
10 KAT6B	CCAGTTCACAAAAGTCCAGCAC	GAGACTGTCCCTGAGTGGTATAGA
10 KAT6B	CATCTGGGAAGGACTCAAGCAG	CAATGAGCCCTTTGGTTTTCTTGTTAA
10 KAT6B	ACCTACATTTCTGCCTCTACACTTAAAG	TTGGACGATAGTGCTTTGAAAAGTCTA
10 KAT6B	GCACTCTTTGATGGGCTTTCTCATA	CTTTTGCCAAAGAAGTGGGCTT
10 KAT6B	GTCGCAGATCACGAGGTGAAATTA	GTGGAGGTTTTAGCTTTTGTGTGG
10 KAT6B	ACTGAATTATCTTCCACGGCAAAATCTA	CATCCTGTGCTTTTTAAGGAAAGTTGT
10 KAT6B	GCAATTGCTCACTTCAAGCGA	GTTGGAGCTTTGACCTTTTGCTTAC
10 KAT6B	CTTCTCATGTGTTGGCTACAGGTA	GCACTGGCTGGAAGAACTCTG
10 KAT6B	ATGCTAATTTGGTGCCATTTTGGT	TCAGTAACGACATCCTTGTTTCCAAT
10 KAT6B	TCAAACAAGAAAGTGCAGATGTAAATGTG	CTTGCTACTACAATGTGGGATCCT
10 KAT6B	AAAGAATTTTTGGCCTTTTACAGTTGGT	ACCTTTTGGCATTCTGGAAAGTG
10 KAT6B	ATTTCATATGGAATGCTGTGACCCA	AACCTGTATACCTCTGAGCTGTCA
10 KAT6B	ACTTAGAGACACTTTGCCATTGATCC	GTGATTGCCTACTGTTAGCTCTAGT
10 KAT6B	GTCACTGGTGAAAGAACCAAAATGT	GAGGAGTACCAGGTTTGGATTTCATATT
10 KAT6B	TGGAAGACTGTGGCCGGTA	CATTCAAGTTATACACCTGCCAGACT
10 KAT6B	GAAGGAGGAACAAGAAATCCTGTCA	cttcACTGCTCTCTTTAGACAGCTC
10 KAT6B	GCTGCTGGAGCTGTCTAAAGA	CTGTGGTTTCGTCAATCTTGGG

10 KAT6B	GCTGTCTAAAGAGAGCAGTgaagaag	GCAACTGACTGTGGTTTCGT
10 KAT6B	CAGTCTGGAATACTCTGTCCATTTGT	ACTCTGTATAAAGTGGGTTTGCAAGTT
10 KAT6B	TGAAGAAGTCATTTGTCAACCATGGTAA	CAGAGACTGTCTTCTTATCCAACCC
10 KAT6B	CTGGGCGCTTTTCATCAGTT	GATACTTCTCTATGTTCTTCAGGGAGGA
10 KAT6B	CATGCGGTCAGTACTTCCCAT	TGACTTAGGAAAAGTGCCTGGTTT
10 KAT6B	GGACTTGAGGAGCCGAATGG	GTACTGCGGTCCGTCTTTCA
10 KAT6B	ATGGTGCTAAAAGCCAAGAAAAAGAG	CAGACTCCATGTGGCTGTCAT
10 KAT6B	CTAAGCTCTGGGAATGCCACA	CTCCAAGATGACGCTCTTCCAA
10 KAT6B	CGCTGTGAATAATGGGAGGTTAC	GCACGAACATTACTCCTTCGCTTA
10 KAT6B	CATGGAAATCGACTCTGAGACTGT	TGGCAATCGTCGAGCGT
10 KAT6B	TGCCCATGGTTATGCTTTCTAAGAC	CCACTTAGATAGACCGCGCTTT
10 KAT6B	CAGCTGCTCCTATAGCAACCT	GAGGCCTCTCCACCACAC
10 KAT6B	AGGACCATGATGCCGATGAC	GCCACAGTCCATTAAGACCTCT
10 KAT6B	GCAGCGTCAAGTCTCCTCAAG	CTCTGACCCATTCGCTCGTA
10 KAT6B	CCTCTTTTGCAACGGAACATGG	TCTGGGAGCGCCCATAGAT
10 KAT6B	GCAACGCCAACATTGGCTTA	GCTGTAAGGCAATGAATGATCAATAAGT
10 KAT6B	AAACAAGTGTGGCCAAAAGGAAC	GGCTGGTTTTTCCTTCTGAAGTC
10 KAT6B	CCGTCTCTCCTACCTGGCATA	TCTTGTCGATCATGTGGAGGTG
10 KAT6B	ACCAAAGTCCACAGATTGCCA	GGCACTTTGATCTGGGCTGATT
10 KAT6B	TTAATGTGCAGCCTGGTCACT	GTTCTTTTGGTCCTGCTTCTGTG
10 KAT6B	GCCAAACTGCAGCAGTTAACTAATAC	GGAGTGTGGAGACTGAGAAAGTT
10 KAT6B	ACATGACATTGCCACCACTCT	ACTGTTAGGGAACCTCAGTCTTTAACT
10 KAT6B	CTGCCACCCATCAGTCACAA	CCATTCATGGCGTTGAGAGTGT
10 KAT6B	GGTAACAGTGGAAGAACAGAAGGA	TTCTGGTTTTTCACCTGGTGATGT
10 KAT6B	CCCTGCTCTGGAAAACAGCTAC	TCGATACTGCCCAGGTCACTA
10 KAT6B	TGTACCCGAATCTGACGAGGA	CTCTCCTGGGTCAAAGACTGAAC
10 KAT6B	ACCATTAAGTAACACAGGGCTTGTT	GTGAGAGATGCCAATATTTGATGCAG
10 KAT6B	TGTGCCCCTGTAAGTCCAAAC	GCTTTTAGCACCATCTGGAtctttttc
10 KAT6B	TGTCAACTCTGTGAACATGAACATGA	CCATCTGCATCTGCATAGGGT
10 KAT6B	AGATGATCTCATCAAACCTgaggaaga	TCTTTTTCCGTGGAGATTTCTGGTT
10 KAT6B	GGCTATATGAATCAAACGCCCCAAT	GAGAGACTGTTTGGACATGCCT
10 KAT6B	CAGCAAGTCGTAGACAGTGGATT	CTCTGTGTCAGACTGCTGGAG
10 KLF6	CCGGGTCTGAACCCCAAA	GGACGTGCTCCCCATGTG
10 KLF6	GATCTGGAGCTCCTGGAAGATG	CGAGCCTGGAGTTTGCATGAA
10 KLF6	CATTTCCCTTGTCACCTGGCT	CGGGAAAATTGAGCTCCTCTGT
10 KLF6	CTCAGTTCCGGAGAAGATGGAG	CAACAGCCTGAACTCAGATGTCA
10 KLF6	GAGCTGTCAGAGGATTCGCT	CCAGGAAGATCTGTGGACCAA

10 KLF6	TTTTTCTCCCGAGCCAGAATGATT	CGTGCCTTCTCTGGTTCATTTTG
10 KLF6	CGTCCGCTGGTGTGCTT	CCTCGCCAGGGAAGGTG
10 KLF6	GCATTGTCCTCAGGCACGTA	TGCTTGTCCTGTGCTCTCTC
10 KLF6	TCTCAGCCTGGAAGCCTTTTAG	CTGTGCTTCTGATTTGCCCTTG
10 KLF6	ATTTGTCTGCCCTGACCACAT	TTTACACCAAAAGCTCCCACTTGA
10 MAPK8	TTTTCTTAGATTGCTGCTGGACACT	GCATCTGATAGAGAAGGTAGGACATTCT
10 MAPK8	GCAGTCAGTGCAGTGCAAGTA	TGAAGACACATCATTGACAGACGAC
10 MAPK8	CCAAGTGATTCAGATGGAGCTAGAT	GACGATAGTTTCTTCTACTAACCCGATG
10 MAPK8	GGCTCTCAGCATCCATCATCATC	GACTAACCGACTCCCCATCC
10 MAPK8	CAGAGTTAAAATACTCCCAGCATACTGA	GTTTTCCCTCTAAGAATCTGACGAATGT
10 MAPK8	TCCACTTAAACCATACATGCGTTGT	GCCCCCGTATAACTCCATTCTTG
10 MAPK8	GGAAGTTATGGACTTGGAGGAGAGA	TTTGTAAATATGGGAAGAACTGCAGGT
10 MAPK8	AATGACCTTTTGCTTTGCTTTTCCTT	GTTCATCGCCTAACTAATTAACTGCTG
10 MAPK8	CGAACAATTAACATGAATGTTTTGCAAGG	AGTCAAGAATCTTCAAAGTGCAATCAGA
10 MAPK8	TCTGATTGCACTTTGAAGATTCTTGACT	GTGTGCTGACCGTTTTCCTTG
10 MAPK8	GTCATCCTTGGCATGGGCTA	ACACATTTAACTCAACGTACTTCTGCTT
10 MAPK8	TGAAGTAATTTCTAATTTTTCTGTCTCTCGACT	GCTAAAGTGTCCAGCAGCAATCTAA
10 MAPK8	CATTAACACCATTATGTTTGCAGCCA	GCTTCAGAAGGATCATACCAGACAT
10 MAPK8	CTCTCCAACACCCGTACATCA	CTCCTCCAAACCTAACTGTCATTCTAAC
10 MAPK8	TGTTTTGTTGCATCTTGCAGCTT	GACTGTGAATGTAGAATCTCCAATCTCT
10 MAPK8	GCAAGCGTGACAACAATTTTTATAGTGT	CGAGAGGAGAAAAGGTAGCAGTTAATGA
10 MAPK8	CTTAGCTACTTGATATTAGATATTGATCAGTGG	TTTAGGTCTGTTTTCAACGTAAGTCCTT
10 MAPK8	ACCATGTCCTGAATTCATGAAGAAACT	GTGTTCTGAGTCAGCTGGGAAAA
10 MAPK8	GTTGAAAACAGACCTAAATATGCTGGAT	ACACACACTACACACCCCAAATG
10 MAPK8	CACTGTCACTCATTTTTATTTTATACTGCTT	TGATGCACCCAACTGACCAAAT
10 MAPK8	GTCATTGCATTTTGTTTTCAGTTGACA	TGACCAAACTGAAGTGCTATCATACATT
10 MAPK8	TGGAAGGGATCCAGTTACTTGTCT	GGCTTAGCTTCTTGATTGCAACATT
10 MAPK8	CAGCTTATGATGCCATTCTTGAAAGA	ATGTTCACTTACATTTTTGTGATTAACACATT
10 MAPK8	CCGACCATTTCAGAATCAGACTCAT	AAATCTTGATTTCATCTATACTTAGGAAACCA
10 MLLT10	TTTGAGTTAAGATAGGTTACTTAGTATACCTGA	TGCTTTCTCTTCCTTGTTCATCACA
10 MLLT10	ACCCCAAGACTTGCTACATTTGT	TCTGTGCAACAATGGAAACATCCTTA
10 MLLT10	TTTCTCAGAGCATGAATGATTGGAAGT	AGCCACAGTATTGGACATTATCGG
10 MLLT10	GGTAATGGTGCCGATAATGTCCAAT	CCAAAAACTCAAGGAAAAAGGAACTGTT

10 MLLT10	GGGTCAGCTTAAGGTAAGAAACTGTA	CTGGAGGCAGATTTTCCAAACTG
10 MLLT10	GCTATGATCAACCAGGCAACAG	TGCCCATCTGTCTTGATTCACATATAAG
10 MLLT10	TTAGACTTCTAAAAATGCATTAGAGGTGTGT	GCTGAAACTGCTGTTGATGAATAAGT
10 MLLT10	ACTTTCTGACCAGCAACGACAAA	CACAAAGGAGAAATCTGTACCCACA
10 MLLT10	TTTGTTAATGTCCTTGTTGTGCATTTAT	TCTGGAATATACAGGGCACAAACC
10 MLLT10	GGGCCCATGTGGTTTGTG	ACAAAAACTTCCTTTCTACTTCCCAGT
10 MLLT10	AAGGTCACAAATTTTGCTTATATTCTTAGGT	CATATGAGATGGTGCCTGACTGA
10 MLLT10	TCTTCTCTCAGTCAGGCACCAT	GTAGGTGAGAGTAGGGTTTTAGAGTTAGT
10 MLLT10	CTGCTCTCACATTTTCAGTAGTTTTGAC	GAAATCTCTGCACTTACCCCCAT
10 MLLT10	GTGAGAGTCACTTTATCAGCTCTGAA	TCCTTTGCCTATACTTTGCAGTGTAAAT
10 MLLT10	AGTGGTTTTGATACGGGCTTCA	AGCTGTTTCCTATATGAGGGCTCT
10 MLLT10	CAGATCCCTGGACCAACACAA	GCTACTTTCTGACTTGCATTATCTCCAT
10 MLLT10	ACCAACCCATTTCTCACCATCC	GGCTGGAAAATGATCATCTCCGATTAA
10 MLLT10	GTAGTTTACTTGAATAGTGACAGGTGGA	GGTCCAGTGGGTACTTGAACAATG
10 MLLT10	CATTTCATTTTTCAACAGCTTGCTATGG	ACAACAGGAGTGCAGTTCCTAATT
10 MLLT10	AGTTCTTCTTATTAATGTGGCACCCAT	CAGTAGTATCTTCCAAGCGCTTCAA
10 MLLT10	CACTAGCAACAACTCTATATCTGGATCA	AGCTGTGAGCTGAAGATTTCTTCC
10 MLLT10	CTAGAGGGTCAGAGGGCAAAG	AACATCACTAATACCTTGAGGAAAAGGG
10 MLLT10	GGAACAACTGTGTCAGCAGCTA	ACATCCATTAGTTATAAGTTCAAAAACAAACCA
10 MLLT10	GGCTATGTATTTCTTTTGTGTGTTTGAGA	AACCTTTGTGATAAACGAACCACTAGA
10 MLLT10	GTTACCGGCACTGCGTAAAAT	CGTTCCTTTTTGGCAGTCAAGTTTTTAA
10 MLLT10	AGTTGAAAACCGAAGATTAGAGGAACAA	CACACTTACCAGTCTGTGCTGAA
10 MLLT10	CTAGTCCGTCTCATCAAATACACACATT	AAACAACCTATAGTTCCTTTTCTACATCTTC
10 MLLT10	GAAGCACTAATCGGCTTGCAT	CAGCCTCCAATCATCTCCTTCATAC
10 MLLT10	GGAGGACGAGGTCTCCCATA	ggcggTTGGGTGTTTACCT
10 MLLT10	TGAATTCCGTGCTTGGGAACT	CCTGCAAGAAGGTTGTTTATGGGTAT
10 MLLT10	TGATTCTTCCTCTCACTCTCAGGAT	CCAAAATGTTTCTATATTTCAATGTCTCCATT
10 MLLT10	AGGAAGTGATTTTTGACAGAAGTATTTGT	TCTGGCTGCTTCTTGTGTTTCT
10 MLLT10	GCCAGAACCATCACCTGCAT	GGTTGAAAAGCTGGGAAAAGTTAGG
10 MLLT10	TGTGTCTGTGACAAATTTGACTGCTA	GGTAAGTGAGAGCTGGAGACAG
10 MLLT10	ATCTGGCTCGGGATCTAGTACTC	ACCCACCTATTACCTGTCACATGATA
10 MLLT10	ACCACATTTTATCTCAGTCACAGTTTCA	AGCAGGATAGGCAATTTCTAGATGTTTC

10 MLLT10	GATTATATCATGTCTGCTGCTTATGGGA	TGCACTGAACTTCCAGAAGATGAC
10 MLLT10	CAGGAACTCCAGGCAGTGTAAA	TCCTTTATGTACATCTTTGGTTGCTGA
10 MLLT10	GCTTCACCAACATCATCTGTAGCA	GTGGTGGGAGTGAAGGAGAAAAATAATA
10 MLLT10	AGTTACTCTCACTCCCAACAGTCA	CTCCAGGTGAATTTTCAAAGCTTTTAGG
10 MLLT10	CTTCAACCTCAGCTGTTACTTCACA	CAGGCCCATGCTTACTTTGTTTATT
10 MLLT10	CTGGCATAGAAGAAGAAACTGTAAAGGA	TTGTTATGCTTCCTGCAGCTGA
10 MLLT10	GGACTTAATGTCCAGTGGACTTAGTT	GCCATTGACCTGCTGAACTCC
10 MLLT10	CAACAAGGCTCAGGAGTGAGT	CAATGCCATTAATTGCCAGTTGGTT
10 MLLT10	GGAATAATTGGAGCTTTGCCAGGT	CTGGCATTGGATGTGTTGCAT
10 MLLT10	CCACACAACCGTACCACCTA	CCCCAATCTAAGGCATAGACAACA
10 MLLT10	TGCACCCCATCATAATATCTTCAGTTT	AACCATGCTGGAGTTTGGTACAT
10 MLLT10	TGGCTTTATGCCATTTATCTCAGTTGT	CAGGATAGACTGAAGACTAGTACTTGGT
10 MLLT10	AGGTGGCCTACTTAACTACACTT	gcaagCTCCATCATGCCGAT
10 NCOA4	GGCTCACTGAACCATATCCCTTTTA	GGGATCTGAAAATTCCCAACGGT
10 NCOA4	TTGAGGGATGATTCAAAGGTGGTTT	TGCATCACTACACCTCAAAAGGG
10 NCOA4	GTGGCAGCTCCAGTAATAGAGAAC	GTCAAGAGTCCAGACAACAGAAGA
10 NCOA4	TGCTGTTTGGTAACTTGAGTATATCTGG	TGTTTTGGGTACACTCCAGTTGATG
10 NCOA4	TTGGGCCAGTTCAATTGTCTTACT	ACAAGCAATGAGTTACAAAATCGACTTTC
10 NCOA4	TCATCCAAGGTTTTCATCTTGACTTTGT	CCTCTAACTGACATGCATGGAGATAGTA
10 NCOA4	CAGAACCATCAGGACCCATGTAA	GCTCAGGTTTGGGTTCCACA
10 NCOA4	GGCTCTGTATAAGTGGCTTCTGAA	CCTTTGGTGCTTTAGTTTGTTCTATTACT
10 NCOA4	CTGAATATGTGGCTCTGTCCTAGAAAAG	TTCCTTGGGACTTCCTTCTTTGTATG
10 NCOA4	GAACAGCCCCTTGTCGGA	CCCAGTCAGCTGTGTTAAAGGAA
10 NCOA4	ACAGAAGTTTAAAAGCCCCATGAATACT	CCAGTGCTATTTTGATGTTTATGCTCA
10 NCOA4	CTATCTTTGGTTGAGTTTCTTAGAGAAGT	GGACAGTTGAATCTTCAGGCTTAAGG
10 NCOA4	CCCAGACTGGGCAGTTTGAC	GCCCAGTGAAGCATATGAGATTACTC
10 NCOA4	GTTGGTCTCATGCCTCCTTGT	AACTTTAAGCTGCATACAGGCAAAG
10 NCOA4	CCTCCCTGCAGTTTGTGATCT	CAGTGAGCCAAGTATGACTTCATATGTA
10 NCOA4	GGAAACATCATTCTTTGGGATTAACACT	CCTGTTCATACAGCCATACCTCAC
10 NCOA4	GTCACCTGGAATGTCTTAGAAGCC	ATTCTAGCCATGCAGTCACCTTAC
10 NCOA4	ctactgcaTTTGACATTCCTTGTTTTGT	TGCTGGGTATGTAAGGAGCTTG
10 NCOA4	AGCAAACCTGCCAGTGGTTAT	ACCTACTGCTGTGTCTACTGTAGAAA

10 NCOA4	CAAAGCAGTCATAATTGTTCTGGCAAA	TGGAATGGCTGTTACACTTTTGATAACT
10 NCOA4	AAAACTGGCTCCTCAAGAGTGAAA	CATCTCATCTTGATCAGGAAGCTCT
10 NCOA4	CATTGAAATGGAAAAGGTTGGAGATCA	AGAGCTTAAACTTCTCACTGGTTTCAC
10 NCOA4	GGAAGCCTGAGAATGGCAGTC	GCACTTCAGATTGCCCAGGTTT
10 NCOA4	CAGCCCAAAGGTGTGGAGATT	GCTCTGCACACCTCCTCTAC
10 NCOA4	TCTATTTTGTAGCAAATTCCTGAGCACT	CCCAAAGGAAGTATAAGCCAAGTAATGA
10 NCOA4	TGTAACAAGGGTATACCTGTATTGGGA	GGCTGCTCAACTCTTGTCCATT
10 NFKB2	CCAGCGAGGGAGACTATGAG	CGTGGTGGATGACATAGACTATCTG
10 NFKB2	GGGCAGACCAGTGTCATTGAG	GAGACCCACTTGCCTTCCAA
10 NFKB2	CACAATGCTACTATGCCCTTGGA	GCATGCTGTCCACTTAGCC
10 NFKB2	CCGAAAGACCTATCCCACTGT	CCAGGTCCACCTCGATCTTG
10 NFKB2	CCCACCCCCATTTAGATCTGA	CATGACTCACTGGGTTGTAGCAA
10 NFKB2	CCTAGCCCAGAGACATGGAGAG	ATCTGCGAGCATACAGGTGTAAG
10 NFKB2	GGCCTTGGCTATTGCATCATC	AAAGTGGGCTCCAGTCTCAC
10 NFKB2	CCCCAGTCTGTCTCCAAACC	GGGAGGTTAGTGAACTTGCTGAC
10 NFKB2	CCAATGGCTTCCTTGAGGAAGT	TGTATCATAGTCCCCATCATGTTCTTCT
10 NFKB2	GGGTGTCCTGCATGTGACTA	CTTCCATGAGCACCTCCCAT
10 NFKB2	CATGGGAGGTGCTCATGGAA	AGCCGCACTATACTCAGATCCA
10 NFKB2	GGCCAAAGAACTGAAGAAGGTGA	ACCCCAGGCAATCAGGATACT
10 NFKB2	GCATGACACAATAACTGGGCTCAA	CTCCTCCAGCTCCTCCGTA
10 NFKB2	CTTCTCCCAGCCCTTCGG	TCCTCCACAGCTTCTCTTACCT
10 NFKB2	CCACAGCCCTGCCTGTATC	GGCTTCCTCCCCGGAGT
10 NFKB2	GCCCCATGGGCTGCTAC	GTGCCCCGGAGTCCATA
10 NFKB2	CCCCGACTTTGCAGTCCTTAAT	CTTCTCAGGCCCTTCAGAGTC
10 NFKB2	CCCCTACCTCTGATAGCGACTC	AGCCTGTGTCTTTAAGTCCAGATACT
10 NFKB2	GGTTGAGCATCCTGCATCCTT	CTAGATGCAAGGCTGTTCGTC
10 NFKB2	CTGGATCTGCTGGTGGACAG	CACTCCTTCCACAATCCTCAGT
10 NFKB2	AGTATCTGGACTTAAAGACACAGGCTTA	GGGATGCCTGATGCTTCTCAC
10 NFKB2	CTATCCCATTCCTGTCCCCATTT	GCAGGCAGCAGGTCAGTG
10 NFKB2	CTAGATCTGTAACTACGAGGGACCA	AGGCGTAGAGGGCACCTA
10 NFKB2	AGACCTCATTCCTCTGTCTTCTCA	TCCAGGTCCAGATCCACCTT
10 NFKB2	CTGCAGCGAGGTATGGACTC	GTGACGCCGTAGTCGAGTA
10 NFKB2	CCCGTAGCTCGAGAGTACAA	CAGGCCCTCTGTTGCCTA
10 NFKB2	GCCAAGCTTCCGTTTTCCTTGTA	GTGTCAGTGAGTCCTTAGCCTAATTT
10 NFKB2	GGGCTAAATTAGGCTAAGGACTCA	CTCCTCGCTTGCGTTTCAG
10 NFKB2	GCCTGTAACAGTGTTTCTGCAA	CTCTTGGGACCTTCTTCTAGCC
10 NFKB2	CTGGCTCACCCTGCTTTCA	GCCAGATGCATGGCTGAGTC
10 NFKB2	CTGCTGGATCGGCATGGA	CCCCTTAGTCAGGTGAGATGG
10 NFKB2	GGACCATGCTGTGGTGTCAA	CCCCTGAGGGTGAGACTGA

10 NFKB2	GGAGATCGTGGCTCAGCAAG	CAGCTACCACTACCCCATTTGT
10 NFKB2	TGTCTGTATGTGTGTCCCCCTAA	CACTGGGTGAGGTTGTCTGTC
10 NFKB2	GCAGCCTGGTAGACACGTA	GTCTGAGTCATGGTTAGGGCAT
10 PTEN	CTGTTAAGTTTGTATGCAACATTTCTAAAGT	TGGGTTATGGTCTTCAAAAGGATATTGT
10 PTEN	GAGGTTATCTTTTTACCACAGTTGCAC	GTGAATTGCTGCAACATGATTGTCA
10 PTEN	AGCTAGAACTTATCAAACCCTTTTGTGA	TGCCCCGATGTAATAAATATGCACAT
10 PTEN	GAAAGGGACGAACTGGTGTAATGAT	CAATAAATTCTCAGATCCAGGAAGAGGAA
10 PTEN	TTAACCATGCAGATCCTCAGTTTGT	AGCATCTTGTTCTGTTTGTGGAAGA
10 PTEN	GTGGTGATATCAAAGTAGAGTTCTTCCA	AGCAAAACACCTGCAGATCTAATAGAAAA
10 PTEN	TGGCTACGACCCAGTTACCAT	CCACTGGTCTATAATCCAGATGATTCTT
10 PTEN	CAGTCAGAGGCGCTATGTGTATTATTATA	ACATGGAAGGATGAGAATTTCAAGCA
10 PTEN	TGCAGAAAGACTTGAAGGCGTAT	CATCACAAAGTATCTTTTTCTGTGGCTT
10 PTEN	ACTTTTTGCAAATGTTTAACATAGGTGACA	AGGTTTCCTCTGGTCCTGGTA
10 PTEN	GCAGTATAGAGCGTGCAGATAATGA	CATACAAGTCAACAACCCCCACA
10 PTEN	TGTTAATGGTGGCTTTTTGTTTGTTTGT	TCTACCTCACTCTAACAAGCAGATAACT
10 PTEN	GCCATCTCTCTCCTCCTTTTTCTT	GTGACAGAAAGGTAAAGAGGAGCA
10 PTEN	TGCTTAAAAATTAATATGTTCATCTGCAAAATG	ATTTGACGGCTCCTCTACTGTTTT
10 PTEN	ATTTTCTTTCTCTAGGTGAAGCTGTACTTC	ATCAGAGTCAGTGGTGTCAGAATATCTA
10 PTEN	CTTTTAGTTGTGCTGAAAGACATTATGACA	TCTCACTCGATAATCTGGATGACTCATT
10 PTEN	TGAGATCAAGATTGCAGATACAGAATC	TTCACCTTTAGCTGGCAGACC
10 PTEN	CACTTTTGGGTAAATACATTCTTCATACCAGGA	CACGCTCTATACTGCAAATGCTATCGA
10 RET	CCCTGTGACCCTGCTTGTC	GGCGTTTCCAGGGCTTAC
10 RET	GTCATTGTTGTGCCCCTACCT	CGTTGAAGTGGAGCAAGAGGAC
10 RET	CTGGTCAATGACTCAGACTTCCAG	TCCCCAGACAGGCAATAGGTA
10 RET	GGACTTAGGCTGTGTGGGAAT	TTGTACTGGACGTTGATGCCA
10 RET	GGAAAACTGCCAGGCATTCAG	ATGTAGTGAAGTTCGGCACACTT
10 RET	GGATCCTGTTTGTGAATGACACCAA	CCCCTCCACTGTTACAAGCAG
10 RET	GTGCCGAACTTCACTACATGGT	TGCTTTTCTCAAAGGGCAGGAG
10 RET	CTGACCTGGTATGGTCATGGAA	TGATGACATGTGGGTGGTTGAC
10 RET	CAGAGTTCAACGTCCTGAAGCA	GGAGAACAGGGCTGTATGGAG
10 RET	CTGAGTTGTATCTAGTTGTGGCACAT	GGGCATTATTACAGTCCACCAG
10 RET	GATTTATGACGACGGCCTCTCA	GAGAGGAAGGATAGTGCAAAGGG
10 RET	GCTCAGATGACAGCCGGTTCT	GAACCAAGTTCTTCCGAGGGAAT
10 RET	ACATAGGAGGATCCAAAGTGGGA	GCAGGTACCTTTCAGCATCTTCA
10 RET	CTTCCATCTGAAAGGCAGAGCA	AGCCGCTCTAGAACAGCATT

10 RET	CTCCTTTACCCCTCCTTCCTAGAG	TGTAACCTCCACCCCAAGAGA
10 RET	GGGACACTGCCCTGGAAATAT	CACTTCTCCTCCTCAGGGAAG
10 RET	GGGATTAAAGCTGGCTATGGCA	CCTTGTTGGGACCTCAGATGT
10 RET	ttttGTCCTTGAAGAAGCCTTATTCTCA	GGCATGGACGTACAGCAAG
10 RET	CTGGGAGAAGCTGTATGTGGAC	GTGTCCTCCTGGATGCAGAT
10 RET	CGGCTGCATGAGAACAACTG	CTGTGATAAGGGCGGCTTGA
10 RET	GGCTCCCACATGGGTGAC	TGCCCTGATTAAACCCTGCTTAC
10 RET	TGTGTCCACCCCCTTACTCAT	CCGTATTTGGCGTACTCCAC
10 RET	GCCCGCTCCTCCTCATC	GTCCAGGGAGCTGGAGTT
10 RET	GCTACCTGGGCAGTGGA	GCTGGGTGCAGAGCCATA
10 RET	GCCTGACGACTCGTGCTAT	TCATAAACATCTCGGGACAAGCC
10 RET	GCGGAAGATGAAGATTTCGGATTTC	GGTATCTTTCCTAGGCTTCCCAA
10 RET	TGCTTGGATCATATTGGCCTGT	ACTTTCAGCTTGTGGGAATTGGA
10 RET	GCCCCTGTCTGCTTGGT	GGGCACCATCACCACCTC
10 RET	GGGAGAAGTACGAGCTGGT	ACCTCCTTCCGCTTGAACTC
10 RET	GTGTACGACGAGGACGACTC	GGTGGCTACACGGACACTAAA
10 RET	CCCCACAGACCTGACTTCTCT	GCTGGAAAGGAGGTGTTGAAGAA
10 RET	TGTGCCCGCGTATACTTCTC	CAGGCGGAACTGGTGGAA
10 RET	CCTTCCGCATTCGGGAGAA	CCACAAGGTCGGCACTCAC
10 RET	ACTCACTGGTCCTTTCACTCTCT	CAGTTGTCTGGCCTCTCCAT
10 RET	GCGGCTCTTCAACCTTCTGAA	GGAGGGAATGCACACAGATGTC
10 RET	ACCAACGCCCTCTGCAT	GGTCTCGTTGGGCCAGT
10 RET	CAGACCTTCCGGGTGGAAC	TGAAGAGCGAGCACCTCATTTC
10 RET	GGGAGTTTTGCCAAGGCCTTA	CCAGTGTTAGTGCCATCAGCTC
10 RET	AGAGTCCTGTACCACTCACGA	CCCTTGTGAGTCCATTACCTTTCA
10 RET	CGGTGCCAAGCCTCACA	GGGTGGCTTGTGGGCAA
10 RET	GCATCCACTGCTACCACAAGT	TCACCAGGATCTTGAAGGCATC
10 RET	CCCTCGCTGGACTCCAT	CTCGTCTGCCCAGCGTT
10 SUFU	GTCGTTTGCCCTCTCCAGTT	CCGTAGATGGCGTGCAGTC
10 SUFU	ccTTCGCTTCGCTCTTTCC	CCTCGGCGCTTTAACCCTTT
10 SUFU	TTCCTCCAGGATGGGTCCTTTA	CTGTACATGCTAACATAGTCCAAGGG
10 SUFU	GGTTGGGTGGCCCAGAC	GGATAAAGAACAGGTGGCATATACTC A
10 SUFU	CGCTTGGTGGTTGGCAAAAAG	CCTGTTGTCAGGAAAAGTCAAACAAG
10 SUFU	GATTTGGATACTGAGGCCACCA	CTCTCTTCAGACGAAAGGTCAACT
10 SUFU	GGACCTAGTGGTTTTGGCTTTG	CAACCAGGCTCATGGCAAAAG
10 SUFU	TGTCTCCATGTTCCCATCTCCA	GGGCCTTCTCACTTGTAACCA
10 SUFU	CCTTACGCGGCTCATGGAC	GCCCTGAATCCATGAGGCATTC
10 SUFU	GCACCACAAGGGCTCAGTAAA	CTGACACCACTCAGGTTGGAG
10 SUFU	TTGACAAAGGCATCGAGACAGAT	TGGTGGAATGCTGAGTGATGG
10 SUFU	tgtgtgtAATGTTCAAGCACGTTTT	CCTCAGGGCAGAAAGACGTT
10 SUFU	GGCCAGCGTGGTAGTTTTTCTTA	GGAAGGACAGGTTTGCTGTTG
10 SUFU	CCTGAGGAGAGGACTCGAGAT	CTCATGCAATCCACAGGGACA

10 SUFU	AGTGAGATCCCAGCCCAGATT	CAGCATGTGCTGAATTCTTGACTC
10 SUFU	GCACAGCCCTTTGGATAACAGT	CTTCCCACAGGAAGGCTTGA
10 SUFU	CTTTTCCTGTGCTTGCTTCACA	ACCTTAGGCAGAGAGGAATGAGG
10 SUFU	AGAGCGTACATCTGAAATTCAACCA	CTGAAGTGCCCCCTGGAG
10 SUFU	CCCCAGACCCTCAGTTACCAT	AGAAAGGTTTTCCTCACTCAAGACATAC
10 SUFU	CAAAGAGTACAGCTGGCCTGAA	AACTGTTACACTGGAAGTCACTGG
10 SUFU	TCTCTGGAAAGACCACGGTGTAT	AGGATGGAGACCTTCAGCTTCTT
10 SUFU	GGGTGGCCATTAACACACAAT	CCACCTTGCATGGGTACTCAC
10 TCF7L2	GTTTGTGACATAAGCAGAACGCTTT	CTTCAGAATCTCTTGCGTTTCCTAC
10 TCF7L2	GTCTTCTAGGTTCCCTCCCCAT	GAAGGTGAGAAAAACAGCAACACTT
10 TCF7L2	CTTGACGGTGTCTTTCTCTGTTC	GTAAGTGTGGAGGTGGGTTTCC
10 TCF7L2	TCACGTACAGCAATGAACACTTCA	CTGTGGAGGCCTCAAAGGTT
10 TCF7L2	GAGCCTTACTCTGTTCCCCTT	GCTCCTTTTCATGTCCCTGAAGA
10 TCF7L2	CTGTAGCTGAGATTTCACATCCAACT	AAAGGCCTCGCAGTGGTAATATAC
10 TCF7L2	AGACTTCACTGTCAGCACTCAAG	CTGCCCTAGAGAGAGGTAAACTTTG
10 TCF7L2	GCCATGTTAGCGGCCAAGA	gCGGAGGCACTTACGGTT
10 TCF7L2	CCCACCCTTGTTTCAAGTCTCTTA	TCACACATGGCCTTGGATGAAG
10 TCF7L2	CCTCTGCTCGCTTCTCTCTTG	AGCACAGCGAATCTGGAAAACTAT
10 TCF7L2	CTGCATAGGCAATCTCAGAGGTC	TGACTCAAAATGACATTAGCACCTGTAA
10 TCF7L2	CCTAAATCCTTGCCTTTCACTTCCT	AAAGAATGTTCAAATGTCATACGTGTTTAA
10 TCF7L2	ATTCTCAGGGAAAGTGTAGGTACTT	TTTAGGGTGTTGAAATACCTACCATTGT
10 TCF7L2	CTGTTCCCATCCCCTAATGGATT	TGCCAGGCGATAGTGGGTA
10 TCF7L2	AGTTGGACCACGACCTTGTTT	CCTTTGCTCTCATTTCCTTCATATACAAC
10 TCF7L2	CACCCTCCAGATATATCCCCGTAT	CATGCCCATGTAAGAAACAAAAAGGAA
10 TCF7L2	AAGCCCCACATAAAGAAACCTCTTAAT	CCTGAGAAGGGCGTCACCTA
10 TCF7L2	TGGGTTGCTTTCATGTGAGTGT	CGTATGATGTGGTGGGACCAT
10 TCF7L2	TTTCAGTTTCTGACCCATGTCACA	GCAGTGGCCATTTCATCTGGA
10 TCF7L2	ACTTTTGTTTCCTCTCGCCAGTATC	CCATCATCTCTGGGAGGTTACTTAC
10 TCF7L2	GCCCAGACACTCTTCTCACAT	CTTCCTCTAACCTGATGATTGTCACTTAC
10 TCF7L2	CTCGACCTCGCCGATTCTT	gggcgTACTCACCTTCTTCC
10 TCF7L2	CTTTCTCATCTGTACCCCACGTC	GACTCCAATCCTAACGAAAATGATCCA
10 TCF7L2	TTTGGCTTTTCTTCCTCCTTCATTTTTC	AGGAGTTTTCGGAGCTCTTCTC
10 TCF7L2	ACCTCTGTGGGACATCCCTTA	CTTGTATGTAGCGAACGCACTTTTT
10 TCF7L2	CGAACTGATTTCCTTCAAAGACGAG	CGGAGGAGCTGTTTTGATTCGTT

10	TCF7L2	GTCAAATCGTCTCTAGTCAATGAATCAGA	CGATTTCTCCCCTTTCAGCAACAT
10	TCF7L2	GATAACTCTCTCCCCTGTTTCTAGGA	GTCTGCTCAGTCTGTGACTTGG
10	TCF7L2	CCCTCCCCGAACCTGCTA	CCTTGTGGGTGGCCTCA
10	TCF7L2	GCTGTCGCTCGTCACCA	AATGTAGCCACATGGCACAAAATTAAG
10	TET1	CAGCAACCAATTGAAAATAAAATGGGA	GCGGTTATCTTCTCGAGTTAAGGT
10	TET1	TTCATGTTTTTCTCCCTATCAGGTTTGT	CCCAGATTTGATCTTGGCTTCCAT
10	TET1	GAGTTTGGCTCCAAGGAAGGA	TCTTTTCCACTGCCCTTATCTTATGTG
10	TET1	CCTTATGCTCTCACACACGTTG	TCAGACTTGAAATGGGTGAGATGAAG
10	TET1	TGCGATGATGACAGAGGTTCTTG	GCAGAGATTACCAGGTTCCATAGG
10	TET1	AACCAACACAACATCAGCGTTTT	TTCGGTTTCACTTTTTACTTCAGGTTG
10	TET1	AGGGAGTAACACTGAGACCGT	GGTGTGGCTGAAGCAGTCTT
10	TET1	GAAAGAGGCATCTCCAGGCTT	CATTGGCACCACTGAGTCTTC
10	TET1	CCACTGTACGATGCCTTCGG	CACCAGTGGAAGGCTCAGAAT
10	TET1	CTGTGATGGAGCCCCTCATTA	GAATGCTGCTCATCTTCTTCCATTG
10	TET1	CCTCAAGACCTTGCCTCTTCTC	GATGTGCTCACTGTCTGACCAAT
10	TET1	AGAAATTGCCCCACATTGATGAGT	GGGTTGGATGATTACGGTTGGG
10	TET1	CACGCTACCACTCCTGTTGA	AGCTTCTTTAGCCTCAAACTTAATCTTGT
10	TET1	AGCCCCAACATGGTTTTGAACTA	GGTTCAATTCATTTACTTCAGAGGACTGT
10	TET1	CAGGCAGCTAATGAAGGTCCA	AAAGCCTTCAGACCCAATGGTTATAG
10	TET1	CATTGCATTCAAATTTGGCATACTTGT	CCACACCATGATGAGCACCA
10	TET1	CACTGTCCAACTGCTGTGATG	ACTTTTCATTGAGGGTGCATCTTCT
10	TET1	CGACAGAAGATGCACCCTCAA	TCCACAATACTCAAATCCTATTCTCCCA
10	TET1	GATTGTGCTCCATGCTTTCTTTTCT	GGTACATACCACAGTGCTTCCATTAT
10	TET1	CCACAGGGACATTCACAACATGA	ATTAAGATTTTTCACAGTAATAGTCATCACAGT
10	TET1	GATGTTCAAAGCCAAAGCCTTCA	CTTCTTTACCGGTGTACACTACTATTTCT
10	TET1	GGTCAAAAAGGAAACGCAATAAGGAT	CAGCTCTGGAATAACAAGCTTCAAAAA
10	TET1	TCTATGGCTGTTATTCAGATATTTAAGTAGATG	TCAACAGTATGTGGGTTCAATTCCAAT
10	TET1	CTCTCTGAGGTATTGTAATGCTTCATCA	ATACTCCATGAACAGCCAAAAGAGAA
10	TET1	CCAGAGACTTGTGGAGCTTCA	ACATGTAAGGGAGAGCTTGGA
10	TET1	GACTACAGTAGATGTGGTCATGGG	ACTAAAATCTCCTTTCACGTGATCAGTT
10	TET1	GTGGACACAAAACAAGAAATCACAGT	ACATGTTTAATGCCATTTCTTACGGTTTG
10	TET1	TCCTCTCCACCTAACCAGTGT	CCTGAGAGTTAGTGCTGACTCC
10	TET1	CCATCAAGAGATCGGCGGAAA	ATCATGTGGACAAAATTGCCCAAAAT
10	TET1	TTCACACTCCATCATAAATCATCATGCT	CACTTGCTCCAATGTTAATCTCCTATCT

10 TET1	AAGAAACGAACCAAATCTCCAAAATTGTT	TCCAATGTCTTGCCGAATTCTTCAATAT
10 TET1	GTACACTGTTTACCAGCTGAAACAAAT	CTCCCCTTGAGATGATCACAACTC
10 TET1	CAAGCATCCACAAAGTCACATGAATAT	CACTTCATTATTTCTTGGTGGCAACTG
10 TET1	GTTGTTATTTCAGACCAAAACCTTGTGT	GACTAATCTGGAAGGCCTTGCAT
10 TET1	TCTTGACAATTGTTCCAATGATTTGCAT	CAACATCTTCCTCAATTTGGGTATGG
10 TET1	GCTATGAGCTCTGTTGCTACTGATAT	TGGTTGATCACCTTCATTGTGTATACT
10 TET1	CCCAGAGTCCTTAACCTGCAAT	ATCACAATCATGTTGCTTTTCTAAACCC
10 TET1	CGTTCAACCAAGTCTCTTATCGTTAATGA	AGCTATTAAGCAAACTGGCTGTTCT
10 TET1	CAGATGATCTATCATGTCAGGATGCAA	AGGCTTGTTGGTGTACTTTCAACT
10 TET1	GCGACATTTGGATAGCATCGAAATT	CTGAGTGAGTGGTGGAAATACTGT
10 TET1	CAGTTGACACAACTTGCTTCGATAATT	ACTTGAAGGTGGTTTCTGTTGTATTGT
10 TET1	GGTATCTCTCATGAAACACCCTTACC	TTTCTGTGCACTGTCCACTGT
10 TET1	CCTTCCTGTTCCTCCAAATCCAAT	CTGAGTTTGATGATGATTGAGGAGTGT
10 TET1	CCAAATCCAAAAAGGTTCCACTTTCTAA	AAGGACTTGGGTGTCTTGCAT
10 TET1	CAGCAAAAATACAATCAGGAGAAGGG	TTGATAACTTACAACTGTGGGCTTCTT
10 TET1	TTTTCCTACTCTGTAGCTATGTCTCGAT	TTCCAGGGCTTAAAGTCTTGACTG
10 TET1	GCACTCAGAAAATGATTCGGTTCCA	CCACTGTGGGTAGGGATATTGATCTT
10 TET1	CGAGGTCCATTATGCAACCCAAA	TTCCCGTTGGATTCCGTTTTAAGA
10 TET1	CTTGCAGTCCAGGGTGCTATAC	GTGTTAGCTGGCAGGAAGCTTAT
10 TET1	GAGCCAACAAAAATGTGGCATCA	CTGCTCCAGCTCTTGTCAGAA
10 TET1	GTTGGGTTCACGAGTAGAATCTCTT	TTTATTACAGAGGTAGGAGACGTAGACC
10 TET1	AGAGAAGGATGAGGAATCAGAGCA	CGTGTTGCATGAACTTTGTTTTTCC
10 TET1	GTTCATCCAGTTTTGGAACATCAGAATT	AGAACACATGCACACAAGTACTCA
10 TET1	TTGGTCCCAAAGAGTTGAGGATTC	CACACTGTGTCCTGAGCAAAC
10 TET1	CTAAACCACCCGTGCCAGT	CTTCGTAGCGCCATTGTAAACC
10 TET1	ACCAACTGTGGTGAATGCACT	GCCCCTTGACTGTTTGCTTAC
10 TET1	AATGATACCAGTGGTTCCCCAAAAA	TGGGATCCAGGTAAGAATCAGATAACT
10 TET1	CAATGTAGAAAATGAGAAGCAGGTTCAT	CTCTGTCTGATTTGCTAGGACCA
10 TET1	GAAACTGCTTGGCTCTTGGTG	CAAAGGCCTGTCCTAGGAAAGA
10 TET1	CACCAAAACTTAATCACTGTCCATCTTT	CATGTGACTTTGTGGATGCTTGG
10 TET1	CCAACTCACATATCAACTCAGCTACTAA	TGAGCAATACTTTTATTGGTGTCAATCT
10 TET1	ACCATTGGATTCACTCAGCTTATTTCA	TCGCCTGCCATCACGTTAG

10	TET1	GGTATAGCCCAACTCTCTCAGG	TCTTCTTTTCCAAAGTCGGTAGCAA
10	TET1	GCTACTGCAAATCAACAGGAAGTTT	AGACCTCTCCTTGGACTGGAAT
10	TET1	CCCCAGATCTACCAGAGATTCCT	ATTTGGAAGGAGCATTAAAGGTAGCA
10	TET1	GTACCTCCTCTTCTTCCTATACCACT	GGCTGTTCTTTCTGTTCTTGCAGTA
10	TET1	AAACAGAGGCTCATTTTGCAATTTGAT	ACTCCAAATATACCCAAGTGCAGAAAAA
10	TET1	TCTCCTGTCCCCTATGCAATTCT	ACAGGGAAGCGTATTTCCTCAC
10	TET1	AGCCATGATTTATGATTCAGTTGTACCT	TGATTTTGGTAAGCTACTGGAGCAT
10	TLX1	GCCCTCTCACCCTTCACTGTAA	GGTACTTCTGGCGGTGGAA
10	TLX1	CCTTCACACGCCTGCAGAT	GGTTCTGGAACCAGGTTTTGAC
10	TLX1	GCCAAGGCGCTCAAAATGA	CAATGAACCGAGAGCTGCCT
10	TLX1	GCCTCACCTTCCCCTGGAT	AAGAGCTGGGCGACCTG
10	TLX1	CCAGCATGGAGCACCTG	AGCAGCCAAGGCCGTATT
10	TLX1	GACCAGGGTGGCTGCAT	ccgggACCTCCAGTACCATAG
10	TLX1	CGGAGGCGCCTACACTTAC	GCCTGCCAAGGCCATGTT
10	TLX1	GCAGCGCTCGTTCAGTTTATCT	GAGGATGCGGTTCGCTTG
10	TLX1	TGGCAGGTAACGGCTTGT	CGAAGAGCGACGAGTTGTG
10	TLX1	GCTGACCCTCTGTGCGT	GGTCCCACAGGGCAGAATG
11	ATM	AGTTCTGTTAAAGTTCATGGCTTTTGTG	AGCGTTTACGATCCTCTTTCAGTG
11	ATM	ggaggttTCTGAAATGTGTATAGCTTGT	TCATCCAAAGTTTCAGGGTTCTCAG
11	ATM	GTCCCATAGTGCTGAGAACCC	ACAAGGTGAGGTTCTAATCCATTCTCT
11	ATM	CGAGAGCTGGAGTTGGATGAATTAG	ACTCCAGAATTTTCAAGCCAGAGG
11	ATM	TGTTTGCCCTGTGTAAATCTGTGAA	TGCCACTCAGAAAATCTAGCTTTAACT
11	ATM	ATGATATGTGGGTATTCCGACTTTGTT	tgtctaAGTTTTTCAAGCACAGGGT
11	ATM	AATTCAGAAACTCTTGTCCGGTGT	CCTACCTTGGCTTTCTGGAATAATTCT
11	ATM	GATAGAGAAAACACTGTCTGCCAAGAA	CTGCAATGTCATTCATTAGCTTTGATGT
11	ATM	GGTGTAATAGCTGAAGAGGAAGCATATA	GGTTAACCTGCATAAAATTTAATGTCTTCCA
11	ATM	AGGTGTGTAAGCAAGAATGCCT	TCCTAAACGTAAGAAGCAACACTCATT
11	ATM	ACTCCTGTTTAGGCCTTGCAG	TCCATGCTGCTTGGTAATGAAGT
11	ATM	AAGACTGGTGTCCTGAACTAGAAGA	TTGTTTAGAATGAGGAGAGAGGCAAAA
11	ATM	CTGAGAAGTTTAAATGTTGGGTAGTTCCT	GCCTCAACACTTCTGACCATCTG
11	ATM	TCGAACAGAGGCTGCAAATAGAATAAT	TCCACTGAGTGGCATCTAAGTTTG
11	ATM	GTTGAGGCACTTTGTGATGCTTATATT	TCTCTACAGAGAGTAACACAGCAAGA
11	ATM	AGAATTAGAGATGCTGAACAAAAGGACTT	CAACAAAGGGAGAAGCTACGTAATGA
11	ATM	ATTAGGCCTTCTTGTATCATGGATGTG	AGTGTACCAACAATAACATGAAGATGGT
11	ATM	CCAGACAGCCGTGACTTACT	ACCATTTTGAAGATGAGTCAGAAAATTACCT
11	ATM	TTAAATTGGTTGTGTTTTCTTGAAGGCA	GAGTATCTGAAAACCGGGCTAATGA

11 ATM	GAGCTAAGAAATGGAAAAATGAAGGCAT	AGACCTACTTCCTCTTTGGCTCTT
11 ATM	TGAAAACAAGCAAGCTCTCCTGAA	TCTTTCTATGCAAAAACACTCACTCAGT
11 ATM	CTGGAATATGCTTTGGAAAGTAGGGT	GCATTTTCAAAAGCTGTTTGCTGAA
11 ATM	CCAGGTTACTGAAAGCACTTCCTT	ACCAAACTTGGTGAAGTAATTTATGGGA
11 ATM	ATTTAAATAGTTGCCATTCCAAGTGTCT	GCTCCGTAAATAGCACCATTAGATGAAT
11 ATM	GCACCTAGGCTAAAATGTCAAGAACTC	TGTTGCTGAGATATTTCACACCAGTATT
11 ATM	GAGCTGATTGTAGCAACATACTACTCAA	AGACAGATACAGACAGAGTGCTTTCT
11 ATM	AGTGCAGTTTTAAAATCCTTTTTCTGTATGG	CAGATAGAGCCTGAAGTACACAGAGA
11 ATM	TGTTCTCTGTGTACTTCAGGCTCT	GCACACTGAATAGCCTTGGAAAAA
11 ATM	GGCTCTATCTGAAACCTTCACAAGAT	AAAGAGATTAGATTACCTCGCACACTG
11 ATM	CCAAGGCTATTCAGTGTGCGA	AGGAATCCACTAGTTCTGTTATGATGGA
11 ATM	CAGCAAAGAAGTAGAAGGAACCAGTT	CAAGTTTTCAGAAAAGAAGCCATGACAT
11 ATM	ACGCTCTACCCACTGCAGTA	GGCCAGTATAATAAACAAAGTGTGATGG
11 ATM	AATTTCAATGGATCACCCCCATCA	GAGAGCTTTGTTTAGGCACATTTTTAGT
11 ATM	ATCACCCCCATCACACTTTGTTTATTAT	GCTGAGAGCTTTGTTTAGGCACA
11 ATM	CTAAAAATGTGCCTAAACAAAGCTCTCA	CCTTGAACCGATTTTAGATGGCATCTTA
11 ATM	TCTGTTAAGCAGTCACTACCATTGTATTC	GAGCTACCTTGGCAACTTCTAGAT
11 ATM	CTAGAAGTTGCCAAGGTAGCTCA	ACCCTTATTGAGACAATGCCAACATTAA
11 ATM	GAAAATTCTTCTTGCCATATGTGAGCA	GCTCCTCCTAAGCCACTTTTTATATCTT
11 ATM	CATATGTGAGCAAGCAGCTGAAA	ACAAAGGCCCAAGCTCCTC
11 ATM	GATATAAAAAGTGGCTTAGGAGGAGCTT	GGAAGAACAGGATAGAAAGACTGCTTAT
11 ATM	CTTTACATGGCTTTTGGTCTTCTAAGTG	CATTCTGGCACGCTTTGGAAAAA
11 ATM	CTCACTATGAAAAACTGTAAAGCTGCAA	AGATGCAGCTACTACCCAGCTA
11 ATM	ACAGGAGAATATGGAAATCTGGTGACTA	CACTAAAACTCTAAGGGCTAAGCCA
11 ATM	CAGTTTGTTCCCCCTGTTATACCC	GCAAAGTGGGAAGAATTTCATCTCCTAA
11 ATM	GACTTTGGCTGTCAACTTTCGAATT	GGGTTTTGGCTCCTTTCGG
11 ATM	CTGCAAATTTATATCCATCATCCGAAAGG	ACCAGAGAAATCCAGAGGAAAGTCATAT
11 ATM	CTACCATGTGACTGGCTTATTTGTATGA	CCTCTGCTGTAAATACAAAGCTTTCAA
11 ATM	CTCTTTGACTGGACCATGAATCCT	ACAGGACCTTCTTAAAATACTGCTCAC
11 ATM	ACTCATTTTTACTCAAACTATTGGGTGGAT	CCCTGAACATGTGTAGAAAGCAGATTT
11 ATM	GTCAGACTGTACTTCCATACTTGATTCAT	GGTTGTGGATCGGCTCGTTT

11 ATM	CCAGCTGTCTTCGACACTTCTC	ATCCTGTAAAGTGCTTTTAGTGGGATT
11 ATM	AAGGCAAAGCATTAGGTACTTGGT	TCCATCTTGTCAAAAGTTGTCTGAAGAA
11 ATM	TCTTGAAGTGAACACCACCAAAAAGATA	CCTTGAGATTCTGGTGGACAGAGA
11 ATM	GCACCAGTCCAGTATTGGCT	TCCTTCCTAACAGTTTACCAAAGTTGAA
11 ATM	CAGTAGCAAAGCCTATGATGAGAACT	GGGATAGAGCGAATACACAGACTC
11 ATM	CAACATTGGTGTGTAACAAAATCCGT	GATGAGATACACAGTCTACCTGGTAAGA
11 ATM	AGATGTGTAAGCGCAGCCTT	ACAGAAAAGCTGCACTTTAGGATAACA
11 ATM	CTGTTCCTCAGTTTGTCACTAAAATCTC	GCAAATTACCTTAATTTCCATAGTAGGGACA
11 ATM	GGCACTGTCCTGATAGATAAAGTCTTT	TGCTCTATTCCCATTTTTACCGTTCC
11 ATM	CACTGACCACCAGTATAGTTCCA	CCTGTGAAGAATTGGAGGCACT
11 ATM	GAGGGTGACTTAGAAAATAGCACAGA	CATCTGGCATCAAATAAGTGGAGAGA
11 ATM	GAGAAGAGTACCCCTTGCCAAT	AAAGAACACACATTGCTGTGGTAAAAA
11 ATM	TTACATGAACTCTATGTCGTGGCATT	CCATGATAGGCTCCTGAAAACTAAAATC
11 ATM	TCCCAGCTTCTCAAGGACAGT	TTCTGGCCAGTATAGAGAGTTCTACAA
11 ATM	TGTATTAAGGACATTCTCACCAAACACC	TCTTTTTCCCTCAGGCTTTCTGTT
11 ATM	TTCTTGAACTTCTGAAACCACTATCGTAA	CAAACATCTTGGTCACGACGATAC
11 ATM	TTTACCACAGCAATGTGTGTTCTTTG	CTGTCCTTGAGCATCCCTTGT
11 ATM	GGTCAAAGCAATATGGACTCTGAGAAC	GCATTCGTATCCACAGATAGCAAAAGAA
11 ATM	TGTTCATAGAACGTAGGTAACATGTGG	AACTGTCTCAGGAGTAGGAAGGATT
11 ATM	TTTTAGGCTACAGATTGCAACCCA	CTTCCGTAAGGCATCGTAACACATAT
11 ATM	ACTATCTCAGCTTCTACCCCAACA	AATCTGACTTTTGTGAGCTTTCTAGGTT
11 ATM	GTGTTGCTTTTGAACAGGGCAA	cccagcCCATGTAATTTTGACA
11 ATM	GCATTGTGTCAAGACAAGAGGTCA	CTAAACTACCCTGAATTATGGCTCCAA
11 ATM	CCATATGTCATTTTCATTTCAGCTCCCT	TGAGAATACTCAGGGCAAGACTCT
11 ATM	CAAATACAAGCTGAAAACTTTGGCTTAC	GTCAGACATAATGCATGCTGAACTTAC
11 ATM	GAAGCACAAGTATTCTGGGCAAAA	TTAAATAACAGTAAAACACTAATCCAGCCAATA
11 ATM	CCTTTCAGTGAGTTTTCTGAGTGCT	ACCTCAAGCAAAGTTTTAAGGCAATTT
11 ATM	TGATTTATTCTGTTTTGTTTGCCACCTT	GCTAGAAATAGTTGTATTCTGGCTTCCT
11 ATM	TCTTGCATTTGAAGAAGGAAGCCA	ACATGGCATCTGTACAGTGTCTATAAC
11 ATM	TTTCTCCAGTTGGTTACATACTTGGAC	GCAAACAACATTCCATGATGACCAAAT
11 ATM	GGCTGTTGTGCCCTTCTCTTA	TGCCCTAAAGGACACAGTATTGG
11 ATM	GTGTTTGTGTGTAACTACTGCTCAGA	AGCAACATCAGATAAAATCCAAGAGCTT

11 ATM	GTACATGAAGGGCAGTTGGGTA	GCTAACCAGTTGCCACAAACC
11 ATM	CCAGCCTAAAACTTACATACACAGAATG	AAGGGTTGCTCCAAAAATCTTACCT
11 ATM	cggccTTAAGGTTAATTCTTGAAGTACA	TCTATAGGACCCACTTCTCCCAAG
11 ATM	CTATATGTAGAGGCTGTTGGAAGCTG	AGCATTATGAAGGTCCACTGAAGTT
11 ATM	ATGATCTGCTTATCTGCTGCC	GCGCTTAAATTTCTCAACTTCTTTCTGA
11 ATM	ACTAGAACATGATAGAGCTACAGAACGA	GCCGATCTAGATGTTTAATTGTTTCAGG
11 ATM	AACTGGTGTACTTGATAGGCATTTGAA	CCAGCATTGGATCTGTTGTCATCTTATA
11 ATM	AGACTGGACATAGTTTCTGGGAGATT	CCACAGCAAACAGAACTGTTTTAGA
11 ATM	TGGAGGATCAGTCATCCATGAATCTA	CAATGAGGCCTCTTATACTGCCAAA
11 ATM	CCTTAAAACTTTGCTTGAGGTGAGT	CCCATTACATTAAGAATGGCCCATTTTG
11 ATM	GGTAATATATGCCTTTTGAGCTGTCTTGA	GCACTAGAATTTGCTGGCTCATGT
11 ATM	CTTTTCCCGTAGGCTGATCCTTATT	GCAGCCAACATGCGAACTTG
11 ATM	TTGTGGTGGAGTTATTGATGACGTT	AGACATTGAAGGTGTCAACCAATAAACT
11 ATM	ACACAATTTCTTGCTGACAATCATCAC	GCAACTGTGAGCTGTTACTATGTAAGAC
11 ATM	TGAAGTCTTCATGGATGTTTGCCA	AAGTAGCTACACTGCGCGTATAAG
11 ATM	GCTATTTGGTTTGAGAAGCGATTGG	CCTGCAACTCAGAATGTAGAAAAAGTG
11 ATM	TTTATGAAGGAGTTATGTGTGTGTAAAACC	ATTGCTGTTTCGAGGTCATATGTTACT
11 ATM	TGGATTACAGGTGCTTATGAATCAACA	ACGGCAATATTACGAAATCCTGAAGAAT
11 ATM	GGATTTCGTAATATTGCCGTCAAAGAAA	GGCTTTTTGTGAGAACACAGGTTTTA
11 ATM	GATCATCAAATGCTCTTTAATGGCCTTT	CCTGTATCTTTTATGAGCACCATCTTCA
11 ATM	TGTCCCCATTGGTGAATTTCTTGTTAA	ACAGCTGTCAGCTTTAATAAGCC
11 ATM	TCATTTTGGAAGTTCACTGGTCTATGAA	GTTGCTTTAATCACATGCGATGGA
11 ATM	CCTGCTCCTAATCCACCTCATTT	TCATCATGTATACTTACAGGGCTTTTGG
11 ATM	AATGCTGATGGTATTAAAACAGTTTTTAAGAA	GCAATGGACTTCACCTCATCAAAATG
11 ATM	GAGACTGTCAAGAGGTGCACA	TCTGGAAGACCTGTTGCATGAC
11 ATM	GGTTTTGATTCCACATCTGGTGATTAGA	GTACCCTCATAGGCAAAATAAGGAAGAAT
11 ATM	ACAGACTGCTTTCCAAAGATTCTTGTAA	ATTGAAGCCATACCTGTTTTCCCA
11 ATM	GTGATGACCTGAGACAAGATGCT	cccaaccAAATGGCATCTTTTATATGTT
11 ATM	CCAAGATCAAAGTACACTGTAAAAAGCAA	CTGCATCATATTTCTCAAGGAACCAAT
11 ATM	GTGCAGGAGAAAGTATCACTCTGTTTA	aagaaaaTCTTACCTTGGTACAGTTGCT
11 ATM	GGAGGCACAAAATGTGAAATTCTTCAAT	GGTGATATTATGTGAAGATGATGTGCAG
11 ATM	GTTCACCTCACTGAAACCTTTGTG	CTATGGCCTGCTGTATGAGCAA

11 ATM	TCAGTGTTGGTGGACAAGTGAAT	GTATGTTGGCAGGTTAAAAATAAAGGCT
11 ATM	GTGCTAATAGAGGAGCACTGTCTT	GTCCCCATTCTAGCAGCCAAT
11 ATM	GAGTTGGGAGTTACATATTGGTAATGATACA	GGCTCCCCTATACTTCTGTAGATTTCT
11 ATM	CCAGATAGTTTGTATGGCTGTGGT	TTGGGTTTTACACACACATAACTCCTT
11 ATM	TCACAGGCTTAACCAATACGTGTT	CTTCGAAGATCCTTTAGTCCTTCAAGTC
11 ATM	GTGTTTATGATGCACTTCCATTGACAA	GAAACAGGTAGAAATAGCCCATGTCAT
11 ATM	TGAAGATACCAGATCCTTGGAGATTTCT	ATTCTGTGACTTCTGAAGGTGATCTTTT
11 ATM	GAAGAAAATAGAACTAGGCTGGGAAGTAA	GTTCTGGTTGAGATGAAAGGATTCCA
11 ATM	AGTTGCTCTTTGTGATGGCATGA	GGCTTGTGTTGAGGCTGATACATT
11 ATM	ACAGAATGTCTGAGAATAGCAAAACCA	GCGACAGTAATCTGTTAAGCCATTTATT
11 ATM	TGTAGGAAAGGTACAATGATTTCCACTT	CACAGATTTATATCATCCAGGCCTTCA
11 ATM	GGCCTGGATGATATAAATCTGTGGAT	ACTAATTAATCTTCTTACTTCACACATTGGCT
11 ATM	ATGATCTGCTAGTGAATGAGATAAGTCATA	ACCTACTTACTGTACCTGGTGACA
11 ATM	GAAGTGGGTCCTATAGATTTCTCTACCA	TCACCAATCTTCTACCAGTGTGT
11 ATM	TGTATTTTTCTTTAAGTGCAAATAGTGTATCTG	CACCTCCTGCTAAGCGAAATTCT
11 ATM	CCATCTTGAACATCTTTGTTTCTCTTC	TGCATCACTAACACTACTATCAGGGTAA
11 ATM	ATATTCTCTGTAAGAATGGCCCTAGT	ACGGGAAAAGAACTGTGGTTAAATATGA
11 ATM	CGAACATATGAACACGAAGCAATGTG	CCAACATACTGAAATAACCTCAGCACTA
11 ATM	AGCTATCCAGGATATGCCACCT	AACACCGGACAAGAGTTTCTGAA
11 ATM	CCGGTTATGCACATCATTTAAGTAGG	TCTATCACCAAGTCCAAGTATGTAACCA
11 ATM	GAAGTACCCAGATTTGACAAAGAAAACC	CACAAGTCAGTGTCTTTATCCAAATGTC
11 ATM	CAATTTTTAATGATGCTTTCTGGCTGGA	CATACTTTTCTTATCTGCATAGATTTCTGC
11 ATM	TTAAATACAGAAGGCATAAATATTCCAGCAGA	TCAAGAGTTAATTGCAAATTACCTTAATTTCCA
11 BIRC2	ATGAAGTATATTCCAACAGAAGATGTTTCA	GTTCTTCCAGTGACAGACCTAATAACAA
11 BIRC2	CCAACAGAAGATGTTTCAGGTAAAACAAA	CGTTCTTCTTGCAACCTCCTCAAT
11 BIRC2	GTTATTAGGTCTGTCACTGGAAGAACA	GATTATACCCCTGCAAATAGGGCAT
11 BIRC2	GGAATGTGCCCCTTCTCTAAGAAA	CTTTAGATGGCTTCAAGTGTTCAACAAT
11 BIRC2	GTGCCTAAATTGTTTGTAGGGTTGG	CAGGATAGGAAGCACACATGTCAAT
11 BIRC2	AAGAACAGAATGGCTCTCTTTCAACA	CCGCAGCATTTCCTTTAACCAAAAT
11 BIRC2	ACAAGCGAGAGAACTGATTGATACC	CATCTGGCCAGTCCTAAGAATTTCTAA
11 BIRC2	ACAAGGTCAAATGCTTCTGTTGTG	TTGGAGACGTATTCTTAGAGGTGGA

11 BIRC2	TCTGGTTTCAGCTAGTCTGGGA	ACTGCTCTAGAATTAAGAGGGTTTGGA
11 BIRC2	AGTGGTTCTTACTCCAGCCTTTC	CTTGCCAATTCTGATGGTGACAAAA
11 BIRC2	CTTACCTACCATATGTGGCCATTAACTT	ACATAGCATCATCCTTTGGTTCCC
11 BIRC2	CTGTGGTGGGAAGCTCAGTAA	TCAGATTTGAAATGCTAAACCTCAGAGT
11 BIRC2	GCATATAGCTCATCTCCTTTACCAGAAT	TCATCATGACAGCATCTTCTGAAGAAC
11 BIRC2	TTCATTTTGGACCTGGAGAAAGTTCTT	GTTTTATAGTTCTCTCCAGTTGTCAGGAT
11 BIRC2	CCTGGTGAAACAAACAGTTCAAAGTAA	GCAGGTGACTAACATCCCCAA
11 BIRC2	CTGGTTTTTATTATGTGGGTAAGAAGCA	GCCACCATCACAACAAAAGCATTT
11 BIRC2	CTGAAGGTCGCAATGATGATGTC	GCCTACTTCTTTTAAGCACAGAGTTTTT
11 BIRC2	CAGTACTGTCACCTACTCATGCAC	GTTTGTTGCTGTTTGTCCAATCTGA
11 BIRC2	GGTCCCTCGTATCAAAACATTAAGAGTA	AGACTCCTTTCTGAGACAGGCA
11 BIRC2	TCCTGTGAACTCTACAGAATGTCTACA	CCTAGTTTCCAGTTATCCAGCATCAG
11 BIRC2	TTCCCAACTGTCCATTTTTGGAAA	CAAGCTGCTCAGGCTGAAC
11 BIRC2	TGTACTGGCCATCTAGTGTTCCA	TCATCATTGCGACCTTCAGGAAAA
11 BIRC3	GCTGCAGATTCGTTCAGAGTCT	AGCCACGGAAATATCCACTGTTT
11 BIRC3	ACACTCATTACTTCCGGGTACAGA	CCATGTCTGAAAAGTAAGTAATCTGGCA
11 BIRC3	CTACCACTGTGCAATGAATAACGAAAA	CCTTCGGTTCCCAATTGCTCA
11 BIRC3	GCTTTGCCTGTGGTGGAAAAT	TGCATGCTCAGATTAGAAACTGTGTA
11 BIRC3	AGAAAATCAGCTTCAAGACACTTCAAGA	ACCCACATAATAAAAACCCGCACTT
11 BIRC3	ATGTGGGTAAGAAACTGAATCTGCtaatt	CAACACCTGAGTCCACCATCA
11 BIRC3	GGTAACAGTGATGATGTCAAATGCTTTT	AGGTGAACACATATAAGCAATCTCATGAT
11 BIRC3	GGAAGGAAGTTTGTGAGCAGAGTT	TCCAGGATTGGAATTACACAAGTCAAAT
11 BIRC3	CGGAAGAATAGAATGGCACTTTTTCAAC	TTGCTTGTAAAGACGTCTGTGTCT
11 BIRC3	GCCGGAATTATTAATGAACAAGAACATGA	TGCTCATATAACACAGCTTCAGCTT
11 BIRC3	ATAATGCCTATACATTTTGTTGGTTTTACATT	CTTCTGAATGGTCTTCTCCAGGTT
11 BIRC3	CCATTTTGAACCTGGAGAAGACCA	GCTAGGATTTTTCTCTGAACTGTCTGTT
11 BIRC3	CCGTGGAAATGGGCTTTAGTAGAA	ACTTGATTCTTTTTCCTCAGTTGCTCT
11 BIRC3	TTTTGCCAGTAAGCAAGTGGTT	GCATTTTCATCTCCTGGGCTGT
11 BIRC3	ACTGATAAAAGCAAAGCCATGCAC	GGTACAGTTCACATGACAAGTCGTAT
11 BIRC3	CGCCAACACGTTTGAACTGAA	CATTTGACCTTGTCATTCACACCAG
11 BIRC3	GGCAAACAACCAGATTTGAAATGGA	AACTGGCTTGAACTTGACGGAT
11 BIRC3	TTGCTCGTGCTGGTTTCTATTACA	GTAGCTTCCAAGTTGTTAACGGAATTT

11 BIRC3	GAATTAAAGGACAGGAGTTCATCCGT	TTGTCTTTTCACTCAAATAGCACTCTCT
11 BIRC3	ATCCTGAGCAGCTTGCAAGT	AGCATTTGACATCATCACTGTTACCTAA
11 BIRC3	TTTTCACTGAAGAAGCAAACTGCC	ACACTTCTTTGTCCATACACACTTTACA
11 BIRC3	AATTGCGGAGACTACAAGAAGAAAGAA	CTGTACCCTTGATTGTACTCCTACAAAT
11 BIRC3	GTAGTATGCAAAGATTGTGCTCCTTCT	AATTAATTCTAAAGTTTAGACGATGTTTTGGTT
11 BIRC3	AGACTTCTGTTGCCTTGAAATGAGT	CTATTACCTGAAACATCTTCTGTGGGAA
11 BIRC3	CTCCCTTAGTGCAACAGGACATAAAATAT	TCCACTGGTAGATCTAGAAAGAGGAAAG
11 CBL	TTCCAAAGATCATTATGGCACTTTCCT	ACTTACCAAAGGTGCTGCTCTC
11 CBL	CCCAGGCGCCATCTATCAC	GCTTAGATCAAGCTATCTCAATTGCCA
11 CBL	CTCTGTTATTTCACTTTATGCCTCCTCT	ACCGAAATATAATCATTGCAGGTCAGAT
11 CBL	CCATGGCTCTGAAATCCACTATTG	CCGAAGTAGCAGTAACCAGTTTAGT
11 CBL	ATTCTTTGCTGTGTACTAGTGGGTTT	CAGAATATGGCCGGTCTGGAG
11 CBL	CTTCGAGATCTTCCACCACCAC	AGTCTCCAAGGCGGCTAGA
11 CBL	CTGTCCCTCCAGAGACAAACTG	AAGTGAGTGCCGGTTGGTTAA
11 CBL	TGATCCCTGGACAGGAAGAGAA	gccCACACATATTTCTTAACAGGTTAGA
11 CBL	ATTTCTGACAATGAACTGAGAGTTGGT	GGCTGTTCCAATTCCTGAGCA
11 CBL	GTTTCTTTAGCCCTGGTCCTCTT	GAATAAATCTCTTTGCGTCATTGAACTGA
11 CBL	CTGTATCTTGCCTTGCCTTCCA	ATCAGCAGTAACATACCCAATAGCC
11 CBL	CTGTACTCGTCTGGGTCAGTG	ACTCTAACAGATGGTATGCAGTAGGT
11 CBL	GGCATGGCATTGAGAGTTAGGTT	GTGGTCACACTCTGGACCTACTA
11 CBL	TCCAGAGTATGAATAGCAGCCCAT	agcTTTTTACAAGTAACAACTCCACTGT
11 CBL	AAATGAATGGCTGCCCCGTA	GGACATCATACCCATCATCCTCAT
11 CBL	TGGTCCCGAGGAGTCAGAAA	CCCAAAGTAGTCTGGAGACTCAC
11 CBL	GATGCATCTGTTACTATCTTTTGCTTCT	GCTGCCACTCCCTCTAGGAT
11 CBL	CCCATCGTGGTAGATCCGTTTG	CCAGTCTCCTAAACTGCCATCTTA
11 CBL	TGGCCAGAAAGAATATTTGAAGAGGT	GAAGATGAGGGACAGTTTGGTTAGG
11 CBL	GCATTCTGATCATTTGTAGGCGAAA	CTTTTCTCCAAAATTCCGCAGCA
11 CBL	TGCTGCGGAATTTTGGAGAAAAG	ACCAAAGCCAGGAAATACATACACAT
11 CBL	TGTTGTTAAATGAGGATTTCCCCAGAT	AGGACCACTACCTTGCTGACA
11 CBL	GAATCAACTCTGAACGGAAAGCTG	CTCGATGTTGTTCTGGGCAATG
11 CBL	AGGACATCCAGAAAGCTTTGGT	CTAAACCTGCAGCAGGGAGAT
11 CBL	GGAAACAAGTCTTCACTTTTTCTGTTAACAT	TTGGAATGTGGAGCCCATCTC

11	CBL	GAATTATACTGTGAGATGGGCTCCAC	AGATCCGTACCTGCCAGGAT
11	CBL	GTAAAGATTGAGCCCTGTGGACA	AGGCCACCCCTTGTATCAGTA
11	CBL	GGGAGCTGTCCTTAAAATTCTCCAA	TCTGGTAGCAGGTCTAAGATATAAGGTG
11	CBL	AGCTGGCGCTAAAGAATAGCC	GGCTTATGGTTTGCTTAGTTTTCTTCAT
11	CBL	GATGGAGACACTTGGAGAAAATGAGTATT	CAGTCACAGACCTGCATAATTATTTGT
11	CBL	CCCAGGGTTGGTTACTCTTTACA	CTAAAGCTTGTGTCCAGTGATATGGTTAT
11	CBL	AGGTGACATGTATTTTGCTCTGTTCA	CCTCTTCACCCTCACATTGCTC
11	CBL	ACCTCTTCCTGTGCCAAAACTG	AGGGTGTCAATTACCCACATAAAGG
11	CBL	GCTTTAGGAGAGTTGAAAGATGCCATT	CTTGAGGGAACACATACTCGCT
11	CBL	CCACCACGACTTGACCTTCTG	AGCAGGGTGAAAGCAAATCAGT
11	CBL	CCTTCACGCCCTGCTTCTC	CGTCCTTCATGAGCCCAATCA
11	CBL	CAACGTGAAGAAGAGCTCTGG	AGCACTTCTCCACCATCTTCTTG
11	CBL	CTCATGAAGGACGCCTTCCA	GCTCAGCCGGCCTTTCA
11	CCND1	CGTGCCTCCGTAGGTCTG	CTTCATCTTAGAGGCCACGAACAT
11	CCND1	CTGGAGCCCGTGAAAAAGAG	CCGGATGGAGTTGTCGGT
11	CCND1	GCCGAGAAGCTGTGCATCTAC	TCGGAGGAGCAGATATGTCAGA
11	CCND1	GCCGCTCACCCTGTGTTC	CCTCCGCCTCTGGCATTTT
11	CCND1	CACGATTTCATTGAACACTTCCTCT	CCGGCCAGGGTCACCTA
11	CCND1	CCTCCTTCCCTCTCTCCTTCTG	ACACTTGATCACTCTGGAGAGGAA
11	CCND1	CCCAACAACTTCCTGTCCTACTAC	TTTCCGTGGCACTAGGTGTC
11	CCND1	CTCTTATAAAGGCTTCCGGGTCAT	GCAGGCTTGACTCCAGCAG
11	CCND1	CTGCCAGGAGCAGATCGAA	GCCCTCAGATGTCCACGTC
11	CCND1	tggaCCTGGCTTGCACAC	GAATGAAGCTTTCCCTTCTGGTATCA
11	CCND1	GGCTGTCGGCGCAGTAG	CACAGGAGCTGGTGTTCCA
11	CCND1	AGCCATGGAACACCAGCTC	GGACCTCCTTCTGCACACA
11	CCND1	CCCTCGGTGTCCTACTTCAAA	CAAGTTGCAGGGAAGTCTTAAGAGA
11	CHEK1	CTTGCATAGAAGACTTGAAAGCATTTGT	TCAGTCTTACCTAGAAGCACTGTTTACT
11	CHEK1	CTCACCCATGTGGCTTAACCTT	AAAAATACCTTAGAAAGCCGGAAGTCA
11	CHEK1	GATAAAATATTGGTTGACTTCCGGCTTT	GCAAGTAACCTATTTACAAATGCCACAT
11	CHEK1	ACTGTAAGCATGAGAACTTGTGTGT	CTGGCTGAGAACTGGAGTACTTC
11	CHEK1	CTTTCTGTCTTAATGCCTCAGTGAAGA	CAAAAGCATATGATCAGGACATGTGG
11	CHEK1	GCCCCTCATACATTGATAAATTGGTACA	AAAAATTAAATTCCCTCACCTGTGAGGA
11	CHEK1	AAGTTCTACAAACCTTAAGTAACACCAG	CGAAATACTGTTGCCAAGCCAAA
11	CHEK1	AATCTCAGACTTTGGCTTGGCA	ACTGGTTCTGCATGAAATTCTCTTCTC
11	CHEK1	TTGAACAAGATGTGTGGTACTTTACCAT	CTCTTACCTCCAGCGAGCATT

11	CHEK1	GCTTCTGTTTGCCTAAGCACATT	GCCATGAGTTGATGGAAGAATCTCT
11	CHEK1	CATGCCTGAACCAGATGCTC	GCAGAGAGAAACACACTAGAATTTGGTA
11	CHEK1	GGGTGGTAGGTATAGTTGTCTATTTCC	ACCCCTTTCATCCAACAGAAGATTTT
11	CHEK1	CATGGTATTGGAATAACTCACAGGGA	AGACTTGATTTTGCCTTGTATGGCtat
11	CHEK1	TTTCCAAGTTGGGATTCTTAGTGTGA	GTCACTGGGTTGGTCCCAT
11	CHEK1	ACTGGGACTTGCTTTGTTTTTAGAATTG	CCAAGAAATCGGTACTCTTTCACTCAAG
11	CHEK1	CCACTTCCTTGGTTTCTCCTTTGT	GCGACTGCTTCTTCAGTTACTCTAT
11	CHEK1	TCCTTTTTAGAGTTCAACTTGCTGTGA	CCTTCTCTCCTGTGACCATAGAATTTTA
11	CHEK1	GCCGTAGACTGTCCAGAAAATATTAAGA	CTATTCTGTCAAAAAGCTCTCCTCCA
11	CHEK1	TTCTGGAGTACTGTAGTGGAGGAG	CATGAGCAATTTTGAAAGGACAACGA
11	CHEK1	GAAAATGTGTTTCTTACCTCAAGCCAT	GGTAATTCTTGCTGATGGATTCTCAACT
11	CHEK1	GTAATGTTTGTTTTTAGCTCTGCTGCAT	CTTTGCAATTACCTTTTCTTCCACAGA
11	CHEK1	AGACCGAAAAGAAAATGGTAGCTAGAC	TCAGGAAGTGTCTCTTGAACTCCA
11	CHEK1	GGGTGATGGATTGGAGTTCAAGA	ACTATATTCACCAGGATTCCCCAGAG
11	CHEK1	AGGGAGGCCTTCATGCAAAAT	GCATCCAATTTGGTAAAGAATCGTGTC
11	CHEK1	GCAGCGGTTGGTCAAAAGAAT	CACACCTGATTCATACAACTTTTCTTCC
11	CHEK1	CTCAGTGGCTTCACTGGTGAT	ACGAAGGCAAGCTTAAAAGAACTCA
11	DDB2	AGAGATTAACCGTGCCGAATGAAA	CCAGAGGCAGTCTGAGTCAC
11	DDB2	TGGCAGGTCCTAGCAGAAGAT	CCCTTGAGGACTTGAGGCAAAAA
11	DDB2	GAAGCAGGGATTCAACCTACCT	TCAGAGACATTCTCCTCCTACTCAC
11	DDB2	CGTGATCCTGCTGAACATGGA	CACATGCGTCACTTTCTTTTTGTG
11	DDB2	CCACCTGAACCGAGCTCTT	GCGCAGGTCCCAAATTTTCAC
11	DDB2	CACAGCCTCCGTAGATCAAACA	GGAGAGATGAGGTCTGGGATATCA
11	DDB2	TTCATATTTGTTTTTGATGTCCCCCTTG	ACGTCGATCGTCCTCAATTCATAAG
11	DDB2	GAGAGTACCCCTGCAGGAGA	TCCCACTGGGAAGCAGAGTA
11	DDB2	CAGAAGAGCGAGATCCGAGTT	CCTCATGATCACAGCCTCCTTC
11	DDB2	CCAGATCCTAATTTCAAAAGTTGTACCC	TCCCTGCTTCCTCCCATCATTAT
11	DDB2	TGTAGTCCCCGCCTTGTTTC	GGGACTCCTGCTCCTCTTGT
11	DDB2	GAGATTGTATTACGCCCCAGGA	CCTAAAAGGCGGAAATATTCAAGCA
11	DDB2	CCCATCATCACTCACTGGCTTT	AGCCTAGTTGTTCCCTCCATTG
11	DDB2	CCAACCAGTTTTACGCCTCCT	TGCTCTTGCCGGAACTACAC
11	DDB2	CCACGGCAAGACAGTTattca	AGACACATCCAGGCTACAAAACC
11	DDB2	cagtCTCTCCCTCCAGCATCT	GAGAACGCACCTCTTTGCC
11	DDB2	CTTTCCTTTGGTGGGAGGACT	GCAGCCTTTTGTAATATCCGGTAAGA

11 DDB2	AGTCCTTTTTGCACACTCTGGAT	GCATGATATCTCCCCCTTTGGAA
11 DDB2	TGACAGGAGGGCTACATCCTT	ccAACAAACACTTCTTTAGCACGA
11 DDB2	CCCAGGCTCTGAGAGATTGGT	GGCCCACACCTTCTTTAGTGT
11 ETS1	ACAAAGTCAGGTAATTCTTGTAAACCCA	ACCTTGCAGAATGACTACTTTGCT
11 ETS1	GGGTGACGACTTCTTGTTTGAT	CCCAGCTTCATCACAGAGTCCTA
11 ETS1	TGATGGGATGGAGCGTCTGA	CTCCTCTCTTTACTTGCAAACCCTT
11 ETS1	ACATAACCTCCACCTCTCTCTGT	CTGAAAGGTGTAGACTTCCAGAAGTT
11 ETS1	AGGGCTGCTCCATTCATACAG	TGGATCTTCATCTGATCTCTTTCCTCA
11 ETS1	CCGCTCCTGAAGAAATGCAC	GCCGACTCTCACCATCATCAA
11 ETS1	GGGAAAAGCTCCAGATCGACTTT	GTCCCCTTCCCCCTGTTACTA
11 ETS1	GGGTCTTTTCCTAGTTTGCTATTATCA	TCTTTGTTCCTTCCCTATCTGTTCATTAC
11 ETS1	GTGTCTTCCCAAAGGGTCTGA	GCAGATGTCCCACTATTAACTCCAAG
11 ETS1	AATGCTTGAGACATCATTTCTTTGCTG	CTTGCCTTCTTACAGCCCATTTG
11 ETS1	CCACAGAAACGTGCAGATGGA	GGGCACCTTCAAGGACTATGTG
11 ETS1	CCTTATTGAGGTCAGCACGGT	GCAGCCAGTCATCTTTCAACAG
11 ETS1	TGTCATAGGAGGGAACACGCT	GTCCTCTTCCTACCTCCTCCTTTA
11 ETS1	ACCCCTTCCAGGAGTTTTCTCT	CAGTCTTTTATCAGCTGGACAGGA
11 ETS1	CAGAAAGTTTGAATTCCCAGCCATC	GGTGTAATGATTCACAATGGCTTGT
11 ETS1	ATCACATTTCCATTGTCCTTCAACTCTA	CTCGAATGAACTGTGGTTTCCAG
11 ETS1	AAGGTCTTTGCTGGTGATAATTGGA	GGCTAAACAACAGAGATTTTCCTCCTAA
11 ETS1	CAATTCAAAATTCAGAGTCCAACCAACA	GCTTTGTGTGTGACCTGCAGA
11 ETS1	CCATCACTCGTCGGCATCTG	CCGTGGCCTACGCTACTAT
11 ETS1	TGTCTTGTGGATGATGTTTTTGTCGTA	CTTGTAGATTTTTGCCTACTCCTCCTTT
11 ETS1	GGGAGAAGATTGTGTGGGCAAA	AACGCCTTCTCTCACACAGG
11 EXT2	GCCCCAGGCAGATTCCTTTATA	CAGATCAGGGAGTTCATTTGGTGAT
11 EXT2	AGAGAGAACTGGTCAGCTGCTA	GCCTTCTCAGCAAAGAGACTAGAGAT
11 EXT2	AGTAATTCCTGTTCCTCTCCACAGT	GCTGACATCGTAGCCTTGCC
11 EXT2	CGGCTTTTCTACGTGGACTTACC	GCCCACCACACTAAACCTCATC
11 EXT2	CTTTCTGTGAAGGGCTGTGTGTA	CATGTCACCTCCCCAAGACTT
11 EXT2	AACTAGTTTGTAATCTCTTGCCTCTTTG	AGTCAAGAACTTCAGAGAAAGGCAAAATA
11 EXT2	CGGTTGTCATTGCAGACTCCTA	GGTATACATTACAATGACCTGAGGAGATT
11 EXT2	TCCTTCCTTTCCTCCTGCGA	TCGTCCCACGGCTTCTG
11 EXT2	GGATGTCCTGCGCCTCA	CCCCCGGCCATCTAGTT
11 EXT2	CCAGGAGTTTGCTTTGCATACC	ACATGTTGAACAACAGGTGATTCGTA
11 EXT2	AATTTCTTGACAGGTGGGATCGAG	CCATCATAAGGACAGCCCCAAA
11 EXT2	CATGTTTGGGTTTGCTGACGATA	ACTCGGTCGTAGGTGAGGAC

11 EXT2	CTCAAGGGTTCACCGCCATA	GAGAGAGGCACTAAGGCTTCTTAC
11 EXT2	CCTGTTTGGATAACTCAGCACTGAA	CGTCCACTCAGACTCATACTTCC
11 EXT2	TCTCTGGGACCATGAGATGAATAAGT	TCTCTCAGTTTTGTCACCTTGCC
11 EXT2	TCAAGTGTCATTTGCCATCCTAAATACT	GGGTGATATAGTAGATTCGGTGCTTG
11 EXT2	GCCCTCATCCCAAGAATGAAGAC	GCCTAACAACCGGCACATC
11 EXT2	GGAATGTAGAGAAGCGCAGCAT	ACACCTTGATTTTGTTCTTTGGGTTG
11 EXT2	TTTGATGTCTATCGCTGTGGCTT	GGTTGATGTCATCAGTGTAGTAGTCA
11 EXT2	GCTCATGGCCATCTCAGACAG	CTGGGCTGTATGAGTGTGAGATA
11 EXT2	CCTTGACTAACATACCAGCTGCAA	ACACTGACTCTCCATGTTTGACC
11 EXT2	GAGGACCTAGAAGCCCTCCA	TTTGCGAGAGGTAATGAATGACACT
11 EXT2	TCAGCTAAAGGGAACTGCTATTTTTGAA	TTGGCCACCAGGAAGTTCAT
11 EXT2	GTGGTTCTGGGAAGCGTACT	TGGCTAGCACTGGAATTCACTTAC
11 EXT2	TGTTATCTCTCAACCTCTTGAACATACT	GTCCCGAAGACTGAAGCAAACT
11 EXT2	CACAGGTCAGAGTGCATCAACA	GACCTCCACCAATGACACGTT
11 EXT2	AGGAATTAGCCTAACCTGGAGTTGA	GGCAATGGCTTTAATTGACTGGA
11 EXT2	GTGTCACAAGCATGATTTTATTGTCCT	ACTTTTGGTTGGAGGCTCACTTAC
11 EXT2	TGGATACAAGCTGATTCTCCCATCT	GGTTACTTAACTTGTTTTCAGCAGTCC
11 EXT2	TCCGGGTTCCATTAAAAGTTGTGA	ACACACTGTGTAAAACCTCCTTACC
11 EXT2	TGACGAGCTGCAATTTGGTTATGA	ACTCTACTGGAAGATTTTAGAAACTCTGA
11 FANCF	GGACGTCACAGTGACCGA	CTACCTGCGCCACATCCAT
11 FANCF	CTCCAGAGCCGTGCGAAT	GATCGCTTTTCCGAGCTTCTG
11 FANCF	GACGTAGGTAGTGCTTGAGACC	AATAGCATTGCAGAGAGGCGTAT
11 FANCF	TGAAGGTCATAGTGCAAACGTTGA	CAAGTGCTAGTCCACTGGCT
11 FANCF	CAAAGACTTCCGAATTCCCCAGA	GGAGCGCTTGCCTCAGAA
11 FANCF	CCGCTATCACCTTCAGGAAGTT	GCTATAGAGAGAACCCAAATCTCCAG
11 FANCF	CTGGGTCTTCATCAGAGAGTCCT	ACCTGGTGCAGCAACTCTTT
11 FANCF	GCTCTCTTGGAGTGTCTCCTCAT	CCGGGATTAGCGAACTTCCAG
11 FANCF	GGACACACGAAGGCATATATTTGGT	GCTCTTCGTAGTGGTGCATTTAGG
11 FANCF	GCTGAGACCCAAAACTTGTCTTTT	GCCCCTCCACCTCTGAAAGATA
11 FANCF	GTCTCCAGGGCAGTTAGAACTT	TCTATCTGGGTCTGCTAACAGACT
11 FLI1	CCTTCCTGCAGTCCTTGCTAA	GCCATCCATGTTCTGGAAAAAGG
11 FLI1	CAGCTTGATGGAGATCGACACAT	GGTACTTGGGCGGCACTTAC
11 FLI1	AACCGGGTCAATGTGTGGAAT	GGAGAGAGGCCACGTCTTC
11 FLI1	AACCCTTATGGTTTTCTTATGGTTGGT	TTGCAGGCCAAGGTAAACTCA

11 FLI1	CCAGCTCAAAGCAAGAGTGTCA	GCTCCGTACGCTGAGTCAAAG
11 FLI1	GTGAGCGACGACCAGTCC	GCTTGACGTTGACCCTCACT
11 FLI1	ACAGCAGGAGTGGATCAATCAG	CCCATCTGCTGCAAAAACCTTA
11 FLI1	AATGAGTGAGAAAGCACATCTGTCA	AGAGATTACATATCCTGGGCAGGT
11 FLI1	GGTGTTCTGTTCTCTCCCGTTT	CATTTTGAACTCCCCGTTGGTC
11 FLI1	CAACGCCAGCTGTATCACCT	CCGTGCACTTTGGTCATAATGTTT
11 FLI1	CGGGCCCTCCGTTATTACTATGATAA	TGTAGGAGATGTCAGAAGGGTACTTG
11 FLI1	CGACCGAGTCGTCCATGTA	AGTATTGTGATGCGGCTCCA
11 FLI1	CTGTCACTTCCTCCAGCTTCTT	AGCTAGAAGGCCACTGATGAGTA
11 FLI1	ACTTTTCTGAGAAGCAGGCGAT	AGAAACATGCTTTCCTCCAAAGAACTA
11 FLI1	GGCTCATGATTCAGAGGCTGA	CTCGTCCATATAGCTGTTGTAGTTCAT
11 FLI1	CAGCGTTAGCAAATGCAGCAA	GGCAGCCTGGTTCTCGAATTA
11 FLI1	AGGAGTCCTCTGTCCCTCTTC	GAGGTTGTATTATAGGCCAGCAGT
11 FLI1	CTGTTTATGTTTTGCCTCTCAGGTTC	GCCTGTGTCATGTCTGGTACA
11 GUCY1A2	CTTGTGCTTTTTGGAGGAGTCTTTG	CTGGGATCTGCTATTTCCTGGAG
11 GUCY1A2	CACTATAAAATCAGGTTGACCTGGGTA	AGACGATACAGCAGACTCTCAAGA
11 GUCY1A2	ACTTGATGTTCATAATACTGCAGTGTCC	TGTCTGCCTACGATACTCACAGT
11 GUCY1A2	CCAGCGAGTCCAGGTTGA	GGCTCTGCTGGAATGGCA
11 GUCY1A2	GCTCCGGCTGCCATTCC	GCAGCATGTCTCGAAGGAAGAT
11 GUCY1A2	GCTGAAGGACTCGGACGAA	GCTTAACGTTGTCGCTTGCC
11 GUCY1A2	TTGGAGAATGACAGGATCAAGAAAAAGA	AATTCGAGTCGGGAAGTCACC
11 GUCY1A2	AAGTGGTTGGGCTGACATTGAT	TGAGGATAGGAATTCACTCAGGCT
11 GUCY1A2	CACAATATGGTGAGGGTGGAAGTA	GTGGCACTTTGCAGGACTTTTT
11 GUCY1A2	CCAACAAAGCATCAAAGCCGTTA	ACTAACATTTGCTTCCCATTTACCTCT
11 GUCY1A2	CCATATTCTCCACATCTCTCTTTGGT	ACCACTCCAACAAAGAAGAAATTGAAGAT
11 GUCY1A2	GCAGTACACTGAAGAATTCCTGAGAC	TGCATTCGCAGATGCTTTCAAAA
11 GUCY1A2	GTCCTCCTTAAGCACCCAACT	GGAACTTTCAGAAGAGGTGCTGAC
11 GUCY1A2	CTGAATCGGTCTTCCATCAGGT	CGTTTGCATTTGATTTGTCTGATTGCT
11 GUCY1A2	TGGCTTTGGACCAGTCCTTAC	GCCTACTTGATTCATGTGCTCTGTATTTT
11 GUCY1A2	CAAAAATCCACACTGGTGGTCAA	CAGGCCAGAAAGTTTGATGATGTC
11 GUCY1A2	CAACAATGTCTGAAAAGAGCATGGT	AGGCAACTTTAGAAAGAACTCACCAG
11 GUCY1A2	CCACTGTCTTCTTTTTCTCTTCTTCCA	GCTACATCTCTCAGACATCCCTATCC
11 GUCY1A2	GACATCTCGGGTGGCATCAT	TGATCCATGTTCCAGAATCAAATTCCA
11 GUCY1A2	CACACATGGAGAGCCCAAAAATAAAA	TGTGAACCTACTATGTGAAGAGTACTGA
11 GUCY1A2	ACATCCAGGGACCAGTAATCTTC	GCTTCTGGCTCTGAAAATAAAGACAA

11	GUCY1A2	CCATCTCAAGTCCATAGCACTTACC	TGACACTCACAAAGTGCTCAAGT
11	GUCY1A2	GATACAATCTCGAAGCAGTCCTCAA	CGGACCTCAGAATTAGCATCAAC
11	GUCY1A2	AAGGGAAGGCTCTACAGAAGGT	CAAATGAGAAGCTATGCTCTGATGTTTC
11	GUCY1A2	ACAGCTACAATTGCCTGGGTTT	CTGAAGGTACTCTCATGCTCCAC
11	HRAS	TGTCAAGGGAGAGGGTCAGT	GAAGCTACGGCATCCCCTA
11	HRAS	TCTTGGCCGAGGTCTCGAT	CTCTCGCTTTCCACCTCTCA
11	HRAS	AGCCCTATCCTGGCTGTGT	TGACCATCCAGCTGATCCAGA
11	HRAS	GGTCGTATTCGTCCACAAAATGGT	GGCAGGAGACCCTGTAGGAG
11	HRAS	CATCCAGGACATGCGCAGA	GCCATCAACAACACCAAGTCTTT
11	HRAS	CCTGTACTGGTGGATGTCCTCA	CGGAAGCAGGTGGTCATTGAT
11	HRAS	CGGTATCCAGGATGTCCAACA	GCATGAGAGGTACCAGGGAGAG
11	HRAS	GCCGTCCCGGGAGACTTA	CACTGACCCTCTCCCTTGAC
11	HRAS	CCTGGGAGTCCCCCTCA	CTGATCCCATCCCTCCTTTCC
11	IGF2	GCAGACTTGCGGCAGTTT	AGCTCGAGGCGTTCAGG
11	IGF2	CTTGGGTGGGTAGAGCAATCAG	GATACCCCGTGGGCAAGTT
11	IGF2	CTGCTTCCAGGTGTCATATTGGA	GTGGACATTAGCTCAAGTCACTGA
11	IGF2	CCGAAGCCCTATTTCTCTGTCT	TCGTTGAGGAGTGCTGTTTCC
11	IGF2	GGGTAGCACAGTACGTCTCCA	GTGCTAACACGGCTCTCTCT
11	IGF2	CGTCCTCACCGGTCACTCTA	GTGCTGCATTGCTGCTTACC
11	IGF2	CGCACAGGGTCTCACTGG	CCGCTCACGCCACTTCT
11	IGF2	CGGGAAACACAGCTCAAATCC	TCTATGCAAGTCCAACGCACT
11	IGF2	GGATAATGGTTACCCCGTCCTC	AGTGCTTTCTTACATCGTTCAAACTCT
11	MAML2	GAACACTTACCAGAATACTGAGCAGTT	GGGAATGATGCAACATAATATGCTTTCA
11	MAML2	TGGGTCAACAGGTTTGTTCAGTAG	CAGCAATCACTGTTGAATCAGCAATT
11	MAML2	CTGTAGAGTCTGCTTCTTTCCCATC	GCTTTTCCTTCCCCCTTTCTCTTC
11	MAML2	TCTCTGGGAACTCTGTATTTGGAGTA	tgcccaATCTCTACCAAGCC
11	MAML2	ttgctgTTGGGTGTAGTGTAGG	CCTCAGGATCTCAGTCGAAGTTT
11	MAML2	GCAAACTCTACCCTCACAAAAGGT	gcaccTGCTGAACAGTAGCA
11	MAML2	ttggtgTGTTTGCCAGCTTTC	ACCTGAGCTGTGAAGGACGATAT
11	MAML2	GCTGCAAGGTGCTTTCTCTTTC	GCTCAGTCACCCCGAGAGT
11	MAML2	GCGCTCCACGATAGCACTG	CATCCCAGAGGCAGCAATCAT
11	MAML2	CCCGTCCTCGACTGATTCAAC	TGCAAATGAGACCCATGAACCAA
11	MAML2	GTTATCTTCATGTAGTCCACCTGAACA	CTGGTAATGATGACTGGATGAAAGACA
11	MAML2	TGTTCCCCAAGATTTCATCAAGATTGA	ACCACAGCAGTGACTTAGCTTT
11	MAML2	TGCCCATTTAGTGTTTGGCTCAT	GTACACCTGCAGCCTATACCC
11	MAML2	TTGCTAACAATTGCTTTGGGTTTCTC	CCAATTCCAGCCTCCTGTCTAC
11	MAML2	TCATTTTGTTGGCTGAGGAAGTCA	GCCTAATTTGAATCAGTCAGGAACAGG
11	MAML2	GCCTGTTGCAGTGACTGATTTG	CACCACAGAGAACATCAAACGTAATG
11	MAML2	GGCATTCTTGTCCCGTGAGAA	GGAGATAACATGCTGCTACGAACTAATA

11 MAML2	GTGCAGTTGTGTTGGATGTGAT	GGGCCAAGTAATAATAACAATGTAGCCA
11 MAML2	ACCAACAGATCCAGCTCCAAAAG	TCAGGAATGAATCAATTGACCCAACA
11 MAML2	CCTGGCTCTGAGGGACTGAA	CCTCCTTACCCATGGAGAAAATAGTGA
11 MAML2	GCTGGGTGCGGGTTGTTAATA	GGCCAGTCGAAAGTAATGGCT
11 MAML2	GAGAGATTCTCCCCAACACGAATT	CTCCAGGGTTCCTTGAAAAGAAAAC
11 MAML2	CTGTTGGCAGGAGATAGGTTAACTA	CACATCACTTTTTATGAGAAGCATCATTGG
11 MAML2	GCCCGGTGAGTATTCACTTTTGA	CCAACATATCTGTGCCTCCCAT
11 MAML2	GCCGGCCTTGTACATGTAGTT	CCTCTTCTGCTGGACCATCAC
11 MAML2	GATTTTCTCCTGCCCAAATGGAC	GCCCAGCAGCTCAAACAGATA
11 MAML2	GGGCATGCTGCTGACGATTA	CTCTCCACTTCGTCTCCAATCC
11 MAML2	CATGTTCTCCAGTTCAAGGTCACT	TCTGTCTTGCAGTAAGCACATGG
11 MAML2	ATTCTCTTGGGTCATTTGGCCAT	GCACCCCTGCGAAAGACTAAC
11 MAML2	GAATGTGTATGGGATGGCAGAGT	GCTTTGTGGACAACTCATTTCTTGATAT
11 MEN1	AACCATGGAGGGTTTTGAAGAAGT	CTGGTGAAGAAGGTCTCCGAT
11 MEN1	GGCTGAGGCTGTTCCATATGAC	GTGGCCGACCTGTCTATCATC
11 MEN1	CGGTGAAGCGGGCATAGAG	GGCTTCGTGGAGCATTTTCTG
11 MEN1	AGGGATGACGCGGTTGAC	GCCCAGAAGACGCTGTTCC
11 MEN1	CACCACGTCGTCGATGGA	GGGCGGGTGGAACCTTAG
11 MEN1	TGCCATCCCTAATCCCGTACA	TCATCCCCAACCTGCTGAAG
11 MEN1	CCTTTCACCTGGCTTTGCT	ACCCTACAGAGACCCCACTG
11 MEN1	GGGTACCTAGGAAAGGATCATAATTCA	GGCTCATAACTCTCTCCTTCGG
11 MEN1	ctgcAGAAGCTCCAGCGA	GGTGGGCCATCATGAGACATAAT
11 MEN1	CCTCAGCCACTGTTAGGGTCT	GCAGCCTGAATTATGATCCTTTCCTA
11 MEN1	GGGTGGTTGGAAACTGATGGA	GGGATGAACACATCTACCCCTACA
11 MEN1	TGGTAGCCAGCCAGGTACA	GATCTTCCTGTGGCCCCTTC
11 MEN1	GCAGAACATGGGCTCAGAGTT	GAAAGTGTCCACCCCTAGTGAC
11 MEN1	CGCTTGAGGAAAGACAGAGTGTA	CCAGTGCTCACTTTCCAGAGT
11 MEN1	GCTGCTGTCACCACCTGTAG	CACCTGCTGCGATTCTACGA
11 MEN1	TCCTCCCATTTGCAGATGCC	GGGTGAGTAAGAGACTGATCTGT
11 MEN1	GTGGCTTGGGCTACTACAGTA	TCATGCCTGGGTAGTGTTTGG
11 MEN1	AGCTGTCTGCTCCCCATTG	ACAGAGGACCCTCTTTCATTACCT
11 MEN1	CTCCTTCATGCCCTTCATCTTCTC	GGCCAGGGTGCAGTGTC
11 MEN1	CCCAGGCCCTTGTCCAG	CGGCAGAAGGTGCGCATA
11 MEN1	CCTTCCCGGGCTTCCTC	GAGCAGGGTCCTGGAGTTC
11 MLL	GGAACTAACAGACCAGGAGAACTT	CCGGCATACTCAATCACCATCTC
11 MLL	TTCTGTAAGAGAAACATTGATGCAGGT	AAAAGAACTGTGTGAGTGCAAGTG
11 MLL	gagcattTGTTACTGCAACCACTATC	GTAATGCTTTCCTTCCGCTTTTGT
11 MLL	TGATGCTTTGGCTTCAGCAAGA	GGGTCAACCTGATTTAATCCACTCAC

11 MLL	GAAATGGTTTTCAGAGCACACTGT	TCACCCGAATGAAGAAGGACTTG
11 MLL	AGCATGGTCAAGTCCTTCTTCATT	CAAAGATGTTTATACACACCACAAAGCA
11 MLL	CCAGTGACTTCTACACATTTGTTCTATCT	GGGTGATAAGGAAGAGGTACTGTGA
11 MLL	ACTTCGAAGCATTGGCTCCAG	ACTACTTTGGCACTTGATTCAAGATCA
11 MLL	AATAATGTTTCCTCAGTCTCCACCAC	GTAACCACTGTCCTTTGCAAATTTGAA
11 MLL	TTTAGGGCAAAACACTTCCACCT	GCACTTTGGTCTTCTCTCCCTTT
11 MLL	GGTGTCCAAGAGCTCCTCTTT	AAAGGAGCCGATTTTACTAACATTCAGT
11 MLL	GGAAACGCACAGTCAAAGTGA	CACTGGACCATCTCCTGGATTT
11 MLL	ACAGAACCAATTTCAGCCTCTGA	GGCCATCTGGAAGCATTAGGTT
11 MLL	ATCAGAATCTTCCAGTACAGGACAGA	ACCATAGGATCTTACTCCATAGAGTGG
11 MLL	CCGTGCACGTTCTAACATGTTTTT	CTTAAGTCATCGGCCCCATCC
11 MLL	ACTGGTGGTTTGTCTTGAAAAGATACA	AGGTGGACAAAACTGAGAGCTTAG
11 MLL	GGAACACCTCCACTTACAAAAGAAGATA	TTTTGCCCTCTGTAAGAGTTGCT
11 MLL	GGACCTCATCATGGTAGGTTTTTGT	GCTGTCAGAACTTGCTTCAGAGAAA
11 MLL	GCCCTTGAAAAAGAGCTGCAGA	AAGACTACCTAAGAACAAGCTCTCAGA
11 MLL	AAGGACAGATGCAACCATTGCTA	AACAGGCATGATGAACTGTCGAATAT
11 MLL	AGAAAGGTGAAGACACAGGTCAAAA	GGGCAATTTTAATTGGAGGGTCATAATC
11 MLL	GCTTTCTTGAGGTTATCTTCACTGGA	GCTGACCACAAAGCACAATTTACA
11 MLL	CTCGGCGGTTTATAGAGGATGAG	GCTACTAGATCGAGAGGAGTCTGAAG
11 MLL	TGGCCAAAATGAGTGGACACA	GGAAACTGAGAGGGAATCTCTTTCATAA
11 MLL	GGAGCGATACCCCTGAAGTT	GAGACGAGGAGGTCTGCTG
11 MLL	CTTCTCAGCACTCCTCTCAAATGT	GGAAATGGGCAGTGGAGGATG
11 MLL	AGAAGTTTTGGATCTAGAACGACGAAAA	GTTGGAGGCATAAGCCAAGGT
11 MLL	CCTCCAGTATCTCTGACCACAC	GTGGCTCTGACCTAGAATGCTTT
11 MLL	GACACTGTCATCTTCCCACATACC	GGAAGTTAAACATGTCAAATGCTGACT
11 MLL	TGTTTTAATCTTTTGGCCCCAGGA	CTGCTCCCCACTCAAGACTTAC
11 MLL	AACCGACATTTAGGTGGACTTCTTT	CCAACGTCCTCGGGAGTTAG
11 MLL	TGATAATTTCCGACCCCCTCCA	TGTGCATATCAAACCTTGTTCCAGAA
11 MLL	AGGTCAGTCAGTACTAAAGTAGTCGT	CCTGAGAAATGGCAGAGAAACAAATCT
11 MLL	GGTTGTTATTGTTTTTGGATTGCCTCA	GTGGGCATGTCATCAGGAAAC
11 MLL	CTTGGCCGAAAACGAGCTG	AATCAAAAGTCTGTTCCGACTCCAA
11 MLL	GATCTTTCTCTTGGTGGCCTGAA	GACTTTTTGACACTGAACCATGGAAA
11 MLL	TGTTTTCTTTTAGCAAATGGAACGTGTT	TCAGATTTCTAGCCGATGAGGAGA

11 MLL	GTTCTTCTCCCTTCTTCTGCATGT	ACAATGTGCTTCTGCCCATCAATAT
11 MLL	aaTGACGCTCATAATCTTCTCTAATCGG	CTGCTCTCTTCTCTTTATAGTGCTACTT
11 MLL	CTAACTGCTATTCTCGGGTCATCA	GTTGCTGGCATCCTCAATGG
11 MLL	AGGAGAGGAACTCACTTACGACTATAAG	CCTTCAGCGTTGCTTTGGAT
11 MLL	CCACAGCTGTATAGAAAATTCTGGGTT	ctaACCTTTTACCCACCCACATACC
11 MLL	TTACTAGGAAATCATCTCAGCAGAGAA	GATCCACAGCTCTTACAGCGA
11 MLL	CTTCTTTTCTAGATCTGTACCAAGTGTGT	CTCCGAAAGCTGGCAAAACTG
11 MLL	TTGTTGCAGACAAAATGAACACTTGT	GGGAAGACCTTTTTAACAACACTACCT
11 MLL	CCTACCTGCAGAAGCAAGCTAA	aatCTACTCCCTGAGAAAGAGTTGTACT
11 MLL	GCAAGTCGAGGGCCGTAAAA	CACATTGCATCATCTTACTCTCATAGTCA
11 MLL	CCCTCTCTGTGACAAATGTTATGATGAT	ACTGAAATAAAAAGACTCCAACCTTCTGT
11 MLL	TTACTTGGGAAGTCTCATTTGCTTCT	AGCTGTGTTTTGACTCTCTTTTGATGA
11 MLL	AGAGTTTTTATTTCCTGCCACAGAAAGT	CATGATAATAACAGGAGCCAGAGGTT
11 MLL	AGACCGACCTCCTCATTCACA	GGCTGATCTTCTTAGGTCAAGTAGGT
11 MLL	GCACTCGTTCAGTTTGGCATTAA	GGCATTGGCAGATCCATGCTA
11 MLL	GTATGTCTTATCTCTTAGGAGGGCAAC	AGCATTTGGTCTGCTTTGATTTTGAA
11 MLL	GGACCATGCTGTTTCCTGTTGA	GGCTTGTGGAAACGGAATTTGTA
11 MLL	GTGCCAAGCACTGTCGAAAT	AACTGCTGACAGAAAAGAAGGGAA
11 MLL	CTCACTATAGACAGATGATGTTGTTGTGT	GCAGGATCCTCTCTTGCTGATG
11 MLL	ACTGGATCTCAAGGTATTGATGGGA	GGCCCTGTGTTGCAAAGTACTTA
11 MLL	GTGGTGGACTCTAGTCAGAAACCTA	TGGTGGCCTGTTTGGATTCAG
11 MLL	GGAAAAGAGTGAAGAAGGGAATGTCT	GAGTGGTTTTGGGAACTTCTTTTCTTG
11 MLL	CTCAGCCACCTACTACAGGAC	TGCTGTTTGAGACATCAGTGCTT
11 MLL	CTTTCCTAAGTGACCTTTCTCTCTCC	GTACAAAGAAGCAGGATGCCTTAC
11 MLL	TTGTTCTTATATTCTGTGAATGGCTCCT	TGGAAGCACTGCGTTTGGTA
11 MLL	gaatcaatTATTTTCAGCAGTGGGATGT	TGGGAGCTGGAATGATTTTCTTCAT
11 MLL	CACACTGAGCAGCCTCCTTTA	CGACTCTCCTTTTGGTGGCTTAC
11 MLL	GGGCCAGGGAACCTAGGATAA	GGCACTCCACTATCTTGCATGTATA
11 MLL	GCTCGCAAGCGCTGTGTA	TCAAGTCCCATCTTGATTCAAGACTAAC
11 MLL	CATAATTGTGTGCTGTACTTACTTGCC	GCACTCAGGGTGATAGCTGTTTC
11 MLL	GCAGCTGCTGGAGTGTAATAAGTG	CATAAGTCCAATAGTCACATGGTGGT
11 MLL	GGATGAGTCTATAGAGGAGACGGT	GGCTCGGGAACACATGAAGT
11 MLL	ACATCCTGCACCAGCAACTAT	CTAAAGCTCTCTCACTTCGCCTT

11 MLL	GCCAGTGGACTACTAAAACCCA	GCGCTCGTTCTCCTCTAAACAAAA
11 MLL	TGTTGTGAGCCCTTCCACAAG	ccagctgTAGTTCTATTACCAAGTTTTG
11 MLL	GGTTTGAATTCAGTACTCCCTTGGA	GCCTCGATCGACGTCCTTT
11 MLL	CCCCAGGAACCTCCAGTAAAG	CATCTCAGAGTGAAACACCCACTTA
11 MLL	CTCAGCAGCCTCCTCGT	GCCGCTCTCCCCAAACA
11 MLL	TCATCGTCCTCAGCCTCTTCA	GGGTTCCTCGCCCCCTTA
11 MLL	GCAAGAGATCAGCTGAAGGACA	GAAATAAATTATGGGATGCCAGTCGT T
11 MLL	CAAGTACCCCCTCCGACAAAAAT	CTGCATGGATGGAGTATTCTTTAGTAC A
11 MLL	CAGTGATTTCTTCAGGTGGAGAGG	CTAGTATCACTCTCTGTCCCATCATCA
11 MLL	CACAGGTTCATAACACAACATCTAGA GA	TGTGTGTTGGTCTCGATCATTCC
11 MLL	CAAAAATCTCACAGTTGGATGGTGT	CCAGCATCTTCAGGTCTATCAATCTTT A
11 MLL	CCTGCCTTCAGACATTATGGACTT	CCCATGTCTTTTTCACGATTACTGTC
11 MLL	CACCTCCATTTGAGAGGGCAAA	TCTCCTATCTCTGGAACTAGATCCCAT A
11 MLL	GCCAGATTCCAAAAAGAAATGGTAA AGA	TTGTCCCTGTGTTTTAACTCTGCT
11 MLL	CCAAAGATGGATAACTGCCATTCTGT A	GGAGCTCAGTATTATAGGTGTCCAGT AA
11 MLL	GAATCTTGGTGAAGGATTGGGTCTT	GATCAGATGGTAGCTCTAGAGGCA
11 MLL	CAACAGATCATCCATTATCAACGAAC A	GCTTCAAGTTTCCTTCCTCACCAG
11 MLL	CATGGATGCAGACCACATCTCTA	CGGGCCAGGACTATCAGTAGAA
11 MLL	GGAACCTGGTCAGGTGACAA	GGCCTTTATCACCAATCTTATCAGAA GA
11 MLL	CTCAGCAGAGGAACAGTTTGAGT	TCACTTTCAGAGGAGGCTACAGAT
11 MLL	GCCAACGACCATGTAACAATGTT	CTCATTTTCCATTTTTAGAGGTGTCAG T
11 MLL	CAGAACCAGAAGTATGTGCCCAA	GGGTCACTCCATTTGGAAGAGT
11 MLL	GGAGAAGAGAGTAACCATCACAGAA AA	TTGCTCTACTGAACCACAAGGAG
11 MLL	GCACATCAACAATAAGCCAGGAT	GTCCTGGAACTGACGCACTA
11 MLL	ATGCAGCCACTTTATGTTCTCCA	GAGAAGTTGGCAAGCTTGGATTT
11 MLL	GGCCCCAGGTATCCAACTTTAC	AGAAGATGGAGAGGATGGAGAACC
11 MLL	GCAAACTACCACAACCCCTACAAG	CTGCTGGCTAGCTTTGATTAAAAGAT TG
11 MLL	CCTTCCCTGCAGCTACTCAAA	TTCTTGAGTCCAGAGGATGGAGT
11 MLL	GGCTTTATCCTCAGCTGTGCAA	AACTTTGTGCTTCTTGCCATTCC
11 MLL	GGTACCCCAGATATTGGCTCAATAAG	TCTGTGATGTCCCCAGTTGTG
11 MLL	TCTCACCCTTACCACAGGACT	GCTGATGTTTGGTGGGAAACTAC
11 MLL	AGACCTCAGTACCACAGTAGCC	GGAGGGTGTGAAGTTAATCAATGTCA T
11 MLL	CCGCCAAGTTCAGGAATGTTTC	TGATAGTGTTCGTCTGCTTCCC

11 MLL	GGTTTCAGCTGCCTCTAGACAAAG	AGCCTAGCTAGTATTTGGATCTCTCA
11 MLL	GCCCCTTCTGATGTGGTTTCTAAT	CCGGTTCAAAATACATGATGCTAGATTT
11 MLL	GCATAACAGCCGCTTCACCTT	CTATTAGGAGCGTCTACCGTCTG
11 MLL	CGAACTGTCCCCAACATCATAAAAGAT	CCCTGATGTGAGGTGGCTAGT
11 MLL	TGTTGCAAATGTGAAGGCAAATAGG	TGCTTAGAACTATTGCCATTGGAGAG
11 MLL	GCACTTTGAACATCCTCAGCACT	CAACACCTTTGGTAGTAGCATTTTCTTT
11 MLL	TCAGTACAAAATGGCCAGTGCTA	TCAGAGCCACTTCTAGGTCTCC
11 MLL	CACAGTTAAAACTAGTCCTCGAAAACCT	TGATCTTATCTCCAGATTTGGTCTCTGA
11 MLL	CCAAGGAAGCAGACTAGTGCT	CGCTCTTGTCAGCATCTCGAT
11 MLL	GATCCATCTGTGTTTTCCCCTCTAAATA	ACTCTGAAATGTCCTTTCCATGTGTT
11 MLL	CGATTGTTTTCGCCACTCCA	CCTGAAGAAGATGTTCCAGCAGAAG
11 MLL	CCACCTTCCCTGGAGTAAAAATCAAAAT	TTGAGAGGAGAGAGTTTACCTGCT
11 MLL	TTGGACCTCGGCCCAAC	GCTGGCAACCCTCTTGTCA
11 MLL	CGTTTCAGCAAGCCACAAAGATT	GGGTCTTTATCCTTTCTGTTGATGGAG
11 MLL	ACAAATTGTACGACGGAGAGGAAG	CCTTCGAGGGCTTTGTCTGA
11 MLL	GTCTGTAACCTTGCCTAGTAATCGAA	CCTTCCACTTCTTGTCCTCATGG
11 MLL	CCCGAGGAGCTGTCCAAAG	CAGGATACAAAGCAGAACTACTCTGAAT
11 MLL	CGAGATGCTGACAAGAGCGT	TTCACCAACAACCTTCTCTTTGGA
11 MLL	TCTGAACCAAGATCTCCTTCTCACT	GCACTAGTGGCAAGAGGACTA
11 MLL	AGGCAGACAAGCTTCCAATGA	ACTGTTAGTTTTAATAGGCAAGGTGGAAA
11 MLL	CTTTGTATCCTGTGGGTAGGGTT	TGTATCCCCAAGAGTCACAGAAGTAATA
11 MLL	GTCTCTTCCTCGTTAAGCATTTCTGT	AGGACTACTTGATGAAAATGGCTCTG
11 MLL	CTTCATCACATGATTCTGGGACTGA	GGTTTTCTCCTTCTCCAGGGATG
11 MLL	TGTCTCCTCTGGGCATTTCTTTG	ACCATGCACTTAATTGGCCGTA
11 MLL	AGTGAAACAGAAGGATGCTTTTGAGAT	GAAAACTTCAAATCCATTCTCAGGAACC
11 MLL	AACAGCTACCATGGGTTTTATTTAAGGT	CCCAATAACCAAGGCTAGGTCATA
11 MLL	TTTACATGGACACCTTGGTTTTAGTGT	CATCCTGTTTGCTGACCTCAGTAA
11 MLL	GAAGGACCTGATCCACCAGTTC	ATTCTGATGGCTCACTGTAGAAACATAC
11 MLL	TGCACATCATTGGTATTAAGAAGGGTT	TGGAATAACACCTCCAGCCATTAC
11 MRE11A	GTGATCACAGTTGACGAGCTT	GAGAAGAACCTGGTCCCAGAG
11 MRE11A	AGCACCTGAGCTTATAAAACAAATCTCT	GGAAATGATACGTTTGTAACACTCGATG
11 MRE11A	ACAAACCACACTCACTTCATTTTCCT	AAAATATTAGTTGCAACAGATATTCATCTTGG
11 MRE11A	CCTCAGCACTTGGCTCAAACTT	TGTAATGTCCATTTCAGGATCCATTCC

11	MRE11A	CCATACACAAGTAATCACTCACTCAAGT	AAAACATTACATACCTGCCTCGAGTTAT
11	MRE11A	GGCCGATCACCCATACAATATTTTCTTA	CACCTTTTTCTGTTGATCAGGTGG
11	MRE11A	GCAACTAGCTGGCAGTCTCtat	TCCAGCAAAATCATGTCCCAGAG
11	MRE11A	TCAAAATCAACCCCTTTCGATACTTGA	CAGAAAGGCACACTTGATGAATTATGT
11	MRE11A	AATAAAGATTCCTTCACAAATCCTATAAGAACA	GATCGGGTAGCTAATCCAAAAGACATT
11	MRE11A	TTTCCTTTTGTTCTCTATGCCTGAAAA	GCAAATTGTTGTTTCCTAGCGTTCA
11	MRE11A	AAAGTCAACTTGACTTTTGACTCAATTTG	TCCAGTGTTTAGTATTCATGGCAATCAT
11	MRE11A	CAATAATTACCCCTGTGGGATCGTC	ACAGCTTTTGTCCCGTATGGAAATT
11	MRE11A	GAGCACTCTCCTCACTACTTTTCAA	GGTAATTCTCACCAGCCAGAGAAG
11	MRE11A	CTTACTCGCAGTCGTACAAGAGG	TGCTATTCAGCCAAGTTAAACTCAGTT
11	MRE11A	TTATCCTGTGATCCTAATTGCCCTTA	GCTGTGTCAGCATCTAGAAATATGTCT
11	MRE11A	TCACTTGCAGTCTATACTCACCATCTAT	GAAATAAACCTTAGCTGCTGATAGGCT
11	MRE11A	CATGGCATAGAAAAATGTGTAACTTTGGA	GTAGATGAATCAGATGTGGAAGAAGACA
11	MRE11A	CCAATAAAGAATAAGGTTTGCCCCATT	GGAAGCACAAAGATTGCGCTAT
11	MRE11A	CTGTCTTACCTAAACCATATAGCGCAA	GGACATTTTAAGTTGTGCTGGATTTGTA
11	MRE11A	ACAGACATTGAACGTCCAAAGTGATT	AGGTGATACGATATTCATGCAGATACCT
11	MRE11A	ACAGGCTTCATGAGAATGTAATCAACAT	CAGATAATCCTAAAGTAACCCAAGCCA
11	MRE11A	CAGAAGCTTTGTATGGCTTGGG	CACTTCAGACATGTTGGTTTGCT
11	MRE11A	CACTGTGTGAAGAGGAATTTTATGCATAT	TTGCACATCACCCTGAGGAAATTTA
11	MRE11A	GTAGTGACATTTCGGGAAGGCT	ATCATTCAGTTTTGCAGATAGGACTCA
11	MRE11A	TTCCACTCAACTGCCAAGTGT	GGCTAATGACTCTGATGATAGCATCTC
11	MRE11A	TCTTCCTTTGTTGGTTGCTGCT	TGTACTTTCTCCTTCTTCTCCCTCTT
11	MRE11A	GTTACTTACCTCCTCATCGATTTTGTCT	CAAGAATTTGTGGACAAGGAGGAGAA
11	MRE11A	TGGTATTTCACTAATTCCTCAATGGCA	TCTGCATCCAGTGATTTGTATCTTACAG
11	MRE11A	CTGCCAGTTAGCGGTAACCT	GCTCAGTGGTTACTTCTCTTTCCC
11	MRE11A	AACACTCACTTCTTTACAGCTTCTCC	GGGCCATGAACATGAGTGTAAAAT
11	MRE11A	CTTCTCTGCGGTTTGAAAGTACTG	ACAACTTGCAATGAGTATGCACATTTT
11	MRE11A	CATTTTTCCTGTATCTTGCATGTTTCTCA	AGCAGGGAACAATCACTAATACAACATT
11	MRE11A	ATAAACACAAGAATTTGCAGCAGAATAAT	GGCAACATGTTTGTGATTTACTCTGT
11	NUMA1	AGTGCAAAGATGCCCTTGAGAG	CCTGGACTCGGGTCGTAAGA
11	NUMA1	TGATGTTGATGATCTGCGTGGT	CCAAGGTAGAATCCCTGGAGAGT
11	NUMA1	CAGGGATGGGAGTGAAGTAGAG	CCTCAGCAGGTTTCCTTTCCATAG

11	NUMA1	ACAGGTCTTTCTGTGCATTCCT	TGTTCACATGGGTCTCATTTACTCC
11	NUMA1	CTGAGCACAGTGCTGGAGTA	AGCATGCAGCCAATCCAGATAG
11	NUMA1	GCTGCCGGGTGGTGATG	TCCATACAGCTATGGTCCACTGA
11	NUMA1	GGACATCCTTCCGGCAAGTC	GCAGCTTGCTGATGAGAGAAGT
11	NUMA1	CAGCTCCAGCTCATCCCTATT	CCAATGCATATGGGCCTTCTCC
11	NUMA1	GGGTCCCTCAGGTACCATCA	TGTTTTCTACGTTGTTGTCATTGCAG
11	NUMA1	GTAAGTCCCTGTAGTCCCCTCA	GAGCCACCTCCTCTACTCAGT
11	NUMA1	AGTTGCCATAATCGGGAGAACC	GACTGTACACTGCAACAGATTCAC
11	NUMA1	ACTTTACATAGCCCTGGCTCCT	GTGACTGTCTGGCATCACCAA
11	NUMA1	GGTCAAGACTGTGGCTGTGA	CCAGTCATCTGCAGCAGCTAC
11	NUMA1	TCAGCTCATTGAGGGCATCC	GTTTGTAGAGGGTTGGCAGGATAA
11	NUMA1	GCTGAGGAAGCAGCTTCTTCATA	CAAAGATTGCTGAATACTCTGGTCCT AA
11	NUMA1	CTCATGCACCAAGACTGGTCA	GCCTCATCTCACTCTGTCTCTGTT
11	NUMA1	GATCAGCAGGTGGGATTCCAA	GCAGCGCAATCGAGTGTG
11	NUMA1	GGGATAGCAGGTCTTCAGATGT	CGACTCCAGGCTCATTTGTTCC
11	NUMA1	TTGGCATCATAATGTGTTTTGGCTTT	GAATGAACTGCAAGCCCAGTTG
11	NUMA1	GCTTATAGTGCTCAGCTGCCT	TTTCCTGAGTGGCCTTTCTTCTC
11	NUMA1	CATCCTGCCAGTGCCTCTA	TCAAAGAAGGCCCTGTCCAAG
11	NUMA1	GGTTCCACTGCGAGTGTTG	CATTGGGTCTCCCGGTCT
11	NUMA1	GGGATTCTCAGGAGTGTGTACC	GCCTAACACCTAAGTGATGCTCTTTTT
11	NUMA1	ACAGCAGGCACTCCATAAACAG	CAAATTCCAGGTGGCAACTGA
11	NUMA1	GGCTCACGGCTCTTTAAAGCA	GCTGCCTTAGGCTTTTCTTCACT
11	NUMA1	CTCCTTCTGCTTCAGGCTACT	CACACTCAATGCCACCATCC
11	NUMA1	GCCAGTTCTTGATCCTGTTGCT	GTCTAGCCTGATCACTGACCTG
11	NUMA1	TGAGGTTGGAGATGGAGCTCT	CCACTCTTGCTGCAAACAACAC
11	NUMA1	TCAGTCTCCAGCATCTCTACCC	GGTTCCTGGTGTTCCTCCATTTC
11	NUMA1	CCTTTTCCGTCAGGGCATGAG	CTGACCTTGCCCTGGAGAA
11	NUMA1	CGCATCTCAAGCTCTGCTCTG	CAGATGGGCAATGAGCTGGAA
11	NUMA1	TGCCCCTGGCTCTCCAT	CCCGAGTGGCTGGAAGAG
11	NUMA1	CAGAACTGGCGTCCCTGTT	CACTCTGCAGGAAAAGATGGCT
11	NUMA1	CTCCTCACGGTGGCGTT	GCGTGGCAGGAGAAGTTCTT
11	NUMA1	GGCCACCTCTTTGCTGGT	CCACAGCGAGCTCCAGATAAG
11	NUMA1	GGAGAGGGCCTGCTCTTT	TCTGCTCTGCGGGAGGA
11	NUMA1	CGCTGTTTCTCAGCCTCCT	GTTGAAGCGGCTGGTGATG
11	NUMA1	AGCTTCTGGCTCTTCTCTGACT	GAAAAATAGCCTCATCAGCAGCTTG
11	NUMA1	CCAGCAATCAGCTTTGCCTTAG	ACTAGGCTGTTCCCTTTGATTTACAG
11	NUMA1	CTGGCGATTCAGGATGGACA	CTTCCGCACCAAGGTACAAGA
11	NUMA1	AGTGGAATAGGAGTGAGGTGAGT	AAAACAAAGAGCTGCGAGCTG
11	NUMA1	TCTCTGGAGAGACTGAAGCATAAGA	GGAGGAGAATGGAGACCTTTCCT
11	NUMA1	CTGTTCAGCCTCCTTGGTCTT	AGAAAGCCAAAACACATTATGATGCC
11	NUMA1	GCTCTTGGTTCTGCTGCTTCTT	CTGAGCACTATAAGCTGCAGGTA

11 NUMA1	CACCACCTTAACACCCACCTTAA	CATACTCTCTTCTCACTGCTTCCC
11 NUMA1	GTCGCTTCATCCCCCTTCAG	TGCCTAACTGTCCTCTTCTCCATAG
11 NUMA1	AGGAATGCAGGGAGAGCTGTA	ACAGATAGCCATGATGCAGCAG
11 NUMA1	CTTCTCATTCAGCAGGGCTAGG	CCCCTTGTGCACATAGTTCTGT
11 NUMA1	CGGCCCCAGAGCTTAGAGAATA	TGAGTCTTCTGTCCTCCTCTTCC
11 NUMA1	AGGCACTGAGAGGGACAGTAG	CGCAAGTTCTCATCTGGCCAT
11 NUMA1	ACTCCCAGAGGCTGGTTAACA	CCAAGAGGGAGATTCGCTTCC
11 NUMA1	GGAGGCAACCTTCTGTAGCTCTA	TGCAAATCTTTACACCTTCTTACTAACCT
11 NUMA1	CACTCATCTTCAGCCTTGCTGT	GGACAGTGCTCTGGAGACTCT
11 NUMA1	CTGGACACAGAGGATCGATGT	CTGGAGGTGATGACTGCCAA
11 NUMA1	CCTGGGCCTTCTCCTCTAACT	GCAGAGGTGAGCAAGCTG
11 NUMA1	CTGCTTCTGGCATTGCTGTT	CAAACCGTGAAGCAACTGAAGG
11 NUMA1	TTGACCTTGGCACCCTCATAC	AGTTTCTGGAAGTGGAGTTGGAC
11 NUMA1	CGCTTCTCCTCTTCAAGGCT	GCCCAGGTTGCAGAGCTA
11 NUMA1	GGAGCTCAGCTAGTTCGTTCT	GTTCCAGGAACAGCTGATGACT
11 NUMA1	TCCTTTTCTTTCTTAGCCAGCTGTT	GGAGTTCGCTACCCTGCAAG
11 NUMA1	GCTGCCAACTCTTGGACATACTT	AGAGAAGGCCAGCTATGCAG
11 NUMA1	CTCTGCATAGCTGGCCTTCT	CACCGTGAGGAGCTGGA
11 NUMA1	CCTTCTGCCGCAGAGGAAT	CAGCTAAGCACCTCTGCCA
11 NUMA1	GACCGCAGCTGCAACTC	GCTAATGAAGCCCGGGACA
11 NUMA1	GCCTTCTCACATTCCTCCTTCAA	TGAGCAGCTCGTCAAAGAAGTAG
11 NUMA1	CTGGGCCTGTGTCACTGA	GGAGCGAGAGGCCTCCTTAA
11 NUMA1	GGCTATCCTCATACCGCTCAC	GAGGCTCGATTACAGCAGCTT
11 NUMA1	GCTGCTTGAGAGCCGCAT	CACCAGGTGGAGCAGCTAA
11 NUMA1	AGGACTTCAGTCTCAGCCTGAT	GTTGTATCTCTGAGCTGAAGGCA
11 NUMA1	CTTTCGTTCCCGCTTATGCTG	CAAAGAGTCCTTGAAGGTCACCAA
11 NUMA1	AAGAAAAACAAGTCAGGTGAGGTGA	CTGCCTCCTGTTTGGCTTCTA
11 NUP98	ACAAAGATCACTCCTTGCCTCAAC	GCAGTTGGTGGACTGGCAT
11 NUP98	GGATGAAGGAGTCTGCTTGGAG	TGTTTGACGAAGTCCATAAACCCTTA
11 NUP98	CTCATGGAGCACTCAAGTGACA	GAGGGTTCTGGCTGTGTGATAG
11 NUP98	GGTGAAAGCAGACATCTCGAAGT	CTTCAGAATACCTCTGACAGTGACA
11 NUP98	GTGCAACTCTCTGGCTTAATCACT	TCACAGGTATTATTCTCACTAAGGTTGGT
11 NUP98	TGAATATCTGCAACAAGCAAGTCATCT	AGTGAAGAAACGTCTTTTCATGATGAGT
11 NUP98	TGGGTTAGAAGAAGAAAAGTGAAAAGCA	CCTGCCTTCCAAAGCAGATACTTC
11 NUP98	CTATTTCTTCTCGGTCATCCTGAAGTG	CCTATTCCTCAAACCCCAGAAAGTG
11 NUP98	GAGTCTGGGAGAACAGATTTCTTGA	CTTTTGATTCTGGGAAGCACATTCATT
11 NUP98	GTTGCTGTTGCTGTGTTTATTTCCA	TCCTTTGCAGATTTAGTTTCCTAAGCA
11 NUP98	ACATAAACCCAAACTGAACCACTCTATC	GCACCAGTGTATTACTGCTATGAAAGAA

11 NUP98	ACTGACCTCTAGTGACTTGCTTTCATA	AGAGAGTAGGAATGTAAAACTTGGACCT
11 NUP98	CATGGAGGTAAGTAAGGGTCTGTTT	CTTTGGCCAACAGAATCAGCAG
11 NUP98	GTTTGCTGAAGAGGCTGGTAGT	CAACAACTGGATAATGGAAGGTTTTGT
11 NUP98	GGCAAACAGTGCCAATCCAAA	GGACTATGCCATGGACGAACTG
11 NUP98	CCACCTAAAGCTTATCAGAGTGGAT	GGTGGCACAGGTGGCTT
11 NUP98	CCTGTGCCACCCCCAAA	CGAATAGGCCTAAGTTTGTGGAATGATA
11 NUP98	GCAGATAGGACTGGGTAAGGCT	TGGTGCTGAGTCTGCATCATC
11 NUP98	TGAGTCGGAGGTTCTATCAGGAG	TGATTATTACACCCCACCCTCAGT
11 NUP98	CGTTGAGTCTTATTTCCAGTCTGACC	GCACTTAGAGGCCCTTTGCTTATT
11 NUP98	GGTTCCAGTGCTCAGCCTTA	AATCTTGGGCTAAAGAGACTTTCCTTAC
11 NUP98	CAGGTACACGGAGCTTCTGG	CTCCATGAATGAGGATGGGATTTATTCT
11 NUP98	AAAGTCAAGCTGTGTCACTTCCT	TACAATCCACTCATTCTTTTGTCTTCCT
11 NUP98	GGTTCAACGGCTTCTCACTCA	GCTGAGGGCTCTTAACTACACC
11 NUP98	CTTCACACTGCGCTGAGAGAT	CGTCTCCTGGCTGTGTATGTTG
11 NUP98	GAAGCCAAAGGGAAATGCACAATT	TGGAGGCTGTATTCAGCTACCT
11 NUP98	CTCACTGATCCTTTTGCCTGTG	CCAGCTAAATGAACCCCGTGAAT
11 NUP98	TCTTCTTCGCTCCAGAATTTGAATGT	TGAAGTTGTTGTAAGACTTTGTCTCgta
11 NUP98	gtgcttACTAGTTCCAAAGGCAGT	GGTACCACAACTGGCTTGTTTG
11 NUP98	CTGGAAGTGGCTGGAGAAGAC	AATATGTTTTCCCTCCCTTCAGGAAC
11 NUP98	AAAAGCCTTCTAAAGTGACTGACACTTA	CTGGACAGGCATCTTTGTTTGG
11 NUP98	CCCTCCAATCTTAGGTTGGTTGTTC	GTAACTGACCTCTGGCACAACAT
11 NUP98	CTCCTGAGGAGATGGAGAAGGA	CATCTGCATTTGGTTCTAGCAACAATAC
11 NUP98	TGAATTTCCAAAGAGGCCTCCA	GCCTTTTCATTTGGTCATCTTACGTAGA
11 NUP98	AAATGAGACTATAACTCTCTACTAAGAAGAACT	GGAAGAGCATTAAGAAGTTGGTTTTGAA
11 NUP98	ACGATTAACAGGAGAAAAGAGATTGCT	AGTTGAGCAGAAGTGCACCAT
11 NUP98	ACAAGCACAGGCATTTACCTACT	TCATTCATGACTATGCAGATTGGGTTAA
11 NUP98	TCTGGTAAGTCTCCTGATGCTTCT	CAAAGAAAGCCAGATGAAGACATGAAATT
11 NUP98	GCAAGCTACAGAAGCATACATCTAAACA	TGTGCATATCCGGAGGAAAGAAG
11 NUP98	TGAGCTCCAGAGGTGTCTGATAT	CCTAGCTTCTGTCTTAGGTTCATTTTCT
11 NUP98	CCGCCACTGGTTGTACCAAA	CCAACCCAGTATACCTGAGGTAAC
11 NUP98	AGGCAGAAAGAGTGAGGAGGTTA	GATTAATTGGCTTCTCTGCTTCTTTGT
11 NUP98	CATACGAATCAGGAGAATAACGTGT	GCACAACCAGTGCACCTTCA
11 NUP98	ACATTCTGCACATTCAGTTTCTCTTTCT	CCAGCAGCACATCAATAGTCTAACATAC

11 NUP98	AGAGGAGAGTCTCCAAAAGGTGA	CGAGCTCTGTGTTACAAATGCATTG
11 NUP98	CTTTCAACTTCGGTATCACGGATTC	GGAGCATCCGTCTAAAACTAGTACAA
11 NUP98	GCAAAGGAGCAGTCTTCAACTTCT	CAGGTATTGGGAAAGCATAATGTTTAAGT
11 NUP98	AAGTGTATACTAAGGCCTTGTGAGGTA	CAGCCTAATTCAGGATTGGGAAAC
11 NUP98	CTGCAAAGAAATATACATGCGGGAA	CTTTGGAACCACAAATACCACCTCTAA
11 NUP98	GTCCAGGTAAACAAGCCCAGAT	GCAGACATTGTGCTCAATACTTTGTTTC
11 NUP98	CCAGATGTGCTGCCAAAAGGA	GCCCAATTCCAATGCATCTTATACATT
11 NUP98	GGGCCCTATCCTCCTGATTAGTTT	GAGAACAGCTGAATGGCTCTCA
11 NUP98	ATCGGCAATCTGATGATTTTCTAGTTCA	AGGCCTAGTCCCTCGTGAAA
11 NUP98	CCTTGCCATAGGTGACAGACT	CAGAGCAGCATCTTTAATGAATATCCC
11 NUP98	GACAGACCAAGAGGATGTGGAT	CCTTCATTCCCGCTTCATTTCAG
11 NUP98	AGACCTGATCCTATGCAATGGAGAT	CCATGTTAGAAGAGAGCATGCCT
11 NUP98	CAGACACAGGTTCCTGATCCTC	GCTTTCCTGATCTTTCTGCTGTCTA
11 NUP98	TCAAATTACAGTCAACTGCAGAAACCTA	CCTGGAGGTCTTTTTGGGACAG
11 NUP98	TCCCAGTGCTGGTGTTTGTAG	TGAGATTCGTTACGGAAACTATTACAGG
11 NUP98	ACTCACTTGGGCATGAATGCT	AGAAGGCTCTTACTACACCTACTCATTAT
11 NUP98	CTTTGGCCGGACTCTAGTGG	TGTGATTTTTAAGGCCAGTGCAGTA
11 NUP98	CCAAATTATTAGATGCAGAACTCCAGTGA	GGTACGTCAACAGGAACAGCAAATA
11 NUP98	TGCTTGCAGTTCCAAACAAGG	TGACTTAGTGGCTCTGATGTACTTAACT
11 NUP98	caagaaccCCATCAATCAATCAATAGAAT	AAGGAAACAGGGAGCTCAATTCAA
11 NUP98	CCAGTTTCAGGCCGGTATTCT	GCCAACAGATAAAACATCTCGTTGTT
11 NUP98	CAAGGCGATCTGGGCTCTTTATTA	CTTTCTGGTCTTATATATGAAGCGCTTTT
11 NUP98	TTTCCTGGCTCCGCTTCAAT	CTATCCATCTTTGGTATTTGCTTCCAC
11 NUP98	GCCCTAGAAATGGAGGCTGTTG	CAAGGTAGAATATAGCCATGGTGATGATT
11 RRM1	AATGTCTTCAAGTTGTCTACAATAAAGTTTTT	TGAGACTCAATGATGGCATACCAAAG
11 RRM1	CGCAAAGTTGTAAAAGCTCAGCA	GCTGGTGTACTCCACTATTTCTGT
11 RRM1	TGGTGTGGAATGTCTAGTATTCTCACA	GCTTCTCCTTATTTAGAGTGAACTGGAT
11 RRM1	CAAGACCAGCGGCTAATCCA	GATCCACACATCAGACATTCATCTCTA
11 RRM1	CCATCAAATGCAGCAACCTGTG	CTTACAACTATGCAAACTCCCTTCACT
11 RRM1	CCATGGTGTGCTCTTTGGAGAA	CCCACTATACCTATCTATGCTCAAGAAGT
11 RRM1	CGGCGAAGATTTAAAGAGATTGCCTTA	ACAGACAAACATGTTCATTTCTCACCT
11 RRM1	TGTCTCATTTGGTACAGAATGTTGCT	CATCTGCCAGACCTTGTACCC

11	RRM1	GCCATCGCCCCATTGGAATT	GCTTCCAGAGCACCATAATAAATAGTTTC
11	RRM1	TCTACAGCAAATTTCCAAGAGAGCTT	CCAGGACACTCATTTGGACACA
11	RRM1	GCCCAGTTACTGAATAAGCAGATCTTT	CACAGGCAAAAACAATTCCATCCATATA
11	RRM1	CCTTTCCTTTTTAGGACTGGTCTTTGA	TTGGACCCCTGAAATAAGAGAAACC
11	RRM1	TGGAACCTAACCCTTCCCACT	GCATCCCCTCACCTTCCTTCT
11	RRM1	AAAGCTGTAGTCTTTTCTAAGCAGTCA	TGCATGCAATAATCTGGTTTTTCATC
11	RRM1	GGGCCTATGGCATGAAGAGA	CATGAGTGAGGAGGTACCACAATG
11	RRM1	GGAGTCAGGGCCGAGAGATA	ACCCATTATTTACCTTAAAGCCGAAGT
11	RRM1	CCTGAATTCTGCTATTATCTATGACCGA	GGTCCATCTCAACTCCAAACAACT
11	RRM1	GGTTGAGATGACAAAAAGGGTTGAGA	GGCCACCATGGGAGAGTG
11	RRM1	GAGGCTCACATGGTGGTGTAA	GTCGTATGTGTGTTCTGATGTGACA
11	RRM1	TTTTTGCATTCCAGGTTGCTGTTT	TCAAGTTTCGGACAACGACTTTAGT
11	RRM1	GTGAAATAGCACTTCATGTTGGTCAAT	GCTTCTGGATTCGAGATGTAATTTTGTC
11	RRM1	CGCCAAGAACGAGTCATGTTT	CCAGGGATAAGAGACAAATGGAAAGA
11	RRM1	TGCTTTGGGAGACATGATGTTTCT	GCTCTTCCTTTCCTGTGTTCTTCTTTAA
11	RRM1	GCCTTGGCATTTAGACATCTTTGAATT	TGCTATTACCAACAAGTACCTATCTCTCA
11	RRM1	acctggcCATGATGGTTTTCTTAA	GCTGTTTCAGCAGCCAAAGTATC
11	RRM1	GGGTCACCACAGTGGAACTA	TTTTACTTTTTCACCCCAGACTTACCA
11	RRM1	CTCAACCTACTTTTTCTGACGTCTCAT	TCAATTGCTGCATCAATGTCTTCTTTG
11	RRM1	TGATGAGAGTATCTGTTGGGATCCA	GCGTATTTCCCTACTCAAAACACCTA
11	RRM1	TTTTTGCCTTAGTGTCTTTGTGTTGAG	CAATGCACATTGCTTTAGAGTGTCATAA
11	RRM1	GAAAGATGACAGCATTGAAGGCAT	TTGCTTTTAGACTTGAATCCCCCTTTAA
11	RRM1	acaGTGACTTAAAGTAGCTGCTTTCC	GATCTGAGCAGTGGAAGCTGTA
11	RRM1	GTTTACTTATTGCCCCGATGCC	TCTCCCTGTAAGGCTGGTTGT
11	RRM1	GCTGAGTGCCCTCTGTCATT	TCCAGTACTTTCAATTCATGGAGCATAC
11	RRM1	CATGGCAGCTAGTCATTTGTGG	ttcactTACTTTGCAATCTTCTCCTTGA
11	RRM1	GATACTGAATGGTAAGGTCAGATCTGG	TTGCTTCAGGTCATCAGGAATTTCT
11	RRM1	TGGGTTTTCCTTCTTTAGAGCATACC	TTTGCCATAGTTAGGCTCAGCA
11	RRM1	CAAAGCCAATCTTTGAACATCCACAT	CCAAAATATCTCAAGCTGGCAATTAAGT
11	SDHD	GGGTGGGAATTGTCGCCTAA	GGCTACGCTAAGCACCTCAG
11	SDHD	GGTCATTTAGAAAGTTTGTCAGTCCTGT	CGGTCCTGAAGAAATGCTGAGA
11	SDHD	CAGTGGTCAGACCTGCTCATA	GTAAAGATGGCTAGAGCCCAGAAAG
11	SDHD	TTGGGTTACTGTGTGGCATATGTT	GGATTCAAATAAGCAGCCGGAAG

11	SDHD	AGTGTTTTGCTCCTGGGTCTG	CATAAGACAAGCTCACAGCAAACAAA
11	SDHD	GCAGCCAAGTTATCTGTATAGTCTTCT	CAAAGGTTAAAGCTGAAAGTGCCA
11	WT1	CCTTTTAGATGTCCTCCTTTGCCT	CAGTTCCCCAACCACTCATTCA
11	WT1	CCCATGGGATCCTCATGCT	CGCTGACACTGTGCTTCTCTC
11	WT1	GCGTCTCGTGCCTCCAAGA	AGGCTTTGCTGCTGAGGAC
11	WT1	CGCCTTCCTACCTGCTGTA	CTCAGCTGTCTTCGGTTCTCTC
11	WT1	TGGATGAAGAAGATCAACTGAAGTTTCC	GCCATCACAACATGCATCAGAGA
11	WT1	CCAGCTGGAGTTTGGTCATGTT	CTGACTGGCAATTGTGTCAACAG
11	WT1	ATTCCTTCTCTCAACTGAGTCTAAACC	CCACACCAGGACTCATACAGGTAA
11	WT1	AATGTGAAGAAAAGTTTACGCACTTGTT	GGCTAGACCTTCTCTGTCCATTTAG
11	WT1	GCTGCCAGCAATGAGAAGTGAA	GAGATCCCCTTTTCCAGTATCATTTTCAA
11	WT1	TCCTGGAAATAACCTGGGTCCT	TTACCCAGGCTGCAATAAGAGATATTTT
11	WT1	GCATCTGTAAGTGGGACAGCTT	TTAAAGCCTCCCTTCCTCTTACTCT
11	WT1	AGGGCCAAAGAGTCCATCAGTA	GAATACACACGCACGGTGTC
11	WT1	GCTCACCTGAATGCCTCTGAA	TGAGCCTTTTTCCCTTCTTTGTGT
11	WT1	GCTACGAAGAAGTTTTCTCTAGACGA	GGACGGACGTCTCGAGAGT
11	WT1	CTGATTACCCACGTCAGTCCT	ACTCCCCACCTCTTCTTCTTTTCTA
11	WT1	TCACAGAGAGCTTTGCCCTTT	GCATGACCTGGAATCAGATGAACT
11	WT1	ACCCCTTTAAGGTGGCTCCTA	CCATTGCTTTTGAAGAAACAGTTGTGT
11	WT1	GGTGTCCTAGAGCGGAGAGT	TTTCCTAACGCGCCCTACC
11	WT1	GAATAGCGGGCTGGCTCT	CCTGAGCGCCTTCACTGT
11	WT1	AGTGAACTGGCCGGAAAAGT	CGGCTTACGGGTCGTTGG
11	WT1	GCTCCTGTTTGATGAAGGAGTGA	CTGTGCCCTGCCTGTGA
11	WT1	GCACGTCGGAGCCCATT	GTGCCTACAGCAGCCAGA
11	WT1	CGCCTAACTTGGCCCAGATG	GCGATCCTGGACTTCCTCTTG
12	ADAMTS20	CATATTGGACATGGAAATCTCGGGAA	CATGACTACTGGTCTCCCAATTCA
12	ADAMTS20	CTTCCCCCTTCTAAATGTTCATATGACT	GCTATTTGCATACAGATACCAGCTTGT
12	ADAMTS20	AGTCATGCAGGAGGGAAGAGTA	GCAACAAACTCAGAATGACCTTGAT
12	ADAMTS20	ATGGTGGGAAGGGTGAACATC	GCTGATAAACTTGGAAGGAAAAGAGGAC
12	ADAMTS20	TCCCACACCTAGAAAGTCTTATTCAGA	CCATCGACTTCGGCCTATAGTTT
12	ADAMTS20	GCACCACAGGGCATTCTTGAT	CCCTGGCTAAAGTTAATGACATTTTCTT
12	ADAMTS20	GTGAAATCTCTAGCAACCATTTTGTTGA	ACCGCTTTATTACCAACTGTTCTCATAA
12	ADAMTS20	CAAGAACCATGTCGCCATTGTG	ACGAGAACCATTTTTCTTCTGAACAGA
12	ADAMTS20	AGTTGGTAATAAAGCGGTCTCAAGTTTA	GGAACGGTTCTTGAGACATACAGA
12	ADAMTS20	AACTCAATAAAGATGGCTTACTGATCCC	GCACACAGCCACTTTCCTAGTT

12	ADAMTS20	GCTTAGATAATAGCTTGGCTGCACA	GCATGAACTACCATCAGCCAATTGA
12	ADAMTS20	GAATTTCAGGCATTCTGTGCAAAGAT	GGTACCATATGTGATCCTTTACAAAGCT
12	ADAMTS20	GCAGCTTTGTAAAGGATCACATATGGT	GCCAATTCTGCATTCTTTTCTAAGTAATAA
12	ADAMTS20	TGGCAGTTTTCGAACAAAATAAAGGAATT	CTTCAGAACTTCCTGGATCACGATATG
12	ADAMTS20	CCAAACGCAAGCTCACACTG	AATCATACTGGAATGCATCCATGTGTA
12	ADAMTS20	TGTTGGAATGCATAAGAACATATGCT	GGAACTGAAACTCATGACATCTGTGT
12	ADAMTS20	GTTTACTTACCATACACTGGCCTTGA	GCACAAAGGATCGTTGTAAACTCTATTG
12	ADAMTS20	AGAAATAATTGGTTCCAGCAACCTGA	TGGCAGTAAGTAAATTATGCTGGTTCTT
12	ADAMTS20	ACGATCCTTTGTGCCAACTGTAA	AATGTGAGGTGGCTTCCAAGATAC
12	ADAMTS20	AACCAGCATAATTTACTTACTGCCACT	GATTCATGTCCAAAAGGCACACAA
12	ADAMTS20	AGCACTGCTTCTCTCGAAAGTC	GCACAAATAATGTTGTACTGATGTGCTT
12	ADAMTS20	CCCCACTTAGCCGCTGA	GGGTCCCCAGGGTGGAA
12	ADAMTS20	CCACAGGAAGCTGAACACTGAT	TTTTACCCCTTGTGGAGAGTGG
12	ADAMTS20	GAATACTTACGGGTGACCAATCCC	AGAGGTACTCAAGCCCGCTAT
12	ADAMTS20	TCAAGAGCATCACGACAGCTTAC	GTGAGTAACAGCTATGTAAAAGGGACAT
12	ADAMTS20	AAACCAAGCCAAATTCCATTGCAT	TGAATCTCTGAGTCCATGTGAACTTC
12	ADAMTS20	CTACTTGCCAGGAAGCACATGTAT	GTTACATGTGGTAAAGGAACAAAGCAG
12	ADAMTS20	ACATTCAGCTGACACCATACCTG	ATTGCATCAGTTTAAGGCATTTGTGT
12	ADAMTS20	CACTTTGTTTTGTGGAGTGTGAAACT	GTTTGTCCAGTGACTTATGTTTAAACCA
12	ADAMTS20	ACCTGAGACCATTCTGAATAATGCC	TCCATTCATGAAGGACAGACTGTTC
12	ADAMTS20	CATAACATTTCTTTTGAGCACCTGGTTT	TCTTTTCACAGTGCTCAGTGACTT
12	ADAMTS20	ACTTGTCTCTTCATTATCCCAATTCCAC	TCCAGAAATACCTGGTCTAAGAAAAGGA
12	ADAMTS20	CACCACAGTAGTGGTCATCAACT	CAAGGTGGCATGTTATTGGCAAA
12	ADAMTS20	ACCACATTGGGATGAACATTCACT	TGGTGGTTTGTCTATCAACTTTTAGGATT
12	ADAMTS20	TGACATGTTAACAGACAAGAGTGCTATC	GTAATATGTCAATTTCCCAATGGCCAAA
12	ADAMTS20	AGGGTTCTCCAAGTACATGTCT	CACATTAGAAAGGATGGTGACTATTACCT
12	ADAMTS20	TTCTCCAAGTACATGTCTGCACAA	AAAACCACATTAGAAAGGATGGTGACT
12	ADAMTS20	AGTCACCATCCTTTCTAATGTGGTTTTT	CAAAGTGTCAGATATTCAGAGTGTAGCA
12	ADAMTS20	GTGGCTTGTTTACAATTTCACAGTTG	AAACCACAGTTTTGAATTTTCCTATTCTTGTT
12	ADAMTS20	ATAGCTCAGAAGTATTTCAAACAGGCT	ATTAATTTGTCAGGAACTGGGATGAAGA
12	ADAMTS20	GCCACTTTGCTGTGCTGGATA	CCCTTGCAATTGAGAAGACTTATTTGT

12	ADAMTS20	ATATTTGATAATGTTAAGCACTTAACCAGTAGT	GAATACAGTGGATCAAATAACGCAGTTG
12	ADAMTS20	ACTGATGACTGAGAATTTACCTGCAAAA	AATCAATGTGCAAGGAACAAGAACTG
12	ADAMTS20	CAACTGCGTTATTTGATCCACTGTATTC	ATTATCTGACGCTGAAGGGAATTTTCT
12	ADAMTS20	GCCAGAATGCACAGTCAGTCTT	GTGTGGGTAATTTATACAACCCTGATGT
12	ADAMTS20	ACTCCTCTCTTCCAAAGGGATATTGA	AAACTACTGGTTAAGTGCTTAACATTATCAAAT
12	ADAMTS20	GCATACCATCATCTGCCCTCA	CCTCCTTGCAATTACATTGTGGTAAC
12	ADAMTS20	ACCTTACCTGTGATGAGTCTGCT	GAGAGAAAAGATTCACATCAACGAATGG
12	ADAMTS20	TGTCTGATTTGGTTATCTGTGCACT	GGCTGAATGTGAGTATCGACATGTAAAT
12	ADAMTS20	CTGTTACACCTTCTTTACAGACTCCAT	GGACAACTCTTCATGCAAGACAATAAC
12	ADAMTS20	CCATAATGAGAACTGTTGAAGACACCT	TGTCTTGAAGGCAGCTGGTT
12	ADAMTS20	AAAAAGCTAAACAGAAATTGAACCACCTT	CTGCTTTAAGTTTTCACATGAGTCCTT
12	ADAMTS20	GCTCCAAGGACTCATGTGAAAACTTAA	AGGATCTATTACTAATATCGTAAGCATCTTTTG
12	ADAMTS20	CCCTGGCTACAACATATACCAACATATT	ACTGTGAATGTGACAATGGACACT
12	ADAMTS20	CTGAAAACAGTGTATCCAGCAGCTA	GGCCTCGTAACTGCATCTAATACTAAAA
12	ADAMTS20	ACATACACGTGTTTAAGGCATACA	ACACAGAATGCCATGAAGCTAGTC
12	ADAMTS20	TGATTCTTTTTACCTGTCTGTCACTGG	TCTTCTTTCAGTGCACAACCACAT
12	ADAMTS20	AGTGAAATCAGATTATTGAACAGTATAAGATGC	GTGGTCCAGGGCCTTGT
12	ADAMTS20	TTACTTCTCCCCAATTTCCGTAGTTC	TGTGGTCTGCCAGGATGAAAAT
12	ADAMTS20	TCGCAGTAACTAGCACTTTGTCC	GGAATCCGCAGTTTGTGCATATTTT
12	ADAMTS20	CGGACTGACCTCCAGTTTTAGAA	CATGGGAACCTTACAGTTCTTGTTCA
12	ADAMTS20	GATTCCGCCTCCACATGTTCT	GAGACACACATCATTTCTTTTTGTGCTT
12	ADAMTS20	TCATCAGCCACCAACTCGAAA	CAGGTCAACTCACAGGAGGATTAC
12	ADAMTS20	CATAAGCTGACGACGGCCTT	CCGCCGATGCATCCTTTCT
12	ADAMTS20	CCAAGTGCACCTCGGTGTA	AGAAGTGTTCCCTCAGAGCCA
12	ADAMTS20	CGTTTCTGCCGGCTGAAGT	TGGAAGAAGTTTAGAGTTGGACAATGAG
12	ADAMTS20	CACATGATGTTGAACACTAGAAATGCAA	CACCCGACCTTGTTCTCAGA
12	ADAMTS20	ACACAGTCCTGACTCCAACATC	GCTAATTGTTCAAATCAACTGGACTCCT
12	ADAMTS20	AAGTATCACTCTGAGGGCTAGAAGTT	GATTTATTGTGCAGACATGTACTTGGAG
12	ADAMTS20	ATCATATACCTCATTCCAGCTATTGGC	AAGTGTTTTGCATTGACCAATTCCA
12	ADAMTS20	TGTGAGTTGGAGGTTTCTGTACTTG	TCTTTTCTAGTGCTCAGCTTCTTGTG
12	ADAMTS20	GCAAATTCGATCCATGAGCAGAAAC	TGAAAGAAAGAGTTTAGGACACACATCA

12	ADAMTS20	GCATCAGCTGTAACCATAATTTCAATGT	CAGTTTACCCTTTCATACCTACAGCA
12	ADAMTS20	GCAGATGATGAGTAATAATTGCATGTC	TGCCACTTCCATCATTTGTTACTCAA
12	ADAMTS20	CCTTAGTTCACAGTCTGTATTGCAACT	TCATTTGACTTTGGCCAAGGTCT
12	ADAMTS20	TGTATGCAAGTTATGTTTCTTCGCTGA	CTGGCTCCTTTAAGCTATTAGCAGAATT
12	ADAMTS20	CCACATACTGGCTGTTGTTTACGA	GCCATCTGCTTTTTGTGAAATTTAACAT
12	ADAMTS20	ACTTATGTTTCTTGCAATATTCTGGTTAAAAA	GGAGATTGCTACAGTGCTTTCAGA
12	ADAMTS20	ACAAATATGAAATACCTGTGGGCATCT	TGCATTATTGTCTCCATGCTAAAGAGT
12	ADAMTS20	AAAGTCTTAGATTACAAAGCTTTACTAACACAA	AGTGACGAGAGAGAATTGCAATGAAT
12	ADAMTS20	GTTTAATGTACCTCGCTCCATTCACTA	GTATGAATAACTTTGGCCATCGTCTTG
12	ADAMTS20	CCCAACTGGGACAGGAAAATTCATT	CAGTGTTCCAGGAGTTGTGGAG
12	ADAMTS20	ACCTTTCCCCTCCTCCACAA	AAGTTGCCCCTTTCTTTCTAATTACGTAT
12	ADAMTS20	TCTTCATACAAGACTCACCCTTCAAGA	CTTGCCTGCCTTTTGTTACTCA
12	ADAMTS20	GAGTTCTATTTCACAGTTTACCTGACACA	GAAGGCAGATGGGAATGAATATGAAGAT
12	ADAMTS20	TGTATATAAGATGTGGCTTGTTGTGACC	TTTTCTTCTTGGCGACTGTTAGGAA
12	ADAMTS20	AGAGTTCCATAATCTCTACGTATTGTCACT	AGGTTGCAACAATCTACAAAGATCCA
12	ADAMTS20	AAATTTTAGAGAAGCAAAACATTCAAGGT	CTGTCAGATGCCCTTCATGGAAA
12	ADAMTS20	GCAAAACACTTCACGGTACTTTCTAC	ATATCAAAGGAATTATGTGTGGATTGTTACA
12	ADAMTS20	TTCTTGTTCCTTGCACATTGATTTCTTT	aaaaaGTTAAACAAGTGTTTTCTGTATTGTTGA
12	ADAMTS20	ATGTTACATTTCTCTTTAGATGAACAAATGTCT	AGTTAGTTTGTGGTAGTAGTCATGTCAT
12	ADAMTS20	GGCGAACTTCAGGATCACAGTAATT	aatGTTGTATATTACCATTGGCTAAATTTTTGA
12	ARID2	ATGTTTGCTGTGCTGTTTTTCTGT	GGAAGATGGAATTGCACCAATAGGAA
12	ARID2	AGTGTCATGTCACAAACTCTGCAT	TGACTGGATCCACTGAATGGAGAA
12	ARID2	GTACCACCAGGCAATCCAAAG	ATTCAAAACTGCGGACATATATCCTGT
12	ARID2	CACCTTCACCAGCTGTACAAGT	TTTTACAAGACTGCCACAGACACA
12	ARID2	AATGAGAAAACCTGGACAGAACTTCA	AAAATTTTCATGCAACTATAGATGACAGAAAC
12	ARID2	GATGTAGTTTTGTTGTTTTTCCAAGCCT	CAGTCTTCTCTTTCCATTCTTCTCCAA
12	ARID2	AGCTTTAGGATCCTTTTCCACTGTATTTG	TCTATTGTTGGCTTAGCTGCATGTAA
12	ARID2	GGCGCTTTTAAAACACCGATCT	CCCCGGTTCTGCTCTCA
12	ARID2	TGTACAACATGTGTTCACCAGTGAT	TGGAACTTGGGTGTTCAATGAGTTT
12	ARID2	CTGATGCACTAGCTGCGGTA	ACACTCGTAACCACACTGAAAAAGAA
12	ARID2	GGTCCCGGCTGACAAGT	GAATCCGCCTAAAGTAGTGACTCTG
12	ARID2	GGGAAGGAGCTGGATCTTCAC	GGGTCAAAGGAGACTTTTTGGA

12	ARID2	tTTAAAGCTGTGTTCTGTATTCAAGGGA	TCCAGATAACAGTGAAAGCACCAATTTA
12	ARID2	CCATGGACTTTAATTCGCCAAATGATTA	GACGTGCTTGCTTTCATTTGATAGG
12	ARID2	GGACTTTGCTATTAACGTATGCACTCT	ATTCAGCTTAAAACTTACTGTCGTCAAAC
12	ARID2	acctagccTGTGTGTGACTATTTCTAA	CCATGAGACTTGTTTCTGTCAGAAATG
12	ARID2	CGTTGATGATAATGAAGTTCGTGACCT	TCCATTCTCCTGATGTACCTTCTACAAT
12	ARID2	GGTATTCATTGTGTTAATGGGTTCAATATCC	ACTATTCCTGGAGGTGGAGCAA
12	ARID2	TTCTTTAGCTTCCAGAGCAGTTGTAG	CTTTGCTTTACTCCTCCCAAGGAT
12	ARID2	TGAGTCATACTGGTTCATACATAAGCA	GGAATCCTCCACTCTCTTCACTGTA
12	ARID2	TGTACTTTACAGAACGGTCTTTCCAAAT	CATCTGAATGGGAAGTGGTATAGCC
12	ARID2	CATATTCATGTGGTAGGAGTAAAACGGA	GCAGGTGAAGGAGCAGGAGATA
12	ARID2	GTTCGTGTTGATTCTGTTCCTGATG	AGAAAATGCTACTGGAGACACAAAAGTTA
12	ARID2	CTGTGCCCATTTCGAACTTACAAA	CTGGAGCAATGAGCTGAGTTG
12	ARID2	GCAACTGTGAGTCAGGGAAATG	GGGAGTAGCAGTAGTACATGATGA
12	ARID2	CAAGCATCTCCTGCTGGACAA	GGTGTTTGTGGCTCCTCGT
12	ARID2	TCTACCTCCTTCAAACTCAGGGAA	CATTAACTAGCGGTTTTCTCAAATCACT
12	ARID2	GATGAGAGGTGAGTTTTCACTGAAGTAT	GTAGGAATCCTGATCCACATATTCACAA
12	ARID2	AGAACTCAGAACAAATAGACATGCAAGA	TCACCATTTCCTACATGATTGGAAGTTT
12	ARID2	TTTGCAAAGCAGAAGATAATGGTGTTTT	GTATAGCACCTCGAGTGTTGAGATTAC
12	ARID2	GGAGATGGTTCTCATTTAAGCAAAAACA	CTTTCTAGTTGATCACCTTTGGCAGTA
12	ARID2	TCATCTCACTTTACCTGATGTGCTG	TCCAGTCTTACCTATGCTCTTTTCTACT
12	ARID2	AGTCATGGAGAACCCGTCCT	TGAGCACTGATTAGTCTCACTTTGAATT
12	ARID2	TGGGAGATGTTGCTTGCACAA	ATAAATTGAGGGTTTTGTACAGTACCATATTA
12	ARID2	AGGGACTTTAGATATCACTCAGCAAGA	CCGCATTTGAAGCCTCCTGTA
12	ARID2	GGTGGTTCATCTGTGAGCAGTA	GACAACTACACTTGGGCGTTG
12	ARID2	GAAGTTCGTGACCTCATTTCTGACA	GAGGTGGATGAAATAAAGACTCCCAA
12	ARID2	TGTAGAAGGTACATCAGGAGAATGGAT	GTACGATTAGCTGCCAAGAGCTTAA
12	ARID2	TCTTTCCTTTGAGGAGGGCAATG	AACTAGTTTAAGGTACAGTCACATGCTT
12	ARID2	GTTGGCAGGGTGGTCATAGTT	TGGGATGATTCACAATGGCCTTTT
12	ARID2	ACAAGCTCAACTCCTAGAGCAC	AGATAAGGGTTTCAAGGCATGCTT
12	ARID2	CAACTCTGGAAGAAGCATAGCTCA	CTTCAGTGAAAACTCACCTCTCATCT
12	ARID2	CGACAGCGGTTTTCTTTTATTACC	CTGCAAGTAGGGCATCCTTTGA
12	ARID2	CTCTTCCTCAAAAAGGATAAGCACTGT	CGTTTCATTTTCTTGTGTTTTGCCTAGT

12	ARID2	GTACAACTGATGATTCCACTTGGAAA	CAGTTGCTGCATGGTAGAAAACC
12	ARID2	GTGGTTTCAGACACCCTCACA	GTGTACCTGCAAGTGGGTAATAAAAGA
12	ARID2	CTGTGACCTTGGAGACTGACA	TCATAAAGGCATTTGGCAAGGGT
12	ARID2	AGTGCTAGCCATTAGTAACATGGAAG	CAGTATGTGAAATGTGGCTGACTTTG
12	ARID2	CTTTTCAGCGACAGCGGTTT	CTTATCCTTTTTGAGGAAGAGGAAGCA
12	ARID2	TTCTTAGATTGTTCACGTTCAGACAACT	CACCGCAAGTAATACTGTTTTAAAGCAA
12	ARID2	AGAAGTTGTTCTAACGCTGCCT	GAACTGTTTGGTGTGCAAGGTTT
12	ARID2	TTCCTACTTGGGATTTTCAGTCATTTCAT	CACCTTGTACAGGAAAAGACATTTGTG
12	ARID2	CAAGTTTGAATTCAGATGTGCCTCAG	CATTTGTAGACCGAACGTCAGATGA
12	ARID2	GCTGGTCAGACAGTTCAGCTAA	TGACTGACAGTAGGAGGTGGTC
12	ARID2	CAACCAATCTTCAAATCTGACTGCAA	ACTTCACAAGGAATTGGAGCTTTACT
12	ARID2	GAACATACCAGCATGTACTTCTACAGT	TGAGACAATATTGGATGCAGAAGTTGTT
12	ARID2	AAGTATCCCATTCTCCTGCCCTA	GGGTCACTGATTGTAACAATTCTAACTC
12	ARID2	TCCTCTACCCCTCAATCACAGG	TGCATTCGACTGTTGAGGTTGA
12	ARID2	AATGCTCCAGTGACTGTCATTCAA	CAGGTTGTGGCCCTGTACTATG
12	ARID2	CAGTCGCAGGAATTCCAAATAAAGTAG	GTTGGTGGATGCTGTTGCTTTT
12	ARID2	GCTAAAGTAGCAATAGAAAGTGCTGTTC	AGATGAGACATGGAAAACAGTGCAT
12	ARID2	TCATAGCACCCCCACAGTATGT	CCCTACCCTTGAGGCTTGTG
12	ARID2	CCCCAACAAGTACAGATGCAAGT	GCTTACCACCTGGTGTTGAAGG
12	ARID2	CTCCACAGAGTTCTGTTGTTCAGAAT	GTCCTGGTACCACTGTGTGTAATG
12	ARID2	TTCTGCTTCCGAAACGTGGT	AATAGCTGCTGAGCTGGAGTTT
12	ARID2	TTACTATTGCTGGTGTCCCAAGTC	TGTTGAGCTGGCTGGCTTAC
12	ARID2	CCATCTGTAATTCCACAGCAGTCTC	GGTGATTAACTGTTGACCCTGTGAA
12	ARID2	AGTAACCAAGCCGCAGGTTT	TGAGATCAGTGGACCTGGCAATA
12	ARID2	CAACCCAACAAAGCGTAGTGATT	GAAGATGGAGTTATGTTAGGTTGTCCAG
12	ARID2	ACCCTTATCTGTAAAAGTCTGAGTCCT	CATATTACTCACCTGCGACCACAT
12	ARID2	CATTGCCTCCACATGGCTTTAG	GCTAATTTACTGCAAGTCGAGAGGTAT
12	ARID2	TGTTTCTCGAGCAGAAATGTATTCTGA	AAGAGATGTTGTAAGTGACGTAAATGGA
12	ATF1	TCTATACCATTGCTACCAGTTCTTTTCC	GTGCTGTCTCTGACGTGGT
12	ATF1	GTTGATTATGGAAGATTCCCACAAGAGT	CTCAGTATTTTGGCCAGTCCCT
12	ATF1	CAGATACTTGTGCCCAGCAATC	TGTAGTTCGGATCTGATATGTTTGCAT
12	ATF1	ACCATCTAGCTGCATCAGGAGAT	GTCATCTGTCTTAGTTGTCTGAGAGG
12	ATF1	TGATGACATCTCCTGTGACTCTCA	tgTATACAGTTCCAGTTAACATCTGGTT

12	ATF1	TGACCACGGAAAATTACTGAAGAACA	TTTTATTTTGATTTTCCAGGACTGCAACT
12	ATF1	TCTTTTCCCACCTGCATTGTTCA	GGGCTTCATACAAGAGAGTACTCAC
12	ATF1	GGAGACGGAGAAAATTCTGGAGTT	CTACAGAAAAGTCAGGTTTTGTGTGTTT
12	ATF1	GGGCTTTTTGCCACTCCTTTG	AGTACTGCCTGAATTTGTCATGGTTA
12	ATF1	TGGAGTACAGGGACTTCAGACAT	ACAGTGTTAGGAGACTTTCTGTAGACA
12	CCND2	TCTCCCCTCCCCTTCCAAAA	TCGTCTCGGAGCAGGTTG
12	CCND2	CTGCTGTGCCACGAGGT	CGCATGTAGGGTTGGATGTC
12	CCND2	CCTACTTCAAGTGCGTGCAGAA	GATTGCCCTCTCCCAGGTTTAG
12	CCND2	CTTTTTCTCTTTTGCTGATGCTATGCT	GCCAAGAAACGGTCCAGGTA
12	CCND2	GTCTTCCCTCTGGCCATGAAT	GGTGTAAATGCACAGCTTCTCC
12	CCND2	GCCTCCAAACTCAAAGAGACCA	GCTCTGGCAGTGGTGATAGAA
12	CCND2	CCAGGTCAGCCTGTGGAATTT	TTGCGCAAGATGTGCTCAATG
12	CCND2	CAGCTGTCACTCCTCATGACTT	CCGGCTCAAGCCTCATCTTAC
12	CCND2	CTGTTTCTGAGTCTCTGCAGTCT	CCCACACTTCCAGTTGCGAT
12	CCND2	CCATGTACCCACCGTCGAT	GACATCTGGACTTAGCCAAGAAGG
12	CCND2	CATGTTTTCTCCGTAGGATGCTCTA	CTCCGACTTGGATCCGTCAC
12	CCND2	GCAGTACCGTCAGGACCAAC	ATTATGGACGCGTCTCTCTCTTTC
12	CDK4	CTGGGTTCAGCAGAAAGAGGA	TGCTACCTTATATCCCTTCTCACCTTAG
12	CDK4	GCTACGGGCAATCACTCTCCTA	CGAGATCTGAAGCCAGAGAACAT
12	CDK4	GTTCCACCACTTGTCACCAGA	CATCTGTACCTCCCCTTTGAAACTA
12	CDK4	CCCACAGCCATCTCCAGTA	TCCCCCACAGCCTGATTG
12	CDK4	GATACATCTCGAGGCCAGTCATC	CTATTCTGAGTCCTCTTTCTGCTGAAC
12	CDK4	CTCAGGGTCCCCACTTCTCTA	CATCAGCACAGTTCGTGAGGT
12	CDK4	CCTCCAGTCGCCTCAGTAAAG	TGGCTGAAATTGGTGTCGGT
12	CDK4	GGCCTTGTACACTGTCCCATAG	CTGCAGGCTCATACCATCCTAA
12	CDK4	CCAGAAGGGAAATGGCAGCTTT	GTTTTCTGACCTTTGCCTCTCC
12	CDK4	GGAATTCAAGGTAGTCCAGGGT	CCCATCCATTCTTCCTATTCCCTTTAG
12	CDK4	CAGATGCACTGGAAACTAGGCA	GAGCATGTAGACCAGGACCTAAG
12	CDK4	GGGTGCCTTGTCCAGATATGTC	GGATTGAAAAGTGAGCATTTACTCTGGT
12	DDIT3	TTCAAGGAAATGAGGAAAGGGAACAT	CAAGAGGTCCTGTCTTCAGATGAAA
12	DDIT3	AGGTGAAACATAGGTACCCCCAT	AGAGATGGCAGCTGAGTCATTG
12	DDIT3	CAGTGTCCCGAAGGAGAAAGG	AACTTGTGGCATAGACTGTTTGCTA
12	DDIT3	CCCTGCGTATGTGGGATTGAG	GCTCTGATTGACCGAATGGTGAA
12	DDIT3	CCAGGGAGCTCTGACTGGA	CTATGTTCCCTTTCCTCATTTCCTTGAA
12	DDIT3	CAATTGTTCATGCTTGGTGCAGA	CTGGAAAGCAGCGCATGAAG
12	DDIT3	ACTTTCCTTTCATTCTCCTGTTCTTTCT	CAGAGCCCTCACTCTCCAGAT

12 EP400	GATCTGAATTCACCTTTTCCTCTACGTT	TGTGACAGGGTGAACTTGCTG
12 EP400	CTCCTGATCCTGAAAGTTCTTGCT	CCATGCTGTAGGCATCCTCTC
12 EP400	CGGAGCTCCTGACCTACAC	GGCATTTTCTCATGTCAATGTACACA
12 EP400	CATCCATCACAGGACCACAGAG	TTTTGGCATCAGTGCTCTCTCA
12 EP400	CGTGTTACAGCAGCATTTTTGGAA	ACGTCCTCGTATAAGGCTTTTTGTC
12 EP400	CATGTTTTGAAGTGTCGCCTTTCTA	AATCCTTGCGGAAAGGAGCTT
12 EP400	GTGACTGACTTAGCATCTTACATCTTTTG	TCAGGCCTCTCATTGAATAAGGAGTA
12 EP400	GGTCTCTGCGGCCCTAATTTC	GGTGTGGTAAGAAACTGGGTCTTAA
12 EP400	GCAGCAGAAGGTTGCCTAC	GCACCCACTCCTGACACT
12 EP400	GTGCATCCAGCTCACTGTGAT	ATTAGGTGTGTGAGCAGGTGAC
12 EP400	GCCTGGACATGACAGATACTTTGA	GAGTTCTCTGTGGCTGCCAATATA
12 EP400	TGCCTTTGAACCTCACAATCGT	GGACAAACGAGACCGTGCTTA
12 EP400	GGACTGAGTTTCCTGGGCAAT	TCACCACCTGCTGCTGAATG
12 EP400	CCAGGTGGTTCAGCAGAAACT	GGTGTCTTTAGCCTGACAGCA
12 EP400	GCCCAAGTTACAGATGAGGGT	TGCTGAATCTCTTGCGTGACA
12 EP400	TCATTATGACTGAGTAGATTGCGTGAAG	TCTTCTGAAAGGGATTCATGCCAAG
12 EP400	GCTGTCTCACTGTCTATACTTCAGG	CACTGAACTTAGATGACACTGGAATGAA
12 EP400	CAGAACGTCTGTAATTCCCATCCT	CAAGCGTGGTGAGCCTTTTT
12 EP400	GCTTGCTGTCTGAGAATGAGGAT	gcCTGAGAAGCTGGAAATGTG
12 EP400	CCCTGGAAAAACCATCACACCT	GCCCTGGATCTGTGGAACTTG
12 EP400	agACGACGACGACCTCTCA	AGACGCACACGTTCATGGT
12 EP400	GGATGATGAGGCGCTCTTGAT	GAGGTAATGGAAGTTCAAGAACATCTCT
12 EP400	GGGTGCTGATTTTATCACAGATGATTCT	GGCATTCAACACCAGTGACTGA
12 EP400	CTGAATTTCTCTGGACTCTCCCATT	TGACTCCCGGTGAGGAGAC
12 EP400	GAAGCCTCCGGTGGTGT	CCTGTGAAACCAGGCTCAAG
12 EP400	TCTCTTTTTGTGCACATACTCCTTTGA	TTCCACTTTGTCTATGCGTCCTAC
12 EP400	CGTGGAATCGCATGGTGTTAG	CCAGCATCATCCATGGGAGAATAA
12 EP400	CCATCCAGGAGCTGTTTGAAGT	AACTCTTCAGCCAGATTCAATGTATTCT
12 EP400	CCTTGGGAACGAACTGTGCT	AGCTGCTTCTACTCCTTCTCACA
12 EP400	CCTCAGATTCCCACCCAGTGA	GCATAGATCTGGCTCGTACGGA
12 EP400	GCAGAGTAAAAACAACCGTCCTC	GAACATAAAATGAGCACTCAGGAAACAT
12 EP400	TGAAATCCTGTGAACTTGGCGTT	GGTCTCAAGCTCAGGTGCTTAC
12 EP400	ACAGGTGGCTGTGTGAATGTC	GGCATGCTCGTCCCAGTAAC
12 EP400	GCCTCTTCATGCAGCAAGAC	GCTGCTGAAGTGGAGATTTCCT
12 EP400	AGATACCGCGGAAACTCATGG	AGCCTTTCTTTTCACCACGCATA
12 EP400	GACGAGCTGTCATTCTTTTATTTGTTCA	CCAATATGCCATTGAGATTCTTCCTGT
12 EP400	GGACTGGCTGGCCAAACTTT	TGGCCTATAGGTAAGTCCTAGCAAATA

12	EP400	CTGGTGGTGACAAAACATGCTT	GCAGCTGCTGGAAGTACGG
12	EP400	CACAGAATGAGGATCTTGAGGCA	CCCCCAACTGCCGCATA
12	EP400	AGCTGCCACACTTGGGAATC	GCAAGCTGGCAGCGTTC
12	EP400	CAGAGTCAGCAGCAGTATGACC	TGTCTGGGCCTCTACCACTT
12	EP400	TCCTCTGCGCTGCAGTTT	CAGGTGTGGGAACGTGGA
12	EP400	AGCAGCCAACTCCCCATC	ACACAGGTAGCCAGCAAATCC
12	EP400	CTGTGGGCGCTCAGGTATT	AGGGACGAGGTAGAAGAGGTG
12	EP400	CCTTACAGATGGTAGCATCGACAA	GCCTTATTGGTGGCTGAGGAA
12	EP400	CTCGATCCTCTCCAGTAAATAGACC	GAGTTATTTGTTCTGTCAGCGTATCCT
12	EP400	CCTCGTCCCAAGACAGTTCTC	CGCCATCACATTCTTGACAATTAAAACT
12	EP400	CCCTTCTTACCGTGGTTCTCCT	CACTGAGAACTCGGCAGGAA
12	EP400	ACCTGATGAAGCTGTACGAAGG	TCTTTGTAAGTACTCCGGCTTCTG
12	EP400	CTTCTCAGTGGCCCTCACTTC	GCAGCTTTGAACTGATCCATGAT
12	EP400	TGGTTCTCATCGACTCGCTTTTC	ACTTGAAAGCTTAGCGCCTCA
12	EP400	CACCATCCCCCATGTATCATCT	GCAAAGTCTGTGGCCATCCA
12	EP400	GGACTATCTGCTGGAGGAGATG	CCACCACTTACACCACTGCAA
12	EP400	TTCTTGTAATGGCCTTCAGTGATCAA	CTAGCCGTGCCGACGTA
12	EP400	CCTGGACCAATGGGCATCT	AGAGAACGTGGCGATGGG
12	EP400	GCCTCTGCTGCTCCACA	CGCGAGAAGAAAGTGCCATGA
12	EP400	CTGCTGTCTTGGTTTCCCATTC	AGCCCTGGAGATGACAAAGATAGT
12	EP400	ctaagaGGCCTCGCCTTGAA	GGTTTCTGGAACCGCAGTGT
12	EP400	CTCCGGAGATGGCACAGA	GGGCATAATGTTTCTTCTTGAAAGCTAA
12	EP400	AGTGTCTTTTCAGTGCTGTTAAGTGT	CAGGCCCTGGACCTCTG
12	EP400	CCCAGAACGTCCAGCATCAG	CTCCTATTCATCAGCTGCTGTATTTGAT
12	EP400	CACCCCAGTCTCCCAGTT	CAGTGTGATCTGCTGGTTTCC
12	EP400	ACACTTTCAAGGGAACATGATGGATTT	CTTACCTCGTCATTGATAACCTCAGATT
12	EP400	CGTGAACATCACCCTGCAGA	GGGACGTGACCTGAATGTATG
12	EP400	GATGAGGTCCTCTGTTTGCCTTT	ACAGAAGTGACCGCTCGAAC
12	EP400	CATGGCAACGACTCAGGGT	GCAGACGTGTGGCAGAGAAA
12	EP400	TCAAATGTTGGTTAGTGGATTGTATAAGGAA	ACTGTTTCTCTCCTTTTCTTGCTCAATA
12	EP400	GGGTTGGTGAAGACAGGAACT	CGCTCGTTGACTAAATAAATCTGATCCA
12	EP400	GAGGAAAAGACCAGACTCTTGAAAGA	CACTGTACCCTTCCATGGCTA
12	EP400	GGCAGAGACTTGCTAAGGATTTGT	GCAGAGACTCTCCACACCGA
12	EP400	GTGAGCTGATGTTGACGCTTTG	AGAAGCCGATGGGAGTAAGATGTA
12	EP400	GCGGTGTCAAATTACTGTCCCTTT	GCTCCAGGAAGGTATTGTGCAG
12	EP400	CAACGTCTGCTTCTGATCGACT	CCCTGTGTAACCTTATGACCACTT
12	EP400	TGAAGAGAGCCAGGATTACTACCATA	TCTTTCCAAAAATGCTGCTGTAACAC
12	EP400	CAGCTTCCTGTGAGTTCCACAT	GGATGGGAGTGGCTTCATACA
12	EP400	CGACTCGGTCATGTGTCTCA	TGGCTGACATGCAGGGATTC

12	EP400	CCTAGAAAATCTGCTCCTGCCT	CAAGGATGCTCAGGACGTTGA
12	EP400	CTTGAAGAGCGGGCACTTTG	TCTCCAGTGCCTTCAGGATTAGA
12	EP400	CTGGAGTATCCGTCCGCATC	GCTGGTTGAGAACCCATTTCCAA
12	EP400	CAAGTGGTGTTGCCTCTCAGT	TGAATGTTTAATAAGGTAGCAACAGCCT
12	EP400	AGGTTTCCCAACATTTTCCAAGAATACT	GTCATAAAACACGACGGTGTCC
12	EP400	CCGTACCACAGGTATAAACCTTGTA	GTCTTTGCATCTCCTGATCCTATCC
12	EP400	TGATTTGTGGCTTATAACATGACACTGA	CATGAGATGTGGAACTCACAGGAA
12	EP400	TGGAGTGTGTGGCTGTGATG	CGCCTGAGACACGGTCTG
12	EP400	CCTGGCCCTGTGGTGAT	ACTCGCCTTAGATGGAAGAGAAAGA
12	EP400	AGAACCTCTAACAGCTTTGCACAA	GCAGTTTCGCAGGATGGG
12	EP400	CTCCAGCACAGCCGCTA	GGAGCACCAGCCTCTGA
12	EP400	GCCCATCCTGCGAAACTG	GGAAAGGTGAGGCCACTTACT
12	EP400	CCTCAGAGGCTGGTGCTC	GGACAGCAAGGCTCCTCCTA
12	EP400	TTTTTCCAGCATGACATAGAGTACAACA	GTCTGCTCTGCTCTTCCTTCTTC
12	EP400	CAGGTGTCTGCTTTGGTATTTAATGG	TCTGCAGTTTCTGCTTCTGCAT
12	EP400	AGAAGCAGCTCCGTGAAGAAAG	CAGCTCCGACAGACTGATTCTTA
12	EP400	TGCTTGTATTTTAGGAACACCTCATCAA	CGTGACCGTCGTGAGCTG
12	EP400	CAACAGCCCAAGTGCAAGTG	ATGCTGCTTTTGGAAAACAAACAGA
12	EP400	AGGCAGGAACCATTAAAACATCA	GTTCACGATCACATTTCCACTCAC
12	EP400	CCATTTCTCCTGGCGCTGTT	CCAGGTGAAGCGCAACTCA
12	EP400	GATTACCTTTATGGAGAGTGGGTGA	GGCATCCTCCTCCAGATACTCAA
12	EP400	TTTCCTCAGGCCCTCAAGAGTA	CCCAAACCTGCTCCATGAAGT
12	EP400	CATCCCAATTAGAGGAGCTAGCTG	CAAGTTCCTTATACAATCCACTAACCAA
12	EP400	CTGGTTTACTCTCTTGCTGAAGACT	GAATATTCTTCTTCTGCTCCTTGCC
12	EP400	GCAACACCAGGACTTCTGAAAATTC	AGTCCTCACTGATGAGCCACT
12	EP400	GCTGAGCCTGGTCAAGACAAC	GTCTTCCCTCTTGCCAAAGACT
12	EP400	TTCTGTAGAGGTGAGTCAGTTGGA	GCCTGCTGCTTCAGCTTCA
12	EP400	GCAGGACAGACCGTGGTG	CTTGGCATAGACCCAGAAGTGA
12	EP400	ACTTGTCTGTCTTAAAACTTGCCTGT	CACATTTTCCCACTCAAGCAGAAG
12	EP400	GTCCTCAAGGTGATTTTGAAAGACAG	CATCAATGACCAGGCACTTCCA
12	EP400	CACGTCCTACACTCAGTTCTTCC	ACACTCAATGACAAGCGCGTAA
12	EP400	CTGGGCCCTTCTGCTTG	CGGCTGCTGTGCCTTCT
12	EP400	GCAGAATGATTTGGACATTgaagaaga	accGCAGGCTCAATAAATTATTACT
12	EP400	TCCCGGACTCAAAATCCTCTCA	CCATGAATAAGCATGTCTTCACATTTTT
12	ERBB3	GTAAGGAAGATGCAAACCCAGGA	CCCACGCCAGTAGAGAAAAGTG
12	ERBB3	AGGATTGGTAGTGATTTTCATGATGCT	AAGAGACTTCCAGGACATGCAAAA

12 ERBB3	CCTTTACCTTATTGACTGGTTTCTACTGT	GCCAGTGGTTCACCTATTCTTCTA
12 ERBB3	TTAGAGGCTACAGACTCTGCCT	GCACTAGCTGCCATTAAATGCT
12 ERBB3	ACTTTCCCCTACCCTCATGAAGT	TTCTTCTTCAGTACCCAGGACAGA
12 ERBB3	CCTTTCTTCAGTGGGTCTCAGT	CATCCATGTACTCATAACCCAGCTC
12 ERBB3	CCTAGGGAGAATGACCTTATGCCA	CGGCATACAGAATTTCCTTCACTTAC
12 ERBB3	CCTAGGCCAAGTTCCCTTGAG	CCATCTCGTTGCCGATTCATATATTCAT
12 ERBB3	GGCACAACTCCAGATGAAGACT	GCCCCTGAAAAGCTCTCATCTC
12 ERBB3	CAGCATCTGAGCAAGGGTATGAA	GCTATGCCAGTAATCAGGGTTATCAA
12 ERBB3	GCTCTTGCCTCGATGTCCTA	TGCTCTCGCGCCACTTAC
12 ERBB3	AGAGTCTTTAATGCCTGAAGGAGGA	TGGGCAATGGTAGAGTAGAGAATTCA
12 ERBB3	CTATGTCCTCGTGGCCATGAA	CGTGGCTGGAGTTGGTGTTATAG
12 ERBB3	CTCTAATGGTGTCCTCCTCCTCTT	ACAACTTCTTACCATCTCCTACCCA
12 ERBB3	GTTTGCCATCTTCGTCATGTTGAA	GTGAGCAGTCTTGGGTCATCA
12 ERBB3	CCTGGGTTCTGAAATTGGGATGT	CCAGCTGGAAAGTTAGCTTGTTG
12 ERBB3	CTGTCCACAGCCTCTTGTCTA	TGTTCCTTCCCCTCAGACACTT
12 ERBB3	CATACCCCGTTGATTAAAACAAGCC	GTATGAGTGAAGTTTTTGGAGGGAATTT
12 ERBB3	CTTTCCTCATCATGTAAATTTCCTTGCAT	CTCAGCCTCAGAGCCTGTTAC
12 ERBB3	ATGCCTGGCATCAGAGTCATC	GTAACAGGAGTCAGCAGACTGT
12 ERBB3	GAGATAGCGCCTACCATTCCC	GAAAAATTGAGGAAAGCCCTAGGAAGA
12 ERBB3	CCCCACTGAACCTCTCTTACATTT	GAGGGAAGGCAGGATCTCTAAC
12 ERBB3	CCCTAACAGCCATGCTTTCTC	CCAGGCAATGGAAAGGGTACATA
12 ERBB3	GAGTTGGCTGGAACCAGGATT	TCTGCTTCCAAGTCTAGGTCTAGG
12 ERBB3	GTAGAGCTGGAGCCAGAACTAGA	GGTGCAAAGTGTTGGGTAATTAGAAG
12 ERBB3	CTGAGTAACTCCTTCCCATTTGCT	CCAGAATTCCTATGGGTCACTCAC
12 ERBB3	AGAATAATGAAGAGAGGGCTTGCTG	ACAGACACCTCCTCGGCTATAAT
12 ERBB3	GTCAGTGCTTGTCCTGTCGAA	CACCTGAACAGTTCCATTGCAG
12 ERBB3	TGTGATACCTCTATCTTTAATCCGCAGA	ATGGTAGAGAGGGCATCCAGAT
12 ERBB3	GTGTATGTGAACCTGTTGGTTTCCTA	ACATGATCCAGCAGAGAACCC
12 ERBB3	AGCTTGTCACTCAATATTTGCCTCT	AGAATTCCTCCAGGCTTCTCTCA
12 ERBB3	GGAATTCCAGATCTCAGTGACTGAT	AATCTGCCACCTGAACCTGAC
12 ERBB3	CGTGCTACTCAAGTCACCCA	TCCCTATCCCCATGCTTCACT
12 ERBB3	CCTGTCACTTCTTTCCCTACCTC	GCAATCTTGATCACGGCCACTTA
12 ERBB3	CCCAAGACTGGTACCTCCTTGA	GTTCCTTTCCCTGGAGCAGAT
12 ERBB3	CACGGTAAGTTCTGAAACAAGCTTT	CCAGAATCTCCAACCCCATCATAC
12 ERBB3	TTTTGTGTTAGATCAGGTTCTGCCTT	TCTCTAGCAGGTCTGGTACTTCAG

12 ERBB3	CCTATGCAGGGCTACGATTGG	GTTGGGCTCAGCAGGTAACT
12 ERBB3	GATTAACTACTCAAAGGCCCCCAT	AGCCTCTCAGCAACAGAACTTAC
12 ERBB3	ATGAAGGTCAGGACTTGGAAGTG	CTTGGAGATCCTGGTGCTACTAGTATA
12 ERBB3	ccggccATGGAATGTATTCTCTTT	GCACCTCCTACTCCCTACTCAC
12 ERBB3	GCCCATATGCCTCTCTCCAAC	GCCATTTCCTCCATACCCTTTACTC
12 ERBB3	TGACCTTGGGATCTGATTCTTCCT	GCCGACATTCATTCTGAACATCTG
12 ERBB3	CAAGGGCCCAATCTACAAGTACC	TGCTTCCCTCACTCTAGTTCTGT
12 ERBB3	GGCAGATGGGCTGAGAATTTG	CGTAGAGCTTGTACAGTGTCTGG
12 ERBB3	CGGCGATGCTGAGAACCAATA	GAATAAAGAGGAGCAGGTTGAGGAA
12 ERBB3	GTTCCCTTAGTAGTCTCTCCTCTCAT	CAGAGCTGCCTATTGGCACTTATATAG
12 ERBB3	AAGGAAATTAGTGCTGGGCGTAT	CTAGCAGACCCTTTCCCTCAC
12 HCAR1	GTAGGTGAAGCTGAGGGTTATGTG	GTTCATCATGGTGGTGGCAAT
12 HCAR1	GGCAGGTAGCATGTGATGAACA	CCAGCTGGAGTTCTTTATGCC
12 HCAR1	CCCCAAAAGCCCAGTGTCTAC	GCTTCCACATGAAGACCTGGA
12 HCAR1	CGGCCAAATTGAAAAGGTAAACAGT	ATGTACAACGGGTCGTGCT
12 HCAR1	AGATGGTGTCCCCCTCGAT	GGTGAGTGCTAACGCTCAGA
12 HCAR1	CTTGAAGGAGCAAAATAAGATGATGCC	GGTCATCCTGGGAACAGTGTATCT
12 HCAR1	GCAGAGATGGTTCTCCAGCAAA	TCGTGTTCCTTACGGTGGTG
12 HCAR1	CGACCCCTTAGCACGAGTTA	ATTTCGAACCTCGGTCGCA
12 HCAR1	GGTGGACCACTTTGAAATACCTGT	CCTTTTCGGACAGACTATTACCTCAGA
12 HCAR1	CTATTTGCCACACTGATGCAACTC	GTATTATTTTTCAAGCCCCTCCTTTCC
12 HCAR1	CTGCAGATTTTGAGCTTGTTGTAGAATT	CTAGACTCTATTTCCTCTGGACGGT
12 HNF1A	GGTGCAAGGAGTTTGGTTTGTG	CTGCAGCTGGCTCAGTTTAGA
12 HNF1A	GCAGCCGAGCCATGGTT	GCAGGACTCCCCCTTGTC
12 HNF1A	CTACCTCCTGGCTGGAGAAG	CGTGAAGTCTTCCCCATCGT
12 HNF1A	GGCTGAGCTGCCCAATG	TCTCCACCACGGCTTTCTG
12 HNF1A	TCCTCAAAGAGCTGGAGAACCT	GGGCTCGTTAGGAGCTGA
12 HNF1A	ACCAATGGAGTTTGAAGTGCTGA	GGGTGTAGACACTGTCACTAAGG
12 HNF1A	TGCAGAAGTACCCTCAAGCAG	AATCTCCCTGCCAAGGAAAGATG
12 HNF1A	AATGAGAAAGAATCAAGGGCAAGGT	CCCCTTCTTGGTTGGTAGCTC
12 HNF1A	GATTGAAGAGCCCACAGGTGAT	CCGTTGTACCTATTGCACTCCTC
12 HNF1A	GGAGCGAGAGACGCTAGTG	ACAGCCTTTTACAGGACCTAGAGT
12 HNF1A	CCACGTCTGCCCCTCTCT	CAGCAGGCCTGGACCTTAC
12 HNF1A	CAGCTGATTCCCTCCCCTT	TCTGCACAGGTGGCATGAG
12 HNF1A	CACCCCTCCTACCAGCAG	CACCCCTTTCCCCTGCAT
12 HNF1A	CTGAGCAGATCCCGTCCTTG	CTTCATGGGAGTGCCCTTGTT
12 HNF1A	TCGATACCACTGGCCTCAAC	GGGTAGGGTCATTACTTACGCT
12 HNF1A	CACCTGGTACGTCCGCAA	CCCACTGACTTCCTTTCCATCTA
12 HNF1A	CCCACCAAGCAGGTAAGGTC	AGATGCCGGCGTGTGAA

12 HNF1A	ACTGAGGCCTCCAGTGAGT	GGAGCCAGGGCCTCTCA
12 HNF1A	TTCTCAGAACCCTCCCCTTCAT	CAAACCAGTTGTAGACACGCA
12 HNF1A	GCTATTTCTGCAGGGCGGAAT	GGCCCGCTGTACGTGTC
12 HNF1A	GCACAAGCTGGCCATGGA	CCTTGTCCCCACATACCACTTAC
12 HNF1A	CCGGACACAGCTTGGCTT	CCAGGCCCGATGGTCAT
12 HNF1A	GAACCTCATCATGGCCTCACTT	GCTCCTGGATTCTGCGGTTG
12 HNF1A	GTGGCTAGCAGCCTTGTTTG	CGTGGTTACTGGGAGGAAGAG
12 HNF1A	AGACCTTCATCTCCACCCAGAT	GGCACAGCTGTCCAGGAAG
12 ING4	GGGAGAGAAAAGTGAGTGAAAGG	CTGACTACTTCTTTCTTCCTCTTCCAG
12 ING4	GAAGAGGATGTCATCCGGCAAA	TGGCTTTCTCCCTCTTCCCTA
12 ING4	CTTTCCAACCTGGTGCCAAAC	GTCAAGTGACTATGACAGCTCTTCC
12 ING4	TCCTCACTCTTTTTGCCTTTGCT	GTACCTCTCCTTTCTCCTTCCTTTC
12 ING4	AGAAGCCACAGTTCTCCAGATCATA	GAGAAACTTTCAGCTCATGAGGGA
12 ING4	CATACCCTCTGTTCTTTGGTCTAGG	ACTTTAAAAGGAGCTGTAAGGCCTATG
12 ING4	CCCAAACAGATCCCAACACAGTT	GAAGCCTATGGCAAGTGCAAG
12 ING4	GCACCTTGTCGTCACCAAATTC	CCAGGCCTCTGTCTCTGTTGTA
12 ING4	GGGATGTGGAAGAAACTGTGTT	GCATTCTCCCTTTCACTCACTTTTC
12 ING4	GTGACCTTCTCGTCACTGGAA	GATCGGAAGTTGCTTTGTTTTGCT
12 ING4	ccagccCATCTTTCTTCTATTCTGA	TTGTCACCAGGTCTCCTATGGA
12 ING4	GGTTGTCACAGCCAATCATCTC	GACACTTTCTCTTCCTCTTGGCAT
12 KRAS	AAAGAATGGTCCTGCACCAGTAA	AGGCCTGCTGAAAATGACTGAATATAA
12 KRAS	CCAGCTCCAACTACCACAAGT	AGGTACTGGTGGAGTATTTGATAGTGT
12 KRAS	AGTGGTTGCCACCTTGTTACC	GGGAGATCCGACAATACAGATTGAAAAA
12 KRAS	CCAGGAGTCTTTTCTTCTTTGCTGATT	CCAATGCAACAGACTTTAAAGAAGTTGT
12 KRAS	CAGATCTGTATTTATTTCAGTGTTACTTACCT	GACTCTGAAGATGTACCTATGGTCCTA
12 KRAS	GTCTACTGTTCTAGAAGGCAAATCACAT	GTGGACAGGTTTTGAAAGATATTTGTGT
12 KRAS	GAAAGCCCTCCCCAGTCC	TGCACTGTAATAATCCAGACTGTGTTT
12 KRAS	CACACTTTGTCTTTGACTTCTTTTTCTTCT	CCTGTACACATGAAGCCATCGTATAT
12 KRAS	GTCAGCTTATTATATTCAATTTAAACCCACC	GCAATGAGGGACCAGTACATGA
12 MDM2	GATATGTTTGCTGCAGGGCCTATA	CTCTTGATTCAGCTTACCTCTTTCATAGT
12 MDM2	CTGTTGGTGCACAAAAAGACACTTAT	TGGCCAATTTCTCCACATGGT
12 MDM2	ATTGCATAAGGGTTTGTGTTAGACTGA	CCTTTTGATCACTCCCACCTTCAAG
12 MDM2	TCAGGTACATCTGTGAGTGAGAACA	AACCAGTAAGCTAACTTGTTGTAAATAACCT
12 MDM2	TGTAGAAGTCTGGTTAGATCCAGCTT	TCCATGATGCTCAAAATTAACTTACCCT
12 MDM2	GCACCGACTTGCTTGTAGCTT	GTTACAGCACCATCAGTAGGTACA
12 MDM2	GCAAATGTGCAATACCAACATGTC	GCCATGCTACAATTGAGGTATACGAAAT

12	MDM2	GGCCCGGAGAGTGGAATG	CCAAAAGTGACCGCTCGCT
12	MDM2	TTGTACAAGAGCTTCAGGAAGAGA	AAAGCTGTGTGAATGCGTCAAATAAAT
12	MDM2	GAGTTAGGAAACAGATACAGAGGTCAAG	CTTCTGAGTCGAGAGATTCAACTTCAAA
12	MDM2	ATTGGTTGGATCAGGATTCAGTTTCA	ACTACCTCATCATCTTCATCTGAGAGTTC
12	MDM2	GGGAGAGTGATACAGATTCATTTGAAGA	ATAAAAATCAGTTCATTTCAGAAAGAAGCTAG
12	MDM2	AGATTTGTTTGGCGTGCCAAG	TCTCTGACAAAAGTGAATGAGGGTAGA
12	MDM2	TGTACAAATAGGTACTCAAAACAGCTCAA	ACAGAGCCAGGCTTTCATCAAA
12	MDM2	AGATGAATTATCTGGTGAACGACAAAGA	TGAGAACATTACCGGATTCGATGG
12	MDM2	GCAGTAGCAGTGAATCTACAGGGA	GCACTGTGTTTTCTATCAATCTCGTAAG
12	MDM2	AGGAATAAGCCCTGCCCAGTA	GTGAATTGAGGCATTTTCTCACTTTGAT
12	MDM2	GGGTTACAGAAACTGACTGTGTGT	GGCTTTCTCAGAGATTTCCCCTT
12	MDM2	GGCTTCCTGAAGATAAAGGGAAAGA	CACATGACTCTCTGGAATCATTCACTAT
12	MDM2	GGGCTTTGATGTTCCTGATTGTAAAAA	CTCTTTCACATCTTCTTGGCTGCTATA
12	MDM2	CTCAGCCATCAACTTCTAGTAGCATTATT	GGTCGACCTTGACAAATCACACA
12	MDM2	GCCCCTTAATGCCATTGAACCT	GCACAATCATTTGAATTGGTTGTCTACA
12	MLL2	AAAACCCTTATACACAAAGAGGTACGG	TGGGTTTCTCCTGGCATTTACAG
12	MLL2	TCAGTCAGTCCCCACCACTTA	CAACTCGGAGTGGTTTGAGAAC
12	MLL2	ACAGCGGTGACAGAGAGAGTA	GCTGTCTGACCAATGCCTGTTT
12	MLL2	GACAGTTTCCAGCCTCCAACA	TCCAGATATTGCAGATGAAGAATCCC
12	MLL2	TCACCTGGTGTATCGGCTTTG	CCTGTTCTGGGCATCAGATGTA
12	MLL2	CCTTGTTCGTCTATCCCCCAGA	CTTCTGCAACCTCTGCCTTTCTA
12	MLL2	CCTATTCCCCAGCCTACACCT	TGGACATTTGGGTTTCTCTGTGTT
12	MLL2	CCACCCACTAGAGGACTGCT	AGACCAAGCACTAGGCCCTAA
12	MLL2	CCATGCTTCCCCAACACTCATT	CATGTGGGAGAGGTCTCTGTC
12	MLL2	GCCTGGGACTCCCAGAACTAA	CGTCTGGTGAGTGGACTTTGTG
12	MLL2	TCAGGGAAGAGGTTGTGGGT	TGATTCTGCCCTCTTCTCTTTTCAG
12	MLL2	GCATGAACTCAGATGGAGGGAA	TCTCTGATCCTGTCCGTCTTGTAG
12	MLL2	CTGTACAGCATACTAACTGGTTTGGA	AGAAGATGGTGAATGGCGTCAC
12	MLL2	GCTCCCCCAGCTCTTCAGAT	TCTCCCTATGCCATCTTGCAC
12	MLL2	TTGTGCCAGGACCAGAAATGT	TCTTTCTTGGCACCAACAGGT
12	MLL2	GGAGGCAAGCTTGGTTATGTCA	ATGTTCTGGGCTCTGAACGAG
12	MLL2	GTTACAGCTGTTCCAGAATAACAGAGT	CATCCTTATCTCCCTTGCTTGGA
12	MLL2	GTTCCCCAGGCTCAGACA	CTGAAAAGCCCCCTGAGGAG
12	MLL2	TCAGGTGCAGGGCATTGG	CCCCAACCTGAGGAATCACA
12	MLL2	GACAGGTGTGATTCCTCAGGTT	GCTGAGGGACCACATCTGT
12	MLL2	CAGGTGCAATTCCTCAGGCT	CCTGAGGAGCCACACCTATCT
12	MLL2	TTCCACAACCCAGATGCTGTT	CTGAACTTCCCAGTCTTCCTCA

12 MLL2	GGGTAATCCCAATCCCTCTTCC	ACCCTTTAGCAGCCTCTCTCAT
12 MLL2	GACAGAGGAGACTCTTCAAATGGT	CACCTGAGGAATTGCCCACT
12 MLL2	GTGGAGACATGGGTGACTCTT	CTGTCCCCACCATTTGAAGAATCT
12 MLL2	GGCAGCAGTGGCATCTC	TGTGCATTGCCCCAGACC
12 MLL2	GGGAAGTGGGCAATTCCTCAG	CCTTGTCTCCACCGGAAGA
12 MLL2	GGGATAGAGGCGTCTCAAGT	GGAATCGCCCCTCTCTCC
12 MLL2	CCAGTGGAGAAAAAGGTGATGATTCA	AGGAATTGCCCGCATCCC
12 MLL2	GGTGCAATGCCTCAGGAAGT	CCCCTAAACGAGGAGATGCC
12 MLL2	CCCAGTGGCATAAGACACAAG	CACTTATCTTCTGTGCTTTGGTGGA
12 MLL2	CCTCAGGCACAGCGCATA	TGAGGAGCCGCAACTCTG
12 MLL2	AGCCTGCGGAGATAGGTGT	ACCGGAGGACTCGCTCAT
12 MLL2	GGGAAGCAGGTGAGTCCT	CCCTGCCTGTGGTGTCA
12 MLL2	GGGATAGGCGCGATACCT	CACCTGAGGAGTCTCCCCTT
12 MLL2	ACATAGGCGAGTCCTCAGGT	CGGAGGCATCTCGTCTGTTC
12 MLL2	TGAGCATATGGGTCAGTGTAGGA	CATCTCAGGTAGAGCCCCAGA
12 MLL2	TTGGAGGAGAAGGTGCCAAAG	CTGGGTGGCCTGGAGTTAAA
12 MLL2	GGGCTTTGAAGACATCAGGTG	CCTAGAGGTGAAGAAGGAAGAGCTT
12 MLL2	TGAGTCAACAAAGCCCAGGTTT	GATAACTTGGCTGTGCCTGAGA
12 MLL2	TTCCGGGACTCCCCAAAAG	ACACTACCCCACCTGGCA
12 MLL2	TGTGGAGGGCTGGTGTCT	GCACCGCCCACCTATCC
12 MLL2	CCCCGTAGGACTAGGATAGGG	CCTGGTGAGCTCTTCCTCAAG
12 MLL2	CCCACATCCAGAGTAGCACATA	CATCCCCTGAGAGCATCTTGG
12 MLL2	CTGAGCCCAGATGAGGGAAAC	TGCAGGGCTGTAGCAACAAA
12 MLL2	CTTTAGGGAAGGCTCCCCTACT	CATGCCAAGGTCCCAAGTG
12 MLL2	ACAAATGCACCCGTCCCA	GCCCCCTGAAGTTGCCTTTAA
12 MLL2	CAGCGAAGTGTGGGCTAGAG	CTTTTTGCCCATCACCCGTTA
12 MLL2	GGACCCTTTACAGGTGGGAAGA	GGTTTCCCTGAGGGCCTTAC
12 MLL2	GTGAGGGTGGGCGAGAATAAG	CGACCCTTTCTCCCGAGTG
12 MLL2	TGGACTGGGACTGAGGACTG	CTCCAAGTCACCCAGACATCTTT
12 MLL2	CATCCACCTGGAGAACAGAGAC	GCCCTATCTGTCATGCTCCTTAC
12 MLL2	TTCTCCTAGGTGGGCAGGT	GGAGCTACGGCGCTTTG
12 MLL2	GGGCCAATCAAATGGCAACT	GTGGTACTGATGCTTGTGTGTC
12 MLL2	AGGCTTCGAAGAGCAAGGTT	CGGAGGTCTTCGCCTGAC
12 MLL2	GCACTGGATTAGTAGGTCCTCTTCTAC	GCTCTGTTTTCCTGCTCCTATCA
12 MLL2	GCTGTCCAGGTAGTGCCATA	caCTCAGCTGTGCTGGCT
12 MLL2	GACTCTGGGAAGGGCTGAG	TCTCCGACCCTCCCATGTTA
12 MLL2	CCCATTTTCTCCACGGGAACT	CGATCAGAGCCTAAAATCCTCACA
12 MLL2	CACAAGCTCACCGTTTGTAGTG	CAGGCTAAGGTGAGTATGAACTGTG
12 MLL2	GGTTTTTGGCCAGGACTCCTT	CATGCTAGAGCGGCCCAT
12 MLL2	GGCTCCAGGGCTAGAAAAGT	cagcagATGGGCCTTTTAAACC
12 MLL2	ctgctgAAGCTGCTGTAAAGAG	CTTATGGGACACAGGCTGGT
12 MLL2	GTGACATCAGACTCTGCTGAAGAT	CAGGTGCTTATGACCCAGTCC
12 MLL2	GAGGTTGTGGCCCTGTATTATTTTG	CTAGGCAGTGGATCATCTTCTGA

12 MLL2	TGACCAGCCTGTGTCCCATA	GCAGCACCCTGGAGCTTT
12 MLL2	GCTGATCCCCTAAGGAAACAGA	CTGAGGCTTCCAGGACAACA
12 MLL2	CTGGGTCATAAGCACCTGTCT	AGGGCCTTATGCCTCCCA
12 MLL2	CTGGACCAGGAGGCCTTG	CCTTCTAGGACAGGTGGCAAT
12 MLL2	CTGTGTGGAGCAGGCTAACTT	GAGGTAAAGCCCTCACTCTCTG
12 MLL2	GAGCTTGGTTTGTCTGTACTCCA	CTGATTCAAGGCTTTTACAGGAAAGG
12 MLL2	ggACAAGCAGGAGTTGTGAGT	CGTAGACCCAGCCGTTTCTT
12 MLL2	CTTCTGCAGCTCCTTCTTCTCAT	GGATCCTCCCTGCTGGAAAAG
12 MLL2	GGGCCAGCTGCATACGTT	CAGCAACAGCAACATTCTGGTG
12 MLL2	CGGCCCTCCCTGATGTGTA	GCCAGCAAAGCCTCTTCAAC
12 MLL2	CCCCACTCTCGAGCTCAAA	TTTTCTAGCAGTGGGCACACA
12 MLL2	CCCAAAGGAGGCCTTCTCA	ACCTGCTGCCAATTCCCTT
12 MLL2	ATCATGCTCTGTCCTGGCTTTAG	TCTGTGCTCCCTGAGGTGAA
12 MLL2	TCCACCCTCCTCCACCTT	CCTGAGGCTGGTAGAATCGG
12 MLL2	GCCTCTGTCACATACTCAACATCAT	GCTGCTCTGTGATGACTGTGATATTAG
12 MLL2	GTCCAGGCAGTATGTGTGGTAG	CACCCTGAGAAGTTCCCATCTT
12 MLL2	CTCCCGTTCAGCCTTCTCATTAG	CCAATGACCCCCACTTGGAT
12 MLL2	ACAATGTTCAGTGTGCCAGGT	CTTTCCATAACTCTCTGCCCATCAG
12 MLL2	CTCGTCTCCATTGAGCAGGT	AGGATCCCGAGCTGGATGA
12 MLL2	CCTTTTTCTCCCCCAGACCT	GGCTCTGATCCACTCCTTTCTG
12 MLL2	AGGGCAGAATCAGGGTACACT	GAGAGGACACTGTAATGTTCCATGT
12 MLL2	GGCCTTGTGGGCATCAAAAT	TCTTCATCCAACACCCCACAC
12 MLL2	CCAGTTGGCAGGGTAGGAC	AGAGACCCCGTTTTTACCCTGTA
12 MLL2	CTGTCTCCCTTGCCCTCAT	GGGCGACGGACTCTCCTA
12 MLL2	CCTATGAGGAGGCAGAGTTGTG	GATGTGGAGCCTGGCAAAGA
12 MLL2	GCTGAAGACTCCGCTGGTTA	GCTTCTTTAGCCCGGAACCC
12 MLL2	GAGTCCGTCGCCCTCAC	GCGCCCTGTGACTCTCT
12 MLL2	CGATATGGTTTACGCTTGCGTTTTT	ACAAGGTTAAAAGGGCCTATAGTTCT G
12 MLL2	ACCTTCCCAAGAACTTCACTATTCC	ATTTTAGCCTCTTTGCCCCCTT
12 MLL2	CATTGACTGGGCAGCCACT	GTGCAAGACAGCGATGCAA
12 MLL2	GCGTTTGCATCGCTGTCTTG	TTGGTGAGCTCCCGAAAGAAG
12 MLL2	CTGTTTCAGCTGTTTCAGCAAGG	CCTTCAATGGGCTTATTTGGCTTA
12 MLL2	CGGACCTAACATGGGAGGGT	AGCTGATGCTGAGAAGCTCAAG
12 MLL2	TCTTGCTCTGCTGCTCTGTAAC	ACCCAGCAGCTCCTACAGAT
12 MLL2	TCCAAGGCACATTTGGTCTCTC	ATGTCCACGGCTTTACCACTT
12 MLL2	CCCACTGGTGCCCTCAC	TCCACAACTCTGCCTCCTCATA
12 MLL2	CCTAACCTGTGTTGTGCCTAAGAC	GTGTGACCTGTTCTTCTGTACCA
12 MLL2	GGGTTAGGCCAAAGTTCTCAGT	TGACATGCTGCGACTCTTCC
12 MLL2	TCTGCTCCTCTAGCACCTTCA	GGGAGTGGTTTGGGTCATATGG
12 MLL2	CAGTGTTTTCATGGATAGGAAGGAAC	GGCTGACACTGAGGCTCTTTT
12 MLL2	TCCTCGCCCCTTCAGATACTCA	TGGAGGTCAGTGAGCAGAAGT
12 MLL2	CCGTGATAGTGATGCCCACAG	TGGAGTGCCTGTTTTGTGTACAG
12 MLL2	CAAGGCTGCTCTTGTCCTAGAA	TGCCTTTGAGCAGCTGAGTC
12 MLL2	CCTACCTGTGTCCCAGCAAAG	TGATTCGGCAGCAGATCCAG
12 MLL2	TGCAGCTGTTTCCTTCTCCTG	CCCCCTATATCGCTCCTGTCT

12 MLL2	TGAGGAAGAAGAGAAAGTGATACTGGA	CAGCCACGCCTTCCTCTATG
12 MLL2	CCTCACCGGCTGTTCACATC	CCACCTTCATCTACCTTGTCTCTTCTA
12 MLL2	CATATTATCCATTTCAAGGGCCCACT	CAATCGTCGCTGCTGCTATC
12 MLL2	GCCCGTTGTTCTCACCAATAGAA	GTTCCACGCCATCGGACA
12 MLL2	CTATGAAAGTCAGCCATCTGGTGA	AGCTGAGCTCTTTTGCTGTCTT
12 MLL2	CCTCGTCCCGCTCAATGTA	CATGCGTTGCCCCAATGTC
12 MLL2	CGGATGGCACAAGCAAAATGG	GGTCCACGGAGGTGTATGAG
12 MLL2	CTCAGCCAAGGGTTCGGT	CACTCAGCACAGCTATACCTACA
12 MLL2	CGCACATCCAGATTGGAGACAT	GGAGGGAGCTAGTTAGATGAGGAG
12 MLL2	CCAGGCTCTGGCTGTGAA	CACCAGTCCTCTCCCTTCC
12 MLL2	ACCTCTGACAGTGGGCTAACT	TCACTGCCAGAGACTGATGACT
12 MLL2	GGGTCAGTGCAGTTAGCTTCT	CTCCACTCTCACCCATCATCAC
12 MLL2	GGGTACTCTAACTCCCCCAAA	GGGATGTTAGTACTCTGTTATTCTGGA
12 MLL2	GCAGGAAAGGTCCCCCATT	CCTCCCCGTTGAGTCCCATA
12 MLL2	TGTCCCAGCATCGCACAATA	GCTCCCTGTCCATGGAGTTG
12 MLL2	ATCTGAGACCCCCACTACCTT	GCTTCTCCCATCCTGATGGA
12 MLL2	AACCAGGGAATGCTGAAGGAG	CCATCCTGGAGACACCCATCA
12 MLL2	CTGGACTAACATCCGTAGAGACC	ACTCTCATCAAATCCGACATCGTT
12 MLL2	CCTGTACCCCACCCTTGTTC	CACCCTCACCTGACTTTTCTTCTC
12 MLL2	CCTGGCTCAGATTAGAGATCTCGTT	TGTGACCCCTATGGAGGTCTAC
12 MLL2	GCTGTCTGCTTGCATTCGG	CCCCTCTGGATGGGATTGATG
12 MLL2	GCCCTTCACCTATGCAATCCC	TTCTCTTTACCTCTTCCCACCTGTAA
12 MLL2	CAGCTCCTAAGCCAGAAGAAAGTT	GTAAGGCCCTTTTTGCAGTTGG
12 MLL2	CCTCCATCTCCCTTGGCTTTTG	GCAGGGCATGAAGAAAACTGTTT
12 MLL2	CTGCTCTCCTGTGACCAATCC	AGAAAAGCACACAATGGTTATCGAGT
12 MLL2	CTACCCAGCAGCTGGTACTC	GCTTATTTCCTCCAGTCTCTGTTCTC
12 MLL2	TCCGAATGATGGTGCCAATGT	CCCTACAGCAAGCAGTTTGTG
12 MLL2	CCGGTACTGAGATGACTTGGAGT	TGAGGGAGAGCTGTCTTGTCA
12 MLL2	CCTCTCAGTTCCCACGCTAAT	AGTCTTCTCTGACTCACTCTCTCTG
12 MLL2	GGCAGTCAAGGAGAAACAGTTTT	GAGCAGAGCTTAGCTGAAGGG
12 MLL2	CGCTGTTGCTTCTTCTTCTCATC	ACAAAGTGTCACCCTGCAAGTT
12 MLL2	CCATTCCAGCCCTGAGTCTTA	ACTTGGTTCTCCCCTCTCCAG
12 MLL2	GCTTTGTCACTCAGTCAGGATACT	GCCTGACATCCCTGACTCTGT
12 MLL2	CTTATCCAGACAAATTCCAGGGACT	TGCCTGTCACAACTTGGGAAA
12 MLL2	TGACACCTCACTTCCTTTGCC	GCGGCCAAGTGTTACACAAATT
12 MLL2	GTCCTGCGAAGGCACTTG	TTTTCATTGGCAGCCCCACTA
12 MLL2	CTTAGCTCCAGGGTGTCAACTT	CTCCTGTCGAAAAGCGAGAAGA
12 MLL2	CGTCCGCAGAGGTAGACAA	AGCCCGTAAGACTGACCGA
12 MLL2	GGGAATGCGGAGATGTAGGG	CTAGTGGGTGGCCTCTCTTG

12	MLL2	TCGCCAAAGAGCTACCCATTC	GGGAGAGCTATGGGCTGTC
12	MLL2	CGACGGAGGGCGTAGTG	CCTTGGGCAAGCCTCAAAG
12	MLL2	CCTGTGGCTACTGTGTAGTTTGT	CCATGCGTTTCACTTTCCCTCA
12	MLL2	GGTTGGCCCCTGAGGTTTG	CCTTATCTACAGGACCAGTCCTTG
12	MLL2	CTCTACCTGCTCCACTCTACTCA	AGAAGCTACTCCGGGCAAAG
12	MLL2	GATGGTGGAGGTGTGGGAT	GGGCTGCCAACCTCAACT
12	MLL2	CTGTCTGTGGTCCAGGGAAG	CAGCTACAGGCACTCCTCAT
12	MLL2	GGCGTACTGCCTGACTCT	TGGGTCAGCTTCGAGCAC
12	MLL2	CCCAGTGCTGAGTTGCACAT	GAGCCCAGTCAGTGAAGAGG
12	MLL2	CCCCTATTGGCTCCCCATTG	CTGGACCAGGTGAATGGACAG
12	MLL2	GAGGTTCCTGCTTGATGCTGA	GGGATCCCCCAGACAACCTA
12	MLL2	CTGCTCTGGCTTCTGGGTTT	TCCCTCCCACCCATCCAG
12	MLL2	CTTCAGGAGCCAGTCGGT	CTGGTCCTGCCCAGTTCATT
12	MLL2	ACTCTGCCGCTCCCTAAGAT	GCAGGATTGGAGGGTTCTGAG
12	MLL2	TCCCAGTCCTTTCTGTACATTGTG	CCATGTCAGCTCGCTTTCCAT
12	MLL2	CACCCTGAGTAACTTGGCTATGT	CAGTGCTATCACAGAAGCCCAT
12	MLL2	CCAAGTTCAGGTCCAGGAGTT	GTAGGAGGCTCCCAAGCTTTC
12	MLL2	CCTTCTTGTCATCAGGGCCAA	GTGTGTGGCTGACAGAAACTG
12	MLL2	tgactcACCTGGCTCTTTCAC	TGGTAAAGCCTGTGGGTGAGTA
12	MLL2	ACATGGAAGGTGGCATGGT	CCAGCTCATGCCCTCCAG
12	MLL2	TGGTCAGGTTACGCAGCAAG	TGTCTCAGCTAAGGACATAGGTTGA
12	MLL2	GGATGGAGCCAGGCGTT	CCTGCCCAGGCCATGTC
12	MLL2	AAGAGCCCTCATGTGGCAAA	GCTCATTGAGGACCTGTTGGAG
12	MLL2	AGGATAGGGTGCTCGCTGATA	TCTATCCTTCCTCACTGCCCTAAG
12	MLL2	CACCAGAATCACTCCCCTCAA	GAGCATGAAGATCCCCAACTCC
12	MLL2	TCTCTGGGAACAGCACCTCATA	ACTTGGCTGATATGTTTCTTCCATCC
12	MLL2	GGATGAATTTCAGGGACCCTCAA	CGATACCCCCACTGACGAG
12	MLL2	CTTGGCATGCAACGTACAGAG	CCAGTGTGCTCTCTAGGATTTGTT
12	MLL2	GGATGGGAGGAATAGGAGGCAT	GTCATTGTCCCCTTCTTTCACTCA
12	MLL2	AGGGCAACCTCCACATTCATC	GTTTCTGTCATGAGGAGGGTGAC
12	MLL2	CAGGTCCAGGTTCAGCAGAC	GGCAGAGTTTATGGAGCAGCTT
12	MLL2	GTCTCGCGGTACCTTGTCA	GTGAAGATTCCCGTCCTCCTC
12	MLL2	GCACTCCTTTCCATTTCTTGAGG	CTACTGAGCCCTTGGTTGAACTT
12	PTPN11	TCTTGAAAGGAACTGAAATACGACGT T	GCTTGAGCTTTCCAATCTGCTC
12	PTPN11	TGTGAAAGAACAACATGAACCCATAG T	CAAAATCTCCAGGGTGGCTCT
12	PTPN11	AACATGGTAGTTTTCTTGTACGAGAG AG	ACATCTTGCCAGACCCATTTTTCA
12	PTPN11	AAATCCTTCCTCATGTCCTGAAAGTA AC	TTGGAACACCATCCGCCAA
12	PTPN11	CTGCAAAACACGGTGAATGACTT	GGCCAATCTGACATGTCTGATACTTT A
12	PTPN11	TTTTGAGTCTGAAACCCCCATGA	TTCTTGCCCACCAGATGACC
12	PTPN11	GCCTCCCTTTCCAATGGACTA	ATGTTCCATGTAATACTGGACCAACT C

12	PTPN11	CCTGAAGCAGTCCAGGACTTATG	AGAAATGTATGGTCAGAAAACACTGTGA
12	PTPN11	ATTTTCTACTCTGCTCATAATGCGTCTT	CAGTAGGTTTTCTGCCTCCACA
12	PTPN11	TCATGTAAGCTTAAACAGCGTGGT	TGCTGAAGTTTTGGCAGGTTTTC
12	PTPN11	AGATGGTTTCACCCAAATATCACTGG	ACACTCTCAGGATCCTCTCTTTTCATT
12	PTPN11	aagaagtAATGCTGATCCAGGCTTT	CTCTTTTCGGCTGTAGAGAAGTTTG
12	PTPN11	CCAGACACTACAACAACAGGAGTG	AGGTACAGAGGTGCTAGGAATCAAA
12	PTPN11	CCTCACATGTGCACTCTTCCAA	CAGGTAAAAAGAATTTCCTTCAGCACT
12	PTPN11	TCTTTTCTGGTTTTTCTTGGCTCTACT	CGGCCCTGCATCCATGAT
12	PTPN11	GAGGTGCACCATAAGCAGGA	TTAAAGAGCTAGGAGTGGGTAGGT
12	PTPN11	CCTGGCTCTGCAGTTTCTCTTTA	CAATATAATGCTGGACCGCCATATAGAT
12	PTPN11	CAGACAGAAGCACAGTACCGATTT	CTAGCAAGAGAATGAGAATCCGCAT
12	PTPN11	ACAGGCCATTTTCCATGTTGGT	ATGACGCCATATTCTTTTAGAGCATACT
12	PTPN11	GTAAATGTGTCAAATACTGGCCTGATG	ACAGTTCCCAAAGTCTCTAGAATACGA
12	PTPN11	CTCTGTCCGTGCCTTTATGAATATCA	GCTTAGTTCTCGAACTCTGCTTTCTATT
12	PTPN11	ACGACTCGTATAAATGCTGCTGA	AGCAACTTCTCCTCCACTAAAAATAGG
12	PTPN11	GGAGAAATTTGCCACTTTGGCT	GCAGCAGACTTTGTGGTCACTAAAATG
12	SMUG1	GAATCGGGCACCACTCACTT	TCATGTCTCTCTCCTTCCTCACAG
12	SMUG1	AATGACTTCGAGGTCTTGAATGTGT	GCCAGAGGTCCAGGTGGAA
12	SMUG1	CTTGTTGGCCTGTGGGTTAC	GATCTGTGATGCAGCCCTCT
12	SMUG1	CTCTGCCAGTCGCCCAA	ACAATCTATGCCCTCTGCTTTTCC
12	SMUG1	CAGCCAAGCATCCACCTAGAA	AGTATGCATGGGAGCCACATC
12	SMUG1	GCAGTAGCGAGTCACGTAGTTG	CCTGGAAGCTTGGCTGAGAG
12	SMUG1	GCATTGAGCCGAAGCTCCTC	GGGCTTCTGTCCATCTCTTTTC
12	SMUG1	ctgactTGCACTCTGTCACACT	GTTACCTTTAGCTGACCCCTTTCC
12	SMUG1	CAGCTCAGCAGGAGTAAGGTT	CTGGAGTGCCCACAGTCAG
12	ZNF384	GCCCCATGTCTCAGACTCAAG	GGTCTTGCTGACATTCTTTCCCTTT
12	ZNF384	ACACAATGAGGGTACAGGGAGAA	AGAAGCGGATGCTGGAATCAG
12	ZNF384	GGAGAGGACATAAGGGTCATTCATCT	CCCTGACCTCTCCAAGAAGGTA
12	ZNF384	CCTTCCTCGGTTAGGGTCGAT	AGCATCATCAGCTCAGACCTTC
12	ZNF384	AACAGAACTTACTACTGAGACTTGGAGA	CCTGAGTGTGACATGTTCTCTCAT
12	ZNF384	GGTGCAGGCAACATGGTCTTA	CAGCACATCCGTATACACTCAGG
12	ZNF384	CACAGAAGTTACAACTGTAGGGCTTA	GTGCTCACTGACATTCTACTCCA
12	ZNF384	AGTGGATCTGCATCTCCGACT	AGGTGGGCTTCTCTTGGAAGTA
12	ZNF384	gctgAAGATCAGGCGGGTT	GCTCCAGAAAACCAGTATAAAGCTTAC

12	ZNF384	GATCATGGAAGATCAACACCTCAGAT	CCTCAGGCATCAGTATGGACAC
12	ZNF384	AGCTGGTCTGACTTGGACTCT	CGAGAACACAATGTTCATCAACAAGAT
12	ZNF384	CCTTCTCTGGCAACAGCTGAT	GAAGTGCTCATCCTTGATCCCT
12	ZNF384	GGGTGAAGTGGACTGTACTCTTA	GATGCAGCCTCACTAGAGGTG
12	ZNF384	CACTGTGTGCGTAGACAGGT	GGCAGGAGGAAAGAGTACAATGT
12	ZNF384	GCATGCGGGTTGTTGTGT	GGGCCAAGCCCTACAACTG
12	ZNF384	AGGCCTTCTGGCAGTAGGAA	GGCAGGATCCACTCCAAGATG
12	ZNF384	GGCTTGATGGTCTCCGTGTG	CATCCGTCAAGCAACGTCATTT
12	ZNF384	CCAGAGTACACAGGAAATCCCA	TCTCTGAATCAAGGAGTCTCCTGTT
12	ZNF384	CAATCATGGGAGCCGAAATGG	TGAAATAGGATTAGGATCAGGACTGAACA
12	ZNF384	AAAGGACTTTTCCCACCAAGAGTT	GTATAAGACGGCGGAGCATCA
12	ZNF384	TGACAGTGAGGCAGATGTCCTTA	CACCACCACACTTCCAGTCTC
12	ZNF384	GGATTGCTGTCCCCACCAC	ggccTCCCAGGCATCAc
13	CDK8	GGCCTCAGAGGCTGTGAC	TTGGCTTTGTAGACGTGACCAT
13	CDK8	AAGTTGGCCGAGGCACTT	CTCTCCCTCCGGGCTAC
13	CDK8	GCACAAAACCTATACATCCTTTCTTCCT	GGGATAGGCAGCATGTGGATTG
13	CDK8	CCTCATCTCCTTTCCAGCGTTC	GCAACAGTGCATGGACAAGATTC
13	CDK8	GATAACTGTGAGAAGCAATACCGTCAT	ATGAGTTAACCAACTAGGCCACTTAC
13	CDK8	AGTTAATGCATCTCCTTTGTTTTGCAG	ATGTCTGCCAGCAATTCACCTTA
13	CDK8	GGTGAAGGTCCTGAGCGA	GGCTTCGATCATATGGAACTACATGAAT
13	CDK8	GTTTCTTCCCCTAGAATTGAGCACAT	CTGCTAAAGGCTTCAAAGGTGAATTAAA
13	CDK8	ACATGGGCTTTGCCCGATTA	CAATAGCTTTGGTATAATGCCTTGCT
13	CDK8	TTTGTTCTCCCTCTGAGCTGAAC	AGGTAGTGGTAGGAGGAACAACTC
13	CDK8	GACCCCCGTTGAAGAAAGTGA	CATATGTAAACACTGACATGCAGTCAAT
13	CDK8	TCAATATCTATGGGAATCCCTGAGTGT	GAGGTAACTGAACTGGCTTCTTGTT
13	CDK8	TCAAGTTTCACAGAGCTTCTAAAGCA	AAATCTCTGTGCAACACCCAGT
13	CDK8	GATGGTATTCACTACCTGCATGCTA	CACTGGTATCTACAGACGACTAGAAAAG
13	CDK8	CTCATCAGAAGGACTTTAGCCAATGTA	CCAAGTGGAATGCTTTACTATCTGGTTT
13	CDK8	AACTGCAGCCTTATCAAGTATATGGAAAA	ACTCTGAGTGCTAGCTAAGTAACCA
13	CDK8	TCTTTTTCTTATGATCAGCGTTTTTGCC	GAAACAAGAAGCTGCTAACAGTACTTT
13	CDK8	GGAAAGAACCACTTTATTTTGCACCt	ACAGTGAAATATTGGTTCTGACGTTAGT
13	CDK8	GGGCTATAGGGTGTATATTTGCAGAAC	ATGTGTACCTGCAGGAAATCCC
13	CDK8	ACCAGCTGGACAGAATATTCAATGTAAT	AACAAAGACTCAAGTAAAAGTAGCTTCCA
13	ERCC5	ACACTGTAAAACTGAATGGTGAGAAGT	GCATCAGGCTCTTTCTCTGTCTTC

13 ERCC5	CGTGAAGTCATCTCCATGTGAAAAACT	GATTTTCACTCTCCAGCTCCTCT
13 ERCC5	CCTGCTGGGAAGTAGCTCAGA	ACACACTTTTACATCTTCGTCATCGT
13 ERCC5	GTCTCATTGCTGTGTAAGTAATTGTTTCC	GGTGAGTTGCAATGTCCGTAATTTT
13 ERCC5	CCTAATCCTCATGACACCAAAGTGAAAA	ATCAGGTTTCCCCCACAGAAAG
13 ERCC5	CCGTGGTGGATGACTCGAA	CCAAGTTTCTCTTCCATAAGTTTTAAACCAA
13 ERCC5	TCTTTCAGCCATTAAGAGAGCTCTTG	CGGTATTCCTTTTCCATCTCTCACTTTA
13 ERCC5	CCGAGAACAGTGATGAAGGACT	CCCCACGTGAACAAGTGACATT
13 ERCC5	GCCAATTGTTCTTTGTTCCCTGTT	AAGCCTTTGAGTTGGTACTGTGA
13 ERCC5	TTTTGCCTTTAGGAGTCTGATGACTTT	GATGTGTCCTGAATGTTGCTGATTC
13 ERCC5	TGAACCAGCATATAGAACATGTCCAAAA	TCTTCAGAGACCACTCTCCTTGA
13 ERCC5	GGGCTTTCTGAAGGAGGTAGAG	GGGTGCTAGTTTATAGAATTCAGAGTCA
13 ERCC5	TTCCTTTCTCTCGGCTGCATT	CTCTTCGAACTTGGGTAAGACTGG
13 ERCC5	GTGTACTTTCCAGAGATGAAGCACTAC	GGTTTAAATGTGTCTGAATGTAGCAATGA
13 ERCC5	GGAAAATCTTAGGAGATACAGGGAATGG	TCGTTCTGTTCCAGCCGAAA
13 ERCC5	GCATGCATATTTTGTCAGCGGTA	ACCCTTCATCCTGAACTTAGGAGAA
13 ERCC5	CGTCACTGTGTACCCCTCACT	CCAAACAGCCAGATATCACTGTCAT
13 ERCC5	AGACTTCCGGAACCATCACTG	CCCAATTGATTGTGAAAGTCCACATATT
13 ERCC5	CGCGGCATGTCTATAGAAACTTTTT	ATGCATCAAACTAGTTCCTTGGAAAGA
13 ERCC5	AATCACAGCAATGTTTCTAGTGGTCTAA	GAAGCTTCTCTGTCGTTTTCCTG
13 ERCC5	GGACTTAGCGTCCAGTGACTC	AAGAGAATCGCAGGAAATCAAGACA
13 ERCC5	AGCTCTTGATGATTGCAGGATCATT	CAGTTCCTCTTCGCCTTGTTC
13 ERCC5	CTGAAACTTCCAAACCTCCCTCA	TGCCATTCATGGAGCGAATCTT
13 ERCC5	GCCACAGGAAGCTGAGAAAGATG	CTCATCCACCAACTCCTTAAAACTCAA
13 ERCC5	AGGAAATTGAAGTTGTGAGGATGAAGAG	GCCGATGAAACAAAGTGAGAAGATG
13 ERCC5	GCCATGGGAACTCAATAGAAAATCCT	AAAACTATACGACACTTACCAAAGTCTGTT
13 ERCC5	AGAGCATGAGAATTTTCTGGAAACCAT	CCAGATTTCTACAAAAGCACAGACAC
13 ERCC5	CCACAGACTCAGTTCCAAAAGAACA	CCCAGGTGCGTCACTATGTC
13 ERCC5	CCTCTGGAGAGTGCAGTGGTTA	CAAAGTTCGTTCTGCTGCTCAAG
13 ERCC5	TCAAAGAATGAAACACATGCTGAAGTG	AAACTGACAGGGCCAATGACT
13 ERCC5	CAAAATGTAAACCGAATTCTGCTTCTGA	TCAGTGGTCTGCTGTTCTTGG
13 ERCC5	GGTTGGATATAGATATAGATATACACACGTACA	CCTTCGGTATAATCACTTCCAAGCAAAT
13 ERCC5	CTCTTTAGGATTGGACCGGAATAAGTT	CATGCCCAGGGAATTCATTGAG

13 ERCC5	GTTGTGTAACCGCCATGGAAATT	TCACATGTTACAGCATGACAGTTCAT
13 ERCC5	GTATGAAATGTAAATTTCATGGTGCTGTGA	TCTGCGCTTGGTGAACTCTTT
13 ERCC5	AGATATTGAGTCTGAGGACTTCAGCA	GTTGCATATTTCACCTCTGGCATT
13 ERCC5	GATCAAGGTTGAGCTTGTTGATTTGG	AGCTCTGTTTAGTCTCTGGCTCTTAA
13 ERCC5	CAACAGGAGAAAGAAGATGCTAAACGTA	CTGGGTTTTTCCTTTTGCCTTATCAAG
13 ERCC5	GTGACCGCCAGATCTGTGTT	CGACAAATTCATTACAAATGGCTGTCA
13 ERCC5	TGCCATGGAGAAAGAATTTGAGCTA	AGATGATTCTGAGAGGCAGGTCT
13 ERCC5	GAATACATGCGGTGGATTTTTGGG	TTCCTTCACTGAGTTCTGCGAAT
13 ERCC5	GAGCCAAAAACCAGTGCTTCAG	CGTCTTAGTTTCCTTCTTTTCTTCCCA
13 ERCC5	GTGCAGTCCGTCGTAGAAGAA	CAACGCGGCGTTAAGGATAC
13 ERCC5	CTTCCTAAGGAGAAAGAGCTTATTGGT	AGTGAATTCTGCTGTGCTAAGAGG
13 ERCC5	GAGTTGGAAACTCTGGAGAGCAA	TCTGAAGAAGGTGGTAAAGGAAACC
13 FLT1	GAAATCTTACCTGACATCATCAGAGCTT	AATCAATGCCATACTGACAGGAAATAGT
13 FLT1	GGCAGGAGTTGAGTATGTAAACCC	AAATTGTTGAAGTCAGAATTGGTGCAA
13 FLT1	GTGATGGGTCAGTTGAAAGCC	AACAAACCAGTGTCTTCTCTTCATTACA
13 FLT1	CTTGCAGTGGTGTTTGTTCTACATTTA	CATGGAGTTCCTGTCTTCCAGAA
13 FLT1	AAACAGTAAACAGCAAGACTGACCT	ACAGTTACGGTTCTAATGCTTTCCA
13 FLT1	CTTATTTGCAGTGAAAGTATGCTGAGA	TCCCCGAAATTATACACATGACTGAAG
13 FLT1	GGGAATGACGAGCTCCCTTC	TGTCTAGATGCTTGCATGTCAATCA
13 FLT1	TGTGGTACAATCATTCCTTGTGCTT	CCTGCAAGATTCAGGCACCTATG
13 FLT1	CCTGTGTATACATTCCTGGCTCTG	TTACTTGGATTTTACTGCGGACAGTTA
13 FLT1	TGCTAATACTGTAGTGCATTGTTCTGTT	CCAAATGGGTTTCATGTTAACTTGGA
13 FLT1	TCTCCTTCCGTCGGCATTTTT	GTGCACGAACAATTCTTAAGATCTTTGA
13 FLT1	CCTGCGGGATTTCACTTGCT	GGCCATACTCTTGTCCTCAATTGTA
13 FLT1	GTGTTCAAGGGAGTGGTAGCAG	CCGAAGTGCTCTGTATTTTTATCTGTGT
13 FLT1	CTACCTTTTTACTCTGCCAGGGAAT	AAGATCCTGAACTGAGTTTAAAAGGCA
13 FLT1	TGCCTTTTAAACTCAGTTCAGGATCT	TGAAAAACTCACCTTCATGGAAAGATAACT
13 FLT1	TTGTAAAAATATCTCAGCGCGTAGGA	AGACTAGATAGCGTCACCAGCA
13 FLT1	CTCAACATCACTCAGACTTTTATCTTCCT	GGGTCCTTTTCGGTCTCTTCAA
13 FLT1	CCTAGAATTGGGAGCTGAGAGTGTA	GGGAAACAGAATTGAGAGCATCAC
13 FLT1	TTTCATACTCACTAGGAAAGCAACCTTT	GCTGCAAATATCTAGCTGTACCTACTTC
13 FLT1	CCTTCTATTATTGCCATGCGCTGA	CTGTAAAGCCACATTGTGACAACTTTT

13 FLT1	AGATTGCAGATTCTGTTTCCTTCTTCTTT	CCTGTGGAAGAAATGGCAAACAAT
13 FLT1	TGTGTTCAAGGTTAAAGTACTGCAGA	GGCCCAGCCATAAATATGTTTTGTT
13 FLT1	TTTCAGGGACTACAGCTGAGGA	AACGTGGTGAAGATTTGTGATTTTGG
13 FLT1	GGTTCTTATAAATATCCCGGGCAAGG	AGTTGGTTAGGTTAAGGAAATGCATTGA
13 FLT1	ACTCTAGACATCAGATCGGGCTT	GACAAGTCTAATCTGGAGCTGATCAC
13 FLT1	GCCACACAGGTGCATGTTAGA	CCATTTAGGGAAACTGAGCCTCTT
13 FLT1	GGCTGTCTCTGGTTATAGGATGTG	CTGTTGCTAGCTAAAGTTTCCTTTTCTT
13 FLT1	CATCATAAGGGAGCCGCTCA	ACAGTGGATGTTAACGGAATATCTAGGA
13 FLT1	CCCTGACGTACATTCAAAGGCATT	GTTGCTGTTTTCCCCACCAG
13 FLT1	AAGGAAAGAAGAACCTTGGCATCTT	GGATGAGAGCTCCTGAGTACTCTA
13 FLT1	CCAAGGGCTCACATTTCAGGAG	TTGGTCTTTTGTACCCTTAAGCCATAA
13 FLT1	TGTTCACTAATTTGTTGCCTACCAGAA	TGAAGAGGATGCAGGGAATTATACAATC
13 FLT1	GGAGACTGGGAGGTGGAAAGTTA	AGTCACAGAAGAGGATGAAGGTGT
13 FLT1	CACATTTGACTGTTTTATGCTCAGCAA	CCGTGTGAAATGACTTACTGATCATGT
13 FLT1	GTTGGTGGCTTTGCAGTGATAG	CATCCAGGCCCACTAATCTAAACAT
13 FLT1	CCTCGTACCCCTCCATCAAC	GTATAACCTTTCTCCTTCTCCCCCTA
13 FLT1	CAATACAGGTTTCTCTTGCTTTATGTTGT	CTGATGGTGATTGTTGAATACTGCAAAT
13 FLT1	TGAGTTGGCAACGCTGAACTA	CCATGCCAACATATTCTACAGTGTTCTT
13 FLT1	TCTTGAGGTAGTTGGAGAGATTTCCAT	GGTTTCTCAGGCAGGCTGAAG
13 FLT1	CCTTTGTCTTTGTTCTGCATTTTGTCAAT	ACTTTAAGCATGAACACTCACTCCTT
13 FLT1	CCCACATGCAGATTCCCATCT	CGCATATGGTATCCCTCAACCTAC
13 FLT1	GGGTGCCAGAACCACTTGATT	CCCGTTCTTCATCTCCTTTCCAG
13 FLT1	GTTCTAATCTAAAGGCTGTCTAACCAA	GACGTGTGGTCTTACGGAGTAT
13 FLT1	CTAAGGAGAAGATTTCCCACAGCA	GTTTTCATGCCCTTCTGCTCAAG
13 FLT1	GGCCCTCCCCTTCTCCATTA	ACTCTTCCTTGCTTCTCACACAG
13 FLT1	AGCCCCGACTCCTTACTTTTACT	CAAATGACCCACTCAGAAAGAGTAGATT
13 FLT1	TGGCTCCCATGGAAAGATAAAGG	ACAACTCGGTGGTCCTGTACT
13 FLT1	AGGCTTCGTGTCAAACTCTAGATG	AGCTGTGGGCACGTCAG
13 FLT1	CAGCGTGGTCGTAGGTGAA	TCCTTTCTTTCTTTAGCTTGAGAGTAACC
13 FLT1	AAATACAGAGCACTTCGGCTTATGT	AAAGAAATAGGGCTTCTGACCTGTG
13 FLT1	GGGTGCACTAGGTACCTTAACT	GCCTTTTCCTGTCTTGATAATTCTTTCTG
13 FLT1	CAAATGCCCATTGACTGTTGCTT	GTTAATGCATGCCTCTTCCTTTTCAG

13 FLT1	TCTCCGGGCTACAGCCT	GCGCTGCTCAGCTGTCT
13 FLT1	ACCCCGGTGTCCCAGTA	GAAGTGGTTGTCTCCTGGCT
13 FLT1	GGTGACGGGACTGTTAAGGG	CATCAGCAGTTCCACCACTTTAGA
13 FLT1	GGGACACCATTAGCATGACAG	CGAGTAATCACAGCTCCCTTTCTTT
13 FLT1	GCCATTTCCTTGTAATTGTTGTACCC	TGACGATCTCTCCTGTCTTCCA
13 FLT1	CATCTCCTCCGAGCCTGAAA	GCTGTTTTCTCTCGGATCTCCAAATTTAA
13 FLT1	TGTCTGAGGAACGTCTCTTGGT	CGGTCTTACCGGCTCTCTATG
13 FLT1	GCGAGGGAAATGCCTTCACTTT	GAGGCCCTAAGGTTTTGTTTATGGA
13 FLT3	TGCATTCATTTCCATTTTAAAGTGCTACTAC	TTTTAATAGCATGCCTGGTTCAAGAGA
13 FLT3	CCAATGGAAAAGAAATGCTGCAGAA	GCCAGCTACAGATGGTACAGG
13 FLT3	TCATTATCTGAGGAGCCGGTCA	ACCCTGCTAATTTGTCACAAGTACAA
13 FLT3	TCAACCAAATTACAGTCTGACTTGAA	GCTGCAATTGTCTAATGTTTTCCTCA
13 FLT3	TGTGTGAGCAGCCTGCATTA	GGCAACTACTTTGAGATGAGTACCTATT
13 FLT3	CCACTGATGATACAAAAGCAAACAGA	CAAGTTTGTATTCTGAAAATGCAGGAACA
13 FLT3	GGAGAAAAGGCAGACTTTAAGGGATA	CATATCAAGTTGCCAAAGGAATGGAATT
13 FLT3	GGAGCATACCGACTTAAATTCCAGA	GCAAAAGTGAAGACTGGCTCTATTT
13 FLT3	GAGAAGCCCATTTAGCCATGGTA	GCCTGTGATCAAGTGTGTTTTAATCAAT
13 FLT3	TTCCCCACTGATGAATCATTGTTCTTAT	GCATGTTTTCTTTTTAAGGTGTGCATGT
13 FLT3	GGCAACCCAGAAAAACTGCAATG	TTTAGGATGTCAGCTGGCAGATG
13 FLT3	GCTGCTACATACCGCTTCTTCTG	TCTTCATCTTGTTCCTTGTTCTATTGCTT
13 FLT3	TCATGAAAGAGTCAATAGGTCAGAGAGT	TCTCGGATGGATACCCATTACCAT
13 FLT3	TGAACACTTCTTCCAGGTCCAAG	ccAGTGAGCTTATTTCACACGTT
13 FLT3	AAGTTGAATGATCACCTACGCAGTT	TCATGAATTATTTGGGACGGACATAAGG
13 FLT3	CAGTTCATTTCTGGCACAGCA	TGTTCTCCTCTTGAGCATTCATTTGTAA
13 FLT3	TCCAGGCTGGAATACTAGTAGCA	GGAGTTGTTTCCATGGTCATTTTGAAAA
13 FLT3	CTCCAGCTTGGGTTTCTGTCA	ACCAAACTGCCTTTTTAGTCACTATGAA
13 FLT3	GGTACTCATCTCAAAGTAGTTGCCCTA	GGGCTCACCTGGGAATTAGAAAA
13 FLT3	CCTATTACCTCCTCGAGTGCTTTG	CTCACTTGGGATGTTTTCATCTCCTA
13 FLT3	CCAGCAGCTGCCCAACTTA	TCTTATCTCCTCTGGGTTTTCCACA
13 FLT3	CCATCTGTAGCTGGCTTTCATACC	TGTTTGTCTCCTCTTCATTGTCGTTTTA
13 FLT3	TTGTACTTGTGACAAATTAGCAGGGT	ATTCTGTTTCATCGCTGAGTGACA
13 FLT3	TCTTGTTTTGGTAATCTACAGCCTGTT	CGTGTTTCGGAATGTCCTCAC
13 FLT3	GGTCGCCTGTTTTGGTAGGT	AATTTGTAATATGGGCGCATAACATGTG

13	FLT3	CCTGATGGTGGAATATCACAAGAACA	GTGTCGAGCAGTACTCTAAACATGAG
13	FLT3	CCAGGAACCCTTTTATGGCTTCA	ATGACAGCTCACCTGTCTAAATTCTTT
13	FLT3	TGATGTGTAATTACGAAACCCTGACC	GAATACTGTTGCTATGGTGATCTTCTCA
13	FLT3	GTCCAAGTCCTGTGAAATTTTTCTCTTT	TGAAATAACAGTTTGCTTTGTGTATGCC
13	FLT3	ACATGAACAGAAACTTGAAACAAAATTCCA	GTCTTTAAGCACAGCTCCCTGAAT
13	FLT3	GGTATTCCTCCACTTACCTGTTTTGTAA	CAGGGACAGTGTACGAAGCTG
13	FLT3	AAGCAGATACATCCACTTCCACAG	CCTTAGACCTGCAGTTTTCATGAATT
13	FLT3	CATTTTTGTGCATCTTTGTTGCTGT	GCACGTACTCACCATTTGTCTTTG
13	FLT3	TCCTTCTCAAGGGCGACAAG	CTGTCAGGTTTAAAGCCTACCCA
13	FLT3	AATAGCAGCTACCATGGATGTGTC	TGATGCTTTAGATACCCTGCTTTACAC
13	FLT3	ACCACAGTGAGTGCAGTTGTTT	GCTCGAGATATCATGAGTGATTCCAAC
13	FLT3	GTTTTGTAGTAGCCCTTCTAAGCCATTA	CATGCAGAAAATGATGATGCCCAA
13	FLT3	TCAGCGTGAACATTTTGGTAAATTGG	TGTAGCAATTGGGCTAAATCTGTCTTTA
13	FLT3	gagagagCAAACATCCTCTTTGTCA	AAAGAGAGGCACTCATGTCAGAAC
13	FLT3	CCAGCTGGGTCATCATCTTGA	GCAGATTGACTCTGAGCTGAGAAAAATT
13	FLT3	CTCACATTGCCCCTGACAACATA	ACAGTAAATAACACTCTGGTGTCATTCT
13	FOXO1	TGGAGGTGGCCGAGTTG	TTCTCCCCCTCTTGGCTCT
13	FOXO1	CCTGCTCCGCACCTTCAG	GCGTGCCCTACTTCAAGGA
13	FOXO1	GAGCTGTTGCTGTCACCCTTA	CGCAAGAGCAGCTCGTC
13	FOXO1	CTGGAAGTCCCCGCACA	TCAGCGCCGACTTCATGAG
13	FOXO1	CCTCCAGCAAGCTCAGGTT	GGCCGGAGTTTAGCCAGTC
13	FOXO1	GGGTGAACTTACCTGCTCACT	GACCTCATGGATGGAGATACATTGG
13	FOXO1	CCCTTCTCCAAGATCATCCTGTTC	GGCTCTCACAGCAATGATGACTTT
13	FOXO1	GCCATAGGTTGACATGACCGAAT	AGTATAACTGTGCGCCTGGAC
13	FOXO1	AGGGCGAAATGTACTCCAGTTATC	GTAAATTTGCTAAGAGCCGAAGCC
13	FOXO1	TGGGCAACACATTGTCAAAGTTAAAAT	AGGAGAAGCTCCCAAGTGACT
13	FOXO1	CAGAAGTCAGCAACTCCTTCAAGA	TGAATTCACCCAGCCCAAACTAC
13	FOXO1	ACTGGAGAGATGCTTTCTTCTTGG	TCGTGTGCAGAATGAAGGAACT
13	FOXO1	GCTCAATGAACATGCCATCCA	GTATGAACCGCCTGACCCAA
13	FOXO1	GAGCATCCACCAAGAACTTTTTCC	GAAGTTTTGCAAGCAGAGAAGTATTGT
13	FOXO1	TCATGCTGGATTGGCCATATGTATATTTT	TCAACCTTCTCTCATCACCAACATC
13	FOXO1	GGCACTTGTACAGGTGTCTTCA	CCAGGCATCTCATAACAAAATGATGAAT
13	FOXO1	GCCCGTTAACTGCAGATGTCT	GAACGTCATGATGGGCCCTA
13	FOXO1	GAGGACTGGGTCGAAACAGTTAAT	CGCAAAGATGGCCTCTACTTTACC

13 FOXO1	GGGATTGCTTATCTCAGACAGACT	GGAGACTCTCACCCATTATGACC
13 IRS2	CCGTACTCGTCCAGGGACAT	CTCCCTGTCGTCCAGCA
13 IRS2	CTGCTGGACGACAGGGA	CGCTCGCACACCCTGAG
13 IRS2	AGTCCACAGATGTTTCCAAACACA	TGCTAACTTCCCTCCTTTCTTTTTCAG
13 IRS2	CTCGCTGCTTTTCCTGAGAGA	GCCACAGTTCCGAGACCTT
13 IRS2	CCCAAAAAGTGGGAGCAGTGAA	CTTGTCCCACCACTTGAAGGA
13 IRS2	GCCCGGCTTAGGAGACTT	GCCTCGTCCTCCTCGCT
13 IRS2	ACCGTCGTGGTGGAGGA	ACGTCGGGCGTGAAGAG
13 IRS2	CTCCTCACCTTTCACGATGGT	GGGTCTCATCAGCGCTGT
13 IRS2	GCTGCTGGCGGACAAGA	CACGAGTACCCACTGCCA
13 IRS2	CACCTGCTCCATGAGGCT	CGCCGCCTCATCGTTGT
13 IRS2	GCCCACAGCGCTGATGA	GGCTTCCAGAATGGTCTCAACTAC
13 IRS2	CGCCAAAGTCGATGTTGATGTACT	GCCCGGAGGGCTTCTTG
13 IRS2	CCGGTGTCCGAGGACAA	AGGCCTCCTCCCCGTATc
13 IRS2	TGGGTACTCGTGCATGCTG	AGCCCCCTCACCCTGTAG
13 IRS2	TGGCCTGAGGCTCCAGA	GCTGCTGCTACAGCTCCTT
13 IRS2	CCTCACGTCGATGGCGAT	CACCTCCCCACGACAGTT
13 IRS2	GCACGTACTGGTCGCTGT	CCCGCCCGACTTCTTCTC
13 IRS2	GCAAGGAGCTGTAGCAGCA	CCAAGCTGTCCATGGAGCAT
13 IRS2	GGGCTGCGGAGAAGAAGTC	CCCGAGGACAGTGGGTACA
13 IRS2	GGCGAGCTGGCCTTGTA	CGCCCCCAAGCAGATCTTG
13 IRS2	TCGCCGGGAATGTCCTG	CGACGACTACATGCCCATGAG
13 IRS2	CCGGCTGCAACTGTCGT	CCGTCCTTCGCCCACAA
13 IRS2	CGGCACGAGCTGCACTT	CGCAGTAAGAGCCAATCGTC
13 IRS2	TGGGCTGCAAGATCTGCTT	GAGATCGGCTCCCACAGG
13 IRS2	GGTTGACCAGGTGGTGGTG	CCATCCTGGAGGCCATGAA
13 IRS2	CGGAACTCGAAGAGCTCCTT	GCCACTCGGACAGCTTCT
13 IRS2	CCCAGGTTGCTGCTGGAG	CTCTGCCTCGCTGGATGA
13 IRS2	CACCAGCCCGTAGCTGT	GGAGCAGGAGGGCTGGTA
13 IRS2	GCTGACCAGGTCGGTGAG	CTGCTGCCTGAACATCAACAAG
13 IRS2	GCCCGCATCAGGGTGTAT	CACTGTGGCCGCTCCTA
13 IRS2	GAGGGCGATCAGGTACTTGTG	TCGAGTACTACGAGAGCGAGAA
13 IRS2	GGCCCACCTCGATGAAGA	GTACCGTCTGTGCCTGTCTG
13 IRS2	GGTCAGGGAGTAGGTCCTCTTG	CGAGTCCATCGCGGAGA
13 IRS2	GCCTTGCTCCGCCACTTT	CGCAAGTGCGGCTACCT
13 IRS2	TGGTCATGTACCCGTAGAACTCA	CGACCCCGGCTTCATGTC
13 IRS2	TCGCAGTTGAGCTTCACGAA	GACAGCTACGGGCTGGT
13 LAMP1	GTATTCTGGAGCCACTAGACCTCT	CCTGGACCCACACTTTGAATATATTG A
13 LAMP1	GTGTCACGAAGGCGTTTTCAG	CGGAAGCGACTAAACGTCCA
13 LAMP1	CATCTTTCTATGAATCTGCTCCGTGAT	TCGCAGAGATTGTGGGTTCTC
13 LAMP1	CCTGAGCTAGGGTGGTGACTT	CACCATCCCGAACTGGAAGAG
13 LAMP1	TTCTCAACATCAACCCCAACAAGA	ACAAAAAGATGTGGTGGAGACACA
13 LAMP1	TCTCCGTCTTCCCTGGAATTGA	AAGTGCCCTGGTGTTTCCTAC
13 LAMP1	CAAGACTGTGGAATCTATAACTGACA TCA	CCAAGGGTCAGCGTCCTA
13 LAMP1	AACATGACCTTTGACCTGCCA	CTCGTGAAATTGAGAGTGAGTGTATG T

13	LAMP1	CTCGTGATTGCTTTTGGAAGAGG	TCATAATCGGCCCATTTTGGTTCTTA
13	LAMP1	CTTACTGTAACTGCTGATGGTTATGAGA	TCACGTTGTACTTGTCCACAGAG
13	LAMP1	CCTCACCCGTGCCCAAG	CCCAGCCCTACCGTGTT
13	LAMP1	ACCTCACCTATGAGAGGAAGGAC	TTAACAGCATGACGTGCAGAGA
13	LAMP1	CTGACACCATCCGTCTGTCTT	AGATAGTCTGGTAGCCTGCGT
13	LAMP1	CCTCATCGTCCTCATCGCCTA	CCCCTTCGACAGGACCCTAA
13	RB1	GCCAACTTGAAATGAAGACTTTTCCT	GAAAGAGAGGTAGATTTCAATGGCTTCT
13	RB1	CTTCCACCAGGGTAGGTCAAA	CCGTAAGGGTGAACTAGGAAACTTG
13	RB1	CTCACATTCCTCGAAGCCCTTA	ACTACTTCCCTAAAGAGAAAACACACAC
13	RB1	AACTTGAGGTTGCTAACTATGAAACACT	CCTTCAGCACTTCTTTTGAGCAC
13	RB1	GATGGTATGTAACAGCGACCGT	GCTACTGGAAAACATTCAAAACTGGTT
13	RB1	TGAGACAACAGAAGCATTATACTGCTTT	CTGGAGTGTGTGGAGGAATTACATT
13	RB1	GATGTATTGTTTGCACTCTTCAGCA	GCTAACATTAAAAGGGACAAGTCTAAGA
13	RB1	GTAAGTCATCGAAAGCATCATAGTTACT	TTCTGCCAGTTTCTGCTGAAATTTG
13	RB1	CAGTAAACATCTCCCAGGAGAGTC	GCATAAACAAACCTGCCAACTGAA
13	RB1	GTCATAATGTTTTTCTTTTCAGGACATGTGA	AGCCTGCTATAATCGATCAAACTAACC
13	RB1	TCTGATTACACAGTATCCTCGACATTGA	CAAGTTACCTGTGATCCAATTTCGAC
13	RB1	GCTGTGGGACAGGGTTGT	GCAGCAGGGATATAGTATCTGACAGTAT
13	RB1	tCCTTTAGCAAACTTCTGAATGACAACA	CTCTCCCCCGACCAAAGAA
13	RB1	CTGTTCTTCCTCAGACATTCAAACG	CCTGGTGGAAGCATACTGCAAAATAT
13	RB1	CGGTCTTCATGCAGAGACTGAAA	GTGAGGTATTGGTGACAAGGTAGG
13	RB1	TCTTATTCCCACAGTGTATCGGCTA	TGCCTGTCTCTCATGAGTTCATACT
13	RB1	GAACATATCATCTGGACCCTTTTCCA	TCAGTTAACAAGTAAGTAGGGAGGAGAG
13	RB1	AGAAGGCAACTTGACAAGAGAAATGATA	AGGTAGATGTTAAGAAACACCTCTCACT
13	RB1	TGAGCTCAGTATGGAAAGAAATAACTCT	TCACTTTGCATATGCCATACATGGA
13	RB1	CATGTATGGCATATGCAAAGTGAAGAAT	AGATCAAATGAATTACCTATGTTATGTTATGGA
13	RB1	TGCGATTTTCTCTCATACAAAGATCTGA	TGGTTCTTTGAGCAACATGGGA
13	RB1	GTGGTTCTAGGGTAGAGGTAACCTT	AAGAAAATACTCACCCCGAATGATTCA
13	RB1	AATACAGTTTTAACATAGTATCCAGTGTGTGAA	CAACTGCTGCAATAAAGATACAGATTCC
13	RB1	CGTCATGCCGCCCAAAA	CGGCTCTGCTCGCTCAC
13	RB1	GTTCCCAGGGAGGTTATATTCAAAAGAA	CGTTTCCTTTTATGGCAGAGGCT
13	RB1	AGTAGAATGTTACCAAGATTATTTTTGACCTA	CCGTGCACTCCTGTTCTGA
13	RB1	CCACTTGCCAACTTACCCAGTA	GGTGTACACAGTGTCCACCAA

13 RB1	TCTTGGACTTGTAACATCTAATGGACTTC	TGGCTAGATTCTTCTTGGGCAAAA
13 RB1	TCATTTGGTAGGCTTGAGTTTGAAGA	TCCACAGATGAAACTTTCTCCCAAG
13 RB1	TGAGTTTGAAGAAACAGAAGAACCTGAT	GAAAATCCTTACCAATACTCCATCCACA
13 RB1	AAATTCTGCATTGGTGCTAAAAGTTTCTT	GCAGAGAATGAGGGAGGAGTACAT
13 RB1	TCTTGATTCTGGAACAGATTTGTCTTT	GGGATTCCATGATTCGATGTTCACAT
13 RB1	CTGGGAAAATTATGCTTACTAATGTGGTTT	ACAAGCAGATTCAAGGTGATCAGT
13 RB1	GGACCAACTGATCACCTTGAATCT	GCACTTATGCTTAAAATCTATTTGCAGT
13 RB1	TATTCTTTCCTTTGTAGTGTCCATAAATTCTTT	GCTGAAGAGTGCAAACAATACATCATAC
14 AKT1	AGGCCGCGAAGTCCATC	AGTTCCAGCTCCCCTTCCTT
14 AKT1	AAGACAGGACATCGTCCCCTA	CACCCAGCCCTGCTTTACA
14 AKT1	CCACCTCGTCCTGTAAAGCA	CGCTACTACGCCATGAAGATCC
14 AKT1	CCTGGCAGCGGGTACTAA	CTTCCTTTGCTTCTCCCAGAGG
14 AKT1	GTGTAGCCTGTAGCTGGGATG	TTCTACAACCAGGACCATGAGAAG
14 AKT1	CCTCCATGAGGATGAGCTCAAAA	CCCTTGATGCCGAGTCCTG
14 AKT1	CTGAATCCCGAGAGGCCAA	CGCTACTTCCTCCTCAAGAATGATG
14 AKT1	GCTCCTTGTAGCCAATGAAGGT	GGGTCTGACGGGTAGAGTGT
14 AKT1	GCAGCAACGCGTATGCAC	GAACCTCATGCTGGACAAGGA
14 AKT1	CAGCCCGAAGTCTGTGATCTTA	ACTCGGAGAAGAACGTGGTG
14 AKT1	GCGAGTGTGTGGGAAATCTGG	AGATCATGCAGCATCGCTTCTT
14 AKT1	CGTGCTGCCACACGATAC	GGCCCTACATCACAGGAGGAA
14 AKT1	CCTCTCCATCCCTCCAAGCTAT	CTGAGCTGTCTACACCCACAG
14 AKT1	GGGAGTGAGGATGGCTACAG	ACTGTCATCGAACGCACCTT
14 AKT1	CCCACGTACCGCTCCTC	GCAGAGTGCCAGCTGATGAA
14 AKT1	CAGCGGATGATGAAGGTGTTG	CCTGGTGGGTGGTATGCAAG
14 AKT1	CACCTTGAGGTCCCGGTA	CGGCTGTGCCTCAGGTT
14 AKT1	CAGCCCTCCACAGTCCAAG	CTGACGGCCTCAAGAAGCA
14 AKT1	GGAAGTCCATCTCCTCCTCCTC	GGTGGAGCTCCTGATCTGGTA
14 AKT1	GCCACGATGACTTCCTTCTTGA	GCCCATAGACCATGAACGAGT
14 AKT1	CCAGCAGCTTCAGGTACTCAA	GTGAAGTGGACGCCTCTCC
14 AKT1	GCCTCTCTGAGTGTGGAGAGAAA	TCGGAGACTGACACCAGGTAT
14 AKT1	GGCCGTGAACTCCTCATCAAA	CTTGAGCACACTTGAGGGTGT
14 BCL11B	GCACCCCAAAAAGCCTTCTG	CGGAGGCTCCCTTTGGAT
14 BCL11B	GTCTGTTACCTGACAACTGACACT	TCCTCCCACCCTCACTCATC
14 BCL11B	GCACGCAGAGGTGAAGTGAT	CCCGACTGACCAGTCTTTTCTATT
14 BCL11B	GCAAGCGCAGCATCCCATA	GAGCTCAGGAAAGTGTCCGA
14 BCL11B	GGTGACTTGGATCCCGATCT	GGGACATCCTGGTTTTTATAGAGCA
14 BCL11B	CGCCACACTGCTTCCTTTTG	CGAAGAAGACGAGGGTCTGGA
14 BCL11B	AGGCCACTTGGCTCCTCTAT	CCGCCTAAGCCCATCTCTATC
14 BCL11B	CGCACTCCGCAGACACTT	CCCATCAGTGCAGCTCTCC
14 BCL11B	CAGGTCAGCATTCTCTCGGTT	CTACAGCACCCTGGAGAAACA
14 BCL11B	CTCGCCGTGCCACTTTTTC	AGTGCGAGCTGTGCAACTA

14 BCL11B	CCAGCCACTGCGAGTACAC	CCTTCCCCGGGCTCTTC
14 BCL11B	GCCTAGGCCCACGTTCT	GGCCCGAGTCGAGCTTC
14 BCL11B	CAGCTCCGAGTCCATGCT	CGGCCGACGGTGACTTC
14 BCL11B	GGGTCGCTCTCGTGGTG	AGCTCAAGCGCCACATGAA
14 BCL11B	CCGGCCTTGTGCATGTG	GCAAGTCGTGCGAGTTCTG
14 BCL11B	CGAGTCGATGGCCATGGG	ACGCCGCCTCTCTTCAG
14 BCL11B	CCATCTCCTCGGCACTGA	TTTCCTGGGCGACAGCAA
14 BCL11B	TCATGCGCAGCAGGTTGA	GCTTCCGCATCTACCTGGA
14 BCL11B	ATTGCTCTGGAACTTGAAGGTCTT	GCGGCAACCCTATGCAC
14 BCL11B	GCCCGTGCGTCTTCATGT	CAGCGACACGTGCGAGTA
14 BCL11B	GCTGTCGCCCAGGAAATTCAT	CTTCAACAGCGCGTGGTTC
14 BCL11B	GTTCAGGAGCCGGTGCAT	GTGCCTTCGACCGAGTCA
14 BCL11B	TGAACACCTTGCCGCAGTA	CAGCCTGCGCTTCTCCA
14 BCL11B	ACGAGTGCTCGGACGAC	GCTCATCCCGTCCGAGAAC
14 BCL11B	GAAGCCGTGCGTGTTCTG	CCGCTTCTCACGCTGTCT
14 BCL2L2	CTCATCTCACTGGGTTGGTCAA	CCCTTCTGCCTCAGCTTATAACC
14 BCL2L2	GCTCTGGTGGCAGACTTTGTA	CCGCCAGATCAGAGAAGGTG
14 BCL2L2	CAGCTGGAGATGAGTTCGAGAC	GGGCCCCCTTGAAAAAGTTC
14 BCL2L2	CTTCACCCAGGTCTCCGAT	CCATCCACTCCTGCACTTGTC
14 BCL2L2	GTGTCAACAAGGAGATGGAACCA	CTGGAGACCAGCTTTGCAGAA
14 BCL2L2	ctctcctGATATCCCTTTCTCCTTCT	CCCCCGTCAGCACTGTC
14 BCL2L2	GGGAACTGGGCATCAGTGAG	CCAAGGGCATTTCTCTGTTCTGTT
14 DICER1	GCCAAGCAGAGGAACTGAGATT	AGGAGCTTGATTTGCATGATGAAGA
14 DICER1	AAGCTGAGTCAATCAGAAGTGCAT	TCGCATTTTGGGACTAACTGCTT
14 DICER1	AATTCCTCTGGATCACATTTCCCATTTAA	TGTTTTAATATGGCGATGTCTCTCTTGT
14 DICER1	TCTTCCTGGAACACTGGTCTCT	GCAAAATCTGGATTGTGTTAATTCTCTCT
14 DICER1	CTCTGCATGTCTAGTGATGTCTGG	CCCCTGAGTATGAAACTTTTGCAGAATA
14 DICER1	TGGTTAGGTCAAGGTTGTACTTTGTTTT	AGATATCGCAATTTTGATCAGCCTCA
14 DICER1	ATCAGCAAGTGAATAGCTCTACAAAA	TTGTAGCTCTCATTTGCTGTGAGAA
14 DICER1	CAAACATCCTTACCAATTTTGTGCAGT	AGCGATGTGCATATATAATGGAAAGGTT
14 DICER1	ACGGGTATTTCCTGCTTGTAGTAATTTAG	TGGTAAGCTCCACGTTGAAGTT
14 DICER1	CAACACTGCAAACCACTTTCAGG	TGAAATGCTTGGCGACTCCTTT
14 DICER1	ACCATTAATTGCTGTAAGATCTGCTGA	TGCTGCACATCAAGGTGCTAAT
14 DICER1	GGTCATGATTTTCTAGAGAGGAGGTTCT	CCTAACTTAGACTTCGGGTGGAAAAA
14 DICER1	GCTGAAGAGGAGTTAGAAATTGAGATGA	AATTGCTGTTGCTCTCAGCCTA
14 DICER1	AGATATGTGGTGATGGCATGCTTTAA	GCAGGATGGATTCTGAGCAGAG
14 DICER1	GAGTCCTTGAGGAGTACCCAATAGAA	AGCGATGAATGTACTCTCCTGAGTAATA
14 DICER1	GGTAGATTTGTTAGCATTTCCATCAAGG	CAGAGACTTTTGCCAAGGAAATCAG
14 DICER1	TTCCTTTTCCTAAGCAAGACGTTTTTG	CAACTTTAGAAGGCGGAAGCTCTA

14 DICER1	CATCTTGTGGTATCTTCAGGAGGA	TGACGCTTAATTTTCCTTTCCAACTCTA
14 DICER1	ccgaccTAGTGCATCTTTTAAAACAA	CATAGGCATTCCCAGTACAAAGTATACAA
14 DICER1	AATGATAACGGCATCTTGGTAATCTTCT	ACTCCAGCACTTTGGATATTGACTTTAA
14 DICER1	GCTTCAGACTTCTCAATATCTTCCATGA	ACTCTAGACACATATACAAACATACAGCTTTG
14 DICER1	ACTCAAACAAATACTAAGTTATGCTAGTACAA	TGACACCTCGACATTTGAATCAGAAG
14 DICER1	CACTGCTTAAAGGAAGCGCTTT	GCCCAAGAGGCTGTAGTTGAAAT
14 DICER1	GTGCTGGAGTCATTAACTTAGAAGAGAA	AGACGCTGATTCAGCATACTGTG
14 DICER1	CATTAAGAGGTAGAACACAGTATGCTGA	AAGTCTGGTTTCATGTTGTCTCTACAAA
14 DICER1	GGTGAAGTCTTGTAATCAACTCAAGCA	CCTTTTGCTAAGATTCCACACTTTCCT
14 DICER1	ACCTCTCCAGAGCGTGTGTA	AGATCGAGGTGCCTCTTCTATTTCT
14 DICER1	ACAGAGACTCCTAGTTAGACCACTTTTT	GCTGTAAAGTACGACTACCACAAGT
14 DICER1	CTCAGGAGAGACAGCTTTGAAGT	GCGATTTTGGACTACCTCATAACCA
14 DICER1	GCGGGTCTTCATAAAGGTGCT	TGTAAATGCTTCTGCACAAGCTTAC
14 DICER1	GAGACCCTATGGGCACTTTGTC	GAAGAATCAGCCTCGCAACAAAC
14 DICER1	CCTGTTTTCTGAATTCTGCTTCCATCT	CAGCAACGTTTAACTTCCAAATGTGTAT
14 DICER1	CTGCCTTCAATTCATTCCACTCAC	TCCGAAGCCTCAAAGCTAATCAA
14 DICER1	GGTTTCAGCTATTGGGAACCTGA	AGAGTCACTGTGGAAGTAGTAGGAA
14 DICER1	CTGTAACTTCGACCAACACCTTTAAATT	GCCTGAGAGTGCACACTAAATG
14 DICER1	TCCAATTTCCCCTGCACAACTT	CAGTACTACTCACTAAAGAGCTGTCCTA
14 DICER1	GCTGAAGTCTCCCCTGATCTGA	ACCATGTTTCTAGGTTGAACTGCT
14 DICER1	GGTATTATGATCCAGAGCTGCTTCAA	GGAGGACGATTTTAGAGAATAGTGAAATTTCC
14 DICER1	CAAGGCTCCTGCTCATGAAAGT	ACTCTTTATCTGCCAATTAACTCACCTC
14 DICER1	AACTTACAACAATGGAGGCTCGAA	TCAGCCTCAGAATTTCTTTTTCTTCACA
14 DICER1	ACTGTGCAACATTCCCAGGTT	ATGATGATGACGTTTTCCCACCA
14 DICER1	CATCGTCAGGCCTCAACACATA	GCAGAGAGGATGTGTTTTCATCATAAGT
14 DICER1	CAAGGCTACAGATCATCTTACAAACCA	AACTCATTCAGATCTCAAGGTTGGG
14 DICER1	TCCAAGATGCATTTACTTCTAGGTTTGA	TGTTAATCATTAGTAGTCCAGAGAGCCA
14 DICER1	GTTCTTTTGCAAACAGGATCTCATGAT	ACTTTATCGCCTTCACTGCCTTT
14 DICER1	CTCTTAGCTCCTCTGCAGTCA	CTGTCCATTTTTCCCCTTACTTGAAGA
14 DICER1	GCTCAGTGTGCAAGTCGTAAGA	GCTCTTGGGATGCAATGTGCTA
14 DICER1	CAACAGCTTTGCTAGGATCCAGA	CTCTCTTTCTCCTTTTTCAACCACTGA
14 DICER1	GGGCATTTTCCATTCATATGCAGAA	TCCGAAGGAAGAGGCTGACTAT

14 DICER1	CCTGATCATACTCCAGGAAATCATCTTC	GCTATGTTTCCCCTCCTTCCC
14 DICER1	CCAATCTGCCGGCACATGTTA	CCCACAGAATATCGATCCTATGTTCAAT
14 DICER1	GGTGCCCTTGCTCTTCCTTTAG	ACCTGCTTATTGCAACAAGTATTGTAGA
14 DICER1	TTGCATTTTGGTATATCAACACCCTCT	TTCTTAGAAGTGCTTTTCTTGGTGGAT
14 DICER1	TCTTTCTAAAGGGAGCCAACAATACC	AGGATATTGAAGTTCCAAAGGCCAT
14 DICER1	CCAGCAAGCGACTCAAAAATATCC	GAAACTACATCTGTGGACTGCCT
14 DICER1	AGCACACCACAGTGTAAATATTTTTCAG	TGTGCGAGAATTGCTTGAAATGG
14 DICER1	CTTACCTAAATTTGGCAGTTTCTGGTT	GGTATTGTTGGCTCCCTTTAGAAAGA
14 DICER1	GTTCCATTTCAAGCAATTCTCGCA	CCCATGATGCGGCCACTAATA
14 DICER1	ATGCTCCAGTATTAGTGTTCGCATT	CATGACCCCTGCTTCCTCAC
14 DICER1	GCAGTCCAAAGAAAGGACCCATT	TTTTCCCTTTTGTAGTAAGCTGTGCTA
14 DICER1	AAGTGCTTCTTCTAATTCCATCAGCA	ACTGCAGTATTGATACCTTTTGTTTCTT
14 DICER1	CTCCCTGTTCTCATGTGAAAGGA	TCTGAGGATGATGATGAGGATGAAGAA
14 DICER1	TGTCTCTGGCTTCTCTTTTTCTTCAAT	CAAACTGCTCGAAATCTTACGCAAATA
14 DICER1	AACTGCTGTCGCTCATATGGTTTA	GGAGCTGCACAGGAAATTTTTATTGT
14 DICER1	GCTCTTCACATAGTGCATGTATTTTCCT	AGACTGTCGTGCCGTATTGG
14 DICER1	ACACCAGGGTCCCAGAACTA	GAGCCATTTGAGAACAACTGAAATCC
14 DICER1	TGGCTCACCGAAAAGTAAATCCC	GCTTCCTCCTGGTTATGTAGTAAATCA
14 DICER1	TCCCATTTATCTGTGTTGCTTTTGTCT	ACTGTAATCTGTATCGCCTTGGAAAAA
14 DICER1	GGGATCAAATATTGACACCACCAT	GCCTTGAATGAATTCCAGCAGTGA
14 DICER1	TGCATAGATCACTTTTACAAGGCCAA	CTTACCTTCTCCAGGCTTTTACACA
14 DICER1	TGATAGTATTGTAGTGGTAGGAGGCA	TGTTTGAAGATTCCACCAAGATGTATGT
14 DICER1	AGTGTTTTATCTGCATCTGGATGATCAA	CCCTACTCGGGAGAATTTCAACAG
14 DICER1	ACTGCAGCCAAACTCCCAATA	TTGATGAGTGTCATCTTGCAATCCT
14 DICER1	GGGTGGTCTAGGATTGCAAGA	GCAGTGAATGCTATGTTTGTTTACTGAT
14 DICER1	CAGCTCACTGAAAGGTTCTTTTGTT	TCAGCTTTTCCTCTGTTCACTGG
14 DICER1	TTTAATTACCGGGAGCACCTTCAG	ACACGGGAAAGCAGTCCATT
14 HIF1A	ACACCAATTTCAAGGTTTTTGGTTGTTT	CCAAGAAAGTGATGTAGTAGCTGCAT
14 HIF1A	GAAACGTGTAAAAGGATGCAAATCTAGT	AAGGAACCAAAGTCATTCTCACTTAG
14 HIF1A	AGGTGTGGCCATTGTAAAAACTCA	TGACAACTGATCGAAGGAACGTAAC
14 HIF1A	CTATATCCCAATGGATGATGACTTCCA	GGTAGTGGTGGCATTAGCAGTA

14 HIF1A	TCCAGCAGACTCAAATACAAGAACC	GTGATGATGTGGCACTAGTAGTTTCTT T
14 HIF1A	ATCTCCATCTCCTACCCACATACAT	ACTCAAAGCGACAGATAACACGTTAG
14 HIF1A	GACAGAAAAATCTCATCCAAGAAGCC	GCGTTAGGGCATATATATCAGAAAGT TT
14 HIF1A	AGAGGTTGAGGGACGGAGATT	CTTCAGGTTTTGAGTTTCCTAGACCTT
14 HIF1A	CCAATGTCGGAGTTTGGAAAACAAAT T	AGTTCAGCCCCTATAACTAGGAACA
14 HIF1A	GTATTTTCTTTAAAAGCGCTCACTGGA T	CTCTGAGCATTCTGCAAAGCTAGTA
14 HIF1A	GTTCCTGAGGAAGAACTAAATCCAAA GA	CACATTAGCTAGAAAAGCAAAACCTA CT
14 HIF1A	CCACCTCTGGACTTGCCTTTC	GCCGAGGGAATGGGCTTAC
14 HIF1A	TCTTGAAATGTTCCTGTCCATAAAGC A	ACACATTCTGTTTGTTGAAGGGAGA
14 HIF1A	AGTGGTATTATTCAGCACGACTTGAT TT	CCAGCAAAGTTAAAGCATCAGGT
14 HIF1A	GTAGCCTCTTTGACAAACTTAAGAAG GA	GCTTGTAGCAACAGACACAATTTAGG
14 HIF1A	GTGTCTGCGAGAAAACTTTGTAAAAA CA	CCATAACAAAACCATCCAAGGCTTTC
14 HIF1A	GACATGAAAGCACAGATGAATTGCT	AGAGCTCTTAATATGTGTGCATTTTAC C
14 HIF1A	CATGCTGAGACTTAATTGAACGGGTA	AGCTTCGCTGTGTGTTTTGTTC
14 HIF1A	TCATTAGGCCTTGTGAAAAAGGGTAA A	CTTACCTTCCATGTTGCAGACTTTATG
14 HIF1A	CCTAACTAGCCGAGGAAGAACTATGA A	ggAACTGTAAACCTTCCCTCACAAT
14 HIF1A	ACATGGCATCTTCTAATCCTTCTGTG	CTGGCTGCATCTCGAGACTTT
14 HIF1A	GTAGGATAAGTTCTGAACGTCGAAAA GA	AATAGCTGATGGTAAGCCTCATCAC
14 HIF1A	GTTCGCATCTTGATAAGGCCTCT	AAAACATTGCGACCACCTTCTAAAAA T
14 HIF1A	CCTATCAGTTAACTTGGGAGGAGAAA	TCATAAATTGAGCGGCCTAAAAGTTC T
14 HIF1A	CGAATTGATGGGATATGAGCCAGA	TGTACAGAGGTTGCAACAGTTAAGTA AT
14 HIF1A	TTTTATCAAAGCTTACTGGCCATGT	ACTGAGGTTGGTTACTGTTGGTATC
14 HIF1A	GCACAGGCCACATTCACGTATAT	TCATATCCAGGCTGTGTCGACT
14 HIF1A	TCCTTTAGATAGCAAGACTTTCCTCA GT	CGCTGAATCTTTGCTATGGTGAATAT
14 HIF1A	CCACAGACACAGAAACTGATGACC	GTTTCAGCGGTGGGTAATGGA
14 HIF1A	CCTCACCCAACGAAAAATTACAGAAT	CCAGTGACTCTGGATTTGGTTCTAA
14 HIF1A	TGCACTCAATCAAGAAGTTGCATTAA AA	ACCTACCTCAGGTGAACTTTGTCT
14 HIF1A	GGGATTAACTCAGGTAAAATGCACAC A	CTCATTTCCTCATGGTCACATGGAT
14 HIF1A	CTGGACACAGTGTGTTTGATTTTACTC	GCCTTGGGTAAGTACAATAGCAAAGT TT
14 HIF1A	GCAAAGTATATGGAAGCTTCTTCAGG AA	GCTTCTGTGTCTTCAGCAAAAAGT
14 HIF1A	CTTTTTACAGCCTAATAGTCCCAGTG A	CCTGAGTAGAAAATGGGTTCTTTGCTT
14 HIF1A	GGTAGAAAAACTTTTTGCTGAAGACA CA	GAGTTTTTACAATGGCCACACCTT

14 HIF1A	TTTCCAAAACAATGATGAACATTCAGCA	ACCTCTTTTGGCAAGCATCCT
14 HIF1A	GACAAGTCACCACAGGACAGTA	TTACTTACCTCACAACGTAATTCACACA
14 HIF1A	TCTCAACCACAGTGCATTGTATGT	TTGGTAGCGATGTAAAAGAATTAAAATTTAGTT
14 HIF1A	GGGCAATCAATGGATGAAAGTGG	CAGTTAACTTGATCCAAAGCTCTGAGTA
14 HIF1A	GAAACCTACTGCAGGGTGAAGAAT	GCAGTATTGTAGCCAGGCTTCTAAAA
14 HSP90AA1	CCCACAGAGCCTACAAGATTGTAA	GCTTTCAGAGCTGTTAAGGTACTACAC
14 HSP90AA1	CATCTCATCACCAGAGGCAGAT	CATTAGTTTTCCAATTGGCCTCTTTAGt
14 HSP90AA1	CCCCAAGAAGTTCACACTGAAAC	GGTTGAGACGTTCGCCTTTC
14 HSP90AA1	TCAACTGGGCAATTTCTGCCT	TGTAGACGTCCTGCAAGGTTTT
14 HSP90AA1	CAAGTTTGGATAACTGAAAGTTCACCAT	TGAAACTGCGCTCCTGTCTTC
14 HSP90AA1	GGATCTTCCAGACTGAAGCCA	ACAATGGGTTACATGGCAGCAA
14 HSP90AA1	CAGGGTTTATCTCCAGGTGTTTCT	ACTTTTTGATTACAGGTGGTTGTGTCA
14 HSP90AA1	TCTTAGGGCTTGAGCTTTCATGATTC	AGTCATCACTTTTTGATTACAGGTGGTT
14 HSP90AA1	GGAGATGTCACCAATCGGTTTGA	AAGTTGTATTTGACAGTAGATAGTATTTTGGA
14 HSP90AA1	GTGTATTCCAAGCTGAAACAAAGTATGT	GAACTGGCGGAAGATAAAGAGAACT
14 HSP90AA1	GTAGTTCTCTTTATCTTCCGCCAGT	CTCGGAGGATCTCCCTCTAAACATA
14 HSP90AA1	GCCAGTTCAGTAAAGAGTTCTAAGCATT	TGGAATGACTCAGTGCATTTGGT
14 HSP90AA1	ACAAGCTTGAAGCACCCATCA	gaaaagTACATCGATCAAGAAGAGCTCA
14 HSP90AA1	GGGCTTTGTTTTGTTGAGCTCT	aaagaGTCGGAAGACAAACCTGAAAT
14 HSP90AA1	TTGAGCTCTTCTTGATCGATGTActttt	agaaagaGTCGGAAGACAAACCTG
14 HSP90AA1	ctcatcAGAACCAACATCTTCAATTTCA	GCAGTGATGTGATTTCGTGTTTTCTTTT
14 HSP90AA1	AGAGAGTGACATTGTTTTAGCTTTCTGT	AGGGCAACACCTCTACAAGGA
14 HSP90AA1	GCCTAAGCAATATAAATGGCTGCAGA	GTGTTTCTCTGGCATCTGATGGT
14 HSP90AA1	AGAGTAGAGTGGTGGATCCAGAC	TGTTTGGTTTGGAAGTCCAGAAGATT
14 HSP90AA1	gcccACCCAGAAAGTACTTCT	GACGTGCTCCTTTTGATCTGTTT
14 HSP90AA1	TGTTTTCAAACAGATCAAAAGGAGCAC	ATCTTTCTCCACCACCCCAAATATC
14 HSP90AA1	TGATCGTTGGGCAAACACAAATTC	AGTACGCTTGGGAGTCCTCA
14 HSP90AA1	GTGCCTACCTGTGTCTGTCC	TTTGCAGGCTGGTGCAGATAT
14 HSP90AA1	GGTTATTGATCAAGTCAGCCTTGGT	TTGTTAACAGGCATTGGACAAAATCC
14 HSP90AA1	TGGGATCTGTCAAGCTTTCATACC	GTAAGTCACTTATTAACCCAGAATCGGA
14 HSP90AA1	TCAATGATCAGGAAATGCTGTATTCACA	GGGAAGACTTTAGTGTCAGTCACC
14 HSP90AA1	ccaGGCCTTCTTTGGTGACT	CAGCCTTTGTGGAACGTCTTC
14 HSP90AA1	CAGTACTCATCAATGGGCTCAATCATATA	GCAAGAAAAGTCAGAGCTAGATGAAAC

14 HSP90AA1	ACAGTCACCCCAATCACCTACA	TGAAAGAAGACCAAACTGAGTACTTGG
14 HSP90AA1	CTTCACAATCTCCTTTATTCTTCGTTCCT	GGCGTTGAGCACTAAATTGAATGTATTA
14 HSP90AA1	GAACACCTATGCTGTACCACATTCT	CGCCCAGAGTGCTGAATACC
14 HSP90AA1	AGTCCCTTGGTACCTTCTCAGAAA	CGGATGCCCCCGTGTTC
14 HSP90AA1	AGAGGAATTGTAGAGTACTGAACAGGTA	TGGAAACAGGTATTGATGAAGATGACC
14 HSP90AA1	GCAGCACTGGTATCATCAGCA	GTCCTGTAATACTGTCTTGAAAGCAGAT
14 NIN	CTCCAGGACACTCTGGAACAAC	GAAAAGGAAGCTCTGAGTGAGGAATTA
14 NIN	ACTTCTACTACCTTATCGACACAGCTATT	GGAGGGCTCATTTGTTTATCTCTTACAG
14 NIN	GTGCCCCAGGTAGCTTCATT	GTGAGAGGCTTGACTCAGGAAC
14 NIN	GCTCCTGGTGAAACTGCTCTA	GCAACATGAGATGGAGCTCAAGG
14 NIN	GCTTGAGCCTGTGTCAGTCTA	GCTCAAGGAGGCACATCATGAG
14 NIN	TCAAGCTTCACTTGCAGTTGTTTTT	AGAACATGAAGCAAAGGCATGAGA
14 NIN	GTTTTTCTAAGGTGTGCGTTTCGT	ATCTTGTGGTGGTGTCACTCTG
14 NIN	GGCATTGCTCCTGTTCTTTTGA	TGGCAGAATATGAGAATCTGACAAACAA
14 NIN	CAGGTTTACCTTTTCTGCTAACACATTT	ACCACATTTAATTTCCCCATACTTGTTCA
14 NIN	GCTCCACACTCATGATCCAGAT	GAGTTTCCTGAAGTGACGGTGA
14 NIN	GCTTCTTCATCCAGTGGCTCAA	CCTCAACTCTTGTCTCCTCTCTCA
14 NIN	AGTAAAGTGCACAAATATGAGTGTACCC	AAGTGACTGAACAGCGACAGAA
14 NIN	CGTGAAGATGAAACAAGAATTGTCCAT	CTTACCTCTTTTTCCCTTCCTGAAACA
14 NIN	AGGAGGTAATTTTTCTTCTCTGCTGTT	AGTCAAGATGTTTCTGGTTCTGTTTCT
14 NIN	ctggaCCACGCTTTCCTG	AGAAAATGGAGGAAGTCACTGAAACATT
14 NIN	CGTAACTCTTTTCCAGGCTGAGG	CGAGAAAATGACTGCCTTCAGGAA
14 NIN	CGTGTCTCCATCATTCTCAGCTC	GGACCTACTTTTTGATGTTTCTGTGCTA
14 NIN	gccgctcATATTCATTTCTCATCTG	TTGGGTCAAAGAAGGGCACT
14 NIN	GGATTCTCTCAAGCATCTTCAGTTTCT	TCCTGGGAGCTGAAGAATCAGAT
14 NIN	CCACTTCCCATAGCCACGTT	GGAGGAACTCACCGACCTTTG
14 NIN	CATTAGCTGTTCCTGAAGCTGACTA	GGAAGTACAGGGACGAGCTCT
14 NIN	TCCTCCAAGCTCAACATGTGG	CTTACACACAGGTGAGCACTGT
14 NIN	GCCTGCTGCATTTCCTTTATTTTACTC	TGGAAAGACTAGAAATGGAACATGACC
14 NIN	GTCCATCCAAGGCATCCGAAT	GGCCCTTGAAAATGAACTTTTGGTT
14 NIN	GGACATCATTTCCTGCCTTTCCT	CAGATACTTGAGCTGAAGAGCAGTC
14 NIN	GCTCCCTCAGTTCCCTTTTGT	GAGAGAGAGATGCTGGAGAAAACAT
14 NIN	CCTGGTGAATGCTGTTCTTGGT	TGAATTTACGCCTTGCTCTGATTTCTA

14 NIN	GACCATGCTGTTCAAATGTTCTTTGT	GCGTGAGGAGAAATCCCAGTG
14 NIN	TGAGCTCGTCCTTCTCAAATTCC	GCCCAGTTTCAGTCTGATTGTCA
14 NIN	TCCTTAGAAGTACACCTGAGAAGAAACA	CCACAGAGCAAGAAAAATTGAGCTTAA
14 NIN	CTTTCTGAAACTGATCACACTCTCTCT	GGCTGGGTGATTGTATTAGCATTTTT
14 NIN	GCCCACTTCCAAAGCTGTAGTT	GGCAAGCAGCGTTTAGAACTTG
14 NIN	TCTGTTTTTGCCTTTTCAATTTCCTGTT	CAGCTTGTCCATTTTTCTTCGTTACAG
14 NIN	aGAACAAATCACCTTGCCACCA	AGAATTTAGAAGACACCGTGCAGAAT
14 NIN	CCGGGACATTTGCAGGTTTAC	GTCCTTATTCATTCCTGAACATGCTAGA
14 NIN	TCCTCAGCTACCAAGCTAGTT	CAGGAAAACCACCTTCTCAAAGATGA
14 NIN	GCCTTACCTGTTTCATTTTCTCCAGT	CCCCAGCCTAAATATGTATGTCTGTT
14 NIN	GGGTGATATGAGGGATAGCAAATC	TCACATTCCAGCTTTAACTCCAGTT
14 NIN	ACGAGTTAATGGCAATAAAGGGATGTAA	GTGAAAAGGCTTCTTCAAGAGAAAGTG
14 NIN	GCTCACTTGTTCTTTGAGCTGATT	ACCAGTGTTTTCAGTGTGACATGAT
14 NIN	GCTTTATCTACAGCCTGTCCGA	AATATCGTGCACAAGGCAGAGT
14 NIN	CTTCTTCTGACGGTGAGTTCTTCAA	CAGTGTTGGTTTTTAAATCCCAGGT
14 NIN	TCAGGTGGCTACAGGCCATA	AAAACCAGGAAAAACTGCAAGAACTTAA
14 NIN	GGCATAGCATTTCTGTTAGACGTTGA	GCACAAGTAGGGTAACAAGTTTGAAAAA
14 NIN	GGCCTGCAGCATAAGAGATCA	CACACTAATCTTCTCTTGCCTTCTCTA
14 NIN	CAGGCTGACCAGAAGAGTCC	TCGGAGGTGGATGATCACCAT
14 NIN	TCATTCCGCCGCTCTATGG	TGCTTAGAAACAGTTTCCCATTAACTCA
14 NIN	TCTTCTTTTGCTTCCCTCTCTTTCC	ACCATCAGCATCTACTCCATATAGACA
14 NIN	GCATGGAAAGGTGCCTTTTTAGT	TCTAGATGCTCGAGGAAGTATTCCATAA
14 NIN	CTCATTGTACCGTCAGGATCAAGA	GTACTGATTACTACTGGCCGGAAGTATA
14 NIN	CTCCCAACAAATACCTCATTCATGGA	AGTCAGATGAATTCCCTTGAACAAGAAT
14 NIN	TTTCTTTTTCAGGCCTTCATTTTCCAA	AAGAGTTGAGATTTCTGACAAGCACA
14 NIN	GCTATATGCTATTCATTACCGTGTGCT	AACGGAAACTGTAAAACAAGAAAATGCT
14 NIN	GTTAGTTTCTCTAGGCACGGCAA	agctgAAGGCAATGATGCATGA
14 NIN	CCTCTATTTTGACTTCCTGTCTCTGAAC	TGGAAATACTGCGGAACAAACAGA
14 NIN	GCTTTCACACCTCTCAGTGACTTTC	GGCAGTTTCAATAATGCTGTATGTTGAC
14 NIN	GCTTCGTTCGGTTTTGCTGTAAAAA	TCTAGATTGCAACAGAGGCTACAAAAG
14 NIN	AGAAGACATTACTAACCCTGGCTCTAA	CCTGTCCCTGCTTCAGCAAG
14 NIN	CGTCCCCATTTTCTTCCAACAG	GAGCACAGAAATCTCCAGACTTCA
14 NIN	AAGTGGATTGGTGGGAAGGAC	AGGATCAATGAGTCCCATGTGTTTT

14 NIN	AGGCAGAGAAGAGAAGTGAGTGA	TGCATTCTTAGAGCTGTGGAAATTAC TTT
14 NIN	CCATGATGTGCAGCTTCTCCAA	TCATCTGATGAATGAGGAACAGCAG
14 NIN	gtctgCCATGGTAAGAAATGCC	GAAGTGTACTACTTGCCTTCTTTCTCT
14 NIN	CTCTCATTCTCTTGCCAAAGCAG	TTTAAGGCTTACACTCTGCAATTCTCT T
14 NIN	GGTTCTTTCTCTCCACCTTTGAACT	CAGAAAGGATAAACCAGCACCTG
14 NIN	GGAGGTCCTGTTTTCAAGTTCCT	GTCCACTGCGACGCCTA
14 NIN	GTGTCCAGTATTCTCTCCACAGATG	CCTGGAATCAAGCTAGGCTTACT
14 NIN	GCTGCAAATCCCAAGCATGAG	GTCTTCATTACTCACTGCCTGACA
14 NIN	TTCAACCCTTCTCCAGTTTGTCATT	GTCTTCTCCTGCCTGGATGATG
14 NIN	CATTCCTGAATTATACTGCGAGGCTT A	GGATAGAAGAGAAACTGCAAGAAGT TTG
14 NIN	CGGGTGATCCCCAAATCTTCA	GTGCAACATATGCTTCTCTCTCAG
14 NKX2-1	GGGCACGGACAGGTCTTTA	TCGCTCATTTGTTGGCGACT
14 NKX2-1	GCCTTCTGGACGGCTCTC	GTACCAGGACACCATGAGGAA
14 NKX2-1	GGTTGGCGCCGTACCAT	CGCCGCCTACCACATGA
14 NKX2-1	GCCGCCGTCATGTGGTA	CTTGAGTCCCCTGGAGGAAAG
14 NKX2-1	CCTCCATGCCCACTTTCTTGTAG	TCCTCTTCCTTCCTCCTCCAG
14 NKX2-1	GGTGGATGGTGGTCTGTGT	GCCAGGTATCCAGCCTGT
14 NKX2-1	AGGTGGGACAGGCTGGATA	GCCATCTCCGTGGGCAG
14 NKX2-1	CGCTGCCCACGGAGATG	gccggTCCTGGTGAAAGA
14 NKX2-1	TGGCCTGGCGCTTCATT	CGGGTGCTCTTCTCGCA
14 NKX2-1	GTCGCTCCAGCTCGTACA	CTTACAGTCTCCCGCTTCATGG
14 NKX2-1	CCCATGCCGCTCATGTTCAT	CTAGGCTGCCTGGGTCA
14 TCL1A	cccATAGTGAGTGCTCCTTGA	TCGTGTATTTGGACGAGAAGCAG
14 TCL1A	CTGGCTGTACCTCGATGGTTAAG	CTTGAGAGGCTCTGGCTCTTG
14 TCL1A	CTGGGATGGAGGGAAGATAAACC	GCCTATCATGTGGCAGCTCTAC
14 TCL1A	GGATCGGTATCGTCCATCAGG	TGAAAGTATGCCCTGTCCTTTTCTT
14 TCL1A	GTGGTCTTTCTTTCTGATACAGCCA	CGATCAATGCCTTCTCTCTCTCTCT
14 TRIP11	CCTTGCATTTTCCATTGCATTAGAGG	CGTTCAAACCAAGAGCTAGAGAGAT
14 TRIP11	GCTTCACGGGTATAAGAATCTTCTGA TT	CAAAATGGTTTGTAGCAGTGGAAAGG
14 TRIP11	CATTGAAAGAGTGACCGTAGAGCTT	GTCATTGCAAGAACAGTTGAATGTAG TT
14 TRIP11	CAGTTTCATCCCTTTGCTTGGA	GGGAAGGTGGCTTCTTCTTGTT
14 TRIP11	TTCAGATGGCTTTCATCGTCCA	CTTGAGCTGTTTCTGCCTTTATTTTCA T
14 TRIP11	TCTACCTGAGCAATATGCCTCCA	GCATCATCTTCATTCGCTTATGGGATT A
14 TRIP11	GTGCTAAGCAGTACATTTAAAAAGAA AGGA	TCTAGGACAAAGGAAATTGAAGCCAT T
14 TRIP11	CTTACCTCTGATCTCAAGATTGCATG A	GCCTACTATTCTGTGTAAACTCAGTG AAA
14 TRIP11	CATCGTCATGGAAAGCTGAAGGA	AGGTAGAAATCAGCCATCTTAAAGCC

14 TRIP11	GGAGTGCAATCTGTCTGGCTTT	TCAGTTGATAGAGGACCATCATTGGAT
14 TRIP11	AATCACACCCACCATTTCTGTGT	AGGAAACACTGATAGCTGAGATAGAAGA
14 TRIP11	GTAGCTTCTTGATTCTGTCTGTCCAAT	GCTACAAGAGACATAAGTTTGGATTCAGA
14 TRIP11	TGCCTCCAAATTAAGTCTTAAGTCATGT	TGGTGTCTCCAATTGAAAGTATGTGT
14 TRIP11	CACTATTAACCACAGATTGCTGATTTGG	CTTTCAGTTGTTTCATGACGATCAGG
14 TRIP11	CTGGTAACACCGCCCTGAT	GTAGGAAACATTTACTAGGACTTGAACTT
14 TRIP11	TCATGAACAGAAAGCTTTGGTGGT	AAGCTGGTACATTTCCTTAGTTGTAGTG
14 TRIP11	GACAAAGACTCTACAAGTAACTGTTCCA	GAGGAAAAAGCTATGTATTCTGCTGAAC
14 TRIP11	GCTATAAGCTGTTTTTGCTTTTCGAGTT	GAGACATTTGAACTGTTTTCGTTGGATT
14 TRIP11	TTGGTCTCACTCAACTATGCCAATT	GCTGCATTGGATTCAGCATCA
14 TRIP11	CTAACTGTTCTGTAAGTCTTGATGCTGA	CAGCTGAAATGTAGAGGTTGACTCT
14 TRIP11	CCTTGGCTGTGACCTGTTACAC	AGTCTTGCTGAAGACAATCTGAAACTTA
14 TRIP11	CACCTTTTCTTGACTCAGTAATGACTTC	AAACACACAATTGTATAGATTGCCAGC
14 TRIP11	TGATTACTATTAACACCTCCAGCCTCAT	GAAAGTAGTGGCCAAGATATGTTTAGAGA
14 TRIP11	CTGACTTAGTGCATCTATTTCGATGTCT	GATGGATATTGTTGCTGCCAAGG
14 TRIP11	TCTTGCAGTTTGATAAGAGCTGCTT	GGCTTATGCTATGGAAAGAGAAAAGGTA
14 TRIP11	GGCTATTTTCCCTAGTCTTCTCATTCAA	AGCAGAAAGATTTGGAGATACAAGCTC
14 TRIP11	TGGGAAGTTGAAGAAATTCTAGCATGAA	GAGCGAGAACTGGAGATTAAACTTCTAA
14 TRIP11	TTCATCTTTGGACAACTGATCAATCTGT	GCATGATTTATCTAAAGCTGAAACGGAA
14 TRIP11	CACTGTCTCTATGCACATAACATTTGTC	CACTTTAATTGGGTTGCATTTCTGAGTT
14 TRIP11	GAGACTTTTACTAATTCAGGCCTATGCT	TTTTCTGATCTAGACTTCCAAAGCACAA
14 TRIP11	GATCAGAGTTATCTGTTCCTTGTGCTT	CGGAATCTGTGAAAATCCCCCTTT
14 TRIP11	TCATCAATTTCCTGACTTCGGTTCT	TCAGCCTTAACATGTTCTGAGAATTCTT
14 TRIP11	CTTTCTCCCCAAAGGCCACTT	CCCACATTTACACCTTTGCCA
14 TRIP11	CCCAGCACTGTTGTCAGGTAA	CTGGTAGAAGAACAGATGTAAATCCGT
14 TRIP11	CCAGCTGGGTTAATAAGAGGTACAG	CTTCTTTGTGAAAGATAGGAGACTAAATCCA
14 TRIP11	CAACTACTATGCAATGAAACAGCAATCT	AAGCAAAGTGATACTATGACAGAAAAGGA
14 TRIP11	TGATGCACTCTGGGCAAGAATT	GAGAACAAGAACAGCTAAATGTGGAAAAG
14 TRIP11	CATTTTCACTGATCACCTTTCCAGTTC	ATGTTGGCCCTGAAACAAAAACAA
14 TRIP11	CTCATTCTGTAGGGCAGTATTTTCCAT	GCAAGACTGGAGAGTTAAATCAGCTTTT
14 TRIP11	CTTCTCCTGCATTGATTTAACTGCATTT	TCGGGAGGCTGCCCtattta
14 TRIP11	ATTCATCACCTTTTCATATTTTTACAGATCTGA	CAGCAATCTACAAGTTACCGAAATCAAC

14 TRIP11	TTTCGGTAACTTGTAGATTGCTGCT	CGTTTTTCATAATGCTGTGCACATAACT
14 TRIP11	ACAAATGCACAGTCCCCTTCAA	GAGTGCCACTCAATGAAGGAGA
14 TRIP11	GCTGTTCAAAAGCAAGAGCCT	GGACATTGGCAAACTAAAAGGAGAAAAT
14 TRIP11	TCCTTTCCCCTGTATGTTTCCACTAT	CCAAAAGTGATCAACTACTTTCTTCCAA
14 TRIP11	TCAGACTTACTTGCTCTAAGACACTCT	CACAAGTTCAGCACAGCATTGG
14 TRIP11	GAAGATCCTTGGTATTGCAAAGCTG	CACAGGTTTTGGTTGACAGTGATAATAA
14 TRIP11	TGCCTCAAAAGTTCGTTTTCATTTACTT	AACACGAACAAACCGATTCAGAAATC
14 TRIP11	AGTTCTCGCTCTTTTATTCCTTTCACTAA	CAAATCAAAACCCAGTTGCATGAAGA
14 TRIP11	CAGAGTTATCTGTTTGAATGTCCTGTCT	GTTTAGATACCAGCATGAGCAAATGAAC
14 TRIP11	ACATCTTGTTTCTCCTTTAGCTGCTT	TGCAACAATTAGAACTCTCCAGGAAAAT
14 TRIP11	CTTCTCTAAAAAGAGCTGGGTGTGT	AAGATCAAAACCAGAGTAAGATGCAACT
14 TRIP11	CAATCGAATCAGACAATCTGTGGTT	TCATTGCTTACTCCCCAGTCT
14 TRIP11	GGCCAGTATAGTCCACTTGTAATTTAGAA	GCAGGTGATGACCACAGTACAAA
14 TRIP11	CATCCATTTCCTTCTTTTGCTCTTGTAAA	AGTGTTACTGAACTAGCATCTGAGGTAT
14 TRIP11	TGTTCCTTGATCGTGTTCAGTTGAG	CAAGAAGAATTGGAACGACTCAGG
14 TRIP11	GGGCTGACTCGTGTTGCATAT	AGTTTTACAAACATCCAGCACTGGTA
14 TRIP11	GCGGTTCGACTCTGCTCTTC	GCTGCAGGAGGAATTGGATAAATATTCT
14 TRIP11	GGTCTTTTTCCTCTATGGTCTGTCTTAA	GTCATCTAGTTTAGAAGAGCAGAAGCA
14 TRIP11	ATTTATCCAATTCCTCCTGCAGCTT	AACTAAGGACGTTTTGTCATCTAGTTT
14 TRIP11	GTGTCAACTGCTTCTGCTCTTC	CCTTACAGCTGGAACATGAGCATTTA
14 TRIP11	TGAGTTCTGCTATTTCCATGTCTTTCTT	AGGCAGAATTGTGTTGGGCTA
14 TRIP11	TGCTTCTTCCAACAGCCTCTTT	TGAGGCATCAGTTAGAAGAATGTCTTG
14 TRIP11	TTTCCAGAGAAAGCTGATTGTTACCA	GCAAAATCTTTCAGAATTAGAACAGCTCAA
14 TRIP11	GAGCTGTTCTAATTCTGAAAGATTTTGCT	GGAGCATATTAGACAAAATGAGGAGGA
14 TRIP11	TCATTCCTTATTCTAGAAAGCTCCTCCT	AGCAGAAACTTGAGGACAAAGTAGAAAA
14 TRIP11	CTCCTTCTGGATGCTTACATTACTTTCT	GCTAATTCAAAGTGAAGTGGCCCTA
14 TRIP11	CAGTTTACTGATGATGCTATCTCCTTCA	TTTTCTTTGAGTACTAAAATGAGAACATGTTGT
14 TRIP11	GACATTCTTCTAACTGATGCCTCACAT	GCAAAATCTTTCAGAATTAGAACAGCTCA
14 TRIP11	TGTCCATTTTATCATCTTCAAGTTGATGAA	ACCATGCAAATTTTTCTTTCAGCACAT
14 TRIP11	GCGTCTCCTGACTGATGGAAG	CTGGCCAGATATCAAACTTTACAAAGG
14 TRIP11	CGTGCCCTCCATCAGCATAT	GACCGGGTCGTTCTCTAAACTC
14 TSHR	CTGCTGCAGAAGGAAAGCATTT	GATGGAGGGAGTAGAACTGGTAATACT

14 TSHR	GTAAAGGCTTCTGCAAGTCCCT	CAGAGCCTCTGTGAGCATGTA
14 TSHR	AGTGGCATTTTCAACACTGGACT	CCCTCATGACTGTATTCTAGTTCTTGTC
14 TSHR	AAGCTTGCTGTGAGTAAGACATACA	GGTAGCCTATACAGCCAGGAAAAG
14 TSHR	GCATGATCTGGGAAGCGCATA	CTCAAATTGTAGAAGGAGTGTGATTCCA
14 TSHR	GACTCTGCAGCAGCTGGA	CCATCTGGAGCCCCAAGATTAT
14 TSHR	gactCTTTTCTGTTGCCTTGCAG	TGGACTTTTCCTTGTACCCAACAA
14 TSHR	CGTCATCGTCTGCTGCTGTTAT	TTCAGAATTGCTGACAGAGCATAGAA
14 TSHR	GAAGAGAATCTGGGTGACAGCA	GTCAAAAGCTTGTAGAGTCTCTTCCT
14 TSHR	CATATGCATGGCCCCAATCTCA	GGCCTTGGTGAAAATAGCATAGAGG
14 TSHR	GATCATTGGTTTTGGCCAGGAG	CAATTCTCAGGAACTTGTAGCCCATTAT
14 TSHR	TGAGTTCAACCCGTGTGAAGAC	TGTAGTGGCTGGTGAGGAGAATA
14 TSHR	ACTTAACTCCTGTGCCAATCCATT	GTGGGTAACCTTTTGAACCTGAATATCA
14 TSHR	GGGCAATGTCTTTGTCCTGCT	GCATGGTTGTAGTACTCAGAGTGA
14 TSHR	GGTTCCTCCAAAGAACAGCAC	TTCTGAGATTTGGCCTTGCTTCT
14 TSHR	GATTGAAAACTCCCATCTAACCCCAA	CATGGGATTGGAATGCATATTCAAGAAA
14 TSHR	GGAGAAATAGCCCCGAGTCC	GTTGAATATCCTTGCAGGTGACTCT
14 TSHR	GCCATCAGGAGGAGGACTTC	GTTATTGAGCTGCTTTTGTGCACTT
14 TSHR	GCACCACTTCTCACCAGTCAC	AGTTTTCATGGCTACCCTTGACTTAC
14 TSHR	ACCTGACAGTTATTGACAAAGATGCATT	TCTCCCCTTAATGTCTCCATTTATTCCa
14 TSHR	CCAACATATTGTGAAAACTGTCATGCT	TGGGCAGATTAGAAAATGCATGACT
14 TSHR	CTGAGAACTATTCCAAGTCATGCATTTT	ACTGCCATTGATTTATGCAAGTATAATACCA
14 TSHR	GAACAGATACGGATGCATTATAGTGACT	CAGAAAAGTAGGATGGGAGAGGATTTAA
14 TSHR	GCTCACTGCCTCTCTGCATTTT	AAGGAAACTCAAGGAAAGTGGAAGT
14 TSHR	CTCATCGCCTCTGTAGACCTCTA	GCGAAGGTGATGGCATACCAG
14 TSHR	GCAAGAAACACCTGGACTCTTAAGAA	CCAAGGTCTTTTGTCACTTATGCC
14 TSHR	TGACGGTCATCACCCTGGA	CAGATACTGACTTTGGCATAGCTACT
14 TSHR	CCTGCTTCCTTTGGTGGGAATA	ATTTCGGACTGTGATGTAGATCTTCAC
15 BLM	GGAAACGTGTGCCAGTGATTCT	GACTTTGGAAACGCTCATGTTTCA
15 BLM	GTCAGCACAAAATTTAGCATCCAGAAAT	GATAAAACAGTCTTCACCAAGCAGTG
15 BLM	GAACTAATCAGCTAGAGGCGATCAAT	GCTTTCATTTAACATCTGCCAATGGA
15 BLM	TGAGTCTAGCCTATAGTATGATTGGCTT	TGCATACTCATTTTCTCAGGTTTTGACA
15 BLM	CTGACAGAAAAGAGGATGTTCTTAGCA	GTCTAAAATATTGCTTGTACCGTCACAG
15 BLM	GCTGAATCCAGAAACCAGCACA	CCATTTTATAACATACAGCCACAGGTT

15 BLM	TCCGTAGGTTTTGGTCTCGGTA	ACAATACTTTTCACATCGTCAGTCACA
15 BLM	AAAACAAGAGATGTGACTGACGATGT	CAAGAAAATGTCGACCAGCATATTCAT
15 BLM	AGATGTGACTGACGATGTGAAAAGTATT	TCAATTGAAGTAACTCAAACATTCAAAACA
15 BLM	TTTGTGATCTATTCCATCTGTCCAAAC	TCGTGAATAAGCAGATCCTTTTCCAAAT
15 BLM	GTAAGAGTGCAAAAATCCAGTCAGGTAT	GGGCTTTATTTCCGAGCATCACA
15 BLM	AATGACCAGGCGATCGCTTA	GCTTGGACAAAGACACTATACTTCACAAT
15 BLM	TCTGTAACTAATGTGTCAGTAGCAAAAACA	AAGTCCTTGACCCTTTGCTGATT
15 BLM	GAACCTCTACCCAACACCACA	TGTGTTTTGGGTAGTGCATACAACT
15 BLM	GATTTCTTGCAGACTCCGAAGGA	CCATATCATCCCAATCATTGATGGTACT
15 BLM	CCGGGATACTGCTCTCAAGAAATTAG	ATTTCTGAGCAGTGCTTACTCTTACAA
15 BLM	GTTACACCACCCCAAAGTCACT	GTCATCCTTCTGTTCCTCAGTCAAAT
15 BLM	TTGTGATGGAATTTGAAGACCACAGA	GCTTCATCAATAACAAAACGTGCCA
15 BLM	GGAGAATCTCTATGAGAGGAAGCTCT	TCGTTCACTCAGTGTGGGTTTT
15 BLM	GCTCTGAAGACAGAACCTGACA	ACTTACATCCAAGGAAGTCAGCTTTT
15 BLM	CTTATCGTAGATCAAGTCCAAAAGCTGA	ACTGCAAATTTAACTGCTGTGCTTTATT
15 BLM	AAATGTTCCTTCAAGTCTGTGCCTTA	TGCCAGATTCTTGGTAGTAACCCT
15 BLM	CCTGTTCTTTCTGTCTCATTAGTGGTT	GAGAAGGTGGAACAAAATCCGTATCA
15 BLM	GCTTCTTCTTCCTCTTCAAAATGCCTTA	TGAGGCTTTCACTTGAATGTAAAATAATTG
15 BLM	CATGCATCTCTCCCTAAATCTGTGG	TCTCTACAATGGAGCCCAGCTTA
15 BLM	TTTCCTTTGATAGGTTTGATATGTGACTAATAA	TCTGAGTCAGTCTTATCACCTGTCA
15 BLM	ACAAGTGCACATATACCCACTCCTA	CAGACTTCTGTAAGTTCTCCAAGACATT
15 BLM	TGTCTCAGAGGGAAGAGATGGT	GGCCTATTAATCTGTGCCACGTA
15 BLM	TTTCTATGATATGCTCTATTTTTCCCCTATAAG	TCCTGCATTCCGTTATATTTTCACAGTA
15 BLM	GGAAACCATCATACAAGAGAAACTCACT	AGCAATTATCACAAGAAACATCTGGGT
15 BLM	TCCTGATTTTTGTAAGAAACACCCAGAT	GAGACCACCTTTTGCAATCTAATTTGTA
15 BLM	CCTGTAAACATCTGCATTTTCCATTTGT	GCATCTTTTTCCTCTTCCTTTCATTTCT
15 BLM	CTTCCCACTACTTTGCAAGTAAAACC	TCCTTTCTTCCCTTGTTCCCTATATTGA
15 BLM	GCCCAGGGAAGTGGTATTGTA	GTGAGGCTGAACTGGATCCAA
15 BLM	CAAAACGAAATCCTCCAGCATCA	GGGTCTATGTACATTGAGATTCGGTT
15 BLM	CTTAATACATTGAGCAGTGTTGGCTTT	CTTCTGGCGAAGCATATTCATTCTTT
15 BLM	GGGACATGATTTTCGTCAAGATTACA	AAATACGTGACGTGCAACAACTTAC
15 BLM	TTAGGTTTAGCATGAGCTTTAACAGACA	AGCTAATCACATGGCTGTACTCAC

15 BLM	TGCAAAGTACCTAACTCCACTGATTTC	ACAGCAAACCAGTCAAAACACTTAC
15 BLM	TGAATTCAGTGGGTTTTCTATGGG	TTTCCAGTTTGTCTTCAGTAACACCAT
15 BLM	TGATCCTGAGGTTTTGCTTCAAATTG	CGCGTCGCTTCACATTAAACAG
15 BLM	ACAGATAAGTTTACAGCAGCAGCTT	GATACCTTATGTTCCGCTGCTGA
15 BLM	GGATTGTGGGAACGAACTGCT	GGCCAGAAAGCCTGAACATTTT
15 BLM	CCTCCTCTGAAAGCGAGCAAATAG	GAGTCACTTTCCTGAGCATCTTCATTTA
15 BLM	GCCCCATTGCTGAAGTGCATA	CATGACTATTCCCAATGGCTAGCTTT
15 BLM	GCTTTTGTGGCCTACCAGAGT	CCCTCCATAGGGCCATCAAG
15 BLM	GATGATGATGATGACTGGGAAGACAT	TTTGAGCAGTAGATGACACTGGAAG
15 BLM	ACAGACTGTCTTCCAGTGTCATCT	GGCAATGATGATTTGCTATGGTTTTTCT
15 BLM	TGTGGAGATACAGGCCTGATTCA	CTTCCTGGTGGCAGAGAATCC
15 BLM	CTGACTACCACCCTAGGAAAGACA	AGAACATTTCCTGGGAAATAAGAGCTT
15 BLM	GGCTGAAACACCAAGACTAGGAAAAA	GAAGGTTGGGTTTCTCTTTCTAAGTCAT
15 BLM	CACTGCTGTGAAAGATCAGAATAAACAT	ATTTGCTGGCTGCTAAATTATGCATT
15 BLM	TCTTGTTTCTCAGTACTCTTGGTTTCTTG	ATCCACTTCTGCTGCACTTCAT
15 BLM	GCCTCAGTGATTCTGCCAGAG	TGCATTCTACATGTGCATGTTTGG
15 BUB1B	CAGCCTGCTCTTCTAATTTGTTTACTTT	GACTCAAAAACTTCCTCCTCTTCTTCT
15 BUB1B	CAAACTCTGTTGGCACTTGAGAA	AGAGCACCTCCTACACGGA
15 BUB1B	AAAGACAGCAAGAGCTCCAATCA	GCAAACTAGAAGTGTCCACAGGTATATT
15 BUB1B	TGTACCCTGTGTCACTGAGCTA	ACAAATTGTTACATTTCTGAAGCCTGTG
15 BUB1B	CCTTGAGCGAGGATGCCATTAT	AGAAGGGAGATCCTTCAAGGACATTA
15 BUB1B	TTTTAGCTGATGGTGCTTTTTGTGATTT	CAGAGACCCTGACATTCATCAATACTTA
15 BUB1B	CGTTTTGTATCCACTCCTTTTCATGAGA	GTAGATAGTGCCACAAGCTGTCTG
15 BUB1B	AGATGTAAAGACCTCTGAGGACCAG	AAGCACATGCAGAATAAGAGAGTAAACA
15 BUB1B	CAATGTCTTACAGGTATCTTCTCAACCT	GAAGCAGTTTATATATTGGTGCCAAACA
15 BUB1B	AGCTGTTATCAATATCAAGATGGCTGT	GCTAAATGGAGCACAAATCTCTCTACT
15 BUB1B	GAGATGAGCTCCAAAGGCAGTT	TGCTGAGTAGTTTGGATTTCTTTTAGCTT
15 BUB1B	GCAGAAACAGATTGAAGAGATGGAGAA	GGGTTGGGAGGACTTTAACCT
15 BUB1B	GAGTATAACTACGATCTGCCAAACTTGA	TCACCATGGACTATTTCTGCTTTGT
15 BUB1B	ACCTTTTGACAATAGTGGAGATGCTAC	TGTTTTAAGAGACAGCTGGCAGAATTAT
15 BUB1B	CAGTTCTTTCTGTGGGTATTGAGCA	TTCTCAAGGCTGTCAAATACAACTCA
15 BUB1B	ACACCAGTGCCTCTATCCCTTAA	CTTGGCGCCACCCAGAATA

15 BUB1B	ATTCTGGGTGGCGCCAA	AAAGATTTGATGGTAACATGAGGACAGT
15 BUB1B	CCTTCCTGCTTTCAAACAACACAA	AACTCATCAAAAATGGAGAAAGGTACACT
15 BUB1B	CAAGACCATGAATAATCACCTTTCGGA	CCTTGCCTTAAAGGTTGTACATTTTCTT
15 BUB1B	AGGGAGATGAATGGGAACTGAGTA	TTGAGACTCAGCCATTCTACACAC
15 BUB1B	ACAGTGCTGACTGTGTAATCTTGATT	GTCTGCGATACTGTGAACACCAT
15 BUB1B	GGCAGAAAACCCTACTCAGTCA	CCAGAGGACATGAGTTCAATGGTA
15 BUB1B	ATTGCTAAGTGAGGTATGTCTTTTTCCA	TTACACAAACATCTGGAGACACATCTT
15 BUB1B	CCTCAGAAAGCATCACCTCAAATG	gtgcttaATTCAAGTCAGTGGTTTCAA
15 BUB1B	GCTGTTGTCACTATTGCATATGCT	AAACATGGCCATAAGGACTTATCTGTT
15 BUB1B	CTGTTGTAGTGGAAATACTGTCCTTGA	AGGAAATGGATTTTGGAGTCCTCTG
15 BUB1B	CTCCTAGCTCCAAGCCAGAA	GTAGGCTTAGACAACTCTGCTGT
15 BUB1B	TGATGAAAATGCTGATGAGGCTTCT	CAAAGCACTGGATCTAAGAGTCCTTAC
15 BUB1B	AGCCATGGTCTATATATGCTGTCTGA	CCACATATGGAGTGAAACTGGGAAG
15 BUB1B	CTGATAGCTGTACCCGCTGTG	CTGCCTTTGGGAAGTAGAATAGGT
15 BUB1B	TTTAAAGACCAGCTATGCAGCTTCT	ACCCTAAGGTCAACACTGTAGGAAA
15 BUB1B	GGCAACTAATAGGCATTCAATACGTGA	GCTTCATATTCTTCTGCCCATGAGATAT
15 BUB1B	TGGTGTTTCACTTGCTCAGTTCT	AGAGTTAAGACAAGCTCCAAAGAAAGTTT
15 BUB1B	AACAATCAAGCTTTGAAGATAGTGGACT	CATATTTGCTCAGGCTTGTTTTACACTT
15 BUB1B	CCCAATTATTGAAGACAGTCGTGAAG	TTCACTAGGCATGGATATAGTGGTATGT
15 BUB1B	tgCAGAACATAATTATTGATGGCCCTT	GTGCTTAGGATGTGGTTTATACTAGGTT
15 BUB1B	AGTAATTTTGCTTCTTTAGGACACCATGT	CTTCTCCTCAGACGCTTGCT
15 BUB1B	GGAGATCCTCTACAAAGGGTTCAGA	AGCCCGAATTTCTTCAAAGGAGAATT
15 BUB1B	GGAGAAGATTTATGCAGGAGTAGGG	TCAGCTCAACCTTCACACAACT
15 BUB1B	CCTTGGTCGCTTCTGTAGCTC	AGCAGCTTTCTCCCGTACCTA
15 BUB1B	TTGTACTGTTAGTGGATGTCTAGGGAA	GCAATTTGCAGTTTAGTTGTCTCCTTTG
15 BUB1B	CAGCAAGAAGAGACGATGCCTA	TGTCCCTTCCACCTATGTAGTCTTAC
15 BUB1B	TGGTGCATAAATGTACCACTGAGTT	GCATTCAGAATCCGCACAAAGAATTT
15 BUB1B	AGGCTAAAAGATGGTGAATTGTGGAAT	TTCCATAAGGCTTTGTTCAGGTGA
15 BUB1B	GGGTTTTTGACACTACATTCCAAAG	CTGAGGCAGCAATCTGTGAGA
15 BUB1B	GGGTATGTCATTGAGGACCTCAAA	CCTTCCCACCTTGAGGATAGTTC
15 BUB1B	AGGTATATCAGCTGGACAGAGCA	TCAGATGGCACTTGAGAAAGACTTAC
15 CASC5	AAACCAACGTGTTATGAAACCATCA	TGACTGTCTGGTCATTTGCATGT

15 CASC5	AAGTTTAGTGATACGACACAAGATCGG	TAATTATTTTCTGCCTACCCGAGACTA
15 CASC5	CCCGTGAAAGGAAACATGCAA	TGATGTGGTATCTATCTTGGTGGACT
15 CASC5	AAGGCCTTTTAGATAATCCCATAAGTGA	AAGAAGTGTTTTGATCCACGGAAAAATT
15 CASC5	GTCCCATACTGTTTTCATTGACTACCA	CCTGGAAAGAAAACACTCTCCTCAG
15 CASC5	CACACCGAGGACTCAAGAATGA	GCACATCAGGAAAAGCACTACATTT
15 CASC5	CGTGGATCAAAACACTTCTTCAGAAAAT	TCGGTTCCTTGGAATAAATAGGTATCTCA
15 CASC5	TGCTTTTCCTGATGTGCCTGATAAA	GGTGAAATTCATCCCATCATCTTTTTCT
15 CASC5	GAACACCAACAGTGATATGTACTCCTA	CCTCTACAGGGCCCCTATTATTAGA
15 CASC5	GCCTCTTCTACACATCAAATGCATGTAT	CCTTTAGATTCCCGTGAGTTGGT
15 CASC5	CTAACCCCTCTGGAGGAATGG	CTCTGACTTTTGCTTGATAGAGGTTCAT
15 CASC5	AAAGATGTACAAAGTCCTGGATTTCTGA	CTCCAGGGCACTTTCGTTATCTAT
15 CASC5	GATATTACCCAGAGTTGTATGGTGGAA	TCCTAGTGATCTCCATGTCATCCTG
15 CASC5	CTGGAGGATAAAGAGGACTTCCATTTG	CGGTTTTGTCCATGGGCCTAATA
15 CASC5	CACACAACTGCCTTAGAATGTAAAACTC	GTGGCTTGACAGTCAATGAAAACA
15 CASC5	TCTGGAAGTCACCGATTCCCATA	TTTCCTACAGCCAGTGCTTGT
15 CASC5	GCTGTGTTCAAGAAATCGCTGAAAA	GCCTGGTCTACAACTTTCCCTAT
15 CASC5	AGTGCTGCTATGGATGAAAAGGTC	AGCAGTTGCATGACTACTTGTAAAGT
15 CASC5	CGCAAGTTGAAAGCTGTCAGTTAAATAA	ACTACCCTCCAAATTATCAGTACAGGAAA
15 CASC5	CTGTTGGTACTGTTTGGGTGGTA	AGCTTGATCATCACTCCACTCAAC
15 CASC5	AGCTTGTCTGAGTGGGATGTC	AGTGAGTGTAGCCATTCATTTTTCTGT
15 CASC5	GAAATCACATTGAATGGTCATGCAAAC	ACATTTTGCCAAGAAACTGCACTTAC
15 CASC5	GTGCTAAGAGAAAGTGATGACCAGTT	TTTCTTACTGAAGAATGCCGTCTTCT
15 CASC5	GAGACCTGTTAGAAGACGGCAT	CCAGTAAGGGTAGGATGGTGAAG
15 CASC5	CTTTTATTCTCTAGCTCCGCAGCA	CTTACCCTGTTGGCCCTTTACTC
15 CASC5	GTGAGCCACTAAGCATTGAAATGTT	TGGTCCCCATCTCTTTAAATACTCAATCT
15 CASC5	TCCCAATGGGTAGCTTCAGGAT	CTCAAGCTCTCTTCACAAATAGTATCTCT
15 CASC5	CAGTAGTCAAATGGAATCACAGTTTCTC	ACGTGTATTAGAAATGAGACGTTTAAACCT
15 CASC5	CTTTGGTCTTCCCTCAGAAAGTAAC	AGCCTCTGAAGACACCCCAT
15 CASC5	GGGTGTCTTCAGAGGCTAATGA	TGGAACACAATGGAAAAAGATCTCAGAA
15 CASC5	AGATTAAAGCTTTCTGCATCGAACCA	TCGTTCAGAATGTCTTCACTGATTCTC
15 CASC5	GGTTTTGTCTGCAAGATACCAGTTC	ACGAGCCTCACAATCTTCTCTATAAATCT
15 CASC5	AAGTCAATATGTTTACCGACCCAAGATAC	AACTATTAACTGATCCCTCCCCTCAA

15 CASC5	CAGAATGAATTTGAGGAGTTTCAGAGTT	CCACCTCTGAGGTCCTGAAGA
15 CASC5	CTAGATATTGAAACCCCCAAGGAGTC	GAAACTCATGGGATGCTTACACATTT
15 CASC5	AGGAGTATATGGCATAGCAAAGAGGAA	CACTATTTTCATATGAGACTCCGTCTGG
15 CASC5	CTAGCCTTCCTAGACCATTTACCTC	GCTTGCCATACAGAGCCACTTT
15 CASC5	TGACTAAAGTCTTCACTCACCAAGGA	GCCCAAGTCTTATTCACATGTCTTCTAA
15 CASC5	GGAAGCCCTTCTGTTCTGAGTT	TGACCTTTGAAGCTCCTCTTCTTC
15 CASC5	CCAAAGAATTGGAACAGCTGAAAACT	TCCCCAAATCCTGTCAGTCTCT
15 CASC5	CTGTTAGTGTTTGTCGTAAGTATACAGTGT	AGAGAGCTGCTGCAACTAGATG
15 CASC5	CAGATTTTTGATCACCATACTGAAGAGGA	AGAGGTCCCATCAGCCTTGA
15 CASC5	GCAGCAGCTCTCTGGATTCAA	CCCCAAAAAGGCATATACAACATTCAAA
15 CASC5	AAGAACCTAAGAAAAGGAAAATCAGGCT	TCCTTCTGTTGCATCTGGGTATG
15 CASC5	CACATTGCTTTCTGCTCCCATT	GCCTTTCCAGTTTATGCTGATTCATTTT
15 CASC5	ATACTGTCTACTTTAGAAACTAAGAATTTGGAG	CCCACACCCATTTATCTCCTTAACTAAT
15 CASC5	GCAGAAATCTCTTAGAACTGGAGGT	ACACAAACAAATGATGCCTGCATT
15 CASC5	GGATCTAACAAAGAGCCACACTGT	TTACAGCTATCTGTCTGTTCATTGTTGT
15 CASC5	ACATTGATTCCCACATCCAGTGAG	TCCATGGCATTCTGAGTTTGATTTTCTA
15 CASC5	CATGGAAATGACCAAATGTCTCTCAAAT	CCTCTCCGGAATAAATAGTTTTCTCTGT
15 CASC5	CTCAGCCTCATTTCTCAACCGA	CCTCACTATTGAGTATTTTGGGCTTGTT
15 CASC5	GCAATCCAGATGCTATGTCTTCTCT	TGCTATTTGCACATTTGATTGATCTTGTT
15 CASC5	TCGTAATGAACCATTTCAGCGATCA	GGCTTGTTGCTATCTCCTGATTACA
15 CASC5	GAACACACTACTGGCCAGCTA	TTTAGGTTTCTGTACACTTTCGTCCTC
15 CASC5	TTTAACCACACTTTGCAGATCTTACCT	GGAAAGGTCTTGAAAAGCAGTATTTTGT
15 CASC5	AAATTCCTCTGCACCCATATGTGAA	TGAGTTACTGTGGAAATCTAGAGCTGTAT
15 CASC5	ATGGACATTACCAAGAGTCATACAGTTG	ATTTTCCCATCTTCTGATGTGGTCATAA
15 CASC5	TGATAAAGATTCTCCTCAGTCAGCTGAT	CCTGATGAAAATAATGGCTTTGTGGTAG
15 CASC5	CCCGAAAAAGAAATGATGCTCCAA	AAAGGATTTGACAGGCTCTGCT
15 CASC5	TGCTGTTTGTGGATCCAGTGATAATT	TGGCAAAATCATTTGCATAAGCAAGA
15 CASC5	ACTGTGGTGGAGTTCTTGATAAACAAAT	CTGGCCTAGTACAGATTTCACTGTATG
15 CASC5	AGGTTATGGAACTAAAGCTTCAGGAAAT	CTGGCATCCTGATTACAAGTCTGT
15 CASC5	CTAAGGAAAGAATACAGCAGAGCCT	TGGTACTCTCCGGTGCTAGATT
15 CASC5	GGGAAGGAAAAGTGTTGGTGGA	TCCTGCCAATGGCACCAAA
15 CASC5	GGTTTGCTGCAGCCTGTAAAAA	CAGCATTGACATGAGTTTGAATGACT

15 CASC5	GGGCAGAAACTCACATAGTCTC	GGTCATTTCCATGGCATCATTACAACTA
15 CASC5	GCCATTATTTTCATCAGGACAGTTCTCT	TCATCTTGTCATGGCAATAGTCAGG
15 CASC5	CGTGATTGTCATTTGGTGCCAT	GGCCTAGTAGTTATTTCATCTGGTGAGA
15 CASC5	CACAGTAACTCAGACGTAACTAAGCA	TGGTAGATGAACAGTTTGAAAGGCTAAA
15 CASC5	CCAATTATTGCCCAGTGCAAAATGA	TGTCCTTTGGTACTGGTTTGAATAACTT
15 CASC5	AGAATATTAGCCATGACCCCAGAATCTA	TGCTTTAGCAAATTTTTCTCCTCTCTCA
15 CASC5	GGAGAAGCACCAGATCCTGTAATT	CATTGTGTGCCTTATTTCCTAATTCAAGT
15 CASC5	GTCAGGTTCCTCTTGCAGCTTAT	CGGTTAAGCTGTTTTTGGCTATTATGTC
15 CASC5	CTCTGCTCCTTGTCCTTTGTTAGA	CATCTCCCCCAGATCTTGACTATATACT
15 CASC5	CCACCCCTTCCAGAGCAATT	TGGTTTCAGCTCCATTATGAGACTTTTT
15 CASC5	TGAGGAAAAGTTTAAGCAATCCCACA	AGTTCAGAAGGACCAAATCCAATGAC
15 CASC5	CCACGAAATCTATTGGCTAATCAAACTTT	AATGGAGGTTGGTCGGTTGAG
15 CASC5	TCTCCAGCAATGCTAAAGATAGTAGAGA	TGTTGGGCAATCTAGGCAAAAAGA
15 CASC5	CTCTCTACCGCCAAAGACAGTTTT	AGTTGTGTCTGCTGGAATCTGTTC
15 CASC5	CACACAACTGCCTTAGAATGTAAAACTG	GTTAGGTCTGTCTGTTCTTTCCTTTTCT
15 CASC5	TGTAGTGTCACTGGTATTGATGACCT	TTCAGTTTACCTGCAACAGATCCAA
15 CASC5	GGTCTCTAGCAAAGATTCAGGCA	AAGGCCTTTTCCTCAGTTTCTATGTTAA
15 CASC5	TCCTGATGAGATCAATTCTTCAGACTCT	TTCTTCTTCTTGTACCCACGTTCTTT
15 CASC5	AATGGGAAAAACTTGCAATAGCCAAA	GGTTCAAGACAGAGCTCACCT
15 CASC5	GGACTTGATCTCTGTCTATAGTACTCTGG	AAAGCTGACTCGACGAGAGTTT
15 CASC5	CCCTTAGGAATCCAATGCTTTGAGAAAT	TTGGTTTGATACACAAAATACCAGTACCT
15 CASC5	CGATTATGTGAGCAGTTTGAACCA	CTTTACCTGTTTCCATCTCAGTGAGG
15 CASC5	AAATACTTAAGAAGATCGATAACTGCCTCAC	TCGTGGTACCAACATTCTCTACTGTA
15 CASC5	TGATTAACCTTTGTTCTTTCCAGCCA	GGTTGTTCCAATGGTGGTCCAA
15 IDH2	CTCAGCCCAGTGGGCTTTA	CCCTGATGGGAAGACGATTGAG
15 IDH2	CTTCTGGTGCTCCCGATAGT	GCCCTGAGAGAAAGGCTTTCTA
15 IDH2	GAGATGAGTGACATGGCCAACT	GCCACCCAGAAGTACAGTGT
15 IDH2	TGATGGTGGCACACTTGACAG	CGTGGACATCCAGCTAAAGTATTTTGA
15 IDH2	ATCAGTCTGGTCACGGTTTGG	CCATGGCCTGCAGACTTCTTAT
15 IDH2	GCAATGAGTCCTGCTCCACTA	TGTGGGCCTGCAAGAACTATG
15 IDH2	GTCTGACTGCACATCTCCGT	CTCTCCCCATAACAGACCTTTTTACTC
15 IDH2	CCCTGTGGGACAGAACAATCC	ACAAAAGGATCAAGGTGGCGAA
15 IDH2	GGTCATCTCATCACCATCCATCT	GCTTTCTGCCACTGTCTCCTG
15 IDH2	CCCAGGGTCTGCCTACCA	CATTCTGCCCCATCCCCATAG
15 IDH2	TCCACTGCAGCCATGGG	TGATGTGAGTGGTGCTCTCTCT

15	IDH2	CCTCGGAGCTGAGCCAAATG	CCAAGAACACCATACTGAAAGCCT
15	IDH2	TCCTTGAAACGCCCATCGT	CGGCAATCGCCAGGCTA
15	IDH2	CCAAGCCAGCCTCACCTC	GCAGTACAAGGCCACAGACTTT
15	IDH2	GTTGTACACTTCCCACTCCTTGA	GCCTAGCCATCCTCTTGTCTCT
15	IDH2	GCCTCTCCCTCCATGCTCA	GGCTCTTGATCTCCCTGCAA
15	IDH2	GGCTGCTCTTGCGAGGT	CTCGTTCGCTCTCCAGCTT
15	IDH2	CAGGTCAGTGGATCCCCTCT	CAATGGAACTATCCGGAACATCCT
15	IDH2	CGTGGGATGTTTTTGCAGATGATG	CTGCAGTGGGACCACTATTATCT
15	IGF1R	CTGTTCTGATACCGTGTGAGAGAG	TGAAGTACTCCGGGTTCACAGA
15	IGF1R	GGGAATGGAGTGCTGTATGCC	GTAGCTCAAAATAATGCAAACCTCCTT
15	IGF1R	ATTGTTCAGTCCATCCCTTTCCAA	CTCCATGACGAAGCGAAGGA
15	IGF1R	GGGCTTGTCCAACGAGCAA	GTATTTGAGAAGAGAACTCAGAAGAGCA
15	IGF1R	TTACACCAAGTGAGCACACAGT	CCTAGGATGAGGCGAAGGTTTTT
15	IGF1R	CCTTGGTCTCCTTGTCCTTCCTA	CTCAGCAACACAGTTTCATAAGCAC
15	IGF1R	CCTGGGAACCCAAATCCAACT	TCAGGTTCCGGCCACTTTAAAA
15	IGF1R	CCAAGGCCTGAAAACTCCATCT	CATATGCTGTCAATGGATGGAAGTACAT
15	IGF1R	CCTGACCCTCTGAGTCTTTCTCTT	CTCCATTTCTGGCCTCAGAGAC
15	IGF1R	GGGCGATCTCAAAAGTTATCTCC	AGAAACACTGGATGCTGGAAAGT
15	IGF1R	ACAGGATTCCTGAAAACCAACTGTA	CTCGATCACCGTGCAGTTCT
15	IGF1R	CAACGACTATCAGCAGCTGAAG	CACTCGGAACAGCAGCAAGTA
15	IGF1R	CAAGCTCACGGTCATTACCGA	AAGATGACCAGGGCGTAGTTG
15	IGF1R	CGCGGCTGGAAACTCTTCTA	GGATCAGGGACCAGTCCACA
15	IGF1R	TGTTTCTGTACCTGCTTTAATTACGGT	GGCCACTCTGGTTTCAGGTT
15	IGF1R	CTGACCTCTGTTACCTCTCCAC	TGTACTCATTGTTGATGGTGGTCTTC
15	IGF1R	CCAAGCAAGACAGGTGCTTTT	GGTACCGGTGCCAGGTTATG
15	IGF1R	CCAAGGGTGTGGTGAAAGATG	CATTTTGGCTTTTCAGGAACTTTCTCTTA
15	IGF1R	CCACGTCGAAGAATCGCATCAT	CAACAGAATGGCATCACCCAAAG
15	IGF1R	CGGTCTCATCTCCGTCTCTCC	ACAGACACCGGCATAGTAGTAGT
15	IGF1R	CAACGACACGGCCTGTGTA	GATCACAAACCCCTCGGAGTC
15	IGF1R	ACTTCTGCGCCAACATCCT	GGCGGGTAGTGACCACAC
15	IGF1R	GCCCCGAACTTTCTCTGAACTTA	CAGGGCTTCAGCCCATGTAG
15	IGF1R	GTGGAGCCCGGCATCTTA	GTGCGAATGTACAAGATCTCACTCTT
15	IGF1R	CCATGGTGGAGAACGACCA	GCTCACTGCTAACCCTACAATGA
15	IGF1R	TGCAAGAAGACAGACTCAATTATGTGT	TGAAGATGGTGCATCCTTGGAG
15	IGF1R	CCATTGATTCTGTTACTTCTGCTCAGAT	CGGGAGCATGAAAAGCATCAAC
15	IGF1R	CTGATAGCCTGACTCTTAAGTTCATTTCA	GCGCACAATGTAGTAACTCAGGTT

15 IGF1R	CAGGTGCGCTAACATCGATT	ATGCGGTAAATTTCGGAAACACATAATT
15 IGF1R	TGAAGTGGAACCCTCCCTCTC	GCACATTACAACCACAGGAATGAA
15 IGF1R	AGCAGGGAAAATGTACTTTGCTT	ACGGTGTTTTGGATGCTGTCA
15 IGF1R	AGTATTGTTTCCTTCGCCCTTGT	CCTCCTCCGGAGCCAGA
15 IGF1R	CATCCCAAATAAAAGGAATGAAGTCTGG	CGGCGGGCACATACTCA
15 IGF1R	CTTAAAAGCCACATTTCTCTCCTCCT	GGTACTCTGTCTCCAGCTCTTC
15 IGF1R	CGCAGACACCTACAACATCAC	GCTGTGGATATCGATGCGGTA
15 IGF1R	CTAACCTTCGGCCTTTCACATTG	GGCCAGCTGGATCATACCATA
15 IGF1R	GAGGAGAAGCCGATGTGTGA	CGGAACTCGTGCACTCagaag
15 IGF1R	CGGTGCCCAGATTGAACAAAGAT	CATGCCGTCTGCAATCTCTC
15 IGF1R	GCCTGAGCAAGATGATTCAGATGG	CCCAGACCTGGAAAGCTAAGG
15 IGF1R	TTCCTTACAAGCATGTATAACGGCTT	CCACCACACACCTCAGTCTTG
15 IGF1R	CATTGAGGAGGTCACAGAGAACC	ATGGAGTTGTGCAGGAAATTCTCA
15 IGF1R	AGGCTGAATACCGCAAAGTCTT	GCTGCTGACTTACACACTACCTG
15 IGF1R	CGTACGCTTGTATGCGGGAAA	CAGGAAGGAAGGCCTCATCTTG
15 IGF1R	GGTTTGAACTGATGCGCATGTG	CAGCTTGTTCTCCTCGCTGTA
15 IGF1R	TTCCGGGAGGTCTCCTTCTAC	GTTCTCGGCCTTGTGTCCT
15 IGF1R	ACTGCCCGACAGACACTC	AAGGCCCGCTCGTTCTT
15 IGF1R	CTTACGCCCACATGAACGG	AGGAGGCTTGTGAATGGATTGTT
15 IGF1R	CTGCATTCATGGGAAATTGACATGTAT	GATCTGTCCACGACCCATTCC
15 IGF1R	GATTCAGGCCACATCTCTCTCTG	ACAGAAAATAAGACAGCCATGAACACT
15 IGF1R	TGAGCAAATTGTTCACCTGGTGATAT	CTTTGCACATCACTGACCTCTTTCTA
15 IGF1R	GGTGATTATGCTGTACGTCTTCCA	GAAGAACATTTGATGGGAAAATCAGGTT
15 IGF1R	GCCACCTTAAAGTGGGACGTG	CTCATAGATATCTCGCGTCATACCAAAA
15 IGF1R	CGCCCTCTCTTCCCTTACAGA	GGCAATGCAGTAAAGGTTCTCATAC
15 LTK	CCTTGGGCAAAGGTAGGACAT	ATCCGAAGGCACCTCAACTG
15 LTK	CCATTGGCAGTCTCTCAAAGGG	cccagccAAAGGCATGTATTTACTA
15 LTK	GGTCTGGTATGGTGAGCGAAA	GGAGACTTTTCTGCGGTCCTC
15 LTK	GGAGGCTGGCTCCAAGATACTA	GCCTTCTCCCAACACCTTTCA
15 LTK	GCCTCTGGAGGAACCACTGA	CTTTTGTTGCCGGGTGGAC
15 LTK	CCTCGCTGTGCTGGTGAC	GGGAGTCGCTGTACATCCT
15 LTK	ACAGGCGTCCTCTCCCT	CGTTCCCCGCAGGATCTC
15 LTK	GGAGAAGATTGCTGAGACGAAGAC	CCTCCTCTGTCGCCAACTT
15 LTK	CCAAAAGACCTGCATCACAAGT	GGCATCTTCACATCCAAGACAGAT
15 LTK	GGAAGGGTGTTATCACCAGGA	CCTTGGTGGTGAACAACTCATCATT
15 LTK	TGCGGTAATAACTGGCCCTAC	GACTTTGGGATGGCACGAGATA
15 LTK	CCGAGCGGTTCTCCAGT	GGGCGCTTCCTGGAACA
15 LTK	GCACGTACCCGGAAAACGTA	GGAGAGCCAGCTCGTCTG
15 LTK	CTCTTCAACGGCTCGAGACT	AGCGTGCTTTCCTCGGT

15 LTK	GGATTCAGAGTGCATAGGTTCTCA	CGAACCTCTGCCATCAGGAC
15 LTK	CCCACCTGGCAATAATAGGGATT	TGGAGGGTGTGCCGTTATTC
15 LTK	TCCATTGGCAGGAGTGAATTCAG	CCCGACCTAATCTAACATCCTGGT
15 LTK	CCCACAAGACCAGGATGTTAGATT	GGCATGAACTCCTGCCTCTC
15 LTK	GGAGAGGAGCAGCCTTGAATC	GGAGGTGTCTCTCTTTCTCCCTA
15 LTK	CAGCCTCAGGGTGAAGAGAAAC	TCAGAAACTGTGCCCTTTTCCT
15 LTK	GCTACCTCCCCAGCTTTTCAA	CCATTACAGGACCCGGATGTG
15 LTK	AGTCCCGCACCGGTAGA	CAAGAACTTCTAGATTCTGGCAGTGA T
15 LTK	TGAGCCAGGACTGCTCCTAA	GAGACCTACATGGACTGATGCC
15 LTK	CCAGAAGTCCCACTCCTCTCA	ACTCCCTTCCAACGTGTCTTC
15 LTK	TGGTTGTGAAGGACCTCCCT	GGAGATCTTCTCACTGGGCTACAT
15 LTK	GTTGGTGCGCCCAGGATA	CCTGAGGCTAATCCTGTCCTTTC
15 LTK	AGTCCATGTAGGTCTCAGACACA	CCTCATTCTGCTGGAACTGATGTC
15 LTK	GAAACTCTTCATGTCCCCTCCA	CCTCCTGGTAACGGGATATGTC
15 LTK	CTCAGGGACTGCGGAAGAAG	CTGACAACCTCTGGGCTGAT
15 LTK	GGGTGTATGAAGGATACTCCATCTTC	GGGATTTCCACCGGGAGATT
15 LTK	GGGTGAAACCTCCAACTAGCA	CCCACACAGACACAATGTGAC
15 LTK	ACAGCTGCACGCCTCTC	GGGTCTTGGCTGTTTTCTACCT
15 LTK	CGTCACATTGTGTCTGTGTGG	CTTTAGACGCCTCCTTCCTCTT
15 LTK	TGCCTCAGTCCTTACCCTCA	CCAGGAACTGAGTCCAGAGAAG
15 MAP2K1	CAAGGAGCCAGGCATTTTTCTT	GTATACGCTTCCAGAGAACAAGGAG
15 MAP2K1	GACTATATCTTTCATCCCTTCCTCCCT	CACCATAGAAGCCCACGATGT
15 MAP2K1	GCATGAGTGCAACTCTCCGT	CCCAACTCTTAAGGCCATTGCT
15 MAP2K1	TGCCAGGCAACAGCTCTTAC	AGATGACTGACAGAGAAGAACAAAT GAAT
15 MAP2K1	CGGAGTTGGAAGCGCGTTA	CGCCCGCCCCATACTTAC
15 MAP2K1	GTCTGTGTGGAATGCTGATCCTT	CCAAAGTCACAGAGCTTGATCTCC
15 MAP2K1	CCTCCAACATCCTAGTCAACTCC	ACCGTACAAGAAGCTCAAGCA
15 MAP2K1	GGGTCCAAGTTAGGTTAGGTGAT	ATCTGGAGGAGGGATGGGATAC
15 MAP2K1	GCATGGGACTGTCTCTGGTAGA	GGACACACCAGGCTACTCAC
15 MAP2K1	GGAGTACTTCTTTGGGTTGACTTCT	TCAGTTCTCCCACCTTCTGCT
15 MAP2K1	CTTGAGGCCTTTCTTACCCAGA	CAATTACCTGTTAATCAAGGCAAACT CA
15 MAP2K1	GGAGGAAGGCAAATTTGTGATGATGA TA	GATCTTGTGCTTCTCCCTCAGATATG
15 MAP2K1	CTTTCCTCTAGGTAATAAAAGGCCTG A	GGAAGCAACAGCCTTTGGATTATATC TA
15 MAP2K1	agccACAGCCGAAAGTTATCA	GTTCAGGAATTCTTCCAGCTTTCTTC
15 MAP2K1	GGAGGTTCTCTGGATCAAGTCCT	CATCTTCCTCTCTTGTCCATTTCTTAC C
15 MAP2K1	CCCAACCCCTTGCCTCATATT	AGTAAATTCCAAGGTGAAGGAAACCA G
15 MAP2K1	TTTTAACACCACGTCCTCTCGTT	GCTCTTTGTTGCTTCCCAAACAC

15 NTRK3	CCCCATGACGCCCTTGAAAA	CTCTCTGTTTCTGGTTCCTCACAG
15 NTRK3	AGAACCCCAGGTACATGGTCTA	CGACACTGTGGTCATTGGCAT
15 NTRK3	GTTCTCAATGACAGGGATGCGA	CAGGTCCTGCGTCTCTCTTG
15 NTRK3	GTGTGTATATGTGTGTGCCAGAGT	GACTTCGGCATGTCCAGAGAT
15 NTRK3	CCCTGTAATAATCCGTGCTGTAGAC	GCTCCACATTGCCAGTCAGAT
15 NTRK3	GGGAGGCCAGGTACACCATA	CTCTCTTCTCCCCATGCTCTTG
15 NTRK3	TGAATTCATGACCACCAGCCA	CCCCAAAGAGGTGTACGATGT
15 NTRK3	GATGTTCAACCGCTGCTGTG	AAGGCTGAGCTTTGGGTGTAA
15 NTRK3	GCTGGCTCTAAATCCCACCTAA	GATCCTCTGGGAGATCTTCACCT
15 NTRK3	GGAACCATGGCTGCTTTCCAT	GTGCCTCTATCTCAACCCCTTTTAG
15 NTRK3	CCCATTACCAGCACTTCAGTACC	GAGCATTATTTGTGCCAGACTTGTTTTA
15 NTRK3	TCTCTGTGGCCGGGTGTA	ctgaccGTTACCTTTCCTCCTC
15 NTRK3	GCGGCCGCCTGACTTAC	GCAAATTGTGTCTGCAGCAAGA
15 NTRK3	CCGCCGGCAATTGATCTCA	GCGATCGGAGATGGATGTCT
15 NTRK3	CACTTGGCTGGGCAAAGAG	CTCGGCGTTTCAAAGGCA
15 NTRK3	CCAAAGAGAACAATGCCTAGAGCTT	CCGGACATTCCAAGCCTCTTA
15 NTRK3	CAAGACCGCAAATTTTCCCTCTT	CTGGATCACCCCTTCCTGATG
15 NTRK3	GCCCAGTGACTATCCAGTCCA	TGCCTGTCTTTTCACCCACTTT
15 NTRK3	GGAAAAGTTAGCAACACAAATGAAGTCT	CCTCCTCCTTTTTGTGTTTGGTTTT
15 NTRK3	CAAGGGTTCGGTGGGACT	GCAGCATGAGCACATTGTCA
15 NTRK3	GCCGCACACTCCATAGAACT	CCTGTGTCCTGCATTGTTCCTA
15 NTRK3	CACAGCCATCCCCCACAATAA	TCAACTTGACGCTGGTGAATGT
15 NTRK3	AAGCCATTGTCCTCACTCGTC	GCCATCCTTGTAGAAGCTATCTGATT
15 NTRK3	CCCATCTCCCAAGCTTGTAGA	CACTACAACAATGGCAACTATACCCT
15 NTRK3	CCCAGTGGGTTTTTGGCAATG	CACTGGCTGCACAATGGG
15 NTRK3	GTAGTATTCCACATGGATGATCTTGGA	GCAGGCTTCTCCCTTGACAG
15 NTRK3	ccCAAGAGTACCTGCCATGTG	CTGCTGATCCTCTTTTTCTCTCTGT
15 NTRK3	GCTCTGGCATCCCAAGGTAA	AGCTCAACAGCCAGAACCTC
15 NTRK3	GCCATCAGCGTTGATGCAGTA	GCCATCTTCGGTTCAGAGGTT
15 NTRK3	CATCCTCAACATAGATGCCATGGT	CCCCTCCTAGTTTTGATCTTCTTTGG
15 NTRK3	TGAGAAAGCCAATTGAGCACTATCTT	GCCGAGTGCTACAACCTCAG
15 NTRK3	CCACAAGCATCTTGTCCTTGGT	CCTCCTCTTTGTCCCCATCCTA
15 NTRK3	CAGTTTCAAAGCAACAGGTAAAGCA	AGGACCAAGTAGTGTGTCTGTTCT
15 PML	CCAATGCCCAGGAACATCCT	ATGTCAAGCTGGGAAGCCAT
15 PML	CCCAGTGGCTCAACAACTTTTT	CAGAGGCACTTGGACTTGAGAG
15 PML	GGACTCTGTCAGAGGCTCCAT	TGTTGGCTGCAAGGTTACAAGA
15 PML	GCCTCTGAGAGTGCTACCCTT	GCATAGCCATTCATTCGTTGattcattc
15 PML	CACAAGTCCCTGGCAGTCA	TCCCCTCCTCAGACTCCATC
15 PML	GCCCCAGGAAGGTCATCAA	CTGTTGGGCAGGAAGACCTC
15 PML	GGAGCCCCGTCATAGGAAGT	TGGCTTTGGGCTTTCTGGAT
15 PML	GGCCTGCAAGGATTCCCATAG	CTGAACTTCTGGGCCACTCA

15 PML	GGAAGACTCAGATGCCGAAAACT	TGTCCTCAGCACAGCTTGG
15 PML	CTATCACTGTCCCAGGTCAGGA	CTTCAGACCTCAAACCCATCTCA
15 PML	CACTCCTCGCCAGTCCA	CGCTGTGAGGCTCAGCAT
15 PML	CCTGCCCTGTGGCACATA	CGAAGGCGGATGGCTCTC
15 PML	CTGACGCTTGGTTTTTCTGTGT	GAGGTCTGTCTTCTGCTTGGG
15 PML	GGACGAGAACCTTGCTGACC	GCACTTCTTTTTGGGACTCAGAGA
15 PML	GCTTCTCTTCACGCACTCCAA	gCTGGCGGCCTTCAGAG
15 PML	GGTCCATGGAGCCTGCAC	GCTTAACCCCATCATCCCCTAAC
15 PML	GGCCTCACCTGCCTCTCT	GCATCCTGAGCACAGCGT
15 PML	CGAAGCTGCTGCCTTGTC	CCGCTGCAGACTCTCGAA
15 PML	CCGCCCTGGATAACGTCTTT	GTGTGCCTCGAAGCACTTG
15 PML	TCTGGTGCTTTGAGTGCGA	GGAGCAGAAGATGTTGTTGGTCTT
15 PML	GGTGCGTGAGTTCCTGGAC	GCCCTCTACCTGGTACTTGGAT
15 PML	GCTACCTGTTGTACAGAGCACA	GCATTGGCAGGATGGTTGAG
15 PML	GTCTCCCCATGTGGTCCAA	CGCACAGCCCCTCGTATC
15 PML	GCTACGCCTCGGACCAG	CCCCTGGGTGATGCAAGAG
15 PML	CGTTTTAAGAGGAAAAAGTCAGGAGA GT	CTGGATCTCTGCGCTGATGT
15 PML	GCTTCGACGAGTTCAAGGTG	CCAGTTTTCTTTTCTGTCAAATGGAGA T
15 PML	GCAGCCACAGTGAGCTCAA	GTGCATCTGCGCGTGAA
15 PML	CAGGAGCAGGATAGTGCCTTT	GTCCACAGCCTCCAGCAG
15 PML	GCGCCAGGTGGTAGCTC	GCTGCAGCACAGCATCCA
15 PML	GCGCGACTACGAGGAGATG	AGGAAACCGTGCATGTCCAG
15 PML	GCTGGCCCAGCATGTCTAC	GTGTGCACTCAGCAGCTC
15 PML	TCCTCACCCTGCCCTTCT	GAGCTCAGAAAGCTGAGGAAGT
15 PML	CATCTACTCCAAGGCCGTGTC	GGAATTCCCACAGCCTGTTAATGT
15 PML	CCCTACACAACGTGAGCTTCAT	CCTTCAAAGTAGGTGCCCAGAG
15 PML	CCCAGCCTGCTTTCAACCT	CCAGAGACTCCAAGCTGGTC
15 PML	CCTGGCCTCCCAAACTTCTTC	GCTCATGTTTCTCGCCAGGTA
15 PML	CTCAAGAACCTGGCCCAGAC	AAAGCACTGCAGGCTACTGAA
15 PML	GCTGCTGCCTAGTCATTTCTGA	TTGATGGAGAAGGCGTACACTG
15 PML	CTATGGCTGTGGTACAGTCAGT	GCTCACTTTCCTGGTGGAACTC
15 TCF12	GTATGTGCTGTTGTCACATGTTGAT	CAAGTAGTCCCACCAGTTAGAAGTAG A
15 TCF12	CCTCCATCATAACCCTGTAACTGCTA	TGTAACAACAGTTCCAGACTGACTTT G
15 TCF12	TTTGCCTCTTTGTTAGGTGGCT	TCTGCCTCTAGATGAAACCTTGATAT CT
15 TCF12	GAAGTCAGATGATGAATCCTCCCAAA A	CAGCTTCTCAAGTATGTTTACCTATGG A
15 TCF12	TGCTCTTTAGCTGTAACTTGTAAATGC T	GTGTTTATGTCTGTTGGTGAAACTGA
15 TCF12	CTCTTTCCATCTAGAGTTATCCTCCAC A	TGTGTGAGGCAGCAACGTAA
15 TCF12	GGCAGTACCAGCAGTTCACC	GGGTCACTGAAAGTCCACAAACTT
15 TCF12	GGAATGAAGTTACACAAAACAACAG CAA	CTTCTCTTAAGGCAGGCTGCT
15 TCF12	GTTTTAGAGAGGAACCTTAACCCCAA	CCGTACTTACCTGGCTGATGT

15	TCF12	CTCTGCCCTAGGACCTGCTA	AGATGCCATGGAAAGCTCTCATAC
15	TCF12	CCATGAATAGTCTAGCAGTTTGATTGTCT	AATCATTAAACTTACTTGACAGCCTGGT
15	TCF12	GGTCAGAAATGACCATGTGTAAGTCT	CACTGTAATGAGGGCTGTCTGTAAA
15	TCF12	TGATCTTTTTCTCCCCCTAGGGT	GAAGACTACTTGCAGAGAATTACCAACT
15	TCF12	AGATGATGATAGAGAACACCCTTGGA	CAATACTCACAGATGCCAAAGCC
15	TCF12	GGTGTGTGGCTACTTTTGGGT	CCAGATTGACCAAAAACAAACAAACTCT
15	TCF12	AGGCCTTTTTAAGCCCCTTACAG	TGGATAACTAGGAGATTCACGGTTGAA
15	TCF12	GCACCATCCCCAAATTCAGATGAT	CAGTAGTATTTGGCAATTGTTTGGTAGT
15	TCF12	TTGCTGCTTACTCCCAAATATTGGA	CAGGAGGCACCTTTCTGACTT
15	TCF12	GCAGATCCCTTGCAAGCAAAAA	AGCAAGGTAATACGGAACACTTGAAAT
15	TCF12	CTGATGGATGACTTTTGGTGGACT	AGACATCATCTTACCTGATCCACTGA
15	TCF12	GAAGACTTACTCCCTTTGCCTGT	GGCTTACCGTTTTCCCATTGTAGTAATA
15	TCF12	GTGGGTAGAAGACTAGGTTGGAA	CCATTTGATGAACTCCAAAGGTCAGAA
15	TCF12	GTGCTTATCCTGTCCCTGGAAT	AAGCGAGTAATGTATACTGCTTTCCTTT
15	TCF12	TTGTCTTAGATGGGACCCACAATTC	ACCCTAGTCACGTGTTATAGCCTAC
15	TCF12	ATCCAGAAAAAGTTGCTGAAATCAGA	TGACAGAGTCTTCCCGATGAGT
15	TCF12	TTGGGTTTTCCTTAACTTTAGGTTGGA	CTGTCTGCATTATTTCTGAAAACCAACA
15	TCF12	TCAATTCAAACTATGGAGGATCAAGCC	TCAGTCTTTGCCTTCCTTTCAGAAA
15	TCF12	GGTATTGATGAAAGAGGAGGTACAACA	CGTAGTCTGCCAAATTACAGCTTTAATC
15	TCF12	CATGCATATCAGCTATTTCCTCATCTCT	AGAAGGACATTGCATCTTGCAAATG
15	TCF12	AATCTAACAGATGCCAGCAATAACCA	AGGCAAACTGGTGGAAGGTC
15	TCF12	AGACTGGATGATGCAATCCATGTG	CTGATCGACTGCTTGCAACAA
15	TCF12	CTCTTCCACGAAAGCGCAAA	GTTGACCACTTGTTCCCCAAGA
15	TCF12	GCAGCAACTTCACGAGCATT	GATGGGAGTCTGTACAGCTTAAAGATTT
15	TCF12	GTGGTTTTCATTTCCATCTTTACGCTT	CTCTCCTTCTCCCTTTCTATCTTCTGT
15	TCF12	CTAATGAAGATGAGGATTTGAACCCTGA	GCCAAGCTCTTTGAATGCTTCATTAA
15	TCF12	GCTTACGCGTGCGGGATA	GTTATACATCTCGGCTGAACCTACTTAC
15	TCF12	AGGTTTGGGTCAGTATCAGCAG	AATGAATAGTATGCTGTCCCAGGTTTT
15	TCF12	CCAGCACAGCTATCTTCTTCAGG	ATGGGATTTGTTGTGTGATACACTCA
15	TGM7	cacaccCCTAAGTGAGTGATCTTT	GGCGAGGACTACTCCAAAGG
15	TGM7	CCTTCCACTCCAGAGGACTGA	GCCATGGTAAGGATGGATGCTT
15	TGM7	CCGCAGGCAGACTCTGTAAAT	CCCTTCTACCTCCGGCTGA
15	TGM7	CTGGAAGGGTCGGCTGAA	GTGACCTGTCCCCTCCTCT

15	TGM7	AGCCTGTTGCAATGGTCCTC	GCTAACATGAATCCTTGTCTCCTTACTC
15	TGM7	AGCTAGGAAGCATCACAAAGAAG	CACCAGTTCCATCGGGAAGG
15	TGM7	CCCACCATCTTAGTGCTGATCT	CCTTTTGTGTATGCCGAGGTG
15	TGM7	CCAAATGACTTCATCGGCGTT	CCCCCATATCAGTAGCCTGTTG
15	TGM7	CCAGGCCCACATTGGGTAATA	CCCTCCACTACTCTTAACTGCTCA
15	TGM7	GTGAGAGGTGGGAACAGTGTC	GGAGGTCCATGCTGGTCCTAA
15	TGM7	GGGAGGCTCCAGACAGATATCTT	ACTCAAGGTGTCTTTTGTATGTGCTTAG
15	TGM7	CCCTTTCCTGAGACCAGCAA	GAGAGTCCATGTCACCCTCAC
15	TGM7	CTCAGAGCCACCATTAAGGTGTT	GGTAACTGAACCAGAGTGCAGA
15	TGM7	TGCTCAGGGCAATCTTGCAATA	GTACTATGACCGAAATGCCGAGA
15	TGM7	GTCTCGTTTCTGAGTTGACAGCA	CCTGAAATGAACTCAGCGATTCTCTATA
15	TGM7	CAGGCCTGCCTCACACT	CGTATCCAGAGGGTGCCA
15	TGM7	CGAGGGTGGGTGCTGTC	TTCTTTGCCCTTCCTGGATCTC
15	TGM7	CCTAAGACCCCCAGACTCCAG	TCCCCATGCATGTCTCTCCTA
15	TGM7	GGAAGCTCAGACTGTCCTCAGA	CCTGAACAAGAGCCTGTATCACT
15	TGM7	CAGTCTTTGGCCGGGTTCTTTA	GCCATCCCCAAAAGTCTGTTAGTG
15	TGM7	GAACTACCAATCATCAATCCAGGCT	TGACTAGTTGAAGGGCCTTGAC
15	TGM7	CAAAGAACCTCACAGGGCCTT	CAGTTATTGGCCATTACACTCTGAAAAT
15	TGM7	TTCCCAGCGGGTAAGTCAC	AATGTCTGGAGCGCTTCTGATT
15	TGM7	GAGAGAGTTGGAGTCAATGGTGA	CCCCTGGAATCATATACCATACAGGTT
15	TGM7	CCAAGACGACATAGCCAGGAGTA	TCAAGGAGATCAAAGGCTACAAGG
15	TGM7	CAGCCACAGTGACGAAGATGT	TCACAGGCTGTTTCTGACTTTCA
15	THBS1	TGAAGGGACCAAATGCCAACTT	TCATCGATGTCTGGCACACTG
15	THBS1	CCTGCAAAGATGATTTTGACCATGA	TTACCCTGATGGCGTACAACC
15	THBS1	GGACATCCCAAAATGACCCTAACTG	CCATGTATGCATCAGTCTCTTAGGATCT
15	THBS1	GTTGATCTTAATTGTTGCCTGTGGTT	TTACATCACCAACGCAGTCCTT
15	THBS1	AAACGTAGTCGTCTCTGCAACAA	GCTGACTGCAGCATTCAGTAAAA
15	THBS1	GGGCTCCGAGTTTCTGGATCTA	GTGTTCTCACACCGGTGCT
15	THBS1	GCATACACGCAACCCCTCTA	CGTTGTTGTCAAGGGTGAGGAG
15	THBS1	CCTGCTTCAACCACAATGGAG	TCTGGTTTACTCGTCTAGTTGACTGTA
15	THBS1	TTTCTTGCCAGCTACCAGTGT	TGGACAGCTCATCACAGGAGAT
15	THBS1	ACAAAGGACTTGCAAGCCATCT	GCGATAAACCTTCCAGGGTTCA
15	THBS1	ACATATTATGCACATGGTTTGAGGCT	TCCCACATCATCTCTGTCATAGTCAT
15	THBS1	CATTCCATTACAACCCAGCTCAGT	CTCTGATCAGGCAGCACCTTA
15	THBS1	ATTAGGCTTTTGTCATTAGAAAAGTAGAGCT	CAGCATTGGTTTATGATCAGTCTTTCAA
15	THBS1	AGGATGTCTAGGAACATGATGGAGAA	GGTAGCCAAAGACAAATCCAGCA
15	THBS1	CACCGAAAGGGACGATGACTA	TGTGGTGGAGTTTACAACTTTCACA

15 THBS1	CAGGGATACTCGGGCCTTTC	TGTTCCAGGGCTTTGCTTCTTA
15 THBS1	CCTGGAAATACTTCTGCGTGCT	GATCACACCATCACCACATGTCA
15 THBS1	GTCCCCGTGGTCATCTTGTT	GCAGCGGACCTCACACTTAC
15 THBS1	GACTGTCTCTCTCTCCTTGTCTCA	GAGCCCCATCAGTTCCTCAC
15 THBS1	CCCCGCTCTGCATTCTACAA	CTGATTCCCTATGTGCCCTAAAAATAGA
15 THBS1	TCTGTGTAACAGCAGCATGGT	GATGCCATTGCCAGCGTAG
15 THBS1	CCACTATAGCGACCCCATGT	CACACAAAGGACCTGGCTCTA
15 THBS1	CCCTCTAAAGAACAGCTTGTCCTA	TTGTTGAGGCTATCGCAGGAG
15 THBS1	CTGTGGCAATGGAATTCAGCA	GACAACAAGGACAGGTCAGTTCT
15 THBS1	GGAGCTGTGTTTCAACCTTTCC	TGTGACTGTAAGTGAAAGTGAATCAGT
15 THBS1	GGAGTTTTCACCCCAACCCTT	GCCCCGGTGAGTTCAAAGATG
15 THBS1	CAGCTGTACATCGACTGTGAAAAG	CAGATGATCCCACGGAGCAG
15 THBS1	CGGAGACAACAGCGTGTTTGA	TGGAACTTGTCATCAGGCACAG
15 THBS1	CAGCTTTCCGCATCGAGGAT	GAGTGGTCTTTCCGCTCCAG
15 THBS1	CCCTGAGGCAGATGAAGAAGAC	GAGAGCTTCTTCCACAGACACC
15 THBS1	GCCTGACCGTCCAAGGAAAG	CGTCCAACTCAGCATTCTCCAT
15 THBS1	CTGCAATTTACCCTCCATTTACATCTCT	CCATCTTTGTCATGGTCAGCCT
15 THBS1	CTATGTGCCCAATGCCAACC	GTCTTAGAAAGTGGCTCCCATGA
15 THBS1	CCTGTTCCCCTCTCACTCTCTT	TCACTGAAGAATAGCTATGCCATTTGAG
15 THBS1	GGAATTCCAGGTACACTCCCT	GCATCACCAATTCCATCCTTGTCA
15 THBS1	CCAACTCAGGGCAGGAAGACTA	GCTGAACTGAAAAGATGAAAAAGGGAT
15 THBS1	CTCCCTGCCTAGCTGATCTCT	CCTAAAGGTGCCACACAAACTCA
15 THBS1	CCCAGCTCTTATTTGTCCCTTGT	TCTGAAAGGAGTCCATCCACCTA
15 THBS1	GGGACACTAATGATATTCTCTCCCATT	TGGCTGGTGAAGGTCCCTTA
15 THBS1	AGGCCAAAGACGGGTTTCATTA	GAGTCAGCCATGATTTTCTTCCCT
15 THBS1	CCTGCAGAGTGGTGATGTATGA	TGTACATTCATGGTGATGTTGCTCT
16 CDH1	GTCCCAAAGTGCAGCTTGTCTA	CATATTTTTGTCAGGGAGCTCAGGAT
16 CDH1	GCTTACACCATCCTCAGCCAAG	CACGGGATTGAGCTAATACACATTTG
16 CDH1	AAGATGCTTTTGTCCCTTCTTCTTT	TGGTCTTTGTCTGACTCTGAGGA
16 CDH1	GCTAGTCTGAGCTCCCTGAAC	GATTTCTGCATTTCCCAGCACAT
16 CDH1	AGAGCCAGGACAAGATCTAGACTT	CAGAAGTGTCCCTGTTCCAGT
16 CDH1	GTATTTTCTCTTAGGTTCTCCAGTTGCT	TCTGCATCAATGATGTTTATGACCTGA
16 CDH1	TTCTGTGAGAGGAATCCAAAGCC	GCAAAGTTGCCAAAATAAAACTCAGGTA
16 CDH1	AGCTTGCGGAAGTCAGTTCA	GCGTCCCTCGCAAGTCAG
16 CDH1	GGTCCTATTCTAAAAGCCAGAGCTT	GATTAGGGCTGTGTACGTGCT
16 CDH1	GAGGATTTTGAGCACGTGAAGAAC	AAAGAATGAACTCTTCCCTCCAAAAGAA

16 CDH1	TTAACTTCATTGTTTCTGCTCTCTAGG G	TTCATTCACATCCAGCACATCCA
16 CDH1	TGGTACCTTTTGAGGTCTCTCTCA	GCCAAAGTCCTCGGACACTT
16 CDH1	GTGCCTCCTGAAAAGAGAGTGG	AGTTGCTGCAAGTCAGTTGAAAAAT
16 CDH1	CTAGTGTTCCTGGTCCTGACTT	GGATTGAAGATCGGAGGATTATCGTT
16 CDH1	GTGATCACAGTCACTGACACC	AGTTTTCAAAGACTTCGCCCATGA
16 CDH1	CAAACTGTACACTGCCCACAGA	CAGGAGAGGAGTTGGGAAATGTG
16 CDH1	TCTGGAATCCAAGCAGAATTGCT	AATCCCTCCCAGAGAAACAGAGA
16 CDH1	TGCTAGCAGTCTTGGTACTTTGTAA	TGTATACAGCCTCCCACGCT
16 CDH1	TGATGCTGATGCCCCCAATAC	GCTGTGAGGATGCCAGTTTCT
16 CDH1	GCCCCTTCTCCCATGTTTTCTT	TCGGTTACCGTGATCAAAATCTCC
16 CDH1	GGGAATGCAGTTGAGGATCCAAT	GGGTCCAAAGAACCTAAGAGTCT
16 CDH1	GGCAGCTAGTGGCTGTCTAA	CTCTCCTCCGAAGAAACAGCA
16 CDH1	CCAGTTCTGATTCTGCTGCTCT	CCACTGAGCTACCAAGGTTTTCAAA
16 CDH1	TGTTCTGTCACCAGATTATGGTTTGT	CCTTTGTCGACCGGTGCAA
16 CDH1	GCCCTGCAGTGAATTTTGAAGA	GGATCTGTGGGTTATGAAACCGT
16 CDH1	GTGTGATTACAGTCAAAAGGCCTCT	CGAACTTCTCAGAAAAATGCCAACAT AC
16 CDH1	AGGGAACCCTCCGAGTCA	GCGGCCTCTCTCCAGGT
16 CDH1	GAGAGCTACACGTTCACGGT	AATTTCTCGGCCCCTTTCCAA
16 CDH1	TTCCTCCCCTGGTCTCATCAT	GTCACACACGCTGACCTCTAA
16 CDH1	GATAACCAGAATAAAGACCAAGTGA CCA	TCACTTGCCAGCTGGACTTAC
16 CDH1	GGAAAAGACCCAGTGTTGGGAT	TTCAGCCATCCTGTTTCTCTTTCAATA A
16 CDH1	CACCCCCTGTTGGTGTCTTTA	TGTTACCCCGGTGTCAACAAG
16 CDH1	CTGAACATAGCCCTGTGTGTATGA	GGACACTCATGAGGGTTGGTG
16 CDH1	TGAAGTGACTCGTAACGACGTTG	AGCTTAGAGATGAGCCATGCTTTG
16 CDH11	CAAAACCATGAGGAGAATACTTACGG A	GTATTCCATCGATCGTCACACTGA
16 CDH11	TCCTCTCTATCCAGAGGTTTTGTAGTT T	GCTGCTCTGAATGTCTTGGTCTA
16 CDH11	GTGGGTTGGAAAGCTCAGGATT	CCTTCCATGGGCACCATGAG
16 CDH11	GGAGCGCTGTAGCACCT	TGTGCCTACCACGTAACCAAA
16 CDH11	CCTACAGGGCTTTCCCAGAAC	GCCCCAAGTTACATCCACGA
16 CDH11	GCAGCTGCATTTTCTTGGACT	GAAGTTTATCAGCAATGGCCCTTTC
16 CDH11	CTTGACGGTCACAGTGTCCTT	TGTCAATCTTCTCTTCATTCTTGCCA
16 CDH11	CCCCTGGTTTCGCAAATTCTCA	GGAATCGTTTGAAATCACAACGGA
16 CDH11	CACCCCCTCCTGTGTTTCATAG	CCAGATGTCTGTGTCAGAAGCA
16 CDH11	TCTTTAGCTTTCACTCTTCCTACTTCC T	CAATGAAATCTATAGTCTTACAGCTG CCA
16 CDH11	TGATACCTCAGTAAAGCAGGAATAGG T	CGGACTCTCAGGGACAACCA

16 CDH11	CGGTCAGTGTGATCGTCACTT	TGATGTTGCCTGCTTACCTGTC
16 CDH11	GTCAGGCCTGGGACTTCTTAT	CAGATGCAGATGACCCCACTTA
16 CDH11	TACACTAACTTGGCGCTATTTCCA	GGTGCCTTCTTCAGGGTTAATTTTAGA
16 CDH11	AGAACAAAACTATGGCTCTGAAACCT	GTGAGAGTGATCAGACCAAGCC
16 CDH11	GCAGGATTACCTGGTTGGAAAGT	CTGTCTGCATTCTGTCTTCCCA
16 CDH11	GTGGCATTATTATTTCCGGTTTCTTCTT	CATTCACAATCCAAATTTCACAGTCAGA
16 CDH11	AACCTTAGTACAAACCTCGGTTGTC	AGCATGATTTATTTATGGGCCACCTT
16 CDH11	CAGCCTGTATTTGAGAAGCATGTG	TCTCCTGCAACGCAGAGG
16 CDH11	GCCGGCGTTCAGAATGTAG	GCAGAAGCAGGACTTGTACCTT
16 CDH11	CCGCCATCGCTGATCACTAT	CGCTGTGATTCCAATGGTCTTG
16 CDH11	CTAGATTCTTGAGAACGCCAGACA	CCACAGATTCAGACTTGGACTATGATT
16 CDH11	CCCTGCCTTCATAACCGTAGA	CCCGCAAAGACATCAAACCTGA
16 CDH11	CGAGGTCCCCAGTTCTGTAGAT	GCTCCTCCTTATGACTCCATTCAAA
16 CDH11	CCAGGTCTAGGCATGTACTGATAC	GGAAGAAGATGTCCGTGAGAACAT
16 CDH11	CCACCCCCTTCATCATCATAAGTAATG	CTGGACTTAACGAATGCTTACCTTCT
16 CDH11	AGCTCTACTTCCTGATTTGGGAGAA	CATTAATGACAACCCTCCGGAGT
16 CDH11	CATGATAGGTCTCGTGCAGGA	ACATTCATGCCACCAAGACGTT
16 CDH11	TGGGCTCTCTCTTCTCGATCC	TGACTCTGGTGATGGGAACATTAAATAC
16 CDH11	AGCTCCTTCCCCTGAGAGAAT	GTAAGTGAGTTTCCCCACTGCTT
16 CDH5	GTAACATGGTTCCTGCTGGGAAT	TGATGTCTTGCAGAGTGACCAG
16 CDH5	TGGTGGAAGCGCGAGATG	CAGAGCCCCAAGTCACTGA
16 CDH5	gcactcaCCATCCTTTTTCCTTAC	AATGACCTGGGCTCTGTTTCC
16 CDH5	ACCATCGACCTCCGATACATGA	GTGCCAATCAGAGGCTTCTTCT
16 CDH5	CACTTCCAGCTGAAGGAAAACC	GATTGTCCTTATCATTCTCATCGACACA
16 CDH5	GCTCACGTCACCTCTTCTTTTCTA	AGCAGTGAGGTGGTACTCTGA
16 CDH5	GGCTGGACCGGGAGAATATC	GAACAACCGATGCGTGAACA
16 CDH5	AGTTCATGACGTGAACGACAACT	CAGTGAAGTGGCTGATGTGAGA
16 CDH5	GCTGAAAGAGGCAATCGCCTT	CTGTACTTGGTCATCCGGTTCTG
16 CDH5	TGTTTGTTGAGGACCCAGATGAG	CCCAAGCCTCACGTTTGACA
16 CDH5	TGTAGACTCAGCAAAGAACAATGGAA	CCTCCACAGTCAGGTTATACCAG
16 CDH5	AGAACTGGACAGAGAAGTCTACCC	AGTTTTGGAAGAATGAGGGAAAAGGAA
16 CDH5	AGATGGCAAAGCCTGACTCC	GACTTGGCATCCCATTGTCTGA
16 CDH5	CACTTCCTACCCGTGGTCATC	AGTAAGATGGCTACCACTGCCT
16 CDH5	GAGTTCACCTTCTGCGAGGATAT	CTTCCACCCACTGTGTCTTCTC
16 CDH5	ACCATCACTGACCATCTCCTGT	gcccagcTGAACTGATTACTTTGAT
16 CDH5	CCTTGTCTGACTCTGCTGCTC	GTAGCTGGTGGTGTCCATCTC
16 CDH5	GGGACCCAGGTTTAAGATGCT	GGGAGTCACTGCCATTTTTGG
16 CDH5	GCAGCTGGTCACCTACGA	CCTCGGTGGCTTCTGCAC
16 CDH5	GCCCGGCCTTCCCTCTAT	GGAGCCCTCGTAGCCGTA

16 CDH5	CCTACGACACGCTGCACAT	GGGTCCGAGCCGTACAG
16 CDH5	AAGTCAGCAACCCCAGGATTC	TGTCCAGGAGTGTTAGTCACACA
16 CDH5	ACAGCCTCCTCCTGCAAAAAG	CTCATCCAAAACTTCAATGTGGACTTG
16 CDH5	CCCACAGGAAAAGAATCCATTGTG	CCTCCCACCTGAACCAGACT
16 CDH5	TGTGTCTCCTCTTTCCCCAGAT	CAGCAGGCTGTGGGTGT
16 CDH5	GCTAACCCTGCCCAACGG	CCAGCTGCTGATGGCTTCT
16 CREBBP	AGTCTGTCCCAACTACATAGATTCCA	GGAAAACCTGGTAGCCTATGCTAAG
16 CREBBP	ATGAACGTGCCTTGCCCTAA	TTGCAGTATGAGTTTTCCCCACA
16 CREBBP	CGTACATGTCCCCTTCCACTTT	ctggACGAAACCCCATGTCTTTA
16 CREBBP	AGGATGGAAAAATAAAAACGCATAAAACT	AGCCGTTTACCATGAGATCCTTATTG
16 CREBBP	CCAAACTCACCCTAATTTCTTCACATACT	CCTGAGTTAAACATGTGCCTCCTT
16 CREBBP	CAGGCTAAGGGATGGCAGTAG	AAACGGAGGTCGCGTTTACATA
16 CREBBP	GGTTCCCCAAGATGCCTTGTT	CACAGATCATTCAGTTGCTTTTTACAGT
16 CREBBP	GTCCAAGAACATGAAAGGGAAAAGG	CCCGAACAGAGTGCAATGCT
16 CREBBP	GGGACAGTTCGCTGGACA	GTCTCACCCCAGACTGGTT
16 CREBBP	CCATGGTGACTGCGAGTCC	ATCGCCACGTCCCTTAGTAAC
16 CREBBP	GCTGGAGACCGCACCTG	CAGCGGATTCTGCAGCAA
16 CREBBP	GGGACCCAATCTGCTGCTT	AGATGGCGGCTCAGATGG
16 CREBBP	CCCATCTGGCCAAGCTGT	CCAGTTCCAGCAGCCTCAA
16 CREBBP	GGTAGCCTCCGGGTCCT	GTATGAATCCACAGTACCGAGAAATGTT
16 CREBBP	CATGCCGGCACTCCCTt	CCAGGCCTTGAACATCATGAAC
16 CREBBP	CTGTGGATTCATACTCGCCATGT	CCAGCCTGCAGAACCTGA
16 CREBBP	GGTCTATCCTAACACGGCTCACT	CCCATATCGAACCAAAGCTCTGTT
16 CREBBP	GCCGTCAATTTCCTCAAAAGCA	GTCAGCAACAGCCTTTGTAAATGTC
16 CREBBP	AACAAGAACCACAGGATTCTCAAGT	AGATGAACAGCATGGGCTCAG
16 CREBBP	AGAGAACACTTACCCCTGGCA	TGAACCGTTGTGGGTACATTTACAG
16 CREBBP	CGGAGCCTTATGAACCAGAGA	CGCATTGTCGAACCATGAAAAAC
16 CREBBP	ACAAGAATTTTATTTCCTAGGGAGCCA	CCCGCTCAGAGCCAGTTTC
16 CREBBP	GATGACGGGAACTGGTTCTGT	CTTCCGAACTACAGCTCTGGTG
16 CREBBP	TGACAATGCGTCATGTGATTCAAAAC	GCAGATCCTGAAAAACGCAAACT
16 CREBBP	AGAACCAGCTGCTGCTGTATC	TGAATATGACTTGGAACTCTGAGAGGTT
16 CREBBP	CTTGGAAGGATGACCTCAAACTCAA	GAGGTTTTTGTCCGAGTGGTG
16 CREBBP	CTTGACCTCCACCGTCTTGT	CCTGCTCCTTCTGGACTTCCTA
16 CREBBP	CAGCTGCGAGTCTTTCCCT	CATGCCCAACGTGTCCAATG
16 CREBBP	CATACAGCTTCTGGGACAGGT	CACAGCCTTGCGTGTTGTT
16 CREBBP	CACCATCAGGTACAGACACCAA	TTTGTCTACACCTGCAACGAGT
16 CREBBP	CGTCTCCACGTGGTGCTT	TCCTCACCCTCGCCAGA
16 CREBBP	GGAGAACTCCCAGTGCTTGTC	GACTAAAGCCCCTCCTCTCCT
16 CREBBP	GCCAATGGGCAACACAGGAATA	CACTATACCAACAGCAGCTCCTC

16 CREBBP	TTTCCTTACACCGGTGCTAGAAG	GTTTGAGTCACTTCAGTAGATGGATGA
16 CREBBP	CACTGGCTCTGTGCAGAACT	GCTTTAACAGTGCGCCTTCTTTG
16 CREBBP	TGTGCAGTCCAGGAAACAGAAA	CTGCATTTTGTTGGTTTGACAATTTACA
16 CREBBP	CCGCGGTTAGGTAGGAAGTATT	GAATGCCGTACCCTACTCCAG
16 CREBBP	AAACCTGCGTTAGGGTCTCAG	CTCAATAGTAACTCTGGCCATAGCTT
16 CREBBP	TGCCCTTGTGAAGCCTGATTAATT	CCAAGCACTGAATCCGCAAG
16 CREBBP	CAGCCCCACTTGCTTTTGTG	TCAGTGCCATGGGCAAGAG
16 CREBBP	TGAAGAATCTCCCTGGCTCAGA	AGGCAGCGGCTCTAGTATCA
16 CREBBP	AATTCTGCTTATCAGCAAAAAGGAATGG	CCTGTCCCTGCCAGTGAA
16 CREBBP	ACCTTGAGAAACTTGCATGCGA	GGACCTATTGGCTGTTTTCCCTTTTAA
16 CREBBP	ACGAAGTTGAGAGTTCCTTCACCTA	CCAGCTCCATGCAGCGA
16 CREBBP	GGGAGTCCGAGAGCAGCATA	TTTGTCTGTCTGTCTGTTTGTCGT
16 CREBBP	GCTTTTAATCCTCCACATGGAATCCTA	GAGAGTAGCAGTAACGGCACAG
16 CREBBP	CGAAGGAGATGTTGACTGAGAGG	AGCTTCCCAAGTTAAAGAAGAAACAGA
16 CREBBP	TGGTTCTGATTTCTGCTCTGCTATG	AGGTTGCGGTAACATAAAGGTGTT
16 CREBBP	AAAAGATAACCTCACACCAGAAATTCCA	GAAGAAAAATGATACCTTAGACCCCGAA
16 CREBBP	CTGAGGGCCAAGGGTAACTT	CTGGAGTGAACCCCCAGTTAG
16 CREBBP	GTTGACCATGCTCTGTTTGCTG	CACCACAGCTGATGTTACCTCTT
16 CREBBP	GGTAATTAACAAGTATGCGAATGCAAGA	CAAAACGACTGCAGGAGTGGTA
16 CREBBP	ACGCCTTGTCCAGCATCTTTT	GCACGTTCATCTGACGTGTGT
16 CREBBP	CGGACGCTCTCTTTCAGGTG	AGCAGGTGAAAATGGCTGAGA
16 CREBBP	GCACATATGCACTCCCAGTATACA	TTGACCCTGTCATGCAGTCC
16 CREBBP	TGCGTCCACAGCAATATCCAA	AGTACGTGGACGACGTCTG
16 CREBBP	GCCAGGCATTGTTGAACATGAG	GGGATTCCCTATACTGAGACCAT
16 CREBBP	CAATTTAAGGTCACCCTCCCTCA	AAGTATGGCCTTCTTGCTGACA
16 CREBBP	TCTGCCACACATTTAGAAAGAATCAGT	AGCCCCCAAACTTGATTTCAGA
16 CREBBP	AGGGAAGTCGGAAGAGCTGAT	GCATGTTGGTTATCTGTCATCAAAACT
16 CREBBP	ggccTGACACACAATTTTTATGGG	AGCCTCAAACCCCAGTTCAG
16 CREBBP	GCTGTTGCTGCGATGACTGA	CAGTGCTACCCAAACCCAGAG
16 CREBBP	GCTGCTGCCTGGACTGTA	TGCCATCTCTCCAGCACAC
16 CREBBP	GAGTCATCCCAGGTGGTGTC	TGCTCTTCCTAACCCTCTCAACAT
16 CREBBP	GGTGCAGTGGTGACTGTGT	ACCATACTCTGTCCATTTCTGGTA
16 CREBBP	GGAGCTCAGAGAAGGGTCTGTA	GTGATTTCTGGCCCAATGTGTT
16 CREBBP	CTTGTTCTAGTTCCTTAATGCTCTCTTCT	CCTCATCTCACTGTTGTGCTTTG
16 CREBBP	CGAGGACACCTGGGTAAAGTTG	CTGACGTACCTGTGCTGGAA
16 CREBBP	GCTTGGGTCTCCGTCTTCAT	CTGAAGTCAGTGCTTTCGGTTTTT
16 CREBBP	GCCAGCCTGCATGGCAT	CAAATCAAACCCGCAGCTAATGG

16 CREBBP	CCGCTCACCTGGTTGGG	CTCCTGCAGCGGTGGAA
16 CREBBP	GGCCTCACGCTCGATCTG	CCCTCACCCGTGAGCAT
16 CREBBP	GGGAAGCCAGCTGGTGA	CGGATGGCCACCATGAACA
16 CREBBP	GCATTTGTTTTCTTGGCAGTGCTT	CGCAACGCCAACTGCTC
16 CREBBP	GCTGTGCGCTGTTTGATGAAA	AGCGCTCTGCAAGACCTG
16 CREBBP	GACTCTGCTGAGGCACGTT	GCCCTCTGCTGCTACCAC
16 CREBBP	GCACCACCCGCTTCATCTT	CTGGGCCTGGATGACGAG
16 CREBBP	CTGGGCGACTTCAGGGT	CTTGCCCAGGCCTGTGA
16 CREBBP	CCTGGGCCTGCATGGATA	CCCCGTGAGCCTGAATGTG
16 CREBBP	GGGCTCTTTGACTGTGGCT	CCTGTCCCCTTTCCTTGCTT
16 CREBBP	AAACAATGGACACTCAGAAGTCACA	CTAGAAGCACTGTATCGACAGGAC
16 CREBBP	GCCGGAAAGGTAATGACTCTGG	AGGGATCTGAGCACCTTTTACAAAG
16 CREBBP	GCTCACATTTCCTATTCCTGGGT	CCAATGGAGGAGAATTAGGCCTTTTAA
16 CREBBP	CTGGAACAAGGTTCCCACTGT	GCTGTAAAGGTTGCTTAGTTTCTCATTT
16 CREBBP	CCTGCACTCCATGGCTCATA	GAAAAATGCCAGTGACAAGCGAA
16 CREBBP	CCGTGCTCACTTACTTTGTTGGT	ACATCTCGTTGTTTTTCTGGTCATAGT
16 CYLD	GACACCACGTTGCTGAAAACAT	AGTAGGGTGAAGTGACTTTTTCTTGG
16 CYLD	CACAGAGTTACTTTTGGCAATTACCAAT	CCCAGATCTCAGCTGTACTTTCAC
16 CYLD	CAAATAGACGTGGGCTGTCCT	CAACTCCAAAGAATATTCCGGAGACT
16 CYLD	TCACAATGAGTTCAGGCTTATGGAG	CGGTACTTTAAGGAGCTTTTGTGTTTG
16 CYLD	AGGACCCCTGTTAGCAGAGAG	TGCATACAAATACACAGACACACATGA
16 CYLD	CAAGAATGCAGCGTTACAGACAAA	ACTGCATGAGGTTGCTCTAGAATTTTTA
16 CYLD	GCAAAAGGCAAGAAAAATCAGATTGGAT	TTTTAAACAGGCTGAACCTCTCCTC
16 CYLD	GGGTCTTCTGTTGACTCACAACC	AGCGGTCTACTTATTCTGTTTCCAAAA
16 CYLD	CTTAGATATGGATATGTGTGTGCCACAA	ACTTTGACATGTGAATATGCTCTTCAGT
16 CYLD	CTGTTCTCAAATACTGCTGGGACA	GGGAACGCCAACTTTCTCATTTTT
16 CYLD	GCAGGTCAAAAGGTACAAGATTGTTACT	TCCCACAGACTTTCACATAAAATACCAC
16 CYLD	GTTCTCCAGACTTTACTTATTTTCCTCTTCT	GTAACACAGTGTCCAGAACAGAACTAAA
16 CYLD	TCCTTTTTCTTTTGCAGCTTATTTGCT	ACAATTTCTGTCCTCAGTAGCTCTTG
16 CYLD	ACACTGTGTTACTTAGACCCAAAGAAAA	CAGAAAACAGCAAATAGAACACCATGT
16 CYLD	AGGACACCAATATTAAGGCAGAAC	AAGTCTTTTCCAAATCGAGGCATCT
16 CYLD	CTGATTATTCAGATGCCTCGATTTGGA	TCTGACAATTTCCACAAAACCAACAAAA
16 CYLD	GAGGGAGTGGTGAGAAAGGGTATA	GGTGAAATACCGAGTGCCTCTG
16 CYLD	GGCTGTACGGATGGAACCTT	ATTGGAAACCGGCTGCAATG
16 CYLD	GGCCTGACTCTAGGTTTGCAT	CACTGTCACCATCACCTAATATAACATGA

16 CYLD	ATCTTTTCAGCATTTGGAGGCTACT	GAATTGTAATGACCCTGGATGCCT
16 CYLD	GCTTGGAGATAATGATTGGGAAGAAGAA	TGCCTCATGGCACTATCTATACCTAT
16 CYLD	CCCTTCCCCTTCTCACATTTCA	TTATCCATTAAGTGAAGGGAAGCTCAC
16 CYLD	AACATGCTTACTGTTTCAGAGAGTGT	GGCAAGTTAGGCATTATGCAAATTACT
16 CYLD	TTTCTCCTAGATCCTGAGGAATTCTTGA	GGAAATCATGTCTGTTGAATAATGGCA
16 CYLD	GCAGAAGACCCTGCAAAATCTC	ACTGAATGGTAAAGAGTGGAATCTGTTC
16 CYLD	GTGAACTCACTGACCACCGA	GTGCAGTGTTTAGCTCTTCCATTAC
16 CYLD	ACTTTCTCTGTCAGCCCAGTCT	GGGTTCTCCTTAACTTCAGCCAA
16 CYLD	GAACCCCTTTTCCTATGGATCGT	AGTTCTAGCTTGTCCAATGCAACA
16 CYLD	ACATGGTCTAGAAGTGGGCTCA	CTGCACAAAACCTCACAGAGAAAAA
16 CYLD	GTGATGAAGATTGTGGCGTGTT	TCTCCAACCTTCAAAGAAACTCTGG
16 CYLD	TCGAACTTCCTCCTTTGGAAATAAACT	CCAAAATTTTCTTACCATGTCCACACC
16 CYLD	TCACTGGCAAAAGGGTTTAGAACTT	GCCTTGGATTCTCCTGGAGTC
16 CYLD	TCTGGAAGACCTGCATTCCTTG	CGTTAGGACTCTGCCTTCAGTT
16 CYLD	ggcctAATGACATTCTTTTCATGGT	TGCAGAGAACAGCAAATAACTCCA
16 CYLD	GCTGCATCCCTTGCCAGAATA	ATGCAGAAGAAAGGCGTTTTCAG
16 ERCC4	gaatgGCAATTACCTACCTGTTCTGT	ACGTTTGTTTTTCTGGCGAAAGAG
16 ERCC4	CGAGTCTTGTCAAGAAGCATTCATCTT	AGATTTCTCATCACTCTTTCCACATGAC
16 ERCC4	GCTGTTGCCTTTGATACTGGTTTTT	GTGCAACTAAGTTACAGTAGATCTCACA
16 ERCC4	AGAAGTGTTGACAATGTTTTCTCCCA	AATGGAGGTGCCTGACAAAGT
16 ERCC4	GAGCGCTCTAGGTTGCTGAT	CTCAGTTGAACCTCCGTATATAAGAAAGT
16 ERCC4	AGTTCTGTCTTAACATGCAGGGTTT	CCAGAGGTTTCCAATGTAGTTGGG
16 ERCC4	TCTGTTGTTTTAAAAGCCTTTGGAAGAC	TCACTCTCCTTATTTTCTGCCTCAATTT
16 ERCC4	GGGAGGCACTGACTGAAGTATTAAAAG	AGGACTCATTTGATAGATTTTGGAAGCA
16 ERCC4	CCCTATGCTCTGACAAGGGTACT	GTGCAACTGAAAGCTGTGATTCTTTATA
16 ERCC4	CAGGATGACAGCCAGTTACGTAT	ATCTGGAAGATGATAAACCCTTGCTC
16 ERCC4	CCAGCACCTCGATGTTTATAAATGCT	ACACATTTAAAGACTTAACCCCACAAGA
16 ERCC4	CTCGGCTCTCTTCGGTTGA	CCATGGCAATCCGTCGAG
16 ERCC4	CGGAAGAGCTTCCATGGAGTC	GCAGTGCAGCTGGAGAAAGT
16 ERCC4	CGGACCGGCTCCTCTAC	TGTCAGAGGCCTCGCATC
16 ERCC4	CACTGGCCATTACAGCAGATTC	GTCTTGTGACAGGGCTGCTAAT
16 ERCC4	CACCACGTTAAGAACATCGCA	GGGATAAGAAAACAGCCATCACTGT
16 ERCC4	CTGTGACCTATTAAAAACTGCCCTGTAT	ACTGTTGCTTGTGATTTCATTTGTTACA

16 ERCC4	CAGCTGAAGATAGAAGGAGTTGAACA	TTCTATCAGTCAAGAAGTCAACCACAAG
16 ERCC4	GTAGGATACTTGTGGTTGACTTCTTGA	ACTGAAGACAGACTATTCTCTGGAGAAA
16 ERCC4	AGATGTCTTCCCTTCGGGTGA	GCCTCCGCTCCAAGAGTGATATA
16 ERCC4	AACATGTTCCCAGCTGAGAGAC	GGGTCTTTAGGTCTTTTGTGAGATT
16 ERCC4	GAAGGAAGACAGTTCAAAGAGAATTAGGA	CTTCCTCTTCCAGTTCTTCAGGTTT
16 ERCC4	GAAGTTGACCTTAACTCAAATGGTAGGA	CAGCATCCGATGACAAATTTACATCAAA
16 ERCC4	CCCGGAAGAAATTAAGCATGAAGAA	CGTATCTTGGCTCCACTTCATGT
16 ERCC4	TGAGAGTTCTTCCCCAGTGACA	CGATGGATCAGAGATGGAAGCTC
16 ERCC4	TGTGGATATGCGTGAATTTCGAAGT	CGGCCGTTATTTAAAGAGCCGATTA
16 ERCC4	GGAGCGCAAGAGTATCAGTGATT	GAGAAAGGCTTGCTAGGGTCA
16 ERCC4	GTCCCGTGCTTCTGATTGAGTT	GGGCACCAGAGAATCCGTAG
16 ERCC4	TCTTCTTACACTTCACTTCCCCAGA	TCTCTGACTCGGGAAGGGTTT
16 ERCC4	TGTTTGTTGTTTTGCTTTTCGTGTTG	CAGGTTTGTGCTGTTCTAAAAATGAGTT
16 ERCC4	TGTCTCTTTAGGTTCCATGTAGCAGTA	GGCATTTTAGTTCCTTTAGACATGCATT
16 ERCC4	ATGCTTGCTATACAGACTGCTATACTG	CCTTGTCAAAAGGTTTTCCAATAGCATT
16 ERCC4	AGCAATACCAAATTTTATTCTTGTTTTAGACA	CTGCAGCAAAGTTCGTAATATCTTCAAA
16 ERCC4	CTTGGAGCCAAGACTAAATCCTTAGTT	TCCCACCTGAATTCTGACCAAAAG
16 ERCC4	GGAATCTCTGAGAGCAACGGAAAAA	GCACTTTTCACACCTGATTCCC
16 FANCA	TGAACTAGAATGATTAGCATAGGCCTCA	ctggccTGGAGCTTGAATTTTT
16 FANCA	CATCTCACCACCCACACGTA	ATCTGTGTGCTGGTGCTCTC
16 FANCA	GAAAGGCAGAAAACTGATACAATTGCT	TGCAGGTATCACACAAATTACAGAAGATT
16 FANCA	GCAGCTGTCAATTCTCATGTCC	TGTGAGTTTGCCTGTTTTCTCTCT
16 FANCA	GAGAAGACGCGACTGTGGAA	GTGCACCTGGGTGAGTCC
16 FANCA	CCCACATCCACCTCTGGGA	CGTCTTCAGTGCCCTGGAA
16 FANCA	GCCTCAGCAGCGTGTTTCTTA	TTGGTCTCCTGCGGTTTACAG
16 FANCA	AGTTTCTGCTGGGACAGGTG	CTGCGCTCTGCTATCTATCCT
16 FANCA	GCATTGTCAGAAGAAACCTGGAAG	TGTGACCAGTTGTGTAGAAAGCAA
16 FANCA	ATTGATTCCTTTCCCCACTAAAGCA	CCTGACGCTGACCTGTCC
16 FANCA	ACGGACACCAGCTTCCTCTTA	CGAACCGACTTCTCTCCGTAG
16 FANCA	CGGTCCGAAAGCTGCGTA	AGCAATTTCTTACTCTCTCTGCAAGTTT
16 FANCA	ACAAAGTATCTCGTGACTGGGAAGA	CAGTTCGGAATGCACTAAATTCAGTTTT
16 FANCA	GCGAAGCACCAGAAATCATGGA	TCTCCACACAGGCCTCCT
16 FANCA	CAAGCCCAGAGAAATAGCACTGA	ACAAGTCAGGGAAGAAAACATCAGG
16 FANCA	AGTCCAGCTTCTTTAGCTGCTT	GGTTGTAAGGCCCTGCAATG
16 FANCA	GGTGTAGCACAACAGACACTCA	ATTGTTTTCCTGTGATCCAGAGCA

16 FANCA	GGGAAGGAACGGTCACCTAC	TCTCTCTGGACACACAGAACCT
16 FANCA	TCCTCTTTCAACACCTCTCGGA	TGTTTCAGTTTCAGTTCCTCATGTTCA
16 FANCA	TCGGGCCTCTGAGAACAATC	GCTGTTGATGGTCTGTTTCCAC
16 FANCA	CGGCTCCTTCCGCTAAACTC	GCTTGGAGACCATGATGTCCATAAATAA
16 FANCA	CCAAGAACCCGCATCTTGTCA	CTTACGTCTGCCTCTGTGTGT
16 FANCA	CTCTGGCGGGAAGGGAT	ACTCGTGGGTCCCGAACT
16 FANCA	TGAAGGAACTCACAGGAAGCTG	CTTTTCTTACCTGCCTTGAACTCTTTTG
16 FANCA	GGACCCAGAGGTGCTGAGAT	GCCACTTCTTGGTGTTGCAG
16 FANCA	CAGCATGAGCTCCCATCCAT	ATCTGGTTTCTTCCTTCTTCTCACAG
16 FANCA	ggccAGGGTAGCTCTTTTCAA	GGATTGTGTGTTCTGTCCTCATTGTAT
16 FANCA	GCCAGAAATCAAACCCGTCTGA	CTTTGTTGCTTGAAGCGGTGT
16 FANCA	CACAATGCCTTGTACGTGAAGATG	TTTCAATACGGCATGGTTAAGAACCTAA
16 FANCA	AAACAGAAATTGAGAGAAGGCTCCA	GACAGCAGCGTTGAGATATCAAAG
16 FANCA	CGTGTTGATGCTGAGCTGAAT	CCCTAACTAGTTTCTTCCTTCGCA
16 FANCA	GCCCAGGTGGTAGTAGGTGTT	CCAGGAGCACTTCCTCTTTGA
16 FANCA	GAGCCGTCTGCGGAAAATC	TGTGCCAGCATACTGCTCTTTT
16 FANCA	CTCACTCACATGACAGAGAATCAGG	CCCTCTGCGTTCCTGTCATAT
16 FANCA	AGGTCACTTACCTTGAACCAGTCT	TGCTGTGACTATGTCATGGTTCTTAAC
16 FANCA	CAGGTACTCTGTTGCCTCCAT	CTTCCCTGTTAGAGTTTGCTCAGTATTT
16 FANCA	GGGAGAACATACTGTGTGCCAAT	GGTTGGGAAAATAGGACATAGCTTTAGTA
16 FANCA	CGGAGACGAGCTCATGAGTC	ACCTCCTGCTGACGTCTTTC
16 FANCA	GCAGCCATCAGGTTCTGACA	GGAGGTCTTTTTGCAAAGCAGAAT
16 FANCA	ACGCGGCTTAAATGAAGTGAATG	GGCGATACTGAGGAGGATTTTATTGTA
16 FANCA	aatacaGTGTGTGCTGCTAACCTT	CTGTTCTAGGCAGGACTTCCAC
16 FANCA	CAGGGAGAAAGTGCTCATGGAT	CAGTCTCAACCCAGCTCTCAC
16 FANCA	TCTTGGCTCTTTAATTTGGCAGACA	TGCTGACATCTGTTTTCTGTTCCT
16 FANCA	GCTGTGCCGGAACATTCTTTG	CTTGAAGCTGGTCCAGCTCTT
16 FANCA	AGGTGAAACTGTGCTTGTATCCC	TTTCCAGCAGTCTGTAGCCATTT
16 FANCA	CCCTACACACACTCTTGACCA	TTGGTTGGCCATTTGCAAGAAG
16 FANCA	GAACCTCCTGCGTTTCCAGAA	GTCATCTGTCGCACATTGGTTTG
16 FANCA	CTATGTCCTATTTTCCCAACCAGCTT	GGTATTCTCTCAGCCGGGATG
16 FANCA	CAGATCTGTCCCACGCTAGA	AGGCATTTTAAACAGCAAGTCTTTGG
16 FANCA	AGAGGACACCTTGGCTGGTAA	GCCTTATTTAGAATGACAGCACAGGTAAT
16 FANCA	CACGAGCTCAGAAGCAGGTA	CTGACTTGAAGTTTTTGTCTGTTTCTCC
16 FANCA	CCCCATGAAGGAGAGCCTCA	TGAATGGCTAGTACTGATGGTTTCAC
16 FANCA	TGAGAGACTGACAAGGAAAGTCCT	GATGCACTGTCTGCTTCTCTGT
16 FANCA	GGCAACAGCAATCCCCAAAATG	ACACACGCTTGGCAGTGTAAT

16 FANCA	ACCTCTTCAGAGGATCTGTGGAA	CACAGTTTGTGCTGGGATTGG
16 FANCA	GTCCCGTCAGAAGAGATGAGG	CTGTGGTCTGAGGCCTAGTG
16 FANCA	CTGAGATGGTAACACCCGTGA	TCGACTTCATACTGGCCAAGTG
16 FANCA	ACCAGAGCAGAGGTCAAAATTAAGG	TCTACTAGTTGTGGGTCCTCACAAA
16 FANCA	TCAACTCAAGAGTCAAAAGAAACTGGA	GTTCTCATTCTGTGTCTTCCCTGAA
16 FANCA	CCGACCTCATCCTCACAGGAA	TGTCTGCCAAATTAAAGAGCCAAGATAT
16 FANCA	GCAGCTGCTAGAGGCCTTTT	TTCCTTTCCTCCCTGTATGGGA
16 FANCA	ACTGACAGATCGGACGGACA	CCAATACAGCCCCTTCTTTCCA
16 FANCA	GGAAACCCATCGCCTGAGAAA	GCAAAGTGATTGACTGTGACAGTTC
16 IL21R	GTCATTCAAAACAATGGCTGGTGT	TCCCCGTGCTTCATGAGAAAG
16 IL21R	CCATGAGCTGTCGCTGCAT	TTTCCCATCCTTCAAGGCTCAG
16 IL21R	ACCCTCCACTGTACGTCTCTT	CCTGGCTCAGCTCTGTGATG
16 IL21R	AGGACTTCCTCCCATCACCAA	GTAGAAGGCAGGGTCTTCGTAATC
16 IL21R	ACAGTATAATATCTCCTGGCGCTCA	GCTGCACTGATCCCCACATT
16 IL21R	CCCTCTGGTGATGTCAGGGT	CTCCGAGGAGCTCATCACA
16 IL21R	CCATATGGCCTCTGGTAGAGTTG	CTCCAGAAGGCAGGAATGAAGA
16 IL21R	GCTTCTCCTCCTGCTTGTCATAG	TGTTTTCCAACCCTGCATGTATCATA
16 IL21R	CATGACCGGCTGCTTTGTC	GAAAATGTCGTCGGCCATGAA
16 IL21R	CTACACCTGCCACATGGATGTAT	attgAGTGCCACCCTAAGCC
16 IL21R	GAAGCCTCCTGGACAGACTAAA	CAAAGCCACTGTCAAACGTGTC
16 IL21R	ACCTTACCCTCATCCTGTGCTA	GGTGGCAGCTGTACACCTC
16 IL21R	CTGGCCGGCCTGGATAT	GGTCCAGGGCTCGAAAGTG
16 IL21R	GAGCTGGGACCCTGGAG	CTTGGGCACACCGTCAGA
16 IL21R	CTCACGGAGCTACAAGAACCA	GCATCTAGCACAGTCACTGTGT
16 IL21R	CATACGGCCTGGTGTCCATT	GCATCCAAGAGTGGGTCCTCTA
16 IL21R	CCCTGGACCTGGATGCTG	CAGTCCTCCCCATCTGCAA
16 IL21R	CCAGTGGGTGGTCATTCCTC	GCTGCAGGTGTCTTCACATCA
16 IL21R	GCCTCTCTCCCCACTGAC	GTGAGGCCCGGCTACTTAC
16 IL21R	CCTGACCTGGTGCATCCTTT	GCACCTGCAGCTCATAGCTC
16 IL21R	CCCTGGAGTTCCGCAAAGAC	TCCATCCCAGCTTCACACCTA
16 MAF	GAGCACTTATCAGGGTGGCTA	GTACAAGGAGAAATACGAGAAGTTGGT
16 MAF	CTCGAGCCGTTTTCTCGGAA	GTCCTGGAGTCGGAGAAGAAC
16 MAF	TGCTTGAGGTGGTCGACTTG	GTGATCCGGCTGAAGCAGAA
16 MAF	GGCATAGCCGCGGTTTTT	GCCTGCACTTCGACGAC
16 MAF	GGGTAGGTGGTTCTCCATGACT	CATTTTGGAAGCCCCAGCATC
16 MAF	TCATTTTGTGAACACACTGGTAAGTACA	CCCTTTACGCTGCGTTTGAT
16 MAF	CGCGCGTAGCCATCGAA	GCGCACCTGGAAGACTACTAC
16 MAF	GGGTAGCCGGTCATCCA	CTCCACCCCCATGAGCAC
16 MAF	TCCAGGTGCGCCTTCTG	AGTGAAAAAGGAACCGGTGGAG
16 MAF	GCTGATGATGCGGTCGGT	TCAGAACTGGCAATGAGCAACT
16 MAF	GGTTCCTTTTTCACTTCAAACTTCATCA	CTCTCCTGCAGCCCATCT
16 MMP2	GCCTGCTTTGGTCAGTACTGT	AGTCCAAAGAACTTCTGCATCTTCT

16 MMP2	TGTTTGTGCTGAAGGACACACTAA	TGATCTGGTTCTTGTCCCACTTG
16 MMP2	AACTACAACTTCTTCCCTCGCAA	AGCCTTCACAAAAGACACCTCAT
16 MMP2	CATTAAGGTCAGCGTCATGTCATTG	CGCTGGTGCAGCTCTCATAT
16 MMP2	TCTGGAGCTGCAGAGAGTCTAA	GATGCCATCAAATACAATGTCCTGT
16 MMP2	CTGTCACTCCTGAGATCTGCAA	CGAAGAGTGAGGGAAAAGAATAATGGT
16 MMP2	CCTTCACTTTCCTGGGCAACAA	TGTCCAGGGTCTCTGTCCAAT
16 MMP2	CAACACACCCTTTGCTTCCAC	GTCTCCAGCAAAGATGTATGTCTTCTT
16 MMP2	CCGCCTTTAACTGGAGCAAAAAC	AGCCCACCAACCTCTGAAC
16 MMP2	GTGGCACAGCTAGACGCTAA	CTCCCAAGGTCCATAGCTCATC
16 MMP2	GGGAGACTCCCATTTTGATGAC	TCACCCAGATGTGTGACTTTCC
16 MMP2	CTTCTCTCTCATCTCTCTTCCTGTCT	ACGGAAGTTCTTGGTGTAGGTG
16 MMP2	GCCCTGATGGCACCCATTTA	CTCCATCCCAGGCTACATGAC
16 MMP2	GGGAGGAAAGTCACACATCTGG	TGTTGTACTCCTTGCCATTGAACA
16 MMP2	GAGTACTGCAAGTTCCCCTTCT	GGGACTAGAGTGGATGCTCA
16 MMP2	ctcacaTGCAGTTCTACCACCTC	GATCATGATGTCTGCCTCTCCAT
16 MMP2	ACTGCGGTTTTCTCGAATCCAT	TGTCCTATCTAAGCCGCCCTT
16 MMP2	CTGCTGGTTCACTGTGTCTGT	CCTAGAAAGCGGAGACAGTCCT
16 MMP2	GCCCATGGAAGCATGTCTCATT	GTCATAGGATGTGCCCTGGAAG
16 MMP2	AAGGACAGCCCTGCAAGTTT	CAGGGCAGAAGCCATACTTCTT
16 MMP2	GCACCACTGAGGACTACGAC	AGATGTCTTAGCACCCCTGTCT
16 MMP2	CCAGGGAGCGCTACGATG	CTGCCAACTCTTTGTCCGTTTT
16 MMP2	CCGTCGCCCATCATCAAGTT	GAGAGACACCCCCATCCTTTG
16 MMP2	CTTCCCTATGCCAGGCAGAAA	CAGGTAATAGGCACCCTTGAAGAAG
16 MMP2	TTTCTCTATCCCAGGTCACAGCTA	GTGTATCGAAGGCAGTGGAGAG
16 MMP2	GCTGCAGGGTGACTGAAGATG	GGCCTCGTATACCGCATCAA
16 MMP2	CCTGAGCTCCCGGAAAAGA	GCATAGGAACTGTGGTTTGAGAGG
16 MYH11	CCAGAGCCACGCGTCATAC	GACCTGGTTGTTGATTTGGACAA
16 MYH11	GGACTTGGGTGTTCAATTCTCCAG	CAAGAATATGGACCCGCTGAATG
16 MYH11	cagccCTCCCAACACATTTCT	GAAGCCTCCGTGCTACACAA
16 MYH11	TCCAGGTTGGACACGAGTTG	AGGTTCCAGAAGGAGATCGAGAA
16 MYH11	GAGCAGGGAAGTCACGTTGT	GCCTTGGTTTCTAAGTTTCTAGTTTCC
16 MYH11	TGAGAAGTACCGCTCCCTCA	GTTGTGGAGCTGGTGGAGAAT
16 MYH11	TCCTCGTACTGCTGGGTGA	CCTGGACGCTGTCCTTGTAG
16 MYH11	CTTTCCCAACCGTGACCTTCTT	AAAAACTTCATCAACAGCCCAGTG
16 MYH11	GGGACCCAGACGAGTCTCTT	GTGGTCTGATTCTGACCCTGTT
16 MYH11	TCATTACCTGTTCTTTTGTCTGAGCTT	TCACGATGTTTCTTTTCTCCATCCAG
16 MYH11	CAAACTCCACATTCCTGGCAAG	CGACATGTACAAGGGCAAGAAGAG
16 MYH11	CGTAGATGTGAGGCGGCATC	GTCTGGAAGTAAGCTGGGTTCT
16 MYH11	CTGCCCCTGTGACACCTTA	GTATGCAGAGGCTGAGGAGATG

16 MYH11	CTCCATCTCATGCAGTATCTCCTC	AGGAGAAGAACCTGCTACAGGAA
16 MYH11	CCTCTGCATACAGCTCTGTCTCT	GAACAGAAGCACTCGCAGGTA
16 MYH11	AGAAAGGCCCCTGTTTGTGATT	GGAGGAGCGAGATTTGAAGATCA
16 MYH11	GGAAGGCCATGATGACATCGG	AGGAGCTCTTCCTTTCCTTCCTT
16 MYH11	ACCACCCTCTTGTCCCTCAA	GGCAACATGGAGGCCATGA
16 MYH11	CCTCCATGTTGCCCTgct	CCCTGAGGCATGACTCCAA
16 MYH11	AAATCTCTTGGTAGCTGGTTTACCTTC	GGAGAGGATTAGTGACTTAACGACAAAT
16 MYH11	TGGTTTACCTTCCAGTTCTGAAATCAT	TTCTCTTTCCCCAAAAAGGAACGA
16 MYH11	CGTTAAGTCACTAATCCTCTCCTCAAG	AACTCTAGTTGTATTTGTTATAATGTGGGT
16 MYH11	GGCCAGACCTTGGGACTTA	CCACTGCCCTCTTTGACCTTT
16 MYH11	GCCCATCTCAGACAACCAAGAC	GCAACATATGAGCGCCTTTTCC
16 MYH11	TTCACGCGGGTGAGTATCC	ACCAGCCTGGCTTATGTGAAAAT
16 MYH11	GGTAAGAACGGTCCCACCAA	GTGACTTCTGCTCTGTGTTTCAAG
16 MYH11	GAGGCTGCTGATGTCACTCTTA	AGAAGGCCCTGGATGAAGAGA
16 MYH11	GTTTCTGCCTCATCTCCTGGAC	AACTGCCATTCACTGTGTTCCT
16 MYH11	GGCTGATGCACGATTTGCTTT	GAGCTGGAGGCCCTAAAGAC
16 MYH11	CTGTGCTGTCCAGTGTGTCTT	AGAAGAACAATGCCCTGAAGAAGAT
16 MYH11	CTCCTGGAGGTCTGAGATGTG	ACTGTCTGGTCCCAGAAAAGTTTT
16 MYH11	AGACCGGTTGGCTAAATCATGG	AGATGATGTTCCCGTGTCTTTCAG
16 MYH11	CATGCCCCAAGCTCCTAGT	GCTGCAGCAAAGAAGAAGCTG
16 MYH11	GCCTGAAGCTCCAGGTCTTTC	GGGCAGCACACATCTCTATTCC
16 MYH11	GAGCCCCCAGTGCTTTTCT	GAGTCCCAGCGCATCAAC
16 MYH11	GCAGCTTCCTGCGGTTG	CCTAACCACCCCTCCAACTCT
16 MYH11	AGAGAAGTTGAGAGGACCCATGA	CTCAACGTGTCTACGAAGCTG
16 MYH11	TGGTCTTGCAGGCTGTTCC	GTGTCCTGCCACATCATCCA
16 MYH11	GCCTGTCGCGGAGAGTTG	GTCGCTGAAGCAGAAAGACAAG
16 MYH11	GCAGCAAGATTTCCTTCAGCTT	AGGAAAGCACTCAAGATGCCA
16 MYH11	GCTGTTCCTGTAGCAGGTTCTT	GAAGGCAGAGAATGAGCTTAAGGAG
16 MYH11	GTACCTGCGAGTGCTTCTGTT	GCCACAAAAACAAATGATGGGCTA
16 MYH11	GCCACACACGTGTACAAGGT	CACCATGTTCATCCTGGAGCA
16 MYH11	CTCGCGCTGGTACTCCTC	GGCTCCTTGTCTTCTGACTTCAT
16 MYH11	ctcctCCCCACAGAACTGG	AGGAGCTGCAGTCCAAGTG
16 MYH11	CACCTGCAGCTTGTGGACT	CCAAGCAGGAGGTGGAACATAA
16 MYH11	GCGCCTCCAGCTTCTTCTTC	GTTATCCCTCCCCTCTGCTTC
16 MYH11	CAAAGTCATTGGTCATCAGGAAAAAGT	CATTGACCTTTGTGTCTGCTCTG
16 MYH11	GTTTTCTTGCCGTGGTGCAAA	GGACGTAGAGTTATTGAAAATGCAGATG
16 MYH11	GTGTCCGTTTCCTCCTCAGAAC	TCCCAACCATGAAGATTTCTTTGCTAA
16 MYH11	GATAGCCTTCCCCACATGGAA	CTTCGTGCGCTGCATCATC
16 MYH11	CTCACCCTCTTCTCGTGGTT	GATGGCCAAGATGACGGAGA
16 MYH11	CATGCCCTTCTTGGTCTTGGA	GCTGAGCCTCTGGCCTATTTAG

16 MYH11	CCCTCTTCCTCCATTCAGTTTCCTA	CCTTTCCCTGGCAGACAACAG
16 MYH11	CCACTGCGAATGAGCGAAT	CCACGAGCAGATCGCTGAC
16 MYH11	GCATCTTGAGCTCTGCGATCTG	CCCTTCCTGAAGAGCACCTTG
16 MYH11	GGATGATGTGGCAGGACACTC	ACCAGTCTCTTGGCACCCTTA
16 MYH11	ACCCCATTTCTACCACCAGCTA	CGCTGACATTTGTTTTATACTGTGTTG TT
16 MYH11	CAAGGTTCTGCCACTCTCAGAC	AGGACAAGACTGAGTTCTCCATCA
16 MYH11	TGGTACCTTCCCAGCATAATGGA	CTGGACGAGGAATGCTGGTT
16 MYH11	AGACTTGTCCGTGGCTTTGG	GATGCCCATGCGTTGTGTATAG
16 MYH11	AGCAGTTCCACTTACGTGCAT	AGCATGGAGAAGCTTTAGAATGGG
16 MYH11	CACCAAAGCTTTTCTGGAAAATCCA	GGTTGCTTTCTCCAATAACTAGATGTG A
16 MYH11	GGAAGGCTCAAGCCATCCAAT	GAATCCCTAACTCCCTCCAAACC
16 MYH11	CTGGTGAATAGCACAGAGGGT	CCCTTGAAGAGGCCTTGGAA
16 MYH11	CCGCTCGAGTTCCTCTTTGG	GAAGTCACCTCTGGGTCTTGTG
16 MYH11	tCACTATGACTCCTGCTGTCCAT	AGTCCAAGTTCAAGTCCACCATC
16 MYH11	CTCCTCCAGCTGTGCAATCTT	CGGCCCAGAAGAATGAGAGTG
16 MYH11	CTCCGGAGCTCCTTGTTCTG	GGTCTCAGTGCGCTCTCTTG
16 MYH11	CACCTCCTGTCCCAAATGTGAA	CCATGAAGGTGATTCAGAGGAACTG
16 MYH11	CAGTTCCGCAGCTTGAGGTA	CTGTGCAGGTTCAGGATGCT
16 MYH11	CGCATTGGGCAGAAAAGAAATG	CCTTCTTGTTTGCTGATGGTAACCT
16 MYH11	CCTCTGGGCAGGAGATTGGTA	CCTGAACTGTGTTTTCCTGTTGG
16 MYH11	ccggccCCTACTCACTTTTG	CTCTTTTCCCTCCACAACAAAGGA
16 MYH11	CTCCTCCAGCTGTTCTTCAAGG	CTACAGAGTTCTGGGCATCTTCAG
16 MYH11	GTTTCCACACCAACCATGAGAGT	CAACATGCAGGCGCTCAAG
16 MYH11	GCTTGGAGATCCCTTTCGAACT	TCCATGAGCTGGAGAAGTCCA
16 MYH11	CTGCGTCTTCATCTCCTCCATC	GGTGGGTTTCTCAATCGTCTTCA
16 MYH11	CCATTGGAGAGGAAGGTGTAGT	CCTTGACCAGATCTCGGTCTCAT
16 MYH11	CAAGCTACCCTCCAGACTCAAG	TGCTTTTGGAGGGCTTCAACA
16 MYH11	CCTAGCAAGGCGAGGCTTT	CCTCCCTGTTGACTCATGCAG
16 PALB2	ACCAAGCAGAGCTTCTTGCAT	ACTGCTTTAGTGCAAAGTACTGACTT
16 PALB2	CCTCCACGGCTACTTTCCTCT	CAGGAAAGTGAGATTCTAAGTCAACC T
16 PALB2	GTTGCTTCCAGGCTAAGACTCTT	ACTTACCAGCAAATGAAAACCAAAA CTT
16 PALB2	CAAGAGTGTCACTGGGAGATTTTAAA GA	GCCCACAAGTTCTAATTTAGAGGCAA AT
16 PALB2	GCTGGTAAGTTATTGTAGGTGAGTTC AT	GACTGTCTCTACAGATAACCTCCTTGT A
16 PALB2	CAGTTGGCCACTTTTACTTATAGCTTT	GTAGCAGTGAACTTACTACTCACGA
16 PALB2	CCTCCAAACTTACAGGTGAAGTAAAT CT	CCTAATTTCACCAGGGCGACTAC
16 PALB2	CCGCTATCTGATAGAGTCTGTAAAGG AA	CAGATTCTCCAGAACCAGTTACAGAA AT
16 PALB2	AAAAACGAGATCCTAGTTACCCAACT TT	ACAAAGAGGAGGCTAGTTAGTAGCA
16 PALB2	CTTGTTGATCAGAAAGGGTCCCA	GTTACTCCTCACATCACCCCATT

16 PALB2	GTTTGCACAGTGCCTTTCAGA	TGTTTCAGCTCATTGTGATTAACCCT
16 PALB2	CCACGCTGAGAGTCGTCTT	CCTGACATACTCTTGACAGTCTATTTGG
16 PALB2	CAGGCCTAAAACCCTGGGAA	CGGCTGCTCTTTTCGTTCTGT
16 PALB2	AGAGGCCCAATATATCCAGAAAATTGTG	TTTTGTGAAATGGTCGGGTACAGA
16 PALB2	TGTCCAGCCAGCAAATGAGAG	CAGCAATCTTGACTTCTGGAACAAT
16 PALB2	CGAGAAGTAAGTCCCAAATGGCA	GTTTTGGGAACATGGTTTTGACCTT
16 PALB2	TTGGGCACGCTAGAGGA	GCATTCTTGAAAAGGGAATACAGCAA
16 PALB2	ACTTACCTGAAGGCGGGCTA	CCTTCCCTCTGACTCCACCTTT
16 PALB2	CAAGTCATGCTGTTTACATTCACTAAGG	CACCCATTGAGTCATTCACTTTTAAAGAA
16 PALB2	GGCATGTGTTTCTACAGAGCTGATT	ACTAAACAATTCGACAGTTCAGGCA
16 PALB2	TTGCAGGGTGGTATGTGGTTT	GCCCTCAAGGCTCCTATGAAAAAG
16 PALB2	TTCGTCCAGCAACTTCTGTAGATG	CGGGCCTTTCTTCATCCATATTACTTTAT
16 PALB2	GCGCAACCGTATTTAAAGGAGT	CATGGATACAGAAATGGAGGACTTAGA
16 PALB2	GCAGGCATAAGTGAATGGTCTAGAT	GCTGTTTTTGTCTCCTCTAGCGT
16 PALB2	TGTAAGACACGAGACACTGGAAGA	TGGGTAATGCAGGCAGACATTAT
16 PALB2	GTGGTCCCAGCCAGTCATTAC	GTTTGTTGGAAGAATGTGATCAGCTTATT
16 PALB2	TTGCATGGTCATAGCTCCCAAT	TGTAGTTTCTCTTTGGAAAGCTCTGG
16 PALB2	GTTTTTCCCACTGCCAAGCAT	ACAGAACTGTTGCCATTGTGTCA
16 PALB2	AGAAAAGGAAGTGCCAGGCAAATA	TTGTTAACAGGTCCAAGGAAGAAGTC
16 PALB2	TTCGTGCTGATATTTGTGTGAGGT	AATGACTTGTCTAGGAAGGCAGTTG
16 PALB2	AGTTGGTGGAATTAATACACTGTCTTCA	AAGGAACAGGAAGAAATCAGTAGCAAA
16 PALB2	TCAGTTACTGGTGATCTAGCAGGATT	GAGGACATTTATTTCACAGGAGAGAGAC
16 PALB2	GTATCTTCTACCAGGTGCTTGGG	CAATGAGGAAACTGACCAAAGTGAAA
16 PALB2	AGTGAATCAGTGCCAAAGACACA	CCAGGAGGATTACCTATACAAAGAACAG
16 PALB2	TGCATGTGCCAGACATCCTAA	CACGAAGCATGTCCAATTGCC
16 PALB2	GGGAAAATGTTCTTGGGTGTCAT	TGACAAGTTACACATCAAAACCCATCT
16 PALB2	TTCCTGGTAGAACAATAAGGTCCTCT	TGAAAAAGTGAAGTCCTGCTCAGAA
16 PALB2	CTCAAAGGGCTCCACTGGTTT	TGATGCTTTCACGGCTCCAT
16 PALB2	TTTAAACTCAGCATTCCATCCCTATGAA	AGATTGTCTGTTTTGTTGGGTTTTGTTAC
16 PALB2	CCTGCACTTAAAACCAGCTGACA	GCTGCTTTGTTTTATTTAGGTTCCAGTAT
16 PALB2	ACATCAGGCACTGGAACTATCTGTA	GCCTTCAAATGATGAAAATTATCCTTGT
16 SOCS1	TCAGGTAGTCGCGGAGGA	CGAGCTGCTGGAGCACTA
16 SOCS1	GGATGCGAGCCAGGTTCT	CCGCGAGAGCTTCGACTG
16 SOCS1	GCTCCAGCAGCTCGAAGA	GTGGGCACCTTCCTGGT
16 SOCS1	GCTAAGGGCGAAAAAGCAGTT	CGTTCGCACGCCGATTA

16 SOCS1	CTCTGCTGCTGTGGAGACTG	CCAGCTCACCTCTTTGTCTCT
16 SOCS1	GCGTGATGCGCCGGTAA	CAGAACCTTCCTCCTCTTCCTC
16 SOCS1	GAACGGAATGTGCGGAAGTG	GGTGGCAGCCGACAATG
16 SOCS1	CCACATGGTTCCAGGCAAGTA	CGAGAACCTGGCTCGCATC
16 TSC2	CACTTCAGGGACTTCTTGGCA	GAAGGTCTTCGTTGGAAGGGTAA
16 TSC2	TGTTTGCATGTCTGAGGGATGT	CTGGTCTTCTTCTCAGAGCCT
16 TSC2	CTTCTGCAGGGAGCCAGA	GCCCCACTCACCTGCTT
16 TSC2	CGTCCTGCTGCAGTGCTT	AGGCTCAGCTCTGAGGGA
16 TSC2	CCCTCTTCTTTAAGGTCATCAAGGA	TGATCGTCAAGGCCAGAGAAAC
16 TSC2	CAACACCGGCTCTTCTTTTGA	GGCTACAGAGGACCCCCTAC
16 TSC2	GGCTCCTTCCTCACCCGATA	CGCTACCTGCGGACCAC
16 TSC2	GCAGCATCAGTGTGTCTGAACAT	GCAGACGATGAGGTCATGCAA
16 TSC2	CCACGCCTCCCAGACTTA	GCTCGGCTGGAGTCTGTG
16 TSC2	CCCTCCGTCCCATAGCAAA	TTATTTCACTGACAGGCAATACCGT
16 TSC2	GGCTCGATACGTCTTCTCCAA	CCAGGTTTTGGTCCTGCCA
16 TSC2	TGTGGGCGAGTTCCTCCTA	GAGGAAAGGAAGGTGCAGTCA
16 TSC2	AGCTCTGTGCCCTGTGTG	AGCACGTCCAGCACCTT
16 TSC2	GAGCTTTGGCCCTTGGTGATA	GTTCTCATCTGCAGACCCCAA
16 TSC2	GCAGGATACAGACGTCCCTCA	AGCCGTGAAGTTGGAGAAGAC
16 TSC2	GTCTGGACATGATGGCTCGATAC	CTAGAAGACAGGGAGCGTGAAA
16 TSC2	GCCCCTGACACGCATTGT	GCAAGGGACACTGGAGCTG
16 TSC2	GCCTGAGGTGTCCTGTCTC	TGAGATGTGCGTGAGCTTCA
16 TSC2	GGAGATGCCTGACATCATCATCAA	GTTACACAGCCCGAACCTCT
16 TSC2	CTCTCTGCTCGACCTGTGT	GCTTCCCTCTGAGAGGCGATA
16 TSC2	CGGTTCCTGAGGAATTGGAAGT	CTTTCGGACCTGGTGGTCTT
16 TSC2	CTCCCACATCCCCGAGGATA	CGGGTACAACGGCTCTCA
16 TSC2	GTGCTACTCTCGGTCCCAA	GATGGACAGGACGATCTCATAGGA
16 TSC2	CATGTCCGAACGAGGTGGT	GCTGAAGGAGCCGTTCGAT
16 TSC2	CGTGGGACATTCTGCTGAACAT	CCCAAGGTACCATCTTCTGAGT
16 TSC2	CCTGGCGTGACCACCAA	CCTGTCCCACCAGCTCAC
16 TSC2	GTACTGGCCTCAGGCCAAA	GCCATGAGGGCGTTAGACA
16 TSC2	CTAGCTTCCGCCTCTGTCT	GCTGGTCCACACTGCAAGG
16 TSC2	TTTCTCCTCGGACATCAACAACAT	CTCACCTGTGTTGGAGCGA
16 TSC2	CAGCCCTGTACAAGTCACTGT	GCCACATTGCCGTCACC
16 TSC2	TGCGCTATAAAGTGCTCATCTTTACTT	CAGAGGCTCGCAGTCTTTTG
16 TSC2	CCAGCCCCCTTCTCATCTCA	CTGGCCTACCGAGAGACAC
16 TSC2	CAGGGCTCTGTGTGCCA	GCCCTGTCCAGGCACCTA
16 TSC2	GCACTGGCCCCTTTTTCTTCTT	CTCTCTAGTAGCTCAACTGGATAAACCT
16 TSC2	GTGTTATTGACGTCATAGAGTGACTAGA	GCACTGACCTCTATGTCCACA
16 TSC2	CTCACTGTCTGGGTGTGCTC	GCCGTACCTCTTGGGTCTCT
16 TSC2	GCCCGGAGTACTAGTCTCAAC	CACAGGACCCATTTCCACTCA
16 TSC2	TGTGTGTAAGTCCTGGCCTTC	AGGCCTCGTGCAGAACAC
16 TSC2	CAGGTGCCACCTGATAGTGA	CTGAGGGAGCCCCATATTCC

16	TSC2	GTTCTTGGAGAGCACATCCTCA	GTCGCCCTGGGCCTTAC
16	TSC2	cttgtcTGCCTCAGGGATCAG	CGTGTACCTGTAGGAGCCAT
16	TSC2	TCGCCATCCTGTCCAATGAG	CCTCACCACACACGTCCAG
16	TSC2	CCAGCCGGACAAGGTGTA	GGTAGCAGGACTGGATGGGA
16	TSC2	CCGTCTCCCTCTCCACCA	CTTCCAGAGCAGTTTCAGAAACC
16	TSC2	GCAGCCCCGTCTGTGTC	GGCTCTGAGCCACACTCA
16	TSC2	GAGGGCCTCAGCACTGG	CTCACCGGAGTCATTGTAGACA
16	TSC2	GGCAACGACTTTGTGTCCAT	CTCCTCGTGACCGAAGCTC
16	TSC2	GGAGAGGTTTCATGCCTGGATT	GTTTTGTCCAGGTAGTTATGGTAAGA GAT
16	TSC2	GGTTCCAGTGCTGACAGCATTA	GGCAGAAACTCGTTTATGAGAAAAT GT
16	TSC2	CATGGAGCTGACAGGTGTCTA	CAGGTTGCACTCGTAGTCCAG
16	TSC2	TGTCCACGTGATCGTCACC	GCTCTTGGCGCCACACT
16	TSC2	GCAGAGGTGTTGCTCAGATGT	TCCCAACAGAATCTTAAACTTCTCCTT C
16	TSC2	CCAACAAGCAAAGATTCAGGCTT	CAAAGACACAGGTAGCTCACTCA
16	TSC2	CCCTGGGATGGAGGACAGAT	GGAGCGACTTCTCCTCCTG
16	TSC2	GTCCTTTCTAGTCGTCCTCAGTCT	AGAGGAGCTGGACTTGCTCA
16	TSC2	TCTGTGGACCTCTCCTTCCA	AGCACTTTCCCCGTCCAG
16	TSC2	CCCGGTCACAGTCAGGGA	TCTTGCCCCTGCGTGATG
16	TSC2	CGAGGTTACACCATCTCCGACT	CCGCAGGAGAGGTGTCA
16	TSC2	CTGTGTCATCGTGCCTGGTA	GGGTCTCACAGGCCTCT
16	TSC2	GTGACGGGTTTGGACACACT	GAATTTGACCAAGTTCACCAGCA
16	TSC2	TTGTCCTCGGAATTCCTTCTGG	AGGACCATTATCAGAACAGGATCTCA A
16	TSC2	CCGTGTTGGCCTTCAGAG	ATGGCGAACACACTGGCATA
16	TSC2	CCGCACCTCTACAGGAACTT	TTTCTGCCGCAAGGCCTA
16	TSC2	CCTGTGCGTGGGATTCTCTTC	GGCTCACCCGACATGGAAC
16	TSC2	GCATGTGAGACAGACCAAGGA	CTGAACACTGGGACCAGCA
16	TSC2	GCTCACATTCCGTCTCTCTGG	AGAAACCGCCTTACCTGGTAAAA
16	TSC2	CCGACATCTGTGTTGCCATCA	CTGGAAAGGCCTAGAAATGCCA
16	TSC2	GGACAAGGGTGCTGTCTTAGG	CTGTGCTTGTAGTGGAGCTGA
16	TSC2	GAGATGCTGGTCAGCCACAT	TCTGCAGCTTCCAGGAACC
16	TSC2	GATGGCCAGGCTCTGTGTT	GCAGCAGGATTGGCTTGTTT
16	TSC2	CCCTTCTTTGGCGACGAGT	GCTGCCACAGGGAGCTTAG
16	TSC2	CCAGTCCTCTGCCCTCTTCTT	GTCTTCTCCTGCACAGGAACC
16	TSC2	CGAAACCTGAGAAGGCCTCA	CTGTGGGTGACTGGCAGAAA
16	TSC2	CTTTCACCATCCTCTTCCTGACA	GTAGTCGCAGACGCAGTAGG
16	TSC2	CCCAACAAGGATGGAGTCGTG	CCTGGGACATCCCTCAGACAT
17	9-Sep	GCGGCTAGCTCTGCACT	GAAGTCCGAAATGATCCCATCCA
17	9-Sep	CTTGTGTGACCTTTGCCCTTTG	CACAAACACACCCATGAGCTG
17	9-Sep	CGGTGTCTCCTTGGTTGTCAT	CCTGGTGTGTCAATCACTGTCA
17	9-Sep	GAGAAAGGCGTCCGGATGAA	GGAAACCTCTAGGGCTGACTC
17	9-Sep	TCCCGCACATGTGTAACCAAT	CCTTCGATGGTACCCCACTTG
17	9-Sep	AGAGGATCCTTGGGAGGAAGAC	GCAAGGCAGTGGATGAGGTTC
17	9-Sep	GCTCTAACTCCTCTGCTGTTCCT	CCACGTAGCCGAAGTCCAC
17	9-Sep	CTGCATCACGGAACGAGAAG	GTTTCCGGCATTGGTTGAGT
17	9-Sep	CAGAGTTTCTCCACTGGGACA	CCGCTGTAGGGCCCTCTTA

17	9-Sep	GCCCATCTCTCTCCCTCCTTAT	CCCTGGGAACAACATCCTAGTG
17	9-Sep	CGCCCACCTCACTGACC	GGGCTTCTGGCTCCTTCTC
17	9-Sep	TGTGAAGCGCCTCAACGA	GGGACGGAAATGACTTGGG
17	9-Sep	CAGGGCATGCATTTCATTGGA	GCAGGTATTTCTCGTACTGGTCA
17	9-Sep	GCAGCCCATCATGAAGTTCATCAA	ATGAGGACGCCGACACTG
17	9-Sep	CCTCGCCGCCACACTTT	AGGCACTCAGGTGAGCAG
17	9-Sep	GGCCTGGAAGGCTGACA	AAATGGCCACATCCCCACA
17	9-Sep	CGGACACTGTCCTCCTAGCAT	AGGCCTTCCCAGAAACTTGAG
17	9-Sep	GACCTGAAGTGCCCCCAT	CAGTCTCCTTACTTTGCCTCGTA
17	9-Sep	CTGCTCCAGTGTGCATTGT	GGTGGCACTCACCACACC
17	9-Sep	CAGATGAGCCTCACGCACAT	GGGCAAGTAGAGATGGAACCCTA
17	9-Sep	CTCCCGCCGCTGCTAAATA	ATGCTTATTCCCCCAGGCATAAAT
17	9-Sep	AGGCACTGCTGAATCTCAGAC	GAAGAGGGTGTTGATTAAGGTGGATT
17	9-Sep	GCAGAGCGGCTTGGGTA	CTGACCGTGCGTGATGGA
17	9-Sep	CCCTAGCCATCCATTCACCAATT	CCACAGTGGCTCGGAGTAG
17	9-Sep	CCAAGAGGGTGGAGATCCAGAT	GTGTAGCCTCAGCCACAGG
17	9-Sep	AGACCTTGGAGAATTCAGAGCCT	CCCATTACCACAGCATCCAAGA
17	9-Sep	GTCGAGACACCCAACTCCAC	GTTGGCTTAGGGAGTCCACAT
17	9-Sep	CTGGGCGTGAAGAACTCAGAA	CACCTGCTTGGACGAGATGT
17	9-Sep	CGCACTGAGCTGTCCATTG	CGGTGGGCTGACTCCTG
17	9-Sep	CGGAGATCACCATCGTCAAACC	TCAGCAGGCTTGGGCATC
17	AURKB	TCAAGGCTTGCCAGGACTAA	GCGCAGAGAGATCGAAATCCAG
17	AURKB	CATCAACCCATACTGCAGGTG	ACTTGGCTCGGGAGAAGAAAAG
17	AURKB	CTTGAGCGCCACGATGAAATG	AACCTCTTAATAACCAAAACCGGTTCT
17	AURKB	GCTCAGTCCTCTCCCCCTT	CAAAGGCACCCTCAGAGCTA
17	AURKB	CCTCCAGCTACAAGCAGCAC	GGAGGAGGATCTACTTGATTCTAGAGTA
17	AURKB	CTCTTCTGCAGCTCCTTGTAGAG	TCATCTTTTCCAGCTCTTTTATGCCTT
17	AURKB	CCTACTCCTCCCGTGCTTAG	CATTCAACCTACCTGTTCTCTCCTTA
17	AURKB	GGACCCGTACTTTGGAGCAT	CCCAGAAGGAGAACTCCTACCC
17	AURKB	ACGCACCCGAGTGAATGA	GGAGCCCAGGACCTCATCT
17	AURKB	GGGTTATGCCTGAGCAGTTTG	AAACAGAATGGGTAGTCAAGGAAGATG
17	AURKB	GAAGGGCCTTACCGTCTGT	CTTCCGGCTCGATCGGT
17	AURKB	CCTGGCTACATCTTCCTTGACT	GAGAGTGCATCACACAACGAGA
17	AURKB	TGACGATGCGGCGATAGG	CTGCCCCCAGAGATGATTGAG
17	AURKB	CAGATCCACCTTCTCATTGTGCAT	CAGGGAAAGGAGAGTTCCTATGTC
17	AURKB	ggccCCTGGAGAGGTTTTAAT	CAGAAAATCTGCTCTTAGGGCTCA
17	AURKB	CAGCAATCTTCAGCTCTCCCT	TGGAGGTCCATCCTTGTCTGATT
17	BIRC5	CTCTGGTTTCAGTGTCATGTGTCT	GAAGTGGTGCAGCCACTCT
17	BIRC5	CCATGGATTGAGGCCTCTGG	CAAAACAAGAGCACAGTTGAAACATCT
17	BIRC5	GCTGCCACGTCCACTCAC	GGCTGGCCAGAGAAGACTTAC
17	BIRC5	CGCCATTAACCGCCAGATTT	AAGAAGGGCCAGTTCTTGAATGTAG

17 BIRC5	CTCAAGGACCACCGCATCT	GTCACAGTGGCCTCGCTA
17 BIRC5	GGAGTGGACTGCCGCTTTAAT	CCAAGGGTTAATTCTTCAAACTGCTTC
17 BIRC5	GTTGCGCTTTCCTTTCTGTCAA	ACAGGGTTTGAGCAGTTCTTATTCC
17 CD79B	GCAGGCGAGGCTACTGA	GGGAACATAGAGGGCATGCAG
17 CD79B	CTCCGAAGCAGTCACTGAGG	AGACAGCCACCTATGAGGACATA
17 CD79B	ACTGACCCCGAGGACTCA	GACACTAACACTCTGATCTCCATCC
17 CD79B	TGGAGACCCTGCATATGCCT	CACGCTGAAGGATGGTATCATCAT
17 CD79B	GATGATCAGCAGCGTCTGGAT	GCTGCCCTTGTAGGCCTAAAA
17 CD79B	CTCACCTACAGACCACTTCACTT	CCACTATCTGCTGGTGTGGTTG
17 CD79B	GCCATGACCATCACCACAAGA	CAATGGCATCTACTTCTGTCAGCA
17 CD79B	GTAGACCTCCGAGGTGTTGTT	CAGGAGATGGACGAGAATCCC
17 CD79B	GATTCGTTCTGGGACTCTTCCAT	CCCACGTTTCATAGCCAGGAAA
17 CD79B	TGTAGCAGTGCATTTTCACCGT	GGAAGATGCCAAGCGGAATG
17 CD79B	CCTCGTCGTGGGTTCTGTA	GCAGTTACACGTTTTCCTCCAAG
17 CDK12	CCCTAACTTGGCGCATGACTT	TGCCTTACCTCTCTTCAGAGTTATAGAG
17 CDK12	TTCACTGCTGCATTCCCTTACAT	CCCTGAGGTAGTTTCTTTTCGAGAA
17 CDK12	AGAGCCACCTCCATCCAAAAC	GCCCCATTCTGGCACTGAC
17 CDK12	GGCTATTTATCTTGGTTGCAATCCGA	CCAGTTCCAGATTGGCTTGAAAAAT
17 CDK12	GGGAACTATTCACAAAGAAGCCT	AACTGGAGGATGAGAATACCTATCACA
17 CDK12	TCCATTGTTCTTGCTTTTGCTTTGTT	GAGATGTTCATTTTCCCCAAACTCAC
17 CDK12	CCCATAGCACTGAAAGAGGAGATT	AGAGCTGGTATTGGAGGCAAAG
17 CDK12	CTACTACCCCTCCACCTCAGA	GGACACAGCAGATGTCTTTGAGT
17 CDK12	GTACTTCAACTTTGCCCCCTTCTA	CAAAGGAGGCAACGTTGAAGTTT
17 CDK12	CAGCTGCTATTCCACACCTGA	CCATGAGGGTAGTGTTGACAAAGATTAA
17 CDK12	TGCTCTGGATTTGTTTTTCAGTCT	GGAGGTCGGTACCACAAAGTAATG
17 CDK12	AGTCGCCCTTACACAAACAAAGT	ACAGAGAAAACAAAGTACAAGTCAACAGT
17 CDK12	CCTTTTCGTAACCAGGCATTATGATCT	GGGAGAGGAAGGTCTGTGAGT
17 CDK12	GGAACAGAGGACACGTCACTT	GAAAGACAGATTCCTCCAAAAACTCAC
17 CDK12	CCTATCTCATCGTCTTATATTGGCTTCA	TCTCTGGGTTGGAGTGGATGT
17 CDK12	CTCAAATGGCACAGCTGCTTA	GCTTCCTTCAAAGACTCCTCTGG
17 CDK12	CTACTTCCCAGCAGCAGGAC	GGGTTTTCATCAGCTGACTCAAGA
17 CDK12	CTGACATGCAGAATATATTGGCAGTTC	TGAGATTCATGACCGGTCTGTTTAC
17 CDK12	CAAATTTGAGAACTGTAGGGTCATTGTG	TGTGCACCGCTTACTAGGATCTA
17 CDK12	ATGGTGTTTAAAAGAAGTAACCCTTCCA	GGAAAGATCGCCTTCAACCAGAG
17 CDK12	CCTCACATTCTTCCACCAGAGAAG	CTGGGATAAAAGTTGCAGCAAGG

17 CDK12	GCAACCATTCCTGAAGGCTGA	GGCCCCCGATAGAGTTTTCC
17 CDK12	GAGTTGAACCCAGCCGTGA	TGTTTCAGGCCCATCAGTGTTT
17 CDK12	GATTTATTGGACCACATGCTGACAC	TCGTCTAAGGCCCAGGGAAA
17 CDK12	CCCCGTCCAAACAGGACTTAT	GTAACTGCTGGACTCCCCAAG
17 CDK12	GCCCAACTCAGTCTTCTGCTTAT	GAAAGGAATCTTTCAGGACACTGAAGT
17 CDK12	GGACCTTCTCAGGCTCTCTGA	GTACCACAGAATTGCTGCTTCC
17 CDK12	gcccggccTTCATTTTCTTAAATAACTA	TCAAACTTGTCCACACAGCGT
17 CDK12	GTTGTCCTCGTTATGGAGAAAGAAGA	TGTGGCAATATTTACCTGTGTCTTTGT
17 CDK12	ACTGTAGTCCATTCATTTAAAACTGGCT	GAAGCCTGACAGGTGAGATACTG
17 CDK12	CCAGTTCACGCAGTCGTCATT	CAAAAATACAGGTGAACCCTTGGAC
17 CDK12	GCAGCAAAGATGGATGGAAAGGA	GGGCAGATTTTTCCAATTTTACACTT
17 CDK12	CAGGTTTGGAGTCTAAAAAGTTACCCA	TTTAACAAGATGCTTCTCGGAGTTCT
17 CDK12	CTTCAGATACAGGGAAAGTAAAGTTGGA	TGATGTTTCTGTCTCCTTTGGAGTAAC
17 CDK12	GTCTTCCAGAGCAAGGGCATAT	GATTTTGATTTCACGAATGGCTGTGA
17 CDK12	CAATGAGAAAGAGGGCTTCCCAA	CCAGTCTACCCTGTAAAAATGTGATTCT
17 CDK12	GACTTGAGGCATTGTTATTTCAGCAT	CAGAAAAGTGCACCAAACCAGATTC
17 CDK12	GACCATGACTTAATGGGACTGCTA	CCGATGCAGGAAATTCTTTTTGTGA
17 CDK12	ACAGCTAATGGAAGGATTGGAATACTG	ACTAAAAAGGTCAGAGGAGAAGAGGAA
17 CDK12	gggtTGCTTTTTGGAGTGCT	GTGACGCTCTCTGCTGTTAGA
17 CDK12	TGGAGGAGCTTCTGGAACTTTG	GGTCACCAACCCCATGTCTTT
17 CDK12	GGTCAGTCCCCCTTACAAGGA	CGATCGCCCGCTGTAAGAG
17 CDK12	CCGGGACTTACTAAAAGCTAAACAGA	TTGCTGCTGCTTTTGGCTAC
17 CDK12	CGTCCAGCTACGAAAGAAGTGG	CCTAAATGAGGAAGGACTGTTAGACAAA
17 CDK12	CGGCATAAGTCCAAACACTCC	TCGTTCTCCCTTCGGTCTAGT
17 CDK12	CTCCGATGACATGGCCTTCA	TGCTGGAGACTTCTTGGCTTTT
17 CDK12	AGGAGACTGATGACTATGGGAAGG	CCACTGTTTTGTAACTTTTGGGTGTT
17 CDK12	CGGAGTAAAAGTCATCGAAAAAGGGA	AGATCGTGAGGGACTAAGGTCATAA
17 CDK12	GGGAGCTTCTTATGGCCAAGA	CTGGACTGGTAGGCCGAAG
17 COL1A1	TCATCCCAGTCTTCCCTCCAA	CTACAGAGGGATCACCATGACC
17 COL1A1	AAGGAAGGGCCATTAGAACACATC	TGATGGTCTTTTCTCTCCCTCTCA
17 COL1A1	GGGAGAACAGCCAACTCATCC	TGATGCCTCTGTGCTCTCCTA
17 COL1A1	GGTCTCAAGTTTGTGGCTCTTTG	TTCACTGGCCTCCTCTCCT
17 COL1A1	ACAGGGAGACATGAACCCCTT	CGCTGACATCTCCATTTCATTCACA
17 COL1A1	ctgGCTCTTCATGGATCCTCA	CTTTCGTGCCTCCCATCCTTAG
17 COL1A1	CCATCTCCTAACACTGGCTCTGA	GCAACATGGAGACTGGTGAGAC
17 COL1A1	TCTTGCTGATGTACCAGTTCTTCTG	TCCCTTTCCCTGACTCCATCTT
17 COL1A1	CAGAGAGAAGGAGAGATGCTGAG	CTTCAGGGAATGCCTGGTGAA

17 COL1A1	GGTTGAACTTACTCTGTCACCCTTAG	AGGCCTCACTCCAGTCTTCTT
17 COL1A1	AGGGTCCCCGAGGTGAG	TGGGCCTGAACTGCCTTTTT
17 COL1A1	GGCAGAAGGGAGAGTTTGGTA	TTTCCCTCTGCTCCTAGGCT
17 COL1A1	tccAGGCTGTCCAGGGAT	AAGGGTAAGCGTTGCACTCT
17 COL1A1	GCCCAGAGTGCAACGCTT	GTAATCTCCTGCCCTCGAATTTTG
17 COL1A1	TGTGAAGGCTGCTCTGGAGATA	TTTGCTCACTGTCTGTTCCTCTC
17 COL1A1	AGACCCTACACGGGATGGT	CGCTGGTGAACGTGGTTCT
17 COL1A1	AGCTTCACCAGGAGATCCTTTG	CCTGGGCATCTTCTTCCTCTTTT
17 COL1A1	CAGGCCTGGGAGTTCTTCTAT	ACTTGTCTTCTAACCCATCTTTTTCCT T
17 COL1A1	GGCTCCTCTTCCTTTCTGGATT	ACTCTCAGCTCATCCTCTTCTCC
17 COL1A1	GAGGCTATGTGTTAGGGCAGAA	TTATCCATGTCTTTAGGGTGATGCTG
17 COL1A1	CATCTTTGCCAGGAGAGCCAT	CAGAGGGCCTCTCAGGAAAC
17 COL1A1	CTAAGGATGGGAGGCACGAAA	CTCATACCTCTCTGCCTCCCTA
17 COL1A1	GACTGAAGCCTGGCAGGATAC	CCACTTTCTAACCTCAGAGTCCTT
17 COL1A1	AGAAGGGAGGGAAGGTTTAGAATCT	CCGTGACTGTAGTGTTCTCTCTG
17 COL1A1	GGGTGCTGTGTGAAGGGA	CCTGACCCTGAGCCTCTTG
17 COL1A1	GACTCTGAGGTTAGAAAGTGGCAAA	GATATGTCCTTTCTTCTGATTCAGGGT
17 COL1A1	CACTCCAAGTCCTGTGATGGT	CAAAGGGTGCTCCTGGTACTC
17 COL1A1	CGCTGTCCAGCAATACCTTGA	GGTGCTGATGGTCCTGCTGTA
17 COL1A1	GATATAGAGTATCCTTGCACTCCCAA A	CACAAAGAGTCTACATGTCTAGGGTC TA
17 COL1A1	CCACCTCCTCTGGATACCCAT	CTCACTCTGACTTCTCTTGTTTGTTCT A
17 COL1A1	CCCCAAGAGCAGATACTGAGAC	GCCAGTGCTCAGTGGACTTAAC
17 COL1A1	CTCTGGAACTGGGCACACTC	TGAACATCATGGTCCTCCACATC
17 COL1A1	CAGGTTAGAGAAGGGAGGACTGT	CAGCATTAGCAAACCTTGGCT
17 COL1A1	GAACTGGAGCCCAGCTACTTA	CTGACCCATATTCCCCTGCTC
17 COL1A1	GGAGGTAGGGATGGAAAGGAGAT	AGTAAATGAGAGGCCCCAGGA
17 COL1A1	GGCACTGTCTGCATCTGTAGAG	GCCAAGAGCTCATGCTTTCCTT
17 COL1A1	CCAGGCAATGAGGGTGGA	GCCCTGTTGGTGTTCAAGGA
17 COL1A1	CCTCGAGCTCCTCGCTTT	AGGAACCCCTGACACTGGA
17 COL1A1	TGAGGGTGTCTCCCCTTTTCT	CCTAACCCTGAGTTCCCCTTTG
17 COL1A1	GCCCCGTTAAGTCCACTGAG	TGACTTCTCTTGGTTTGGTTGTCA
17 COL1A1	GGTCATGGAGTGTTGCCATCTTA	GTACCCTCAGCATGGCCATT
17 COL1A1	CCTGGCATGGCAGGAGTAG	ACCACCCTCAAGAGCCTGA
17 COL1A1	GCTCCGGATGTTCTCGATCTG	CAAGAGAAGGCTCACGATGGT
17 COL1A1	GCATCATCAGCCCGGTAGTAG	GCCGGTAATCCCCACTCTCTT
17 COL1A1	CTGACGCCTTTGTCCTCATTC	ATCTCTCCAATCTGACTCCTTTTCTTC TA
17 COL1A1	CTGGGCCACTTGCCAGA	TGCCCTGTCCTTCCCTTCTA
17 COL1A1	AGAGGCATCAAGCCTGGAC	GCTAAAGGCGAACCTGGTGAT
17 COL1A1	CCAGCATCGCCTTTAGCAC	GGATTGTTTCCCACCCAAGCA
17 COL1A1	GCATTACCACGTGGGAGTGA	GCTCCAACGAGATCGAGATCC
17 COL1A1	ACAGTGACGCTGTAGGTGAAG	CCTCCCAGAACATCACCTACCA
17 COL1A1	GCCACGCTGTTCTTGCAG	CGCATAGCGGCTCACTCTT

17 COL1A1	GGGATTACCGGCATCCAAGTG	CTCCTGGCAAAGATGGACTCAA
17 COL1A1	ACATACAACAGGACCAGCATCAC	CTTCTGGCAGCCTTTCTCAAAC
17 COL1A1	GGGAGCTTAAATGACTCAAAGGT	TGTTCTAATGGCCCTTCCTTGTC
17 COL1A1	tgATGGCTGTCTGATTAGCTAGGA	TCCCCCATTTCCCACCTACA
17 COL1A1	CCTATCCCACAGCACAGCAT	GTGGTGACAAGGGTGAGACAG
17 COL1A1	GGTGACCCTTTATGCCTCTGTC	GTGTCCCCCATGCCTTTAGAA
17 COL1A1	GCGTTAGAGCCAAGGTTTGCTA	CGAGTGCTGTCCCGTCT
17 COL1A1	GGGCAGTTCTTGGTCTCGT	CTACTGACAACGCCCCTCTTC
17 COL1A1	CGAAGCCGAATTCCTGGTCTG	TCCATCACTCCCACGTGGTA
17 COL1A1	GTTCAGTTTGGGTTGCTTGTCT	CCTGCCCATCATCGATGTG
17 COL1A1	GCATCATTGGGTCCTCAGTCA	CTGTTCCTATGTTCTCTCCTTCCAG
17 COL1A1	GGGTCAGATGGTATCTTCTTGCT	TTCCCTTCAATCCCAGCCTTC
17 COL1A1	GGCCATGCTGAGGGTACT	GCCTGACTCTTTCTTCTCCCTT
17 COL1A1	GTGAGGTGCCAGAGAGCAG	TGGACGTCCTGGTGAAGTTG
17 COL1A1	GGATCCTTTCTCGCCAGCA	GAGTCTGACAGCCCCTCCTAT
17 COL1A1	GGCTCACCCTGTAGATCAGAGAATAA	ACCTTCTACCTCTTTCCTGCTTTAG
17 ERBB2	CCCTCACCACTGTCCCTTCT	CCCTCCACCCAGCTCTGA
17 ERBB2	CCAACTAAGGGCCTGATCCTACT	GTAGGAGAGGTCAGGTTTCACAC
17 ERBB2	CCACTATAAGGACCCTCCCTTCT	TGCAGAAAAGACCGTTGGACTC
17 ERBB2	CGGTCCCTTCCTCCTCACT	TCTCCTCCGCCCCTCATAC
17 ERBB2	CTGCCTGGTACTGCCCTATTG	GGCATGGACTCAAACGTGTCT
17 ERBB2	GCCCTGGTCACCTACAACAC	CCTCCCCACCAAAATGAGAAAACT
17 ERBB2	GTGCCCACCTTTCTCCCATA	GGGTTCCGAAAGAGCTGGTC
17 ERBB2	CCATAACACCCACCTCTGCTT	CACTGGGCTCCCTGTCTTAC
17 ERBB2	AGTGATGTCCACCCTGTTCCT	ACCTGTGATCTCTTCCAGAGTCT
17 ERBB2	CAGAGCAGCTCCAAGTGTTTG	CCATGCTGAGATGTATAGGTAACCTG
17 ERBB2	CAAACTAGCCCTCAATCCCTGA	CAGCAGTCTCCGCATCGT
17 ERBB2	GCAGCAGAAGATCCGGAAGTA	CATCGCTCCGCTAGGTGT
17 ERBB2	CCTGCCCTTTGCCCAAC	GGTAGAGCACATTGGGCACAAA
17 ERBB2	GGGAAGGAGAGATGAGTCCAGTA	CCCATGTCATCGTCCTCCAG
17 ERBB2	GCACCTTCTACCGCTCACT	GTGTGACCGAGGGCACT
17 ERBB2	TCTCACTGCCTGTCTCTGGTT	CCTGTATGACACCTGCATTCCAC
17 ERBB2	CGGAGACTGCTGCAGGAAAC	GTCTCTTTCAGGATCCGCATCT
17 ERBB2	TGGAGCCGCTGACACCTA	CAGGACCTGGCCCTGAC
17 ERBB2	CTATTTCAGCCCCACTCTGCTT	CGGGTCTCCATTGTCTAGCAC
17 ERBB2	AGCTCTTTGAGGACAACTATGCC	tgGTCAGCTCTGAATAACCAAGAGA
17 ERBB2	GTCTGTCTCCTGGCATCACAT	AAAGTCATCAGCTCCCACACAG
17 ERBB2	CCTGTCTCTGCCTTAGGTGTGA	CCACGCACATTTGACCATGA
17 ERBB2	CATCTGCACCATTGATGTCTACATGA	TCTACATACATCCTGGTCCCCTTT
17 ERBB2	GCTCTTCTCACTCATATCCTCCTCTT	TCCTTCCTGTCCTCCTAGCAG
17 ERBB2	TGTCCTCTGACCCATCTGCT	GGACAGGCTGGCATTGGT
17 ERBB2	GAAACCTGGAACTCACCTACCTG	TCTTCTGCCACCCACCTGTA
17 ERBB2	CCATCCCAGATCCGTGAGTG	CCATTCCCAGGTCACCATCAA

17 ERBB2	GGGCTGGCTCCGATGTAT	CAACGTAGCCATCAGTCTCAGA
17 ERBB2	TCTACAGCGGTACAGTGAGGA	TGCCCATCAGTCTCTCTGCTTA
17 ERBB2	GGTGTCTAGCCCATGGGAGAA	CCCATCACACACCATAACTCCAC
17 ERBB2	GTTCACCCACCAGAGTGATGT	TCCCTCCCTTCACATGCTGA
17 ERBB2	CAGAAGGTCTACATGGGTGCTT	AGTCTGTAATTTTGACATGGTTGGGA
17 ERBB2	CGGAACGTGCTGGTCAAGAG	TGCTCCTTGGTCCTTCACCTAA
17 ERBB2	CTGGAAAGGTGGTTCCCAAGA	GTGAAGGGCAATGAAGGGTACAT
17 ERBB2	CCAGGGTATGTGGCTACATGTT	ACTTCTCACACCGCTGTGTTC
17 ERBB2	CAAGAGGTGACAGCAGAGGATG	AAGAGGGTCTGAGGAAGGATAGG
17 ERBB2	TTCCCCTAATGGGTCACCTTCT	ACGTCTTTGACGACCCCATTC
17 ERBB2	CAAGACTCTCTCCCCAGGGAA	GTGGGTCCTGGTCCCAGTA
17 ERBB2	CCAGCCTTCGACAACCTCTAT	GAGGACACATCAGGGCTTCTG
17 ERBB2	TGTCCTGTCCCCACTCCTTTAA	CCCCTCTTTCTCAGTGCTCA
17 ERBB2	CTGTTGACCTGTCCCGGTA	AGATCTTCTTGCAGCCAGCAAA
17 ERBB2	GTTACCAGTGCCAATATCCAGGAG	CATCTGGATCCTCAGGACTCTGT
17 ERBB2	GGGAAAGGGTCCTCTGATCAT	GGTTGTTCTTGTGGAAGATGTCCT
17 ERBB2	CTACCAGGACACGATTTTGTGGA	GGTCAGGCTGTCTGAGAGAAGA
17 ERBB2	GCTGTCCCTTGGGACTGTCTA	CATGCGGGAGAATTCAGACAC
17 ERBB2	GGCCAAGATTCCGGGAGTTG	GGCATACTGGACTCATCTCTCCTT
17 ERBB2	GCTCCTCCTCGCCCTCTT	TCGGGATAGGAGAGCTCGTC
17 ERBB2	GTGGTCTCCCATACCCTCTCA	GTCTAAGAGGCAGCCATAGGG
17 ERBB2	CAGCTGGTGACACAGCTTATG	CCTAGCCCCTTGTGGACATAG
17 ETV4	AACAAGCTGCTCTCTTGTGATTCT	CCAAACTCACCTCTGACTTCTCTCT
17 ETV4	CACCACCTTGTCCCTAGACC	GTAGCATCTCTACCCCAAAACTGTTT
17 ETV4	GGAGAGGAGAAGTGTAGGGCTTA	CTCCAATTCCACTATTTACCCCTCAG
17 ETV4	CCTACCCTTCACCAGGTCCA	CCCCCACAGTAGCTTTCCA
17 ETV4	CTCCTTCTTGATCCTGGTGGTG	ACCCAGCTGGTCCTCAAAG
17 ETV4	GGTCGCGGTTTGTCTCTCTT	AGACTAAGCGCCTCAGGGT
17 ETV4	GTGGGTGGGTTGGATGTATGA	GGTGGTGATCAAACAGGAACAG
17 ETV4	GTCGTAGGCGAAGTCCGT	GCCCTGCCCACCCTATC
17 ETV4	GGTATTCTTGCTTAAAGCTCTGCTG	CTTCCTTCTCCATCCAACCACAT
17 ETV4	CCCAACATGGAGGGCTTGG	CACTTCTCACATCACCCCTTCT
17 ETV4	GGGTCCTCGGGTGACTCA	CCGGATACTTGGACCAGCAA
17 ETV4	CTCACGCTGCTGAAGGTGTA	CCTGGACGGTGTGCGAA
17 ETV4	AGTGGGTTTCCCTGTCTCCTTA	CTTGGACAGTCGCCCCTAC
17 ETV4	GGATCTCAGGAAATTCCGTTGCT	GGTTCAAAGATGCTTTCCAGCTT
17 ETV4	TTGCAACAATGAAACGTGATGTGA	ACCCCCAATTTCCTCCACAAG
17 ETV4	ATTCATTTATATGTACACAGGGCAGCA	TGTCAGTGAGGAGGACACAGT
17 ETV4	TCATCCAAGTGGGACAAAGGG	CCAGACCTTCTCTCTCCCCATA
17 ETV4	TCCAGGCTGTAAACTTTGGAAAGG	CAGCTGTGGCAATTTCTGGTG
17 ETV4	CATTTGTTGGGTCATCCAGCAAG	CACAAACCCCACACCTCCTATTT

17 FANCJ	TTTATGATAACACCTTATATTACAAACCACCAT	ACCAAGTAGCATTATCTCCAGAAAGGA
17 FANCJ	CTCCTTTCTGGAGATAATGCTACTTGG	ACCTCTTTAAAGTACAGTACCTCACCTT
17 FANCJ	GATAGATGACTTGCTGCTTCCAGTAAA	GGCTGAATTTTCCAAAAAGCATCAAAAA
17 FANCJ	CATTGTCCTGTATATTGGTTCTGTCCTTT	CTAAATGGGTACGGCAGCAGAT
17 FANCJ	AGTTATTACCTTGTTGCCTCTACCCT	CCAGACTGTGAGCCAAGGAATT
17 FANCJ	ACCTTGTAAGATGGCAAGAAACACAA	GAATCTCTGTGCTACCTTCCAGAATAC
17 FANCJ	CTCCCACTTCATCTTGGAACTCAAAT	CTAATTTCCATGCCTTTTTCAGGCATA
17 FANCJ	TCTGGGTTACTCACTAGATTTAATCTGGAT	GGAGTGGAAATGAAATGCTCTTAACTTT
17 FANCJ	GCAGTGGTGATACCCATTTTGTGT	GTGTGTGTAAGGATGATACTGGTTGA
17 FANCJ	CCAAGTGAACCCAGAAAATATTCTCCAT	CTTTCCTTATAAAGCTTACCCGTCACA
17 FANCJ	CAGAATTCATCATAGCAAGCTGTGAC	GTAAGGCGTGTCTCAATATTTGACATTT
17 FANCJ	GCTGGTACTGAGCAAGAAGACAA	CAGACAAAAATGGGTTGTTGGTTCTAC
17 FANCJ	CAGTTTTCTGTCGTGAACGTTTCTTATTT	CCACTTAATTAGCACTTTCTTTGCATCT
17 FANCJ	AAAGGCAGCACAAATACACTAATAGACA	GGAATCAGCAAGTTACAGTGTAACAG
17 FANCJ	GAGCAAACCGAAGCTGAACTT	CCTGCTGTTGTTAAATCTATTTTGCCA
17 FANCJ	ACATACCAGTTCCTATGGTTCCAGTTA	AGCATACAGGGCCTTAAACCAG
17 FANCJ	AAATTTCACTCCACTTACCTACCAAGG	ACTAAAACGACAATACAATGACCACCAT
17 FANCJ	TCACCGACCATGAAATAATTTCCAGT	GGTGAAGACAGTCATTGTAGAACCA
17 FANCJ	TTGTATTTGATTGCGTCATAGTACACCT	GAACGTTGGCTCTCTACTGGTT
17 FANCJ	CAGATATCAACTGACCCAGGCA	TTGTGCATGCCATTCAAAGGATTTT
17 FANCJ	GTTCCTTGGTTCATGTCATTGTTTGT	tgggCTGTAGAGAATGATTGATGTTTATT
17 FANCJ	CAACAGAGTTTAACATAAGCATGATGACAT	TGGATAAATGAACTGGAACTGGGAAAA
17 FANCJ	GCCTTTATTTTTGGAAGGAGATGGTTT	GCTGAGGATTGCATAGATACAAAGTTGA
17 FANCJ	TCATCTTCTGCTTCTGTTTCAAAATCTCT	AATCATTCTGAACATCCGCTCTGT
17 FANCJ	TGGATCCAGGGCTTCTTCAGA	CCCTTCACTGATAAATGTGAATCCTCAA
17 FANCJ	CAGTTCCAGTTCATTTATCCAAGTTGTTT	AGAAGTAGATTCAGCCAGAGAAGTGA
17 FANCJ	AACTTTGTATCTATGCAATCCTCAGCTT	TGACCTAGCTGAAACTGATAGAGGAAAT
17 FANCJ	AATCCTCAGCTTTCACTTCTCTGG	TGACCTAGCTGAAACTGATAGAGGAA
17 FANCJ	CCTCTATCAGTTTCAGCTAGGTCATTTT	TGGATCCAGACATTGAATTGTCTCT
17 FANCJ	CTCTAAGCCCAGCTGAGATCTTAC	TGTCCCACTGGAAAACTGGATTTATT
17 FANCJ	ATCCAAACGATGTGTTTACTGTCAGA	CCTGAGCTCGGGTCATCAGA
17 FANCJ	GGGAGGACTAGAGGCACTATTC	GATCCACAAGCCCAACTTTCAAC

17 FANCJ	TGACCAGCTAACTCTCTTTGTTTGTTT	GCAATTATGTTAGCTAGGAGCAGAAAGT
17 FANCJ	GCATTAATACATACTTTCTGTGGCGAA	CACCTTAAACTTCATTTTGGTTCTGTGT
17 FANCJ	CTAGCAGTTAATTTGATTTTCCGAAGTTGA	CAACAGAAATGAGAAGTGCATGGAATT
17 FANCJ	TTTACTCACGTTTTTCCCATCTAGCA	CGGAGGACGGCATATTCAGG
17 FANCJ	TGCTGGAAAGAATAGTCATTGGAACC	AAACAGTCAAGAGTCATCGAATACCATT
17 FANCJ	CTTGGATTTCCCTGTATGATCCTTCTT	GATTCCATGTGAGGTTTGATAACGTTTT
17 FANCJ	AAACTTAAGGTTTTGATGGCCTACCT	AGTTTGTCGTGGTAAAGTGAGTGA
17 FANCJ	CCTGTAGATCTTTCACATTTGGAAAAGG	TGTTGTTTAAGATGGAGCTCTCCTG
17 FANCJ	TTACATCTCCATGAGTAGGAAGAAGGT	CCATATTACACAGCCCGAGAACTAAT
17 FANCJ	GTAGGGACAAAATATGATGTCAGCATCT	GTGATCAGCACACATTACAGACTTTC
17 FANCJ	TCCCTTGGAAAGTCTGTAATGTGTG	CTTGTGGCTTTAATGATGTTCCTCAAA
17 FANCJ	CATGGCTGAACCAGTCTGGAT	AACATTGTTTGTTGGAGAGTCCCA
17 FANCJ	GGCTAAGCTTTTTCCACTTCCTG	CAATCTCACTTTTCCTTGCTGTTCTATT
17 FANCJ	TGCTAGCATCCAAATTAGGCTATTT	CCAATTTATGGTAAAGAGGAGGCAAGA
17 FANCJ	TGAGTTGATGCACTAATAACAGGTACTTC	AATTCCCTCCCAACCCCTCTATA
17 FANCJ	GATACTTGACTACCATGTTCAGCTGT	CTCTGGCTGCAAAGTTATCTGCTA
17 FANCJ	CATCTCTGTATATGGATGCCTGTTTCT	CCTTACATCCTGGTTCCAGTTTTCTTC
17 FANCJ	CTGGCACTTCAGGTATCTTCTAACT	TCACCAATGAAATCCTTTTCGTCAGA
17 FLCN	CCGGCACCTCATCTCTGAATT	CTGGTACAGCATCATCACCATCAT
17 FLCN	TTGATGAGGTAGATCCGGTCCAT	TCTTCGGAGATGAGCAGCAC
17 FLCN	GAAGGTGTGGCTGAACACAAAG	TTTGTGCCAGCTGACTCTGAA
17 FLCN	GTGGGCTCCCACAGAGAC	AGGGCACCCGGGATATATCA
17 FLCN	GCTGACGTATTTAATGGAGGTCTCTTTA	AAATAGCTTGAGTTTTCCGAGCTCA
17 FLCN	CCGCACACCTAAGGAAAAGATG	TGAAAGCACTGCACTCTCTTGTT
17 FLCN	CTCGTTCTGGGCTGATTCAGA	GGATACCTTGGTCCAGATGGAGA
17 FLCN	AGGAGAGCAGACAGCTGGTA	gtGTTCTCCAGGCTCCTGAA
17 FLCN	CCTAACAGATATGCCAAAAGCAGAGA	CGTGCTCAGAGGATGAACACAG
17 FLCN	TGGTGTAGGAATGGCGTGAAG	GGAGTTGGCTGTGAACGAGTA
17 FLCN	CGCTTCAATCTTATTCAGGATGGT	AAGTACGAGTTTGTGGTGACCAG
17 FLCN	CCTCGGTCTGCAGCTACA	CCACGGGCCTTGTGTTGTTA
17 FLCN	ACACCTACTGCAGGGATCACA	AAGAAGAGGAAGGTGGCATTCAG
17 FLCN	GCACGCATCCGACTGTTCAT	CTCTCTGCCACTTCTGCGA
17 FLCN	ACCTCCGTGCAGAAGAGAGT	GGGAGGTTTCATGGAGTCAATAGG
17 FLCN	GTCTTTAGGCAGGTGTGTGTGA	GTCCGAGGAGGACAATGTCAAG

17 FLCN	CCAGTCATCCAGAACTTCAGCAG	TGTCTGAGTGTTTTGTTTTGGTTTTCTT
17 FLCN	CTTTTGGAAACAGCTCCAGGTTTT	CTGAATAAGATTGAAGCGGCTCTG
17 FLCN	CACAGACAGGTTCTGGTTGGT	CAGAGTGTCCTTTTTGTGAGAAGATG
17 FLCN	TCTCCTCCTGAGCTCCTGATG	TGTGTTGCCAGAGAGTACAGAAG
17 FLCN	CCTGAGAGAGAGGAGGACTCT	GAGACTGTTTGTATCTGTAACTGTCCT T
17 FLCN	AGATTCTTTCACATGGCGGTCAA	AATCGCTGAGGGAGGGAAAC
17 FLCN	CAGAGATCTGGTTCCACTTTGGG	CCATACAGCAGCCAGTACGA
17 FLCN	CCAGGAAGTTGCACCGATAGG	GGTTTGGGTAGTAGAGCATGGA
17 HLF	CGCCTTGAGAGCGAGTACTTT	GTAGGGAGGCGGGATAAAGG
17 HLF	GACCGCTCCCCCTGAATC	GGAGCGTCGTCCCTTCC
17 HLF	TTTTTAAGTCCAGCTTTCCCTTCTTCT	GTATCTCCGTCATAGGGAAGGGT
17 HLF	GGGCCTACCTTATGGGACAAA	GCTGTGGTCATGCTGAGATGG
17 HLF	AGGAGTTTTTGTCAGAAAATGGCATT C	GCTCTGCATACAGTTCGGAGAT
17 HLF	CCCTCGGTCATGGACCTCA	CTCTACCTTGCCTGTCCCATTC
17 HLF	GGTGTTCATGAATTAAAACTCCTGTG T	GCGAGGGTCAAACATTTCCTG
17 HLF	AGATCTTGCCCTTTCCAGCATC	TTGCCCAAAGACTGATTACCAGTT
17 HLF	CTGGCACCTCTGCACAATC	TTGGCTGCCATGTTGTTCTTTC
17 HLF	CTGTAACTAATGAGCAGGATGACAAG TA	CTTCCTCAAGTCAGCCACCTC
17 HLF	TCGTTCCTGGAGAAGGAGAACT	ACAGGAAACAAACTGTCCATCTATTC C
17 ITGB3	TTCAAGTGACTCCTGCTTCATTCA	TTTTCGGTCGTGGATGGTGAT
17 ITGB3	CCTGCTCATCTGGAAACTCCTC	GCTGCTTCCACTTAAGAGTCTCA
17 ITGB3	GTCCCAACTGTATCCAAATCTGCTTA	TGGAGTAAGACAGGTCCATCAAGTAG
17 ITGB3	GGAGGATTACCCTGTGGACATCTA	GACACAGGCTTGTCCACAAATG
17 ITGB3	AGTAACCTGCGGATTGGCTT	GAGCTAGAAATAGGAACCAGGACTTG
17 ITGB3	GGAGACCACCAGCTTCCTTTG	GACTATTCCCAGCACAAGACACTT
17 ITGB3	CTACTTGCCAGATTTGGCTTGG	CAGGTGGCATTGAAGGATAGAGA
17 ITGB3	CGTGACCTCCCTGAAGAGTTG	CCACAGATGCTCCAGGACAAAG
17 ITGB3	CTGGCCTTGGCATACCACTA	TGGTCAGTTAGCGTCAGCAC
17 ITGB3	GCACAGGGTTTCCTGTCACATA	CGACACAGTCATCCTCATTCTTATAG G
17 ITGB3	CTTGCCCATGTTTGGCTACAAA	GCACTCCAAGTCCTCCAAACTC
17 ITGB3	TGGCAAGGATGCAGTGAATTGTA	CCTAACATGGTTCTCCCTCTCC
17 ITGB3	CAGGAGGTAGAGAGTCGCCATA	CTAGTACTCGGGCCTCACTCA
17 ITGB3	GCCCCAGAATCCATCGAGTTC	GTCTCTCCCCGCAAAGAGTC
17 ITGB3	ACAGCCCAAGCAAGATAAGTTCT	CAGTCACTGCAAAGATCAAATTGATG TT
17 ITGB3	TGATGACTGAGAAGCTATCCCAGAAA	GTATCCAATGCTAGCACAGGCT
17 ITGB3	ACATTCAGGGTAGGGAAGGACT	AGGCTGATAATGATCTGAGGATGACT
17 ITGB3	CTGGGCCCAACTGTGTCTAA	GGCAGGCACAGTCACAATCA

17 ITGB3	CTGATCGTCCAGGTCACCTT	GGAAGGGCGATAGTCCTCCT
17 ITGB3	GATCCCAGTGTGAGTGCTCAG	TCGTCACACTCGCAGTACTTG
17 ITGB3	CACAGCAGTGACTTTGGCAAG	CCTTCACCTATGTTTCCAGTGGTT
17 ITGB3	GGCTTGCTCCTTCTTTGCCTTA	TGCAACTCTAAGATGTGCTGAACTTAC
17 ITGB3	TCTCTACCAGTGACATGGCTGAA	TGGCATCAGTGGTAAACACCAG
17 ITGB3	GGAGGAATGATGCATCCCACTTG	ACCATAGGTGGTGCCAGAGAT
17 ITGB3	GGCAGGCAAGTACCTTCACT	CAAGGTCTGGTATCTGCTCCTTAC
17 ITGB3	cTCTGGGCGACTGTGCT	GGCTCCAAGTCCGCAACTT
17 ITGB3	CTTCCTGGGCTGTGTGTTTTC	ACAGCTGCCACATTCACACTT
17 ITGB3	GTACTGACACCTGCATGTCCA	CTCTCTCCAGACTCCACACTCA
17 MAP2K4	ACCTGGAGGTCAGACTATTTTAGTAA	GGAGATCTTCAGTTTTCCTGATGACT
17 MAP2K4	AGAGACTGAGAACACACAGCATTG	CTTGGTTTGTGGACCATTTTGTTGA
17 MAP2K4	GGACGAGGAGCTTATGGTTCTG	ACAGTGGTATTACTGCACCATGAAATT
17 MAP2K4	GTCTCGTAACGGTTTTTCTCTACCA	CCGCATTACTACATCCAAATCCATAAGA
17 MAP2K4	GGTCAACAGTGGATGAAAAAGAACAAAA	TGCCATCTTCATTTGTCCCAATTAGA
17 MAP2K4	GGAGTAGTAAATGATGCCTGGTGT	GAATTACTCAGCTGCGGAGGAT
17 MAP2K4	TCAACTAACACAAGTCGTGAAAGGAG	CGTGTTCGACCATTCATAAATCAGGTA
17 MAP2K4	GGGTGACTGTTGGATCTGTATGG	AGACCAGGTACTTACTGCTAAAGTGA
17 MAP2K4	GAACTCATGTCTACCTCGTTTGATAAGT	GCATTCAAAACTCCAGCAGCATC
17 MAP2K4	CGTTGTTACTTTGGATCTGAAGGA	ACTCACCAGAAGCTCTTTATACTTTGG
17 MAP2K4	CCTTACGAAGGATGAATCCAAAAGG	CCACTGCTTGCTTAGTGAACCA
17 MAP2K4	GCAAGTTCTCTTGCCTTTTGGT	CTTGCTGTAGATTTGAAAGGTGGATTTG
17 MAP2K4	CGCAAAGCACTGAAGTTGAATTTTG	TCTTTGCTTGAGTTGAGCCTTTGATTA
17 MAP2K4	CTGTTGCTTCCATTTGCCTATTCC	GGCAAGGTTGAATCAGCATAAACATAC
17 MAP2K4	ATGCAGAGGACTACACGGGATA	ACCCACCTCTGTGAATAATTTTCAAGTT
17 MAP2K4	AGACTGTGAAAGCACTAAACCACTTAAA	ACAGTGACACTGTAATTTTCAATAACCGT
17 MAP2K4	GACTTGACCACTTATGTTGTCTAAGGT	AAGTCACAGAGCTTAATATTTCCACTTC
17 MAP2K4	TGTCTTGCAGATATCAAACCTTCCAAT	CTATCAAGCAAGAAGGCCTGGA
17 MAP2K4	GGGAGCCTGGAGTTCTATGTTCT	CAGGATTTTACAAACATAGCATGCGA
17 MAP2K4	GAAGAACGTGCCGTTGAGG	ACACTGTGATACGGGATGAAAATTCTTT
17 MAP2K4	GCCGCTCGGCTCTTCAC	GGCCGCGTTCCTTACCC
17 NF1	TGGGTGTATCTGGTGTTGAAAATTCTAA	CCCAATTTTCTCCTGATTGTTCCAG
17 NF1	ATGTGCTTGCTTTACATCGTCTACT	CCAAGAAGATGCAAAGTAAAAAGCACTA
17 NF1	AGTATGAGTTTTAGAGGCTGTTAATTTGCT	TCAGCTGCCTACTTCCTCCAT

17 NF1	TGGACAGTCTACGAAAAGCTCTTG	GGACTGAACAAGTAGGAAAATGACAGA
17 NF1	GCTGACAGAAAGTGCTGCAATT	GCATGTTACCTTAAGATCAACCACCA
17 NF1	TGTCATTTTCCTACTTGTTCAGTCCAT	CAAGTCCTATGAACTTATCAACGAAGAG
17 NF1	TTTTCCATTACAGCAAACACAAATTCCA	GATACTAAGCTGGCTCTGCAGT
17 NF1	CCACATCTCCTTACCCTCCTG	CAAGGGAATTCCTAATGTTGGTGTCT
17 NF1	GACCTTTGAACTCTTTGTTTTCATGTCT	TGATAAATCTGAGGAACATGGCACTTC
17 NF1	CAGAACAGCATCGGTGCAGTA	CTTTGACATTAACTTCAAGCCCCTT
17 NF1	AAAAAGCCACCACCTAGAATCGAA	CAAAAGCACATAACTGAAAACCATAGGG
17 NF1	CATACTCAGTAGACAACATAAAGCCTCAT	GAAGTCCCAGCTTGGTAGAAAATACT
17 NF1	AGGCATCAGGTACATGAAAAAGAAGAAT	CATAGTATTTCTTACCTCCGTGCAACA
17 NF1	GACTTCCAAAGCTGGGAATCCT	GGAAAAACCAAACTTGCCATCTCTCTAT
17 NF1	GAATGCCTTCTCTTTTGTCTATATCTGATAATT	CTCTCCTTGCCATCATTACTTCAACTA
17 NF1	AGTTGAAGTAATGATGGCAAGGAGAG	CTCCTTTCTACCAATAACCGCATATCTA
17 NF1	ATGTGGATTGTGCAAAATTAAAACGACT	AGTTTGGTGTTCTAGTTCAGCACAATTA
17 NF1	AAAGCTACTGTGTGAACCTCATCAA	TTTTAAAAGGTGTCCAACCAATGCAA
17 NF1	CTTTGCATTGGTTGGACACCTTT	gccTAGTTTGCATTTAAAGTAAGACATAAG
17 NF1	TTTCTTTGAGTCCTCAGTGAAAGCTTAA	CCTTTTAGGAGCCTTTGTGTCTGATA
17 NF1	GGAACAAGGAAAAGTTTTGATCACTTGA	ACCCATTTCATAATCAGTTTCTGCTACT
17 NF1	ATGGAATCAGGGATCACAACACC	TGACTTTCATGTACTCTCCCACCTT
17 NF1	GAAGAAGACCTCAGCAGATGCTT	CACAATTTCTGTGTTTGTTAACCAGAGT
17 NF1	GGCATCCTTCACCTGCTATTGTT	CTGCTAAGTAGGCCACGCT
17 NF1	CACAGAGCGTGGCCTACT	TGTGAACTTTCTGCTCTGCCA
17 NF1	AGTTGCAAATATATGTCTTCCACCCTT	GCTTCAGTGTCAGGGTTCCA
17 NF1	AGTGGCCCTGTACATGTTTCTG	AGAGGCAAACTCCATGAATGTGTTAT
17 NF1	TGTGTGTTTGCATGGTCTTAGAAAGT	CGAGTGACAATACACAGCATCAATC
17 NF1	TGAAGTGTCAGTGCATAACCTCTTG	CCATGTGCTTTGAGGCAGACT
17 NF1	CGCATTGGATTGGTGGCCTAA	AAGGAGGTGAGATTCAAAATTCGTGAT
17 NF1	CCATTCACACCATGCACATATGATT	CCTGTTTGCGCACTTTCATCTTC
17 NF1	GATTTGTTGCAGGTTTTGGTTTTAATGG	TGTGTGTTCTTAAAGCAGGCATACTAAT
17 NF1	CCTTTTGAATGACTGCAGTGAAGTT	CACTGTCTACGTTGGCATTGAGTA
17 NF1	TGTACGGTCCTTGCAATGTCA	CTCTTACATGCCAGTTCTCTAGGTTT
17 NF1	TGTCATGGAAGAAATGTTGGATAAAGCA	ATGGATCCTCCTCCACTCACAT

17 NF1	GCTGAAAGCCTTCACAAGACCAT	CCAGGCCAGAATTGCTTCTCT
17 NF1	GGAATGGATCAACATGACTGGCT	GTGTATCTGCGTTTCCCTCTGAA
17 NF1	CAGTCAGTGAACGTAAGGGTTCT	CAGATCCTTAACATTGGTCCGTATTTGA
17 NF1	GCAGATACACCTGTCAGCAAATT	GTTAAATAGCATTGGATACAGAGCAGGA
17 NF1	TGTTGATTGGTAGCAGAAAGTGAAACT	AAAGCCTGCCTACTTAAGATCAATTTACT
17 NF1	CTGACAGTAAAAGGAAAAGCAACCA	GAGGACCCAGGTATGCAAGAAG
17 NF1	TCCTGCTCTGTATCCAATGCTATTTAAC	AGGCTTATTTCAAACAAGTCACTCTATTCA
17 NF1	ACGACCTTTTGATAAGATGGCAACA	CTTCTCAACCAAAAAGAAAGCAATCAAC
17 NF1	GGGAAGTGAAAGAACTTGAAAGATTCA	CTGTGATTTGGCTTCCATCAATTCC
17 NF1	CAGCCTGAAGAAGGAGATGGTG	CCTATCCTAGTCCTGTCATGGGTAT
17 NF1	CAACCAAAGGACACAATGAGATTAGATG	AGCTGAGAGAAAATAAAACCCCAGAG
17 NF1	TGCAGCTGAACTTCGGAATTCT	TCATATTACTTCAGTAGTCCCATGTGGA
17 NF1	TTGCTGTTCTTTTTGGCTTCATTTGTA	CCTTTTGGACTTACATTGGTGATGATTC
17 NF1	TGGCTCAGAATTCACCTTCTACATTT	AACCTTTTTGAAAACCAAGAGTGCAT
17 NF1	AATGTGCAGGGCTGATTGTCT	CACTTCAAAGCTAACATGTTGCCA
17 NF1	CTCCTGGATCCTTTATTACGAATTGTGA	GGAGAAGGTGCCTTGCTTCAT
17 NF1	TTCTGTTTTCCTAAAAGGCACTTGAGA	ACACTGATACCCAAAATGAATGCACT
17 NF1	AAGGCCATGTTAGTAAATTTGCATCTG	CTCTCAAACCGATCAGCCAATACT
17 NF1	ACAGAATTTGACACACTTGCAGAAAC	ctTCCCCGCTTACTCTAATCACTTAC
17 NF1	CCTTGCAGAATCCAAGAAAACAGG	CATTTTCCCCTTACCTCCATTGCT
17 NF1	CATGCCAGAGATTGCTCAGGA	CCTGTTACTCACAGAAACCACACA
17 NF1	CCTAAAACGTCATGATTTTCAATGGCA	ATTAGACATTCCTTGTTGTGCTCAGT
17 NF1	GCAGAACACACATACCAAAGTCAGT	TTCCCCAAAACACAGTAACCCAA
17 NF1	AGCAGAAATTATATCAATGAGAAAATTCATGTT	GCCGCATATGTTCTTCTTTTGTGA
17 NF1	ACTTCAGAGTATTGCCAATCATGTTCTC	AGCAACTGAGTAAGTGGCAAGAAA
17 NF1	GAGTCTCAAACAGGAAGACAACTCAA	AGGAATATCACATCCTACCCCGTAAAA
17 NF1	AGATAGAAGTTCCTGTCACTTTCTCCTT	AGAAAAAGTACAGAAGAAGCTGACCAT
17 NF1	GCTCTAGACTAAGTTGCTTTCAAGTGA	GCAGATCAGTTAACAGACAAAAGTCAAC
17 NF1	TGTGGAATCCTGATGCTCCTGTA	CAAAACCATAAAACCTTTGGAAGTGTAAGT
17 NF1	ACTTTAATGCCAGGGATTTTGTTCCT	TCTGGACACAAGATAAGGAGAATGATTTG
17 NF1	AGCAGCAGTTTGGCCACTA	CACCTTGAGTAAAATAAACTGCTTCACAT

17 NF1	GCTGCTTTATTTTTAACTGCAGTGTGT	TGCGGTACCTGCTGAATACAAG
17 NF1	GCTGCAGCTTGATGAGGTCAA	GTAATCTAGGAACCTCAAGGCAAAGT
17 NF1	ATTCTCAAGTGGTTGCGGGAA	GGAGGAAGGAGAAGCTGAAATAGAAC
17 NF1	TTGATTGCTGTTGTTAGGAAATAGGACA	TGGATAGGTGGCTGCAAGGTA
17 NF1	CTCTCCCAAAGGTTCTGAAGGA	CTAACCAAGCAACTTCTTAGTGTTGG
17 NF1	GGCAACCTTCTCAGGCCAA	AAAAACTTTGCTACACTGACATGGAAAA
17 NF1	TCATATTGATTAGGCTGTTCCAATGaa	CCTGTTGCACTGGTTTTGATGAA
17 NF1	GATCTGCTTGATGTTGTACTAGACAGT	CTTGCTTGAAACCAATTTCACATTTCC
17 NF1	GATACTGCTGTAGCTTTGGCTTCT	aCATGGGACTCAAAGTTTTAGCACAA
17 NF1	AATTTACAAAACTGTACCAGATCCCACA	TGACACCAGTTGACAATAGAAGATGTA
17 NF1	actcatCTCCCTTTAATTTTGGCACAT	TGGATCTTGGCACAATGATAACAGG
17 NF1	TGGACTCTAAGATCAACACCCTGT	CATCAACCATCCTTCTCCAGATACATTT
17 NF1	GGTGTGTGTGTGGCTTCAAAAAC	AGACATTTTACATCATCATCTGCTGCT
17 NF1	AAAATGGGATTGTTTGCACTAACCTG	CATGGAGTCTGCCAATTCTACTTCTTTA
17 NF1	CCAACTGCTCTGGAACATGTTTTC	GACATACCTCAGCACACACATACA
17 NF1	TATTTTCTGTCTTTACTTGTTCCTTTATTCTCT	GCAGCTGACTTGACTTTGCTAATG
17 NF1	GTCTGACAGAGTTCTCATTACCCAAATT	CTCTCTCTCATAGGAGCCAGGA
17 NF1	TCATTGCCTTCCGTTCCAGTTAC	TCATTTTGGCTTCTATACCTCCATGAT
17 NF1	TGTCATTTTCCTTAGGTTCAAAACTGGT	CGATTGCTAGGCCCGGTATG
17 NF1	GCCATATGAAATTGTAGTGGACCTTACC	ACTCCCTGACCCAGGAGTTAC
17 NF1	AACGTCTCCGCAGTCTATATCTATAACT	CAGGTAGTTTCTGTTGTTCATGCTC
17 NF1	GGGAAACTGGCTGAGCACATA	TCGTTTACAAAACACAGACTGGAACT
17 NF1	ACTTGCATGGACTGTGTTATTGGT	GAGCTAGTTCTGTCCACTGGTC
17 NF1	GCTGGACCAGTGGACAGAACTA	TCCACAGAACTTATAAAAACCTTTGTGt
17 NF1	ACCTGCCACCGTTTTCCTTTTA	CACCATGATGAATGGGATATGTATCCAT
17 NF1	TCTCCACTTCACCCCGTCA	GGTTAAGGTGAACTGGTTCTCATCT
17 NF1	GCAGCCTAAAACATAGAAAGTCACTTCT	GCTGCTTGCCTCCATTAGTTG
17 NF1	TTCGGAAATTGAAGAAATCTGCCTAGT	GCGGGTCCGGATATGAATGA
17 NF1	GTGTGAAGCCATTGTCCAGTCTA	ACCTACCGTAAACTCGGGTCA
17 NF1	TCGCATTACTTAATTTAGGCAGTTCTGA	CAACACTGCATACCTTCCAATTTACAAT
17 NF1	TGTGCTAAAACTTTGAGTCCCATGt	AGCATGTAGCGTGCTAAAATAGCAATA
17 NF1	TCTCCAACTCCTACTTTAGAACAACATCT	CGGACCTGTGGCTACTAAGAA

17 NF1	CCTACCTCTTCCACGTTGTTACT	TGGTAAACACAAATTCCTTTCCTAGAACT
17 NF1	GGAGCAAAAGTAATACGTAAATGGAAAGTT	TGCTAACTGCGCAACCTTCTT
17 NF1	GCAGTTAGCAGTTATAAATAGCCTGGAA	TCCTGAAAATGAATGTGAAACTATCACTCA
17 NF1	GCATTCCAATATAATCCATCCCTGCAA	AGTGAGGGCGGAACAGGTA
17 NF1	AACATGAATAGGATACAGTCTTCTACTTCTCA	ATTCGTTGATCAAACTCATCTGTGGTAT
17 NF1	TTGGCAGGCTACACTGGTAAAAT	GCACGAAGGTGAATTAAAATCAAAAGGA
17 NF1	GTTAGGTGAAGTGATTATCCAGGTGT	GGATCTTCGGATCAGTAAGTACTTCTTC
17 NF1	AGTGTCTGAATCAAATGTTCTCTTGGAT	GGCCTCCTAAAAGTAGACTGGAATAAAA
17 NF1	GTGCAGTAACTTGATTTGCTGTTGTAT	AAGAGGCTTCCAAATTGAGGGT
17 NF1	AAATAGTTGATCATACTTTGTAACAGAATCACA	GGTACAAGTTAAGGCACACAGAAGATTA
17 NF1	AACTTCATTTGTGTTTTCTCCTAGGTCA	GTGTTGTTGGCAGGGATACATAAAC
17 NF1	AGCCACACCTCACGTTAGAATTT	AGTAAAAATGTAAGTGAAAGTCTTCACTGGA
17 NF1	CCAATGAAGTCTACACGTTGCAC	GAAGGAGGTTCCGCTGGTTT
17 NF1	ACCATCAGAGAGCCTTGAGGA	TGGTATAAACAGTGGCACACACTT
17 NF1	CATCAGTTCCTCCTCAGAATTCCC	ACACCATCAGCAGCTAGATCCT
17 NF1	AGGGCCAGTTACTAGAGACATCAG	TTGCTAAATCCAGAAATACACTCTTCCAA
17 NF1	TGTTCTGTAGGCAACTTGCCA	GAAGGTCAAATAGGCTGAAGTGAAGATA
17 NF1	ACTGAAGGCAGCTCTGAACATC	TGTGCAGTAAAGAATGGCCAGT
17 NF1	GAAAAACCTACAGACCTGGAGACA	GGCGTTTCAGCTAAACCCAATT
17 NF1	AGTAACTGGCTGTTCTCTTTTTCTCC	AGCTTCACACGATCTTCTTAATGCT
17 NF1	CGCTGGCAGTTTCAAACGTAAT	GGGTTCATTATAAAACAGGAAGTGCAG
17 NF1	GAGCCTGCACTCCACAGA	CTGACCACGGCCTGGAC
17 NF1	CACAGGCCGGTGGAATG	CTCCTACCTCCCCTCACCTA
17 NF1	TTCATTGACCATCACATGCTAATAGTGT	GCATCATCATTATGCTTGCAAAAACGA
17 NF1	GACTCCATGGCTGTCAAATCTAGT	AGCAAACTCAATACCTGCCCAA
17 NF1	GCAGCTGGATTTTACTGCCATT	CAGGCTGACTGCCTCTTGA
17 NF1	CCTGCTTTTTAATCCAAGTAAGCCATT	AACCAATGCTCTCACCTTAAAGTGT
17 NF1	CGTATAAGCCCTCACAACAACCAA	GTCTTGAAACGAACATCAATACATAATAGTG
17 NLRP1	CCAGTGGTGAGTGTGAGTTGG	CCTGTGGCTACTGAGGTAGTTG
17 NLRP1	TCACCGGTACAAGTTCTTTTCTTTGT	ACTCTTGTGCGTGCCTTCTC
17 NLRP1	CCAAAGGCTTCGTATGCAGGT	CCCCTTACAGTGCAGCCATAG
17 NLRP1	CAGGAGCTCAGTAGGGTCTCA	AGTCTCCAAGCCAGGAGTCA
17 NLRP1	GCTCTCTGGGTGCTAGGCT	AGAAATCTCTGCCTCACTCCTCTA
17 NLRP1	GGGTCCTCTGTCCACTCA	GACGAGTGGCATGGAGGT
17 NLRP1	CCCATACTGAGCCACCAGGTA	GCCTGTTACTTGGAGTTCCTGAAG

17 NLRP1	GGAACTCCTTCAGCTCCTCCTT	CTGTTCTCTGCCTGCCTGATAC
17 NLRP1	CCTCCCTATCCTTCCCTGTTGT	CGTTGAGATTGAATTCTGTGTGTGG
17 NLRP1	ACTCACTTTCTGTTCAACCTCGATT	CGTTACACTGTGTCTGGGTCTG
17 NLRP1	GATCTCACCCAGGAACTGGTC	tgTCTGCCTCTCCCTCTTCTT
17 NLRP1	GGAGTATTTCCAGCATCCCTGAAC	CTCCCTTAATCATTGTTCCCTATGTTC<br>T
17 NLRP1	GGTCCCAGTTGAAGACCCTCTA	CTTCCACCTCTACCTGATCCCA
17 NLRP1	CTTCCGAATGGAGCAGTCACT	CATAGTTCTGGAAAACCCCAGCTT
17 NLRP1	GATCATTTTCAGGAGGACTCCCAAG	CTGTGCCTCTTCAATCCATTCTTTT
17 NLRP1	GGTCAGCCAGAGGGAGTTCT	CGGCTCCCTTTGTGCTTTCA
17 NLRP1	GCAGTGATGAGTCCCTTCCTTTC	GATGCATCTTCATCTCTGTTTTCCTC
17 NLRP1	GGTCTACTCAGGCCTCCTTGT	GTGATTGTTCTCCCTCCATTCCA
17 NLRP1	CCTTCTCATCCTCCAAGACATAGGA	CCCTTTTCAGTCCAGATGACCT
17 NLRP1	CCCATCTAACCCATGCTTCCT	CTGACTTCCAAGACCACCACAA
17 NLRP1	GGGCAAGGTAATGTAGACAGAGG	AAGCAATTAGAGCCTTTAGGTTGGT
17 NLRP1	GCCCAGAGCTCTTTGTTTGATT	CACGTTGGGTAGAGGTCCTG
17 NLRP1	CTTCCTGCTGGACTCAGAGAAC	AAAACTATACTTCCCGAGGCATCC
17 NLRP1	CTCCACATAATCAGGCCAGCTT	CCATGGGAGCCTTCTGTGAG
17 NLRP1	CATGTGGAACAGAGGCTCTCT	TGTTCCTTCTTTCCCTCCCTTACTTA
17 NLRP1	GGTCCGAGCCGTGATCAG	TGCTCTTCATCCTCGATGGTGTA
17 NLRP1	CAAGACCCATCCTGGCTCAT	GTCTTCTACTTCAGCTGCAGAGAG
17 NLRP1	CGAGACTCACCACCTTGGACT	CCAAGAACCTCGCATAGTCATACTG
17 NLRP1	GTTGACTTCCCAATTCCAGCAG	CCAAGATCCCCTGGTCAAGAG
17 NLRP1	GGTCTCACCTTCTCTCTGCTCTT	CAATGTGCTCACGGATGCTG
17 NLRP1	GTCTCTGGCAAAGGTGTTTGG	TGAGTAACCTGAAGACCATGGGT
17 NLRP1	CAGCCTTGCCCCTTCAAACTA	CCAAGATCTTAGGCAGAATGAACAAT<br>TC
17 NLRP1	GCCCACCAGCTGATTCACAG	CCTCCACCGCAGTGCTAATG
17 NLRP1	CCGGCAATTCATGGATCCAG	CCCCTCTGATGTGTCTCCAA
17 NLRP1	CCATCCCTGCTTCAGAACAGAA	CGTCCTCAAGGTCACCAGAAAC
17 NLRP1	CACTTAGGTCCAGCTCCTTCAG	ACACATCACATCTCTCCTGTCCTT
17 NLRP1	tccaTCCCCTGTCCGATACC	AGCCTGAAGGAGCTAGACCT
17 NLRP1	CAACGTCATCCAGGTTGTTCTG	CCTGAAGAGCGAGCCCTTAG
17 NLRP1	GCCAGGACTCAAGGGTCAAG	GGACCGGAAGTGCAAAGATG
17 NLRP1	CCTTCAGGGCTTGGTAGAGTC	GAGTGACATCGGTGGAGGTT
17 NLRP1	TGTCCATGCAGTTTGTCCAAGA	CTGTGGTTGGAGCCCTTCTTT
17 NLRP1	gcccagcTCCTTCATTGATTTTCT	CCAGCTGTTCTCGGAGTTCTAC
17 NLRP1	CCTGATCCCAAGTGGCCAAC	GAAAGACCTTCCATGCAAGATGAAC
17 NLRP1	CTCAGGTCACTCGGGCTTA	GCTCTTAGTGTGCACTTTCTGCAT
17 NLRP1	TCACGTGGCGGCTGAATTTA	CAGCCACACTCTCTGGAGTC
17 NLRP1	GAGTCTCGTACAAGCAGTGGA	GCATCAACCACACGTTTCCT

17 NLRP1	CCTCATCACTTAACAGGCCCAAT	TTTCCAAGAGTTCTTTGCAGCAATG
17 PER1	GGAGCTCTAGCTGGGAGACC	GCCAATAAGGCGGAGAGTGT
17 PER1	TGGAGCTGAAGCTACACTGACT	TGGTAACTGGCACCATCTCTCT
17 PER1	TGTCTGGCCATGAGGAAATGTC	GCCTAACCCCGTATGTGACC
17 PER1	CCCATCTGAGACCCGGATCTT	CTCTCTGTTGAAAGTCCCTAATGCT
17 PER1	CCTCGTCTCCTCTAGGTCCTAT	CCAGTGACCACACTCTCTGTG
17 PER1	AGAGATAAAGACATTAGTCCCAGAGT GT	ACAAACTCTCAGAGCCCATCC
17 PER1	GCTGTAGGCAATGGAACTGCT	CAGCAAGAGGTGTGTATGGATGT
17 PER1	CCCGTCCCAGGCTTCTC	GCTCCGAGCCATGCAGAA
17 PER1	GCTGGTCCTCAGAAAACCGA	GGAAGCTGTGGTAGAGGAAGGAT
17 PER1	GACCCAACATACACATCCATACACA	GATGACACCGATGCCAACAG
17 PER1	GACTCATTGCCACTTGAACCATTG	ACATGAGTGGCCCCCTAGAA
17 PER1	CCCCCAGGACAAAATGATTCC	ATCTTCCACCTCACTCCCTCA
17 PER1	CCCTACCCTGGGAGATGCT	ACCTGCTCGAACTTCTGCTG
17 PER1	GGAGCGCGAGTCCTCTTG	CCAGTAAGAGCAGTTGAAGGGA
17 PER1	AGCAGGGTGCATTGGATCTTTA	GGTACCTGGAGAGCTGCAAC
17 PER1	GAGGCACATTTACGCTTAGTGG	CTGGACAGCATCCTCAGGTAA
17 PER1	GTCTGTCTCTTCACCCACACATC	GTTCTGCTCACTGTGGTGCT
17 PER1	GGGAGTGAACACGTCCTCATT	GCAAGGTAGCCTTCGTGTTG
17 PER1	CACTTACGTGCGTACTTTGTGG	CACTCCCCTATCCGCTTCTG
17 PER1	CATGGTGACATACTCCCCGTT	GCCTTGGTCTCCTGGATCTCTA
17 PER1	CCTCTTGGGACACACCACTTA	TCAGAAGGAACTCATGACAGCAC
17 PER1	GTCGAAGCTTGAGCTCTCGAA	GGCCACACTCTGGGACTAATG
17 PER1	GATGCCTGGCAGGCCTTA	GGTTCTGCTCCCGTCCAG
17 PER1	CCTCAGGGACCAAGGCTAGT	CCATGTGAGCTTGAGAAGGCTAT
17 PER1	CAGATTCAAGAGCTGTGGGAGAA	GTCCACCTATACCCTGGAGGAG
17 PER1	CTCAGACGTGATGTGCTCCA	CCTCCTTACTGAATGCCTCACT
17 PER1	GCGAAGAATCCACTAAGGGAAAGT	CCCAGGATGTGGGAGTCTTCT
17 PER1	GAGATGGAGCAGTGGAACCAT	CTTCTCAGTGGCTGTCTCCTTC
17 PER1	CCTGCTCCGAAATGTAGACGATT	GAGTACACACTTCAGAACCAGGTC
17 PER1	TGGAGCAGAAACACCTCCCTTA	GCTGCGTGGACTCGACAG
17 PER1	CTGAGGGAGCTGTGGAAGAG	cagACGCCTACCGTCCA
17 PER1	AGCACGGCCTTGGTCAG	CCTGAAGTGGTTCTGATGACCTT
17 PER1	AGGCAGAGGTCTTCTCCAGTT	CTTAGTGGATTCTTCGCAGCCTA
17 PER1	GGGTCAGTGCTCACCAGT	AGCTCTCCACTCCAGCTCAA
17 PER1	GGCCTCCTGCAACAGCA	TCGCACTCCCCTTCTCCAT
17 PER1	AGAGAACACTGGGAGAGGGTAG	ACACCACTGCCGATCCAAAG
17 PER1	GAGACATAGCAGGGCGCTT	GGGTACTAACCCCAGGTTGA
17 PER1	GCTGCATCTCGAGTTGAACAGT	CTAACTATCTGTTCCCAACCCCATC
17 PER1	TCTGGAGTGCCCCATAAGGATA	CCCTACCCTCTCCCAGTGTTC
17 PER1	GAACAGATAGTTAGGGAGCACCAA	CTTCCCAGCGGTTGTCCA

17 PER1	GCTCCCTCTCCGCTACTCTC	GGCTGGATTCACTCTTCACTCTAC
17 PER1	CATCTGAGTTCTGAGAATTGGGACAT	AAGGCTTCAAGCTCTCAGGAC
17 PER1	GCCTTCTTCCTCCTCCTCCATA	CTTCTCAGAGCTGGATGGACTG
17 PER1	CCTCTTCCATGGGCTCCAG	CTGGCAGGTTAGCAGTGAGA
17 PER1	GTGCTGGTTCCAAGAAGGAGAG	CCCTGGGTCCTCCAGTGATAG
17 PER1	TCTGCATCACCCCCGTTG	CGGGTCAAGCCATCTAACCT
17 PER1	GAGGCCATGCCACTAGAGAT	CCTCATGCTGGCTATCCACAA
17 PER1	GGCGAGAGGAACTCACTCTTC	GCCACTCTGATGCCTCCTTC
17 PER1	AGAAAAGAAAACTGCCCCCACTA	ACCAGGTGATTAAGTACGTGCTC
17 PER1	GAGCAGCCAAATGGGATCCT	GCCTGACTCCCATTGGTCT
17 PGAP3	CCCCCAATCTCCCCTAAGGA	CCATGCCATCTGGCACATCA
17 PGAP3	CCTGAAAAAGAGGACGTGGACA	CGTGGTGGTGGTCTTGCT
17 PGAP3	AAGTCAAGCAGCTCGAGCA	AGGAGCCGAAGGAATGAGTCT
17 PGAP3	CAGGGATTGCAGAGGCGTATT	GGGCGCTCTGAATCACTTCC
17 PGAP3	CTGCTAGACTCATGTAGATTGGCT	CGCGGTTGGTCCTGCTA
17 PGAP3	GTACGCAGTCGCGGTACA	CTCCGGAAGTGGAAGCTTCATA
17 PGAP3	CCTCTACCCCAAGAGTCTCTCT	CGACTATGGCTACAACCTGGTG
17 PGAP3	GCCTCACCAATAGCCACGTT	CCTCTGAGCAACCCTGGAC
17 PGAP3	GCCCCCAGGGTTCGATTTT	CGTGAAGCCTCATGCTGGT
17 PGAP3	GGATGTAGAGCTCCACCTTCCT	CGTGGCCTCGTTTCTCAATG
17 PGAP3	GGTAGCGGCAGAGCATCAC	CAAACCTCCTTCATCTGCTCCTTT
17 PGAP3	CTACCACGCTACTACCATATGCC	GGAGTTCACCAGGCCACATTT
17 PGAP3	GGGAGTTGGCATGACCCTCTA	CCTCCTTAGGTGTCCCTCAATG
17 PGAP3	GGAAAACTGTGGACCAGAACCAT	TTCCCAAAGGTGAAATGGATGTGA
17 PGAP3	GGGCAGACTCGCTCCAA	AGAGCTGTGTGCCTCAATGG
17 PRKAR1A	ACAGCACCAAATAATACAGAGCAGTTAT	ACCACAATCTTCTGCCCATCTTC
17 PRKAR1A	GCATTGGAACCAGTGCAGTTT	TTTCCCTCTTAGAGCGTACAACTTTTT
17 PRKAR1A	TTTGTAACACACTCTCACAGTACCAC	TGGTAAATCTTACATCCGTCTCTCCTT
17 PRKAR1A	TGTGATTGATCAAGGAGAGACGGA	GGGATGTAATGACCCTTCAAAATCACTA
17 PRKAR1A	TGTTACCCATCTTTGCTTTCTCCA	TGTTGTACTGCTGGATGTTTCGT
17 PRKAR1A	CCCATGCTCAGACATCCTCAA	GTTTGCATGAGTGAAGCATGGAT
17 PRKAR1A	CTTGACATTTAATTGAAGCGCAGGTT	CACATTCTTTTCAATGGCTTTGGCTA
17 PRKAR1A	AGATTACAAGACAATGGCCGCTT	GGATCACACCCTTACTTGAAAAATAGTG
17 PRKAR1A	AAATCCCTGTGAATCAGTTGTCTAATGAA	GACGTAGAGCTCACATTCTCGAA
17 PRKAR1A	AGAACCATGGAGTCTGGCAGTA	GGTCTCTCAGGTCGAGCAGT
17 PRKAR1A	GCTCAAAGATTCTATTGTGCAGTTGT	CAACTGTCACAATCACCTCATCATCT
17 PRKAR1A	GGAACATGAGAGTGCCAGCTTTA	CCTTTAACCACTGGGTTGGGT
17 PRKAR1A	AGACTCAAGGGAGGATGAGATTTCT	CCAAGTGGGTCCCAAAAGCAT
17 PRKAR1A	TTTGATGTCACTTGCACTTTAGGTCT	CGATGCCCCACAATTTCACATTT

17	PRKAR1A	CAGCCACTGTCAAAGCAAAGAC	GTTACGTAGATGAGAATTCTAAATCACACTCT
17	PRKAR1A	CACACTGAGAAAGCGGAAGATG	CCCAAGTCCATCCAATTCTAATTCAGG
17	PRKAR1A	TGTGTGTTTGTTTAGCTTTTTGGTGA	CACTGAGAGGTGACTTAAAGAAACCAT
17	PRKAR1A	GCTTAATGTTTGAAATTCACGGAAGAGA	GTAACAATATTGCATGCTCCAGAGG
17	RARA	CAGTCCCTGAACCCAAGCAT	CAGCTGGTTGGCGAAGG
17	RARA	CCTCACCGACCTGGTCTTT	AGCTCCCTGGAAGAGACTCC
17	RARA	CCTGCAGCTGCCCTCTTA	GCAGGGAAGGTTTCCTGGTG
17	RARA	CTCATTGAGAAGGTGCGCAAA	AGAGATCTTGCCTGGCACAAG
17	RARA	TTGGTACTAAGGATGGCGACCTA	CTGAGGACTTGTCCTGACAGAC
17	RARA	CGCATCTACAAGCCTTGCTTT	CATTGACCTCATCAAGCTGTCCTT
17	RARA	TCTGCCTCCCTTCTGACTGT	GAGAGTCCACCCAGCATAGG
17	RARA	CCTCCCTACGCCTTCTTCTT	CCACCCTCCACAGTCCCT
17	RARA	CATGGAGAAGAAGGCCCTCA	CTTGGCGAACTCCACAGTCTTAA
17	RARA	GAACTCTCCACCAAGTGCATCA	GACATCTACCCAGGACACAGG
17	RARA	CCTAATCCCTTCCTAGTGGTGGAT	CCCCTACAACAGCAGACTGAGA
17	RARA	CAGCTGCTCTGCTCGGAT	CAGGCCCGGTTCTGGTTATAAAA
17	RARA	CTCCCCTAGACTGAGACCGT	CGGGTCACCTTGTTGATGATG
17	RARA	TGTCACCGGGACAAGAACTG	CTTGGTCTTTAGGAGCATCTGAGA
17	RARA	ACCTCTGCCCCCTCCTTT	GGCTGTCCGCTCAGAGTG
17	RARA	AGAACTCAGAGGGCCTGGA	CGAGGGCTGTGTCCATGTG
17	RARA	GGGTGGTTCTGCTTCCTCA	ATGCTTCGCAGGTCAGTAATCTT
17	RARA	CACATGTTCCCCAAGATGCTAATG	TCGTATTGTCCTGGCACAAAGA
17	RNF213	GTGGACAGATCTCACGGGAAT	GGTCAGGATTGAATGGGAAATGAAGA
17	RNF213	TGTTCTTCCACGCCATCATCTC	GATTCCTCCCAAGCCTACAGAC
17	RNF213	GCCCTGCTGTCCGTCTC	GCTCCAGCGGCATGGAAA
17	RNF213	CCTTCAAGGTCAGGGTTTGGT	CCTCTGGCTTCTTGAGGTCTTC
17	RNF213	CGTGGCTACAGTTGGTGAAGAAT	CCAGATGAAGCAGTGGGTGAG
17	RNF213	GAAAAAGTCGGTAAGAATGAACAAGGG	CATCCCTACCTTGCCTTCACTT
17	RNF213	AAAAACCAGGAAGCAGATGTCCA	GTTAAACTTGCCAAAACAGCTGACT
17	RNF213	GGTACTGCTCCAGAAGTGGTT	ACTTAGGAAATAGAATCCTGACCCACAT
17	RNF213	CTGGCATCAGTGGCAGATCTT	GAAAGGTTCATGTGGTTCTGCAC
17	RNF213	GGTGAGATGGCCTACATCGTG	CTGTGCTGAATCTGACCTGTCTT
17	RNF213	TCAAAGCATTGCCATGGAAAATGG	AATCCAGTGCTTCCTCCACAG
17	RNF213	TCACCCCAGGATGAACAATGTG	CCTCAACTTTGTTCACTTCTCAGGAATA
17	RNF213	GTCTTCTGGCCCCAAGAAAGT	AGACATCAGCATCAACTTGTTCAAAAG
17	RNF213	AGTACTCTTTAGAGGAGGTGAAGGAG	CTTAGCGCCACTGCAAAGATG
17	RNF213	AGAATTTTCATCCCATAGAGCACTGG	CTTGGCCACACTGTCCTCTAAG

17 RNF213	CAATAGTTGCTGGGACACCAAAG	TCATAGCCCTTAGCTGGGTCAT
17 RNF213	GCCAGCAGTCAGCCTACTT	CCTCCAAATACTGAGACTTTGAACAC A
17 RNF213	ccagcCTTGCCACATCAACTTAT	GGAAATGCAGACAATGCCTTCA
17 RNF213	GTCATGACCGCGTTCTTGT	GGTTGACGTACTCGCCCTTC
17 RNF213	GTTCATTTACAAGCACCAGCAGAA	GAGAGACGCGTCCAGTACC
17 RNF213	GCAGTGAGCTGCCTTTTTCTTTC	GCTTTCCAGAAGCAAGTCTCCAT
17 RNF213	AGTGCTTGCAGCTCACAGATT	GATGTCCTGGAGCAGCTTTTTG
17 RNF213	GATCAACCAGGAGCTCATCCAG	ATCTGAAAACTTTCTCCACACTTCCA
17 RNF213	TCAGCTCTGTTTTAGCAATGACAGT	ACTGCATCGTGCTTTCCACTTA
17 RNF213	CCTCAAATGGTGGTGATTTCTCCATA	GAAAGGATCTTGCATTCGCCAAT
17 RNF213	CAATGTGAAGCTGTGAGCAAATTC	GCCATTTCTGTCACCGTTCCAT
17 RNF213	GGCCTAGTGACAGCAACCTTG	CGATGGAATTGTGGCCTACTCTTA
17 RNF213	aaaaGTGAAATGCTGTGCAAATCTCA	GTGGACACCAGGTCTACGAA
17 RNF213	CAGTGCGTTTTTCATAAAGGTCCAT	GTGACTTTTGGGAAGATGTCAAGAAA
17 RNF213	AAGCACATGGTCACCATGGAT	CGACACTGCCGTTGATGTTG
17 RNF213	CAGCTCACTGCGGGATCTTTA	CTGTCTCCAGGCATCCTTGT
17 RNF213	GAGTACTTTCACAGACAGAGGCA	TGACACGAGGGATTTAGGACTCT
17 RNF213	CACGTGTACCCCAGTTCAGT	CGTCTTAAATACTGGTAAGGTCTTTGG A
17 RNF213	CGGCAGATGTGCAACAGTTTC	GGCTAGGTTTACAGGAAGAAAGCAA
17 RNF213	ATGACATTCATCGGCTTCCATCT	TGTGTCTGGGCAGTTTATCAAAGTC
17 RNF213	TGCACTGCTGTATGGAGCTG	AATGTAGGAGTCAGTCTTCCCAGAT
17 RNF213	CTCTCAGGGTTTCATGAGGAAAACA	ATCTCTCTCGGTGTGATTTTTGCT
17 RNF213	CAGAGGGTGCCCTTCAATGT	CGCATCTCGATGGCAAGGATTTTA
17 RNF213	GGGAGTTCTGCAGCGAAACTT	CTCTGACCAGGATGGGTTTATTACC
17 RNF213	AGCTCACAACCGACAATATGCT	CCGCAGGTCGCTAAGGAATTTA
17 RNF213	CTTTCTCTCCAAGTCCAGCGT	GCAGCCTGCTGTCGTCATA
17 RNF213	CTCCAGCATTTCCTGTTTCACTG	AAGGTCACCACGAACTTCTTGAA
17 RNF213	GCTGTGGGAAAACCAGGCTTAT	ATTTTCAGCCTCCCTGACTCTG
17 RNF213	CGCCAGGTTCTTTCCGAAAC	GAATCTTCAGCTCGATGCAGAC
17 RNF213	CGAGTTTTATTGGCGACACACTGA	CGCTAGATCTTCTTCCCTAACCC
17 RNF213	GAAGGAGAACGTCTTCATGATGGT	CCAGGTGGACCTGCTTCA
17 RNF213	GCAGACTGCTGTGTTCTTTGTG	CAGCTGTCAGCACTTACTGCTTA
17 RNF213	GCTTAAGCATAACCCTGTCGTTTA	ACAATTTCATTGTGTAGGCGAATCAA G
17 RNF213	GCTACCGCTCTCGTCAGCTA	ACTGGGCTTAGGGACTCTGG
17 RNF213	CAGTCTCAAAGTCTGTAACCCCAAT	CTGGAACTGCTTCACTAGATCCC
17 RNF213	CCTGAGATTTTGGCCTTGCAAA	GATCCTTTCGAACCGCCAAATG

17 RNF213	GTCTACGAGCTCCCATTCAACA	GAAACCTGCCCAGAGGAGTC
17 RNF213	AGTTCGTGGACATCTTTCAGCA	CTGACCCTTAGCCAGTATCTAAACAT
17 RNF213	ACTCACCCGCTTTCTCGTT	GCCCTGCACGAACTCCATC
17 RNF213	ACCGGGTGTACCTGGTG	TTCTCACCTGCTGCTTGACAA
17 RNF213	GGTTATCTGGAATTCCCACGGTAA	CACCAACAACTTTCGTCAACACAG
17 RNF213	GCTGATAGCTGTTGCCGACT	GTGCGTATCGACTCGATGCT
17 RNF213	CAGGGCCAGAGAGTAGAGAGTTA	CCACCCCTCAAACCCTATCTT
17 RNF213	ACTTGCAACCTTTCCTTGAAGTC	GAGTACTCGGTGTCAAACTGCT
17 RNF213	GCCATCGTGTCGTGTGTTTAC	GACGTCCTCCTTTCCAGGATT
17 RNF213	CTCCGAAACCCACACTCCTG	CGTGACTGTGGCGCTCATTA
17 RNF213	CCCCTGCCATTTGTATATCGTTGA	CCACTTCAGTAAGACCAGAAAGAACC
17 RNF213	TCCAGATCTGAGAGAACCAGGAA	AGAGTTCACTTACCTCCAGGCA
17 RNF213	ACTGAACTGTACATGCTCTTCATCAA C	GCACGGAACAGAGCATTCGTTAATTA TA
17 RNF213	CCAGGCTCACCATCTTCTCTACA	CCAACTGCTCCAGGTCCTTC
17 RNF213	TTCTGCAGCAGCACATCCT	CCCTTGCCCCTTTCCTCAC
17 RNF213	GGGTTGCTATCATACGGTTCTTAC	CCTGGAACGCTTAGTGGTACA
17 RNF213	GGAAAGTCTCTGTACGTGAAGAGG	CTGATACTGCGCATCCAGGAA
17 RNF213	TGGCTGCCTTTAAACCTCGAA	TGTATTCATCGCCGTACACCAG
17 RNF213	AAAACAATTCGACTGATCGACCCT	GGGACCACGTCTTTACTGATG
17 RNF213	CCTGGCCCATTTTGTCACCT	CGCACCTGGCAGTGTGT
17 RNF213	CCCAGGCCAGATGGATAGGTA	CAAGGGATGCCACCTGGTAT
17 RNF213	GCCCGTGTTCTCTCCTTTTG	CTCACCTTTTGGCCACCTTTG
17 RNF213	CCAAAGAGGCATCTATGTGATCCAG	TCTGTGAAGAGCATAGGTTAGAAGTC A
17 RNF213	GGCCAGTTTCTCTGCTTCAGAA	TCATCTGCTCTGAGGCAAACC
17 RNF213	CAAAGGACCCCGTCTGTCTG	CTTGAGAGAAAGGGTGTTGTACCT
17 RNF213	ATTCTCTCCAGCTGTTTCCCAAG	TCTTATGATAGTTCTCTGAGGTCAGGT T
17 RNF213	ACCATGCTGAGGAGACTGTTTT	GCAGTATCTCTTCTGGGAACTGAGA
17 RNF213	GAAAGTGAAATTCTTCCTGAAATGGC A	AAAGTCATCCAAAGATAGCTGAGGAA AT
17 RNF213	CCCTTCCTTCATCTGCCGTTA	GCTTTGGAACAGGGCTCCTC
17 RNF213	CTCAGACAGTTGGCAAGAAAACC	CAGCTGTGCAGTGAGATTTTCC
17 RNF213	GAGTGGTAACGCAATCACGTTT	GAGGTTTCCTTTCCCACCTTCTC
17 RNF213	AGGCCATGGAAACAGAAAGTTCT	GTTTAAAGATGCAGCTCAGAAGCAT
17 RNF213	TCTGGTCTCTTCCTCTCTGCTG	ATTTAGGAATGTGCTGAGGAGCTTAG
17 RNF213	CGGATCTGAGCCCGGAAAATG	CCCCATAGGTGTGCAAATCCATA
17 RNF213	TCTGTTTGTCATCTGCTGGAAATGT	ACAGGATCAGGTTTATCGGGATCA
17 RNF213	GATGACCCAACCGTCTCCAC	GCACGCGTCATCCTCATTCAA
17 RNF213	GAGCTGCACGCGCAATAT	CCCCCAACACACAGTGAAATGA
17 RNF213	GCCTTCTAGGCTAGTGTCTAGGT	CGTCTGTGATGTGGTTCATGACT
17 RNF213	GTGGCCCATCTCTCTTCTGT	TGGCCTGGTCAGTAACTCTAGG

17 RNF213	ATTCATCGACAGAGACGGCAA	GGGAAAGAAAGCCTTCCACTCA
17 RNF213	CGTCTCTTTTCTCCGTCCCTA	AGGTGCTCAATGAAGTTTTCCATATACA
17 RNF213	GCCTCATGGGAGACTCCAGAA	AAACTGCTCGAACTGGGTGAA
17 RNF213	ACACCATCAACCTGAACAGCTT	GCTGGTGGCAAGCCTGATAC
17 RNF213	GCTACCTCTGAGTCACCTGGTTA	CTCCAGAGCCGACTTCAGAC
17 RNF213	GATGACTGACCCTCCCTCTTG	CTGAAGATGTGAGCATGTTCTTTGTAAA
17 RNF213	AAAAGGTGGCTCCGAGAAGTT	CGATCAGTCACATCCCTACCCA
17 RNF213	TTCTTCTATCCTTCCTTCTCCACAGT	ACCTTCCTCTTCTAGGTGGTTCAG
17 RNF213	CCAGTGCTTACTCCAGAAATGATGAA	AGAGGAAATCTGCAGCTCTGTC
17 RNF213	CCTCGGTTGAATACCTGCAAGA	TCGAAGAACTAGTTTCCCATACTTCCT
17 RNF213	GTAGGGTTTTCCACTGCTCCAA	CCCGCTCCACTTCATAACTGAT
17 RNF213	CGAGGTCACTGAACTGCATGTC	TGACGATCTGCCGCTGAATC
17 RNF213	GCAGGAGTTCGATCTGGAGAA	GCTTGTTCACAACAGACACACC
17 RNF213	TGCTATCGAGTAGGTAATTTGCTTTTGTT	GGACTTAGTGATCACAGCAGACAAG
17 RNF213	ATTTGCGGAAATTTGGCATCGT	CCTTGCCTGAGCTGATGTTCC
17 RNF213	GCTCGGAGAACACCCTTCTG	ACGGTCCTCATTTTCTCGTATTTTGT
17 RNF213	GCTGTGATTTCACTTTCGCAGAA	TTTCCTCCTTGGAGACATGCTG
17 RNF213	CCCATGGAGTGTCCTTCGTG	TTTCCTGGAAGGGAGGTGAGA
17 RNF213	ACAGTTGTGGGTGGCTTCA	GCTCCGCCTCGTGACTC
17 RNF213	GTCCATGGTTCATGCATCTTCCT	CAAAAAGGACAATCAGGCCTGTT
17 RNF213	GATGCCAGCTCACCAGATGA	CTTTCAGTCACTCTAGTCAAGCACTTAT
17 RNF213	CAAGGAGGGAGATCACATTAGTACA	GCAATGGTGTGTTTTAACTGGCAT
17 RNF213	TCCTTCTGCAGGCCTTAAACAG	AAGCATTAAAGCCTGCAGGGA
17 RNF213	CCCCCTGCTAATGACTCCTGT	CGCCTTCACAGATCCAAACCTAA
17 RNF213	GGTTTTCCAATAGTTCGGATTTCCCA	CGCCGCTTTCAGTTTCCTAGT
17 RNF213	TGCAGAACTTTTCCAGAATCCTGA	CATTCCCAGAGTGGAGTTGGA
17 RNF213	TTGTAGCCTTTATGTTTCCCATCACTT	CGGATTCCATCTTCAAAATCTGTCTGA
17 RNF213	ACGAATACAGCCAAATGTAAAATCCTCA	CTAGGACTTCCTACGTTTTGGGTTT
17 RNF213	GCCCAGTGAGGAGTAAACTGAA	TGGCTTAGGTCACGGTGAAAC
17 RNF213	CAGTTTCTGCAGATGGCTCTGT	TGGAGAACCTGAGGGTAGATGG
17 RNF213	CCCAGGTGACAAGCTCACTTA	GGTCATCTAGACCCCAACACTTAC
17 RNF213	AAACAAATGCCCTGTTGGTAGTTA	GCAGTTTCAGACACACAGTCAGG
17 RNF213	GGAGGCCTTGTCACCATCCTA	ACCTTGTGTGTTGACTCCACTTAC
17 RNF213	CAGGTGGTGAGATGCCAGAAA	TGACTTCTACGACAGACAGCAC
17 RNF213	CCTACAGCGATGCCTGTGAA	CTGATTTCCTTTTCGGTGTGAGAC
17 RNF213	CCGTGGCATTTAGTCGTGAG	CCTGAAAAGACTGCCCCCAAA
17 RNF213	TCCTCAGACATCCTCGTCCAG	AAAGACCATTTTGATGAGGCTGTAGT
17 RNF213	CGGCTGCCTACTCAGATCTCT	CACCTCATCTAACACCACCACA

17 RNF213	GGAACAACTGCAGACATGATCTACTC	GCTGGCCATCCACCATATGAT
17 RNF213	GGAAGGACCTGCAGCAGTA	GGCCCAGTTGGAGATGCC
17 RNF213	AATTCTTCGGGCTTCGTGACT	CCAGAAAGATGTCCAAAGCTTGGAT
17 RNF213	GCATGGAAACAGGCAAGATGGT	TGACAATCAGGCGGAAGTTGG
17 RNF213	CACAAAAAGGTCGGCTTCGT	CAAAGTACCCTTGGACTCGGT
17 RNF213	GCGTCAAATGTCGGGTTCAC	CCAGCACCGTGTTGATATCCA
17 RNF213	GGAACTTCAGTGGCAAGGATGA	CCTTCTGAGAAGGCCCAAAGATG
17 RNF213	AGCTGTATCAAAGAAGTCCTGTGTG	CGTCTCCTCCATACTAACCCTGTAG
17 RNF213	CCATGCAGCTGATCAAACAGAA	AATAATCTCCGGCTGCTGGTC
17 RNF213	CCGTTTGGAGTCAGCTGGTTT	TGTAGAGCTTTTCAGCAACGTCA
17 RNF213	CCGGCTGGAGAAGCACTATC	CGAGTGGTAGCCGATGAAGAC
17 RNF213	AGATCCTGCAGCAGACATTCTTC	GTTCTGCAGGTTGAGAAGCAAC
17 RNF213	GGCACAAATACAGCCCCTCT	CAGTTCAGCAGGATCGATTTGG
17 RNF213	TGTGGGACTTTGGACAACTGAG	CTGACAAAGCTGCACTCATCTT
17 RNF213	CTTCACCAGAAGGTGTCTGAGG	CCTGAAGGAAATCTGCAAAGGAGTT
17 RNF213	GGACAAACGCCCTAATATGCAG	CGCTTATCTTGGCTGATGAGATTATTC A
17 RNF213	CATCTAGATAAAACCCTTCCCACCA	TCTTTTCCTGCAGCTCCAAATCTTAG
17 RNF213	GGAAAAGTGTGGTCCATTGCT	GAGCCACAGTAAGCCAGACATA
17 RNF213	AAACGGATTCGGGCTCACA	ACACTGAGGAATCCGGAGAGAA
17 RNF213	TTGGTCGAGCCAAGCTTGA	CTCTGAGGAAGCGGActtgtt
17 RNF213	AGTGTCTCTCTTTCTGTTTAGAGCaaaa	CCTGAGGCCACGGGTTTG
17 RNF213	CCCTGTCACCTGACTTTGCTTT	GGGCTGTCGCCAACCTC
17 RNF213	GCTGGTGAAGGCCATTCTAGG	CCATCTCTCCCGGATGACTCA
17 RNF213	GGAGGGTGACGGCCTCT	TCCGTGTGGTCCTGGAATTG
17 RNF213	CCACAGGAAGTGAGGCTCAG	CTCCTGGGCAGCATCTTCA
17 RNF213	CTCTTATCAGAGTTCAGCCTGGT	GCAGACCTCTCGGGAGACAA
17 RNF213	GCTCACCTGGCAGGGAT	AGCAGCACCTCATCGTAAGTG
17 RNF213	AACCTCATGGTTCTGCCTCTC	ACCAGGTCCAAGCCAAAGTC
17 RNF213	GAAGACTACCAGCTCGTCATGG	CGGTAGTGACCTGCCAGGTA
17 RNF213	CTGGCTGTCTACATGCAAACC	GTAGCTCAGCTGATCTGCGAA
17 RNF213	CCCTCGAGGCCATCCAAG	ACCCTGCCGCTCTCCTTA
17 RNF213	GGCACAAGGTCTACAGCCT	TCCCAGTCCCCATCACAGA
17 RNF213	AGGGTGTGTCCCTCCAAATG	AGGTAAAAGTGCTCCATTCGCTT
17 RNF213	TTGACAGCTGGAAGAGATTTGTGA	CTCAGGGTGCAGTTGCTTTTG
17 RNF213	CAGCTGTGTGCTCTTGAGTTC	GCTGAGGTCTTGTGAGTGTCTT
17 RNF213	GCCCTAACGATGCTATCCTTCAT	CCATGATGATCCTCAGCTTGTCC
17 RNF213	GATCAAGGACCGAGTCGAACAG	CCACAGTCCCCAGCAAATAACTTA
17 RNF213	GGACTTTGGAGTGCTTGCAGT	TGGCTGTCTCTGAGCAAGTCTA
17 RNF213	GTCCAAGAAATGGCTGGGAAGA	AGCTCAAGGATGTGCCTTTCTG
17 RNF213	CTGAGTGAGCCCGAAGAAGAAT	TCACAAAGTCCTTGAAGATGACATCA A
17 RNF213	AGAGGTCACCCTTGCAGAGA	GTGCAGGTGATGGTATTCCTTGAT
17 RNF213	GTGAGTGGCGCCCTCTTT	GAGGCCAGCCCAAGTACTC
17 RNF213	GTGGAATAGTCCCACGCTGAA	AATAGCAGGGATGCGTAGCC

17 RNF213	GGGAGAATGACATTGATGTGGAC	AGAGCCTTCCACAGCTTTTTCA
17 RNF213	CTCAGCCTCTGTCTTGTGTTCT	GAGCCAAGTGAGTGAGTAGCC
17 RNF213	GACTTCAGTGCATTCATGAAGCAT	GCCACCATTTCCACAGTCACT
17 RNF213	CCAGTGGTCTTCCTCCTTATCC	AGAAGCGAAAGCAGGTTCTTAACT
17 RNF213	GAGCATCTCTCAGATTTTGCTCTAGAA	AGACACTGAGGCGCATCTTG
17 RNF213	AGCCTCTTTCTTGCGGGTATC	CTCCTTGCTTCCTTTAGTCACAGTA
17 TP53	gcatctgTATCAGGCAAAGTCATAGA	GTAGCTAACTAACTTCAGAACACCAACTT
17 TP53	GTGCTTCTGACGCACACCTA	GTTCAAGACAGAAGGGCCTGA
17 TP53	CAAGAAGTGGAGAATGTCAGTCTGAG	CAGACCCTCTCACTCATGTGATG
17 TP53	CAAGAAGAAAACGGCATTTTGAGTGT	CACCTTTCCTTGCCTCTTTCCTA
17 TP53	CTCCTCCACCGCTTCTTGTC	CTACTGGGACGGAACAGCTTT
17 TP53	TGCCCTTCCAATGGATCCAC	TCATGCTGGATCCCCACTTTTC
17 TP53	TGCGGAGATTCTCTTCCTCTGT	AATGGGACAGGTAGGACCTGAT
17 TP53	GGGATGTGATGAGAGGTGGAT	CCATCCTCACCATCATCACACTG
17 TP53	GGCTCCTGACCTGGAGTCTT	CTCATCTTGGGCCTGTGTTATCT
17 TP53	GCCACTGACAACCACCCTTAA	CGTGTGGAGTATTTGGATGACAGAAA
17 TP53	ACCACCACACTATGTCGAAAAGTG	AGGCCTCTGATTCCTCACTGAT
17 TP53	GCCAGACCTAAGAGCAATCAGT	GGCCATCTACAAGCAGTCACAG
17 TP53	CCTCACAACCTCCGTCATGTG	CAACTCTGTCTCCTTCCTCTTCCTA
17 TP53	GGGACTGTAGATGGGTGAAAAGA	GGGACTGACTTTCTGCTCTTGT
17 TP53	GATACGGCCAGGCATTGAAG	TCATCTTCTGTCCCTTCCCAGA
17 TP53	GTAGCTGCCCTGGTAGGTTT	AACAATGGTTCACTGAAGACCCA
17 TP53	CATTCTGGGAGCTTCATCTGGAC	GGTCCTCTGACTGCTCTTTTCA
17 TP53	CCTATGGCTTTCCAACCTAGGAA	CAGATCCGTGGGCGTGA
17 TP53	TGAGTTCCAAGGCCTCATTCAG	TGAACCATCTTTTAACTCAGGTACTGTG
18 BCL2	CCGGGAAGCAACAACTCTGAT	GGATCCAGGATAACGGAGGCT
18 BCL2	ACATCACCAAGTGCACCTACC	GGATTGTGGCCTTCTTTGAGTTC
18 BCL2	GACGCTCTCCACACACATGA	CGACTTCGCCGAGATGTCC
18 BCL2	GTTCACCCCGTCCCTGAA	CGGTGCCACCTGTGGTC
18 BCL2	GCGGAGGGTCAGGTGGA	cgcACCGGGCATCTTCT
18 BCL2	TTCATGGTACATCACTGACAATGCATA	GATTTCTCCTGGCTGTCTCTGAA
18 BCL2	GGGCCAAACTGAGCAGAGTC	GGCATTCTCTGCTCTCTCTCTAATACT
18 BCL2	CGGGATGCGGCTGGATG	CGATAACCGGGAGATAGTGATGAAGT
18 BCL2	CTCTGCGACAGCTTATAATGGATGT	AGCTGGATTATAACTCCTCTTCTTTCTCT
18 CDH2	GCAGCGCTCCCGCTATC	AACTGCCTGGAGCCGTTT
18 CDH2	CATGACCAACCTTCTCAGTAACGA	AAGCTACAGACATGGAAGGCAA
18 CDH2	TGTTTGAAAGGCCATATGTGGGA	CTTGCATTATAAAGAGCCGTTTACAGAA

18 CDH2	TATGAAAACAGTTAAAATTGTGCCACCTT	GTAAGCACAGTGGCCACCTA
18 CDH2	ACCCAGTCTCTCTTCTGCCTTT	TGTTTCCTAGTACTCAGGAATGTCTTCT
18 CDH2	GCACAGCATAGAACATAAGTAAGCTACA	ACCCTTATTTTGCCCCCAATCC
18 CDH2	CCCTTCTTCTTGGCGAATGATCTTA	GCTGCAGAAAATCAAGTGCCA
18 CDH2	GGTGCTGAATTCCCTTGGCTAA	ATGCAAGCATTCTTATTTCCTGCAAAA
18 CDH2	TCAGTTTTCCAGGCAATTGAGTTTTG	CCGAGAATCACCAAATGTGAAAAACAAT
18 CDH2	CATACCATTGTCAGAAGCAAGGAAAGTA	CCAATTGGCTAAAAATAGATCCTGTGAA
18 CDH2	CGGTCCAAAACAGCAATTGTAGTTATTT	GCCAGTTGCATTTGGAACTAAACC
18 CDH2	TGAAGCACATAAAATTAACTTTTCATGCCA	GCTTTTGATCTTCCTTTATCTCCAGTGA
18 CDH2	CCGAGTGATGGTCCAATTTCTCTTAATAG	TGACAATGCCCCTCAAGTGTT
18 CDH2	CAAGTCTCTGCCTCTTGAGGT	GGAGAGAACTGTTCAGCATCATTTG
18 CDH2	GAAAGAAAAGGATCCAAAAAGCCTCAT	CACCAGGTTTGGAATGGGACA
18 CDH2	AGGCTTTGATCCCTCAGGAAC	GTTGAGGGCACATGCAGTAGATATT
18 CDH2	GGGTTCTCCACTTGATTTCCATT	GTTTTTCAGCTGTGAGTTTATTCTTGCT
18 CDH2	ATGGCTAATTTGTTTCATGAGCATGG	CCTGAAGATGTTTACAGTGCAGTCTT
18 CDH2	GTCCTTCATGCACATCCTTCGAT	AGGTAGCAATCATTGCACACATTTTT
18 CDH2	CCTACAGAGATCTAACCATTAGCATGAA	CCTGTGGGAATCCGACGAATG
18 CDH2	GGCGTGGATGGGTCTTTCAT	GTTGTAATTTTCTATCTTTGTGCCCATCT
18 CDH2	GCAGCTGTTGGAGAGATCAGT	CCTGGAACGCAGTGTACAGAATC
18 CDH2	CCAGTAGGATCTCCGCCACT	CCTCTGACTGAGGTTTGTCTTTCTC
18 CDH2	ACACAGAATTATCAGCTGGTTGGAAT	ACTGCGGTACAGTGTAACTGG
18 CDH2	GATAATGAAGATACCAGTTGGAGGCT	GAAGTGTGATGTGCCCCATCT
18 CDH2	GCTGAACATCCTAGAAGCCAAGA	GTTTGCCAGTGTGACTCCAAC
18 CDH2	CCACAATCCTGTCCACATCTGT	CTTGAAGCTGGTATCTATGAAGTTCCC
18 CDH2	CAGCAAGCACTGTGCTAGTAGA	ACGGTTCAAGAAACTTGCTGACA
18 CDH2	TCAGTCATCACCTCCACCATACA	CACCATATGACTCCCTGTTAGTGTT
18 CDH2	GAGCCACTGCCTTCATAGTCA	TGGTTTTGTAAAGTGTAACCCTCTCTT
18 CDH2	CAGCCCTAAAGCCATATTCGGAT	CTGAGCATGCCAAGTTCCTGATA
18 CDH2	GGGTCTCTTTGTCTTGGGCATA	CTTTTACAGTGAAGTTTAGCAACTGCAA
18 CDH2	CCAAATGACTGATAACAGGAGGGAA	TGATGAAGAAGGTGGAGGAGAAGAA
18 CDH2	AAGCACTGCTCACCTGGTC	TGTGCTGATGTTTGTGGTATGGAT
18 CDH2	CGTTCTTTATCCCGGCGTTTC	GCATTTCAGGCCTTCAGTTCATATAAGT

18 CDH2	CACTGTGAGTATATTGTGATGTGGAGA	TCACCCAACATGTTTACAATCAACAATG
18 CDH2	CACTGTGATGATGTCACCAGTCT	CAATTGATGCTGACGATCCCAATG
18 CDH2	GATTCTGTACCTCAACATCCCATTGA	GGTCCCTGGAATGAAGGGTAA
18 CDH20	cccagtcGTAAATACCGACTTCTA	GGAGCATTGTCATTCACATCTAAGACTT
18 CDH20	TTGGAAGTGTTCCTGTCACAATCA	GGTTAAGCAGAGTGCACCAC
18 CDH20	TCCTGGAACCCCTCCTTCTTT	GTCATCACAGCTGCACACTTG
18 CDH20	CACAGGCACACTGACCATC	GGGAAAGCAGCCCCTTACTC
18 CDH20	TGACGGATTATCAAGAATGCGTGT	GCACCATCTCCATCCACAATAGTATAT
18 CDH20	TGAAGGCATCAATGCAGAGATGAA	AGCACCCATGAAAGTGGTTTAGAA
18 CDH20	TTTCATATGGTAGGCACTCAGTTGTC	GCACCTGTTGTAATGTCAACATAGAAAA
18 CDH20	GAAGCAGTGACCCTGGAAGAT	GGAGACCAACTTACTGTGCTACTTAC
18 CDH20	CAACTCACCTGATTTCATGTCCCT	ATAGCCAACCATCCACCTGATTAC
18 CDH20	CTTCGACTTCCTGACGGACTG	CGGAGAACGTCTGGATTTGGA
18 CDH20	CAGGGAGACACCTAGAAACCAATC	GGCTTGGATAATCACTTCGTAGTATTCTT
18 CDH20	CATGAACATGGACAGAGAAGCCA	TGTGCTCTCTTGGGTTAGGTACT
18 CDH20	CTCAGGATTGCATCCAGCCTTTA	AGAAAAATATGGCTGGCCCTGAA
18 CDH20	CCAGGGTGGTGTACAGCATTC	GGGTCTGTCATAGCAGCAGTTATAAATT
18 CDH20	GACTTGAAACGGGATCTCATTTAGGA	GCTCATTCTACCAGAAGTCCACA
18 CDH20	TTCTGAAAACCTGGCAATCCCA	GCTTGTCTGACAGGAGTGCTT
18 CDH20	GGCATCCTTTTGAACGTTGACA	TGAAATGGGCCCAAGTTCAGAA
18 CDH20	CCCAACACCACAAGGTGAATTAG	CGGGTCATTTCTACTTGGCATTTCTT
18 CDH20	CAATCCTCACCTAGAGATGCGTT	CTCAGGCACCTCCACAAAGT
18 CDH20	CCTGTGTTTGAACCTGGCTTTT	GAAGGGAAGATTCCAGCATATAAGGA
18 CDH20	GAATTAGGTGCTTTCCTCTCCCA	CCTCTGAATGGCGTGGATGTC
18 CDH20	CATCGACGACACCACTGGA	GGCTCATTGTCATTGATGTCTTGAATTT
18 CDH20	CCCGAGTCAGAGTTCATCATCA	AATCAGCGAGTCACCACCTTAC
18 CDH20	TCGGTAAACACACTCTCCTTTTTGT	GATGTCGAAGGCCTCGGT
18 CDH20	GAGAACATCGTCCGCTACGA	GCACGTCTGAGGCACGTA
18 CDH20	GCTGCCCGAGATCGAGAG	CCGTCCCCCTCGAACAT
18 CDH20	TTCGACTCCCTCCAGACGTAT	GTAGAGCTCGGCCAGCTTC
18 DCC	AGAACTGTTGGTTTGATATGTCAGCTT	CAAAATGACAGATTGAGCCACCATAC
18 DCC	GGCATGTTTCTCAGGAGTGTGTA	CATTGTGGCACCAATATTGACTCAC
18 DCC	ACACTCTCTTTTTACTAGACAGGTGGTA	AATGGAGTAGTGAACATTTAGCCAACTT
18 DCC	TTCACATCCAATGACAAGGCCTTAT	GGGAGAGAAAGTGGAGATGCTTAC
18 DCC	aagggAACCTAGTTGATACTATGAAGA	ACTGGGCACTTTCTAGCGTTG

18 DCC	CTTTTTCTTTTGCAGCTGTCGTGA	ACTTCTTCCTGCTCCGAAACC
18 DCC	CAACACTCTCAGTGGACCGA	CTAAGGGATAAGGTCAGAATGCAACA TA
18 DCC	GCGATTATTGTGCTTTATTTGGAAGAC T	GGGCTCCCCAATGACTTCAC
18 DCC	GGAGACACAGTGCTACTCAAGT	AAATTCCAATGTCCCCCGGTT
18 DCC	CCTCTGGAGCATTGCAGATCA	AGGCAACAAAGAGCATTGCAATAC
18 DCC	TCATGGCCTCTCCTCTTGTTTC	GACTTCACCACCCCAAGTATCC
18 DCC	CCCTAGGGAGGAAGCAACTTAC	CCAAAGCAAGGTTCCCATTGTC
18 DCC	TGCCATGCATTTAACTCATTGTGATG	AGAGATCTGGGACCGAGGTG
18 DCC	CCCAGTTGATTATTATCCTTTGCTTGA TG	CGCACCTCAGACGTCTTTTG
18 DCC	GCAGACAACTCTGTCCCTAAGAA	TCCAGGAGAGGTTTAAAACAATGTGT TA
18 DCC	CCCGAAGGTGTTGGCTGAAA	AAGAGCCACTTACCGGTTACTTG
18 DCC	TCTTCGGAGCTTCCTTGTTCAG	CAAGCGCCAAATCTCTTGCTA
18 DCC	TGGTGTTCTGCCGTGCTAC	TCAAGGGAGGAGTCCAACTCAT
18 DCC	GCCCCAGACTAACTGCATCATC	ATCACAGATACTCACCTAACCTCTCA AT
18 DCC	GACAGCAAGCAGCGATATTATTCC	GAAACCATCATACCAGGGAACAGTT
18 DCC	GTTCACCTCACTACTCTTGCACAT	CGAAAGGTGTACATGGCTTCTGG
18 DCC	ACTTGGAGAAGAGACTGACCACAT	CAGCATAGGTGGTTGTTGGG
18 DCC	GCTCACTGTGGGAAACCTGAAG	GGCCCGTTCCTTTTCATACTCA
18 DCC	GCAGTGAGTGAAGGACCAACTA	ACTCATTGGTGGAGGTAGCAAAG
18 DCC	TCACACTTACGTTTGGGTTACTTCTTT	GGGTCGTCTTTCTGTGTCGAATTTTA
18 DCC	CACTCCGCAGCTTTGCTAATC	TCATGCACTTAAATAGAGGCACCAAT AA
18 DCC	GAACACAAAATGGATTTATTACCGGC TA	CAAGACTACACCATGTGAACACTGA
18 DCC	CCCAGAGCAGTGTTTTAAACTCAGT	GGCATACAGGTTGGAAGGATGATTT
18 DCC	GCAGTTCCGCCATGGTTTTT	TCACTAGGAATGACCACATCTCCA
18 DCC	GGAAGTGCCATTGAATAGAGGTTCT	AGTGCCAGATGGCTTTTCAATATTTT C
18 DCC	CTGGGTGTTTGGAAGCAGAAC	GCCTATCACCACTGATTGTTTCCAT
18 DCC	TTTGGATCCATCATGAAGAAATGGAG AT	GCTTCCCAGTTGGGTTTCTGA
18 DCC	AAGAACATCCCAATTGATGACTGGAT T	TGAAGAGGATAGGATCAGAGAGTGG
18 DCC	CAAAGTTGCCAAGACCTCACAC	GGGAAGACATTTCCATAAAAGGAGTG AA
18 DCC	AAGCACGAAATTCAAAAGGAGTGG	TGCAGCCATGTTGCTAATAGAGAT
18 DCC	GAACATATTTCCCTGTGCTCTCTTGT	CCATCTTTCTTCCACTTGATCACTG
18 DCC	GAGTCCGACCGAGGAGTTC	TGGTGTCTGGAATGAAGTATGTTTTGT
18 DCC	AACTTTCAAATGGGTCTCTGCTGAT	ggaagaATCCACCTACCTGCTAC
18 DCC	GATGCTTGCTAATAGGTTGGCTCT	CGGACAAATCGGCTGGAAAC

18 DCC	CAGAGATGTGGTCCCTGTCTTG	AGCTGAATTTTATTGGTAGCATCACCT
18 DCC	TGTTACAATTTTGCTGAAGCTTTTGGA	GTACCATTGACTGTCATGGCTGA
18 DCC	GGAAGTCAGTACAGTTTCCAGGTG	ACCTTTTGAAGCTGAGATAACAAGAAGA
18 DCC	CCTCTGCTGTTTGGGAGTAGATT	GTGTTTGGTTTGAGGCCTGTTG
18 DCC	CAGAAGACACAACATCTCTAAGTTACACA	GGTCCAAAGTAGACATTTCCTCACA
18 DCC	TTTGTTACTAACTCTTGGCTCTCATTCTC	GGAAAATTCACCTCATCCATTCAGAAAT
18 DCC	CCTAAAATCAATGCCAATCTCCTGGATA	GATCTTGCTTTTCCAGCCAACC
18 DCC	CATGTGAAAACAGCCTCCCTTG	TCTGTCCCTTGATGAAGCATGAATTTTA
18 DCC	AAGCCCAGAACATTAGGAACTTTGT	TGATCACAAGCAGGTTGCTGTT
18 DCC	GCAAGTTCCAGGGCCAGTA	AGTGTGGTCCATTCCTTCACC
18 DCC	GGCAGTGTCACTCCTCAGAAG	TCTTGGGAACCAGCTGTTACCTA
18 DCC	TGTTTTGAGGAAAATGTTTGCCTTGAG	CTCCTTCTCCGGCATTGTTAAAAG
18 DCC	CTCCAGAGTCAAGTTCCCATTATGTAAT	ACTTTGTCAGAGGAAGATTAACAATTTCACT
18 DCC	AGTGTCTTAATTTGAGCTTTGGTGGA	CATCATCTGTCACATTGGAGATAAGCA
18 DCC	AAGTATTCTTTATTGGGTGGAAGCAACT	CAATATAAACTCATCTCCAGCCACTAACT
18 DCC	TTGTACTGACATTGTGACATGCTCT	CCCCAGACACTCTTGAATTAACCATTTA
18 DCC	TCATATGATACTGTGTTTTCCCCTCAT	GATAGCCAGAAACACAACATTCCAG
18 DCC	CAAAGACCATCCAATGTAGTAGCCATT	AATGTGAAATACCTGAGTTGGATGACTT
18 DCC	GCTCATTAGAAGGCTGGAGTGT	GCCTTCCAGTTTATAAGATAGTCCATCAA
18 DCC	ACAGTTTTGGTGTTTTATGTCTCCAGA	CCGTCAGAAAGTGTAACCACTGTT
18 DCC	CCGGGCGTCTCTACTGATGATAT	TTAGTCAGGAAGCCTTGAAAATGGAA
18 MALT1	ATTCTGTAAGTGATGCTGCTGATGTTAT	TGATAATGCATTTTAAAGTTCTACTCACCCA
18 MALT1	GCAAACAACTGACCAGCCTTT	CATTTGGTCCCAATGTTATTCACATTTCT
18 MALT1	AGGTGTGTTTTTGATGAGTCATCCA	CTGGCTGAATTCAAAGGTGAAATTGTTA
18 MALT1	GCAGGCTTTTATGTCTGTCGAGTT	GGGAATCCCCATCCCAACATTC
18 MALT1	CCACCGTGGTCCAGATATATAGCA	CTGTGCAACCTTTTTCACCCAT
18 MALT1	CAGCCTGTGTCTGCTGAAGTTA	CCATGAACTCCTGGAGAATAATCCTAAG
18 MALT1	CGAAGGACAAGGTTGCCCTTT	CCAACAGTGAAACCACTTTGAAGTC
18 MALT1	AAAGCTCCTTTGGTGGATGTGTA	AAGGAGTAAAAACTCATCCACAGCAT
18 MALT1	AGATGCGTAATGCTGTGGATGA	ACTCTTCCATTCAACAACATGTTTACCT
18 MALT1	CGCGCTGCCAGATTTGTTC	TGTAGCGGGTCCCCCAA
18 MALT1	GAGGGCCATGTCGCTGT	TGATCCAGGAGCTCGCTGA
18 MALT1	AGGGCCATGTCGCTGTT	GGCGCCTGATCCAGGAG

18 MALT1	GCCTCTTATCATCAGAAAACCTACCA	CTCCGGTGGTTTGTAAACTATACTTGTAT
18 MALT1	CAGCTGAGTTTTCCAATGTCATGAT	TCACTGTATTTTAAATAAAAGAAGAGACTCACA
18 MALT1	GGGTGTGTATGTGTGAATATTTTCAGA	CCCAACATTCACTTCCTGCTTGT
18 MALT1	ACTCAGGATTGGAAGATACTGTAGAGG	TCTGGTAAGGACCATTAGACATAAGACA
18 MALT1	CCAGAATTCACGAGAGGATAGTATCAGT	GATACCAAGTAGCTGCCAGTTTCT
18 MALT1	TGCAAATAAAGGCACACCTGAAGA	GTCTATGCCAAAATAAGTTTCCAGTTTCA
18 MALT1	GGGAAGGAAGACTTGCTTTCAAACT	GACAGCTATCTGCTGGTGTAACATT
18 MALT1	CCATTCACATCCTGGTAATCCAAGT	TGTCTCTACTGGCACATTACTTCTACTAA
18 MALT1	GCTCACCATGCTTCATGTCATT	GTGCAGTACTATTTCACAGGCATCTA
18 MALT1	ggtGTCAAGGAGCAGAAGCTTT	TGCTCTCCTTTTTCTGAGTACTTTTCTC
18 MALT1	GCAACTGGACATCCTTTTGTTCA	ACCTAGCAATGTCTATCAGACTCTTTTA
18 MALT1	ACTGAGAATATAACCTGATAATGTAGGATAGT	CTACTTCGAATCTCTAGAGCCTGTTTG
18 MALT1	AAGTGTCACCTTACCAAAGGCAA	TGTAGATTCCGCACAAGAGATTCAG
18 MALT1	ACAGGCTCTAGAGATTCGAAGTAGTTTA	AAAATGGGCTTTACCGTACCATGA
18 MALT1	GGGAACTTGACACATCTCTTTAATTATAGG	ACACAGATTTGCAACTTGGATTCAGA
18 MALT1	TTTAAGGAAGTGTTGATGGCGTCT	GGTAGTGAGGAATAGGGCTTCCA
18 MALT1	CATTGGTTTTACAGTGTGTTGCTGT	CCAAAAGACCCCAAAATCAACTTCCTA
18 MALT1	CTTGACGGCTGCCCTTCTTA	GCATGCAGGAGATTTGGTTTCAAA
18 MALT1	TTCTCATTATTTATACCCTTTTAAGAATGCTCT	GGTGACTTTTAGTGCATCCAAGATTG
18 MALT1	CCAAGCCATTAAAATAGAAGAGGAAGCA	CTTGCTATCTTGACTGTCTCGATCATTA
18 MALT1	CCAAGGAACCTACTGGTGTCATGTATA	TCCTTTGCTTAATTAAACGTTGCAGTTT
18 MALT1	GCAGTCTGTATGTTCAGAGTGCATT	TCAGACCTATATGGATTTGGAGCATCA
18 MALT1	CGTTGATGCTCCAAATCCATATAGGT	TGTCATGGTCGATATTTAACTCCAACAA
18 MALT1	AACACTATAACATGATTCACTGGCTCAA	GTTTGTATTACTCACCAGGATGACCA
18 MBD1	AACCTATCCCGTCCACCTGA	CTACGGCGGATGGACTGT
18 MBD1	CAGAATGAGCTCTGCTCCCT	AGCCGAGGGTGTGGAGTA
18 MBD1	CACAATCCTCTTGGGTCTGACA	TGAGTTGGGCAAGTGGAAAGA
18 MBD1	GCTTGTCGCAGCAGAAGTC	CACTGGAATAGGCGTTGAGGAT
18 MBD1	TCTTCATCCTGTGGACCCATCA	CCACTTTTCTTCTTCCCTCTTCCTC
18 MBD1	cctaacATAAATGGTCAGCAGAAGCTTA	TGGATAGACTGATACTGCTTTGTTTCC
18 MBD1	CCAATCACAACCCTCACCCAT	CTCGGAAACGTCAGGTTGGA
18 MBD1	GCCTTCCCTCCACCCACT	GCTGAAACCACCCTTTCTGATCTT
18 MBD1	CCTTCCTGACCTCACCACTCT	CACCCAACCACTGACCCATATT

18	MBD1	ACCTAGGTATCTCATGTTGTGAAGT	GCTAACCCATCATGGCCCATC
18	MBD1	ACTCCTGACTCTGCCCACTA	CCTTTCAGGCTACTGCTGCTT
18	MBD1	GGCATCCCCAGCACTGA	TGCCTGTTTCCTACTTCTCCCA
18	MBD1	GGCTCTTTCCACTTGCCCAA	CTGCCAGGTAACAGAAGACTGT
18	MBD1	CCGGCTATACTTTCAAAACTCAGG	ACCACATTGACCTATGGTGTTAATTCTT
18	MBD1	CCGTTCACACTTGCAGAACAG	CCGTGATCTCGTCCCTACCA
18	MBD1	CCTGGCAACCAATCTAGGGT	CATACAGACCTGCTCCCTTTTCC
18	MBD1	GGACCTGCTCCAGCTCCTAT	CCACACCTGCCTTTCCCATC
18	MBD1	GCTCATTCCAGCATCTGCTTCTT	CCACCCCATTTGCCTCTACAG
18	MBD1	CTCAAGAGAACCATCATGGCTCAT	GATACCCAGGACGAGTGGAC
18	MBD1	ACCAATACGGGAGAAGTCAGGA	CTGAGACCAAGTCTGCTGCAA
18	MBD1	GGGAGGATTGCAGCAGACTTG	CTGACCTTGTGTTTTTACGGGAAG
18	MBD1	CATGCATGGGACAGGCAGA	CCTAACCCTATGCTCGCCTTT
18	MBD1	ACCTGCACAGGACTGCTTG	CCCGACGGCACCATCTT
18	MBD1	CAAGGTGGGCTTCAAGGTAGG	TCAGGACACCAGGTAGCCATA
18	MBD1	GTCCAGTAACTGGTTTCTGAGAATCA	CTTTAGAATGGGAGTATCACAGTGTCAT
18	SMAD2	TGATCAAACCTGGGATCTAACAAAACTT	AGAATGGGCAGGAAGAAAAGTGG
18	SMAD2	CCAGACTTTTCACTGCTTTCTCACA	GCTTGCTGCCTTTGGTAAGAAC
18	SMAD2	CCAAGTTTTAGGAGATTCAGAAGGCAAA	CTAACAGAACTTCCGCCTCTGG
18	SMAD2	GTTTTCTGGAATGGAGTGAGTATAGTCAT	CTCTTTGCCATTAACTTGGATTTCTTGA
18	SMAD2	AGCAACAAGAAAAACTAAGCAAGTTGAC	AGTTTTTGCTGAGTGCCTAAGTGA
18	SMAD2	GCTCTGCACAAAGATTGCACTA	TGCTTCCAAAGTCACACTGAAATAGTAA
18	SMAD2	TTTTGGTATGCGTCTCAACTTCTCT	GTGAAGATGGAGAAACAAGTGACCA
18	SMAD2	CCTGTGTCCATACTTTGATTCAACTGT	GGTAGTGAGATTATGTTGCAGCTGT
18	SMAD2	CTCCAGAATATGCAAGAATGCAATGAAA	GAATGTGCACCATAAGAATGAGTTTTGT
18	SMAD2	GTATTCTGCTCCCCACCCTTTC	AGATCTTCAACAACCAGGAATTTGCT
18	SMAD2	AGAAGAGCAGCAAATTCCTGGTT	GGAATCAGTAGATGATTGCATGCTCATA
18	SMAD2	GAAAGCTGGTTTTACTGCACACA	GGTTCTGCTTAGGTTTACTCTCCAA
18	SMAD2	ACCGTGGCATTTCGGTTAACA	CTGCATTTTGGTGTTCGATAGCATATTA
18	SMAD2	AAGGTTTCTCCAACCCTCTGATTTAATT	TTGGCTTGAATTGTTCACGCATT
18	SMAD2	TCTAAGATTCGACAGAGGGCAGA	CCATGCTTCCATGTTCACATCAATTTT
18	SMAD2	GGATAGTAAACAGTCCATAGGGACCA	TGGACCTCTACAGTGGTTGGA
18	SMAD2	GGGATCCCATCTGAGTTAATACTTTG	CCTGCCTGTGGACTTGAATTTCATAAT
18	SMAD2	ACACTAAAATTTTCCTGGGTCACAAGA	ACCCTTACCACTATCAGAGAGTTGAG
18	SMAD2	AAACTTGCAATATTCCTACCTGGTGT	GGCCTGATCTTCACAGTCATCA

18 SMAD2	GCAGTTTTCAATTGCCTTGAGTTCA	CTTTTCCCCTTGCTTCCCAAC
18 SMAD2	GCTATTCATTAGGATCCCTTTCTCGGA	CCAGCAGAACTATCTCCTACTACTCTTT
18 SMAD2	GCAACTTACCCAAGCTATGATTAACAG	TAAAGCTAGTTTTATGATTACTTGATCAGAAGT
18 SMAD4	CAGATAGCATCAGGGCCTCAG	TGCTGTCTAAAAATTAAGGCCCACAT
18 SMAD4	AGGTCTTTGATTTGCGTCAGTGT	GGAGCTATTCCACCTACTGATCCT
18 SMAD4	TTCCTAAGGTTGCACATAGGCAAA	CCCAGCTTCTCTGTCTAAGTAGTAACT
18 SMAD4	ACCACGCGGTCTTTGTACAG	CACCCAGATTTCAATTCTTTTGACAACA
18 SMAD4	GCATAAGCTTGTTTTAAACAATTCCTAACCT	AGGCTGGAATGCAAGCTCAT
18 SMAD4	TCTTTTCTTTAGGGCCTGTTCACA	GTGCGTTTCAATCACCACTAAATCAAT
18 SMAD4	GCTCCTGAGTATTGGTGTTCCA	CCCAAACAAAAGCGATCTCCTC
18 SMAD4	ATGATGGTGAAGGATGAATATGTGCAT	GCAGTGCTGGTAGCATTAGACTC
18 SMAD4	CAGCTCTGTTAGCCCCATCT	CAATGATTAGGAAAACTCAACTTGCTGA
18 SMAD4	TTAGCTGTTGTTTTTCACTGTTTCCAAA	ACCTTGTCTATGGCACATCAAACT
18 SMAD4	GCCTGTCTGAGCATTGTGCAT	GATGAGCTCCATTTGTAGTTATAGCTGT
18 SMAD4	GTTTGGTAAAGAAGCTGAAGGAGAAAAA	AATCTGCCACCATAGAGGGTATAGAA
18 SMAD4	ACCCTGTCCCTCTGATGTCTT	GAATTTCAATCCAGCAAGGTGTTTCTTT
18 SMAD4	CGGATTACCCAAGACAGAGCATC	TTAAGGGCCCCAACGGTAAAA
18 SMAD4	tCTAGGTGGCTGGTCGGA	AGGTGATACAACTCGTTCGTAGTGATA
18 SMAD4	AAAATGTGATAGTGTCTGTGTGAATCCA	TCGCGGGCTATCTTCCAAATTTATAATT
18 SMAD4	TGCTACTTCTGAATTGAAATGGTTCA	GATTACCTACCATTACTCTGCAGTGTT
18 SMAD4	AGATCTCTCAGGATTAACACTGCAGA	GCCTGCCGCTCACACAA
18 SMAD4	CAAATTAACCCATGTGGGCCTTAA	GCCCTTACAACAAAAACAAGAGCTTAC
18 SMAD4	AATGTACCATGTTAATGTCTTCTTGTTCCT	TGGCTGACCAGTAAATCCATTCTG
18 SMAD4	CGTGGCAGGAAACATCCCT	ACTGCTCAAAGAAACTAATCAACTGA
18 SMAD4	AAAAACTGTGTTGTGGAGTGCAA	CCTGAACATCCATTTCAAAGTAAGCAA
18 SMAD4	GATGGATACGTGGACCCTTCTG	ACATGGGAAAACATAACCTTGAATAAAAG
18 ZNF521	CCCCCAAATTGAACTGAAGTCATGT	GGGAGGCACCTTCAAGTGTC
18 ZNF521	CTTCCCACCTGTAAAGCAGACT	CATTTCTCTTCATGTGTTTTCCTTGACA
18 ZNF521	CGGCGACAAATGGCACATTTA	GCAGTAGGCTGTTCAAACACAAG
18 ZNF521	GGACCGTTTCTTGAGGAGGTTA	AAGTGAACTCCAGAACCACATCC
18 ZNF521	TGTGGAGTTTTATGTGGCGATCTC	GGCTTCCATACCCGTGTCAA
18 ZNF521	GAGCTCTCGGTGGATGGTTT	GGGCCAGAATGAGAATCTGAGTG

18 ZNF521	CCTTGCCTTTCCCCTCAATGG	CCAAGCAAGATCTGGTGAAACTTGA
18 ZNF521	CGGCTAAACGACTTGTCACAGAA	GCAGATGGAGTGGATGTTGAAGA
18 ZNF521	GACCATATGGCAGGCCATTGATA	GACGTTCCACATGCAAAAGACAG
18 ZNF521	GCCAAGAGCAAGTCGGATCA	ACTCTGCGTGCTGGTTGAAAT
18 ZNF521	TCTGAACTGCAGACCCATTCC	GAGGAGTTTTAGAGCATTGCCAAAT
18 ZNF521	TTCTGTGATATCGCTCAGCGATT	ACTGAAGATGGAGAGGCACTAGATT
18 ZNF521	CGTCTTCCGGCCTCTTCTTAC	GTGTTTCTGTTTTTGGTAGCAAGAAGA T
18 ZNF521	CGCTACCCACAGTGACACTC	GGAAGTATAGTTTCTTTCAGGGCTGA AT
18 ZNF521	CCAATACACACAAAGCATCATGGT	TGTACACAATGTCCACAGAAGTTTTT CT
18 ZNF521	ATTACCTGCAGCTCTGTTTGGA	CGGAAACAAAATAACCCAAGTTCATC ATT
18 ZNF521	CGAAAGAAGACAAAGGTGTGCAT	GTGCAACAAGGAATTCCCCAAC
18 ZNF521	ATCGTGACTGTTGTGGGACTC	GCCTTCCATGCGATCATTTTGT
18 ZNF521	GTCAGGGAATTCCTCAAGTCAGG	TCCCTCCCTTTTAACTCTTACTGAACA
18 ZNF521	CATGGTTGAGCTGTCCACTGA	CATTTGTTCTGAGAGTTTCCACACAG
18 ZNF521	CAATGAACCTGTCTAATATGCTCTTCC A	CCGAAGTTGTCAACGACCTCA
18 ZNF521	GTAACATGCTTCAGCAAGGATTCTT	TGCACGTCCCACTGGAGAATATA
18 ZNF521	CGGATGTGTTCCTGAAGAGTGT	CATCTTAAGCAAGTGCATGAAGCTC
18 ZNF521	CATGTGGCTGTACAGTTCCTCAA	CAGTGTGTCTACTGCCACGAG
18 ZNF521	TCAAGACTCTTACTATGCGTGACTTTG	CTCTCGAACCTTCTTCTCCGA
18 ZNF521	CAGTGTTTTTCTCGCAAGTGTTTTTCT A	GAAAACCAAGGGAAAGTGCATAAGT G
18 ZNF521	GAAACAATCAGACCTGGGTCCT	GCAGATTCACCTGAAAACTATGCACT TA
18 ZNF521	GGGAGGTCTCCTCTACGAAGA	AGGACTGGAAGATGAAGGACACT
18 ZNF521	GTGTACTTAGCACCACATTGATTACA GA	AACATTCCCTTGGCCCTGAATTAT
18 ZNF521	CAAAGGACTCACCGCAGAAAATG	AGCACAGTAACGAAAAGAAAGTCTA TAGG
18 ZNF521	AATATGGGCCTGTTCTGGCTTATC	ACATGACTGGTCCCTCGAGTA
18 ZNF521	CCTGGATTTCTTCCCATTGTGGAT	CTCCCAAAGAACCAGTAGTAGAAGTC TA
18 ZNF521	CCTCACACTGACTGCACTTCTG	TCACACGTCCAACAAGCCATA
18 ZNF521	CATATGTTCCCGGAGGCCATTT	CTATGGAAACTTTGCTGCAGAATCAC
18 ZNF521	TGGCGAATTTGTACAATAGGAACAAG AA	GGGTTTCTCACTGACTCTTCCC
18 ZNF521	ACAGCTGTAGGTAACTTTTGCTTGTT	GTACGACTCCAGATTCCAACCTC
18 ZNF521	CGGAAGTCCCAGTTGCAAGAT	CCTTCAGAAACACCTGCTGGAC
18 ZNF521	GTCTGATGTTGTGGTCTCGGA	GCAGACTTTGCTGACCAACAG
18 ZNF521	CGTGCAAAGAGTAAAACATGTTCCC	AATCACTGAAGTTTGTTTTCCCTCTCt

18 ZNF521	CCGAGGAAAAACAATGATGGTTGA	GTGTCAGATGGTTTTCTACAATGAATGG
18 ZNF521	TGCAACATGAACCTGAATATCCCA	GCAACATAGGTAAGATCAGCATCTCA
19 AKT2	CCAGCGTGGGAGAAAGAATCT	GCTGTCCCGAGCTAGGTGA
19 AKT2	CGCACACCCTGCCACTAA	AGCTGTTCTTCCACCTGTCC
19 AKT2	CTCCTCTGTGAAGACACGCT	AGCTGTCACTTGCTGGTTCAT
19 AKT2	ACCCACAGCTCCTCTCCAT	ACCCTGCTGCCTTCATCC
19 AKT2	TTGGCCTCACACGTTCCTAC	TGGAAAACCTCATGCTGGACAA
19 AKT2	CAGTGATCTTGATGTGGCCATCT	GCATGGGAGGGTTGATGTCC
19 AKT2	TTGTACCAGAAGATTAGGGCTCTCT	CTCACCCGCAGTCTGTCT
19 AKT2	GCCCTCCTTGAGAAGTGAGTTA	GTCGACACAAGGTACTTCGATGAT
19 AKT2	TGATGGACTGGGCGGTAAATTC	CACTCGTGGTGACCTTGGTT
19 AKT2	GAGCCCCCTGAACTGTGTTAT	AGTGACTCCTCCACGACTGA
19 AKT2	GCTGACCGCCACTTCCAT	GGACTCACGTGTGTCCCT
19 AKT2	CAGGGAAAATCTCTCCAGCTGTA	GTCATCGAGAGGACCTTCCAC
19 AKT2	ACTGACCTCTCGTCTGGAGAATC	GCAGGCTAGCAGGGCTATC
19 AKT2	CTCGCGAGCGCAATTCC	CAGACCATGAATGACTTCGACTATCT
19 AKT2	GTTCCCTTGCCAAGGAGTTTG	ACAGTGTCTTTTGGTTTTCCTCTTTCTT
19 AKT2	CGAGCGTGCGTCCTCTG	CCAGGCCCTCTCCTGACT
19 AKT2	CGACACACTGCGACCCTAC	GCTTGCTGGGCTGCTTAAGAA
19 AKT2	CAGCCCTCACCTCTGCTT	GACCACGAGCGCCTCTT
19 AKT2	CGGATCTCTTCCATGAGGATGA	CCTCATTTCTCCTCCATCCTCAG
19 AKT2	GAGGCCTCAACCAAGGTCA	GCGATGCCAAGGAGGTCAT
19 AKT2	GCTGAGGAAGAACCTGTGCTC	tgGTTTGTGGTCAAGACAGAATCA
19 AKT2	TGACCCACTCATCCCAAGACA	CTCCCTTCCTGCCTCATTTCAG
19 AURKC	TCCACCTCAGACGGAAATTGTG	CTCTGGCTTAATATCTCTGTGAATCACTT
19 AURKC	CTGACCTACTGCCATGACAAGA	GTACTCAGAACTCACCCTACCAC
19 AURKC	GGCGTCAGGAATTTCAGCCAATA	GCTTCCAATCCTGCTCGTC
19 AURKC	tcctcCTCTCTTTCAGTGGCT	ACACACCCAGTCTGTTCTTCATC
19 AURKC	ACCCCTTTCAGGACCCTGT	CTTCCCTGCGTCCTTTCTCT
19 AURKC	CCTCCCAAGCTGAGGCTTTTT	CTATCTGCGACTTGAAGAGAACCTT
19 AURKC	GCTCAAGGAAAGCCATTTCATTGT	CTGGGCTCAGACGTCAAAGAT
19 AURKC	CTTAACAAGGCTCAAAGAAGAGGGA	TCTCTCCAAGGGCTGGTATCT
19 AURKC	GGGACTTGATTTCCAGGCTTCTC	AGCTCCCTGAACACACACAAA
19 AURKC	GTGTCCTAGGGTCTCCACATC	CCTTTTCATCATATGTTCTCCCCTCAA
19 AURKC	ACTACTTGCCGCCAGAAATGA	TGAATGCCCAAAGGTGACTGT
19 AURKC	ATGATAGGCCTCAGGGAGAAATCT	GCTCACCCCTTGGAGCATAT
19 AURKC	CCGGGTGTACCTGATTCTGGA	CAGCCCACAGTAAACTCAGAGA
19 AXL	CTGTGTGACCATCTCGTGTGATT	TGCCTAGCCGAAGCTGATG
19 AXL	CCCTCACCTCGCAAGCAT	TCCCTCCCCAACCCTTCTTA
19 AXL	ACTCCCACCCAACTATAGGAAATACA	CTACACATGTGCACGTAACTCAC

19 AXL	CCCATGAACCTGGGTATTGGT	ATTTCCCTGGCGCAGATAGTC
19 AXL	GCGTGGAGAACAGCGAGATTTAT	GGGTTTGGAATGAATTCTGGGTTG
19 AXL	GGTTCCTAAGCTAACTCTTCCCATCT	ATCCCGAAGCCAATGTACCTC
19 AXL	GTGTCAGCTCCAGGTTCAGG	ACCACTATCCAGTCATCCTGTTCAT
19 AXL	CTTCGGGATGGACAGATCCT	gctTTCTGCCCTCTGTGTGAC
19 AXL	GCTGAGATGGTGCTTGCTCAA	CCAGTTCACCTCTTTCCACTGT
19 AXL	CCCCAGAGAAGTGTTTGAACCA	GCCAGATTCTCTACAGCCTTTAGAT
19 AXL	CACCCACTTCCTGGGAACAG	CTCTTGCCAATGCACGAAGG
19 AXL	GAACATTAGTGCTACGCGGAATG	CCAAGCCACTCCACTCCAAT
19 AXL	CCCGAGAGTGAAGGCTTAACAG	CACACTGTCAGATTGGACACAGA
19 AXL	CATAGGGCTAAGGCAAGAGGTG	TTGGGCATGGCTTTGGACTTA
19 AXL	CAGGAGTGACAGGGCTACC	tgaatgaTTAAACGCCCCTTACCA
19 AXL	CCTAGTGGTGAGTCAGGCATCT	GTAGGTCCACGGGCTCTG
19 AXL	CAACACCCCCTTCAACCTGA	CTATTCGGAGCTGACCCACAT
19 AXL	CAGGGTCAATCTCTCCCGTT	AAATGAACCAAGTTTGGGAGTCTCA
19 AXL	CTAGGCTTGCACAGAGGGAT	TCCCGGTGTATGAATCTCTTGGTA
19 AXL	CCAGTGGCATGGAGTATCTGAG	TCTAGCCCTGCTTCCATCCTAA
19 AXL	CAGACTGGACACCCTCTTTCC	GGTCTGATGTCCCAGAAACACC
19 AXL	CACGGGACAGTACCAGTGTTT	CTATGAGCGCCACATGTCCT
19 AXL	ctgCTCCATGACTCTGTCCAC	GAAGTTCAGAAGGATGTGTGTATGCT
19 AXL	GCTGGGAGCCCAACAACTT	AGGAGCCCCCATCACTCA
19 AXL	CCTGTTCCTTTCCCCAATCCAAA	AGACTCTCAATGGCAATCCACTTG
19 AXL	CGTATCGCCAAGATGCCAGT	CCAGGCATAGTGTGTGATGGT
19 AXL	AAGGCTTCATCCATGACCTGTT	TCTTTCGCCGGTGGACAAG
19 AXL	GTGTCCTCATCTTGGCTCTCTTC	ATTCTGATTCTTAGTCATCACTCCTCC<br>T
19 AXL	CTCCTCCTCACGACCTTTCTC	GGCTTGTAGACCCCTTGAATCT
19 AXL	CCCCTCATTTTGCTGCCCTA	CCACCCTCATCCATGTTGACA
19 AXL	AGGAGCCTGACGAAATCCTCTA	ACATAGCGTCCAGCAGGATG
19 AXL	GATTCCTGTAGCTGCCTCACT	TTGGATCCTGAGAGGGAGTACC
19 AXL	CCAGTCTTGTCCTCTCTGAGCA	TGCAATGAGTATGCATGGTGACA
19 AXL	TCTGATATTGGACCCTTCCCTCAT	CCACCTCCCAACTCTGTCAC
19 BCL3	GCTGGACCCCGCGAATG	AGCAGCTGCACCATGCT
19 BCL3	GAAAACAACAGCCTTAGCATGGT	AAAGGATCAGAGCTGGACGC
19 BCL3	GGGCCTAGGTTTCACCGA	GCTCCCTCCCAGCTCCTA
19 BCL3	GTGAGAGGTGAACAACCCCTAA	gagCCTCACCTGCCGTA
19 BCL3	CGCTCCTACCTTCCTGTGAC	GGAGTACATTTGCGCGTTCA
19 BCL3	CAACGTGAACGCGCAAATG	CGTGTCGTTGTGGCAGTT
19 BCL3	GCTGACAGCAGCCTCAAG	ACACTCGCCTGCAACCA
19 BCL3	GCTCTATGGTCACGCCCATC	ggaGGTTGGGAACGGTTTCTC
19 BCL3	GGAAGACTGCAGGATGAGGAA	CACCAGCTGTCACCAGGAG
19 BCL3	GTGATCACCACATTACCGTCTGT	GCCTCCAGGTCCAACGTG
19 BCL3	TCCGGCTCCTGGTGACA	GTCATAATTGCGGGCCTCCA

19 BCL3	CGGGCACGTTGGACCTG	AGAGGAGGGCTCGGTGAAA
19 BCL3	TGTCCCTTCTCTGTCCTCCATT	CATGGCGATGTCAGCAGAAAG
19 BCL3	CATGATGTGCCCCATGGAAC	GTGTTATGGACGGCTGGTCTT
19 BCL3	ccTTGTCGTCCCCCTGGA	TGGTGACCCTATGCTCTGTGT
19 BCL3	ccctctGTCTCTCTTCCTTCCT	GGAAGGCGGGTGGAGATG
19 BCL3	CTCCTCCCAATTTCTTCCTTCCTT	CCCCTCCTCATGAGTCCAAGAT
19 CCNE1	TGGACACATTGTGGCATGGAA	TGTATACATTCAGCCAGGACACA
19 CCNE1	GGCGTTTAAGTCCCCTGACTAT	GGACGTGATGGAGCCACTTAC
19 CCNE1	CTGAGTTGCAGGTGAATTTGTAATGT	GGGTAAACCCGGTCATCATCTT
19 CCNE1	TGATCCCCACACCTGACAAAG	GTAAGAGACCTGGAAGCCATGTG
19 CCNE1	GCAGGGAGCGGTGAGTG	CCGCCGTCCTCCTTCAT
19 CCNE1	GGTCCACAGGGATGCGAAG	CTCCCCAACCTGTCTCTTTTGTA
19 CCNE1	AGTCTATAAACTTCACAGGGAGACCT T	TTTACCTCAAGTTTGGCTGCAATAAA T
19 CCNE1	GCGACACAAGAAAATGTTGTAAAAA CTC	GCCAACCTCTTCCCCCAATC
19 CCNE1	GGCATCCATCTCAGCGTTCTT	CATGCACTGGAAAGCCAACTTAC
19 CCNE1	GGCAGGAACGGAGCTCATAA	CGATTTTGGCCATTTCTTCATCTGG
19 CCNE1	GGCTTCATGTTAGTTTTTGCAGGAT	ACAGCATGAAACCCCATGTGA
19 CCNE1	CTGTTAGAGATTGGCTTTGGTGCTA	GGCCGGAAAATTCACTGGAAC
19 CCNE1	CCTCATAGCATGGACGCATTCTTA	GCATTTTTGGCTGCAGAAGAGG
19 CCNE1	GATCAGCACTTTCTTGAGCAACAC	AGGTCCTAATACACAAAAATGCTGGA AA
19 CCNE1	GTAAGTTGGCTTTCCAGTGCAT	CGTCTCCCTTATAACCATGGCAA
19 CCNE1	CTGTGTCAAGTGGATGGTTCCAT	GAATTTGGCCAAGAAGGACTGAAAG
19 CCNE1	CCTATGGTAATTGAAGCCCAAGGA	GAGAGGAGAAGCCCTATTTTGTTCA
19 CCNE1	CGAGCAAAGAAAGCCATGTTGTC	CAACTGTCTTTGGTGGAGAAGGAT
19 CD79A	GCATCCAGGAGGGTCTGAAAGATA	CATCCCCACCCTTCACTCAC
19 CD79A	CTGATGTTCGCTGCCTCATTT	TGGACATCTCCTATGTTGAGGCT
19 CD79A	CTGCTCCATGTATGAGGACATCTC	AGGGAAGTGAGCTGAGACACT
19 CD79A	AATGTGTCACCATCCCCAGTC	GTTGGCGTTGTTGCTGCTATT
19 CD79A	GAAGACGCCCACTTCCAATG	CCATGGCTCTTGTTCACATTCTG
19 CD79A	CCCCAATGGTACGCTGATCAT	GCGGGACAGTGGGAGTAG
19 CD79A	GCTCTGAGCCATACTACCTCCTT	GGCTGACTCAGAGGTCCGA
19 CD79A	GGGACTGCTGCAACTCAAACT	CTGCCCTTTGGCCACATAC
19 CEBPA	gcggcTGGTAAGGGAAGAG	AGGCTGGAGCCCCTGTA
19 CEBPA	CGGCCGGGTCGATGTAG	CCGATGAGCAGCCACCTG
19 CEBPA	GTTGCCCATGGCCTTGAC	AGAAGGTGCTGGAGCTGAC
19 CEBPA	CGCAGGCGGTCATTGTCA	GGCCAAGAAGTCGGTGGA
19 CEBPA	GGCTGCAGGTGCATGGT	CGGCCTCTTCCCTTACCAg
19 CEBPA	AGTGCGCGATCTGGAACTG	CGCTGGTGATCAAGCAGGAG
19 CEBPA	GGAGGCACCGGAATCTCCTA	GCTGCCAGAGAGCTCCTT
19 CIC	GACTCTGTGGACAAGTGAGCAT	CCGAAACTCAGGCAACTCAG

19 CIC	TGGACTTCGAAGAGCGCTTT	TGCCCAATGCTCGCCTAC
19 CIC	ATCTAGCCCCCTCCCCATAC	GATGGGACAGCACCGTGA
19 CIC	ACAGGAACTGACGCAGGTCTA	CATACTGGGTAGAAGGCTTTGGG
19 CIC	GGCCCTGCATCGTACTCTG	TCATCACTCGTCATGTCCTCACT
19 CIC	GAGCTTCTCGTTCTCAGCGT	CTGGGTCCTGGGCAAGATT
19 CIC	ACAGTGAGACAGAGAGTGACCAT	ACTGGGTCCGACGTTTTCC
19 CIC	AGAGACATGGCCCTCACTGT	CTGCGTCCATCCTTCTCAGA
19 CIC	CCAAGGAACGGGACTCATCT	TCTCCCGCTGCATTAAACACA
19 CIC	AGGTGTCGGAGTGTCCTGA	GGAACCATAGCCTTTGCCAAA
19 CIC	CCCCCGGATCCTCCTGTAG	TGGGCCTTGAAGGTTCCTG
19 CIC	GTGCTGCCCCCAAACAAG	TTAGAGGCGATGGGCACAG
19 CIC	CAGCCACCTCCTTCTCACT	GCAGGATCCATTGGGAGGAAC
19 CIC	GTCCAGCGACACCTTCCAA	GTCTGCAGGATGTTTCCTCCTC
19 CIC	GGCCTGTCAGCAGCACTC	GCCGACTTCTTGTCCGAATACAC
19 CIC	GGGAATCACCCAGGTACAGT	ACTTTGCCGTTGGTGGAAGT
19 CIC	GAGACCCGAAAGTGTGGGT	GCCCCATAGGCCAGTGATG
19 CIC	CATCCGTTTCACCCTCCCA	CTATGGCCCCTCCCCTAGT
19 CIC	CCGCTGGGTGTCAGCTTA	GGTGCCCACCAGTAGGT
19 CIC	CGCCTGCCACTGTCACTA	CACTCCCAGCACCTGCTC
19 CIC	CCCCTGCTGTCCAGTTCATTG	CAGCGTGGGCAGGATGT
19 CIC	GGTCCAGGTGGCCTAGGA	CGGGTCCTGGGCTCTCA
19 CIC	CCCTGGAGCCACATGCC	TCAGGGAACTCACGCATCATC
19 CIC	GCCTGGCTCAGCAAACAAT	AGAGGCACCTGCCTCAAAG
19 CIC	GCCATCGCCAGCATTCC	GCCCCTCAAGCTCAGACTCT
19 CIC	CCAGTCCGAGAGCCAACTG	CGCTCACCATGGTCTCAGG
19 CIC	CCCCCACCTGCTGAGGA	ACACAGGCATGGAAAGAAGAGTAG
19 CIC	CTGTCATAGCGCCACTCTCT	CCGCTGTGGGAGAAAGCTC
19 CIC	AGAACGGCTACACACAGTTGG	CCTAGACCTGCGTCAGTTCCT
19 CIC	AGAAGTAGACAGTCAGGCGCTA	AGACACCATCTGCAGGATAGGT
19 CIC	TGCACTGAGTCTGCTTCTGTTT	ACGGTCAAAGGTGAAGATGTCC
19 CIC	CTGCGCCGGACCATGTA	CTTAGCACCCCGACACCTT
19 CIC	CCAAGAGTGCCAAGTGCGA	CCACCCTACCTCTCTCACTCTCTA
19 CIC	CCTATGGGTGCCCTTCTCCA	AGTATGGGCTCAGCTGCAATTAC
19 CIC	GGTAACGCTGTTGCCTCTTG	CTGAGCTGGACTTCTTTCGGT
19 CIC	CAGATTGGAAGTGGTGCAACAA	GCAGCAGTGCCCGTCTC
19 CIC	CGGGAGCGGAGCATGTC	AGGGTCGCCAAATTAAAGCGAT
19 CIC	GCTTACCTCACTCCTCCCCATT	CAGCGAGGTGAAGCCTAGAG
19 CIC	GGCCCACGAGCTCTGTAG	CAAGTTAGGGCCCGGACTG
19 CIC	CCTCTCTGACTCCCCTGTGAAA	CATACTCTAGCTCCCCAAGCAC
19 CIC	GCCCTGCACAGGTACAGAA	CGAGTGACCCCAAGACTCC
19 CIC	GTCCTGTTTGGCTCCCTTGTAA	CCCGCACTGACATGCTAGG
19 CIC	GGGAAGGTCCTAGTGCCTCT	CAACATCCAGCAGGTAGAGAGAA
19 CIC	CACCTGGCTCCTTTCCACA	CTCACCTTTGCTCTGGTTGGA
19 CIC	GTTCCCTTGGCACTCCTTAGT	CTGGACCCCACACCCTT
19 CIC	CCATCTCCCTGCCCATCT	CCACTGCTGCTCTCGCT
19 CIC	CCGCTCTGGGCTGTGTTTAAT	CCAGGATCTTGCTGACGGT

19 CIC	CAGCTGTAGCCCCTGGT	CAGCCCATGCTCACTTGTC
19 CIC	CCTGAAGAAGACCTTTGACTCTGT	ACTTCTGACAGGACCCTGCT
19 CIC	CGTCATCCCAACCAGGACAAC	GCAGGGCAACCCTTCACTC
19 CIC	CCTAACTTGGTCTCCTGCTTCTT	GCTGCCAGAGTAAAATGCTGTT
19 CIC	CCCCGGAGGTCCTGTCAT	AGAATGGTGGCTGCAGGTG
19 CIC	CAAGCCTGGTCTACACTGTGG	ACTCACTCTGCTCTGGGTTGA
19 CIC	CTGTGCTCCCCACCGTTT	GCTGCTCCGTGGGAGTG
19 CIC	CCCCAGGTCCCTCCACA	TTTACCCGGGAGGAGATAACCT
19 CRTC1	CCACGCATCCCGCCTTT	GGGCTCATTTACCAGAGAGACAG
19 CRTC1	TTTCAGCCCAGCGGATTTCT	GGAGCTCAGGGATCCCCA
19 CRTC1	CACTTGGGTCTTTTTGTGCCTTT	GTTCACGTTGGGCAGGGA
19 CRTC1	CAGCCGAGGCCAGTACTATG	GATAACCCCAGCTGTCCTCAAT
19 CRTC1	CACAGCAGGCCTCTCTTCTG	GGTCCCAGCAAGCATCTGT
19 CRTC1	GCCATGACTCCGTGTGTCAG	TCTCTTCCATTCCTGGGACTGTT
19 CRTC1	CCACCTCCCCAGTCTTACTGT	CCAGGTGATGCTTGAGAAGAGAA
19 CRTC1	GGACTATCGCATTGTCTGCCT	CCCGAGTCCCCTGTCCATA
19 CRTC1	TCAGGCTCTCCTGTCTTCTCTTT	CAACCTCAAGAGAGTGGACAGAAT
19 CRTC1	CCTCGCCTGCTGAGCAT	CCGCCTGGGTGAAGAAGG
19 CRTC1	CGCCAGCCTGACTCGTG	ACCCCACCCTGGGCTTA
19 CRTC1	CTCTCTCTGTTTCCATCTCCTCCT	GGATATCTGCCGTCCGTGTT
19 CRTC1	GCCCCTGTCAGTGGACA	AGATGGTGCTGCCCTGATTC
19 CRTC1	CCCTCCCTCCTACTTCCTCTCTA	CCCTCGCACCTGAGTGATG
19 CRTC1	CCTGTCCCCGCTGTCAC	AACTAAAAGTGTTGGGAGCATTCTCA
19 CRTC1	GGGCTGATCAGGCTGCT	CGAGGTGGGAGACTGGTT
19 CRTC1	CTGCAGCAGTACCGCACTA	CATGAACAAGACAAGCGTGGA
19 CRTC1	gTGGCGGCGAGAAGATG	CGTCAGGCTCAGGTCCTT
19 CRTC1	CCTGGTGTGCCTCCCTTC	GGCTGGCGTATCCCAGTT
19 CRTC1	GGGCTCCACACTCAACTACTC	TGGCCTCACCTGTGAGGAT
19 CRTC1	ACAGTGGCATCCCCAACATC	CCATCAAGCTTGGGTCACAAC
19 CRTC1	CCTGCCTCTGGCCTTTTC	GGGAAGGTGGGCTCCTC
19 CRTC1	CGACCTGACCAACATCCACTTC	CGGAGTGTCCCTGCCTTA
19 CRTC1	GCACTGCCCCCTTAACTCAC	TCCTGCTTTTCCACATGTAACCAT
19 CRTC1	AGGGCTAAGCAGTGCCTTTT	GGTCGATCTTGAGTTCGTCCAG
19 CRTC1	CGACTCCGACAGCCAGTTT	CCATCCGGAAGGTGTCCTC
19 CRTC1	CAACGACCCCGACATGGTT	TCACAAGCTCGGCCGTT
19 ERCC1	ATTTCCTCTTGGAAGGGATTCAACA	CCTGCACCCAGACTACATCC
19 ERCC1	CTTACCACATCCACCTGGACAAG	TCTTGAAGAGACTGAGACCTCTCAA
19 ERCC1	AGGCTTCTCATAGAACAGTCCAGAA	GCAATGTGCCCTGGGAATTTG
19 ERCC1	CCCAGCACATAGTCGGGAATTAC	TGTGGTTATCAAGGGTCATCCCTA
19 ERCC1	GGGTCTCAGGTTGTGTTTATTTGG	CCCCTCGCTTTCACCTTTCAG
19 ERCC1	CAGGGACCAATCCCAGGTG	CCTGGCTTCTAGGTTTCTAATTCTGAT T
19 ERCC1	AGGGAGATGGAAGGAAATGGGT	AGTCAACAAAACGGACAGTCAGA

19 ERCC1	CCATTCCTTACTCCAAATGTGGTCA	aGTCTTGCTGGCCTTTCTTCTT
19 ERCC1	CCCCGCATCTCCTTGTCCTA	CCTTCCCTTTCAGGCTCCAG
19 ERCC1	GGAGAACAAAGTGGCTGGAACT	GGGCAAAATCCAACAGCATCA
19 ERCC1	CTGCCGAGGGCTCACAA	CGGCCCCTCAGACCTAC
19 ERCC1	AGAGGCTGTGAGATGGCATATTC	GAACTCACAGGGCCTCAGAT
19 ERCC1	GGCAGGGATGGGAGGTGA	GCTGCCCTGTATCCTGTTATCC
19 ERCC1	GGCCTGGGAGGACGATTTAT	GCTCCTTTAATGACTGGTTCCTCATTT T
19 ERCC2	AGGACTTGTGGTTGGACGAAA	TTCGAAAGTTGCTCAACTTCTATGAG AA
19 ERCC2	ACCTCAGGGTGAATACACAAGTTTTT	GTGCCTCCAATGAGCACAAG
19 ERCC2	ACTCCTCCCTTGATCCTAACACT	CCACCAGCCACACTTGTAAC
19 ERCC2	GAGAAGCTCAGCCTGGGA	CACAGATGCCAACCTCAACCT
19 ERCC2	CAGGAAGTACTTGGCCACCTG	CTCCCCGAATGACCTTCTGT
19 ERCC2	GGGCATCAAATTCCTGGGACAA	GCAGTACCAGCATGACACCAG
19 ERCC2	GTAACCTCATAGAATCGGCAGTGG	ACCCTGTCTGGGTGCTAAGAT
19 ERCC2	CACAGAGCATTCACACCCTCA	GGGTAATCTCACCCCTCCTT
19 ERCC2	GGAGGGAGGTCAGGGACTA	ACTGCCCTCATCTCTCTCCAG
19 ERCC2	GGAGTCACCAGGAACCGTTTA	CCTCTCCCTTTCCTCTGTTCTCT
19 ERCC2	CCCCTTCCTTTGTCTGCCTTTA	GGTTCTGAGACCCTGTGTGTTG
19 ERCC2	gatacaCCTCCCCTCTTGGAAC	GCTTATCGTGACCTCTGTTGCT
19 ERCC2	CACAGGAAACCTGTCACCATCA	ATGGCATCGTGGCCTTCTT
19 ERCC2	TCTCCATGTACTGGTAGCTGGT	CCAGAAACTCCTAGTTCTAAGACAGA GA
19 ERCC2	CTCTGCCCATCCCACCAG	CTGACCCCTGGCACTCTT
19 ERCC2	GCTCACCCTGCAGCACT	CAGCCCCAGCTCATCTCT
19 ERCC2	GGACCAATAGGGCCTAGGGAA	GGGACTGAGTCCGCTTGTTATC
19 ERCC2	GGAGTCGATGCAGACGTTGT	CCTGGTGTCCAAGGAACTGG
19 ERCC2	GTGGGCCTCGTCGAAGAC	GCCCTGGTAACCCTGCTC
19 ERCC2	CACCGATGACCCCATCTTCAA	CCGGCCTCTCGCTGAATAT
19 ERCC2	CTCTGCATAACCGGGACCT	GCTGCTGGAGTACGTGAAGTG
19 ERCC2	CTCTCCTGCACCACATGCT	CGCCGCTGATAGCGTCT
19 ERCC2	GGAAACAGCCTGGTTCTTGGA	AATGACTTTCTTACCTTCGATGCCAT
19 ERCC2	GAGGCCGTAGTCCGTCTTG	CCCAGCTTCTCATCCTCCGTA
19 ERCC2	TCCAGAGAGCTCTGGGAAGAC	GTGGCTCTGAGGAGTGACCTA
19 ERCC2	cctgCCAACAACCCTCTAGAC	AGATCACCGACCTTGCTGAC
19 ERCC2	GGGTGGCAAAGTTAGCAAGGA	CCTGCTCATGCTTCCGTCTG
19 ERCC2	CCAGGGACCTCGGACTTCTA	ACATCTACCCCGAGCAGTTCT
19 ERCC2	CGTTTGAGCTCCCGCATGTA	CTTCTCTGGGCTCGACGAC
19 ERCC2	GCCCTAGCCTCTCCCACT	TGCCCAGTCCTGCTGAGA
19 ERCC2	GCTGCTCGTCTGTCTCTTTG	ACCTGGAGACCCTGCAGAA
19 ERCC2	CCACCTGAGCACCGTCT	GCCAGCCCCTCTGAGTGA
19 ERCC2	CGAGACGCAAGTTAGGTCACT	AGAACATCCAGAGGAACAAGCTG
19 ERCC2	CACCATCCTGGGTCTCAATAAAGAG	CCCAGAAGAGTTGGATGTAACCAG
19 FZR1	GTGCTTCCCTCCTGTCCAC	GCGAGTGGCGCTGATACC
19 FZR1	AGCTGTACCGGGAGCGT	ACAGGGAGTAGGGAGACACATC
19 FZR1	TGCAAGAGGTGACCCAAGTG	CGACCAGTCCACCAGATTGAG

19 FZR1	GAGCTGCAGGACGACTTCTAC	GTACATTGAGGTCTGCTGGGA
19 FZR1	CGCCTCACTGTGCTTGGT	CCGCAGGCTTACCGTGAA
19 FZR1	CCTGTGAGGACCGGCCTA	CCAGGTGCTCCGTGTACTG
19 FZR1	TCTGGAATCACTCGAGCCTGA	GGTTGTCCTGTCAGCGTGTT
19 FZR1	GACCGCTGTATCCGCTTCT	CACATCCTGAGGCCTGGT
19 FZR1	GCTCTAGGGCCGGGAACA	GATGGGCTGGGACCCTAAC
19 FZR1	GGCAAAGCTCACACTGACC	ACTGTCAAGAGCCCCATCAGA
19 FZR1	GGTCCCATGGCTGGTGA	GGATGTCCCTCTGCAGGAT
19 FZR1	TGGAATGCTGAGCAGCTGTC	GAGGAGCTGGTGGTCTGT
19 FZR1	AGGTGTGCGGGCTCAAG	CTCTTCCGTTCCTGCCAGT
19 FZR1	GCTCAGCGCATCTGCCAT	GGAAGGCAGAGTCCATGCAA
19 FZR1	CCTCGTTGCCCCTCACC	AGTGCCTGGCGTGAACTC
19 FZR1	CTCTCGGTGCTGAGAGCAA	CCTCACGTTAATCCTGTGGAAGT
19 FZR1	GAGCCAACTGGAGCGTGA	CGAGGAGCCCGCTTGTT
19 FZR1	CTCTGCCCATGCCTTCCA	GCCTCAGGGAGCTCACCA
19 FZR1	CTGCCCCACTCACCTCTC	GGCATCGTGTTCTCATTCTGGAT
19 FZR1	CCTGCTTCGCCAGATCGT	GGACACCTGAGGCTGCT
19 GNA11	CCTCTGATTCCCTCTGCCTTC	ACCAGGTGCGAGTACAGGAT
19 GNA11	CCTCAACAAGAAGGACCTGCT	TGTATGAGCCCCTCTCCCATATC
19 GNA11	CCCCCTCCTGGTGGCTTT	GGCAGGTAGCCCAAGGTG
19 GNA11	CCGACGTTGACCGCATC	gggcAAATGAGCCTCTCAGT
19 GNA11	CGTCCTGGGATTGCAGATTGG	TCACGTTCTCAAAGCAGTGGAT
19 GNA11	GTCGGAGCGGAGGAAGTG	CCCAGGCTTGGCAGGTG
19 GNA11	CGAGCTCTCGACGTCTCC	GCCTGCGGTCGTAGCATT
19 GNA11	CAGTACGTCAGTGCCATCAAGA	CCTTCCCGAAGGGTCCCA
19 GNA11	CGGCAGGGTCTGGGTAAGA	CGCTTGTCCTCCTCCGAGTA
19 GNA11	AGAGCACGTTCATCAAGCAGAT	GCTCACCTTGTTCTGCTCGTA
19 GNA11	CGCCATGCAGGCCATGAT	CACCATGCTCACCACCAAG
19 GNA11	GGCCCACGAGTCCCTTG	CGGTGGCACACGTGAAGT
19 GNA11	GACAGCGACAAGATCATCTACTCA	CGTGTCTCCATCCCGTCT
19 JAK3	CTCATTTCCCAGGGCCTCTTA	CTCCCTCATCCTCTCCCCATAG
19 JAK3	ccaccCTAGCAGTAGACCGA	TCTCTGACTCCTCCCCATTCC
19 JAK3	GGGATAGCGGGACTGATGTC	GCTCTATGCCTGCCAAGACC
19 JAK3	TTGAGGTGTCTCTCCTCGAAGAT	GGATGAGAGGCGCTGCTTAC
19 JAK3	CTGGCAGGAGGGTAAGAATGTG	GGGACTTTCAGCGGGAGATT
19 JAK3	TGTGCAGTGCTTTGAGGATCT	GACATCAGTCCCGCTATCCC
19 JAK3	CCTCCTGCGAGGGACAAG	TGGTCACTGTTACCAGGACAGA
19 JAK3	CTGCACCCACTAAAATCTGGTTG	CTTCGGTACTCCCCCTCCTT
19 JAK3	CCACTCCTCAGCCTTCACC	TGCTTGCGCGACTTCCT
19 JAK3	AGATCTGCGAGGAATAGAGAAGGA	TGACAGATCCTGCCTTCTCCA
19 JAK3	CACTGGGCCCAATATGACATCA	GCCTATGAGCCGGTCCAGA
19 JAK3	GAATGACGGCTCGGAAGGA	GGATTACCGACTGCTCCTCTCA
19 JAK3	CCTGGAAGGTGAGGACACTGA	GCACTTCTTCTGCAAGGAGGT
19 JAK3	GGCCACTTCCTCCAGCAG	CTGGGCTCTGACGCTTGT
19 JAK3	ACCTCGAAATGCAAGGAGATGAT	GTACCACTCCCTCTTTGCTCTG
19 JAK3	CATCCTCCACGGAGAAGATGT	tgatgCTGGCACTCCTGAAG
19 JAK3	GGGTTCCCCACCAGAAAATG	ACATCCTCGTGGAGAGCGA
19 JAK3	GCCGAAGTCAGCGATCTTGA	GGGTACCTGCCGGATTATCCT

19 JAK3	GTCTGGTCCACATTGCTCTCA	GCTGTCCCCTCACCATTCAG
19 JAK3	CCAGGTGACCCCATGCTAAA	CGTCCTGTACGAGCTCTTCAC
19 JAK3	ACAGACTGGGAAACTGAGGCTA	CCACATGCGCTCCTCCTT
19 JAK3	CTGCAGCTTTTGTCGCAGTAG	TCTACACCCTCGCATCTCAAGA
19 JAK3	GCCCCACATCCCCTACCA	CCCTTGGTGGCCACGAC
19 JAK3	CCTCCTGTTCTCCCTGGGT	GACCGGCACTCGCTCAT
19 JAK3	GCTCCAGGTCCATGATGTACT	CAGCTACAAGGCCTGCCTAC
19 JAK3	GAATACGCCTCCGCGTCA	GGCTAAAGCCTGGGTTTGTGT
19 JAK3	TCCTCCACCTCCAGGAACTT	CTGTGTCTGGCCCCCTTAG
19 JAK3	CGTTGCTCACTCCCAAGCA	CGTGGGCCTCAGTCTCAAG
19 JAK3	CTGAGACACTCACCCTGCTC	AGGTTAACAACAGGGCTTGAAGTT
19 JAK3	GTTCCTGAAGTAGAGGACCCTCat	ACTCATGCCTTCACTGCTCAC
19 JAK3	GATCCACTAGGGATGCACTCA	TCCCTCAGCGTTCATGCAG
19 JAK3	CAGCCTCCGTGGACAAGAG	CTCCCTGCTCAGAAGTCCAAT
19 JAK3	TGGATGTCAGTCTGCCCTTCT	ACTTAGCTTGGAAGCTGACAAGTG
19 JAK3	TGGTGACGCCACTAAACACTTC	AGTGGATCCCTGATCCCACTT
19 JAK3	TTGCATGCCAGTCCTCATGT	AATTGGTTTGGGCTGGAGAAGT
19 JAK3	AAATCCTTGCGTAGCCCGAA	GCCCCCACCATAATGTCACT
19 JAK3	GGAAGAACAGCCTAGACTTGGG	ATCTTCTCTCCCTTCCCACCTT
19 JAK3	CAATTCCTCTTCCACCCAGAGAA	CATAGACATGTATCTGCGAAAACGTG
19 JAK3	TGTTTGACCACCTGCAGCTT	TGTTGGCAGAACCTCCTCAAC
19 JAK3	GAAAAGGACAGGGAGTGGTGTT	TTCACGAGCTCATGAAGCTGT
19 JAK3	GAATGATGGCCGGTCCTGT	caaaTACGCTGAATGGGAGTTGTG
19 JAK3	GTCCCAGATCAGCCACTCATT	CCCACAGCAGTCTTCGAGAG
19 JAK3	CATCCCAGCAGGTTGCCA	TGTCGCTCAGTCCCACTCA
19 KEAP1	TGTGGTTACCCCAGCATGAG	AACGAGTGGCGAATGATCACA
19 KEAP1	CCGCTTCGGATGGTGTTCATT	TCCTCAATCGTCTCCTTTATGCC
19 KEAP1	GCGGTTTGTCCCGTCAAAG	TGGCCATTGTCCCCATTTTTCTTA
19 KEAP1	CATTGGACTGTATTTTTGCCCAAGA	AGTGTTACGACCCAGATACAGACA
19 KEAP1	CGATGTCATTCGGGTCACCT	TCACAGCTGCATCTCTCTCTTTC
19 KEAP1	GCAGTCCACAAAAGATGGGCTA	GAGCGCTACGATGTGGAAAC
19 KEAP1	GCTACGAAAGTCCACGTCTCT	CTTCTCTGCATGGTGCCCTTTA
19 KEAP1	CCGGATCTCAGTGTCTTGGGA	TGAGCGTGCCCCGTAAC
19 KEAP1	GCATAGATGTGGCCATCGATGA	GTGGGCGGGCTGTTGTA
19 KEAP1	GTCGGGCGAGTTGTTCCT	GCTACTTCCGACAGTCGCT
19 KEAP1	GGGTTGTAAGCCTCCAGGTA	ACTCCCGCTGCAAGGAC
19 KEAP1	TCCTCGAAGATCTTGACCAGGTA	CATCAACTGGGTCAAGTACGACT
19 KEAP1	GACGTAGAACCGTCGCTGTT	GGAGTTCTTCAACCTGTCCCA
19 KEAP1	GCTGATGAGGGTCACCAGTTG	CGGTCCTGCTTGGTGAGG
19 KEAP1	GCCGCAGCTCGTTCATGAT	GCAGTCACAGTGCCCTGAG
19 KEAP1	CCTGGTCCTTCTCCTGACACT	CATCGGCATCGCCAACTTC
19 KEAP1	CTCAGTGGAGGCGTACATCAC	CAGAGGTGGTGGTGTTGCTTA
19 KEAP1	GCAACTCCACACAGCCAATCT	CCATGGGCGAGAAGTGTGTC
19 KEAP1	CACCGTTCATGACGTGGAG	CTCATCCAGCCCTGTCTTCAA
19 KEAP1	GCAGCCCGTTGGTGAACAT	TCATACCAAGCAGGCCTTTGG
19 MAP2K2	CCGGAAGGAGTGGCACATC	ACCCTCTGTTCTCCTCCACA

19	MAP2K2	CCCGGCCACTGTCACAC	GCTCACGGCTCCCCTTTC
19	MAP2K2	CTTCTCCCCAACATGCTCTGTT	GTCCTGCACGAATGCAACTC
19	MAP2K2	CCGTAGAAGCCCACGATGTA	CCCAAATGCCTTCATCCCGTT
19	MAP2K2	GGACAGAGCCTGGAGCTAAT	GTCACCAAAGTCCAGCACAGA
19	MAP2K2	CGCAGTGCTCACCTTCCT	GGCTGGAAGCCTTTCTCACC
19	MAP2K2	CTTTGAGTTCGCCGACCTTG	CCAGATGGAGTCTCCCTAGGTA
19	MAP2K2	GCGTCCAGACCGGAAGTT	CGTCGGAAGGTACCCCATC
19	MAP2K2	AAGATGGCCTCCAGCTCTTTG	TCTGGGCTCTTTCCTCCCT
19	MAP2K2	AGGCCCAGCAGCCTTATTT	GGTCCTGCCTCTTGGAACC
19	MAP2K2	AAAAGGCATCAAGCACAAACCTC	GGCCATCGGCCCATCTC
19	MAP2K2	TGACCCCTGCCCACTCA	CCTATGGGCCCCGGCTA
19	MAP2K2	TGTTGGAGGGCTTCACATCTG	TGGCGTACCTCCGAGAGAA
19	MAP2K2	GCCTTACCTCGGTGCATGAT	CTCTGACTGCTCAGCTCTGAC
19	MAP2K2	GAGAGCTGGCTGGCAGA	CCATGGCCAACTCCTTCGT
19	MAP2K2	CGAAGGAGTTGGCCATGGA	CGGCAGGCAACTGTTGG
19	MAP2K2	TGGAAGAGGTCCGTGCAGA	TCACTCCTTGTGTGCCCTCTA
19	MARK4	GACTGAGAAGACGCTGTACCTG	CGACACGAGGTAGTCAAACACTT
19	MARK4	GCAGGAGAAGTGTTTGACTACCT	CCATGCCCAGCTCTCAGAC
19	MARK4	CGTGGTGATGCCAGCTCTA	AGCCCTCGACTCCCACT
19	MARK4	CCTCCAGCCCCATGGTC	GCAGGGCCTGAGGAATGT
19	MARK4	CCTCACTCTCCTCTGTCTTCCTT	tttgCAAGTGTTCCAGTTGGTG
19	MARK4	CACGGAAGGTGGGATTGGAT	GACAGGAGGCGATGGAGTTC
19	MARK4	CTCACTGGGTGCCCGTT	CCACCCTGTGCCCATACT
19	MARK4	CCCACGTCCATGAAAGGCTTT	CAGAACGTGTCCAGCTTCGAT
19	MARK4	CTTTGGCTTCAGCAACGAGTT	GCTGACGAGGGTGTACAGG
19	MARK4	TCTGGAGCCTGGGAGTCAT	CCAGTCCCACCACTGAGG
19	MARK4	GGGTGTTATGGTTGGCACATT	AGCACTTGCGTACTCCATCAC
19	MARK4	CTGGTGATGGAGTACGCAAGT	GAGCTTCCTTCTCCTTCATGC
19	MARK4	TGGTTTCACAGTCCACTGAGG	ACCTTCAGGTCCCTGTGTACAA
19	MARK4	CTGTGCACTATTGTCACCAGAAAAAT A	GGAGAAAAGGGCTGGAGGAATTT
19	MARK4	CCACATGAGCCCCTGACTTATTC	GTTCTGTGCAAACGTAGGTGTT
19	MARK4	CTAGCATGATGACCCGCAGA	AAGGGAGGTCCTGTTCACCTA
19	MARK4	GGTGGGTTCCCTATGTCCAGAT	GTAAGTGTCGGGAGGTGCTG
19	MARK4	AGACCCCCTCCTCCAGTAAC	TCACCTCGAGAGTACAGCGTTTA
19	MARK4	GGAGATTTTTGGTGCTGAACCCA	AGGAGAGGAAAAGTGTGTCAAGAAG
19	MARK4	CCACCCTGACTTGTCTGTCT	GACCTGGCCACCATGGAA
19	MARK4	TTCCACCATCCGCAGCA	GGTCAGCTTGGTGAAGAGGTT
19	MARK4	CCCACACTGGCCCATGAG	GAGCAGAGTGGAAGACAGGTT
19	MARK4	gaatGTGTCCCGAATTGGGAAGA	GGTTTCCGTTTGATCCCAAGG
19	MARK4	CTCGCCTCAGTTGCCATCTA	CAGGAACGGCTGTGGCT
19	MARK4	CCCTCCTGAGGCCCTGAT	GCTGGCAGACCTCCACTTC
19	MARK4	GAGCCCCTGTCCCACTT	GGACCGTGGTGGCTCAG
19	MARK4	CACCCGCATCTCCAACGA	GGACAATCTTTGCCCCCAAAT
19	MARK4	GGTTCCTTCTGACACCTGTCTT	cagGATGGCTCTCCCTGAC
19	MARK4	GGGTGCAGAAGAGCCTCA	GGCGGTGGTAGGTGGAA
19	MARK4	TCTGTCTCCTGTACCCCAACA	GCGGTTAGAGTTCTGCCGTT
19	MARK4	GGGTTACCCTCGATCCCTCTA	ACACACAGGTCAGACATGCAG

19 MARK4	GGGCTTGTGGCACCTTGA	AGGAAGGGTCAGGTGGGT
19 MARK4	GATGGAAGTTAAAGTTTCTGGGAGTCT	TTCGTTGTACTTCTGGCTGGTC
19 MARK4	CACGGGAAGAAATCAAAGAGTCCTT	CGAGAGGATGACAGATGAGGCT
19 PIK3R2	CTGAGGTTCAGCCAGCAAAGA	TGTCCTTTCAATGGCCTCCAC
19 PIK3R2	GCTCCCCCTCTTCTGGTGAA	CTGTTTCCGTGCAGCatgag
19 PIK3R2	CCAGCCCTGCTTCAACCAA	CAGCAGCTCCAGGTCCTC
19 PIK3R2	CCAGTACCGCGCTCTGT	GCATCCAGCCCACGCTC
19 PIK3R2	AGGCTATGCATCTCCCCTCAT	TCCGTGTGTACTCTTCATAAAGCTG
19 PIK3R2	ACAAGAGCCGCGAGTATGAC	GCAACTGGATATTCATCCTCAAGTCC
19 PIK3R2	GGTGCTCAGGATGCATTTGTTT	CAGCAACCTACCTTGAAATGTCC
19 PIK3R2	GCAGGATGCTGAGTGGTACTG	CCCTGTGGGAACATCCAACAT
19 PIK3R2	GTGCCTTTACCCCTCACGA	CCTTGCTGCATTTCTCTTGAGTCT
19 PIK3R2	GGCCTTCAATGAGACTATCAAGATCTTT	tgtactGCACCAGATCAGGGT
19 PIK3R2	GGCAGCATTTGAGAGCTGACT	ACACGTCCAACTCATGTGTACTG
19 PIK3R2	GCACCGCGTACAGGTGAA	AGGAAGCTCTTAATGCCGTCAG
19 PIK3R2	GACGTGGATCAGTGGGACA	CCCTCCCACCCGCTACA
19 PIK3R2	ccgagatAAAACCCTGAGGTCTCT	TGTCGATCTCTCTGTTGTCCGA
19 PIK3R2	CCGCATTGCCGAGATCCA	GAAGTATGGAGCCGCCACTT
19 PIK3R2	CCCTCACCACACTCCCCTT	CGGGAAGAAATGACAGCAATACAG
19 PIK3R2	CAGGGATTGAGGGTCAGGTG	CGTGGCGGTAGTGATTGATGAG
19 PIK3R2	TCACCTTCTGCTCCGTTGTG	CAGCCAGGTGCTACCCT
19 PIK3R2	GCGACGTGCTGGTAGTGAG	ccccAGGAACTCCACATAGGT
19 PIK3R2	CGGCAGCGAGGTGACTT	CCCTGCTTGCTGCTCAC
19 PIK3R2	GGCTGGCAGGTGAGTGAC	GCTTGCCACTCAGCATCTC
19 PIK3R2	TGGTACGTGGGCAAGATCAAC	GCATTCAATGAGAGCTCTGGGA
19 PIK3R2	CGACTCCCCCTCTCGTCT	GTGAGCGCGTCGTTGTG
19 PIK3R2	GGCAGTCCCAATGTTGGATGTT	GTGACCCCCGGTTATCCTTG
19 PPP2R1A	CTCCCTGTCTCCTTTCGCTTT	GCTCCGTGCTTTCTAGGTCTT
19 PPP2R1A	CCAGGAGCTTTGCATACTCACC	GCCCTATCTTCTGCAGAGACTT
19 PPP2R1A	TTGCCAATGTCCGCTTCAATG	GGTCAAAGATTAAGGTGCCCACAA
19 PPP2R1A	GGGTCAGAGTTGAGATGGGATA	GAGGACTTCCTGCCTTCCAC
19 PPP2R1A	CACTGTTCCTCTCCTCTCCCTA	TGAACTTGTCAGCCACCATGT
19 PPP2R1A	GCCGCTGAAGACAAGTCCTG	CCAATGGTTCCATCGGCCTAA
19 PPP2R1A	TGGGTGTAGGTTCCATGGGAT	CTGCTCCTGTGAAGGTACTCA
19 PPP2R1A	GGGAGCTGTCCAGTGACTTTG	GCGAGTGCTCGTGTGAGATG
19 PPP2R1A	GACAAGGCAGTGGAGTCCTTA	CCTTCACAGCACTGGACACTC
19 PPP2R1A	GGCCTCTTCTCCGTCTGCTA	CCCAGCTAGACCAAAAGGAGTC
19 PPP2R1A	CTGGAGGTGGAACTAGCACA	GTTCCTCCCATCAAACTCTCTGTTAC
19 PPP2R1A	GGAGGTACCTTGGCATTGTGG	TGTCTCCCCAAAGAGGGACTT
19 PPP2R1A	CCCCTCCTCTTCTTGTTCTCTCA	AAAACCACCTCCCAAGAGTCC
19 PPP2R1A	CCCTCGGGAGATGTCCATAAAAG	CGGATGCCAATCACCTCGTT
19 PPP2R1A	ACATCATCTCTAACCTGGACTGTGT	CTCCTCACTCACCAGCTGTC
19 PPP2R1A	CATTGAGTACATGCCCCTCCTG	CAGTTGTCACAGCCTCCACTA

19 PPP2R1A	ccctAATTCTGGTGCCTTCACTTT	TCACAGTCTTTCATCAGgttctgg
19 PPP2R1A	GGGCCTGAGATCACcaagac	GATGTGCTAGTTCCACCTCCA
19 PPP2R1A	CCACATCAGTTCTTCACCTCCAAAT	GCCCACTCCTTCCCAAACTT
19 PPP2R1A	GCAACCTGAAGAAGCTAGTGGAA	CTAGGGATGGGCCAGCAG
19 PPP2R1A	GCCGCAGTCTGACAGGAAA	CCAAGTCCCCCGTAGCCT
19 PPP2R1A	gCCTGCTGCCTCAGGAT	ACTCTTGACGTTGTCCAGCTC
19 PPP2R1A	GGGAGTTTGCCAAGGTGCT	CCTTGCTGAGCTCTGGGATTC
19 PPP2R1A	TTATTGTCTCTTAGGAGTTGGCATCTG	TTGTCTTTGCCCAAGATGGGA
19 PPP2R1A	GCCTCAGTCATCATGGGTCTCT	CAAACCCAGAGCTGAGATCCT
19 SMARCA4	CCAGAGCTCAAGGCTGTCTTTC	TCCTCACTGTCATCGTCACTCA
19 SMARCA4	AAGATCAAGCTTGGCCGGAA	CGGCCTCACCTCCTCTTG
19 SMARCA4	GTTTGAATTCCCGGCAGGTTT	GAAAGCCAGGAAATGCACTCTTAC
19 SMARCA4	GGCTTGTCCTCTTCCCTCCTA	CTCCTGCTCGATCTTCTGCTG
19 SMARCA4	GCATCACTGAGAAGCTGGAGAA	GCAGCATGTTTAGGAACAAAGCA
19 SMARCA4	GCCACCTCACGTTCCACAT	GCATCCCCATTCCTTTCATCTG
19 SMARCA4	CATGCATGAGAAGGGCATGTC	GGACTCTGGAGTATGACAAACACA
19 SMARCA4	CAGAAGCAGGTGTTCCTTGGT	TGAGCAGGAAGATGAAGTACTCAGA
19 SMARCA4	GCTGCTGAAAACCTTCAACGA	CCTTCCTTCTCCTCGACCTG
19 SMARCA4	GGCTATGGGTTTGCACAGTGA	CGTAGCAAGAGAGGAAACCTCAG
19 SMARCA4	TGGTGCCTGCATGCTGAT	TCCGCGTCGTCCTTGATG
19 SMARCA4	CGAGCTCCCCTCGTGGAT	TGGGCCTCTCCAGCATGTA
19 SMARCA4	GGTAACGCTTGCTTCTCCTGT	GCTCCCCTCAAAACTCCATCA
19 SMARCA4	GGGTGCTGGCTGTCCTAT	CGTCGCTGTCTCGCTTG
19 SMARCA4	GGCAGAAGAAATCATCACGGAAG	TGGTGAGGTTGGGTGGGTTA
19 SMARCA4	CGCCTGCCGAGAAACTC	GTGCAGAGTCAGAGTGCAGAT
19 SMARCA4	GAACTGAGGTGACATGGGCTT	CTGTGTCGGGACCCCTTAC
19 SMARCA4	GGACTGACTGGCACCTCTT	GCCTGGATCACCTTCTGGTC
19 SMARCA4	GCAGCCAAGTACAAGCTCAAC	GCGTCCACCATTCACGCT
19 SMARCA4	CTGAAGGGTCAGCCTTCTCTT	CCTTGGTCAGCTTCTGGATTTTG
19 SMARCA4	GGAATATCACAGATCCGTCACAGG	TGTAGAAGAGCGCACGTCAAG
19 SMARCA4	CGGGCCCATCCACTCAA	ACATTCGGATTCGGAGCcttc
19 SMARCA4	GTGCGGCAGAAAATCgagaa	GGAGGGCTGTGAAAAGCTG
19 SMARCA4	CATGTTTGCCTGCCATTTTCTGT	CTCATCTGGCTGGTCTCGTC
19 SMARCA4	ACGAATGACTCTTTTTCAGCCTCT	GGAATGCAAGGCCCAACTCA
19 SMARCA4	TGGCCCCTTGCCTTCTC	GGGTCTGCGGTGGCATC
19 SMARCA4	GCACCCCTCAGAAGCTGATT	GGTGATGCGGCTCTGCTT
19 SMARCA4	CGTGATGCCACCGCAGA	CCCGCCCTCACCTGTACT
19 SMARCA4	GCCTCGACCCTGTGGAGAT	GCACAGGGCACTGTTTGCTA
19 SMARCA4	GTGATAGCCGCCGGTTCT	GCAGGTACAACGTCAGGGTTAC
19 SMARCA4	CCATTTGGGTCCCTCTCATCT	GCCTAGGAGCTCAGCAGACA
19 SMARCA4	CATAAACCTGGGACGCACTGT	CCACCATAGAAGAGGGAGCTCA
19 SMARCA4	GCCCTCGTGAGCATTATGTGT	CTTGTCCTTCTCGGAGCCATC
19 SMARCA4	GAGTGCTCTACCGCCACAT	AGATCATGACCCGATGGTCAGA

19	SMARCA4	CGGCACTGACAGTTTGCAAT	GCAGAGTGGGATGCCTGAAC
19	SMARCA4	GCCTGACCGTGTCTCTCTCTA	CCTTAGGGCTAGCTTGATACTTCCA
19	SMARCA4	GCGGTGTCTTGACTCTCTGAAG	ATGCCGTTCAGGTTGTTGTTG
19	SMARCA4	AGTGGCTGGTGTCCCTGTA	TGGCAGCGGGTACTCAC
19	SMARCA4	GGTCCTGAGGTAAGACCTGCT	GTTGAAGGTCTGTGCGTTCTG
19	SMARCA4	GAAGGACGTCATGCTCCTGT	cacAGCTAGCGGGACTTGTT
19	SMARCA4	GTGCCACCACATTGCAGTAAC	GAGGTTAGCCACGTACTCGTC
19	SMARCA4	CCTACCTCTTGCAGCAGACA	ACGTGAAGACGGCTGCTC
19	SMARCA4	GCCAGTGGCCCGTCTTC	CCCTGGCCAGCATCTTGTA
19	SMARCA4	AGCCAGCACATTGTCACAGATA	TCCACTCTCTCAGTGACAGCA
19	SMARCA4	GTGGCCTGCAGTCCTACTATG	cacCACCTGGGAACACCTG
19	SMARCA4	GGGAAGAGGCTGTAAAAATCACA	CTGGCCCGGAAGACATCT
19	SMARCA4	ACCAGCTCAGAGCTCAGATCAT	CCTGTTGCGGACACCGA
19	SMARCA4	GCAGATGCCAACGCTACCT	CAGCCATTCGCTCTGAGACA
19	SMARCA4	CAAAAGCCGAGCTGTGCATC	ATTTCCCACAGGGACACGTT
19	SMARCA4	GCCCCCTGCCCTGATTG	CTTGTTCTGCAGCGGTGTG
19	SMARCA4	GCTCAACACGCACTATGTGG	CGTTAAACCACTGCTCGAAGGT
19	SMARCA4	GCTGCCCACCATCTTCAAGAG	AGCTGGTGCTCAACACGTT
19	SMARCA4	CCAACGCACACTCTCTCCTC	GCTAAGGCCTATGCCATCTCA
19	SMARCA4	GTCTGCAGCTCCCGTGAA	GGGCCCCATCATGCTGT
19	SMARCA4	CCTTCCCCTGGAGCCAT	GGATCCCTACCTTGTGCATCTG
19	SMARCA4	GGGTACCCTCAGGACAACAT	ACACCTCCAACAAGAAGGCTT
19	SMARCA4	GGCATCTCTTGCCTCATGACTA	ACCCGGGAAGGTTTTCAAGTTC
19	SMARCA4	GCATCGCACACCGAATTCAG	CAGCAGTGTTGGTGACAAAGG
19	SMARCA4	GCTTTGTCCTCTGAGCACGA	CCATCCAGTTGGGAAGCTTCT
19	SMARCA4	CCTGCAGGGTTCCAGGTTTAA	GCTCGGAGTTTGGGAAGAATTCTATC
19	SMARCA4	GCCTCGGGTAAATTTGAGCTTCTT	GCCTCACTGGCTCATACTCAC
19	STK11	CTGTGGTGTTTGGGAGGCT	GCCCTGGCTATGCAGGTA
19	STK11	GCAGGTACTTCTGTCAGCTGATT	CCCCTAGCACGTGCCTAC
19	STK11	CGAGGTAGGCACGTGCTA	GAAGGTGTCCAGGCCGTT
19	STK11	GACACCTTCTCCGGCTTCAA	TGTGGCCAGAGAGGGTCT
19	STK11	TCTTCCCTCCCCTCGAAATGA	CACCTTTCAGCAGGTCAGAGA
19	STK11	TGTTTGAGAACATCGGGAAGGG	CCAACCCTACATTTCTGCACAAAA
19	STK11	CAGGCCATCATCCTGACGTT	CCGGAAAGGATATATTTCTGCTTCTC<br>T
19	STK11	GCTGGTGGATGTGTTATACAACGA	CGTGTGCCAGTCTCCTTCAAG
19	STK11	CTGGGCAGCAGCTGTAAGT	TCATACACACGGCCTTGGG
19	STK11	GGAGGCCAGTCACAATGGA	GACAGCCGGCGGATCTT
19	STK11	CGCAGCTGAGCACCAAATC	CAGGCGTTGTCCCCACAT
19	STK11	CCCTTAGGAGCGTCCAGGTAT	CTCTTGGCCGGTTCGTACT
19	STK11	GCTTCTCCTCAGGGATGCTT	TCTAGCGCCCGCTCAAC
19	STK11	TCCCTCCAGAGCCCCTTTT	GCACACTGGGAAACGCTTCT
19	STK11	AGGAAATGCTGGACAGCGT	aaaaacTTGGGCCTTCATGTCAATG
19	STK11	AAATTTTGGAGAAGGGAAGTCGGAA	GGTGGATGAACGTGTCCATACC
19	STK11	GGAGGGCGAGCTGATGTC	GAGTCCAGCACCTCCTTCAC
19	STK11	GGGAAGGCTCTTACGGCAA	ACCCCAGCAAGCCATACTTAC

19 STK11	AGGAGCTGGGTCGGAAAAC	CAAGTACGGCACCACAGTCAT
19 STK11	GAAGAAACATCCTCCGGCTGAA	ACCGTGAAGTCCTGAGTGTAGA
19 STK11	CTTCGACATCGAGGATGACATCA	AAGCTGTCCTTGTTGCAGACA
19 TCF3	GGGCCTTCAGCTCCTTCT	CCTGACCCTAGGCCTGTTG
19 TCF3	CCCACGTCTGCACATCTGT	TCTCATCCCCACCCCATCTC
19 TCF3	CCATGTGTGTTCCCAAGCTTC	GTTCTCACCCCTTTTGCTTCTAAC
19 TCF3	CACCACTAGAGTGCCTCAGTTT	CACCAGCCTCATGCACAAC
19 TCF3	GCTCAAGGGCTTACCACTGTA	GGCTCCCCCTGATGCAC
19 TCF3	GAGGCTCCATCGCTGAAGTA	CACAGACAAGGAGCTCAGTGAC
19 TCF3	CACCATGCTGAAGTCCAGGAG	AACTCTTCTCCCCTCCCTGTT
19 TCF3	AGAGAGGGTGGGTGACAGATT	CACTAACCTTCCTTCTCCCCTTG
19 TCF3	GGACCCCAGCATCTGCAC	ATGTGCCAGATGCACCTCAA
19 TCF3	GGTCTGCGCTTTGTCCGA	CTGTCCCTGGAGGAGAAAGAC
19 TCF3	GCTCCCGCGCGTTATTG	ACACAAGTAGCTGGATACCCTCT
19 TCF3	AGCTAACGGGAAGCCTCCTA	CTGTCCCCTTCGGGCAT
19 TCF3	GTAGTACTGGGAGGTCCCCTT	GCTTCGGCCTTTGCAGTTTT
19 TCF3	GTCACTGCAAGGAGGCAACT	GCTGTCTCGGTCATCCTGAAC
19 TCF3	TCGCACTTGCTGCTCCAA	TGCGGGTCCGTGACATC
19 TCF3	CAGCTCCTTAAAGGCCTCGTT	ACCTCACTGTCTCCCTCTGG
19 TCF3	GTCTCGAGTGGCCGTTCT	GGAAAAGGTGTCAGGTGTGGTT
19 TCF3	GGGAGCTGAAAGCACCATCTG	ACCTCACATCTTTCTCCTCCCT
19 TCF3	GGGAGACGGGTGCATCA	GACATGCACACGCTGCT
19 TCF3	GGGCCGGTGAAACCTGAG	CCACCCTGCAGCAGAGTA
19 TCF3	TCGTCCAGGTGGTCTTCTATCT	CCTGGCATATGCAGCTTCCT
19 TCF3	CCCTGCCCTGGGTCCTA	CAGCCGAGCTCTGGAGTC
19 TCF3	GTGCTGCTGCTTCCACTG	ACTCGGCCCCCACCTTA
19 TCF3	ACTCCAGAGCTCGGCTGA	CCCCAGCAGCACCTATCC
19 TCF3	CCTTCTCAAGGAGCGTCTGT	TCACGTCCCCACTCCCTT
19 TCF3	GCAAAGCCTTCACAGACCTCA	CCGGATCACTCAAGCAATAACTTC
19 TCF3	GGGTAGAAGGGCTGGACGA	GGGATGCCCTCGGCAAA
19 TCF3	GGGACAGGAAACTCAGGGTCT	GGGTGACCCATAACTCTCTCTGT
19 TCF3	CCAGTGTCCCCTCGGAGA	TGCATGGAGCAGAGGTGAAC
19 TCF3	GAGGAGAAGGAGGATGCAGATG	CCAGCACGAGCGTATGGTA
19 TCF3	CCATGTACCTGCCACGTAGAAG	TGATCGTGGTCCCTCCGT
19 TCF3	AGATCTGCGAGGAGGACCA	GGGCCTCACCTCTCTTCAC
19 TCF3	CACTGTCTTCAACAGACCCTTGA	CCTGTCACCAACGGGAAGG
19 TCF3	CCTACCTGAACCTCCGAACTG	GGTTTGAACCCGGGACTGTA
20 ASXL1	GGGTGGGCTTCTGTTCTCTTTTAA	TCTGAGCCGCATGGGTAAAAA
20 ASXL1	GCAGCAGTGCACTAAATAACGAGT	GGGTTTTAAATACCAGGAGACATGCA A
20 ASXL1	TGTATAACCCTCATCCATTCTTTTGTG G	tgCACCAAAAGAGAGTTCATATCACTT
20 ASXL1	CTGGGAGAAATGAGCTTGTCTGA	GCCTGGCCACGAATAGCAG
20 ASXL1	GCTGTGCCTTCAGGGTTCT	TCACCTGTGGCTGGCTTC
20 ASXL1	CCGCTCCTGAGAGGCTT	GGCCACCCAAGAAGTAAAAATAAAC T
20 ASXL1	CAATTGAGCAGATTGTGTGCAGAT	GTGTCTCAAAATCTATTTCTTCCCCTC T

20	ASXL1	AAATCATAGGTCAAATGAAGCGCAAC	TGTCTACTTCAGGCAGGAGGAA
20	ASXL1	CCTTACCATCACACTTCCAGCAG	CATCCGTCCCCACCTAGAAG
20	ASXL1	ACAGCAGGCACTAGGGACTAA	GGCTAGGCTCTACCTCTTCCTTAC
20	ASXL1	CCCGAATGCACTTACTAGAAGAGG	CCAGGACTCATAGCTCCAACTG
20	ASXL1	tgtaATTTGGCTCTGGGCAGAA	GCAGGTTCATACAATGGTGTTTATTCAAA
20	ASXL1	CTGTGGATCAGGAACCCAAGG	CGGTGTGAACACTTTCAATTGTGT
20	ASXL1	CTTGAAGATCGTCAGTCCTTTCGTA	ACCCTCAAAGAAAACCTGGCT
20	ASXL1	CTGTATGCCATGACCCTTAAGCTA	AGTGGGCTGACCTTTAACCAC
20	ASXL1	TCACCTGAGTTGTACCTTGCTG	TCACCCATCCATTAAAGGGTCCTTA
20	ASXL1	CAGATTCAACTTTCACGTATCAAACCA	CCCCTCGGACCTGCAGA
20	ASXL1	AGGACCCTCGCAGACATTAAAG	ACCACCATCACCACTGCTG
20	ASXL1	CGATGAGGGAGGTGGCAGA	CCTGGGTATGCTCCCCATT
20	ASXL1	AACTACTGCCGCCTTATCCTCTA	TCCTAGCCCATCTGTGAGTCC
20	ASXL1	GAAATTGCACACTGAAATTAGGACGTT	CTATGAAACCCTCATGTTAAGCAACAAG
20	ASXL1	AGTTGACTTGGGCTCTCTTTTGT	AGCATCACAACCATGAGTTTACACAT
20	ASXL1	CATGTCCACTCTGGCCTGAAA	ACTGATTCTCCTGGGACACACA
20	ASXL1	GAGGCTGAAATCAAAAGTGGCT	CCTGCTTGTTCTGACTCCTGTTTT
20	ASXL1	CAGAGCCAGAAGGAATCTGTACAA	CCCTGCTGGGTCAGTCTTAG
20	ASXL1	GATGTTCCCCTCTACAAGGATGG	CTCAGCCTGGATCCGAGAAG
20	ASXL1	CGAATTCCCAGTTGAGTCTGTGG	CTGCTCAAAGGATTTCCTCTTCTGAT
20	ASXL1	GAGGGAAAGTGATACTAGACAAGAAAACT	GCTTTCTCCCACTCCTCTCCAA
20	ASXL1	GCCTACTACAGAGGGCTACAGTT	GGCTGCTCCACTAATCTCTCAG
20	ASXL1	CCATCCAGTGACAAATCCCATTACAT	TGTTCTCGAGGTAGTAAGATCTCCTG
20	ASXL1	TCCGCCAAAAGATCCCAGATT	TGGTGGAAGAACCTTCTCTAGGG
20	ASXL1	CCTTCTGGAAATTACCCCGAGAG	AAAGGTGGGAGAGCTAGAGGAAT
20	ASXL1	GAATCCAGAGAACACATACCATCTGT	CTCCTCGAGATGGCACAGTC
20	ASXL1	CTGTCCTCCCAAACCTCAGTAG	CCATTGTCTGCAGGAACGGT
20	ASXL1	AGTTGCTGCAGGGTAGCTTG	CCATCAACCATGCCACTGTTTTT
20	ASXL1	AGTAATTCAAATGCTGCTCCAGGAA	GAGCCAAAAAGCTTCTTCCCTTG
20	ASXL1	CTCTTGACAGCCTTACTTCACTCT	GAGGTGACCTTCAAAGTCAGAGG
20	ASXL1	GAAAGTGATGATGAGGAGCAAGGA	GTGAGGATTCAGGTGTGGAACT
20	ASXL1	CCAACTGAGTTCCACCAGCTTTAATT	TGCTGTCAGTGAACATTTGCAAG
20	ASXL1	CGCTACGCATGGGATCTTTACAT	TCAATCCTGGCAAGACCAGTG
20	ASXL1	CTGGGTGATCAGAGCAATGTTACA	GTTTGTGCTTGGTCCCAACTTC
20	ASXL1	GAGCCACAGTGCATCACTTTC	ACAAGGCACAATACACAGAGCTT

20	ASXL1	TCCCACTATGAAGGATCCTGTAAATGT	AGAAACGAGAGCCTTGGTTTTCA
20	ASXL1	GATCCCTCCAGTTTTTCCCAGT	TGTTCTCGGCATTTGCCTTAAGA
20	ASXL1	ACGATGACTGTATTGGACCCTCA	AGAGTGCTCCTGCCTAAAGAGTATT
20	ASXL1	CGGTGAGTCCACGGATACAG	GTCACCATTCACCTTGGACAGT
20	ASXL1	TTCTGCCAGATAGCTGTGAAACA	GGAATCCATTTCTTCCAGTTTCCTAGAG
20	ASXL1	AGCGTCAAGAATGAGAAGACTTTTGT	TCACTGAGCCCCTTCCCT
20	ASXL1	GGATGAGAAACCCAATTGGAACCAA	CTCAGTACTGGCCAGCAGTAG
20	AURKA	GAAAGGTACATTGCTATGGAGGACA	ATGATGCTACCAGAGTCTACCTAATTCT
20	AURKA	ACTGTTCCAAGTGGTGCATATTCC	AGCTCCAAACCAAGTGTTAGAATGTTTA
20	AURKA	CGACAAGACCAACCTCCATGT	GGTGGCTTTGTTCAAATTAAAGGTTCTT
20	AURKA	CCAGAGTTCCCACAACGAAATTTG	CTGAGGAGGAACTGGCATCAAAA
20	AURKA	CCTGCAGTTCAGGCTGATAAGG	AGCCTTGGAGTTCTTTGCTATGAATT
20	AURKA	TCAAAAGGAGGCTTCCCAACTAAA	AACGAGAAGCACATCAACCTGA
20	AURKA	GGTGCAGCATTGGTGAACATT	GCAAAGCAAGTTTATTCTGGCTCTTA
20	AURKA	TTCTCCAGCTGAGCTTTAAATAACACTT	CTAGGAGGCAGTGGGCTTT
20	AURKA	GGCGACCAATTTCAAAGTCTTCC	ATGCATATTGAGTGAAGTTGGAATACGA
20	AURKA	CTAAGGCTCCAAACAATAAGTGCAA	CAGTGTACCTCATCCTGTCTCC
20	AURKA	CTTGCTCTTTTGGGTGTTATTCAGT	GGTCTTGTGTCCTTCAAATTCTTCC
20	AURKA	AAGCTTTTGTGCTTGCAAAGGAA	CTCTTCCATTCTAGGCTACAGCTC
20	AURKA	CACGTTTTGGACCTCCAACTG	CCTTCCATCTCTTATCAGTGACATTCAT
20	AURKA	GGTAAACCAAACAACTTCCACCTCTAAA	CTGTCTTACTGTCATTCGAAGAGAGTTA
20	AURKA	GATCCAAGAAGTAAGTTCTCTGGCTTAA	TGTGTTCCTTTCGGAAGAGATGTC
20	AURKA	CAGTAGCATGTTCCTGTCAGGTTATATG	CACCCCTGGATCACAGCAAAT
20	AURKA	TGTTTTGGCAATTTGATGGTTTTGATGA	CATTTGTTGTACTCCAGGTTGAATTCAC
20	AURKA	CCCTCTGTTACAAAGTCAGGGAAT	TGGAATTCTTTCCTTATCAGCCTGAAC
20	BCL2L1	CTAAACTGACTCCAGCTGTATCCTTTC	GGTGTTTTGGACAATGGACTGGT
20	BCL2L1	GGATCTCTGACCAGAGGCCAA	CGTAGACAAGGAGATGCAGGTA
20	BCL2L1	CTGCGATCCGACTCACCAA	CATATCAGAGCTTTGAACAGGTAGTGAA
20	BCL2L1	CCCATCCCGGAAGAGTTCA	CCGGGAGGTGATCCCCAT
20	BCL2L1	CCCTCAGCGCTTGCTTTACT	GACTGAATCGGAGATGGAGACC
20	BCL2L1	CCAGGATGGGTTGCCATTGA	CTTTCTCTCCTACAAGCTTTCCCA
20	BCL2L1	GGTAGAGTGGATGGTCAGTGTCT	CTGCAGGATACTTTTGTGGAACTC
20	BCL2L1	GCTGCTGCATTGTTCCCATA	GTTTTTCACATCACCAAGGCCATAG

20 GNAS	TGTTGATTAGTTCAAGCTCTTGCCT	GGTTGGGTGGCGGTTACTTAC
20 GNAS	CAGAGATCATGGTTTCTTGACATTCA C	GTTCTTTACGAACAGCCAAGCC
20 GNAS	CGAACCCACAACTCCCTGAAG	CAATCGCCTCTTTCAGGTTGTTTTT
20 GNAS	GCAACCAAAGTGCAGGACATC	CCACAATGGTCTGAAAATGATTTAGC AA
20 GNAS	TGCTCACGCTCTTGGCTT	GGCGGTCACTCCACAAAC
20 GNAS	GTTTCGGTTGGCTTTGGTGAG	TGAATGTCAAGAAACCATGATCTCTG TT
20 GNAS	CACGGCCTCCCTTCTTGTA	CAAGAGTGGAAGCCATACACAC
20 GNAS	GCCGCCGCGTGTACTAC	GAAGGCTTGCACGAGAAAGTT
20 GNAS	CCCCAGCGCAACTTACTCC	GCTTCTTTGCGCATCTGTCT
20 GNAS	CCTCCCTGCCCAAAGTGTTA	CCCTGCACAGATTTGACACTTAC
20 GNAS	GTACCCCTGGCGGAGAAG	GCAGAGTCCGCCGCATT
20 GNAS	CCATCTTACGGAGCCCCAAA	GGGCTGTTCCCCGATTTCAG
20 GNAS	GCAATAATATGTCAGGACAACGCGAT AT	GCCTCGCCATCATTCTCGAC
20 GNAS	ACTTCAGGAGCTACAGAGATGCT	GAAACTAAAGACAGGCTCGACTCA
20 GNAS	GGTGCCTTGCAGATTAGGTGA	CTCAGTTTCCCAGTATGATCTTCATGT T
20 GNAS	CGTCGCTGCAAGCCAAA	GTTTAGCGGTGGGCAACTAAC
20 GNAS	TTCCCTGACCGCTTTGCTAAA	CAAAGTCAGGCACGTTCATCAC
20 GNAS	TTCAGAGTGGACTACATCCTGAGT	TGCCTGAAGTGTGGTTTGGAT
20 GNAS	TCTGGCTCTCCTGCTCCAT	GCCTCCTCGTGCTCGTG
20 GNAS	CCCCCGAATCGGAATCTGAC	TCTCAGGCTCGGTCTCGAA
20 GNAS	CCGAAATCGAGTCCGAGACC	GGGCTTCGCAACTTGAGA
20 GNAS	CTCCACCTTCGGCCAGT	GATCTTTGTCCTCGGGCTTGA
20 GNAS	CGACGCCTCCCCAAGTC	GGCGGGTGGGCTTCTTT
20 GNAS	GAGACACAAATAGTTGGCAAATTGAT GT	TTTGGAAAGAGGGCTCAGAGTC
20 GNAS	CCTCACAACGAGCCCATCC	TTCAGCCTCGAAGGGCATT
20 GNAS	CTGGCTCCAGAGGAGGCTA	CTGCTGCCGTCGACTTG
20 GNAS	GCTACAGCCCACCTCCTGA	CTGGTCTGGCACCACTGAA
20 GNAS	GGCTTTCCCGAGTTATCGCA	CTGCTGCCCGTGAAGTTAGA
20 GNAS	CATCCCAAGAGGCTGTCAGA	CCGTCAAGCGCGATTGG
20 GNAS	CACTCCCGTCAACATGGACA	CCCCGGAGAGGGTACTTTTC
20 GNAS	CTTCGGCCCAGCACTCAT	AGCAAGGTCTGGGACCTGTAA
20 GNAS	TGGAAGGAGCCGCTGATG	TCCCGGAGTCGGGATCTT
20 GNAS	GGCAGCCTCAGCGGATAC	CGGGATCGGCTGCGAAT
20 GNAS	GCCAGACGCAAGATCCATC	GCTGGCCACCCCTTCAT
20 GNAS	AGAGGCTGCAGTCAACTTCTC	GCTCAAATGGCATAGTCTCCTCA
20 GNAS	CCGGCTTCTCGGTGTGT	CGTTGGAGGTGGCAAGGG
20 GNAS	GTCACTGACAAGTCCCCTTGTT	ATGTCACGGCAGTCGTTGA
20 GNAS	TGAGAACATCCGCCGTGTG	CTATGGTGGGTGATTAACTGCTTGT
20 MAFB	CGGCGACGCTTGGTGAT	CAGCCGCTGCAAAGCTTC
20 MAFB	CCGCGAAAGCTGTCGAA	CTCGAGGATCTGTACTGGATGG
20 MAFB	GGTTCATCTGCTGGTAGTTGCT	GGCTCGGTGTCCTCCAC
20 MAFB	GAGCTACACGGAGTGCTGAG	CCGCTGGCCATGGAGTA
20 MAFB	CAGGTCGAAGTCGTTGACATACT	GCTCTTCTCCGCTCTTCCC

20 MAFB	GTCGAAGTCGTTGACATACTCCAT	CCAGCTCTTCTCCGCTCT
20 MAFB	gCCACGACTCACAGAAAGAACTC	GCAGCTTAAGCAGGAGGTGTC
20 MAFB	CTTGACCTTGTAGGCGTCTCTC	GGTATAAACGCGTCCAGCAGAA
20 MAFB	GTCTTCTCATTCTCCAGGTGGT	ACCAGCTCGTGTCCATGTC
20 MAFB	GCGGATCACCTCGTCCTT	CAGCTGCCCACTAGCCA
20 MAFB	GAGCTGGTCGTCGGAGAA	ACCCGCACCATCACCATC
20 MAFB	tcgctcAAGTCAAACAGGTCAA	CCCCTCCTCTCCCGAGT
20 PLCG1	GTGAGATGTCTATTCCCAGCTGTT	GCAACATCCAGGGACCAGTG
20 PLCG1	CTCTTCGTCTTCTCCATCAGCAT	CCAGAAGGTTCCAGCAGAATCT
20 PLCG1	CTGGCTCACAAGTCCCTCTT	GACCTGAGCTGGTTCCTCAC
20 PLCG1	CCTTTGTGTGCCCAGACATCT	CTACAGCCACAGCTCAGTACTTA
20 PLCG1	AGCTGTTGGGCCTAAACCTG	ATGCCATTCTTGATAGAGTTGCTGAT
20 PLCG1	CATCCATGATGTACTCTGAGAACGAC	GGGATACCATTCCTGGAACAGA
20 PLCG1	CAGCTGGACTGGAATACACCAT	GGAGGCTGCTCTTGTCAAAGG
20 PLCG1	GGCCCTTCTTTGTCTGCCTAC	AGGATGACTGGGAAGACAGACT
20 PLCG1	AAGCACCATGCGGGATGA	ACTTTTGGAGAACTCAACATAAACAG ACT
20 PLCG1	CATGAACAACCCTCTTTCCCACTA	AGGAGGACTCACTGGAGAACTG
20 PLCG1	CCTCTCCTTGCCTATCCAGGTA	GGAGCAACATCACCATGGTCAA
20 PLCG1	TCTAGAAGTGCAGAGGAGTCATTGA	GCGGGATTCAAAGGAGCCTT
20 PLCG1	TCACTGTCTCTTCCCTTCCACAT	CCAGTAGTGGGAAAGAGGGTTG
20 PLCG1	CCTCAGGCCAGCTGTTTCAT	TCTAGCACCCCTCCATCTTCC
20 PLCG1	GTATGCCCGGACACCATGAA	CAGGTAGGTATGGTCAGATGGG
20 PLCG1	CTCCTTGAGTTCCACCCTCAT	GAGGTTGTCTGTCAAGAAGAACTTG
20 PLCG1	GTATCCACTCCCGGCAAGAT	GGCTCTGAAAGTCGCATCTCA
20 PLCG1	CCCCTGCGCTGTAATGAGTT	CACCTGAGAACAGGCAGAGTC
20 PLCG1	CTGCTCTAGCCTGCCTTCTTA	CCTCCCCACTCCAAACCAAAC
20 PLCG1	GGGCTACTTAGACCCAGAGAA	GGCTTGGTGAGGAGTGTGTC
20 PLCG1	GCCCAATACTTCAAGAAGGTGCT	GGCAAGGCACTGAGAAGTGT
20 PLCG1	AGAGAGAGTGTAAGAATGAGGAAAC CA	CTGGGTCCTCTTGATAGCGA
20 PLCG1	GAAGACCTCACGGGACTTTGA	CCTCTACCCCCTTAACTCCCA
20 PLCG1	AGAGGTCATGAGAAGCTGGATGA	CCTGCCCCCATCTACAGACA
20 PLCG1	ATGGGAACCCCTACCAAAGGATA	GGTTGACCATCTCTTCCACGTAG
20 PLCG1	GCAGCTGTGGTTCCCATCAAA	GCCAAGGAGAATCTGGGAATAGG
20 PLCG1	CCTGACCCTGTGTGACTGTTTT	CGGTATAGCGGGTGTTTCTCATAG
20 PLCG1	GCCTTGTTGACCTCATCAGCTA	TGGTAAGATTCTGGGTCTCTCCTG
20 PLCG1	CCGCTTGACCATGGTGATGT	TCATCCAGCCTCCGACTCA
20 PLCG1	GCTACAGGGCCTTGTGTGT	CCTTACTCTCTTCATCAAAGGGAACA G
20 PLCG1	GGAAGATAATGGAACGGAGGAAGAA	CTAAATACTCTTTTTGCACCCACCAG
20 PLCG1	CCCAGGGCTGTCTGTAGATG	TTCTGGGCGCTGTACATGAG
20 PLCG1	GCAGTTTGCTCAGCTGTACC	ACTGGAGAAATGGCACCCAAAA

20 PLCG1	GGAGAGCCTTTCTGCTCTGA	CCTACCACACCACTGGCTTAC
20 PLCG1	GGGCAGCTATTGATACCTTGCT	CCCCTTCAGGAGTGGTTCTTAC
20 PLCG1	GCACCTTGTTGTCTGTTGACCAT	CAATGTCAGCCCAGCCATAC
20 PLCG1	GGGCATTAACATATCCCATTGTGT	CCAGCTTCCTATGCCCAGAG
20 PLCG1	GGCCAGGGTCACAGTATCTTTG	CTCGATGCAGTACTCAGTAAGCAG
20 PLCG1	CGTGACGGGCGTCACAT	GTGTACACACGCTGACTGAAC
20 PLCG1	CCTGGTGGGTGCAAAAAGAGTA	TTGGTTTCCGGGAAGGATGAC
20 PLCG1	GTGCTTGCTACCGGGACAT	CCACATGGGCAAAGGATCGTAG
20 PLCG1	CAGCGACTGGATTCCTCCAA	GCAGATTATGGTGTATTCCAGTCCA
20 PLCG1	CCTGCCAGTAAGGACACTCT	GTTCGCGGCATTCTACAAGG
20 PLCG1	GAGGTGGGCACCGTCAT	CAGGAAGTGGGCGCACTTA
20 PLCG1	CCTGACTGGAGGCTTCTCTCA	GGTGAAACATCGGGCTCAC
20 PLCG1	CTGAAGCCTTTTGTCGTTGCC	AGCCAGGAAATTCTGGTCACTAAAC
20 PLCG1	CTTCGTGGTGTATGAGGAAGACAT	CACCCTGCCAGCAAGATTG
20 PLCG1	TATTTGTCCCATGCACACGGATA	AAGAGAAACAGATGGGAGCTCAC
20 PLCG1	AGTAAAAGGCCTGAAGACAGGTG	TCCAGGTCCTCACTGTAGTTGT
20 PLCG1	GGCCTGACTCTGCCTGTT	CAGTGCCACACCCCTCA
20 PLCG1	GATACAGAGCAGTGCCTTTGAAGA	CAATGACTCCTCTGCACTTCTAGAG
20 PTPRT	CAGCACAAGGGACCTCCTTA	GTGGAAGGTGACATCAAGTTCATAAC T
20 PTPRT	GCTCTATCACCCACCACCCTAT	TCAAAGTGGTCCGACGACTG
20 PTPRT	aaaCAAGCTGGCTCTCATGGT	ACGGAGGTCTACGGAGACATTAAA
20 PTPRT	CATACTGCTCCTGCCACTTCTC	GAACAGCCCTAACAGCCTCTC
20 PTPRT	GCTCTGTTTCAATCAGGGTGAC	GATGAGACAGCTGCTCACACT
20 PTPRT	CTCTAGAGCCCTGCTGACTTA	TCCCCTGAAAAGCTACAGCATC
20 PTPRT	CTTTGCTGAGTGCCTGGAAGTA	TCCAGCCTCGATTCTCTACACT
20 PTPRT	GCTTCAACTCAGCAGCAAAGT	ACCATCTGTGTCTCCTCTTTCCT
20 PTPRT	CTCTTTTCCCCTGCATCCTCAA	CTCTAACCACTCTGTCCTTGCTTTTTA
20 PTPRT	GTCCATATCAGGCAAGCATCCAT	CAATGATGAAGGCTTCTCTTCTAGTTC T
20 PTPRT	ACTGAATCCGTTGACGTCCTG	CCCTTGCACATGTCCCAAAG
20 PTPRT	CTTTCTTGTTTTGAGTTACTGTTGCCT	ATTGGCATCTCATTTTTGTTGTTCACA
20 PTPRT	cCCCCATGATTCACGGTAAAAAC	TTCACCTGGGAGCAGATTAACAC
20 PTPRT	TCCAGCATTGGTTTCTCCCAT	CACGGGAATGTATTTACCCATATCTC TT
20 PTPRT	GGTTGGAGTAACAGATATAGGAGGAG AG	CACAGTGCTTTTCTCCTGTTATCCA
20 PTPRT	AGAGAAGAAAAACTGCAGACTCCAA	CCTGCACTCTCTGTTTCCTGTC
20 PTPRT	CGAGAGTGCATCTCCAGCAT	CTCACTGTCTTCTCTGTTGGCA
20 PTPRT	GAGAAGCACAGTAAACACGAGTGA	TCAGCACTTTCTGGCCACATT
20 PTPRT	CCATGGTAAAATGCCATACCTGATAG A	CGTCTACGTGAAGGTGAATGGTG
20 PTPRT	CATTCCACACAGGGTTCCCTT	CCAACCCTGAAGGAGAATGACA

20 PTPRT	GAAGTAGTAATGGAAGTCGATGCAGT	TGTCTCTACTTCCCACCTGCTT
20 PTPRT	AGTGAAAATGAGAAACTAACAGACA CTT	TCCTTCATCTGACTGCCCATCAATATA
20 PTPRT	aggtgAAGGACCTTTGACTTACCT	GCTCCGCCTCTTCCACTTC
20 PTPRT	GGGAACGCCGTGGTCAG	TGCGGGAGGTTCCTCAGT
20 PTPRT	CTACAGGTGGCCAACTGTCTT	GCTTCATTGCCATTGACACCA
20 PTPRT	ATTCTCGGCCATGTCAAGCA	GGATAGGGCTGAATGCTAGGG
20 PTPRT	ACTCACTAGGGACTGGATCGT	CACTCGGATTGCCACCAAAATT
20 PTPRT	TCCTTTTTCCTTTCTAGCAGAGATACC T	GGAAAGTGTTCAAGCTCCGGAA
20 PTPRT	ACAAAGAGGTGGTGGGTTTCA	CTTCCTGAGTGAGGCATTTATTTGTTT
20 PTPRT	GAATATTTGCCCTAGAATGCCAGCTA	CCCGGAACCATGATAAGAATCGAA
20 PTPRT	AGAGGCAGCACGTCCATAC	GCAGGGCCTATCTATTCACCTTT
20 PTPRT	GTGCATACCAAGGGCACAGAAT	GCGTAACAACAAATCCAACATGGTG
20 PTPRT	TGGGATACTCACCAGGGTCTC	GCTCACCTCTTTGCTCTGTGTTTA
20 PTPRT	GGTAAACCACAGATCTTGAGCCT	TCCCCTTGATTTCTGCTCTCTCT
20 PTPRT	AATAAAAGCCTCATAACTGACTGCCA	TGCGACTCTTGCTGTCTAACC
20 PTPRT	CAGCTCCTCGCTCTCCATTC	CTACAACCTCACCGTGCAGTA
20 PTPRT	CTGCTGGTTGAACACATACTGG	CTTGTTTCTTGCCTGCATGTTTTG
20 PTPRT	CCCCACAGGCTGGTCTCTTA	ACGATCCAGACTCATCCCTACC
20 PTPRT	GGTAGCTCATCTCCACAGGGT	GACAAAGGGCAAAGCTTCTATAGGT
20 PTPRT	CTGAGTGTGGGCAACTCTCC	TCATGTGTGTTTCTTTCTCTCCTTCTC
20 PTPRT	GGCAATAGCACAGCCTCTCATA	AACTATAAGCTGTGGCATCTGGAC
20 PTPRT	CTCGGGTCAGCAGAGTCTTG	CACCCAGAATTCTAACACTGTGGAG
20 PTPRT	CTCGGATCTCATACTCAACATCGG	CCCCATCATCCTGAAGGAAGT
20 PTPRT	CCTGTGGTGGTGCGATATTCC	TGACAGTCCTTTTTGATTGCTTGTCTT A
20 PTPRT	GTGTTGTCCACCTGCTTCTCT	aggaagctGTTGTCATTATGATGATTGT
20 PTPRT	CATGGATCAAGGCTGTGTTGG	CTGCAACTGTTTCTGTGAAATTGACT
20 PTPRT	GGGAGGATCAGCAGATGAGAAA	CGGAGCGTCAGCAAGTACC
20 PTPRT	CCATCAGAGCGGATCACACAG	CTATGATTCCTCCTCTCCCTCTCTT
20 PTPRT	AAGGGATCTCCCTCCAGGTTT	TTCGATTACAACTGCTCCTCTGTG
20 PTPRT	AGTGTCCATCTCATTCAGCATCAC	AAGCCTGTGTCATTCCTTTGTCA
20 PTPRT	CGAGCCTGGGATTTGCTCATA	GTGTTTGTGCACGATGCCAT
20 PTPRT	AGTAGAGAGAACGGAACTCACACA	CCACAACACTAAATGTGGACAGCTA
20 PTPRT	AGAGCTCCTGAGCCCAGTTA	cgcctctcAGGAACAGTATAAATTTGT
20 PTPRT	AATGGCGGCCGTGTACTTA	CTTCTGTGACCTCTTTCCTTATCTCAG
20 PTPRT	TCTGAGCAGAGGCTTCTCTCAT	ATTCTCTTGTGTTCTTTGGTTTCTCTCT

20 SRC	CTGCCCTGACCTTTCTCGTT	GGACCTTCTTACCAGGGTAGGG
20 SRC	GCTGACTGAGCTCACCACAAA	GAGCCTAACATCAGACgctcaa
20 SRC	GCACAGAACGGTGTCCAGAG	CAGCTTGCGGATCTTGTAGTG
20 SRC	CCAAGGGCCTCAACGTGAA	CCCAGAGTCTGCAGCTGAG
20 SRC	GCCTGCATCCCTCCTCAA	GCTCCTCTGAAACCACAGCATAC
20 SRC	GCATGAGAAGCTGGTGCAGTT	TCTAGACATGAGAGGAAATGAGGCT
20 SRC	TGACGGCTCCCTTCTCCTT	GCAGCCAGAGACGGTACTC
20 SRC	AGCTGGAGCTGGGTCTCT	GCTTGAGGACCAAGGGTGTG
20 SRC	GAAGACCCGCCTAACTGCT	CCCTGGCCCAGCTTGAC
20 SRC	GGGAGATCCCTCGGGAGT	CATCCAGCAGAGGCAGCTAAA
20 SRC	CTTCCCCCACCCCACTT	CTCCTTCCGCCAGCACT
20 SRC	CCCTGCACGACCTCATGT	GCCGAGAAGCCGGTCTG
20 SRC	CCTGGACAGTCAGCACCATC	AGTATCCGTTGGCCAGCAA
20 SRC	ccCTGCTGCCTCTCCTT	CGCTGGCTGGCATCCTT
20 SRC	ACTTGCGTGTGGGAGACAAAT	GTCTGTCTCCGTCCTAGACTCATAG
20 SRC	CACCTTTGTGGCCCTCTATGA	CTGATGCCAGATCCTGCATGA
20 SRC	GAACTGACCTCACTTGCCTGA	GTAGTTCATCCGCTCCACGTA
20 SRC	AGATCGCCTCAGGCATGG	CCGCGCCGTGTACTCAT
20 TOP1	GTCCCAGGAACTGATGCTACA	CACGCAGCAGCAAAGTCTTAC
20 TOP1	AGAATTCGCTAGCCCTGGGTA	TCATGGTCGAGCATTTTTGCAAAG
20 TOP1	CAAAGCAGAGGAAGTAGCTACGTT	ACTAGGATATTAACACATGGGCTACA GT
20 TOP1	AGTCTGTCTTTACTACACTGTCCCAA	GGACTTCCAGTCTTCTCGATACTG
20 TOP1	GTTCTAGCACTCTGACCAGCAA	TCACTCATCCTCATCTCGAGGT
20 TOP1	TGTGTGGACAAGATCCGGAAC	TCCCTTTGTTAAGCTCAACACTAGAC
20 TOP1	TGGCTGTTGTTTGATATTCTCTCCTT	ACTTAGTCTCTGGGACGCCAT
20 TOP1	AACCTACAACTATTTATGGAGAACAA GCA	CCCTCCCCTTTAGGTACTGTGA
20 TOP1	GCCATTGCTGAGTCACCCTAA	GAACTCTGCCTCTTGAGACTGG
20 TOP1	GAAACTTGGAAAATTATGCTCAGCAG AT	CTTTCCACTTATGTCCTGGAGGAG
20 TOP1	CAGAGATGCCAAGGTTCCTTCTC	GCAGCTCTCAACTATCCCCTTAC
20 TOP1	GGTCTCCTATGAGTGAGAACTCCT	CCCCAAAGGACCTAAGTCTCTT
20 TOP1	GTAAGATAAGAGCCAGCAAAATCATG G	AAAGGATCTTCGCTGGGATGTT
20 TOP1	TGTTCCTTTCTTTACAGCGGATGAG	ACACAGTTCCCTTCATTTTATCCAAG AT
20 TOP1	TGAATGTGAGGCCCAGAAGTCA	CTTCAGGATAGCGCTCTTCTTCC
20 TOP1	GGCTGTGCTTTGTCTTTTAAAGGTG	CCTGGCCAAGAATCAAGAACCT
20 TOP1	TCACTTCGTGTGGAGCACAT	CAAGTCAGACAGTACAGGTACATTAA CT
20 TOP1	GCTTTGACCTAAATCTGTTGCTTTGT	CCCTATCTGGCCAAGCAATACTTA
20 TOP1	ACAGAAGATAGCTCATGACTCCTGTT	GGACTTGCTTGATTCCACCCTTAC
20 TOP1	AACGAAATTGTGTAAGGATCATGTCT CT	CCATCCAACTCTGGGTGTAGATTG
20 TOP1	GTGCTTTCTCACCATGTTTCTTTGT	GCATTTTAACCCAGTGCTGAGATGTT A

20	TOP1	CCCTACTCTGGGCTAACGCT	AACTCTGTACCTTTTCCTCttttcgtt
20	TOP1	CGCTTTGTGACTTAACTTTTTACAGTGA	CCTCCCCTACTAGAAAACTCTGTACC
20	TOP1	GTGCTTGGGTGTATTTGCAAAGAA	TCTGGGCTTTGAAATACTGGCT
20	TOP1	CCTAAGCAAATGTGATTTTACCCAGATG	CACAATTCCAGAATTCTCCAGAGGTT
20	TOP1	CACAGTCACCGCCGCTTA	GGAATCGTTGTGGAGGTGGTC
20	TOP1	CTCCGGGCCGACATGAG	GGCTCCGGCCTGTCAAA
20	TOP1	GAGCCATTTTTCCTCTACAGCTCAT	CCCAACTTTCACAATACCAGGTACATA
20	TOP1	CTTTTATTGACTCAAAGTGGCAGGAT	TCAACTGTTCCTCCAGTCTCTGA
20	TOP1	GTAGTAGAGTCAAAGAAGAAGGCTGT	CTCAAGGAGGGCTCAACACTTA
20	TOP1	AAATGCATTAGTAGAGAACAGCAATCTGA	TCCAGGAGGCTCTATCTTGAAGTT
20	TOP1	GGATAACCACAAAGAGAGGATTGCT	CATAGGATGAAGTGCGAGCTCA
21	ERG	TGACTGGTTTCCCATGAAAGCA	CCTCTTCCACATTTGACTTCAGATGA
21	ERG	TGGAGAGTTTTGTAAGGCTTTATCAACA	AGAAAGGTAATTCTCTTTCTCTCTCTCTT
21	ERG	AATGGGAGGTCAGGGAGAGAAA	TCCTCCAGCGACTATGGACA
21	ERG	CGTGGGCTCATCTTGGAAGTC	GTTGGAGTGTCTTGAATTATGTGTTTCTT
21	ERG	ACCCTCTGAAGCTCAGGTCATA	AGGAACTGTGCAAGATGACCAAG
21	ERG	GTGAGCCTCTGGAAGTCGTC	AATGTGTTCCCACCCCTTTCA
21	ERG	TCTGCCTTGATAAAGGAAACCAAAGAA	CATCAAAACTACTTTCTGGTCAGAGAGA
21	ERG	CGTCAATGGAACAAACCCATCAC	TCAGCCTGGACCGGTCA
21	ERG	TCTGTTCAGTTGAAGGAATTTCTCACC	GATCCTTATCAGATTCTTGGACCAACAA
21	ERG	GGATTTGCAAGGCGGCTAC	GGAGGACATTTTCGGCTCTGAAAT
21	ERG	GAATGCACGCTGATGGGAAAAG	CCCCGTGACATCTTCCAGTTTTT
21	ERG	AGTTGGTGAATTCCAGTATGGGTTT	TGTACAAGTACCCCTCAGACCTC
21	ERG	GCTGCTGAGCCTGAATCTTCT	TGACCAAATCTGTTATAGCTAATTGCCT
21	ERG	CACGGATCTCAGCAGGACATC	CTTCACATGTTTGTCTTTCCTTTTGTCT
21	ERG	CTCCCTGAGGCTCAGCCTA	CCAGACACCGTTGGGATGAA
21	ERG	CCTCCATGTAGCTGCCGTAG	CGATGGAGACATGCTCTTTGATTG
21	ERG	gctgtGTTGACTGACCCCTA	GTAGATGGGCTGGCTTACTGAAG
21	ERG	GTGATAGGAGCCCATGTACGG	CGCCCTCCGTTACTACTATGACA
21	ERG	CATGGACCTTGGTCATGATGTTCT	TCCAACTCCAGCTGCATCAC
21	ERG	TCATCTTGAACTCCCCGTTGG	AACCTGTGCTCACTGGAGTTTT
21	ITGB2	GGAATCTGCTCCTTGGGTCAGAA	GTCACCTGGAGGACAACTTGT
21	ITGB2	CGAATTCGTTGCTCCTCTTGT	CCTTCTTCTCAGGAGGAAATCGG
21	ITGB2	CGAAATGGAAGCCGTCATCAGT	AGCTCTGCATCGTCTCCTCT
21	ITGB2	CCCTGTTCTCCCTGATTGGAA	CTGAGACCGTCACCAAGACC
21	ITGB2	CACCTCACCTGCCGACA	AGGTTCTTCCCCAGTGTGAGT

21	ITGB2	AGATGCCGCACTCCAAGAA	GTGAAGGTCACGGCCACA
21	ITGB2	GACAAACGACTGCTCCTGGAT	TCCTCCCCACGCTGAAC
21	ITGB2	CCTGACGGCCTTGTCTTCAC	ACACTCACAAGCCCCTGTTT
21	ITGB2	CCCCACCCAATGGATGCT	AACTGAGCAGGACCTCCTCT
21	ITGB2	CCAGGACCCCTCAGTGTG	CAGCCCAGAGGTGACTGT
21	ITGB2	GACATTGATCTGCACGCCAT	CCCCAAGAGTAACCGCTCTCT
21	ITGB2	CCACTCGTTGAATGGTCCAGAA	CCCGGCAAGCTGATATACGG
21	ITGB2	GATGGTGTCACACTCGCAGTA	CAGGAGCTGGAAGGAAGCTG
21	ITGB2	TGAGCAGATGATGGAGTTGTTGTC	TGTCCCTTGTCCCACATCAC
21	ITGB2	GGTGCAGAGGTGCTCACTG	CCTCACCCTGTGGCAAGTA
21	ITGB2	CTCCGCCTGCACTCACAT	GTGCGAGAGGACCACTGAG
21	ITGB2	GCCACGACCACTACACTCAA	AGGAGCTTAGCCGTGCTG
21	ITGB2	GAGTTGTTGGTCCTCTGGGT	GGAGTGCACGAAGTTCAAGGT
21	ITGB2	GCCCCGACTCGATGCATTC	TCCTCTGACCTTCCTGCTCTAC
21	ITGB2	CAGCAGGAGGTCGCATAGT	GGAAGGCTCTGATCCACCTGA
21	ITGB2	CCTTCTCAAAGCGCCTGTACTC	GGTGTGCTGCTTCTTCCCTA
21	ITGB2	CATGGGTCCCAGCTGAGTG	CGTGCCATGTCTGGTCTTCTT
21	ITGB2	GCGGCGACCTGCATCAT	TTCAGGCACGTGCTGAAG
21	ITGB2	TGAAACTGGTTGGAGTTGTTGGT	CCTTCGTGGACAAGACCGT
21	ITGB2	GTGCGTGTTCACGAACGG	CCCTGCTAGGAGAGAGGAAACA
21	ITGB2	ACAACACTGGAGGTGAGATCCT	GGATGGACCGCTACCTCATCT
21	ITGB2	ACCTCGGCTCTCATCCACAT	CTGCAGCTGTCGAACAACC
21	ITGB2	GCAGGTCCTGCCCTTCAC	TGGCACCGATGCTCACA
21	ITGB2	ACCCGGACACATGCCTTCT	GAAGCAGCTGTCCCCACA
21	ITGB2	CGCAGGTAAAGCGTCACTTTT	GGATCCTGACTCCATTCGCT
21	ITGB2	ATGTCGTCAGCCGCACA	AGTCCTCCATGGCCAGTCA
21	ITGB2	CTGAGTGCGAGGAGTTGTGT	GCTTGATGACCTCAGGAATGTCA
21	ITGB2	AGGTCGCCACCTAGCTTCT	TGTGGCTCCTTTTGTTCTGTCC
21	RUNX1	GTACCAGCCCCAAGTGGATG	AACCTCATTCTGTTTTGTTCTCTATCG T
21	RUNX1	TCAGCAGAAACAGCCTTAATTATTTG GT	TGCATACTTGGAATGAATCCTTCTAG AGA
21	RUNX1	ACATACCGTGGACGTCTCTAGAA	TGTCAAAGCTATAAAAATCTCCAGTG CT
21	RUNX1	GCATTCTGAGGGCTGTCATCTT	CGAACTTTCTCCCTGGTCACAC
21	RUNX1	GTTGGTCTGGGAAGGTGTGT	CTTTAACCCTCAGCCTCAGAGTC
21	RUNX1	CATCTGGTACTTACCCTGCATCT	GGGAGCTTGTCCTTTTCCGA
21	RUNX1	CAGCTGCTCCAGTTCACTGA	GCCCCAGTTTTAGGAAATCCACAA
21	RUNX1	TCTGCACCGAGGTGAAACAAG	GAGGGTGCATTTTCAGGAGGAA
21	RUNX1	GTTAAGACAGACCGAGTTTCTAGGG	CATGAAGAACCAGGTTGCAAGATTTA
21	RUNX1	GACCGACAAACCTGAGGTCAT	GCATTTGTCCTTTGACTGGTGTTTAG
21	RUNX1	CCCTCTCCGGGCCAGTA	ACCGCAGCATGGTGGAG
21	RUNX1	CAGCACCTCCACCATGCT	CCGTAGATGCCAGCACGA
21	RUNX1	CAACGCCTCGCTCATCTTG	GAGCTGTTTGCAGGGTCCTAA
21	RUNX1	CTCCACCACGTCGCTCT	AGCTCGCCCTCCTACCA
21	RUNX1	CCCACCATGGAGAACTGGTA	CATCGGCATCGGCATGTC
21	RUNX1	CGGCAGGTAGGTGTGGTA	CTGCCCTCCATCTCCGA
21	RUNX1	GTGAAGGCGCCTGGATAGT	CCGCAACCTCCTACTCACTTC

21 RUNX1	CGGGCTTGTCGCGAACA	AACTCCCCCACCAACATGG
21 RUNX1	CCCAGCTCAGCTGCAAAGAAT	CCTACGATCAGTCCTACCAATACCT
21 RUNX1	GAAGGAGAGGCAATGGATCCC	AGGGCCACTCATTTCTTATTAAAAGACA
22 BCR	AGTATGGATGCTCCCTTCTCTGT	ATGAGGAGGGCGAGTCATC
22 BCR	CAAGATGGGCTGCCCTACATT	GCCACACGTACACCATGGG
22 BCR	TTGTGTGGAGCGCTGGAAT	ACATAAGCAGCAGCAGTGACTC
22 BCR	GAAGAAGAAGCTGTCGGAGCA	AAGTGAAAACGCACTGCCATTAG
22 BCR	TCGGTGCATGTGAACGTTCTT	CAAAGCGTCCAGCTCCTCAT
22 BCR	GCTTCCAGATGGTGGATGAACT	CCACTCTGACCACACCAAACC
22 BCR	TCAGAGGACCCTTCAGCCATAA	GATCTTCGTCTTGTTGTAACACTTTTCA
22 BCR	CAGACCCTGAGGATACTGTGCTA	ATGAAAAAGGACGCCATGCATC
22 BCR	GTGATGTGGAAAAGACCTGTGAC	GGGCCCTTGTGGAATCAGTTC
22 BCR	CCTTCCAGGCTGACTTCTGTCTA	CAGCTCCCTAAATCCTCCTCAC
22 BCR	ACAGCACCCGCTGCTTT	CCAGCTGTCAGTCATGGTGATA
22 BCR	ACCTCGCCACGGTCTTTG	CCACCTCTGTCAGGTCAGG
22 BCR	GGTCTCTGGCTCGTTGTGA	CATGGGAGAGCTGGGCTAC
22 BCR	GCGGAGATAACTGGGTGTGTT	CCCTCTCAGGACAGAGGATACT
22 BCR	CACTCACCCTTGCACCGA	GGCGCTGTGACTTACATGGA
22 BCR	GCGAGGGTTCTCCGGGTA	CGGATCCGCTCGAAGTTG
22 BCR	GAGGGTTCTCCGGGTAAGG	CCATGGCCCTTGCGGAT
22 BCR	CGCTCAGGTCCAACTTCGAG	CACCTCTTTGTCGTTGACCTTCA
22 BCR	CCTTCTACGTGAACGTCGAGTTT	GATGCATCCCCCACGCT
22 BCR	ATGCAGATGGAGCGCAAAAAG	CCATTGGCGTCGATCAGG
22 BCR	CCCGCTTCCTGAAGGACAA	AGGTAAGGCGCTTCTCCTG
22 BCR	GGAGACTCACTCGGGATCCT	GGGTAGAACTCGTCAGTGAAGA
22 BCR	GCACCTGTCCCACTCTCTCTT	CTGAACGGCATCAAGGCATTAC
22 BCR	ACCTCCGGGTGTGCAGATAT	AGATGCCCACCTCCTCCA
22 BCR	AGCAGAGTTAGCTTGTCACCTG	CCTCCTCCCCAAACCAGTACTTA
22 BCR	ACCCCAGCCTTCCCTGT	GGGCCACATCGTCACTCA
22 BCR	CCAGTGCTCCTCTGTGTCTAA	TGATCTCCTCATTGATGCTGGAC
22 BCR	CCTCCGCATCTCACAGAACTT	CTCAGCAGGATGGGTGGAC
22 BCR	CACCTGTGCTACCTCACTTGT	CCATCATAGAACTCCTTGTGGATCTC
22 BCR	CTTCTTCAAAGTGCCTGAGCTCTA	CGGCCCTGGGTTACTCAC
22 BCR	CTGAGCCACTCTTCTCTTCCCTA	AGATGGACTGGGACCTTTAGACT
22 BCR	GCAAGAGACAGAGCATCCTGTT	CAAGGACACCTCAGGAAGGTT
22 BCR	GAAAGCTGAAATTGTTGCCAAAGG	GAGACCTCAGCATGCAGAACT
22 BCR	CGTGGAAGGCGCAGTTC	CGGAAGCGCTCCTGGTT
22 BCR	CAGCCAGAGCACCTCTGA	CTCGCTGGAGGTGAGGTT
22 BCR	CATCGAGCAGGAGCTGGAG	CCATCGCTGCCGGTCAT
22 BCR	GCTATGACCGGCAGCGA	CTGGCCTTACCCGGAGAA
22 BCR	CGGACTGCAGCTCCAATGAG	TGCTGCTGTCGAAGGACTG
22 BCR	CCCTCGCAGAACTCGCAA	CACCTGCGTCTCCATGGAA
22 BCR	GCAGATCTGGCCCAACGATG	ACAAGTGCACCTGATACACTCAA

22	BCR	CAATTGGTGCACCTCTTTTCCA	GCCCTGTACACGGAAACGG
22	CHEK2	GGATGAGAAAGGCAAGCCTACA	TTTCTCCCCCTCTTCTTAGCCT
22	CHEK2	ACGTGTTAATAAAAGGTGATCAGCCTT	TGGTGCCTGTGGAGAGGTA
22	CHEK2	GGGTAGAGCTGTGGATTCATTTT	GACGGACATTTTTCCTCCCTCTTT
22	CHEK2	CCTGAAGCCACGGGTGT	CCTGTTAATTCTGGCATACTCTTACTGA
22	CHEK2	GGACCCACTTCCTAAAATAGAGAACATT	CATACAGCAAGAAACACTTTCGGATT
22	CHEK2	ACATGGGTATTCATTACCTACCCTGAA	GCCCTCTGATGCATGCTTTTATATTACA
22	CHEK2	AAAGGGTCTTACCAAGATTGGCA	ACAGTGTCCACTCAGGAACTCTA
22	CHEK2	AGGTTCTTGGTCCTCAGGAATAGAA	CAGGGCATATCCAGCTCCTC
22	CHEK2	AGTTTGGCATCGTGCTGGTA	TGGACACTTTCTTTTTGAGGTCGT
22	CHEK2	TCAGAAATGAGAAACCACCAATCACA	GATGATCAGTCAGTTTATCCTAAGGCATT
22	CHEK2	ACCTTCCAAGAGTTTTTGACATGATGTA	ATTTTAGAATCAGTGATCGCCTCTTGT
22	CHEK2	GTAATTCAACTAAAAGAAAGGCAGCTGT	TTGCCTTGCCTTTGTGTTTCAG
22	CHEK2	CCTACCAGTCTGTGCAGCAA	CCTTATGTGGAACCCCCACCTA
22	CHEK2	AAGAACTTCAGGCGCCAAGTA	GGCAAGTTCAACATTATTCCCTTTTGT
22	CHEK2	GAACAAGAATCTACAGGAATAGCCACA	GTCATGCCTGCCTTTCTGTGTA
22	CHEK2	ACCACAGCACATACACATTTTAGCA	GGTATCCACCTTTCTCTGAGCAT
22	CHEK2	GTAAGGACAGGACAAATTTTCCTCCTA	AAAACGCCGTCCTTTGAATAACAATT
22	CHEK2	CTGCTTAGTGACAGTGCAATTTCAG	TGGGTCCTAAAAACTCTTACATTGCATA
22	CHEK2	TCCATTGCCACTGTGATCTTCTATG	GAGAGCTGGTAATTTGGTCATTGTTTT
22	CHEK2	ACATGTTTTCCTCTCGAAAGCCA	GGACAATGGAAAGGCTGTTATATGGA
22	CHEK2	TGTTTCAACATTGAGAGCTGGGT	TGTATAAGTAGAGCTGGGTTTGGAACT
22	CRKL	AGAGTCCCCGGTCCAACA	CCAGGGCAGGTGGAAGAATC
22	CRKL	GCCACGGTATGTTCCTCGT	AAATTCCTGGTCCCCGATCTTAAAA
22	CRKL	CATCAACTCGCTGCCCAAC	AGACCCGGTTCTCGACCTT
22	CRKL	AAGTTTGACAGGCACTGGCTTA	CGGCATCATTCCCAGGAAAATC
22	CRKL	ACCTGGAATATGTACGGACTCTGTAT	CAAGCTTTTCGACATAAGGGACAG
22	CRKL	GGCCGGGTTGGGATGATTC	TGCAGGTAGAGGAGTTGTGGT
22	CRKL	TCATGCATACGCTCAACCTCAG	CTCTAATGCCAAGGCAGTCTTGT
22	CRKL	CCAGAAAAGAGTACCCTGTGCTTATG	AATCATCCAGTGCTCTAATTCCATGAAT
22	CRKL	GTTGCAGAGAAATGCTAACTTTGTCTT	CTCGTTTTCATCTGGGTTTTGAGG
22	CRKL	CCCTTTACGCACGTCAAAATCTT	GACTTCGGCAATGCAGTGTG
22	CYP2D6	GGGTGTCCCAGCAAAGTTCAT	GGTGACCACCTCGACCAC
22	CYP2D6	GGATGTAGGATCATGAGCAGGAG	CCCCGTTCTGTCCCGAGTAT
22	CYP2D6	GAGTTGGAACTACCACATTGCTTTATTG	ATGGTGTCTTTGCTTTCCTGGT

22	CYP2D6	CCTTCTCAGCCTGGCTTCTG	TGGGCAACCTGCTGCAT
22	CYP2D6	TGGAAGTCCACATGCAGCAG	ATTTGGTAGTGAGGCAGGTATGG
22	CYP2D6	TGGTGGGTAGCGTGCAG	CCATTTGGTAGTGAGGCAGGTA
22	CYP2D6	GTCCCACGGAAATCTGTCTCT	CCGGTGGTCGTGCTCAAT
22	CYP2D6	ATCTGGGTGATGGGCACAG	TGACCCTCCCTCTGCAGTT
22	CYP2D6	ACTCCTCGGTCTCTCGCT	CCCCAACGGTCTCTTGGA
22	CYP2D6	AGCCACTCTCACCTTCTCCA	AAGGTCCTACGCTTCCAAAAGG
22	CYP2D6	GCTCAGTTAGCAGCTCATCCA	GCCTGAGACTTGTCCAGGT
22	CYP2D6	CACCCACCGGAGTGGTT	TGTTCCTGGCGCGCTAT
22	CYP2D6	CAAGTTGCGCAAGGTGGA	GTTGGAGTGGGTGGTGGAT
22	CYP2D6	AAGCCCTGTACTTCGATGTCAC	GCCGTGTCCAACAGGAGAT
22	CYP2D6	ATGAATCACGGCAGTGGTGTA	GGAGGCAAGAAGGAGTGTCAG
22	EP300	aGAGACTGTCTGTTTTTCAGGTCTTC	AAGAGAACTCCAGGTGCTTGTC
22	EP300	GATCTGATGGATGGTCGGGAT	TCTCCACATGGTGCTTGCATT
22	EP300	CGCTTTGTCTACACCTGCAATG	CTCTATAAAATACGTGGCTGCATGG
22	EP300	TTGCATGAGAAAGGGTGTTCAGA	GGCTGGTCTTCCTCCTCTTTTATT
22	EP300	AAGAGAGAAGCACTGAGTTAAAAACTGA	CCAGAACGAATGGCTTCAGAGA
22	EP300	CAGCGGACTGGTGTGGTT	AGCTTGAGTCCTGGGCAAG
22	EP300	CCCAATAGCATGCCACCCTA	TCTCCGCTGCTCTCTGAATCT
22	EP300	GCAGCAGTGGAAATGGCAAT	AAATGCCCACTGGGACCTC
22	EP300	GTATGAACCCACCTCCCATGAC	GGCCACTGACATCATGGCT
22	EP300	CAACTACAGTCTGGGATGCCAA	ACCTGAGAGTCCGCAAAAGG
22	EP300	ACTGTGTCTCAACAAGCCTTACAAAA	GGATTAGAGTTGGCATACTTGGCA
22	EP300	GTTGGCTGCATTCATCAAGCA	CGCCTGCATTGGATTCATGTTC
22	EP300	GTCCACTCCAATCCAGCCAT	AATTGTGAAGGCATGGTGTTGTG
22	EP300	CAGGCTCAGCAGATGAACATGA	TGCTGGAACTGGTTATGGTTGG
22	EP300	CCTTTGGTTAGAACAGCAGTCAGA	CCAAAGCGTACTGACTATTCAAGGG
22	EP300	TCAGAAGTCATGGGAAATATTGCAAGT	ACAACGTTAAGACTTACCCAGCAG
22	EP300	CAGGAATAGGCCCTGGAATGG	TGTCCCATATTTCCTTGTTGCATCT
22	EP300	CAGAATATGCAGTACCCAAACCCA	CTCACATGCATGCCGATTGT
22	EP300	GGATGCAGCATCACATGCAA	CTGTTGCTGAAGGAGTCGCT
22	EP300	TGCCAGTCTACAGGCCTATCA	GAGAGCGCACTTGATTGGAG
22	EP300	GCCAGCAGATCCCTAATTCTCT	CAGCTACCAGTCCAGGATGTG
22	EP300	TCTTGAGATGCTTTTAGAGCTTCAGG	CGGTAAAGTGCCTCCAAAGTTG
22	EP300	AGAAGAACTACGACAGGCACTG	ACCACCAAATACTTACAGGGATTCCTA
22	EP300	GTTTCCCCACAGACAAGTTCC	TTGCCATGCCTGGATTGCTA
22	EP300	CCATTCTTACTGTTCTAGCTTGTCCT	CACACCTGGCCAAGCTTAC
22	EP300	GCTTTCATGTTTCTTGTCAGCCAT	GGCCAGAAATCACCCTCGAAA
22	EP300	GATTAACAAGTGCAAAGGAATTGCCTTA	TCAAAGGAAACTCAATGCCCTTACA
22	EP300	CGGAACAGTTCACCCCAGTA	CTGGACAACAAACTAATTAGCACTTTTCA

22	EP300	ACTCTGCGTGTGTCTCACCTA	CATTTTGGCCTCCATCTTCACTTC
22	EP300	CCATTGCTGAGAAGCAGCCTT	TGGGTTCCTATACTGAGGTCCTAAC
22	EP300	AGTGTCAGCTTGAATTAAATGAGGTCTT	TTTCCGAATTCCAGTAGTGGATGG
22	EP300	CCTATGCCAACAGCAGCTCAA	ATGAGACGTGTCCACAATCTTACTTAC
22	EP300	GGGAGATATTCTGTGCTATTCCCAAA	GCTGCTCTTATTTTTGCTGGTTTTCTTA
22	EP300	GGAGACAGCAAAAATGCTAAAAAGAAGA	CTCTGGGTGGCTGCATCTTA
22	EP300	AATGAAGCAGTTTGGTGATTTGTGTTT	GTTACATTCATTCCCTGTGATGGGA
22	EP300	CCAGTTCCTTCCTCAGACTCAGT	GCCACTCTCTCCTATAAAAACCTACATC
22	EP300	TTTGTCTTCTCTAGCAATTTTGACTGGA	CTTTCTATGGAGCTGGGATCAATCTG
22	EP300	CCCCAACCTAAGCACTGTTAGT	CTGATTCTGCTGGTTCTTTGCTTG
22	EP300	TCAAGTAAATCAGATGCCGACACAA	TCATTTACTGACACAACCAATACCATGT
22	EP300	AATGCTGACATGATATTACAGTGGTAGG	TTTCCGAGCATATGCAACTAGGTT
22	EP300	TGCTTTAAAAGACAGACGGATGGAA	CCACAACAGGTTCAATCTTGGCTATTAA
22	EP300	TGTTTTCTTCTCTTTAGTGGCACACT	TTCATCACTTACGCTGTTGATTTCTCT
22	EP300	CCCCCTCAAAAATGCTGGTGATA	ACAGGTCCCTCACATAATCAAGTAGT
22	EP300	TCTGTTCTGAATTGCTGTCTTGTTATGT	GGTACAGGCGAGGGTGAATT
22	EP300	TTCCTCAACCAGCTCTTCATCAG	GGATGTAGAGCCTGGGACTGT
22	EP300	TGTTCCTACACCTCCTGCCAT	AGGTGCAGCAGGAAGTGAAG
22	EP300	CAACACAACTTCCCCAACAAGTG	TCTACTTACTGGAGTTGCAGGCT
22	EP300	TGTTCCTACCCCAACAGCAC	AAAGAGAATGGAAATGGCCCAGAA
22	EP300	TTCCTTTGAAACTGTCTTTGTGAACTTG	GCTGTCCAGGATTCTGAGTATATGG
22	EP300	AACAACCCCAATCCTTATGGTTCA	AGTACTCACCATGTTGGGCATTC
22	EP300	AAAAAGGCAGTTCCTGGTGGA	TCAATTTAGGCTCCTAGTGGGTACAA
22	EP300	GAGGTTGAACCTTAAGACTAACAACAGT	CCTCAGAAAGTCATTCACACGATTCTC
22	EP300	TCTACCAGACTTGGCACCTTTCTA	CGAGGAGAAGAAAAGTGAAATGACTAGAT
22	EP300	TGGATGATACTCCATCTCCCGTAAA	GCCAGGGCTCCTGATACTGT
22	EP300	CTACCATTAAGAGGAAGTTAGACACTGG	CATCACTGGGTCAATTTCTTGTTCAA
22	EP300	GCTCCAAGCTCTCTGAGGTCT	AAAGCCAGAAGCAGGATATTCTCTTATC
22	EP300	GGTTTGTGTGTGCAGTGAGTTTT	CAGATTGTGGAGTTATTCGAGGCAT
22	EP300	GCAGTGTGCCAAACCAGATG	GCATAAAGCGGGTGTTCAGGTA
22	EP300	TCAACGGTTTATCTAAGTTGTGTAAGCA	TCTAGGCTTCAGATAAGTTTTGCCAAA
22	EP300	TCTGTTACCTGGTGGTAGTTCCT	TGGTCCTTCGTTTTTCTTCTAGTTCTTT
22	EP300	ACTACCACCTTCTAGCTGAGAAAATCT	TTCCTGGTTGCGGCTGT

22	EP300	CTGGGAGAGTGAGAGGGTGTTA	ACTGCAGTCCTCAAGCATTTAG
22	EP300	CCTCGATAGTGTTCATTTCTTCCGT	CTATATCTCACCAGAATGCCATGCT
22	EP300	AGGCATTCAGATCTAACATTTTGCTCAT	CGTCAAACTAACCGGGTTGAGAG
22	EP300	CATGGACAGTTGGCTCAACCT	CAGAAGAACTAGGAGTTATGGTCTGTTT
22	EP300	GGTGGCTGTTGTATTTATTTCTGTCTC	ACCCAGGGTATAAATCACGTTAAGACT
22	EP300	AGAGGCTAGTTTTTGTTCTACGAAAGG	TTTCTCCATTTTGTGGTCATGGTTTTT
22	EP300	GACTTGTGTATCACCTGCTATAACACT	AGCATGGACCAGAGACTGGAT
22	EP300	CCAGAATTCAATGCTTTCTCAGCTT	TGCTCCCAAAATACTACAAGGTGTC
22	EP300	GATTCTCGCCGCCTGAGTAT	CATTGGTTTTCCGTTTGCAACC
22	EP300	TGGTTTTGTCATACTTTGACCTTTGTCT	GCCAAGACTTGTCTGAAGCTGATTAA
22	EP300	AGCGGGTTGTGCAGCATA	TGCCGGAGCTTCTGCTTG
22	EP300	TGGGACTAACCAATGGTGGTGATA	GGGCCACCAACTCCCATATTG
22	EP300	GGTGCCGTTCTGCCTAAACAT	CCAGTTGGTGTCGTTGGAGT
22	EP300	CGATCTGGTAGTTCCCCTAACCT	CATGTTGGGAGAAGTCAAGCCT
22	EP300	TGGTCAAAAGCCCAATGACACA	CCAGGATTCATGCCCGCAT
22	EP300	CATGGGAATGAACACAGGGATGA	TCAGTAAGTTGCCAGCACTTCC
22	EP300	CCTCGCTTGTATCTCCGAAAGAAT	CGAGTAGAAAAGATTAAAGGGAACGTG
22	EP300	TCCAACTTGTGGTTTAAAATGTAGCCT	CAGGAGGATGGCAATGGAAGATATAATC
22	EP300	TGTCCACCAAGTGAGGGAGAT	GCCCCTATGTCCCAACTGAC
22	EP300	ATTTCACTTTTCTTCTCCTCGTGGAT	GGCTTTGGTTCGGTATGGAAAG
22	EP300	GACAGTGGAGAGATGGCAGAATC	GCTAGCCACTGTGAGCTAGTCATA
22	EP300	TCTCTCAGGGTCAACAGCCA	GTGAGCATGCAAAAGGAGAACAAG
22	EP300	CAGAGAAGCGCAAGCTCATC	AGTGTGTCATGTGGTTTAGGACATTC
22	EP300	CCCCACTGTCGCACAATGAA	CTTCTTCTCCCTGGCTGTAAAAATTG
22	MAPK1	CAGCCAGGAACATGAGCTCTTA	TCTCACTACTGCAAAACCTTTCTCTTT
22	MAPK1	GAAGTCCGGGTTCGAGGTC	GCTACACCAACCTCTCGTACA
22	MAPK1	CGCCGATGTACGAGAGGTT	CCGAGCGTCGAGCTCAG
22	MAPK1	CCTCCCTTGCTAGAGCTCACT	AAGTTCGACATGGAATTGGATGACT
22	MAPK1	AGTTCTTTGAGCTTTTCCTTAGGCA	TGAGCAGTGGTCATTTTATGAATCAGT
22	MAPK1	CTTACGGCTTACTGCAATGCATTC	GCCCGTGTTGCAGATCC
22	MAPK1	GAACCCTGTGTGATCATGGTCT	CAGAAAGTATCTGCCACTTAGTAGCATT
22	MAPK1	AAAATTCACACCTACCACAGGTTAAAGA	GGAAGCATTATCTTGACCAGCTGA
22	MAPK1	GGGAACCTTACCCAAAATGTGGT	CTGTAACACCAGATACCATTTTCAGTCT
22	MAPK1	AACATGTTTTTGGGTATTTCTGGTGT	GACCTCAAGCCTTCCAACCT
22	MAPK1	TTGAGATCACAGGTGGTGTTGAG	TCATGGAAACAGATCTTTACAAGCTCTT

22 MAPK1	CATTGCTGAGGTGTTGTGTCTTC	TTTTCAATGCTTTGCTAAATGTGACCA
22 MAPK1	CCATAGGCTCATTTTTCAGGCTAGT	GAACTATTTGCTTTCTCTTCCACACAA A
22 MAPK1	GCCTGTTCCATGGCACCTTATT	TGTGTAGTCATTTACCTTGGTCCTGAT A
22 MAPK1	ACAAGGTTACCAAGCAGTGGAAT	AGAGAACCCTGAGGGAGATAAAAAT CTT
22 MAPK1	TTCTCATGTCTGAAGCGCAGT	GCTGTTTTTAATGCCATCTCTCTCTCT
22 MN1	GGGAAGTGAGAGGAAAACGAGTG	ATTGACCTGGACTCGCTGATG
22 MN1	CCTTGTCAGCGGGCATGTA	GGTGGCAAGAAGGGTGAGTG
22 MN1	GCTCGCTGTCGCCATTCT	GAGATCCACCCCCTGGAGAT
22 MN1	GCTGGATCTGCGCCTGAA	GCATCATGTCTAACTCTACCTCGAC
22 MN1	CCGCCGTAGCTGTCAGG	CAACCCCCAGGCACTAGTT
22 MN1	CCTCTTGGCCACGTTCATGTAG	CGCAATTCGAGTATCCCATCCA
22 MN1	GTGCATGCTCCGGTTCTC	ccaccCGGGCTTCTAGTC
22 MN1	GGCGGGCACGAATTTTGTC	GGGTCTGGAGCCCTCAGT
22 MN1	GCATTAACGGGTGCCTGGA	CATGGTGGGCTTGTCCAAAAT
22 MN1	TTTCTGGCCCCACTGAACC	GGCATGGTGGGCTTGTC
22 MN1	GGCTGGGCGTGCATTTTG	TCGCCCTCTGACTCCGA
22 MN1	CCTGCGTGCAACTCCGA	TGAGCATGAACACCCACTTTAAGG
22 MN1	CGCTCATAGCAGGATCCACAG	AAGTATGTTTGGGCTGGACCAAT
22 MN1	TCCGGTCTCGTTAAAGTTCCTCT	TCCTGGGCTGGAACGAGTA
22 MN1	GGGACTCCTGCTCGCTTT	GGGCCAAGTCGGACAGTA
22 MN1	GGCGAACTCACCCACGTTT	CGAAAAGGCGCTCACGTC
22 MN1	CCCAGGATGGCGACGTG	AGCCCTGGCACCTTCTTTG
22 MN1	GAGCCGCCGAGTACTTGT	CCCCCAACTACACCCTGGA
22 MN1	GTCATTCCCCGACGTGGAT	CCCGGGCTCGGATTACTTC
22 MN1	GCTTTGGAGCCGCTGCTA	CGTGACCTTCAACAAGAAGAACC
22 MN1	CAGACTGCCCCCGAACTG	TCTGCCCGCTGACTGTG
22 MN1	CCTCTCCGGGCATCCTCAT	GACCTGCTGCCCCGTAG
22 MN1	CGAGCCGCCCATCCTAC	TTGGCCCAGCCGAACTT
22 MN1	CTCAGTTTCCTCTTGCCCTCT	TCGCTGGGCTCCTTCAAC
22 MN1	GCCTGCTGCTCGAAGGTG	CCCAACCTGGCTCAGCTA
22 MN1	GCCTTCGCGCTCAAAGTTC	AACGCGGCCCTCATGATTAA
22 MN1	ATTCCGCGACGCCATCT	GACCACCAGTCCCTgcaa
22 MN1	GTCCTTGGAGCTGGGCTT	CGGTCCAGGCGTGAACT
22 MN1	GGCTGGGCTGGAAATCAGG	AGCCGGGCTTTCCGTTT
22 MN1	TCATGAGGGCCGCGTTTT	GCCCGACAGCTTCCCTTC
22 MN1	gttgcAGGGACTGGTGGT	ACAACGGCGCTCTGGATAAT
22 MN1	CTGGGTAGGCGGAAGGG	CAGCGGCTGCAGCATTT
22 MN1	GCGACCCGCTGCATAATGA	TCCGATTCCCACAGTCTGGA
22 MN1	GACGGCTCCTTGGTTCGT	CGGACTTCCACAGTTCAGGTG
22 MN1	CTCGGTTAGGGCTCTGGT	CTTCGCCGAGGGCTATGAG
22 MN1	CTGGCTCTCCGCCATGT	GGAAGTCATCATCCCCACCA
22 MN1	TCAGCAATAGTGGCCCTTTCAA	CATTCTGACATCTCCAACAGATTTGG
22 MN1	AGTTAGGGCAGCCACGAATG	AGTGCCTGTCTGTCCACTG

22 MN1	GTCACCCACGTCGTCTGT	GCCACTTTGGCCCCCATTAT
22 MYH9	ATAGGAAACTCAGTTGTAGAAAACTCCT	GAGAACAGGCTCACGGAGATG
22 MYH9	ACTGACACCCACCTGAGACT	GCATGTCTCTACCTCTATCTCCTCT
22 MYH9	GGTGGGCACACACTACGTC	AGAACCTGCCCATCTACTCTGAA
22 MYH9	GCCCTTGTACATTTCCACAATCTC	GAGAATCAAACTTCCTAACCTGTTCCTA
22 MYH9	CCTGGCCCCAGAGGACATA	GTGTCCCATCTCTTGGGTATCAATG
22 MYH9	GATTCCTCTGGTGAAATCGGTCA	CCTCTCTGCTCCCAAGTCAAG
22 MYH9	CTGCAAGGGTGACCACACT	GCTGAAAAAGCTGGAGGAGGA
22 MYH9	TTCTGGTCCTCCAGGATGATCT	TGCTGTCCTTACCGTGTCTCT
22 MYH9	CCCAGTTGTGGTTTCAGTAGGA	CGGGAGTTAATCTGATGTCTTCTTTGTTT
22 MYH9	ACACCGACCCTCTGTGATGA	GCTGGAGCGGCTCAACAA
22 MYH9	TCATAAGGTCCTCCATCTCCGT	GAGAAGACCATCTCTGCCAAGTATG
22 MYH9	ACAGAGCCTTGGTCTCCTTCT	GTAGCTCTGAGCCTCATGGTG
22 MYH9	GGAAAGAATGCTCACAGCTCACT	CCAGATCGCGGAGCTCA
22 MYH9	TTCTTGGCCAGCTGCATCT	GGGCTCCCCCTGCATTG
22 MYH9	AATTCCTGGAGCCCAGTAGGA	CGAGAAGAAGCTGAAGAGCATGG
22 MYH9	CCTCCTGCAACTGGATCATCTC	GGGTGCCAAGCCTCTGTC
22 MYH9	GTGACATTCGTGCCTTGCTT	GCTGGAGGACGCCACTG
22 MYH9	CATGGCATCGGCCGTCT	GGGCCACACGTCCATCTC
22 MYH9	GGCACATGTGTACCCCTTGTT	GAATGACAACATCGCCACACTG
22 MYH9	CAGCTCCGAGACAAACTTGTCA	GAGACCCTGTGAGCACACAT
22 MYH9	CTCACCCGTGAGCTGAGAA	CAAGAACAAGCATGAGGCAATGAT
22 MYH9	CCCTCACCTTCCAAGTCAGTG	CCAACACTAAATGCCAGCCTACT
22 MYH9	TGCTTGAAGGAGAGGAAATGCAA	CCGCCTACGACAAGCTG
22 MYH9	GCCGCGTCTTGGTCTTCTC	CCAGGTGGCCGACATGAA
22 MYH9	CCACACTGTCCTCCATCTTCTTT	TTCATAACTGGGCAGATCCCT
22 MYH9	ATGTTCACAGCAGTCCCAAGAA	AGGTAGATGGCAAAGCGGATG
22 MYH9	AAGAGGCTTATTCGGCAGGTTT	TGACCCTCTGCTTCTACCTCTG
22 MYH9	GTTAAGACACCTCCACAACCAACA	AGGGCATCGAGTGGAACTTC
22 MYH9	GGTCGAGGCCAAAGTCGAT	CTGACATTGTTTTCTCTCCTTGCAG
22 MYH9	CCAGATAGCACCGAGTCTGAAC	GACCGTGAAGAATGACAACTCCT
22 MYH9	GCCGCGCTACTCACGAA	GCCTTCACTGCGTCCCT
22 MYH9	GCACTTGGCCTGTGCTTCT	ACACGGAGCTGATCAACGAC
22 MYH9	TGCAGGTTGGCCTTCTTCA	AGGTGTGTCCCCAGGGTA
22 MYH9	CCTTCAAGCCCCCTTCTCAAC	GCACAACCTCAAGGAGCGTTA
22 MYH9	ACTTACGTAGATGAGCCCTGAGTAG	GGGAAGAAGGTGAAGGTGAACA
22 MYH9	GGTTCATCTTCTGGATGTCATCCT	GCCAAGAAGCTGGTATGGGT
22 MYH9	TCAAAGCCACTCTTGTCGGAAG	CCCGCTTAGGTCCTGGCTATA
22 MYH9	GTCCACCTTCTGGGAACCT	GTCCATGGTCCTTTGTGTCTCC
22 MYH9	GTTCTCCCAGCTCCTGGTT	CCTAGAGGCCAGGGTGGA

22	MYH9	TGTTCTGCTGCATCTTCTTCTTCTC	GGCAGAAACCGAGCTGTGT
22	MYH9	CAGATCTCTTCTAATTCCTGCTTCTTGG	AGGAGCTTGTGTTTTCCACTTGT
22	MYH9	CCCCACACTCGACCATAGGA	TCCTGTTGGAGCCGTACAAC
22	MYH9	CCATTGGACAGGAAGCGGTATT	TGTCCCAGTCTCTCCAACCTTT
22	MYH9	GGCTGCAGGCCAACTACTC	CCAGCTTGTGTTGCTCCTTAATATTT
22	MYH9	CATCATCCCACCAAGGCCAA	GACTTCCTTGCCTCTCTTGGTA
22	MYH9	GCTGGTTGTGGCCCAGATTT	AAGTACAAGGCCTCCATCACC
22	MYH9	GCACGTACTTGGTCTCGTTGT	CCAGAACAAGGAGCTTAAGGTCAA
22	MYH9	GACTTGACAGTGCCCTCCAT	AAGGTCACTGGCCCTTCTTC
22	MYH9	GCCCTGGCACCTTCATATGTA	ACATCGACTCGGCCAACAAG
22	MYH9	GATGGCTTCGTCCCGGTT	CTTCCCAAGTTGTTGATGTTCCTT
22	MYH9	CCCTAACACAATCCAGGTGGAA	CCATGAAGGCCCAGTTCGAG
22	MYH9	CTGACCAGCTGCTTCTTCTTCT	GAGATGAAGACGCAGCTGGAA
22	MYH9	GCAGCTTGGCATCTTCGGT	AGGTACCTGCCAGTCCCTTT
22	MYH9	GGAGTATCTCCCGGGACTCA	GGAGCTCGACAGCAATCTGTA
22	MYH9	GAAGACTTTGCTCTGGCCAATG	CTCGTCAGCCAGTGAGAAGAA
22	MYH9	ggcCCTTCTAGCACGCA	GACCATGTTCCTCACCACACA
22	MYH9	TGTGCAAGAACCGTACTCAGG	TGAAGGACAAAGCTGATTTCTGCAT
22	MYH9	CCTTGCCGGCATAGTGGATA	GGACGAGGAGTGCTGGTTC
22	MYH9	CCACGAAGCTCTTGTCGGT	TCTGATGTCCGGGCTTTGG
22	MYH9	GGGACAGCAGCTCAACTCA	GGGTAAAATGACCAGGCTTCTCTTT
22	MYH9	GATCGATGCAGGACCATGAGA	GGCTCTGGACAAGACCAAGAG
22	MYH9	CAATGTCCAGGATCCCGATGAA	cctttgTATCTGACACCACGCT
22	MYH9	GCTCCCTCACGACAGGAT	CTCGAGGGCATCCGTATCTG
22	MYH9	CCTGGAAGACCACCCTGTTG	CCCTGACTATTCTCCGACTGTTCT
22	MYH9	AGGAGGCAGCTTCTTCTCTACA	CTCTGATCTCTTGCTATCCTTTCTTACTT
22	MYH9	GGGCCACAACTGCTGCTA	GCAGGAGCTGCAGGTCAAG
22	MYH9	GCGCTCTCCCTCGTTGAA	AGGTGAAAGCAAACCTCGAGAAG
22	MYH9	CTCGTTCTCCAGAGTCTGCTTT	GAAGCAGCGATGTCTGGATCT
22	MYH9	CCAACTCTGCCCCCTCAC	GCTGCCTTTAGGTCACACTCT
22	MYH9	GCAGAGAAACGACTGAAGGCTCT	CAACTTTGTCCGCTGCATCATC
22	MYH9	CGCACCTTCTTCTCGTGGTT	AAGGGCATGTTCCGCACT
22	MYH9	GCCAGCTGCTCCTTGTAAAG	GGCACTCACGTGTTCTCTGT
22	MYH9	CCCCATCCCGAGGGACTT	TGAGCGTGCTTCCAGGAAT
22	MYH9	CCCGTTTCTGCTTCTCAGCTTT	CTGATGTTGCTGGTCGCTTTG
22	MYH9	CTTCTCACTGCCCCACCAG	CTGAGCCACCTTGTCTGTCAT
22	MYH9	GCGGTCTCAGGGAGGCT	ACGTGAGCAGGAGGTGAAC
22	MYH9	CCTCCAGGGTCTTCTTCAGGAT	GGTAGAAAAGCTGCCTGGAGTG
22	MYH9	CCCCAGCCTGCAGAGTC	GTGGAGGACGAGAAGAATTCCT
22	MYH9	TGCTTCTCCAGGTTGTGCTT	CCTCTCAACCCGCGAGTAG
22	MYH9	CCAAGCACCTGCCCCATTATT	GCTTCTGAGCTGCCTCTGATTG
22	NF2	TGGCAAACAATACCAAATTTACTTCATGT	CGGTGCTCTGCGTACCAA

22	NF2	GGAAATGTGGGAGGAGAGAATTACTG	CAAGCATGTCCTAGTTTTGCAGTT
22	NF2	CGCTCTCCACCCATCTCACTTA	AGCTCAGAGAGGTTTCAACACAC
22	NF2	CACTCATCACGATTTCAGGCCTA	GTACCTGGGAACAACCTACTTTGAG
22	NF2	CACTCTATGCATTCATCTTCACGTT	CTCATAAATAGATCATGGTTCCCGATACA
22	NF2	AGCTCTGTTCAGAAATGGCAGT	TGCTATGTCTTCCTGAAAAGGGAATTT
22	NF2	TGGCCACAGATTCTCCAGCTA	CATCAGTTAAAACAAGGTTGTGCTCA
22	NF2	TGTTTTTCTTCACCCCTCGCA	CCCAGCCCCTCAGAAATCAC
22	NF2	CTCACAGAGTATCATGTCTCCCTTG	CCAAATTAACGCCCAGGAAAAATACAT
22	NF2	TCAGCTGGCGCTTACAGTAG	ACTGTACGAGATGTTTCGGATTTCA
22	NF2	CAAGATCTCCTTCCCGTGGAA	GCCTGGGAATTAAAGGACTCTATGAAT
22	NF2	TCAGCTCTCTCAAGAGGAAGCA	AGAGAACCTCTCGAGCTTCCA
22	NF2	CCACTTCAGCTAAGAGCACTGT	CCTCCGTGCGAATCGCT
22	NF2	AGGAAATGCAGCGCATCAAG	CCTCCTCGCCAGTCTGGT
22	NF2	GCACAGGAGGAAGTGCCAATA	TCTCAGGATAAAATTTGGCCAAGAAGT
22	NF2	TCAAAGGAAGAACCAGTCACCTTTC	ACTCCTGGGCAAGTTCTCTCA
22	NF2	GCTGGAGGATCGGTTGTCAA	GCTGTCACCAATGAGGTTGAAG
22	NF2	CGTTGCCTCCTGACATACCAAG	CCCAATCACTCAGTCTAGTTCACA
22	NF2	CCTCTCAGCTTCTTCTCTGCTTT	CTCTCTTGATATCTGGTCCATCCC
22	NF2	AATTCAGTCCTCATCAGCTGTCT	GAGGTCCTTCCCTTTCCACTTC
22	NF2	TCCCCATTGGTTTGTTATTGCAGAT	AGTTTCATCGAGTTCTAGCCCAAC
22	NF2	GAGCCGTGTCTCACTGTCTG	TCTTTCAGTTTCAAGGCCTCGATTT
22	NF2	GCAGCTCAATGAACTCAAGACAG	TCCTGATCAGCAAAATACAAGAAAGAGA
22	NF2	GGGTGGTGTCTTTTCCTGCTA	CAATCTCCAGGAGCTTCTGCTT
22	NF2	GCAGATCAGCTGAAGCAGGAC	AGGAAAGAGAACATCACCAGGACTA
22	NF2	TGGTTGCGCATTTGTGGAATTT	AGGATCTCAACTTACCAGCTTATTAACAC
22	NF2	GTCTTCAAGTTTAACTCCTCAAAGCTTC	GCCAAGTGAGATACCATTCTATACTTCA
22	NF2	GCTAAAGGGCTCAGAGTGCAG	TCCTCACGGTGAACGTCTTG
22	PDGFB	GCCCCTCCCAGATCTCTTAAAGT	GCTTTATGAGATGCTGAGTGACCA
22	PDGFB	ATCATCAAAGGAGCGGATCGAG	CTTGGAGCTTTCCACAGGAGAAA
22	PDGFB	GGGCGAAGGTAATGAATGAAGA	CGGCGCGCTCGATCTAC
22	PDGFB	GGAAGACAAGACGTGGAGAGGTA	ATTTGGAGGATCTGGTGCAAGAG
22	PDGFB	GGAAGCCTGGTCAGGTATGAG	CCCTGTGTGGAGGTGCAG
22	PDGFB	CTGCACGTTGCGGTTGTT	GGTTCCCTGACCATTGCTGAG
22	PDGFB	TCTTGCACTCGGCGATCAT	gGGTTGAAGGGCGTGAGAAA
22	PDGFB	TGGCCCCCATGCTAATTTGA	GGTGTCTGACTGTGACTTCTCCT
22	PDGFB	ACTCTCCTGCCGATGCC	GCTGACGAGGTCTTTCTTTTTCTTGT

22	PDGFB	CTGAGCCTGGAAAGGTGGTTA	CCTCCAGGTGAGAAAGATCGAGA
22	PDGFB	AGATTGGCTTCTTCCGCACAA	CTCCGGGCTTTCGAGGAAA
22	SMARCB1	CCTCAAGGCCTGTTTGTCTGTT	CAGAGAACCTCGGAACATACGG
22	SMARCB1	GCTTTACTCATAGGTGGGAAACTACCT	AAACCCTCGTGAAGACGCA
22	SMARCB1	CTCCTATACTGACTGGGAGGACTT	CTCTTCTTGTCTCGGCCCAT
22	SMARCB1	GCTCCACAACCATCAACAGGAA	GCACAAGGTCAAAGCAGAGTG
22	SMARCB1	GCTGACTGTTGCTTCCATTTCA	TGTACCATTCATGTTCCAGGTGAAG
22	SMARCB1	CATGGAGATCGATGGGCAGAAG	CTGCGTGTCAGGTCCAGAAT
22	SMARCB1	GACCCAGTGATGTTTGTCTGTTACA	CTGGCCAGGAACGCATTAC
22	SMARCB1	CCCTCCTGATCCCTCGCA	CTACCTCGGAGCCGATCAT
22	SMARCB1	TGTGTCCTTTGGTTGTTGCCT	GTATGCGATGGTGGTGACAAAC
22	SMARCB1	ACCAGAGAAGTTTGCCCTGAAG	CTCAAAACAGTTTTGCAGGGAGATG
22	SMARCB1	TCTATAGCTGGAAAAGTCATTCCTCTCA	GGTCGCGGATCTTCTTCTCC
22	SMARCB1	AGACTCTGACAGACGCTGAGAT	CTGTCTCAGAGACCATGGGAGA
22	SMARCB1	CACTTGGCTGCCCTGTAGAG	CCAATCTTCTGAGATGCTCCGT
22	SMARCB1	GCATCCGGAGATGTTTGGCT	CAAACGTCAGCGGGTTCAAA
22	SMARCB1	AGAAATCCTCTGTGACGATCTGGA	CAGTGCTGGGTGAGAAGTCA
22	TIMP3	CCTGAGATGCTGTTCCTGATGT	AGCCAGGGTGATGACATTACCTA
22	TIMP3	GAGTCCATCAACTGCTGCCT	GCAGTAGCCGCCCTTCTG
22	TIMP3	TCCAAACACTACGCCTGCAT	CTGGGAAGAGTTAGTGTCCAAGG
22	TIMP3	AGCGGCAATGACCCCTT	CAGGAGCGCTTACCGATGT
22	TIMP3	GGGAAAGAAGAAGTCATGATGTTCCTT	CCTAGAAGCTAGAGTTGGCCATTAC
22	TIMP3	CTCTCCTTGTTTTCTTCTTTCCTCCT	CCAGTGACCCCAGAGCTTAC
X	AR	TCAACCCGTCAGTACCCAGA	CGAAGTAGAGCATCCTGGAGTT
X	AR	TGGCGATCCTTCACCAATGTC	AAGCTTCACTGTCACCCCATC
X	AR	GATAAATTCAAGTCTCTCTTCCTTCCCA	GGGCTGACATTCATAGCCTTCAA
X	AR	CAGAAGCTGACAGTGTCACACA	GCTAGAGAGCAAGGCTGCAAA
X	AR	GTGCTGGACACGACAACAAC	CCCTTATCTCATGCTCCCACTT
X	AR	GCTGGCTTTGTACCGGACTT	ACAACACTCACTACCCTAGAAACTGA
X	AR	AGCAAGCTCTTCTTGGAAAACCT	ACTCTTGAGAGAGGTGCCTCAT
X	AR	GTACAGCCAGTGTGTCCGA	GCAAAAGTGGTCCTCTCTGAATCT
X	AR	GGATGAGGAACAGCAACCTTCA	AGGGACAACGTGGATGGG
X	AR	ATTGCATGTACGCCCCACT	GCCTTCTAGCCCTTTGGTGTA
X	AR	CCTCCGGACGAGGATGACT	TTCGGATACTGCTTCCTGCTG
X	AR	CCGCCGCTTCCTCATCCT	GGCCGAGTGTAGCCGTA
X	AR	cgAGGCGGGAGCTGTAG	CCATTTCGCTTTTGACACAAGTGG
X	AR	AGTATTCCCCTTTCAAGGGAGGT	CCAGTGCTCCGGACTTGTAG
X	AR	GGGTAAGGGAAGTAGGTGGAA	GCCCGGGTTCTGGATCA
X	AR	CAGAGTGCCCTATCCCAGTC	AGGTAGGAGCCGCTAGATACC
X	AR	agcagGGTGAGGATGGTTCT	TCTGGGACGCAACCTCTCT

X	AR	CATGCAACTCCTTCAGCAACA	ACCGACACTGCCTTACACAAC
X	AR	TGCCGTCTACCCTGTCTCT	CCGTAGTCCAGCGGGTT
X	AR	GCTCGCATCAAGCTGGAG	CTTCGGCTGTGAAGAGAGTGT
X	AR	CCATTTCTGACAACGCCAAGGA	GCGGGTGGAACTCCCAAA
X	AR	ACTTCACTTGCCTATTTCTGCCATT	CTCCACAGATCAGGCAGGTC
X	AR	CACCTCCTTGTCAACCCTGTTT	CAGAAAGGATCTTGGGCACTTG
X	AR	CATTGACTATTACTTTCCACCCCAGAA	GTGCATGTGCAAGACCCTTTAC
X	AR	CCGGAAATGATGGCAGAGATCA	TGCAGAGGAGTAGTGCAGAGTTA
X	AR	CGAAGAAAGAGACTCTGGAAACTCA	CAGAGTCATCCCTGCTTCATAACATTT
X	AR	TGTCCATCTTGTCGTCTTCGG	CCCTTGGAAGCATCAAAGAAGAAAAT
X	AR	CCCATTCTGTCTTCATCCCACA	GAGCTTGGTGAGCTGGTAGAAG
X	AR	AATCCCACATCCTGCTCAAGAC	GGAATGGAAGCACCTCCCAAC
X	ATRX	GCATTTGCTAAAAACATTCAAATACACAA	CACTACTGCACAGTCAAGGAAGAA
X	ATRX	TCATCATTAAATTCTTCAGCCCACTTCT	TGTTCTTACCAGTAAGCAGAGCGATATA
X	ATRX	TCAACTTGCTTCTTTATGTCACTGTCT	GGCAAGAAGATGGTGGTGTTATGAT
X	ATRX	ACATTCCTTCCTTGAGCAAGATTTCTAT	CCACAAAAGGTTTCTGAATTAGCAACTG
X	ATRX	CTTCTCTCCTGAGGACGTTTCA	CCAGTGATGCATATTTCAGTGGGAATA
X	ATRX	CCTGACAATCAGCACCTTTAATTGG	AGCAGTGGAACTGAACAAGAAGT
X	ATRX	GATGAACTCTCCACTTCTTGTTCAGT	ACCTGCCAACACTTCTGAAGATTT
X	ATRX	CCTTTGAGTCAATTAGTCAGGTAAGTGA	TGTGGATGAAACAGGAAACAATCCTT
X	ATRX	AGGCAACATTCATTAGACTCACTTTCTT	AAAAAGATAGTAGCTCAAGTGGAAGTGG
X	ATRX	ACTTCAACATCATTGTCACTGCCA	TGCTGATTGACCTCTTATAATTGACGTT
X	ATRX	CTGGAACTGAGGAAGGAACAGAC	GCATCAGAATGTTCCAACAGAGGAA
X	ATRX	CACCACCGGTACTTTTATTTGTTCTTTG	ACCTTGTGCTTTGGAAAAGAAGGATATT
X	ATRX	GTAAGCACATCCGATTTTCCAATATGG	CAGTGCTGAATGAAGACAAAGATGATTT
X	ATRX	CTGTTTTCTTGAAAGTTTAGCTTCTGACT	CGATGGATGCTGTAAACAAAGAGAA
X	ATRX	TTCTGCTTCTAAATTCAGGCCCTTTA	GGCTTTATCTGAACTGAAGTCTTGTACT
X	ATRX	CGTGCTTTTGTTTCAAACTTAGCATCTA	GCTTTTAAGTCTGTGTTGGCTGA
X	ATRX	CGACTCAGACGGTGCCT	ACTCCAGTGCATTTCTATCGTAACC
X	ATRX	GCAATACCATTGGTTGTTTTCATCCAT	TTTCAGGTGGTGTGCGGAA
X	ATRX	GGGAGCTCACCCTCATCTC	CTCAAGAATTGCCAAGAAGATGCTTTTA
X	ATRX	TCATCATCTGAAGATCCATCCTCATCA	TGGTGTATTTCACCCCCAGATATATAGT
X	ATRX	CACTGAAATATGCATCACTGGttaaaaa	TGGATGAATGAATTTGAGAAGTGGCA
X	ATRX	CATCTTTTAATCCCTCTTGCCACTTCT	GGTGACAATCTGTTTTCTTATAGGTGGT

X	ATRX	TGAAATCCAGTTTGTCACACAAAAGAAC	TGGAAATTCTGGCCGTTTATTGTTTA
X	ATRX	CTTTTTGTCCCTTCAACATCAACCA	GATGCCGTTTCGAGAATTTTATTCACTT
X	ATRX	ATGACTATCCATCCCTCCATAGTAGG	ACGAAGAGAAGCAATCTACAATGATGT
X	ATRX	CAGTTGAGTTCTGTTAAGTCATTGATTCC	GTATGCAGCCACCACCATTAC
X	ATRX	ACGTCAGAATTCTTACCATCTGTTGTTT	AGTGGAAGGAGAACATGAATCTCTCA
X	ATRX	ACAGGATACCTTCATATTCTTCAGCTCTT	GGTGTTCAGTTTATGTGGGATTGCT
X	ATRX	TTCACAGACTCACAGCAGCAAT	TTTTAATGCTGTTTCTTAGAAGTTTTGGTTATG
X	ATRX	TTATTCAAATGCAAAACTGAAAAAGAACAAC	TCAGTGCAAGCTCTATCAGTACTACTTA
X	ATRX	TCATTCTCACAGCTAACACATATTCGT	AAGCAATGAGAACTTGTAGTTCATTCCT
X	ATRX	AAAATGCCATGTTTGGTCGTTTGT	AAATGATATTACTATGCAGAGCTTGCCA
X	ATRX	AGTAAAATTTCCTTAGTCCACGTTGGTT	GTGGAGCGTCATTTTACTATGAATGAG
X	ATRX	AGACGCATCTTCATTTACAGTTTCATCA	GGGTATTGTTCCAGTTGTCAGAAACAT
X	ATRX	GGTCATCTAATAAGTCTGGCTCAAAAGT	TCTTTTTACAAAGGGAACCATGGAAGAT
X	ATRX	TGCTTAGTTACTTGCCGATCATAAATCT	ACCATAAAGTTTTACCATTTCATATTAACCAGT
X	ATRX	GGGTGGAGGGTACAAATTGGAG	TCAAGGAAGAGAAAAAGTTGTAGAAGCA
X	ATRX	TCACTGCTGTCACACTGTTTGT	GGCAAATGGAAGGATTCCCTATACTTTA
X	ATRX	ccactgTCTGGTCCAATATGTTCT	TGCGGCACAAATTGACTGTG
X	ATRX	TCTTCTCCAGATTCTCCGTCACT	GGCATTTTAAAGGCTGAAGAAGGTTTTA
X	ATRX	TCTACTGAAAGAGCGGGAAAGA	CGTGCTCACATTCTCTATGAGATGT
X	ATRX	CATATTTGTACCTGAACACATCCAGCTA	ACTTGGATCCATTAAGGAGTTCAGGA
X	ATRX	TCCTGAACTCCTTAATGGATCCAAGT	TGCATGTGTTTTTGACATGAGCATTT
X	ATRX	CAACTTTGTTTCCCTCTCTGTAATACAC	TCGCTTTGGACAAACTAAGCCT
X	ATRX	AGGCTTAGTTTGTCCAAAGCGATAAA	CTAAAGCAGGATCTCTAGGAATTAATCTGG
X	ATRX	ATGGATTCCAAGAAGCGTCGAA	GCATTTATTCTATATTTTCATTTTCAGAGGACG
X	ATRX	AGCTGCCTCACACCCAAATAT	AGATGAAGATGAAGAAACCAAAGAACCTT
X	ATRX	ACCTGCACTAAAGGTTCTTTGGTT	CCATTGCTTGAATGATTTCTTTGAGCTT
X	ATRX	GGCAGGCACTCCTGGTTTTTA	GCCTTATCTAAATCAGTGCCTGTCA
X	ATRX	TGTCGTCATCATCATCATCCACTG	CCAGAGTTTAGACCCCACTTAGTTTG
X	ATRX	ACAATGAATGAATGGAACAGAGAGGTAAC	GGAAGAAAATCAGCGGAGCTATAAACA
X	ATRX	AACCTTAATACGTCGCCTTTTCTTTTTC	GGAATGCATACAGAGGTAGAACAGTTTT
X	ATRX	GTCTAAGTTTATTCTGCTTCCAATAGATGCT	CAGGTGCAAAGCTTTTCCAAGATT
X	ATRX	TGTAGTCTAGCTGCAAACACCAAG	TCTTTTGATCAGGTGTGGGCAAT

X	ATRX	ACATACATGTGTCCAGAAGCAATCTAA	GTGGTTCTGGAAGTAACTCTGATATGATG
X	ATRX	TCCTTACCTCTTCCTTGCTGTTTTC	GGGTTTGTGGAGTTATAGGTATTGATGTT
X	ATRX	GCTGCTCTCTGAATAAAATAAATGGTTACCT	TCTCCTCCACGACTTGCAATG
X	ATRX	AGATTCTTCTGATGAGTGTGCAAGG	TGGCTATATTTTCAGTTTGCTAGAGCTT
X	ATRX	GTTACCTCTCTCAATTTTTCTCGCTCA	AAAGGCAGAAAGAAAATTCGGAAGATTC
X	ATRX	GCTCCCTCTCAGCAATACGTTTT	CAAGTCTCCTGGAAAAGGCAGA
X	ATRX	ATGACCAAATAAATCACATCTTTAGGAAGGA	CTTCGAATGGCAGAGGAAATTGG
X	ATRX	GCACTTACACTTTATCCCCAATTTCC	GCTCCAGACTGGTACAAAGATTTTGTT
X	ATRX	AAACCTCAGCATCAGCATCTGT	TCTCTTGCTCTCTTTCCATTTCTTTTCT
X	ATRX	CAAATAAGAACTTGCAATGAAGGGTGT	ATGTGTGAATAATAGCCACTCCTTTCTC
X	ATRX	TCATTTGAAGGCATGGTCATTCAGA	TCAGGAATCAGGAGTTAGTGAAGAAGT
X	ATRX	CGCTGTTCATCTTCGGATTCACTA	TCTGTTTTAATGTCGAGTCAGTGGTAAA
X	ATRX	TCAATTAGGTTATTTTGAAGTGGTGTTCCT	TGTTGTTTGTGATGAAGGCCATATTCTA
X	ATRX	GCTTTAGAAACAGCAGATGCTTCATTTT	CCTGTAAGATCAGCACTTTAAAGGGATT
X	ATRX	CTAAGTAGTAACCCTAAATTTGATTAGCTTCA	TGTCTTCACATACTGGATTTTGCCAAT
X	ATRX	TTGCATACCTGAAGATTGGCAAA	CTGACATTACATCCTCATCTTCTTCTGA
X	ATRX	AGTATCAGATGATGAACAATCTTGTCTC	AGGGAAAGGAGATAGTTGTGACTCTT
X	ATRX	GAAGTAAAACTCTGCTCTTTCCCAGAA	TGATGATGCTGAAAGAAAACAAGAGAGA
X	ATRX	TTCTTCTCTCTACCATATGCTCCATTCT	TGGAACAACTGATGGAGAAAAGAAAAGT
X	ATRX	CCTGTTGACTTCTCAGCATAATCAGATA	CTTCATCTGATGGCACTGAAAAGTTAC
X	ATRX	GGAAAATGACAAATTTCTTCTCGCTCA	AGAGCGATGAAACTTCTGAAGATGAT
X	ATRX	CGCCTGAGTCTTTAAATCATACAAAGTC	AGATTCTGATGAAATGCTAGCAATCCT
X	ATRX	CTCTTAATTCCATGATGGTCGTGTCTT	CTCTTCTGAAGATGAGAAACACAGCA
X	ATRX	GACTCATCCTGCTCACCTCTTT	AGGATAAGCGTAATTCTTCTGACAGTG
X	ATRX	CTATAGCACTGTCAGAAGAATTACGCT	AGATCCCCACGTGTAAAGACTACA
X	ATRX	CAGTTCCCTTTTTGCTCTGCTTTTT	GCAAGCATCTCAAAACCAAAACATGTA
X	ATRX	GCTTCCCTCACCTATAGAATTCTGATCAT	AAAGAAGGCAGTCATTGTCAAGGA
X	ATRX	TTCTGTCGGTCGCCTCAAG	TTGTGGTCTGAACCCCAAGTT
X	ATRX	GCTTGTTCTTAGGGAGTTTCTCTTTTTC	AAGGAAATACAAAGTGGCTCATCATCA
X	ATRX	AGCTCTTAGCTGATTTGCCCTT	GATGAGTCACAGTTCTTCTTCAGATACT
X	ATRX	GCAATATCAGATAAGCCATCCTGTACTT	GCTTCCACTGATGGTGTCGATA
X	ATRX	GATGATGATGAGCCACTTTGTATTTCC	GGAAGAGACAAGATTGTTCATCATCTGA

X	ATRX	CCTGTCCAAGTCCACATTTCTCT	TCACTCCTGTTTCCCTTTCTAATTCC
X	ATRX	ATGATGAGCCACTTTGTATTTCCTTAGT	CTTGGAAAGAGTTCAAGGAAGAGACA
X	ATRX	TTCCAATGCAAGATGAGCCTTCTTAATA	AGCCAACATGAACTCCAGTTATGT
X	ATRX	GATTTCTGAATTATCTGTTGCCTGCTT	GAAATTAGATGATTCCTGTTCTGGCTCT
X	ATRX	AGTGCGGAATAAGAGTAGGTTACAGA	CAGTTGTTGCAGCAAAATAAGAAGAAGAT
X	ATRX	GAGCCAGAACAGGAATCATCTAATTTCT	GAGAATTTAGAACAGTTGTTGCAGCAAA
X	ATRX	TCTTCTTCTTATTTTGCTGCAACAACTG	GGTCGAAAGGAGTTGTCCACAATA
X	ATRX	TGATAGAGTTGTGAGACTGGGTAGTT	TGAATTAGCTAGTAGGGAGAAGACAGAAG
X	ATRX	CGACACAGAAATATTTGTAGCTCACCTTT	TTGTTTTCAGCCAGTCCCTCAT
X	ATRX	GTCCTGGATTTTTGCTTCTCATTGG	CCCCCAAATTTGATCATGAATCCTTCT
X	ATRX	TCCTCTCATATCAATCTGCTGGTAGTT	GCTCCAGCAGCAGTACAATCAG
X	ATRX	TGATAAGTCATTTGTTGCTGTTGCTG	TTTTGAGACAGGTCATGCACTTGTA
X	ATRX	ACCCTTTTCTTCTTCATTGCTCTTTCA	GAACATGATTCTCTTTTGGACCACAAAG
X	ATRX	GCTGCTTTTCTTTCTTCTTCAGTCAA	TTGTCTCTTATTTCAACCCCTCTAAACAG
X	ATRX	GGAGAATGTGATATTTCTGCATAGGGAA	GAGCCCTGTCAGCAATGAGTA
X	ATRX	CAACTCACCTCCAGCTGTTGAT	GGGACTGACCATGCGTTTCAA
X	ATRX	GGGTAAATTGGTCCCAGTTGGTATG	CCTGGATCTGAGAATGTGGATTCTTTT
X	ATRX	caCATTCAGATTGTTAGAAAAGCATAGG	CCTCAGATTCTGATGAAACCTCCA
X	ATRX	AGCTTAAACTCATGGAGGTTTCATCA	ctCAGCTCGTGCCAATTAACTTATTC
X	BTK	GCTGAATTTGAAAGAACTTAGGTCCATT	GTGCACAAACTCTCCTACTATGAGT
X	BTK	AACTTACCCCACGTTCAAAGTCAT	ACAGGTGAACTCCAGAAAGAAGAAG
X	BTK	TCAGTCTTTGGTGGCTGAATGG	CTGCTGAACACATTGCCCAAG
X	BTK	TGAGGCCTGTAGAGACGTAGG	ACTAGGACCCCTGCTATCCAAA
X	BTK	atgaaAGCAAGAACAATATCTCTGTGGA	CAAGTTCAGCAGCAAATCTGACAT
X	BTK	AATCTTATCCACTTACCAAAAGCCCAA	TCCTAATGCAACAAGTCCTGAATCC
X	BTK	ACAGACCCTTGGGAGAGTGAT	CTGCTCTCAGACAGCCAAAAATG
X	BTK	CTCCAAAATTTGGCAGCCCATAG	TGATCTCTCTCTTCTTTCCTGCTACA
X	BTK	GAGAGAGAGAGTTCCTCCTGGAA	ACTGCCACATTGTTTCCTTCACA
X	BTK	CACCGTTTTTGAGCTGGTGAAT	CTGAATCCTAACTGCTGAAGTCTGT
X	BTK	GCATCAAGGAGCTATTAGGAGGGT	AGACAGCTTCTTTTTCGTTGTTTCAG
X	BTK	GTCATCTTTAAGCCTCAGTGGGT	ACGTGGCCATCAAGATGATCAA
X	BTK	CATCTTCAGACATGGAGCCTTCT	CCAAAGAATCACACCAAGACTTTATTGT
X	BTK	CAAGGCCAGCAAAGGTAGTTT	GCATGACCTCTCTCTCTGTTTCA

X	BTK	CACCACCCCTTCTAATTGTGTTACA	AGCAATGCATCAACCAATAACCATTTTT
X	BTK	GAGGATTAAAACTGTAACACCTACCCA	TCACTGGTCTCTGTTTGCACTAC
X	BTK	CAAATCTGCTGTTCCCCATCTCA	CTCCATTTTCTTCCTCATTCTACTTCTCA
X	BTK	GCTTCTACCCAGTATCACAGGGATT	GAGCTCATTAACTACCATCAGCACA
X	BTK	CCTGGTACTCACCTGCAGAGT	AAGTTACTGACTAAGCATCCACTTCTTC
X	BTK	AGACTCTAGTGTGTTCTTAGGGCTT	CCATATCATCACTGGCTTCTTGTTTTG
X	BTK	GGGCTTGTGGAGAAGAGAAGT	TCTGGATTCTAGCCACTCTAACACTTTA
X	BTK	TCCTAGGCCAATCCTTCTAAGGT	CTGAGTACATGGCCAATGGCT
X	BTK	GCATCTCCCTCAGGTAGTTCAG	GACTAGTTCCTTGCCTTTCCTGTAG
X	BTK	AGTACTTGGAGGGAGAGCATGT	CATGGTGGAGAGCACGAGATA
X	BTK	TGGTGTGGACTCACCCATTTT	GCCCTTTATGATTACATGCCAATGAATG
X	BTK	CTTCCGCAGCTGTAGATCATTTG	CCCTCCTACCTTTTCTCCTAACTACATA
X	BTK	CAGAACAGGCCCTCAGTTCAA	CTTATGACCAGGAGCCACTCAAG
X	FAM123B	GGTCTCTGCAGTGTCGAGAG	CTACTATCACAAACATGCCTTCAACAA
X	FAM123B	GGCCTTGGTAGAATCGACTATGGTA	GGGAAGGCAAGTGTGAAGAGAA
X	FAM123B	GATCTTCATCATTGTGGAACTCAGGA	GCAGCTCTGACTCTGACTCAT
X	FAM123B	GCTCAGGGAGGTTTTGAGTGAAAG	ACCCTACTTATTCACCCCCTGAAG
X	FAM123B	CTCAACCTCCTCTTCCTCTGGAT	GTTATGAAGGAACCTGCTCCAAGA
X	FAM123B	CCCGTGTCCCTACTCCAGAAT	CTCAGCCCACTCACTATGGG
X	FAM123B	TTGACAGGTCAAGGCTGGAAG	CCTGTTGATGAGCCCAGTTG
X	FAM123B	CGAAGCTCCCAATACAACAACTGT	CTGGAGATGACTGCCTTTATGACC
X	FAM123B	TCTCAGAGCTTCGACCATGGA	CTGGATTTGAGGATGATTCAGGTGA
X	FAM123B	AGACAATCCCTGCGGACAAG	CCAGTTAGGTCTTATCCTGGCCTA
X	FAM123B	GGTCACTCTGAGGAGTCAAAAGT	CCAAATGTATCCACGGCCCAA
X	FAM123B	GGGATGGTAGCCCAGGTTCATA	CCTTGCCAGATGATGATGACgag
X	FAM123B	GCAGTTTCCCACAGATATTCTAAGTCAT	GTTCCTGCCTGGTGACCTAC
X	FAM123B	GAAGCCTCCAGAACTGGAAGAG	CTAGCTCGTCCCTCACACCTA
X	FAM123B	GAGGTTATAGCAAGGGCCCAT	CCTTCCAGCCAGTCTCCATATAG
X	FAM123B	GCATCAGGCAGGTACTTTGATCTT	GGGTATCAGGGACCTCTCAGA
X	FAM123B	GTCATAGGAGGTATGCAACAGGTT	CAGTCCCCTTTCCCTCTATACTGA
X	FAM123B	GGTAATTCCCCGGTGGGAAA	CCCGAGAGGTTCGTTGTAGAG
X	FAM123B	CCCAAGCAGGCCAATCATAGG	CTACCTCGAGTCTGATGAGCTG
X	FAM123B	GGGTCTCTCGGACTTGAGTCT	GCCTATACTCGAGAGGCTTATGG
X	FAM123B	CTTGGAAGGTCTCCTCAAAGCA	TTAGCAGTATCCGCCGTCAC
X	FAM123B	CGTCCTCCTCATCTGAATCTTCCT	CTATGGCCCTCAACAGGAGAA

X	FAM123B	CCTGGCATAGGCTTCCCT	ACGGCTAGTGACCATCCAGAA
X	FAM123B	CCCAGTGACCTTGCTCTTCC	AGACAGGCTCCAGATGTAAGACAT
X	FAM123B	CCCCTCCAAAGAAACTAGGCA	GGGAGTACCCGTGAACAAACAG
X	FAM123B	CATCATCTGGCAAGGCCATCT	GCTTTGATTCATTGACAGGTTGTGG
X	FAM123B	AAACTTCTCAGCCACAGCTTTCT	CATGGCCCTGAAGATGTTGTC
X	FAM123B	CCATGTCCTGTTCTGCTATTATGTCA	CCTAGAGGAGCCCCATAGCC
X	FAM123B	CCTTGTTCTTGGCTCCTTTTTCT	GCTGGACGTACCTCAGTTGTC
X	FAM123B	GGAGAAGCCAGTTCCTTCACT	GTGGCAAGAAGGGTATCTGTACTC
X	FAM123B	CTACTACCTTCTCCCCTGTTTCTG	AGAACCTTCTCCACCAGCTACT
X	FAM123B	TTTCTGGATCTTTACAGGCCATTTTCT	GCTCAGCCCCTCAGGTG
X	G6PD	GCATGAGGTAGCTCCACCCT	GAGGCCTGGCGTATTTTCAC
X	G6PD	GCCCTGCAACAATTAGTTGGAA	CCATCAGTCGGATACACACATATTCA TC
X	G6PD	CTTGGGCTTCTCCAGCTCAAT	CCTGCATACCTGTGGGCTATG
X	G6PD	CTCACCGATGCACCCATGAT	CTTGTTAACGAGCCTTTCTTCCAC
X	G6PD	GCAGTGGTGGGACACACTTA	TGGAGGATGATGTATGTAGGTCGT
X	G6PD	CTCGATTCTGCTCGGTTCTCA	CCCCGGAAACGGTCGTA
X	G6PD	GGAATGTGCAGCTGAGGTCAAT	GAGCTGATGAAGAGAGTGGGT
X	G6PD	TGTAGGTGCCCTCATACTGGAA	TTTATGGCAGGTGAGGAAAGGG
X	G6PD	GGACACGCTCATAGAGTGGTG	CCCCCGACCGTCTACGA
X	G6PD	GGACTCGTGAATGTTCTTGGTGA	TTTGCCCGCAACTCCTATGT
X	G6PD	GGAGGCTGCATCATCGTACT	GGGCTGACATCTGTCTGTGTG
X	G6PD	CTCCCCCAAGATAGGGAAGAGT	AACATCGCCTGCGTTATCCT
X	G6PD	CCAAAGGGCTCCTTGAAGGT	CCTCCAGAACTCAGACAAGGG
X	G6PD	ACCCTCCACACTGCTCCTT	TGGTGATCCGCGTGCAG
X	G6PD	GGTGTACACGGCCTCGTT	GCCCTTTCCGCCACGTA
X	G6PD	GCTGGTAGAGAGGGCAGAAC	GGCCTTCTGCCCGAAAACA
X	G6PD	ACCCCATAGCCCACAGGTAT	CAAGACACTCTCTCCCTCACAG
X	G6PD	GGCATAGCCCACGATGAAGG	ACCCCACCATCTGGTAAGTGT
X	G6PD	GGCCTCGAAGGCATCACC	TGTGGTCCTGGGCCAGTA
X	G6PD	CGTCCAGGTACCCTTTGGTG	CATTCTCTCCCTTGGCTTTCTCT
X	G6PD	GGTGAGGCTCCTGAGTACCA	GCTGTCCAACCACATCTCCTC
X	G6PD	AGATCTGGTCCTCACGGAACA	GATGCAGCTCTGATCCTCACT
X	G6PD	CTCAGTGCCTCGTCACAGAT	ACCTACTGCAGATGCTGTGTC
X	G6PD	GACGTCATCTGAGTTGGTGGA	GCCCTTGAACCAGGTGAACAG
X	GATA1	CCTGTGGAAAAGCTGGGAACTT	CTGGGATCCCCTCCATACAGT
X	GATA1	CTTTCAGGTGTACCCATTGCTCA	GAAGCTGGTGCTGCCTTTTC
X	GATA1	GGCCGTGGAAGATCTGGATG	CGGTGGGAGAAAAGAAGGTACT
X	GATA1	CCCTGTCCCCAATAGTGCTTATG	CACCTCAACTTCCCTGTCCTTTTT
X	GATA1	AGCACCCAAAAATTATCTTACCCTGA	CTTGCGGTTTCGAGTCTGAATAC
X	GATA1	CACTGACCATGCGGAAGGAT	TCCCCACAATTCCCGCTAC
X	GATA1	CTGGTGGCTTTATGGTGGTG	AAGGCATGAGGTGGCTAACAG
X	GATA1	GCCCTGTGGTGCTGTCA	TCCTCTGGAGGCCATGCT
X	GATA1	CATCCCCAGGGCACTGAT	GGGCGTCACCTGGTGTA

X	GATA1	CCTTCTGTCCTCGCAGGTTA	GCTGCATCCAAGCCCTCAG
X	GATA1	GTGTCCTCCACACCAGAATCA	AAGACAGCCACTCAATGGAGTTAC
X	GATA1	GGTGGCCCAAAGTAAAGCTG	TTCTGCCCATTCATCTTGTGATAGAG
X	GATA1	ACAGGCCACTACCTATGCAAC	TCCTATATAATGGGAAGATGTGGCTTCT
X	KDM5C	GGTGCTTGACAAAAACCTGTTCT	GCCTCTTTGAGCCATTTCTTCTTCA
X	KDM5C	AGGACTACATGGGTCACTATGGT	CCCCTACCTATTCTTGCTCTCCTC
X	KDM5C	CCTGCCCACACACACAGATAG	ACTTTGCTCCTGTCCTGGGTA
X	KDM5C	CTTCCTCCAGAAAGGCCCAT	GCCAGTGTATCAAGTGCAAGAC
X	KDM5C	GGGCTGACAGGAAACACGTA	GGATTTTGCTACCTGGTTCTGGTTATAT
X	KDM5C	TCCCCGAACTTCCACCAGAATA	CTTATCTGCAAGATGGCTGCCT
X	KDM5C	CAGGTTCAGGTCTAGCTTCTCTG	TTGGCCACACTCAACCTTGA
X	KDM5C	GGGAATGCTTATTGAAGGGACAA	GCCTAAGACCTTCCTGGAGAG
X	KDM5C	CTGTGACTCAGCTCCTCCTTG	CCCTAAGTGTCCTAGACCTGTGTTTA
X	KDM5C	CCTCTTCTGGGTCTCCACTCAA	CCTAGCCCTGCTGTGGATAAAG
X	KDM5C	GTTCAGCAATGGTCAGCAGTTC	GGATGAGGTGAAACGCACACT
X	KDM5C	CGCATGACAGCCAAGGTG	CCTCCAGTCCAGGGCTACT
X	KDM5C	CCCCTCTCCAACAGGGACT	GCCCAGCTGAGGAGTTTGT
X	KDM5C	CTGCCCTCAATAAGGCCAGAT	GTGTGATGGCTGTGATGACAAC
X	KDM5C	CTCATCAGCACTCCAAGCGT	GAGTTTGGACCTGAAACCTCACAT
X	KDM5C	CAGCAGGCAGAAGATGTGGTA	GATTTCCAATGGAGAACTTTTGCTCTT
X	KDM5C	CAGGAGCTGAGGTCTGAACAAT	TGACCATCAAGCCTGCTTCTT
X	KDM5C	GCAGGCTTGATGGTCAGAAAGA	CCCTTTCTCCACTCTGACTCC
X	KDM5C	CCACTGTCACAACTGTTGCT	CACCCATCCCCACCACTG
X	KDM5C	CGGTTCCAAGCCATTCTGGTT	GGAGCTAGAGCCAAAGAGGGTA
X	KDM5C	TGGCCCTGAGCTCCGTA	TGTCTTTCTGCCTGTCTGTAATCAC
X	KDM5C	CACTACCTCAGCCCTCCATC	TGACATGTTCTCCTTTGGGTTTCA
X	KDM5C	GGGTCAGAGTACAGAAGAAAGGG	CTTCCTGCCATGCTGCATAAG
X	KDM5C	GACTCAGCCCGAACCTTCAG	CATGGGCCTTTCTGGAGGAA
X	KDM5C	acgtaacCATGAATCATCCACTCAC	GGTAAATAGCATTGAGGAAGATGTGACT
X	KDM5C	AATGGATGTCAGCTCCATACTCAAC	GCCATAGTCATCTGCTACCTGTCT
X	KDM5C	CTGCCATATGCTGTGGTTCTC	GCTTGTTCCTGCAACCTCCTA
X	KDM5C	CGTTAAGAGACGCTGTAGGTCAA	GGAGGAGCTGAGACAGCTAGAG
X	KDM5C	GTGCGCTGTCAGTACCTGTAG	TGGCAAGTTGAACTGAGCTGT
X	KDM5C	GACAGGTTGTGGGCCAGT	GCTCGAGGTGACCCTGGAT
X	KDM5C	agctCAGACACTCTATCATCACCAA	CAGCCGGTGGATGGAGAAG
X	KDM5C	TCAGATTTGTACAACCCCAGCTC	GATGTAAGGGAAGCCCAGTCAT
X	KDM5C	CACTGACTTGATTCCCTGGCT	CAGTCTGTACTGTGCCACATCAAT
X	KDM5C	GGCACCTTCATGCCAGAGATAT	TCTCAGTTCCTTAGCATAACCCTCA

X	KDM5C	CCCAGCCTGTTAGGCCTTA	TCTTGATTCATTTCCTTCTTTCCTTCC A
X	KDM5C	cCACCACCAGCTCCTAGTCTT	TTTCCCCTCTCTACCCTCTCAG
X	KDM5C	CCACCATCCCACCACATTCTA	CCATGGCCTTTTGTGTTCTTCAG
X	KDM5C	GGGCTAGGAGACAAGAATCTGA	ACGGCTACAGGCTGAACCTA
X	KDM5C	GTAGTTAGGAGGCTCCTCAGGT	CTCACAGAGAGGGCCATCAG
X	KDM5C	CCAAAAGAGCAGTCACATCTTCAGA	CATCCTGGCACTGCTGGTAG
X	KDM5C	CCGCACAGGCAGTCTCT	GCCTCCTCAGCTCTCCGA
X	KDM5C	GGGATGAGGTGGGATTGGG	AGCACGGCCTCCTCTACA
X	KDM5C	GCTGGCTATCCTACTGCTTCAT	GAGTTCCGAGACCCTCTTGG
X	KDM5C	GGGCCTGATTTTCGCGATGTA	AGTCCTTAAGGGCGGTCCA
X	KDM5C	GCACCTTATGTGTGCCCTAACT	GAGGTTTCCTAATAGTGAGCTGCTG
X	KDM5C	ACTCAGGCAGTTCTTTAGTTGCTG	CTCTATTCAATACTGCCTACTCTTTGC T
X	KDM5C	TCATTAAGAGCAAGTGTGGAAATCTG T	CCTCAACTATCCACCAGGCAAAAA
X	KDM5C	GAGCGTAGCAAGGAGCCAATA	CACATCCATGTCTTTTCCTACTTGTTT T
X	KDM5C	CACCTCAAAGCTCTAAGTTCGAAGA	GTGTAACACACGTCCATTTGATAATG AG
X	KDM5C	GGGTTTGTATTCCTTGTCCTTCTC	GAGGGTAGAGGGAAGTTATTGCTTTT
X	KDM5C	CACCAGCCCATCCATCTATCTG	CTTGACTTTTGTATTCTGACTCCAAGG T
X	KDM5C	ACCACCATCACAAAGGACATCA	GATGAAGAAGCTGACACCTGAACT
X	KDM5C	ACCCAGCACATCCCAGTGTATAT	GCGGAAAACATCCCTGTTCAC
X	KDM5C	GAGGTCAGGCTGGCTATCAAAT	GTCCCTTTTCCACTTGTGCAG
X	KDM5C	CCTTGAGAGCCTGGATGTTGG	CCACATCAGACTGAGCGTTCTT
X	KDM6A	GAAGTCATCATAAAGACCACTCAGAT AG	TCCTCCTCCTCCCAGAACTTAAAAA
X	KDM6A	AAGGCATGTTTCTAATACTGTGTCTCT TT	GCACAGGATAACTCTTTGCATCAGT
X	KDM6A	GGAACTGGATACAGTGCCGTAA	CATTGTTAGCTTCCACCAAAGTTTTAG T
X	KDM6A	GTATTCATGAAGACCTGGGACTTTTCT	CCAGATTTTCTTTGTTCCAGTGGGAT
X	KDM6A	GTTGTTGCAGCCAGCAGATG	CTTACTCTCAATGATTCCTGGAATGA GG
X	KDM6A	ACCAGGCCTCCTCATTCCA	TGAGATGCATATAAAAGTAATGTGTC CTTTCA
X	KDM6A	CTTCCCTATAGATTACACAACCAGCA T	CCAGAGACTGAGTTTGTAGGCA
X	KDM6A	GCCAACAGCTGACTCATCAC	GTTCTTGATGTGCTACAGGGAACAT
X	KDM6A	CTTTGGCCAATGGACCCTTTTC	GTAGAGTGAGTGCGTTTCGCT
X	KDM6A	TGGAAGTAATGGAAACGTGCCTTA	AAAACATTTATGTGAAAAGCCAACAA GGA
X	KDM6A	CCACAGGTATTTGTAGCAGAGTTTCA	CCAATATGGTATGACCAACATGGCTT A
X	KDM6A	GTGTCGTATCAGCAGGAAATCTTC	GGCTGTCTTTGCATGTTTCATATTTTG AT
X	KDM6A	TGGACTTGTGCAAATGCCTAGT	GGACATTTATCCAACAAAATGCTACT GT
X	KDM6A	TTTCCAGGAATCTAGGTAAAAATGGC TT	ACGTAAATACTAGGTGTAGGTGGATT CA
X	KDM6A	CTCCCTTGCCAAAGGACAAGT	AATGTTTCCTAAAGGGCATCCATGTA A

X	KDM6A	TTTCTTCCAGTCTTACTAGGAGCTTCT	CCAAATCTCCAGGTCGCTGAATA
X	KDM6A	TGAAGCAAATGTTCCAGTGTATAGGTT	CCTGTAAGTGGACCAACATTCCAAG
X	KDM6A	CTATTGGCTGGTGCAACAACATT	ATTCTATGCAAGGAGTCATTCTTCTTAC
X	KDM6A	CACCTGAGCAGGTGATAATGGT	ACACTTTGCAATTTGTTCCATTCGTA
X	KDM6A	CAGTATAAATTGGCAGTGGAACGG	AGAGGGACTACAGCAGTTTTAGAAAAG
X	KDM6A	TTGTCTTTCCTCATCCTTTGCAGT	CGCCCAGAACTCCATTAAATTATCTGTA
X	KDM6A	GTGTTTGACCAGATAGTGGTTCTGA	GCATCAGTTTCTGTGCTGGATTTAAATT
X	KDM6A	TCTTCTTCTAGCATTTGGAACAGCTC	AGGGAAAAATATGCTATACACACTTGGT
X	KDM6A	GGAAAGTCAGTTTGTCTTAATGCAACA	TCTGTCAAATACCAGTAGAAAGTAAAAATGAAC
X	KDM6A	TCTTTTCACATTTCAGGTCATCAGGAA	TTGGAAACCTACTTTTCACAGAAGTCAT
X	KDM6A	GTTTGTTGTTCCTGAAGGTTACTGG	TTCTTCGCTGAATGGTAAGTGAAT
X	KDM6A	cTTTCGGTGATGAGGAAAAGAAAATGG	CGGGAGAGCGGCTACTG
X	KDM6A	CATAGTCATTTGGCCTCCTCTAACC	GTGTCAAACACCATGAATGAGCTT
X	KDM6A	TCTTAAGATGCTTTTGTGTGACTCTAAA	CCATGAAGTGATCGGAATACATGTAGAA
X	KDM6A	GGGCTTCACAAAGGTCAGAG	CCCCCTGTGTTACTGAATTGCTA
X	KDM6A	CAGAATCAGAACGGACATCCCA	GCACCGTCAATATGTTTCCTGAAG
X	KDM6A	GGCATTACCTTAACCAAAGAGAGCAA	ACTTAGAATCTCCATGGCTAGGACT
X	KDM6A	TCACTGTCCACAATTTCATTCAAGTAGAA	GCAATGTACTATGTAGGTCTTTCGTGAA
X	KDM6A	GATGACGGCAGATGCTGTTTG	ACAGGTTCCAGTACCCACATTG
X	KDM6A	TGATCTGCTTTTTGTCACTAATGAGAGT	CAGGTCCTCCATTTTGTACTGTTCT
X	KDM6A	GAAACTTGGAAAACTTTGTGGTGCT	GCAAACACTGCTGCTTCATAACC
X	KDM6A	CCTTGTTGATGGGAAAAGCCAATAA	GTGGTTGTCTGCTCAGTGGAT
X	KDM6A	GCATTTAGGAAGATTTAGTGGACTTGCT	CGGGAATTGGTAGGCTCCA
X	KDM6A	CCTAGTATTGAGGAGGCGTGGA	GCTTGACCACTAGAGAATTGAAAAGAAC
X	KDM6A	AAGTCTCTTGGCATTATTTCTAGTTGGT	CAATGAGAGCTTCCCTCAATGTCT
X	KDM6A	TGTCTTCTAAGAACTCTGAAGCAATGTC	AGGGAAGAGGCTTTATAAAACAACTGT
X	KDM6A	TCTTTTTCCCTTCCTTTACAGAATGCT	ACAAGCAAACAAGCCATTTACTGAAAAT
X	KDM6A	GGGTGTGGTGGGAATCTTGTTA	CATGAAATCAGCATGCAAATGTACACT
X	KDM6A	TCTTAGGTGATCGAATGGAGGCTAT	AAAGAAATCAGACTCCACTTTTCCTTCA
X	KDM6A	GTGGAGTCTGATTTCTTTTGTCAATTAGG	TTTGCTCATGCACTTACAATTTCTTAGG
X	KDM6A	TTTTGGTTTGTTTTCTGCTTCGTATTCTT	AGCTTTCCTTGGTGGCTTTATCTC
X	KDM6A	TCATTTACTGTAGGTTGGATGCATCAC	CAGGACTGGCCAGAATTAGGAT

X	KDM6A	GGAAAGCTATGCTATTCAGTATCTCCAA	ATCTCTTAGCTGGAATAGACAAAATCAAACA
X	KDM6A	GAGTTATTTCCCCTAACTTCACAAGCA	TTCCTGAATAGCAGAAAAGGTCTCATTT
X	KDM6A	GTTCCCTGAGCGTTAACGAGTAAA	CGACTCGCAGCTGCCTTAC
X	KDM6A	TGATATATAGTCCATCCTTTCAGCCGAT	AGGCATCCTGAACTTTCCCAATAC
X	KDM6A	CCTTCCCTTCTCAGGTGCTATTC	ACAAAGGCTTACCCTATTGAACACC
X	KDM6A	AGGCAGTCTATTGATAAATCAGAAGCAA	CTATGAAGTACTATATGTGATGCAACCATCT
X	KDM6A	GGTGTCGGCGTTGGAGT	CTCGCCGCTCGCTTTTC
X	KDM6A	ACAGAAGTTTCAATGTACTACCAAGCA	AAAAGCTGGGATCAACATAAAGCAC
X	KDM6A	CAGGGCAATTAAAGCATTTCAGGAG	CCAACAACCTACCTTTAAACTAGACTCA
X	KDM6A	CATTTACGACTTGGGCTTATGTTCAAAG	TTCTTTTAAAGTGCCAATATAAATTCCAACATT
X	KDM6A	TCAACAGCAACACCTTCTCCAAA	GATCTGTGGACTAGGTTTGTGGTTA
X	KDM6A	CCATGAAAACAGATCTGCTTCTGGT	TCACTTTGATGAACTTTCCCACACT
X	KDM6A	AACCAAGCAGTTCTTCTGAGTTGA	CATGGTCCAATTGTACAGCACAAATA
X	KDM6A	CCATGGATGCTTTACAGGCCTA	GCAAGTGCAGAGGTATTACTACAACT
X	KDM6A	CCTCAGGATGCCATTAAATGCTACTTAA	CCTCCTTCTGCCTGAGTGTTC
X	KDM6A	AGAGGAAATATCATTCTGCAAAAGAAGCT	TGCCACACTCGACATGGAAAA
X	KDM6A	CTCTGTTTTCCTGAGATCTAACCACAT	CAGCAAGGCCACGTATTACTGTA
X	KDM6A	TGTACAAATCCGAACAACCCTGT	TCTGCCAGTGCTGGAAAAGTTAA
X	MAGEA1	GCTTCCTCAGGCTTGCAGT	AGAGTCTCCAAGGTTCAGTTCTCA
X	MAGEA1	GGCCTTGGCTGCAACTC	TGAAACCAGCTATGTGAAAGTCCTT
X	MAGEA1	AGCAGGCCATCATAGGAGAGA	ACAAGCACTGTTTTCCTGAGATCTT
X	MAGEA1	CTCTTGCACTGACCTTGATCACATA	GGAAGCTGCTCACCCAAGATTT
X	MAGEA1	CTGCAAGGACTCAGAGGCTTT	TCCTTGTTCCGAGCAGTAATCAC
X	MAGEA1	TGCCGGTACTCCAGGTACT	GATAATTGTCCTGGTCATGATTGCAATG
X	MAGEA1	CCAACCAAATCAGCCACCTTCT	CTTTCCCACTACCATCAACTTCACT
X	MAGEA1	CAGATTTCCTCCTCAGGAGCAT	CCTATGTCCTTGTCACCTGCCTA
X	MAGEA1	CTCACTGGGTTGCCTCTGT	CCTCCTCCTCTCCTCTGGTC
X	MAGEA1	GGATCTGTTGACCCAGCAGTG	ATGTCTCTTGAGCAGAGGAGTCT
X	PAK3	GTAGTTTCCTCCTATCCCTACATGCT	GGGCCAATGGAGGCTCA
X	PAK3	TGTCACAAGAGTACAAAAACAGCATCT	GGTCTTGGTGCGATAACTGGT
X	PAK3	GCCCCTCCTGTGTCtgaaga	CTCTATGACTATTCCAAAGGGCATCTTT
X	PAK3	AGTTTCCCAAGTGTAATCCTACAGGTA	CCAATTTTGTTATGTTGGAAGTTTGGA
X	PAK3	CAGAGCAATGGGCACGATTAC	CCTTTGATACCACAGACTTCCTCTTAC

X	PAK3	CATCCTTTATATTGCCATTTTTGTCCCA	GCACGATATAACAGGCCAAGCATTTA
X	PAK3	GTATGTTTCACTCTATGTCCCCCAAA	TTCCTTCTTGGGTTGCTGTTGA
X	PAK3	TGGTCATGAGGGAAAATAAGAACCC	GCAGAATGTTAAATGAACGAATCCCAAA
X	PAK3	AGCATTCAGTAATCGATTTCACTTGACA	ACCTGGTTTGAGTGCAGGAAA
X	PAK3	ATTTAATAGTGCCTGCAAGCTTTGG	AATTCCCTGATACTCTAATACCACCTGAA
X	PAK3	GAGTTTGCTAATTTTCCTCCCCTCA	CCTGTTGCAATGTCTAGTGCTGTATA
X	PAK3	CACAGGGCATCAGGTACTGTT	GCGAAGTTGCCATCGAAGAAGATA
X	PAK3	TGATGAACCTAATTGTACAACTTGTGGAT	CATCAAACCCCACATGAATCGTATG
X	PAK3	GAGATCTCTCTTCCTTCAGACTTTGAG	ACATGGCAATTCTTGCTTGAAAACTATT
X	PAK3	AAAGGTAATGGGCATAGCACAACT	CAAGTATTCCATGACTACCCATAGTTCA
X	PAK3	TCTTGTTATAGCTACTTGGTGGGTGA	GGTCTTCTCAGCTAGAAATGCTCTATTT
X	PAK3	CCCCATTCAGACCCATTGGTT	CAAGCTTCTTTTGGGCCTGTG
X	PAK3	TGGGCTTATTTTAACTGGCTTTTCTTCT	GACCAGATATCAACTTTCGGACCA
X	PAK3	GAGGTGGTGACTCGAAAAGCTTA	GGGAATAAAGTACCTGACTTCTCTTCTG
X	PAK3	TTTTTCTGGTGTGGTCTCTGAATGAA	GGAAACACCAATCATGTGAAACAGT
X	PAK3	GTCTGTGCCAAGCCATGATTT	GAATACAGCTGACAGTCTCTCAGG
X	PAK3	TGGAACTCCAGAGCTCCAGAAT	GGAAAAGCTGAGTATAATGCCTCTCTT
X	PAK3	ACAATACATTGACTCCACACTCTTTCC	AGAGGGTGGTGTGACCTCTTTA
X	PAK3	TGCTTCACCAGCAGTACCAAA	CGTGTAGAAAGTAATCATGGCAAGAAGT
X	PAK3	AGGTTTTTAGACCTCTTCTCCCTCAA	GCTGAAGAATCCCGGTTGTTACTA
X	PAK3	GGCTCCTCCACTGAGGATGAA	GCCCGGCCACTGATACTTTAC
X	PAK3	TTCAAAATATATCTTAATTCAAAAGGGCTGCTT	CTGGAGAGAGGCTTGGCTAATTTTA
X	PAK3	TCCTTCCTTTTGCAGCATCCATT	TCTTTCCTGCAGCAGAGTTTTATTTCA
X	PAK3	CTTCTCTCCCCACCCATCTCTTA	CAGAAACCCAAGCTCTTACTGAATTTT
X	SH2D1A	TTGTTATTTTTCTTTAGGGATAAGAGAAGATCC	GCATTTGTAGCTCACCGAACTGTATTAT
X	SH2D1A	GCATCCCTAGCACATTTTGTAGGT	GCAGAGGTATTACAATGCCTTGATCT
X	SH2D1A	AAATCTCATTTCAGCATTTCAGAAGCC	TGAGCTTCCAAACCCTGTCAAAA
X	SH2D1A	AAGTTTATTCTTTCACAGGTATCACGGT	AACCAGCCTAAATTTCAAATACCTCCTT
X	SH2D1A	GAACTGGGAGTCAGGTGGTTG	CCGGTTTCCCTGCTGATTTTG
X	SH2D1A	GGACGCAGTGGCTGTGTATC	GCCCATGTCCACCGTATCAT
X	SSX1	CATACCTAGCTGAGTCACTGACaaat	CTGTTACCTAGTTTAGTCATGGCCTT
X	SSX1	CAACGGAAGTCTCTCCAGAGT	CCATGAAAGGAAAAAGGTTCTCTGGT
X	SSX1	CTTCAACGTACCCAACCCTCAT	GGTGAGCCGAAGGTTCACTTAC

X	SSX1	CTGTTTCTCTTGCAGGTGAGACT	CAGAAACCTGGTCACCCTACTC
X	SSX1	ggccTAAGTTTGTCCCTTAAGGAA	CCTGCGGTTATGGTCATTATCAAAATC
X	SSX1	AAGATCATGCCCAAGAAGCCA	AGAGAAGTTGTTCCCAAATCATTTCCT
X	TAF1	GGGCTGTATCTAACTAAAGTGTTTTGA	ATCAGAAGGAAGCAATGCACATACT
X	TAF1	CCACTGCCCTGAGAATCTTTTTC	GTGTTGGTTTGTTTGGAATCTTCACA
X	TAF1	GGGTGTGGTGAAGGATTCTCCTA	ACTACATTCCAAGTCCTCCACTCA
X	TAF1	GTGTGTGTATCTGAGTGCCTGA	AGATGGACGGATTCATGGAAAGAAAAT
X	TAF1	CTGATCCTTTGGTGGCACTGA	CTGCTGAAGCTTGTGTCTTGG
X	TAF1	GCCTGATCCCAAGTCGAACA	GAGTGGAAATCCTCACTGTCCTC
X	TAF1	TCCACCTGTCAGAGGACGAG	TGAACCACCTAGGCTGGAGAA
X	TAF1	GTACTGCTAAAGAAGCAGCTTTGG	CGGATTTGTTAACATGCTCCCAATT
X	TAF1	GGGTTTCTCTTCCTTGTTGCAGT	CCATCTTCAGATTCTGCCTGTGT
X	TAF1	AGCTTGGTATTTCTTTCTCAGCAGT	CCCTGTCTTGTCCGAATGATCAAG
X	TAF1	GAACAACCTTTTTCGTGCTCCAATT	CGTATTGGCCCTTTTGGAGTTAGG
X	TAF1	AGATGGGACCTCAGGAAGCA	TGCAATCTGAGCATTCTCCACAG
X	TAF1	CAGTGTCCCTTGTTTGAAGTTCCT	ggccCCAACATACCCTTTCTG
X	TAF1	CTTTTGCCTGCAACCATATTTCTTTAGT	CATGTTCTCAATGTTCTTTCCCATTTCT
X	TAF1	GCTCAGCTGAAGATAGTGACTTTGA	TCTGTGAAGCCAAGATCCTCAC
X	TAF1	cccggcAAATTGTCCTTAACTTT	ACGCCACTTGGTCATCATCATC
X	TAF1	AGAAAGCTATCAACCCCTTGCTG	TTTTCCTTTCCCCTGCACGAT
X	TAF1	GTAAAGTGCTATGGTCATATTGTGTAGGA	AATTGGCCAGGATGGAGAGAAC
X	TAF1	TTTACTGCCATACATCTCCTTTCCTG	TCTGAGCTGACTGGTTCCTCAT
X	TAF1	TGTTCTGACGGAGGATATGGGAAA	CTTTTTCAGAGGTGGGCGATG
X	TAF1	CCATCAAACTCCGGCAGTTC	ACCAGAATTCAATTATACCTTGGCCTT
X	TAF1	ACTGGATACTTGTCTGTAGGAAAGGT	CGACTCTTCTTCAGAGATGATTCCTTCT
X	TAF1	CTCCCCCTCCAAGGAGAGTA	TCTTCTAAACACCAAGCCCCTTTT
X	TAF1	TGGATGGATTACTTTAGTCCTGAGATGT	TTGAATCCTCCGCCGTTCTTT
X	TAF1	CAACATCGGGAAGAGATGCGA	CCGTTAGCTGTAATCACACCACAT
X	TAF1	TGTTGCGATTCTCCCTGCTTT	GTTCAAGCCAATACACCAAAGAATCAC
X	TAF1	TCTCTTTAACTGAATTGCTCTGCATGA	GCATCTCGAGACATACTGAGGGA
X	TAF1	CCTCCTGATTTGTATGATACCAACACA	CACTTGCAGGCACAATAACAATCTTA
X	TAF1	GTGGCTAGGCCTGTTACAGT	CAGACTTAAGCACCCACCAGTT
X	TAF1	GTCTTTTCCAGGGATGGACTCA	TGGTGTCTAAGAGGAAGGTGTTCA
X	TAF1	CAAGCTTAACTCTTGATGACCCAGAA	GCTTTCTTCATTTTCCATGTCCATCCT
X	TAF1	CGCATGCTTCAGGAGAACACA	CTGTAGCATGTTGCTGTCGATTAC

X	TAF1	GCTGTTTTCCCATCTGACTTTGAGT	TCCTGGATCTGCTCTTCCTGT
X	TAF1	AAGAAGCACCGTGAGCTGAT	CCTTAATCTCAGGTCTCTATTTTGCCTA
X	TAF1	GCATTAAAAGGATAAAGAAGTGTTGGCA	CTGCCTTTCTCTCATTCTAACTGTCTTA
X	TAF1	GGCTTGTCCTTTGAAATCCCTGAA	AGAACGAGCCTGTTCTGTTGAC
X	TAF1	GATTGATGTGGTGCGCACAAT	ACTAAAACGACCCACTTGTTCTGTAG
X	TAF1	AGAGGAATGTCAGCGCATCTTT	GTCAAACTTTTAGGTGACCTCCTCTT
X	TAF1	GGAATTGGCTCTGGCAACTGTTA	GTTTTCGCGGAGTGTTTGTAGG
X	TAF1	AAATCATCACTCGGCCAATGGA	TGCAATCACGGAACATTTGATTCTTAC
X	TAF1	GTGGTGGCTTCTGGTCTCTTAT	GGCTGTTGTACAGTTCCTTCCTC
X	TAF1	GGAGATGGTGATCTTGCAGATGAA	AGAAGCAATGGTTGAGGCCTAC
X	TAF1	GAAATGGGTGAGTGGAGGACTT	CGAAGGTCTGCATCTGTTCCTG
X	TAF1	CAGCCAGTGAAGAAGACAGTGA	TTCCAACTCTATACAAAGCCCCTAGTAT
X	TAF1	cTCTGTTTTGAGCTTCCGGTAGA	GCAGTGATGGTAGCTGCTGT
X	TAF1	ACTGTTGTTTTATTTCCGGTCTATGGG	CCATTGATGTTGCCGAAAAGGAA
X	TAF1	GAGGCGGCCCATTTTCTTTAG	GCACCTCCTTCCTCTGCTTAG
X	TAF1	AACGTTTGGAAATCTTTTCTACCTACCT	TCTTCATCTCGAAACGTGCGATAAAT
X	TAF1	CCACTGGACGCTGTCTCAAG	aaGGAAGTGGTAAATCAAACTCACATGA
X	TAF1	TCAGGAAATTCTCCCTGCTCTACT	CACCCAGCATATCATACCACAGTC
X	TAF1	TGGCTGAGTGGCGTTATGG	ATTCCTTTCTTACATACCAGTGTTGCT
X	TAF1	GAGGAAGCATTCTGATTTCACATTTCTC	GCCTTGACATCCCTACTATAGATATCCA
X	TAF1	AGCCTACTTACCTCTGACGTGTT	CTTTTCCAACTCCTCCTCCTGTTC
X	TAF1	AACCCTGTTGCCATGACAGAA	CACTTCCTGCCTGGTATCTCTTAC
X	TAF1	GGGCTTGGTGTTTAGAAGAACA	CCCTGTTGCTTGGGATAATAATACTCAT
X	TAF1	TGAAAGATCCATGGAATCTCTCCAATG	CCCATATCCTCCGTCAGAACAAAG
X	TAF1	CTCTGTTACTATTTCCTCCTTGCACTT	CTGAGCATCCCAAATGATATTGTCC
X	TAF1	TCTGGTATATGGACGCTGGGA	GTTGACATTAACAGCAAGTTTTCAAAGG
X	TAF1	CGACTCGTGCTGTCCCTTTTT	CCTACTCCCCACAACCCAGA
X	TAF1	GGAAACCTTGACCAGTGACCAAT	TGTTTGACATCCTCCCCATCC
X	TAF1	CAGCTGCATTGGGAGGATGATAT	GCTAGACACAGCTGGCACAG
X	TAF1	GTAACCCAACAAAGGTGGTGAAATT	CACCTTACGTATGGTCTCTAAATCCATT
X	TAF1	AAGGAAGCTTAAGGCGGTTGT	GGAGGCATCAACTTGCAATCAAT
X	TAF1	GCCATTTCATCACCCAGTTAATAAGAAA	TGATGAAAAGAATCAGGCACTCAGA
X	TAF1	AGATGACTATGATGCTGATTGTGAAGAC	GCTCCTCAGACAAAAGTAGACAATCTC

X	TAF1	GGAGCACATCGGGAAAGATGAAAATA	GTGGCCGATCTTTACTTTTCCAGA
X	TAF1	GGCAGGTTTTTATTTACCGCCTTT	AGACAAATAACTAGTCACAACAGACGAC
X	TAF1	GGGACACACTGCTGATGACAG	ctTAGGACCTCCATCTTTACCACCTA
X	TAF1	TTTTGGTGTGTTTGTCTCAGATTGAAC	TCCTGAGGTGTGCGCATAAAAA
X	TAF1	CTTCAGGTGGTGGAGAGATGT	CAGAGACTCACCCGTTTATAATAGTTCTT
X	TAF1	GTTGGCATGGCAACCAAGATA	GCAGTTTGTCTCACTACAGCTATGT
X	TAF1	GATTTGGGTGCTTGGGCCTATA	AGATGATGCCTTCTCCATTTTCAGAC
X	TBX22	GTGAACTGTGACGCTCTCTCA	TTCAGATCCTTGAAGCTCCATTTGAATA
X	TBX22	GCTCTGAAAGTCTGGAAGAGAAAGA	TGACCCTTGTGAACAGCTTGG
X	TBX22	ACTGGGCTAATCTGGAGTGAAGT	TGAACATAGAATCTAGGAATGATGCACAA
X	TBX22	TGGTAGCTGGGAATACAGACCAT	GGGTAAAGGATTGTGCTCACGTA
X	TBX22	GCTGCAAAGAATCCCTTCACC	TCTTCCTCTCCGCCCTTTTTC
X	TBX22	CAAAAGAAAACTCCAAGACCCAATACAG	GGAGAGATGCGGAACCAGTA
X	TBX22	CACTATAAGCAATGGCAACAGTGTTC	GATGTCATTGCTATGCTGCTTACC
X	TBX22	AGCTCTTGGGTCAGTCCTTATTTTC	GCCTGGGCATAGAGTACCATTCTA
X	TBX22	GGGATGCTGAAAGTTGACTCTCT	AAACGTTCCAAGTTTTCTTACTTTGTGT
X	TBX22	GATTTTAAAACCTTTGGCGCAGAC	GCATGTACATATCCCCTTGTGTAGTATT
X	TBX22	CCATCACCCCAGAAGCACTTAG	TGCTGGATACCAATGAACATGATTACA
X	TBX22	GCAGGAACATCAAATGTCAAGTTCA	GGGAGCTTGTAGGTAAATGAAACATAGG
X	TBX22	GTCAAGTTTCTTTTGGAGAAGGCAAA	GATGCTTTTTAAGCCCAATAACTGTTGA
X	TBX22	TCCTTACTTTCTCCACTTTGCTTTTCA	CTTTCAGGAGCCGGTAAAACAAG
X	TBX22	AACTAATTTTTGGCAACAGCAACCT	GTAGATACTGATCACTGGAGTCTTCAGA
X	TBX22	GTCACCAATTCAAAAAGCGGTTCA	GTCATAGGAAGGATGAAAGGAGCAA
X	TBX22	GCCAGTTTTTCTAACAGCATTGATCAT	GTTTTAACACCTTCAGTGGGCAAG
X	TBX22	GTTGACCTGTCCCAGATTCAGTC	GCCTCTATGCCTCTAATCCAAGGT
X	TFE3	CCCACCACCATCTCCTCTTTTC	TCCTGAAGGCCTCTGTGGATTA
X	TFE3	CCTTCTGCAGCTTGCGGATA	AGCTCCTGAGAAACTCCTCATGT
X	TFE3	CTCCTAGGATCACTCCTCAGTCA	ATCTCTGTGGTTGGCGTCTC
X	TFE3	TGGACGGCTCAATGTGTGG	CTGCGGCAGCAGCTAATG
X	TFE3	CCTGTTCCCGACGCTCA	CTACACTCTCTGCATCGTCTTCT
X	TFE3	ATGAAGAAGATGACGACATGGCA	AGACCCTTTAGGCTTGGGATGA
X	TFE3	GAAAGAATGGGTTGGGCTGGTA	CGTCCTTGATCCTGACAGCTTC
X	TFE3	GGGTTGGCTTTTGAGCTCGTA	ATTCAATCACTGACCACCACTCC
X	TFE3	GCACACGCTCTCTGGCTTA	CATGTGTTTCTTGCCTTCCTGTG
X	TFE3	GGGCCAAGACCCTATCACCTA	GGTGGTTCTCTGCCCTCTCT

X	TFE3	GGGAATGTGGGAGGAGGTT	CTGAGGCTGCCCACACTAC
X	TFE3	CTCTGAGCTGGACCCGATG	ACAGTACCTGTCCACCACACT
X	TFE3	GCCTGGGAAGCCAGCTTG	CACCCCTTTGAGTCCTGTTCT
X	TFE3	AGGCAAGGGTCTCCTATACACA	GCCTGGAGTCCAGTTACAATGAT
X	TFE3	GGGCAGATAGCTGAGCATTTC	CACATTCCCCTCCCAAATTCTTCTAA
X	TFE3	GACGTCCTCCAAGGCTAGG	AGCCGTGTTCGTGCTGTT
X	TFE3	CGCACCTGAGCAGCTGA	CCGGGCGTCATGTCTCAT
X	TFE3	GCCATCCCGAGCTGGTTC	GCGCAGTGCTAGCTCCAT
X	TFE3	CCCACCAAGGCACAATTTAGTAG	CCTCTCCTCCCCTCTCCAAAG
X	TFE3	CTGCTGGCCTTGGAGACA	CTGGGAGACCCCTTCCAC
X	TFE3	CCTCCATCAGAATGTCCTCCAG	CGCAGCAACGTTCCATGTAG
X	TFE3	GGAGCATTCTGGGCAGGT	CTCCAGGGCTGCTTTCCTT
X	TFE3	TGTCAGAAGCCGAAGTCGTG	CTGAGGGACTCTGAACCCTACTTA
X	TFE3	TCATCCTGACTGGTCCTCCTTT	CCCTGCTCTCTTCAGTGTCC
X	USP9X	TCTGCAATGCTTGTCTATGTTGGT	ACCTTGACCTTGCTCATCTGG
X	USP9X	GAAAATTCCCCGGCAACTCC	GGTTACTTTCCTGTCTGCTCTCT
X	USP9X	CCCTCTCCCCCTCTTCTCTATTT	CCATATCTGCATTCGGTAGGAAGTTATT
X	USP9X	TCATAATGCACTGAAAGGAATTCCAGA	ACCTGCAGGATTTGGTAAGCA
X	USP9X	CCCCTTGTACTGAGTATGCTAACC	ACACCTTCTACACCTGGCTGA
X	USP9X	TGGTAGCTCTATTTAGTAACTGTCCTGT	CCCCAAGTTCTCATGATTCTCTTCTTTT
X	USP9X	TGTTCGAGCTGTGGCAGAAG	CACTTTGAACATTGAGTCAACCTTGA
X	USP9X	GTACGAACTCTTCTGGTTACAACAGA	TGAAGTTGATTGATTAAGTCATGGCGAT
X	USP9X	AGCGAAGTCCCCATGTGTTTT	AGGTCCCTGCTATTTCCCATAGTT
X	USP9X	AGGCTATTTTCTATCGTGAAAGTTGTGT	TGCTGAATTGGCATTATAGGTTGTGATA
X	USP9X	TCTTCCCAATCCTTTTGGTGATCC	GAGGATTCTCCCAGCAGCAA
X	USP9X	GGAAACCGTCAAATTGCTTCGTTT	GCAACCTAAAAACAAGTAAGACATGGT
X	USP9X	CTTCTCTGGCAGGTAAAAGGAAAATAAC	GCCGCAGTTCATAGGTATAGGAAT
X	USP9X	CAGAGTCTGCTGAATATGATGATGTCT	GTCGTACTGAACCAATTGTATCATTTGT
X	USP9X	TGATGACTTGGAGGTATGGTCTCAT	CCAATCAACTTTCTATCATCTGCAGGA
X	USP9X	CTGAGGCCAGGTGGTTTAAGT	AATGCTTCACAGCAAACCTGTTC
X	USP9X	CAATGAGCCAGACTTGGAAGAC	TGATGAATGTCTGCCAAGCCTTT
X	USP9X	CAACAGCCTTAGATGCTCTTAGTAAAGA	GCCAGCCTAAATTAATTCACTCCATTT
X	USP9X	GCATTGCTAATATGTAATCCCTTTTTCAACT	CAGACCGGAATAGCCTGTTCA
X	USP9X	ACCTACAGTATATGAGAAATGGAGAGCTT	AGATTCCTCACACAGCCAACAG
X	USP9X	CCACCTACAATTAATGCTGGTTTTGAAT	ACTTACTAGTTATTGCTGTGCCAATGT
X	USP9X	AGAATGGCCGTGTTCATGCTA	GAATCATGTAGAGTTGCTGAATCACAGA

X	USP9X	GCCGGTGCTACTTGTTACATGAAT	ACTCAAATTTACCTCATTGTCCTGCT
X	USP9X	GGGATGAGAAGCAGGACAATG	CGGCCCATCCCTAACTTTCAA
X	USP9X	CATTTCCAGAGACTTCAGACCATGAT	TTGTTCATGTGATGTGCTGCTG
X	USP9X	TGCATGGACAGCCATATACAGG	CCTGGGTGCACAGTCTTGAT
X	USP9X	TCAGCTTATTACAGCCAAACTTACACA	TCAAAACTGTATCTACTCACCACTCCA
X	USP9X	GCCCTGATAGCTCTTCTGATTCCT	gTTCAAAACTGTATCTACTCACCACTCC
X	USP9X	CAGTGTTTTGATCTTGTAAATCGCCTT	GAAGTTCTAAAAGTGGAAACCAGTCTTG
X	USP9X	ACTCATCGTCTGGTGGAGCTA	CAAAGAGTTCATCTTCAGGCAACTG
X	USP9X	CATGTGAATCAGTTTCCTCAAGTGTT	CAGATCACTGAAGATTCTGTGTCAGA
X	USP9X	GGTTCTCTCCAGCAGTAGAGGAA	CCAAAAACGTACATTCTTGCTGTGA
X	USP9X	TGATGCATTGTGTATTCTCCTTCGT	GCAAACGCACCCCTCACTT
X	USP9X	CGAATACCTTCTGGAGTGCCCTA	CTCATCATTTAGAAAGAAAGCTGTGCAA
X	USP9X	AAATGCAATGTTTTGTGGTCCTTT	GCCAAATTTGTTGAGAAGATCCACT
X	USP9X	TCATTAATTGTGTTACAGGGTTGGCT	TCTACGGTGCACTAACCATTGATTTT
X	USP9X	TGAGTTTTTCAAGTGTGGTTTCTTCAAC	AGAGTGAGAAACTCATAGCATTGCC
X	USP9X	TCAGACCATTTGGGCAATGCT	TCATTTTTGGCTTCATTCTTTGCTTCTT
X	USP9X	GGCAATGCTATGAGTTTCTCACTCT	ATTGAAAGAGCATCATTTTTGGCTTCA
X	USP9X	AAGCCAAAAATGATGCTCTTTCAATGA	TCGGCATTAACAATTATTTCAAGAGGTAAGT
X	USP9X	TCATTTGTTTAAGGACCGTGATACACA	GCGCTTCACCAAACAAACTACAAATT
X	USP9X	TTCCCGCACTGAAACAAATTAGAGA	CCAGCCAATATGCTGTTTCTTTTCAATA
X	USP9X	GGTACGAATGTGAAGAATCTTTTACGAC	AGCCCGTACCTTTTTATTGCATTTTTC
X	USP9X	ACTAGAAGGTGCAAATGCATATCATTGT	GGTGACTTGTGAGGATGAACCAAAA
X	USP9X	CCATGTCTTACTTGTTTTTAGGTTGCAT	TGTTTCCAGTCACAAAAAGAGTATCCA
X	USP9X	CAAAATTGCAAAGATCTGCCCTGT	gccCCACTGTAGCTTTAACTAGTATCT
X	USP9X	AGTGGGTGATGAATGAAGAGCAAT	AGGGCACTTTGTAGCACCAC
X	USP9X	AACCAAGTTACCCATGATCAAGCA	GCCTAGGAAAGTTAAAATTGTGACAGAAG
X	USP9X	GTAGCTCATTTGTAGTGCCTCTTTTAGT	CAGCTTCTAAAAGCACTTCTAATTCCC
X	USP9X	GTGGTTCCAGTTTTGCCGAAAG	CCACTTCATCCGCTTTTGGGAT
X	USP9X	CCCAAGAGGGAACAATGTTACAATCAT	TGTCCCATGCAACATCTGGTG
X	USP9X	GCACAGGAGCAGTTCTTTTTAATGT	TGCAAGCTAAAACCACAAATTTCTGAAT
X	USP9X	TTTTCCTGTGGATTTTAATAGAACATCTGGT	CCAGTCATAGTCAAATCGCTTTAGTTGT
X	USP9X	GATACCGTAAAGCGCTTGCTG	ACACCTGCAACTGTGTAAGGTT
X	USP9X	GAATTTCCTCGAGAGCTGGACAT	AGTCTGTATTTTGTGCTTCCTGCT

X	USP9X	AGAGTGAGCAGTCTGAAAGTGAGA	TCACCATCATCAAATTTATACCAGCGA
X	USP9X	GGAATGGTGGAGATGGTGAGAGA	CCTTTTCTGGCGCCTGTATG
X	USP9X	CTGGCCTAATTTCTTTGGTTGAAGTC	TGACTATATCTACTTCCCAGCCTCAC
X	USP9X	ACCATGAAGATTATGACCCACAAACT	CCAACAAAGACCAAGCTATACAGTCTA
X	USP9X	GTTTTAAAAGAAGAGAATCATGAGAACTTGGG	TCTTTCAAGTTCATCTCCAAGCCATT
X	USP9X	CAGGGCAATGGAGATCTTAAAAGAAAG	ACCATTGGACGTTTCATTGCTTTG
X	USP9X	AATCCTCAGTACACTTACAACAATTGGT	CTCTTCCATTTCTATCTGCTGCACA
X	USP9X	TGAACTCTGTCCAGAGGAGGTAAAA	ccagccGAATAAACATTTTTCAAAACC
X	USP9X	GTTAGCTGTCGGTGAAGTTTCCA	TGATATTGTTAGCCCATCACGGAAAAA
X	USP9X	GTTAAAAGTGAAGCATGTCAGCGA	CTCACAGGCATCACTATGTAAATCTGAT
X	USP9X	ATTAACACAGAGAAACTTACTTGGGCTT	GGGTGGCATATTTAGGCTGCTA
X	USP9X	CTTTGGCAAGTTGCAGACTTAGG	ACGGTCCATTTAGAAACAGAATTTGCTA
X	USP9X	GTGGATTTTGAGGATGGACGTGTA	CATTATTGAGAAGAACTTCAGCATTGGG
X	USP9X	AGTTGCTAATGGGTCAGTTTTGTAGATT	CGACTCATGTTCATCTGGAGCATC
X	USP9X	TTCAAAATTAGGAGCCAGATGACCAA	GGGATAAAAGCATAAACTGACTCCTGT
X	USP9X	TGAATCATTCTGTGCTTAGCTACGTT	CTCAAGTAGCTTTTCACGTTGCTTTT
X	USP9X	GCCAGTTGGACAAATGCGAGTA	ATCACTGTGAGCCAGATTCCAC
X	USP9X	GTTTGAGCCTATAGCCCAACTGA	TGGCATGGTCTAAACAAATAGCTCTTAAT
X	USP9X	CCTCCAGATAGCACAACGATAGAAAA	CCTCTGTTAGATATAGCACTTGTGAGG
X	USP9X	AGCCTCACAAGTGCTATATCTAACAGA	ACCCAAATCATTCACCTTACCATATGAAA
X	USP9X	GCACACAAAGTGTTGAACCTTCT	AGTGAGCAAAGCAGTACACTCTTAC
X	USP9X	TCATTTGTTCAATAACTGCTTGGCATTT	GCTGGTTTATCTTCAAATTGTTGAGGAT
X	USP9X	CAATGTTGATCCCAGGGATGATGT	GGGCACATAGTATTGCAGTCGAG
X	USP9X	GTCATCTTTGGTCATTTAGCTGCTT	AGATGCCTTCCAACATGTCTTTTAGAG
X	USP9X	GGGCATCAGGATGTGGGT	ACCACATGCACAATTAAGAAACATTTGT
X	USP9X	ATGATACTTAGGTAAGATACTTACCTCTTGAAA	CTTCATTCAGTGCATTCATCTTTCCAT
X	USP9X	CAATGGAAAGATGAATGCACTGAATGA	TGAAAGACTCACTGCCATTCGT
X	USP9X	GGAAAGATGAATGCACTGAATGAAGTT	TCCTATGAAGAAGAACTGAAAGACTCACT
X	USP9X	GTAGCCTGAGAAGGGTTCTTAACAA	AGTGATTTCTTCTGCTTCAGGCAA
X	USP9X	GCAAGATCACCTGTTGCCTGA	GGAATGAAAATCCAAAGCAATGTACCA
X	USP9X	TGCTACAATTAAATCAGCCATTGCC	AGACGATCAAAACAAGACTGAATGAAGT
X	USP9X	CTGATATTTGTAGGTGGTGATCCATGAA	CGAACCATTCGAACAGCTTCC

X	USP9X	CAAAGACAGTGTTAATTGTGCAAGACA	GTTATTCACAAACCTCGACATAGGGA
X	USP9X	AGATTCTTGTGCATTGTGATTTCGTTT	GATCAAGTTGTTCAGGAGAAAAATCCC
X	USP9X	TTGGCATGGGATTTTTCTCCTGA	CACCACAGACTGTTTAATGGGATTTCTA
X	USP9X	TGTTGTTTGTGGTCTTGTTGCTTT	GCATCGTGTTGTTCACGCA
X	USP9X	GGTGAGCCTGTTAATCTGCGT	CAGCAAAGGAACCTCCTAAGACTTTA
X	USP9X	CGTGAACAACACGATGCTTTAGAA	ACTTACCTATGTGGGCAGCCT
X	USP9X	TGAATTAACATTTAGGAATGGATACAGCAGAA	GCTGCCCAGATATTATCAAGATCCT
X	USP9X	GTCATCAAAGAAAAAGCTCTGACCTTAC	TGCATCCATTTCATGACAGCTACT
X	USP9X	CAGATTGCCTTTCCGGATAAAAATTACA	CTTAAAACAGGCTTCACGATCACAAA
X	USP9X	GAAATGCTTAGCTGAGAATGCAGTTTAC	AGAAGGATCAAGCTGAAGCACATTA
X	USP9X	GGGATGAACCAGACTTAGATCCTGATAT	TGCTACTAGTTTTCCTTCTCGACAATTC
X	USP9X	TGTTTGATCACATGATGAAGCGTATGT	TGGCTGATGGCATAATAATCTGATGAG
X	USP9X	GGAATGAAGTGTTTTGAGCGATTCTTC	ACATCAGACACCATTTACAGAGTTCCTA
X	USP9X	AGCTTGCTATCACCACCAGAC	GGAGGGTTTAAGTAAACGCCATTACAT
X	USP9X	CCGAATGCAGTACAGTATGGAGTATTTT	TGAACTTAAACATGTTGACGCCTACT
X	USP9X	CCTTGTTGCTGTAAAACAGGTAATGT	TACAAACAGGTTGAAATACTGCTGTAA
X	USP9X	CTTTTATCATTTTGAAACCAGGTCGTGA	GGGCCGAAAGAATTATATATGGAAGTTT
X	USP9X	ATTCATGAAGCCCTTGCCATCT	GGACCTTCATCTAAAGACACAAGCATAAA
X	USP9X	CTTCTGAAATTGAGTGTACCTGCTACT	ACCATTGATTGAAGACTGCATTCTTGA
X	USP9X	CTCTTTACCTCCCTTTCATAAGCTAGAC	TCAGAAGTTTGAAAGGCCAGTAACTC
X	USP9X	GCCACCTTGGAGTGACAAAG	TGATATGTAGCTTACAACCAAGAACACAG
X	WAS	CCCTGACCAGACTCCACTGA	GTAAAGGCGGATGAAGTAGGACT
X	WAS	TCGTGAAGGATAACCCCCAGA	GGTCTGAGGTCTTGAAGCTATGGA
X	WAS	GGGAACTAGAAAAGTCCCCTCTCA	CCTTATTACCCCTACTTGCACTAGAG
X	WAS	GCTCATTGCGGAAGTTCCTCTT	GTCGCTGGTTCTCGTGGT
X	WAS	TCCACCCTCCTCCAGGAC	GGAGATGATGGAGAGGGAAagga
X	WAS	AGAGGGCAAGAGGGTTTCACTA	GTCCAAGAGACTCTGAAGTTACTCAC
X	WAS	AGCCAATGAAGGTGAGTCCTCTA	GAAGAATCAGCACGCACCTC
X	WAS	TCCCTGCCCAGCTAACAAAAG	CCAGCGAAGGTGTGGAAGAAG
X	WAS	TGTACTCACAGCTTGTCTACTCCA	GCTTGGCAGTCCTGCACATA
X	WAS	GCTTGTCTCCTCGCCTTATTCC	CTGGTCCTCAATGAAGTCGTAGATAA
X	WAS	CGACGCCGAGACCTCTAAAC	GCTATGAGCTGCTTGGGCTA
X	WAS	AGGCCCTAAGCCCTCTGT	CCGGCAGGATCAGCTCA

X	WAS	CGGCTGACCCCAAGGTATG	CAACTTCCTTTCCTCCCCTGTG
X	WAS	AGCTATGTGTTATACCCCCTCCA	GGGCAATCCCCAAAGGTACAG
X	WAS	GGTAACAAGGGTCGTTCTGGT	AGTGGTCCAGAACGTCCAGTA
X	WAS	GGTCCACTGCCCCCTGTA	GGCAGTGGTCCAGAACGTC
X	WAS	gccCAGCTCCGGGAATG	CCTCCATCCTGCCTGTCCT
X	WAS	GTCAATGAGCCAACCACCCTATT	GATCTTCTTCTTCCCTGAGCGTTT
X	WAS	GGACCTAGCCCAGCTGATAAG	TCAGCTGTCCACTTGTTCATGT
X	WAS	ACCTCCCAGGCCCTATGAAG	ACAGGGCAGCAAGTAACTCAG
