# Supplementary material for: Cancer genomic profiling identified dihydropyrimidine dehydrogenase deficiency in bladder cancer promotes sensitivity to gemcitabine
Source: Sci Rep. 2022 May 20;12:8535. doi: 10.1038/s41598-022-12528-3 (PMC9122908; doi:10.1038/s41598-022-12528-3)
Supplement: Supplementary file 11 — Supplementary Information 11. [file 41598_2022_12528_MOESM11_ESM.pdf]

Supplementary Information

Cancer genomic profiling identified dihydropyrimidine dehydrogenase deficiency in bladder cancer promotes sensitivity to gemcitabine

Shigehiro Tsukahara[1,2], Masaki Shiota[1]*, Dai Takamatsu[1,3], Shohei Nagakawa[1], Takashi Matsumoto[1], Ryo Kiyokoba[2], Mikako Yagi[2], Daiki Setoyama[2], Nozomi Noda[2], Shinya Matsumoto[2], Tetsutaro Hayashi[4], Alberto Contreras-Sanz[5], Peter C. Black[5], Junichi Inokuchi[1], Kenichi Kohashi[3], Yoshinao Oda[3], Takeshi Uchiumi[6]*, Masatoshi Eto[1], Dongchon Kang[2]
